# How Genome-Wide SNP-SNP Interactions Relate to Nasopharyngeal Carcinoma Susceptibility

**DOI:** 10.1371/journal.pone.0083034

**Published:** 2013-12-23

**Authors:** Wen-Hui Su, Yin Yao Shugart, Kai-Ping Chang, Ngan-Ming Tsang, Ka-Po Tse, Yu-Sun Chang

**Affiliations:** 1 Department of Biomedical Sciences, Chang Gung University, Taoyuan, Taiwan; 2 Graduate Institute of Biomedical Sciences, College of Medicine, Chang Gung University, Taoyuan, Taiwan; 3 Chang Gung Molecular Medicine Research Center, Chang Gung University, Taoyuan, Taiwan; 4 Genomic Research Branch, Division of Neuroscience and Behavioral Sciences, National Institute of Mental Health, NIH, Bethesda, Maryland, United States of America; 5 Department of Gastroenterology, Johns Hopkins Medical School, Baltimore, Maryland, United States of America; 6 Department of Otolaryngology - Head and Neck Surgery, Chang Gung Memorial Hospital at Lin-Kou, Taoyuan, Taiwan; 7 Department of Radiation Oncology, Chang Gung Memorial Hospital at Lin-Kou, Taoyuan, Taiwan; Sanjay Gandhi Medical Institute, India

## Abstract

This study is the first to use genome-wide association study (GWAS) data to evaluate the multidimensional genetic architecture underlying nasopharyngeal cancer. Since analysis of data from GWAS confirms a close and consistent association between elevated risk for nasopharyngeal carcinoma (NPC) and major histocompatibility complex class 1 genes, our goal here was to explore lesser effects of gene-gene interactions. We conducted an exhaustive genome-wide analysis of GWAS data of NPC, revealing two-locus interactions occurring between single nucleotide polymorphisms (SNPs), and identified a number of suggestive interaction loci which were missed by traditional GWAS analyses. Although none of the interaction pairs we identified passed the genome-wide Bonferroni-adjusted threshold for significance, using independent GWAS data from the same population (Stage 2), we selected 66 SNP pairs in 39 clusters with *P*<0.01. We identified that in several chromosome regions, multiple suggestive interactions group to form a block-like signal, effectively reducing the rate of false discovery. The strongest cluster of interactions involved the *CREB5* gene and a SNP rs1607979 on chromosome 17q22 (*P* = 9.86×10^−11^) which also show *trans*-expression quantitative loci (eQTL) association in Chinese population. We then detected a complicated *cis*-interaction pattern around the NPC-associated *HLA-B* locus, which is immediately adjacent to copy-number variations implicated in male susceptibility for NPC. While it remains to be seen exactly how and to what degree SNP-SNP interactions such as these affect susceptibility for nasopharyngeal cancer, future research on these questions holds great promise for increasing our understanding of this disease’s genetic etiology, and possibly also that of other gene-related cancers.

## Introduction

Nasopharyngeal carcinoma (NPC; MIM 161550) mainly occurs in ethnically Chinese populations living in Southern China, Hong Kong, and Taiwan [1]. NPC tumors are believed to arise when Epstein-Barr virus (EBV) infects the nasopharyngeal epithelia of persons with certain (as yet ill-defined) genetic abnormalities that increase their susceptibility for developing the disease [2].

Genes associated with NPC include class I genes of the major histocompatibility complex (MHC), such as *HLA-A*, *HLA-B*, and *HLA-C*, as well as *RAD51L1*, *MDM2*, *TP53*, and *MMP2* genes [3]. In an earlier genome-wide association study (GWAS), we identified a span of approximately 400 kb on chromosome 6p21, characterized by associations with *HLA-A*, *GABBR1*, and *HCG9* genes, as a consistent NPC-susceptibility locus [4]. According to these findings, validated in two subsequent NPC GWAS studies [5], [6], these associations increase the odds of contracting NPC almost two-fold [4]. High-resolution molecular typing of HLA class I genes further indicates that on both the *HLA-A* and *HLA-B* genes, the association signals occur in the antigen-recognition groove [6].

Although GWAS analysis has linked different medical disorders with thousands of genetic variants [7], known variants still account for only a small fraction of the heritability of complex diseases [8], [9]. Much of the rest, it has been suggested, may stem from genetic interaction [10]. In the case of Crohn’s disease, for instance, when considering multiple interactions among different pathways, genetic interactions have been implicated in roughly 80 percent of heritability that is currently unexplained [11].

Interaction analysis successes include associating *HLA-C* interaction with *ERAP1* with susceptibility for psoriasis [12], and *HLA-B27* interaction with *ERAP1* with susceptibility for ankylosing spondylitis [13]. Analysis of genome-wide genetic interactions has been used to investigate the genetic architecture of heritability in prostate cancer [14], [15], type 2 diabetes [16], levels of high-density lipoprotein cholesterol [17], the body mass index [18], serum uric acid concentration [19], and various complex diseases [20]. Yet most interactions identified in the discovery stage of these investigations cannot be replicated or validated in independent samples. This failure continues to impede genome-wide genetic interaction research, with the result that the genetic architecture responsible for most inherited diseases remains to be explored.

Because analyzing for genetic interactions throughout the genome imposes a heavy computational burden, most epistasis investigators try to narrow their focus. Therefore, in the discovery stage, prior to the initiation of interaction analysis, investigators typically prioritize single nucleotide polymorphisms (SNPs) according to the significance of their marginal effects [21], [22]. Perhaps as a result of this targeting, however, very few convincing genome-wide genetic interactions between complex disease loci have been identified.

In this study, we used a genome-wide analytical approach to identify possible SNP-SNP interactions involved in the development of NPC. Many of the interacting pairs of SNPs were analyzed using data drawn independently from an ethnically similar population. We found that in several chromosome regions, many suggestive interactions group together to form a block-like signal, effectively reducing the rate of false discovery. In addition to identifying several regions where multiple signals interact, we also discovered a complicated pattern of *cis*-interactions within MHC region. Since this region is linked with immunity and susceptibility for NPC, this finding clearly merits further functional analysis. Our study shows how targeted analysis of GWAS data can be used to uncover interactions between and among genes, providing new avenues for research into the genetic etiology of NPC.

## Results

### Stage 1: Genome-wide Two-locus SNP-SNP Interaction Analysis

We conducted a genome-wide two-locus analysis of SNP-SNP interactions for associations with NPC using our previous NPC GWAS data set [4]. To lessen the computational burden of conducting genome-wide SNP-SNP interaction analysis, we split the data into 24 sets according to chromosome location for the PLINK “epistasis analysis”. These analyses, which were run in parallel on a 48-processor machine and took over four months to complete, identified a total of 4,244,943 unique interactions with *P* values<1.00×10^−4^.

When subjected to 1.15×10^11^ statistical tests, however, none of the 66 pairs of SNP-SNP interactions with *P* values<1.00×10^−8^ identified by our initial analysis achieved a genome-wide level of significance of *P*_interaction_≤4.34×10^−13^. The strongest interaction (*P*_interaction_ = 1.97×10^−10^) detected was between SNPs rs17233815 and rs10871618. (See *Table S1* for the top 10,000 interaction pairs identified, *P*_interaction_<6.78×10^−7^).

We further observed that 99.72% of the top 10,000 SNP pairs identified as interacting in our Stage 1 dataset contained one SNP that produced an only small single-locus effect (*P*_single_>0.05). Most of the stronger interacting SNP pairs contained SNPs with moderate single-locus effects paired with SNPs with small single-locus effects. The highest single-locus association *P* value we observed occurred in the pair rs1884008 (*P*_single_ = 7.87×10^−1^) and rs4561414 (*P*_single_ = 1.09×10^−5^), which produced a moderate interaction *P* value (*P*_interaction_ = 2.39×10^−7^).

In Stage 1, the interaction SNP pairs tended to be grouped together in clusters. Our top 100 interaction pairs, for example, contained 16 such clusters. The largest clustering of interaction pairs (19 out of 100) involved MHC-region SNPs interacting with SNPs in nearby *HLA-B/C* genes. A second potentially cluster we identified contains SNPs in the *PDGFD* gene (a member of the platelet-derived-growth-factor family) that interacts with SNPs in a ‘gene desert’ area of 8q24.

### Stage 2

To test our initial results, we analyzed a smaller NPC dataset, collected independently from an ethnically similar population, for the top 10,000 interacting SNP pairs identified in Stage 1. Of these 10,000 interacting pairs, 467 (4.67%) could not be tested in the Stage 2 dataset, possibly due to differences in the genotyping platforms that caused SNPs included in the Stage1 dataset to be omitted from the Stage 2 dataset (*Table S1*). In our Stage 2 analyses, the lowest *P* value achieved (*P*_interaction_ = 1.68×10^−5^) was from an interacting SNP pair ranked 8761^st^ in the Stage 1 analysis. All of the top 100 interaction pairs we had initially identified failed to replicate (*P*_interaction_>0.01) in Stage 2.

### Combined Analysis

In the second stage of our study, we sought to increase the power of our analysis by increasing the size of our sample. We therefore combined data from our Stage 1 and Stage 2 datasets and analyzed for SNP-SNP interaction in the 10,000 top pairs identified from Stage 1. All the SNP pairs in the second stage failed to achieve Bonferroni corrected p-value<0.05 threshold, therefore, we only selected SNP pairs with interaction *P* values<0.01 in the Stage 2 dataset and 5.00×10^−7^ in the combined analysis. We then performed permutation tests 10,000 times in all three datasets (Stage 1, Stage 2, and combined), keeping only results above the permutation *P* value threshold (*P*_permutation_<0.01).

Due to the limited sample size, we recognized that our study could be under-powered and therefore is likely to have missed many true positives. Our study can achieve the power of 0.92 when using Epistasis Power Calculator suggested by PLINK; which were very similar to the result of two previous publications [23], [24]. However, the power calculated might not be applied to our situation since those power calculations usually assume the variants are causal and ours are unlikely to be the causal variants. The approach we used may detect the surrogate markers for the effective SNPs. On the other hand, the power of our study can only achieve 0.0063 when using powerGWASinteraction [25].

Although the power achieved in these analyses was unlikely to allow for solid conclusions, we were able to identify 66 interacting SNP pairs of potential interest for further analysis (*Table S2*). Of even greater interest is the fact that these 66 potentially interacting SNP pairs cluster into 39 interacting groups (*Table S3*), of which 12 (31%) are associated with at least two nearby SNPs from one of the interacting chromosomes.

*Table 1* lists the 10 suggestive interacting regions with the strongest levels of interaction in the combined analysis and the two suggestive interacting regions within the MHC region. *Figure S2* shows detailed odds ratios for interactions listed in *Table 1*. To compare interacting regions with interaction signals, we plotted linkage disequilibrium (LD) structures (*Figure S3*).

**Table 1:** The top 10 and MHC interaction regions associated with NPC susceptibility.

Region A						Region B						Interaction *p*-value			Suggestive Interaction Number^d^
Chr.	SNP^a^	Location	Distance (bp)^b^	NearestGene	Single- locus *P* c	Chr.	SNP^a^	Location	Distance (bp)^b^	Nearest Gene	Single- locus *P* c	Stage 1	Stage 2	Combined	
7p15	rs2237353	intron		*CREB5*	5.03E-01	17q22	rs1607979	upstream	566,730	*KIF2B*	3.78E-01	9.96E-08***	5.87E-04**	9.86E-11***	2
7q11	rs6460664	intron		*WBSCR17*	2.18E-01	9q33	rs2300932	intron		*C5*	6.11E-01	9.63E-09***	9.76E-03*	8.86E-10***	3
4q26	rs6821696	downstream	5,428	*MIR1973*	2.49E-01	10q26	rs1380439	intron		*ADAM12*	6.89E-01	2.12E-07***	8.37E-04**	8.90E-10***	1
2q36	rs10933155	upstream	13,764	*RHBDD1*	5.33E-01	21q22	rs1888469	intron		*ERG*	9.91E-01	3.67E-08***	1.45E-03*	1.04E-09***	1
7p21	rs10487781	intron		*DGKB*	4.38E-01	7q31	rs17154507	upstream†	54,319	*DLD*	8.30E-01	4.58E-08***	2.35E-03*	1.43E-09***	1
9p21	rs1332173	upstream	175,899	*TUSC1*	8.01E-01	16p13	rs2127065	intron		*RBFOX1*	3.49E-01	3.64E-07***	4.24E-04**	1.76E-09***	6
2p23	rs6726261	upstream†	11,931	*ADCY3*	4.51E-01	9q21	rs11140659	upstream	120,855	*NTRK2*	4.23E-01	2.21E-07***	3.85E-03*	1.92E-09***	1
2p16	rs7589636	upstream	444,753	*VRK2*	5.47E-01	2p16	rs730402	upstream	513,286	*MIR4432*	2.73E-02	4.59E-07***	1.47E-03*	2.11E-09***	5
1p34	rs4660176	intron		*KCNQ4*	3.08E-01	7q36	rs740576	intron		*DPP6*	8.98E-01	6.33E-08***	2.97E-03*	2.11E-09***	1
5p15	rs4571472	intron		*FBXL7*	3.10E-01	10q21	rs7075349	intron†		*ZNF365*	2.74E-01	6.28E-07***	1.93E-03*	3.38E-09***	1
**MHC**															
6p21	rs2523849	intron		*HCG22*	4.48E-01	6p21	rs4947296	downstream	20,822	*C6orf15*	1.90E-01	4.28E-07***	1.97E-03*	8.42E-09***	3
6p21	rs7761965	downstream	48,764	*HLA-B*	8.90E-01	6p21	rs2596501	downstream	1,298	*HLA-B*	4.68E-02	2.64E-07***	7.40E-03*	1.81E-08***	2

^a^ The most significant SNP pairs within the region.

^b^ Distance between the most significant SNP and its nearest gene.

^c^ Single-locus association *p*-value from the discovery data set.

^d^ The number of significant interactions within this region. For detailed information, see *Table S3*.

SNP located within the putative transcription regulatory region annotated by the UCSC genome browser (http://genome.ucsc.edu/).

Permuted *p-*value<0.01 after 10 000 permutations.

Permuted *p*-value<0.001 after 10 000 permutations.

Permuted *p*-value<0.0001 after 10 000 permutations. For detailed permutation *p-*values, see *Table S3.*

### Suggestive Interacting Regions Identified

**Figure 1:**
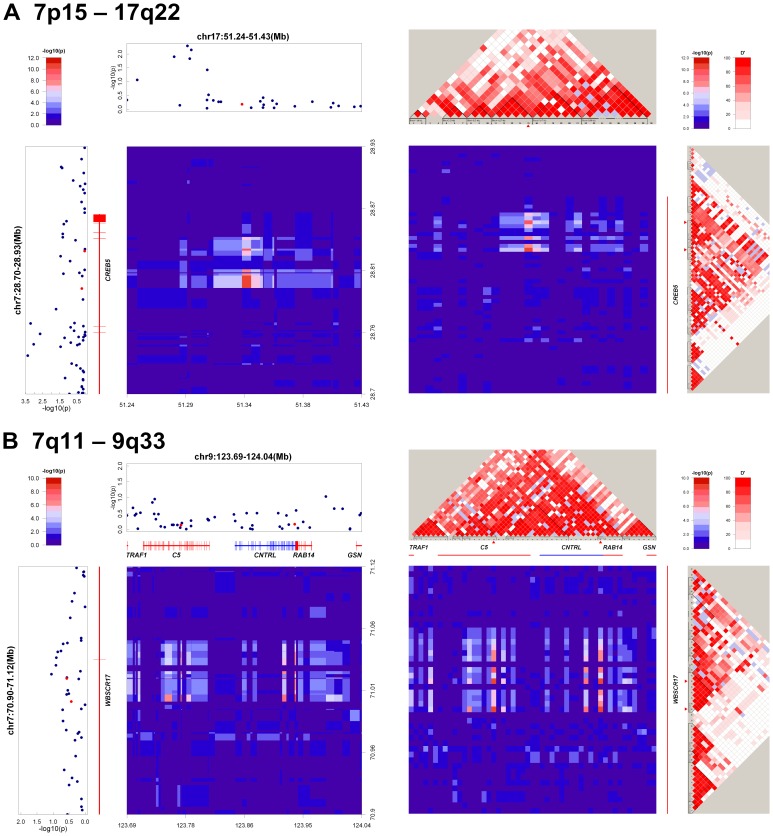
Regional signal plots of all SNPs within 100 kb of the top 2 interactive. SNP pairs identified. In each figure, the left panel shows the interaction signal heat-map and single-locus signal plots. The heat-map is aligned by chromosome positions based on NCBI build 36. Single-locus signal plots with gene annotations *(lower left and upper-right)*. In the single-locus signal plots, red is the position of suggestive-interacting SNP pairs in the corresponding regions. In the single-locus signal plots, solid black dots denote logistic regression test *P* values. In the interaction-signal heatmap plot, interaction *P* values, transformed by a negative logarithm, are coded by color *(key in upper-left box)*. *Interaction signal heatmap and LD plot (right panel)*. Heatmap is aligned with each SNP equidistant from LD plot positions. Interaction-signal heatmap and LD plots are color coded *(key upper right)*. Single-locus signal plots *(lower left)* same as above, except that coordination is not based on chromosome position. In detailed LD structures estimated in control samples *(upper left and lower right)* increasing intensities of red represent higher D’ values, and solid red triangles denote the positions of potentially interacting pairs of SNPs. Genes annotated in this region are also depicted according to their relative positions on the LD plot. On the heatmap, yellow dot is position of suggestive interacting pairs of SNPs in their corresponding regions.

The strongest levels of interaction identified by our initial analysis occurred between rs2237353 (on the *CREB5* intron) and rs1607979 (566 kb upstream of *KIF2B*), with interaction *P*_combined_ = 9.86×10^−11^ (*Table 1* and *Figure 1A*). Patients who carry double homozygotes AA/GG (odds ratio *[OR]* = 3.57, 95% with a confidence interval [CI] of 1.69–8.10) and CC/AA (*OR* = 2.68, 95% CI 1.44–5.16) for both SNPs are, in fact, at higher risk for developing NPC (*Figure S2A*). Within the *CREB5* intron, we identified another SNP, rs2237361, interacting at relatively high levels (*P*_combined_ = 7.44×10^−10^) with rs1607979. These two more interactive SNP pairs joined with nearby SNPs to produce a block-like signal with strong LD (*Figure 1A*). Earlier GWAS analyses did not identify single-locus associations between NPC and these interacting SNPs (*P*_single_>0.01; *Figure 1A* and *Table S2*). The expression quantitative loci (eQTL) analysis between rs1607979 genotype and *CREB5* expression (performed using Genevar [26] in HapMap3 dataset [27]) indicated suggestive *trans*-eQTL associations (*P*<0.05) in Han Chinese populations (*Figure S4*).

Our analysis also identified interaction between chromosome 7q11 and 9q33. As shown in *Table S2*, our results indicate that two SNPs within the *WBSCR17* intron (rs6460664 and rs6460671) interact with SNPs in two adjacent genes (rs2300932 in *C5,* rs3789311 in *CNTRL*). In *C5* and *CNTRL*, these interacting SNPs formed two separate blocks that appear to interact with *WBSCR17* independently (Although, given the strong LD between *C5* and *CNTRL,* perhaps not. See *Table S4* and *Figure 1B* *,* for LD’s potential effects on blocks of interactions within other interaction groups).

### Complicated Pattern of Interactions in MHC Region

Previous GWAS studies have shown that SNPs located within the chromosome 6p MHC class I region can profoundly affect susceptibility to NPC [4]–[6]. After our Stage 2 and combined analyses, two interaction regions in the MHC passed our filtering criteria (detailed in *Tables 1*, *S2,* and *S3*).

**Figure 2:**
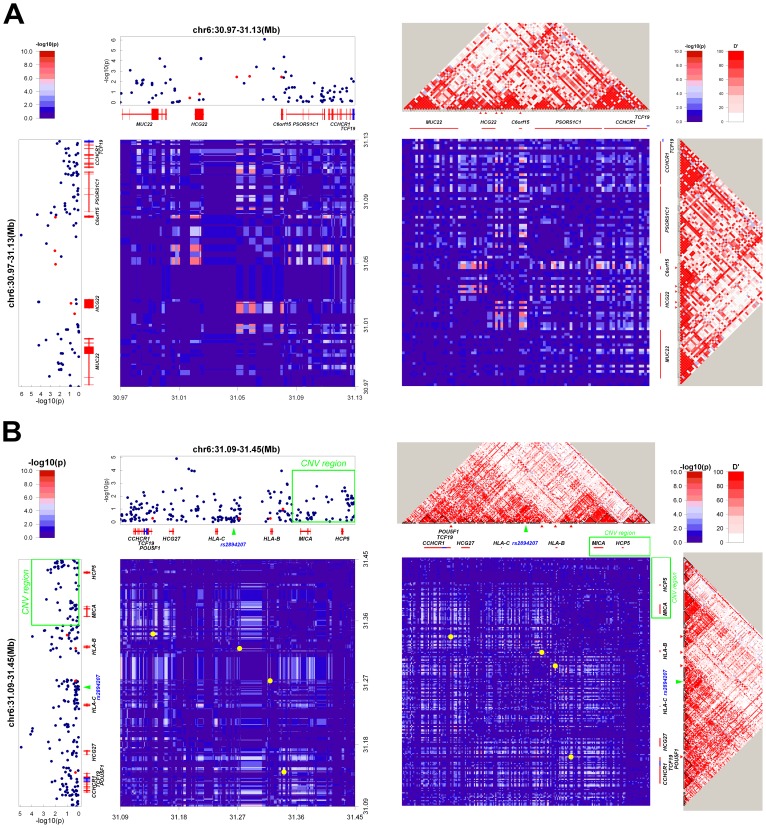
Regional signal plots of the interactions observed in the MHC region. The layout of this figure is similar to that described in Figure 1. Because these SNP pairs are located in a small region with a complicated interaction pattern, the same chromosome region is plotted in the 2 axes of the heatmap. On the heatmap, green arrow is position of the NPC-susceptibility SNP (rs2894207) identified by a previous GWAS study [5], green box is the copy number variations related to NPC susceptibility in men [37].

Interestingly, a small area of SNPs within those two MHC regions showed complicated patterns of *cis-*interaction. Three SNPs (rs4947296, rs9380215, and rs2233984) near *C6orf15* interacted with two upstream SNPs (rs2523849 and rs2523864) near *HCG22*. Although the level of interaction did not reach statistical significance, SNPs located near *C6orf15* also interacted with SNPs downstream of chromosome 6. (*Figure 2A*).

In each case, all of the SNPs located within this small MHC region formed an interaction block characterized by complicated patterns of *cis*-interaction. Haplotype analysis revealed a higher level of significance (*P*_combined_ = 8.35×10^−10^) for the association between SNPs with increased interaction in this region and NPC susceptibility (*Table S4*). The complicated *cis*-interaction patterns we detected may therefore reflect some type of haplotype effect.

Immediately downstream of this small MHC region, another *cis*-interaction region identified by our analysis contains (1) a SNP (rs879882) upstream of the *POU5F1* gene that interacts a SNP (rs7770216) close to the *HLA-B* gene, and (2) two SNPs (rs7761965 and rs2596501) downstream of the *HLA-B* gene that also interact with each other (*Figure 2B* *, Table S2*). Results from haplotype analysis suggest a potentially association with NPC (*P*_combined_ = 2.00×10^−6^), (*Table S5*).

### Interaction Profiles of GWAS Significant SNPs

We next analyzed the genome-wide interaction profile of 18 significant SNPs identified in previous GWAS analyses [4], [5]. (Note that SNP rs28421666, located near the *HLA-DQ/DR* gene, was excluded because it had not been included in the genotyping platform of our Stage 1 dataset.) When tested in our Stage 1 dataset, all 18 SNPs obtained good interaction *P* values (*P*_interaction_<1.00×10^−4^, *Table S6*). Most interacting counterparts of GWAS-significant SNPs, however, were located outside the MHC class I regions where single-locus GWAS *P* values were non-significant (*P*_single_>0.05). In chromosome 21, for example, both *GABBR1* and *HLA-F* genes interact with *SLC37A1* while the *HLA-A* gene interacts with *RTCD1* (in chromosome 1), *KCNMA1* (in chromosome 10), *PGM2L1* (in chromosome 11), and *NUDT7* (in chromosome 16). Interesting as the findings potential are, however, most failed to replicate when subjected to further analyses in the Stage 2 (*P*_interaction_>0.05) and combined (*P*_interaction_>1.00×10^−4^) datasets (*Table S5*), and we found no significant *cis*-interaction signal in the NPC-associated region near the *HLA-A* gene (*Figure S3M*). Our analyses have therefore failed to identify interactions in the GWAS-significant MHC class I region rising to the level of significance.

## Discussion

To explore SNP-SNP interactions implicated in NPC, we divided genome-wide SNPs into subsets and performed a full pairwise scan. In this way we generated a complete profile of SNP-SNP interactions associated with NPC, which we analyzed in two different stages.

Our analyses revealed a number of interaction regions, each containing many interactive pairs of SNPs. The multiplicity of interaction signals produced per region reduced our likelihood of false discovery. Although we also identified two regions of *cis*-interaction close to an *HLA-B* locus known to be important for NPC susceptibility, none of the interaction pairs identified rise to the level of genome-wide significance.

The fact that the strongest interaction *P* value identified in this study (9.86×10^−11^) failed to achieve the Bonferroni-adjusted threshold for genome-wide significance (*P*_interaction_≤4.34×10^−13^, considering 1.15×10^11^ statistical tests) may be due to the limited size of our sample. It has further been suggested that–considering the LD structure among SNPs–the cutoff for genome-wide significance in SNP-SNP interaction analysis of approximately 500,000 SNPs should rather be *P*_interaction_≤4.2×10^−11^ [14]. Using this standard, our combined analysis finding of interaction between rs2237353 and rs1607979 (*P*_interaction_ = 9.86×10^−11^) comes close to achieving significance.

Still another suggestion is that rather than the conservative Bonferroni correction for multiple testing, a permutation-based strategy should be used to verify interaction analyses [21], [28]. In our study, all selected interactions were *P*_permutation_<0.01 in the 10,000-permutation test in all 3 analytic conditions and datasets (Stage 1, Stage 2, and combined). However, the permutation *P* values are not corrected ones and should be interpreted carefully.

In addition, most of the interactions we identified grouped together to form stronger, block-based signals. The detection of many potentially interaction SNPs within a region of strong LD indicates that these interactions are neither sporadic nor the spurious artifacts of genotyping. Other genome-wide interaction-based association analyses have reported similar interaction patterns [20]. The observation of multiple interaction signals within a chromosome region effectively reduces the likelihood of false positives.

Because the assessment of all pairwise interactions demands extensive computational resources, researchers generally prioritize which SNPs identified by GWAS are to be subjected to further testing [21]. Statistical examination suggests, however, that modifying thresholds to account for reduced SNP numbers does not protect studies from falsely identifying large numbers of interactions. The need to eliminate these false positives makes replication and permutation studies doubly important [29].

In our study, interactions identified using high-impact GWAS SNPs could not be successfully replicated (*Table S5*), and our highest interacting pairs all displayed small, single-locus effects (*Table 1*). That similar results have been reported by other genome-wide interaction-based association analyses [14], [16], [20] suggests that it may be counter-productive to use high-impact GWAS SNPs in interaction analyses seeking to identify significant interaction signals.

Another recognized issue is the number of interaction pairs selected for validation analysis. Most other SNP-SNP interaction studies that have failed to replicate their findings selected only a few top interaction pairs for validation. One prostate cancer study, for instance, conducted validation tests on only 16 of 1,325 pairs of top SNP-SNP interactions [14]. In this study, however, although we tested all top 100 interaction pairs, none achieved significant *P* values in our Stage 2 analysis (*Table S1*).

Our pair-wise genome-wide search of interacting SNPs revealed several suggestive two-locus associations (*Table 1*). The strongest one was the interaction between two *CREB5* intron SNPs (rs2237353 and rs2237361) and a SNP (rs1607979) located in a gene desert region of 17q22, where the nearest gene *(KIF2B)* is 566 Kb away (*Figure 1A*). *CREB5* is a member of the cAMP-responsive element (CRE)-binding protein family implicated in tumorigenesis in acute myeloid leukemia and prostate cancer.

Although interaction between the 17q22 and *CREB5* regions is not currently documented and we know little about the molecular function of the 17q22 region, our finding suggests a possible regulatory function for this locus that need be validated using molecular experiments. Studies have already associated the gene desert locus of 8q24 with increased susceptibility for prostate, colorectal, and breast cancer; and subsequent molecular experiments have revealed a tissue-specific long-range *cis*-interaction between this region and the proto-oncogene *MYC* [30], [31]. These findings suggest that, as with 8q24 and *MYC,* the *trans*-regulation of *CREB5* may well lie in region 17q22. Further support for this hypothesis comes from the eQTL analysis of a HapMap3 Chinese population, which indicates a suggestive association between rs1607979 and *CREB5* expression (*Figure S4*). In addition, the provirus integration site for xenotropic murine leukemia virus-related virus (XMRV), an infectious retrovirus associated with a predisposition for prostate cancer, has also been mapped to *CREB5* [32]. The relationships among EBV, *CREB5,* and 17q22 in nasopharyngeal cancer are therefore well worth further investigation.

Another plausible interaction identified in this study involves the *WBSCR17* intron and the chromosome region in 9q33 that contains *C5* and *CNTRL* (*Figure 1B*). *WBSCR17* is known to play important roles (through O-glycosylation, controlled by GlcNAc concentrations) in the formation of lamellipodia and the regulation of macropinocytosis [33]. *C5* is involved in the formation of the membrane-attack complex [34]. *CNTRL* encodes a centrosomal protein required for abscission mediated by secretory vesicles [35]. All three molecules are involved in the membrane-trafficking function. How these interactions affect NPC susceptibility, perhaps by regulating the EBV or cancer metastasis, has yet to be elucidated.

Previous genetic studies have described the MHC region, especially MHC class I genes, as the major susceptibility locus for NPC [3], [36]. The most significant NPC susceptibility locus, identified in multiple GWAS analyses, is a chromosomal region (of approximately 400kb) comprising the *GABBR1*, *HLA-F, HCG9*, and *HLA-A* genes [4]–[6]. Two GWAS studies suggest that another independent signal for NPC-susceptibility may be located in the *HLA-B* gene [5], [6], although whether the HLA-NPC association is directly related to HLA genes or to other susceptibility SNPs in LD with the HLA genes remains to be investigated.

We also identified a complicated SNP *cis*-interaction pattern in the chromosome 6p21 region (30.97–31.45 Mb) located near the *HLA-B* gene. In fact, two *cis*-interaction clusters can be found in this region. In region 1, SNPs downstream of *C6Orf15* and upstream of *HCG22* interact with SNPs between the two genes. In region 2, SNPs rs7761965 and rs2596501 (both located upstream of *HLA-B* gene) interact with each other, whereas SNP rs7770216 (located downstream of *HLA-B*), interacts with SNP rs879882 (located upstream of the *POU5F1* gene).

It should be noted that region 2 coincides with the previously GWAS-identified susceptibility locus for NPC upstream of the *HLA-B* gene [5], [6]. In addition, a CNV region related to NPC susceptibility in males [37] is located adjacent to this region. The complicated *cis-*interaction pattern identified in the MHC region could result from strong LD or haplotype associations with the MHC region. Indeed, since substantial LD occurs in areas where chromosomes interact, the LD effect cannot be ignored (*Figure 2*).

High-resolution molecular typing of HLA class I genes suggests that in the genes *HLA-A* (the major NPC susceptibility locus identified by GWAS [4]–[6]) and *HLA-B*, the signal associated with NPC is located in the recognition groove. It further suggests that other significant associations in strong LD with the *HLA-A* gene are only proxies for HLA-A*11∶01 [6]. In this study, we saw no evidence of block-like interaction signals in the strong LD region close to the *HLA-A* gene (*Figure S3M*), possibly because strong LD may produce proxy effects without affecting SNP-SNP interactions in nearby genes.

Alternatively, the lack of gene interaction in the strong LD region near the *HLA-A* gene may be due to haplotype associations within the chromosome region. Increased risk for NPC has so far been associated with HLA-A*0207 and HLA-B*4601, as well as with the extended haplotype: HLA-A*3303-B*5801/2-DRB1*0301-DQB1*0201/2-DPB1*0401 [38]. Advanced molecular typing also associates NPC with *HLA-A-B-C* haplotypes, which exhibit both a susceptibility effect (HLA-A*02∶03-B*38∶02-C*07∶02) and a protective effect (HLA-A*11∶01-B*13∶01-C*03∶04) [6]. Our own haplotype analysis revealed increased risk for NPC when the *HLA-B* locus contains interacting SNPs, suggesting that in some cases, *cis*-interaction might reflect an underlying haplotype effect (*Figure 2A* *, Table S4*).

In sum, our genome-wide two-locus SNP-SNP interaction analysis provides a feasible approach that, when refined, should increase the potential for successful replication. The *trans*-eQTL association observed in Han Chinese suggests that rs1607979 may play some role in the regulation of the *CREB5* gene. This study extends the spectrum of possible NPC-susceptibility signals. It also identifies a complicated pattern of *cis*-interaction in the *HLA-B* locus, which HLA molecular typing, GWAS, and CNV analysis have shown to contain many signals related to NPC susceptibility.

## Materials and Methods

### Ethics Statement

This study was reviewed and approved by the Institutional Review Board and Ethics Committee of Chang Gung Memorial Hospital, Taiwan. Written informed consent was obtained from all study participants.

### Genome-wide Two-locus SNP-SNP Interaction Analysis

This study is a two-stage search for SNP-SNP interactions in persons with nasopharyngeal cancer whose aim is to identify novel loci associated with elevated susceptibility for the disease (*Figure S1*).

### Stage 1

#### Sample

For our initial analyses, we used a previously published NPC GWAS dataset [4] collected from 277 NPC patients and 285 healthy controls. All subjects are of Han Chinese descent living in Taiwan. Genotyping was performed using Illumina Hap550v3_A BeadChips, which provided 480,365 SNPs for GWAS analysis. A series steps were also performed for quality control, as previously described [4]. The inflation factor lambda of the original GWAS that corresponds to the discovery dataset was 1.039, suggesting the absence of major population structure associated with case-control status.

#### Whole-genome two-locus SNP-SNP interaction analysis

A PLINK epistasis analysis (v1.07) [39] was used to identify SNP-SNP interactions on a genome-wide scale. The “epistasis” option in PLINK provides a logistic regression test for interaction that assumes an allelic model for interactions and their principal effects. PLINK constructs a model based on allele dosage for each SNP A and B, and fits the model in the form of: Y ∼ b0+ b1.A+b2.B+b3.AB+e. The test for interaction is based on the coefficient b3 and therefore considers allelic-by-allelic interaction only. Because the output could contain millions or even billions of lines, the default is to output tests with *P* values<1.00×10^−4^. Testing for all for all two-locus interactions, we split 480,365 SNPs into 24 chromosome sets, then analyzed for all possible interacting SNP pairs located within 2 chromosome pairs and within individual chromosomes. For chromosome pairs with set files containing 2 chromosome SNP sets, we used a SET1 × SET2 test in PLINK epistasis (as in: chromosome 1 vs. chromosome 2, vs. chromosome 3, vs. chromosome 4, etc.). For each individual chromosome, we used a “SET1 × SET1” test with set files containing one chromosome SNP set. Due to the limitation of the software setting, covariates such as age and gender were not included in the analysis.

### Stage 2

#### Sample

The dataset used to test our results from Stage 1 included data from an additional 181 NPC cases and 187 controls. Data were collected independently from subjects unrelated to the earlier set but similarly of Han Chinese descent living in Taiwan. Stage 2 data additionally contain information on family history and clinical outcome as follows: 73 cases were resistant to radiotherapy, 33 cases had a family history of NPC, and 18 cases had distal metastasis. Subjects were genotyped using Illumina Human610-Quad BeadChips from the Illumina-certified service provider Genizon Biosciences (Genizon BioSciences, Canada). GWAS analysis was conducted using the same data-processing criteria as specified for Stage 1 above. Quality control criteria included low call rate (<99%), failure on PLINK tests for identity-by-state (IBS) or identity-by-descent (IBD), or failure on the EIGENSOFT package [40] analysis for principal components (PCA).

Nine samples and 14 duplicate samples failed quality control assessments and were eliminated. We also eliminated cases and controls where the SNPs missing data rate was >3% or a minor allele frequency (MAF)<0.1, and controls with a Hardy-Weinberg Equilibrium (HWE) *P* value<0.00005. The quality control process therefore removed 120,113 markers from the original 620,901 markers, leaving 500,788 markers for use in future tests.

#### SNP-SNP interaction analysis

We then pooled all two-locus SNP-SNP interaction results in Stage 1 and ranked them by interaction *P* value. Because a similar genotyping platform was used in both Stages, we could in most cases select exact SNP combinations from the Stage 2 dataset for replication. Using PLINK epistasis analysis, we analyzed the top 10,000 SNP pairs identified from Stage 1 in the Stage 2 dataset.

### Combined Analysis

Stage 1 and Stage 2 data sets contained 464 cases and 478 controls. Following the sample quality control process, samples that failed to pass the call rate (<99%), IBS, IBD, or PCA tests were removed. This left a combined sample with data from 454 cases and 477 controls. We then used this combined dataset to calculate interaction *P* values for the top 10,000 interaction pairs identified in Stage 1.

### Permutation Test

Permutation test was performed 10,000 times using permuted phenotype sets generated by “–make-perm-pheno 10,000” command in PLINK. Pseudo-interaction *P* values were calculated using the permuted phenotypes. Permutation *P* values were calculated for each interaction pair as *P*_permutation_ = (b+1 )/(m+1 ), where b was the number of permutations yielding a pseudo-interaction *P* value at least as extreme as that observed using the original data, and m was the number of permutation tests.

### Haplotype and LD Analysis

To examine the haplotypes of interacting SNPs, we used the R (version 2.13.1) [41]/haplo.stats package (version 1.5.5) [42]. The minimum haplotype frequency was set at 0.01 and missing values were excluded from the analysis. The Haploview package [43] was used to analyze patterns of LD and identify haplotype blocks.

## Supporting Information

Figure S1 Data-processing flowchart. GWAS data were divided into 24 sets according to chromosomal position. PLINK epistasis analysis by pairing two sets on different chromosomes or one set on one chromosome. The 10,000 pairs with the highest interaction scores were then tested in independent GWAS samples, and the 66 most suggestive interaction pairs were selected.
(PDF)

Figure S2 Odds ratios for the top 10 interaction pairs and pairs in the MHC region. X and Y axes show interacting SNP genotypes. Z axis shows SNP pair odds ratios estimated relative to the baseline double-homozygote of major alleles in the combined data set.
(PDF)

Figure S3 Regional signal plots of all SNPs within 100 kb of the suggestive interactive SNP pairs identified. In each figure, the left panel shows the interaction signal heat-map and single-locus signal plots. The heat-map is aligned by chromosome positions based on NCBI build 36. Single-locus signal plots with gene annotations *(lower left and upper-right)*. In the single-locus signal plots, red is the position of suggestive-interacting SNP pairs in the corresponding regions. In the single-locus signal plots, solid black dots denote logistic regression test *P* values. In the interaction-signal heatmap plot, interaction *P* values, transformed by a negative logarithm, are coded by color *(key in upper-left box)*. *Interaction signal heatmap and LD plot (right panel)*. Heatmap is aligned with each SNP equidistant from LD plot positions. Interaction-signal heatmap and LD plots are color coded *(key upper right)*. Single-locus signal plots *(lower left)* same as above, except that coordination is not based on chromosome position. In detailed LD structures estimated in control samples *(upper left and lower right)* increasing intensities of red represent higher D’ values, and solid red triangles denote the positions of potentially interacting pairs of SNPs. Genes annotated in this region are also depicted according to their relative positions on the LD plot as follows: The top 10 interactions regions selected, shown in Table 1 (A to J); the two suggestive interaction regions located in the chromosome 6p21 MHC region (K and L). Because these SNP pairs are located in a small region with a complicated interaction pattern, the same chromosome region is plotted in the 2 axes of the heatmap. On the heatmap, yellow dot is position of suggestive interacting pairs of SNPs in their corresponding regions, green arrow is position of the NPC-susceptibility SNP (rs2894207) identified by a previous GWAS study [5], green box is the copy number variations related to NPC susceptibility in men (L). Plot of regional signals from GWAS-identified NPC-associated SNPs in the HLA region (M).
(PDF)

Figure S4 HapMap3 eQTL analysis between rs1607979 genotype and CREB5 gene expression profile. Genevar [26] analysis of HapMap3 data of lymphoblastoid cell lines collected from unrelated individuals of diverse ethnicity [27]. Total sample (N = 726) includes (CEU = 109) Caucasians from Utah, USA; (CHB = 80) Han Chinese from Beijing, China; (GIH = 82) Gujarati Indians from Houston, TX, USA; (JPT = 82) Japanese from Tokyo, Japan; (LWK = 82) Luhya in Webuye, Kenya; (MEX = 45) Mexican ancestry from Los Angeles, CA, USA; (MKK = 138) Maasai from Kinyawa, Kenya; and (YRI = 108) Yoruba from Ibadan, Nigeria. We performed Spearman’s rank correlation coefficient (rho) to estimate the strength of relationship between alleles and gene expression intensities and used linear regression to model the relationship between the two variables. A t-statistic with n−2 degrees of freedom was used to test the significance of the relationship in both correlation and regression analyses.
(PDF)

Table S1 Top 10 000 SNP pairs identified in Stage 1 analysis ranked by interaction significance.
(PDF)

Table S2 Suggestive interactions associated with NPC susceptibility.
(PDF)

Table S3 Suggestive regions associated with NPC susceptibility.
(PDF)

Table S4 LD analysis of SNPs in interacting region.
(PDF)

Table S5 Haplotype analysis of the interaction regions located in the MHC region.
(PDF)

Table S6 Interaction P values for SNP pairs identified by other GWAS studies.
(PDF)
