# Supplementary material for: How Genome-Wide SNP-SNP Interactions Relate to Nasopharyngeal Carcinoma Susceptibility
Source: PLoS One. 2013 Dec 23;8(12):e83034. doi: 10.1371/journal.pone.0083034 (PMC3871583; doi:10.1371/journal.pone.0083034)
Supplement: Figure S1 — Data-processing flowchart. GWAS data were divided into 24 sets according to chromosomal position. PLINK epistasis analysis by pairing two sets on different chromosomes or one set on one chromosome. The 10,000 pairs with the highest interaction scores were then tested in independent GWAS samples, and the 66 most suggestive interaction pairs were selected. (PDF) [file pone.0083034.s001.pdf]

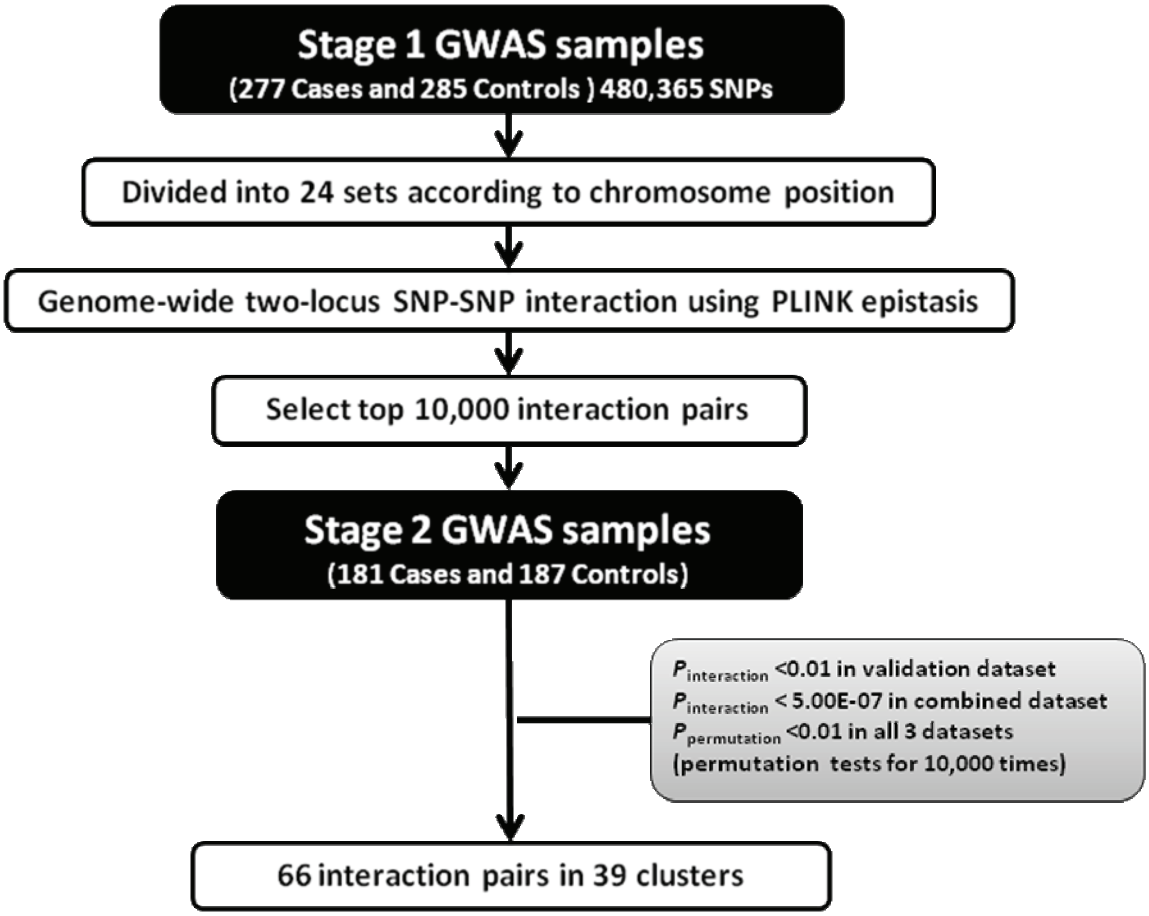

Figure S1. Data-processing flowchart. GWAS data were divided into 24 sets according to chromosomal position. PLINK epistasis analysis by pairing two sets on different chromosomes or one set on one chromosome. The 10,000 pairs with the highest interaction scores were then tested in independent GWAS samples, and the 66 most significant interaction pairs were selected.
