# Supplementary material for: How Genome-Wide SNP-SNP Interactions Relate to Nasopharyngeal Carcinoma Susceptibility
Source: PLoS One. 2013 Dec 23;8(12):e83034. doi: 10.1371/journal.pone.0083034 (PMC3871583; doi:10.1371/journal.pone.0083034)
Supplement: Figure S2 — Odds ratios for the top 10 interaction pairs and pairs in the MHC region. X and Y axes show interacting SNP genotypes. Z axis shows SNP pair odds ratios estimated relative to the baseline double-homozygote of major alleles in the combined data set. (PDF) [file pone.0083034.s002.pdf]

A 7q15-17q22

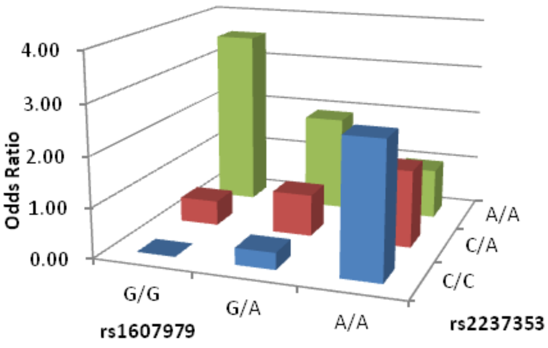

		rs1607979		
		G/G	G/A	A/A
rs2237353	C/C	NA	0.33(0.07, 1.08)	2.68(1.44, 5.16)
	C/A	0.50(0.17, 1.26)	0.84(0.55, 1.29)	1.55(1.07, 2.23)
	A/A	3.57(1.69, 8.10)	1.92(1.33, 2.79)	1.00

B 7q11-9q33

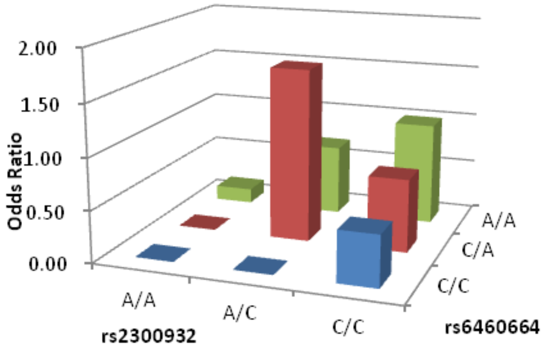

		rs2300932		
		A/A	A/C	C/C
rs6460664	C/C	NA	NA	0.50(0.19, 1.18)
	C/A	NA	1.68(1.04, 2.77)	0.71(0.51, 0.99)
	A/A	0.144(0.03, 0.43)	0.69(0.48, 0.98)	1.00

C 4q26-10q26

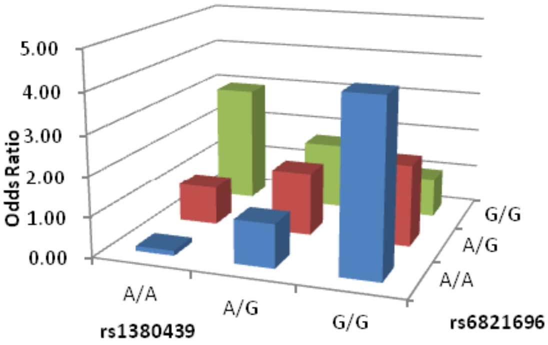

		rs1380439		
		A/A	A/G	G/G
rs6821696	A/A	0.12(0.01, 0.63)	1.07(0.55, 2.06)	4.28(1.75, 11.68)
	A/G	0.97(0.54, 1.72)	1.59(1.03, 2.46)	2.04(1.27, 3.32)
	G/G	2.97(1.70, 5.28.)	1.68(1.11, 2.57)	1.00

D 2q36-21q22

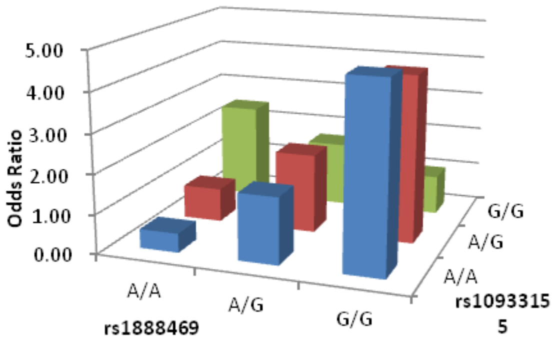

		rs1888469		
		A/A	A/G	G/G
rs10933155	A/A	0.48(0.06, 2.07)	1.69(0.70, 4.01)	4.69(1.26, 23.35)
	A/G	0.86(0.47, 1.55)	2.02 (1.30, 3.16)	4.25(2.43, 7.62)
	G/G	2.45 (1.52, 4.00)	1.65(1.10, 2.51)	1.00

E 7p21-7q31

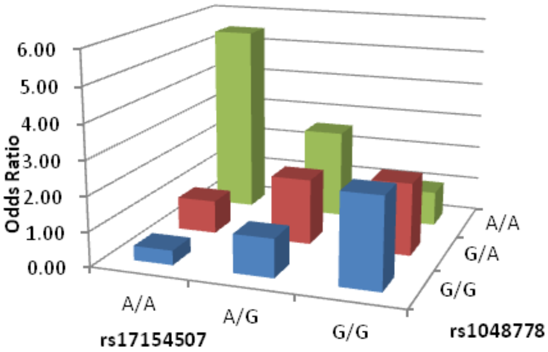

		rs17154507		
		A/A	A/G	G/G
rs10487781	G/G	0.42(0.09, 1.38)	1.11(0.61, 1.98)	2.60(1.45, 4.74)
	G/A	0.96(0.49, 1.85)	1.90(1.22, 3.00)	2.09(1.33, 3.33)
	A/A	5.49(2.45, 13.41)	2.57(1.58, 4.22)	1.00

F 9p21-16p13

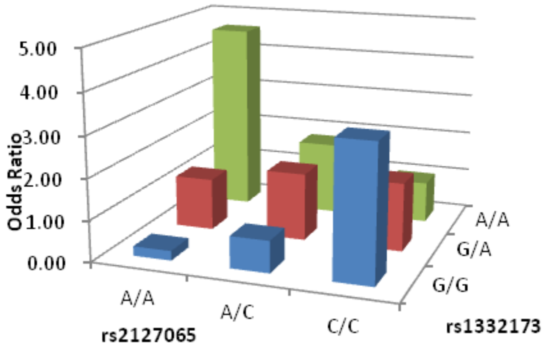

		rs2127065		
		A/A	A/C	C/C
rs1332173	G/G	0.23(0.01, 1.35)	0.77(0.41, 1.44)	3.28(1.79, 6.15)
	G/A	1.28(0.69, 2.34)	1.65(1.06, 2.59)	1.67(1.08, 2.59)
	A/A	4.62(2.25, 10.05)	1.78(1.13, 2.83)	1.00

G 2p23-9q21

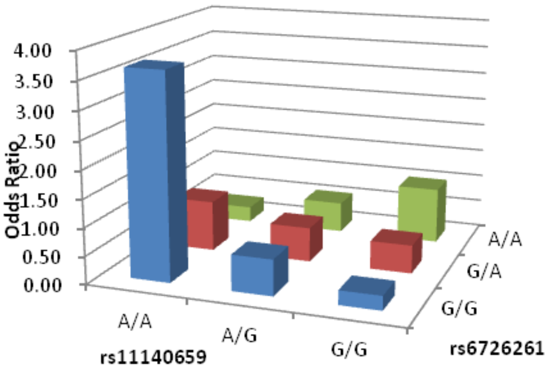

		rs11140659		
		A/A	A/G	G/G
rs6726261	G/G	3.66(1.16, 16.90)	0.64(0.35, 1.17)	0.25(0.10, 0.58)
	G/A	0.91(0.49, 1.70)	0.62(0.40, 0.95)	0.51 (0.31, 0.82)
	A/A	0.28(0.15, 0.50)	0.55(0.36, 0.85)	1.00

H 2p16-2p16

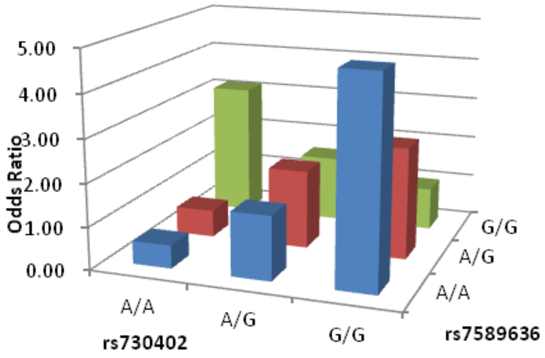

		rs730402		
		A/A	A/G	G/G
rs7589636	A/A	0.54(0.19, 1.37)	1.50 (0.83, 2.73)	4.80(2.32, 10.38)
	A/G	0.64(0.31, 1.31)	1.84(1.11, 3.12)	2.61(1.52, 4.54)
	G/G	3.13 (1.58, 6.34)	1.55(0.90, 2.71)	1.00

I 1p34-7q36

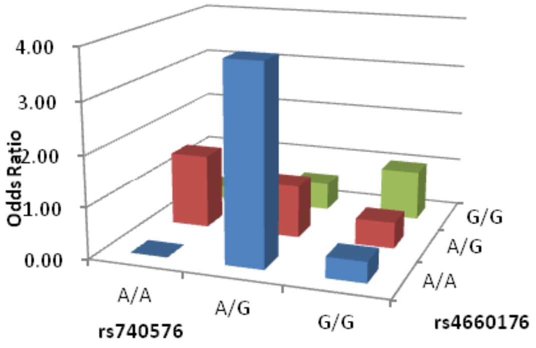

		rs740576		
		A/A	A/G	G/G
rs4660176	A/A	NA	3.85(0.99, 27.57)	0.40(0.20, 0.78)
	A/G	1.47(0.55, 4.41)	1.04(0.68, 1.58)	0.51(0.3, 0.73)
	G/G	0.25(0.09, 0.63)	0.55(0.38, 0.79)	1.00

J 5p15-10q21

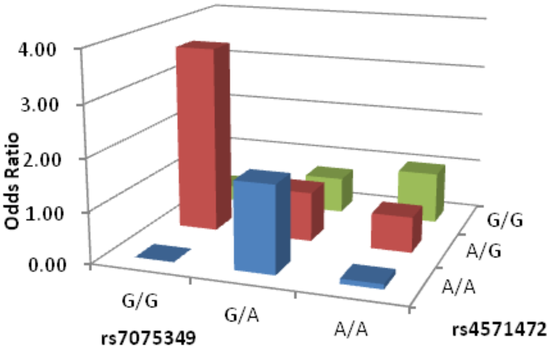

		rs7075349		
		G/G	G/A	A/A
rs4571472	A/A	NA	1.69(0.58, 5.68)	0.10(0.00, 0.53)
	A/G	3.64(1.54, 10.16)	0.97(0.63, 1.48)	0.70(0.46, 1.04)
	G/G	0.30(0.16, 0.55)	0.7(0.50, 0.99)	1.00

K 6p21-6p21 (I)

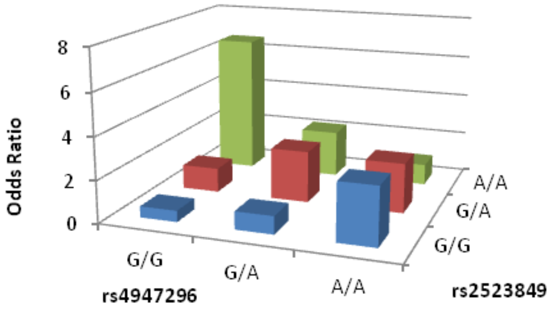

		rs4947296		
		G/G	G/A	A/A
rs2523849	G/G	0.53 (0.02, 3.92)	0.85(0.37, 1.82)	2.74(1.49, 5.15)
	G/A	1.17 (0.47, 2.75	2.47 (1.60, 3.82)	2.36(1.62, 3.45)
	A/A	6.65 (3.24, 14.85)	2.23(1.51, 3.32)	1.00

L 6p21-6p21 (II)

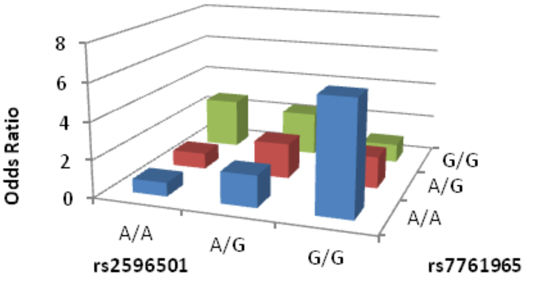

		rs2596501		
		A/A	A/G	G/G
rs7761965	A/A	0.74 (0.24, 1.96)	1.69 (0.92, 3.14)	6.04 (2.82,13.99)
	A/G	0.86 (0.48, 1.52)	1.91 (1.23, 2.99)	1.70 (1.05, 2.79)
	G/G	2.70 (1.45, 5.11)	2.36 (1.48, 3.78)	1.00

Figure S2. Odds ratios for the top 10 interaction pairs and pairs in the MHC region. X and Y axes show interacting SNP genotypes. Z axis shows SNP pair odds ratios estimated relative to the baseline double-homozygote of major alleles in the combined data set.
