# Supplementary material for: How Genome-Wide SNP-SNP Interactions Relate to Nasopharyngeal Carcinoma Susceptibility
Source: PLoS One. 2013 Dec 23;8(12):e83034. doi: 10.1371/journal.pone.0083034 (PMC3871583; doi:10.1371/journal.pone.0083034)
Supplement: Figure S3 — Regional signal plots of all SNPs within 100 kb of the suggestive interactive SNP pairs identified. In each figure, the left panel shows the interaction signal heat-map and single-locus signal plots. The heat-map is aligned by chromosome positions based on NCBI build 36. Single-locus signal plots with gene annotations (lower left and upper-right). In the single-locus signal plots, red is the position of suggestive-interacting SNP pairs in the corresponding regions. In the single-locus signal plots, solid black dots denote logistic regression test P values. In the interaction-signal heatmap plot, interaction P values, transformed by a negative logarithm, are coded by color (key in upper-left box). Interaction signal heatmap and LD plot (right panel). Heatmap is aligned with each SNP equidistant from LD plot positions. Interaction-signal heatmap and LD plots are color coded (key upper right). Single-locus signal plots (lower left) same as above, except that coordination is not based on chromosome position. In detailed LD structures estimated in control samples (upper left and lower right) increasing intensities of red represent higher D’ values, and solid red triangles denote the positions of potentially interacting pairs of SNPs. Genes annotated in this region are also depicted according to their relative positions on the LD plot as follows: The top 10 interactions regions selected, shown in Table 1 (A to J); the two suggestive interaction regions located in the chromosome 6p21 MHC region (K and L). Because these SNP pairs are located in a small region with a complicated interaction pattern, the same chromosome region is plotted in the 2 axes of the heatmap. On the heatmap, yellow dot is position of suggestive interacting pairs of SNPs in their corresponding regions, green arrow is position of the NPC-susceptibility SNP (rs2894207) identified by a previous GWAS study [5], green box is the copy number variations related to NPC susceptibility in men (L). Plot of region [file pone.0083034.s003.pdf]

A 7p15 – 17q22

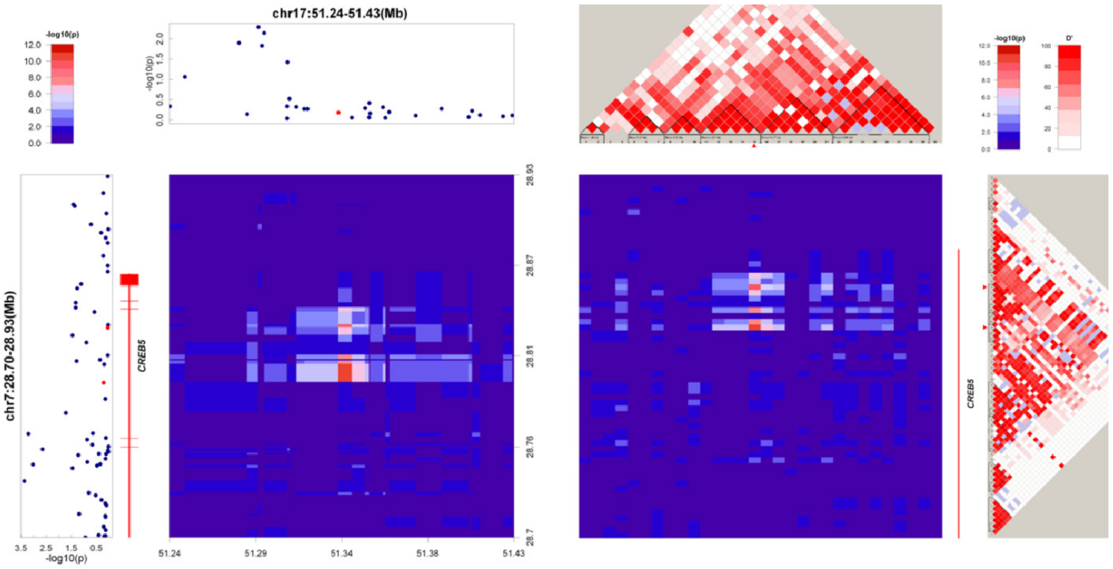

B 7q11 – 9q33

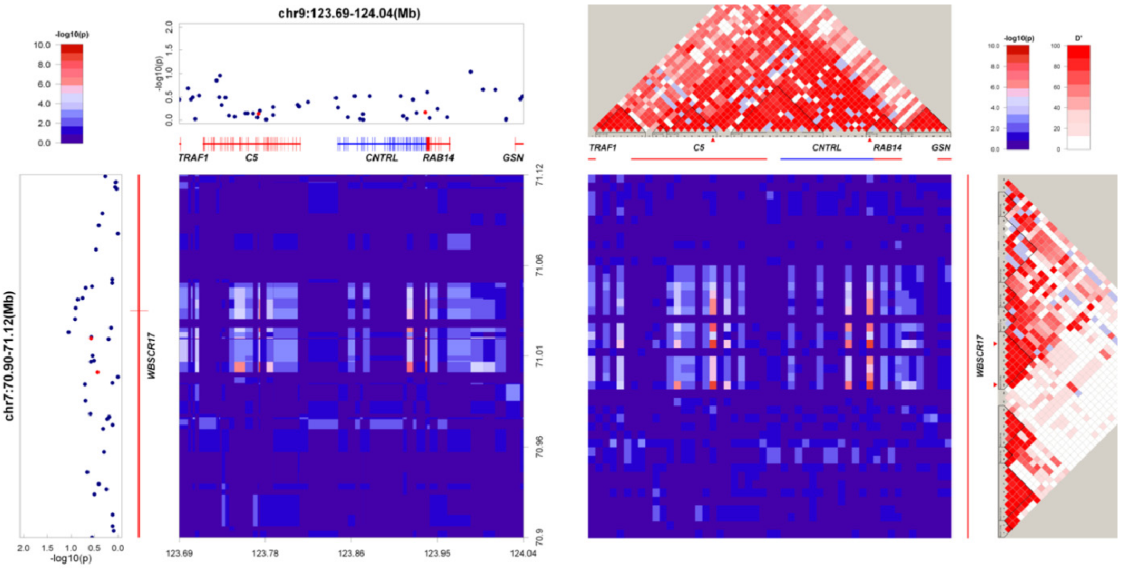

C 4q26 – 10q26

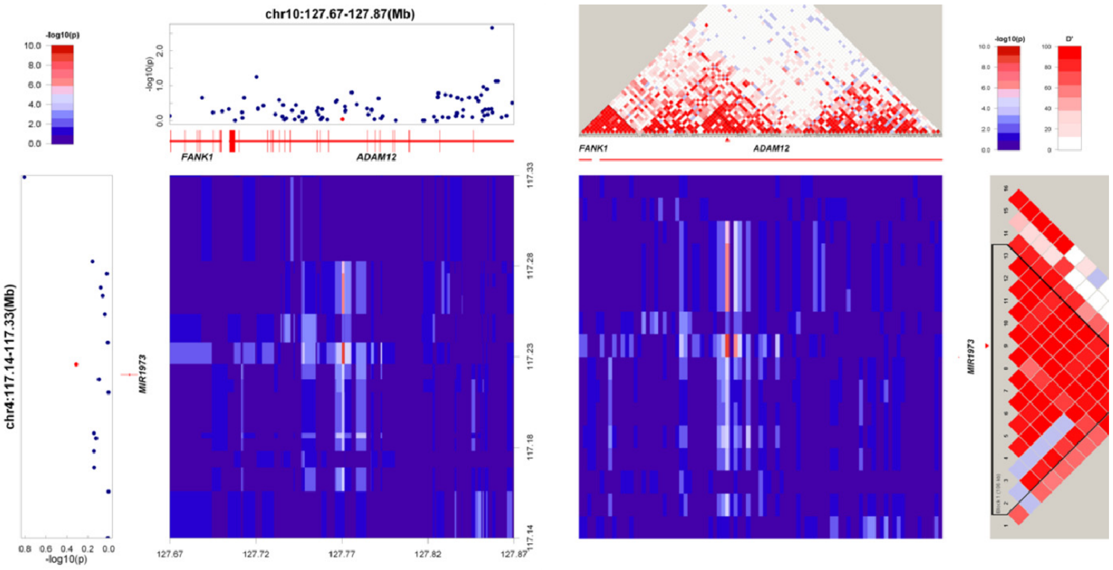

D **2q36 – 21q22**

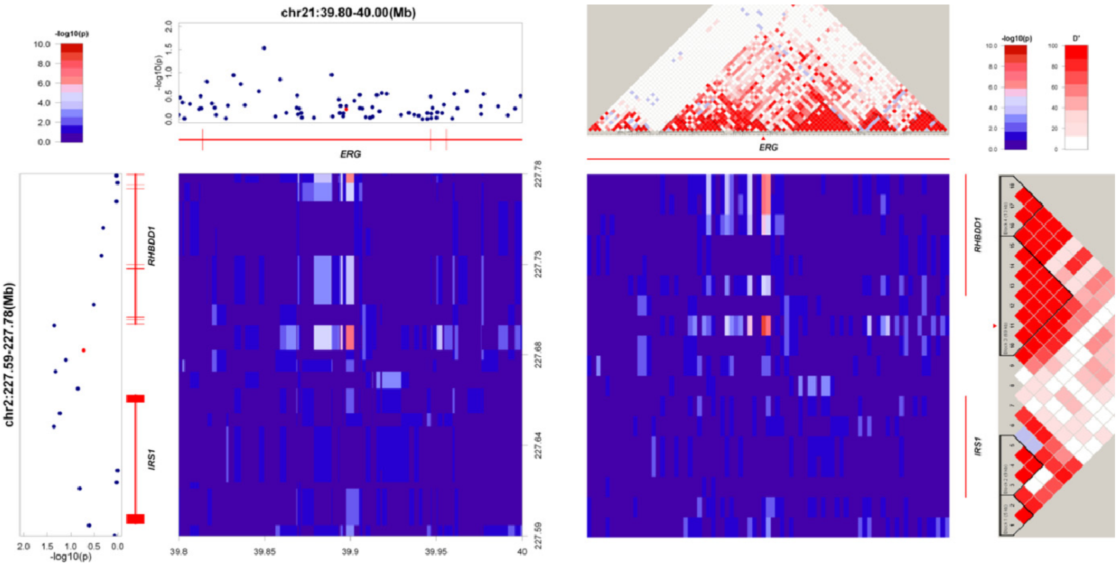

E **7p21 – 7q31**

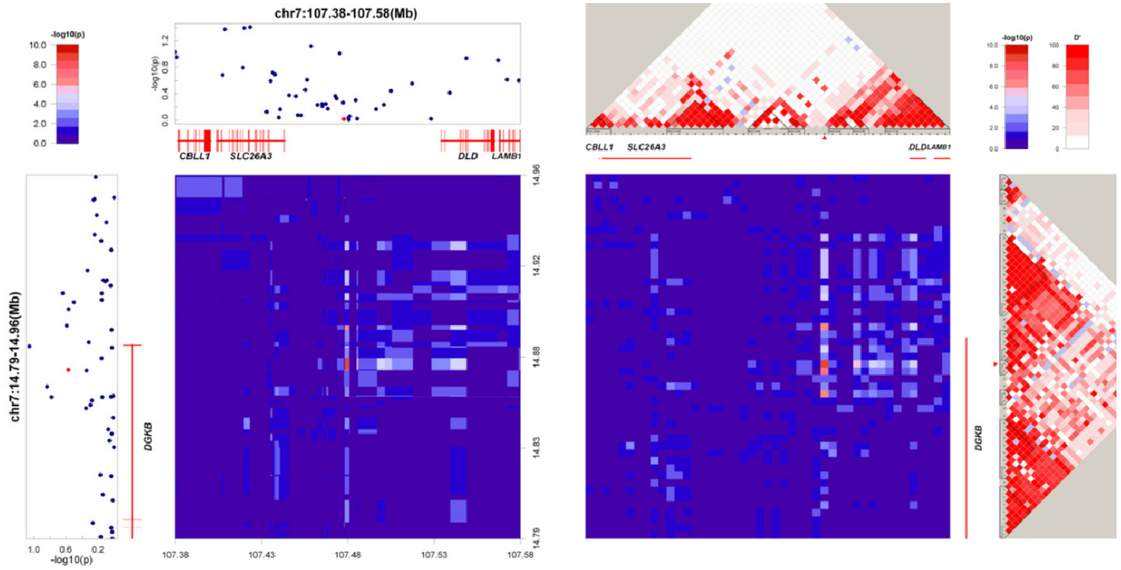

F **9p21 – 16p13**

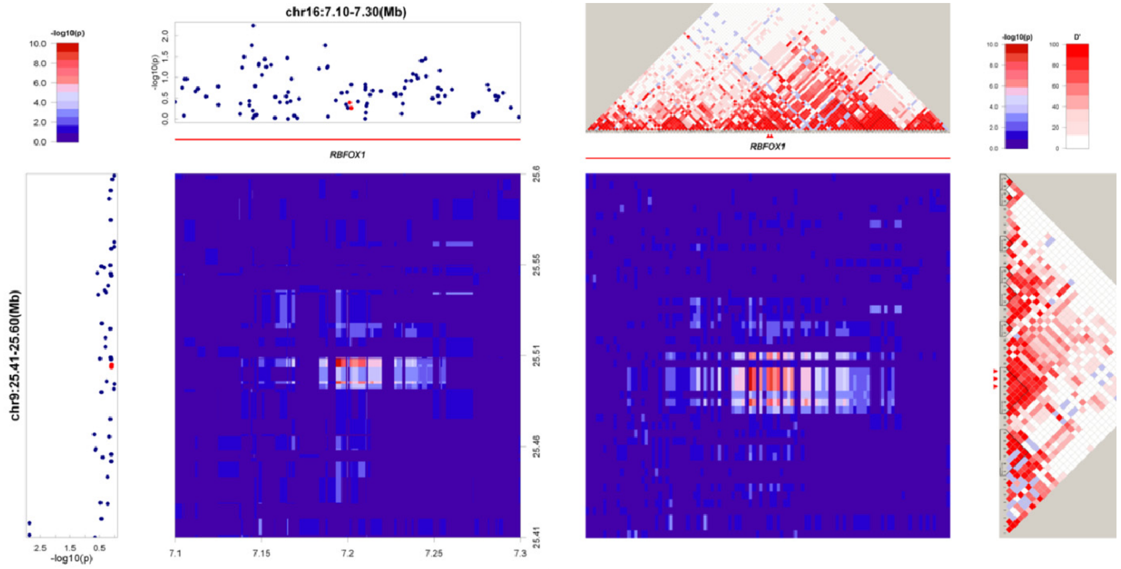

G 2p23 – 9q21

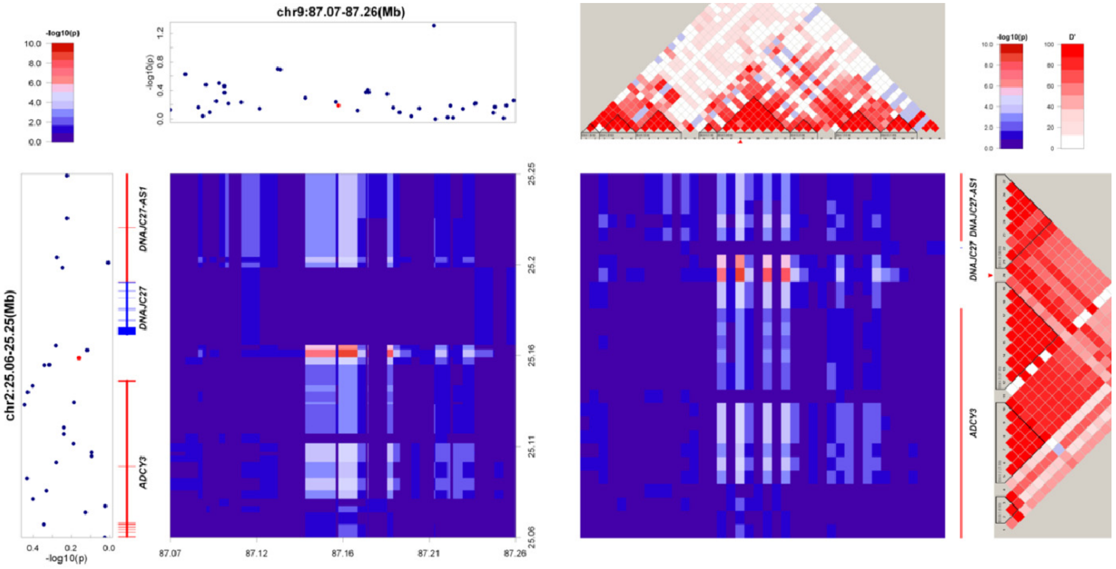

H 2p16 – 2p16

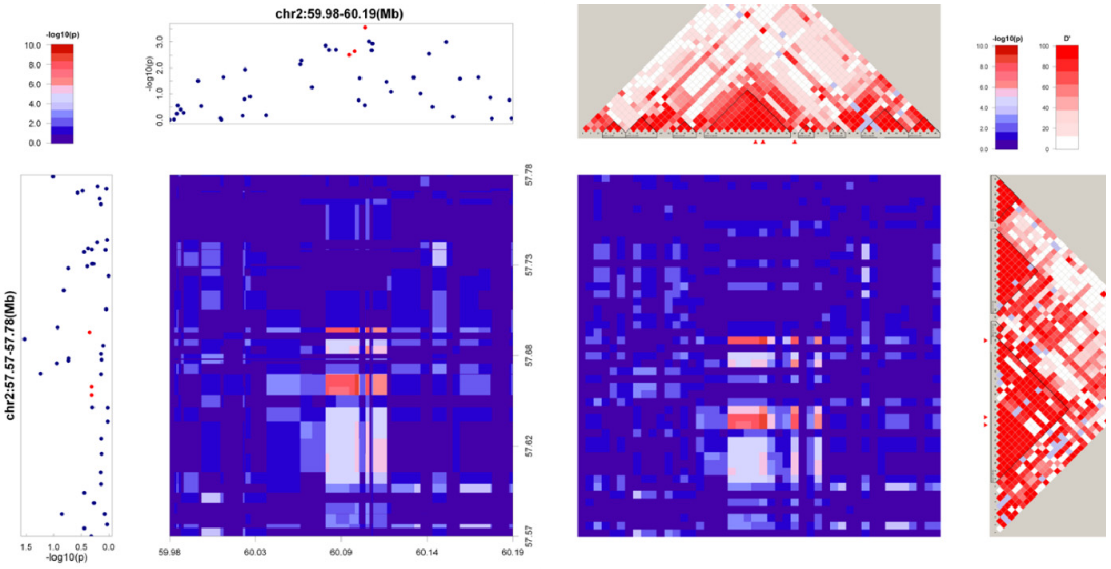

I 1p34 – 7q36

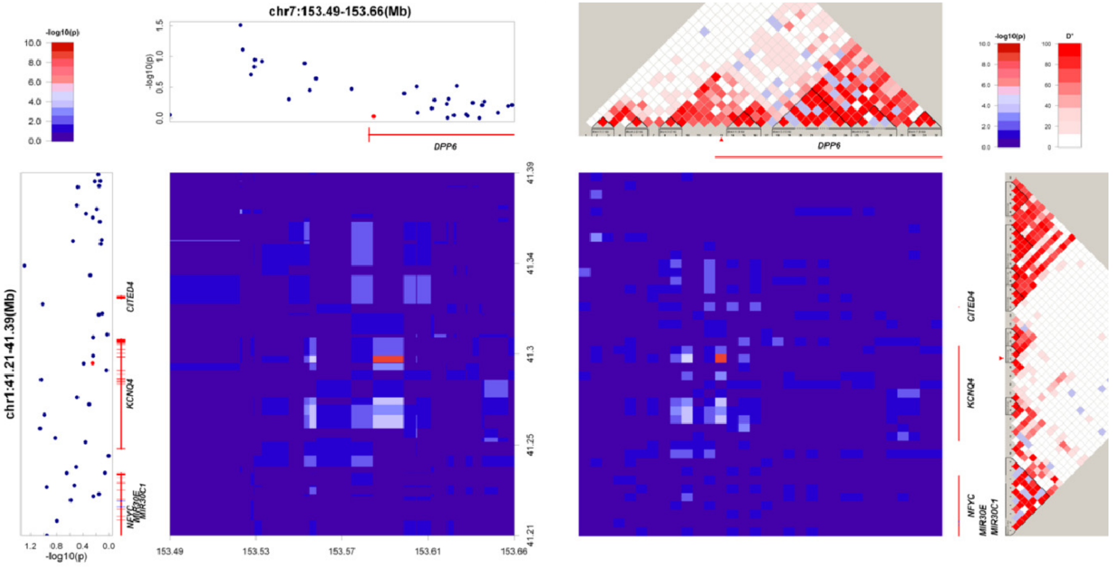

J 5p15 – 10q21

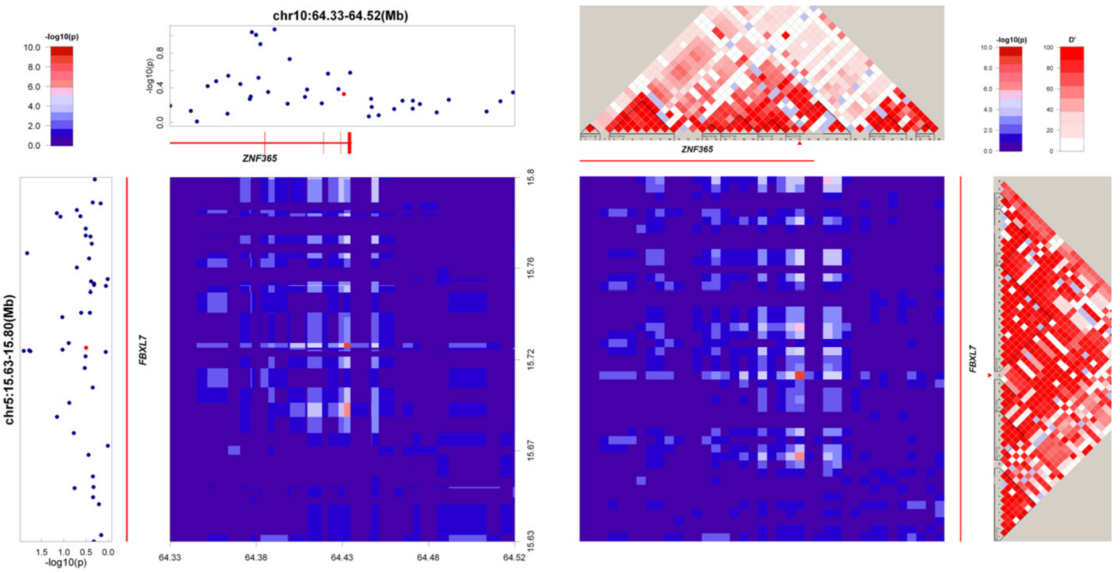

K 6p21 – 6p21 (I)

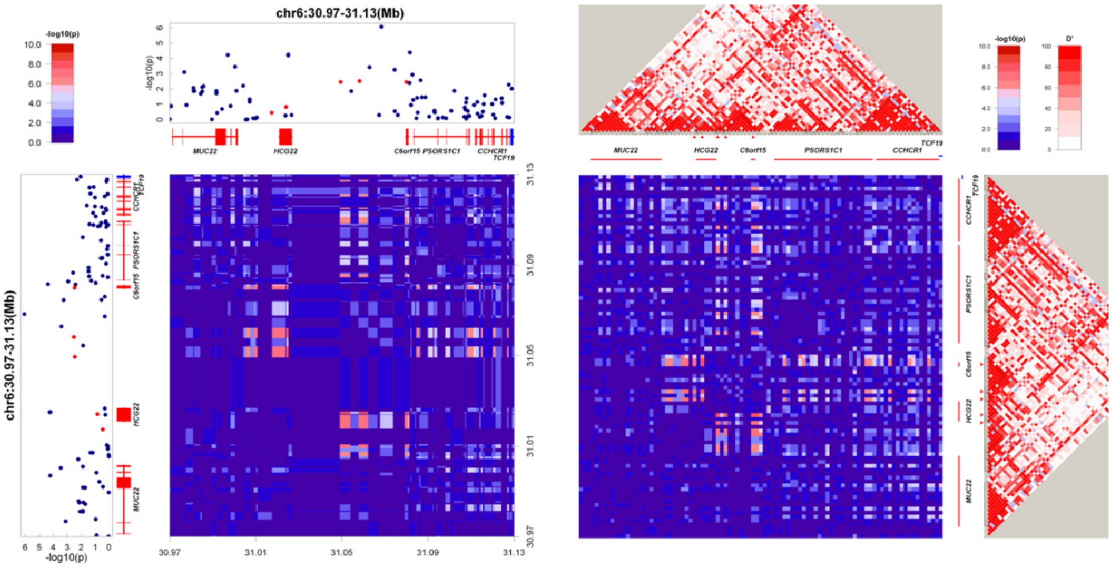

L 6p21 – 6p21 (II)

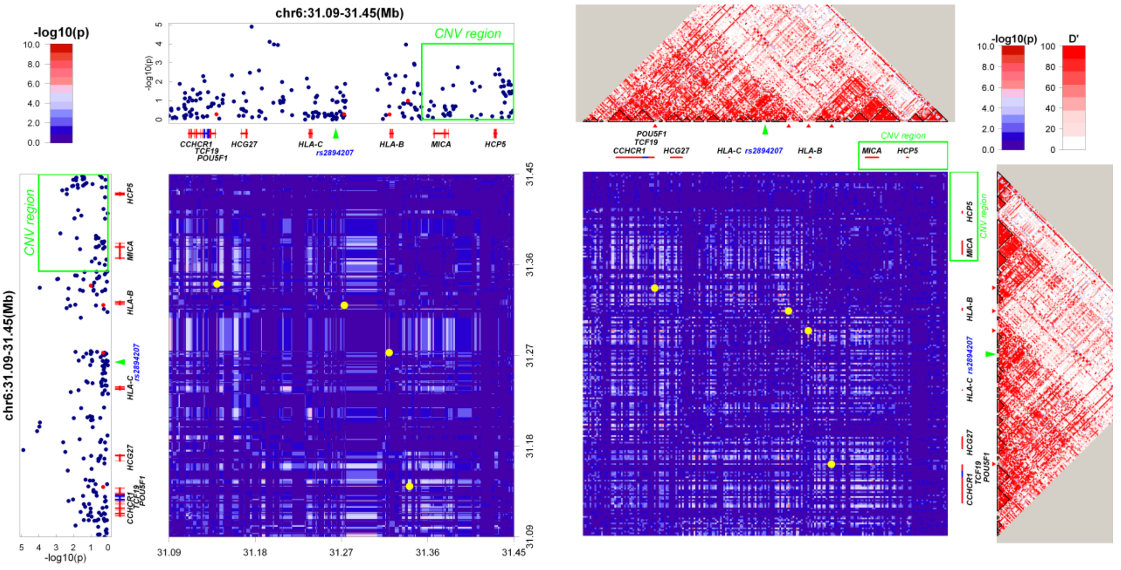

M GWAS SNPs

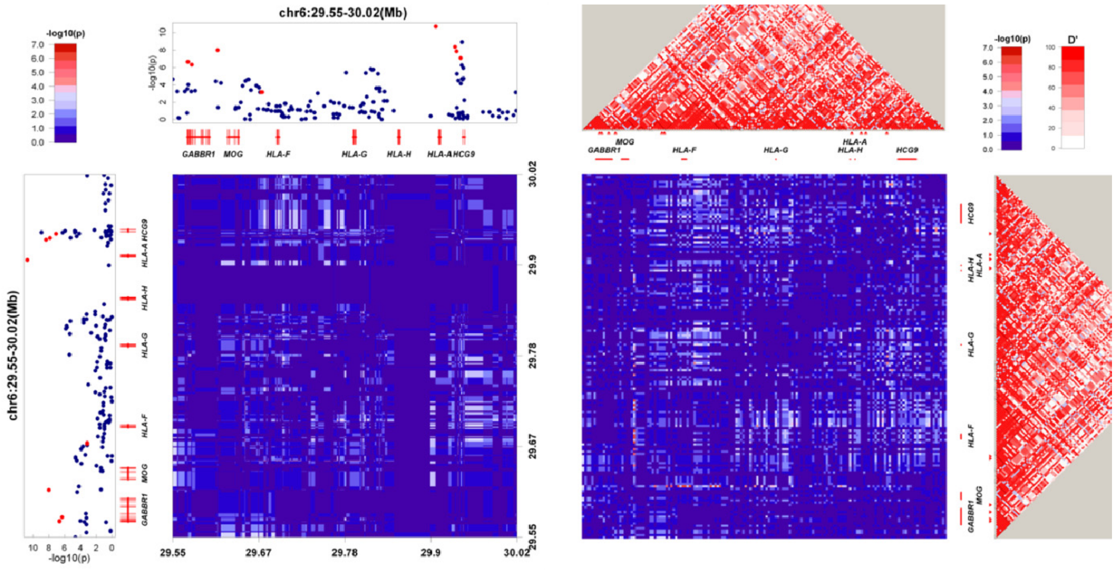

Figure S3. Regional signal plots of all SNPs within 100 kb of the most significant interactive SNP pairs identified. In each figure, the left panel shows the interaction signal heat-map and single-locus signal plots. The heat-map is aligned by chromosome positions based on NCBI build 36. Single-locus signal plots with gene annotations *(lower left and upper-right)*. In the single-locus signal plots, red is the position of significant-interacting SNP pairs in the corresponding regions. In the single-locus signal plots, solid black dots denote logistic regression test *P* values. In the interaction-signal heatmap plot, interaction *P* values, transformed by a negative logarithm, are coded by color *(key in upper-left box)*.

Interaction signal heatmap and LD plot (right panel). Heatmap is aligned with each SNP equidistant from LD plot positions. Interaction-signal heatmap and LD plots are color coded *(key upper right)*. Single-locus signal plots *(lower left)* same as above, except that coordination is not based on chromosome position. In detailed LD structures estimated in control samples *(upper left and lower right)* increasing intensities of red represent higher D' values, and solid red triangles denote the positions of potentially interacting pairs of SNPs. Genes annotated in this region are also depicted according to their relative positions on the LD plot as follows: The top 10 interactions regions selected, shown in Table 1 (A to J); the two most-significant interaction regions located in the chromosome 6p21 MHC region (K and L). Because these SNP pairs are located in a small region with a complicated interaction pattern, the same chromosome region is plotted in the 2 axes of the heatmap. On the heatmap, yellow dot is position of significant interacting pairs of SNPs in their corresponding regions, green arrow is position of the NPC-susceptibility SNP (rs2894207) identified by a previous GWAS study [1], green box is the copy number variations related to NPC susceptibility in men (L). Plot of regional signals from GWAS-identified NPC-associated SNPs in the HLA region (M).

1. Bei JX, Li Y, Jia WH, Feng BJ, Zhou G, et al. (2010) A genome-wide association study of nasopharyngeal carcinoma identifies three new susceptibility loci. Nat Genet 42: 599-603.
