# Supplementary material for: How Genome-Wide SNP-SNP Interactions Relate to Nasopharyngeal Carcinoma Susceptibility
Source: PLoS One. 2013 Dec 23;8(12):e83034. doi: 10.1371/journal.pone.0083034 (PMC3871583; doi:10.1371/journal.pone.0083034)
Supplement: Figure S4 — HapMap3 eQTL analysis between rs1607979 genotype and CREB5 gene expression profile. Genevar [26] analysis of HapMap3 data of lymphoblastoid cell lines collected from unrelated individuals of diverse ethnicity [27]. Total sample (N = 726) includes (CEU = 109) Caucasians from Utah, USA; (CHB = 80) Han Chinese from Beijing, China; (GIH = 82) Gujarati Indians from Houston, TX, USA; (JPT = 82) Japanese from Tokyo, Japan; (LWK = 82) Luhya in Webuye, Kenya; (MEX = 45) Mexican ancestry from Los Angeles, CA, USA; (MKK = 138) Maasai from Kinyawa, Kenya; and (YRI = 108) Yoruba from Ibadan, Nigeria. We performed Spearman’s rank correlation coefficient (rho) to estimate the strength of relationship between alleles and gene expression intensities and used linear regression to model the relationship between the two variables. A t-statistic with n−2 degrees of freedom was used to test the significance of the relationship in both correlation and regression analyses. (PDF) [file pone.0083034.s004.pdf]

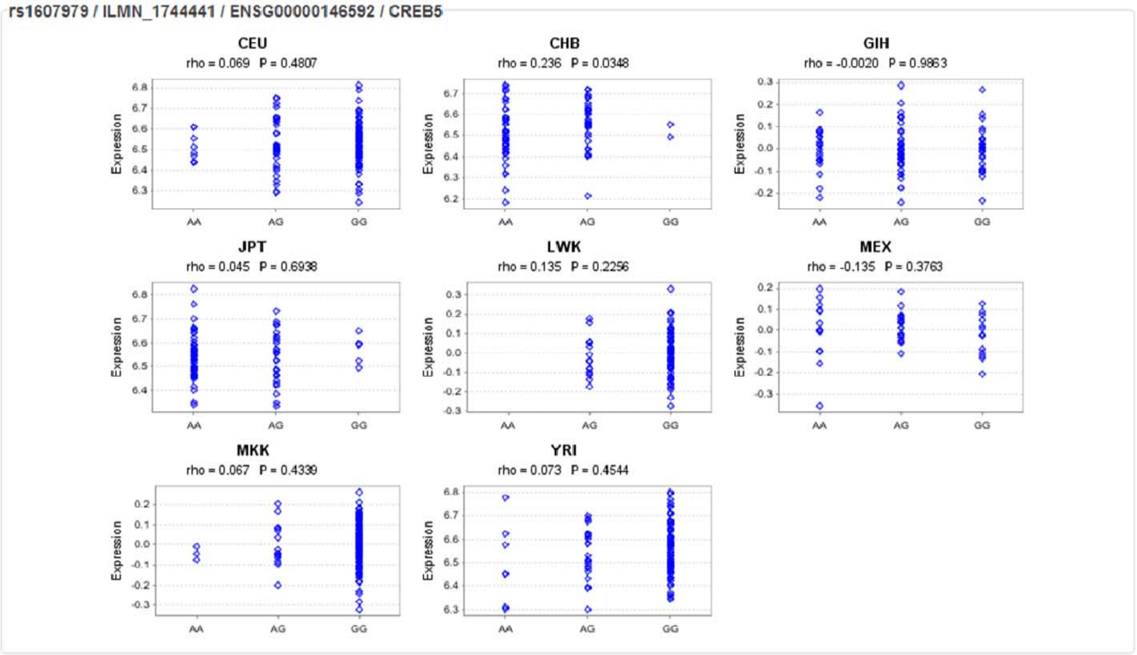

Figure S4. HapMap3 eQTL analysis between rs1607979 genotype and CREB5 gene expression profile. Genevar [1] analysis of HapMap3 data of lymphoblastoid cell lines collected from unrelated individuals of diverse ethnicity [2]. Total sample (N=726) includes (CEU=109) Caucasians from Utah, USA; (CHB=80) Han Chinese from Beijing, China; (GIH=82) Gujarati Indians from Houston, TX, USA; (JPT=82) Japanese from Tokyo, Japan; (LWK=82) Luhya in Webuye, Kenya; (MEX=45) Mexican ancestry from Los Angeles, CA, USA; (MKK=138) Maasai from Kinyawa, Kenya; and (YRI=108) Yoruba from Ibadan, Nigeria. We performed Spearman's rank correlation coefficient (rho) to estimate the strength of relationship between alleles and gene expression intensities and used linear regression to model the relationship between the two variables. A t-statistic with n−2 degrees of freedom was used to test the significance of the relationship in both correlation and regression analyses.

References

1. Yang TP, Beazley C, Montgomery SB, Dimas AS, Gutierrez-Arcelus M, et al. (2010) Genevar: a database and Java application for the analysis and visualization of SNP-gene associations in eQTL studies. Bioinformatics 26: 2474-2476.
2. Stranger BE, Montgomery SB, Dimas AS, Parts L, Stegle O, et al. (2012) Patterns of cis regulatory variation in diverse human populations. PLoS Genet 8: e1002639.
