# Supplementary material for: How Genome-Wide SNP-SNP Interactions Relate to Nasopharyngeal Carcinoma Susceptibility
Source: PLoS One. 2013 Dec 23;8(12):e83034. doi: 10.1371/journal.pone.0083034 (PMC3871583; doi:10.1371/journal.pone.0083034)
Supplement: Table S1 — Top 10 000 SNP pairs identified in Stage 1 analysis ranked by interaction significance. (PDF) [file pone.0083034.s005.pdf]

Supplementary Table S1. The top 10 000 interaction pairs from the discovery data set

SNP A					SNP B						Interaction P			Ranking	Cluster in top 100 interactions
CHR	SNP	Location	gle locus P va	Gene	CHR	SNP	Location	Single locus P valu	Gene	MHC region	Stage 1	Stage 2	Combined		
13	rs17233815	92156875	7.20E-01	GPC5	18	rs10871618	63228905	1.89E-01	N/A	-	1.97E-10	6.96E-01	1.68E-06	1	
6	rs9368675	31380136	7.91E-01	N/A	6	rs2253907	31444849	7.98E-01	HLA-B	MHC	4.91E-10	1.57E-01	2.28E-08	2	1
6	rs7750269	31379136	8.58E-01	N/A	6	rs2253907	31444849	7.98E-01	HLA-B	MHC	6.77E-10	1.57E-01	3.02E-08	3	1
6	rs9368675	31380136	7.91E-01	N/A	6	rs7743761	31444079	8.81E-01	HLA-B	MHC	1.44E-09	1.55E-01	4.26E-08	4	1
6	rs7761965	31381474	9.15E-01	N/A	6	rs2253907	31444849	7.98E-01	HLA-B	MHC	1.67E-09	5.78E-02	7.98E-09	5	1
6	rs887464	31253899	8.59E-02	TCF19	6	rs3130467	31295054	5.92E-02	HCG27	MHC	1.69E-09	3.29E-01	2.27E-07	6	1
6	rs7750269	31379136	8.58E-01	N/A	6	rs7743761	31444079	8.81E-01	HLA-B	MHC	1.95E-09	1.55E-01	5.53E-08	7	1
6	rs396243	31383153	2.17E-01	N/A	6	rs7743761	31444079	8.81E-01	HLA-B	MHC	1.97E-09	2.77E-01	1.20E-07	8	1
8	rs4907391	1.43E+08	7.74E-01	N/A	11	rs10895575	1.03E+08	6.08E-01	PDGFD	-	2.01E-09	5.74E-01	2.07E-05	9	2
8	rs4907391	1.43E+08	7.74E-01	N/A	11	rs11226161	1.03E+08	6.08E-01	PDGFD	-	2.01E-09	5.74E-01	2.07E-05	10	2
8	rs4907391	1.43E+08	7.74E-01	N/A	11	rs11226163	1.03E+08	5.86E-01	PDGFD	-	2.20E-09	5.74E-01	2.18E-05	11	2
6	rs9348862	31360726	7.72E-01	HLA-C	6	rs2253907	31444849	7.98E-01	HLA-B	MHC	2.24E-09	3.37E-01	4.19E-07	12	1
6	rs9357122	31368324	7.72E-01	N/A	6	rs2253907	31444849	7.98E-01	HLA-B	MHC	2.24E-09	3.37E-01	4.19E-07	13	1
6	rs9348862	31360726	7.72E-01	HLA-C	6	rs7743761	31444079	8.81E-01	HLA-B	MHC	2.30E-09	8.81E-02	3.06E-08	14	1
6	rs9357122	31368324	7.72E-01	N/A	6	rs7743761	31444079	8.81E-01	HLA-B	MHC	2.30E-09	8.81E-02	3.06E-08	15	1
2	rs17013592	77088275	7.17E-01	LRRTM4	5	rs468828	3205858	8.16E-01	N/A	-	2.52E-09	4.04E-01	2.69E-05	16	
6	rs7768444	825234	5.45E-01	N/A	1	rs11207426	59458507	2.71E-01	N/A	-	2.74E-09	8.73E-01	1.50E-06	17	3
6	rs3868542	31253818	6.85E-02	TCF19	6	rs3130467	31295054	5.92E-02	HCG27	MHC	2.86E-09	5.17E-01	1.01E-06	18	1
8	rs868480	1.43E+08	6.79E-01	N/A	11	rs10895575	1.03E+08	6.08E-01	PDGFD	-	2.92E-09	5.74E-01	3.01E-05	19	2
8	rs868480	1.43E+08	6.79E-01	N/A	11	rs11226161	1.03E+08	6.08E-01	PDGFD	-	2.92E-09	5.74E-01	3.01E-05	20	2
10	rs10828047	20716201	2.54E-01	N/A	21	rs2070413	31424285	5.67E-01	TIAM1	-	3.10E-09	N/A	N/A	21	
5	rs307177	6140348	3.52E-01	N/A	5	rs6868098	1.54E+08	8.15E-01	LARP1	-	3.11E-09	N/A	N/A	22	4
5	rs307177	6140348	3.52E-01	N/A	5	rs10039709	1.54E+08	8.14E-01	LARP1	-	3.14E-09	N/A	N/A	23	4
8	rs868480	1.43E+08	6.79E-01	N/A	11	rs11226163	1.03E+08	5.86E-01	PDGFD	-	3.20E-09	5.74E-01	3.18E-05	24	2
6	rs7761965	31381474	9.15E-01	N/A	6	rs7743761	31444079	8.81E-01	HLA-B	MHC	3.32E-09	8.18E-02	1.91E-08	25	1
5	rs4348193	77671476	5.91E-01	N/A	11	rs826044	73445145	5.08E-01	C2CD3	-	3.56E-09	9.04E-01	5.97E-06	26	5
9	rs11791889	35850942	3.60E-01	TMEM8B	14	rs2144039	22799242	2.41E-01	HOMEZ	-	3.74E-09	9.15E-01	1.18E-05	27	6
5	rs1435194	1.05E+08	1.04E-01	N/A	12	rs4763075	61962124	4.02E-01	N/A	-	3.92E-09	7.62E-01	6.30E-06	28	7
3	rs4684717	10598391	7.53E-01	N/A	14	rs2075493	21701518	7.45E-02	N/A	-	4.00E-09	9.63E-01	1.39E-05	29	8
9	rs10814287	35850483	3.37E-01	TMEM8B	14	rs2144039	22799242	2.41E-01	HOMEZ	-	4.20E-09	8.53E-01	1.56E-05	30	6
3	rs1378661	1.62E+08	8.01E-01	N/A	20	rs858140	24009345	2.02E-01	N/A	-	4.29E-09	5.81E-01	2.34E-07	31	
3	rs10935384	1.42E+08	7.96E-01	CLSTN2	16	rs17680065	19988272	8.62E-02	GPR139	-	4.30E-09	7.45E-01	3.75E-06	32	
1	rs10919242	1.68E+08	8.00E-01	C1orf156	12	rs7980095	17131098	7.26E-02	N/A	-	4.39E-09	4.51E-02	9.05E-04	33	9
1	rs12035144	1.68E+08	8.00E-01	C1orf156	12	rs7980095	17131098	7.26E-02	N/A	-	4.39E-09	5.24E-02	7.96E-04	34	9
18	rs12961809	55117108	8.71E-01	CPLX4	20	rs6061928	59971673	4.29E-01	TAF4	-	4.39E-09	4.28E-01	7.16E-08	35	
5	rs4348193	77671476	5.91E-01	N/A	11	rs11235995	73484091	3.56E-01	C2CD3	-	4.46E-09	9.54E-01	4.68E-06	36	5
5	rs4348193	77671476	5.91E-01	N/A	11	rs7125032	73484690	3.56E-01	C2CD3	-	4.46E-09	9.54E-01	4.68E-06	37	5
5	rs4348193	77671476	5.91E-01	N/A	11	rs7110734	73530035	3.56E-01	C2CD3	-	4.46E-09	9.86E-01	3.76E-06	38	5
1	rs12041287	1.65E+08	2.21E-01	GPA33	12	rs2632226	75535673	3.99E-01	N/A	-	4.67E-09	7.64E-01	1.88E-06	39	10
1	rs4656520	1.65E+08	6.30E-01	GPA33	12	rs2632226	75535673	3.99E-01	N/A	-	4.99E-09	3.49E-01	1.69E-05	40	10
2	rs1346722	1.68E+08	6.75E-01	XIRP2	19	rs889362	18619255	5.85E-02	KLHL26	-	5.03E-09	3.89E-02	3.13E-04	41	
6	rs7768444	825234	5.45E-01	N/A	1	rs12044604	59427090	5.87E-01	N/A	-	5.17E-09	5.72E-01	2.14E-07	42	3
6	rs7768444	825234	5.45E-01	N/A	1	rs3850543	59426218	5.46E-01	N/A	-	5.21E-09	5.72E-01	2.13E-07	43	3
3	rs4684717	10598391	7.53E-01	N/A	14	rs1894371	21704535	1.04E-01	N/A	-	5.31E-09	9.33E-01	2.01E-05	44	8
4	rs3762876	95808500	7.86E-03	PDLIM5	4	rs10489022	1.38E+08	9.82E-01	N/A	-	5.43E-09	8.74E-01	2.89E-06	45	
6	rs887464	31253899	8.59E-02	TCF19	6	rs3130473	31307187	2.29E-01	N/A	MHC	5.74E-09	2.18E-01	3.00E-07	46	1
7	rs219827	98486005	1.84E-01	SMURF1	12	rs10843756	30507005	6.47E-01	N/A	-	5.86E-09	2.13E-01	2.31E-08	47	
14	rs12050150	31637758	2.91E-01	ARHGAP5	19	rs296364	53067510	3.31E-01	SULT2A1	-	5.90E-09	8.96E-01	1.45E-05	48	
5	rs4568402	2150011	7.54E-01	N/A	8	rs1430849	1.26E+08	1.05E-01	MTSS1	-	6.10E-09	1.54E-01	1.47E-03	49	11
5	rs4348193	77671476	5.91E-01	N/A	11	rs1800849	73397813	6.34E-01	UCP3	-	6.31E-09	9.18E-01	7.06E-06	50	5
1	rs12041287	1.65E+08	2.21E-01	GPA33	12	rs922380	75600329	7.23E-01	N/A	-	6.43E-09	1.73E-01	7.20E-05	51	10
1	rs12140479	70595826	2.66E-01	ANKRD13C	8	rs12676876	69767325	1.32E-01	C8orf34	-	6.53E-09	3.69E-01	1.09E-06	52	
6	rs4122189	31275906	7.70E-02	HCG27	6	rs3130473	31307187	2.29E-01	N/A	MHC	6.53E-09	7.02E-01	4.87E-05	53	1
5	rs4299769	2150562	6.31E-01	N/A	8	rs1430849	1.26E+08	1.05E-01	MTSS1	-	6.63E-09	1.84E-01	1.52E-03	54	11
5	rs4348193	77671476	5.91E-01	N/A	11	rs11236029	73555872	3.42E-01	C2CD3	-	6.98E-09	9.99E-01	5.35E-06	55	5
2	rs16986953	19805954	3.64E-01	N/A	10	rs11258082	13039121	1.64E-01	CCDC3	-	7.19E-09	8.99E-01	2.39E-06	56	
6	rs12660608	1.23E+08	8.18E-02	CLVS2	1	rs1552991	1.5E+08	1.63E-01	HRNR	-	7.24E-09	3.13E-01	4.87E-05	57	12
6	rs2078901	67052116	7.04E-01	N/A	9	rs11169848	88671816	1.22E-01	N/A	-	7.54E-09	3.82E-01	1.94E-06	58	
6	rs11753072	811963	7.32E-01	N/A	1	rs11207426	59458507	2.71E-01	N/A	-	8.06E-09	7.53E-01	1.92E-06	59	3
3	rs2049276	1.5E+08	3.00E-01	N/A	11	rs720749	1.09E+08	5.35E-01	N/A	-	8.41E-09	6.35E-01	1.87E-07	60	13
6	rs4122189	31275906	7.70E-02	HCG27	6	rs3130467	31295054	5.92E-02	HCG27	MHC	8.45E-09	3.42E-01	1.23E-06	61	1
1	rs2817178	3037112	8.46E-01	PRDM16	16	rs2058529	6621545	9.82E-01	A2BP1	-	8.79E-09	3.68E-01	2.85E-07	62	
6	rs13201721	1.42E+08	5.49E-01	N/A	4	rs1875341	7287993	9.59E-01	SORCS2	-	8.91E-09	9.38E-01	1.88E-06	63	
6	rs879882	31247431	7.61E-01	TCF19	6	rs1131896	31487094	3.40E-01	MICA	MHC	9.38E-09	6.40E-02	2.72E-08	64	1
4	rs13111857	1.67E+08	9.24E-01	N/A	9	rs6559402	80521159	9.45E-01	N/A	-	9.41E-09	5.14E-01	1.45E-06	65	
7	rs6460664	70638550	2.18E-01	WBSCR17	9	rs2300932	1.23E+08	6.11E-01	C5	-	9.63E-09	9.76E-03	8.86E-10	66	
6	rs3868542	31253818	6.85E-02	TCF19	6	rs3130473	31307187	2.29E-01	N/A	MHC	9.74E-09	3.59E-01	1.19E-06	67	1
4	rs6836201	1.81E+08	5.52E-01	N/A	8	rs16904685	1.34E+08	3.57E-01	KCNQ3	-	1.00E-08	4.68E-01	3.63E-05	68	
2	rs17039713	60190093	1.70E-02	N/A	12	rs708837	1.15E+08	3.47E-01	N/A	-	1.04E-08	4.42E-01	1.80E-04	69	
1	rs2117905	76825184	3.98E-01	ST6GALN/AC3	1	rs2815217	2.16E+08	4.00E-01	N/A	-	1.05E-08	8.77E-01	2.00E-05	70	
1	rs11161944	87427396	7.61E-01	LOC339524	16	rs1981492	296690	4.85E-01	AXIN1	-	1.07E-08	7.11E-02	1.36E-04	71	
6	rs2233984	31187243	4.20E-02	C6orf15	6	rs887464	31253899	8.59E-02	TCF19	MHC	1.09E-08	3.34E-02	2.44E-08	72	1
3	rs2639369	1.5E+08	2.81E-01	N/A	11	rs720749	1.09E+08	5.35E-01	N/A	-	1.11E-08	3.63E-01	6.17E-08	73	13
3	rs2687660	1.5E+08	2.81E-01	N/A	11	rs720749	1.09E+08	5.35E-01	N/A	-	1.11E-08	3.63E-01	6.17E-08	74	13
8	rs12676446	18560492	3.77E-01	PSD3	9	rs914355	1.12E+08	6.68E-01	PALM2-AKAP2	-	1.11E-08	5.31E-01	1.52E-06	75	
10	rs7907344	10998527	5.91E-01	LOC254312	10	rs7084752	97060105	9.11E-01	SORBS1	-	1.12E-08	4.24E-03	5.49E-02	76	
8	rs10448145	11327179	9.55E-01	C8orf12	15	rs11247349	96851676	8.76E-01	FAM169B	-	1.12E-08	3.94E-01	2.07E-07	77	14
8	rs12549796	11327107	9.55E-01	C8orf12	15	rs11247349	96851676	8.76E-01	FAM169B	-	1.12E-08	3.94E-01	2.07E-07	78	14
1	rs10922563	89319391	2.84E-01	GBP1	13	rs2321995	58577784	3.86E-01	N/A	-	1.12E-08	4.64E-01	6.46E-07	79	
6	rs12212773	1.55E+08	1.06E-01	IPCEF1	10	rs10904177	4102318	9.30E-01	N/A	-	1.14E-08	7.76E-01	2.95E-06	80	15
3	rs4684717	10598391	7.53E-01	N/A	14	rs6572313	21751816	7.24E-02	N/A	-	1.14E-08	8.53E-01	1.95E-05	81	8
2	rs17031450	66156704	4.16E-01	N/A	2	rs930365	2.4E+08	4.16E-02	N/A	-	1.15E-08	2.80E-01	4.07E-07	82	
1	rs12041287	1.65E+08	2.21E-01	GPA33	12	rs2669013	75547079	6.30E-01	N/A	-	1.16E-08	N/A	N/A	83	10
3	rs12489904	20359631	4.21E-01	N/A	14	rs1152490	55865987	1.95E-01	N/A	-	1.16E-08	8.52E-01	1.91E-06	84	
5	rs307176	6140464	4.26E-01	N/A	5	rs6868098	1.54E+08	8.15E-01	LARP1	-	1.17E-08	4.74E-01	2.62E-05	85	4
6	rs11753072	811963	7.32E-01	N/A	1	rs12044604	59427090	5.87E-01	N/A	-	1.17E-08	3.03E-01	1.98E-07	86	3
5	rs307176	6140464	4.26E-01	N/A	5	rs10039709	1.54E+08	8.14E-01	LARP1	-	1.19E-08	5.05E-01	2.10E-05	87	4
2	rs976904	1.33E+08	1.22E-01	NCKAP5	11	rs2512568	1.15E+08	5.17E-01	N/A	-	1.19E-08	7.77E-01	7.13E-07	88	
1	rs4656520	1.65E+08	6.30E-01	GPA33	12	rs2669013	75547079	6.30E-01	N/A	-	1.20E-08	N/A	N/A	89	10
6	rs11753072	811963	7.32E-01	N/A	1	rs3850543	59426218	5.46E-01	N/A	-	1.20E-08	3.03E-01	2.00E-07	90	3
2	rs7566955	1.41E+08	1.25E-02	N/A	12	rs11172506	56921388	2.79E-01	N/A	-	1.20E-08	6.54E-01	4.96E-05	91	
8	rs3864665	62093567	7.26E-01	N/A	22	rs3788535	36863991	9.84E-02	PLA2G6	-	1.24E-08	8.74E-01	6.91E-06	92	16
8	rs3864665	62093567	7.26E-01	N/A	22	rs4820315	36848677	9.84E-02	PLA2G6	-	1.24E-08	8.74E-01	6.91E-06	93	16
8	rs3864665	62093567	7.26E-01	N/A	22	rs2076118	36859446	9.84E-02	PLA2G6	-	1.24E-08	N/A	N/A	94	16
3	rs4684717	10598391	7.53E-01	N/A	14	rs3811285	21693095	9.36E-02	N/A	-	1.27E-08	9.78E-01	4.50E-05	95	8
6	rs12212773	1.55E+08	1.06E-01	IPCEF1	10	rs2069178	4118024	7.87E-01	N/A	-	1.27E-08	8.78E-01	3.86E-06	96	15
6	rs1796520	26518779	8.92E-01	BTN3A1	15	rs813299	29225595	8.06E-01	N/A	MHC	1.28E-08	1.34E-01	3.47E-04	97	
5	rs10059955	1.05E+08	1.38E-01	N/A	12	rs4763075	61962124	4.02E-01	N/A	-	1.29E-08	6.41E-01	6.25E-06	98	7
6	rs7774522	1.23E+08	6.95E-02	CLVS2	1	rs1552991	1.5E+08	1.63E-01	HRNR	-	1.30E-08	2.37E-01	1.07E-04	99	12
2	rs2359964	2.08E+08	4.54E-01	KLF7	17	rs3764899	4669572	2.36E-01	PLD2	-	1.31E-08	9.84E-02	3.27E-03	100	
10	rs1057549	49537333	8.44E-01	N/A	12	rs7311906	1.14E+08	9.39E-02	N/A	-	1.32E-08	2.65E-01	6.82E-08	101	
4	rs11726511	24919158	5.11E-01	ZCCHC4	9	rs626365	1.35E+08	2.54E-01	RALGDS	-	1.34E-08	9.55E-02	2.92E-04	102	
7	rs599574	70776464	3.50E-01	WBSCR17	10	rs946028	8388335	8.75E-01	N/A	-	1.35E-08	5.97E-01	3.43E-05	103	
1	rs16860429	1.81E+08	6.09E-01	LAMC2	11	rs4244525	26652919	2.96E-01	SLC5A12	-	1.35E-08	4.34E-01	6.50E-07	104	
4	rs17040984	1.63E+08	9.50E-02	FSTL5	8	rs2035919	18151237	4.61E-01	N/A	-	1.36E-08	1.94E-01	1.43E-07	105	
6	rs4495304	31188697	4.80E-02	C6orf15	6	rs887464	31253899	8.59E-02	TCF19	MHC	1.39E-08	3.34E-02	2.94E-08	106	
1	rs12041287	1.65E+08	2.21E-01	GPA33	12	rs11114901	75581238	5.64E-01	N/A	-	1.40E-08	4.06E-01	1.85E-05	107	
4	rs1379410	1.8E+08	9.15E-02	N/A	5	rs2416349	1.13E+08	9.44E-01	N/A	-	1.47E-08	9.13E-01	1.49E-05	108	
7	rs6460671	70658243	2.65E-01	WBSCR17	9	rs2300932	1.23E+08	6.11E-01	C5	-	1.47E-08	8.46E-03	1.49E-09	109	
9	rs13284995	35832854	2.53E-01	TMEM8B	14	rs2144039	22799242	2.41E-01	HOMEZ	-	1.48E-08	7.49E-01	5.64E-05	110	

SNP A					SNP B						Interaction P			Ranking	Cluster in top 100 interactions
CHR	SNP	Location	gle locus P va	Gene	CHR	SNP	Location	ingle locus P valu	Gene	MHC region	Stage 1	Stage 2	Combined		
9	rs7869423	1624618	8.04E-01	N/A	17	rs11650227	52864287	4.11E-01	MSI2	-	1.49E-08	3.15E-01	2.56E-07	111	
1	rs2358994	1.14E+08	9.12E-01	BCL2L15	16	rs8044476	71358068	7.56E-01	ZFHX3	-	1.50E-08	3.79E-01	6.20E-05	112	
5	rs1435194	1.05E+08	1.04E-01	N/A	12	rs12824017	61935103	2.85E-01	N/A	-	1.52E-08	7.77E-01	1.27E-05	113	
7	rs219827	98486005	1.84E-01	SMURF1	10	rs10771730	30507860	5.51E-01	N/A	-	1.52E-08	4.64E-02	4.10E-09	114	
1	rs3813982	2.05E+08	5.44E-01	N/A	19	rs17361438	53540514	6.59E-01	TMEM143	-	1.53E-08	4.22E-01	1.44E-04	115	
3	rs6790154	1.26E+08	4.27E-01	SLC12A8	3	rs1430408	1.58E+08	2.53E-01	N/A	-	1.55E-08	2.12E-01	1.42E-06	116	
3	rs4684717	10598391	7.53E-01	N/A	14	rs2242542	21740815	8.39E-02	N/A	-	1.56E-08	8.53E-01	2.36E-05	117	
7	rs6460664	70638550	2.18E-01	WBSCR17	9	rs3789311	1.23E+08	7.66E-01	CEP110	-	1.56E-08	1.01E-02	1.50E-09	118	
1	rs1410244	1.65E+08	4.30E-01	DUSP27	12	rs2632226	75535673	3.99E-01	N/A	-	1.56E-08	1.61E-01	1.45E-04	119	
4	rs13144404	44130442	8.14E-01	KCTD8	12	rs10858403	75142247	1.81E-03	N/A	-	1.59E-08	2.38E-01	1.94E-04	120	
4	rs7660000	89970881	2.31E-01	FAM13A	5	rs6863774	55730873	1.10E-01	N/A	-	1.60E-08	1.45E-01	4.38E-07	121	
17	rs17247848	28008297	5.07E-01	MYO1D	18	rs1893489	46449659	8.82E-01	MAPK4	-	1.63E-08	3.49E-01	4.21E-04	122	
1	rs6673606	1.81E+08	6.15E-01	LAMC2	11	rs4244525	26652919	2.96E-01	SLC5A12	-	1.64E-08	4.88E-01	9.44E-07	123	
8	rs352810	15666890	6.99E-01	TUSC3	18	rs2432842	68860542	1.84E-01	N/A	-	1.65E-08	5.31E-01	1.71E-06	124	
2	rs6712787	2.21E+08	4.12E-01	N/A	5	rs6596352	1.36E+08	5.11E-01	N/A	-	1.66E-08	7.78E-01	3.28E-05	125	
2	rs7599725	2.21E+08	4.12E-01	N/A	5	rs6596352	1.36E+08	5.11E-01	N/A	-	1.66E-08	7.78E-01	3.28E-05	126	
2	rs12694536	2.21E+08	4.14E-01	N/A	5	rs6596352	1.36E+08	5.11E-01	N/A	-	1.67E-08	7.13E-01	4.91E-05	127	
1	rs10922563	89319391	2.84E-01	GBP1	13	rs2321997	58561359	4.61E-01	N/A	-	1.69E-08	4.64E-01	8.60E-07	128	
5	rs4348193	77671476	5.91E-01	N/A	11	rs1944971	73601066	3.55E-01	PPME1	-	1.70E-08	9.99E-01	8.96E-06	129	
1	rs4554704	1.94E+08	9.33E-01	N/A	20	rs1052653	49438061	4.58E-01	NFATC2	-	1.75E-08	5.42E-01	8.62E-05	130	
1	rs1597732	1.94E+08	9.22E-01	N/A	20	rs1052653	49438061	4.58E-01	NFATC2	-	1.77E-08	6.50E-01	7.00E-05	131	
6	rs6911781	1.14E+08	3.17E-01	N/A	2	rs1383413	1.8E+08	8.86E-01	ZNF385B	-	1.77E-08	N/A	N/A	132	
1	rs556596	28281983	6.43E-02	EYA3	19	rs2290669	16356774	8.42E-01	EPS15L1	-	1.79E-08	8.55E-01	6.05E-06	133	
1	rs384642	91403679	1.49E-01	N/A	11	rs10832843	1354595	4.92E-02	BRSK2	-	1.79E-08	9.69E-01	3.02E-06	134	
6	rs12660608	1.23E+08	8.18E-02	CLVS2	1	rs17596572	1.5E+08	1.20E-01	HRNR	-	1.81E-08	3.74E-01	4.81E-05	135	
3	rs2712353	1.15E+08	5.03E-01	ATP6V1A	12	rs4762758	20505926	3.12E-01	PDE3A	-	1.82E-08	3.30E-01	2.69E-04	136	
8	rs12676761	64030368	9.01E-01	NKAIN3	10	rs7091494	14887155	9.95E-01	CDNF	-	1.87E-08	6.81E-01	8.68E-06	137	
5	rs2560412	5202621	4.31E-01	ADAMTS16	12	rs862231	89243803	5.47E-02	N/A	-	1.87E-08	N/A	N/A	138	
5	rs4348193	77671476	5.91E-01	N/A	11	rs1685333	73405807	6.89E-01	C2CD3	-	1.88E-08	7.28E-01	3.47E-05	139	
6	rs7761965	31381474	9.15E-01	N/A	6	rs2523608	31430538	3.16E-01	HLA-B	MHC	1.89E-08	1.51E-01	N/A	140	
7	rs886972	1.48E+08	7.02E-01	CNTN/AP2	20	rs6063634	49376385	4.82E-01	N/A	-	1.89E-08	8.85E-01	8.11E-06	141	
3	rs12492728	40601372	6.26E-01	N/A	17	rs9905601	37268737	2.20E-01	KLHL10	-	1.90E-08	5.81E-01	1.14E-04	142	
1	rs3013101	13672722	6.40E-01	N/A	13	rs7331424	22484366	5.73E-01	N/A	-	1.92E-08	6.56E-01	8.29E-05	143	
1	rs3908575	98636348	6.82E-01	N/A	9	rs17083209	82166375	1.33E-01	N/A	-	1.94E-08	2.04E-01	6.05E-07	144	
2	rs7566955	1.41E+08	1.25E-02	N/A	12	rs12298303	56947982	3.10E-01	N/A	-	1.94E-08	7.21E-01	6.44E-05	145	
9	rs1463014	22934927	9.91E-01	N/A	15	rs691	22915888	2.47E-01	SNORD116-27	-	1.95E-08	4.33E-01	3.61E-07	146	
2	rs6737780	2.39E+08	2.74E-01	TRAF3IP1	10	rs17097516	1.2E+08	8.73E-01	N/A	-	1.95E-08	7.42E-01	2.19E-05	147	
6	rs4896219	1.37E+08	8.74E-01	MAP3K5	8	rs2979419	62167326	2.62E-01	N/A	-	1.95E-08	9.90E-01	1.41E-05	148	
5	rs2162928	6137255	3.48E-01	N/A	5	rs6868098	1.54E+08	8.15E-01	LARP1	-	1.96E-08	4.22E-01	5.25E-05	149	
3	rs1522948	1.5E+08	3.82E-01	N/A	11	rs720749	1.09E+08	5.35E-01	N/A	-	1.96E-08	6.37E-01	2.81E-07	150	
12	rs1872521	1.27E+08	8.77E-01	N/A	22	rs8136069	35052436	4.00E-01	MYH9	-	1.97E-08	4.81E-01	2.65E-06	151	
1	rs950302	1.65E+08	5.39E-01	DUSP27	12	rs2632226	75535673	3.99E-01	N/A	-	1.97E-08	3.57E-01	4.03E-05	152	
8	rs12056725	1.46E+08	1.28E-01	SCRT1	10	rs1471824	72405746	7.51E-01	N/A	-	2.00E-08	6.12E-01	1.06E-04	153	
5	rs2162928	6137255	3.48E-01	N/A	5	rs10039709	1.54E+08	8.14E-01	LARP1	-	2.00E-08	4.52E-01	4.20E-05	154	
1	rs7523434	1.65E+08	1.28E-01	GPA33	12	rs2632226	75535673	3.99E-01	N/A	-	2.02E-08	8.61E-01	5.64E-06	155	
12	rs2268009	6210649	8.30E-01	CD9	12	rs748380	1.16E+08	7.37E-01	C12orf49	-	2.05E-08	7.84E-01	1.58E-05	156	
6	rs7768444	825234	5.45E-01	N/A	1	rs331635	59438130	5.19E-01	N/A	-	2.05E-08	8.44E-01	1.24E-05	157	
3	rs1871493	1.42E+08	8.68E-01	CLSTN2	5	rs2052007	1.29E+08	9.44E-01	CHSY3	-	2.06E-08	9.48E-01	1.96E-05	158	
2	rs2052955	82182372	1.41E-02	N/A	17	rs2240774	72222167	7.40E-01	JMJD6	-	2.07E-08	2.39E-01	1.52E-04	159	
1	rs680925	66355709	3.11E-01	PDE4B	1	rs6685742	1.7E+08	2.31E-01	N/A	-	2.08E-08	9.30E-01	1.57E-05	160	
2	rs7577337	1.69E+08	1.27E-01	N/A	12	rs10877902	61330448	8.81E-01	PPM1H	-	2.12E-08	6.64E-01	2.46E-04	161	
3	rs3845906	66609292	2.77E-01	LRIG1	18	rs2132918	14586020	1.90E-01	N/A	-	2.13E-08	9.96E-01	8.88E-06	162	
2	rs4669572	10392759	5.05E-02	HPCAL1	11	rs1346118	19176075	6.24E-01	CSRP3	-	2.15E-08	4.25E-01	5.96E-05	163	
2	rs17012765	76565481	9.53E-02	N/A	5	rs874547	66180569	1.71E-01	MAST4	-	2.15E-08	5.45E-01	1.25E-06	164	
3	rs7648359	1.36E+08	5.09E-01	EPHB1	13	rs7322307	36248679	9.25E-01	N/A	-	2.15E-08	6.53E-01	1.53E-04	165	
10	rs1110399	72770272	3.81E-01	N/A	13	rs963943	53356272	1.90E-01	N/A	-	2.16E-08	1.96E-01	5.16E-04	166	
5	rs274362	53372577	5.22E-01	ARL15	17	rs2305529	71213848	4.16E-01	ITGB4	-	2.17E-08	5.83E-01	6.89E-05	167	
3	rs823028	1.96E+08	1.97E-01	N/A	9	rs11138025	80848452	4.85E-01	N/A	-	2.17E-08	6.44E-01	1.83E-04	168	
6	rs9295957	31265572	2.18E-01	POU5F1	6	rs3130467	31295054	5.92E-02	HCG27	MHC	2.18E-08	4.13E-01	3.03E-06	169	
2	rs205628	74814524	5.13E-01	N/A	5	rs752283	1.78E+08	8.21E-01	COL23A1	-	2.21E-08	8.58E-01	1.14E-05	170	
1	rs478665	65288121	8.38E-01	MIR101-1	10	rs4748702	21053851	8.02E-01	N/A	-	2.21E-08	5.73E-01	1.43E-06	171	
6	rs2233984	31187243	4.20E-02	C6orf15	6	rs4122189	31275906	7.70E-02	HCG27	MHC	2.21E-08	1.22E-01	2.37E-07	172	
10	rs11006830	28396093	1.45E-01	MPP7	19	rs1661926	58324708	8.62E-01	ZNF415	-	2.22E-08	9.93E-01	7.79E-06	173	
2	rs12471133	1.56E+08	6.61E-01	N/A	4	rs10014448	27369239	7.92E-01	N/A	-	2.23E-08	2.05E-01	1.59E-03	174	
1	rs1688582	1.02E+08	4.62E-01	N/A	3	rs4688645	61595936	3.39E-01	PTPRG	-	2.25E-08	9.91E-01	3.54E-05	175	
1	rs1125953	1.96E+08	9.32E-01	N/A	7	rs194834	1.04E+08	9.34E-01	ORC5L	-	2.26E-08	8.07E-01	5.70E-06	176	
11	rs555097	56919292	3.56E-01	PRG3	14	rs8020693	69670487	5.66E-01	SLC8A3	-	2.27E-08	5.99E-01	2.61E-06	177	
11	rs555097	56919292	3.56E-01	PRG3	14	rs8022074	69670478	5.66E-01	SLC8A3	-	2.27E-08	5.99E-01	2.61E-06	178	
11	rs3851114	56923237	3.99E-01	PRG3	14	rs8020693	69670487	5.66E-01	SLC8A3	-	2.27E-08	6.73E-01	5.69E-06	179	
11	rs3851114	56923237	3.99E-01	PRG3	14	rs8022074	69670478	5.66E-01	SLC8A3	-	2.27E-08	6.73E-01	5.69E-06	180	
21	rs2829160	24840460	2.46E-01	N/A	22	rs17210001	18294854	7.97E-02	TXNRD2	-	2.28E-08	8.66E-01	4.71E-05	181	
1	rs12040932	28183811	8.69E-02	EYA3	19	rs2290669	16356774	8.42E-01	EPS15L1	-	2.29E-08	9.01E-01	3.32E-06	182	
6	rs6900659	1.06E+08	8.04E-01	N/A	2	rs11682423	1.63E+08	7.51E-03	KCNH7	-	2.30E-08	2.97E-01	2.79E-06	183	
3	rs823028	1.96E+08	1.97E-01	N/A	9	rs7853145	80848802	4.69E-01	N/A	-	2.31E-08	6.44E-01	1.90E-04	184	
2	rs7566955	1.41E+08	1.25E-02	N/A	12	rs1553380	56897140	2.79E-01	N/A	-	2.34E-08	7.16E-01	6.37E-05	185	
3	rs11719694	20363109	3.03E-01	N/A	14	rs1152490	55865987	1.95E-01	N/A	-	2.35E-08	8.52E-01	3.13E-06	186	
5	rs1865508	1.05E+08	1.73E-01	N/A	12	rs4763075	61962124	4.02E-01	N/A	-	2.36E-08	6.39E-01	4.94E-06	187	
3	rs10514694	36283073	2.94E-01	N/A	16	rs6500892	6953116	9.44E-01	A2BP1	-	2.36E-08	3.45E-01	1.23E-06	188	
3	rs3845906	66609292	2.77E-01	LRIG1	18	rs11080775	14572736	1.07E-01	N/A	-	2.37E-08	9.27E-01	1.22E-05	189	
21	rs1541289	24844319	3.92E-01	N/A	22	rs17210001	18294854	7.97E-02	TXNRD2	-	2.38E-08	5.93E-01	2.25E-04	190	
6	rs2811231	55263663	7.58E-01	HCRTR2	5	rs5326	1.75E+08	8.38E-01	DRD1	-	2.39E-08	9.08E-01	4.07E-05	191	
6	rs2811232	55264771	7.58E-01	HCRTR2	5	rs5326	1.75E+08	8.38E-01	DRD1	-	2.39E-08	9.08E-01	4.07E-05	192	
8	rs7000979	17210139	1.32E-01	MTMR7	15	rs767387	51385518	8.23E-01	N/A	-	2.39E-08	7.94E-01	1.66E-06	193	
1	rs12037569	1.1E+08	4.37E-01	SORT1	5	rs10069547	1.55E+08	1.33E-01	N/A	-	2.40E-08	5.52E-01	1.62E-04	194	
1	rs12037569	1.1E+08	4.37E-01	SORT1	5	rs13354678	1.55E+08	1.33E-01	N/A	-	2.40E-08	5.52E-01	1.62E-04	195	
5	rs10477862	1.06E+08	8.56E-02	N/A	12	rs4763075	61962124	4.02E-01	N/A	-	2.41E-08	6.08E-01	9.25E-06	196	
2	rs17013592	77088275	7.17E-01	LRRTM4	5	rs160890	3187879	9.49E-01	N/A	-	2.44E-08	9.81E-01	1.48E-05	197	
3	rs823028	1.96E+08	1.97E-01	N/A	9	rs6559443	80837613	3.04E-01	N/A	-	2.45E-08	N/A	N/A	198	
7	rs886972	1.48E+08	7.02E-01	CNTN/AP2	20	rs761685	49373633	4.46E-01	N/A	-	2.46E-08	8.56E-01	2.20E-05	199	
1	rs384642	91403679	1.49E-01	N/A	11	rs4881745	1354892	6.43E-02	BRSK2	-	2.47E-08	N/A	N/A	200	
13	rs16968691	1.06E+08	8.10E-01	N/A	19	rs718258	15627419	2.61E-01	CYP4F3	-	2.48E-08	9.95E-01	2.45E-05	201	
6	rs9295957	31265572	2.18E-01	POU5F1	6	rs3130473	31307187	2.29E-01	N/A	MHC	2.55E-08	4.64E-01	3.68E-06	202	
1	rs4652781	1.81E+08	5.30E-01	LAMC2	11	rs4244525	26652919	2.96E-01	SLC5A12	-	2.55E-08	1.76E-01	2.15E-07	203	
3	rs860309	1.96E+08	1.06E-01	N/A	9	rs11138025	80848452	4.85E-01	N/A	-	2.56E-08	8.01E-01	1.14E-04	204	
5	rs4921132	1.6E+08	8.00E-02	C1QTNF2	7	rs2001012	1.42E+08	8.11E-01	TRYX3	-	2.56E-08	6.80E-01	8.65E-06	205	
3	rs11709533	66622318	4.47E-01	LRIG1	18	rs11080775	14572736	1.07E-01	N/A	-	2.60E-08	8.66E-01	1.77E-05	206	
6	rs7750201	1.06E+08	9.29E-02	N/A	1	rs2938616	1.1E+08	8.22E-01	N/A	-	2.60E-08	6.07E-01	4.12E-05	207	
5	rs10069382	31957022	1.47E-01	PDZD2	17	rs12452082	43299000	3.60E-01	SP6	-	2.61E-08	N/A	N/A	208	
2	rs11898850	73053127	9.44E-01	SFXN5	11	rs340938	22446557	4.91E-01	N/A	-	2.62E-08	7.61E-01	2.37E-05	209	
2	rs2052955	82182372	1.41E-02	N/A	17	rs7211963	72208041	5.06E-01	JMJD6	-	2.62E-08	3.24E-01	1.10E-04	210	
2	rs10167584	82185384	2.12E-02	N/A	17	rs2240774	72222167	7.40E-01	JMJD6	-	2.63E-08	2.64E-01	1.30E-04	211	
8	rs12679296	1.16E+08	4.04E-01	N/A	15	rs7173054	25550656	4.83E-01	N/A	-	2.64E-08	7.23E-01	2.52E-05	212	
6	rs9285484	1.37E+08	7.87E-01	MAP3K5	8	rs2979419	62167326	2.62E-01	N/A	-	2.65E-08	7.07E-01	2.83E-06	213	
7	rs6460671	70658243	2.65E-01	WBSCR17	9	rs3789311	1.23E+08	7.66E-01	CEP110	-	2.67E-08	8.49E-03	2.68E-09	214	
6	rs3130688	31318195	3.32E-01	N/A	6	rs9394047	31344229	1.23E-01	HLA-C	MHC	2.67E-08	7.56E-01	3.19E-06	215	
6	rs887464	31253899	8.59E-02	TCF19	6	rs7750269	31379136	8.58E-01	N/A	MHC	2.70E-08	2.64E-01	1.33E-04	216	
2	rs1402774	82195241	2.08E-02	N/A	17	rs2240774	72222167	7.40E-01	JMJD6	-	2.70E-08	1.91E-01	5.10E-07	217	
3	rs860309	1.96E+08	1.06E-01	N/A	9	rs7853145	80848802	4.69E-01	N/A	-	2.75E-08	8.01E-01	1.18E-04	218	
6	rs9348868	11476104	7.56E-01	NEDD9	1	rs7540340	30138444	8.33E-01	N/A	-	2.75E-08	4.51E-01	6.16E-07	219	
5	rs274362	53372577	5.22E-01	ARL15	17	rs3744000	71215928	4.53E-01	ITGB4	-	2.76E-08	5.83E-01	7.85E-05	220	
1	rs10489667	1.15E+08	9.12E-01	N/A	12	rs1950049	1.18E+08	4.35E-01	N/A	-	2.78E-08	3.91E-01	4.79E-05	221	
8	rs4301474	1.2E+08	2.83E-01	N/A	18	rs9646553	47274701	8.69E-01	N/A	-	2.81E-08	1.82E-01	6.42E-07	222	

SNP A					SNP B						Interaction P			Ranking	Cluster in top 100 interactions
CHR	SNP	Location	gle locus P va	Gene	CHR	SNP	Location	Single locus P valu	Gene	HC region	Stage 1	Stage 2	Combined		
6	rs9355586	1.67E+08	6.60E-01	RPS6KA2	12	s12580153	91342613	1.82E-01	CLLU1	-	2.83E-08	2.34E-01	3.05E-04	223	
6	rs4495304	31188697	4.80E-02	C6orf15	6	rs4122189	31275906	7.70E-02	HCG27	MHC	2.83E-08	1.22E-01	2.90E-07	224	
1	rs4658931	2.29E+08	4.05E-01	FAM89A	12	s10858625	86517374	8.47E-01	N/A	-	2.84E-08	3.92E-01	8.16E-06	225	
14	rs4584745	53097749	5.78E-01	N/A	20	rs1736493	43964041	5.37E-01	PLTP	-	2.84E-08	9.93E-01	3.00E-05	226	
8	rs1598294	15675662	7.57E-01	TUSC3	18	rs8093852	68855454	1.39E-01	N/A	-	2.85E-08	2.87E-01	3.07E-07	227	
5	rs252155	1.5E+08	6.37E-01	RBM22	8	s10094729	62798716	2.87E-01	ASPH	-	2.85E-08	2.23E-01	7.83E-07	228	
2	rs11898850	73053127	9.44E-01	SFXN5	11	rs340936	22444915	4.47E-01	N/A	-	2.86E-08	7.61E-01	2.53E-05	229	
2	rs12622589	45072318	7.61E-02	SIX2	2	rs6740621	2.35E+08	7.12E-01	ARL4C	-	2.87E-08	3.43E-01	2.68E-07	230	
5	rs1363292	92207389	5.85E-01	N/A	11	s11042978	2154994	3.22E-01	N/A	-	2.88E-08	1.65E-01	2.11E-04	231	
3	rs7651855	1.62E+08	4.74E-01	ARL14	20	rs858140	24009345	2.02E-01	N/A	-	2.89E-08	9.13E-01	9.24E-06	232	
5	rs3903000	52208612	8.96E-01	N/A	22	rs2142831	41131396	2.54E-01	NFAM1	-	2.92E-08	5.77E-01	2.31E-06	233	
12	rs12184484	96036626	3.87E-01	N/A	19	rs2334253	50808317	2.61E-01	N/A	-	2.93E-08	5.54E-01	1.36E-05	234	
12	rs17751083	96037531	3.87E-01	N/A	19	rs2334253	50808317	2.61E-01	N/A	-	2.93E-08	5.54E-01	1.36E-05	235	
8	rs2341797	29387170	6.22E-01	N/A	11	s10500862	19821432	7.09E-01	N/AV2	-	2.93E-08	2.41E-01	2.39E-07	236	
2	rs4669572	10392759	5.05E-02	HPCAL1	11	rs793272	19204188	4.66E-01	E2F8	-	2.93E-08	3.29E-01	1.39E-04	237	
12	rs1872521	1.27E+08	8.77E-01	N/A	22	rs1557540	35062483	3.50E-01	MYH9	-	2.93E-08	3.57E-01	1.67E-06	238	
10	rs11598564	1.03E+08	4.52E-02	N/A	12	rs3858666	22795317	8.86E-01	N/A	-	2.93E-08	4.30E-01	3.10E-06	239	
8	rs352810	15666890	6.99E-01	TUSC3	18	rs8093852	68855454	1.39E-01	N/A	-	2.95E-08	5.62E-01	2.03E-06	240	
1	rs10157812	98656129	2.35E-01	N/A	9	s17083209	82166375	1.33E-01	N/A	-	2.95E-08	3.24E-01	2.36E-06	241	
1	rs7542173	98656811	2.35E-01	N/A	9	s17083209	82166375	1.33E-01	N/A	-	2.95E-08	3.24E-01	2.36E-06	242	
3	rs7648359	1.36E+08	5.09E-01	EPHB1	13	rs9603097	36242187	6.41E-01	N/A	-	2.96E-08	5.22E-02	1.02E-07	243	
6	rs4947296	31166157	4.31E-02	N/A	6	rs887464	31253899	8.59E-02	TCF19	MHC	2.96E-08	6.31E-01	1.46E-04	244	
5	rs13183847	1.25E+08	6.50E-01	N/A	12	s10862213	79893973	2.43E-01	N/A	-	2.96E-08	8.83E-01	5.37E-05	245	
5	rs31226	53363328	3.70E-01	ARL15	17	rs2305529	71213848	4.16E-01	ITGB4	-	2.97E-08	8.29E-01	3.06E-05	246	
6	rs3094205	31199841	5.91E-01	C6orf15	14	rs2809121	97504272	2.57E-01	C14orf64	MHC	2.98E-08	1.08E-01	5.95E-08	247	
2	rs1263595	2.08E+08	4.03E-01	KLF7	17	rs3764899	4669572	2.36E-01	PLD2	-	2.98E-08	1.18E-01	3.68E-03	248	
6	rs512778	79401865	6.51E-02	N/A	17	rs8076949	23763267	2.71E-01	SLC46A1	-	2.99E-08	7.44E-01	7.27E-07	249	
7	rs509834	70775938	3.81E-01	WBSCR17	10	rs946028	8388335	8.75E-01	N/A	-	3.02E-08	6.06E-01	5.64E-05	250	
12	rs825090	1.01E+08	3.89E-01	MYBPC1	14	rs1958249	43362891	9.69E-02	N/A	-	3.03E-08	4.82E-01	4.69E-06	251	
13	rs16801	37878738	5.64E-01	N/A	18	rs568080	32094999	5.37E-01	MOCOS	-	3.05E-08	9.43E-01	8.01E-05	252	
6	rs887464	31253899	8.59E-02	TCF19	6	rs9368675	31380136	7.91E-01	N/A	MHC	3.07E-08	8.90E-01	1.41E-05	253	
7	rs17367928	77934930	9.71E-01	MAGI2	20	s12625612	42279088	8.94E-01	C20orf111	-	3.07E-08	8.90E-01	1.41E-05	254	
7	rs17367928	77934930	9.71E-01	MAGI2	20	rs4812799	42280108	8.94E-01	C20orf111	-	3.07E-08	1.91E-01	5.58E-07	255	
1	rs4140432	1.85E+08	6.49E-01	N/A	5	rs7715102	2427305	5.42E-01	N/A	-	3.07E-08	1.99E-01	3.89E-07	256	
5	rs33376	1.71E+08	8.11E-01	N/A	21	rs7275493	16971867	9.00E-01	N/A	-	3.08E-08	6.14E-01	5.26E-05	257	
8	rs1598294	15675662	7.57E-01	TUSC3	18	rs2432842	68860542	1.84E-01	N/A	-	3.10E-08	2.87E-01	4.48E-07	258	
3	rs1871493	1.42E+08	8.68E-01	CLSTN2	5	s17692371	1.29E+08	9.90E-01	CHSY3	-	3.12E-08	8.59E-01	1.86E-05	259	
1	rs10889978	41634712	1.59E-01	N/A	12	rs1807783	93504732	5.57E-01	TMCC3	-	3.12E-08	6.27E-01	1.10E-04	260	
1	rs578271	65277859	8.36E-01	MIR101-1	10	rs4748702	21053851	8.02E-01	N/A	-	3.12E-08	7.42E-01	3.90E-06	261	
4	rs2980089	4318199	8.86E-01	LYAR	5	s13166683	1.21E+08	7.34E-01	N/A	-	3.15E-08	4.47E-01	2.09E-06	262	
6	rs7774522	1.23E+08	6.95E-02	CLVS2	1	s17596572	1.5E+08	1.20E-01	HRNR	-	3.17E-08	2.86E-01	1.06E-04	263	
1	rs2686228	2.39E+08	9.03E-01	RGS7	12	rs4765461	1.26E+08	8.78E-02	N/A	-	3.18E-08	5.89E-01	7.72E-05	264	
5	rs4921132	1.6E+08	8.00E-02	C1QTNF2	7	rs7801392	1.42E+08	9.06E-01	MOXD2	-	3.19E-08	6.84E-01	1.02E-05	265	
3	rs2611623	1.89E+08	1.80E-01	N/A	8	rs7460495	59596784	7.15E-01	N/A	-	3.20E-08	8.19E-01	6.71E-07	266	
12	rs1872521	1.27E+08	8.77E-01	N/A	22	rs6000239	35045975	3.04E-01	MYH9	-	3.22E-08	4.47E-01	2.82E-06	267	
2	rs10167584	82185384	2.12E-02	N/A	17	rs7211963	72208041	5.06E-01	JMJD6	-	3.24E-08	3.54E-01	9.53E-05	268	
5	rs4457046	1.06E+08	5.53E-02	N/A	12	rs4763075	61962124	4.02E-01	N/A	-	3.27E-08	6.99E-01	1.56E-05	269	
1	rs1804506	91920601	5.28E-01	N/A	1	s12405469	2.25E+08	7.98E-02	PSEN2	-	3.28E-08	9.21E-01	2.09E-05	270	
1	rs9287077	1.65E+08	8.02E-01	GPA33	12	rs922380	75600329	7.23E-01	N/A	-	3.28E-08	1.32E-01	2.17E-04	271	
2	rs1402774	82195241	2.08E-02	N/A	17	rs7211963	72208041	5.06E-01	JMJD6	-	3.30E-08	3.54E-01	9.67E-05	272	
6	rs9399364	1.42E+08	3.94E-01	N/A	10	rs1904382	65220692	1.59E-01	N/A	-	3.32E-08	8.32E-03	4.94E-09	273	
6	rs9403288	1.42E+08	3.94E-01	N/A	10	rs1904382	65220692	1.59E-01	N/A	-	3.32E-08	8.32E-03	4.94E-09	274	
2	rs12714081	82144466	1.29E-02	N/A	17	rs7211963	72208041	5.06E-01	JMJD6	-	3.32E-08	5.89E-01	4.23E-05	275	
1	rs2996016	41633970	2.03E-01	N/A	12	rs1807783	93504732	5.57E-01	TMCC3	-	3.34E-08	7.75E-01	7.59E-05	276	
3	rs7648359	1.36E+08	5.09E-01	EPHB1	13	s10507442	36245848	6.87E-01	N/A	-	3.36E-08	5.98E-01	1.74E-04	277	
6	rs2395471	31348671	7.52E-01	HLA-C	6	rs2253907	31444649	7.98E-01	HLA-B	MHC	3.36E-08	6.25E-02	1.08E-07	278	
6	rs9396677	16574200	5.82E-02	ATXN1	8	rs4871101	1.22E+08	1.17E-01	SNTB1	-	3.37E-08	2.35E-01	7.20E-05	279	
1	rs6662401	1.67E+08	7.53E-01	XCL1	22	rs2294209	30970057	6.97E-01	SLC5A4	-	3.37E-08	4.10E-01	9.48E-07	280	
2	rs12471723	2.19E+08	7.72E-02	IL8RBP	13	rs1164502	75766685	6.62E-01	N/A	-	3.38E-08	6.58E-01	3.40E-06	281	
6	rs9380215	31157634	4.29E-02	N/A	6	rs887464	31253899	8.59E-02	TCF19	MHC	3.39E-08	5.24E-01	2.73E-06	282	
1	rs11799843	1.85E+08	2.96E-01	MIR548F1	5	rs7715102	2427305	5.42E-01	N/A	-	3.39E-08	5.24E-01	2.73E-06	283	
1	rs3817586	1.85E+08	2.96E-01	MIR548F1	5	rs7715102	2427305	5.42E-01	N/A	-	3.39E-08	3.34E-02	6.39E-08	284	
1	rs1886690	39058140	5.37E-01	RRAGC	15	s12904751	52772402	8.03E-02	N/A	-	3.40E-08	9.10E-01	1.74E-05	285	
1	rs7541537	2.37E+08	8.14E-01	N/A	3	rs33036	1.61E+08	9.89E-01	SCHIP1	-	3.41E-08	9.84E-01	2.01E-05	286	
1	rs16858460	1.65E+08	8.22E-01	DUSP27	12	rs922380	75600329	7.23E-01	N/A	-	3.42E-08	1.02E-01	2.84E-04	287	
8	rs1002905	64020514	7.19E-01	NKAIN3	10	rs7091494	14887155	9.95E-01	CDNF	-	3.43E-08	4.97E-01	4.15E-06	288	
10	rs7903921	1.32E+08	8.20E-01	N/A	15	rs8030479	97712007	6.31E-01	LRRC28	-	3.48E-08	4.88E-01	6.91E-06	289	
2	rs1405193	1.85E+08	6.01E-01	N/A	18	rs955427	57041053	6.97E-01	N/A	-	3.50E-08	3.13E-02	2.48E-08	290	
2	rs7561814	1.69E+08	1.94E-01	N/A	12	s10877902	61330448	8.81E-01	PPM1H	-	3.51E-08	1.07E-01	7.08E-08	291	
6	rs1062470	31192414	5.89E-01	C6orf15	14	rs2809121	97504272	2.57E-01	C14orf64	MHC	3.51E-08	7.21E-01	2.45E-04	292	
3	rs4684717	10598391	7.53E-01	N/A	14	rs1983521	21688320	9.27E-02	N/A	-	3.51E-08	7.94E-01	3.54E-05	293	
6	rs3130559	31205280	7.50E-01	C6orf15	6	rs1131896	31487094	3.40E-01	MICA	MHC	3.55E-08	4.24E-01	3.46E-06	294	
1	rs11590309	79058813	1.11E-01	N/A	3	rs6790145	1.9E+08	9.06E-01	TPRG1	-	3.56E-08	5.73E-01	1.47E-05	295	
1	rs12733799	1.85E+08	2.68E-01	C1orf27	5	rs7715102	2427305	5.42E-01	N/A	-	3.57E-08	5.24E-01	2.82E-06	296	
1	rs2336938	2.05E+08	7.38E-01	N/A	19	s17361438	53540514	6.59E-01	TMEM143	-	3.60E-08	3.70E-01	1.92E-04	297	
2	rs7558942	2.42E+08	1.12E-01	THAP4	5	s12515734	35656957	3.74E-01	SPEF2	-	3.63E-08	1.42E-01	5.03E-07	298	
1	rs1093920	2.44E+08	9.05E-01	KIF26B	2	rs207431	31406898	6.50E-01	XDH	-	3.66E-08	4.65E-01	5.88E-05	299	
2	rs10933155	2.27E+08	5.33E-01	RHBDD1	21	rs1888469	38821374	9.91E-01	ERG	-	3.67E-08	1.45E-03	1.04E-09	300	
3	rs816151	1.26E+08	8.18E-01	SLC12A8	3	rs1430408	1.58E+08	2.53E-01	N/A	-	3.71E-08	7.14E-01	5.16E-05	301	
1	rs1410244	1.65E+08	4.30E-01	DUSP27	12	rs2669013	75547079	6.30E-01	N/A	-	3.71E-08	N/A	N/A	302	
1	rs10753949	1.89E+08	7.06E-01	N/A	10	rs7090538	1.27E+08	7.36E-01	CTBP2	-	3.72E-08	4.60E-01	9.30E-05	303	
1	rs11207036	57677441	2.77E-01	DAB1	5	s12515754	3398843	6.84E-01	N/A	-	3.73E-08	9.98E-01	2.11E-05	304	
6	rs10457834	1.49E+08	2.16E-01	N/A	9	rs7023466	92045628	8.06E-01	N/A	-	3.73E-08	5.36E-01	2.30E-05	305	
6	rs4897043	1.49E+08	2.16E-01	N/A	9	rs7023466	92045628	8.06E-01	N/A	-	3.73E-08	5.36E-01	2.30E-05	306	
1	rs4653175	36529078	4.78E-01	THRAP3	15	s12912234	27334602	9.54E-01	FAM189A1	-	3.74E-08	3.26E-01	1.96E-04	307	
14	rs10145210	53090485	3.84E-01	N/A	20	rs1736493	43964041	5.37E-01	PLTP	-	3.75E-08	N/A	N/A	308	
5	rs12523655	1.7E+08	5.99E-01	KCNIP1	7	s10215566	1.28E+08	8.76E-01	N/A	-	3.76E-08	N/A	N/A	309	
1	rs3013101	13672722	6.40E-01	N/A	13	rs1926845	22484809	5.50E-01	N/A	-	3.77E-08	6.56E-01	1.21E-04	310	
6	rs6911781	1.14E+08	3.17E-01	N/A	2	rs2367797	1.8E+08	8.78E-01	ZNF385B	-	3.77E-08	N/A	N/A	311	
1	rs1493081	2.27E+08	9.40E-01	N/A	7	rs7804628	18059883	4.19E-01	N/A	-	3.78E-08	9.00E-01	9.66E-06	312	
5	rs31226	53363328	3.70E-01	ARL15	17	rs3744000	71215928	4.53E-01	ITGB4	-	3.79E-08	8.29E-01	3.50E-05	313	
5	rs17457012	38792000	9.44E-01	N/A	10	rs4747211	73296229	7.50E-01	PSAP	-	3.79E-08	4.35E-01	2.28E-06	314	
1	rs3002307	2.13E+08	6.80E-01	PTPN14	13	s10492664	1.08E+08	6.04E-02	N/A	-	3.83E-08	6.64E-01	5.21E-05	315	
3	rs860309	1.96E+08	1.06E-01	N/A	9	rs6559443	80837613	3.04E-01	N/A	-	3.83E-08	N/A	N/A	316	
6	rs6921516	1.61E+08	4.89E-01	LPA	7	s10951991	6688186	5.78E-01	ZNF12	-	3.84E-08	N/A	N/A	317	
3	rs7613069	10600996	5.88E-01	N/A	14	rs2075493	21701518	7.45E-02	N/A	-	3.88E-08	6.69E-01	2.35E-04	318	
6	rs2233984	31187243	4.20E-02	C6orf15	6	rs3868542	31253818	6.85E-02	TCF19	MHC	3.88E-08	5.03E-02	1.22E-07	319	
3	rs7630274	20693128	3.67E-01	N/A	10	rs2253755	1.24E+08	4.10E-01	HTRA1	-	3.89E-08	2.95E-01	6.44E-04	320	
10	rs10998957	71198936	5.17E-01	N/A	22	rs6010044	49448804	4.82E-01	SHANK3	-	3.89E-08	3.46E-01	3.54E-04	321	
9	rs10814287	35850483	3.37E-01	TMEM8B	14	rs977870	22794987	1.31E-01	HOMEZ	-	3.90E-08	N/A	N/A	322	
2	rs1470504	77076318	7.97E-01	LRRTM4	5	rs7726783	3187719	3.47E-01	N/A	-	3.91E-08	6.61E-01	1.46E-05	323	
4	rs1517945	1.38E+08	4.72E-01	N/A	5	rs187462	1.09E+08	6.84E-01	N/A	-	3.91E-08	1.67E-01	6.00E-05	324	
5	rs1904163	52221753	7.92E-01	ITGA1	12	rs6581981	69680831	8.57E-01	N/A	-	3.92E-08	8.90E-01	1.55E-05	325	
6	rs2233965	31188878	4.20E-03	C6orf15	6	rs3131003	31201461	6.51E-01	C6orf15	MHC	3.94E-08	4.74E-01	2.31E-06	326	
4	rs2178603	85309773	8.34E-01	N/A	5	rs1344690	91190974	8.83E-01	N/A	-	3.96E-08	3.70E-01	1.01E-04	327	
3	rs11709533	66622318	4.47E-01	LRIG1	18	rs2132918	14586020	1.90E-01	N/A	-	3.98E-08	9.43E-01	1.81E-05	328	
4	rs3733548	24765581	8.25E-02	SEPSECS	5	rs6881214	24873656	6.85E-01	N/A	-	4.00E-08	7.48E-01	2.57E-05	329	
8	rs1548353	17211136	5.14E-01	MTMR7	15	rs767387	51385518	8.23E-01	N/A	-	4.00E-08	7.02E-01	1.90E-05	330	
6	rs7764600	1860827	7.88E-01	GMDS	7	rs337331	1.26E+08	4.71E-02	N/A	-	4.02E-08	2.95E-01	1.07E-04	331	
6	rs7764600	1860827	7.88E-01	GMDS	7	rs337335	1.26E+08	4.71E-02	N/A	-	4.02E-08	2.95E-01	1.07E-04	332	
7	rs6974901	47512004	5.04E-01	TNS3	10	rs1336197	56167665	1.58E-01	PCDH15	-	4.04E-08	2.96E-01	3.44E-07	333	
12	rs11063708	5464664	1.77E-01	NTF3	13	rs9558979	1.07E+08	8.79E-01	FAM155A	-	4.05E-08	9.34E-01	6.02E-06	334	

SNP A					SNP B						Interaction P			Ranking	Cluster in top 100 interactions
CHR	SNP	Location	gle locus P va	Gene	CHR	SNP	Location	Single locus P valu	Gene	HC region	Stage 1	Stage 2	Combined		
3	rs7630274	20693128	3.67E-01	N/A	10	rs2300433	1.24E+08	4.84E-01	HTRA1	-	4.06E-08	2.95E-01	6.45E-04	335	
8	rs2648883	1.29E+08	8.39E-01	PVT1	11	s11236552	75222813	1.91E-01	UVRAG	-	4.07E-08	7.35E-01	1.22E-04	336	
6	rs3132571	31013292	5.35E-02	DPCR1	6	rs1265086	31217861	3.70E-01	TCF19	MHC	4.08E-08	2.95E-02	6.04E-08	337	
1	rs474348	98683758	2.10E-01	N/A	9	s17083209	82166375	1.33E-01	N/A	-	4.10E-08	3.83E-01	4.20E-06	338	
2	rs16853057	1.68E+08	5.73E-01	XIRP2	19	rs889362	18619255	5.85E-02	KLHL26	-	4.11E-08	3.91E-01	2.28E-05	339	
12	rs825090	1.01E+08	3.89E-01	MYBPC1	14	rs2182977	43348830	1.33E-01	N/A	-	4.16E-08	6.57E-01	7.70E-06	340	
6	rs3805994	70823225	2.48E-01	COL19A1	8	rs2242093	1.45E+08	1.98E-02	ZC3H3	-	4.16E-08	5.85E-01	1.56E-05	341	
7	rs2280659	1.54E+08	4.99E-01	DPP6	8	rs4841005	8538640	4.39E-01	N/A	-	4.17E-08	3.16E-01	6.18E-07	342	
1	rs10889403	63334538	9.86E-01	N/A	11	s11041391	7432637	5.65E-01	SYT9	-	4.17E-08	8.42E-01	2.21E-06	343	
6	rs3805994	70823225	2.48E-01	COL19A1	8	s12679598	1.45E+08	1.98E-02	ZC3H3	-	4.18E-08	6.16E-01	1.36E-05	344	
9	rs10819907	97938109	8.46E-01	N/A	12	rs1196836	1.04E+08	3.24E-01	KIAA1033	-	4.18E-08	6.35E-01	8.92E-05	345	
6	rs2233965	31188878	4.20E-03	C6orf15	6	rs887464	31253899	8.59E-02	TCF19	MHC	4.19E-08	4.64E-01	3.47E-06	346	
3	rs762308	37740699	1.26E-02	ITGA9	4	rs2881015	1.1E+08	5.34E-01	COL25A1	-	4.19E-08	2.01E-01	2.52E-06	347	
5	rs12523655	1.7E+08	5.99E-01	KCNIP1	7	rs6958788	1.28E+08	8.77E-01	FAM71F2	-	4.21E-08	3.79E-01	2.50E-06	348	
6	rs6937783	1.06E+08	8.02E-01	N/A	2	s11682423	1.63E+08	7.51E-03	KCNH7	-	4.21E-08	2.97E-01	3.06E-06	349	
1	rs2686228	2.39E+08	9.03E-01	RGS7	12	s12228054	1.26E+08	7.53E-02	N/A	-	4.22E-08	5.89E-01	9.15E-05	350	
1	rs1268910	2.13E+08	3.18E-01	N/A	5	rs4320273	31072368	6.09E-05	N/A	-	4.22E-08	4.76E-01	1.17E-05	351	
2	rs12994560	75658556	9.34E-01	FAM176A	8	s11990002	79263139	1.94E-01	N/A	-	4.22E-08	6.80E-01	3.15E-05	352	
1	rs2249665	47459671	9.73E-01	TAL1	15	rs1579821	20501269	8.54E-01	CYFIP1	-	4.23E-08	4.22E-01	2.62E-05	353	
6	rs2237236	26559532	9.88E-01	BTN3A3	15	rs813299	29225595	8.06E-01	N/A	MHC	4.24E-08	7.83E-01	3.34E-06	354	
1	rs16860429	1.81E+08	6.09E-01	LAMC2	11	s10835057	26653876	2.24E-01	SLC5A12	-	4.24E-08	1.17E-01	7.75E-04	355	
11	rs1371351	96030639	6.33E-01	N/A	11	rs9633935	1.08E+08	4.46E-01	N/A	-	4.24E-08	N/A	N/A	356	
12	rs11063703	5459670	1.77E-01	NTF3	13	rs9558979	1.07E+08	8.79E-01	FAM155A	-	4.25E-08	6.29E-01	2.24E-06	357	
19	rs919803	35678944	1.57E-01	ZNF536	22	rs1024338	47919286	7.26E-02	N/A	-	4.26E-08	6.16E-01	5.82E-06	358	
4	rs4698742	14085628	5.31E-01	N/A	8	rs2978010	1.35E+08	8.56E-01	ST3GAL1	-	4.26E-08	1.04E-01	1.18E-07	359	
2	rs6712787	2.21E+08	4.12E-01	N/A	5	s10155508	1.36E+08	2.98E-01	N/A	-	4.27E-08	4.83E-01	4.26E-06	360	
2	rs7599725	2.21E+08	4.12E-01	N/A	5	s10155508	1.36E+08	2.98E-01	N/A	-	4.27E-08	4.83E-01	4.26E-06	361	
9	rs7025588	1.39E+08	9.67E-01	N/A	10	s11253229	5590202	6.02E-01	N/A	-	4.30E-08	2.19E-01	1.02E-06	362	
6	rs3130534	31317024	3.50E-01	N/A	6	rs7761965	31381474	9.15E-01	N/A	MHC	4.30E-08	N/A	N/A	363	
2	rs12694536	2.21E+08	4.14E-01	N/A	5	s10155508	1.36E+08	2.98E-01	N/A	-	4.31E-08	5.38E-01	6.70E-06	364	
3	rs2724693	1.39E+08	3.64E-01	N/A	8	rs904924	96599824	2.18E-01	N/A	-	4.31E-08	3.07E-01	5.37E-04	365	
7	rs599574	70776464	3.50E-01	WBSCR17	10	rs7896001	8397305	6.35E-01	N/A	-	4.33E-08	4.63E-01	9.51E-05	366	
7	rs599574	70776464	3.50E-01	WBSCR17	10	rs1892064	8401217	6.35E-01	N/A	-	4.33E-08	5.52E-01	7.02E-05	367	
12	rs12424340	96130353	3.48E-02	N/A	19	rs2334253	50808317	2.61E-01	N/A	-	4.35E-08	8.43E-01	1.73E-06	368	
5	rs10059955	1.05E+08	1.38E-01	N/A	12	s12824017	61935103	2.85E-01	N/A	-	4.37E-08	6.54E-01	1.21E-05	369	
6	rs3777932	1.13E+08	4.82E-02	LAMA4	14	rs2798819	94492794	9.86E-01	N/A	-	4.40E-08	6.05E-01	3.20E-06	370	
6	rs3798221	1.61E+08	6.44E-01	LPA	7	s10951991	6688186	5.78E-01	ZNF12	-	4.41E-08	2.00E-01	3.30E-07	371	
3	rs6781052	4576969	1.34E-01	ITPR1	3	rs2324938	86096826	5.01E-01	CADM2	-	4.42E-08	4.21E-01	1.99E-06	372	
1	rs2249636	47459120	5.73E-01	TAL1	15	rs1579821	20501269	8.54E-01	CYFIP1	-	4.46E-08	8.33E-01	4.45E-06	373	
6	rs4122189	31275906	7.70E-02	HCG27	6	rs7750269	31379136	8.58E-01	N/A	MHC	4.48E-08	3.66E-01	2.58E-06	374	
1	rs9287077	1.65E+08	8.02E-01	GPA33	12	rs2632226	75535673	3.99E-01	N/A	-	4.48E-08	5.52E-01	1.63E-05	375	
12	rs1468894	96058471	6.16E-01	N/A	19	rs2334253	50808317	2.61E-01	N/A	-	4.48E-08	5.67E-01	1.97E-05	376	
17	rs17247848	28008297	5.07E-01	MYO1D	18	rs3911593	46436326	7.35E-01	MAPK4	-	4.49E-08	3.76E-01	5.28E-04	377	
2	rs12714081	82144466	1.29E-02	N/A	17	rs2240774	72222167	7.40E-01	JMJD6	-	4.49E-08	4.42E-01	1.33E-04	378	
10	rs10764818	1.31E+08	6.80E-01	N/A	15	rs1558390	95320425	1.04E-01	N/A	-	4.50E-08	9.00E-01	2.58E-05	379	
3	rs7632399	1.23E+08	5.33E-01	CASR	10	rs2589446	56634203	2.40E-01	N/A	-	4.51E-08	8.09E-01	2.19E-05	380	
1	rs12042641	1.65E+08	5.58E-01	GPA33	12	rs922380	75600329	7.23E-01	N/A	-	4.52E-08	1.39E-01	2.51E-04	381	
9	rs10819907	97938109	8.46E-01	N/A	12	rs1196865	1.04E+08	3.01E-01	KIAA1033	-	4.53E-08	6.35E-01	9.54E-05	382	
8	rs4876860	1.2E+08	2.21E-01	N/A	13	rs2985947	45056322	2.08E-02	FAM194B	-	4.54E-08	6.28E-02	1.53E-03	383	
1	rs3002307	2.13E+08	6.80E-01	PTPN14	13	s12584299	1.08E+08	7.00E-02	N/A	-	4.54E-08	6.30E-01	6.66E-05	384	
13	rs7986907	78628865	9.19E-01	N/A	15	rs4886616	71876786	5.12E-01	N/A	-	4.55E-08	6.62E-01	4.25E-06	385	
8	rs4841530	11326556	7.06E-01	C8orf12	15	s11247349	96851676	8.76E-01	FAM169B	-	4.56E-08	3.19E-01	4.00E-07	386	
6	rs9404133	1.02E+08	3.61E-01	GRIK2	5	s16868534	34893202	1.12E-02	TTC23L	-	4.58E-08	9.39E-01	4.57E-06	387	
7	rs10487781	14835730	4.38E-01	DGKB	7	s17154507	1.07E+08	8.30E-01	N/A	-	4.58E-08	2.35E-03	1.43E-09	388	
4	rs7700133	1.86E+08	1.53E-02	N/A	14	rs179724	30553512	8.70E-01	STRN3	-	4.58E-08	7.39E-01	1.02E-05	389	
1	rs10489397	1.85E+08	6.96E-01	N/A	5	rs7715102	2427305	5.42E-01	N/A	-	4.59E-08	2.92E-01	1.06E-06	390	
4	rs10010758	37614913	9.45E-01	TBC1D1	18	rs8092807	3013264	3.23E-01	LPIN2	-	4.59E-08	6.69E-01	4.49E-06	391	
6	rs1555772	1.22E+08	4.39E-01	N/A	3	rs524431	74383584	3.74E-01	CNTN3	-	4.60E-08	1.80E-02	3.12E-08	392	
8	rs1077701	6457008	3.44E-01	MCPH1	15	rs6492914	37822048	4.15E-02	FSIP1	-	4.60E-08	5.26E-01	3.47E-04	393	
1	rs950302	1.65E+08	5.39E-01	DUSP27	12	rs2669013	75547079	6.30E-01	N/A	-	4.60E-08	N/A	N/A	394	
4	rs17040984	1.63E+08	9.50E-02	FSTL5	8	rs6586726	18144500	4.58E-01	N/AT1	-	4.60E-08	2.52E-01	7.57E-07	395	
9	rs2383022	17475598	2.76E-01	CNTLN	12	s10847417	1.27E+08	9.96E-01	N/A	-	4.61E-08	5.36E-01	1.92E-04	396	
8	rs2648883	1.29E+08	8.39E-01	PVT1	11	s12280464	75246798	1.67E-01	UVRAG	-	4.63E-08	9.31E-01	9.31E-05	397	
6	rs6923877	1.61E+08	5.49E-01	LPA	7	s10951991	6688186	5.78E-01	ZNF12	-	4.64E-08	2.00E-01	3.44E-07	398	
6	rs9378310	1671161	4.51E-02	GMDS	2	s10209739	13290142	2.31E-01	N/A	-	4.64E-08	5.37E-01	2.07E-04	399	
6	rs9378310	1671161	4.51E-02	GMDS	2	rs7573456	13292214	2.31E-01	N/A	-	4.64E-08	5.37E-01	2.07E-04	400	
5	rs11134856	1.74E+08	3.40E-01	N/A	9	rs1331885	28332179	1.93E-02	LINGO2	-	4.64E-08	4.33E-01	2.95E-04	401	
1	rs614448	1.17E+08	2.89E-01	N/A	3	rs4679208	1.27E+08	3.62E-01	ALDH1L1	-	4.67E-08	5.88E-01	1.47E-04	402	
6	rs6455688	1.61E+08	6.19E-01	LPA	7	s10951991	6688186	5.78E-01	ZNF12	-	4.67E-08	2.00E-01	3.48E-07	403	
8	rs16893262	87293814	1.58E-01	SLC7A13	20	rs1005351	7020555	8.94E-01	N/A	-	4.70E-08	2.77E-01	5.67E-04	404	
6	rs1006012	53966408	8.93E-01	N/A	1	rs1188483	2.27E+08	3.41E-01	TRIM17	-	4.71E-08	8.88E-01	2.53E-05	405	
11	rs508205	1.2E+08	4.93E-01	N/A	22	rs228918	35836626	9.71E-01	TMPRSS6	-	4.72E-08	2.94E-01	2.39E-04	406	
9	rs7867457	78427138	6.15E-01	PRUNE2	13	rs1572371	19785860	4.76E-01	N/A	-	4.74E-08	6.79E-01	4.27E-06	407	
2	rs17013592	77088275	7.17E-01	LRRTM4	5	rs597099	3192158	7.14E-01	N/A	-	4.75E-08	7.29E-01	3.00E-05	408	
2	rs883844	1.92E+08	8.59E-01	GLS	2	rs4332918	2.28E+08	2.15E-01	COL4A3	-	4.75E-08	6.89E-01	5.86E-06	409	
11	rs12574854	1.28E+08	7.65E-01	N/A	19	rs2033259	17557689	8.20E-01	GLT25D1	-	4.78E-08	4.45E-01	2.84E-04	410	
7	rs6973800	94202716	3.00E-01	N/A	11	rs1996352	1.02E+08	4.37E-01	MMP7	-	4.78E-08	5.03E-01	1.39E-06	411	
1	rs2990678	13709800	6.50E-01	N/A	8	rs3802269	12633550	6.72E-01	LONRF1	-	4.79E-08	4.31E-01	1.14E-04	412	
3	rs2622717	1.39E+08	3.26E-01	N/A	8	rs904924	96599824	2.18E-01	N/A	-	4.79E-08	3.07E-01	5.78E-04	413	
17	rs9910055	39638563	5.02E-01	C17orf65	19	rs483808	7863481	2.21E-01	N/A	-	4.79E-08	7.81E-01	3.19E-06	414	
3	rs1381065	1.17E+08	6.02E-01	LSAMP	7	rs219822	98427987	1.21E-01	TRRAP	-	4.79E-08	4.13E-01	7.93E-06	415	
3	rs6764210	36237237	5.32E-01	N/A	16	rs6500892	6953116	9.44E-01	A2BP1	-	4.79E-08	5.26E-01	6.17E-06	416	
14	rs9323099	44019800	4.11E-01	N/A	14	rs7156399	95104682	7.38E-01	N/A	-	4.80E-08	3.30E-01	6.16E-05	417	
4	rs11097059	86683004	6.51E-01	ARHGAP24	13	rs3783257	29004466	1.48E-01	SLC7A1	-	4.80E-08	6.04E-01	1.58E-05	418	
4	rs11097059	86683004	6.51E-01	ARHGAP24	13	rs3783260	29000167	1.48E-01	SLC7A1	-	4.80E-08	6.04E-01	1.58E-05	419	
4	rs11097059	86683004	6.51E-01	ARHGAP24	13	rs3825536	29001194	1.48E-01	SLC7A1	-	4.80E-08	6.04E-01	1.58E-05	420	
8	rs2019131	1.38E+08	9.48E-01	N/A	17	rs1390247	4307913	1.67E-02	SPNS3	-	4.81E-08	2.36E-01	8.26E-08	421	
1	rs7523434	1.65E+08	1.28E-01	GPA33	12	rs2669013	75547079	6.30E-01	N/A	-	4.81E-08	N/A	N/A	422	
1	rs2281960	1.65E+08	6.11E-01	GPA33	12	rs922380	75600329	7.23E-01	N/A	-	4.82E-08	1.36E-01	2.71E-04	423	
1	rs2281962	1.65E+08	6.11E-01	GPA33	12	rs922380	75600329	7.23E-01	N/A	-	4.82E-08	1.36E-01	2.71E-04	424	
9	rs1663762	74466718	1.36E-01	TMC1	17	rs2323661	15241034	1.56E-01	N/A	-	4.82E-08	3.71E-01	4.10E-04	425	
2	rs1470504	77076318	7.97E-01	LRRTM4	5	rs160890	3187879	9.49E-01	N/A	-	4.82E-08	7.57E-01	4.51E-06	426	
2	rs12618307	10414395	8.04E-01	HPCAL1	12	rs4761378	76330817	6.54E-02	N/A	-	4.84E-08	5.70E-01	1.33E-05	427	
1	rs1287634	5847958	3.75E-01	NPHP4	12	s10848943	3814283	7.50E-01	PARP11	-	4.85E-08	8.14E-01	2.08E-05	428	
4	rs4862432	1.86E+08	2.49E-02	N/A	14	rs179724	30553512	8.70E-01	STRN3	-	4.85E-08	8.56E-01	1.65E-05	429	
12	rs1872521	1.27E+08	8.77E-01	N/A	22	rs4820234	35059020	3.19E-01	MYH9	-	4.85E-08	3.56E-01	2.43E-06	430	
6	rs2032544	1.67E+08	5.68E-01	RPS6KA2	12	s12580153	91342613	1.82E-01	CLLU1	-	4.85E-08	1.89E-01	2.55E-04	431	
12	rs10506938	85833028	7.43E-01	N/A	16	rs4997772	80462796	1.94E-01	PLCG2	-	4.85E-08	9.40E-01	3.62E-05	432	
12	rs2897395	85829951	7.43E-01	N/A	16	rs4997772	80462796	1.94E-01	PLCG2	-	4.85E-08	9.62E-01	2.69E-05	433	
7	rs6972380	1.21E+08	7.32E-01	N/A	17	rs1388164	29068522	6.13E-01	ACCN1	-	4.86E-08	7.85E-01	1.04E-05	434	
1	rs281966	91511350	9.12E-01	HFM1	2	s13386612	77841057	5.99E-01	N/A	-	4.86E-08	8.91E-01	4.05E-05	435	
1	rs281968	91507901	9.12E-01	HFM1	2	s13386612	77841057	5.99E-01	N/A	-	4.86E-08	9.66E-01	5.21E-05	436	
6	rs2636597	1.34E+08	1.99E-01	N/A	13	rs9634445	96921033	8.02E-01	RAP2A	-	4.87E-08	9.05E-01	4.99E-05	437	
7	rs10229295	1.21E+08	6.96E-01	N/A	17	rs1388164	29068522	6.13E-01	ACCN1	-	4.88E-08	7.95E-01	9.59E-06	438	
3	rs4684717	10598391	7.53E-01	N/A	14	s12433895	21692350	8.97E-02	N/A	-	4.88E-08	8.04E-01	4.40E-05	439	
12	rs10506937	85825624	7.99E-01	N/A	16	rs4997772	80462796	1.94E-01	PLCG2	-	4.88E-08	8.69E-01	5.25E-05	440	
5	rs2089192	1.7E+08	5.56E-01	KCNIP1	7	s10215566	1.28E+08	8.76E-01	N/A	-	4.88E-08	N/A	N/A	441	
6	rs4122189	31275906	7.70E-02	HCG27	6	rs9368675	31380136	7.91E-01	N/A	MHC	4.89E-08	3.66E-01	2.73E-06	442	
6	rs4495304	31188697	4.80E-02	C6orf15	6	rs3868542	31253818	6.85E-02	TCF19	MHC	4.89E-08	5.03E-02	1.47E-07	443	
3	rs1400166	7193175	3.14E-01	GRM7	15	rs6598416	96676803	9.34E-01	N/A	-	4.90E-08	3.91E-01	1.40E-06	444	
14	rs4074077	1.04E+08	8.74E-01	N/A	21	rs1557275	31247458	5.84E-02	N/A	-	4.90E-08	4.34E-01	6.36E-06	445	
9	rs10819907	97938109	8.46E-01	N/A	12	rs1196804	1.04E+08	2.93E-01	KIAA1033	-	4.91E-08	6.35E-01	9.90E-05	446	

SNP A					SNP B						Interaction P			Ranking	Cluster in top 100 interactions
CHR	SNP	Location	gle locus P va	Gene	CHR	SNP	Location	Single locus P valu	Gene	MHC region	Stage 1	Stage 2	Combined		
5	rs12523655	1.7E+08	5.99E-01	KCNIP1	7	rs1109552	1.28E+08	8.96E-01	FAM71F2	-	4.93E-08	3.36E-01	2.19E-06	447	
9	rs7044405	35860001	1.82E-01	TMEM8B	14	rs2144039	22799242	2.41E-01	HOMEZ	-	4.95E-08	6.29E-01	1.33E-04	448	
3	rs7651261	1.83E+08	3.46E-01	N/A	18	rs273363	30848701	1.25E-02	MAPRE2	-	4.95E-08	8.17E-01	9.67E-06	449	
12	rs4930767	5467642	2.06E-01	NTF3	13	rs9558979	1.07E+08	8.79E-01	FAM155A	-	4.96E-08	8.87E-01	5.80E-06	450	
11	rs11820229	59959174	8.62E-01	MS4A14	18	rs920783	42152426	1.23E-01	RNF165	-	4.96E-08	3.24E-01	3.74E-07	451	
1	rs2294723	1.7E+08	1.53E-01	DNM3	7	rs7799650	98683305	1.13E-02	N/A	-	4.97E-08	5.07E-01	7.54E-06	452	
1	rs2294723	1.7E+08	1.53E-01	DNM3	7	rs4255064	98684620	1.13E-02	N/A	-	4.97E-08	5.09E-01	7.52E-06	453	
12	rs10128762	72968165	7.81E-01	N/A	21	rs928250	17977401	4.43E-01	N/A	-	4.97E-08	5.70E-03	9.21E-08	454	
8	rs12676446	18560492	3.77E-01	PSD3	9	rs1969944	1.12E+08	8.02E-01	PALM2-AKAP2	-	4.97E-08	5.24E-01	3.47E-06	455	
9	rs4146797	11941983	7.65E-01	N/A	22	rs5992838	16644831	1.46E-01	BID	-	4.98E-08	7.79E-02	1.06E-07	456	
1	rs1354221	2.15E+08	6.49E-01	ESRRG	3	rs2257056	9163615	6.52E-01	SRGAP3	-	5.00E-08	2.09E-01	5.23E-07	457	
13	rs9512365	26218189	8.63E-01	GPR12	22	rs5993935	18600764	6.79E-01	MIR1286	-	5.02E-08	9.38E-01	2.25E-05	458	
11	rs2186760	95730814	7.60E-01	MAML2	15	rs2573612	98271513	1.21E-01	N/A	-	5.04E-08	5.40E-01	8.95E-06	459	
9	rs11791889	35850942	3.60E-01	TMEM8B	14	rs977870	22794987	1.31E-01	HOMEZ	-	5.04E-08	N/A	N/A	460	
5	rs4700718	1.8E+08	3.35E-01	RASGEF1C	8	rs13267425	1.02E+08	2.72E-02	N/A	-	5.04E-08	7.08E-01	3.02E-05	461	
4	rs1433634	66440061	3.55E-01	N/A	12	rs16946571	1.15E+08	2.61E-01	N/A	-	5.05E-08	5.58E-01	5.27E-05	462	
4	rs6551968	66433172	3.55E-01	N/A	12	rs16946571	1.15E+08	2.61E-01	N/A	-	5.05E-08	5.58E-01	5.27E-05	463	
2	rs7566955	1.41E+08	1.25E-02	N/A	12	rs10877090	56963223	1.71E-01	N/A	-	5.05E-08	5.06E-01	1.84E-04	464	
7	rs7806531	70661813	1.54E-01	WBSCR17	9	rs2300932	1.23E+08	6.11E-01	C5	-	5.06E-08	3.49E-02	1.92E-08	465	
1	rs1808806	1.18E+08	8.04E-01	FAM46C	2	rs820780	57756125	3.10E-01	N/A	-	5.06E-08	6.20E-01	4.23E-05	466	
12	rs20508	96606718	5.71E-01	N/A	15	rs12594522	30855341	1.35E-01	FMN1	-	5.06E-08	3.35E-01	1.83E-06	467	
10	rs7908946	1.28E+08	8.46E-01	ADAM12	19	rs1849003	21934101	6.55E-01	ZNF208	-	5.07E-08	2.63E-01	5.82E-04	468	
10	rs4993019	1.33E+08	6.54E-01	N/A	15	rs1036003	31828733	9.82E-01	RYR3	-	5.07E-08	6.46E-01	5.88E-06	469	
10	rs4993019	1.33E+08	6.54E-01	N/A	15	rs2278311	31829265	9.82E-01	RYR3	-	5.07E-08	6.46E-01	5.88E-06	470	
4	rs17074371	1.84E+08	4.00E-01	N/A	16	rs12149621	80702549	1.52E-01	HSD17B2	-	5.07E-08	1.29E-01	3.72E-08	471	
17	rs12450534	61762419	9.72E-01	PRKCA	20	rs6107832	655880	3.32E-01	N/A	-	5.08E-08	4.98E-01	1.50E-04	472	
2	rs2298873	1393966	8.05E-01	TPO	17	rs9898832	29926052	3.40E-01	TMEM132E	-	5.08E-08	9.44E-01	1.73E-05	473	
4	rs11726511	24919158	5.11E-01	ZCCHC4	9	rs626713	1.35E+08	2.50E-01	RALGDS	-	5.10E-08	1.22E-01	4.79E-04	474	
1	rs10903122	25176163	2.26E-01	RUNX3	15	rs12907348	46978032	9.33E-01	SHC4	-	5.11E-08	1.43E-01	4.93E-04	475	
1	rs1609996	25174456	2.26E-01	RUNX3	15	rs12907348	46978032	9.33E-01	SHC4	-	5.11E-08	1.43E-01	4.93E-04	476	
1	rs6673606	1.81E+08	6.15E-01	LAMC2	11	rs10835057	26653876	2.24E-01	SLC5A12	-	5.12E-08	8.58E-01	4.85E-06	477	
12	rs11059460	1.27E+08	2.00E-01	N/A	18	rs1893523	41735073	3.29E-01	KIAA1632	-	5.13E-08	4.35E-01	2.40E-04	478	
2	rs1470504	77076318	7.97E-01	LRRTM4	5	rs1038046	3198652	5.89E-01	N/A	-	5.13E-08	8.79E-01	5.38E-06	479	
5	rs1030179	1.23E+08	6.89E-01	N/A	10	rs981155	58243244	1.71E-01	N/A	-	5.14E-08	3.68E-01	1.13E-04	480	
1	rs2806403	58296573	4.23E-01	DAB1	1	rs155552	1.81E+08	6.99E-01	RGSL1	-	5.18E-08	2.60E-01	9.76E-04	481	
7	rs10233816	17941651	6.54E-01	SNX13	14	rs734606	94272240	2.92E-01	N/A	-	5.20E-08	7.38E-01	6.98E-05	482	
7	rs10233816	17941651	6.54E-01	SNX13	14	rs8006054	94273061	2.92E-01	N/A	-	5.20E-08	7.38E-01	6.98E-05	483	
3	rs10514695	36283102	3.53E-01	N/A	16	rs6500892	6953116	9.44E-01	A2BP1	-	5.21E-08	3.45E-01	2.15E-06	484	
1	rs6683416	1.85E+08	7.18E-01	PLA2G4A	18	rs2023096	69807945	3.84E-01	N/A	-	5.21E-08	4.93E-01	1.53E-06	485	
1	rs1832787	18499504	2.02E-01	IGSF21	3	rs1440512	29707866	7.32E-01	N/A	-	5.22E-08	6.78E-01	7.34E-06	486	
6	rs1796520	26518779	8.92E-01	BTN3A1	15	rs1378847	29234640	5.84E-01	N/A	MHC	5.23E-08	7.06E-01	6.45E-06	487	
18	rs12961809	55117108	8.71E-01	CPLX4	20	rs6061396	59971956	8.75E-01	TAF4	-	5.23E-08	3.34E-01	1.45E-04	488	
2	rs1346722	1.68E+08	6.75E-01	XIRP2	19	rs889363	18622276	1.44E-01	KLHL26	-	5.24E-08	6.98E-02	5.07E-04	489	
6	rs9480096	1.51E+08	4.85E-01	PPP1R14C	5	rs880944	53827131	1.23E-01	N/A	-	5.24E-08	6.08E-01	5.28E-07	490	
2	rs2894593	2.27E+08	3.87E-01	N/A	5	rs6885825	31125851	8.70E-01	N/A	-	5.28E-08	9.91E-01	8.15E-06	491	
7	rs10215210	88206842	9.61E-01	ZNF804B	13	rs9508666	29645725	6.46E-01	N/A	-	5.29E-08	N/A	N/A	492	
5	rs4704506	77690583	5.04E-01	SCAMP1	11	rs826044	73445145	5.08E-01	C2CD3	-	5.30E-08	7.49E-01	9.41E-06	493	
4	rs1105912	25677116	7.90E-01	N/A	9	rs10992658	94958464	6.02E-01	N/A	-	5.30E-08	6.52E-01	6.68E-06	494	
5	rs3816325	34718524	2.08E-01	RAI14	16	rs11646351	5473293	4.19E-01	N/A	-	5.30E-08	6.93E-01	1.89E-05	495	
2	rs4505564	2.41E+08	3.55E-01	N/A	7	rs12718545	51777395	2.99E-01	N/A	-	5.31E-08	2.14E-01	1.41E-06	496	
1	rs12567677	2.39E+08	6.75E-01	RGS7	11	rs2640738	1.08E+08	8.73E-01	EXPH5	-	5.31E-08	4.88E-01	1.25E-06	497	
11	rs1378357	11758937	8.32E-02	N/A	12	rs17107607	68731117	7.94E-01	N/A	-	5.32E-08	6.13E-01	1.35E-05	498	
8	rs2648883	1.29E+08	8.39E-01	PVT1	11	rs10219339	75162498	1.45E-01	DGAT2	-	5.33E-08	7.60E-01	1.40E-04	499	
6	rs2636597	1.34E+08	1.99E-01	N/A	13	rs1159278	96883350	9.56E-01	RAP2A	-	5.33E-08	8.51E-01	4.12E-05	500	
5	rs11749226	53989021	4.52E-01	N/A	9	rs1326930	86275002	2.61E-01	N/A	-	5.35E-08	1.63E-01	1.90E-04	501	
2	rs6760087	2.05E+08	8.79E-01	PARD3B	19	rs4807863	6257971	8.37E-02	ACER1	-	5.36E-08	1.19E-01	6.88E-07	502	
8	rs7825721	82397176	2.99E-01	N/A	8	rs2218609	94224423	5.53E-01	N/A	-	5.36E-08	N/A	N/A	503	
8	rs7841723	82397098	2.99E-01	N/A	8	rs2218609	94224423	5.53E-01	N/A	-	5.36E-08	N/A	N/A	504	
2	rs12471133	1.56E+08	6.61E-01	N/A	4	rs4692256	27353816	7.45E-01	N/A	-	5.36E-08	1.21E-01	3.36E-03	505	
6	rs4947172	1.13E+08	4.19E-02	LAMA4	14	rs2798819	94492794	9.86E-01	N/A	-	5.37E-08	4.44E-01	1.94E-06	506	
6	rs6937889	6704016	9.78E-01	N/A	14	rs11159198	75937134	4.39E-02	ESRRB	-	5.38E-08	N/A	N/A	507	
5	rs4704506	77690583	5.04E-01	SCAMP1	11	rs1800849	73397813	6.34E-01	UCP3	-	5.38E-08	7.44E-01	7.68E-06	508	
10	rs4917420	1.06E+08	2.66E-01	N/A	12	rs11169821	50062992	6.53E-01	GALNT6	-	5.38E-08	2.82E-02	5.91E-08	509	
2	rs13390436	25524281	2.01E-01	DTNB	3	rs1092103	1.46E+08	1.72E-01	N/A	-	5.38E-08	3.50E-01	8.78E-04	510	
3	rs7632399	1.23E+08	5.33E-01	CASR	10	rs2120763	56645255	3.94E-01	N/A	-	5.39E-08	9.09E-01	1.99E-05	511	
3	rs7632399	1.23E+08	5.33E-01	CASR	10	rs2680327	56663822	3.94E-01	N/A	-	5.39E-08	9.13E-01	2.00E-05	512	
6	rs9355586	1.67E+08	6.60E-01	RPS6KA2	12	rs406117	91323127	2.14E-01	CLLU1	-	5.40E-08	2.64E-01	3.48E-04	513	
1	rs633995	1.7E+08	6.98E-01	DNM3	10	rs1360817	1938864	3.21E-01	N/A	-	5.40E-08	3.66E-02	7.91E-03	514	
5	rs2089192	1.7E+08	5.56E-01	KCNIP1	7	rs6958788	1.28E+08	8.77E-01	FAM71F2	-	5.46E-08	3.61E-01	2.55E-06	515	
3	rs1225047	1.33E+08	1.58E-01	CPNE4	19	rs8108738	18116359	3.36E-01	MAST3	-	5.49E-08	2.67E-01	6.93E-04	516	
9	rs10819907	97938109	8.46E-01	N/A	12	rs1196814	1.04E+08	2.67E-01	KIAA1033	-	5.50E-08	6.35E-01	1.07E-04	517	
8	rs2648883	1.29E+08	8.39E-01	PVT1	11	rs1458836	75202444	1.68E-01	DGAT2	-	5.52E-08	7.35E-01	1.43E-04	518	
8	rs17222409	13430193	3.86E-01	DLC1	12	rs7136562	1.26E+08	2.26E-01	N/A	-	5.53E-08	5.65E-02	1.61E-07	519	
1	rs523744	2.44E+08	8.55E-01	KIF26B	2	rs207431	31406898	6.50E-01	XDH	-	5.54E-08	4.94E-01	6.92E-05	520	
6	rs2237238	1.13E+08	4.12E-02	LAMA4	14	rs2798819	94492794	9.86E-01	N/A	-	5.54E-08	5.15E-01	2.58E-06	521	
2	rs2457407	2.21E+08	8.86E-01	N/A	16	rs7200316	5860065	5.46E-01	N/A	-	5.55E-08	5.00E-01	4.13E-06	522	
5	rs2632151	1.13E+08	2.65E-01	N/A	10	rs7893835	1.22E+08	6.43E-01	N/A	-	5.55E-08	7.11E-01	2.16E-05	523	
6	rs3130712	31317489	3.95E-01	N/A	6	rs9394047	31344229	1.23E-01	HLA-C	MHC	5.56E-08	7.81E-01	5.78E-06	524	
6	rs12216269	1.38E+08	9.09E-01	N/A	20	rs6074422	12357264	3.92E-01	N/A	-	5.57E-08	3.95E-01	2.03E-04	525	
11	rs660375	1.07E+08	1.13E-01	N/A	17	rs16962555	61059850	6.18E-01	CCDC46	-	5.58E-08	9.51E-01	9.81E-06	526	
6	rs2179560	52196142	7.62E-01	IL17F	1	rs10157110	15215681	2.82E-01	KIAA1026	-	5.58E-08	4.94E-01	1.05E-05	527	
7	rs799084	42992986	5.12E-03	N/A	14	rs12588834	60770795	1.15E-01	N/A	-	5.58E-08	1.78E-01	3.09E-04	528	
2	rs2304401	11678760	4.93E-01	GREB1	3	rs10510382	7972953	2.32E-01	N/A	-	5.58E-08	2.85E-01	1.99E-04	529	
1	rs6657936	58301408	4.86E-01	DAB1	1	rs155552	1.81E+08	6.99E-01	RGSL1	-	5.58E-08	3.21E-01	8.75E-04	530	
4	rs3733545	25287494	4.72E-02	N/A	10	rs10826248	60338567	9.22E-01	N/A	-	5.59E-08	7.93E-01	7.36E-05	531	
1	rs3002307	2.13E+08	6.80E-01	PTPN14	13	rs11069721	1.08E+08	5.06E-02	N/A	-	5.59E-08	6.75E-01	6.62E-05	532	
3	rs4684717	10598391	7.53E-01	N/A	14	rs10872983	21691822	1.07E-01	N/A	-	5.61E-08	7.94E-01	4.66E-05	533	
2	rs291329	2.4E+08	9.20E-01	HDAC4	7	rs7787734	7618897	2.88E-01	MIOS	-	5.64E-08	2.18E-01	4.79E-04	534	
2	rs12994560	75658556	9.34E-01	FAM176A	8	rs10095615	79276255	1.72E-01	N/A	-	5.66E-08	6.14E-01	4.64E-05	535	
6	rs396243	31383153	2.17E-01	N/A	6	rs2253907	31444849	7.98E-01	HLA-B	MHC	5.67E-08	6.00E-01	1.41E-05	536	
8	rs2648883	1.29E+08	8.39E-01	PVT1	11	rs7108178	75221946	2.16E-01	UVRAG	-	5.67E-08	7.71E-01	1.28E-04	537	
6	rs1624440	26541308	9.34E-01	BTN3A3	15	rs813299	29225595	8.06E-01	N/A	MHC	5.70E-08	1.85E-01	4.81E-04	538	
10	rs11244909	1.28E+08	4.97E-01	ADAM12	13	rs9535618	50681315	1.59E-01	FAM124A	-	5.71E-08	1.57E-01	5.06E-07	539	
1	rs863455	1.66E+08	1.66E-02	CD247	4	rs878456	38208152	6.65E-01	N/A	-	5.71E-08	3.86E-03	1.35E-02	540	
2	rs1979043	1.01E+08	4.86E-01	TBC1D8	7	rs10954664	81613491	8.67E-01	CACN/A2D1	-	5.72E-08	5.93E-01	4.19E-05	541	
4	rs11735774	62239467	5.66E-01	LPHN3	9	rs10809144	10637333	3.47E-01	N/A	-	5.72E-08	7.17E-01	1.73E-05	542	
8	rs1351424	1.36E+08	8.70E-01	N/A	13	rs1340741	68418063	2.94E-01	N/A	-	5.73E-08	2.50E-02	5.25E-08	543	
1	rs6593732	1.45E+08	6.06E-02	N/A	2	rs935865	1.75E+08	7.80E-01	CHN1	-	5.75E-08	6.29E-01	2.44E-04	544	
2	rs10497873	2.04E+08	5.35E-01	N/A	7	rs11760550	54075875	1.22E-01	N/A	-	5.75E-08	3.69E-01	1.06E-04	545	
13	rs1171080	35385286	5.95E-01	DCLK1	13	rs1283207	1E+08	8.76E-01	N/A	-	5.78E-08	2.65E-01	2.30E-06	546	
6	rs4947296	31166157	4.31E-02	N/A	6	rs4122189	31275906	7.70E-02	HCG27	MHC	5.80E-08	1.68E-01	8.28E-07	547	
15	rs2034247	78399486	8.59E-01	N/A	19	rs2288887	57187615	4.93E-01	ZNF350	-	5.83E-08	4.59E-01	1.05E-06	548	
3	rs1817789	26686295	6.08E-01	LRRC3B	5	rs7380062	1.39E+08	2.47E-01	ECSCR	-	5.85E-08	7.38E-01	1.61E-04	549	
12	rs7970351	5191067	9.81E-01	N/A	17	rs7210806	59843219	8.77E-01	N/A	-	5.86E-08	7.82E-01	1.32E-04	550	
1	rs10889271	62173075	8.63E-01	IN/ADL	3	rs7628487	1.3E+08	9.73E-01	N/A	-	5.86E-08	3.74E-01	3.47E-06	551	
16	rs387138	76525505	2.56E-01	VAT1L	22	rs17004824	22778398	3.48E-01	CABIN1	-	5.90E-08	8.61E-01	5.55E-05	552	
9	rs10991224	1.06E+08	9.76E-01	N/A	14	rs417054	29617886	7.89E-01	N/A	-	5.91E-08	7.73E-01	6.39E-06	553	
3	rs6776685	35708268	5.08E-01	ARPP-21	9	rs7853654	1.33E+08	5.42E-01	ABL1	-	5.92E-08	8.13E-01	6.76E-05	554	
3	rs6776486	59383218	8.15E-01	N/A	4	rs10033037	1.15E+08	3.38E-02	CAMK2D	-	5.93E-08	3.41E-01	3.32E-06	555	
8	rs7002130	1.39E+08	3.23E-01	N/A	10	rs7897	1.04E+08	6.62E-01	NOLC1	-	5.94E-08	9.98E-01	2.86E-05	556	
1	rs1385665	1.15E+08	9.23E-01	N/A	9	rs7854368	1.14E+08	1.57E-02	N/A	-	5.95E-08	6.55E-01	1.59E-05	557	
10	rs7908946	1.28E+08	8.46E-01	ADAM12	19	rs1859093	21935179	6.73E-01	ZNF208	-	5.99E-08	2.51E-01	6.75E-04	558	

SNP A					SNP B						Interaction P			Ranking	Cluster in top 100 interactions
CHR	SNP	Location	gle locus P va	Gene	CHR	SNP	Location	Single locus P valu	Gene	MHC region	Stage 1	Stage 2	Combined		
3	rs9290235	1.66E+08	1.91E-01	N/A	17	rs1867295	50109062	8.79E-01	N/A	-	5.99E-08	1.42E-01	6.55E-04	559	
4	rs2132834	91787002	9.39E-01	FAM190A	17	rs8081176	75898582	3.89E-01	RNF213	-	6.01E-08	1.58E-01	4.43E-07	560	
2	rs12615696	4940344	5.19E-01	N/A	5	rs672413	78313985	6.12E-01	ARSB	-	6.01E-08	1.08E-01	1.53E-07	561	
2	rs4144195	4935066	5.19E-01	N/A	5	rs672413	78313985	6.12E-01	ARSB	-	6.01E-08	1.08E-01	1.53E-07	562	
2	rs6712088	4935287	5.19E-01	N/A	5	rs672413	78313985	6.12E-01	ARSB	-	6.01E-08	1.08E-01	1.53E-07	563	
10	rs1907336	77824762	6.70E-01	C10orf11	11	rs670342	78329325	9.25E-01	ODZ4	-	6.03E-08	N/A	N/A	564	
6	rs3130473	31307187	2.29E-01	N/A	6	rs3130534	31317024	3.50E-01	N/A	MHC	6.03E-08	8.63E-01	6.93E-05	565	
1	rs4659904	2.37E+08	7.64E-01	N/A	3	rs33036	1.61E+08	9.89E-01	SCHIP1	-	6.07E-08	9.84E-01	2.89E-05	566	
2	rs13384421	2.32E+08	2.35E-02	C2orf57	13	rs9534563	46656237	5.59E-01	N/A	-	6.09E-08	9.00E-01	1.16E-05	567	
7	rs6460519	68551289	6.83E-01	N/A	13	rs342594	59176565	9.16E-01	DIAPH3	-	6.10E-08	5.36E-01	7.23E-06	568	
2	rs1463990	2.21E+08	9.68E-01	N/A	16	rs7200316	5860065	5.46E-01	N/A	-	6.11E-08	6.53E-01	8.31E-06	569	
1	rs3766793	53363218	8.50E-01	SLC1A7	2	rs243083	60427374	8.80E-01	N/A	-	6.12E-08	6.84E-01	1.45E-04	570	
11	rs1371351	96030639	6.33E-01	N/A	11	rs4258339	1.08E+08	3.72E-01	N/A	-	6.13E-08	N/A	N/A	571	
2	rs11888197	1.95E+08	5.61E-01	N/A	4	rs9999662	7475846	4.47E-01	SORCS2	-	6.14E-08	3.07E-01	1.58E-04	572	
10	rs4993019	1.33E+08	6.54E-01	N/A	15	rs2288608	31821713	8.87E-01	RYR3	-	6.17E-08	5.88E-01	5.24E-06	573	
7	rs7788751	1.53E+08	4.99E-01	N/A	9	rs10125911	1.28E+08	9.82E-01	N/A	-	6.18E-08	8.72E-02	5.10E-08	574	
3	rs2276745	1.29E+08	6.27E-01	MGLL	7	rs7793131	33094064	9.86E-01	RP9	-	6.18E-08	6.31E-01	3.21E-06	575	
8	rs1685101	32317455	6.83E-01	NRG1	11	rs11232924	81304990	9.08E-01	N/A	-	6.19E-08	8.80E-01	2.93E-05	576	
6	rs12528284	1.67E+08	6.57E-01	RPS6KA2	12	rs12580153	91342613	1.82E-01	CLLU1	-	6.26E-08	1.90E-01	3.91E-04	577	
3	rs1356792	20353157	1.25E-01	N/A	14	rs1152490	55865987	1.95E-01	N/A	-	6.26E-08	5.30E-01	2.11E-05	578	
9	rs920901	74419068	7.34E-02	TMC1	17	rs2323661	15241034	1.56E-01	N/A	-	6.31E-08	4.05E-01	7.32E-04	579	
1	rs11804163	36757321	2.49E-01	N/A	3	rs1868504	54397089	9.19E-01	CACN/A2D3	-	6.32E-08	6.98E-01	1.22E-05	580	
1	rs4660176	41066236	3.08E-01	KCNQ4	7	rs740576	1.53E+08	8.98E-01	DPP6	-	6.33E-08	2.97E-03	2.11E-09	581	
11	rs549289	1.17E+08	8.02E-01	DSCAML1	15	rs3145	56999403	1.93E-01	SLTM	-	6.34E-08	1.54E-01	7.15E-04	582	
3	rs475050	22998578	6.54E-01	N/A	13	rs9567867	46931402	2.50E-01	N/A	-	6.35E-08	4.28E-01	4.89E-07	583	
6	rs9384817	1.12E+08	4.04E-01	WISP3	3	rs9820339	1.51E+08	7.77E-01	WWTR1	-	6.35E-08	3.80E-01	8.65E-04	584	
2	rs1419950	2.3E+08	3.09E-01	PID1	18	rs2541874	61792527	4.60E-01	N/A	-	6.36E-08	2.85E-01	1.96E-06	585	
5	rs4704506	77690583	5.04E-01	SCAMP1	11	rs7110734	73530035	3.56E-01	C2CD3	-	6.37E-08	7.49E-01	8.84E-06	586	
5	rs4704506	77690583	5.04E-01	SCAMP1	11	rs11235995	73484091	3.56E-01	C2CD3	-	6.37E-08	8.03E-01	1.09E-05	587	
5	rs4704506	77690583	5.04E-01	SCAMP1	11	rs7125032	73484690	3.56E-01	C2CD3	-	6.37E-08	8.03E-01	1.09E-05	588	
5	rs2089192	1.7E+08	5.56E-01	KCNIP1	7	rs1109552	1.28E+08	8.96E-01	FAM71F2	-	6.38E-08	3.21E-01	2.25E-06	589	
10	rs2163673	90477499	7.09E-01	LIPK	21	rs2837682	40826362	6.19E-01	DSCAM	-	6.38E-08	8.49E-01	2.40E-06	590	
6	rs17317669	25514010	7.37E-01	N/A	5	rs3853114	13534754	6.45E-01	N/A	MHC	6.38E-08	6.86E-01	3.55E-05	591	
7	rs4472413	15380099	2.56E-01	TMEM195	10	rs10762833	80441226	4.81E-01	LOC283050	-	6.38E-08	9.67E-01	2.83E-06	592	
3	rs7613069	10600996	5.88E-01	N/A	14	rs1894371	21704535	1.04E-01	N/A	-	6.39E-08	7.63E-01	2.49E-04	593	
16	rs12918570	56825937	7.58E-01	CCDC113	19	rs2108622	15851431	2.19E-01	CYP4F2	-	6.39E-08	7.86E-01	2.29E-05	594	
2	rs978673	1.61E+08	8.99E-01	N/A	11	rs6592878	79405363	6.40E-01	N/A	-	6.42E-08	6.17E-01	7.35E-05	595	
7	rs1548210	3493211	3.57E-01	SDK1	12	rs10773263	1.25E+08	8.37E-01	N/A	-	6.42E-08	1.75E-01	1.95E-03	596	
7	rs1548210	3493211	3.57E-01	SDK1	12	rs7958738	1.25E+08	8.37E-01	N/A	-	6.42E-08	1.75E-01	1.95E-03	597	
20	rs6140971	9515361	1.23E-01	PAK7	20	rs1015581	14467512	4.24E-01	MACROD2	-	6.42E-08	2.24E-01	5.09E-04	598	
2	rs2115366	8381482	9.89E-01	N/A	17	rs1328120	47802041	2.49E-01	N/A	-	6.44E-08	8.48E-01	7.20E-06	599	
4	rs7686686	1.11E+08	3.40E-01	N/A	5	rs13160797	1.62E+08	3.80E-01	N/A	-	6.45E-08	1.43E-01	3.15E-07	600	
18	rs593234	27933920	3.68E-01	N/A	18	rs7232315	53717230	6.60E-01	N/A	-	6.45E-08	6.15E-01	2.77E-06	601	
13	rs9511484	24339945	5.54E-01	N/A	14	rs11847318	30287393	7.26E-01	SCFD1	-	6.47E-08	4.04E-01	3.09E-05	602	
5	rs2289285	1.49E+08	3.32E-01	ABLIM3	19	rs654765	59282648	1.52E-01	NDUFA3	-	6.47E-08	9.14E-01	2.30E-04	603	
3	rs12152230	35728583	4.12E-01	ARPP-21	9	rs7853654	1.33E+08	5.42E-01	ABL1	-	6.48E-08	7.04E-01	2.48E-05	604	
1	rs12140479	70595826	2.66E-01	ANKRD13C	8	rs4737915	69810468	3.77E-01	C8orf34	-	6.48E-08	2.70E-01	3.25E-04	605	
6	rs658376	1.38E+08	6.00E-01	N/A	10	rs10881886	92870563	4.99E-01	N/A	-	6.50E-08	2.72E-01	4.44E-07	606	
7	rs11983822	13067583	7.10E-02	N/A	19	rs1859955	62514181	6.47E-01	ZNF460	-	6.50E-08	5.85E-01	1.85E-06	607	
4	rs10020207	58401219	1.97E-01	N/A	16	rs2938723	61142271	8.85E-01	N/A	-	6.52E-08	3.20E-01	3.84E-04	608	
3	rs6764210	36237237	5.32E-01	N/A	16	rs12149452	6977388	9.69E-01	A2BP1	-	6.52E-08	8.39E-01	1.90E-05	609	
1	rs12726519	1.85E+08	6.72E-01	PLA2G4A	18	rs2023096	69807945	3.84E-01	N/A	-	6.52E-08	6.45E-01	3.53E-06	610	
1	rs489933	5848388	1.86E-01	NPHP4	12	rs10848943	3814283	7.50E-01	PARP11	-	6.52E-08	6.42E-01	1.76E-05	611	
6	rs1009014	1.58E+08	2.41E-01	SYNJ2	2	rs11685822	64502642	9.82E-01	N/A	-	6.53E-08	7.38E-01	4.27E-05	612	
1	rs2576262	2.15E+08	5.36E-01	ESRRG	19	rs11669215	17867995	6.04E-01	SLC5A5	-	6.53E-08	1.41E-02	4.02E-09	613	
2	rs2627857	66155597	9.35E-01	N/A	8	rs12164192	1.05E+08	1.69E-01	N/A	-	6.53E-08	3.25E-01	2.51E-04	614	
2	rs1901753	11060172	5.04E-01	N/A	8	rs1534863	11705562	8.14E-01	FDFT1	-	6.53E-08	2.97E-01	4.50E-07	615	
4	rs11099345	1.37E+08	5.26E-01	N/A	12	rs3916142	1.28E+08	3.78E-02	TMEM132D	-	6.55E-08	8.58E-01	8.68E-06	616	
1	rs1393299	2.4E+08	3.10E-01	PLD5	7	rs5024722	1.42E+08	3.67E-01	LOC93432	-	6.59E-08	2.71E-01	3.22E-04	617	
7	rs17164162	11266179	6.32E-01	N/A	17	rs1815198	52715584	4.21E-02	MSI2	-	6.59E-08	9.12E-01	1.47E-05	618	
2	rs13419210	22805243	1.19E-01	N/A	9	rs10812695	27917133	8.78E-01	N/A	-	6.62E-08	7.47E-01	7.23E-05	619	
3	rs285299	36242533	5.64E-01	N/A	16	rs6500892	6953116	9.44E-01	A2BP1	-	6.63E-08	5.79E-01	6.91E-06	620	
3	rs285299	36242533	5.64E-01	N/A	16	rs12149452	6977388	9.69E-01	A2BP1	-	6.65E-08	9.69E-01	2.23E-05	621	
5	rs1347679	1.06E+08	6.91E-02	N/A	9	rs10758314	35584724	1.76E-01	TESK1	-	6.66E-08	5.23E-01	4.43E-06	622	
10	rs12772794	20714659	4.84E-01	N/A	21	rs2070413	31424285	5.67E-01	TIAM1	-	6.66E-08	N/A	N/A	623	
8	rs1823506	1.11E+08	9.86E-01	N/A	12	rs876594	3531820	6.92E-01	PRMT8	-	6.71E-08	6.20E-01	2.07E-06	624	
2	rs11898850	73053127	9.44E-01	SFXN5	11	rs10833760	22472317	8.82E-01	N/A	-	6.71E-08	8.10E-01	3.16E-05	625	
8	rs4735729	77471658	3.79E-01	N/A	17	rs11871749	14593162	8.66E-01	N/A	-	6.72E-08	1.58E-01	1.96E-06	626	
4	rs1105889	1.54E+08	5.31E-01	FHDC1	14	rs4983413	1.05E+08	3.35E-01	MTA1	-	6.74E-08	1.61E-01	1.83E-08	627	
6	rs2252551	34730654	3.57E-01	C6orf106	11	rs12420859	44681103	6.76E-01	N/A	MHC	6.74E-08	1.61E-01	1.83E-08	628	
6	rs2815003	34743787	3.57E-01	C6orf106	11	rs12420859	44681103	6.76E-01	N/A	MHC	6.74E-08	6.35E-01	2.22E-04	629	
6	rs6570957	1.5E+08	2.74E-01	N/A	9	rs1668978	25342327	1.10E-01	N/A	-	6.77E-08	7.60E-01	4.97E-05	630	
3	rs2724706	1.39E+08	3.24E-01	N/A	8	rs904924	96599824	2.18E-01	N/A	-	6.77E-08	3.42E-01	6.47E-04	631	
5	rs307177	6140348	3.52E-01	N/A	5	rs4117784	1.54E+08	8.70E-01	LARP1	-	6.77E-08	N/A	N/A	632	
5	rs3797937	9244362	9.13E-01	SEMA5A	9	rs7863071	18521748	6.63E-01	ADAMTSL1	-	6.78E-08	8.41E-02	6.99E-08	633	
2	rs1065643	42874697	8.71E-01	HAAO	4	rs11730156	22095646	5.10E-01	GPR125	-	6.78E-08	1.78E-01	9.70E-04	634	
2	rs332723	1.19E+08	4.72E-01	N/A	20	rs6020814	48922587	5.42E-01	BCAS4	-	6.79E-08	3.49E-01	6.49E-07	635	
1	rs11208115	63294343	3.44E-01	N/A	8	rs1209992	13168685	5.37E-01	DLC1	-	6.80E-08	3.03E-02	2.28E-07	636	
6	rs2490050	3807592	2.85E-01	FAM50B	18	rs2032761	1474362	4.53E-01	N/A	-	6.80E-08	6.45E-02	1.86E-07	637	
6	rs2490052	3810945	2.85E-01	FAM50B	18	rs2032761	1474362	4.53E-01	N/A	-	6.80E-08	6.45E-02	1.86E-07	638	
8	rs2648883	1.29E+08	8.39E-01	PVT1	11	rs6592615	75226420	2.01E-01	UVRAG	-	6.81E-08	7.58E-01	1.51E-04	639	
2	rs11892554	2.09E+08	2.91E-01	N/A	7	rs1989886	20549647	1.18E-01	N/A	-	6.81E-08	8.09E-01	9.89E-05	640	
2	rs4044513	2.06E+08	3.64E-01	PARD3B	7	rs4588783	1.03E+08	7.15E-01	SLC26A5	-	6.82E-08	2.37E-01	1.45E-03	641	
2	rs291335	2.4E+08	9.65E-01	HDAC4	7	rs7787734	7618897	2.88E-01	MIOS	-	6.83E-08	2.18E-01	5.36E-04	642	
1	rs2358756	34252509	1.57E-01	CSMD2	17	rs1990197	65582129	8.57E-01	KCNJ16	-	6.89E-08	2.73E-01	1.31E-03	643	
6	rs965719	5271733	9.61E-01	FARS2	4	rs12647248	1.23E+08	8.83E-01	N/A	-	6.89E-08	N/A	N/A	644	
6	rs9380215	31157634	4.29E-02	N/A	6	rs4122189	31275906	7.70E-02	HCG27	MHC	6.90E-08	1.22E-01	6.01E-07	645	
9	rs1231343	25732531	4.72E-01	N/A	17	rs12603672	48470242	8.59E-01	N/A	-	6.93E-08	1.44E-02	1.54E-08	646	
3	rs899983	1.39E+08	3.57E-01	N/A	8	rs904924	96599824	2.18E-01	N/A	-	6.93E-08	3.57E-01	6.10E-04	647	
2	rs6756027	64573758	5.05E-01	N/A	3	rs7429875	27565426	7.42E-01	N/A	-	6.93E-08	5.68E-01	1.29E-04	648	
3	rs2218746	28991443	3.16E-01	N/A	15	rs4842935	81431271	5.68E-01	HOMER2	-	6.94E-08	2.55E-01	5.36E-04	649	
3	rs12631386	1.35E+08	4.49E-02	N/A	5	rs988306	1.37E+08	5.16E-01	SPOCK1	-	6.94E-08	3.47E-01	1.53E-03	650	
1	rs1391819	63343139	8.94E-01	N/A	11	rs11041391	7432637	5.65E-01	SYT9	-	6.94E-08	6.63E-01	8.53E-07	651	
2	rs6746082	25512748	2.21E-01	DTNB	3	rs1092103	1.46E+08	1.72E-01	N/A	-	6.95E-08	4.13E-01	8.80E-04	652	
2	rs788172	1.73E+08	4.78E-01	MAP1D	7	rs10245949	47844464	3.59E-01	PKD1L1	-	6.95E-08	4.90E-01	1.29E-04	653	
14	rs2255627	61309786	9.71E-01	SN/APC1	17	rs10775405	42543089	8.16E-01	CDC27	-	6.96E-08	4.16E-01	1.40E-05	654	
14	rs6573400	61309325	9.29E-01	SN/APC1	17	rs10775405	42543089	8.16E-01	CDC27	-	6.96E-08	4.34E-01	1.25E-05	655	
2	rs10198393	2.19E+08	1.00E-01	IL8RBP	13	rs1164502	75766685	6.62E-01	N/A	-	6.96E-08	4.29E-01	5.76E-06	656	
6	rs2169100	93477143	5.01E-01	N/A	2	rs10173035	1.68E+08	3.08E-02	N/A	-	6.99E-08	2.24E-01	5.82E-04	657	
2	rs1974677	10415869	8.58E-01	HPCAL1	12	rs4761378	76330817	6.54E-02	N/A	-	6.99E-08	5.01E-01	1.33E-05	658	
10	rs3861027	72961172	3.65E-01	CDH23	17	rs755803	69439887	2.14E-01	N/A	-	6.99E-08	3.28E-01	4.87E-06	659	
13	rs4586303	37883824	6.11E-01	N/A	18	rs568080	32094999	5.37E-01	MOCOS	-	7.00E-08	9.43E-01	1.30E-04	660	
2	rs6707252	1000713	3.04E-01	SNTG2	8	rs2111570	1.41E+08	5.71E-01	KCNK9	-	7.00E-08	3.36E-01	2.35E-06	661	
5	rs1904163	52221753	7.92E-01	ITGA1	12	rs6581982	69684055	2.64E-01	N/A	-	7.02E-08	9.83E-01	2.77E-05	662	
11	rs4980704	69501109	3.49E-01	N/A	13	rs1185049	28124634	2.13E-01	POMP	-	7.03E-08	8.67E-01	1.77E-05	663	
5	rs2368097	39694775	1.13E-01	N/A	9	rs10971632	33655229	6.52E-01	PTENP1	-	7.04E-08	5.82E-02	1.35E-03	664	
1	rs12564233	91637710	9.59E-01	HFM1	2	rs13386612	77841057	5.99E-01	N/A	-	7.05E-08	9.21E-01	9.85E-05	665	
1	rs10754178	1.94E+08	9.18E-01	N/A	2	rs3911080	70573427	5.09E-01	TGFA	-	7.05E-08	3.85E-01	3.90E-05	666	
6	rs6926443	17204333	5.36E-01	N/A	6	rs1870488	63991038	7.41E-01	N/A	-	7.05E-08	N/A	N/A	667	
8	rs9643233	1.29E+08	2.00E-01	N/A	16	rs4985065	8910173	9.96E-01	USP7	-	7.07E-08	9.63E-01	8.86E-05	668	
6	rs9352376	77471874	1.23E-01	N/A	3	rs9848911	16248562	1.97E-01	GALNTL2	-	7.08E-08	1.74E-01	1.04E-03	669	
7	rs2188513	92382009	5.14E-01	N/A	8	rs2249536	69486976	2.13E-01	N/A	-	7.09E-08	6.70E-01	1.86E-06	670	

SNP A					SNP B						Interaction P			Ranking	Cluster in top 100 interactions
CHR	SNP	Location	gle locus P va	Gene	CHR	SNP	Location	Single locus P valu	Gene	MHC region	Stage 1	Stage 2	Combined		
1	rs3827730	50710436	1.77E-01	FAF1	13	rs2324606	39882601	9.48E-01	LOC646982	-	7.15E-08	1.45E-01	2.75E-07	671	
7	rs17144110	21246477	4.04E-01	N/A	8	rs13250348	18617744	9.93E-01	PSD3	-	7.16E-08	5.51E-01	3.90E-04	672	
5	rs10037741	1.06E+08	9.07E-02	N/A	12	rs4763075	61962124	4.02E-01	N/A	-	7.16E-08	N/A	N/A	673	
3	rs4684717	10598391	7.53E-01	N/A	14	rs12434307	21771960	9.12E-02	N/A	-	7.17E-08	7.60E-01	5.83E-05	674	
6	rs11753072	811963	7.32E-01	N/A	1	rs331635	59438130	5.19E-01	N/A	-	7.17E-08	9.64E-01	1.78E-05	675	
2	rs1000582	1.69E+08	6.52E-01	N/A	5	rs188693	42975987	7.34E-02	N/A	-	7.18E-08	1.55E-01	9.46E-04	676	
1	rs2148682	65642077	2.92E-01	LEPR	12	rs2286007	841552	6.78E-01	N/A	-	7.18E-08	4.63E-01	3.61E-06	677	
7	rs6460519	68551289	6.83E-01	N/A	13	rs2226042	59188076	8.65E-01	DIAPH3	-	7.20E-08	4.37E-01	5.28E-06	678	
9	rs10820080	1.04E+08	8.07E-01	N/A	13	rs9543928	74916808	3.12E-01	TBC1D4	-	7.20E-08	6.77E-01	1.91E-05	679	
1	rs1008455	47446389	9.44E-01	PDZK1IP1	15	rs1579821	20501269	8.54E-01	CYFIP1	-	7.20E-08	3.51E-01	7.78E-05	680	
1	rs11260963	18436036	8.74E-01	IGSF21	2	rs7562418	7260610	8.88E-01	N/A	-	7.21E-08	7.24E-01	5.01E-05	681	
6	rs6900659	1.06E+08	8.04E-01	N/A	2	rs1435009	1.63E+08	9.35E-03	KCNH7	-	7.22E-08	3.59E-01	7.78E-06	682	
5	rs1001869	1.69E+08	8.32E-01	N/A	9	rs817829	1.09E+08	4.38E-02	N/A	-	7.23E-08	8.42E-01	6.24E-06	683	
1	rs3908575	98636348	6.82E-01	N/A	9	rs11138630	82170736	1.86E-01	N/A	-	7.25E-08	4.08E-01	4.97E-06	684	
9	rs13297228	81788576	5.45E-01	N/A	22	rs2213767	25700782	6.92E-04	N/A	-	7.26E-08	2.78E-01	2.02E-06	685	
11	rs4980704	69501109	3.49E-01	N/A	13	rs9551517	28128045	1.92E-01	POMP	-	7.26E-08	8.67E-01	1.82E-05	686	
1	rs2276401	1.57E+08	7.22E-01	SPTA1	21	rs2833492	32052953	7.21E-01	N/A	-	7.27E-08	3.96E-01	1.74E-06	687	
4	rs1105912	25677116	7.90E-01	N/A	9	rs2398829	94961237	5.90E-01	N/A	-	7.29E-08	8.61E-01	1.63E-05	688	
2	rs12996793	70503279	3.44E-01	N/A	12	rs4077193	80002092	4.19E-01	ACSS3	-	7.32E-08	1.21E-01	7.90E-04	689	
2	rs4852178	70501406	3.44E-01	N/A	12	rs4077193	80002092	4.19E-01	ACSS3	-	7.32E-08	1.21E-01	7.90E-04	690	
7	rs2280659	1.54E+08	4.99E-01	DPP6	8	rs1547559	8536538	4.10E-01	N/A	-	7.32E-08	N/A	N/A	691	
2	rs2627857	66155597	9.35E-01	N/A	8	rs2028945	1.05E+08	8.30E-02	RIMS2	-	7.34E-08	3.86E-01	2.42E-04	692	
3	rs9870986	1.48E+08	5.00E-01	N/A	8	rs12114032	20891612	8.29E-01	N/A	-	7.36E-08	6.00E-01	1.65E-04	693	
3	rs2350279	1.41E+08	3.47E-02	CLSTN2	4	rs2598306	42494347	7.18E-01	N/A	-	7.37E-08	4.66E-01	2.36E-06	694	
6	rs10782186	1.18E+08	2.24E-01	DCBLD1	8	rs6586746	18325984	2.70E-01	N/A	-	7.37E-08	4.06E-01	2.00E-06	695	
13	rs1171080	35385286	5.95E-01	DCLK1	13	rs2786953	1E+08	9.26E-01	N/A	-	7.38E-08	2.18E-01	1.89E-06	696	
1	rs994712	1.06E+08	5.15E-01	N/A	16	rs2407912	60158141	2.11E-01	N/A	-	7.38E-08	3.22E-01	3.75E-07	697	
12	rs1872521	1.27E+08	8.77E-01	N/A	22	rs713839	35063884	4.44E-01	MYH9	-	7.40E-08	2.43E-01	1.70E-06	698	
12	rs1872521	1.27E+08	8.77E-01	N/A	22	rs2413398	35060893	4.44E-01	MYH9	-	7.40E-08	2.80E-01	2.20E-06	699	
8	rs2190162	5342127	1.62E-01	N/A	14	rs8018930	94309119	5.71E-01	GSC	-	7.45E-08	4.55E-01	2.94E-04	700	
6	rs12216269	1.38E+08	9.09E-01	N/A	20	rs2208973	12342990	4.41E-01	N/A	-	7.46E-08	5.59E-01	1.35E-04	701	
7	rs7795683	1.14E+08	9.10E-01	N/A	14	rs1315728	67013662	9.59E-01	TMEM229B	-	7.47E-08	6.64E-01	1.70E-04	702	
3	rs2581786	53101183	1.65E-01	RFT1	17	rs2301775	34594761	8.82E-01	CACNB1	-	7.47E-08	N/A	N/A	703	
14	rs1959375	44021538	2.55E-01	N/A	14	rs7156399	95104682	7.38E-01	N/A	-	7.47E-08	2.84E-01	1.01E-04	704	
1	rs2131384	1.93E+08	3.15E-01	N/A	5	rs27114	14498585	4.08E-01	TRIO	-	7.51E-08	9.14E-01	2.70E-05	705	
1	rs1268910	2.13E+08	3.18E-01	N/A	5	rs13172112	31068066	4.99E-05	N/A	-	7.53E-08	4.01E-01	9.51E-06	706	
1	rs1268910	2.13E+08	3.18E-01	N/A	5	rs16900572	31069693	4.99E-05	N/A	-	7.53E-08	4.01E-01	9.51E-06	707	
2	rs9309723	1009753	3.36E-01	SNTG2	8	rs2111570	1.41E+08	5.71E-01	KCNK9	-	7.53E-08	2.88E-01	1.83E-06	708	
2	rs17013592	77088275	7.17E-01	LRRTM4	5	rs7726783	3187719	3.47E-01	N/A	-	7.54E-08	7.69E-01	3.07E-05	709	
2	rs1470504	77076318	7.97E-01	LRRTM4	5	rs597099	3192158	7.14E-01	N/A	-	7.55E-08	7.37E-01	3.31E-05	710	
6	rs7764600	1860827	7.88E-01	GMDS	7	rs337901	1.26E+08	2.65E-02	N/A	-	7.56E-08	2.95E-01	1.70E-04	711	
3	rs6791296	62406455	7.93E-01	CADPS	20	rs1205693	49750938	1.27E-01	ATP9A	-	7.58E-08	2.58E-01	9.43E-05	712	
1	rs1125953	1.96E+08	9.32E-01	N/A	7	rs1035169	1.04E+08	7.40E-01	ORC5L	-	7.62E-08	7.39E-01	1.14E-05	713	
7	rs893785	12471898	1.57E-02	N/A	7	rs5004318	47765998	9.25E-01	PKD1L1	-	7.62E-08	9.56E-01	7.72E-05	714	
7	rs893785	12471898	1.57E-02	N/A	7	rs6953403	47768997	9.25E-01	PKD1L1	-	7.62E-08	9.56E-01	7.72E-05	715	
6	rs4714674	43352692	6.53E-03	TTBK1	17	rs9895907	5521311	2.74E-01	N/A	-	7.63E-08	9.54E-01	2.23E-05	716	
6	rs4714674	43352692	6.53E-03	TTBK1	17	rs9915238	5520461	3.13E-01	N/A	-	7.63E-08	9.54E-01	2.23E-05	717	
6	rs2744974	34687409	3.36E-01	C6orf106	11	rs12420859	44681103	6.76E-01	N/A	MHC	7.65E-08	4.65E-01	1.30E-06	718	
7	rs2188513	92382009	5.14E-01	N/A	8	rs10104993	69509383	1.91E-01	N/A	-	7.68E-08	N/A	N/A	719	
3	rs6779886	1.17E+08	3.97E-01	LSAMP	7	rs219822	98427987	1.21E-01	TRRAP	-	7.72E-08	1.64E-01	1.17E-06	720	
10	rs4746372	77608248	4.90E-01	C10orf11	20	rs1470351	5097713	6.38E-02	CDS2	-	7.73E-08	6.65E-01	1.19E-05	721	
5	rs7718088	1.8E+08	3.52E-01	RASGEF1C	8	rs13267425	1.02E+08	2.72E-02	N/A	-	7.73E-08	7.76E-01	3.20E-05	722	
3	rs10514694	36283073	2.94E-01	N/A	16	rs12923619	6951319	7.89E-01	A2BP1	-	7.74E-08	3.00E-01	3.73E-06	723	
2	rs797703	47801295	8.31E-01	N/A	3	rs6438537	1.21E+08	4.07E-01	CD80	-	7.74E-08	7.88E-01	1.03E-05	724	
3	rs13094598	35709235	3.90E-01	ARPP-21	9	rs7853654	1.33E+08	5.42E-01	ABL1	-	7.75E-08	6.98E-01	5.43E-05	725	
2	rs1918876	1.45E+08	8.78E-01	N/A	13	rs8181851	58498359	3.14E-01	N/A	-	7.76E-08	6.69E-01	6.58E-05	726	
2	rs904162	47363828	4.44E-01	N/A	7	rs1859032	1.17E+08	2.28E-01	N/A	-	7.76E-08	3.19E-01	6.47E-07	727	
8	rs4384011	1.2E+08	2.67E-01	N/A	13	rs2985947	45056322	2.08E-02	FAM194B	-	7.77E-08	5.00E-02	2.69E-03	728	
3	rs12233598	1.51E+08	2.23E-01	N/A	8	rs4504596	11149302	6.37E-01	N/A	-	7.77E-08	7.68E-01	1.22E-04	729	
10	rs2766070	1.29E+08	5.64E-01	DOCK1	13	rs400532	1.1E+08	1.75E-01	COL4A2	-	7.79E-08	2.32E-01	8.54E-07	730	
6	rs1006012	53966408	8.93E-01	N/A	1	rs1188473	2.27E+08	2.53E-01	TRIM11	-	7.80E-08	8.70E-01	1.12E-04	731	
5	rs1865508	1.05E+08	1.73E-01	N/A	12	rs12824017	61935103	2.85E-01	N/A	-	7.80E-08	6.51E-01	9.66E-06	732	
3	rs6764210	36237237	5.32E-01	N/A	16	rs12923619	6951319	7.89E-01	A2BP1	-	7.84E-08	4.72E-01	1.14E-05	733	
1	rs16858460	1.65E+08	8.22E-01	DUSP27	12	rs2632226	75535673	3.99E-01	N/A	-	7.86E-08	5.66E-01	2.21E-05	734	
9	rs10759387	1.12E+08	5.73E-01	PALM2-AKAP2	22	rs4823336	43304667	1.50E-01	N/A	-	7.86E-08	8.37E-02	2.38E-03	735	
3	rs4377455	29403191	6.47E-01	N/A	15	rs11634230	91503829	2.60E-02	N/A	-	7.87E-08	5.85E-01	1.48E-06	736	
2	rs6543606	31208012	2.20E-01	GALNT14	7	rs13228939	53674043	6.93E-01	N/A	-	7.87E-08	9.45E-01	2.16E-05	737	
1	rs11806304	1.85E+08	3.57E-01	C1orf27	5	rs7715102	2427305	5.42E-01	N/A	-	7.88E-08	5.24E-01	5.00E-06	738	
1	rs559798	2.43E+08	9.42E-01	C1orf100	11	rs2004933	43432394	6.42E-01	TTC17	-	7.89E-08	6.94E-01	1.09E-04	739	
1	rs559798	2.43E+08	9.42E-01	C1orf100	11	rs4354680	43345907	6.42E-01	TTC17	-	7.89E-08	7.92E-01	7.18E-05	740	
2	rs4675124	2.28E+08	5.93E-01	RHBDD1	17	rs4239112	10096227	3.32E-01	N/A	-	7.90E-08	2.77E-01	2.50E-07	741	
2	rs1527418	1.19E+08	1.90E-01	N/A	9	rs4842173	1.37E+08	9.22E-01	COL5A1	-	7.91E-08	2.68E-01	5.85E-06	742	
1	rs680925	66355709	3.11E-01	PDE4B	1	rs9943293	1.7E+08	3.36E-01	N/A	-	7.91E-08	9.49E-01	2.42E-05	743	
1	rs1287635	5847333	4.74E-01	NPHP4	12	rs10848943	3814283	7.50E-01	PARP11	-	7.92E-08	8.56E-01	3.18E-05	744	
6	rs10498871	70969556	5.00E-01	COL19A1	13	rs7990263	35359216	6.07E-01	DCLK1	-	7.92E-08	8.00E-02	2.78E-07	745	
15	rs12324234	92930509	1.35E-02	N/A	16	rs7342694	80461301	1.98E-01	PLCG2	-	7.92E-08	4.45E-01	5.39E-04	746	
1	rs896549	2E+08	8.77E-01	LGR6	10	rs7092996	16371668	5.58E-01	N/A	-	7.92E-08	3.13E-01	5.53E-04	747	
5	rs10477862	1.06E+08	8.56E-02	N/A	12	rs12824017	61935103	2.85E-01	N/A	-	7.93E-08	6.18E-01	1.73E-05	748	
6	rs6938042	93890157	9.07E-02	N/A	4	rs4543114	6797326	3.62E-01	N/A	-	7.94E-08	N/A	N/A	749	
6	rs6926443	17204333	5.36E-01	N/A	6	rs10755383	64014326	7.92E-01	N/A	-	7.95E-08	N/A	N/A	750	
1	rs6664353	1.81E+08	6.55E-01	C1orf14	3	rs184452	64125246	5.83E-01	PRICKLE2	-	7.97E-08	7.49E-01	6.98E-05	751	
6	rs2496698	81422854	5.51E-01	N/A	1	rs4661539	15065503	3.32E-01	KIAA1026	-	7.98E-08	5.19E-01	3.14E-06	752	
6	rs13197089	1.11E+08	1.62E-01	CDK19	1	rs10798126	1.86E+08	8.20E-01	N/A	-	7.98E-08	8.63E-01	3.75E-05	753	
4	rs17040984	1.63E+08	9.50E-02	FSTL5	8	rs2172427	18153536	9.32E-01	N/A	-	8.00E-08	2.09E-01	3.56E-07	754	
2	rs2892769	1.62E+08	2.36E-01	N/A	4	rs12648491	37910380	2.87E-01	N/A	-	8.01E-08	2.90E-01	3.62E-04	755	
3	rs7613069	10600996	5.88E-01	N/A	14	rs6572313	21751816	7.24E-02	N/A	-	8.02E-08	7.60E-01	2.68E-04	756	
15	rs12324234	92930509	1.35E-02	N/A	16	rs4997772	80462796	1.94E-01	PLCG2	-	8.02E-08	6.66E-01	3.11E-04	757	
2	rs4663126	2.35E+08	4.68E-02	N/A	13	rs7985561	23963550	9.92E-01	PARP4	-	8.03E-08	5.48E-01	1.72E-05	758	
16	rs11645645	1444150	8.37E-01	CCDC154	16	rs7188174	53009218	6.93E-01	N/A	-	8.03E-08	5.07E-01	1.63E-05	759	
2	rs11684876	1.07E+08	2.31E-02	N/A	3	rs566216	1.76E+08	5.95E-01	N/A	-	8.04E-08	6.81E-01	2.35E-05	760	
6	rs3130473	31307187	2.29E-01	N/A	6	rs2253907	31444849	7.98E-01	HLA-B	MHC	8.04E-08	6.44E-02	1.69E-07	761	
2	rs11674577	2.4E+08	4.12E-02	N/A	9	rs12551186	1.19E+08	3.32E-01	ASTN2	-	8.07E-08	1.64E-01	2.54E-06	762	
5	rs4479880	1.6E+08	5.55E-01	ATP10B	16	rs12925541	83990006	5.38E-01	N/A	-	8.08E-08	7.74E-01	3.49E-04	763	
6	rs2032544	1.67E+08	5.68E-01	RPS6KA2	12	rs406117	91323127	2.14E-01	CLLU1	-	8.09E-08	2.15E-01	2.87E-04	764	
6	rs10485435	69672336	1.32E-01	BAI3	10	rs605089	78845394	8.31E-01	KCNMA1	-	8.09E-08	9.40E-01	5.47E-05	765	
1	rs1125953	1.96E+08	9.32E-01	N/A	7	rs6948583	1.04E+08	6.84E-01	ORC5L	-	8.09E-08	7.39E-01	1.19E-05	766	
2	rs10204096	46737243	7.14E-01	N/A	20	rs10485815	51096895	8.85E-02	TSHZ2	-	8.13E-08	8.12E-01	3.89E-05	767	
1	rs876761	91552996	8.98E-01	HFM1	2	rs13386612	77841057	5.99E-01	N/A	-	8.13E-08	9.22E-01	1.05E-04	768	
3	rs2590453	1.89E+08	8.62E-01	N/A	20	rs2206641	19445646	2.63E-01	SLC24A3	-	8.13E-08	5.42E-02	1.85E-03	769	
2	rs291329	2.4E+08	9.20E-01	HDAC4	7	rs7804360	7621768	3.21E-01	MIOS	-	8.13E-08	2.13E-01	5.34E-04	770	
6	rs2490050	3807592	2.85E-01	FAM50B	18	rs17382680	1477818	4.95E-01	N/A	-	8.13E-08	5.54E-02	1.83E-07	771	
6	rs2490050	3807592	2.85E-01	FAM50B	18	rs4327127	1478322	4.95E-01	N/A	-	8.13E-08	5.54E-02	1.83E-07	772	
6	rs2490052	3810945	2.85E-01	FAM50B	18	rs17382680	1477818	4.95E-01	N/A	-	8.13E-08	5.54E-02	1.83E-07	773	
6	rs2490052	3810945	2.85E-01	FAM50B	18	rs4327127	1478322	4.95E-01	N/A	-	8.13E-08	5.54E-02	1.83E-07	774	
9	rs10819907	97938109	8.46E-01	N/A	12	rs7134360	1.04E+08	7.16E-01	N/A	-	8.14E-08	8.66E-01	7.74E-05	775	
2	rs1834619	17764966	5.69E-01	SMC6	8	rs2685603	34780638	7.11E-01	N/A	-	8.14E-08	3.41E-02	7.10E-03	776	
3	rs2699971	1.1E+08	3.37E-01	N/A	5	rs1423127	61463894	2.09E-01	N/A	-	8.15E-08	4.01E-01	1.17E-03	777	
3	rs2699971	1.1E+08	3.37E-01	N/A	5	rs3906400	61474161	2.09E-01	N/A	-	8.15E-08	4.01E-01	1.17E-03	778	
4	rs4697450	23904932	1.74E-01	N/A	8	rs7821815	25907378	6.29E-01	EBF2	-	8.16E-08	5.11E-01	4.74E-06	779	
15	rs11854769	36289535	6.23E-01	N/A	16	rs3764276	29580704	3.61E-01	QPRT	-	8.17E-08	7.32E-01	1.53E-04	780	
2	rs13422673	1.55E+08	8.42E-01	N/A	8	rs1996193	83563903	7.25E-01	N/A	-	8.18E-08	5.45E-01	2.14E-06	781	
12	rs11059460	1.27E+08	2.00E-01	N/A	18	rs3744996	41744600	2.82E-01	KIAA1632	-	8.19E-08	5.35E-01	2.38E-04	782	

SNP A					SNP B						Interaction P			Ranking	Cluster in top 100 interactions
CHR	SNP	Location	gle locus P va	Gene	CHR	SNP	Location	Single locus P valu	Gene	MHC region	Stage 1	Stage 2	Combined		
1	rs478665	65288121	8.38E-01	MIR101-1	10	rs7075837	21066895	6.94E-01	N/A	-	8.19E-08	1.45E-01	3.50E-07	783	
6	rs7755677	17120649	1.29E-01	N/A	9	rs1090130	1.33E+08	8.00E-01	FIBCD1	-	8.21E-08	8.66E-01	3.10E-05	784	
6	rs6903707	1.43E+08	7.17E-01	GPR126	3	rs7641032	23118465	3.81E-01	N/A	-	8.21E-08	6.20E-01	4.73E-05	785	
1	rs281966	91511350	9.12E-01	HFM1	2	rs1487036	77843115	6.24E-01	N/A	-	8.22E-08	7.56E-01	5.27E-05	786	
1	rs281968	91507901	9.12E-01	HFM1	2	rs1487036	77843115	6.24E-01	N/A	-	8.22E-08	8.25E-01	6.69E-05	787	
3	rs9874792	55835566	2.37E-01	ERC2	19	rs1860565	62026834	7.53E-01	PEG3	-	8.23E-08	3.35E-01	1.53E-05	788	
5	rs459760	1.02E+08	3.28E-01	N/A	10	rs7907048	80357575	4.83E-01	LOC283050	-	8.25E-08	6.56E-01	5.55E-06	789	
6	rs9271568	32698441	6.10E-01	HLA-DQA1	19	rs1004246	16317260	8.23E-01	EPS15L1	MHC	8.26E-08	N/A	N/A	790	
14	rs2071047	53488161	8.49E-01	BMP4	19	rs4805363	34477688	2.20E-01	N/A	-	8.27E-08	7.38E-01	4.25E-05	791	
9	rs10820080	1.04E+08	8.07E-01	N/A	13	rs4597197	74915102	4.86E-01	TBC1D4	-	8.27E-08	6.65E-01	1.99E-05	792	
15	rs1424711	92933574	2.68E-02	N/A	16	rs4997772	80462796	1.94E-01	PLCG2	-	8.27E-08	8.72E-01	1.37E-04	793	
6	rs7773278	1.44E+08	7.07E-01	PLAGL1	3	rs6772225	55251558	1.06E-01	N/A	-	8.27E-08	4.54E-02	1.86E-03	794	
6	rs483076	69605819	1.12E-01	BAI3	10	rs605089	78845394	8.31E-01	KCNMA1	-	8.27E-08	9.49E-01	6.58E-05	795	
6	rs513357	69615419	1.12E-01	BAI3	10	rs605089	78845394	8.31E-01	KCNMA1	-	8.27E-08	9.49E-01	6.58E-05	796	
6	rs544495	69604733	1.12E-01	BAI3	10	rs605089	78845394	8.31E-01	KCNMA1	-	8.27E-08	9.49E-01	6.58E-05	797	
1	rs11208057	40658419	8.83E-01	SMAP2	22	rs1005518	43463948	2.86E-01	PRR5	-	8.27E-08	1.39E-01	3.26E-06	798	
15	rs1424711	92933574	2.68E-02	N/A	16	rs7342694	80461301	1.98E-01	PLCG2	-	8.27E-08	6.25E-01	2.44E-04	799	
6	rs6929407	82540364	9.93E-01	N/A	14	rs1115944	79675956	4.17E-01	N/A	-	8.28E-08	7.00E-01	1.02E-04	800	
18	rs1639365	3639896	9.82E-01	DLGAP1	20	rs6080604	17136874	7.42E-01	PCSK2	-	8.28E-08	3.34E-01	3.32E-06	801	
13	rs7986907	78628865	9.19E-01	N/A	15	rs1695826	71877087	5.55E-01	N/A	-	8.28E-08	6.62E-01	6.12E-06	802	
2	rs11892554	2.09E+08	2.91E-01	N/A	7	rs916790	20539225	9.56E-02	N/A	-	8.29E-08	5.11E-01	2.31E-04	803	
9	rs4269622	28231422	6.45E-01	LINGO2	11	rs901553	11310922	4.51E-01	GALNTL4	-	8.29E-08	7.44E-01	1.95E-06	804	
3	rs922939	25440012	5.85E-01	RARB	11	rs3758729	45165411	7.74E-01	PRDM11	-	8.31E-08	1.82E-01	2.00E-04	805	
10	rs1701470	12194839	1.58E-02	SEC61A2	19	rs7257033	62299942	5.37E-01	N/A	-	8.31E-08	1.63E-01	4.79E-07	806	
10	rs1246758	30254709	9.96E-01	N/A	11	rs893949	1.34E+08	8.40E-01	N/A	-	8.33E-08	2.26E-01	5.05E-04	807	
2	rs4514909	69031848	9.50E-01	GKN2	8	rs754234	1.36E+08	9.77E-01	ZFAT	-	8.34E-08	8.01E-01	6.22E-06	808	
6	rs9295957	31265572	2.18E-01	POU5F1	6	rs7750269	31379136	8.58E-01	N/A	MHC	8.35E-08	4.77E-01	5.47E-06	809	
2	rs17039713	60190093	1.70E-02	N/A	12	rs7964855	1.15E+08	7.67E-01	N/A	-	8.35E-08	4.41E-01	5.80E-04	810	
2	rs4851390	1.01E+08	2.74E-01	NPAS2	10	rs1032317	1.07E+08	8.29E-01	SORCS3	-	8.35E-08	4.73E-01	3.84E-06	811	
2	rs6541877	1.24E+08	5.97E-01	N/A	8	rs7818430	21854375	3.40E-01	XPO7	-	8.36E-08	7.11E-02	2.02E-03	812	
8	rs6471603	58517186	9.19E-01	N/A	18	rs1421265	52617413	6.52E-01	WDR7	-	8.36E-08	4.50E-01	3.34E-04	813	
1	rs2803238	63132148	6.82E-01	N/A	4	rs1313470	31417795	6.99E-01	N/A	-	8.36E-08	6.43E-01	2.88E-05	814	
2	rs10932775	2.19E+08	8.40E-01	PNKD	4	rs2292837	1.4E+08	2.54E-01	ELF2	-	8.37E-08	6.08E-02	2.67E-03	815	
9	rs9644893	8915157	4.20E-01	PTPRD	16	rs3803662	51143842	4.09E-01	TOX3	-	8.42E-08	4.31E-01	1.33E-06	816	
9	rs1368684	8914538	4.20E-01	PTPRD	16	rs3803662	51143842	4.09E-01	TOX3	-	8.42E-08	4.99E-01	1.71E-06	817	
3	rs1920363	1.17E+08	6.04E-01	LSAMP	7	rs219822	98427987	1.21E-01	TRRAP	-	8.43E-08	3.35E-01	7.76E-06	818	
17	rs8077733	28043066	3.83E-01	MYO1D	18	rs1893489	46449659	8.82E-01	MAPK4	-	8.43E-08	5.25E-01	3.73E-04	819	
1	rs12568614	86152984	1.32E-01	COL24A1	5	rs1820167	52155113	6.97E-02	ITGA1	-	8.45E-08	8.19E-01	1.21E-05	820	
6	rs2844665	31114834	8.49E-01	HCG22	6	rs7761965	31381474	9.15E-01	N/A	MHC	8.46E-08	1.17E-01	5.72E-07	821	
4	rs4862612	1.87E+08	6.27E-04	N/A	22	rs882432	24677844	3.56E-02	MYO18B	-	8.48E-08	4.01E-01	2.24E-04	822	
8	rs13255959	1.19E+08	3.59E-01	EXT1	21	rs989554	38708797	6.64E-01	ERG	-	8.53E-08	9.78E-01	4.37E-05	823	
4	rs6551816	59533224	4.90E-01	N/A	11	rs1228026	20119894	7.06E-01	DBX1	-	8.54E-08	7.40E-01	3.98E-06	824	
4	rs7678440	1.06E+08	6.25E-01	TET2	16	rs1185930	56194179	3.39E-02	GPR56	-	8.55E-08	7.01E-01	3.95E-04	825	
15	rs4419043	98563203	4.76E-01	ADAMTS17	18	rs1296180	55117108	8.71E-01	CPLX4	-	8.57E-08	4.81E-01	6.52E-05	826	
4	rs14773	77252749	8.34E-01	ART3	5	rs2289876	36074074	4.59E-01	UGT3A2	-	8.59E-08	2.37E-01	4.42E-06	827	
6	rs3095250	31316319	6.90E-02	N/A	6	rs9394047	31344229	1.23E-01	HLA-C	MHC	8.60E-08	7.37E-01	1.55E-05	828	
6	rs2233984	31187243	4.20E-02	C6orf15	6	rs9295957	31265572	2.18E-01	POU5F1	MHC	8.62E-08	1.32E-01	6.60E-07	829	
5	rs1001869	1.69E+08	8.32E-01	N/A	9	rs817828	1.09E+08	7.35E-02	N/A	-	8.62E-08	9.12E-01	2.00E-05	830	
5	rs9293478	86130471	4.96E-01	N/A	13	rs4769136	20381892	3.46E-01	XPO4	-	8.65E-08	4.80E-01	3.04E-05	831	
6	rs1355460	23999853	1.51E-01	N/A	13	rs4287433	1.06E+08	2.09E-01	N/A	-	8.65E-08	2.05E-01	1.77E-06	832	
10	rs7477069	54212144	4.45E-01	MBL2	18	rs3826582	50187014	1.17E-01	N/A	-	8.67E-08	3.75E-01	2.61E-06	833	
3	rs7651261	1.83E+08	3.46E-01	N/A	18	rs1165994	30843312	1.06E-02	MAPRE2	-	8.67E-08	8.19E-01	1.96E-05	834	
2	rs6741437	28720705	1.36E-02	N/A	5	rs1713720	1.01E+08	7.00E-01	N/A	-	8.67E-08	9.76E-01	5.83E-05	835	
5	rs10514108	78141103	4.51E-01	ARSB	10	rs1090337	1161734	9.26E-01	WDR37	-	8.67E-08	6.39E-01	2.87E-04	836	
7	rs4472413	15380099	2.56E-01	TMEM195	10	rs1060628	80436013	7.88E-01	LOC283050	-	8.68E-08	6.88E-01	4.97E-05	837	
1	rs10157812	98656129	2.35E-01	N/A	9	rs1113863	82170736	1.86E-01	N/A	-	8.69E-08	4.36E-01	7.42E-06	838	
1	rs7542173	98656811	2.35E-01	N/A	9	rs1113863	82170736	1.86E-01	N/A	-	8.69E-08	4.36E-01	7.42E-06	839	
3	rs1356802	1.33E+08	1.83E-01	N/A	15	rs7183261	92129998	3.06E-01	N/A	-	8.70E-08	1.03E-01	3.01E-07	840	
11	rs1371351	96030639	6.33E-01	N/A	11	rs1121288	1.08E+08	4.66E-01	N/A	-	8.70E-08	N/A	N/A	841	
14	rs4901534	54267775	5.88E-01	SAMD4A	14	rs1162809	94818764	4.63E-01	CLMN	-	8.71E-08	2.38E-01	7.96E-07	842	
1	rs3002303	2.13E+08	8.18E-01	PTPN14	13	rs1049266	1.08E+08	6.04E-02	N/A	-	8.72E-08	6.64E-01	8.73E-05	843	
6	rs2265919	1.46E+08	3.00E-01	SHPRH	10	rs1318662	26860966	6.27E-01	APBB1IP	-	8.73E-08	8.41E-01	1.12E-04	844	
6	rs2265477	1.46E+08	3.00E-01	SHPRH	10	rs1318662	26860966	6.27E-01	APBB1IP	-	8.73E-08	8.81E-01	9.52E-05	845	
8	rs6984212	11328376	4.24E-01	C8orf12	15	rs1124734	96851676	8.76E-01	FAM169B	-	8.74E-08	8.94E-01	8.83E-06	846	
5	rs11749226	53989021	4.52E-01	N/A	9	rs6559808	86278852	2.38E-01	N/A	-	8.74E-08	1.85E-01	2.19E-04	847	
11	rs674485	64953969	4.53E-01	NEAT1	12	rs7132140	77260928	3.36E-01	N/A	-	8.74E-08	7.57E-01	5.48E-05	848	
2	rs1344375	80508405	1.95E-01	CTNN/A2	3	rs9847612	95573832	4.78E-02	N/A	-	8.75E-08	9.13E-01	2.08E-05	849	
11	rs716950	1.1E+08	8.91E-01	ARHGAP20	21	rs2026220	25436520	7.73E-01	N/A	-	8.77E-08	6.83E-01	7.50E-05	850	
3	rs9877416	1.53E+08	1.78E-03	N/A	17	rs1396216	50963616	7.69E-01	N/A	-	8.78E-08	5.05E-02	3.32E-08	851	
7	rs1525500	1.1E+08	3.04E-01	N/A	17	rs1165133	11799674	1.96E-02	DN/AH9	-	8.78E-08	5.21E-01	4.53E-05	852	
2	rs10200743	33589785	2.75E-01	RASGRP3	3	rs903056	14785294	1.95E-01	N/A	-	8.78E-08	1.33E-01	7.07E-07	853	
11	rs553484	82599186	2.28E-01	ANKRD42	22	rs743185	27433217	6.71E-01	CHEK2	-	8.78E-08	1.55E-01	2.28E-07	854	
3	rs6775796	74786379	7.90E-02	N/A	13	rs694322	48401261	6.19E-01	N/A	-	8.78E-08	1.78E-01	5.71E-08	855	
6	rs2517552	31115569	8.54E-01	HCG22	6	rs7761965	31381474	9.15E-01	N/A	MHC	8.78E-08	5.76E-02	3.04E-07	856	
8	rs2623638	3616003	2.52E-01	CSMD1	9	rs690364	92396585	6.74E-01	N/A	-	8.79E-08	4.21E-01	7.19E-05	857	
10	rs11245515	1.27E+08	5.03E-01	CTBP2	18	rs1380711	71644131	6.79E-02	N/A	-	8.79E-08	5.57E-01	3.09E-06	858	
10	rs11245515	1.27E+08	5.03E-01	CTBP2	18	rs1115091	71645804	6.79E-02	N/A	-	8.79E-08	5.66E-01	3.17E-06	859	
17	rs8069445	376995	2.52E-01	VPS53	17	rs1860178	11070954	8.72E-01	N/A	-	8.79E-08	6.59E-01	3.06E-04	860	
11	rs7941607	1.22E+08	6.25E-01	C11orf63	18	rs2364890	65595729	6.97E-01	DOK6	-	8.79E-08	8.60E-01	3.20E-05	861	
1	rs4970606	38854416	5.43E-01	N/A	9	rs7858089	28399300	3.65E-01	LINGO2	-	8.81E-08	6.57E-01	4.04E-06	862	
6	rs10457834	1.49E+08	2.16E-01	N/A	9	rs2798058	92063000	7.55E-01	N/A	-	8.81E-08	5.51E-01	4.87E-05	863	
6	rs4897043	1.49E+08	2.16E-01	N/A	9	rs2798058	92063000	7.55E-01	N/A	-	8.81E-08	5.51E-01	4.87E-05	864	
16	rs12918570	56825937	7.58E-01	CCDC113	19	rs1261018	15839641	1.73E-01	CYP4F2	-	8.82E-08	7.29E-01	2.19E-05	865	
6	rs7748966	1.67E+08	9.59E-01	PRR18	2	rs6707530	2.16E+08	3.67E-01	FN1	-	8.83E-08	2.76E-01	7.20E-07	866	
18	rs1639365	3639896	9.82E-01	DLGAP1	20	rs1578411	17126434	6.99E-01	N/A	-	8.84E-08	4.61E-01	6.10E-06	867	
2	rs1918876	1.45E+08	8.78E-01	N/A	13	rs6562004	58517819	2.73E-01	N/A	-	8.84E-08	7.26E-01	6.33E-05	868	
10	rs11599750	1.02E+08	9.36E-01	CPN1	13	rs2116412	74725315	5.10E-02	LOC647288	-	8.86E-08	3.33E-01	1.55E-04	869	
2	rs4668246	1.71E+08	6.32E-01	MYO3B	4	rs6840580	1.3E+08	3.73E-01	N/A	-	8.88E-08	5.10E-01	7.98E-05	870	
2	rs7608475	2.41E+08	3.02E-01	N/A	9	rs1873738	80967681	1.18E-01	N/A	-	8.90E-08	7.96E-01	3.07E-05	871	
2	rs2368502	2.41E+08	3.02E-01	N/A	9	rs1873738	80967681	1.18E-01	N/A	-	8.90E-08	8.13E-01	2.91E-05	872	
2	rs10207571	2.41E+08	3.02E-01	N/A	9	rs1873738	80967681	1.18E-01	N/A	-	8.90E-08	8.46E-01	2.54E-05	873	
4	rs1443207	32981890	5.50E-01	N/A	11	rs2041466	22769111	4.37E-02	GAS2	-	8.91E-08	2.73E-01	9.16E-06	874	
7	rs160342	28630355	1.95E-01	CREB5	9	rs4350058	24024072	5.39E-01	N/A	-	8.92E-08	2.98E-02	2.87E-08	875	
1	rs11207039	57696027	8.61E-01	DAB1	5	rs1251575	3398843	6.84E-01	N/A	-	8.95E-08	8.03E-01	5.94E-05	876	
7	rs7806531	70661813	1.54E-01	WBSCR17	9	rs3789311	1.23E+08	7.66E-01	CEP110	-	8.96E-08	3.75E-02	3.28E-08	877	
6	rs9295957	31265572	2.18E-01	POU5F1	6	rs9368675	31380136	7.91E-01	N/A	MHC	8.96E-08	4.77E-01	5.74E-06	878	
13	rs3885907	30212455	9.28E-01	ALOX5AP	16	rs1293323	85669131	9.81E-01	N/A	-	8.97E-08	2.62E-01	4.56E-04	879	
8	rs7845730	58512321	9.18E-01	N/A	18	rs1421265	52617413	6.52E-01	WDR7	-	8.99E-08	4.50E-01	3.45E-04	880	
8	rs6991253	58521794	9.18E-01	N/A	18	rs1421265	52617413	6.52E-01	WDR7	-	8.99E-08	6.69E-01	1.07E-04	881	
8	rs6980937	58516550	9.18E-01	N/A	18	rs1421265	52617413	6.52E-01	WDR7	-	8.99E-08	N/A	N/A	882	
3	rs716534	1.11E+08	2.91E-01	N/A	16	rs1834016	56833286	1.34E-01	CCDC113	-	8.99E-08	4.51E-01	2.15E-04	883	
10	rs12219393	18795615	8.52E-01	CACNB2	13	rs1106924	96313752	1.29E-01	N/A	-	9.00E-08	4.23E-01	5.93E-07	884	
3	rs17042584	5468089	9.34E-02	N/A	5	rs4540217	1.59E+08	1.01E-01	PWWP2A	-	9.00E-08	1.07E-01	4.69E-03	885	
1	rs633995	1.7E+08	6.98E-01	DNM3	10	rs7923705	1952508	3.76E-01	N/A	-	9.00E-08	2.85E-02	1.23E-02	886	
1	rs1655200	1.18E+08	6.07E-01	N/A	2	rs7421653	2.34E+08	8.14E-01	INPP5D	-	9.00E-08	3.44E-01	3.15E-04	887	
4	rs3923993	1.53E+08	3.61E-01	N/A	7	rs2188324	88162442	3.41E-01	N/A	-	9.02E-08	4.28E-01	7.33E-07	888	
2	rs12694536	2.21E+08	4.14E-01	N/A	5	rs1770242	1.36E+08	6.28E-01	N/A	-	9.02E-08	9.30E-01	7.93E-05	889	
7	rs11981351	7298240	4.09E-01	N/A	14	rs1959335	61549704	8.67E-01	SYT16	-	9.03E-08	4.61E-01	5.94E-05	890	
2	rs10518819	57568221	2.59E-01	N/A	13	rs475992	22772150	6.82E-02	SGCG	-	9.06E-08	3.50E-01	5.12E-04	891	
8	rs13274016	15642573	4.45E-01	TUSC3	18	rs2432842	68860542	1.84E-01	N/A	-	9.06E-08	6.96E-01	7.16E-06	892	
5	rs3903000	52208612	8.96E-01	N/A	22	rs8137110	41141106	1.63E-01	NFAM1	-	9.08E-08	6.42E-01	6.91E-06	893	
3	rs6444269	1.89E+08	9.23E-01	N/A	8	rs1995317	1.33E+08	2.79E-01	EFR3A	-	9.08E-08	4.60E-02	1.08E-03	894	

SNP A					SNP B						Interaction P			Ranking	Cluster in top 100 interactions
CHR	SNP	Location	gle locus P va	Gene	CHR	SNP	Location	ingle locus P valu	Gene	HC region	Stage 1	Stage 2	Combined		
3	rs10514694	36283073	2.94E-01	N/A	16	rs12149452	6977388	9.69E-01	A2BP1	-	9.09E-08	5.70E-01	8.32E-06	895	
6	rs9360723	74784936	3.52E-01	N/A	2	rs16822401	1.83E+08	9.60E-01	PPP1R1C	-	9.10E-08	6.24E-01	8.21E-05	896	
6	rs4706565	74785439	3.52E-01	N/A	2	rs16822401	1.83E+08	9.60E-01	PPP1R1C	-	9.10E-08	6.61E-01	7.04E-05	897	
7	rs11767427	1.44E+08	9.06E-01	N/A	9	rs7865913	90107350	7.91E-01	N/A	-	9.16E-08	4.43E-01	2.33E-05	898	
1	rs4927133	54726111	5.44E-01	N/A	5	rs439274	79454132	6.22E-01	SERINC5	-	9.17E-08	9.70E-01	1.57E-05	899	
1	rs2094026	1.94E+08	8.67E-01	N/A	2	rs3911080	70573427	5.09E-01	TGFA	-	9.18E-08	3.48E-01	5.60E-05	900	
1	rs4658018	1.94E+08	8.67E-01	N/A	2	rs3911080	70573427	5.09E-01	TGFA	-	9.18E-08	N/A	N/A	901	
9	rs10114153	11920627	9.70E-01	N/A	22	rs5992838	16644831	1.46E-01	BID	-	9.18E-08	6.03E-02	1.27E-07	902	
2	rs6721434	25523021	6.21E-01	DTNB	8	rs7821595	20925819	4.09E-01	N/A	-	9.19E-08	4.05E-01	6.48E-06	903	
6	rs2265917	1.46E+08	2.90E-01	SHPRH	10	rs1318662	26860966	6.27E-01	APBB1IP	-	9.19E-08	8.41E-01	1.17E-04	904	
7	rs509834	70775938	3.81E-01	WBSCR17	10	rs7896001	8397305	6.35E-01	N/A	-	9.20E-08	4.63E-01	1.45E-04	905	
7	rs509834	70775938	3.81E-01	WBSCR17	10	rs1892064	8401217	6.35E-01	N/A	-	9.20E-08	5.55E-01	1.08E-04	906	
2	rs1871780	1.58E+08	2.96E-01	N/A	9	rs356669	97087614	2.60E-01	FANCC	-	9.22E-08	5.77E-01	7.92E-05	907	
1	rs6704127	55842970	8.76E-01	N/A	16	rs4371172	63228918	4.22E-01	N/A	-	9.22E-08	6.32E-01	1.10E-04	908	
11	rs10488768	1.1E+08	8.96E-01	ARHGAP20	21	rs2026220	25436520	7.73E-01	N/A	-	9.22E-08	8.26E-01	4.72E-05	909	
11	rs997275	1.1E+08	8.96E-01	ARHGAP20	21	rs2026220	25436520	7.73E-01	N/A	-	9.22E-08	8.26E-01	4.72E-05	910	
10	rs2493765	12882926	5.83E-01	CAMK1D	12	rs12313609	97856730	2.80E-01	ANKS1B	-	9.24E-08	N/A	N/A	911	
5	rs7720463	1.8E+08	6.54E-01	RASGEF1C	7	rs160357	28635027	3.56E-01	CREB5	-	9.25E-08	5.88E-01	1.05E-04	912	
5	rs10464035	1.07E+08	5.27E-01	N/A	8	rs980238	4270026	1.10E-01	CSMD1	-	9.27E-08	4.73E-01	2.24E-04	913	
4	rs7667668	1.39E+08	4.67E-01	N/A	16	rs1463462	17161560	9.44E-01	XYLT1	-	9.28E-08	2.74E-01	6.27E-07	914	
1	rs12044804	2.05E+08	4.22E-01	IL19	2	rs6726292	55010134	1.06E-01	EML6	-	9.28E-08	5.18E-02	2.24E-03	915	
4	rs1159315	46697833	4.76E-01	GABRA4	7	rs41825	1.06E+08	5.84E-01	N/A	-	9.29E-08	7.59E-01	1.43E-05	916	
8	rs2981060	70121659	5.30E-01	N/A	14	rs10140426	99446255	7.38E-01	EML1	-	9.29E-08	N/A	N/A	917	
6	rs2064501	1.38E+08	5.73E-02	IL22RA2	7	rs4728404	1.36E+08	1.65E-01	N/A	-	9.31E-08	1.46E-01	7.64E-07	918	
6	rs2395185	32541145	6.23E-01	N/A	19	rs1004246	16317260	8.23E-01	EPS15L1	MHC	9.32E-08	9.57E-01	2.05E-05	919	
4	rs1451634	1.52E+08	6.54E-01	LRBA	20	rs856333	50750148	9.42E-01	N/A	-	9.32E-08	9.57E-01	2.05E-05	920	
4	rs4696222	1.52E+08	6.54E-01	LRBA	20	rs856333	50750148	9.42E-01	N/A	-	9.32E-08	9.31E-01	3.58E-05	921	
6	rs2233984	31187243	4.20E-02	C6orf15	6	rs3094205	31199841	5.91E-01	C6orf15	MHC	9.33E-08	7.32E-02	2.73E-07	922	
1	rs10921046	1.9E+08	7.83E-01	N/A	17	rs8079495	67829583	2.00E-01	N/A	-	9.34E-08	2.90E-01	1.60E-06	923	
3	rs2251423	62514776	5.94E-01	CADPS	9	rs10982110	1.16E+08	9.39E-01	COL27A1	-	9.35E-08	2.12E-01	3.96E-03	924	
5	rs1030179	1.23E+08	6.89E-01	N/A	10	rs4935672	58292847	2.85E-01	N/A	-	9.35E-08	4.40E-01	9.72E-05	925	
15	rs2034247	78399486	8.59E-01	N/A	19	rs1978717	57189062	4.91E-01	ZNF350	-	9.35E-08	4.59E-01	1.48E-06	926	
5	rs274362	53372577	5.22E-01	ARL15	17	rs820206	71153309	5.26E-01	RECQL5	-	9.36E-08	5.09E-01	2.12E-04	927	
8	rs9657404	4768285	8.75E-01	CSMD1	13	rs9556587	95877619	1.76E-01	HS6ST3	-	9.36E-08	2.64E-01	3.11E-06	928	
6	rs6919894	6440521	9.16E-01	LOC285780	9	rs11103281	1.38E+08	8.32E-01	CAPN8	-	9.39E-08	N/A	N/A	929	
4	rs1110956	1.54E+08	4.94E-01	FHDC1	14	rs4983413	1.05E+08	3.35E-01	MTA1	-	9.40E-08	5.90E-01	3.20E-04	930	
5	rs10941404	38785588	5.07E-01	N/A	10	rs4747211	73296229	7.50E-01	PSAP	-	9.42E-08	4.35E-01	1.13E-05	931	
5	rs4128777	38785799	5.07E-01	N/A	10	rs4747211	73296229	7.50E-01	PSAP	-	9.42E-08	4.35E-01	1.13E-05	932	
10	rs1317790	28419610	4.31E-01	MPP7	19	rs1661926	58324708	8.62E-01	ZNF415	-	9.44E-08	6.82E-01	5.96E-05	933	
5	rs967771	1.61E+08	4.73E-01	GABRB2	22	rs9306511	47201283	2.35E-01	N/A	-	9.45E-08	5.95E-01	1.04E-05	934	
5	rs729129	1.8E+08	1.04E-01	N/A	7	rs851814	1.47E+08	7.68E-01	CNTN/AP2	-	9.46E-08	6.35E-01	1.17E-04	935	
5	rs17745927	1.7E+08	9.72E-01	KCNIP1	7	rs10215566	1.28E+08	8.76E-01	N/A	-	9.47E-08	N/A	N/A	936	
6	rs9350088	10203442	4.07E-01	N/A	12	rs4148665	21926514	3.93E-01	ABCC9	-	9.48E-08	3.09E-02	1.14E-07	937	
13	rs7986907	78628865	9.19E-01	N/A	15	rs4886617	71876846	4.23E-01	N/A	-	9.48E-08	4.97E-01	3.18E-06	938	
5	rs1368298	1.58E+08	4.33E-01	EBF1	8	rs10503585	16758568	6.56E-01	N/A	-	9.49E-08	1.10E-01	8.18E-04	939	
4	rs6812217	1.59E+08	4.71E-01	N/A	10	rs10826158	59558004	9.04E-01	N/A	-	9.50E-08	3.66E-01	1.67E-06	940	
4	rs7668273	26781226	5.93E-01	N/A	12	rs7977912	91087603	3.57E-01	N/A	-	9.51E-08	6.46E-01	9.30E-05	941	
1	rs4657399	1.63E+08	5.39E-01	N/A	9	rs1877480	2648311	9.25E-02	VLDLR	-	9.52E-08	8.59E-01	3.49E-05	942	
9	rs1452336	28114180	9.45E-01	LINGO2	20	rs4911234	30522023	9.26E-01	C20orf112	-	9.52E-08	9.96E-02	1.42E-06	943	
4	rs2061231	45333604	5.46E-01	N/A	8	rs10504828	87986352	2.68E-01	CNBD1	-	9.53E-08	1.86E-01	7.16E-03	944	
1	rs11161943	87427194	7.56E-01	LOC339524	16	rs1981492	296690	4.85E-01	AXIN1	-	9.54E-08	9.80E-02	3.00E-04	945	
3	rs1568573	1.62E+08	5.45E-01	PPM1L	20	rs858140	24009345	2.02E-01	N/A	-	9.55E-08	5.14E-01	2.85E-06	946	
11	rs665714	82588909	2.06E-01	PCF11	22	rs743185	27433217	6.71E-01	CHEK2	-	9.55E-08	1.46E-01	2.29E-07	947	
11	rs555586	82589527	2.06E-01	PCF11	22	rs743185	27433217	6.71E-01	CHEK2	-	9.55E-08	1.55E-01	2.47E-07	948	
2	rs2551214	2335000	2.21E-01	N/A	3	rs7648149	45220637	1.21E-01	N/A	-	9.56E-08	9.20E-01	3.60E-05	949	
5	rs4704506	77690583	5.04E-01	SCAMP1	11	rs11236029	73555872	3.42E-01	C2CD3	-	9.56E-08	7.54E-01	1.23E-05	950	
11	rs11028806	3633400	9.35E-01	NUP98	22	rs695504	32330230	8.08E-01	LARGE	-	9.57E-08	2.81E-01	4.00E-07	951	
15	rs723434	72130383	7.63E-01	PML	18	rs7505930	4082001	5.75E-01	N/A	-	9.58E-08	1.72E-01	1.33E-04	952	
8	rs2278092	30721347	5.96E-01	N/A	22	rs7290191	17902510	5.00E-01	N/A	-	9.59E-08	7.22E-01	2.74E-04	953	
1	rs4658104	91860110	9.84E-01	HSP90B3P	7	rs329580	35058400	4.68E-01	DPY19L1	-	9.60E-08	5.58E-01	3.91E-06	954	
1	rs1504698	1.64E+08	9.22E-01	N/A	5	rs4701081	1.79E+08	6.14E-02	ADAMTS2	-	9.61E-08	4.26E-01	9.23E-06	955	
8	rs975729	1.29E+08	2.88E-01	N/A	16	rs1657091	8902915	5.57E-01	USP7	-	9.62E-08	1.82E-01	1.65E-03	956	
8	rs975730	1.29E+08	2.88E-01	N/A	16	rs1657091	8902915	5.57E-01	USP7	-	9.62E-08	2.07E-01	1.46E-03	957	
1	rs10489667	1.15E+08	9.12E-01	N/A	12	rs956025	1.18E+08	6.26E-01	N/A	-	9.62E-08	4.77E-01	7.27E-05	958	
1	rs12042641	1.65E+08	5.58E-01	GPA33	12	rs2632226	75535673	3.99E-01	N/A	-	9.63E-08	5.78E-01	2.60E-05	959	
7	rs2062403	68146581	5.93E-01	N/A	7	rs9638581	69189129	9.37E-02	AUTS2	-	9.67E-08	9.08E-01	1.94E-05	960	
2	rs11889825	2.39E+08	4.38E-01	N/A	5	rs561738	84650492	5.07E-01	N/A	-	9.67E-08	9.45E-01	3.71E-05	961	
3	rs2590453	1.89E+08	8.62E-01	N/A	20	rs6081622	19441514	2.89E-01	SLC24A3	-	9.69E-08	7.26E-02	1.17E-03	962	
6	rs9355736	1.6E+08	5.17E-01	N/A	7	rs10234191	1.03E+08	4.38E-01	N/A	-	9.71E-08	9.32E-01	2.31E-05	963	
3	rs2375840	1.47E+08	1.56E-01	N/A	18	rs1551899	11543868	1.10E-01	N/A	-	9.73E-08	2.53E-01	9.22E-05	964	
3	rs6440364	1.47E+08	1.56E-01	N/A	18	rs1551899	11543868	1.10E-01	N/A	-	9.73E-08	2.53E-01	9.22E-05	965	
16	rs2526269	2996585	4.66E-01	CLDN9	22	rs133986	26354182	8.54E-01	N/A	-	9.74E-08	5.65E-02	6.82E-04	966	
11	rs6578868	7469166	9.70E-01	OLFML1	19	rs6510092	35463597	6.57E-01	N/A	-	9.75E-08	1.71E-02	1.23E-02	967	
7	rs12701156	31910279	1.53E-01	N/A	9	rs9314841	70823865	9.59E-01	FXN	-	9.77E-08	4.79E-01	1.60E-05	968	
7	rs6962770	31922742	1.53E-01	N/A	9	rs9314841	70823865	9.59E-01	FXN	-	9.77E-08	4.79E-01	1.60E-05	969	
2	rs11899692	47321835	6.25E-01	N/A	3	rs6440278	1.46E+08	5.79E-01	N/A	-	9.80E-08	6.07E-01	3.71E-04	970	
2	rs1405719	1.19E+08	1.68E-01	N/A	9	rs4842173	1.37E+08	9.22E-01	COL5A1	-	9.81E-08	2.67E-01	7.40E-06	971	
9	rs10960376	11919890	9.64E-01	N/A	22	rs5992838	16644831	1.46E-01	BID	-	9.81E-08	5.86E-02	1.21E-07	972	
2	rs291335	2.4E+08	9.65E-01	HDAC4	7	rs7804360	7621768	3.21E-01	MIOS	-	9.82E-08	2.13E-01	5.97E-04	973	
6	rs727811	1.65E+08	3.59E-02	N/A	7	rs6947977	77979596	5.32E-01	MAGI2	-	9.83E-08	6.65E-01	9.61E-06	974	
2	rs6749677	1.19E+08	2.42E-01	N/A	9	rs4842173	1.37E+08	9.22E-01	COL5A1	-	9.84E-08	3.56E-01	1.27E-05	975	
6	rs11968290	1.65E+08	4.52E-02	N/A	7	rs6947977	77979596	5.32E-01	MAGI2	-	9.85E-08	9.80E-01	2.99E-05	976	
6	rs6937258	93450044	5.04E-01	N/A	2	rs10173035	1.68E+08	3.08E-02	N/A	-	9.85E-08	2.76E-01	5.25E-04	977	
2	rs7561066	2.15E+08	2.97E-01	SPAG16	13	rs9516948	96928731	8.96E-01	RAP2A	-	9.87E-08	7.51E-01	7.26E-05	978	
4	rs7682883	32593729	1.05E-01	N/A	20	rs6037513	3171225	7.60E-01	C20orf194	-	9.88E-08	7.04E-01	8.99E-05	979	
19	rs4802856	56917579	4.09E-01	NCRN/A00085	22	rs738322	36898952	9.90E-01	PLA2G6	-	9.90E-08	9.59E-01	3.19E-05	980	
10	rs7902949	53602572	1.24E-01	PRKG1	15	rs12913071	69598123	9.91E-01	THSD4	-	9.92E-08	4.33E-01	1.93E-06	981	
3	rs1628472	1.18E+08	8.75E-01	N/A	8	rs2280880	1.44E+08	5.62E-02	JRK	-	9.92E-08	9.23E-02	4.18E-04	982	
16	rs1024647	7732249	3.56E-02	N/A	17	rs4128646	10022981	5.72E-02	GAS7	-	9.94E-08	N/A	N/A	983	
7	rs2237353	28764739	5.03E-01	CREB5	17	rs1607979	48688258	3.78E-01	N/A	-	9.96E-08	5.87E-04	9.86E-11	984	
1	rs12091627	2.18E+08	9.26E-01	N/A	7	rs1076283	1.49E+08	2.59E-01	N/A	-	9.98E-08	8.63E-01	4.30E-05	985	
7	rs10246727	1.53E+08	4.18E-01	N/A	9	rs10125911	1.28E+08	9.82E-01	N/A	-	1.00E-07	8.72E-02	7.73E-08	986	
13	rs1025410	1.09E+08	8.54E-01	MYO16	17	rs3744734	399648	8.60E-01	VPS53	-	1.00E-07	7.80E-01	1.51E-05	987	
7	rs11983822	13067583	7.10E-02	N/A	19	rs3746228	62496174	5.82E-01	ZNF460	-	1.00E-07	6.59E-01	4.10E-06	988	
6	rs2747690	1.64E+08	7.58E-01	PACRG	12	rs1044825	1771746	3.51E-01	ADIPOR2	-	1.00E-07	6.63E-01	6.87E-06	989	
3	rs6766004	28988908	2.46E-01	N/A	15	rs4842935	81431271	5.68E-01	HOMER2	-	1.01E-07	2.89E-01	5.64E-04	990	
1	rs4656520	1.65E+08	6.30E-01	GPA33	12	rs2669010	75533191	8.86E-02	N/A	-	1.01E-07	N/A	N/A	991	
2	rs6760087	2.05E+08	8.79E-01	PARD3B	19	rs4807078	6255967	8.30E-02	ACER1	-	1.01E-07	1.59E-01	1.85E-06	992	
7	rs1868757	1.14E+08	8.12E-01	N/A	14	rs1315728	67013662	9.59E-01	TMEM229B	-	1.01E-07	5.32E-01	3.69E-04	993	
5	rs2069187	54433806	6.90E-01	CDC20B	9	rs1509186	1085922	8.28E-01	N/A	-	1.01E-07	2.22E-01	1.15E-03	994	
3	rs1499153	7123907	9.84E-01	GRM7	12	rs1565728	75952082	6.02E-01	E2F7	-	1.01E-07	9.81E-01	1.03E-05	995	
7	rs7789215	77933529	9.47E-01	MAGI2	19	rs734379	63590994	3.57E-01	ZNF837	-	1.01E-07	9.19E-01	5.85E-05	996	
3	rs2581830	53109138	1.13E-01	RFT1	17	rs2301775	34594761	8.82E-01	CACNB1	-	1.01E-07	N/A	N/A	997	
5	rs2368097	39694775	1.13E-01	N/A	9	rs1856197	33688114	5.73E-01	N/A	-	1.01E-07	5.77E-02	1.67E-03	998	
2	rs1455162	1.38E+08	4.22E-01	HNMT	5	rs1025482	1.67E+08	1.28E-01	ODZ2	-	1.02E-07	6.84E-01	2.15E-05	999	
5	rs4386763	1.16E+08	5.37E-01	COMMD10	18	rs7240382	72579628	1.92E-01	N/A	-	1.02E-07	6.43E-01	8.77E-06	1000	
3	rs2581628	1.44E+08	8.77E-01	N/A	8	rs6996111	1.21E+08	1.55E-01	N/A	-	1.02E-07	N/A	N/A	1001	
4	rs2955256	1.71E+08	8.93E-01	AADAT	13	rs17068084	18566347	1.92E-01	DKFZp686A162	-	1.02E-07	9.38E-01	8.34E-05	1002	
5	rs6869329	2138954	5.10E-01	N/A	8	rs1430849	1.26E+08	1.05E-01	MTSS1	-	1.02E-07	3.15E-01	2.25E-03	1003	
1	rs1874807	85551981	7.14E-01	DDAH1	5	rs7447258	35792452	2.83E-01	SPEF2	-	1.02E-07	1.82E-02	3.22E-02	1004	
6	rs9395787	13552717	7.23E-01	GFOD1	6	rs4307206	1.19E+08	8.19E-01	N/A	-	1.02E-07	2.01E-01	1.13E-06	1005	
1	rs3002303	2.13E+08	8.18E-01	PTPN14	13	rs12584299	1.08E+08	7.00E-02	N/A	-	1.02E-07	6.30E-01	1.10E-04	1006	

SNP A					SNP B						Interaction P			Ranking	Cluster in top 100 interactions
CHR	SNP	Location	gle locus P va	Gene	CHR	SNP	Location	ingle locus P valu	Gene	HC region	Stage 1	Stage 2	Combined		
7	rs2888889	92376825	4.75E-01	N/A	8	rs2249536	69486976	2.13E-01	N/A	-	1.02E-07	6.39E-01	2.30E-06	1007	
4	rs1993616	1.74E+08	5.91E-01	GALNTL6	22	rs7293017	18113811	5.60E-02	TBX1	-	1.02E-07	6.60E-02	4.00E-07	1008	
6	rs4947296	31166157	4.31E-02	N/A	6	rs3868542	31253818	6.85E-02	TCF19	MHC	1.02E-07	4.15E-01	1.32E-05	1009	
9	rs1231343	25732531	4.72E-01	N/A	17	rs9898180	48469648	8.18E-01	N/A	-	1.03E-07	9.69E-02	3.62E-07	1010	
2	rs11695281	28540072	3.33E-02	N/A	17	s17636929	14698780	4.93E-01	N/A	-	1.03E-07	4.19E-01	5.69E-04	1011	
7	rs10951658	41755259	4.59E-01	LOC285954	17	rs895690	70296244	2.37E-01	SLC9A3R1	-	1.03E-07	5.00E-01	2.11E-04	1012	
5	rs252155	1.5E+08	6.37E-01	RBM22	8	s11787089	62783393	3.57E-01	ASPH	-	1.03E-07	5.61E-01	1.61E-05	1013	
2	rs4611674	638899	2.98E-03	TMEM18	9	rs3812541	1.4E+08	3.64E-01	CACN/A1B	-	1.03E-07	3.45E-01	2.28E-06	1014	
3	rs1628472	1.18E+08	8.75E-01	N/A	8	rs3802232	1.44E+08	2.77E-01	JRK	-	1.03E-07	3.46E-02	1.10E-03	1015	
11	rs952131	1.29E+08	6.41E-01	N/A	19	rs2255598	24248960	9.93E-01	N/A	-	1.03E-07	9.06E-01	1.39E-04	1016	
2	rs4281898	2.28E+08	5.50E-01	RHBDD1	17	rs4239112	10096227	3.32E-01	N/A	-	1.03E-07	4.05E-01	5.73E-07	1017	
10	rs7074840	1.28E+08	1.63E-01	N/A	12	rs1566575	41963432	3.88E-01	N/A	-	1.03E-07	2.24E-01	8.12E-04	1018	
10	rs4746372	77608248	4.90E-01	C10orf11	20	rs6107589	5098051	5.46E-02	CDS2	-	1.03E-07	6.65E-01	1.44E-05	1019	
10	rs12243844	55532600	6.59E-01	PCDH15	11	rs657339	73886240	6.83E-02	LIPT2	-	1.04E-07	5.96E-02	2.09E-03	1020	
3	rs9811344	1.14E+08	9.41E-01	N/A	5	s10479044	1.33E+08	2.60E-01	FSTL4	-	1.04E-07	6.52E-02	2.66E-07	1021	
1	rs1808806	1.18E+08	8.04E-01	FAM46C	2	rs2695616	57761698	3.10E-01	N/A	-	1.04E-07	7.76E-01	3.66E-05	1022	
6	rs9480096	1.51E+08	4.85E-01	PPP1R14C	5	s12520518	53820991	1.16E-01	N/A	-	1.04E-07	5.09E-01	8.12E-07	1023	
18	rs767887	3643422	4.12E-01	DLGAP1	20	rs6080604	17136874	7.42E-01	PCSK2	-	1.04E-07	4.67E-01	4.83E-06	1024	
4	rs7690190	16128817	8.77E-01	LDB2	8	s11136715	4118520	9.92E-01	CSMD1	-	1.04E-07	6.05E-01	5.98E-06	1025	
2	rs10207182	2.14E+08	4.13E-01	SPAG16	14	rs1885147	28063661	9.81E-01	N/A	-	1.04E-07	1.43E-01	5.92E-07	1026	
3	rs2724693	1.39E+08	3.64E-01	N/A	8	rs1500906	96601801	1.79E-01	N/A	-	1.04E-07	1.51E-01	2.07E-03	1027	
3	rs2724693	1.39E+08	3.64E-01	N/A	8	rs1500909	96602267	1.79E-01	N/A	-	1.04E-07	1.51E-01	2.07E-03	1028	
8	rs4875428	4841962	7.27E-01	CSMD1	8	rs4581170	1.43E+08	8.94E-01	TSN/ARE1	-	1.04E-07	3.92E-02	4.23E-08	1029	
8	rs7833343	58513774	8.93E-01	N/A	18	rs1421265	52617413	6.52E-01	WDR7	-	1.04E-07	1.88E-01	8.72E-04	1030	
2	rs2253680	1.7E+08	8.71E-02	FASTKD1	11	rs4757326	15444590	6.88E-01	N/A	-	1.04E-07	4.50E-01	3.77E-04	1031	
6	rs3131003	31201461	6.51E-01	C6orf15	6	rs3130467	31295054	5.92E-02	HCG27	MHC	1.04E-07	5.06E-01	3.95E-05	1032	
2	rs2253680	1.7E+08	8.71E-02	FASTKD1	11	rs7479920	15430164	6.88E-01	N/A	-	1.04E-07	8.14E-01	3.46E-05	1033	
3	rs9852746	55778372	2.40E-01	ERC2	19	rs1860565	62026834	7.53E-01	PEG3	-	1.04E-07	8.65E-01	4.17E-05	1034	
9	rs10117492	11924556	8.13E-01	N/A	22	rs5992838	16644831	1.46E-01	BID	-	1.05E-07	7.36E-02	1.72E-07	1035	
3	rs1871493	1.42E+08	8.68E-01	CLSTN2	5	s17769571	1.29E+08	8.95E-01	CHSY3	-	1.05E-07	8.64E-01	3.25E-05	1036	
7	rs11764649	12537614	3.90E-01	N/A	19	rs2074879	13233589	5.33E-01	CACN/A1A	-	1.05E-07	3.35E-01	2.71E-04	1037	
1	rs10864702	2.3E+08	4.93E-01	TSN/AX-DISC1	2	s10197319	2.04E+08	7.93E-01	N/A	-	1.05E-07	4.75E-01	3.54E-05	1038	
1	rs10864702	2.3E+08	4.93E-01	TSN/AX-DISC1	2	s12616245	2.04E+08	7.93E-01	N/A	-	1.05E-07	4.75E-01	3.54E-05	1039	
11	rs952131	1.29E+08	6.41E-01	N/A	19	s10405242	24166259	8.92E-01	N/A	-	1.05E-07	9.68E-01	7.46E-05	1040	
6	rs12528284	1.67E+08	6.57E-01	RPS6KA2	12	rs406117	91323127	2.14E-01	CLLU1	-	1.05E-07	2.15E-01	4.39E-04	1041	
11	rs3901366	1.2E+08	7.75E-01	N/A	16	s11640875	81278925	3.67E-02	CDH13	-	1.05E-07	6.10E-01	1.31E-05	1042	
7	rs4543487	82641622	3.47E-01	PCLO	12	rs1495778	40272610	1.98E-01	PDZRN4	-	1.05E-07	2.28E-01	9.17E-06	1043	
5	rs17745927	1.7E+08	9.72E-01	KCNIP1	7	rs6958788	1.28E+08	8.77E-01	FAM71F2	-	1.05E-07	2.71E-01	2.17E-06	1044	
5	rs4615276	1.06E+08	1.15E-01	N/A	12	rs4763075	61962124	4.02E-01	N/A	-	1.05E-07	7.97E-01	6.08E-05	1045	
17	rs17810148	9658805	1.38E-01	GLP2R	22	rs2318406	47713629	2.45E-01	N/A	-	1.05E-07	6.00E-01	1.95E-04	1046	
9	rs4877228	83790792	6.16E-02	FLJ46321	21	rs6517385	37156142	7.73E-01	HLCS	-	1.05E-07	6.05E-01	3.31E-06	1047	
5	rs4348193	77671476	5.91E-01	N/A	11	rs3741135	73391608	7.68E-01	UCP3	-	1.05E-07	7.61E-01	4.35E-05	1048	
19	rs12971499	7165282	4.79E-01	INSR	19	rs2071572	60378042	1.78E-01	PTPRH	-	1.06E-07	8.64E-01	1.48E-04	1049	
6	rs2025866	85453231	9.61E-01	N/A	1	rs6656361	1.45E+08	7.69E-01	LOC728989	-	1.06E-07	N/A	N/A	1050	
6	rs4407684	85500368	9.61E-01	TBX18	1	rs6656361	1.45E+08	7.69E-01	LOC728989	-	1.06E-07	N/A	N/A	1051	
1	rs3002297	2.13E+08	7.72E-01	PTPN14	13	s10492664	1.08E+08	6.04E-02	N/A	-	1.06E-07	6.64E-01	9.62E-05	1052	
5	rs11949188	1.68E+08	3.45E-01	WWC1	8	rs2670027	57586721	2.94E-01	N/A	-	1.06E-07	5.37E-01	3.87E-06	1053	
9	rs2183124	33105556	6.29E-01	B4GALT1	9	s11143080	73846129	2.00E-01	C9orf57	-	1.06E-07	9.71E-01	1.79E-04	1054	
2	rs11691849	85248122	3.01E-01	TCF7L1	7	s10951853	5249937	9.05E-01	WIPI2	-	1.06E-07	9.78E-01	2.38E-05	1055	
2	rs4853333	77772523	7.72E-01	N/A	7	s11543821	1.34E+08	3.66E-01	AGBL3	-	1.06E-07	5.98E-01	2.10E-04	1056	
5	rs1382342	1.16E+08	8.12E-01	COMMD10	18	rs7240382	72579628	1.92E-01	N/A	-	1.06E-07	7.04E-01	3.28E-05	1057	
4	rs1824864	22214157	6.39E-01	N/A	16	rs4344741	49000892	1.50E-01	N/A	-	1.06E-07	9.15E-01	3.30E-05	1058	
6	rs9358131	17661992	3.30E-01	CAP2	7	s11772815	28357572	3.06E-01	CREB5	-	1.06E-07	7.70E-01	9.60E-05	1059	
12	rs319284	11276538	2.09E-01	N/A	13	rs9559465	1.08E+08	1.14E-01	MYO16	-	1.06E-07	2.55E-01	3.76E-06	1060	
1	rs1832899	56974459	8.89E-01	C1orf168	8	rs1460399	3018385	7.11E-01	CSMD1	-	1.06E-07	6.87E-01	1.58E-04	1061	
5	rs10515241	95988347	3.03E-01	N/A	14	rs1010452	94249523	6.44E-01	N/A	-	1.07E-07	2.36E-01	3.63E-06	1062	
17	rs9909852	20231611	2.03E-01	CCDC144C	18	s11876558	29643580	4.76E-01	N/A	-	1.07E-07	5.81E-01	3.06E-04	1063	
6	rs9368675	31380136	7.91E-01	N/A	6	rs2844575	31442924	5.47E-01	HLA-B	MHC	1.07E-07	9.48E-01	9.41E-05	1064	
6	rs4897358	1.3E+08	1.08E-01	N/A	14	rs1397163	91173949	1.20E-01	CATSPERB	-	1.07E-07	9.86E-01	3.82E-05	1065	
7	rs4731401	1.28E+08	3.69E-01	N/A	18	s11872471	49114530	1.56E-01	DCC	-	1.07E-07	5.33E-01	5.00E-06	1066	
6	rs9355443	1.64E+08	5.34E-01	N/A	17	rs4794031	44697152	8.27E-01	N/A	-	1.07E-07	3.20E-01	1.29E-03	1067	
2	rs976904	1.33E+08	1.22E-01	NCKAP5	11	s10502208	1.15E+08	5.14E-01	N/A	-	1.07E-07	1.19E-01	6.15E-04	1068	
3	rs1350415	55888277	3.06E-01	ERC2	3	s11718099	1.87E+08	1.60E-01	N/A	-	1.07E-07	9.14E-01	7.36E-06	1069	
3	rs6550484	37603838	4.94E-01	ITGA9	8	rs7009980	4488686	4.40E-01	CSMD1	-	1.07E-07	4.13E-01	7.83E-06	1070	
3	rs6550483	37603715	4.94E-01	ITGA9	8	rs7009980	4488686	4.40E-01	CSMD1	-	1.07E-07	8.90E-01	9.73E-05	1071	
5	rs1432679	1.58E+08	5.12E-01	EBF1	8	s10503585	16758568	6.56E-01	N/A	-	1.07E-07	8.90E-01	9.73E-05	1072	
2	rs1466180	1.69E+08	7.53E-01	N/A	5	rs188693	42975987	7.34E-02	N/A	-	1.07E-07	1.54E-01	1.13E-03	1073	
9	rs7039047	11936421	7.63E-01	N/A	22	rs5992838	16644831	1.46E-01	BID	-	1.08E-07	4.53E-02	8.70E-08	1074	
5	rs10462803	5201635	8.83E-01	ADAMTS16	12	rs862231	89243803	5.47E-02	N/A	-	1.08E-07	N/A	N/A	1075	
3	rs1628472	1.18E+08	8.75E-01	N/A	8	rs4545075	1.44E+08	1.06E-01	N/A	-	1.08E-07	2.44E-01	2.02E-04	1076	
5	rs4457046	1.06E+08	5.53E-02	N/A	12	s12824017	61935103	2.85E-01	N/A	-	1.08E-07	7.10E-01	2.89E-05	1077	
2	rs6742683	1.07E+08	1.69E-02	N/A	3	rs566216	1.76E+08	5.95E-01	N/A	-	1.08E-07	6.95E-01	1.54E-05	1078	
2	rs11124141	1.07E+08	1.97E-02	N/A	3	rs566216	1.76E+08	5.95E-01	N/A	-	1.08E-07	6.95E-01	1.88E-05	1079	
1	rs1125489	2.38E+08	7.77E-01	CHRM3	5	rs163446	1.12E+08	8.34E-01	N/A	-	1.08E-07	2.26E-01	9.84E-07	1080	
3	rs4484141	1.51E+08	5.93E-01	N/A	16	rs397599	61204411	7.03E-01	N/A	-	1.09E-07	4.34E-01	8.22E-05	1081	
4	rs2871159	1.82E+08	8.18E-01	N/A	19	rs7259371	38226481	8.16E-01	RHPN2	-	1.09E-07	6.23E-01	2.05E-06	1082	
5	rs251022	1.41E+08	3.93E-01	DIAPH1	18	rs646700	74198598	4.72E-01	N/A	-	1.09E-07	6.98E-02	4.25E-03	1083	
11	rs952131	1.29E+08	6.41E-01	N/A	19	rs7253132	24170037	8.91E-01	N/A	-	1.09E-07	9.91E-01	6.45E-05	1084	
6	rs4235996	19576099	4.19E-01	N/A	1	s12027362	1.98E+08	2.62E-01	N/A	-	1.09E-07	4.70E-01	1.79E-05	1085	
14	rs1205106	71339421	5.44E-01	N/A	20	rs226834	46292121	6.68E-01	N/A	-	1.09E-07	6.00E-01	1.19E-05	1086	
10	rs7091494	14887155	9.95E-01	CDNF	16	rs9929588	19954457	7.98E-02	GPR139	-	1.09E-07	9.32E-01	1.51E-05	1087	
10	rs7477069	54212144	4.45E-01	MBL2	18	rs1523868	50216824	1.45E-01	N/A	-	1.09E-07	1.15E-01	4.63E-07	1088	
15	rs294544	92649372	1.37E-01	MCTP2	20	rs6085256	5655548	3.06E-01	N/A	-	1.09E-07	7.47E-01	1.32E-05	1089	
6	rs4495304	31188697	4.80E-02	C6orf15	6	rs9295957	31265572	2.18E-01	POU5F1	MHC	1.10E-07	1.32E-01	8.02E-07	1090	
11	rs1939904	1.3E+08	1.47E-02	N/A	13	rs3015361	1.05E+08	8.93E-01	N/A	-	1.10E-07	6.97E-01	3.40E-05	1091	
1	rs3820604	2.33E+08	9.13E-01	TARBP1	7	rs562850	1.05E+08	3.44E-01	ATXN7L1	-	1.10E-07	9.73E-01	1.64E-05	1092	
3	rs4857185	97394779	1.60E-01	N/A	15	rs8031186	82285073	2.16E-01	ADAMTSL3	-	1.10E-07	7.45E-01	3.09E-05	1093	
18	rs6566862	52950493	7.29E-01	N/A	20	rs2426214	49007217	9.38E-01	MOCS3	-	1.10E-07	2.45E-01	2.78E-04	1094	
1	rs2743208	18890442	2.92E-01	PAX7	3	s12630126	1.06E+08	1.14E-01	N/A	-	1.10E-07	2.93E-01	1.05E-06	1095	
2	rs1898716	2.16E+08	6.69E-01	N/A	17	rs8081956	5444682	5.44E-01	NLRP1	-	1.11E-07	7.32E-02	3.18E-07	1096	
6	rs4495304	31188697	4.80E-02	C6orf15	6	rs3094205	31199841	5.91E-01	C6orf15	MHC	1.11E-07	1.31E-01	3.00E-04	1097	
4	rs6533498	1.11E+08	6.67E-01	ELOVL6	7	rs6949694	32439081	4.04E-01	N/A	-	1.11E-07	1.89E-01	1.99E-06	1098	
13	rs736596	32674506	6.84E-01	STARD13	17	s11078220	13898143	9.76E-01	COX10	-	1.11E-07	7.61E-01	1.38E-05	1099	
7	rs2888889	92376825	4.75E-01	N/A	8	s10104993	69509383	1.91E-01	N/A	-	1.11E-07	N/A	N/A	1100	
9	rs1928202	7263813	6.54E-01	N/A	18	rs1553803	43377597	2.98E-01	N/A	-	1.11E-07	5.68E-01	4.08E-04	1101	
8	rs16886345	1.16E+08	4.30E-01	N/A	15	rs7173054	25550656	4.83E-01	N/A	-	1.11E-07	8.79E-01	3.39E-05	1102	
1	rs12033655	57685815	7.94E-01	DAB1	5	s12515754	3398843	6.84E-01	N/A	-	1.11E-07	8.09E-01	1.29E-05	1103	
8	rs579096	20846271	9.14E-01	N/A	9	s10974837	4885391	6.23E-01	N/A	-	1.11E-07	5.72E-01	5.38E-06	1104	
3	rs16851254	1.42E+08	2.89E-01	ACPL2	4	rs180146	1.74E+08	8.31E-01	GALNTL6	-	1.11E-07	7.67E-01	3.76E-05	1105	
1	rs12027754	63362033	5.97E-01	N/A	11	s11041391	7432637	5.65E-01	SYT9	-	1.12E-07	3.29E-01	9.12E-04	1106	
4	rs1110956	1.54E+08	4.94E-01	FHDC1	14	rs9671696	1.05E+08	3.73E-01	MTA1	-	1.12E-07	5.51E-01	1.75E-05	1107	
6	rs887464	31253899	8.59E-02	TCF19	6	rs396243	31383153	2.17E-01	N/A	MHC	1.12E-07	9.75E-01	5.01E-06	1108	
5	rs1030179	1.23E+08	6.89E-01	N/A	10	s11005559	58288085	2.59E-01	N/A	-	1.12E-07	4.71E-01	9.63E-05	1109	
13	rs736596	32674506	6.84E-01	STARD13	17	rs8079772	13890445	8.89E-01	N/A	-	1.12E-07	8.92E-01	7.86E-06	1110	
1	rs10493291	60909871	7.99E-01	N/A	20	rs11960	17548357	4.22E-01	DSTN	-	1.12E-07	9.55E-01	6.92E-05	1111	
10	rs7097370	19049704	2.15E-01	N/A	12	rs27633	28015572	7.56E-02	PTHLH	-	1.12E-07	4.81E-01	3.04E-05	1112	
5	rs4479880	1.6E+08	5.55E-01	ATP10B	16	rs6564118	84005439	7.46E-01	N/A	-	1.12E-07	2.90E-01	1.57E-03	1113	
4	rs12233914	19906678	2.98E-01	SLIT2	14	rs1676284	76133569	2.50E-02	N/A	-	1.12E-07	7.58E-01	1.38E-06	1114	
1	rs11207166	58307728	7.14E-01	DAB1	1	rs155552	1.81E+08	6.99E-01	RGSL1	-	1.12E-07	3.59E-01	6.68E-04	1115	
9	rs2417682	1.08E+08	5.72E-01	N/A	22	rs1883993	23541229	5.59E-01	SGSM1	-	1.12E-07	9.41E-01	4.67E-05	1116	
8	rs972665	5340694	2.22E-01	N/A	14	rs8018930	94309119	5.71E-01	GSC	-	1.12E-07	6.88E-01	1.45E-04	1117	
1	rs12564019	1.58E+08	9.29E-01	OR10J1	3	s10511074	85736123	5.53E-01	CADM2	-	1.12E-07	2.42E-01	1.79E-06	1118	

SNP A					SNP B						Interaction P			Ranking	Cluster in top 100 interactions
CHR	SNP	Location	gle locus P va	Gene	CHR	SNP	Location	ingle locus P valu	Gene	HC region	Stage 1	Stage 2	Combined		
3	rs10804521	1.17E+08	4.00E-01	LSAMP	7	rs219822	98427987	1.21E-01	TRRAP	-	1.13E-07	2.14E-01	2.27E-06	1119	
4	rs10020207	58401219	1.97E-01	N/A	16	rs2938721	61138773	9.26E-01	N/A	-	1.13E-07	3.32E-01	5.51E-04	1120	
8	rs6984354	77447663	3.95E-01	N/A	17	rs1187174	14593162	8.66E-01	N/A	-	1.13E-07	1.71E-01	3.14E-06	1121	
2	rs12328213	78409751	8.91E-01	N/A	9	rs871807	1.16E+08	9.50E-01	ZNF618	-	1.13E-07	4.59E-01	1.66E-04	1122	
6	rs9498131	1.49E+08	4.50E-01	N/A	9	rs7023466	92045628	8.06E-01	N/A	-	1.13E-07	4.84E-01	2.47E-05	1123	
2	rs2264132	2.39E+08	9.80E-01	SCLY	13	rs9524661	94332852	9.91E-01	N/A	-	1.13E-07	6.60E-01	3.86E-06	1124	
1	rs10889978	41634712	1.59E-01	N/A	12	rs4019549	93503803	5.98E-01	TMCC3	-	1.13E-07	8.49E-01	1.12E-04	1125	
2	rs601400	1.66E+08	5.54E-01	N/A	12	rs2359895	5369951	9.03E-02	N/A	-	1.13E-07	6.57E-02	2.94E-03	1126	
8	rs11167147	1.44E+08	3.22E-01	BAI1	9	rs4740990	9795134	7.52E-01	PTPRD	-	1.13E-07	7.96E-01	2.89E-05	1127	
3	rs6772642	1.38E+08	9.15E-01	N/A	7	rs1496770	78096946	2.41E-01	MAGI2	-	1.13E-07	9.36E-01	1.69E-04	1128	
1	rs9287077	1.65E+08	8.02E-01	GPA33	12	rs2669013	75547079	6.30E-01	N/A	-	1.13E-07	N/A	N/A	1129	
7	rs219822	98427987	1.21E-01	TRRAP	12	rs1084375	30507005	6.47E-01	N/A	-	1.13E-07	3.14E-01	4.32E-07	1130	
3	rs285299	36242533	5.64E-01	N/A	16	rs1292361	6951319	7.89E-01	A2BP1	-	1.13E-07	5.18E-01	1.28E-05	1131	
12	rs759391	94984098	5.37E-01	N/A	21	rs2837914	41348235	7.00E-02	N/A	-	1.13E-07	9.15E-01	5.06E-05	1132	
8	rs12546391	22351173	8.12E-01	SLC39A14	12	rs1105956	1.27E+08	6.30E-01	N/A	-	1.13E-07	1.03E-01	2.52E-03	1133	
4	rs1379977	84769783	7.71E-01	N/A	11	rs2047443	80303491	5.66E-01	N/A	-	1.13E-07	8.22E-01	4.70E-06	1134	
1	rs2168924	2.15E+08	8.89E-01	USH2A	17	rs9905659	68208939	3.75E-01	SLC39A11	-	1.13E-07	9.65E-01	7.82E-05	1135	
6	rs7763910	26580634	9.24E-01	BTN3A3	15	rs813299	29225595	8.06E-01	N/A	MHC	1.13E-07	1.31E-01	1.79E-03	1136	
8	rs1049874	17971607	2.79E-01	ASAH1	8	rs1473482	18641042	6.14E-02	PSD3	-	1.14E-07	9.91E-02	2.05E-07	1137	
2	rs6705773	2.21E+08	2.92E-01	N/A	5	rs6596352	1.36E+08	5.11E-01	N/A	-	1.14E-07	7.64E-01	1.33E-04	1138	
2	rs4675812	2.42E+08	7.38E-01	FARP2	15	rs1771000	51119600	4.74E-02	N/A	-	1.14E-07	2.57E-03	1.11E-02	1139	
2	rs11679686	11653488	9.49E-01	GREB1	8	rs1610131	1.35E+08	4.46E-01	N/A	-	1.14E-07	2.50E-01	1.70E-06	1140	
1	rs872171	2.16E+08	7.60E-01	SPATA17	3	rs1488109	1.7E+08	8.17E-01	MECOM	-	1.14E-07	6.38E-01	5.71E-05	1141	
6	rs6900659	1.06E+08	8.04E-01	N/A	2	rs6736615	1.63E+08	7.22E-03	KCNH7	-	1.14E-07	2.62E-01	6.37E-06	1142	
6	rs6916194	1.36E+08	7.45E-01	PDE7B	19	rs1040884	7195884	5.30E-01	INSR	-	1.14E-07	3.21E-01	2.87E-04	1143	
1	rs10857926	1.12E+08	4.25E-01	N/A	19	rs731826	55072670	5.41E-01	AKT1S1	-	1.14E-07	6.45E-01	6.37E-05	1144	
5	rs2269954	1.38E+08	8.02E-01	BRD8	18	rs1893152	2831862	7.81E-02	EMILIN2	-	1.14E-07	9.52E-01	1.46E-04	1145	
6	rs9395787	13552717	7.23E-01	GFOD1	6	rs1219455	1.19E+08	9.44E-01	N/A	-	1.14E-07	2.83E-01	2.54E-06	1146	
1	rs680925	66355709	3.11E-01	PDE4B	1	rs2073484	1.7E+08	3.24E-01	N/A	-	1.14E-07	7.88E-01	1.99E-05	1147	
16	rs2326282	83143633	1.81E-01	COTL1	17	rs232110	61109297	9.29E-01	CCDC46	-	1.14E-07	2.11E-02	1.17E-02	1148	
16	rs2326282	83143633	1.81E-01	COTL1	17	rs232104	61106259	9.29E-01	CCDC46	-	1.14E-07	2.41E-02	1.06E-02	1149	
5	rs307176	6140464	4.26E-01	N/A	5	rs4117784	1.54E+08	8.70E-01	LARP1	-	1.15E-07	4.44E-01	1.07E-04	1150	
6	rs483366	1.62E+08	3.18E-01	PARK2	8	rs4740033	85925158	5.24E-01	RALYL	-	1.15E-07	2.64E-01	3.64E-07	1151	
4	rs1713985	57481207	3.75E-01	REST	11	rs4757841	19724190	8.89E-02	N/AV2	-	1.15E-07	6.71E-01	7.74E-05	1152	
6	rs6911915	1.18E+08	1.66E-01	DCBLD1	7	rs6586746	18325984	2.70E-01	N/A	-	1.15E-07	8.33E-01	1.69E-05	1153	
9	rs10780849	89327729	3.43E-01	DAPK1	12	rs1008283	1.24E+08	4.76E-01	DHX37	-	1.15E-07	7.42E-01	7.66E-05	1154	
13	rs1171080	35385286	5.95E-01	DCLK1	13	rs9557474	1E+08	8.71E-01	TMTC4	-	1.15E-07	2.47E-01	3.58E-06	1155	
5	rs1229724	1.37E+08	3.71E-01	SPOCK1	10	rs973237	63195307	7.50E-01	C10orf107	-	1.16E-07	9.30E-01	2.69E-04	1156	
13	rs4885005	72002057	9.00E-01	N/A	14	rs2180388	1E+08	1.75E-01	N/A	-	1.16E-07	7.96E-02	5.00E-07	1157	
8	rs11785815	59472940	1.94E-01	UBXN2B	20	rs6126251	49597547	2.43E-01	NFATC2	-	1.16E-07	2.05E-01	4.35E-07	1158	
16	rs2326282	83143633	1.81E-01	COTL1	17	rs232150	61131964	8.00E-01	CCDC46	-	1.16E-07	4.09E-02	1.46E-02	1159	
5	rs1506174	40169821	1.82E-01	N/A	17	rs6503080	7944237	6.92E-01	N/A	-	1.16E-07	6.12E-01	6.17E-05	1160	
2	rs7558031	1.93E+08	4.84E-02	N/A	8	rs7159	1.24E+08	9.22E-01	DERL1	-	1.16E-07	2.83E-01	9.69E-07	1161	
2	rs7558031	1.93E+08	4.84E-02	N/A	8	rs7824629	1.24E+08	9.22E-01	DERL1	-	1.16E-07	N/A	N/A	1162	
4	rs4833022	37927687	8.23E-01	N/A	8	rs2163372	1.26E+08	6.16E-01	MTSS1	-	1.16E-07	8.44E-01	3.99E-05	1163	
8	rs2981060	70121659	5.30E-01	N/A	14	rs7160423	99369996	6.39E-01	EML1	-	1.17E-07	5.85E-01	1.12E-04	1164	
8	rs352774	15692048	8.87E-01	N/A	18	rs8093852	68855454	1.39E-01	N/A	-	1.17E-07	6.34E-01	5.58E-06	1165	
3	rs938115	1.18E+08	8.50E-01	N/A	14	rs2877489	22300914	9.64E-01	SLC7A7	-	1.17E-07	3.09E-01	2.14E-06	1166	
5	rs295209	94349360	8.59E-01	MCTP1	9	rs2230806	1.07E+08	6.23E-01	ABCA1	-	1.17E-07	8.12E-01	6.73E-05	1167	
2	rs698811	44557180	8.42E-01	C2orf34	3	rs1093367	1.96E+08	7.25E-01	GP5	-	1.17E-07	7.68E-01	1.42E-04	1168	
5	rs1353103	25036316	4.67E-01	N/A	9	rs4977871	23633682	9.19E-01	N/A	-	1.17E-07	N/A	N/A	1169	
5	rs7711285	25032921	4.67E-01	N/A	9	rs4977871	23633682	9.19E-01	N/A	-	1.17E-07	N/A	N/A	1170	
2	rs13011280	1.39E+08	1.77E-01	N/A	20	rs6139963	6243914	3.24E-01	N/A	-	1.17E-07	8.41E-01	7.56E-06	1171	
6	rs2269646	1.13E+08	3.23E-01	LAMA4	2	rs1299594	16226902	7.82E-01	N/A	-	1.17E-07	2.02E-01	1.19E-04	1172	
3	rs7613069	10600996	5.88E-01	N/A	14	rs2242542	21740815	8.39E-02	N/A	-	1.17E-07	7.60E-01	3.28E-04	1173	
3	rs2622717	1.39E+08	3.26E-01	N/A	8	rs1500906	96601801	1.79E-01	N/A	-	1.17E-07	1.51E-01	2.23E-03	1174	
3	rs2622717	1.39E+08	3.26E-01	N/A	8	rs1500909	96602267	1.79E-01	N/A	-	1.17E-07	1.51E-01	2.23E-03	1175	
10	rs2478425	1.22E+08	9.97E-01	PPAPDC1A	20	rs6106298	20791441	2.43E-01	N/A	-	1.17E-07	5.73E-01	4.69E-05	1176	
6	rs9380215	31157634	4.29E-02	N/A	6	rs3868542	31253818	6.85E-02	TCF19	MHC	1.17E-07	5.03E-02	3.08E-07	1177	
5	rs10071906	1.16E+08	4.18E-01	COMMD10	18	rs7240382	72579628	1.92E-01	N/A	-	1.17E-07	6.71E-01	1.38E-05	1178	
2	rs4669614	10792122	5.50E-02	ATP6V1C2	3	rs2168718	14059158	2.27E-01	TPRXL	-	1.18E-07	2.08E-01	1.16E-04	1179	
7	rs4291161	12535092	2.80E-01	N/A	19	rs2074879	13233589	5.33E-01	CACN/A1A	-	1.18E-07	2.83E-01	4.43E-04	1180	
10	rs4511241	61879261	7.19E-01	N/A	14	rs1013335	25788992	2.88E-01	N/A	-	1.18E-07	6.28E-01	1.97E-04	1181	
9	rs10820441	1.05E+08	2.55E-01	N/A	12	rs4246220	23998069	8.42E-01	SOX5	-	1.18E-07	8.67E-01	3.59E-05	1182	
2	rs13407074	2.35E+08	4.89E-01	ARL4C	8	rs1198643	1.17E+08	3.20E-02	N/A	-	1.18E-07	6.57E-01	1.58E-05	1183	
8	rs7006696	1.19E+08	2.58E-01	EXT1	21	rs989554	38708797	6.64E-01	ERG	-	1.18E-07	9.82E-01	8.65E-05	1184	
5	rs6896019	1.16E+08	4.58E-01	COMMD10	18	rs7240382	72579628	1.92E-01	N/A	-	1.18E-07	6.71E-01	1.37E-05	1185	
6	rs11155954	1.55E+08	4.51E-03	IPCEF1	3	rs9881504	1.97E+08	7.17E-01	N/A	-	1.18E-07	9.06E-01	4.80E-05	1186	
6	rs397139	1.33E+08	4.13E-01	N/A	16	rs1697509	83577116	7.46E-01	ZDHHC7	-	1.19E-07	8.90E-02	2.46E-03	1187	
9	rs7868963	7937001	1.76E-01	N/A	11	rs4754875	96022834	4.42E-01	N/A	-	1.19E-07	9.36E-02	1.45E-03	1188	
6	rs525153	1.33E+08	4.13E-01	N/A	16	rs1697509	83577116	7.46E-01	ZDHHC7	-	1.19E-07	1.06E-01	2.03E-03	1189	
3	rs11711597	1.43E+08	7.57E-01	RASA2	20	rs6131220	11421176	6.47E-01	N/A	-	1.19E-07	7.10E-02	3.32E-07	1190	
1	rs12564233	91637710	9.59E-01	HFM1	2	rs1487036	77843115	6.24E-01	N/A	-	1.19E-07	9.40E-01	1.25E-04	1191	
2	rs7574345	1.8E+08	3.83E-01	ZNF385B	4	rs4864021	1.32E+08	6.81E-02	N/A	-	1.19E-07	N/A	N/A	1192	
3	rs7429462	1.33E+08	6.21E-01	CPNE4	16	rs2098747	69916438	7.10E-01	N/A	-	1.19E-07	1.66E-01	4.17E-07	1193	
2	rs7566839	25524317	5.24E-01	DTNB	8	rs7821595	20925819	4.09E-01	N/A	-	1.19E-07	5.01E-01	1.16E-05	1194	
12	rs7296020	61595594	4.84E-01	PPM1H	13	rs7331302	91492070	6.88E-01	GPC5	-	1.19E-07	7.73E-01	3.66E-05	1195	
12	rs7296020	61595594	4.84E-01	PPM1H	13	rs9523482	91476434	6.88E-01	GPC5	-	1.19E-07	8.00E-01	3.98E-05	1196	
12	rs2239193	1.12E+08	7.82E-02	OAS2	16	rs889592	85076757	1.31E-01	N/A	-	1.19E-07	7.12E-01	1.83E-05	1197	
6	rs1390735	38142442	5.36E-01	ZFAND3	18	rs4075498	47826748	5.02E-02	N/A	-	1.19E-07	4.31E-01	1.26E-06	1198	
4	rs12644174	1.5E+08	2.86E-01	N/A	18	rs650329	42533890	5.68E-02	ST8SIA5	-	1.19E-07	5.05E-01	5.03E-05	1199	
10	rs931028	71043773	3.34E-01	C10orf35	11	rs7928566	21093139	4.17E-01	NELL1	-	1.20E-07	3.06E-01	2.40E-06	1200	
10	rs649364	14487345	9.79E-01	N/A	10	rs1915440	62866140	7.54E-01	TMEM26	-	1.20E-07	2.32E-01	1.06E-06	1201	
7	rs6465375	92727684	1.22E-01	CCDC132	13	rs9541313	67737117	4.18E-01	N/A	-	1.20E-07	1.72E-01	1.10E-06	1202	
11	rs4938222	1.15E+08	2.15E-01	N/A	14	rs7159774	32295737	3.04E-01	AKAP6	-	1.20E-07	5.11E-01	3.51E-05	1203	
3	rs6442019	46696322	2.62E-01	ALS2CL	4	rs4597805	46759253	3.50E-02	GABRB1	-	1.20E-07	6.84E-01	8.91E-05	1204	
2	rs11686460	2.41E+08	6.93E-01	N/A	3	rs9851577	1.27E+08	1.08E-01	ALDH1L1	-	1.20E-07	6.16E-01	1.21E-05	1205	
1	rs3008204	2.12E+08	3.28E-01	N/A	3	rs1702411	87404447	1.06E-02	CHMP2B	-	1.20E-07	7.51E-01	1.30E-05	1206	
2	rs2118836	96526699	8.13E-01	NEURL3	8	rs2517105	17369695	3.36E-01	N/A	-	1.20E-07	5.99E-02	2.83E-03	1207	
6	rs3805994	70823225	2.48E-01	COL19A1	8	rs2294119	1.45E+08	1.95E-02	ZC3H3	-	1.20E-07	9.00E-01	2.02E-05	1208	
6	rs2395471	31348671	7.52E-01	HLA-C	6	rs9266722	31457901	1.13E-01	N/A	MHC	1.20E-07	5.20E-01	4.10E-05	1209	
7	rs2190529	1.1E+08	9.37E-01	IMMP2L	8	rs1687944	32444084	9.18E-01	NRG1	-	1.21E-07	8.10E-01	7.03E-06	1210	
7	rs12672177	1.02E+08	7.98E-02	PRKRIP1	21	rs1304825	34658036	3.39E-01	FAM165B	-	1.21E-07	9.21E-02	2.99E-07	1211	
2	rs797703	47801295	8.31E-01	N/A	3	rs4330287	1.21E+08	4.44E-01	CD80	-	1.21E-07	8.48E-01	1.62E-05	1212	
1	rs474348	98683758	2.10E-01	N/A	9	rs1113863	82170736	1.86E-01	N/A	-	1.21E-07	5.10E-01	1.30E-05	1213	
6	rs6932496	11497723	6.95E-01	NEDD9	1	rs7540340	30138444	8.33E-01	N/A	-	1.21E-07	6.47E-01	7.30E-06	1214	
12	rs10771219	25514113	3.02E-01	IFLTD1	18	rs1944992	55349322	5.60E-01	CCBE1	-	1.21E-07	6.62E-01	3.96E-06	1215	
12	rs10771219	25514113	3.02E-01	IFLTD1	18	rs4566280	55348853	5.60E-01	CCBE1	-	1.21E-07	6.62E-01	3.96E-06	1216	
6	rs9395787	13552717	7.23E-01	GFOD1	6	rs3734381	1.19E+08	7.10E-01	N/A	-	1.21E-07	3.72E-01	5.23E-06	1217	
5	rs2057831	1.38E+08	8.16E-01	CDC23	18	rs1893152	2831862	7.81E-02	EMILIN2	-	1.21E-07	9.52E-01	1.51E-04	1218	
5	rs2864	1.38E+08	8.16E-01	BRD8	18	rs1893152	2831862	7.81E-02	EMILIN2	-	1.21E-07	9.52E-01	1.51E-04	1219	
6	rs6901854	1.01E+08	3.80E-01	N/A	8	rs9643154	1.22E+08	6.50E-01	N/A	-	1.21E-07	7.70E-01	3.17E-05	1220	
1	rs289697	76370797	9.50E-01	ST6GALN/AC3	18	rs1296276	65388622	7.65E-02	DOK6	-	1.21E-07	7.91E-01	1.43E-04	1221	
1	rs1125953	1.96E+08	9.32E-01	N/A	7	rs4265124	1.04E+08	7.94E-01	N/A	-	1.21E-07	5.84E-01	6.83E-06	1222	
3	rs9870986	1.48E+08	5.00E-01	N/A	8	rs1879571	20918847	2.88E-01	N/A	-	1.21E-07	8.77E-01	1.28E-04	1223	
6	rs2770333	1.02E+08	6.19E-01	N/A	14	rs1957011	31962436	2.42E-01	AKAP6	-	1.21E-07	N/A	N/A	1224	
4	rs1532013	1.42E+08	2.65E-01	TBC1D9	16	rs1164030	3932784	8.21E-01	ADCY9	-	1.22E-07	9.61E-01	6.48E-06	1225	
2	rs9679386	30806491	9.60E-01	CAPN13	16	rs1038106	7105351	2.71E-01	A2BP1	-	1.22E-07	1.85E-01	6.04E-07	1226	
1	rs1321623	34079292	3.36E-01	CSMD2	11	rs1122527	1.02E+08	4.08E-01	N/A	-	1.22E-07	2.16E-01	6.49E-04	1227	
2	rs1489635	1.64E+08	9.00E-01	N/A	10	rs2767438	1.34E+08	7.26E-01	INPP5A	-	1.22E-07	9.52E-01	3.07E-05	1228	
6	rs9476962	15967233	9.29E-01	N/A	2	rs1914728	88297195	5.33E-01	N/A	-	1.22E-07	5.04E-01	2.11E-05	1229	
6	rs6937531	1.44E+08	8.76E-01	PLAGL1	3	rs6772225	55251558	1.06E-01	N/A	-	1.22E-07	9.79E-02	1.36E-03	1230	

SNP A					SNP B						Interaction P			Ranking	Cluster in top 100 interactions
CHR	SNP	Location	gle locus P va	Gene	CHR	SNP	Location	ingle locus P valu	Gene	HC region	Stage 1	Stage 2	Combined		
1	rs2336645	1.08E+08	2.56E-01	VAV3	17	s12452093	29114217	5.70E-01	ACCN1	-	1.22E-07	2.60E-01	2.70E-06	1231	
7	rs174930	29835729	8.43E-03	WIPF3	11	rs1012849	1.23E+08	8.08E-01	N/A	-	1.22E-07	5.79E-01	7.66E-06	1232	
18	rs1940659	2019423	3.51E-01	N/A	19	s10424255	51750826	3.01E-01	N/A	-	1.22E-07	6.15E-01	2.03E-04	1233	
9	rs10819907	97938109	8.46E-01	N/A	12	rs1196883	1.04E+08	6.59E-01	KIAA1033	-	1.22E-07	7.91E-01	6.58E-05	1234	
2	rs976904	1.33E+08	1.22E-01	NCKAP5	11	s11215687	1.15E+08	5.15E-01	N/A	-	1.22E-07	9.95E-01	1.12E-05	1235	
1	rs827497	97467030	9.20E-01	DPYD	9	s10966450	24760659	3.09E-02	N/A	-	1.22E-07	1.56E-01	1.12E-03	1236	
5	rs295209	94349360	8.59E-01	MCTP1	9	rs2253174	1.07E+08	6.17E-01	ABCA1	-	1.22E-07	8.12E-01	6.97E-05	1237	
6	rs1199389	8336115	7.69E-01	N/A	17	rs236525	65725420	9.55E-01	N/A	-	1.22E-07	1.32E-01	1.05E-03	1238	
2	rs2241736	47040897	1.38E-01	TTC7A	12	rs7304239	73840326	3.77E-01	KCNC2	-	1.22E-07	4.82E-01	1.27E-06	1239	
6	rs736348	36859223	7.24E-01	CPNE5	5	rs6891999	75585263	8.20E-01	SV2C	MHC	1.22E-07	7.45E-01	1.26E-05	1240	
14	rs4900251	94361899	6.38E-01	N/A	16	rs4129044	6841514	8.11E-01	A2BP1	-	1.23E-07	8.47E-02	1.75E-07	1241	
5	rs17745927	1.7E+08	9.72E-01	KCNIP1	7	rs1109552	1.28E+08	8.96E-01	FAM71F2	-	1.23E-07	2.37E-01	1.89E-06	1242	
5	rs1875972	1.68E+08	1.33E-01	SLIT3	10	s10764706	27874675	3.89E-01	RAB18	-	1.23E-07	4.39E-01	2.70E-06	1243	
8	rs2981060	70121659	5.30E-01	N/A	14	rs3783324	99419770	8.71E-01	EML1	-	1.23E-07	6.01E-01	9.09E-05	1244	
5	rs11960332	1.8E+08	6.87E-01	FLT4	12	s12229875	52458569	1.62E-01	N/A	-	1.23E-07	4.26E-01	1.38E-06	1245	
10	rs755228	76499380	8.99E-01	DUPD1	12	rs1317389	1.02E+08	9.37E-01	C12orf42	-	1.23E-07	7.57E-01	2.40E-05	1246	
6	rs2747690	1.64E+08	7.58E-01	PACRG	12	rs7294540	1769975	3.23E-01	ADIPOR2	-	1.23E-07	8.81E-01	1.75E-05	1247	
6	rs4715607	56264084	2.92E-01	N/A	16	s12148999	12755335	1.10E-01	CPPED1	-	1.23E-07	9.52E-01	1.39E-05	1248	
4	rs1105889	1.54E+08	5.31E-01	FHDC1	14	rs9671696	1.05E+08	3.73E-01	MTA1	-	1.23E-07	3.60E-01	8.56E-04	1249	
14	rs11157994	53521052	6.80E-01	N/A	18	rs7244678	11987197	3.75E-01	IMPA2	-	1.23E-07	5.22E-01	6.92E-06	1250	
11	rs4980704	69501109	3.49E-01	N/A	13	rs7329410	28122208	2.58E-01	POMP	-	1.23E-07	8.67E-01	2.78E-05	1251	
11	rs4980704	69501109	3.49E-01	N/A	13	rs8002632	28120286	2.58E-01	POMP	-	1.23E-07	8.67E-01	2.78E-05	1252	
5	rs4484406	77280768	3.52E-01	N/A	10	s12778872	89346913	6.02E-01	N/A	-	1.23E-07	1.35E-01	1.66E-03	1253	
6	rs9378310	1671161	4.51E-02	GMDS	2	s10193292	13285236	4.63E-01	N/A	-	1.23E-07	4.40E-01	4.18E-04	1254	
6	rs9378310	1671161	4.51E-02	GMDS	2	rs2046266	13284325	4.63E-01	N/A	-	1.23E-07	4.40E-01	4.18E-04	1255	
8	rs626913	61711048	5.84E-01	RAB2A	9	rs7858618	18997561	4.33E-01	FAM154A	-	1.23E-07	N/A	N/A	1256	
1	rs3002303	2.13E+08	8.18E-01	PTPN14	13	s11069721	1.08E+08	5.06E-02	N/A	-	1.24E-07	6.75E-01	1.10E-04	1257	
10	rs10824414	77834518	7.60E-01	C10orf11	11	rs670342	78329325	9.25E-01	ODZ4	-	1.24E-07	N/A	N/A	1258	
3	rs9843440	1.86E+08	2.04E-01	EHHADH	8	rs6993070	1.25E+08	8.97E-01	N/A	-	1.24E-07	1.49E-01	4.88E-04	1259	
3	rs6804571	7111588	6.13E-01	GRM7	12	rs1565728	75952082	6.02E-01	E2F7	-	1.24E-07	8.90E-01	8.26E-06	1260	
1	rs1874930	63279983	3.78E-01	N/A	8	rs1209992	13168685	5.37E-01	DLC1	-	1.24E-07	3.03E-02	3.55E-07	1261	
3	rs2712353	1.15E+08	5.03E-01	ATP6V1A	12	s10841535	20493515	4.33E-01	PDE3A	-	1.24E-07	1.73E-01	2.69E-03	1262	
11	rs7946496	1.26E+08	9.27E-01	KIRREL3	16	rs4785358	48397283	1.14E-01	ZNF423	-	1.24E-07	5.04E-01	1.27E-05	1263	
1	rs3002297	2.13E+08	7.72E-01	PTPN14	13	s12584299	1.08E+08	7.00E-02	N/A	-	1.24E-07	6.30E-01	1.21E-04	1264	
1	rs17115070	96628806	8.62E-01	N/A	5	rs931454	67287006	9.75E-01	N/A	-	1.24E-07	3.20E-01	5.07E-04	1265	
6	rs4707585	90763751	8.82E-01	N/A	1	s12037071	77672382	4.42E-01	AK5	-	1.24E-07	7.46E-02	3.28E-03	1266	
8	rs10957052	59472451	1.72E-01	UBXN2B	20	rs6126251	49597547	2.43E-01	NFATC2	-	1.24E-07	2.04E-01	4.65E-07	1267	
1	rs12409333	65190427	5.81E-01	JAK1	3	rs6795065	1.54E+08	2.79E-01	N/A	-	1.24E-07	3.07E-01	9.23E-06	1268	
1	rs4915675	65200064	5.81E-01	JAK1	3	rs6795065	1.54E+08	2.79E-01	N/A	-	1.24E-07	3.07E-01	9.23E-06	1269	
1	rs7553101	65192778	5.81E-01	JAK1	3	rs6795065	1.54E+08	2.79E-01	N/A	-	1.24E-07	3.07E-01	9.23E-06	1270	
1	rs7546545	65196548	5.81E-01	JAK1	3	rs6795065	1.54E+08	2.79E-01	N/A	-	1.24E-07	3.28E-01	1.03E-05	1271	
6	rs2882694	74574401	9.53E-01	CD109	21	rs1625694	21975417	8.49E-01	N/A	-	1.24E-07	3.85E-01	6.93E-06	1272	
5	rs2089192	1.7E+08	5.56E-01	KCNIP1	11	s11234874	86306884	6.83E-01	N/A	-	1.24E-07	8.47E-01	7.90E-05	1273	
7	rs968141	53769374	4.90E-01	N/A	7	rs2140878	1.23E+08	2.02E-01	IQUB	-	1.24E-07	3.41E-02	1.37E-07	1274	
2	rs11890872	52111013	2.63E-02	N/A	3	s10937160	1.85E+08	6.88E-01	HTR3D	-	1.24E-07	4.25E-01	1.44E-04	1275	
5	rs17745927	1.7E+08	9.72E-01	KCNIP1	11	s11234874	86306884	6.83E-01	N/A	-	1.24E-07	6.28E-01	4.77E-04	1276	
7	rs968141	53769374	4.90E-01	N/A	7	rs7779352	1.23E+08	2.02E-01	IQUB	-	1.24E-07	6.71E-01	2.23E-04	1277	
6	rs2844665	31114834	8.49E-01	HCG22	6	rs9368675	31380136	7.91E-01	N/A	MHC	1.24E-07	6.71E-01	2.23E-04	1278	
6	rs6932930	34785081	2.64E-01	C6orf106	5	rs2338637	72037229	8.11E-02	N/A	MHC	1.25E-07	4.94E-01	1.03E-06	1279	
9	rs7857883	1.26E+08	7.53E-01	N/A	15	rs1372839	96622820	2.25E-01	N/A	-	1.25E-07	7.17E-01	1.80E-05	1280	
13	rs828187	90129301	8.25E-01	N/A	15	rs6576636	24634499	6.05E-01	N/A	-	1.25E-07	2.74E-02	1.31E-06	1281	
9	rs12682705	20508923	9.59E-02	MLLT3	14	s12887478	26929914	9.39E-02	N/A	-	1.25E-07	2.55E-01	7.27E-04	1282	
14	rs910795	22702346	3.98E-01	SLC7A8	22	rs382013	16656101	3.48E-01	BID	-	1.25E-07	8.92E-02	9.26E-04	1283	
1	rs2383513	1.85E+08	7.84E-01	N/A	5	rs7715102	2427305	5.42E-01	N/A	-	1.25E-07	3.30E-01	2.63E-06	1284	
12	rs7970351	5191067	9.81E-01	N/A	17	rs8080666	59840612	8.26E-01	N/A	-	1.25E-07	7.12E-01	2.50E-04	1285	
2	rs6707657	33748322	6.15E-01	N/A	5	rs2591724	33867772	1.12E-01	ADAMTS12	-	1.25E-07	7.58E-01	8.78E-06	1286	
1	rs2996016	41633970	2.03E-01	N/A	12	rs4019549	93503803	5.98E-01	TMCC3	-	1.25E-07	9.95E-01	8.00E-05	1287	
1	rs326910	96624140	9.19E-01	N/A	5	rs931454	67287006	9.75E-01	N/A	-	1.25E-07	3.40E-01	5.49E-04	1288	
5	rs4145160	1.53E+08	6.37E-01	GRIA1	8	rs2673557	1.34E+08	1.26E-01	KCNQ3	-	1.25E-07	6.20E-01	5.61E-05	1289	
1	rs11161944	87427396	7.61E-01	LOC339524	16	rs7359414	302639	5.67E-01	AXIN1	-	1.26E-07	6.60E-02	6.79E-04	1290	
5	rs2968019	58380905	8.06E-01	PDE4D	18	rs9319948	55193376	1.06E-01	LMAN1	-	1.26E-07	4.42E-01	2.29E-06	1291	
6	rs157695	91275194	1.31E-01	MAP3K7	7	rs2344701	1.33E+08	2.79E-01	N/A	-	1.26E-07	5.55E-01	7.62E-05	1292	
1	rs2276401	1.57E+08	7.22E-01	SPTA1	21	s12626903	32046986	7.18E-01	N/A	-	1.26E-07	4.00E-01	2.74E-06	1293	
1	rs10754178	1.94E+08	9.18E-01	N/A	2	rs3771494	70578860	2.49E-01	TGFA	-	1.26E-07	3.99E-01	4.12E-05	1294	
3	rs2366672	1.94E+08	6.44E-02	FGF12	15	rs631864	68163495	4.95E-01	TLE3	-	1.27E-07	N/A	N/A	1295	
2	rs4663126	2.35E+08	4.68E-02	N/A	13	rs7140044	23927295	5.66E-01	PARP4	-	1.27E-07	3.96E-02	1.67E-07	1296	
1	rs556596	28281983	6.43E-02	EYA3	19	rs1870071	16366106	3.56E-01	EPS15L1	-	1.27E-07	6.23E-01	3.41E-05	1297	
5	rs4921132	1.6E+08	8.00E-02	C1QTNF2	7	rs993191	1.42E+08	8.93E-01	TRYX3	-	1.27E-07	9.83E-01	1.92E-05	1298	
5	rs17079868	1.79E+08	1.86E-01	RNF130	11	s11605215	30399824	1.62E-01	MPPED2	-	1.27E-07	6.91E-01	3.04E-05	1299	
6	rs2517552	31115569	8.54E-01	HCG22	6	rs9368675	31380136	7.91E-01	N/A	MHC	1.27E-07	4.65E-01	1.29E-05	1300	
6	rs4710491	62565148	8.98E-01	KHDRBS2	2	rs2421916	63301186	3.38E-01	C2orf86	-	1.28E-07	5.32E-01	3.02E-04	1301	
6	rs9395787	13552717	7.23E-01	GFOD1	6	rs6906287	1.19E+08	8.61E-01	N/A	-	1.28E-07	1.82E-01	1.48E-06	1302	
5	rs4296777	1.06E+08	2.30E-01	N/A	12	rs4763075	61962124	4.02E-01	N/A	-	1.28E-07	5.07E-01	1.89E-05	1303	
2	rs6727188	66879646	5.64E-02	N/A	8	rs2071575	27454349	3.68E-01	EPHX2	-	1.28E-07	6.79E-01	6.34E-05	1304	
5	rs31226	53363328	3.70E-01	ARL15	17	rs820206	71153309	5.26E-01	RECQL5	-	1.28E-07	7.21E-01	1.03E-04	1305	
2	rs1348837	1.34E+08	8.10E-01	N/A	7	rs9792016	13823760	1.01E-02	N/A	-	1.28E-07	8.40E-01	1.39E-05	1306	
1	rs281966	91511350	9.12E-01	HFM1	2	rs1872787	77844332	5.73E-01	N/A	-	1.28E-07	N/A	N/A	1307	
1	rs281968	91507901	9.12E-01	HFM1	2	rs1872787	77844332	5.73E-01	N/A	-	1.28E-07	N/A	N/A	1308	
6	rs11965794	6440030	8.73E-01	LOC285780	9	s11103281	1.38E+08	8.32E-01	CAPN8	-	1.28E-07	N/A	N/A	1309	
5	rs3101875	1.71E+08	6.93E-01	N/A	13	rs4771539	1.06E+08	3.59E-01	N/A	-	1.28E-07	N/A	N/A	1310	
5	rs1030179	1.23E+08	6.89E-01	N/A	10	s16909484	58227959	1.72E-01	N/A	-	1.28E-07	4.12E-01	1.57E-04	1311	
5	rs3822476	77749577	4.95E-01	SCAMP1	11	rs826044	73445145	5.08E-01	C2CD3	-	1.28E-07	7.00E-01	1.61E-05	1312	
2	rs11892554	2.09E+08	2.91E-01	N/A	7	s10273717	20519346	1.20E-01	N/A	-	1.28E-07	4.53E-01	4.48E-04	1313	
4	rs4546199	1.64E+08	9.51E-01	N/A	9	rs3818518	1.37E+08	4.09E-01	N/A	-	1.28E-07	N/A	N/A	1314	
11	rs669775	87444947	1.27E-01	N/A	12	rs1353360	69793828	6.32E-01	TSPAN8	-	1.28E-07	3.48E-01	2.00E-06	1315	
6	rs2395471	31348671	7.52E-01	HLA-C	6	rs7743761	31444079	8.81E-01	HLA-B	MHC	1.28E-07	2.04E-01	1.53E-06	1316	
12	rs4766567	1.1E+08	1.30E-01	CUX2	20	rs1999602	51545423	1.17E-01	N/A	-	1.29E-07	5.52E-01	1.23E-03	1317	
5	rs6862119	27393113	6.08E-02	N/A	18	rs961560	71821808	2.36E-01	N/A	-	1.29E-07	6.81E-01	4.70E-04	1318	
5	rs325339	27388996	6.08E-02	N/A	18	rs961560	71821808	2.36E-01	N/A	-	1.29E-07	N/A	N/A	1319	
7	rs10263457	80822767	5.81E-01	N/A	21	s10854450	21364059	9.77E-01	NCAM2	-	1.29E-07	3.43E-02	1.12E-07	1320	
2	rs4669614	10792122	5.50E-02	ATP6V1C2	3	s12490233	13952366	2.71E-01	TPRXL	-	1.29E-07	1.41E-01	1.62E-04	1321	
4	rs17040984	1.63E+08	9.50E-02	FSTL5	8	rs6586725	18144157	3.06E-01	N/AT1	-	1.29E-07	3.80E-01	1.64E-06	1322	
8	rs352773	15691569	9.77E-01	N/A	18	rs8093852	68855454	1.39E-01	N/A	-	1.29E-07	7.41E-01	8.87E-06	1323	
6	rs9343222	75526636	9.59E-01	N/A	11	rs2919040	96085117	2.21E-01	N/A	-	1.29E-07	9.12E-01	3.92E-05	1324	
10	rs2183164	1.06E+08	2.46E-01	N/A	12	s11169821	50062992	6.53E-01	GALNT6	-	1.29E-07	2.57E-02	9.16E-08	1325	
6	rs6937783	1.06E+08	8.02E-01	N/A	2	rs1435009	1.63E+08	9.35E-03	KCNH7	-	1.29E-07	3.59E-01	8.50E-06	1326	
2	rs11127202	29093564	5.47E-01	FAM179A	7	rs6963746	77612423	4.89E-02	MAGI2	-	1.29E-07	9.85E-01	3.98E-05	1327	
1	rs11203423	17780092	4.50E-01	ARHGEF10L	11	rs2173553	36048464	8.18E-01	LDLRAD3	-	1.29E-07	6.77E-01	5.53E-05	1328	
1	rs4654619	4852515	8.63E-01	N/A	11	s10835810	31679060	7.96E-01	ELP4	-	1.29E-07	8.05E-01	5.01E-05	1329	
2	rs788172	1.73E+08	4.78E-01	MAP1D	7	rs6967514	47862683	2.34E-01	PKD1L1	-	1.30E-07	9.52E-01	2.35E-05	1330	
2	rs4234085	2.09E+08	2.12E-01	N/A	7	rs916790	20539225	9.56E-02	N/A	-	1.30E-07	5.26E-01	1.50E-04	1331	
9	rs522560	77289112	8.31E-01	MIR548H3	13	rs7999126	86769337	9.01E-01	N/A	-	1.30E-07	3.58E-02	3.37E-03	1332	
2	rs1455164	1.38E+08	4.43E-01	HNMT	5	rs1025482	1.67E+08	1.28E-01	ODZ2	-	1.30E-07	7.38E-01	5.06E-05	1333	
17	rs4413004	40462370	1.63E-02	DCAKD	21	s12483287	35027749	8.90E-01	CLIC6	-	1.30E-07	3.66E-01	6.14E-07	1334	
10	rs10906861	15292403	4.94E-02	FAM171A1	13	s12864883	34922993	8.65E-01	NBEA	-	1.30E-07	8.13E-01	1.07E-05	1335	
1	rs9287077	1.65E+08	8.02E-01	GPA33	12	s11114901	75581238	5.64E-01	N/A	-	1.30E-07	3.32E-01	9.93E-05	1336	
9	rs7048902	7266671	5.62E-01	N/A	18	rs1553803	43377597	2.98E-01	N/A	-	1.30E-07	5.21E-01	5.37E-04	1337	
4	rs12374234	1.5E+08	5.59E-01	N/A	18	rs650329	42533890	5.68E-02	ST8SIA5	-	1.30E-07	5.62E-01	4.37E-05	1338	
6	rs833621	44008421	8.94E-01	LOC100132354	8	s11987462	84491475	3.91E-01	N/A	-	1.30E-07	8.24E-01	4.11E-05	1339	
4	rs12641913	63377531	3.55E-01	N/A	12	rs1436124	94807563	6.05E-01	CCDC38	-	1.30E-07	6.04E-02	3.60E-07	1340	
12	rs17834211	14293625	7.78E-03	N/A	18	rs4572512	18204117	5.51E-02	N/A	-	1.30E-07	3.57E-01	3.19E-04	1341	
1	rs10159239	2.46E+08	7.76E-02	NLRP3	7	rs1016019	9945064	8.61E-01	N/A	-	1.30E-07	5.33E-01	2.34E-06	1342	

SNP A					SNP B						Interaction P			Ranking	Cluster in top 100 interactions
CHR	SNP	Location	gle locus P va	Gene	CHR	SNP	Location	ingle locus P valu	Gene	HC region	Stage 1	Stage 2	Combined		
6	rs4707585	90763751	8.82E-01	N/A	1	rs12031867	77672763	5.08E-01	AK5	-	1.30E-07	6.00E-02	4.11E-03	1343	
17	rs4795715	27966241	7.52E-01	MYO1D	18	rs1893489	46449659	8.82E-01	MAPK4	-	1.31E-07	4.92E-01	2.71E-04	1344	
2	rs17031450	66156704	4.16E-01	N/A	2	rs11124220	2.4E+08	7.66E-02	N/A	-	1.31E-07	2.77E-01	3.86E-06	1345	
8	rs2616496	89130414	4.68E-01	MMP16	8	rs7826824	1.37E+08	2.46E-01	N/A	-	1.31E-07	2.83E-01	1.30E-03	1346	
6	rs1296391	6444221	9.77E-01	LOC285780	9	rs11103281	1.38E+08	8.32E-01	CAPN8	-	1.31E-07	N/A	N/A	1347	
6	rs1986394	3724954	8.79E-01	N/A	5	rs1500619	84196609	9.46E-01	N/A	-	1.31E-07	4.81E-01	4.91E-06	1348	
9	rs1853195	14010982	5.75E-01	N/A	15	rs723049	36837208	1.22E-01	N/A	-	1.31E-07	7.63E-01	9.57E-06	1349	
13	rs16968691	1.06E+08	8.10E-01	N/A	19	rs1063803	15633233	4.21E-02	CYP4F3	-	1.31E-07	9.62E-01	1.54E-04	1350	
3	rs17020557	2819653	3.47E-01	CNTN4	12	rs2629751	1.03E+08	4.14E-01	GLT8D2	-	1.31E-07	N/A	N/A	1351	
1	rs6701764	2.13E+08	6.27E-01	SMYD2	13	rs10492664	1.08E+08	6.04E-02	N/A	-	1.32E-07	4.43E-01	2.12E-04	1352	
2	rs4669614	10792122	5.50E-02	ATP6V1C2	3	rs4685064	14060592	2.40E-01	TPRXL	-	1.32E-07	2.22E-01	1.14E-04	1353	
2	rs7592671	1.68E+08	9.06E-01	XIRP2	19	rs889362	18619255	5.85E-02	KLHL26	-	1.32E-07	2.82E-01	2.72E-04	1354	
2	rs1193629	1.76E+08	8.87E-01	CHN1	5	rs6898404	83237939	4.08E-01	N/A	-	1.32E-07	4.83E-01	1.08E-06	1355	
9	rs7049038	1.37E+08	7.82E-01	N/A	16	rs8045887	1994156	4.32E-01	ZNF598	-	1.32E-07	1.06E-01	4.04E-03	1356	
3	rs11714736	20663883	7.95E-01	N/A	7	rs6952125	6336719	8.56E-01	C7orf70	-	1.32E-07	2.41E-01	1.25E-06	1357	
7	rs10241562	92800291	1.77E-01	CCDC132	13	rs9541313	67737117	4.18E-01	N/A	-	1.32E-07	3.05E-01	2.66E-06	1358	
7	rs2106432	92808783	1.77E-01	CCDC132	13	rs9541313	67737117	4.18E-01	N/A	-	1.32E-07	3.05E-01	2.66E-06	1359	
10	rs6480813	77813831	4.04E-01	C10orf11	11	rs670342	78329325	9.25E-01	ODZ4	-	1.32E-07	N/A	N/A	1360	
1	rs3128678	4925039	7.99E-01	N/A	13	rs9547847	36877154	3.09E-01	N/A	-	1.32E-07	7.09E-03	3.10E-02	1361	
3	rs931567	31410581	7.00E-01	N/A	14	rs1953462	43867484	7.56E-01	N/A	-	1.32E-07	4.41E-01	1.14E-04	1362	
6	rs408359	32249861	9.41E-01	PPT2	4	rs13116332	1.49E+08	9.20E-01	NR3C2	MHC	1.33E-07	1.96E-01	3.44E-04	1363	
15	rs12595305	87700628	6.25E-01	MIR9-3	17	rs2292068	7194383	2.91E-01	TMEM95	-	1.33E-07	6.88E-01	2.14E-05	1364	
6	rs6912191	1.44E+08	8.86E-01	SF3B5	22	rs565979	19358946	3.62E-01	POM121L4P	-	1.33E-07	8.15E-01	3.70E-05	1365	
16	rs387138	76525505	2.56E-01	VAT1L	22	rs3827321	22794377	3.22E-01	CABIN1	-	1.33E-07	9.32E-01	5.33E-05	1366	
10	rs10788275	1.24E+08	5.25E-01	BTBD16	13	rs1932745	58831501	8.64E-01	N/A	-	1.33E-07	7.43E-01	3.00E-05	1367	
6	rs9348050	1.66E+08	6.98E-01	N/A	5	rs7730471	1.74E+08	9.43E-01	N/A	-	1.33E-07	8.00E-01	1.67E-04	1368	
6	rs1332849	1.54E+08	3.27E-01	N/A	8	rs1367088	27318112	7.79E-01	PTK2B	-	1.33E-07	3.63E-01	9.54E-06	1369	
15	rs7162279	61474347	8.42E-01	CA12	15	rs7177001	93019031	9.89E-01	N/A	-	1.33E-07	4.47E-01	3.34E-04	1370	
2	rs16984239	18097927	4.25E-01	N/A	16	rs344363	1912549	5.50E-01	HS3ST6	-	1.33E-07	4.99E-01	5.52E-04	1371	
1	rs7881161	1.69E+08	3.19E-02	C1orf129	20	rs13041247	38702488	3.09E-01	N/A	-	1.33E-07	9.91E-01	3.16E-04	1372	
5	rs4130135	84403300	5.94E-01	N/A	13	rs9552462	21119261	3.91E-01	N/A	-	1.33E-07	8.33E-01	2.66E-05	1373	
6	rs879882	31247431	7.61E-01	TCF19	6	rs9266409	31444547	1.40E-01	HLA-B	MHC	1.34E-07	1.92E-01	8.07E-04	1374	
6	rs241424	32912912	2.10E-01	TAP1	15	rs4923770	36137300	4.04E-02	N/A	MHC	1.34E-07	6.10E-01	2.38E-04	1375	
7	rs2392362	35367972	5.04E-02	N/A	8	rs2613646	1.43E+08	9.44E-01	FLJ43860	-	1.34E-07	2.69E-02	9.39E-08	1376	
10	rs10509957	1.14E+08	5.37E-01	TECTB	15	rs11632715	30791539	3.09E-01	GREM1	-	1.34E-07	5.11E-01	1.16E-05	1377	
3	rs729820	1.96E+08	2.73E-01	C3orf21	7	rs976681	24496541	9.07E-01	N/A	-	1.34E-07	2.90E-01	3.83E-07	1378	
6	rs204231	13767705	2.88E-01	RANBP9	3	rs1521293	1.2E+08	8.56E-01	IGSF11	-	1.34E-07	6.36E-01	1.67E-04	1379	
8	rs2981060	70121659	5.30E-01	N/A	14	rs2273704	99453985	7.37E-01	EML1	-	1.34E-07	4.48E-01	2.15E-04	1380	
8	rs2981060	70121659	5.30E-01	N/A	14	rs4905917	99452945	7.37E-01	EML1	-	1.34E-07	N/A	N/A	1381	
6	rs1925220	1.22E+08	3.73E-01	N/A	3	rs7651173	74390042	1.87E-01	CNTN3	-	1.34E-07	2.89E-02	9.26E-08	1382	
5	rs2567818	1.21E+08	9.02E-01	N/A	7	rs10464419	1.54E+08	1.52E-01	DPP6	-	1.34E-07	1.98E-01	4.77E-06	1383	
6	rs4320356	26531539	6.27E-01	BTN3A3	15	rs813299	29225595	8.06E-01	N/A	MHC	1.35E-07	1.30E-01	3.60E-07	1384	
18	rs767887	3643422	4.12E-01	DLGAP1	20	rs1578411	17126434	6.99E-01	N/A	-	1.35E-07	4.09E-01	2.33E-04	1385	
4	rs11730677	14059685	9.31E-01	N/A	8	rs2978010	1.35E+08	8.56E-01	ST3GAL1	-	1.35E-07	6.30E-01	1.05E-05	1386	
3	rs4857185	97394779	1.60E-01	N/A	15	rs4128911	82290193	1.96E-01	ADAMTSL3	-	1.35E-07	5.84E-01	5.48E-05	1387	
2	rs4368280	1.93E+08	2.54E-01	TMEFF2	13	rs9599553	69605722	7.62E-01	ATXN8OS	-	1.35E-07	9.03E-01	7.13E-06	1388	
10	rs10788275	1.24E+08	5.25E-01	BTBD16	13	rs1853920	58840169	9.22E-01	N/A	-	1.35E-07	6.95E-01	2.86E-05	1389	
3	rs9869437	1.98E+08	3.31E-01	RNF168	9	rs7029906	14575052	5.51E-01	N/A	-	1.35E-07	9.86E-03	5.76E-03	1390	
3	rs878809	1.96E+08	2.65E-04	N/A	17	rs3025160	34522004	6.02E-01	PLXDC1	-	1.35E-07	4.86E-01	6.95E-04	1391	
4	rs12640278	1.83E+08	3.29E-01	MGC45800	18	rs8086766	11775174	9.81E-01	GN/AL	-	1.35E-07	5.14E-01	1.86E-04	1392	
10	rs11256915	11004494	7.47E-01	LOC254312	10	rs7084752	97060105	9.11E-01	SORBS1	-	1.35E-07	1.35E-02	6.79E-02	1393	
3	rs10510956	66597327	4.03E-01	LRIG1	18	rs2132918	14586020	1.90E-01	N/A	-	1.36E-07	8.29E-01	7.00E-05	1394	
8	rs12544854	17973091	3.06E-01	N/A	8	rs1473482	18641042	6.14E-02	PSD3	-	1.36E-07	9.91E-02	2.40E-07	1395	
7	rs12701102	31522149	5.92E-01	CCDC129	14	rs721910	1E+08	5.46E-01	MEG3	-	1.36E-07	8.82E-01	1.05E-04	1396	
6	rs1842129	1.25E+08	1.93E-01	NKAIN2	5	rs4866385	33810171	5.37E-01	ADAMTS12	-	1.36E-07	8.91E-01	8.30E-05	1397	
9	rs12351893	74417310	5.19E-02	TMC1	17	rs2323661	15241034	1.56E-01	N/A	-	1.36E-07	7.16E-01	2.11E-04	1398	
5	rs374683	2575431	3.66E-01	N/A	9	rs10758063	31429249	4.81E-02	N/A	-	1.36E-07	8.40E-01	5.77E-06	1399	
18	rs749452	7796676	7.87E-01	PTPRM	21	rs2825324	19343367	9.30E-01	N/A	-	1.36E-07	2.69E-01	3.36E-04	1400	
9	rs1854131	77293735	9.27E-01	MIR548H3	13	rs7999126	86769337	9.01E-01	N/A	-	1.36E-07	2.06E-02	5.43E-03	1401	
2	rs11688000	75146665	2.02E-01	TACR1	5	rs11953463	1.55E+08	5.55E-01	N/A	-	1.36E-07	8.43E-01	2.22E-05	1402	
6	rs11969459	1.48E+08	2.29E-03	N/A	9	rs881660	1.11E+08	4.23E-01	N/A	-	1.36E-07	5.93E-01	5.91E-06	1403	
12	rs11059460	1.27E+08	2.00E-01	N/A	18	rs9946933	41767260	2.82E-01	KIAA1632	-	1.36E-07	5.71E-01	2.82E-04	1404	
12	rs7302874	1.19E+08	6.75E-01	CCDC64	16	rs2340988	23891757	1.22E-01	PRKCB	-	1.36E-07	1.67E-01	5.35E-07	1405	
12	rs7977281	1.19E+08	6.75E-01	CCDC64	16	rs2340988	23891757	1.22E-01	PRKCB	-	1.36E-07	1.67E-01	5.35E-07	1406	
4	rs6820738	1.02E+08	7.05E-01	PPP3CA	14	rs2148692	84870539	7.10E-01	N/A	-	1.36E-07	8.23E-01	8.85E-06	1407	
11	rs10840404	10382705	3.76E-01	N/A	17	rs917538	14975566	6.48E-01	N/A	-	1.37E-07	3.45E-01	5.22E-04	1408	
9	rs2093483	7919372	3.80E-01	N/A	10	rs2981579	1.23E+08	4.98E-01	FGFR2	-	1.37E-07	5.75E-01	4.45E-07	1409	
6	rs6911781	1.14E+08	3.17E-01	N/A	2	rs259833	1.8E+08	8.75E-01	ZNF385B	-	1.37E-07	N/A	N/A	1410	
1	rs16858460	1.65E+08	8.22E-01	DUSP27	12	rs11114901	75581238	5.64E-01	N/A	-	1.37E-07	2.73E-01	1.31E-04	1411	
8	rs7017581	1.21E+08	8.34E-01	ENPP2	9	rs12346753	1.12E+08	3.59E-01	SVEP1	-	1.37E-07	5.82E-01	6.84E-06	1412	
2	rs4668976	15818201	1.72E-01	N/A	10	rs7894289	1.34E+08	9.60E-01	N/A	-	1.37E-07	8.20E-01	3.11E-05	1413	
1	rs876761	91552996	8.98E-01	HFM1	2	rs1487036	77843115	6.24E-01	N/A	-	1.37E-07	9.32E-01	1.32E-04	1414	
11	rs2434484	43905584	8.47E-01	ALKBH3	14	rs1187628	94733739	3.56E-01	CLMN	-	1.37E-07	9.68E-01	3.19E-05	1415	
7	rs7804782	12525324	4.08E-01	N/A	19	rs2074879	13233589	5.33E-01	CACNA1A	-	1.37E-07	4.81E-01	3.21E-04	1416	
5	rs395157	38903489	7.15E-01	OSMR	8	rs2326244	1.2E+08	6.00E-01	N/A	-	1.37E-07	4.73E-02	1.81E-07	1417	
6	rs6938044	1.13E+08	5.21E-01	LAMA4	2	rs12995942	16226902	7.82E-01	N/A	-	1.37E-07	N/A	N/A	1418	
1	rs10874906	95403246	9.20E-01	TMEM56	12	rs4930758	5506838	4.57E-01	N/A	-	1.38E-07	6.45E-01	3.75E-04	1419	
13	rs1325360	32625148	9.71E-01	STARD13	16	rs3892156	48877496	1.96E-01	ADCY7	-	1.38E-07	6.69E-01	1.64E-04	1420	
1	rs3007217	36651819	4.96E-01	LSM10	16	rs982205	27306957	2.20E-01	IL21R	-	1.38E-07	5.81E-01	2.19E-06	1421	
5	rs294953	1.52E+08	8.40E-01	N/A	7	rs10808241	1.29E+08	1.34E-01	N/A	-	1.38E-07	1.24E-01	4.09E-07	1422	
1	rs6670349	2.13E+08	8.24E-01	PTPN14	13	rs10492664	1.08E+08	6.04E-02	N/A	-	1.38E-07	7.35E-01	1.02E-04	1423	
15	rs12594710	50368636	5.51E-01	MYO5C	17	rs4291960	23329968	2.49E-01	N/A	-	1.38E-07	5.43E-01	1.42E-05	1424	
2	rs987987	1.07E+08	5.90E-02	N/A	3	rs566216	1.76E+08	5.95E-01	N/A	-	1.38E-07	7.63E-01	5.92E-05	1425	
9	rs10819907	97938109	8.46E-01	N/A	12	rs2374448	1.04E+08	4.83E-01	APPL2	-	1.38E-07	9.29E-01	3.59E-05	1426	
6	rs7766847	1.69E+08	1.67E-01	N/A	3	rs2399248	1.1E+08	9.05E-01	GUCA1C	-	1.38E-07	6.94E-01	9.09E-05	1427	
6	rs658376	1.38E+08	6.00E-01	N/A	10	rs6583757	92895794	9.69E-01	NUDT9P1	-	1.38E-07	1.78E-01	7.97E-07	1428	
10	rs10788275	1.24E+08	5.25E-01	BTBD16	13	rs4884334	58839005	8.89E-01	N/A	-	1.38E-07	6.95E-01	2.90E-05	1429	
7	rs2058152	13100464	3.88E-02	N/A	19	rs10411733	62482800	4.72E-01	ZNF460	-	1.38E-07	7.97E-01	6.78E-06	1430	
7	rs7784901	13097171	3.88E-02	N/A	19	rs10411733	62482800	4.72E-01	ZNF460	-	1.38E-07	7.97E-01	6.78E-06	1431	
3	rs2712353	1.15E+08	5.03E-01	ATP6V1A	12	rs10841523	20479842	3.36E-01	PDE3A	-	1.38E-07	2.64E-01	5.91E-04	1432	
1	rs2358756	34252509	1.57E-01	CSMD2	17	rs2215006	65561388	7.39E-01	N/A	-	1.38E-07	2.76E-01	1.44E-03	1433	
11	rs952131	1.29E+08	6.41E-01	N/A	19	rs17000660	24166697	9.47E-01	N/A	-	1.38E-07	9.68E-01	8.87E-05	1434	
6	rs2327351	1.34E+08	3.81E-01	EYA4	19	rs11668571	51729460	8.35E-01	N/A	-	1.39E-07	1.26E-01	9.30E-04	1435	
11	rs4753751	95766009	7.08E-01	CCDC82	15	rs2573612	98271513	1.21E-01	N/A	-	1.39E-07	2.09E-01	8.07E-04	1436	
6	rs2327351	1.34E+08	3.81E-01	EYA4	19	rs10417062	51725531	7.39E-01	N/A	-	1.39E-07	2.09E-01	7.31E-04	1437	
6	rs2233984	31187243	4.20E-02	C6orf15	6	rs1062470	31192414	5.89E-01	C6orf15	MHC	1.39E-07	5.83E-01	1.43E-05	1438	
2	rs1921627	77026201	5.37E-01	LRRTM4	5	rs7726783	3187719	3.47E-01	N/A	-	1.39E-07	9.08E-02	5.25E-07	1439	
9	rs765931	7943199	6.61E-02	N/A	11	rs4754875	96022834	4.42E-01	N/A	-	1.39E-07	7.65E-01	2.96E-05	1440	
8	rs6980573	1.26E+08	7.73E-01	N/A	14	rs7154073	90837143	5.09E-01	CCDC88C	-	1.39E-07	8.46E-01	5.58E-05	1441	
7	rs4731401	1.28E+08	3.69E-01	N/A	18	rs11082983	49157825	1.78E-01	DCC	-	1.39E-07	9.86E-01	4.44E-05	1442	
10	rs2862928	1.02E+08	6.79E-01	CPN1	16	rs386031	83130745	4.41E-01	N/A	-	1.39E-07	4.33E-01	5.63E-05	1443	
9	rs2250057	1.35E+08	6.26E-01	TSC1	17	rs227666	52167514	2.34E-01	N/A	-	1.39E-07	8.09E-01	3.07E-05	1444	
2	rs17012409	33042874	1.63E-01	LTBP1	11	rs2237929	17853990	8.97E-01	SERGEF	-	1.39E-07	5.64E-01	2.53E-05	1445	
1	rs11807525	1.89E+08	2.42E-02	N/A	15	rs12440690	93999392	5.36E-01	N/A	-	1.39E-07	7.44E-01	5.58E-05	1446	
1	rs11807892	1.89E+08	2.42E-02	N/A	15	rs12440690	93999392	5.36E-01	N/A	-	1.39E-07	7.44E-01	5.58E-05	1447	
2	rs6546118	65065682	4.25E-01	N/A	2	rs10864912	1.14E+08	9.98E-01	LOC440839	-	1.39E-07	7.79E-01	7.30E-05	1448	
3	rs12106926	1829867	9.51E-01	N/A	18	rs4450488	52868023	8.68E-01	WDR7	-	1.39E-07	N/A	N/A	1449	
3	rs2253685	53094743	1.87E-01	RFT1	17	rs2301775	34594761	8.82E-01	CACNB1	-	1.39E-07	N/A	N/A	1450	
3	rs2336725	53093779	1.87E-01	RFT1	17	rs2301775	34594761	8.82E-01	CACNB1	-	1.39E-07	N/A	N/A	1451	
6	rs9389085	1.34E+08	2.08E-01	EYA4	19	rs1990575	61753859	5.11E-01	ZNF470	-	1.39E-07	4.85E-01	6.26E-05	1452	
9	rs13284995	35832854	2.53E-01	TMEM8B	14	rs977870	22794987	1.31E-01	HOMEZ	-	1.39E-07	N/A	N/A	1453	
1	rs1361812	1.02E+08	4.10E-01	N/A	3	rs4688645	61595936	3.39E-01	PTPRG	-	1.39E-07	9.48E-01	4.90E-05	1454	

SNP A					SNP B						Interaction P			Ranking	Cluster in top 100 interactions
CHR	SNP	Location	gle locus P va	Gene	CHR	SNP	Location	ingle locus P valu	Gene	HC region	Stage 1	Stage 2	Combined		
2	rs3856559	2.16E+08	2.62E-03	N/A	3	s16853481	1.71E+08	2.61E-01	MECOM	-	1.39E-07	5.85E-01	5.76E-05	1455	
2	rs3856558	2.16E+08	2.62E-03	N/A	3	s16853481	1.71E+08	2.61E-01	MECOM	-	1.39E-07	5.85E-01	5.74E-05	1456	
7	rs13228720	37555747	4.44E-01	N/A	12	rs4963932	25952950	1.69E-01	N/A	-	1.40E-07	9.75E-01	4.88E-05	1457	
7	rs10487725	31506482	5.17E-01	CCDC129	14	rs721910	1E+08	5.46E-01	MEG3	-	1.40E-07	8.18E-01	2.73E-04	1458	
8	rs6995779	98544092	5.16E-01	N/A	15	rs7178039	91394651	1.76E-01	RGMA	-	1.40E-07	N/A	N/A	1459	
10	rs731016	71206453	8.01E-01	N/A	22	rs5770953	49409804	9.14E-01	ARSA	-	1.40E-07	N/A	N/A	1460	
4	rs4835617	1.51E+08	1.76E-01	N/A	22	rs2281122	34273930	3.31E-01	RASD2	-	1.40E-07	5.50E-01	2.70E-06	1461	
9	rs4979309	1.16E+08	7.14E-01	ZNF618	12	rs1690918	67560497	6.15E-01	CPM	-	1.40E-07	6.64E-01	1.45E-04	1462	
3	rs2255214	1.23E+08	5.02E-01	CD86	17	s16960503	19709516	1.85E-01	ULK2	-	1.40E-07	6.99E-01	1.79E-04	1463	
8	rs4419794	87298855	1.30E-01	SLC7A13	20	rs1005351	7020555	8.94E-01	N/A	-	1.40E-07	1.64E-01	2.28E-03	1464	
6	rs2636601	1.34E+08	3.18E-01	N/A	13	rs9634445	96921033	8.02E-01	RAP2A	-	1.40E-07	8.97E-01	1.72E-04	1465	
6	rs2677818	1.34E+08	3.18E-01	N/A	13	rs9634445	96921033	8.02E-01	RAP2A	-	1.40E-07	9.10E-01	1.65E-04	1466	
6	rs2677820	1.34E+08	3.18E-01	N/A	13	rs9634445	96921033	8.02E-01	RAP2A	-	1.40E-07	9.10E-01	1.65E-04	1467	
17	rs17247848	28008297	5.07E-01	MYO1D	18	rs8097986	46445557	7.03E-01	MAPK4	-	1.41E-07	2.89E-01	1.85E-04	1468	
6	rs1265110	31227401	8.90E-01	TCF19	6	rs1131896	31487094	3.40E-01	MICA	MHC	1.41E-07	4.81E-01	1.12E-03	1469	
2	rs17039713	60190093	1.70E-02	N/A	12	rs708826	1.15E+08	6.92E-01	N/A	-	1.41E-07	2.90E-01	2.95E-06	1470	
12	rs12828323	82279482	5.14E-01	N/A	16	rs4782589	82760123	8.24E-01	LRRC50	-	1.41E-07	4.81E-01	5.35E-04	1471	
2	rs3106708	1.8E+08	8.73E-01	ZNF385B	3	rs358741	1.57E+08	8.76E-01	C3orf33	-	1.41E-07	6.05E-01	8.71E-05	1472	
11	rs549289	1.17E+08	8.02E-01	DSCAML1	15	rs4775101	57005386	1.71E-01	SLTM	-	1.41E-07	1.59E-01	1.14E-03	1473	
2	rs12613279	1.96E+08	8.37E-02	DN/AH7	9	rs4880117	1.39E+08	1.43E-01	AGPAT2	-	1.41E-07	9.83E-01	4.53E-06	1474	
5	rs9327438	1.27E+08	5.38E-01	MEGF10	7	rs1670338	1.58E+08	1.57E-02	PTPRN2	-	1.41E-07	8.88E-01	2.39E-05	1475	
7	rs174930	29835729	8.43E-03	WIPF3	11	rs3016471	1.23E+08	5.81E-01	N/A	-	1.41E-07	N/A	N/A	1476	
2	rs748519	1.49E+08	7.09E-03	N/A	21	rs2837640	40760614	5.78E-01	DSCAM	-	1.41E-07	1.42E-01	2.41E-04	1477	
18	rs508232	7517485	5.02E-01	N/A	20	rs7264433	17638239	1.45E-01	BANF2	-	1.41E-07	6.41E-01	9.63E-06	1478	
11	rs12223069	1.04E+08	5.73E-01	N/A	16	rs7188834	10628697	5.52E-01	TEKT5	-	1.41E-07	8.93E-01	6.50E-05	1479	
2	rs776	18449496	8.00E-01	N/A	2	rs1608590	76259467	5.61E-01	N/A	-	1.41E-07	9.45E-01	5.06E-05	1480	
7	rs1558052	20277792	1.40E-01	N/A	8	s13256011	6474739	6.29E-01	MCPH1	-	1.42E-07	2.58E-01	3.16E-04	1481	
13	rs1757387	1.03E+08	3.23E-01	N/A	20	rs6131659	15237069	9.69E-01	MACROD2	-	1.42E-07	4.34E-01	6.42E-06	1482	
3	rs6785269	1.72E+08	4.08E-01	TNIK	21	rs7277210	46327739	3.35E-01	COL6A2	-	1.42E-07	N/A	N/A	1483	
2	rs7563917	77066611	7.93E-01	LRRTM4	5	rs597099	3192158	7.14E-01	N/A	-	1.42E-07	1.12E-01	1.53E-03	1484	
7	rs7777166	32362822	5.03E-01	N/A	12	rs918130	22201720	4.53E-01	N/A	-	1.42E-07	2.70E-02	9.72E-08	1485	
1	rs11208380	64429559	8.74E-02	ROR1	15	s12437597	25855901	9.67E-02	OCA2	-	1.42E-07	2.75E-01	1.61E-06	1486	
6	rs879882	31247431	7.61E-01	TCF19	6	rs9266395	31443545	1.35E-01	HLA-B	MHC	1.42E-07	3.78E-02	1.23E-07	1487	
10	rs7477069	54212144	4.45E-01	MBL2	18	rs9646557	50216164	1.53E-01	N/A	-	1.43E-07	1.20E-01	6.13E-07	1488	
2	rs11889825	2.39E+08	4.38E-01	N/A	5	rs305722	84668872	4.05E-01	N/A	-	1.43E-07	8.13E-01	2.61E-05	1489	
7	rs1522545	94187775	5.45E-01	N/A	11	rs1996352	1.02E+08	4.37E-01	MMP7	-	1.43E-07	8.92E-01	1.93E-05	1490	
9	rs7866897	1.31E+08	2.24E-01	CRAT	11	rs1532710	1E+08	8.83E-01	N/A	-	1.43E-07	4.31E-02	1.42E-03	1491	
3	rs2289271	1.17E+08	7.32E-01	LSAMP	9	rs721862	1.27E+08	7.35E-01	NR6A1	-	1.43E-07	3.91E-01	7.10E-04	1492	
9	rs10814822	4040630	8.17E-01	GLIS3	10	rs7082854	1.33E+08	6.90E-01	TCERG1L	-	1.43E-07	2.56E-01	6.75E-04	1493	
10	rs10824274	76538473	2.01E-01	N/A	12	s11111591	1.02E+08	7.56E-01	C12orf42	-	1.43E-07	7.81E-01	1.26E-04	1494	
8	rs6583602	1.43E+08	1.47E-01	TSN/ARE1	17	rs4789374	72423955	4.59E-01	MGAT5B	-	1.43E-07	9.37E-02	1.14E-03	1495	
4	rs12233914	19906678	2.98E-01	SLIT2	14	rs8022093	76146916	7.72E-02	N/A	-	1.43E-07	9.82E-01	4.16E-06	1496	
3	rs11719906	10511005	1.00E-01	ATP2B2	8	rs6993679	37780229	8.46E-01	GPR124	-	1.43E-07	1.30E-01	1.35E-03	1497	
6	rs6932960	1.57E+08	1.59E-01	N/A	2	rs4674511	2.21E+08	7.58E-01	N/A	-	1.43E-07	7.92E-02	1.46E-07	1498	
4	rs2305934	89963265	3.83E-02	FAM13A	5	rs6863774	55730873	1.10E-01	N/A	-	1.43E-07	2.73E-01	2.90E-06	1499	
8	rs7815674	16104297	7.76E-01	MSR1	11	rs7950814	71905497	2.40E-01	N/A	-	1.43E-07	5.13E-01	2.07E-04	1500	
5	rs1826676	62103131	2.86E-02	N/A	9	rs7043121	4204847	8.60E-01	GLIS3	-	1.44E-07	5.61E-01	3.67E-06	1501	
1	rs12134633	2.2E+08	5.92E-01	DUSP10	2	rs1902241	1.26E+08	7.39E-01	N/A	-	1.44E-07	6.67E-01	3.72E-06	1502	
9	rs7858074	1.28E+08	4.77E-02	N/A	11	rs962199	3811341	4.43E-01	RHOG	-	1.44E-07	6.83E-01	1.59E-04	1503	
4	rs4688994	6387028	6.54E-01	PPP2R2C	10	s11254509	17380348	8.39E-01	N/A	-	1.44E-07	7.89E-01	1.25E-05	1504	
1	rs4661089	1.55E+08	2.78E-01	CYCSP52	11	s10892178	1.17E+08	3.31E-01	DSCAML1	-	1.44E-07	2.20E-01	4.27E-06	1505	
6	rs9395787	13552717	7.23E-01	GFOD1	6	s11153748	1.19E+08	9.94E-01	N/A	-	1.44E-07	2.83E-01	3.01E-06	1506	
2	rs4669614	10792122	5.50E-02	ATP6V1C2	3	s11718142	13993794	2.64E-01	TPRXL	-	1.44E-07	2.72E-01	8.57E-05	1507	
8	rs10956218	1.26E+08	4.70E-02	N/A	14	rs1481420	84229253	1.16E-01	N/A	-	1.44E-07	5.16E-01	7.09E-06	1508	
3	rs4530474	1.25E+08	8.94E-01	MYLK	12	rs1293739	1.12E+08	1.91E-01	OAS2	-	1.44E-07	9.74E-01	1.94E-05	1509	
8	rs4872390	25966892	3.89E-01	EBF2	19	rs1275221	56178879	2.12E-01	KLK9	-	1.44E-07	1.18E-01	2.99E-03	1510	
3	rs1523113	1.68E+08	4.29E-01	N/A	15	rs8034215	55521306	7.59E-01	CGNL1	-	1.45E-07	9.06E-01	7.85E-05	1511	
11	rs10767936	32422338	3.72E-01	WT1	17	rs7226259	66592303	4.72E-01	N/A	-	1.45E-07	9.63E-01	4.81E-05	1512	
5	rs13165943	92165388	3.59E-01	N/A	11	s11042978	2154994	3.22E-01	N/A	-	1.45E-07	1.89E-01	5.22E-04	1513	
4	rs1519313	1.87E+08	3.01E-03	N/A	11	s11233078	81706676	6.44E-02	N/A	-	1.45E-07	3.81E-01	7.88E-05	1514	
1	rs578271	65277859	8.36E-01	MIR101-1	10	rs7075837	21066895	6.94E-01	N/A	-	1.45E-07	1.12E-01	3.44E-07	1515	
13	rs4142313	42995658	4.52E-01	ENOX1	14	rs4900550	1.02E+08	2.80E-01	RCOR1	-	1.45E-07	4.82E-01	1.92E-06	1516	
1	rs7545117	1.06E+08	1.69E-01	N/A	2	rs1374274	46908673	9.45E-01	N/A	-	1.45E-07	5.65E-01	3.95E-04	1517	
6	rs6901414	1.3E+08	6.10E-01	L3MBTL3	7	rs1569007	53998890	6.85E-01	N/A	-	1.45E-07	9.32E-01	1.56E-04	1518	
6	rs6901414	1.3E+08	6.10E-01	L3MBTL3	7	rs7791859	53982955	6.85E-01	N/A	-	1.45E-07	9.32E-01	1.56E-04	1519	
6	rs9346979	1.64E+08	5.80E-01	N/A	17	rs4794031	44697152	8.27E-01	N/A	-	1.45E-07	5.34E-01	5.82E-06	1520	
5	rs10055705	65978304	3.62E-01	MAST4	10	s11197219	84837248	9.50E-01	N/A	-	1.45E-07	8.49E-01	1.35E-04	1521	
10	rs12269161	31612679	9.41E-01	N/A	13	rs9567077	42442964	1.36E-01	EPSTI1	-	1.45E-07	N/A	N/A	1522	
3	rs9878757	1.86E+08	1.84E-01	N/A	5	rs891970	1.46E+08	4.35E-01	POU4F3	-	1.46E-07	5.77E-01	1.28E-05	1523	
3	rs9878757	1.86E+08	1.84E-01	N/A	5	rs2277016	1.46E+08	4.35E-01	POU4F3	-	1.46E-07	7.66E-01	6.65E-05	1524	
6	rs7750269	31379136	8.58E-01	N/A	6	rs2844575	31442924	5.47E-01	HLA-B	MHC	1.46E-07	7.66E-01	6.65E-05	1525	
4	rs1379410	1.8E+08	9.15E-02	N/A	5	rs6594771	1.13E+08	7.23E-01	N/A	-	1.46E-07	9.29E-01	2.90E-05	1526	
2	rs4368280	1.93E+08	2.54E-01	TMEFF2	13	rs167255	69606706	7.29E-01	ATXN8OS	-	1.46E-07	4.13E-01	1.00E-05	1527	
10	rs3895536	1.06E+08	1.35E-01	N/A	14	rs7157243	51685256	3.40E-01	N/A	-	1.46E-07	6.47E-01	3.64E-05	1528	
5	rs1435194	1.05E+08	1.04E-01	N/A	12	s10506439	61930601	4.64E-01	N/A	-	1.46E-07	N/A	N/A	1529	
1	rs2276401	1.57E+08	7.22E-01	SPTA1	21	rs7279195	32036597	6.01E-01	SFRS15	-	1.46E-07	2.45E-01	1.11E-06	1530	
3	rs2289271	1.17E+08	7.32E-01	LSAMP	9	s10760374	1.27E+08	7.36E-01	NR6A1	-	1.46E-07	3.36E-01	8.63E-04	1531	
6	rs9344747	88714274	5.64E-01	N/A	10	rs2537626	59345205	2.98E-01	N/A	-	1.46E-07	8.06E-01	7.68E-05	1532	
6	rs9344747	88714274	5.64E-01	N/A	10	rs7913320	59343720	2.98E-01	N/A	-	1.46E-07	8.06E-01	7.68E-05	1533	
6	rs9344747	88714274	5.64E-01	N/A	10	rs2658623	59347689	2.98E-01	N/A	-	1.46E-07	N/A	N/A	1534	
6	rs833623	44006755	8.37E-01	LOC100132354	8	s11987462	84491475	3.91E-01	N/A	-	1.46E-07	4.62E-01	4.53E-04	1535	
11	rs3741337	1.1E+08	8.98E-01	ARHGAP20	21	rs2026220	25436520	7.73E-01	N/A	-	1.47E-07	8.78E-01	5.54E-05	1536	
2	rs10933155	2.27E+08	5.33E-01	RHBDD1	21	rs1888470	38821405	5.39E-01	ERG	-	1.47E-07	1.65E-02	4.77E-08	1537	
4	rs10049820	31957285	4.57E-01	N/A	18	s11873667	3815114	9.20E-01	DLGAP1	-	1.47E-07	3.74E-01	3.66E-04	1538	
7	rs2529243	1.01E+08	3.57E-01	N/A	13	rs9315757	39597795	8.01E-01	N/A	-	1.47E-07	N/A	N/A	1539	
2	rs2339564	22837681	2.48E-01	N/A	2	rs4673218	2.03E+08	6.49E-01	N/A	-	1.47E-07	3.52E-01	6.23E-06	1540	
2	rs10932775	2.19E+08	8.40E-01	PNKD	4	rs3762864	1.4E+08	4.37E-01	ELF2	-	1.47E-07	6.11E-02	4.71E-03	1541	
4	rs2218313	1.43E+08	2.64E-02	N/A	5	s17776554	1.48E+08	5.82E-01	SPINK9	-	1.47E-07	3.52E-01	4.17E-04	1542	
5	rs10037780	26331435	8.69E-02	N/A	9	s12338373	81178142	9.50E-01	N/A	-	1.47E-07	9.74E-01	9.10E-05	1543	
3	rs1154631	16667935	3.44E-01	N/A	8	rs2514730	1.19E+08	2.12E-01	N/A	-	1.47E-07	3.88E-01	1.87E-04	1544	
6	rs7764600	1860827	7.88E-01	GMDS	7	rs1719115	1.26E+08	1.26E-01	N/A	-	1.47E-07	5.64E-01	8.92E-05	1545	
1	rs678640	2.11E+08	6.05E-01	ATF3	1	rs1619856	2.33E+08	3.89E-01	TARBP1	-	1.47E-07	6.07E-01	8.86E-06	1546	
5	rs984509	59330629	3.64E-01	PDE4D	17	rs3785902	65619713	5.26E-02	KCNJ16	-	1.47E-07	6.99E-01	4.12E-05	1547	
6	rs313210	86065675	5.72E-01	N/A	10	rs7477268	5360986	1.33E-01	N/A	-	1.47E-07	8.72E-01	5.64E-05	1548	
10	rs359080	1.32E+08	4.50E-01	N/A	22	rs629429	25067966	2.49E-01	N/A	-	1.47E-07	7.67E-01	7.34E-05	1549	
5	rs4868506	1.75E+08	8.40E-01	N/A	7	s10488668	1.32E+08	3.56E-03	N/A	-	1.48E-07	1.32E-02	2.17E-02	1550	
5	rs4704506	77690583	5.04E-01	SCAMP1	11	rs1685333	73405807	6.89E-01	C2CD3	-	1.48E-07	9.25E-01	3.64E-05	1551	
6	rs2268640	39158362	9.09E-01	GLP1R	11	rs1885667	35976313	7.09E-01	LDLRAD3	-	1.48E-07	9.72E-01	3.70E-06	1552	
2	rs1906901	66250961	2.76E-02	N/A	12	rs273791	59364762	9.95E-01	N/A	-	1.48E-07	4.41E-01	1.37E-04	1553	
16	rs149156	65470207	5.29E-01	PDP2	16	s12598715	79020900	4.17E-01	N/A	-	1.48E-07	3.85E-01	1.13E-06	1554	
1	rs1125953	1.96E+08	9.32E-01	N/A	7	rs194849	1.04E+08	8.54E-01	ORC5L	-	1.48E-07	7.39E-01	1.58E-05	1555	
11	rs1216192	99712710	6.35E-01	CNTN5	17	rs2247989	78229671	1.24E-01	RAB40B	-	1.49E-07	3.10E-02	2.40E-08	1556	
6	rs1410171	85459030	9.09E-01	N/A	1	rs6656361	1.45E+08	7.69E-01	LOC728989	-	1.49E-07	N/A	N/A	1557	
1	rs2276401	1.57E+08	7.22E-01	SPTA1	21	rs2833489	32012629	5.93E-01	SFRS15	-	1.49E-07	2.42E-01	1.10E-06	1558	
1	rs2276401	1.57E+08	7.22E-01	SPTA1	21	rs7282914	32026828	5.93E-01	SFRS15	-	1.49E-07	2.45E-01	1.13E-06	1559	
12	rs2893812	1.19E+08	6.73E-01	CCDC64	16	rs2340988	23891757	1.22E-01	PRKCB	-	1.49E-07	1.67E-01	5.60E-07	1560	
7	rs228582	1.44E+08	1.19E-02	TPK1	21	rs2838906	45646335	6.11E-01	COL18A1	-	1.49E-07	5.39E-01	4.83E-06	1561	
11	rs674485	64953969	4.53E-01	NEAT1	12	rs7980937	77250807	9.01E-01	N/A	-	1.49E-07	7.66E-01	1.43E-05	1562	
7	rs1908268	24125473	5.57E-01	N/A	14	rs1474965	25946860	6.39E-01	N/A	-	1.49E-07	6.53E-01	1.46E-04	1563	
1	rs4129186	2.13E+08	6.85E-01	PTPN14	13	s10492664	1.08E+08	6.04E-02	N/A	-	1.49E-07	5.74E-01	1.04E-05	1564	
2	rs12478812	2.3E+08	4.41E-01	PID1	3	rs6442831	3795517	9.99E-02	N/A	-	1.49E-07	1.28E-01	1.59E-06	1565	
1	rs6667354	2.32E+08	7.67E-01	KCNK1	2	s11688696	40160244	5.01E-01	N/A	-	1.49E-07	4.39E-01	9.95E-06	1566	

SNP A					SNP B						Interaction P			Ranking	Cluster in top 100 interactions
CHR	SNP	Location	gle locus P va	Gene	CHR	SNP	Location	ingle locus P valu	Gene	MHC region	Stage 1	Stage 2	Combined		
2	rs16986953	19805954	3.64E-01	N/A	10	rs2580894	13035894	9.18E-02	CCDC3	-	1.49E-07	8.41E-01	1.60E-05	1567	
2	rs6712787	2.21E+08	4.12E-01	N/A	5	s17702421	1.36E+08	6.28E-01	N/A	-	1.49E-07	8.85E-01	7.97E-05	1568	
2	rs7599725	2.21E+08	4.12E-01	N/A	5	s17702421	1.36E+08	6.28E-01	N/A	-	1.49E-07	8.85E-01	7.97E-05	1569	
1	rs1874113	2.19E+08	5.67E-01	MOSC1	2	rs7564066	7955969	6.95E-01	N/A	-	1.49E-07	3.43E-01	1.53E-05	1570	
19	rs917649	61616573	4.05E-02	ZNF583	21	rs363524	29943260	5.01E-01	GRIK1	-	1.49E-07	8.96E-01	1.30E-05	1571	
1	rs6701764	2.13E+08	6.27E-01	SMYD2	13	s12584299	1.08E+08	7.00E-02	N/A	-	1.50E-07	4.10E-01	2.67E-04	1572	
1	rs1039126	2.32E+08	5.24E-01	KCNK1	16	rs1423087	26416466	9.80E-01	N/A	-	1.50E-07	9.12E-01	3.63E-05	1573	
3	rs1628472	1.18E+08	8.75E-01	N/A	8	rs6988393	1.44E+08	2.30E-01	JRK	-	1.50E-07	3.46E-02	1.37E-03	1574	
6	rs6907109	1.13E+08	3.82E-01	LAMA4	2	s12995942	16226902	7.82E-01	N/A	-	1.50E-07	1.92E-01	1.52E-04	1575	
13	rs1325360	32625148	9.71E-01	STARD13	16	rs3760012	48877392	1.93E-01	ADCY7	-	1.50E-07	6.81E-01	1.34E-04	1576	
7	rs40848	1.08E+08	7.89E-01	PNPLA8	8	s10505223	1.16E+08	5.52E-01	N/A	-	1.50E-07	N/A	N/A	1577	
5	rs162758	16586624	7.25E-01	FAM134B	19	rs4804000	51968294	4.75E-01	SLC1A5	-	1.50E-07	8.67E-01	1.05E-05	1578	
2	rs1918876	1.45E+08	8.78E-01	N/A	13	s10507621	58396330	3.44E-01	N/A	-	1.50E-07	6.60E-01	1.20E-04	1579	
2	rs10490511	80802274	8.48E-02	N/A	4	rs2222427	1.82E+08	8.82E-01	N/A	-	1.50E-07	6.65E-01	2.53E-04	1580	
1	rs6678619	54534938	7.12E-02	SSBP3	8	rs1452806	78251077	6.20E-01	N/A	-	1.50E-07	7.88E-01	2.27E-05	1581	
2	rs4522658	1.34E+08	6.49E-01	N/A	7	rs9785055	8821948	5.87E-01	N/A	-	1.50E-07	6.55E-01	1.83E-04	1582	
11	rs10908195	69140597	5.91E-01	N/A	22	rs2413485	36523866	3.78E-02	H1F0	-	1.50E-07	7.66E-01	9.20E-05	1583	
3	rs9883600	1.46E+08	9.15E-01	N/A	9	rs1771780	1.24E+08	4.65E-01	N/A	-	1.50E-07	2.91E-01	8.89E-07	1584	
4	rs490325	12756720	5.07E-01	N/A	22	rs228947	35856475	7.24E-02	IL2RB	-	1.51E-07	6.78E-02	7.40E-07	1585	
2	rs4605324	29895170	3.52E-01	ALK	4	rs6811343	5132896	1.05E-02	STK32B	-	1.51E-07	1.84E-01	6.48E-07	1586	
1	rs2054269	4409197	1.00E+00	N/A	7	s10262722	29506730	9.03E-01	CHN2	-	1.51E-07	5.94E-01	1.38E-04	1587	
2	rs976904	1.33E+08	1.22E-01	NCKAP5	11	s11215686	1.15E+08	4.70E-01	N/A	-	1.51E-07	7.85E-01	2.59E-05	1588	
1	rs358693	91180775	5.40E-01	ZNF644	11	rs1039038	40468187	6.59E-01	N/A	-	1.51E-07	6.94E-02	2.43E-07	1589	
1	rs2240335	17547124	1.17E-02	PADI4	13	rs481792	71834251	7.43E-01	N/A	-	1.51E-07	5.77E-01	1.45E-04	1590	
2	rs721250	59630520	8.75E-01	N/A	3	rs6439301	1.33E+08	8.79E-01	MRPL3	-	1.51E-07	3.57E-01	1.52E-06	1591	
3	rs6763419	62192561	8.25E-01	PTPRG	10	s10904104	3877689	3.83E-01	N/A	-	1.51E-07	9.08E-01	6.13E-05	1592	
2	rs1470504	77076318	7.97E-01	LRRTM4	5	rs468828	3205858	8.16E-01	N/A	-	1.51E-07	2.26E-01	4.26E-04	1593	
2	rs11892554	2.09E+08	2.91E-01	N/A	7	rs2214244	20527828	1.05E-01	N/A	-	1.51E-07	5.83E-01	2.77E-04	1594	
6	rs1265256	4429854	2.13E-02	N/A	16	s11648589	23203475	4.34E-01	SCNN1B	-	1.51E-07	4.28E-01	1.17E-04	1595	
3	rs7618050	68424104	9.63E-01	FAM19A1	8	rs4872137	23378139	3.00E-02	ENTPD4	-	1.51E-07	9.96E-01	7.09E-05	1596	
11	rs669775	87444947	1.27E-01	N/A	12	rs1798066	69794395	5.84E-01	TSPAN8	-	1.51E-07	3.48E-01	2.30E-06	1597	
1	rs3002297	2.13E+08	7.72E-01	PTPN14	13	s11069721	1.08E+08	5.06E-02	N/A	-	1.51E-07	6.75E-01	1.22E-04	1598	
6	rs833621	44008421	8.94E-01	LOC100132354	8	rs4739941	84490303	4.16E-01	N/A	-	1.51E-07	8.51E-01	4.98E-05	1599	
1	rs12754064	76543227	4.33E-01	ST6GALN/AC3	5	rs2973737	1.78E+08	2.89E-01	COL23A1	-	1.51E-07	6.89E-01	1.65E-05	1600	
6	rs543645	13401089	1.20E-01	PHACTR1	4	rs2062844	29392135	8.91E-03	N/A	-	1.51E-07	1.28E-01	1.19E-03	1601	
8	rs1869831	1.21E+08	5.23E-01	COL14A1	22	s16985531	26491703	8.13E-01	MN1	-	1.51E-07	3.12E-01	2.46E-04	1602	
8	rs2198747	1.21E+08	5.23E-01	COL14A1	22	s16985531	26491703	8.13E-01	MN1	-	1.51E-07	3.21E-01	2.44E-04	1603	
8	rs6469915	1.21E+08	5.23E-01	COL14A1	22	s16985531	26491703	8.13E-01	MN1	-	1.51E-07	3.37E-01	2.21E-04	1604	
7	rs6974676	40974255	6.77E-01	N/A	8	s10108175	88832035	6.38E-01	N/A	-	1.52E-07	4.86E-01	7.43E-06	1605	
6	rs9355736	1.6E+08	5.17E-01	N/A	7	rs7456498	1.03E+08	4.75E-01	N/A	-	1.52E-07	8.66E-01	4.38E-05	1606	
5	rs4538612	33628213	7.02E-01	ADAMTS12	13	rs1329520	40536611	1.54E-01	WBP4	-	1.52E-07	3.93E-01	4.14E-04	1607	
6	rs7747415	1.02E+08	5.76E-01	N/A	14	rs1957011	31962436	2.42E-01	AKAP6	-	1.52E-07	N/A	N/A	1608	
6	rs9498521	1.02E+08	5.76E-01	N/A	14	rs1957011	31962436	2.42E-01	AKAP6	-	1.52E-07	N/A	N/A	1609	
2	rs12993541	41092961	5.50E-01	N/A	2	rs7593053	1.13E+08	2.94E-01	FBLN7	-	1.52E-07	2.01E-02	2.05E-07	1610	
3	rs1284718	1.04E+08	2.05E-01	N/A	13	rs9534981	47653682	6.92E-01	N/A	-	1.52E-07	1.40E-01	3.83E-07	1611	
3	rs1284718	1.04E+08	2.05E-01	N/A	13	rs7998964	47686991	6.92E-01	ITM2B	-	1.52E-07	1.58E-01	4.47E-07	1612	
3	rs1284718	1.04E+08	2.05E-01	N/A	13	rs9534995	47684077	6.92E-01	N/A	-	1.52E-07	1.58E-01	4.47E-07	1613	
3	rs4677353	73981204	5.53E-02	N/A	13	s17058611	60173532	5.84E-01	N/A	-	1.52E-07	4.69E-01	6.34E-04	1614	
2	rs10178695	1.33E+08	1.61E-01	GPR39	11	rs2512568	1.15E+08	5.17E-01	N/A	-	1.52E-07	6.89E-01	2.83E-06	1615	
8	rs7004626	17287631	5.90E-01	MTMR7	15	rs1704405	40031093	7.64E-01	EHD4	-	1.52E-07	N/A	N/A	1616	
5	rs10060475	3854906	4.42E-01	N/A	9	rs4276770	25246654	4.63E-01	N/A	-	1.53E-07	2.15E-01	4.00E-04	1617	
2	rs10496132	66251051	2.35E-02	N/A	12	rs273791	59364762	9.95E-01	N/A	-	1.53E-07	4.35E-01	1.46E-04	1618	
2	rs13383903	66254338	2.35E-02	N/A	12	rs273791	59364762	9.95E-01	N/A	-	1.53E-07	4.35E-01	1.46E-04	1619	
1	rs10926137	2.38E+08	2.16E-01	FMN2	10	rs533480	97222195	6.25E-02	SORBS1	-	1.53E-07	7.22E-01	6.37E-06	1620	
1	rs10926137	2.38E+08	2.16E-01	FMN2	10	rs4917690	97224981	6.25E-02	SORBS1	-	1.53E-07	7.79E-01	7.87E-06	1621	
11	rs612010	63347581	7.70E-01	MARK2	20	rs2297056	44429857	7.67E-01	SLC35C2	-	1.53E-07	3.67E-01	3.23E-06	1622	
11	rs612010	63347581	7.70E-01	MARK2	20	s10211808	44419171	7.67E-01	SLC35C2	-	1.53E-07	4.04E-01	3.88E-06	1623	
1	rs10911898	1.85E+08	8.55E-01	N/A	5	rs7715102	2427305	5.42E-01	N/A	-	1.53E-07	4.19E-01	7.25E-06	1624	
11	rs2472528	5116672	3.86E-01	OR52A1	13	rs2497410	84746002	1.84E-01	N/A	-	1.53E-07	N/A	N/A	1625	
7	rs677442	42979035	2.14E-02	N/A	11	rs475870	58208834	2.51E-02	N/A	-	1.53E-07	9.27E-01	1.23E-04	1626	
4	rs13101823	26784600	1.91E-01	N/A	8	s10503851	29164298	5.91E-01	KIF13B	-	1.53E-07	9.29E-01	5.97E-05	1627	
1	rs12078797	2.18E+08	8.87E-01	N/A	2	rs1519108	66470030	2.88E-01	N/A	-	1.53E-07	2.52E-01	6.69E-07	1628	
2	rs4667014	1.86E+08	5.42E-01	N/A	11	rs2725421	1.32E+08	4.30E-01	OPCML	-	1.53E-07	4.79E-01	1.84E-04	1629	
4	rs4697450	23904932	1.74E-01	N/A	8	rs4269551	25914609	5.44E-01	EBF2	-	1.53E-07	4.48E-01	5.65E-06	1630	
6	rs708039	6432930	3.23E-01	LOC285780	5	rs1353797	95715579	1.51E-01	N/A	-	1.53E-07	2.56E-01	1.58E-03	1631	
1	rs12137718	56975814	7.74E-01	C1orf168	8	rs1460399	3018385	7.11E-01	CSMD1	-	1.54E-07	6.25E-01	2.39E-04	1632	
5	rs1875972	1.68E+08	1.33E-01	SLIT3	10	rs2505331	27816417	5.15E-01	RAB18	-	1.54E-07	7.93E-01	1.16E-05	1633	
6	rs1768997	1.54E+08	6.38E-01	N/A	4	s10937820	7480682	8.93E-01	SORCS2	-	1.54E-07	3.45E-01	2.08E-03	1634	
7	rs17417090	77885009	6.79E-02	MAGI2	19	rs257675	63176021	6.07E-01	ZNF606	-	1.54E-07	4.51E-01	3.63E-05	1635	
2	rs4669614	10792122	5.50E-02	ATP6V1C2	3	rs2607761	14066429	1.95E-01	TPRXL	-	1.54E-07	2.08E-01	1.36E-04	1636	
7	rs599574	70776464	3.50E-01	WBSCR17	10	s11255628	8390349	5.17E-01	N/A	-	1.54E-07	5.37E-01	2.71E-04	1637	
6	rs9402524	1.34E+08	6.11E-01	N/A	13	rs9634445	96921033	8.02E-01	RAP2A	-	1.54E-07	8.90E-01	1.99E-04	1638	
2	rs4234085	2.09E+08	2.12E-01	N/A	7	rs1989886	20549647	1.18E-01	N/A	-	1.54E-07	8.57E-01	7.77E-05	1639	
4	rs2298999	1.11E+08	9.63E-01	N/A	7	rs3778876	38502727	6.95E-01	AMPH	-	1.54E-07	9.76E-01	2.61E-04	1640	
4	rs2298999	1.11E+08	9.63E-01	N/A	7	rs3943706	38501284	6.95E-01	AMPH	-	1.54E-07	9.76E-01	2.61E-04	1641	
2	rs10928295	1.47E+08	5.39E-01	N/A	17	rs7221011	6182156	6.70E-01	N/A	-	1.54E-07	3.10E-01	4.47E-06	1642	
8	rs13274016	15642573	4.45E-01	TUSC3	18	rs8093852	68855454	1.39E-01	N/A	-	1.55E-07	7.26E-01	8.57E-06	1643	
9	rs4336672	1.13E+08	2.11E-01	MUSK	14	rs1957173	45476666	7.46E-01	N/A	-	1.55E-07	6.21E-01	3.22E-04	1644	
3	rs7620240	81845477	9.73E-02	GBE1	10	s12266077	1.24E+08	2.84E-01	C10orf120	-	1.55E-07	8.22E-01	6.57E-05	1645	
7	rs1728648	1.05E+08	3.05E-01	PUS7	19	s10405511	9156591	8.95E-01	OR7D2	-	1.55E-07	8.54E-01	9.37E-05	1646	
6	rs4129277	1.68E+08	3.07E-01	N/A	9	s12352882	6845838	3.67E-01	KDM4C	-	1.55E-07	9.88E-01	2.04E-05	1647	
4	rs11097059	86683004	6.51E-01	ARHGAP24	13	rs883055	28990675	2.11E-01	MTUS2	-	1.55E-07	4.92E-01	3.25E-05	1648	
4	rs17042642	1.12E+08	3.41E-01	N/A	11	rs2291842	5676243	8.66E-02	TRIM5	-	1.55E-07	7.99E-01	2.65E-05	1649	
4	rs6823804	1.12E+08	3.41E-01	N/A	11	rs2291842	5676243	8.66E-02	TRIM5	-	1.55E-07	N/A	N/A	1650	
8	rs2450286	50994092	7.95E-01	SNTG1	20	rs6014949	55186217	1.65E-01	BMP7	-	1.55E-07	1.70E-01	5.22E-04	1651	
2	rs1517915	1.44E+08	7.49E-02	ARHGAP15	5	s13158163	95652626	5.39E-01	N/A	-	1.55E-07	4.79E-01	3.13E-05	1652	
3	rs7637878	1.35E+08	5.18E-02	BFSP2	4	rs4627935	1.42E+08	1.59E-01	RNF150	-	1.55E-07	7.08E-01	1.32E-05	1653	
6	rs2636597	1.34E+08	1.99E-01	N/A	13	rs7322222	96933162	7.88E-01	RAP2A	-	1.56E-07	8.38E-01	7.81E-05	1654	
12	rs11058897	1.26E+08	8.06E-04	N/A	16	s12931881	83436787	4.28E-03	CRISPLD2	-	1.56E-07	9.43E-01	4.87E-05	1655	
12	rs11058900	1.26E+08	8.06E-04	N/A	16	s12931881	83436787	4.28E-03	CRISPLD2	-	1.56E-07	9.93E-01	3.76E-05	1656	
2	rs13000922	18869695	4.13E-01	N/A	5	rs718291	67387836	5.06E-01	N/A	-	1.56E-07	2.37E-01	3.34E-07	1657	
10	rs2163673	90477499	7.09E-01	LIPK	21	rs2837693	40832232	1.95E-01	DSCAM	-	1.56E-07	9.22E-01	7.99E-06	1658	
9	rs1381919	22927343	7.70E-01	N/A	15	rs691	22915888	2.47E-01	SNORD116-27	-	1.56E-07	9.93E-01	8.44E-06	1659	
5	rs11749226	53989021	4.52E-01	N/A	9	s12554805	86282593	2.25E-01	N/A	-	1.56E-07	1.53E-01	4.97E-04	1660	
6	rs1148	1.64E+08	8.69E-01	N/A	3	rs9836895	1611410	9.75E-01	N/A	-	1.56E-07	2.34E-01	1.00E-06	1661	
9	rs4744750	77274328	3.54E-01	N/A	13	rs7999126	86769337	9.01E-01	N/A	-	1.56E-07	9.91E-03	1.06E-02	1662	
1	rs10493673	80887077	7.44E-01	N/A	7	rs9649757	53280204	8.40E-03	N/A	-	1.56E-07	2.61E-01	4.57E-04	1663	
6	rs2636601	1.34E+08	3.18E-01	N/A	13	rs1159278	96883350	9.56E-01	RAP2A	-	1.56E-07	9.54E-01	1.43E-04	1664	
6	rs2677818	1.34E+08	3.18E-01	N/A	13	rs1159278	96883350	9.56E-01	RAP2A	-	1.56E-07	9.68E-01	1.37E-04	1665	
6	rs2677820	1.34E+08	3.18E-01	N/A	13	rs1159278	96883350	9.56E-01	RAP2A	-	1.56E-07	9.68E-01	1.37E-04	1666	
6	rs6926443	17204333	5.36E-01	N/A	6	rs1504377	64005381	7.08E-01	N/A	-	1.56E-07	N/A	N/A	1667	
13	rs421595	1.12E+08	5.06E-01	ATP11A	19	rs3764611	51515856	1.51E-01	HIF3A	-	1.57E-07	2.69E-01	4.63E-04	1668	
1	rs7881161	1.69E+08	3.19E-02	C1orf129	20	s11696257	38704230	3.38E-01	N/A	-	1.57E-07	9.91E-01	3.44E-04	1669	
1	rs7881161	1.69E+08	3.19E-02	C1orf129	20	s17820943	38701930	3.38E-01	N/A	-	1.57E-07	9.91E-01	3.44E-04	1670	
1	rs2795425	2.31E+08	5.94E-01	N/A	8	rs459578	88584509	1.20E-01	N/A	-	1.57E-07	8.17E-02	1.81E-07	1671	
5	rs295209	94349360	8.59E-01	MCTP1	9	rs2472384	1.07E+08	4.64E-01	ABCA1	-	1.57E-07	6.34E-01	1.35E-04	1672	
3	rs6776559	26700125	4.78E-01	LRRC3B	5	rs7380062	1.39E+08	2.47E-01	ECSCR	-	1.57E-07	6.76E-01	3.09E-04	1673	
7	rs6415328	97034600	9.99E-01	N/A	12	rs7313327	43825650	2.87E-01	N/A	-	1.57E-07	9.36E-01	1.08E-04	1674	
2	rs1193629	1.76E+08	8.87E-01	CHN1	5	rs4077503	83249374	5.14E-01	N/A	-	1.57E-07	4.47E-01	1.15E-06	1675	
2	rs1193629	1.76E+08	8.87E-01	CHN1	5	s10805814	83238685	5.14E-01	N/A	-	1.57E-07	4.94E-01	1.37E-06	1676	
2	rs1193629	1.76E+08	8.87E-01	CHN1	5	s13163997	83236774	5.14E-01	N/A	-	1.57E-07	5.76E-01	1.94E-06	1677	
16	rs9928609	7725725	8.97E-02	N/A	17	rs4128646	10022981	5.72E-02	GAS7	-	1.57E-07	6.65E-01	6.51E-06	1678	

SNP A					SNP B						Interaction P			Ranking	Cluster in top 100 interactions
CHR	SNP	Location	gle locus P va	Gene	CHR	SNP	Location	ingle locus P valu	Gene	MHC region	Stage 1	Stage 2	Combined		
2	rs1581035	28539836	2.83E-02	N/A	17	s17636929	14698780	4.93E-01	N/A	-	1.57E-07	4.19E-01	7.15E-04	1679	
9	rs4977617	23593120	3.28E-03	N/A	13	rs2329151	79498340	8.29E-01	N/A	-	1.57E-07	1.04E-01	1.23E-03	1680	
5	rs3822476	77749577	4.95E-01	SCAMP1	11	rs7110734	73530035	3.56E-01	C2CD3	-	1.57E-07	6.99E-01	1.54E-05	1681	
1	rs2479415	55271333	7.21E-01	PCSK9	7	rs528528	1.03E+08	3.46E-01	RELN	-	1.57E-07	7.25E-01	4.06E-05	1682	
5	rs3822476	77749577	4.95E-01	SCAMP1	11	s11235995	73484091	3.56E-01	C2CD3	-	1.57E-07	7.51E-01	1.88E-05	1683	
5	rs3822476	77749577	4.95E-01	SCAMP1	11	rs7125032	73484690	3.56E-01	C2CD3	-	1.57E-07	7.51E-01	1.88E-05	1684	
8	rs2450286	50994092	7.95E-01	SNTG1	20	rs6025422	55183188	3.01E-01	BMP7	-	1.57E-07	5.63E-02	3.93E-03	1685	
1	rs10914802	34117836	7.49E-01	CSMD2	7	rs4731317	1.26E+08	1.01E-01	GRM8	-	1.57E-07	9.10E-01	2.53E-05	1686	
1	rs10914802	34117836	7.49E-01	CSMD2	7	rs1419482	1.26E+08	1.01E-01	GRM8	-	1.57E-07	9.13E-01	2.49E-05	1687	
1	rs10914802	34117836	7.49E-01	CSMD2	7	s12706739	1.26E+08	1.01E-01	GRM8	-	1.57E-07	9.67E-01	2.07E-05	1688	
7	rs11983822	13067583	7.10E-02	N/A	19	s10411733	62482800	4.72E-01	ZNF460	-	1.58E-07	8.96E-01	1.01E-05	1689	
2	rs6741437	28720705	1.36E-02	N/A	5	s12109235	1.01E+08	7.45E-01	N/A	-	1.58E-07	9.36E-01	8.84E-05	1690	
10	rs6482940	1.29E+08	5.87E-01	N/A	16	s10431977	75573781	6.40E-01	N/A	-	1.58E-07	2.82E-02	3.59E-03	1691	
2	rs2916490	80045863	6.83E-01	CTNN/A2	7	rs6977749	1.37E+08	8.67E-01	PTN	-	1.58E-07	9.91E-02	3.73E-03	1692	
11	rs603021	1.28E+08	6.79E-01	FLI1	14	s12436815	62304839	7.60E-01	KCNH5	-	1.58E-07	6.54E-01	1.57E-05	1693	
1	rs10910288	2.32E+08	1.00E-01	N/A	14	rs1037295	42398071	1.61E-01	N/A	-	1.58E-07	9.45E-01	5.17E-05	1694	
5	rs1826676	62103131	2.86E-02	N/A	9	s10758570	4197963	6.55E-01	GLIS3	-	1.58E-07	3.72E-01	2.05E-06	1695	
3	rs4128782	89320422	6.97E-02	EPHA3	7	s10259462	1.3E+08	5.50E-01	FLJ43663	-	1.58E-07	6.60E-01	2.08E-05	1696	
4	rs2110898	12468854	4.23E-01	N/A	10	rs2503872	43113631	8.14E-01	N/A	-	1.58E-07	2.41E-01	2.44E-04	1697	
1	rs6670349	2.13E+08	8.24E-01	PTPN14	13	s12584299	1.08E+08	7.00E-02	N/A	-	1.59E-07	7.02E-01	1.25E-04	1698	
3	rs1881364	10946358	1.30E-01	SLC6A11	5	rs6875376	10684219	4.34E-01	ANKRD33B	-	1.59E-07	4.95E-01	1.67E-04	1699	
3	rs1321635	1.99E+08	5.66E-01	N/A	5	rs4580739	59691437	9.44E-01	PDE4D	-	1.59E-07	3.38E-01	4.40E-04	1700	
4	rs17066390	1.8E+08	7.22E-01	N/A	7	rs2699467	1.44E+08	7.20E-01	N/A	-	1.59E-07	7.54E-02	2.24E-07	1701	
7	rs217164	31528639	5.80E-01	CCDC129	7	rs675242	47556332	3.71E-01	TNS3	-	1.59E-07	1.86E-01	1.69E-03	1702	
12	rs7296020	61595594	4.84E-01	PPM1H	13	rs1819421	91359715	5.45E-01	GPC5	-	1.59E-07	7.70E-01	3.44E-05	1703	
4	rs7656250	23475114	8.58E-01	PPARGC1A	15	s11636570	30044127	6.97E-01	N/A	-	1.59E-07	2.73E-01	4.49E-04	1704	
3	rs7613069	10600996	5.88E-01	N/A	14	rs3811285	21693095	9.36E-02	N/A	-	1.59E-07	6.42E-01	5.88E-04	1705	
8	rs3808403	1.41E+08	2.47E-01	KCNK9	17	rs612720	44273203	4.77E-01	CALCOCO2	-	1.59E-07	8.09E-01	6.20E-05	1706	
2	rs17013592	77088275	7.17E-01	LRRTM4	5	rs1038046	3198652	5.89E-01	N/A	-	1.59E-07	9.86E-01	6.26E-05	1707	
6	rs708039	6432930	3.23E-01	LOC285780	5	s10476546	95578352	1.07E-01	N/A	-	1.60E-07	2.43E-01	1.40E-03	1708	
3	rs1321635	1.99E+08	5.66E-01	N/A	5	rs1423246	59697213	9.45E-01	PDE4D	-	1.60E-07	3.01E-01	5.19E-04	1709	
3	rs570253	1.26E+08	3.23E-01	SLC12A8	3	rs1430408	1.58E+08	2.53E-01	N/A	-	1.60E-07	4.52E-01	3.72E-05	1710	
4	rs2132834	91787002	9.39E-01	FAM190A	12	s10876168	50066903	7.04E-01	GALNT6	-	1.60E-07	6.43E-01	1.51E-04	1711	
9	rs11140522	86125249	9.92E-01	SLC28A3	12	s11170448	51834254	9.91E-01	CSAD	-	1.60E-07	7.91E-01	9.10E-05	1712	
5	rs4700436	56023055	7.81E-01	N/A	5	rs255806	1.19E+08	2.50E-01	N/A	-	1.60E-07	N/A	N/A	1713	
2	rs1193629	1.76E+08	8.87E-01	CHN1	5	rs4274957	83243219	4.41E-01	N/A	-	1.60E-07	4.44E-01	1.10E-06	1714	
1	rs4908382	28156065	2.56E-01	EYA3	3	rs1466941	1.45E+08	9.40E-01	N/A	-	1.60E-07	7.13E-02	1.81E-03	1715	
1	rs714839	1.7E+08	7.35E-01	FMO4	12	rs313327	1.03E+08	4.68E-02	N/A	-	1.60E-07	3.22E-01	2.47E-06	1716	
6	rs7743761	31444079	8.81E-01	HLA-B	1	rs805112	67843363	2.56E-01	N/A	MHC	1.60E-07	2.29E-01	1.51E-03	1717	
12	rs11172506	56921388	2.79E-01	N/A	21	rs2257906	18085674	7.74E-01	C21orf91	-	1.60E-07	2.60E-01	8.18E-07	1718	
5	rs1875972	1.68E+08	1.33E-01	SLIT3	10	rs592818	27808006	5.02E-01	N/A	-	1.61E-07	7.10E-01	8.72E-06	1719	
16	rs2326282	83143633	1.81E-01	COTL1	17	rs232101	61105175	8.81E-01	CCDC46	-	1.61E-07	2.74E-02	1.07E-02	1720	
2	rs10197796	2.12E+08	1.66E-01	N/A	11	rs2199482	42461438	6.61E-01	N/A	-	1.61E-07	2.20E-01	8.39E-07	1721	
14	rs945012	19965486	9.74E-01	TEP1	14	rs718433	21305730	2.94E-01	N/A	-	1.61E-07	2.52E-01	6.65E-07	1722	
8	rs4738873	62248730	8.62E-01	N/A	22	rs5755606	33869717	7.37E-01	N/A	-	1.61E-07	7.90E-01	5.98E-05	1723	
1	rs747658	2.25E+08	4.83E-01	PARP1	16	rs2908668	5013774	7.33E-01	SEC14L5	-	1.61E-07	3.90E-01	1.68E-03	1724	
3	rs2200392	1.57E+08	1.72E-01	N/A	3	rs6798531	1.91E+08	8.74E-01	N/A	-	1.61E-07	9.10E-01	8.10E-05	1725	
7	rs181047	87962850	6.03E-01	N/A	18	rs242685	56914766	3.20E-01	N/A	-	1.61E-07	9.34E-01	2.19E-04	1726	
7	rs181049	87960886	6.03E-01	N/A	18	rs242685	56914766	3.20E-01	N/A	-	1.61E-07	9.83E-01	1.73E-04	1727	
4	rs11944575	1.87E+08	8.49E-01	SNX25	8	rs2439525	97606217	4.01E-01	SDC2	-	1.61E-07	6.38E-01	8.14E-06	1728	
7	rs757359	75205312	3.05E-01	HIP1	8	s10112728	68736913	7.10E-02	CPA6	-	1.61E-07	8.93E-03	1.93E-08	1729	
6	rs9462209	36736020	4.64E-01	CDKN1A	22	rs4361209	37611582	4.88E-01	CBX6	MHC	1.61E-07	7.96E-01	2.75E-05	1730	
6	rs13216342	1.68E+08	3.38E-01	N/A	9	s12352882	6845838	3.67E-01	KDM4C	-	1.61E-07	9.05E-01	1.23E-05	1731	
2	rs1515306	1.8E+08	7.18E-01	ZNF385B	8	s10089145	17281979	2.83E-01	MTMR7	-	1.62E-07	6.92E-01	2.17E-05	1732	
4	rs4956211	1.1E+08	1.47E-02	COL25A1	7	rs2288645	1.5E+08	4.54E-01	ABCB8	-	1.62E-07	7.22E-01	2.45E-05	1733	
16	rs387138	76525505	2.56E-01	VAT1L	22	rs2073396	22781035	6.54E-01	CABIN1	-	1.62E-07	8.58E-01	9.96E-05	1734	
6	rs9402205	1.3E+08	1.46E-01	N/A	14	rs1397163	91173949	1.20E-01	CATSPERB	-	1.62E-07	2.40E-02	1.22E-07	1735	
6	rs1111737	22016072	5.06E-01	FLJ22536	15	rs8034348	47932099	3.18E-01	ATP8B4	-	1.62E-07	5.77E-01	3.24E-04	1736	
2	rs16909	45405034	1.45E-01	N/A	19	rs4926252	13245131	8.82E-01	CACN/A1A	-	1.62E-07	8.87E-01	7.26E-05	1737	
1	rs796486	2.19E+08	6.83E-01	C1orf115	3	rs7628951	56432745	5.26E-01	ERC2	-	1.62E-07	7.73E-01	9.08E-06	1738	
1	rs1497840	73947366	1.58E-01	N/A	10	rs1227079	73122633	3.67E-01	CDH23	-	1.62E-07	2.55E-01	2.17E-03	1739	
1	rs17515948	73948694	1.58E-01	N/A	10	rs1227079	73122633	3.67E-01	CDH23	-	1.62E-07	2.75E-01	2.07E-03	1740	
6	rs9402524	1.34E+08	6.11E-01	N/A	13	rs1159278	96883350	9.56E-01	RAP2A	-	1.62E-07	9.55E-01	1.18E-04	1741	
3	rs4894332	1.4E+08	8.95E-01	FOXL2	3	rs2363961	1.92E+08	5.75E-01	N/A	-	1.63E-07	N/A	N/A	1742	
3	rs4894332	1.4E+08	8.95E-01	FOXL2	3	rs7641416	1.92E+08	5.75E-01	N/A	-	1.63E-07	N/A	N/A	1743	
3	rs2133952	1.58E+08	1.90E-01	N/A	8	s17669929	96377320	5.93E-02	N/A	-	1.63E-07	3.50E-01	5.56E-04	1744	
12	rs12828323	82279482	5.14E-01	N/A	16	rs4782898	82760075	8.88E-01	LRRC50	-	1.63E-07	6.05E-01	1.07E-04	1745	
1	rs12042641	1.65E+08	5.58E-01	GPA33	12	s11114901	75581238	5.64E-01	N/A	-	1.63E-07	3.52E-01	1.04E-04	1746	
1	rs2336645	1.08E+08	2.56E-01	VAV3	17	rs902568	29108642	8.76E-01	ACCN1	-	1.63E-07	6.72E-01	1.11E-05	1747	
2	rs1523300	70622873	3.40E-01	TGFA	20	rs6018564	45641776	4.87E-01	N/A	-	1.63E-07	6.83E-01	1.24E-04	1748	
2	rs531864	1.69E+08	7.48E-01	N/A	8	s10108984	1.24E+08	1.05E-01	ZHX2	-	1.63E-07	9.21E-01	3.10E-05	1749	
6	rs397139	1.33E+08	4.13E-01	N/A	16	rs7192417	83591618	7.39E-01	ZDHHC7	-	1.63E-07	1.01E-01	2.76E-03	1750	
6	rs525153	1.33E+08	4.13E-01	N/A	16	rs7192417	83591618	7.39E-01	ZDHHC7	-	1.63E-07	1.20E-01	2.28E-03	1751	
5	rs1181983	1.28E+08	6.83E-01	SLC27A6	9	rs4740650	1788737	7.67E-01	N/A	-	1.63E-07	9.18E-01	4.18E-05	1752	
4	rs1443207	32981890	5.50E-01	N/A	11	rs2109681	22770039	8.58E-02	GAS2	-	1.63E-07	5.17E-02	3.92E-07	1753	
2	rs2058552	71056585	1.69E-01	ATP6V1B1	3	rs7648874	30561417	8.07E-01	N/A	-	1.63E-07	7.70E-01	2.92E-05	1754	
2	rs4664030	1.62E+08	5.40E-01	N/A	18	rs1788904	55572070	2.92E-01	N/A	-	1.63E-07	6.92E-01	2.62E-04	1755	
7	rs4291161	12535092	2.80E-01	N/A	19	rs735386	13225932	3.41E-01	CACN/A1A	-	1.64E-07	2.49E-01	7.33E-04	1756	
2	rs2176186	2.28E+08	6.36E-01	C2orf83	10	rs787337	29174711	9.90E-01	N/A	-	1.64E-07	5.59E-01	9.45E-04	1757	
12	rs10771219	25514113	3.02E-01	IFLTD1	18	rs1944993	55347999	5.28E-01	CCBE1	-	1.64E-07	4.72E-01	2.07E-06	1758	
4	rs13110363	1.63E+08	2.81E-01	FSTL5	13	rs7328013	1.01E+08	1.86E-01	N/ALCN	-	1.64E-07	6.35E-01	6.81E-06	1759	
2	rs6432436	14131715	1.30E-01	N/A	16	rs9941131	77421314	9.00E-02	WWOX	-	1.64E-07	5.14E-01	3.50E-05	1760	
2	rs6744253	14132136	1.30E-01	N/A	16	rs9941131	77421314	9.00E-02	WWOX	-	1.64E-07	5.14E-01	3.50E-05	1761	
8	rs9987116	1.3E+08	6.52E-01	N/A	19	rs8112582	8148511	4.19E-01	N/A	-	1.64E-07	3.45E-01	3.89E-04	1762	
6	rs4495304	31188697	4.80E-02	C6orf15	6	rs1062470	31192414	5.89E-01	C6orf15	MHC	1.64E-07	9.08E-02	6.11E-07	1763	
8	rs899920	1.36E+08	8.74E-01	N/A	13	rs1340741	68418063	2.94E-01	N/A	-	1.65E-07	3.58E-02	1.85E-07	1764	
3	rs2724706	1.39E+08	3.24E-01	N/A	8	rs1500906	96601801	1.79E-01	N/A	-	1.65E-07	1.88E-01	2.25E-03	1765	
3	rs2724706	1.39E+08	3.24E-01	N/A	8	rs1500909	96602267	1.79E-01	N/A	-	1.65E-07	1.88E-01	2.25E-03	1766	
3	rs881753	1.97E+08	4.44E-01	TNK2	10	rs1806437	49468469	2.62E-01	ARHGAP22	-	1.65E-07	4.74E-01	5.01E-04	1767	
7	rs10250954	22441474	5.74E-01	MGC87042	16	rs4785755	88565329	3.88E-01	AFG3L1	-	1.65E-07	9.29E-01	2.89E-05	1768	
18	rs1489169	44129658	2.17E-02	N/A	22	rs361893	16974931	3.34E-01	TUBA8	-	1.65E-07	5.61E-02	1.52E-06	1769	
1	rs746503	54842574	3.17E-01	ACOT11	7	rs2732757	84648146	3.36E-01	N/A	-	1.65E-07	1.05E-01	2.64E-07	1770	
1	rs4641275	1.81E+08	6.05E-01	C1orf14	3	rs184452	64125246	5.83E-01	PRICKLE2	-	1.65E-07	8.62E-01	4.80E-04	1771	
11	rs10791048	1.29E+08	2.10E-01	N/A	18	rs280985	4441032	8.03E-01	N/A	-	1.65E-07	4.60E-01	7.82E-04	1772	
10	rs1515415	79106217	4.46E-01	N/A	13	rs7985826	1.02E+08	2.18E-01	BIVM	-	1.65E-07	9.72E-01	9.33E-05	1773	
7	rs2059407	53620102	4.01E-01	N/A	17	rs7207017	50834065	6.37E-01	MMD	-	1.65E-07	9.07E-01	1.61E-04	1774	
3	rs13068994	4769128	1.33E-01	ITPR1	10	rs1933961	86771947	7.76E-01	N/A	-	1.66E-07	9.18E-01	2.76E-05	1775	
1	rs12078845	54375565	3.80E-01	CDCP2	18	s16978310	41077512	9.65E-01	N/A	-	1.66E-07	2.59E-01	1.96E-05	1776	
10	rs11189926	1.01E+08	8.77E-01	HPSE2	12	rs979553	25104849	6.05E-02	LRMP	-	1.66E-07	2.69E-01	3.77E-06	1777	
1	rs2094026	1.94E+08	8.67E-01	N/A	2	rs3771494	70578860	2.49E-01	TGFA	-	1.66E-07	3.58E-01	6.02E-05	1778	
6	rs1925227	1.02E+08	3.61E-01	GRIK2	5	s16868534	34893202	1.12E-02	TTC23L	-	1.66E-07	9.46E-01	1.18E-05	1779	
1	rs4658018	1.94E+08	8.67E-01	N/A	2	rs3771494	70578860	2.49E-01	TGFA	-	1.66E-07	N/A	N/A	1780	
3	rs6440172	1.45E+08	5.76E-01	SLC9A9	10	rs6585740	1.23E+08	2.89E-01	FGFR2	-	1.66E-07	1.74E-01	1.91E-06	1781	
3	rs9825812	1.28E+08	8.33E-01	N/A	10	rs972570	70942024	1.83E-01	TSPAN15	-	1.66E-07	9.01E-01	2.15E-04	1782	
11	rs11025056	19186741	7.22E-01	CSRP3	16	s12930687	9528370	8.01E-04	N/A	-	1.66E-07	8.35E-01	4.55E-05	1783	
8	rs10089434	3210534	1.83E-01	CSMD1	12	s11180455	73981709	7.99E-01	CAPS2	-	1.66E-07	8.70E-01	8.67E-06	1784	
4	rs872883	6515448	9.72E-01	PPP2R2C	18	rs649361	30997514	8.74E-03	N/A	-	1.66E-07	9.63E-01	7.03E-05	1785	
7	rs2690897	1.01E+08	3.73E-01	N/A	13	rs9315757	39597795	8.01E-01	N/A	-	1.66E-07	6.64E-01	3.83E-04	1786	
6	rs674327	1.53E+08	5.82E-01	N/A	20	rs6055075	7445194	9.55E-01	N/A	-	1.66E-07	1.50E-01	2.37E-07	1787	
11	rs10898133	82910180	2.08E-01	DLG2	13	rs9532449	39369698	1.50E-01	N/A	-	1.67E-07	4.90E-01	2.76E-06	1788	
17	rs9891027	32617791	4.47E-01	ACACA	17	rs4789106	69034532	1.94E-01	SDK2	-	1.67E-07	1.55E-01	2.75E-06	1789	
9	rs2997657	71852509	4.53E-01	MAMDC2	12	rs7962536	61590288	4.55E-01	PPM1H	-	1.67E-07	5.43E-01	5.04E-04	1790	

SNP A					SNP B						Interaction P			Ranking	Cluster in top 100 interactions
CHR	SNP	Location	gle locus P va	Gene	CHR	SNP	Location	Single locus P value	Gene	MHC region	Stage 1	Stage 2	Combined		
4	rs17725302	1.78E+08	6.09E-01	N/A	5	rs1582416	1.43E+08	7.86E-01	N/A	-	1.67E-07	1.44E-01	4.17E-07	1791	
3	rs899983	1.39E+08	3.57E-01	N/A	8	rs1500906	96601801	1.79E-01	N/A	-	1.67E-07	1.86E-01	2.26E-03	1792	
3	rs899983	1.39E+08	3.57E-01	N/A	8	rs1500909	96602267	1.79E-01	N/A	-	1.67E-07	1.86E-01	2.26E-03	1793	
8	rs10092844	72375844	9.01E-01	EYA1	15	rs9920560	58835222	5.46E-01	RORA	-	1.67E-07	4.41E-01	4.77E-06	1794	
5	rs6877153	1.22E+08	8.47E-01	SNCAIP	11	rs11037217	43006402	8.33E-01	N/A	-	1.67E-07	8.84E-01	2.29E-05	1795	
11	rs1124847	56712658	3.71E-01	LRRC55	16	rs1345872	72058301	6.68E-01	N/A	-	1.67E-07	5.71E-01	6.98E-04	1796	
4	rs1377347	41067359	3.26E-01	LIMCH1	19	rs16979595	50169221	7.74E-01	CLPTM1	-	1.67E-07	2.10E-01	7.67E-04	1797	
6	rs3130473	31307187	2.29E-01	N/A	6	rs7743761	31444079	8.81E-01	HLA-B	MHC	1.67E-07	8.02E-01	4.17E-05	1798	
3	rs1870709	1.17E+08	3.37E-01	LSAMP	8	rs6984840	10169975	9.13E-01	MSRA	-	1.68E-07	5.71E-01	1.57E-04	1799	
1	rs11203366	17530121	9.93E-03	PADI4	13	rs481792	71834251	7.43E-01	N/A	-	1.68E-07	5.97E-01	1.42E-04	1800	
7	rs13239224	35360576	2.86E-02	N/A	8	rs2613646	1.43E+08	9.44E-01	FLJ43860	-	1.68E-07	6.10E-01	2.80E-04	1801	
9	rs7029452	74397149	3.15E-02	TMC1	17	rs2323661	15241034	1.56E-01	N/A	-	1.68E-07	3.87E-01	1.64E-03	1802	
1	rs7550122	1.06E+08	1.03E-01	N/A	10	rs10826790	30683878	9.60E-01	LOC729668	-	1.68E-07	8.27E-01	5.86E-05	1803	
4	rs1550057	1.42E+08	8.62E-01	N/A	16	rs11642883	81629115	1.95E-01	CDH13	-	1.68E-07	1.32E-01	9.03E-07	1804	
6	rs9384086	1.54E+08	3.79E-01	N/A	10	rs1914191	64032229	6.47E-01	ZNF365	-	1.68E-07	4.21E-01	1.74E-04	1805	
1	rs7417097	8916454	6.73E-01	CA6	8	rs2949554	88121048	6.90E-01	CNBD1	-	1.68E-07	6.67E-01	3.59E-05	1806	
2	rs4605324	29895170	3.52E-01	ALK	4	rs1447284	5138454	9.87E-03	STK32B	-	1.68E-07	3.34E-01	2.90E-06	1807	
3	rs9872768	1.48E+08	1.86E-01	N/A	8	rs1879571	20918847	2.88E-01	N/A	-	1.68E-07	2.66E-01	1.52E-06	1808	
1	rs710139	10767145	5.13E-01	CASZ1	5	rs10512948	8286351	8.91E-02	N/A	-	1.68E-07	8.92E-01	7.92E-05	1809	
6	rs9295957	31265572	2.18E-01	POU5F1	6	rs396243	31383153	2.17E-01	N/A	MHC	1.68E-07	8.20E-01	2.48E-05	1810	
6	rs9271568	32698441	6.10E-01	HLA-DQA1	19	rs6512102	16315110	8.67E-01	EPS15L1	MHC	1.69E-07	3.76E-01	7.33E-04	1811	
2	rs13409846	2.19E+08	2.63E-01	PNKD	4	rs2292837	1.4E+08	2.54E-01	ELF2	-	1.69E-07	4.24E-01	2.43E-04	1812	
2	rs2627857	66155597	9.35E-01	N/A	8	rs2166646	1.05E+08	1.59E-01	N/A	-	1.69E-07	6.94E-01	5.23E-05	1813	
5	rs6452143	23756112	3.87E-01	N/A	10	rs4748247	16167564	5.20E-01	N/A	-	1.69E-07	N/A	N/A	1814	
10	rs2804503	33694722	1.37E-01	N/A	18	rs1367865	48215053	8.55E-01	DCC	-	1.69E-07	5.90E-01	1.29E-04	1815	
5	rs988364	59247667	2.68E-03	PDE4D	7	rs12670356	81840844	5.95E-01	CACN/A2D1	-	1.69E-07	1.78E-01	5.13E-04	1816	
9	rs10967200	26093057	9.42E-01	N/A	9	rs10869977	79647096	1.83E-01	GN/AQ	-	1.69E-07	2.55E-01	2.39E-06	1817	
6	rs6922684	1.5E+08	8.87E-01	PPP1R14C	9	rs10780770	88373314	7.97E-01	N/A	-	1.69E-07	2.22E-01	8.82E-04	1818	
5	rs346660	1.36E+08	2.88E-01	N/A	17	rs1358175	36011315	8.03E-01	N/A	-	1.69E-07	8.75E-01	1.21E-05	1819	
1	rs535988	1.63E+08	6.38E-01	N/A	8	rs4876153	2291741	2.11E-01	N/A	-	1.69E-07	N/A	N/A	1820	
9	rs7025302	91943342	9.76E-01	N/A	19	rs10408252	39694615	4.34E-02	WTIP	-	1.70E-07	6.65E-02	1.28E-03	1821	
4	rs749493	1.75E+08	8.95E-01	N/A	12	rs10505892	23569819	9.11E-01	SOX5	-	1.70E-07	8.83E-01	9.41E-05	1822	
7	rs10256077	2137976	4.92E-01	MAD1L1	13	rs4436648	22118204	4.36E-01	N/A	-	1.70E-07	3.64E-01	3.84E-04	1823	
3	rs1559018	1.92E+08	9.42E-01	IL1RAP	8	rs4871724	1.28E+08	8.85E-03	N/A	-	1.70E-07	7.31E-01	1.77E-05	1824	
1	rs11203368	17539095	1.03E-02	PADI4	13	rs481792	71834251	7.43E-01	N/A	-	1.70E-07	5.60E-01	1.62E-04	1825	
4	rs1519313	1.87E+08	3.01E-03	N/A	11	rs7928596	81675813	7.26E-02	N/A	-	1.70E-07	3.06E-01	1.28E-04	1826	
1	rs1501228	60680048	7.26E-02	N/A	9	rs1810887	1.34E+08	6.35E-01	NTNG2	-	1.70E-07	6.71E-01	7.03E-06	1827	
5	rs1156684	1.21E+08	7.86E-01	N/A	7	rs10464419	1.54E+08	1.52E-01	DPP6	-	1.70E-07	1.81E-01	5.05E-06	1828	
5	rs10043664	54464067	6.89E-01	CDC20B	9	rs1509186	1085922	8.28E-01	N/A	-	1.70E-07	2.67E-01	1.18E-03	1829	
5	rs4703197	1.01E+08	9.53E-01	N/A	12	rs529446	5091580	5.54E-01	N/A	-	1.70E-07	1.41E-01	4.50E-07	1830	
1	rs556596	28281983	6.43E-02	EYA3	19	rs11878602	16416153	3.89E-01	EPS15L1	-	1.70E-07	8.65E-01	1.19E-05	1831	
2	rs10169420	1.83E+08	4.31E-01	N/A	12	rs1603572	82627746	9.51E-01	N/A	-	1.71E-07	1.57E-01	1.75E-06	1832	
3	rs6791296	62406455	7.93E-01	CADPS	20	rs6126303	49740539	1.32E-01	ATP9A	-	1.71E-07	1.44E-01	2.42E-04	1833	
1	rs12023371	1.57E+08	8.45E-01	SPTA1	15	rs415799	56478046	6.34E-01	N/A	-	1.71E-07	2.26E-01	6.76E-06	1834	
8	rs7829982	23262643	9.83E-01	LOXL2	12	rs10778080	99760460	8.30E-01	ANO4	-	1.71E-07	2.35E-01	1.09E-06	1835	
11	rs10891914	1.15E+08	8.34E-01	N/A	13	rs12585823	45434292	1.83E-01	ZC3H13	-	1.71E-07	5.91E-01	3.07E-04	1836	
3	rs563275	1.89E+08	9.63E-01	N/A	20	rs2206641	19445646	2.63E-01	SLC24A3	-	1.71E-07	6.28E-02	2.31E-03	1837	
13	rs1171080	35385286	5.95E-01	DCLK1	13	rs9518128	1E+08	8.21E-01	TMTC4	-	1.71E-07	1.52E-01	2.14E-06	1838	
2	rs13415138	46935055	2.36E-01	LOC100134259	3	rs4685976	5716702	7.91E-01	N/A	-	1.71E-07	8.79E-01	3.00E-05	1839	
4	rs7686686	1.11E+08	3.40E-01	N/A	5	rs13169504	1.62E+08	3.04E-01	N/A	-	1.71E-07	3.76E-01	4.50E-06	1840	
6	rs1750603	94958120	1.09E-02	N/A	10	rs2565190	42850077	8.76E-01	N/A	-	1.71E-07	3.38E-01	1.41E-06	1841	
2	rs532290	1.35E+08	6.84E-01	TMEM163	10	rs6602096	16405073	8.34E-01	N/A	-	1.71E-07	5.98E-01	1.08E-04	1842	
5	rs6451438	39673322	1.26E-01	N/A	9	rs10971632	33655229	6.52E-01	PTENP1	-	1.71E-07	7.09E-02	1.70E-03	1843	
10	rs2025468	10656491	3.55E-01	N/A	19	rs384842	52070865	3.46E-01	N/A	-	1.71E-07	6.14E-01	1.34E-04	1844	
5	rs1875972	1.68E+08	1.33E-01	SLIT3	10	rs2505300	27830113	3.98E-01	RAB18	-	1.71E-07	7.25E-01	1.15E-05	1845	
9	rs4130590	1.29E+08	9.17E-01	GARNL3	10	rs11255243	7701244	2.59E-01	ITIH5	-	1.71E-07	9.56E-01	3.33E-05	1846	
9	rs4130590	1.29E+08	9.17E-01	GARNL3	10	rs1970183	7701459	2.59E-01	ITIH5	-	1.71E-07	9.74E-01	3.20E-05	1847	
11	rs10831769	12201044	6.12E-01	MICAL2	18	rs2726252	35434744	5.29E-03	LOC647946	-	1.71E-07	9.48E-01	4.91E-06	1848	
6	rs6907357	96218093	7.07E-01	N/A	22	rs9617490	47445992	4.81E-03	FAM19A5	-	1.72E-07	6.38E-03	1.17E-08	1849	
5	rs12523655	1.7E+08	5.99E-01	KCNIP1	7	rs6976331	1.28E+08	4.24E-01	N/A	-	1.72E-07	4.55E-01	8.90E-06	1850	
1	rs1808806	1.18E+08	8.04E-01	FAM46C	2	rs1405819	57712498	4.28E-01	N/A	-	1.72E-07	5.66E-01	1.40E-04	1851	
7	rs1002029	1.55E+08	7.90E-01	CNPY1	18	rs1791350	55596838	3.80E-01	N/A	-	1.72E-07	2.26E-01	8.87E-04	1852	
17	rs7209936	78519504	6.65E-01	B3GNTL1	18	rs8088164	53979610	6.35E-02	NEDD4L	-	1.72E-07	6.49E-01	5.05E-06	1853	
7	rs6974363	47599712	6.43E-01	N/A	19	rs11880383	17231190	4.56E-01	USHBP1	-	1.72E-07	8.03E-01	1.03E-04	1854	
3	rs6419833	37595485	4.95E-01	ITGA9	8	rs7009980	4488686	4.40E-01	CSMD1	-	1.72E-07	8.32E-01	1.50E-04	1855	
15	rs10152718	36270856	9.11E-01	N/A	16	rs3764276	29580704	3.61E-01	QPRT	-	1.72E-07	9.35E-01	7.65E-05	1856	
4	rs4695718	44107694	8.51E-01	KCTD8	12	rs10858403	75142247	1.81E-03	N/A	-	1.72E-07	3.56E-01	3.53E-04	1857	
1	rs10493673	80887077	7.44E-01	N/A	7	rs10276450	53275750	7.85E-03	N/A	-	1.72E-07	2.34E-01	5.72E-04	1858	
2	rs896637	2.09E+08	5.59E-01	N/A	7	rs1989886	20549647	1.18E-01	N/A	-	1.72E-07	5.87E-01	2.85E-04	1859	
2	rs2203163	2360793	2.53E-01	N/A	9	rs10817758	1.17E+08	9.89E-01	1-Dec	-	1.72E-07	5.84E-01	3.39E-05	1860	
6	rs4896668	1.44E+08	4.96E-01	PHACTR2	14	rs4982398	20599037	5.69E-01	RN/ASE7	-	1.73E-07	1.14E-01	4.17E-03	1861	
4	rs4697648	10307363	6.42E-01	CLNK	7	rs1017045	1.05E+08	7.20E-01	N/A	-	1.73E-07	1.54E-01	2.86E-03	1862	
1	rs11807525	1.89E+08	2.42E-02	N/A	15	rs8033029	94018392	7.53E-01	N/A	-	1.73E-07	7.15E-01	5.84E-05	1863	
1	rs11807892	1.89E+08	2.42E-02	N/A	15	rs8033029	94018392	7.53E-01	N/A	-	1.73E-07	7.15E-01	5.84E-05	1864	
1	rs4846904	2.28E+08	6.90E-01	GALNT2	9	rs2055772	15592101	2.20E-01	C9orf93	-	1.73E-07	1.95E-01	4.25E-03	1865	
7	rs11983822	13067583	7.10E-02	N/A	19	rs4801457	62485375	9.79E-01	ZNF460	-	1.73E-07	7.26E-01	5.92E-06	1866	
1	rs3933012	94968020	1.77E-01	N/A	9	rs4078338	91237253	4.16E-01	SEMA4D	-	1.73E-07	9.98E-01	1.90E-05	1867	
8	rs2469764	22475136	9.92E-01	SORBS3	16	rs1566183	74589238	8.45E-01	N/A	-	1.73E-07	4.70E-01	9.28E-06	1868	
6	rs9395787	13552717	7.23E-01	GFOD1	6	rs13220480	1.19E+08	9.44E-01	N/A	-	1.73E-07	2.58E-01	3.87E-06	1869	
3	rs9857526	1.24E+08	7.05E-02	SEC22A	12	rs10747874	59144947	1.68E-01	N/A	-	1.73E-07	6.36E-02	1.22E-03	1870	
2	rs6742645	75386274	5.90E-01	N/A	10	rs953920	1.18E+08	1.93E-01	GFRA1	-	1.73E-07	6.07E-01	4.47E-05	1871	
5	rs1645732	39273844	3.51E-01	FYB	13	rs12430962	84516283	6.14E-01	N/A	-	1.73E-07	9.78E-01	1.25E-05	1872	
2	rs1834619	17764966	5.69E-01	SMC6	8	rs1317084	34796689	6.57E-01	N/A	-	1.74E-07	3.41E-02	1.02E-02	1873	
2	rs1834619	17764966	5.69E-01	SMC6	8	rs2254888	34789074	6.57E-01	N/A	-	1.74E-07	3.41E-02	1.02E-02	1874	
2	rs1834619	17764966	5.69E-01	SMC6	8	rs2685592	34774314	6.57E-01	N/A	-	1.74E-07	3.41E-02	1.02E-02	1875	
2	rs1834619	17764966	5.69E-01	SMC6	8	rs2685609	34794461	6.57E-01	N/A	-	1.74E-07	3.41E-02	1.02E-02	1876	
2	rs1834619	17764966	5.69E-01	SMC6	8	rs2687543	34799762	6.57E-01	N/A	-	1.74E-07	3.41E-02	1.02E-02	1877	
2	rs12464087	15820363	8.09E-01	N/A	8	rs11782215	1.43E+08	5.01E-01	N/A	-	1.74E-07	8.20E-01	1.09E-04	1878	
2	rs1834619	17764966	5.69E-01	SMC6	8	rs2685589	34769092	6.57E-01	N/A	-	1.74E-07	N/A	N/A	1879	
2	rs1834619	17764966	5.69E-01	SMC6	8	rs2685593	34783010	6.57E-01	N/A	-	1.74E-07	N/A	N/A	1880	
3	rs10514695	36283102	3.53E-01	N/A	16	rs12923619	6951319	7.89E-01	A2BP1	-	1.74E-07	3.00E-01	6.56E-06	1881	
5	rs1345592	1.69E+08	1.10E-01	SLIT3	9	rs1444835	74442041	1.62E-01	TMC1	-	1.74E-07	7.26E-01	3.33E-05	1882	
2	rs12052617	1.03E+08	3.74E-01	N/A	10	rs10796161	14230820	4.82E-01	FRMD4A	-	1.74E-07	6.06E-01	3.89E-04	1883	
11	rs10895059	96411255	9.68E-01	N/A	13	rs1928955	65715323	8.99E-01	N/A	-	1.74E-07	7.91E-01	5.51E-05	1884	
9	rs1107108	70508637	4.38E-01	PIP5K1B	17	rs1869934	74746266	4.52E-01	HRNBP3	-	1.74E-07	3.10E-01	3.93E-06	1885	
7	rs6962380	17940428	4.89E-01	SNX13	14	rs734606	94272240	2.92E-01	N/A	-	1.74E-07	6.62E-01	1.45E-04	1886	
7	rs6962380	17940428	4.89E-01	SNX13	14	rs8006054	94273061	2.92E-01	N/A	-	1.74E-07	6.62E-01	1.45E-04	1887	
3	rs4370006	1.8E+08	2.46E-02	KCNMB2	20	rs2296903	17653226	3.85E-02	BANF2	-	1.74E-07	1.12E-01	6.42E-03	1888	
1	rs16822888	36656533	5.34E-01	OSCP1	16	rs982205	27306957	2.20E-01	IL21R	-	1.74E-07	5.60E-01	2.39E-06	1889	
5	rs12523655	1.7E+08	5.99E-01	KCNIP1	11	rs11234874	86306884	6.83E-01	N/A	-	1.74E-07	8.37E-01	3.02E-04	1890	
6	rs2844665	31114834	8.49E-01	HCG22	6	rs7750269	31379136	8.58E-01	N/A	MHC	1.75E-07	3.41E-02	1.82E-07	1891	
1	rs11208641	38440246	2.93E-01	N/A	11	rs4340049	11805098	2.22E-01	USP47	-	1.75E-07	3.27E-02	4.20E-03	1892	
1	rs2281960	1.65E+08	6.11E-01	GPA33	12	rs2632226	75535673	3.99E-01	N/A	-	1.75E-07	4.76E-01	5.98E-05	1893	
1	rs2281962	1.65E+08	6.11E-01	GPA33	12	rs2632226	75535673	3.99E-01	N/A	-	1.75E-07	4.76E-01	5.98E-05	1894	
6	rs957387	15429838	7.84E-01	JARID2	21	rs2248626	41034004	4.38E-01	DSCAM	-	1.75E-07	1.75E-01	1.47E-03	1895	
1	rs12748225	33528130	8.72E-01	ZNF362	11	rs4620675	24998331	5.92E-01	LUZP2	-	1.75E-07	4.75E-01	3.67E-03	1896	
3	rs10510956	66597327	4.03E-01	LRIG1	18	rs11080775	14572736	1.07E-01	N/A	-	1.75E-07	8.60E-01	7.27E-05	1897	
11	rs1787666	65005665	3.80E-01	MALAT1	12	rs7132140	77260928	3.36E-01	N/A	-	1.75E-07	8.74E-01	4.36E-05	1898	
10	rs1949884	50441425	2.11E-01	N/A	10	rs1898355	1.19E+08	9.14E-01	N/A	-	1.75E-07	9.54E-01	2.12E-05	1899	
12	rs11610805	96658862	5.35E-01	N/A	16	rs4476188	11382829	2.44E-01	N/A	-	1.75E-07	1.88E-01	1.34E-03	1900	
8	rs16939382	77948002	9.53E-01	ZFHX4	8	rs2443786	1.09E+08	8.95E-01	RSPO2	-	1.75E-07	7.78E-01	5.74E-05	1901	
8	rs7007712	11328212	7.52E-01	C8orf12	15	rs11247349	96851676	8.76E-01	FAM169B	-	1.75E-07	6.97E-01	7.58E-06	1902	

SNP A					SNP B						Interaction P			Ranking	Cluster in top 100 interactions
CHR	SNP	Location	gle locus P va	Gene	CHR	SNP	Location	Single locus P valu	Gene	MHC region	Stage 1	Stage 2	Combined		
4	rs11132007	1.82E+08	9.06E-01	N/A	18	rs1430916	38525965	9.25E-01	N/A	-	1.75E-07	4.30E-01	8.82E-06	1903	
10	rs11101464	49643764	9.14E-01	WDFY4	10	rs522549	80305869	5.07E-01	N/A	-	1.75E-07	4.32E-01	2.11E-04	1904	
6	rs796716	93746305	3.02E-01	N/A	14	rs1204982	71289922	4.06E-02	SIPA1L1	-	1.75E-07	8.38E-01	2.31E-04	1905	
6	rs554089	93735234	3.02E-01	N/A	14	rs1204982	71289922	4.06E-02	SIPA1L1	-	1.75E-07	9.06E-01	1.87E-04	1906	
6	rs9380215	31157634	4.29E-02	N/A	6	rs3094205	31199841	5.91E-01	C6orf15	MHC	1.76E-07	7.32E-02	4.57E-07	1907	
2	rs11888670	31174338	9.34E-01	GALNT14	11	rs746490	86083732	8.55E-01	N/A	-	1.76E-07	1.23E-01	4.50E-07	1908	
1	rs7552260	30624265	7.04E-01	N/A	18	rs13381162	55096022	9.11E-01	RAX	-	1.76E-07	2.02E-01	1.36E-03	1909	
4	rs17725302	1.78E+08	6.09E-01	N/A	5	rs4513726	1.43E+08	8.03E-01	N/A	-	1.76E-07	1.44E-01	4.38E-07	1910	
4	rs1519313	1.87E+08	3.01E-03	N/A	11	rs17143395	81687956	6.24E-02	N/A	-	1.76E-07	3.79E-01	9.33E-05	1911	
1	rs1340592	67793691	4.96E-01	N/A	12	rs11168514	46985543	9.11E-01	N/A	-	1.76E-07	7.15E-01	1.01E-04	1912	
1	rs6662607	67794090	4.96E-01	N/A	12	rs11168514	46985543	9.11E-01	N/A	-	1.76E-07	7.15E-01	1.01E-04	1913	
1	rs6693292	67795756	4.96E-01	N/A	12	rs11168514	46985543	9.11E-01	N/A	-	1.76E-07	7.15E-01	1.01E-04	1914	
1	rs4655733	67798226	4.96E-01	N/A	12	rs11168514	46985543	9.11E-01	N/A	-	1.76E-07	7.73E-01	8.36E-05	1915	
12	rs10772019	32976571	9.65E-01	N/A	12	rs11615997	1.28E+08	3.34E-01	TMEM132D	-	1.76E-07	2.01E-01	1.16E-03	1916	
1	rs4129186	2.13E+08	6.85E-01	PTPN14	13	rs12584299	1.08E+08	7.00E-02	N/A	-	1.76E-07	6.06E-01	1.36E-05	1917	
5	rs2731714	1.72E+08	5.77E-01	SH3PXD2B	22	rs9609684	31759708	2.52E-01	SYN3	-	1.76E-07	5.19E-01	2.64E-04	1918	
5	rs4360024	77719962	5.37E-01	SCAMP1	11	rs826044	73445145	5.08E-01	C2CD3	-	1.76E-07	6.47E-01	1.61E-05	1919	
1	rs6678713	1.89E+08	5.35E-01	FAM5C	15	rs685935	53558203	4.55E-01	DYX1C1	-	1.77E-07	6.04E-01	1.73E-05	1920	
13	rs1924578	96267186	2.15E-01	HS6ST3	22	rs2027855	46216177	7.20E-01	N/A	-	1.77E-07	9.73E-01	1.81E-05	1921	
3	rs2037184	1.89E+08	2.28E-01	N/A	8	rs3134133	1.08E+08	2.41E-01	OXR1	-	1.77E-07	4.60E-01	2.62E-04	1922	
3	rs2037184	1.89E+08	2.28E-01	N/A	8	rs1350984	1.08E+08	2.41E-01	OXR1	-	1.77E-07	5.04E-01	2.11E-04	1923	
18	rs948655	52924341	7.33E-01	N/A	20	rs2426214	49007217	9.38E-01	MOCS3	-	1.77E-07	6.84E-01	1.70E-04	1924	
3	rs2037184	1.89E+08	2.28E-01	N/A	8	rs3101525	1.08E+08	2.41E-01	OXR1	-	1.77E-07	N/A	N/A	1925	
1	rs10489639	1.59E+08	7.88E-01	CD48	16	rs10514566	81385638	7.82E-02	CDH13	-	1.77E-07	3.44E-01	2.74E-04	1926	
7	rs971818	1.47E+08	1.79E-01	CNTN/AP2	11	rs10894710	1.33E+08	4.13E-01	N/A	-	1.77E-07	3.79E-01	4.27E-05	1927	
7	rs17166711	13075823	7.09E-02	N/A	19	rs1859955	62514181	6.47E-01	ZNF460	-	1.77E-07	5.08E-01	3.04E-06	1928	
7	rs12533128	13071663	7.09E-02	N/A	19	rs1859955	62514181	6.47E-01	ZNF460	-	1.77E-07	5.80E-01	4.62E-06	1929	
7	rs7778887	13071534	7.09E-02	N/A	19	rs1859955	62514181	6.47E-01	ZNF460	-	1.77E-07	5.80E-01	4.62E-06	1930	
12	rs904647	1.23E+08	2.43E-01	FAM101A	17	rs2529378	67326448	2.71E-02	N/A	-	1.77E-07	8.93E-02	1.88E-07	1931	
12	rs7953959	96052271	8.83E-01	N/A	19	rs2334253	50808317	2.61E-01	N/A	-	1.77E-07	9.47E-01	4.15E-05	1932	
1	rs2281960	1.65E+08	6.11E-01	GPA33	12	rs11114901	75581238	5.64E-01	N/A	-	1.77E-07	3.45E-01	1.14E-04	1933	
1	rs2281962	1.65E+08	6.11E-01	GPA33	12	rs11114901	75581238	5.64E-01	N/A	-	1.77E-07	3.45E-01	1.14E-04	1934	
7	rs7804782	12525324	4.08E-01	N/A	19	rs735386	13225932	3.41E-01	CACN/A1A	-	1.77E-07	3.66E-01	6.32E-04	1935	
2	rs2059412	1752354	7.59E-01	MYT1L	8	rs4562370	90704907	8.65E-01	N/A	-	1.78E-07	1.35E-01	1.48E-06	1936	
5	rs6869061	25051322	5.04E-01	N/A	9	rs4977871	23633682	9.19E-01	N/A	-	1.78E-07	3.84E-01	1.12E-05	1937	
6	rs2233965	31188878	4.20E-03	C6orf15	6	rs4122189	31275906	7.70E-02	HCG27	MHC	1.78E-07	4.89E-01	2.57E-05	1938	
5	rs37249	76052731	5.41E-01	IQGAP2	8	rs932649	18833654	9.10E-01	PSD3	-	1.78E-07	N/A	N/A	1939	
6	rs1158747	1.13E+08	4.15E-01	LAMA4	2	rs12995942	16226902	7.82E-01	N/A	-	1.78E-07	1.93E-01	1.67E-04	1940	
9	rs4879930	35815413	1.47E-01	TMEM8B	14	rs2144039	22799242	2.41E-01	HOMEZ	-	1.78E-07	6.91E-01	2.85E-04	1941	
3	rs6782326	19327455	8.22E-02	KCNH8	19	rs2302600	17802143	8.06E-01	JAK3	-	1.78E-07	6.46E-01	1.97E-04	1942	
5	rs11949188	1.68E+08	3.45E-01	WWC1	7	rs1181728	1.41E+08	3.90E-02	N/A	-	1.78E-07	7.35E-01	2.65E-04	1943	
6	rs2517552	31115569	8.54E-01	HCG22	6	rs7750269	31379136	8.58E-01	N/A	MHC	1.78E-07	3.96E-02	2.21E-07	1944	
17	rs1631484	39346388	4.09E-01	MPP2	18	rs4891904	66962989	9.00E-01	N/A	-	1.78E-07	4.61E-02	8.53E-08	1945	
17	rs3760360	39344518	4.09E-01	MPP2	18	rs4891904	66962989	9.00E-01	N/A	-	1.78E-07	4.61E-02	8.53E-08	1946	
3	rs6779681	1.39E+08	8.78E-02	N/A	15	rs12902878	40131086	7.80E-01	PLA2G4D	-	1.79E-07	8.32E-01	8.43E-05	1947	
4	rs17021918	95781900	5.69E-03	PDLIM5	4	rs10489022	1.38E+08	9.82E-01	N/A	-	1.79E-07	8.45E-01	1.67E-05	1948	
6	rs1265110	31227401	8.90E-01	TCF19	6	rs9368675	31380136	7.91E-01	N/A	MHC	1.79E-07	9.59E-01	6.02E-05	1949	
4	rs4690563	1.79E+08	5.72E-01	LOC285501	19	rs7249604	58709070	6.35E-01	ZNF813	-	1.79E-07	2.54E-01	2.54E-06	1950	
6	rs1265256	4429854	2.13E-02	N/A	16	rs756813	23198310	4.85E-01	N/A	-	1.79E-07	3.78E-01	2.59E-04	1951	
3	rs2046227	1.02E+08	6.40E-01	GPR128	10	rs2306409	1036712	7.95E-01	GTPBP4	-	1.79E-07	1.01E-01	1.56E-07	1952	
2	rs6717788	6546864	7.04E-01	N/A	4	rs1027655	1.08E+08	8.60E-01	N/A	-	1.79E-07	2.35E-01	2.63E-06	1953	
1	rs1808806	1.18E+08	8.04E-01	FAM46C	2	rs2245273	57618825	3.40E-01	N/A	-	1.79E-07	5.68E-01	1.06E-04	1954	
6	rs6932549	75548849	4.83E-01	N/A	11	rs2919040	96085117	2.21E-01	N/A	-	1.79E-07	9.55E-01	8.11E-05	1955	
1	rs4845733	1.5E+08	2.42E-01	N/A	9	rs10757753	28506764	2.45E-01	LINGO2	-	1.79E-07	4.66E-01	7.34E-06	1956	
3	rs11713033	1.59E+08	7.34E-01	N/A	5	rs250210	50328781	4.18E-01	N/A	-	1.79E-07	8.41E-02	9.94E-08	1957	
9	rs1444826	74536439	2.27E-02	TMC1	17	rs2323661	15241034	1.56E-01	N/A	-	1.79E-07	2.30E-01	2.03E-06	1958	
6	rs1989634	1.11E+08	5.73E-01	N/A	13	rs2318058	1.12E+08	5.76E-01	N/A	-	1.79E-07	5.45E-01	4.96E-04	1959	
6	rs3130534	31317024	3.50E-01	N/A	6	rs9368675	31380136	7.91E-01	N/A	MHC	1.79E-07	2.88E-01	7.49E-06	1960	
4	rs6831971	6418368	2.47E-01	PPP2R2C	8	rs3134369	1.02E+08	5.86E-01	N/A	-	1.80E-07	6.04E-01	6.95E-06	1961	
12	rs549433	61467083	8.12E-01	PPM1H	12	rs11178988	70606698	5.61E-03	TPH2	-	1.80E-07	3.77E-01	4.79E-04	1962	
12	rs7969363	3173346	4.25E-02	TSPAN9	21	rs468725	27108137	9.34E-01	N/A	-	1.80E-07	1.03E-01	3.14E-03	1963	
8	rs352806	15662778	7.12E-01	TUSC3	22	rs735455	20743793	9.48E-01	N/A	-	1.80E-07	3.74E-01	1.48E-06	1964	
14	rs11158751	68101178	8.01E-01	RAD51L1	20	rs1555300	42863904	8.23E-01	RIMS4	-	1.80E-07	6.88E-01	2.30E-05	1965	
6	rs6901012	1.39E+08	8.65E-01	NHSL1	12	rs7958878	99646096	2.32E-01	N/A	-	1.80E-07	7.99E-01	4.01E-05	1966	
2	rs4675812	2.42E+08	7.38E-01	FARP2	15	rs17659026	51120707	5.54E-02	N/A	-	1.80E-07	2.24E-03	1.46E-02	1967	
1	rs10918609	1.65E+08	9.07E-01	MAEL	4	rs7657542	20374965	2.32E-01	KCNIP4	-	1.80E-07	8.85E-01	1.60E-04	1968	
6	rs6929407	82540364	9.93E-01	N/A	14	rs9806105	79676933	3.82E-01	N/A	-	1.80E-07	6.16E-01	2.21E-04	1969	
6	rs1265110	31227401	8.90E-01	TCF19	6	rs7750269	31379136	8.58E-01	N/A	MHC	1.81E-07	1.86E-01	4.48E-06	1970	
3	rs6765225	59534529	6.78E-01	N/A	10	rs1247419	29948134	9.45E-01	SVIL	-	1.81E-07	4.97E-02	5.28E-03	1971	
13	rs7318560	23579068	8.60E-01	N/A	22	rs714006	48233723	6.49E-01	N/A	-	1.81E-07	9.78E-01	9.48E-05	1972	
5	rs4645374	1.09E+08	1.52E-01	N/A	10	rs12262014	11379015	1.26E-01	CUGBP2	-	1.81E-07	7.60E-01	2.72E-04	1973	
3	rs11709882	1.61E+08	2.24E-01	N/A	20	rs722829	117701	5.20E-01	DEFB128	-	1.81E-07	2.54E-01	2.58E-06	1974	
6	rs9396677	16574200	5.82E-02	ATXN1	7	rs17792244	9200867	2.38E-01	N/A	-	1.81E-07	5.55E-01	3.59E-06	1975	
4	rs17012312	1.28E+08	5.47E-02	N/A	8	rs1440332	73897644	2.54E-01	KCNB2	-	1.81E-07	2.82E-01	4.56E-04	1976	
2	rs7600191	2.21E+08	4.76E-01	N/A	5	rs6596352	1.36E+08	5.11E-01	N/A	-	1.81E-07	9.84E-01	4.73E-05	1977	
4	rs675465	20152915	3.83E-01	SLIT2	7	rs2054788	46218211	5.35E-01	N/A	-	1.81E-07	7.19E-01	1.45E-04	1978	
14	rs12887958	22652409	9.48E-02	C14orf119	21	rs7281211	42618010	6.76E-02	TFF3	-	1.81E-07	7.99E-01	3.14E-04	1979	
1	rs695117	1.86E+08	6.04E-01	N/A	11	rs1943620	1.13E+08	4.66E-01	NCAM1	-	1.81E-07	9.45E-01	3.21E-05	1980	
6	rs3095250	31316319	6.90E-02	N/A	18	rs2732229	71742290	5.44E-01	N/A	MHC	1.82E-07	8.74E-02	6.05E-07	1981	
6	rs4947296	31166157	4.31E-02	N/A	6	rs3094205	31199841	5.91E-01	C6orf15	MHC	1.82E-07	9.43E-01	1.34E-04	1982	
5	rs10515789	1.58E+08	5.21E-01	EBF1	12	rs7133991	24200930	1.83E-01	SOX5	-	1.82E-07	1.83E-02	3.44E-07	1983	
5	rs3842965	1.26E+08	7.39E-01	N/A	14	rs12879626	33790885	6.08E-01	N/A	-	1.82E-07	7.74E-01	1.05E-05	1984	
1	rs1483198	4617251	2.86E-01	AJAP1	14	rs2287650	77435365	7.96E-02	ADCK1	-	1.82E-07	6.44E-01	3.20E-05	1985	
1	rs12564233	91637710	9.59E-01	HFM1	2	rs1872787	77844332	5.73E-01	N/A	-	1.82E-07	6.86E-01	3.64E-05	1986	
1	rs1483198	4617251	2.86E-01	AJAP1	14	rs7147735	77430207	7.96E-02	ADCK1	-	1.82E-07	7.86E-01	8.96E-05	1987	
8	rs4355773	26121818	1.03E-01	N/A	10	rs941828	89447015	3.40E-01	PAPSS2	-	1.82E-07	N/A	N/A	1988	
6	rs6570957	1.5E+08	2.74E-01	N/A	9	rs980462	25343887	1.40E-01	N/A	-	1.82E-07	4.07E-01	7.53E-04	1989	
2	rs892464	1.1E+08	9.32E-02	10-Sep	3	rs1032164	1.19E+08	3.38E-01	N/A	-	1.82E-07	N/A	N/A	1990	
5	rs152907	50182686	4.22E-01	N/A	19	rs10403684	34552727	2.52E-01	N/A	-	1.82E-07	1.72E-01	2.77E-03	1991	
8	rs1982401	1.11E+08	9.82E-01	N/A	12	rs876594	3531820	6.92E-01	PRMT8	-	1.82E-07	3.54E-01	1.28E-06	1992	
1	rs1021737	70677388	8.98E-01	CTH	8	rs16887011	37657180	9.85E-01	ZNF703	-	1.82E-07	3.58E-02	3.23E-07	1993	
4	rs9308115	1.67E+08	3.78E-01	N/A	9	rs6559402	80521159	9.45E-01	N/A	-	1.83E-07	8.20E-01	5.66E-05	1994	
1	rs2228552	31938082	9.44E-01	COL16A1	10	rs12571983	1.29E+08	8.92E-01	N/A	-	1.83E-07	5.93E-05	5.93E-05	1995	
8	rs3808521	23220312	8.95E-01	LOXL2	19	rs12052065	17102649	9.51E-01	MYO9B	-	1.83E-07	1.80E-01	6.33E-07	1996	
14	rs12887958	22652409	9.48E-02	C14orf119	21	rs9981660	42611007	7.44E-02	TFF3	-	1.83E-07	7.06E-01	2.10E-04	1997	
2	rs12989626	1.39E+08	2.83E-01	N/A	16	rs1544357	19117111	1.90E-01	SYT17	-	1.83E-07	4.46E-01	5.65E-06	1998	
1	rs12144807	79945399	6.54E-01	N/A	3	rs4858060	23167699	6.43E-04	N/A	-	1.83E-07	8.26E-01	2.29E-04	1999	
1	rs12144677	79944988	6.54E-01	N/A	3	rs4858060	23167699	6.43E-04	N/A	-	1.83E-07	9.84E-01	9.83E-05	2000	
5	rs2901821	1.64E+08	2.17E-01	N/A	12	rs1521381	77392088	8.19E-01	N/A	-	1.83E-07	9.87E-01	4.70E-05	2001	
1	rs6671507	41730026	7.62E-01	HIVEP3	2	rs929390	1.59E+08	8.17E-01	PKP4	-	1.83E-07	4.53E-01	5.67E-06	2002	
5	rs10059955	1.05E+08	1.38E-01	N/A	12	rs10506439	61930601	4.64E-01	N/A	-	1.83E-07	9.08E-01	2.82E-05	2003	
4	rs13110363	1.63E+08	2.81E-01	FSTL5	13	rs597294	1.01E+08	1.50E-01	N/ALCN	-	1.84E-07	8.25E-01	1.16E-05	2004	
7	rs6616	16790503	2.76E-02	AGR2	8	rs2126128	1.41E+08	9.14E-01	TRAPPC9	-	1.84E-07	1.21E-01	1.25E-03	2005	
4	rs4555724	1.42E+08	3.18E-01	TBC1D9	16	rs11640300	3932784	8.21E-01	ADCY9	-	1.84E-07	9.61E-01	8.82E-06	2006	
3	rs9637471	59978630	3.65E-01	FHIT	7	rs11773422	47698241	5.62E-02	N/A	-	1.84E-07	5.44E-01	3.22E-04	2007	
1	rs1417144	14338672	4.77E-03	N/A	5	rs166078	1.63E+08	5.00E-01	N/A	-	1.84E-07	7.07E-01	1.29E-04	2008	
5	rs1030179	1.23E+08	6.89E-01	N/A	10	rs11005556	58284273	2.88E-01	N/A	-	1.84E-07	4.71E-01	1.26E-04	2009	
5	rs305722	84668872	4.05E-01	N/A	17	rs1719480	70062993	6.34E-01	CD300C	-	1.84E-07	7.34E-01	2.50E-04	2010	
5	rs10064153	17838281	8.07E-01	N/A	10	rs1414397	13474981	4.65E-01	N/A	-	1.84E-07	3.32E-01	1.46E-06	2011	
5	rs1904163	52221753	7.92E-01	ITGA1	12	rs11178549	69720071	2.01E-01	N/A	-	1.84E-07	9.85E-01	2.87E-05	2012	
10	rs7477069	54212144	4.45E-01	MBL2	18	rs940311	50169447	1.44E-01	C18orf54	-	1.84E-07	7.50E-02	3.27E-07	2013	
16	rs7500355	84634601	7.85E-01	N/A	17	rs2176134	22166482	9.37E-01	N/A	-	1.84E-07	2.42E-01	7.22E-04	2014	

SNP A					SNP B						Interaction P			Ranking	Cluster in top 100 interactions
CHR	SNP	Location	gle locus P va	Gene	CHR	SNP	Location	Single locus P valu	Gene	HC region	Stage 1	Stage 2	Combined		
8	rs11780291	16802532	9.16E-02	N/A	10	rs10509252	67491726	6.44E-01	CTNN/A3	-	1.84E-07	2.82E-01	5.85E-04	2015	
3	rs13068994	4769128	1.33E-01	ITPR1	10	rs10788392	86742325	7.41E-01	N/A	-	1.84E-07	6.92E-01	3.09E-05	2016	
5	rs378228	94353608	9.58E-01	MCTP1	9	rs2230806	1.07E+08	6.23E-01	ABCA1	-	1.84E-07	8.19E-01	6.65E-05	2017	
6	rs17058986	99537921	6.53E-01	N/A	11	rs10895701	1.04E+08	7.12E-01	N/A	-	1.85E-07	5.18E-01	7.66E-05	2018	
6	rs17058986	99537921	6.53E-01	N/A	11	rs1352283	1.04E+08	7.12E-01	N/A	-	1.85E-07	5.18E-01	7.66E-05	2019	
2	rs4611634	2.21E+08	2.80E-01	N/A	9	rs12378755	29308695	7.58E-01	N/A	-	1.85E-07	4.05E-01	1.77E-04	2020	
2	rs7585061	1.68E+08	8.77E-01	XIRP2	19	rs889362	18619255	5.85E-02	KLHL26	-	1.85E-07	2.96E-01	1.34E-04	2021	
6	rs833623	44006755	8.37E-01	LOC100132354	8	rs4739941	84490303	4.16E-01	N/A	-	1.85E-07	4.45E-01	5.53E-04	2022	
3	rs1153462	3132051	8.71E-03	IL5RA	20	rs2425656	42939661	5.30E-01	YWHAB	-	1.85E-07	1.50E-01	1.89E-03	2023	
7	rs722263	92724748	1.09E-01	CCDC132	13	rs9541313	67737117	4.18E-01	N/A	-	1.85E-07	2.11E-01	2.10E-06	2024	
6	rs9379638	10744123	6.89E-01	GCNT2	14	rs9323573	24467061	8.89E-01	STXBP6	-	1.85E-07	5.78E-01	2.31E-06	2025	
1	rs489969	2.36E+08	7.22E-02	RYR2	17	rs11868833	74621639	8.77E-02	HRNBP3	-	1.85E-07	9.93E-01	1.62E-04	2026	
1	rs490057	2.36E+08	7.22E-02	RYR2	17	rs11868833	74621639	8.77E-02	HRNBP3	-	1.85E-07	9.93E-01	1.62E-04	2027	
8	rs7008075	68514834	2.12E-01	CPA6	16	rs12600104	54624250	8.64E-01	N/A	-	1.85E-07	2.37E-01	3.14E-06	2028	
7	rs10255418	6037382	2.49E-03	EIF2AK1	14	rs1958645	53391156	3.94E-02	N/A	-	1.85E-07	2.49E-01	4.34E-06	2029	
2	rs1476698	2.42E+08	7.09E-01	FARP2	15	rs17710001	51119600	4.74E-02	N/A	-	1.85E-07	4.48E-03	1.10E-02	2030	
2	rs6437294	2.41E+08	1.05E-02	OTOS	5	rs1504998	82236917	8.18E-01	N/A	-	1.85E-07	2.43E-01	8.35E-04	2031	
6	rs11155954	1.55E+08	4.51E-03	IPCEF1	3	rs966199	1.97E+08	9.10E-01	TNK2	-	1.86E-07	4.56E-01	9.91E-06	2032	
11	rs1187177	91624103	5.29E-01	N/A	11	rs7105053	1.3E+08	9.37E-01	N/A	-	1.86E-07	1.01E-02	9.15E-09	2033	
6	rs9455531	1.69E+08	7.14E-01	N/A	11	rs1540082	85979365	8.72E-01	ME3	-	1.86E-07	3.03E-01	4.22E-04	2034	
10	rs723212	61395919	8.45E-02	C10orf40	10	rs2817694	98856512	3.60E-01	SLIT1	-	1.86E-07	2.04E-01	1.81E-03	2035	
2	rs13006682	1.05E+08	8.30E-01	N/A	20	rs2423967	15529484	1.83E-01	MACROD2	-	1.86E-07	4.02E-01	1.99E-05	2036	
2	rs13027051	42871962	5.81E-01	HAAO	4	rs11730156	22095646	5.10E-01	GPR125	-	1.86E-07	6.15E-02	4.08E-03	2037	
5	rs29775	1.72E+08	1.97E-01	C5orf41	12	rs1881227	87562931	9.76E-01	N/A	-	1.86E-07	2.87E-01	4.02E-06	2038	
3	rs1395006	1.33E+08	6.48E-01	N/A	15	rs7183261	92129998	3.06E-01	N/A	-	1.87E-07	2.69E-01	2.66E-06	2039	
1	rs2117905	76825184	3.98E-01	ST6GALN/AC3	1	rs2815209	2.16E+08	5.43E-01	N/A	-	1.87E-07	8.77E-01	8.52E-05	2040	
2	rs4673807	2.15E+08	5.15E-01	SPAG16	13	rs9516948	96928731	8.96E-01	RAP2A	-	1.87E-07	9.98E-01	5.91E-05	2041	
2	rs17012765	76565481	9.53E-02	N/A	5	rs17214697	66153861	2.63E-01	MAST4	-	1.87E-07	5.44E-01	5.61E-06	2042	
3	rs4331688	67916439	6.44E-01	N/A	20	rs6097667	51965103	4.71E-01	N/A	-	1.87E-07	8.12E-02	1.18E-06	2043	
6	rs1830642	1.53E+08	3.98E-04	SYNE1	7	rs6959814	1.41E+08	8.80E-02	N/A	-	1.87E-07	5.66E-01	1.16E-04	2044	
6	rs2758805	1.53E+08	3.98E-04	SYNE1	7	rs6959814	1.41E+08	8.80E-02	N/A	-	1.87E-07	5.66E-01	1.16E-04	2045	
6	rs2758811	1.53E+08	3.98E-04	SYNE1	7	rs6959814	1.41E+08	8.80E-02	N/A	-	1.87E-07	5.66E-01	1.16E-04	2046	
10	rs1888606	90447613	9.07E-01	LIPF	21	rs2837693	40832232	1.95E-01	DSCAM	-	1.87E-07	3.76E-01	8.04E-05	2047	
14	rs179090	29899759	6.97E-01	N/A	15	rs1387556	66094514	2.22E-01	N/A	-	1.87E-07	7.32E-01	4.43E-05	2048	
10	rs1890628	1.3E+08	7.51E-01	N/A	11	rs2306729	12194691	1.79E-01	MICAL2	-	1.87E-07	5.39E-01	2.22E-05	2049	
9	rs1326809	1.19E+08	6.83E-01	ASTN2	17	rs657672	34572591	2.17E-03	PLXDC1	-	1.87E-07	4.22E-01	1.35E-04	2050	
9	rs1326809	1.19E+08	6.83E-01	ASTN2	17	rs657723	34572629	2.17E-03	PLXDC1	-	1.87E-07	4.22E-01	1.35E-04	2051	
10	rs472664	1.08E+08	6.69E-01	N/A	17	rs1860413	56869243	3.49E-01	TBX4	-	1.88E-07	2.15E-02	3.63E-07	2052	
2	rs11692657	1.33E+08	9.46E-01	NCKAP5	3	rs755292	24521271	6.63E-01	THRB	-	1.88E-07	1.64E-02	1.24E-02	2053	
5	rs521135	80323930	9.31E-01	RASGRF2	11	rs644497	81488295	3.86E-01	N/A	-	1.88E-07	4.46E-01	6.37E-04	2054	
3	rs4831090	1.17E+08	5.50E-01	LSAMP	8	rs6984840	10169975	9.13E-01	MSRA	-	1.88E-07	7.22E-01	1.41E-04	2055	
7	rs10239606	28362592	4.29E-01	CREB5	13	rs485988	74807197	2.17E-01	TBC1D4	-	1.88E-07	5.61E-01	1.63E-05	2056	
7	rs4721998	21228763	7.69E-01	N/A	12	rs3782637	5703791	1.17E-01	ANO2	-	1.88E-07	N/A	N/A	2057	
1	rs12130711	2.46E+08	7.87E-02	NLRP3	7	rs1016019	9945064	8.61E-01	N/A	-	1.88E-07	4.04E-01	1.64E-06	2058	
1	rs4657349	1.62E+08	6.73E-01	N/A	22	rs5766879	46571296	4.51E-02	N/A	-	1.88E-07	5.78E-01	8.20E-04	2059	
13	rs4473069	43009971	4.52E-01	ENOX1	14	rs4900550	1.02E+08	2.80E-01	RCOR1	-	1.88E-07	6.38E-01	4.90E-06	2060	
1	rs16860429	1.81E+08	6.09E-01	LAMC2	11	rs11029672	26657670	5.54E-01	SLC5A12	-	1.88E-07	8.74E-01	1.29E-05	2061	
12	rs549433	61467083	8.12E-01	PPM1H	12	rs17110426	70601249	5.13E-03	TPH2	-	1.88E-07	1.24E-01	8.82E-07	2062	
5	rs2632151	1.13E+08	2.65E-01	N/A	10	rs7071792	1.22E+08	8.01E-01	N/A	-	1.88E-07	7.58E-01	6.08E-05	2063	
6	rs1362070	29850278	4.50E-02	HCG4	10	rs7097635	31000212	5.63E-01	N/A	MHC	1.88E-07	4.55E-02	3.91E-07	2064	
8	rs1351424	1.36E+08	8.70E-01	N/A	13	rs1340745	68414215	1.43E-01	N/A	-	1.88E-07	4.63E-01	3.35E-04	2065	
6	rs3130267	33414772	3.22E-01	DAXX	2	rs10495592	12165935	8.57E-01	N/A	MHC	1.89E-07	9.57E-01	2.61E-05	2066	
6	rs7761965	31381474	9.15E-01	N/A	6	rs6929796	31630648	6.69E-01	NFKBIL1	MHC	1.89E-07	N/A	N/A	2067	
3	rs11919151	1.59E+08	8.51E-01	VEPH1	18	rs4617954	68770627	7.45E-01	N/A	-	1.89E-07	1.47E-01	1.16E-03	2068	
5	rs2567803	1.21E+08	8.46E-01	N/A	7	rs10464419	1.54E+08	1.52E-01	DPP6	-	1.89E-07	8.61E-01	5.75E-05	2069	
3	rs7629215	1.95E+08	6.17E-01	N/A	20	rs12479923	38039524	2.06E-01	N/A	-	1.89E-07	N/A	N/A	2070	
8	rs2981060	70121659	5.30E-01	N/A	14	rs4905904	99378672	6.39E-01	EML1	-	1.89E-07	4.80E-01	2.41E-06	2071	
4	rs6850000	1.67E+08	1.89E-02	CPE	5	rs26784	80085648	6.39E-01	MSH3	-	1.89E-07	2.41E-01	5.81E-07	2072	
6	rs1953635	1.41E+08	2.53E-01	N/A	18	rs559687	5970877	3.36E-01	L3MBTL4	-	1.89E-07	4.45E-01	2.15E-05	2073	
8	rs452325	88574482	6.21E-02	N/A	9	rs7031605	7074558	5.37E-01	KDM4C	-	1.89E-07	6.51E-01	1.57E-04	2074	
7	rs2691576	17921601	4.83E-01	SNX13	14	rs734606	94272240	2.92E-01	N/A	-	1.89E-07	9.03E-01	9.30E-05	2075	
7	rs2691576	17921601	4.83E-01	SNX13	14	rs8006054	94273061	2.92E-01	N/A	-	1.89E-07	9.03E-01	9.30E-05	2076	
7	rs2714854	17921819	4.83E-01	SNX13	14	rs734606	94272240	2.92E-01	N/A	-	1.89E-07	9.03E-01	9.30E-05	2077	
7	rs2714854	17921819	4.83E-01	SNX13	14	rs8006054	94273061	2.92E-01	N/A	-	1.89E-07	9.03E-01	9.30E-05	2078	
10	rs1949884	50441425	2.11E-01	N/A	10	rs10886031	1.19E+08	8.25E-01	N/A	-	1.89E-07	9.74E-01	2.62E-05	2079	
9	rs1444835	74442041	1.62E-01	TMC1	17	rs2323661	15241034	1.56E-01	N/A	-	1.89E-07	4.90E-01	5.35E-04	2080	
6	rs2395185	32541145	6.23E-01	N/A	19	rs6512102	16315110	8.67E-01	EPS15L1	MHC	1.90E-07	6.58E-01	9.65E-05	2081	
4	rs10026454	6083357	6.62E-01	JAKMIP1	7	rs1525459	1.44E+08	7.31E-01	N/A	-	1.90E-07	7.28E-01	2.91E-05	2082	
7	rs10262194	6241110	2.02E-02	CYTH3	12	rs1469999	1.03E+08	4.32E-01	NT5DC3	-	1.90E-07	6.08E-01	1.97E-05	2083	
4	rs2023829	1.06E+08	9.64E-02	N/A	13	rs1981058	38731403	7.56E-01	N/A	-	1.90E-07	7.76E-01	2.34E-04	2084	
4	rs3109836	26017925	9.71E-01	RBPJ	5	rs3105236	1.02E+08	1.28E-01	N/A	-	1.90E-07	1.51E-01	2.30E-03	2085	
7	rs518136	17047682	4.62E-01	N/A	15	rs7497302	59658128	8.65E-01	N/A	-	1.90E-07	8.55E-01	8.61E-05	2086	
6	rs4709792	1.64E+08	5.80E-01	N/A	17	rs4794031	44697152	8.27E-01	N/A	-	1.90E-07	6.09E-01	1.05E-05	2087	
1	rs4844678	2.07E+08	7.95E-02	N/A	10	rs1333217	84569810	7.04E-01	NRG3	-	1.90E-07	9.39E-01	7.92E-05	2088	
9	rs4381051	1.26E+08	1.81E-01	N/A	13	rs7324647	1.03E+08	1.29E-01	N/A	-	1.90E-07	7.75E-02	1.12E-02	2089	
4	rs9992295	64901642	5.50E-01	TECRL	7	rs10950503	2006120	4.02E-01	MAD1L1	-	1.90E-07	5.01E-01	1.13E-03	2090	
2	rs6710668	18443299	8.29E-01	N/A	2	rs1608590	76259467	5.61E-01	N/A	-	1.90E-07	9.41E-01	6.57E-05	2091	
9	rs4977867	23603083	7.49E-04	N/A	13	rs2329151	79498340	8.29E-01	N/A	-	1.90E-07	4.61E-02	4.04E-03	2092	
4	rs236754	1.03E+08	8.32E-01	N/A	10	rs4747895	11277515	3.41E-01	CUGBP2	-	1.90E-07	8.93E-01	1.22E-04	2093	
12	rs10859809	93888058	9.27E-02	NDUFA12	20	rs6017995	44770445	6.23E-01	SLC2A10	-	1.90E-07	N/A	N/A	2094	
2	rs11694256	1.16E+08	2.76E-01	DPP10	9	rs2006996	1.17E+08	2.03E-01	N/A	-	1.91E-07	8.76E-01	6.83E-05	2095	
1	rs3008204	2.12E+08	3.28E-01	N/A	3	rs300978	87406024	5.62E-02	CHMP2B	-	1.91E-07	8.10E-01	1.25E-05	2096	
7	rs16878220	14557271	8.50E-01	DGKB	12	rs6538288	90583215	5.92E-01	N/A	-	1.91E-07	8.61E-01	5.61E-05	2097	
6	rs4896364	1.39E+08	7.74E-01	NHSL1	9	rs1984008	27319792	6.92E-01	MOBKL2B	-	1.91E-07	1.33E-01	7.84E-04	2098	
13	rs1171080	35385286	5.95E-01	DCLK1	13	rs2297943	1E+08	9.54E-01	TMTC4	-	1.91E-07	2.95E-01	7.64E-06	2099	
6	rs1876606	54320923	7.46E-01	TIN/AG	11	rs609920	1.32E+08	6.21E-02	OPCML	-	1.91E-07	2.29E-01	1.02E-05	2100	
3	rs1284746	1.04E+08	4.76E-01	N/A	13	rs7998964	47686991	6.92E-01	ITM2B	-	1.91E-07	2.77E-01	2.17E-06	2101	
3	rs1284746	1.04E+08	4.76E-01	N/A	13	rs9534995	47684077	6.92E-01	N/A	-	1.91E-07	2.77E-01	2.17E-06	2102	
3	rs1356106	1.04E+08	4.76E-01	N/A	13	rs7998964	47686991	6.92E-01	ITM2B	-	1.91E-07	2.77E-01	2.17E-06	2103	
3	rs1356106	1.04E+08	4.76E-01	N/A	13	rs9534995	47684077	6.92E-01	N/A	-	1.91E-07	2.77E-01	2.17E-06	2104	
3	rs1284746	1.04E+08	4.76E-01	N/A	13	rs9534981	47653682	6.92E-01	N/A	-	1.91E-07	2.89E-01	2.39E-06	2105	
3	rs1356106	1.04E+08	4.76E-01	N/A	13	rs9534981	47653682	6.92E-01	N/A	-	1.91E-07	2.89E-01	2.39E-06	2106	
11	rs2275998	66083157	1.21E-01	ACTN3	16	rs4786876	6505930	9.35E-01	A2BP1	-	1.92E-07	6.91E-01	1.49E-05	2107	
4	rs7665555	74737693	2.69E-01	N/A	8	rs3812440	1.45E+08	6.45E-01	C8orf51	-	1.92E-07	3.30E-01	1.87E-03	2108	
16	rs187431	65464372	4.90E-01	PDP2	16	rs12598715	79020900	4.17E-01	N/A	-	1.92E-07	3.50E-01	1.09E-06	2109	
5	rs4379190	1.26E+08	2.45E-01	ALDH7A1	19	rs10412487	8834217	8.77E-01	MUC16	-	1.92E-07	8.90E-01	3.87E-05	2110	
12	rs7958980	41545011	1.55E-01	N/A	13	rs2120065	75380231	8.39E-01	N/A	-	1.92E-07	5.17E-02	1.54E-03	2111	
17	rs8077733	28043066	3.83E-01	MYO1D	18	rs8097986	46445557	7.03E-01	MAPK4	-	1.92E-07	6.85E-01	5.34E-04	2112	
13	rs9542990	71993272	9.49E-01	N/A	14	rs2180388	1E+08	1.75E-01	N/A	-	1.92E-07	1.30E-01	1.66E-06	2113	
3	rs10514695	36283102	3.53E-01	N/A	16	rs12149452	6977388	9.69E-01	A2BP1	-	1.92E-07	5.70E-01	1.37E-05	2114	
12	rs3181301	6216045	9.20E-01	CD9	12	rs748380	1.16E+08	7.37E-01	C12orf49	-	1.92E-07	6.51E-01	1.43E-04	2115	
4	rs17798302	1.82E+08	8.28E-01	N/A	18	rs1430916	38525965	9.25E-01	N/A	-	1.92E-07	3.84E-01	8.13E-06	2116	
3	rs6665	1.56E+08	3.92E-02	MME	11	rs668095	59756876	5.75E-03	N/A	-	1.92E-07	6.25E-01	4.63E-05	2117	
3	rs6665	1.56E+08	3.92E-02	MME	11	rs668134	59756902	5.75E-03	N/A	-	1.92E-07	6.25E-01	4.63E-05	2118	
1	rs600647	1.08E+08	1.60E-01	NTNG1	3	rs6778609	17163396	5.38E-01	TBC1D5	-	1.92E-07	8.49E-01	8.02E-05	2119	
5	rs378228	94353608	9.58E-01	MCTP1	9	rs2253174	1.07E+08	6.17E-01	ABCA1	-	1.92E-07	8.19E-01	6.89E-05	2120	
1	rs10926185	2.38E+08	9.52E-01	FMN2	9	rs2278278	92648560	5.18E-01	N/A	-	1.93E-07	1.81E-01	8.17E-07	2121	
6	rs9386913	1.11E+08	8.71E-01	N/A	13	rs2318058	1.12E+08	5.76E-01	N/A	-	1.93E-07	3.53E-01	1.13E-05	2122	
1	rs2050656	2.36E+08	2.48E-01	RYR2	10	rs7086823	56607632	3.27E-01	N/A	-	1.93E-07	N/A	N/A	2123	
3	rs1920083	1.11E+08	1.17E-01	N/A	5	rs3936585	1.33E+08	4.50E-01	N/A	-	1.93E-07	1.75E-01	8.68E-07	2124	
1	rs1603141	2.32E+08	1.14E-01	N/A	14	rs1037295	42398071	1.61E-01	N/A	-	1.93E-07	8.01E-01	3.74E-05	2125	
1	rs10797493	2.32E+08	1.14E-01	N/A	14	rs1037295	42398071	1.61E-01	N/A	-	1.93E-07	9.45E-01	5.68E-05	2126	

SNP A					SNP B						Interaction P			Ranking	Cluster in top 100 interactions
CHR	SNP	Location	gle locus P va	Gene	CHR	SNP	Location	Single locus P valu	Gene	HC region	Stage 1	Stage 2	Combined		
8	rs6991392	1893230	9.90E-01	ARHGEF10	14	rs1956174	93885874	9.65E-01	N/A	-	1.93E-07	4.11E-01	6.45E-06	2127	
2	rs4599058	2.36E+08	4.76E-01	N/A	4	s12508123	1.81E+08	7.86E-01	N/A	-	1.93E-07	8.70E-01	1.16E-05	2128	
5	rs12519172	1.61E+08	6.94E-01	N/A	21	rs220170	42428369	1.12E-01	UMODL1	-	1.93E-07	N/A	N/A	2129	
6	rs3130559	31205280	7.50E-01	C6orf15	6	rs9267649	31932807	1.17E-02	C6orf48	MHC	1.94E-07	5.45E-01	4.02E-06	2130	
6	rs9348050	1.66E+08	6.98E-01	N/A	5	rs7718690	1.74E+08	6.85E-01	N/A	-	1.94E-07	8.99E-01	1.45E-04	2131	
1	rs922114	4411281	9.54E-01	N/A	7	s10262722	29506730	9.03E-01	CHN2	-	1.94E-07	5.94E-01	1.63E-04	2132	
1	rs885098	68506066	2.59E-01	N/A	5	rs6892958	1.47E+08	1.16E-01	JAKMIP2	-	1.94E-07	6.05E-01	2.31E-05	2133	
1	rs10489667	1.15E+08	9.12E-01	N/A	3	rs3805015	4797145	5.43E-01	ITPR1	-	1.94E-07	8.37E-01	9.49E-06	2134	
19	rs919803	35678944	1.57E-01	ZNF536	22	rs6009503	47918047	1.89E-01	N/A	-	1.94E-07	9.62E-01	1.63E-04	2135	
3	rs12487078	1.28E+08	4.87E-01	N/A	5	s13188321	1.12E+08	7.84E-01	N/A	-	1.94E-07	1.05E-01	8.89E-07	2136	
8	rs391916	88581402	6.22E-02	N/A	9	rs7031605	7074558	5.37E-01	KDM4C	-	1.94E-07	7.43E-01	1.20E-04	2137	
8	rs463809	88582958	6.22E-02	N/A	9	rs7031605	7074558	5.37E-01	KDM4C	-	1.94E-07	N/A	N/A	2138	
13	rs9318743	80677414	1.71E-01	N/A	22	s12628683	24795266	3.91E-01	N/A	-	1.95E-07	3.46E-01	9.42E-06	2139	
6	rs1361429	1.65E+08	8.00E-01	N/A	2	s13387662	34016918	2.26E-01	N/A	-	1.95E-07	8.22E-01	1.17E-05	2140	
1	rs16858460	1.65E+08	8.22E-01	DUSP27	12	rs2669013	75547079	6.30E-01	N/A	-	1.95E-07	N/A	N/A	2141	
3	rs9828692	1.35E+08	5.16E-02	BFSP2	4	rs4627935	1.42E+08	1.59E-01	RNF150	-	1.95E-07	8.08E-01	2.16E-05	2142	
1	rs2240335	17547124	1.17E-02	PADI4	13	rs486299	71844010	8.94E-01	N/A	-	1.95E-07	6.89E-01	1.13E-04	2143	
3	rs17822079	77403430	2.55E-01	ROBO2	16	rs2347642	27017929	8.43E-01	N/A	-	1.95E-07	3.07E-01	5.75E-06	2144	
1	rs6670349	2.13E+08	8.24E-01	PTPN14	13	s11069721	1.08E+08	5.06E-02	N/A	-	1.95E-07	6.50E-01	1.75E-04	2145	
10	rs1679414	4495344	2.71E-01	N/A	16	rs4782717	81167218	2.78E-01	N/A	-	1.95E-07	2.05E-01	2.95E-07	2146	
3	rs9814549	1.01E+08	6.90E-01	C3orf26	8	rs7812526	16933751	6.37E-01	EFHA2	-	1.95E-07	7.84E-01	7.33E-06	2147	
3	rs11131064	7189945	5.95E-01	GRM7	20	rs6020994	49178718	1.03E-01	N/A	-	1.95E-07	9.71E-01	7.65E-05	2148	
11	rs538371	1.07E+08	4.50E-01	CWF19L2	18	rs1550716	35493076	1.30E-03	LOC647946	-	1.95E-07	1.11E-01	1.02E-06	2149	
15	rs12909171	97796941	3.15E-01	N/A	17	rs9914954	28872301	1.11E-01	ACCN1	-	1.95E-07	2.69E-01	1.40E-06	2150	
5	rs262749	1.65E+08	6.94E-01	N/A	16	rs274073	24795616	7.80E-01	SLC5A11	-	1.95E-07	5.12E-01	4.71E-04	2151	
7	rs4723037	30966625	1.87E-01	GHRHR	15	s12913547	65254561	3.52E-01	SMAD3	-	1.95E-07	8.96E-01	4.51E-05	2152	
8	rs4873274	49401130	6.75E-01	N/A	12	rs975085	43457609	1.41E-01	NELL2	-	1.95E-07	9.96E-01	6.48E-05	2153	
5	rs4921086	1.16E+08	3.80E-01	COMMD10	18	rs7240382	72579628	1.92E-01	N/A	-	1.95E-07	6.71E-01	1.96E-05	2154	
10	rs10998957	71198936	5.17E-01	N/A	22	rs5770953	49409804	9.14E-01	ARSA	-	1.96E-07	3.10E-01	1.60E-06	2155	
6	rs3798349	34879798	2.18E-01	UHRF1BP1	11	s12420859	44681103	6.76E-01	N/A	MHC	1.96E-07	N/A	N/A	2156	
18	rs2032165	8978701	6.78E-01	N/A	20	rs232291	43767808	8.10E-01	WFDC10B	-	1.96E-07	4.99E-01	8.99E-04	2157	
5	rs2914723	9994041	4.07E-02	N/A	14	rs2208432	25201299	6.84E-03	N/A	-	1.96E-07	5.59E-01	6.30E-04	2158	
6	rs1555035	9930468	7.62E-01	N/A	19	rs7255568	556985	1.86E-01	POLRMT	-	1.96E-07	4.83E-01	4.09E-06	2159	
3	rs2049233	1.98E+08	6.09E-01	PAK2	4	rs4693491	83846607	9.47E-01	SCD5	-	1.96E-07	1.23E-01	3.39E-08	2160	
8	rs4355773	26121818	1.03E-01	N/A	10	rs3781191	89452970	5.28E-01	PAPSS2	-	1.96E-07	7.27E-01	1.88E-05	2161	
2	rs13407109	2.18E+08	9.06E-02	TNS1	12	rs1245810	78161526	9.82E-01	SYT1	-	1.96E-07	7.34E-01	1.83E-04	2162	
1	rs6427807	1.98E+08	3.10E-01	FAM58B	8	s10956670	1.34E+08	9.94E-01	HPYR1	-	1.96E-07	5.02E-01	3.98E-05	2163	
5	rs6873909	9340073	7.25E-01	SEMA5A	19	rs2301661	18898053	3.09E-01	HOMER3	-	1.96E-07	9.34E-01	1.61E-04	2164	
6	rs1570055	1.37E+08	9.72E-01	MAP3K5	8	rs2979419	62167326	2.62E-01	N/A	-	1.96E-07	1.96E-01	1.30E-06	2165	
6	rs9483945	1.37E+08	9.72E-01	MAP3K5	8	rs2979419	62167326	2.62E-01	N/A	-	1.96E-07	2.49E-01	1.79E-06	2166	
2	rs4234085	2.09E+08	2.12E-01	N/A	7	s10273717	20519346	1.20E-01	N/A	-	1.96E-07	4.75E-01	2.86E-04	2167	
3	rs1881364	10946358	1.30E-01	SLC6A11	5	rs900531	10689151	1.97E-01	ANKRD33B	-	1.96E-07	6.74E-01	1.27E-04	2168	
9	rs2808707	86748114	9.60E-01	NTRK2	11	rs503591	58435036	7.11E-01	N/A	-	1.96E-07	6.59E-01	7.56E-06	2169	
3	rs17331632	1.27E+08	1.32E-01	SNX4	15	rs813299	29225595	8.06E-01	N/A	-	1.97E-07	1.62E-01	5.76E-07	2170	
2	rs1067350	44523450	9.60E-01	C2orf34	3	s10933677	1.96E+08	7.25E-01	GP5	-	1.97E-07	7.98E-01	4.58E-05	2171	
7	rs17166689	13068758	6.44E-02	N/A	19	rs1859955	62514181	6.47E-01	ZNF460	-	1.97E-07	N/A	N/A	2172	
2	rs6541877	1.24E+08	5.97E-01	N/A	8	rs878873	21890527	2.78E-01	XPO7	-	1.97E-07	7.67E-02	3.10E-03	2173	
5	rs1030179	1.23E+08	6.89E-01	N/A	10	rs1914568	58171445	1.89E-01	N/A	-	1.97E-07	3.13E-01	2.99E-04	2174	
10	rs619778	3634879	1.18E-01	N/A	18	rs9954817	11431243	1.86E-01	N/A	-	1.97E-07	4.59E-01	7.38E-04	2175	
2	rs16847299	1.34E+08	9.78E-01	NCKAP5	12	rs917634	6501430	4.78E-01	N/A	-	1.97E-07	5.22E-01	1.08E-04	2176	
2	rs16847409	1.34E+08	9.78E-01	NCKAP5	12	rs917634	6501430	4.78E-01	N/A	-	1.97E-07	6.00E-01	7.65E-05	2177	
1	rs559798	2.43E+08	9.42E-01	C1orf100	11	rs7107014	43264387	8.69E-01	N/A	-	1.97E-07	7.77E-01	1.41E-04	2178	
3	rs9827214	1.59E+08	8.27E-01	N/A	5	rs250210	50328781	4.18E-01	N/A	-	1.97E-07	8.09E-02	1.05E-07	2179	
12	rs10859809	93888058	9.27E-02	NDUFA12	20	rs4810539	44757415	6.23E-01	SLC2A10	-	1.97E-07	1.52E-01	6.31E-07	2180	
7	rs6948739	2899721	1.13E-02	CARD11	12	rs7309495	1.12E+08	2.29E-01	RPH3A	-	1.97E-07	3.69E-01	2.63E-04	2181	
7	rs1544521	1.24E+08	7.96E-01	N/A	13	rs4769507	26092813	9.42E-01	WASF3	-	1.97E-07	9.13E-01	4.86E-05	2182	
4	rs1513697	1.23E+08	6.86E-01	N/A	5	rs474807	1.11E+08	3.71E-01	C5orf13	-	1.98E-07	2.42E-02	1.40E-07	2183	
6	rs674026	1.51E+08	7.55E-02	IYD	10	s10787025	1.09E+08	5.62E-01	N/A	-	1.98E-07	1.72E-01	6.28E-07	2184	
7	rs270904	1.37E+08	7.97E-01	DGKI	7	s12534759	1.41E+08	3.69E-01	AGK	-	1.98E-07	7.19E-01	2.80E-05	2185	
5	rs1514858	17776087	3.99E-01	N/A	11	s10160599	21369124	4.12E-01	NELL1	-	1.98E-07	N/A	N/A	2186	
11	rs7930159	69133252	5.40E-01	N/A	22	rs2413485	36523866	3.78E-02	H1F0	-	1.98E-07	7.66E-01	1.10E-04	2187	
3	rs696067	1.91E+08	6.85E-01	LEPREL1	11	rs1057128	2753813	7.31E-01	KCNQ1	-	1.98E-07	N/A	N/A	2188	
11	rs12577015	10381085	4.10E-01	N/A	17	rs917538	14975566	6.48E-01	N/A	-	1.98E-07	3.45E-01	6.55E-04	2189	
2	rs698811	44557180	8.42E-01	C2orf34	3	s13082706	1.96E+08	7.66E-01	ATP13A3	-	1.99E-07	8.02E-01	6.21E-05	2190	
3	rs4858021	21827143	8.93E-03	N/A	3	rs1497762	1.61E+08	9.59E-01	SCHIP1	-	1.99E-07	2.54E-01	1.80E-06	2191	
3	rs6776685	35708268	5.08E-01	ARPP-21	9	rs3802347	1.33E+08	8.87E-01	ABL1	-	1.99E-07	3.16E-01	1.60E-03	2192	
1	rs12748225	33528130	8.72E-01	ZNF362	11	rs1446201	25010231	6.48E-01	LUZP2	-	1.99E-07	4.75E-01	3.70E-03	2193	
1	rs2293464	2.25E+08	5.22E-01	PARP1	16	rs2908668	5013774	7.33E-01	SEC14L5	-	1.99E-07	3.90E-01	1.89E-03	2194	
2	rs6547035	75945152	1.08E-01	N/A	11	rs7948432	1.01E+08	4.46E-01	N/A	-	1.99E-07	7.50E-01	4.64E-05	2195	
1	rs6413913	1.88E+08	6.92E-01	N/A	12	rs796041	88164489	9.89E-01	N/A	-	1.99E-07	4.20E-01	4.43E-06	2196	
4	rs17034632	1.06E+08	3.31E-01	N/A	11	rs446134	36364621	8.61E-02	PRR5L	-	1.99E-07	4.49E-01	8.97E-05	2197	
13	rs9534651	46919997	2.74E-01	N/A	19	rs7252293	11225495	1.50E-01	DOCK6	-	1.99E-07	2.33E-01	1.93E-06	2198	
5	rs10866630	1.68E+08	8.98E-01	SLIT3	22	rs5765545	44400710	7.09E-01	N/A	-	1.99E-07	2.73E-01	9.01E-06	2199	
7	rs1525500	1.1E+08	3.04E-01	N/A	17	s12940825	11795027	1.94E-02	DN/AH9	-	1.99E-07	6.47E-01	4.10E-05	2200	
6	rs372578	7832222	6.84E-01	BMP6	1	s11249395	1.21E+08	2.57E-02	LOC647121	-	2.00E-07	3.73E-01	4.10E-06	2201	
11	rs621922	93994962	2.89E-01	PIWIL4	21	s13047224	16961894	1.26E-01	N/A	-	2.00E-07	4.17E-01	3.53E-06	2202	
11	rs621922	93994962	2.89E-01	PIWIL4	21	rs2823950	16961016	1.26E-01	N/A	-	2.00E-07	4.17E-01	3.53E-06	2203	
11	rs11233500	82559055	6.63E-01	PCF11	22	rs743185	27433217	6.71E-01	CHEK2	-	2.00E-07	7.31E-01	1.04E-05	2204	
6	rs7755781	47807689	2.88E-02	GPR115	15	s12905507	56908905	7.07E-01	FAM63B	-	2.00E-07	9.96E-01	1.50E-05	2205	
4	rs4697177	20369187	1.66E-01	KCNIP4	18	rs8082808	54469013	4.70E-01	N/A	-	2.00E-07	8.16E-01	8.17E-05	2206	
2	rs13387224	51910176	9.60E-01	N/A	21	rs2825728	19969339	3.81E-01	N/A	-	2.00E-07	1.64E-01	3.07E-06	2207	
16	rs4784502	53756719	3.41E-01	N/A	22	rs2282667	21554058	9.35E-01	N/A	-	2.00E-07	9.78E-01	1.92E-05	2208	
5	rs9327438	1.27E+08	5.38E-01	MEGF10	7	s10081235	1.58E+08	2.26E-03	PTPRN2	-	2.00E-07	7.82E-01	4.00E-05	2209	
5	rs9327438	1.27E+08	5.38E-01	MEGF10	7	rs4909239	1.58E+08	2.26E-03	PTPRN2	-	2.00E-07	7.82E-01	4.00E-05	2210	
5	rs295677	58186560	8.26E-01	RAB3C	9	rs4838267	1.19E+08	2.98E-01	ASTN2	-	2.00E-07	N/A	N/A	2211	
8	rs1376842	83055073	6.14E-01	N/A	12	s10785630	45166647	1.36E-02	N/A	-	2.00E-07	1.16E-02	4.21E-08	2212	
6	rs7742508	70834071	2.99E-01	COL19A1	8	rs2242093	1.45E+08	1.98E-02	ZC3H3	-	2.00E-07	5.92E-01	4.09E-05	2213	
16	rs864837	86164322	6.67E-01	N/A	22	rs7287612	32607434	9.42E-01	LARGE	-	2.01E-07	2.69E-02	1.32E-07	2214	
6	rs879882	31247431	7.61E-01	TCF19	6	rs6933050	31451611	1.57E-01	HLA-B	MHC	2.01E-07	5.99E-01	9.07E-06	2215	
7	rs39099	29259620	4.15E-01	CHN2	16	rs6564068	83302251	2.72E-01	USP10	-	2.01E-07	9.95E-01	7.67E-06	2216	
10	rs3861027	72961172	3.65E-01	CDH23	17	s11077708	69442873	2.03E-01	N/A	-	2.01E-07	2.00E-01	4.40E-06	2217	
7	rs10255671	1.51E+08	4.48E-01	CRYGN	20	rs1926076	54314184	7.18E-01	N/A	-	2.01E-07	4.17E-01	2.48E-05	2218	
7	rs6966644	19360045	3.98E-01	N/A	13	rs7331320	71573196	7.15E-02	N/A	-	2.01E-07	6.17E-01	1.63E-05	2219	
7	rs6946884	15846661	9.92E-01	N/A	7	s11973288	52306768	2.61E-02	N/A	-	2.01E-07	1.46E-01	3.65E-08	2220	
9	rs2808707	86748114	9.60E-01	NTRK2	11	rs474253	58469528	9.29E-01	N/A	-	2.01E-07	7.93E-01	1.80E-04	2221	
6	rs6932930	34785081	2.64E-01	C6orf106	11	s12420859	44681103	6.76E-01	N/A	MHC	2.01E-07	8.59E-01	1.49E-05	2222	
13	rs4773539	89356301	5.20E-01	N/A	14	rs1449115	84073885	1.97E-01	N/A	-	2.02E-07	6.54E-02	2.97E-07	2223	
13	rs4773539	89356301	5.20E-01	N/A	14	rs895889	84082471	1.97E-01	N/A	-	2.02E-07	7.01E-02	3.28E-07	2224	
6	rs313211	86065034	6.16E-01	N/A	10	rs7477268	5360986	1.33E-01	N/A	-	2.02E-07	8.72E-01	6.92E-05	2225	
6	rs12528811	5732399	1.57E-01	FARS2	2	rs6715170	42941808	4.89E-01	N/A	-	2.02E-07	2.11E-01	2.17E-06	2226	
6	rs7742508	70834071	2.99E-01	COL19A1	8	s12679598	1.45E+08	1.98E-02	ZC3H3	-	2.02E-07	6.23E-01	3.61E-05	2227	
1	rs3125923	68631067	2.98E-01	N/A	20	rs1926076	54314184	7.18E-01	N/A	-	2.02E-07	7.91E-02	2.94E-03	2228	
10	rs814620	90412116	8.85E-01	LIPF	21	rs2837686	40828369	2.51E-01	DSCAM	-	2.02E-07	3.51E-01	1.35E-06	2229	
11	rs715849	1.19E+08	9.16E-01	PVRL1	13	rs1041329	60197135	8.46E-01	N/A	-	2.02E-07	1.19E-01	6.07E-07	2230	
1	rs11807227	3799283	9.49E-01	DFFB	7	s10486650	34607010	9.14E-01	N/A	-	2.02E-07	8.17E-01	1.20E-04	2231	
11	rs715849	1.19E+08	9.16E-01	PVRL1	13	rs1329699	60196350	7.97E-01	N/A	-	2.02E-07	N/A	N/A	2232	
6	rs6937783	1.06E+08	8.02E-01	N/A	2	rs6736615	1.63E+08	7.22E-03	KCNH7	-	2.02E-07	2.62E-01	6.96E-06	2233	
11	rs341058	72063373	2.52E-01	PDE2A	11	rs1873886	1.3E+08	1.82E-01	N/A	-	2.03E-07	1.79E-01	3.40E-07	2234	
7	rs13222586	1.45E+08	9.48E-02	N/A	8	rs410487	8775015	6.14E-01	MFHAS1	-	2.03E-07	1.53E-01	9.96E-07	2235	
5	rs160874	3114140	3.77E-01	N/A	10	rs1710309	1.28E+08	6.75E-01	ADAM12	-	2.03E-07	7.83E-02	4.20E-03	2236	
2	rs2306797	1.73E+08	2.91E-01	N/A	10	rs7071660	1.29E+08	4.83E-01	DOCK1	-	2.03E-07	4.93E-01	1.67E-05	2237	
3	rs563275	1.89E+08	9.63E-01	N/A	20	rs6081622	19441514	2.89E-01	SLC24A3	-	2.03E-07	8.43E-02	1.47E-03	2238	

SNP A					SNP B						Interaction P			Ranking	Cluster in top 100 interactions
CHR	SNP	Location	gle locus P va	Gene	CHR	SNP	Location	Single locus P valu	Gene	MHC region	Stage 1	Stage 2	Combined		
4	rs2061231	45333604	5.46E-01	N/A	8	rs7017482	87990792	2.17E-01	CNBD1	-	2.03E-07	1.85E-01	1.05E-02	2239	
4	rs2061231	45333604	5.46E-01	N/A	8	rs4557720	88001939	2.17E-01	CNBD1	-	2.03E-07	2.29E-01	8.71E-03	2240	
11	rs10894710	1.33E+08	4.13E-01	N/A	14	rs393142	45393416	8.43E-01	N/A	-	2.03E-07	3.51E-01	3.35E-06	2241	
8	rs4129294	1.18E+08	2.34E-01	N/A	10	rs10887363	86515945	6.96E-01	N/A	-	2.04E-07	4.00E-01	1.99E-06	2242	
1	rs3008204	2.12E+08	3.28E-01	N/A	3	rs2642318	87430156	6.31E-02	N/A	-	2.04E-07	7.79E-01	1.36E-05	2243	
1	rs3008204	2.12E+08	3.28E-01	N/A	3	rs300989	87427487	6.31E-02	POU1F1	-	2.04E-07	7.79E-01	1.36E-05	2244	
1	rs3008204	2.12E+08	3.28E-01	N/A	3	rs903292	87434070	6.31E-02	N/A	-	2.04E-07	7.79E-01	1.36E-05	2245	
1	rs3008204	2.12E+08	3.28E-01	N/A	3	rs1845427	87440279	6.31E-02	N/A	-	2.04E-07	N/A	N/A	2246	
8	rs10503617	18474753	6.82E-01	PSD3	10	rs4750217	5745906	1.02E-01	ASB13	-	2.04E-07	5.07E-01	1.75E-04	2247	
7	rs4731401	1.28E+08	3.69E-01	N/A	18	rs11874922	49140166	2.06E-01	DCC	-	2.04E-07	9.86E-01	5.29E-05	2248	
9	rs11792210	92069102	2.88E-01	N/A	22	rs3810597	17660403	1.85E-01	CLTCL1	-	2.04E-07	5.61E-01	1.17E-05	2249	
16	rs2526269	2996585	4.66E-01	CLDN9	22	rs133985	26353875	7.62E-01	N/A	-	2.04E-07	5.65E-02	1.05E-03	2250	
18	rs7238355	32291755	3.85E-01	FHOD3	19	rs2013899	57360509	2.10E-01	ZNF836	-	2.04E-07	8.63E-01	6.52E-05	2251	
9	rs10976178	7276273	6.08E-01	N/A	18	rs1553803	43377597	2.98E-01	N/A	-	2.04E-07	4.01E-01	9.59E-04	2252	
8	rs2980932	6713422	4.48E-01	DEFB1	11	rs6483763	21302450	2.80E-02	NELL1	-	2.04E-07	6.45E-01	1.78E-04	2253	
10	rs7098507	14484566	9.71E-01	N/A	10	rs1915440	62866140	7.54E-01	TMEM26	-	2.04E-07	N/A	N/A	2254	
2	rs12990794	10409436	7.42E-01	HPCAL1	12	rs4761378	76330817	6.54E-02	N/A	-	2.04E-07	3.23E-01	9.23E-06	2255	
5	rs2162928	6137255	3.48E-01	N/A	5	rs4117784	1.54E+08	8.70E-01	LARP1	-	2.04E-07	3.94E-01	2.15E-04	2256	
7	rs509834	70775938	3.81E-01	WBSCR17	10	rs11255628	8390349	5.17E-01	N/A	-	2.04E-07	5.20E-01	3.29E-04	2257	
13	rs9315923	41968894	6.51E-01	N/A	14	rs12437348	35676301	7.54E-01	N/A	-	2.04E-07	5.59E-01	8.61E-06	2258	
13	rs9315923	41968894	6.51E-01	N/A	14	rs10220323	35652691	7.54E-01	N/A	-	2.04E-07	6.58E-01	1.28E-05	2259	
13	rs9315923	41968894	6.51E-01	N/A	14	rs12431579	35664148	7.54E-01	N/A	-	2.04E-07	6.58E-01	1.28E-05	2260	
10	rs1409332	95395804	4.38E-01	PDE6C	15	rs9806697	45858085	1.91E-01	SEMA6D	-	2.04E-07	3.94E-01	6.61E-06	2261	
2	rs11897520	2.07E+08	7.57E-01	N/A	8	rs1127379	41240437	3.05E-01	SFRP1	-	2.04E-07	3.88E-01	1.59E-04	2262	
4	rs2583636	83372203	6.35E-01	N/A	8	rs11774937	2082418	3.95E-01	MYOM2	-	2.04E-07	7.48E-01	4.69E-05	2263	
9	rs8999	1.34E+08	6.99E-01	TTF1	9	rs943423	1.37E+08	6.11E-01	N/A	-	2.04E-07	8.99E-01	1.29E-04	2264	
1	rs12027674	1.61E+08	5.21E-01	NOS1AP	22	rs713821	25059005	1.71E-01	N/A	-	2.05E-07	1.27E-01	2.91E-07	2265	
1	rs6677154	1.61E+08	5.21E-01	NOS1AP	22	rs713821	25059005	1.71E-01	N/A	-	2.05E-07	1.27E-01	2.91E-07	2266	
3	rs9872307	1.88E+08	2.51E-01	N/A	5	rs2695978	1.66E+08	7.80E-03	N/A	-	2.05E-07	9.04E-01	2.80E-05	2267	
3	rs6807545	1.28E+08	3.79E-01	N/A	5	rs13188321	1.12E+08	7.84E-01	N/A	-	2.05E-07	9.92E-02	9.38E-07	2268	
6	rs2327478	1.35E+08	2.47E-01	N/A	1	rs659070	85687737	4.86E-01	DDAH1	-	2.05E-07	9.34E-01	2.20E-05	2269	
10	rs1327014	1.3E+08	7.85E-01	N/A	20	rs448294	184802	9.54E-01	C20orf96	-	2.05E-07	6.52E-01	8.33E-06	2270	
4	rs1994983	20986195	3.61E-01	KCNIP4	20	rs6139011	3042175	2.03E-01	UBOX5	-	2.05E-07	6.56E-01	1.78E-05	2271	
6	rs17058986	99537921	6.53E-01	N/A	11	rs7944328	1.04E+08	9.72E-01	N/A	-	2.05E-07	6.00E-01	6.61E-05	2272	
2	rs1369066	2.32E+08	3.86E-01	C2orf57	18	rs1521422	4132736	3.16E-01	N/A	-	2.05E-07	6.37E-01	1.30E-05	2273	
13	rs2485773	37868825	5.48E-01	N/A	18	rs568080	32094999	5.37E-01	MOCOS	-	2.05E-07	7.40E-01	5.54E-04	2274	
2	rs7558031	1.93E+08	4.84E-02	N/A	8	rs12677755	1.24E+08	9.68E-01	DERL1	-	2.05E-07	2.02E-01	9.02E-07	2275	
3	rs11720652	14636424	8.72E-01	N/A	3	rs4678512	33267546	2.84E-01	N/A	-	2.05E-07	9.66E-01	6.03E-05	2276	
3	rs475050	22998578	6.54E-01	N/A	13	rs959866	46915771	8.04E-01	N/A	-	2.05E-07	9.88E-01	1.68E-05	2277	
11	rs10502145	1.11E+08	6.59E-01	SIK2	22	rs133467	47329193	6.70E-01	FAM19A5	-	2.05E-07	N/A	N/A	2278	
12	rs1038712	25477846	4.69E-01	N/A	15	rs3825915	99736820	6.82E-01	PCSK6	-	2.05E-07	2.73E-01	6.21E-07	2279	
6	rs6924759	82529275	8.54E-01	FAM46A	14	rs11159448	79675956	4.17E-01	N/A	-	2.05E-07	6.78E-01	1.92E-04	2280	
5	rs4705285	1.48E+08	4.98E-01	N/A	18	rs10514157	70994152	7.49E-01	N/A	-	2.06E-07	5.12E-01	3.15E-06	2281	
5	rs2731714	1.72E+08	5.77E-01	SH3PXD2B	22	rs9609679	31718996	2.96E-02	SYN3	-	2.06E-07	5.26E-01	2.76E-04	2282	
9	rs10985354	1.23E+08	3.54E-01	DAB2IP	10	rs6585481	1.2E+08	4.80E-01	C10orf84	-	2.06E-07	9.39E-01	9.91E-05	2283	
1	rs874349	53404490	8.63E-01	N/A	9	rs10760397	1.27E+08	7.80E-01	GAPVD1	-	2.06E-07	9.50E-01	2.25E-05	2284	
8	rs2409096	8729887	1.70E-01	MFHAS1	21	rs1012766	27329010	3.63E-02	N/A	-	2.06E-07	4.29E-01	6.66E-04	2285	
8	rs2409096	8729887	1.70E-01	MFHAS1	21	rs2830643	27329894	3.63E-02	N/A	-	2.06E-07	4.29E-01	6.66E-04	2286	
9	rs4557815	99251690	5.18E-01	TDRD7	18	rs11876166	33216840	4.40E-01	BRUNOL4	-	2.06E-07	2.85E-01	5.29E-04	2287	
7	rs2160193	13070161	6.14E-02	N/A	19	rs1859955	62514181	6.47E-01	ZNF460	-	2.06E-07	4.45E-01	2.79E-06	2288	
7	rs7793289	13073946	6.14E-02	N/A	19	rs1859955	62514181	6.47E-01	ZNF460	-	2.06E-07	5.39E-01	3.88E-06	2289	
2	rs11902129	66945345	7.04E-01	N/A	10	rs10872825	1.33E+08	4.87E-01	N/A	-	2.06E-07	5.87E-01	3.37E-05	2290	
7	rs11981351	7298240	4.09E-01	N/A	14	rs7161726	61552632	9.18E-01	SYT16	-	2.06E-07	3.80E-01	1.28E-04	2291	
3	rs9875063	1.04E+08	1.90E-01	N/A	10	rs7901814	24886122	6.72E-01	KIAA1217	-	2.06E-07	4.12E-01	6.26E-04	2292	
3	rs6442019	46696322	2.62E-01	ALS2CL	4	rs4396968	46783941	2.29E-01	GABRB1	-	2.06E-07	8.16E-01	1.19E-04	2293	
6	rs9503867	3927524	4.76E-01	N/A	3	rs1991248	1.08E+08	4.61E-01	LOC344595	-	2.06E-07	8.67E-01	2.91E-05	2294	
3	rs2133952	1.58E+08	1.90E-01	N/A	8	rs1492656	96371431	5.16E-02	N/A	-	2.06E-07	2.80E-01	1.04E-03	2295	
5	rs2269290	83420906	6.74E-01	EDIL3	8	rs4736716	1.35E+08	4.61E-01	N/A	-	2.06E-07	3.66E-01	6.93E-06	2296	
11	rs11228726	56208679	4.22E-01	OR8U8	12	rs11060333	1.21E+08	7.60E-02	LRRC43	-	2.06E-07	9.90E-01	1.15E-04	2297	
2	rs896637	2.09E+08	5.59E-01	N/A	7	rs916790	20539225	9.56E-02	N/A	-	2.06E-07	3.43E-01	6.67E-04	2298	
10	rs705468	3648434	1.32E-01	N/A	18	rs9954817	11431243	1.86E-01	N/A	-	2.07E-07	1.35E-01	1.03E-06	2299	
6	rs1737031	29845460	4.76E-02	IFITM4P	10	rs7097635	31000212	5.63E-01	N/A	MHC	2.07E-07	4.59E-01	7.59E-04	2300	
13	rs525884	74689394	3.75E-01	N/A	16	rs8059445	80329239	9.10E-01	N/A	-	2.07E-07	1.60E-01	2.70E-07	2301	
6	rs4463239	1.66E+08	3.48E-01	N/A	4	rs17674102	27223561	1.59E-01	N/A	-	2.07E-07	3.85E-01	5.53E-04	2302	
3	rs6776486	59383218	8.15E-01	N/A	4	rs916913	1.15E+08	6.53E-02	CAMK2D	-	2.07E-07	2.44E-01	2.66E-06	2303	
7	rs1052981	37913026	3.09E-01	TXNDC3	13	rs9533422	42665104	5.00E-01	N/A	-	2.07E-07	6.94E-02	1.59E-03	2304	
6	rs10945364	1.69E+08	7.72E-01	N/A	11	rs1540082	85979365	8.72E-01	ME3	-	2.07E-07	3.57E-01	3.87E-04	2305	
8	rs1457784	1.33E+08	6.39E-01	KCNQ3	22	rs135279	46846234	3.65E-01	N/A	-	2.07E-07	3.67E-01	5.29E-04	2306	
2	rs6546183	25525310	2.93E-01	DTNB	3	rs1092103	1.46E+08	1.72E-01	N/A	-	2.07E-07	3.65E-01	1.39E-03	2307	
5	rs274362	53372577	5.22E-01	ARL15	17	rs820215	71205405	4.61E-01	SAP30BP	-	2.08E-07	5.06E-01	4.25E-04	2308	
3	rs4530474	1.25E+08	8.94E-01	MYLK	12	rs10492029	1.12E+08	1.95E-01	OAS2	-	2.08E-07	9.54E-01	1.91E-05	2309	
3	rs2367605	1.95E+08	9.79E-01	ATP13A5	18	rs1786767	33348572	9.13E-01	BRUNOL4	-	2.08E-07	3.27E-01	7.26E-06	2310	
6	rs2504853	12643097	3.49E-01	N/A	7	rs1159329	67261520	9.33E-01	N/A	-	2.08E-07	8.43E-01	3.70E-05	2311	
9	rs10760846	1.04E+08	7.20E-01	N/A	13	rs9543928	74916808	3.12E-01	TBC1D4	-	2.08E-07	6.73E-01	2.96E-05	2312	
3	rs1153462	3132051	8.71E-03	IL5RA	20	rs2179069	42940668	5.30E-01	YWHAB	-	2.08E-07	1.05E-01	3.70E-03	2313	
5	rs2269290	83420906	6.74E-01	EDIL3	8	rs4736713	1.35E+08	6.87E-01	N/A	-	2.08E-07	6.30E-01	1.91E-05	2314	
2	rs7589659	1.28E+08	2.64E-01	N/A	4	rs13117816	36740726	2.99E-01	N/A	-	2.08E-07	8.46E-01	4.01E-05	2315	
2	rs905656	1.28E+08	2.64E-01	N/A	4	rs13117816	36740726	2.99E-01	N/A	-	2.08E-07	8.46E-01	4.01E-05	2316	
1	rs11578784	2.22E+08	5.54E-02	N/A	10	rs17155529	14670748	5.89E-01	FAM107B	-	2.08E-07	2.26E-01	1.21E-06	2317	
6	rs7752376	1.63E+08	7.31E-01	PACRG	9	rs2065423	12912330	5.00E-01	N/A	-	2.08E-07	2.61E-01	6.06E-06	2318	
2	rs12713665	68881970	3.10E-01	ARHGAP25	5	rs11747611	56045013	9.54E-01	N/A	-	2.08E-07	6.19E-01	4.06E-06	2319	
3	rs1920370	1.17E+08	4.47E-01	LSAMP	7	rs219822	98427987	1.21E-01	TRRAP	-	2.08E-07	1.06E-01	2.15E-06	2320	
1	rs807247	25993456	5.33E-01	MAN1C1	11	rs2282612	1.01E+08	7.87E-01	KIAA1377	-	2.08E-07	3.21E-01	1.07E-05	2321	
18	rs2032165	8978701	6.78E-01	N/A	20	rs232286	43771100	9.09E-01	WFDC10B	-	2.08E-07	7.69E-01	3.64E-04	2322	
2	rs6546862	73713856	4.42E-01	N/AT8	5	rs1911009	8294011	1.89E-01	N/A	-	2.08E-07	4.47E-01	1.02E-05	2323	
2	rs6546862	73713856	4.42E-01	N/AT8	5	rs6555529	8285117	1.89E-01	N/A	-	2.08E-07	4.70E-01	1.12E-05	2324	
6	rs6923034	74493546	8.15E-01	CD109	2	rs2371504	35163479	1.41E-01	N/A	-	2.08E-07	6.82E-01	2.29E-05	2325	
10	rs914672	20687518	2.79E-01	N/A	16	rs2012766	79295143	6.06E-01	CDYL2	-	2.08E-07	8.43E-01	8.17E-05	2326	
1	rs10494667	1.91E+08	2.81E-01	N/A	7	rs4609107	21251829	8.00E-01	N/A	-	2.08E-07	9.57E-01	6.25E-06	2327	
8	rs4631498	1.39E+08	3.84E-01	N/A	12	rs2196488	29190816	7.06E-02	N/A	-	2.08E-07	7.85E-01	1.67E-04	2328	
8	rs7828112	4848045	8.11E-01	CSMD1	8	rs4581170	1.43E+08	8.94E-01	TSN/ARE1	-	2.09E-07	3.29E-01	8.04E-04	2329	
7	rs13235604	47631897	6.05E-01	N/A	20	rs1203938	22542958	4.96E-01	N/A	-	2.09E-07	9.47E-01	9.34E-05	2330	
7	rs13235604	47631897	6.05E-01	N/A	20	rs1203943	22544825	4.96E-01	N/A	-	2.09E-07	9.47E-01	9.34E-05	2331	
7	rs13235604	47631897	6.05E-01	N/A	20	rs1203930	22538835	4.96E-01	N/A	-	2.09E-07	9.96E-01	1.11E-04	2332	
5	rs30359	55870159	1.37E-01	N/A	13	rs1325968	93673950	4.00E-01	GPC6	-	2.09E-07	9.66E-01	1.46E-04	2333	
9	rs7860062	84736837	8.07E-01	N/A	12	rs7968515	28587114	7.22E-01	CCDC91	-	2.09E-07	1.28E-01	2.49E-03	2334	
4	rs4693498	83859356	9.47E-01	SCD5	12	rs3819526	2306783	4.68E-01	CACN/A1C	-	2.09E-07	7.25E-01	1.67E-05	2335	
4	rs6535384	83858517	9.47E-01	SCD5	12	rs3819526	2306783	4.68E-01	CACN/A1C	-	2.09E-07	7.25E-01	1.67E-05	2336	
2	rs4853948	2925053	9.25E-01	N/A	3	rs12492635	3476945	9.36E-01	N/A	-	2.09E-07	N/A	N/A	2337	
5	rs12523655	1.7E+08	5.99E-01	KCNIP1	7	rs6467202	1.28E+08	4.41E-01	FAM71F2	-	2.10E-07	4.74E-01	1.15E-05	2338	
6	rs6907188	1.5E+08	6.86E-01	RAET1K	3	rs6801138	1.73E+08	5.70E-01	PLD1	-	2.10E-07	5.10E-01	3.08E-04	2339	
3	rs12493171	1.1E+08	1.38E-02	MYH15	7	rs2285505	92825381	7.07E-02	CCDC132	-	2.10E-07	2.80E-01	1.71E-03	2340	
3	rs1456722	1.1E+08	1.38E-02	MYH15	7	rs2285505	92825381	7.07E-02	CCDC132	-	2.10E-07	2.80E-01	1.71E-03	2341	
1	rs6701764	2.13E+08	6.27E-01	SMYD2	13	rs11069721	1.08E+08	5.06E-02	N/A	-	2.10E-07	3.92E-01	3.84E-04	2342	
1	rs876761	91552996	8.98E-01	HFM1	2	rs1872787	77844332	5.73E-01	N/A	-	2.10E-07	N/A	N/A	2343	
2	rs6737780	2.39E+08	2.74E-01	TRAF3IP1	10	rs180481	1.2E+08	2.46E-01	N/A	-	2.10E-07	8.69E-01	1.11E-04	2344	
10	rs4545434	64545751	2.93E-01	NRBF2	11	rs584994	1.17E+08	1.36E-02	TMPRSS13	-	2.10E-07	4.87E-01	5.46E-04	2345	
6	rs7453313	9069974	4.28E-01	N/A	20	rs6038729	7085757	3.08E-01	N/A	-	2.10E-07	5.25E-01	2.31E-05	2346	
7	rs736198	13069584	5.91E-02	N/A	19	rs1859955	62514181	6.47E-01	ZNF460	-	2.10E-07	4.47E-01	2.85E-06	2347	
7	rs10255671	1.51E+08	4.48E-01	CRYGN	20	rs6024786	54324078	7.54E-01	N/A	-	2.11E-07	8.72E-02	2.63E-06	2348	
7	rs2214810	26606626	9.64E-01	N/A	12	rs7315384	1.03E+08	7.05E-01	STAB2	-	2.11E-07	2.12E-01	2.63E-03	2349	
2	rs1530941	2.07E+08	7.11E-01	N/A	8	rs1127379	41240437	3.05E-01	SFRP1	-	2.11E-07	3.88E-01	1.61E-04	2350	

SNP A					SNP B						Interaction P			Ranking	Cluster in top 100 interactions
CHR	SNP	Location	gle locus P va	Gene	CHR	SNP	Location	Single locus P valu	Gene	MHC region	Stage 1	Stage 2	Combined		
3	rs716534	1.11E+08	2.91E-01	N/A	16	rs2731766	56828823	4.67E-01	CCDC113	-	2.11E-07	6.95E-01	1.38E-04	2351	
7	rs10255671	1.51E+08	4.48E-01	CRYGN	20	rs4810019	54344324	7.54E-01	N/A	-	2.11E-07	N/A	N/A	2352	
12	rs10506937	85825624	7.99E-01	N/A	16	rs7342694	80461301	1.98E-01	PLCG2	-	2.11E-07	4.55E-01	3.30E-04	2353	
6	rs9347882	1.65E+08	4.33E-01	N/A	1	rs12402711	41651668	5.18E-01	N/A	-	2.11E-07	6.03E-01	3.41E-06	2354	
5	rs1501761	4811455	6.99E-01	N/A	15	rs7178749	59841189	1.69E-01	N/A	-	2.11E-07	8.54E-01	8.74E-05	2355	
2	rs13002864	76580554	8.73E-02	N/A	3	rs1482038	14296181	5.73E-01	N/A	-	2.11E-07	N/A	N/A	2356	
3	rs814753	65758136	2.75E-01	MAGI1	4	rs4695894	1.75E+08	5.74E-01	N/A	-	2.11E-07	4.38E-01	1.68E-03	2357	
6	rs283548	51074433	1.77E-01	N/A	3	rs3755702	1.26E+08	6.67E-02	KALRN	-	2.11E-07	8.89E-01	4.34E-05	2358	
12	rs10506938	85833028	7.43E-01	N/A	16	rs7342694	80461301	1.98E-01	PLCG2	-	2.11E-07	5.26E-01	2.32E-04	2359	
12	rs2897395	85829951	7.43E-01	N/A	16	rs7342694	80461301	1.98E-01	PLCG2	-	2.11E-07	6.07E-01	1.78E-04	2360	
18	rs7238355	32291755	3.85E-01	FHOD3	19	rs7246914	57362717	1.36E-01	ZNF836	-	2.11E-07	9.89E-01	1.04E-04	2361	
3	rs6798734	1.6E+08	7.04E-01	N/A	4	rs7690462	24033252	3.90E-01	N/A	-	2.11E-07	3.88E-01	1.61E-03	2362	
11	rs690847	1.29E+08	2.35E-01	N/A	18	rs280985	4441032	8.03E-01	N/A	-	2.11E-07	4.69E-01	8.98E-04	2363	
5	rs16877062	79034659	4.92E-01	CMYA5	9	rs10867879	71481798	9.44E-01	APBA1	-	2.11E-07	9.91E-01	6.04E-05	2364	
13	rs1415242	33614568	8.36E-01	N/A	18	rs949292	56319787	7.64E-01	N/A	-	2.11E-07	7.44E-01	1.30E-04	2365	
3	rs7622003	1.33E+08	1.52E-01	N/A	4	rs1598859	1.04E+08	8.57E-01	NFKB1	-	2.11E-07	7.83E-01	2.15E-05	2366	
3	rs7622210	1.33E+08	1.52E-01	N/A	4	rs1598859	1.04E+08	8.57E-01	NFKB1	-	2.11E-07	7.83E-01	2.15E-05	2367	
12	rs12426177	1.06E+08	2.40E-02	N/A	17	rs3744345	12831997	8.17E-01	RICH2	-	2.11E-07	4.13E-02	1.13E-07	2368	
3	rs10510896	63330132	9.81E-01	SYNPR	15	rs4779536	29574400	4.24E-01	OTUD7A	-	2.12E-07	7.28E-02	1.01E-07	2369	
6	rs4122189	31275906	7.70E-02	HCG27	6	rs396243	31383153	2.17E-01	N/A	MHC	2.12E-07	5.46E-01	1.21E-04	2370	
1	rs11203366	17530121	9.93E-03	PADI4	13	rs4885001	71857395	7.48E-01	N/A	-	2.12E-07	6.67E-01	1.65E-05	2371	
3	rs1024940	1.92E+08	8.48E-01	IL1RAP	8	rs4871724	1.28E+08	8.85E-03	N/A	-	2.12E-07	8.58E-01	1.20E-04	2372	
5	rs10520846	16954752	1.98E-01	MYO10	19	rs379327	61061978	6.42E-01	NLRP4	-	2.12E-07	N/A	N/A	2373	
7	rs10215210	88206842	9.61E-01	ZNF804B	13	rs609045	29644051	5.64E-01	N/A	-	2.12E-07	8.22E-01	1.21E-05	2374	
4	rs13110363	1.63E+08	2.81E-01	FSTL5	13	rs2274085	1.01E+08	1.30E-01	N/ALCN	-	2.12E-07	6.94E-01	4.98E-05	2375	
4	rs6821696	1.17E+08	2.49E-01	MIR1973	10	rs1380439	1.28E+08	6.89E-01	ADAM12	-	2.12E-07	8.37E-04	8.90E-10	2376	
3	rs12635848	59371840	7.61E-01	N/A	4	rs10033037	1.15E+08	3.38E-02	CAMK2D	-	2.12E-07	4.18E-01	1.44E-05	2377	
3	rs1398776	1.47E+08	8.31E-01	N/A	9	rs4742429	806742	2.20E-01	N/A	-	2.12E-07	2.72E-01	1.31E-03	2378	
6	rs3130534	31317024	3.50E-01	N/A	18	rs2732229	71742290	5.44E-01	N/A	MHC	2.13E-07	9.18E-01	3.71E-05	2379	
1	rs12760232	1.85E+08	8.07E-01	PLA2G4A	15	rs522933	38046796	1.29E-01	EIF2AK4	-	2.13E-07	N/A	N/A	2380	
4	rs4566706	1.89E+08	7.37E-01	N/A	5	rs38067	10037208	2.02E-01	N/A	-	2.13E-07	6.27E-01	2.31E-04	2381	
8	rs1047398	22626963	9.53E-01	PEBP4	11	rs513438	82645611	9.07E-01	ANKRD42	-	2.13E-07	1.06E-01	8.05E-04	2382	
8	rs1047406	22626880	9.53E-01	PEBP4	11	rs513438	82645611	9.07E-01	ANKRD42	-	2.13E-07	1.06E-01	8.05E-04	2383	
10	rs617738	84312795	8.13E-01	NRG3	10	rs7097525	1.23E+08	6.14E-02	N/A	-	2.13E-07	1.55E-01	1.87E-06	2384	
7	rs10280848	67742124	8.09E-01	N/A	10	rs3737275	26350416	3.53E-01	MYO3A	-	2.13E-07	2.35E-01	5.99E-04	2385	
2	rs10497291	1.67E+08	6.91E-01	N/A	22	rs17462966	28463579	9.64E-01	ZMAT5	-	2.13E-07	7.06E-01	2.50E-05	2386	
2	rs2600660	61403104	3.92E-01	USP34	15	rs971804	83345385	4.00E-01	PDE8A	-	2.13E-07	1.57E-01	1.79E-03	2387	
12	rs17604051	40543278	8.84E-01	N/A	22	rs5750511	20785321	7.79E-01	N/A	-	2.13E-07	8.45E-01	9.84E-05	2388	
4	rs1509620	95782995	5.64E-02	PDLIM5	4	rs10489022	1.38E+08	9.82E-01	N/A	-	2.13E-07	9.40E-01	6.39E-05	2389	
7	rs17144110	21246477	4.04E-01	N/A	8	rs755683	18604967	8.77E-01	PSD3	-	2.13E-07	4.54E-01	7.48E-04	2390	
4	rs4690142	81286316	6.24E-02	N/A	20	rs6090443	61624230	7.46E-01	PPDPF	-	2.13E-07	4.98E-01	1.90E-04	2391	
15	rs1424712	92931134	1.27E-02	N/A	16	rs7342694	80461301	1.98E-01	PLCG2	-	2.13E-07	5.65E-01	5.61E-04	2392	
2	rs17016629	35506420	1.82E-01	N/A	7	rs1544733	1.38E+08	5.55E-01	TRIM24	-	2.13E-07	8.06E-01	1.49E-04	2393	
3	rs11128068	89289912	8.95E-02	EPHA3	7	rs10259462	1.3E+08	5.50E-01	FLJ43663	-	2.13E-07	6.30E-01	2.31E-05	2394	
7	rs228587	1.44E+08	7.02E-03	TPK1	21	rs2838906	45646335	6.11E-01	COL18A1	-	2.13E-07	5.40E-01	6.32E-06	2395	
3	rs1447738	1.45E+08	7.27E-01	SLC9A9	8	rs2816489	5849941	8.18E-01	N/A	-	2.13E-07	6.36E-01	1.16E-03	2396	
5	rs4348193	77671476	5.91E-01	N/A	11	rs3741132	73655891	3.98E-01	PPME1	-	2.14E-07	9.07E-01	4.25E-05	2397	
7	rs10215210	88206842	9.61E-01	ZNF804B	13	rs7997640	29642080	5.24E-01	N/A	-	2.14E-07	N/A	N/A	2398	
3	rs2350279	1.41E+08	3.47E-02	CLSTN2	4	rs10517048	42443021	3.91E-01	N/A	-	2.14E-07	1.39E-01	9.46E-07	2399	
3	rs2350279	1.41E+08	3.47E-02	CLSTN2	4	rs2345904	42447945	3.91E-01	N/A	-	2.14E-07	1.39E-01	9.46E-07	2400	
3	rs2350279	1.41E+08	3.47E-02	CLSTN2	4	rs6831756	42446582	3.91E-01	N/A	-	2.14E-07	1.39E-01	9.46E-07	2401	
3	rs2350279	1.41E+08	3.47E-02	CLSTN2	4	rs6850284	42441381	3.91E-01	N/A	-	2.14E-07	1.39E-01	9.46E-07	2402	
3	rs2350279	1.41E+08	3.47E-02	CLSTN2	4	rs7667384	42445273	3.91E-01	N/A	-	2.14E-07	1.39E-01	9.46E-07	2403	
3	rs2350279	1.41E+08	3.47E-02	CLSTN2	4	rs9291222	42445972	3.91E-01	N/A	-	2.14E-07	1.39E-01	9.46E-07	2404	
12	rs10850903	1.17E+08	6.29E-01	KSR2	17	rs3744516	29390113	4.10E-01	ACCN1	-	2.14E-07	9.32E-01	1.33E-05	2405	
1	rs3901950	1.85E+08	4.52E-01	N/A	5	rs7715102	2427305	5.42E-01	N/A	-	2.14E-07	7.24E-01	3.53E-05	2406	
12	rs10128762	72968165	7.81E-01	N/A	21	rs2824444	17985065	2.72E-01	N/A	-	2.14E-07	1.56E-03	3.39E-08	2407	
1	rs12044451	75464916	4.91E-01	SLC44A5	5	rs828718	1.62E+08	5.73E-01	N/A	-	2.14E-07	5.38E-01	1.79E-05	2408	
2	rs6545648	57511448	3.54E-01	N/A	2	rs730402	59948210	2.73E-02	N/A	-	2.14E-07	3.61E-03	2.72E-09	2409	
8	rs2008566	70967766	7.12E-01	N/A	11	rs552007	1.14E+08	4.00E-01	ZBTB16	-	2.14E-07	6.19E-01	7.42E-06	2410	
3	rs165571	13025094	6.28E-01	IQSEC1	14	rs2236184	68140901	8.23E-01	RAD51L1	-	2.14E-07	9.12E-01	2.08E-05	2411	
4	rs4306906	1.77E+08	4.50E-01	GPM6A	11	rs2949861	93451687	4.79E-02	HEPHL1	-	2.15E-07	4.32E-01	9.46E-05	2412	
1	rs11203366	17530121	9.93E-03	PADI4	13	rs486299	71844010	8.94E-01	N/A	-	2.15E-07	7.07E-01	1.10E-04	2413	
3	rs2102801	29176464	5.09E-01	N/A	17	rs11656277	50726046	8.22E-02	HLF	-	2.15E-07	8.36E-01	2.92E-05	2414	
13	rs879681	1.06E+08	2.38E-01	N/A	19	rs1946612	46459827	8.46E-01	HNRNPUL1	-	2.15E-07	3.98E-02	8.44E-03	2415	
6	rs9367511	53118141	1.47E-01	GCM1	3	rs9841414	1.65E+08	7.88E-01	N/A	-	2.15E-07	9.30E-01	1.99E-04	2416	
3	rs7615473	55825018	2.41E-01	ERC2	19	rs1860565	62026834	7.53E-01	PEG3	-	2.15E-07	3.86E-01	3.73E-05	2417	
1	rs12564019	1.58E+08	9.29E-01	OR10J1	3	rs11127905	85793026	2.44E-01	CADM2	-	2.15E-07	1.90E-01	2.77E-06	2418	
1	rs12564019	1.58E+08	9.29E-01	OR10J1	3	rs9860249	85770242	2.44E-01	CADM2	-	2.15E-07	1.90E-01	2.77E-06	2419	
1	rs1832787	18499504	2.02E-01	IGSF21	3	rs1344314	29708376	6.95E-01	N/A	-	2.15E-07	7.14E-01	2.70E-05	2420	
6	rs693612	1.07E+08	8.96E-01	PRDM1	9	rs296663	92749882	6.95E-01	N/A	-	2.15E-07	8.05E-01	2.38E-05	2421	
16	rs1072123	50596711	4.09E-01	N/A	22	rs5755464	33686322	5.89E-01	N/A	-	2.15E-07	9.17E-01	9.60E-05	2422	
6	rs9400997	1.18E+08	9.69E-01	ROS1	10	rs10828819	25879438	3.78E-01	GPR158	-	2.15E-07	3.48E-01	3.58E-06	2423	
1	rs11203368	17539095	1.03E-02	PADI4	13	rs4885001	71857395	7.48E-01	N/A	-	2.15E-07	6.56E-01	7.58E-05	2424	
4	rs10049820	31957285	4.57E-01	N/A	18	rs7232492	3820245	9.57E-01	DLGAP1	-	2.15E-07	3.48E-01	5.19E-04	2425	
6	rs6926443	17204333	5.36E-01	N/A	6	rs9350790	63976392	6.50E-01	N/A	-	2.15E-07	N/A	N/A	2426	
3	rs6792812	26709968	5.62E-01	LRRC3B	5	rs7380062	1.39E+08	2.47E-01	ECSCR	-	2.15E-07	6.64E-01	3.75E-04	2427	
15	rs1424712	92931134	1.27E-02	N/A	16	rs4997772	80462796	1.94E-01	PLCG2	-	2.15E-07	8.07E-01	3.23E-04	2428	
3	rs9814549	1.01E+08	6.90E-01	C3orf26	8	rs2014286	16951496	5.93E-01	EFHA2	-	2.15E-07	9.44E-01	1.13E-05	2429	
4	rs12646565	1.91E+08	4.80E-01	FRG1	10	rs1380459	61767337	1.41E-01	ANK3	-	2.15E-07	6.72E-02	7.10E-07	2430	
3	rs11128564	11654509	3.40E-01	VGLL4	13	rs9543844	74554919	2.24E-01	N/A	-	2.15E-07	1.64E-01	6.93E-04	2431	
4	rs4975057	80707369	7.87E-02	N/A	7	rs9886302	70389420	4.37E-01	WBSCR17	-	2.15E-07	2.93E-01	4.64E-06	2432	
5	rs4360024	77719962	5.37E-01	SCAMP1	11	rs11235995	73484091	3.56E-01	C2CD3	-	2.16E-07	6.47E-01	1.54E-05	2433	
5	rs4360024	77719962	5.37E-01	SCAMP1	11	rs7110734	73530035	3.56E-01	C2CD3	-	2.16E-07	6.98E-01	1.88E-05	2434	
10	rs6537539	50426040	3.66E-01	ERCC6	10	rs1898355	1.19E+08	9.14E-01	N/A	-	2.16E-07	6.98E-01	1.88E-05	2435	
6	rs2233984	31187243	4.20E-02	C6orf15	6	rs1265156	31250276	6.76E-01	TCF19	MHC	2.16E-07	7.36E-01	6.04E-05	2436	
5	rs4360024	77719962	5.37E-01	SCAMP1	11	rs7125032	73484690	3.56E-01	C2CD3	-	2.16E-07	7.83E-01	1.89E-05	2437	
3	rs11926147	54170136	9.40E-01	CACN/A2D3	8	rs2978107	1.02E+08	8.21E-01	N/A	-	2.16E-07	8.33E-02	3.54E-07	2438	
6	rs10456181	55148655	1.14E-01	HCRTR2	7	rs11760565	95582203	6.07E-01	DYNC1I1	-	2.16E-07	9.36E-01	3.69E-04	2439	
2	rs11892554	2.09E+08	2.91E-01	N/A	7	rs6461503	20527521	1.19E-01	N/A	-	2.16E-07	5.83E-01	3.38E-04	2440	
6	rs658376	1.38E+08	6.00E-01	N/A	10	rs7088963	92895662	8.35E-01	NUDT9P1	-	2.16E-07	1.97E-01	5.61E-07	2441	
8	rs559167	96915454	9.48E-01	N/A	13	rs4500590	34979108	7.80E-01	NBEA	-	2.16E-07	4.63E-01	3.47E-05	2442	
5	rs13170704	25044243	4.67E-01	N/A	9	rs4977871	23633682	9.19E-01	N/A	-	2.16E-07	N/A	N/A	2443	
1	rs12564019	1.58E+08	9.29E-01	OR10J1	3	rs12494196	85796570	2.19E-01	CADM2	-	2.16E-07	1.90E-01	2.45E-06	2444	
2	rs4293574	1.61E+08	8.64E-01	N/A	8	rs7010864	58947443	3.33E-02	N/A	-	2.16E-07	3.46E-01	4.96E-04	2445	
14	rs4903794	78272079	4.28E-01	NRXN3	14	rs10484062	98398213	6.47E-02	N/A	-	2.16E-07	4.17E-01	2.96E-06	2446	
6	rs2858331	32789255	1.42E-01	N/A	10	rs7913408	52265121	2.80E-01	A1CF	MHC	2.16E-07	8.89E-01	2.44E-04	2447	
3	rs2600330	12943104	6.20E-01	IQSEC1	3	rs10511409	1.24E+08	7.06E-01	KPN/A1	-	2.17E-07	7.78E-01	2.00E-04	2448	
7	rs10233816	17941651	6.54E-01	SNX13	14	rs2896294	94276900	2.54E-01	N/A	-	2.17E-07	9.05E-01	9.70E-05	2449	
3	rs12638958	99425571	6.69E-01	N/A	16	rs6500343	49448844	5.45E-01	N/A	-	2.17E-07	1.27E-01	2.05E-03	2450	
2	rs7560152	31209932	1.82E-01	GALNT14	7	rs13228939	53674043	6.93E-01	N/A	-	2.17E-07	9.45E-01	4.52E-05	2451	
2	rs2042542	2.18E+08	1.32E-01	TNS1	12	rs1245810	78161526	9.82E-01	SYT1	-	2.17E-07	7.56E-01	9.64E-06	2452	
1	rs10889403	63334538	9.86E-01	N/A	11	rs11041390	7432565	6.89E-01	SYT9	-	2.17E-07	8.70E-01	8.16E-06	2453	
2	rs723504	12348891	3.55E-01	N/A	10	rs2297643	99349977	3.43E-01	DHDPSL	-	2.17E-07	8.98E-01	5.42E-05	2454	
5	rs1366315	67292325	7.30E-01	N/A	5	rs34992	80302822	3.48E-01	RASGRF2	-	2.17E-07	3.44E-01	3.58E-04	2455	
4	rs2522469	1.06E+08	7.41E-02	N/A	13	rs1981058	38731403	7.56E-01	N/A	-	2.17E-07	7.17E-01	2.93E-04	2456	
1	rs6673606	1.81E+08	6.15E-01	LAMC2	11	rs11029672	26657670	5.54E-01	SLC5A12	-	2.17E-07	9.51E-01	1.79E-05	2457	
6	rs9377299	1.02E+08	8.12E-01	GRIK2	17	rs11655926	32317483	1.58E-01	N/A	-	2.17E-07	2.34E-01	5.27E-04	2458	
6	rs1011088	4426420	1.19E-01	N/A	16	rs756813	23198310	4.85E-01	N/A	-	2.17E-07	3.48E-01	5.45E-04	2459	
6	rs4541741	41772010	1.22E-01	TFEB	11	rs4937186	1.26E+08	2.63E-01	KIRREL3	-	2.17E-07	5.52E-01	1.31E-04	2460	
3	rs4054727	3075615	7.99E-01	IL5RA	16	rs322567	62486605	4.63E-01	N/A	-	2.17E-07	7.75E-01	2.71E-06	2461	
5	rs12054892	57981007	6.54E-01	RAB3C	14	rs11621843	92185877	4.54E-01	RIN3	-	2.17E-07	5.93E-01	1.93E-05	2462	

SNP A					SNP B						Interaction P			Ranking	Cluster in top 100 interactions
CHR	SNP	Location	gle locus P va	Gene	CHR	SNP	Location	Single locus P valu	Gene	IHC region	Stage 1	Stage 2	Combined		
5	rs4704506	77690583	5.04E-01	SCAMP1	11	rs1944971	73601066	3.55E-01	PPME1	-	2.17E-07	7.54E-01	2.02E-05	2463	
5	rs10052776	11231648	2.10E-01	CTNND2	5	rs1363902	64475999	8.14E-01	ADAMTS6	-	2.17E-07	N/A	N/A	2464	
7	rs17159305	1.12E+08	4.85E-01	DOCK4	11	rs7127610	84306633	5.16E-01	DLG2	-	2.17E-07	1.36E-01	1.41E-03	2465	
3	rs4530474	1.25E+08	8.94E-01	MYLK	12	rs1635135	1.12E+08	2.15E-01	OAS2	-	2.17E-07	5.05E-01	1.28E-03	2466	
6	rs2233965	31188878	4.20E-03	C6orf15	6	rs3868542	31253818	6.85E-02	TCF19	MHC	2.18E-07	9.53E-01	1.99E-05	2467	
3	rs1528898	1.11E+08	4.65E-01	N/A	10	rs4880885	1632428	1.34E-01	ADARB2	-	2.18E-07	8.82E-02	4.30E-03	2468	
6	rs6899410	87230340	6.28E-01	N/A	1	rs748142	48281640	6.07E-01	N/A	-	2.18E-07	8.82E-02	4.30E-03	2469	
3	rs336564	1.63E+08	7.69E-01	C3orf57	8	rs4977114	1.44E+08	6.95E-01	N/A	-	2.18E-07	1.71E-01	3.12E-06	2470	
3	rs2701069	1.11E+08	4.65E-01	N/A	10	rs4880885	1632428	1.34E-01	ADARB2	-	2.18E-07	1.93E-01	3.93E-06	2471	
9	rs1796993	74505258	4.66E-02	TMC1	17	rs2323661	15241034	1.56E-01	N/A	-	2.18E-07	7.16E-01	3.13E-04	2472	
9	rs2501917	74505943	4.66E-02	TMC1	17	rs2323661	15241034	1.56E-01	N/A	-	2.18E-07	7.16E-01	3.13E-04	2473	
3	rs336592	1.63E+08	7.69E-01	C3orf57	8	rs4977114	1.44E+08	6.95E-01	N/A	-	2.18E-07	7.16E-01	3.13E-04	2474	
9	rs2793150	74509273	4.66E-02	TMC1	17	rs2323661	15241034	1.56E-01	N/A	-	2.18E-07	4.60E-01	1.12E-05	2475	
6	rs12216269	1.38E+08	9.09E-01	N/A	20	rs1884358	12392714	4.44E-01	N/A	-	2.18E-07	2.69E-01	1.31E-05	2476	
1	rs10889272	62175137	8.34E-01	IN/ADL	3	rs7628487	1.3E+08	9.73E-01	N/A	-	2.18E-07	3.95E-01	4.23E-04	2477	
6	rs7772313	55646969	8.81E-03	N/A	14	rs2104152	95087829	2.57E-03	SCARN/A13	-	2.18E-07	6.53E-01	5.28E-04	2478	
8	rs16892035	1.2E+08	3.39E-01	COLEC10	18	rs9646553	47274701	8.69E-01	N/A	-	2.18E-07	7.05E-01	6.36E-05	2479	
8	rs6469809	1.2E+08	3.39E-01	COLEC10	18	rs9646553	47274701	8.69E-01	N/A	-	2.18E-07	7.05E-01	6.36E-05	2480	
6	rs1541227	37609844	1.24E-01	N/A	14	rs8004382	20225233	5.18E-01	RN/ASE4	-	2.18E-07	8.96E-01	3.34E-05	2481	
1	rs9425991	34336673	6.36E-01	CSMD2	3	rs9875900	65246265	5.43E-02	N/A	-	2.18E-07	3.15E-01	1.75E-04	2482	
1	rs9428015	89041928	5.42E-01	PKN2	16	rs12443910	72711976	1.06E-01	N/A	-	2.18E-07	3.29E-01	5.88E-06	2483	
1	rs11579628	54356866	7.39E-01	C1orf83	17	rs7211140	69685505	6.98E-01	N/A	-	2.18E-07	5.05E-01	1.59E-05	2484	
6	rs9485099	1.47E+08	2.81E-02	C6orf103	5	rs2085448	1.68E+08	2.55E-01	WWC1	-	2.18E-07	7.02E-01	1.34E-05	2485	
8	rs4076510	4774854	8.11E-01	CSMD1	13	rs9556587	95877619	1.76E-01	HS6ST3	-	2.18E-07	6.35E-01	1.74E-05	2486	
1	rs11203368	17539095	1.03E-02	PADI4	13	rs486299	71844010	8.94E-01	N/A	-	2.18E-07	6.69E-01	1.26E-04	2487	
9	rs7044405	35860001	1.82E-01	TMEM8B	14	rs977870	22794987	1.31E-01	HOMEZ	-	2.18E-07	N/A	N/A	2488	
1	rs6678619	54534938	7.12E-02	SSBP3	8	rs1993196	78213269	5.77E-01	N/A	-	2.19E-07	8.12E-01	3.00E-05	2489	
3	rs9857526	1.24E+08	7.05E-02	SEC22A	12	rs12425300	59135229	1.89E-01	N/A	-	2.19E-07	6.36E-02	1.37E-03	2490	
3	rs1284718	1.04E+08	2.05E-01	N/A	13	rs9534986	47665333	6.35E-01	N/A	-	2.19E-07	1.58E-01	6.05E-07	2491	
1	rs1112903	2.37E+08	7.02E-01	N/A	14	rs1950927	51148960	9.29E-01	FRMD6	-	2.19E-07	2.86E-01	6.75E-06	2492	
3	rs2289271	1.17E+08	7.32E-01	LSAMP	9	rs10120039	1.26E+08	7.39E-01	NR6A1	-	2.19E-07	5.44E-01	4.50E-04	2493	
1	rs2359899	14175747	6.47E-01	N/A	3	rs723765	78740688	2.82E-01	ROBO1	-	2.19E-07	3.60E-01	2.71E-04	2494	
9	rs1329562	81988930	6.71E-01	N/A	14	rs1002579	51653822	1.58E-01	N/A	-	2.19E-07	2.75E-01	6.79E-04	2495	
6	rs4330570	3927897	4.88E-01	N/A	3	rs1991248	1.08E+08	4.61E-01	LOC344595	-	2.19E-07	8.67E-01	3.03E-05	2496	
13	rs9576292	36998697	4.66E-01	N/A	14	rs11627212	29675166	7.06E-01	N/A	-	2.19E-07	N/A	N/A	2497	
6	rs4947296	31166157	4.31E-02	N/A	6	rs9295957	31265572	2.18E-01	POU5F1	MHC	2.20E-07	2.90E-01	2.53E-06	2498	
4	rs13152512	1.66E+08	9.00E-01	N/A	12	rs10506250	43268257	3.53E-01	NELL2	-	2.20E-07	8.24E-01	3.16E-05	2499	
5	rs4348193	77671476	5.91E-01	N/A	11	rs7117465	73671304	4.00E-01	P4HA3	-	2.20E-07	5.52E-01	7.07E-05	2500	
4	rs10032050	93860117	8.96E-01	GRID2	7	rs10266126	69013326	2.41E-01	AUTS2	-	2.20E-07	3.65E-01	3.01E-06	2501	
4	rs12646171	24688021	2.40E-01	N/A	15	rs6606897	25122055	6.89E-01	GABRG3	-	2.20E-07	1.15E-01	1.08E-03	2502	
3	rs7625947	58458670	4.77E-01	KCTD6	13	rs7990984	66217726	2.89E-01	PCDH9	-	2.20E-07	1.82E-01	2.21E-06	2503	
3	rs10510199	855594	5.53E-01	N/A	20	rs17316539	40353727	6.49E-01	PTPRT	-	2.20E-07	2.53E-01	5.64E-04	2504	
8	rs2898295	11633378	8.50E-01	GATA4	8	rs17620722	16029553	9.43E-01	MSR1	-	2.20E-07	1.35E-01	1.56E-06	2505	
4	rs1491411	1.76E+08	7.69E-01	N/A	8	rs4288398	6869048	3.91E-01	DEFA3	-	2.20E-07	5.89E-01	1.15E-05	2506	
15	rs11247231	99016603	8.43E-01	ASB7	20	rs3818217	18460739	1.59E-01	SEC23B	-	2.20E-07	6.77E-01	1.40E-05	2507	
11	rs328271	1.1E+08	8.44E-01	N/A	21	rs2026220	25436520	7.73E-01	N/A	-	2.20E-07	8.97E-01	7.29E-05	2508	
8	rs1025161	64988980	2.73E-01	N/A	10	rs11202059	81944823	3.91E-01	ANXA11	-	2.20E-07	4.22E-01	1.51E-04	2509	
9	rs1326809	1.19E+08	6.83E-01	ASTN2	17	rs544198	34572555	1.93E-03	PLXDC1	-	2.20E-07	4.33E-01	1.70E-04	2510	
4	rs2130938	1.76E+08	7.69E-01	N/A	8	rs4288398	6869048	3.91E-01	DEFA3	-	2.20E-07	5.05E-01	3.53E-04	2511	
4	rs923677	1.76E+08	7.69E-01	N/A	8	rs4288398	6869048	3.91E-01	DEFA3	-	2.20E-07	5.05E-01	3.53E-04	2512	
6	rs9487771	1.12E+08	8.81E-01	N/A	18	rs684680	11982265	2.34E-01	IMPA2	-	2.20E-07	5.21E-01	3.34E-04	2513	
5	rs37181	1.16E+08	5.66E-01	COMMD10	10	rs512064	84613706	3.18E-01	NRG3	-	2.20E-07	8.56E-01	1.35E-04	2514	
6	rs879882	31247431	7.61E-01	TCF19	6	rs2523454	31475844	1.69E-01	MICA	MHC	2.20E-07	4.84E-03	7.30E-09	2515	
2	rs6726261	25007490	4.51E-01	ADCY3	9	rs11140659	86352181	4.23E-01	N/A	-	2.21E-07	3.85E-03	1.92E-09	2516	
5	rs1370473	1.06E+08	1.05E-01	N/A	9	rs10758314	35584724	1.76E-01	TESK1	-	2.21E-07	5.96E-01	1.35E-05	2517	
1	rs3008204	2.12E+08	3.28E-01	N/A	3	rs2646229	87446270	4.95E-02	N/A	-	2.21E-07	8.70E-01	1.92E-05	2518	
3	rs7630566	1.68E+08	2.22E-01	N/A	8	rs6473482	84902684	5.45E-01	N/A	-	2.21E-07	3.12E-01	2.57E-04	2519	
18	rs11660621	73145738	2.09E-01	N/A	22	rs5755093	33270942	1.36E-02	N/A	-	2.21E-07	3.71E-01	3.76E-05	2520	
2	rs698811	44557180	8.42E-01	C2orf34	3	rs9874152	1.96E+08	8.70E-01	ATP13A3	-	2.21E-07	8.14E-01	6.85E-05	2521	
12	rs4913277	66868439	5.36E-02	IL26	17	rs16974282	65203957	5.52E-01	N/A	-	2.21E-07	8.90E-01	3.08E-05	2522	
4	rs7673099	60869879	3.58E-01	N/A	5	rs3909307	94580009	2.77E-01	MCTP1	-	2.21E-07	6.09E-01	4.56E-06	2523	
9	rs11788425	34323855	8.29E-01	NUDT2	11	rs10833529	21396052	8.17E-01	NELL1	-	2.21E-07	1.26E-01	1.14E-06	2524	
8	rs4428652	56080984	8.06E-01	N/A	17	rs8531	8033419	7.63E-01	TMEM107	-	2.21E-07	6.96E-01	2.39E-05	2525	
15	rs8023252	58916073	3.13E-01	RORA	18	rs9963153	42094062	1.14E-01	C18orf25	-	2.21E-07	7.38E-01	1.92E-04	2526	
6	rs197123	7926670	2.82E-01	MGC26597	9	rs7853173	78796827	6.78E-01	N/A	-	2.21E-07	7.66E-01	3.28E-05	2527	
1	rs6702660	4923290	9.64E-01	N/A	13	rs9547847	36877154	3.09E-01	N/A	-	2.21E-07	3.80E-03	4.75E-02	2528	
6	rs9348687	25700468	5.86E-01	N/A	6	rs2240064	31222552	4.73E-02	TCF19	MHC	2.21E-07	2.11E-01	1.18E-05	2529	
12	rs11610805	96658862	5.35E-01	N/A	16	rs7203055	11381157	2.71E-01	N/A	-	2.22E-07	1.21E-01	8.69E-07	2530	
12	rs11610805	96658862	5.35E-01	N/A	16	rs11864333	11383077	2.71E-01	N/A	-	2.22E-07	1.88E-01	1.47E-03	2531	
6	rs2233984	31187243	4.20E-02	C6orf15	6	rs3130534	31317024	3.50E-01	N/A	MHC	2.22E-07	2.29E-01	1.15E-03	2532	
1	rs12077262	4069908	1.13E-01	N/A	8	rs10104116	62216253	9.12E-01	N/A	-	2.22E-07	2.80E-01	2.85E-06	2533	
18	rs11660621	73145738	2.09E-01	N/A	22	rs135162	33200990	1.44E-02	N/A	-	2.22E-07	3.21E-01	2.72E-05	2534	
2	rs10197232	1.14E+08	9.60E-01	LOC440839	22	rs4823613	44976971	8.51E-01	PPARA	-	2.22E-07	3.51E-01	1.02E-05	2535	
2	rs12711768	1.14E+08	9.60E-01	LOC440839	22	rs4823613	44976971	8.51E-01	PPARA	-	2.22E-07	3.91E-01	1.23E-05	2536	
1	rs11585289	63378189	7.10E-01	N/A	11	rs11041391	7432637	5.65E-01	SYT9	-	2.22E-07	8.22E-01	2.83E-05	2537	
5	rs334485	16582595	6.72E-01	FAM134B	19	rs4804000	51968294	4.75E-01	SLC1A5	-	2.22E-07	6.95E-01	7.46E-06	2538	
2	rs9287545	1.29E+08	4.92E-01	N/A	8	rs11136914	5520796	9.17E-01	N/A	-	2.22E-07	7.77E-01	4.15E-05	2539	
5	rs11750377	1.04E+08	2.36E-01	N/A	21	rs2836746	39203153	6.21E-01	N/A	-	2.22E-07	1.82E-01	1.35E-06	2540	
6	rs4713646	33714501	7.65E-01	ITPR3	3	rs10513628	1.68E+08	1.48E-01	N/A	MHC	2.22E-07	3.30E-01	4.97E-06	2541	
11	rs7121739	1.1E+08	9.85E-01	N/A	14	rs10139051	91949818	5.95E-01	SLC24A4	-	2.23E-07	7.83E-01	8.38E-05	2542	
2	rs4675252	2.03E+08	3.78E-01	FZD7	14	rs1255579	62853747	2.51E-01	GPHB5	-	2.23E-07	7.09E-01	5.22E-05	2543	
9	rs7869846	1767728	3.44E-01	N/A	14	rs1669794	45975802	8.14E-01	N/A	-	2.23E-07	2.72E-01	6.49E-06	2544	
5	rs2089192	1.7E+08	5.56E-01	KCNIP1	7	rs6976331	1.28E+08	4.24E-01	N/A	-	2.23E-07	4.25E-01	8.64E-06	2545	
3	rs9869824	33898549	5.55E-01	PDCD6IP	13	rs2038712	21757616	3.79E-02	N/A	-	2.23E-07	3.10E-01	9.54E-05	2546	
2	rs7601930	1.66E+08	8.04E-01	N/A	10	rs7895521	55526027	3.65E-01	PCDH15	-	2.23E-07	8.82E-01	5.66E-05	2547	
5	rs988364	59247667	2.68E-03	PDE4D	7	rs1544461	81843406	5.62E-01	CACN/A2D1	-	2.23E-07	7.33E-02	2.05E-03	2548	
11	rs10908195	69140597	5.91E-01	N/A	22	rs4821714	36522398	3.20E-02	TRIOBP	-	2.23E-07	7.66E-01	1.18E-04	2549	
7	rs270900	1.37E+08	9.20E-01	DGKI	7	rs12534759	1.41E+08	3.69E-01	AGK	-	2.23E-07	8.47E-01	4.62E-05	2550	
3	rs11715446	74774139	5.22E-02	N/A	13	rs694322	48401261	6.19E-01	N/A	-	2.23E-07	9.49E-01	9.14E-06	2551	
2	rs698809	44556482	9.07E-01	C2orf34	3	rs10933677	1.96E+08	7.25E-01	GP5	-	2.23E-07	9.65E-01	1.21E-04	2552	
4	rs10440412	1.27E+08	5.31E-01	N/A	10	rs10764279	21079396	2.50E-01	N/A	-	2.23E-07	4.74E-02	2.16E-03	2553	
12	rs2673730	1.07E+08	5.03E-01	N/A	17	rs4789997	74799181	1.71E-01	HRNBP3	-	2.23E-07	9.59E-01	6.42E-05	2554	
15	rs11071185	53482122	5.92E-01	CCPG1	20	rs4401268	11893421	4.17E-01	N/A	-	2.23E-07	5.02E-01	5.13E-04	2555	
9	rs1547663	1.19E+08	3.75E-01	ASTN2	17	rs657672	34572591	2.17E-03	PLXDC1	-	2.24E-07	1.27E-01	6.47E-04	2556	
9	rs1547663	1.19E+08	3.75E-01	ASTN2	17	rs657723	34572629	2.17E-03	PLXDC1	-	2.24E-07	1.27E-01	6.47E-04	2557	
4	rs4698434	15577672	6.28E-01	PROM1	9	rs12342805	80536668	6.32E-01	N/A	-	2.24E-07	6.99E-01	1.78E-05	2558	
4	rs9991981	1.3E+08	4.48E-01	PHF17	12	rs10843910	31261896	5.54E-01	N/A	-	2.24E-07	8.91E-01	1.20E-04	2559	
2	rs1859712	1.02E+08	7.93E-01	MAP4K4	16	rs11645360	53631989	1.78E-01	N/A	-	2.24E-07	5.96E-01	1.86E-04	2560	
2	rs897144	2.21E+08	8.86E-01	N/A	9	rs1485184	7569004	7.52E-01	N/A	-	2.24E-07	8.79E-01	1.37E-04	2561	
1	rs11166135	99121424	8.10E-01	LPPR5	13	rs1018595	38893969	8.97E-01	LHFP	-	2.24E-07	6.06E-01	3.70E-04	2562	
5	rs37181	1.16E+08	5.66E-01	COMMD10	10	rs2475793	84629629	5.30E-01	NRG3	-	2.24E-07	1.98E-02	4.32E-08	2563	
1	rs924080	67532728	1.51E-01	IL12RB2	10	rs11007106	28761283	1.83E-01	N/A	-	2.24E-07	7.74E-01	2.10E-05	2564	
6	rs9372627	1.21E+08	6.85E-01	N/A	9	rs2026001	98055916	3.12E-01	HSD17B3	-	2.24E-07	2.05E-01	1.05E-06	2565	
3	rs2131495	1.43E+08	5.28E-01	RASA2	20	rs2224184	11449142	5.72E-01	N/A	-	2.24E-07	1.83E-01	8.16E-07	2566	
1	rs10493673	80887077	7.44E-01	N/A	7	rs6946803	53293659	6.26E-03	N/A	-	2.24E-07	2.14E-01	7.88E-04	2567	
8	rs4355773	26121818	1.03E-01	N/A	10	rs4933453	89448889	4.41E-01	PAPSS2	-	2.25E-07	7.51E-01	2.18E-05	2568	
1	rs10800936	1.88E+08	4.27E-01	FAM5C	7	rs12536500	50775527	8.10E-02	GRB10	-	2.25E-07	1.18E-01	2.92E-03	2569	
14	rs2190631	71657625	8.31E-01	RGS6	19	rs2195310	58337103	7.73E-01	ZNF415	-	2.25E-07	2.59E-01	3.14E-06	2570	
12	rs719948	1.26E+08	1.34E-03	N/A	16	rs12931881	83436787	4.28E-03	CRISPLD2	-	2.25E-07	7.69E-01	1.06E-04	2571	
9	rs2992140	1.17E+08	9.73E-02	1-Dec	20	rs1569940	35962992	8.38E-01	VSTM2L	-	2.25E-07	9.94E-01	8.90E-05	2572	
8	rs2514809	95250422	7.09E-01	CDH17	17	rs2945395	22909096	8.14E-01	KSR1	-	2.25E-07	4.73E-01	5.57E-06	2573	
7	rs2237361	28798822	3.48E-01	CREB5	17	rs1607979	48688258	3.78E-01	N/A	-	2.25E-07	1.60E-03	7.44E-10	2574	

SNP A					SNP B						Interaction P			Ranking	Cluster in top 100 interactions
CHR	SNP	Location	gle locus P va	Gene	CHR	SNP	Location	Single locus P valu	Gene	MHC region	Stage 1	Stage 2	Combined		
2	rs1448561	33707848	9.39E-01	N/A	5	rs2591724	33867772	1.12E-01	ADAMTS12	-	2.25E-07	4.97E-01	4.90E-06	2575	
7	rs13235604	47631897	6.05E-01	N/A	20	rs747912	22581060	8.24E-01	N/A	-	2.25E-07	5.30E-01	2.19E-05	2576	
7	rs1575847	94308081	1.73E-02	N/A	14	rs17096088	58861960	9.40E-02	DAAM1	-	2.25E-07	6.37E-01	6.98E-05	2577	
1	rs1688582	1.02E+08	4.62E-01	N/A	3	rs17624623	61595506	5.50E-01	PTPRG	-	2.25E-07	7.90E-01	4.88E-05	2578	
4	rs1519313	1.87E+08	3.01E-03	N/A	11	rs11233070	81677116	6.36E-02	N/A	-	2.26E-07	3.06E-01	1.60E-04	2579	
13	rs1325360	32625148	9.71E-01	STARD13	16	rs1078151	48875317	1.90E-01	ADCY7	-	2.26E-07	8.00E-01	1.50E-04	2580	
1	rs10920678	1.89E+08	2.97E-01	FAM5C	7	rs12536500	50775527	8.10E-02	GRB10	-	2.26E-07	1.04E-01	3.27E-03	2581	
1	rs10920679	1.89E+08	2.97E-01	FAM5C	7	rs12536500	50775527	8.10E-02	GRB10	-	2.26E-07	1.04E-01	3.27E-03	2582	
7	rs289988	25480258	3.86E-02	N/A	15	rs4072280	96762153	7.64E-01	N/A	-	2.26E-07	8.97E-01	8.53E-05	2583	
1	rs16834573	1.63E+08	9.82E-01	PBX1	5	rs17072586	1.7E+08	2.92E-01	KCNIP1	-	2.26E-07	9.39E-01	3.19E-05	2584	
10	rs7477069	54212144	4.45E-01	MBL2	18	rs11083046	50035017	1.26E-01	POLI	-	2.26E-07	1.56E-01	8.09E-07	2585	
5	rs1025324	35381254	3.56E-01	N/A	10	rs2127355	62204222	8.76E-01	CDK1	-	2.26E-07	4.53E-01	1.01E-03	2586	
1	rs10494739	1.94E+08	7.09E-01	N/A	1	rs12119834	2.04E+08	9.58E-01	CDK18	-	2.26E-07	4.18E-01	1.53E-05	2587	
1	rs9651142	1.01E+08	2.84E-01	N/A	7	rs4283980	1.04E+08	1.62E-01	LHFPL3	-	2.27E-07	5.59E-02	2.37E-03	2588	
18	rs11660621	73145738	2.09E-01	N/A	22	rs130710	33233306	1.49E-02	N/A	-	2.27E-07	3.15E-01	2.73E-05	2589	
2	rs2001202	1.33E+08	1.60E-01	GPR39	11	rs2512568	1.15E+08	5.17E-01	N/A	-	2.27E-07	7.34E-01	7.47E-06	2590	
1	rs4465197	2.36E+08	7.79E-01	RYR2	7	rs11768588	1.55E+08	1.13E-01	N/A	-	2.27E-07	4.50E-01	1.34E-03	2591	
1	rs12022475	80075562	4.18E-02	N/A	10	rs715094	3574566	5.44E-01	N/A	-	2.27E-07	3.01E-01	5.11E-06	2592	
2	rs17041596	51416820	9.03E-01	N/A	10	rs7922042	1.33E+08	6.63E-02	N/A	-	2.27E-07	6.86E-01	1.80E-05	2593	
10	rs9421683	82446303	7.30E-01	N/A	12	rs7316595	92026319	8.23E-02	N/A	-	2.27E-07	2.40E-01	1.54E-03	2594	
6	rs2636597	1.34E+08	1.99E-01	N/A	13	rs9513249	96957705	6.69E-01	N/A	-	2.27E-07	7.78E-01	1.12E-04	2595	
11	rs11025056	19186741	7.22E-01	CSRP3	16	rs4780486	9532033	1.05E-03	N/A	-	2.27E-07	7.86E-01	5.86E-05	2596	
2	rs13018470	59548253	7.71E-01	N/A	11	rs385987	1.31E+08	3.63E-01	NTM	-	2.27E-07	8.87E-01	1.16E-04	2597	
12	rs10849969	1.11E+08	9.52E-01	ACAD10	14	rs10141858	97886923	1.51E-01	N/A	-	2.27E-07	5.32E-01	1.85E-05	2598	
6	rs9296461	45659449	2.80E-01	N/A	4	rs754397	1.2E+08	5.05E-01	SYNPO2	-	2.27E-07	3.44E-01	7.26E-06	2599	
6	rs2233984	31187243	4.20E-02	C6orf15	6	rs3131003	31201461	6.51E-01	C6orf15	MHC	2.27E-07	6.89E-01	1.49E-05	2600	
2	rs12617257	1.43E+08	9.92E-01	LRP1B	15	rs17673100	29838780	1.84E-01	N/A	-	2.28E-07	7.80E-01	1.30E-05	2601	
5	rs6451161	17780897	6.54E-01	N/A	15	rs1392857	51101682	1.87E-02	N/A	-	2.28E-07	6.07E-01	4.34E-04	2602	
15	rs10152718	36270856	9.11E-01	N/A	16	rs11150564	29574237	6.15E-01	SPN	-	2.28E-07	6.35E-01	3.34E-05	2603	
12	rs10859814	93902069	5.59E-01	NDUFA12	15	rs10518996	57164738	4.83E-01	RNF111	-	2.28E-07	N/A	N/A	2604	
12	rs4923674	93910102	5.59E-01	NDUFA12	15	rs10518996	57164738	4.83E-01	RNF111	-	2.28E-07	N/A	N/A	2605	
6	rs4869991	1.52E+08	4.13E-01	N/A	10	rs7085165	91618499	9.82E-01	N/A	-	2.28E-07	8.36E-01	6.81E-05	2606	
7	rs2237361	28798822	3.48E-01	CREB5	9	rs2073577	1.35E+08	8.65E-01	GFI1B	-	2.28E-07	2.37E-01	1.08E-03	2607	
9	rs10760846	1.04E+08	7.20E-01	N/A	13	rs4597197	74915102	4.86E-01	TBC1D4	-	2.28E-07	3.56E-01	7.17E-06	2608	
1	rs1417144	14338672	4.77E-03	N/A	5	rs158842	1.63E+08	4.53E-01	N/A	-	2.28E-07	6.58E-01	3.10E-05	2609	
2	rs12469135	75352306	2.76E-01	N/A	7	rs6947156	52573003	6.72E-01	N/A	-	2.28E-07	3.33E-01	6.97E-04	2610	
6	rs1265110	31227401	8.90E-01	TCF19	6	rs7761965	31381474	9.15E-01	N/A	MHC	2.28E-07	3.56E-01	1.44E-03	2611	
2	rs11902634	2.24E+08	3.79E-02	N/A	5	rs9313889	1.61E+08	7.98E-03	GABRB2	-	2.28E-07	7.07E-01	1.48E-04	2612	
1	rs1417144	14338672	4.77E-03	N/A	5	rs158845	1.63E+08	4.53E-01	N/A	-	2.28E-07	7.07E-01	1.48E-04	2613	
1	rs2024788	61659856	4.03E-01	NFIA	4	rs2531198	10229443	9.18E-01	CLNK	-	2.29E-07	5.66E-01	1.01E-04	2614	
4	rs1713985	57481207	3.75E-01	REST	11	rs4757842	19727630	1.74E-01	N/AV2	-	2.29E-07	7.54E-01	2.03E-05	2615	
9	rs4978964	1.13E+08	1.97E-01	LPAR1	15	rs1002612	99471420	6.89E-01	N/A	-	2.29E-07	8.48E-01	6.12E-05	2616	
2	rs920247	2.4E+08	3.37E-01	N/A	5	rs925203	8381783	4.74E-01	N/A	-	2.29E-07	8.19E-02	3.50E-07	2617	
6	rs6916194	1.36E+08	7.45E-01	PDE7B	2	rs16847122	2.12E+08	8.55E-01	ERBB4	-	2.29E-07	8.46E-01	1.88E-05	2618	
1	rs10919325	1.68E+08	5.16E-01	N/A	11	rs7112378	9038502	5.97E-02	SCUBE2	-	2.30E-07	5.85E-01	1.11E-05	2619	
3	rs9883600	1.46E+08	9.15E-01	N/A	9	rs2793190	1.24E+08	4.28E-01	N/A	-	2.30E-07	4.08E-01	2.29E-06	2620	
5	rs6874186	71512871	4.97E-01	MAP1B	14	rs2277481	23657385	2.73E-01	DCAF11	-	2.30E-07	3.67E-01	3.41E-06	2621	
8	rs10503617	18474753	6.82E-01	PSD3	10	rs4750228	5746028	1.14E-01	ASB13	-	2.30E-07	5.07E-01	1.88E-04	2622	
6	rs4715902	14573278	7.81E-01	N/A	10	rs10786637	1.03E+08	4.49E-01	BTRC	-	2.30E-07	4.59E-01	3.17E-04	2623	
2	rs6750921	85824619	7.42E-01	ATOH8	3	rs921160	63063832	9.79E-01	LOC285401	-	2.30E-07	6.26E-01	3.25E-05	2624	
1	rs10489960	2.19E+08	3.88E-01	N/A	3	rs9862107	1.14E+08	8.40E-01	N/A	-	2.30E-07	8.40E-01	1.06E-04	2625	
8	rs9773092	3203432	2.05E-01	CSMD1	12	rs11180455	73981709	7.99E-01	CAPS2	-	2.30E-07	7.69E-01	7.88E-06	2626	
2	rs5541	40195351	5.93E-01	SLC8A1	22	rs2051489	21092651	2.66E-04	N/A	-	2.30E-07	4.64E-01	2.69E-04	2627	
2	rs5541	40195351	5.93E-01	SLC8A1	22	rs2288090	21093526	2.66E-04	N/A	-	2.30E-07	4.69E-01	2.70E-04	2628	
4	rs1874572	1.47E+08	3.16E-01	N/A	9	rs11145712	79932626	4.80E-01	N/A	-	2.30E-07	3.82E-01	5.20E-06	2629	
4	rs1874572	1.47E+08	3.16E-01	N/A	9	rs12235401	79935893	4.80E-01	N/A	-	2.30E-07	4.25E-01	6.38E-06	2630	
12	rs9783446	86330283	2.25E-01	N/A	22	rs5758913	41478203	8.66E-01	N/A	-	2.30E-07	4.96E-01	2.23E-04	2631	
2	rs10211429	12496630	7.53E-01	N/A	10	rs10763707	29634797	7.87E-01	LYZL1	-	2.30E-07	7.44E-01	8.08E-05	2632	
4	rs6531560	37337010	8.79E-01	RELL1	12	rs34322	12770837	1.24E-01	CDKN1B	-	2.30E-07	7.76E-01	6.18E-05	2633	
7	rs993312	84585780	7.11E-01	SEMA3D	12	rs1976935	52380303	8.48E-01	CALCOCO1	-	2.30E-07	8.89E-01	2.08E-05	2634	
8	rs1912470	14474524	8.69E-01	SGCZ	16	rs8046650	77923674	8.10E-01	N/A	-	2.30E-07	1.01E-01	6.01E-03	2635	
6	rs2844651	31008643	1.58E-01	DPCR1	6	rs1265086	31217861	3.70E-01	TCF19	MHC	2.31E-07	2.60E-01	1.47E-06	2636	
5	rs2247450	79417552	5.34E-01	THBS4	15	rs10520672	86367621	4.16E-01	NTRK3	-	2.31E-07	3.55E-01	1.89E-04	2637	
7	rs1722052	1.1E+08	3.09E-01	N/A	17	rs11651333	11799674	1.96E-02	DN/AH9	-	2.31E-07	4.54E-01	1.83E-06	2638	
4	rs9312212	1.79E+08	6.60E-01	LOC285501	19	rs7249604	58709070	6.35E-01	ZNF813	-	2.31E-07	1.35E-01	3.42E-06	2639	
1	rs17162257	26801589	2.64E-01	N/A	2	rs6751801	2.33E+08	2.27E-02	GIGYF2	-	2.31E-07	1.57E-01	4.32E-06	2640	
11	rs4309121	94230937	2.88E-01	AMOTL1	16	rs6500840	6795938	6.00E-01	A2BP1	-	2.31E-07	2.82E-01	1.67E-03	2641	
3	rs1395407	27955318	6.71E-01	N/A	3	rs11129967	42682285	6.73E-01	KBTBD5	-	2.31E-07	3.10E-01	2.78E-06	2642	
10	rs6584753	1.08E+08	2.05E-01	N/A	15	rs17116917	21465730	3.33E-01	NDN	-	2.31E-07	1.21E-01	1.97E-06	2643	
4	rs9993173	93564163	7.92E-01	GRID2	20	rs1361514	17060688	5.93E-01	N/A	-	2.31E-07	1.86E-01	6.33E-07	2644	
8	rs352774	15692048	8.87E-01	N/A	18	rs2432842	68860542	1.84E-01	N/A	-	2.31E-07	6.07E-01	1.03E-05	2645	
7	rs10235893	1.52E+08	8.93E-01	LOC100128822	12	rs11060321	1.28E+08	1.94E-01	TMEM132D	-	2.31E-07	7.85E-01	2.34E-05	2646	
7	rs6950343	1.51E+08	3.48E-01	PRKAG2	15	rs11631210	29603612	6.47E-01	OTUD7A	-	2.31E-07	2.64E-01	4.07E-04	2647	
14	rs885845	95799481	1.37E-02	BDKRB2	20	rs17217119	52175997	8.36E-01	N/A	-	2.31E-07	5.39E-01	4.70E-06	2648	
2	rs16847914	1.42E+08	2.08E-01	LRP1B	15	rs17673100	29838780	1.84E-01	N/A	-	2.31E-07	8.60E-01	2.64E-05	2649	
11	rs12226062	1.1E+08	7.79E-01	N/A	20	rs6053798	593853	1.67E-01	SCRT2	-	2.32E-07	1.85E-01	3.25E-07	2650	
4	rs6850000	1.67E+08	1.89E-02	CPE	5	rs1650647	80006179	6.89E-01	MSH3	-	2.32E-07	4.69E-01	3.10E-05	2651	
7	rs12673367	48304115	6.37E-02	ABCA13	10	rs11015882	27901946	4.28E-01	N/A	-	2.32E-07	7.51E-01	4.65E-06	2652	
9	rs1341032	74541943	8.79E-03	TMC1	17	rs2323661	15241034	1.56E-01	N/A	-	2.32E-07	4.29E-01	1.83E-03	2653	
5	rs11739635	19883130	4.56E-01	CDH18	19	rs4803892	51238863	4.00E-01	IGFL4	-	2.32E-07	5.27E-01	3.45E-04	2654	
11	rs10831769	12201044	6.12E-01	MICAL2	18	rs2848791	35497911	5.09E-03	LOC647946	-	2.32E-07	9.47E-01	9.13E-06	2655	
9	rs329376	36490619	2.82E-01	N/A	13	rs11620165	1.04E+08	6.59E-02	N/A	-	2.32E-07	N/A	N/A	2656	
9	rs329376	36490619	2.82E-01	N/A	13	rs4772659	1.04E+08	6.59E-02	N/A	-	2.32E-07	N/A	N/A	2657	
10	rs7477069	54212144	4.45E-01	MBL2	18	rs17558111	50217951	1.99E-01	N/A	-	2.32E-07	3.92E-01	1.96E-06	2658	
6	rs11760199	9068727	3.40E-01	N/A	20	rs6038729	7085757	3.08E-01	N/A	-	2.32E-07	5.25E-01	2.49E-05	2659	
4	rs2140945	83862422	9.95E-01	SCD5	12	rs3819526	2306783	4.68E-01	CACN/A1C	-	2.32E-07	9.46E-01	6.23E-05	2660	
10	rs12269161	31612679	9.41E-01	N/A	13	rs10492659	42440382	9.49E-02	EPSTI1	-	2.32E-07	N/A	N/A	2661	
8	rs7017116	10351627	4.86E-01	N/A	11	rs896618	40285758	8.61E-01	LRRC4C	-	2.32E-07	1.05E-01	3.09E-06	2662	
10	rs7477069	54212144	4.45E-01	MBL2	18	rs12604509	50211533	1.52E-01	N/A	-	2.32E-07	2.00E-01	2.03E-06	2663	
10	rs7477069	54212144	4.45E-01	MBL2	18	rs4940331	50189175	1.52E-01	N/A	-	2.32E-07	2.00E-01	2.03E-06	2664	
9	rs10814092	34283063	8.38E-01	KIF24	11	rs10833529	21396052	8.17E-01	NELL1	-	2.33E-07	1.39E-01	1.37E-06	2665	
9	rs10972048	34300927	8.38E-01	NUDT2	11	rs10833529	21396052	8.17E-01	NELL1	-	2.33E-07	1.39E-01	1.37E-06	2666	
3	rs1348994	1.1E+08	2.37E-01	TRAT1	11	rs1397600	7919643	2.50E-01	NLRP10	-	2.33E-07	1.70E-01	8.06E-04	2667	
7	rs11767698	24046485	4.50E-01	N/A	8	rs1714701	4112474	9.67E-01	CSMD1	-	2.33E-07	1.39E-01	2.19E-03	2668	
1	rs678640	2.11E+08	6.05E-01	ATF3	1	rs270500	2.33E+08	8.13E-01	TARBP1	-	2.33E-07	4.70E-01	3.99E-06	2669	
1	rs7519595	1.75E+08	5.81E-02	ASTN1	4	rs6448388	25263664	5.34E-01	N/A	-	2.33E-07	1.45E-01	1.91E-06	2670	
1	rs2331886	1.79E+08	1.08E-01	XPR1	18	rs10502778	38015340	7.16E-01	N/A	-	2.33E-07	1.72E-01	1.59E-06	2671	
3	rs7651713	1.13E+08	9.45E-01	N/A	7	rs10272887	30795140	2.94E-01	FAM188B	-	2.33E-07	5.24E-01	1.54E-05	2672	
2	rs7588391	2.36E+08	9.85E-01	N/A	10	rs10901701	1.28E+08	4.30E-01	N/A	-	2.33E-07	2.91E-01	9.58E-04	2673	
7	rs7808892	31900490	1.25E-01	N/A	9	rs9314841	70823865	9.59E-01	FXN	-	2.33E-07	5.67E-01	1.32E-05	2674	
2	rs4234085	2.09E+08	2.12E-01	N/A	7	rs2214244	20527828	1.05E-01	N/A	-	2.33E-07	6.07E-01	1.74E-04	2675	
7	rs677442	42979035	2.14E-02	N/A	11	rs609036	58210105	8.98E-03	N/A	-	2.33E-07	6.34E-01	2.82E-04	2676	
1	rs1125953	1.96E+08	9.32E-01	N/A	7	rs2299408	1.04E+08	9.14E-01	ORC5L	-	2.33E-07	8.20E-01	2.90E-05	2677	
6	rs6911781	1.14E+08	3.17E-01	N/A	2	rs12328338	1.8E+08	7.62E-01	ZNF385B	-	2.33E-07	N/A	N/A	2678	
1	rs16837337	35655246	8.61E-01	ZMYM4	17	rs4795343	34472851	4.95E-01	PLXDC1	-	2.33E-07	7.35E-01	9.27E-06	2679	
7	rs6460519	68551289	6.83E-01	N/A	13	rs1353523	59215544	8.67E-01	DIAPH3	-	2.33E-07	5.13E-01	1.65E-05	2680	
15	rs4775065	56589235	3.12E-01	LIPC	22	rs2399168	15884945	3.06E-03	CECR7	-	2.33E-07	N/A	N/A	2681	
9	rs7860062	84736837	8.07E-01	N/A	9	rs10991799	91843986	5.47E-01	N/A	-	2.34E-07	8.04E-02	1.36E-06	2682	
2	rs12992690	2.25E+08	3.23E-01	FAM124B	10	rs2418810	1.08E+08	3.10E-02	N/A	-	2.34E-07	6.95E-01	3.70E-05	2683	
2	rs6746998	2.09E+08	1.89E-01	N/A	7	rs916790	20539225	9.56E-02	N/A	-	2.34E-07	3.75E-01	3.65E-04	2684	
1	rs10493817	88899475	6.20E-01	N/A	16	rs12443910	72711976	1.06E-01	N/A	-	2.34E-07	7.77E-01	3.83E-05	2685	
2	rs11889825	2.39E+08	4.38E-01	N/A	5	rs4920799	84642284	5.62E-01	N/A	-	2.34E-07	8.02E-01	3.84E-05	2686	

SNP A					SNP B						Interaction P			Ranking	Cluster in top 100 interactions
CHR	SNP	Location	gle locus P va	Gene	CHR	SNP	Location	ingle locus P valu	Gene	HC region	Stage 1	Stage 2	Combined		
2	rs788172	1.73E+08	4.78E-01	MAP1D	7	s11769398	47840222	3.10E-01	PKD1L1	-	2.34E-07	9.85E-01	1.38E-04	2687	
2	rs2373000	37446132	6.54E-01	QPCT	4	rs896577	75967145	2.26E-01	N/A	-	2.34E-07	5.72E-01	4.60E-05	2688	
13	rs953905	73891001	4.40E-01	N/A	18	rs1373179	48098425	4.96E-01	N/A	-	2.34E-07	7.89E-01	2.85E-05	2689	
4	rs1519313	1.87E+08	3.01E-03	N/A	22	rs882432	24677844	3.56E-02	MYO18B	-	2.34E-07	1.94E-01	6.99E-04	2690	
8	rs7818942	37026803	6.65E-01	N/A	12	rs2701129	50715744	4.59E-01	GRASP	-	2.34E-07	8.63E-01	3.85E-05	2691	
7	rs2079460	14195474	4.10E-01	DGKB	16	rs8057015	76717963	8.42E-01	WWOX	-	2.35E-07	1.42E-02	2.89E-08	2692	
4	rs7656930	1.47E+08	5.35E-01	N/A	7	s10248926	69995265	1.12E-01	N/A	-	2.35E-07	6.18E-01	9.95E-06	2693	
6	rs197123	7926670	2.82E-01	MGC26597	9	rs1929538	78793838	6.80E-01	N/A	-	2.35E-07	7.66E-01	3.30E-05	2694	
1	rs7418365	9457890	1.94E-01	N/A	3	rs822793	22978054	1.42E-01	N/A	-	2.35E-07	3.03E-01	2.49E-06	2695	
17	rs12149977	74854205	8.26E-01	HRNBP3	18	rs8091916	3182682	6.18E-02	MYOM1	-	2.35E-07	5.77E-01	4.75E-04	2696	
4	rs7677258	1.89E+08	4.00E-01	N/A	20	rs6021796	50280251	3.36E-01	N/A	-	2.35E-07	6.64E-02	2.11E-03	2697	
6	rs1877771	1.63E+08	9.76E-01	PACRG	8	s13259780	78161214	2.33E-01	N/A	-	2.35E-07	4.55E-01	8.96E-06	2698	
3	rs6793085	3117423	4.20E-01	IL5RA	20	s16998899	17209305	4.47E-01	PCSK2	-	2.35E-07	2.88E-01	7.53E-06	2699	
4	rs2202507	1.45E+08	5.74E-01	N/A	12	s10860784	1.01E+08	6.45E-01	GNPTAB	-	2.35E-07	7.40E-01	1.33E-05	2700	
2	rs11691660	85245727	6.78E-01	TCF7L1	7	s10951853	5249937	9.05E-01	WIPI2	-	2.35E-07	8.51E-01	1.83E-05	2701	
1	rs4844484	2.08E+08	4.98E-01	LAMB3	9	s12004142	22856134	4.20E-01	N/A	-	2.36E-07	5.27E-01	1.27E-05	2702	
2	rs6714495	23691653	2.48E-01	KLHL29	10	s10885995	1.18E+08	2.64E-02	PNLIPRP2	-	2.36E-07	3.91E-01	4.11E-04	2703	
9	rs11788110	1.16E+08	1.06E-01	N/A	16	rs4260044	83829745	1.48E-01	N/A	-	2.36E-07	3.98E-01	8.99E-04	2704	
2	rs4852275	72014973	1.08E-01	N/A	19	s10419133	61856128	2.24E-01	ZNF835	-	2.36E-07	6.77E-01	1.83E-04	2705	
3	rs6792170	56933398	1.80E-01	ARHGEF3	14	rs4982254	34825488	5.72E-01	KIAA0391	-	2.36E-07	2.66E-01	5.08E-04	2706	
5	rs3822476	77749577	4.95E-01	SCAMP1	11	s11236029	73555872	3.42E-01	C2CD3	-	2.36E-07	7.02E-01	2.13E-05	2707	
10	rs6537539	50426040	3.66E-01	ERCC6	10	s10886031	1.19E+08	8.25E-01	N/A	-	2.36E-07	7.20E-01	7.37E-05	2708	
13	rs184249	25408069	4.36E-01	ATP8A2	13	rs9529874	70831653	2.82E-01	N/A	-	2.36E-07	7.32E-02	6.01E-07	2709	
2	rs4667823	1.66E+08	8.28E-01	N/A	10	rs7895521	55526027	3.65E-01	PCDH15	-	2.36E-07	8.82E-01	5.81E-05	2710	
5	rs10875641	1.48E+08	3.41E-01	N/A	18	s10514157	70994152	7.49E-01	N/A	-	2.36E-07	3.81E-01	1.52E-06	2711	
2	rs4580409	3121507	2.94E-01	N/A	3	rs9850126	7962521	1.42E-01	N/A	-	2.36E-07	N/A	N/A	2712	
1	rs4920502	18687921	3.22E-01	KLHDC7A	12	s10845458	12084254	9.73E-01	N/A	-	2.36E-07	7.05E-01	2.06E-05	2713	
1	rs4652781	1.81E+08	5.30E-01	LAMC2	11	s11029672	26657670	5.54E-01	SLC5A12	-	2.36E-07	4.48E-01	3.63E-06	2714	
8	rs7001238	70081936	8.32E-01	N/A	13	rs985740	72743821	2.58E-01	N/A	-	2.37E-07	3.26E-01	2.98E-06	2715	
9	rs974484	78912590	7.68E-01	N/A	14	rs7149528	31834970	3.31E-01	N/A	-	2.37E-07	7.75E-01	1.61E-05	2716	
1	rs1321776	1.76E+08	2.94E-01	N/A	7	s10228842	11931877	8.76E-01	N/A	-	2.37E-07	8.84E-01	1.23E-04	2717	
3	rs11713033	1.59E+08	7.34E-01	N/A	5	rs6450490	50334219	4.55E-01	N/A	-	2.37E-07	9.93E-02	1.59E-07	2718	
4	rs17005223	1.41E+08	5.71E-01	MAML3	15	s12904449	92616974	6.77E-01	N/A	-	2.37E-07	2.03E-01	5.62E-04	2719	
9	rs10819907	97938109	8.46E-01	N/A	12	s10778374	1.04E+08	5.50E-01	N/A	-	2.37E-07	9.96E-01	1.04E-04	2720	
10	rs1983907	14302025	9.76E-01	FRMD4A	13	rs750989	1.12E+08	6.09E-01	C13orf28	-	2.37E-07	2.77E-01	5.05E-07	2721	
2	rs6714466	2.25E+08	5.02E-01	DOCK10	10	rs7895918	43712074	1.50E-02	N/A	-	2.37E-07	7.32E-01	1.08E-05	2722	
2	rs7558808	2.26E+08	5.02E-01	DOCK10	10	rs7895918	43712074	1.50E-02	N/A	-	2.37E-07	7.32E-01	1.08E-05	2723	
11	rs475688	64120867	8.12E-01	NRXN2	12	s10785513	43278997	1.63E-01	NELL2	-	2.37E-07	1.31E-01	1.03E-03	2724	
6	rs12196606	1.41E+08	2.80E-01	N/A	18	rs559687	5970877	3.36E-01	L3MBTL4	-	2.37E-07	5.11E-01	3.29E-06	2725	
4	rs6812381	1.89E+08	4.06E-01	N/A	20	rs6021796	50280251	3.36E-01	N/A	-	2.37E-07	6.64E-02	2.14E-03	2726	
3	rs6442565	15993474	4.83E-01	N/A	4	rs1395652	60231878	6.00E-01	N/A	-	2.37E-07	2.08E-01	4.25E-07	2727	
5	rs2974491	1.13E+08	1.20E-01	N/A	9	rs7022165	1.15E+08	7.24E-01	C9orf110	-	2.37E-07	2.58E-01	5.21E-06	2728	
6	rs9368675	31380136	7.91E-01	N/A	6	rs2394990	31448538	6.80E-02	HLA-B	MHC	2.37E-07	1.26E-01	1.64E-03	2729	
6	rs9360723	74784936	3.52E-01	N/A	2	rs3754930	1.83E+08	5.20E-01	PPP1R1C	-	2.38E-07	9.40E-01	5.10E-05	2730	
6	rs4706565	74785439	3.52E-01	N/A	2	rs3754930	1.83E+08	5.20E-01	PPP1R1C	-	2.38E-07	9.80E-01	4.38E-05	2731	
10	rs1327014	1.3E+08	7.85E-01	N/A	20	rs399083	187688	9.87E-01	C20orf96	-	2.38E-07	4.01E-01	2.56E-06	2732	
5	rs2545581	1.02E+08	5.91E-01	N/A	10	rs7907048	80357575	4.83E-01	LOC283050	-	2.38E-07	9.71E-01	1.00E-04	2733	
2	rs13393408	75380569	6.27E-01	N/A	10	rs953920	1.18E+08	1.93E-01	GFRA1	-	2.38E-07	6.94E-01	7.17E-05	2734	
16	rs11862729	14053599	8.99E-01	MKL2	19	rs7250902	768708	8.58E-01	N/A	-	2.38E-07	6.97E-01	1.43E-04	2735	
7	rs7791689	31532741	7.47E-01	CCDC129	7	rs675242	47556332	3.71E-01	TNS3	-	2.39E-07	1.37E-01	3.03E-03	2736	
14	rs1884008	85054657	7.87E-01	FLRT2	15	rs4561414	93973328	1.09E-05	N/A	-	2.39E-07	6.43E-01	5.55E-05	2737	
12	rs11610805	96658862	5.35E-01	N/A	16	rs8056559	11377567	3.22E-01	N/A	-	2.39E-07	1.62E-01	2.04E-03	2738	
7	rs9654994	3583940	1.03E-01	SDK1	20	s11086468	36229826	8.53E-01	TGM2	-	2.39E-07	8.73E-02	1.04E-07	2739	
7	rs9654994	3583940	1.03E-01	SDK1	20	rs761002	36230414	8.53E-01	TGM2	-	2.39E-07	8.73E-02	1.04E-07	2740	
6	rs4960136	5733000	2.41E-01	FARS2	2	rs6715170	42941808	4.89E-01	N/A	-	2.39E-07	2.77E-01	5.09E-06	2741	
8	rs9314532	4401471	9.20E-01	CSMD1	18	rs7240702	44391897	5.86E-01	KIAA0427	-	2.39E-07	6.60E-01	9.78E-05	2742	
3	rs2222255	96608609	7.35E-01	N/A	4	rs7690164	1.12E+08	9.22E-01	N/A	-	2.39E-07	5.50E-01	4.19E-04	2743	
6	rs4709802	1.64E+08	9.49E-01	N/A	10	rs4880241	1.34E+08	6.94E-01	JAKMIP3	-	2.39E-07	N/A	N/A	2744	
9	rs4978050	26139006	4.68E-01	N/A	9	rs1030828	79595252	9.48E-01	GN/AQ	-	2.39E-07	4.70E-01	3.41E-04	2745	
6	rs2789588	73200418	8.84E-01	N/A	20	rs6040155	1079119	1.41E-01	PSMF1	-	2.39E-07	9.70E-01	1.04E-04	2746	
1	rs17014199	1.08E+08	1.42E-01	NTNG1	16	s16962919	13808838	9.95E-01	N/A	-	2.39E-07	1.08E-01	9.73E-08	2747	
3	rs2276805	1.74E+08	5.87E-01	NCEH1	18	rs1557320	22365762	4.83E-01	KCTD1	-	2.39E-07	1.41E-01	2.03E-06	2748	
6	rs1361429	1.65E+08	8.00E-01	N/A	2	s12618269	34017690	2.17E-01	N/A	-	2.39E-07	6.85E-01	7.77E-06	2749	
3	rs717478	21240113	4.43E-01	N/A	7	rs1917939	96554800	3.11E-01	N/A	-	2.39E-07	8.65E-02	5.19E-07	2750	
4	rs2114148	1.38E+08	2.87E-01	N/A	10	s10829413	1.3E+08	9.02E-01	N/A	-	2.39E-07	5.83E-01	1.99E-05	2751	
6	rs1265156	31250276	6.76E-01	TCF19	6	rs7761965	31381474	9.15E-01	N/A	MHC	2.39E-07	7.40E-02	1.41E-02	2752	
9	rs1536510	1.26E+08	1.81E-01	N/A	13	rs7324647	1.03E+08	1.29E-01	N/A	-	2.39E-07	2.94E-01	9.36E-07	2753	
4	rs1032818	1.21E+08	7.26E-01	N/A	20	rs6094783	45755083	7.93E-01	SULF2	-	2.40E-07	1.35E-01	5.39E-07	2754	
1	rs3008204	2.12E+08	3.28E-01	N/A	3	rs9822911	87401448	4.90E-02	CHMP2B	-	2.40E-07	7.63E-01	1.24E-05	2755	
2	rs3796058	1.73E+08	9.99E-01	MAP1D	3	rs168587	1.57E+08	3.89E-01	C3orf33	-	2.40E-07	6.11E-01	1.57E-05	2756	
4	rs10939459	12471574	3.89E-01	N/A	10	rs2503872	43113631	8.14E-01	N/A	-	2.40E-07	1.88E-01	4.50E-04	2757	
1	rs12042641	1.65E+08	5.58E-01	GPA33	12	rs2669013	75547079	6.30E-01	N/A	-	2.40E-07	N/A	N/A	2758	
6	rs9487771	1.12E+08	8.81E-01	N/A	18	rs638063	11980505	3.37E-01	IMPA2	-	2.40E-07	4.28E-01	1.78E-04	2759	
3	rs11131064	7189945	5.95E-01	GRM7	20	rs6020997	49179732	1.30E-01	N/A	-	2.40E-07	9.71E-01	8.72E-05	2760	
4	rs1384725	64898070	5.08E-01	TECRL	7	s10950503	2006120	4.02E-01	MAD1L1	-	2.40E-07	5.01E-01	1.28E-03	2761	
3	rs7625947	58458670	4.77E-01	KCTD6	13	rs9540880	66204784	2.64E-01	PCDH9	-	2.41E-07	2.14E-01	6.93E-04	2762	
1	rs10489227	1.76E+08	2.67E-01	N/A	7	s10228842	11931877	8.76E-01	N/A	-	2.41E-07	8.84E-01	1.26E-04	2763	
1	rs12038173	1.76E+08	2.67E-01	N/A	7	s10228842	11931877	8.76E-01	N/A	-	2.41E-07	8.84E-01	1.26E-04	2764	
1	rs1885026	1.76E+08	2.67E-01	N/A	7	s10228842	11931877	8.76E-01	N/A	-	2.41E-07	8.84E-01	1.26E-04	2765	
3	rs9637471	59978630	3.65E-01	FHIT	7	s10224672	47698428	5.42E-02	N/A	-	2.41E-07	5.56E-01	3.59E-04	2766	
3	rs9637471	59978630	3.65E-01	FHIT	7	rs4720608	47696036	5.42E-02	N/A	-	2.41E-07	5.56E-01	3.59E-04	2767	
4	rs10517643	1.58E+08	4.54E-02	N/A	7	s12707053	1.32E+08	3.25E-01	N/A	-	2.41E-07	6.57E-01	2.44E-04	2768	
6	rs1001965	1.5E+08	3.44E-01	N/A	9	rs1668978	25342327	1.10E-01	N/A	-	2.41E-07	8.86E-01	1.63E-04	2769	
3	rs2200392	1.57E+08	1.72E-01	N/A	3	rs9882745	1.91E+08	8.23E-01	N/A	-	2.41E-07	9.76E-01	1.53E-04	2770	
3	rs9845807	1.14E+08	2.80E-01	N/A	19	s12462307	63476544	3.48E-01	ZNF544	-	2.41E-07	8.85E-01	2.09E-05	2771	
6	rs13203975	1.52E+08	4.88E-01	ESR1	3	rs2568911	1.96E+08	3.66E-01	N/A	-	2.41E-07	N/A	N/A	2772	
6	rs4235996	19576099	4.19E-01	N/A	1	rs6695659	1.98E+08	1.25E-01	N/A	-	2.41E-07	3.46E-01	2.66E-05	2773	
8	rs6985259	84374122	2.17E-01	N/A	18	rs8096215	63817997	8.49E-01	N/A	-	2.41E-07	N/A	N/A	2774	
5	rs6893628	1828600	2.84E-01	N/A	8	rs2467799	80874618	1.56E-01	N/A	-	2.42E-07	7.13E-01	6.19E-05	2775	
17	rs10208	39655804	4.20E-01	UBTF	19	rs483808	7863481	2.21E-01	N/A	-	2.42E-07	7.75E-01	4.57E-05	2776	
2	rs10931519	1.93E+08	5.31E-01	TMEFF2	4	rs1459413	66716398	6.61E-01	N/A	-	2.42E-07	4.37E-01	7.55E-06	2777	
3	rs9824299	1.81E+08	8.01E-01	PEX5L	5	rs1384449	44412817	4.80E-01	FGF10	-	2.42E-07	9.19E-01	8.92E-05	2778	
3	rs9824299	1.81E+08	8.01E-01	PEX5L	5	rs2973644	44419940	4.80E-01	FGF10	-	2.42E-07	9.19E-01	8.92E-05	2779	
2	rs10178695	1.33E+08	1.61E-01	GPR39	11	s11215687	1.15E+08	5.15E-01	N/A	-	2.42E-07	9.45E-01	1.29E-05	2780	
7	rs10215210	88206842	9.61E-01	ZNF804B	13	rs9508667	29645950	7.87E-01	N/A	-	2.42E-07	N/A	N/A	2781	
6	rs208704	1.49E+08	7.40E-01	N/A	12	s10431270	27806212	6.37E-01	LOC100133893	-	2.42E-07	2.35E-01	5.30E-04	2782	
1	rs17808157	2.31E+08	2.08E-01	N/A	2	rs935480	1.35E+08	8.20E-01	MGAT5	-	2.42E-07	4.68E-01	6.37E-05	2783	
6	rs9377299	1.02E+08	8.12E-01	GRIK2	17	s11652230	32317613	1.05E-01	N/A	-	2.42E-07	1.92E-01	9.88E-04	2784	
1	rs1321117	30400206	5.29E-01	N/A	13	rs9594987	43128994	5.58E-01	ENOX1	-	2.42E-07	2.03E-01	3.22E-04	2785	
12	rs550434	71157865	4.27E-01	TRHDE	16	rs2046532	55840586	1.11E-01	RSPRY1	-	2.42E-07	5.95E-01	1.85E-05	2786	
3	rs892761	1.66E+08	1.01E-01	N/A	17	rs1867295	50109062	8.79E-01	N/A	-	2.42E-07	7.89E-02	2.33E-03	2787	
14	rs4900026	89698387	4.65E-01	KCNK13	20	rs2276483	9399032	7.31E-01	N/A	-	2.42E-07	5.10E-01	1.08E-03	2788	
14	rs4900026	89698387	4.65E-01	KCNK13	20	rs2276484	9399327	7.31E-01	N/A	-	2.42E-07	5.10E-01	1.08E-03	2789	
12	rs11066038	1.11E+08	9.30E-01	C12orf47	14	s10141858	97886923	1.51E-01	N/A	-	2.42E-07	3.11E-01	5.92E-06	2790	
4	rs1452402	1.1E+08	1.85E-01	N/A	8	rs7845649	87942336	3.53E-01	CNBD1	-	2.42E-07	5.16E-01	1.73E-05	2791	
2	rs2369271	2.32E+08	1.16E-02	N/A	10	rs7895297	69004205	9.86E-02	CTNN/A3	-	2.43E-07	1.97E-01	1.01E-06	2792	
4	rs13131680	8072492	4.84E-01	ABLIM2	11	s10832368	15132561	3.47E-01	INSC	-	2.43E-07	2.88E-01	4.48E-06	2793	
1	rs17538183	38631451	3.05E-02	N/A	2	rs1400131	1.84E+08	2.94E-01	NUP35	-	2.43E-07	4.87E-01	7.39E-05	2794	
6	rs4896224	1.37E+08	2.28E-01	NHEG1	1	rs3767392	2.01E+08	6.53E-01	PPP1R12B	-	2.43E-07	8.46E-01	2.62E-05	2795	
7	rs10255671	1.51E+08	4.48E-01	CRYGN	20	rs1886956	54313803	5.96E-01	N/A	-	2.43E-07	4.04E-01	2.70E-05	2796	
2	rs2861680	79006430	1.64E-01	N/A	11	rs6591324	55941921	3.16E-01	OR8U8	-	2.43E-07	5.97E-01	1.44E-04	2797	
1	rs1325258	34250168	8.71E-01	CSMD2	2	rs2888114	35097562	1.97E-01	N/A	-	2.43E-07	9.90E-01	1.00E-04	2798	

SNP A					SNP B						Interaction P			Ranking	Cluster in top 100 interactions
CHR	SNP	Location	gle locus P va	Gene	CHR	SNP	Location	Single locus P valu	Gene	MHC region	Stage 1	Stage 2	Combined		
7	rs6461617	21912212	6.34E-01	DN/AH11	7	rs7787227	43189520	2.66E-01	HECW1	-	2.43E-07	3.67E-01	4.72E-06	2799	
4	rs4975057	80707369	7.87E-02	N/A	7	s10272997	70402452	5.44E-01	WBSCR17	-	2.43E-07	1.67E-01	1.98E-06	2800	
15	rs1520941	27440581	5.01E-02	FAM189A1	21	rs456874	27761403	7.31E-02	N/A	-	2.43E-07	9.95E-01	9.33E-05	2801	
3	rs12490397	21451300	5.76E-01	ZNF385D	5	rs367842	19588755	3.69E-01	CDH18	-	2.43E-07	1.80E-01	5.22E-06	2802	
3	rs12630629	28973345	1.64E-01	N/A	15	rs4842935	81431271	5.68E-01	HOMER2	-	2.43E-07	2.54E-01	1.12E-03	2803	
2	rs11563194	2.35E+08	5.74E-01	SPP2	16	rs1861532	53235014	1.44E-01	N/A	-	2.43E-07	6.26E-01	9.67E-05	2804	
2	rs17865706	2.35E+08	5.74E-01	SPP2	16	rs1861532	53235014	1.44E-01	N/A	-	2.43E-07	6.26E-01	9.67E-05	2805	
2	rs9636246	2.35E+08	5.74E-01	SPP2	16	rs1861532	53235014	1.44E-01	N/A	-	2.43E-07	7.13E-01	7.56E-05	2806	
5	rs378228	94353608	9.58E-01	MCTP1	9	rs2472384	1.07E+08	4.64E-01	ABCA1	-	2.43E-07	6.39E-01	1.33E-04	2807	
6	rs9321202	1.3E+08	6.31E-02	N/A	14	rs1397163	91173949	1.20E-01	CATSPERB	-	2.44E-07	8.88E-02	1.41E-06	2808	
1	rs6683585	2.39E+08	4.30E-01	RGS7	14	rs4906067	1.01E+08	4.73E-01	N/A	-	2.44E-07	7.93E-01	1.32E-04	2809	
3	rs1485272	3702589	9.14E-01	N/A	7	rs313829	65189932	5.53E-01	ASL	-	2.44E-07	5.82E-01	2.61E-04	2810	
5	rs6451438	39673322	1.26E-01	N/A	9	rs1856197	33688114	5.73E-01	N/A	-	2.44E-07	7.08E-02	2.08E-03	2811	
8	rs3940483	62165144	6.29E-01	N/A	12	s11045790	21195636	9.12E-02	SLCO1B1	-	2.45E-07	7.89E-01	3.50E-05	2812	
17	rs231485	39337538	4.93E-01	MPP2	18	rs4891904	66962989	9.00E-01	N/A	-	2.45E-07	3.91E-02	7.47E-08	2813	
9	rs4130590	1.29E+08	9.17E-01	GARNL3	10	rs1536492	7702849	3.08E-01	ITIH5	-	2.45E-07	9.27E-01	3.25E-05	2814	
7	rs17166711	13075823	7.09E-02	N/A	19	s10411733	62482800	4.72E-01	ZNF460	-	2.45E-07	8.24E-01	1.13E-05	2815	
7	rs12533128	13071663	7.09E-02	N/A	19	s10411733	62482800	4.72E-01	ZNF460	-	2.45E-07	8.98E-01	1.63E-05	2816	
7	rs7778887	13071534	7.09E-02	N/A	19	s10411733	62482800	4.72E-01	ZNF460	-	2.45E-07	8.98E-01	1.63E-05	2817	
8	rs17079568	3037517	4.46E-01	CSMD1	17	rs2233362	44642066	3.62E-02	ABI3	-	2.45E-07	2.14E-01	1.97E-03	2818	
10	rs2420724	1.22E+08	1.21E-02	PPAPDC1A	11	s11228700	56175691	2.47E-01	OR8U8	-	2.45E-07	9.75E-01	1.01E-04	2819	
7	rs12056134	1.27E+08	9.39E-02	N/A	8	rs2575735	97603827	1.89E-01	SDC2	-	2.46E-07	5.24E-01	1.83E-05	2820	
3	rs2130369	1.01E+08	7.50E-01	C3orf26	8	rs7812526	16933751	6.37E-01	EFHA2	-	2.46E-07	7.35E-01	5.06E-06	2821	
11	rs1996352	1.02E+08	4.37E-01	MMP7	20	rs6042778	14451743	5.18E-01	MACROD2	-	2.46E-07	3.97E-01	1.68E-06	2822	
4	rs12233914	19906678	2.98E-01	SLIT2	14	rs4903435	76131256	2.15E-02	N/A	-	2.46E-07	7.60E-01	2.41E-06	2823	
3	rs12636001	55839545	1.06E-01	ERC2	19	rs1860565	62026834	7.53E-01	PEG3	-	2.46E-07	7.77E-01	7.04E-05	2824	
2	rs1476698	2.42E+08	7.09E-01	FARP2	15	s17659026	51120707	5.54E-02	N/A	-	2.46E-07	3.98E-03	1.34E-02	2825	
1	rs6540817	2.13E+08	6.69E-01	SMYD2	13	s10492664	1.08E+08	6.04E-02	N/A	-	2.46E-07	5.18E-01	2.16E-04	2826	
1	rs6540819	2.13E+08	6.69E-01	SMYD2	13	s10492664	1.08E+08	6.04E-02	N/A	-	2.46E-07	5.18E-01	2.16E-04	2827	
3	rs6438537	1.21E+08	4.07E-01	CD80	14	rs1885754	92238463	4.61E-01	RIN3	-	2.46E-07	5.15E-01	4.08E-06	2828	
15	rs8037033	31251826	2.94E-01	N/A	19	rs2292455	1477613	6.42E-01	ADAMTSL5	-	2.46E-07	6.71E-01	1.24E-05	2829	
9	rs4977868	23613958	1.30E-03	N/A	13	rs2329151	79498340	8.29E-01	N/A	-	2.46E-07	4.69E-02	3.49E-03	2830	
5	rs10037741	1.06E+08	9.07E-02	N/A	12	s12824017	61935103	2.85E-01	N/A	-	2.46E-07	N/A	N/A	2831	
8	rs4876860	1.2E+08	2.21E-01	N/A	13	rs3014939	45068610	1.62E-02	FAM194B	-	2.46E-07	8.23E-02	2.87E-03	2832	
8	rs4876860	1.2E+08	2.21E-01	N/A	13	rs3014940	45070829	1.62E-02	FAM194B	-	2.46E-07	8.23E-02	2.87E-03	2833	
9	rs10116050	79252094	6.46E-02	GN/A14	12	rs2162028	27647755	1.40E-01	PPFIBP1	-	2.46E-07	9.65E-02	1.73E-06	2834	
1	rs4078315	2.17E+08	1.56E-01	N/A	20	rs989804	7901919	9.14E-01	TMX4	-	2.47E-07	3.59E-01	8.54E-04	2835	
5	rs170519	27412439	4.74E-02	N/A	18	rs961560	71821808	2.36E-01	N/A	-	2.47E-07	3.31E-01	1.78E-03	2836	
5	rs2434676	27424316	4.74E-02	N/A	18	rs961560	71821808	2.36E-01	N/A	-	2.47E-07	3.31E-01	1.78E-03	2837	
5	rs325355	27409619	4.74E-02	N/A	18	rs961560	71821808	2.36E-01	N/A	-	2.47E-07	3.31E-01	1.78E-03	2838	
5	rs1459742	27426727	4.74E-02	N/A	18	rs961560	71821808	2.36E-01	N/A	-	2.47E-07	N/A	N/A	2839	
16	rs12448972	48308060	4.46E-01	ZNF423	16	s11649469	84711365	9.63E-02	N/A	-	2.47E-07	6.60E-01	1.61E-04	2840	
1	rs1125953	1.96E+08	9.32E-01	N/A	7	rs3808008	1.04E+08	8.53E-01	ORC5L	-	2.47E-07	7.39E-01	2.21E-05	2841	
3	rs11131064	7189945	5.95E-01	GRM7	20	rs6067653	49176578	1.30E-01	N/A	-	2.47E-07	9.71E-01	8.78E-05	2842	
5	rs397655	1.02E+08	5.46E-01	SLCO4C1	12	rs529446	5091580	5.54E-01	N/A	-	2.47E-07	7.82E-01	9.24E-05	2843	
13	rs1998678	71988485	2.27E-01	N/A	13	rs2260892	1.13E+08	3.67E-01	TMCO3	-	2.47E-07	8.39E-01	1.13E-05	2844	
6	rs9359271	78222839	2.79E-01	HTR1B	9	rs2242187	99177215	4.38E-01	N/A	-	2.47E-07	9.51E-01	1.45E-04	2845	
6	rs9359271	78222839	2.79E-01	HTR1B	9	rs4366181	99170166	4.38E-01	N/A	-	2.47E-07	9.51E-01	1.45E-04	2846	
5	rs4895293	1.2E+08	7.54E-01	N/A	20	rs557446	47632326	8.78E-01	PTGIS	-	2.47E-07	N/A	N/A	2847	
2	rs17016629	35506420	1.82E-01	N/A	7	rs3807155	1.38E+08	3.43E-01	TRIM24	-	2.48E-07	7.34E-01	2.22E-04	2848	
6	rs2342761	69667002	9.55E-02	BAI3	10	rs605089	78845394	8.31E-01	KCNMA1	-	2.48E-07	9.03E-01	1.03E-04	2849	
12	rs2162734	20250937	6.87E-01	N/A	17	rs2779252	23155497	5.83E-01	NOS2	-	2.48E-07	1.77E-01	9.35E-07	2850	
6	rs10499229	1.44E+08	5.95E-01	PHACTR2	16	rs4786482	4299422	8.17E-01	N/A	-	2.48E-07	8.51E-01	4.50E-05	2851	
3	rs10513354	1.51E+08	8.72E-01	WWTR1	17	rs758633	46350777	1.22E-01	N/A	-	2.48E-07	9.27E-01	8.92E-05	2852	
12	rs1551845	77838929	6.92E-01	SYT1	17	rs6504618	45041188	4.31E-01	SPOP	-	2.48E-07	8.03E-01	6.41E-06	2853	
5	rs7444529	17835564	7.62E-01	N/A	10	rs1414397	13474981	4.65E-01	N/A	-	2.48E-07	3.32E-01	1.84E-06	2854	
2	rs16846210	2.12E+08	8.11E-01	ERBB4	10	rs1227079	73122633	3.67E-01	CDH23	-	2.48E-07	5.18E-01	7.77E-05	2855	
14	rs3291	72100163	2.90E-01	RGS6	16	rs7186021	84043560	8.02E-01	N/A	-	2.48E-07	8.23E-01	1.48E-04	2856	
1	rs12402993	57551809	8.51E-01	DAB1	2	rs1427454	49416040	8.90E-01	N/A	-	2.48E-07	2.07E-01	3.15E-06	2857	
4	rs11726511	24919158	5.11E-01	ZCCHC4	9	rs658853	1.35E+08	3.21E-01	RALGDS	-	2.49E-07	1.33E-01	1.22E-03	2858	
14	rs1884008	85054657	7.87E-01	FLRT2	15	rs7170179	93964516	3.09E-05	N/A	-	2.49E-07	5.53E-01	3.66E-05	2859	
2	rs6431850	7165870	3.51E-01	N/A	5	rs1593902	28368592	9.09E-01	N/A	-	2.49E-07	N/A	N/A	2860	
8	rs2272712	1833703	6.99E-01	ARHGEF10	13	rs9576999	31273453	7.30E-01	RXFP2	-	2.49E-07	5.21E-01	4.49E-04	2861	
20	rs2741396	51597685	3.61E-01	ZNF217	20	rs767528	55478690	7.11E-01	N/A	-	2.49E-07	6.87E-02	3.55E-03	2862	
2	rs777589	61279125	3.60E-01	USP34	15	rs971804	83345385	4.00E-01	PDE8A	-	2.49E-07	1.27E-01	2.56E-03	2863	
6	rs4945787	1.08E+08	1.42E-01	SCML4	4	s13116347	1.49E+08	8.09E-01	NR3C2	-	2.49E-07	9.61E-01	2.34E-04	2864	
3	rs1962532	1.23E+08	5.01E-01	ILDR1	17	s16960503	19709516	1.85E-01	ULK2	-	2.49E-07	7.42E-01	2.26E-04	2865	
7	rs518136	17047682	4.62E-01	N/A	15	rs6494284	59625718	6.60E-01	N/A	-	2.49E-07	7.58E-01	5.93E-05	2866	
12	rs12812221	55605603	5.43E-01	SDR9C7	15	rs3098513	25479604	1.32E-01	N/A	-	2.49E-07	4.03E-01	4.45E-06	2867	
6	rs953034	65374016	8.15E-01	EYS	13	s12428930	1.08E+08	5.73E-01	TNFSF13B	-	2.49E-07	4.72E-01	3.77E-04	2868	
5	rs4957696	1.07E+08	4.75E-01	N/A	8	rs980238	4270026	1.10E-01	CSMD1	-	2.49E-07	5.19E-01	4.61E-04	2869	
1	rs16843795	1.7E+08	1.76E-01	DNM3	8	rs2278144	25689994	9.77E-01	N/A	-	2.49E-07	3.06E-01	3.56E-06	2870	
10	rs1409332	95395804	4.38E-01	PDE6C	15	rs1439323	45860350	1.85E-01	SEMA6D	-	2.49E-07	4.48E-01	1.05E-05	2871	
7	rs194825	1.04E+08	9.16E-01	ORC5L	13	rs4885537	77875455	1.46E-01	N/A	-	2.49E-07	7.15E-01	3.85E-05	2872	
11	rs2275998	66083157	1.21E-01	ACTN3	16	rs1476968	6492801	7.70E-01	A2BP1	-	2.49E-07	5.51E-01	2.07E-05	2873	
3	rs7619096	84281765	7.67E-02	N/A	13	rs3015350	1.05E+08	3.67E-01	N/A	-	2.49E-07	6.77E-01	7.25E-05	2874	
6	rs1555772	1.22E+08	4.39E-01	N/A	3	rs7651173	74390042	1.87E-01	CNTN3	-	2.49E-07	1.56E-02	8.94E-08	2875	
1	rs310172	61109878	8.79E-01	N/A	8	s10156248	1.22E+08	8.72E-01	N/A	-	2.50E-07	5.01E-02	2.93E-07	2876	
6	rs2635471	1.53E+08	6.32E-01	SYNE1	21	rs761231	15980909	6.26E-01	N/A	-	2.50E-07	8.58E-01	1.38E-04	2877	
5	rs10521001	32846548	2.55E-01	C5orf23	21	rs2298686	41854057	4.85E-02	N/A	-	2.50E-07	3.51E-01	1.49E-03	2878	
1	rs1125953	1.96E+08	9.32E-01	N/A	7	rs194867	1.04E+08	7.95E-01	ORC5L	-	2.50E-07	7.39E-01	2.01E-05	2879	
2	rs484968	2.39E+08	5.01E-01	ASB1	10	s17097516	1.2E+08	8.73E-01	N/A	-	2.50E-07	8.31E-01	1.42E-05	2880	
6	rs2023071	1.63E+08	8.61E-01	PARK2	13	s16972098	1.08E+08	1.62E-02	N/A	-	2.50E-07	3.71E-01	1.49E-04	2881	
1	rs10779581	2.11E+08	8.30E-01	N/A	1	rs1619856	2.33E+08	3.89E-01	TARBP1	-	2.50E-07	8.08E-01	3.10E-05	2882	
4	rs10461355	55616292	9.13E-01	N/A	7	rs320088	29049787	7.65E-01	CPVL	-	2.50E-07	9.26E-01	8.94E-05	2883	
1	rs7516085	1.89E+08	7.28E-01	N/A	3	rs2197126	19398565	5.56E-01	KCNH8	-	2.50E-07	7.56E-01	1.08E-05	2884	
5	rs2963134	1.64E+08	9.87E-01	N/A	7	s12669418	1.46E+08	1.95E-02	CNTN/AP2	-	2.50E-07	8.55E-01	5.48E-05	2885	
7	rs11984007	37557232	4.51E-01	N/A	12	rs4963932	25952950	1.69E-01	N/A	-	2.50E-07	9.75E-01	7.65E-05	2886	
3	rs1580470	1.98E+08	5.70E-01	PIGX	4	rs4693491	83846607	9.47E-01	SCD5	-	2.50E-07	7.81E-01	1.92E-04	2887	
3	rs9857526	1.24E+08	7.05E-02	SEC22A	12	s10877431	59132466	2.07E-01	N/A	-	2.50E-07	6.36E-02	1.48E-03	2888	
3	rs11129702	36273457	6.02E-01	N/A	16	rs6500892	6953116	9.44E-01	A2BP1	-	2.50E-07	9.32E-01	2.49E-05	2889	
3	rs4683068	45167977	6.76E-01	CDCP1	18	rs1539919	67403665	2.80E-01	N/A	-	2.51E-07	N/A	N/A	2890	
6	rs9392671	5252431	7.99E-01	FARS2	4	s12647248	1.23E+08	8.83E-01	N/A	-	2.51E-07	5.28E-01	2.79E-04	2891	
1	rs600734	75457991	2.70E-01	SLC44A5	5	rs828718	1.62E+08	5.73E-01	N/A	-	2.51E-07	6.59E-01	1.96E-05	2892	
6	rs550675	71035914	2.05E-01	COL9A1	5	rs735129	65576085	9.23E-01	N/A	-	2.51E-07	2.10E-01	7.99E-04	2893	
2	rs6546272	66998937	7.40E-01	N/A	10	s10872825	1.33E+08	4.87E-01	N/A	-	2.51E-07	3.29E-01	6.72E-06	2894	
15	rs11247231	99016603	8.43E-01	ASB7	20	rs6081256	18555297	1.48E-01	DTD1	-	2.51E-07	6.62E-01	1.13E-05	2895	
5	rs12054892	57981007	6.54E-01	RAB3C	14	rs3829948	92186579	5.37E-01	RIN3	-	2.51E-07	4.75E-01	1.26E-05	2896	
2	rs3791691	2.12E+08	3.66E-01	ERBB4	3	rs4677287	73598809	1.07E-01	PDZRN3	-	2.51E-07	6.76E-01	1.28E-05	2897	
13	rs1325360	32625148	9.71E-01	STARD13	16	rs2302715	48885095	1.64E-01	ADCY7	-	2.51E-07	6.71E-01	1.89E-04	2898	
9	rs12379650	83615761	7.53E-02	N/A	14	s10467770	20969471	8.71E-01	N/A	-	2.51E-07	9.46E-01	1.02E-04	2899	
8	rs17074981	5685852	4.27E-02	N/A	18	rs1822462	46590325	8.54E-01	MRO	-	2.51E-07	2.79E-01	3.78E-07	2900	
3	rs9850624	1.05E+08	6.04E-01	N/A	15	rs7169735	77090508	7.37E-02	RASGRF1	-	2.51E-07	4.12E-01	1.06E-04	2901	
2	rs10187044	2.26E+08	4.99E-01	N/A	4	rs2159972	18666528	1.66E-01	N/A	-	2.51E-07	7.76E-01	2.00E-04	2902	
11	rs885430	1.19E+08	9.58E-01	PVRL1	13	rs1041329	60197135	8.46E-01	N/A	-	2.52E-07	1.18E-01	7.12E-07	2903	
2	rs264625	1.6E+08	5.69E-01	TANC1	9	rs3008731	15993441	4.69E-01	N/A	-	2.52E-07	5.59E-01	1.28E-05	2904	
2	rs13396164	2.35E+08	5.70E-01	SPP2	16	rs1861532	53235014	1.44E-01	N/A	-	2.52E-07	6.26E-01	9.87E-05	2905	
11	rs885430	1.19E+08	9.58E-01	PVRL1	13	rs1329699	60196350	7.97E-01	N/A	-	2.52E-07	N/A	N/A	2906	
3	rs13094598	35709235	3.90E-01	ARPP-21	9	rs3802347	1.33E+08	8.87E-01	ABL1	-	2.52E-07	3.21E-01	1.60E-03	2907	
2	rs2715933	1.61E+08	4.24E-01	N/A	3	s11129577	34040753	7.33E-01	N/A	-	2.52E-07	9.99E-01	2.63E-04	2908	
6	rs7752376	1.63E+08	7.31E-01	PACRG	8	s13259780	78161214	2.33E-01	N/A	-	2.52E-07	1.98E-01	1.29E-06	2909	
3	rs7613069	10600996	5.88E-01	N/A	14	rs1983521	21688320	9.27E-02	N/A	-	2.52E-07	6.43E-01	7.08E-04	2910	

SNP A					SNP B						Interaction P			Ranking	Cluster in top 100 interactions
CHR	SNP	Location	gle locus P va	Gene	CHR	SNP	Location	ingle locus P valu	Gene	HC region	Stage 1	Stage 2	Combined		
1	rs1745371	46757779	6.23E-01	DMBX1	19	rs575756	61386317	1.81E-01	GALP	-	2.52E-07	8.56E-01	8.52E-05	2911	
2	rs6430516	1.35E+08	7.45E-01	TMEM163	4	s13117732	1.78E+08	4.03E-01	N/A	-	2.52E-07	1.34E-01	5.48E-07	2912	
19	rs17836364	59539399	3.11E-01	LILRA4	22	rs6004913	24768495	3.58E-01	MYO18B	-	2.52E-07	9.45E-01	8.47E-05	2913	
12	rs10859814	93902069	5.59E-01	NDUFA12	15	rs6494069	57173642	5.26E-01	RNF111	-	2.52E-07	N/A	N/A	2914	
12	rs4923674	93910102	5.59E-01	NDUFA12	15	rs6494069	57173642	5.26E-01	RNF111	-	2.52E-07	N/A	N/A	2915	
3	rs9819666	30529216	7.22E-01	N/A	10	s11001958	78410283	7.45E-01	KCNMA1	-	2.52E-07	N/A	N/A	2916	
6	rs7741863	11479601	5.95E-01	NEDD9	1	rs7540340	30138444	8.33E-01	N/A	-	2.52E-07	4.56E-01	5.50E-06	2917	
1	rs709688	41025772	4.06E-01	KCNQ4	16	s11649548	48396749	3.71E-01	ZNF423	-	2.52E-07	4.95E-01	1.73E-04	2918	
3	rs1496650	60164511	6.42E-01	FHIT	21	rs443684	39368104	8.26E-01	N/A	-	2.53E-07	3.16E-01	3.56E-04	2919	
1	rs1483198	4617251	2.86E-01	AJAP1	14	rs734654	77436720	8.01E-02	ADCK1	-	2.53E-07	6.86E-01	4.62E-05	2920	
8	rs4631498	1.39E+08	3.84E-01	N/A	12	s10492385	29248257	1.53E-01	FAR2	-	2.53E-07	9.52E-01	1.07E-04	2921	
15	rs3809482	41449094	5.41E-02	ADAL	19	rs7256487	19716654	6.42E-02	ZNF14	-	2.53E-07	8.17E-01	3.80E-05	2922	
15	rs6493084	41434919	5.41E-02	ADAL	19	rs7256487	19716654	6.42E-02	ZNF14	-	2.53E-07	8.17E-01	3.80E-05	2923	
15	rs9920879	41427062	5.41E-02	ADAL	19	rs7256487	19716654	6.42E-02	ZNF14	-	2.53E-07	8.17E-01	3.80E-05	2924	
1	rs4147825	94333526	4.49E-01	ABCA4	14	rs2430368	91457294	7.01E-02	FBLN5	-	2.53E-07	1.46E-01	2.20E-03	2925	
2	rs2601053	1.69E+08	7.06E-01	N/A	5	rs188693	42975987	7.34E-02	N/A	-	2.53E-07	2.62E-01	1.08E-03	2926	
6	rs9389037	1.33E+08	9.95E-01	N/A	16	s16975090	83577116	7.46E-01	ZDHHC7	-	2.53E-07	1.84E-01	2.31E-03	2927	
6	rs9375927	1.33E+08	9.95E-01	N/A	16	s16975090	83577116	7.46E-01	ZDHHC7	-	2.53E-07	2.37E-01	1.67E-03	2928	
11	rs986656	19488217	3.12E-01	N/AV2	18	rs1031287	18600005	1.85E-01	N/A	-	2.53E-07	6.28E-01	2.50E-04	2929	
11	rs986656	19488217	3.12E-01	N/AV2	18	rs1072782	18611634	1.85E-01	N/A	-	2.53E-07	6.28E-01	2.50E-04	2930	
7	rs7788751	1.53E+08	4.99E-01	N/A	9	rs888229	1.28E+08	7.76E-01	N/A	-	2.53E-07	8.99E-02	1.71E-07	2931	
8	rs352773	15691569	9.77E-01	N/A	18	rs2432842	68860542	1.84E-01	N/A	-	2.53E-07	6.97E-01	1.56E-05	2932	
8	rs3753115	17975052	3.40E-01	ASAH1	8	rs1473482	18641042	6.14E-02	PSD3	-	2.53E-07	1.45E-01	6.20E-07	2933	
7	rs4722286	23880149	8.36E-01	N/A	20	rs726207	15962926	6.80E-01	MACROD2	-	2.53E-07	6.09E-01	3.59E-04	2934	
15	rs341392	58911527	5.34E-01	RORA	19	s11880782	42128946	8.70E-01	ZNF568	-	2.53E-07	7.07E-01	1.27E-04	2935	
1	rs12022475	80075562	4.18E-02	N/A	10	rs1890583	3580269	9.20E-01	N/A	-	2.54E-07	4.14E-01	7.79E-06	2936	
11	rs673330	94464314	9.16E-01	ENDOD1	15	rs1124962	92601453	3.30E-02	N/A	-	2.54E-07	3.43E-02	1.03E-02	2937	
7	rs2237353	28764739	5.03E-01	CREB5	17	rs1521448	48695858	5.05E-01	N/A	-	2.54E-07	4.35E-02	2.29E-07	2938	
1	rs17538183	38631451	3.05E-02	N/A	2	rs2675045	1.84E+08	2.93E-01	NUP35	-	2.54E-07	5.43E-01	6.96E-05	2939	
1	rs2743208	18890442	2.92E-01	PAX7	3	rs9880276	1.06E+08	3.59E-02	N/A	-	2.54E-07	1.71E-01	6.85E-07	2940	
5	rs4704506	77690583	5.04E-01	SCAMP1	11	rs3741135	73391608	7.68E-01	UCP3	-	2.54E-07	7.82E-01	1.94E-05	2941	
8	rs2648847	1.29E+08	8.44E-01	MIR1208	17	rs7211638	69465455	6.86E-01	N/A	-	2.54E-07	8.63E-01	1.08E-04	2942	
9	rs1547663	1.19E+08	3.75E-01	ASTN2	17	rs544198	34572555	1.93E-03	PLXDC1	-	2.54E-07	1.27E-01	7.03E-04	2943	
6	rs4706713	69654952	8.04E-02	BAI3	10	rs605089	78845394	8.31E-01	KCNMA1	-	2.54E-07	9.08E-01	1.24E-04	2944	
5	rs244548	50965715	1.02E-01	N/A	18	rs1566819	24374735	1.49E-01	N/A	-	2.54E-07	7.37E-01	2.50E-05	2945	
5	rs244548	50965715	1.02E-01	N/A	18	rs879500	24378686	1.49E-01	N/A	-	2.54E-07	7.37E-01	2.50E-05	2946	
7	rs10239917	77993275	9.59E-02	MAGI2	18	s12966793	1450455	7.09E-01	N/A	-	2.54E-07	8.08E-01	3.92E-05	2947	
1	rs513086	72082573	4.73E-01	NEGR1	1	rs6680997	2.09E+08	4.83E-01	KCNH1	-	2.54E-07	8.40E-01	6.63E-07	2948	
1	rs10493425	67782715	4.50E-01	N/A	12	rs2732439	47030466	7.19E-01	N/A	-	2.54E-07	1.46E-01	1.24E-03	2949	
10	rs755228	76499380	8.99E-01	DUPD1	12	rs7956770	1.02E+08	9.24E-01	C12orf42	-	2.55E-07	6.52E-01	7.16E-05	2950	
6	rs9390358	1.46E+08	3.41E-01	FBXO30	10	rs1318662	26860966	6.27E-01	APBB1IP	-	2.55E-07	9.51E-01	1.21E-04	2951	
6	rs1199389	8336115	7.69E-01	N/A	17	rs236530	65729066	3.95E-01	N/A	-	2.55E-07	4.58E-02	4.09E-03	2952	
11	rs6590660	1.32E+08	9.90E-01	OPCML	14	rs4904156	83589239	6.19E-01	N/A	-	2.55E-07	7.05E-01	2.83E-05	2953	
10	rs506657	1.21E+08	1.90E-01	GRK5	11	rs990081	13851048	1.56E-01	N/A	-	2.55E-07	8.38E-01	1.36E-04	2954	
7	rs10247053	26603887	9.24E-01	N/A	12	rs7315384	1.03E+08	7.05E-01	STAB2	-	2.55E-07	1.98E-01	2.65E-03	2955	
5	rs10462345	72846913	4.45E-02	BTF3	20	rs232651	7015037	3.21E-01	N/A	-	2.55E-07	8.12E-01	2.50E-04	2956	
2	rs932559	31406406	5.28E-01	XDH	3	rs9866001	1.47E+08	1.54E-01	N/A	-	2.55E-07	2.77E-01	6.07E-06	2957	
13	rs732613	21376317	3.73E-01	N/A	14	rs225902	29529202	3.35E-01	N/A	-	2.55E-07	9.88E-01	5.43E-05	2958	
3	rs301564	11778927	9.11E-01	N/A	4	rs1448817	1.12E+08	2.85E-01	N/A	-	2.55E-07	4.07E-01	4.00E-04	2959	
6	rs9459207	1.65E+08	7.57E-01	N/A	15	s11632715	30791539	3.09E-01	GREM1	-	2.55E-07	7.61E-01	3.34E-04	2960	
6	rs1925220	1.22E+08	3.73E-01	N/A	3	rs524431	74383584	3.74E-01	CNTN3	-	2.55E-07	4.94E-02	3.54E-07	2961	
7	rs10280848	67742124	8.09E-01	N/A	10	rs993398	26326947	2.98E-01	MYO3A	-	2.55E-07	2.24E-01	6.59E-04	2962	
15	rs11247231	99016603	8.43E-01	ASB7	20	rs6081232	18519572	1.29E-01	DTD1	-	2.55E-07	8.41E-01	2.11E-05	2963	
11	rs10891175	1.1E+08	3.76E-01	N/A	20	rs6053798	593853	1.67E-01	SCRT2	-	2.56E-07	2.26E-01	5.62E-07	2964	
12	rs1377003	43218535	7.93E-02	NELL2	18	rs535796	22574324	9.92E-01	N/A	-	2.56E-07	7.18E-01	1.34E-05	2965	
5	rs11750377	1.04E+08	2.36E-01	N/A	21	rs2000639	39202392	2.39E-01	N/A	-	2.56E-07	3.77E-01	1.13E-05	2966	
3	rs9869824	33898549	5.55E-01	PDCD6IP	13	rs2038711	21757408	3.12E-02	N/A	-	2.56E-07	2.95E-01	1.15E-04	2967	
1	rs4568808	1.91E+08	7.52E-01	RGS13	13	rs2803218	1E+08	3.87E-01	N/A	-	2.56E-07	4.06E-01	1.23E-05	2968	
3	rs9852724	77734675	7.82E-01	ROBO2	5	rs9327428	1.26E+08	5.43E-01	3-Mar	-	2.56E-07	5.63E-01	1.55E-05	2969	
6	rs12211869	1.51E+08	4.62E-01	MTHFD1L	11	rs674827	10889767	1.10E-01	N/A	-	2.56E-07	N/A	N/A	2970	
12	rs772562	61591648	5.31E-01	PPM1H	14	rs847297	71742850	9.21E-01	RGS6	-	2.56E-07	3.01E-01	4.27E-04	2971	
4	rs1379410	1.8E+08	9.15E-02	N/A	5	s10053880	1.13E+08	4.61E-01	N/A	-	2.56E-07	7.25E-01	1.30E-04	2972	
3	rs6550901	24788964	7.02E-01	N/A	11	rs1387153	92313476	4.04E-01	N/A	-	2.56E-07	8.79E-01	1.37E-05	2973	
2	rs7575751	84549514	9.28E-02	SUCLG1	2	s10804253	2.18E+08	2.64E-01	N/A	-	2.56E-07	8.52E-01	6.07E-05	2974	
11	rs1790474	1.19E+08	4.13E-01	N/A	15	rs4777466	69923926	8.62E-01	MYO9A	-	2.56E-07	8.55E-01	5.72E-05	2975	
6	rs3094204	31199971	3.72E-01	C6orf15	9	rs3844046	1.32E+08	4.02E-01	PRRX2	MHC	2.57E-07	9.80E-01	8.50E-05	2976	
1	rs6703099	75306907	6.03E-01	N/A	5	rs828718	1.62E+08	5.73E-01	N/A	-	2.57E-07	4.71E-01	5.81E-06	2977	
1	rs12730329	85254288	5.38E-01	MCOLN2	8	s17270769	1.25E+08	6.56E-01	FER1L6	-	2.57E-07	6.83E-01	1.11E-04	2978	
1	rs3765692	3574631	4.04E-01	TP73	7	rs201171	6573680	5.34E-01	TAS1R1	-	2.57E-07	5.40E-02	1.93E-02	2979	
1	rs10800319	1.66E+08	9.62E-01	N/A	18	s12964156	24831978	4.76E-01	N/A	-	2.57E-07	1.33E-01	5.30E-07	2980	
11	rs3134909	44059753	9.61E-01	EXT2	12	rs7971473	69568041	6.83E-01	PTPRR	-	2.57E-07	2.29E-01	8.09E-04	2981	
8	rs2648883	1.29E+08	8.39E-01	PVT1	11	rs3060	75189220	2.61E-01	DGAT2	-	2.57E-07	3.78E-01	9.92E-04	2982	
8	rs10503296	4803438	6.89E-01	CSMD1	11	s11222955	1.32E+08	2.65E-01	NTM	-	2.57E-07	7.43E-01	4.96E-05	2983	
16	rs274074	24795955	1.13E-01	SLC5A11	17	rs9908225	52969779	4.64E-01	MSI2	-	2.57E-07	9.37E-01	3.35E-05	2984	
1	rs356285	80750041	9.43E-01	N/A	4	s16880691	28082146	6.88E-01	N/A	-	2.57E-07	5.05E-01	2.17E-04	2985	
5	rs7720237	1.09E+08	1.14E-01	N/A	10	s12262014	11379015	1.26E-01	CUGBP2	-	2.57E-07	9.78E-01	1.28E-04	2986	
2	rs1834619	17764966	5.69E-01	SMC6	8	rs4358797	34751512	7.25E-01	N/A	-	2.57E-07	2.95E-02	1.39E-02	2987	
3	rs4078054	1.8E+08	1.71E-01	KCNMB2	5	s13362504	56014886	9.88E-01	N/A	-	2.57E-07	6.04E-01	3.00E-05	2988	
1	rs2494523	1.58E+08	4.30E-01	FCRL6	12	rs756502	531292	1.24E-01	B4GALNT3	-	2.57E-07	8.00E-01	6.85E-05	2989	
10	rs3793854	61523062	3.68E-01	ANK3	12	rs1514661	41965940	3.57E-01	N/A	-	2.57E-07	9.54E-01	9.04E-05	2990	
6	rs4235996	19576099	4.19E-01	N/A	1	s17664308	1.98E+08	5.53E-01	N/A	-	2.57E-07	1.74E-01	4.74E-06	2991	
6	rs9380215	31157634	4.29E-02	N/A	6	rs9295957	31265572	2.18E-01	POU5F1	MHC	2.58E-07	1.32E-01	1.63E-06	2992	
6	rs9261564	30282569	1.60E-01	TRIM26	12	rs699573	61577631	6.44E-01	PPM1H	MHC	2.58E-07	5.96E-02	3.53E-03	2993	
10	rs12243844	55532600	6.59E-01	PCDH15	11	rs601904	73878387	5.93E-02	LIPT2	-	2.58E-07	4.85E-01	3.02E-04	2994	
6	rs7750881	1.65E+08	8.79E-01	N/A	2	s13387662	34016918	2.26E-01	N/A	-	2.58E-07	5.96E-01	5.28E-06	2995	
2	rs4674875	2.25E+08	6.75E-01	N/A	16	rs7195698	7256644	3.46E-01	A2BP1	-	2.58E-07	9.23E-01	4.15E-05	2996	
8	rs7014579	439706	3.51E-01	C8orf42	12	s12427354	22096664	4.42E-01	CMAS	-	2.58E-07	6.48E-01	1.34E-04	2997	
5	rs28235	3504196	9.75E-01	N/A	10	s11000792	53534408	2.27E-01	PRKG1	-	2.58E-07	7.37E-01	1.35E-04	2998	
6	rs9498131	1.49E+08	4.50E-01	N/A	9	rs2798058	92063000	7.55E-01	N/A	-	2.58E-07	4.96E-01	5.34E-05	2999	
4	rs2378847	1.8E+08	7.19E-01	N/A	7	rs2699467	1.44E+08	7.20E-01	N/A	-	2.58E-07	3.46E-02	1.14E-07	3000	
6	rs10457834	1.49E+08	2.16E-01	N/A	9	rs2798060	92068763	9.20E-01	N/A	-	2.58E-07	7.18E-01	1.49E-04	3001	
6	rs4897043	1.49E+08	2.16E-01	N/A	9	rs2798060	92068763	9.20E-01	N/A	-	2.58E-07	7.18E-01	1.49E-04	3002	
6	rs9380215	31157634	4.29E-02	N/A	6	rs1062470	31192414	5.89E-01	C6orf15	MHC	2.59E-07	9.08E-02	8.70E-07	3003	
2	rs1829731	19525417	6.93E-01	N/A	12	rs1683163	25124885	2.18E-01	LRMP	-	2.59E-07	1.48E-02	3.45E-08	3004	
8	rs754539	18631372	5.96E-01	PSD3	18	s11081675	26834675	8.47E-01	DSC3	-	2.59E-07	5.47E-01	2.41E-04	3005	
8	rs10090702	59864937	3.48E-01	TOX	9	rs7855088	99782074	6.54E-01	ANP32B	-	2.59E-07	6.25E-01	3.37E-04	3006	
1	rs789099	2.37E+08	7.39E-01	N/A	8	rs1026349	15006865	9.73E-01	SGCZ	-	2.59E-07	6.84E-01	1.74E-04	3007	
6	rs12525909	1.65E+08	7.54E-01	N/A	2	s13387662	34016918	2.26E-01	N/A	-	2.59E-07	8.90E-01	1.71E-05	3008	
10	rs7915236	60924819	9.66E-01	N/A	14	rs1950806	88310509	8.80E-01	EML5	-	2.59E-07	3.14E-01	9.27E-04	3009	
10	rs1751291	4482612	2.98E-01	N/A	16	s12598271	81163012	3.75E-01	N/A	-	2.59E-07	2.42E-01	5.93E-07	3010	
5	rs4296777	1.06E+08	2.30E-01	N/A	12	s12824017	61935103	2.85E-01	N/A	-	2.59E-07	5.28E-01	3.00E-05	3011	
8	rs1516969	1.3E+08	5.70E-01	N/A	19	rs8112582	8148511	4.19E-01	N/A	-	2.59E-07	6.18E-01	7.66E-05	3012	
8	rs987926	15667229	7.65E-01	TUSC3	22	rs735455	20743793	9.48E-01	N/A	-	2.59E-07	6.58E-01	7.76E-06	3013	
13	rs1324408	25414460	2.67E-01	ATP8A2	13	rs9529874	70831653	2.82E-01	N/A	-	2.59E-07	4.94E-01	2.66E-05	3014	
8	rs3824250	1.25E+08	7.91E-01	WDYHV1	14	rs7157201	73818930	3.00E-01	VSX2	-	2.59E-07	4.94E-01	2.66E-05	3015	
3	rs10460960	42283739	7.63E-01	N/A	20	rs963487	20769624	4.61E-01	N/A	-	2.59E-07	5.84E-01	6.98E-05	3016	
8	rs6999234	1.25E+08	7.91E-01	WDYHV1	14	rs7157201	73818930	3.00E-01	VSX2	-	2.59E-07	7.65E-02	4.07E-07	3017	
8	rs7014678	1.25E+08	7.91E-01	WDYHV1	14	rs7157201	73818930	3.00E-01	VSX2	-	2.59E-07	4.64E-01	9.49E-06	3018	
6	rs9348862	31360726	7.72E-01	HLA-C	6	rs2844575	31442924	5.47E-01	HLA-B	MHC	2.59E-07	4.64E-01	9.49E-06	3019	
6	rs9357122	31368324	7.72E-01	N/A	6	rs2844575	31442924	5.47E-01	HLA-B	MHC	2.59E-07	4.64E-01	9.49E-06	3020	
7	rs2058163	34301616	2.61E-01	N/A	13	rs693039	53944792	9.51E-01	N/A	-	2.60E-07	9.15E-01	5.33E-05	3021	
5	rs1826675	62102994	2.07E-02	N/A	9	rs7043121	4204847	8.60E-01	GLIS3	-	2.60E-07	5.61E-01	5.50E-06	3022	

SNP A					SNP B						Interaction P			Ranking	Cluster in top 100 interactions
CHR	SNP	Location	gle locus P va	Gene	CHR	SNP	Location	Single locus P valu	Gene	MHC region	Stage 1	Stage 2	Combined		
6	rs9395232	47189989	2.33E-01	N/A	6	rs2064712	1.61E+08	3.03E-01	N/A	-	2.60E-07	3.06E-01	1.49E-06	3023	
16	rs4072137	85488196	5.16E-01	N/A	19	rs2902970	34212410	1.85E-01	N/A	-	2.60E-07	8.87E-01	7.59E-05	3024	
6	rs531815	10898737	4.31E-01	MAK	18	rs10454095	18031438	5.72E-02	GATA6	-	2.60E-07	5.28E-01	6.48E-04	3025	
8	rs10504091	50923329	7.21E-01	N/A	20	rs6025422	55183188	3.01E-01	BMP7	-	2.60E-07	1.97E-02	1.58E-02	3026	
8	rs13255117	50943966	7.21E-01	N/A	20	rs6025422	55183188	3.01E-01	BMP7	-	2.60E-07	2.44E-02	1.21E-02	3027	
2	rs4503980	2.27E+08	1.85E-01	N/A	9	rs10120210	94324803	9.58E-01	ECM2	-	2.60E-07	1.51E-01	9.82E-07	3028	
9	rs10813878	32849234	7.62E-01	N/A	12	rs1489418	1.28E+08	2.29E-01	TMEM132C	-	2.60E-07	4.39E-01	3.75E-04	3029	
7	rs219822	98427987	1.21E-01	TRRAP	12	rs10771730	30507860	5.51E-01	N/A	-	2.60E-07	7.91E-02	8.85E-08	3030	
7	rs537276	17491609	8.35E-01	N/A	14	rs11624983	33824468	6.29E-01	N/A	-	2.60E-07	9.68E-01	6.52E-05	3031	
7	rs6949899	1.27E+08	3.53E-01	N/A	11	rs1478750	1.34E+08	4.25E-01	N/A	-	2.60E-07	2.92E-01	1.83E-03	3032	
1	rs12731127	53398214	9.24E-01	SLC1A7	9	rs10760397	1.27E+08	7.80E-01	GAPVD1	-	2.60E-07	9.22E-01	2.74E-05	3033	
6	rs2076513	1536010	9.71E-01	FOXC1	11	rs17333828	10961741	2.29E-02	N/A	-	2.61E-07	5.62E-01	4.66E-05	3034	
8	rs7833351	95251354	6.61E-01	CDH17	17	rs2945395	22909096	8.14E-01	KSR1	-	2.61E-07	4.41E-01	5.53E-06	3035	
3	rs9827214	1.59E+08	8.27E-01	N/A	5	rs6450490	50334219	4.55E-01	N/A	-	2.61E-07	9.55E-02	1.68E-07	3036	
13	rs4055938	33191277	7.40E-01	N/A	13	rs3015345	1.05E+08	7.02E-01	N/A	-	2.61E-07	3.62E-01	2.49E-04	3037	
18	rs7238168	32291551	4.02E-01	FHOD3	19	rs2013899	57360509	2.10E-01	ZNF836	-	2.61E-07	8.63E-01	7.54E-05	3038	
2	rs4853669	1.93E+08	8.90E-02	N/A	10	rs7077246	1.27E+08	1.38E-01	N/A	-	2.61E-07	6.39E-01	5.71E-05	3039	
6	rs10943465	78293015	1.26E-01	N/A	14	rs4899065	61571458	2.01E-01	SYT16	-	2.61E-07	4.62E-01	4.23E-06	3040	
6	rs3130467	31295054	5.92E-02	HCG27	6	rs3130534	31317024	3.50E-01	N/A	MHC	2.61E-07	7.60E-01	2.87E-04	3041	
5	rs6555368	5793248	9.15E-01	N/A	18	rs10502527	24834125	3.55E-01	N/A	-	2.62E-07	3.01E-02	6.89E-08	3042	
5	rs2895450	5790475	9.15E-01	N/A	18	rs1941193	24840424	3.55E-01	N/A	-	2.62E-07	3.03E-02	6.93E-08	3043	
5	rs2895450	5790475	9.15E-01	N/A	18	rs10502527	24834125	3.55E-01	N/A	-	2.62E-07	5.41E-02	1.47E-07	3044	
6	rs7761965	31381474	9.15E-01	N/A	6	rs2071596	31614670	4.97E-01	NFKBIL1	MHC	2.62E-07	5.43E-02	1.48E-07	3045	
5	rs6555368	5793248	9.15E-01	N/A	18	rs1941193	24840424	3.55E-01	N/A	-	2.62E-07	N/A	N/A	3046	
5	rs7722440	5793672	9.15E-01	N/A	18	rs10502527	24834125	3.55E-01	N/A	-	2.62E-07	N/A	N/A	3047	
5	rs7722440	5793672	9.15E-01	N/A	18	rs1941193	24840424	3.55E-01	N/A	-	2.62E-07	4.00E-01	1.27E-03	3048	
3	rs3821767	10898941	4.55E-01	SLC6A11	8	rs4736681	1.35E+08	8.76E-01	ST3GAL1	-	2.62E-07	5.56E-01	3.79E-04	3049	
1	rs2358757	34273393	1.74E-01	CSMD2	17	rs1990197	65582129	8.57E-01	KCNJ16	-	2.62E-07	8.32E-01	7.81E-05	3050	
3	rs9637471	59978630	3.65E-01	FHIT	7	rs10227002	47694132	5.19E-02	N/A	-	2.62E-07	1.80E-01	2.03E-06	3051	
6	rs12213837	1.5E+08	2.47E-01	ULBP3	12	rs7970515	82050093	5.66E-01	TMTC2	-	2.62E-07	4.39E-01	2.59E-06	3052	
6	rs11155700	1.5E+08	2.47E-01	ULBP3	12	rs7970515	82050093	5.66E-01	TMTC2	-	2.62E-07	4.70E-01	2.64E-05	3053	
2	rs4622658	1.3E+08	2.51E-01	N/A	5	rs4349709	1.24E+08	1.05E-01	N/A	-	2.62E-07	5.60E-01	3.92E-05	3054	
8	rs1685101	32317455	6.83E-01	NRG1	11	rs1112922	81286092	2.79E-01	N/A	-	2.62E-07	9.17E-01	1.66E-04	3055	
5	rs2909920	1.2E+08	8.44E-01	N/A	20	rs557446	47632326	8.78E-01	PTGIS	-	2.62E-07	4.02E-01	7.44E-06	3056	
12	rs11104208	85869527	6.40E-01	N/A	16	rs4997772	80462796	1.94E-01	PLCG2	-	2.62E-07	9.57E-01	7.95E-05	3057	
12	rs1873154	85870225	6.40E-01	N/A	16	rs4997772	80462796	1.94E-01	PLCG2	-	2.62E-07	9.57E-01	7.95E-05	3058	
10	rs7922151	14738516	6.40E-01	FAM107B	18	rs2939414	58135802	6.53E-01	KIAA1468	-	2.62E-07	9.13E-01	2.37E-04	3059	
8	rs11779713	17208445	1.94E-01	MTMR7	15	rs767387	51385518	8.23E-01	N/A	-	2.62E-07	9.81E-01	2.27E-05	3060	
12	rs4131955	73597602	9.03E-01	N/A	14	rs12436039	78521772	1.14E-01	NRXN3	-	2.62E-07	8.87E-01	5.21E-05	3061	
4	rs17084662	64882812	4.69E-01	TECRL	7	rs10950503	2006120	4.02E-01	MAD1L1	-	2.62E-07	5.48E-01	1.16E-03	3062	
6	rs3130534	31317024	3.50E-01	N/A	6	rs7750269	31379136	8.58E-01	N/A	MHC	2.63E-07	2.30E-01	2.79E-06	3063	
9	rs3011289	1.35E+08	8.24E-01	TSC1	17	rs227666	52167514	2.34E-01	N/A	-	2.63E-07	4.35E-01	6.22E-04	3064	
3	rs4857355	99444827	7.71E-01	N/A	16	rs6500343	49448844	5.45E-01	N/A	-	2.63E-07	2.03E-01	1.02E-03	3065	
6	rs515077	8125285	5.80E-01	N/A	7	rs2282965	86239740	3.03E-01	GRM3	-	2.63E-07	6.51E-01	8.87E-05	3066	
6	rs2172784	1.66E+08	1.60E-01	N/A	15	rs471751	64122891	9.02E-01	MEGF11	-	2.63E-07	8.88E-01	3.45E-05	3067	
9	rs10124779	84708950	8.65E-01	N/A	10	rs2078276	97077049	2.17E-01	SORBS1	-	2.63E-07	5.40E-01	4.16E-06	3068	
6	rs9458499	1.63E+08	9.55E-01	PARK2	3	rs7646021	62469648	7.86E-02	CADPS	-	2.63E-07	6.44E-01	3.39E-04	3069	
6	rs7759209	44560135	2.19E-01	N/A	19	rs1017219	54265250	7.76E-01	CGB7	-	2.63E-07	3.69E-01	8.26E-04	3070	
7	rs12705744	1.1E+08	3.82E-01	IMMP2L	8	rs12676463	32459740	6.39E-01	NRG1	-	2.63E-07	7.07E-01	3.82E-06	3071	
5	rs244548	50965715	1.02E-01	N/A	18	rs1857705	24385115	1.45E-01	N/A	-	2.63E-07	8.33E-01	3.50E-05	3072	
6	rs1399221	1.63E+08	6.79E-01	PACRG	4	rs16840358	7529531	5.11E-01	SORCS2	-	2.63E-07	5.54E-01	1.96E-05	3073	
6	rs10946578	22854096	8.77E-01	N/A	3	rs968861	1.95E+08	4.22E-01	N/A	-	2.63E-07	9.31E-01	5.55E-05	3074	
3	rs7646289	24776800	7.23E-01	N/A	11	rs1387153	92313476	4.04E-01	N/A	-	2.64E-07	5.32E-01	2.08E-05	3075	
6	rs4495304	31188697	4.80E-02	C6orf15	6	rs3131003	31201461	6.51E-01	C6orf15	MHC	2.64E-07	6.77E-01	5.78E-06	3076	
6	rs7761965	31381474	9.15E-01	N/A	6	rs2596501	31429190	2.85E-01	HLA-B	MHC	2.64E-07	9.69E-01	1.26E-04	3077	
5	rs6555878	1.69E+08	1.66E-01	FAM196B	13	rs10507804	71928655	1.09E-01	N/A	-	2.64E-07	4.27E-01	4.87E-04	3078	
1	rs2968485	96642521	6.69E-01	N/A	5	rs931454	67287006	9.75E-01	N/A	-	2.64E-07	8.69E-01	4.20E-05	3079	
3	rs1362772	1.71E+08	8.90E-01	MECOM	8	rs9642980	1.04E+08	4.68E-02	N/A	-	2.64E-07	7.40E-03	1.81E-08	3080	
1	rs4846609	2.15E+08	5.21E-01	ESRRG	4	rs6822279	99964728	1.96E-01	N/A	-	2.64E-07	8.29E-01	1.36E-05	3081	
2	rs11896237	32882823	1.40E-01	N/A	4	rs4552542	75818014	9.42E-01	N/A	-	2.64E-07	8.98E-01	7.12E-05	3082	
4	rs8340	56899534	6.06E-01	KIAA1211	16	rs13332555	54442500	6.18E-01	N/A	-	2.64E-07	2.33E-01	2.13E-06	3083	
9	rs7026124	74384798	3.10E-02	TMC1	17	rs2323661	15241034	1.56E-01	N/A	-	2.64E-07	4.35E-01	1.75E-03	3084	
1	rs12120414	56615717	7.99E-01	N/A	5	rs12659150	25967191	4.97E-02	N/A	-	2.64E-07	1.15E-01	5.23E-04	3085	
3	rs2204261	1.68E+08	4.72E-01	N/A	11	rs11024039	16753836	7.68E-01	C11orf58	-	2.64E-07	2.69E-01	1.03E-05	3086	
7	rs2058163	34301616	2.61E-01	N/A	13	rs9316728	53978870	8.29E-01	N/A	-	2.64E-07	7.74E-01	2.46E-05	3087	
7	rs4728351	75202053	2.44E-01	HIP1	8	rs10112728	68736913	7.10E-02	CPA6	-	2.65E-07	5.82E-03	1.51E-08	3088	
4	rs11737590	1.52E+08	5.36E-01	LRBA	20	rs856333	50750148	9.42E-01	N/A	-	2.65E-07	8.16E-01	6.00E-05	3089	
4	rs877378	1.85E+08	5.29E-01	N/A	11	rs1502259	88675893	1.46E-01	TYR	-	2.65E-07	2.04E-01	1.34E-06	3090	
3	rs17070650	64347702	5.24E-01	N/A	17	rs7211270	46870132	9.91E-01	N/A	-	2.65E-07	2.47E-01	3.36E-06	3091	
6	rs2798960	78279947	1.39E-01	N/A	14	rs4899065	61571458	2.01E-01	SYT16	-	2.65E-07	5.24E-01	7.82E-04	3092	
6	rs9367264	47186418	3.24E-01	N/A	19	rs1506684	56063231	8.35E-01	KLK2	-	2.65E-07	7.97E-01	3.96E-05	3093	
6	rs9367264	47186418	3.24E-01	N/A	19	rs2569739	56064207	8.35E-01	KLK2	-	2.65E-07	7.97E-01	3.96E-05	3094	
11	rs11037175	42927616	1.95E-01	N/A	13	rs9576228	36717029	8.55E-01	N/A	-	2.65E-07	3.45E-01	5.99E-06	3095	
10	rs2420724	1.22E+08	1.21E-02	PPAPDC1A	11	rs998544	55941464	4.68E-01	OR8U8	-	2.65E-07	8.62E-01	4.25E-05	3096	
2	rs17026540	85766673	9.06E-01	SFTPB	10	rs1338431	1.15E+08	5.13E-01	N/A	-	2.65E-07	7.96E-02	4.45E-03	3097	
6	rs483366	1.62E+08	3.18E-01	PARK2	8	rs4568622	85942553	7.97E-01	RALYL	-	2.65E-07	8.59E-01	2.39E-05	3098	
12	rs12422318	85902122	7.44E-01	N/A	16	rs4997772	80462796	1.94E-01	PLCG2	-	2.65E-07	9.68E-01	1.12E-04	3099	
5	rs17136136	1.13E+08	4.18E-01	N/A	9	rs11789633	4311558	2.29E-03	N/A	-	2.65E-07	N/A	N/A	3100	
3	rs7629489	6756909	9.90E-01	N/A	16	rs1105355	82633064	2.50E-01	MBTPS1	-	2.65E-07	4.62E-01	3.35E-06	3101	
5	rs11749422	25003793	9.86E-01	N/A	9	rs4977871	23633682	9.19E-01	N/A	-	2.65E-07	9.35E-01	7.04E-05	3102	
4	rs9307532	80650245	5.15E-02	N/A	7	rs2390961	24266172	2.98E-01	N/A	-	2.66E-07	1.48E-01	1.76E-06	3103	
5	rs6892217	1.71E+08	4.33E-01	STK10	11	rs2298525	1.21E+08	5.90E-01	SORL1	-	2.66E-07	4.87E-01	1.33E-04	3104	
10	rs755228	76499380	8.99E-01	DUPD1	12	rs10860999	1.02E+08	8.42E-01	C12orf42	-	2.66E-07	7.01E-01	5.19E-05	3105	
9	rs10521449	74412721	5.80E-02	TMC1	17	rs2323661	15241034	1.56E-01	N/A	-	2.66E-07	7.16E-01	3.44E-04	3106	
7	rs10250954	22441474	5.74E-01	MGC87042	16	rs8049897	88551703	3.59E-01	AFG3L1	-	2.66E-07	3.74E-01	7.05E-06	3107	
2	rs10174856	1.34E+08	9.29E-01	NCKAP5	12	rs917634	6501430	4.78E-01	N/A	-	2.66E-07	6.74E-01	6.64E-05	3108	
3	rs6783300	1.77E+08	1.38E-01	N/AALADL2	14	rs8008645	61612978	9.48E-01	SYT16	-	2.66E-07	N/A	N/A	3109	
2	rs2057347	32846440	6.82E-01	N/A	14	rs3861621	77478622	7.86E-02	ADCK1	-	2.66E-07	1.96E-01	2.67E-03	3110	
4	rs2290870	42271177	9.93E-01	ATP8A1	8	rs2610935	21119368	8.96E-02	N/A	-	2.66E-07	1.44E-01	3.65E-06	3111	
4	rs6826949	1.15E+08	9.93E-01	ANK2	11	rs1567523	7808485	8.85E-01	OR5P3	-	2.66E-07	5.74E-01	2.73E-04	3112	
1	rs12035946	39177455	8.05E-01	RHBDL2	3	rs1564406	1.4E+08	3.36E-01	MRAS	-	2.66E-07	5.97E-01	8.21E-05	3113	
8	rs3110150	60212394	7.67E-02	TOX	9	rs1359637	1.16E+08	7.69E-01	N/A	-	2.66E-07	N/A	N/A	3114	
16	rs9925231	86895608	5.95E-01	N/A	21	rs2834484	34738811	1.90E-01	KCNE1	-	2.67E-07	2.52E-02	2.50E-08	3115	
2	rs17012765	75565481	9.53E-02	N/A	5	rs9918275	66165571	4.87E-01	MAST4	-	2.67E-07	6.00E-01	7.34E-06	3116	
12	rs10771219	25514113	3.02E-01	IFLTD1	18	rs9957878	55358933	5.69E-01	CCBE1	-	2.67E-07	7.76E-01	1.00E-05	3117	
2	rs4670648	33595788	2.17E-01	RASGRP3	3	rs903056	14785294	1.95E-01	N/A	-	2.67E-07	1.56E-01	2.43E-06	3118	
2	rs4998208	1133477	1.83E-01	SNTG2	3	rs2173911	1.92E+08	4.07E-01	LOC647309	-	2.67E-07	3.77E-01	3.47E-04	3119	
5	rs3797937	9244362	9.13E-01	SEMA5A	9	rs6475227	18528877	7.10E-01	ADAMTSL1	-	2.67E-07	1.38E-01	2.57E-07	3120	
2	rs401974	13999468	5.71E-01	N/A	18	rs11080490	11086618	3.39E-01	FAM38B	-	2.67E-07	5.02E-01	6.92E-05	3121	
4	rs7673236	83044195	3.78E-01	N/A	8	rs17177644	12812392	1.25E-01	N/A	-	2.67E-07	8.60E-01	1.52E-04	3122	
1	rs506813	1.64E+08	4.40E-01	UCK2	11	rs2276039	56893947	2.75E-01	PRG3	-	2.67E-07	9.87E-01	2.03E-04	3123	
7	rs6973203	1.47E+08	1.84E-01	CNTN/AP2	19	rs376620	6896861	5.37E-01	EMR1	-	2.67E-07	9.69E-03	7.76E-08	3124	
5	rs31464	16991569	7.87E-01	MYO10	5	rs11135037	1.58E+08	3.17E-01	N/A	-	2.67E-07	6.28E-02	4.03E-08	3125	
7	rs1653876	32327144	3.17E-01	N/A	12	rs918130	22201720	4.53E-01	N/A	-	2.67E-07	1.03E-01	6.31E-07	3126	
5	rs16869748	90789632	6.85E-01	N/A	19	rs11671204	14513278	9.17E-02	TECR	-	2.67E-07	3.40E-01	3.64E-06	3127	
1	rs7534662	2.29E+08	2.88E-02	CAPN9	7	rs11980237	6474042	6.53E-01	KDELR2	-	2.67E-07	3.63E-01	1.54E-05	3128	
1	rs7537410	2.29E+08	2.88E-02	CAPN9	7	rs11980237	6474042	6.53E-01	KDELR2	-	2.67E-07	3.63E-01	1.54E-05	3129	
6	rs4947296	31166157	4.31E-02	N/A	6	rs1062470	31192414	5.89E-01	C6orf15	MHC	2.68E-07	4.96E-02	1.34E-02	3130	
10	rs1327014	1.3E+08	7.85E-01	N/A	20	rs419762	187294	9.04E-01	C20orf96	-	2.68E-07	1.07E-01	1.14E-06	3131	
1	rs3767178	1.78E+08	9.88E-01	QSOX1	21	rs879502	24456529	2.43E-02	N/A	-	2.68E-07	5.20E-01	5.99E-06	3132	
11	rs2707098	19868309	3.39E-01	N/AV2	13	rs9301254	1.07E+08	2.44E-01	FAM155A	-	2.68E-07	7.43E-01	8.40E-05	3133	
8	rs2285275	17215970	3.71E-01	MTMR7	15	rs767387	51385518	8.23E-01	N/A	-	2.68E-07	8.68E-01	1.40E-05	3134	

SNP A					SNP B						Interaction P				
CHR	SNP	Location	gle locus P va	Gene	CHR	SNP	Location	Single locus P valu	Gene	MHC region	Stage 1	Stage 2	Combined	Ranking	Cluster in top 100 interactions
6	rs2328537	1.44E+08	7.35E-01	PLAGL1	8	s10107668	33643721	2.78E-02	N/A	-	2.68E-07	4.19E-02	1.06E-07	3135	
6	rs7763726	1.6E+08	9.04E-02	FNDC1	2	s11894795	1.23E+08	9.77E-01	N/A	-	2.68E-07	4.83E-01	3.19E-05	3136	
6	rs9472138	43919740	3.62E-01	N/A	13	rs527337	32459520	8.32E-01	N/A	-	2.68E-07	5.96E-01	2.91E-05	3137	
12	rs6539316	1.06E+08	2.41E-02	N/A	17	rs3744345	12831997	8.17E-01	RICH2	-	2.68E-07	3.08E-02	7.73E-08	3138	
7	rs2394936	98251592	6.88E-01	N/A	17	rs6504468	62318279	6.45E-01	CACNG5	-	2.68E-07	7.62E-01	1.96E-05	3139	
6	rs1684802	1.54E+08	7.41E-01	N/A	4	s1093782C	7480682	8.93E-01	SORCS2	-	2.68E-07	3.45E-01	2.59E-03	3140	
11	rs1790474	1.19E+08	4.13E-01	N/A	15	rs7497104	69941054	6.21E-01	MYO9A	-	2.68E-07	9.83E-01	9.23E-05	3141	
3	rs6768946	28968511	1.89E-01	N/A	15	rs4842935	81431271	5.68E-01	HOMER2	-	2.68E-07	2.54E-01	1.16E-03	3142	
7	rs736198	13069584	5.91E-02	N/A	19	s1041173	62482800	4.72E-01	ZNF460	-	2.68E-07	6.30E-01	6.82E-06	3143	
6	rs4299797	87002427	8.52E-01	N/A	14	rs2023955	70340808	1.57E-02	MAP3K9	-	2.68E-07	6.61E-01	4.60E-04	3144	
6	rs9344586	87005974	8.52E-01	N/A	14	rs2023955	70340808	1.57E-02	MAP3K9	-	2.68E-07	6.61E-01	4.60E-04	3145	
6	rs13217719	1.24E+08	1.39E-01	NKAIN2	7	rs7777398	1.55E+08	6.14E-02	N/A	-	2.69E-07	1.52E-01	1.25E-06	3146	
5	rs1826675	62102994	2.07E-02	N/A	9	s1075857C	4197963	6.55E-01	GLIS3	-	2.69E-07	3.72E-01	2.98E-06	3147	
3	rs6801602	1.9E+08	8.85E-01	LPP	14	rs2332909	72233067	4.87E-01	DPF3	-	2.69E-07	4.70E-01	1.58E-03	3148	
2	rs10201495	2.36E+08	1.09E-01	N/A	22	rs1894605	34855927	1.50E-01	APOL3	-	2.69E-07	5.60E-02	4.79E-03	3149	
10	rs11015882	27901946	4.28E-01	N/A	13	s17591848	39642051	7.51E-01	N/A	-	2.69E-07	8.67E-01	6.15E-05	3150	
5	rs6860945	16600195	7.78E-01	FAM134B	9	rs940120	14896868	8.68E-01	FREM1	-	2.69E-07	9.25E-01	9.95E-05	3151	
6	rs7453313	9069974	4.28E-01	N/A	20	rs719563	7090997	2.75E-01	N/A	-	2.69E-07	4.48E-01	1.76E-05	3152	
14	rs1294552	90063039	2.68E-02	TTC7B	20	rs2143861	11127758	3.38E-01	N/A	-	2.69E-07	5.99E-01	1.11E-04	3153	
4	rs6448112	21678477	1.19E-01	N/A	7	s12673206	25212304	2.25E-02	NPVF	-	2.69E-07	8.50E-01	1.43E-04	3154	
12	rs9667991	93703993	2.43E-01	N/A	13	rs4771358	99694039	9.79E-01	PCCA	-	2.69E-07	4.01E-01	4.08E-06	3155	
12	rs9667991	93703993	2.43E-01	N/A	13	rs6491555	99701543	9.79E-01	PCCA	-	2.69E-07	4.92E-01	5.45E-06	3156	
1	rs7551015	18146429	8.73E-01	N/A	4	rs6533629	1.14E+08	6.65E-01	C4orf21	-	2.69E-07	6.78E-01	2.60E-04	3157	
3	rs13325052	1.49E+08	1.88E-01	N/A	13	rs9550222	1.12E+08	3.46E-01	N/A	-	2.69E-07	2.37E-01	2.36E-03	3158	
2	rs4663968	2.34E+08	5.73E-01	UGT1A10	14	s10137862	50518114	5.13E-01	TRIM9	-	2.69E-07	3.85E-01	6.97E-04	3159	
4	rs580399	1.5E+08	4.63E-01	N/A	5	rs9293829	80351685	8.49E-01	RASGRF2	-	2.69E-07	6.20E-01	1.19E-05	3160	
3	rs6791296	62406455	7.93E-01	CADPS	20	rs4811223	49742333	1.48E-01	ATP9A	-	2.69E-07	9.23E-02	4.54E-04	3161	
1	rs12022475	80075562	4.18E-02	N/A	10	s10795059	3582082	5.85E-01	N/A	-	2.69E-07	3.01E-01	5.84E-06	3162	
10	rs814628	90419632	7.73E-01	LIPF	21	rs2837682	40826362	6.19E-01	DSCAM	-	2.69E-07	1.79E-01	5.37E-04	3163	
13	rs9554473	97892903	8.40E-01	STK24	15	rs9646211	68162014	4.01E-01	TLE3	-	2.69E-07	7.02E-01	8.61E-06	3164	
4	rs17007266	1.43E+08	6.19E-01	N/A	13	rs622486	1.1E+08	1.61E-01	COL4A1	-	2.69E-07	1.99E-02	1.24E-02	3165	
6	rs9385259	1.23E+08	3.50E-01	PKIB	17	rs3744375	55504527	3.56E-01	HEATR6	-	2.69E-07	8.11E-01	2.75E-04	3166	
3	rs357161	13182536	8.23E-01	N/A	9	s10819994	1.04E+08	9.58E-01	N/A	-	2.70E-07	7.51E-02	6.62E-07	3167	
1	rs4908382	28156065	2.56E-01	EYA3	3	rs1992093	1.45E+08	9.41E-01	C3orf58	-	2.70E-07	8.24E-02	2.05E-03	3168	
7	rs334523	47532029	7.13E-01	TNS3	19	rs2035970	6932763	4.72E-01	EMR4P	-	2.70E-07	8.58E-02	4.23E-03	3169	
17	rs11654515	19736366	6.49E-01	AKAP10	18	rs2897347	72330759	1.71E-01	ZNF516	-	2.70E-07	4.63E-01	6.79E-05	3170	
3	rs500607	8156844	8.97E-01	N/A	9	rs6415812	98927782	6.88E-01	N/A	-	2.70E-07	N/A	N/A	3171	
2	rs12998820	2.03E+08	8.88E-01	N/A	10	rs584933	14435417	5.40E-01	N/A	-	2.70E-07	6.50E-03	9.17E-03	3172	
1	rs10914890	34478745	5.83E-01	N/A	8	rs4242345	1.25E+08	2.11E-01	ANXA13	-	2.70E-07	8.19E-02	2.10E-07	3173	
3	rs6776572	3068366	9.42E-01	IL5RA	17	rs925607	74892827	7.76E-01	HRNBP3	-	2.70E-07	4.85E-01	5.60E-04	3174	
1	rs2050656	2.36E+08	2.48E-01	RYR2	10	s12265891	56617236	2.96E-01	N/A	-	2.70E-07	7.98E-01	3.29E-05	3175	
18	rs7238168	32291551	4.02E-01	FHOD3	19	rs7246914	57362717	1.36E-01	ZNF836	-	2.70E-07	9.89E-01	1.20E-04	3176	
12	rs4149061	21241935	7.70E-01	SLCO1B1	21	rs6586245	41927730	2.66E-01	N/A	-	2.70E-07	3.95E-01	7.59E-06	3177	
3	rs833660	62527834	8.93E-02	CADPS	20	rs365516	14785965	7.92E-01	MACROD2	-	2.70E-07	9.23E-01	7.47E-05	3178	
5	rs3733989	1.68E+08	2.01E-01	ODZ2	18	rs2732229	71742290	5.44E-01	N/A	-	2.70E-07	2.03E-01	3.24E-06	3179	
7	rs17168036	14274731	2.43E-01	DGKB	16	s17137208	4706598	2.15E-01	C16orf71	-	2.70E-07	5.33E-01	3.50E-04	3180	
7	rs10951658	41755259	4.59E-01	LOC285954	17	rs2016126	70296527	2.16E-01	SLC9A3R1	-	2.70E-07	7.24E-01	8.88E-05	3181	
6	rs1156086	27295334	5.88E-01	N/A	15	rs813299	29225595	8.06E-01	N/A	MHC	2.70E-07	8.22E-02	1.86E-07	3182	
5	rs2968019	58380905	8.06E-01	PDE4D	18	rs4806	55146376	7.48E-02	LMAN1	-	2.71E-07	5.72E-01	7.97E-06	3183	
2	rs11894115	2.02E+08	6.70E-01	ALS2CR4	2	rs4851988	2.4E+08	7.37E-01	N/A	-	2.71E-07	4.77E-01	4.70E-06	3184	
3	rs9289423	1.33E+08	7.24E-01	N/A	9	rs1334069	3592018	4.85E-01	N/A	-	2.71E-07	5.60E-01	2.91E-06	3185	
2	rs6709267	1.16E+08	4.03E-01	DPP10	9	rs2006996	1.17E+08	2.03E-01	N/A	-	2.71E-07	8.81E-01	7.86E-05	3186	
1	rs1025653	2.15E+08	6.15E-01	ESRRG	4	rs6832923	1.56E+08	1.41E-01	N/A	-	2.71E-07	9.68E-01	4.55E-05	3187	
6	rs539298	1.61E+08	8.47E-02	SLC22A3	22	rs196067	37183623	7.63E-01	KDELR3	-	2.71E-07	3.62E-01	5.44E-04	3188	
4	rs8192049	1.41E+08	5.80E-02	MGST2	5	rs4554194	92536080	4.92E-01	N/A	-	2.71E-07	N/A	N/A	3189	
16	rs11644041	82873782	3.22E-01	WFDC1	22	rs3747225	43658162	1.69E-01	PHF21B	-	2.71E-07	2.24E-01	1.60E-03	3190	
2	rs4637047	1.92E+08	5.70E-01	N/A	11	rs7946750	70361142	8.51E-02	SHANK2	-	2.71E-07	3.83E-01	1.95E-05	3191	
1	rs2863991	1.07E+08	1.63E-01	N/A	7	rs6977428	53653312	3.71E-01	N/A	-	2.71E-07	5.61E-01	8.43E-05	3192	
8	rs6530946	16099299	8.70E-01	MSR1	11	rs7950814	71905497	2.40E-01	N/A	-	2.71E-07	4.00E-01	4.78E-04	3193	
5	rs1501727	33599372	3.76E-01	ADAMTS12	8	rs1907548	85324969	9.17E-01	RALYL	-	2.71E-07	6.81E-01	9.97E-05	3194	
18	rs2032165	8978701	6.78E-01	N/A	20	rs980984	43766466	9.79E-01	WFDC10B	-	2.71E-07	7.69E-01	5.40E-04	3195	
7	rs7789227	12089416	2.68E-01	N/A	7	rs1076303	71056394	3.08E-01	CALN1	-	2.71E-07	7.77E-01	3.82E-04	3196	
9	rs17195438	79320366	2.00E-01	GN/A14	14	s17126867	72861024	4.41E-01	NUMB	-	2.71E-07	1.12E-01	1.88E-03	3197	
9	rs10813978	33426471	5.45E-01	AQP3	12	rs7310294	1.06E+08	9.82E-01	N/A	-	2.71E-07	2.73E-01	1.38E-05	3198	
11	rs7131167	1.28E+08	6.56E-01	N/A	19	rs2033259	17557689	8.20E-01	GLT25D1	-	2.71E-07	7.14E-01	3.09E-04	3199	
4	rs16869740	20152890	4.66E-01	SLIT2	7	rs2054788	46218211	5.35E-01	N/A	-	2.71E-07	8.31E-01	1.63E-04	3200	
6	rs130067	31226490	8.62E-01	TCF19	6	rs7750269	31379136	8.58E-01	N/A	MHC	2.72E-07	4.47E-01	1.14E-05	3201	
5	rs2089192	1.7E+08	5.56E-01	KCNIP1	7	rs6467202	1.28E+08	4.41E-01	FAM71F2	-	2.72E-07	2.85E-01	3.67E-04	3202	
8	rs10503998	37046107	9.54E-01	N/A	12	rs2701129	50715744	4.59E-01	GRASP	-	2.72E-07	7.34E-01	2.08E-05	3203	
2	rs6745725	1.72E+08	7.67E-01	N/A	2	rs6434780	1.96E+08	6.88E-01	N/A	-	2.72E-07	7.34E-01	2.08E-05	3204	
11	rs10891585	1.13E+08	3.70E-02	TMPRSS5	11	s11827471	1.2E+08	3.90E-01	POU2F3	-	2.72E-07	6.66E-01	2.21E-04	3205	
1	rs6693292	67795756	4.96E-01	N/A	12	s11168459	46882508	6.23E-01	OR10AD1	-	2.72E-07	6.89E-01	2.08E-04	3206	
11	rs11214696	1.13E+08	3.70E-02	TMPRSS5	11	s11827471	1.2E+08	3.90E-01	POU2F3	-	2.72E-07	8.91E-01	1.96E-04	3207	
1	rs1340592	67793691	4.96E-01	N/A	12	s11168459	46882508	6.23E-01	OR10AD1	-	2.72E-07	9.58E-01	3.06E-05	3208	
1	rs4655733	67798226	4.96E-01	N/A	12	s10875770	46886787	6.23E-01	OR10AD1	-	2.72E-07	9.58E-01	3.06E-05	3209	
1	rs4655733	67798226	4.96E-01	N/A	12	s10875772	46895820	6.23E-01	OR10AD1	-	2.72E-07	9.58E-01	3.06E-05	3210	
8	rs9649891	37062813	9.54E-01	N/A	12	rs2701129	50715744	4.59E-01	GRASP	-	2.72E-07	9.84E-01	3.71E-05	3211	
1	rs1340592	67793691	4.96E-01	N/A	12	s10875770	46886787	6.23E-01	OR10AD1	-	2.72E-07	9.84E-01	3.71E-05	3212	
1	rs1340592	67793691	4.96E-01	N/A	12	s10875772	46895820	6.23E-01	OR10AD1	-	2.72E-07	9.84E-01	3.71E-05	3213	
1	rs4655733	67798226	4.96E-01	N/A	12	s11168459	46882508	6.23E-01	OR10AD1	-	2.72E-07	9.84E-01	3.71E-05	3214	
1	rs6662607	67794090	4.96E-01	N/A	12	s10875770	46886787	6.23E-01	OR10AD1	-	2.72E-07	9.84E-01	3.71E-05	3215	
1	rs6662607	67794090	4.96E-01	N/A	12	s10875772	46895820	6.23E-01	OR10AD1	-	2.72E-07	9.84E-01	3.71E-05	3216	
1	rs6662607	67794090	4.96E-01	N/A	12	s11168459	46882508	6.23E-01	OR10AD1	-	2.72E-07	9.84E-01	3.71E-05	3217	
1	rs6693292	67795756	4.96E-01	N/A	12	s10875770	46886787	6.23E-01	OR10AD1	-	2.72E-07	9.84E-01	3.71E-05	3218	
1	rs6693292	67795756	4.96E-01	N/A	12	s10875772	46895820	6.23E-01	OR10AD1	-	2.72E-07	9.84E-01	3.71E-05	3219	
8	rs7828550	82813796	9.93E-01	N/A	17	rs7208536	76364381	4.91E-01	RPTOR	-	2.72E-07	1.59E-01	3.89E-03	3220	
15	rs563940	41986601	4.96E-01	FRMD5	16	rs9933029	66860911	4.02E-01	PLA2G15	-	2.72E-07	2.15E-01	2.28E-06	3221	
1	rs1260773	64307510	2.49E-01	ROR1	9	rs4606122	73376130	5.42E-01	N/A	-	2.72E-07	4.36E-01	8.66E-06	3222	
8	rs10954922	33649641	2.34E-02	N/A	16	rs130021	3772472	7.22E-01	CREBBP	-	2.72E-07	8.48E-01	6.14E-05	3223	
1	rs6683927	2.22E+08	5.68E-02	N/A	10	rs7093694	1.35E+08	7.66E-01	GPR123	-	2.72E-07	1.34E-02	8.92E-03	3224	
4	rs1982202	1.62E+08	3.76E-01	N/A	20	rs6136735	19375522	5.41E-01	SLC24A3	-	2.72E-07	5.33E-01	9.84E-05	3225	
1	rs9729649	5494891	8.59E-01	N/A	7	s12536225	16892608	1.73E-01	AGR3	-	2.72E-07	2.93E-01	2.15E-04	3226	
4	rs11732223	1.54E+08	7.67E-01	ARFIP1	14	rs4983413	1.05E+08	3.35E-01	MTA1	-	2.72E-07	6.90E-01	7.25E-04	3227	
10	rs898716	14165659	4.31E-01	FRMD4A	12	rs2263487	45537094	2.68E-02	N/A	-	2.72E-07	7.16E-01	7.08E-05	3228	
2	rs2068801	17981411	6.34E-01	KCNS3	3	rs6809730	68678897	2.43E-01	FAM19A1	-	2.72E-07	8.34E-01	6.69E-05	3229	
2	rs6737230	1.61E+08	8.97E-01	N/A	8	rs7010864	58947443	3.33E-02	N/A	-	2.72E-07	4.17E-01	3.62E-06	3230	
2	rs2292834	1.44E+08	3.05E-01	ARHGAP15	9	s10816959	1.12E+08	2.82E-01	TXNDC8	-	2.72E-07	6.26E-01	7.35E-06	3231	
3	rs7373528	37725628	8.99E-02	ITGA9	3	s16846607	1.05E+08	1.09E-01	N/A	-	2.72E-07	7.04E-01	6.59E-05	3232	
3	rs1284746	1.04E+08	4.76E-01	N/A	13	rs9534986	47665333	6.35E-01	N/A	-	2.72E-07	2.77E-01	2.83E-06	3233	
3	rs1356106	1.04E+08	4.76E-01	N/A	13	rs9534986	47665333	6.35E-01	N/A	-	2.72E-07	2.77E-01	2.83E-06	3234	
4	rs7682286	7471476	2.08E-01	SORCS2	14	rs6575528	95133483	1.34E-02	N/A	-	2.72E-07	3.84E-01	1.73E-06	3235	
11	rs1025438	8862037	1.09E-01	ST5	13	rs1289556	1.01E+08	7.33E-03	N/ALCN	-	2.73E-07	4.96E-01	9.09E-06	3236	
2	rs17404655	76770070	2.26E-01	N/A	14	rs4983213	26555027	4.09E-02	N/A	-	2.73E-07	5.37E-01	2.04E-03	3237	
11	rs1025438	8862037	1.09E-01	ST5	13	rs9805515	1.01E+08	7.33E-03	N/ALCN	-	2.73E-07	5.74E-01	1.19E-05	3238	
13	rs1041052	25417045	2.66E-01	ATP8A2	13	rs9529874	70831653	2.82E-01	N/A	-	2.73E-07	1.10E-01	7.38E-07	3239	
14	rs11624899	95730089	6.42E-01	BDKRB2	20	rs8120755	44404617	3.83E-01	SLC35C2	-	2.73E-07	1.95E-01	3.90E-03	3240	
9	rs11145032	78538013	2.46E-01	PRUNE2	11	s11026477	22234428	1.81E-01	ANO5	-	2.73E-07	5.07E-01	6.78E-06	3241	
3	rs13097576	1.91E+08	4.75E-01	TP63	17	rs7207422	28478501	8.29E-02	ACCN1	-	2.73E-07	6.06E-01	2.03E-05	3242	
5	rs4348193	77671476	5.91E-01	N/A	11	s11236042	73661102	3.63E-01	PPME1	-	2.73E-07	8.82E-01	4.42E-05	3243	
2	rs1834619	17764966	5.69E-01	SMC6	8	s11992870	34734996	7.10E-01	N/A	-	2.73E-07	2.95E-02	1.44E-02	3244	
1	rs10493583	76343514	9.55E-01	ST6GALN/AC3	18	s12962764	65388622	7.65E-02	DOK6	-	2.73E-07	3.22E-01	1.37E-03	3245	
6	rs2874759	80449007	7.52E-01	SH3BGRL2	7	rs2692136	1.47E+08	1.19E-01	CNTN/AP2	-	2.73E-07	4.51E-01	4.90E-04	3246	

SNP A					SNP B						Interaction P			Ranking	Cluster in top 100 interactions
CHR	SNP	Location	gle locus P va	Gene	CHR	SNP	Location	ingle locus P valu	Gene	MHC region	Stage 1	Stage 2	Combined		
2	rs4485562	2.34E+08	6.39E-01	UGT1A10	9	s10814680	38345452	7.63E-02	N/A	-	2.73E-07	7.32E-01	8.83E-06	3247	
3	rs2130369	1.01E+08	7.50E-01	C3orf26	8	rs2014286	16951496	5.93E-01	EFHA2	-	2.73E-07	8.94E-01	7.81E-06	3248	
3	rs17020557	2819653	3.47E-01	CNTN4	12	rs2722175	1.03E+08	2.80E-01	GLT8D2	-	2.73E-07	N/A	N/A	3249	
12	rs10128762	72968165	7.81E-01	N/A	21	rs2824447	17987327	2.47E-01	N/A	-	2.73E-07	1.56E-03	4.24E-08	3250	
8	rs6995339	62099853	6.64E-01	N/A	22	rs3788535	36863991	9.84E-02	PLA2G6	-	2.73E-07	9.32E-01	1.65E-05	3251	
8	rs6995339	62099853	6.64E-01	N/A	22	rs4820315	36848677	9.84E-02	PLA2G6	-	2.73E-07	9.32E-01	1.65E-05	3252	
8	rs6995339	62099853	6.64E-01	N/A	22	rs2076118	36859446	9.84E-02	PLA2G6	-	2.73E-07	N/A	N/A	3253	
4	rs2385895	8068659	2.36E-01	ABLIM2	11	s10832368	15132561	3.47E-01	INSC	-	2.73E-07	4.29E-01	6.81E-06	3254	
6	rs4495304	31188697	4.80E-02	C6orf15	6	rs3130534	31317024	3.50E-01	N/A	MHC	2.73E-07	1.21E-01	1.04E-06	3255	
6	rs7745656	32788948	1.70E-01	N/A	10	rs7913408	52265121	2.80E-01	A1CF	MHC	2.74E-07	6.04E-02	1.74E-03	3256	
3	rs6444269	1.89E+08	9.23E-01	N/A	8	rs2334792	1.33E+08	2.80E-01	EFR3A	-	2.74E-07	1.08E-01	2.19E-03	3257	
4	rs2218313	1.43E+08	2.64E-02	N/A	5	rs4274968	1.48E+08	6.89E-01	HTR4	-	2.74E-07	6.72E-01	1.57E-04	3258	
4	rs1824864	22214157	6.39E-01	N/A	16	rs9922832	49001660	1.60E-01	N/A	-	2.74E-07	8.29E-01	7.59E-05	3259	
7	rs17166689	13068758	6.44E-02	N/A	19	s10411733	62482800	4.72E-01	ZNF460	-	2.74E-07	N/A	N/A	3260	
1	rs12120863	89274110	9.04E-02	GBP1	13	rs2321997	58561359	4.61E-01	N/A	-	2.74E-07	1.55E-01	5.92E-07	3261	
12	rs17624000	39698479	2.97E-02	CNTN1	18	rs168206	3683772	8.93E-01	DLGAP1	-	2.74E-07	4.14E-01	2.69E-05	3262	
6	rs6935051	1.5E+08	5.88E-01	RAET1L	3	rs9866473	1.73E+08	6.06E-01	PLD1	-	2.74E-07	8.04E-01	4.38E-05	3263	
9	rs11999361	1.18E+08	1.84E-01	PAPPA	17	rs9901111	28070943	8.78E-01	MYO1D	-	2.74E-07	8.32E-01	1.55E-05	3264	
2	rs2118836	96526699	8.13E-01	NEURL3	8	rs2705034	17370850	2.09E-01	N/A	-	2.74E-07	9.78E-01	4.02E-05	3265	
8	rs10504091	50923329	7.21E-01	N/A	20	rs6014949	55186217	1.65E-01	BMP7	-	2.74E-07	8.33E-02	4.29E-07	3266	
8	rs13255117	50943966	7.21E-01	N/A	20	rs6014949	55186217	1.65E-01	BMP7	-	2.74E-07	9.60E-02	1.80E-03	3267	
6	rs1739671	71103938	8.00E-01	N/A	10	rs7904018	7834300	6.41E-01	KIN	-	2.74E-07	1.07E-01	1.50E-03	3268	
11	rs11036893	42422682	8.93E-01	N/A	15	rs598729	55848087	9.06E-01	N/A	-	2.74E-07	7.65E-01	2.47E-05	3269	
10	rs2420724	1.22E+08	1.21E-02	PPAPDC1A	11	s10896271	55870151	4.79E-01	OR8K1	-	2.74E-07	8.62E-01	4.35E-05	3270	
10	rs2420724	1.22E+08	1.21E-02	PPAPDC1A	11	s10896272	55870169	4.79E-01	OR8K1	-	2.74E-07	8.62E-01	4.35E-05	3271	
10	rs2420724	1.22E+08	1.21E-02	PPAPDC1A	11	rs1945205	55943006	4.79E-01	OR8U8	-	2.74E-07	8.62E-01	4.35E-05	3272	
10	rs2420724	1.22E+08	1.21E-02	PPAPDC1A	11	rs1945222	55923662	4.79E-01	OR8U8	-	2.74E-07	8.62E-01	4.35E-05	3273	
11	rs454465	5445416	1.81E-01	OR51I2	13	s17058611	60173532	5.84E-01	N/A	-	2.74E-07	N/A	N/A	3274	
10	rs7907344	10998527	5.91E-01	LOC254312	10	rs3814148	97061354	3.64E-01	SORBS1	-	2.74E-07	5.73E-01	7.36E-06	3275	
6	rs6921980	71096916	8.00E-01	N/A	10	rs7904018	7834300	6.41E-01	KIN	-	2.74E-07	8.31E-01	1.15E-04	3276	
6	rs696988	71095665	8.00E-01	N/A	10	rs7904018	7834300	6.41E-01	KIN	-	2.74E-07	8.31E-01	1.15E-04	3277	
8	rs4831766	15641064	5.83E-01	TUSC3	22	rs735455	20743793	9.48E-01	N/A	-	2.74E-07	N/A	N/A	3278	
17	rs10521258	13954919	4.46E-01	COX10	21	rs2236431	35335609	4.65E-01	RUNX1	-	2.74E-07	2.26E-03	1.34E-01	3279	
6	rs2322841	81319707	6.20E-01	N/A	2	s10930467	1.72E+08	2.77E-01	N/A	-	2.74E-07	2.08E-01	4.44E-07	3280	
6	rs6903716	22064383	7.82E-01	FLJ22536	22	rs3827281	16964433	4.11E-01	TUBA8	-	2.74E-07	4.55E-01	1.19E-05	3281	
2	rs2861680	79006430	1.64E-01	N/A	11	rs1397056	56177773	4.24E-01	OR8U8	-	2.74E-07	1.26E-02	1.19E-02	3282	
9	rs12551990	77303509	6.87E-01	MIR548H3	13	rs7999126	86769337	9.01E-01	N/A	-	2.74E-07	6.26E-01	1.88E-04	3283	
6	rs4495304	31188697	4.80E-02	C6orf15	6	rs1265156	31250276	6.76E-01	TCF19	MHC	2.74E-07	1.76E-01	1.06E-05	3284	
5	rs1345592	1.69E+08	1.10E-01	SLIT3	9	rs1444826	74536439	2.27E-02	TMC1	-	2.74E-07	3.62E-01	6.62E-06	3285	
7	rs4543487	82641622	3.47E-01	PCLO	12	s10506198	40260625	2.23E-01	PDZRN4	-	2.74E-07	5.64E-01	1.06E-04	3286	
3	rs11720652	14636424	8.72E-01	N/A	3	rs4678787	33248482	1.70E-01	SUSD5	-	2.74E-07	6.05E-01	1.45E-04	3287	
4	rs2132834	91787002	9.39E-01	FAM190A	17	rs8066993	75894625	5.64E-01	RNF213	-	2.74E-07	8.03E-01	4.55E-05	3288	
6	rs6940982	81826357	2.92E-01	N/A	5	rs4703613	73144329	3.07E-01	N/A	-	2.75E-07	6.59E-01	1.76E-05	3289	
3	rs4684686	10395875	7.32E-01	ATP2B2	3	rs2173911	1.92E+08	4.07E-01	LOC647309	-	2.75E-07	7.95E-01	5.12E-05	3290	
6	rs957387	15429838	7.84E-01	JARID2	21	rs2223006	41035292	4.42E-01	DSCAM	-	2.75E-07	1.75E-01	1.89E-03	3291	
1	rs9436816	68619836	3.94E-01	N/A	20	rs1926076	54314184	7.18E-01	N/A	-	2.75E-07	2.02E-01	1.31E-03	3292	
2	rs897877	2.19E+08	6.02E-01	PNKD	4	rs2292837	1.4E+08	2.54E-01	ELF2	-	2.75E-07	3.25E-01	8.98E-04	3293	
3	rs1471217	48359899	8.17E-01	SPINK8	5	rs1366397	23476753	2.74E-01	N/A	-	2.75E-07	1.75E-01	3.52E-06	3294	
6	rs4960016	4633860	2.29E-01	CDYL	2	rs1368056	1.38E+08	7.82E-01	THSD7B	-	2.75E-07	5.70E-01	5.69E-04	3295	
10	rs11595366	11454821	7.08E-01	N/A	11	rs2186598	1.05E+08	9.64E-01	GRIA4	-	2.75E-07	9.17E-01	1.19E-04	3296	
2	rs6746998	2.09E+08	1.89E-01	N/A	7	rs1989886	20549647	1.18E-01	N/A	-	2.75E-07	6.81E-01	1.91E-04	3297	
7	rs1723593	1.1E+08	5.08E-01	N/A	17	s11651333	11799674	1.96E-02	DN/AH9	-	2.75E-07	1.72E-01	4.27E-04	3298	
14	rs2282277	44017413	1.65E-01	N/A	14	rs7156399	95104682	7.38E-01	N/A	-	2.75E-07	2.85E-01	1.80E-04	3299	
7	rs17166711	13075823	7.09E-02	N/A	19	rs3746228	62496174	5.82E-01	ZNF460	-	2.75E-07	5.83E-01	6.75E-06	3300	
7	rs12533128	13071663	7.09E-02	N/A	19	rs3746228	62496174	5.82E-01	ZNF460	-	2.75E-07	6.57E-01	1.02E-05	3301	
7	rs7778887	13071534	7.09E-02	N/A	19	rs3746228	62496174	5.82E-01	ZNF460	-	2.75E-07	6.57E-01	1.02E-05	3302	
9	rs7029669	1.09E+08	4.90E-01	N/A	10	s11187969	96221159	4.96E-01	TBC1D12	-	2.75E-07	8.57E-01	5.40E-04	3303	
3	rs9830749	1.17E+08	3.37E-01	LSAMP	8	rs6984840	10169975	9.13E-01	MSRA	-	2.76E-07	5.50E-01	2.32E-04	3304	
14	rs10148477	61340572	9.87E-01	SN/APC1	17	s10775405	42543089	8.16E-01	CDC27	-	2.76E-07	5.96E-01	7.04E-05	3305	
1	rs4908382	28156065	2.56E-01	EYA3	3	rs1354496	1.45E+08	8.35E-01	N/A	-	2.76E-07	3.09E-02	3.82E-03	3306	
8	rs879638	41770625	1.78E-01	ANK1	9	s16925271	7056807	3.11E-01	KDM4C	-	2.76E-07	1.55E-01	1.52E-03	3307	
2	rs925751	1.8E+08	9.55E-01	ZNF385B	3	rs358741	1.57E+08	8.76E-01	C3orf33	-	2.76E-07	2.31E-01	4.83E-04	3308	
3	rs17220622	46554129	2.14E-01	LRRC2	12	s11058480	1.22E+08	3.49E-01	N/A	-	2.76E-07	5.59E-01	3.92E-05	3309	
2	rs6755940	1.61E+08	1.47E-01	RBMS1	19	rs8100011	49128573	3.05E-01	ZNF45	-	2.76E-07	6.19E-01	7.27E-05	3310	
3	rs12485670	67534807	6.83E-01	N/A	13	rs1157851	76205508	6.26E-01	N/A	-	2.76E-07	6.38E-01	1.15E-05	3311	
1	rs3738372	2.22E+08	6.49E-01	N/A	7	rs2402056	1.16E+08	1.89E-01	TES	-	2.76E-07	4.17E-01	3.42E-04	3312	
12	rs2955503	22090132	7.55E-01	CMAS	19	rs1008076	3919605	7.65E-01	EEF2	-	2.76E-07	6.16E-01	7.49E-06	3313	
12	rs264514	1.29E+08	5.54E-01	TMEM132D	19	rs352493	4131836	4.10E-01	ANKRD24	-	2.76E-07	3.01E-01	2.98E-03	3314	
1	rs1501509	1.89E+08	3.10E-01	FAM5C	15	rs8040756	53585891	1.08E-01	DYX1C1	-	2.76E-07	2.96E-01	1.99E-05	3315	
8	rs2142200	1.29E+08	3.16E-01	N/A	16	rs1657091	8902915	5.57E-01	USP7	-	2.77E-07	2.04E-01	2.23E-03	3316	
10	rs1751291	4482612	2.98E-01	N/A	16	rs4782717	81167218	2.78E-01	N/A	-	2.77E-07	2.54E-01	5.78E-07	3317	
6	rs1408913	1.65E+08	3.43E-01	N/A	10	s11101460	49638602	2.64E-01	WDFY4	-	2.77E-07	5.89E-01	1.41E-04	3318	
1	rs6702842	1.5E+08	1.51E-01	SNX27	5	rs1553544	1.19E+08	1.53E-01	DMXL1	-	2.77E-07	5.92E-01	3.28E-05	3319	
6	rs9376020	1.35E+08	5.91E-01	SGK1	19	rs919275	7212441	4.14E-01	INSR	-	2.77E-07	9.20E-01	1.11E-04	3320	
10	rs3750749	50354234	2.12E-01	ERCC6	12	rs763948	66442193	9.93E-01	N/A	-	2.77E-07	9.79E-01	6.84E-05	3321	
1	rs4908382	28156065	2.56E-01	EYA3	19	rs2290669	16356774	8.42E-01	EPS15L1	-	2.77E-07	7.30E-01	1.10E-05	3322	
8	rs4737435	57561106	2.29E-01	N/A	12	s10779113	83536444	6.09E-01	N/A	-	2.77E-07	6.48E-01	1.84E-05	3323	
10	rs10883931	1.06E+08	4.09E-01	SH3PXD2A	21	rs2836028	38161920	1.79E-01	KCNJ6	-	2.77E-07	4.23E-01	4.36E-04	3324	
6	rs6928944	1.64E+08	6.31E-01	N/A	14	rs9323964	96724402	2.66E-01	N/A	-	2.77E-07	8.20E-01	4.40E-05	3325	
4	rs9997597	1.75E+08	7.28E-01	N/A	21	rs2212870	19863784	2.10E-02	N/A	-	2.77E-07	N/A	N/A	3326	
14	rs7141906	78737063	7.62E-01	NRXN3	21	rs150792	42334322	1.02E-01	C21orf121	-	2.77E-07	3.69E-01	9.58E-06	3327	
6	rs1331644	1.46E+08	3.38E-01	GRM1	10	rs1318662	26860966	6.27E-01	APBB1IP	-	2.77E-07	8.76E-01	1.73E-04	3328	
8	rs1393978	1.07E+08	9.64E-01	N/A	11	rs2370643	1.21E+08	2.16E-01	N/A	-	2.77E-07	1.32E-02	3.22E-08	3329	
5	rs2526150	1.35E+08	5.12E-01	FBXL21	7	rs4357206	5369132	4.48E-01	TNRC18	-	2.77E-07	6.82E-01	1.40E-04	3330	
2	rs1568120	1.35E+08	3.72E-02	TMEM163	13	rs9506820	21758453	5.80E-01	N/A	-	2.78E-07	7.11E-01	7.68E-04	3331	
2	rs1949914	6113298	7.25E-01	N/A	11	rs1945921	1.3E+08	2.19E-01	N/A	-	2.78E-07	8.13E-01	3.19E-05	3332	
2	rs6705916	1.35E+08	3.72E-02	TMEM163	13	rs9506820	21758453	5.80E-01	N/A	-	2.78E-07	8.40E-01	5.11E-04	3333	
6	rs397139	1.33E+08	4.13E-01	N/A	16	rs1566458	83576432	5.21E-01	ZDHHC7	-	2.78E-07	8.90E-02	4.05E-03	3334	
6	rs525153	1.33E+08	4.13E-01	N/A	16	rs1566458	83576432	5.21E-01	ZDHHC7	-	2.78E-07	1.06E-01	3.38E-03	3335	
3	rs2350279	1.41E+08	3.47E-02	CLSTN2	4	rs6832992	42456190	4.21E-01	N/A	-	2.78E-07	1.39E-01	1.18E-06	3336	
2	rs868251	1.02E+08	6.04E-01	N/A	4	rs4692990	94891726	1.44E-01	GRID2	-	2.78E-07	2.23E-01	3.73E-06	3337	
4	rs2570096	58820719	5.52E-01	N/A	9	s10868806	72143304	3.29E-01	SMC5	-	2.78E-07	8.01E-01	1.32E-05	3338	
2	rs13002663	1.45E+08	2.06E-01	ZEB2	17	s12451200	18719218	2.22E-01	PRPSAP2	-	2.78E-07	8.13E-01	1.35E-05	3339	
2	rs13002663	1.45E+08	2.06E-01	ZEB2	17	rs4393623	18716625	2.22E-01	PRPSAP2	-	2.78E-07	8.13E-01	1.35E-05	3340	
3	rs10865655	70940116	2.58E-01	N/A	20	rs6026169	56288487	1.95E-01	PPP4R1L	-	2.78E-07	9.95E-01	2.89E-05	3341	
10	rs431673	90484145	5.54E-01	LIPK	21	rs2837682	40826362	6.19E-01	DSCAM	-	2.78E-07	4.11E-01	6.53E-05	3342	
5	rs3828699	1.26E+08	5.49E-01	LMNB1	19	rs245111	55220709	4.22E-02	ZNF473	-	2.78E-07	8.63E-01	3.18E-05	3343	
6	rs2524123	31373293	7.02E-01	N/A	19	rs312181	52025044	1.96E-01	AP2S1	MHC	2.78E-07	8.29E-01	2.15E-05	3344	
1	rs863455	1.66E+08	1.66E-02	CD247	4	s17615522	38204296	7.12E-01	N/A	-	2.79E-07	2.98E-03	2.94E-02	3345	
2	rs6546862	73713856	4.42E-01	N/AT8	5	rs6892570	8279339	1.71E-01	N/A	-	2.79E-07	4.70E-01	1.36E-05	3346	
2	rs13407109	2.18E+08	9.06E-02	TNS1	12	rs1245819	78166488	8.71E-01	SYT1	-	2.79E-07	5.26E-01	6.43E-06	3347	
2	rs13407109	2.18E+08	9.06E-02	TNS1	12	rs1268463	78167625	8.71E-01	SYT1	-	2.79E-07	5.26E-01	6.43E-06	3348	
8	rs7822457	1.21E+08	7.84E-01	ENPP2	9	s12346753	1.12E+08	3.59E-01	SVEP1	-	2.79E-07	7.02E-01	1.82E-05	3349	
1	rs4951488	2.09E+08	9.37E-01	HHAT	20	rs6044850	17425751	3.78E-02	PCSK2	-	2.79E-07	4.03E-02	2.80E-08	3350	
5	rs4242215	1.65E+08	6.13E-01	N/A	22	rs2587113	16708503	1.18E-01	MICAL3	-	2.79E-07	3.58E-01	1.29E-06	3351	
6	rs942723	1.65E+08	8.26E-01	N/A	5	rs3857397	71269359	8.70E-01	N/A	-	2.79E-07	6.86E-01	7.06E-06	3352	
8	rs12550138	1.07E+08	9.62E-01	N/A	11	rs2370643	1.21E+08	2.16E-01	N/A	-	2.79E-07	1.08E-02	2.64E-08	3353	
5	rs7446783	38042733	1.54E-01	N/A	11	s10751670	934466	7.32E-01	AP2A2	-	2.79E-07	3.28E-01	4.61E-04	3354	
4	rs6852233	32440679	5.49E-01	N/A	5	rs2921571	5592894	6.48E-01	N/A	-	2.79E-07	6.62E-01	2.25E-05	3355	
10	rs10740809	30098021	4.47E-01	N/A	10	s10826831	30958430	9.75E-01	LYZL2	-	2.79E-07	4.69E-02	2.40E-07	3356	
5	rs6874186	71512871	4.97E-01	MAP1B	14	rs2277483	23661732	2.47E-01	DCAF11	-	2.79E-07	6.61E-01	2.84E-05	3357	
6	rs2233984	31187243	4.20E-02	C6orf15	6	rs3130453	31232828	5.90E-01	TCF19	MHC	2.79E-07	3.67E-01	4.04E-06	3358	

SNP A					SNP B						Interaction P			Ranking	Cluster in top 100 interactions
CHR	SNP	Location	gle locus P va	Gene	CHR	SNP	Location	Single locus P valu	Gene	MHC region	Stage 1	Stage 2	Combined		
6	rs130067	31226490	8.62E-01	TCF19	6	rs9368675	31380136	7.91E-01	N/A	MHC	2.79E-07	2.15E-01	2.30E-06	3359	
3	rs7641916	1.4E+08	8.44E-01	FOXL2	3	rs7641416	1.92E+08	5.75E-01	N/A	-	2.80E-07	6.76E-01	7.02E-05	3360	
3	rs7641916	1.4E+08	8.44E-01	FOXL2	3	rs2363961	1.92E+08	5.75E-01	N/A	-	2.80E-07	6.97E-01	7.62E-05	3361	
2	rs272118	1.31E+08	3.39E-01	FAM123C	15	rs4932557	89919267	5.50E-01	N/A	-	2.80E-07	9.37E-01	2.59E-05	3362	
3	rs2581618	1.44E+08	9.56E-01	N/A	8	rs6996111	1.21E+08	1.55E-01	N/A	-	2.80E-07	4.72E-01	6.70E-06	3363	
5	rs1345592	1.69E+08	1.10E-01	SLIT3	9	rs1796993	74505258	4.66E-02	TMC1	-	2.80E-07	6.06E-01	9.31E-05	3364	
5	rs1345592	1.69E+08	1.10E-01	SLIT3	9	rs2501917	74505943	4.66E-02	TMC1	-	2.80E-07	6.06E-01	9.31E-05	3365	
5	rs1345592	1.69E+08	1.10E-01	SLIT3	9	rs2793150	74509273	4.66E-02	TMC1	-	2.80E-07	6.06E-01	9.31E-05	3366	
7	rs11764649	12537614	3.90E-01	N/A	19	rs735386	13225932	3.41E-01	CACN/A1A	-	2.80E-07	2.90E-01	5.27E-04	3367	
2	rs16846100	2.12E+08	9.37E-01	ERBB4	3	rs4677287	73598809	1.07E-01	PDZRN3	-	2.80E-07	8.21E-01	5.38E-05	3368	
1	rs6540817	2.13E+08	6.69E-01	SMYD2	13	s12584299	1.08E+08	7.00E-02	N/A	-	2.80E-07	4.84E-01	2.70E-04	3369	
1	rs6540819	2.13E+08	6.69E-01	SMYD2	13	s12584299	1.08E+08	7.00E-02	N/A	-	2.80E-07	4.84E-01	2.70E-04	3370	
5	rs1551050	16586551	9.95E-01	FAM134B	9	rs940120	14896868	8.68E-01	FREM1	-	2.80E-07	9.15E-01	5.52E-05	3371	
7	rs7802654	93731669	6.96E-01	N/A	18	rs273717	21420569	6.23E-01	N/A	-	2.80E-07	4.51E-01	1.52E-05	3372	
6	rs1161761	1112422	7.67E-01	N/A	7	s17172297	54147376	3.01E-01	N/A	-	2.80E-07	6.59E-01	1.33E-05	3373	
9	rs12235312	3874879	9.00E-02	GLIS3	15	rs7172407	71637454	6.54E-01	C15orf60	-	2.80E-07	2.18E-01	8.82E-06	3374	
4	rs6816078	1.47E+08	6.65E-01	ZNF827	8	s13260791	1105146	5.19E-01	N/A	-	2.81E-07	7.03E-01	2.02E-05	3375	
5	rs1865508	1.05E+08	1.73E-01	N/A	12	s10506439	61930601	4.64E-01	N/A	-	2.81E-07	9.12E-01	2.24E-05	3376	
4	rs13152512	1.66E+08	9.00E-01	N/A	12	s12810632	43268514	2.54E-01	NELL2	-	2.81E-07	1.52E-01	1.09E-06	3377	
1	rs6680751	2.38E+08	8.03E-01	FMN2	18	rs1646613	31580770	2.17E-01	N/A	-	2.81E-07	2.41E-01	5.70E-04	3378	
2	rs744556	1.9E+08	7.77E-01	WDR75	8	rs4921712	20314771	2.94E-01	N/A	-	2.81E-07	7.13E-01	3.62E-05	3379	
2	rs10192133	1.61E+08	8.05E-01	N/A	8	rs7010864	58947443	3.33E-02	N/A	-	2.81E-07	N/A	N/A	3380	
4	rs1880716	83866763	6.59E-01	SCD5	12	rs3819526	2306783	4.68E-01	CACN/A1C	-	2.81E-07	7.49E-01	2.64E-05	3381	
6	rs199090	23641601	3.11E-01	N/A	21	rs2836524	38867699	2.31E-01	ERG	-	2.81E-07	8.01E-01	1.36E-05	3382	
10	rs1262099	1.08E+08	1.81E-01	N/A	15	s17116917	21465730	3.33E-01	NDN	-	2.81E-07	1.48E-01	5.12E-07	3383	
10	rs1418396	87690805	5.61E-01	GRID1	18	rs1511939	34221480	5.42E-01	N/A	-	2.81E-07	7.06E-01	1.61E-04	3384	
7	rs7811880	70344437	5.47E-01	WBSCR17	18	rs4939970	46195712	4.86E-01	N/A	-	2.82E-07	6.01E-02	4.16E-03	3385	
3	rs2046227	1.02E+08	6.40E-01	GPR128	10	rs947403	1006601	8.95E-01	GTPBP4	-	2.82E-07	8.32E-02	1.77E-07	3386	
7	rs6974582	70343390	5.47E-01	WBSCR17	18	rs4939970	46195712	4.86E-01	N/A	-	2.82E-07	N/A	N/A	3387	
4	rs975777	15927164	2.23E-01	N/A	12	rs7968452	23653255	2.09E-01	SOX5	-	2.82E-07	7.16E-01	1.65E-05	3388	
2	rs1000582	1.69E+08	6.52E-01	N/A	5	rs2548373	42963857	9.66E-02	N/A	-	2.82E-07	1.31E-01	2.45E-03	3389	
6	rs4946936	1.09E+08	2.98E-01	FOXO3	13	rs1105576	99178430	6.33E-01	CLYBL	-	2.82E-07	6.01E-01	2.14E-03	3390	
8	rs39503	90848944	1.30E-01	RIPK2	17	s10451228	73195845	8.35E-02	N/A	-	2.82E-07	9.34E-02	1.33E-06	3391	
8	rs447618	90854822	1.30E-01	RIPK2	17	s10451228	73195845	8.35E-02	N/A	-	2.82E-07	9.34E-02	1.33E-06	3392	
7	rs7792262	23882268	8.84E-01	N/A	20	rs726207	15962926	6.80E-01	MACROD2	-	2.82E-07	5.32E-01	4.68E-04	3393	
4	rs6812217	1.59E+08	4.71E-01	N/A	10	rs1930494	59537766	9.24E-01	N/A	-	2.82E-07	3.54E-01	2.35E-06	3394	
7	rs205764	1.3E+08	9.73E-01	N/A	16	s12444248	51987801	1.87E-01	N/A	-	2.83E-07	9.29E-01	3.46E-05	3395	
7	rs2158629	4334226	1.19E-01	N/A	14	rs2238233	71761360	6.63E-01	RGS6	-	2.83E-07	2.46E-01	4.63E-04	3396	
2	rs4954160	1.35E+08	3.71E-02	TMEM163	13	rs9506820	21758453	5.80E-01	N/A	-	2.83E-07	6.10E-01	9.68E-04	3397	
1	rs807247	25993456	5.33E-01	MAN1C1	11	s11225049	1.01E+08	8.75E-01	ANGPTL5	-	2.83E-07	4.43E-01	2.13E-05	3398	
6	rs1006012	53966408	8.93E-01	N/A	1	rs4653960	2.27E+08	9.98E-02	HIST3H2A	-	2.83E-07	8.75E-01	1.09E-04	3399	
2	rs6432974	1.68E+08	4.29E-01	XIRP2	19	rs889362	18619255	5.85E-02	KLHL26	-	2.83E-07	7.64E-02	1.15E-03	3400	
8	rs4870982	1.28E+08	2.89E-01	N/A	16	rs2968161	83529138	3.80E-04	N/A	-	2.83E-07	2.45E-01	3.47E-06	3401	
2	rs6729727	1.01E+08	8.25E-01	NPAS2	8	rs9329273	8509914	3.41E-02	N/A	-	2.83E-07	9.19E-01	1.04E-04	3402	
3	rs735659	15198998	4.45E-01	DVWA	22	rs7364173	49023156	5.00E-01	HDAC10	-	2.83E-07	6.52E-01	1.44E-05	3403	
2	rs7558364	1.8E+08	8.23E-01	ZNF385B	3	rs358741	1.57E+08	8.76E-01	C3orf33	-	2.83E-07	7.67E-01	6.10E-04	3404	
8	rs2003423	11340421	5.15E-01	C8orf12	15	s11247349	96851676	8.76E-01	FAM169B	-	2.83E-07	2.37E-01	2.11E-05	3405	
6	rs2243384	1.18E+08	3.68E-02	ROS1	9	s10820964	94081457	9.62E-01	IARS	-	2.83E-07	4.90E-01	3.12E-06	3406	
2	rs1364058	8067078	9.94E-01	N/A	3	rs6549282	70287533	7.17E-01	N/A	-	2.83E-07	8.70E-01	2.59E-05	3407	
11	rs11234627	85776380	8.56E-01	CCDC81	16	rs1461184	62565898	5.10E-01	N/A	-	2.83E-07	N/A	N/A	3408	
2	rs6705138	25441355	5.87E-01	DTNB	11	rs6483327	93743115	6.80E-01	GPR83	-	2.83E-07	1.22E-02	1.29E-07	3409	
8	rs4732690	29460028	5.69E-01	N/A	17	rs6502931	6061766	3.16E-01	N/A	-	2.83E-07	1.77E-01	1.43E-06	3410	
3	rs3755579	1.21E+08	5.30E-01	KTELC1	18	rs7240925	20484577	1.06E-01	N/A	-	2.83E-07	3.88E-01	8.59E-06	3411	
4	rs12498340	56906109	5.52E-01	KIAA1211	16	s13332555	54442500	6.18E-01	N/A	-	2.83E-07	3.88E-01	8.59E-06	3412	
6	rs9348687	25700468	5.86E-01	N/A	6	rs2239524	31233548	1.08E-02	TCF19	MHC	2.83E-07	9.58E-01	1.12E-04	3413	
3	rs1599796	1.21E+08	5.30E-01	CD80	18	rs7240925	20484577	1.06E-01	N/A	-	2.83E-07	9.59E-01	1.01E-04	3414	
6	rs9455997	1.68E+08	8.19E-01	N/A	11	rs1284108	93138481	8.91E-01	MED17	-	2.84E-07	2.63E-01	5.58E-04	3415	
10	rs4253060	50397793	2.08E-01	PGBD3	12	rs763948	66442193	9.93E-01	N/A	-	2.84E-07	N/A	N/A	3416	
2	rs6734108	1.05E+08	1.25E-01	N/A	9	rs4240425	1.3E+08	1.35E-01	SLC25A25	-	2.84E-07	2.41E-01	2.75E-03	3417	
4	rs2857960	3710410	7.21E-01	N/A	5	rs6881045	1.24E+08	9.79E-01	N/A	-	2.84E-07	3.25E-01	1.49E-06	3418	
13	rs9533456	42791004	9.75E-01	ENOX1	14	rs8015016	95192161	2.86E-02	TCL6	-	2.84E-07	4.54E-01	5.33E-06	3419	
11	rs10741762	19179719	7.95E-01	CSRP3	16	s12930687	9528370	8.01E-04	N/A	-	2.84E-07	8.29E-01	6.65E-05	3420	
2	rs934748	1.13E+08	2.21E-01	CHCHD5	8	s12678304	87729756	1.07E-01	CNGB3	-	2.84E-07	1.48E-01	1.08E-06	3421	
1	rs12078797	2.18E+08	8.87E-01	N/A	2	rs7604762	66478879	3.18E-01	N/A	-	2.84E-07	2.49E-01	1.00E-06	3422	
3	rs7617433	78966169	8.31E-02	ROBO1	21	rs2007397	41438062	1.27E-02	C21orf130	-	2.84E-07	9.32E-02	5.61E-07	3423	
4	rs11942220	93558414	7.39E-01	GRID2	20	rs1361514	17060688	5.93E-01	N/A	-	2.84E-07	3.32E-01	3.81E-06	3424	
11	rs1790474	1.19E+08	4.13E-01	N/A	15	s12898868	69899972	9.08E-01	MYO9A	-	2.84E-07	9.13E-01	7.66E-05	3425	
5	rs1544028	1.37E+08	7.42E-01	SPOCK1	18	s12960235	59404470	6.14E-01	SERPINB12	-	2.84E-07	1.14E-01	1.25E-06	3426	
12	rs6488713	14834183	9.01E-01	WBP11	13	rs9541996	34200700	9.17E-01	N/A	-	2.84E-07	1.94E-01	3.37E-06	3427	
6	rs2243384	1.18E+08	3.68E-02	ROS1	9	s10739927	94091902	9.03E-01	IARS	-	2.84E-07	9.55E-01	1.43E-04	3428	
6	rs2243384	1.18E+08	3.68E-02	ROS1	9	rs7023004	94138533	9.03E-01	CENPP	-	2.84E-07	9.55E-01	1.43E-04	3429	
5	rs1506174	40169821	1.82E-01	N/A	17	rs3809882	7940314	7.25E-01	N/A	-	2.84E-07	6.83E-01	1.17E-04	3430	
2	rs4671839	67774434	5.44E-01	N/A	3	rs1523752	77814664	9.39E-01	N/A	-	2.84E-07	1.28E-01	2.92E-03	3431	
7	rs10226053	35491630	1.65E-01	N/A	20	rs6128287	56210845	1.17E-02	N/A	-	2.85E-07	1.14E-01	4.85E-03	3432	
9	rs2996472	1.37E+08	9.46E-02	N/A	18	rs8088785	43806288	1.38E-01	ZBTB7C	-	2.85E-07	N/A	N/A	3433	
8	rs39767	90889143	2.76E-01	RIPK2	17	s10451228	73195845	8.35E-02	N/A	-	2.85E-07	8.68E-02	8.52E-07	3434	
6	rs6908208	1.25E+08	2.27E-01	NKAIN2	21	rs2826728	21484517	8.73E-01	NCAM2	-	2.85E-07	8.85E-01	2.65E-05	3435	
3	rs2056123	1.59E+08	9.69E-01	N/A	5	rs250210	50328781	4.18E-01	N/A	-	2.85E-07	1.06E-01	2.20E-07	3436	
12	rs2158133	45736697	6.22E-01	AMIGO2	18	rs1941526	37906937	9.68E-01	PIK3C3	-	2.85E-07	3.74E-01	2.43E-04	3437	
6	rs7756097	55142383	1.84E-01	HCRTR2	7	s11760565	95582203	6.07E-01	DYNC1I1	-	2.85E-07	9.70E-01	7.41E-05	3438	
12	rs11178226	69089941	9.20E-01	KCNMB4	15	rs2305445	97471323	5.20E-01	SYNM	-	2.85E-07	5.21E-01	5.07E-04	3439	
2	rs925798	1.62E+08	5.39E-01	N/A	18	rs1788904	55572070	2.92E-01	N/A	-	2.85E-07	6.92E-01	3.43E-04	3440	
6	rs1018401	22954031	8.97E-01	N/A	5	rs4957751	1.08E+08	6.50E-01	FBXL17	-	2.85E-07	4.27E-01	2.00E-04	3441	
9	rs944591	83372048	1.28E-01	TLE1	11	rs7945490	1.25E+08	9.13E-01	EI24	-	2.85E-07	8.29E-01	2.29E-05	3442	
2	rs932559	31406406	5.28E-01	XDH	3	rs6440373	1.47E+08	1.54E-01	N/A	-	2.85E-07	2.06E-01	6.22E-06	3443	
6	rs241424	32912912	2.10E-01	TAP1	15	rs8024631	36148921	5.98E-02	N/A	MHC	2.86E-07	6.71E-01	2.74E-05	3444	
2	rs10190732	5382212	9.94E-01	N/A	13	rs9565575	35287317	4.52E-01	DCLK1	-	2.86E-07	7.75E-02	1.01E-06	3445	
2	rs10190732	5382212	9.94E-01	N/A	13	rs956330	35276075	4.52E-01	DCLK1	-	2.86E-07	1.76E-01	4.32E-06	3446	
6	rs11155364	1.45E+08	7.90E-02	UTRN	13	rs9540711	65788311	9.70E-01	PCDH9	-	2.86E-07	1.98E-01	5.26E-06	3447	
4	rs13101260	1.84E+08	6.07E-01	DCTD	15	rs2462847	50499221	6.86E-01	MYO5A	-	2.86E-07	2.21E-01	1.80E-03	3448	
5	rs10036474	54405988	9.61E-01	N/A	9	rs1509186	1085922	8.28E-01	N/A	-	2.86E-07	2.21E-01	1.80E-03	3449	
5	rs1876681	54425155	9.61E-01	CDC20B	9	rs1509186	1085922	8.28E-01	N/A	-	2.86E-07	2.21E-01	1.80E-03	3450	
5	rs3111191	54413949	9.61E-01	N/A	9	rs1509186	1085922	8.28E-01	N/A	-	2.86E-07	2.97E-01	4.29E-07	3451	
1	rs4650166	69508597	8.79E-01	N/A	4	s11723316	1.85E+08	4.89E-01	N/A	-	2.86E-07	2.22E-01	1.11E-06	3452	
8	rs2458287	1.04E+08	8.84E-01	N/A	18	rs7505519	33426314	6.58E-01	N/A	-	2.86E-07	7.15E-01	6.72E-05	3453	
8	rs2458287	1.04E+08	8.84E-01	N/A	18	s12956949	33425651	6.58E-01	N/A	-	2.86E-07	7.35E-01	6.40E-05	3454	
9	rs7852834	28802865	8.33E-02	N/A	15	rs4513061	84772636	8.10E-01	AGBL1	-	2.86E-07	9.28E-01	1.30E-05	3455	
3	rs7373528	37725628	8.99E-02	ITGA9	3	rs7629646	1.05E+08	7.51E-02	N/A	-	2.86E-07	9.89E-01	5.65E-05	3456	
12	rs12812221	55605603	5.43E-01	SDR9C7	15	rs7182244	25484561	1.74E-01	N/A	-	2.86E-07	5.07E-01	7.56E-06	3457	
9	rs10992568	94793140	5.05E-01	FGD3	22	rs713781	44608887	5.75E-01	ATXN10	-	2.86E-07	8.66E-01	2.51E-05	3458	
9	rs4743322	1.01E+08	7.66E-01	COL15A1	15	rs869884	75078653	4.88E-01	PSTPIP1	-	2.86E-07	6.52E-02	8.76E-08	3459	
3	rs16943	51849719	7.98E-01	IQCF3	9	rs4540481	29980455	4.04E-01	N/A	-	2.86E-07	4.97E-01	5.80E-06	3460	
9	rs10781324	77829016	8.37E-01	N/A	15	s12909300	44336035	5.01E-01	N/A	-	2.86E-07	5.75E-01	1.49E-05	3461	
6	rs6917441	1.38E+08	1.31E-01	N/A	15	rs3522	72031397	8.99E-01	LOXL1	-	2.86E-07	3.17E-01	4.60E-06	3462	
11	rs2283155	2504751	1.86E-01	KCNQ1	11	rs4939284	58887475	3.10E-01	OR5AN1	-	2.86E-07	7.16E-01	1.65E-04	3463	
11	rs2283155	2504751	1.86E-01	KCNQ1	11	rs881834	58890943	3.10E-01	OR5AN1	-	2.86E-07	7.16E-01	1.65E-04	3464	
10	rs4460733	1.3E+08	5.02E-01	N/A	14	rs7152071	70222186	1.46E-01	TTC9	-	2.86E-07	9.25E-01	1.60E-04	3465	
6	rs9462935	43936551	2.00E-01	N/A	13	rs527337	32459520	8.32E-01	N/A	-	2.86E-07	6.09E-01	2.82E-05	3466	
13	rs9508766	29977837	4.82E-01	N/A	17	rs202578	10729031	6.05E-01	N/A	-	2.86E-07	6.52E-01	3.76E-04	3467	
1	rs10908730	1.58E+08	8.98E-01	OR10J5	3	s12494196	85796570	2.19E-01	CADM2	-	2.86E-07	2.11E-01	6.89E-06	3468	
2	rs4953408	46740069	4.21E-01	N/A	20	s10485815	51096895	8.85E-02	TSHZ2	-	2.87E-07	6.66E-01	1.12E-04	3469	
3	rs2611623	1.89E+08	1.80E-01	N/A	8	rs1050488	59659044	4.12E-01	NSMAF	-	2.87E-07	4.91E-02	2.27E-08	3470	

SNP A					SNP B						Interaction P			Ranking	Cluster in top 100 interactions
CHR	SNP	Location	gle locus P va	Gene	CHR	SNP	Location	Single locus P valu	Gene	MHC region	Stage 1	Stage 2	Combined		
7	rs7793289	13073946	6.14E-02	N/A	19	rs10411733	62482800	4.72E-01	ZNF460	-	2.87E-07	7.28E-01	9.77E-06	3471	
7	rs2160193	13070161	6.14E-02	N/A	19	rs10411733	62482800	4.72E-01	ZNF460	-	2.87E-07	7.53E-01	1.09E-05	3472	
8	rs10504517	72980330	1.44E-01	N/A	10	rs2014307	1.24E+08	1.18E-01	HTRA1	-	2.87E-07	7.94E-01	5.23E-05	3473	
5	rs389333	1.25E+08	6.22E-01	N/A	14	rs4465523	19983363	4.03E-01	TMEM55B	-	2.87E-07	9.99E-01	7.65E-05	3474	
5	rs420280	1.25E+08	6.22E-01	N/A	14	rs4465523	19983363	4.03E-01	TMEM55B	-	2.87E-07	9.99E-01	7.65E-05	3475	
7	rs6971207	1.39E+08	3.93E-01	TBXAS1	15	rs10519219	76977187	4.02E-01	MORF4L1	-	2.87E-07	2.11E-01	9.24E-04	3476	
8	rs6991392	1893230	9.90E-01	ARHGEF10	14	rs2749527	93896821	7.03E-01	SERPIN/A1	-	2.87E-07	2.48E-01	2.64E-06	3477	
8	rs2278092	30721347	5.96E-01	N/A	22	rs2013516	17752656	5.10E-01	HIRA	-	2.87E-07	9.27E-01	1.51E-04	3478	
1	rs4412572	1.61E+08	5.04E-01	N/A	4	rs4401481	60397850	3.91E-01	N/A	-	2.87E-07	N/A	N/A	3479	
6	rs1367211	1.61E+08	9.60E-01	LPA	18	rs12967775	65421552	6.14E-01	DOK6	-	2.87E-07	5.40E-01	5.49E-05	3480	
7	rs6966644	19360045	3.98E-01	N/A	13	rs6562694	71573602	8.06E-02	N/A	-	2.87E-07	6.17E-01	2.01E-05	3481	
2	rs13382215	2.15E+08	7.25E-01	VWC2L	14	rs10483719	59369282	2.76E-01	RTN1	-	2.87E-07	4.11E-01	1.64E-05	3482	
1	rs668800	1.15E+08	4.26E-02	N/A	11	rs648067	1.19E+08	4.25E-01	N/A	-	2.87E-07	6.16E-01	7.44E-06	3483	
4	rs7680027	15974946	3.22E-01	N/A	12	rs7968452	23653255	2.09E-01	SOX5	-	2.87E-07	7.80E-01	2.04E-05	3484	
5	rs985765	13447171	2.43E-01	N/A	13	rs14067	1.13E+08	4.50E-01	ADPRHL1	-	2.87E-07	4.28E-01	5.74E-04	3485	
1	rs12742458	30349160	3.34E-01	N/A	13	rs906790	75633401	6.15E-01	N/A	-	2.87E-07	1.85E-01	8.58E-04	3486	
4	rs13142103	55594043	9.87E-01	N/A	7	rs320088	29049787	7.65E-01	CPVL	-	2.87E-07	9.30E-01	9.27E-05	3487	
4	rs4864949	55595404	9.87E-01	N/A	7	rs320088	29049787	7.65E-01	CPVL	-	2.87E-07	9.30E-01	9.27E-05	3488	
6	rs9473594	49642288	8.88E-01	C6orf141	1	rs12048512	1.91E+08	7.87E-01	RGS21	-	2.87E-07	6.04E-01	2.14E-04	3489	
2	rs7605424	80155080	6.72E-01	CTNN/A2	7	rs1029465	11478639	1.64E-01	THSD7A	-	2.87E-07	8.14E-01	1.03E-04	3490	
2	rs3791696	2.12E+08	5.89E-01	ERBB4	3	rs4677287	73598809	1.07E-01	PDZRN3	-	2.87E-07	6.50E-01	1.44E-05	3491	
4	rs12506975	30096720	7.56E-01	N/A	19	rs892023	19524850	7.75E-01	YJEFN3	-	2.88E-07	1.57E-01	1.53E-06	3492	
2	rs7582124	1.6E+08	9.56E-01	7-Mar	18	rs9948462	7066836	6.08E-01	LAMA1	-	2.88E-07	8.72E-01	1.02E-04	3493	
1	rs1342780	90971264	5.87E-01	BARHL2	9	rs2182549	12785679	3.78E-01	C9orf150	-	2.88E-07	9.61E-01	9.34E-05	3494	
1	rs1924250	2.07E+08	6.70E-01	N/A	15	rs1484197	44455257	4.98E-02	N/A	-	2.88E-07	6.62E-01	4.33E-05	3495	
1	rs758518	1.17E+08	9.71E-02	CD58	4	rs13116208	15039724	6.35E-01	C1QTNF7	-	2.88E-07	7.19E-01	4.23E-04	3496	
4	rs12233914	19906678	2.98E-01	SLIT2	14	rs11159221	76190497	8.25E-03	N/A	-	2.88E-07	3.93E-01	4.21E-07	3497	
1	rs2075973	9278484	5.68E-01	SPSB1	3	rs1349008	76804103	8.47E-01	N/A	-	2.88E-07	3.53E-01	1.14E-05	3498	
5	rs12657273	1.07E+08	7.80E-01	N/A	10	rs538037	29481749	5.75E-01	N/A	-	2.88E-07	3.89E-01	5.41E-04	3499	
16	rs330149	20959502	4.26E-01	DN/AH3	16	rs2582597	58683286	3.67E-01	N/A	-	2.88E-07	5.16E-01	1.39E-04	3500	
3	rs4530474	1.25E+08	8.94E-01	MYLK	12	rs13311	1.12E+08	1.12E-01	OAS2	-	2.88E-07	7.50E-01	1.02E-05	3501	
1	rs2240335	17547124	1.17E-02	PADI4	13	rs4885001	71857395	7.48E-01	N/A	-	2.88E-07	7.85E-01	5.56E-05	3502	
12	rs9971641	86289970	2.82E-01	N/A	22	rs5758913	41478203	8.66E-01	N/A	-	2.88E-07	4.96E-01	2.51E-04	3503	
2	rs4146082	1.61E+08	5.88E-01	N/A	8	rs7010864	58947443	3.33E-02	N/A	-	2.89E-07	8.84E-01	1.36E-05	3504	
3	rs1732187	1.11E+08	4.81E-01	N/A	10	rs4880885	1632428	1.34E-01	ADARB2	-	2.89E-07	1.05E-01	3.79E-03	3505	
4	rs4693052	83873487	9.94E-01	SCD5	19	rs3787058	6184431	7.90E-01	MLLT1	-	2.89E-07	7.01E-01	1.72E-05	3506	
10	rs4751939	1.18E+08	5.40E-01	ATRNL1	15	rs1814767	21249101	1.49E-01	N/A	-	2.89E-07	8.99E-01	3.07E-05	3507	
4	rs4148155	89273691	8.33E-01	ABCG2	9	rs10739694	1.3E+08	7.73E-01	SH2D3C	-	2.89E-07	5.20E-01	4.56E-05	3508	
7	rs2058152	13100464	3.88E-02	N/A	19	rs1859955	62514181	6.47E-01	ZNF460	-	2.89E-07	6.13E-01	5.87E-06	3509	
7	rs7784901	13097171	3.88E-02	N/A	19	rs1859955	62514181	6.47E-01	ZNF460	-	2.89E-07	6.13E-01	5.87E-06	3510	
3	rs4687253	1.93E+08	8.71E-01	N/A	20	rs6012416	46467236	9.43E-01	N/A	-	2.89E-07	2.37E-01	1.92E-03	3511	
2	rs13422673	1.55E+08	8.42E-01	N/A	8	rs7829925	83586789	7.73E-01	N/A	-	2.89E-07	3.85E-01	3.02E-06	3512	
8	rs7003953	21660734	6.22E-02	GFRA2	14	rs179247	80502299	3.28E-01	TSHR	-	2.89E-07	4.50E-01	2.99E-04	3513	
5	rs2896245	17182725	3.89E-01	LOC285696	9	rs4879801	34499746	6.35E-01	DN/AI1	-	2.89E-07	5.43E-01	5.33E-04	3514	
1	rs3125923	68631067	2.98E-01	N/A	20	rs1886956	54313803	5.96E-01	N/A	-	2.90E-07	3.86E-02	6.78E-03	3515	
2	rs6705773	2.21E+08	2.92E-01	N/A	5	rs10155508	1.36E+08	2.98E-01	N/A	-	2.90E-07	4.95E-01	2.04E-05	3516	
3	rs11130751	59993955	7.42E-01	FHIT	5	rs4078802	1063083	2.79E-01	NKD2	-	2.90E-07	9.18E-01	7.77E-05	3517	
14	rs2239578	22973220	5.10E-01	MYH7	16	rs12599325	47343323	3.80E-01	N/A	-	2.90E-07	9.24E-01	3.14E-05	3518	
1	rs4926345	68733064	5.87E-01	DEPDC1	14	rs10133218	87424875	4.38E-01	N/A	-	2.90E-07	3.31E-01	2.90E-04	3519	
1	rs7527841	68708712	5.87E-01	DEPDC1	14	rs10133218	87424875	4.38E-01	N/A	-	2.90E-07	3.31E-01	2.90E-04	3520	
5	rs244603	50958653	1.09E-01	N/A	18	rs1566819	24374735	1.49E-01	N/A	-	2.90E-07	6.59E-01	1.98E-05	3521	
5	rs244603	50958653	1.09E-01	N/A	18	rs879500	24378686	1.49E-01	N/A	-	2.90E-07	6.59E-01	1.98E-05	3522	
7	rs6946058	1.16E+08	1.44E-02	TES	22	rs4822134	41197276	7.40E-01	N/A	-	2.90E-07	7.28E-01	1.29E-05	3523	
5	rs244552	50978106	1.09E-01	N/A	18	rs1566819	24374735	1.49E-01	N/A	-	2.90E-07	7.37E-01	2.71E-05	3524	
5	rs244552	50978106	1.09E-01	N/A	18	rs879500	24378686	1.49E-01	N/A	-	2.90E-07	7.37E-01	2.71E-05	3525	
5	rs36271	50983810	1.09E-01	N/A	18	rs1566819	24374735	1.49E-01	N/A	-	2.90E-07	7.70E-01	2.95E-05	3526	
5	rs36271	50983810	1.09E-01	N/A	18	rs879500	24378686	1.49E-01	N/A	-	2.90E-07	7.70E-01	2.95E-05	3527	
1	rs6424449	72056794	4.47E-01	NEGR1	1	rs17131515	91888607	9.27E-01	HSP90B3P	-	2.90E-07	4.23E-01	4.82E-04	3528	
5	rs745749	1.8E+08	5.30E-01	MAPK9	12	rs1921484	74609258	7.98E-01	N/A	-	2.90E-07	N/A	N/A	3529	
3	rs1348994	1.1E+08	2.37E-01	TRAT1	11	rs7947377	7923790	2.26E-01	NLRP10	-	2.90E-07	1.40E-01	1.11E-03	3530	
4	rs6850999	80720962	6.97E-02	N/A	7	rs9886302	70389420	4.37E-01	WBSCR17	-	2.90E-07	2.93E-01	5.79E-06	3531	
4	rs2218313	1.43E+08	2.64E-02	N/A	5	rs10515610	1.48E+08	5.36E-01	FBXO38	-	2.90E-07	2.73E-01	9.65E-04	3532	
11	rs986656	19488217	3.12E-01	N/AV2	18	rs12954266	18612312	2.06E-01	N/A	-	2.90E-07	6.28E-01	2.69E-04	3533	
2	rs12612135	1.39E+08	9.60E-02	N/A	13	rs4770601	23608111	1.76E-02	N/A	-	2.91E-07	2.50E-01	5.58E-06	3534	
3	rs13317017	1.01E+08	6.80E-01	FILIP1L	9	rs4877434	90080934	9.39E-01	N/A	-	2.91E-07	2.91E-01	3.46E-03	3535	
3	rs793440	1.01E+08	6.80E-01	FILIP1L	9	rs4877434	90080934	9.39E-01	N/A	-	2.91E-07	2.91E-01	3.46E-03	3536	
6	rs4839850	97191959	4.09E-01	N/A	2	rs6752043	75858330	6.70E-03	N/A	-	2.91E-07	3.49E-01	1.03E-04	3537	
4	rs3113765	1.82E+08	5.10E-01	N/A	18	rs1430916	38525965	9.25E-01	N/A	-	2.91E-07	3.97E-01	1.09E-05	3538	
18	rs12969440	65333980	9.48E-01	DOK6	20	rs6123631	54828815	2.98E-01	N/A	-	2.91E-07	7.54E-01	4.41E-05	3539	
6	rs1555064	1.64E+08	2.00E-01	PACRG	14	rs2293702	21071787	8.99E-01	SALL2	-	2.91E-07	7.31E-01	2.81E-05	3540	
8	rs9643233	1.29E+08	2.00E-01	N/A	16	rs30781	8891770	8.45E-01	USP7	-	2.91E-07	5.38E-01	8.03E-04	3541	
5	rs31226	53363328	3.70E-01	ARL15	17	rs820215	71205405	4.61E-01	SAP30BP	-	2.91E-07	7.26E-01	2.09E-04	3542	
11	rs7121739	1.1E+08	9.85E-01	N/A	14	rs12435024	91951404	7.13E-01	SLC24A4	-	2.91E-07	7.82E-01	6.77E-05	3543	
3	rs4128691	72432553	5.85E-01	N/A	11	rs479244	95572771	2.71E-01	MAML2	-	2.92E-07	6.46E-01	1.65E-05	3544	
3	rs4686910	1.89E+08	9.80E-01	N/A	20	rs2206641	19445646	2.63E-01	SLC24A3	-	2.92E-07	1.32E-01	1.39E-03	3545	
6	rs9322700	1.04E+08	8.74E-01	N/A	21	rs8130641	42173423	2.84E-01	PRDM15	-	2.92E-07	6.09E-01	8.56E-04	3546	
6	rs9404314	1.04E+08	8.74E-01	N/A	21	rs8130641	42173423	2.84E-01	PRDM15	-	2.92E-07	6.09E-01	8.56E-04	3547	
12	rs4762369	96103195	1.62E-01	N/A	19	rs2334253	50808317	2.61E-01	N/A	-	2.92E-07	9.49E-01	2.00E-05	3548	
3	rs7632823	54290709	1.09E-01	CACN/A2D3	20	rs6137352	2146288	5.96E-02	N/A	-	2.92E-07	N/A	N/A	3549	
1	rs10800938	1.88E+08	2.70E-01	FAM5C	7	rs12536500	50775527	8.10E-02	GRB10	-	2.92E-07	1.04E-01	3.70E-03	3550	
7	rs12535253	1.38E+08	5.51E-02	SVOPL	11	rs7103919	14209927	9.14E-01	SPON1	-	2.92E-07	2.32E-01	2.54E-06	3551	
10	rs1326983	19976417	3.86E-01	N/A	14	rs238402	57151094	7.76E-01	SLC35F4	-	2.92E-07	5.35E-01	1.75E-05	3552	
6	rs887464	31253899	8.59E-02	TCF19	6	rs3130685	31314185	9.60E-02	N/A	MHC	2.92E-07	1.49E-01	5.99E-06	3553	
6	rs11155364	1.45E+08	7.90E-02	UTRN	13	rs2324908	65741222	9.23E-01	N/A	-	2.92E-07	7.67E-01	3.92E-05	3554	
6	rs241424	32912912	2.10E-01	TAP1	15	rs7176106	36121556	3.74E-02	N/A	MHC	2.93E-07	1.02E-01	7.23E-07	3555	
3	rs12631386	1.35E+08	4.49E-02	N/A	5	rs6886072	1.37E+08	7.80E-01	SPOCK1	-	2.93E-07	8.30E-02	8.14E-04	3556	
2	rs843713	54359574	7.46E-01	ACYP2	3	rs9310279	73793635	1.87E-01	N/A	-	2.93E-07	1.14E-01	7.36E-03	3557	
6	rs2623966	1.53E+08	8.47E-01	SYNE1	5	rs1563551	83057582	8.70E-01	HAPLN1	-	2.93E-07	2.44E-01	5.88E-06	3558	
3	rs7617433	78966169	8.31E-02	ROBO1	10	rs1797	1.2E+08	8.98E-01	N/A	-	2.93E-07	1.80E-01	2.17E-07	3559	
13	rs2483397	24457548	8.14E-01	LOC646405	13	rs9584439	96375828	9.22E-01	N/A	-	2.93E-07	6.16E-01	2.93E-04	3560	
12	rs1152937	67591725	4.46E-01	CPM	20	rs2145280	55788630	7.42E-02	N/A	-	2.93E-07	7.66E-01	1.41E-04	3561	
11	rs7930159	69133252	5.40E-01	N/A	22	rs4821714	36522398	3.20E-02	TRIOBP	-	2.93E-07	8.65E-01	2.21E-04	3562	
17	rs231487	39336826	4.50E-01	MPP2	18	rs4891904	66962989	9.00E-01	N/A	-	2.93E-07	3.91E-02	8.70E-08	3563	
17	rs7503251	1691753	5.94E-01	SMYD4	20	rs235711	6688089	9.70E-02	BMP2	-	2.93E-07	8.64E-02	1.43E-06	3564	
6	rs4997129	856832	2.83E-01	N/A	5	rs9313585	1.72E+08	1.34E-01	UBTD2	-	2.93E-07	9.04E-01	4.29E-05	3565	
5	rs41135	96191782	6.54E-01	N/A	9	rs7860423	1.4E+08	7.74E-01	CACN/A1B	-	2.93E-07	3.47E-01	1.57E-03	3566	
2	rs2216253	2.06E+08	2.75E-01	N/A	3	rs6802186	1.33E+08	5.45E-02	CPNE4	-	2.93E-07	5.82E-01	2.18E-04	3567	
17	rs2107565	20029084	5.41E-01	CYTSB	18	rs11876558	29643580	4.76E-01	N/A	-	2.93E-07	9.15E-01	1.31E-04	3568	
17	rs2703807	20049781	5.41E-01	CYTSB	18	rs11876558	29643580	4.76E-01	N/A	-	2.93E-07	9.15E-01	1.31E-04	3569	
1	rs7523434	1.65E+08	1.28E-01	GPA33	12	rs2669010	75533191	8.86E-02	N/A	-	2.93E-07	N/A	N/A	3570	
1	rs3128678	4925039	7.99E-01	N/A	13	rs9576264	36886791	9.77E-01	N/A	-	2.94E-07	1.38E-02	1.94E-02	3571	
4	rs1513697	1.23E+08	6.86E-01	N/A	5	rs508089	1.11E+08	4.42E-01	C5orf13	-	2.94E-07	3.82E-02	3.01E-07	3572	
21	rs2833275	31411628	9.50E-01	TIAM1	22	rs5748285	17923952	1.37E-01	LOC150185	-	2.94E-07	4.21E-01	7.49E-04	3573	
1	rs10493808	88396583	4.41E-01	N/A	13	rs9515042	1.09E+08	8.71E-01	N/A	-	2.94E-07	6.58E-01	8.98E-04	3574	
9	rs2009306	77275310	3.91E-01	N/A	13	rs7999126	86769337	9.01E-01	N/A	-	2.94E-07	9.91E-03	1.31E-02	3575	
1	rs10908730	1.58E+08	8.98E-01	OR10J5	3	rs11127905	85793026	2.44E-01	CADM2	-	2.94E-07	2.11E-01	6.95E-06	3576	
1	rs10908730	1.58E+08	8.98E-01	OR10J5	3	rs9860249	85770242	2.44E-01	CADM2	-	2.94E-07	2.11E-01	6.95E-06	3577	
1	rs746503	54842574	3.17E-01	ACOT11	7	rs1029563	84576431	2.69E-01	SEMA3D	-	2.94E-07	3.43E-02	1.75E-08	3578	
14	rs12587564	53643881	6.78E-01	N/A	18	rs7244678	11987197	3.75E-01	IMPA2	-	2.94E-07	5.90E-01	1.73E-05	3579	
14	rs713423	53651669	6.78E-01	N/A	18	rs7244678	11987197	3.75E-01	IMPA2	-	2.94E-07	6.31E-01	2.05E-05	3580	
2	rs17033166	67262298	7.40E-02	N/A	9	rs7038501	1.16E+08	1.57E-01	ZNF618	-	2.94E-07	5.50E-02	3.99E-07	3581	
2	rs1037205	1.16E+08	2.99E-01	DPP10	16	rs11863271	82980316	3.69E-01	ATP2C2	-	2.94E-07	1.42E-01	9.91E-04	3582	

SNP A					SNP B						Interaction P			Ranking	Cluster in top 100 interactions
CHR	SNP	Location	gle locus P va	Gene	CHR	SNP	Location	Single locus P valu	Gene	HC region	Stage 1	Stage 2	Combined		
2	rs7584395	1.16E+08	2.99E-01	DPP10	16	's11863271	82980316	3.69E-01	ATP2C2	-	2.94E-07	1.49E-01	9.41E-04	3583	
11	rs7104953	1.31E+08	6.58E-01	NTM	20	rs6114758	2453124	2.23E-01	TMC2	-	2.94E-07	8.12E-01	3.81E-04	3584	
5	rs1173773	32786740	3.89E-01	NPR3	5	's11242599	1.06E+08	2.21E-01	N/A	-	2.94E-07	7.58E-01	9.58E-05	3585	
6	rs2146178	1.14E+08	8.83E-01	FLJ34503	7	rs9638081	1.56E+08	5.16E-02	N/A	-	2.94E-07	8.47E-01	7.06E-05	3586	
6	rs2273006	34959112	4.80E-01	ANKS1A	9	rs372957	31962985	1.19E-01	N/A	MHC	2.94E-07	8.60E-01	2.23E-05	3587	
6	rs2273006	34959112	4.80E-01	ANKS1A	9	rs453019	31975376	1.19E-01	N/A	MHC	2.94E-07	4.40E-01	1.23E-03	3588	
5	rs6870010	1.54E+08	8.69E-01	GALNT10	7	rs4719913	27899757	3.28E-01	JAZF1	-	2.95E-07	6.12E-01	1.02E-04	3589	
10	rs1413615	99793187	9.45E-01	CRTAC1	13	rs2018259	52421953	7.74E-02	N/A	-	2.95E-07	2.62E-03	1.10E-08	3590	
4	rs1513765	1.39E+08	1.21E-01	N/A	15	rs745103	65222129	3.01E-01	SMAD3	-	2.95E-07	3.28E-01	8.26E-06	3591	
3	rs902982	4568188	8.29E-02	ITPR1	5	rs2324938	86096826	5.01E-01	CADM2	-	2.95E-07	4.04E-01	7.62E-06	3592	
9	rs1155556	1.19E+08	8.36E-01	ASTN2	17	rs801259	34580691	9.50E-04	PLXDC1	-	2.95E-07	5.49E-01	1.37E-04	3593	
10	rs10736121	99796738	9.45E-01	CRTAC1	13	rs2018259	52421953	7.74E-02	N/A	-	2.95E-07	N/A	N/A	3594	
10	rs11189926	1.01E+08	8.77E-01	HPSE2	12	's11047795	25103308	7.94E-02	LRMP	-	2.95E-07	1.88E-01	3.13E-06	3595	
2	rs13014016	2.34E+08	6.19E-01	DGKD	10	rs751498	13969136	5.59E-01	FRMD4A	-	2.95E-07	9.78E-02	4.18E-03	3596	
2	rs1523300	70622873	3.40E-01	TGFA	20	rs2868802	45608177	5.62E-01	N/A	-	2.95E-07	8.05E-01	1.20E-04	3597	
1	rs17012944	2.07E+08	8.92E-01	N/A	16	rs2865585	12636088	7.51E-01	N/A	-	2.95E-07	3.37E-01	3.06E-06	3598	
2	rs17676196	2.3E+08	2.82E-01	PID1	18	rs2541872	61783932	3.81E-01	N/A	-	2.95E-07	4.57E-01	1.08E-04	3599	
3	rs7616728	26680641	8.92E-01	LRRC3B	5	rs7380062	1.39E+08	2.47E-01	ECSCR	-	2.95E-07	9.89E-01	2.03E-04	3600	
8	rs471757	1.03E+08	7.69E-01	GRHL2	11	rs483027	1.26E+08	5.79E-01	KIRREL3	-	2.95E-07	3.38E-01	1.52E-06	3601	
6	rs2876453	17678979	2.75E-01	CAP2	7	's11772815	28357572	3.06E-01	CREB5	-	2.95E-07	9.89E-01	6.68E-05	3602	
11	rs1784359	1.23E+08	2.38E-01	GRAMD1B	18	rs9958503	53362220	6.07E-01	FECH	-	2.95E-07	1.16E-01	1.54E-06	3603	
2	rs10191556	2.42E+08	2.87E-01	THAP4	4	rs905955	76955454	1.71E-01	USO1	-	2.95E-07	1.74E-01	1.56E-06	3604	
12	rs7970396	59254503	6.20E-01	N/A	16	rs7205180	10103085	8.12E-01	GRIN2A	-	2.95E-07	9.91E-01	5.91E-04	3605	
10	rs1679414	4495344	2.71E-01	N/A	16	's12598271	81163012	3.75E-01	N/A	-	2.95E-07	1.94E-01	4.45E-07	3606	
8	rs13254837	14217144	1.15E-02	SGCZ	8	rs4917229	1.43E+08	2.30E-03	N/A	-	2.96E-07	4.36E-01	3.81E-04	3607	
11	rs10751026	80152305	7.48E-01	N/A	11	rs2406347	98028284	2.05E-01	N/A	-	2.96E-07	3.44E-01	8.40E-04	3608	
6	rs7770797	6566961	8.95E-01	LOC285780	10	rs7896883	98777344	6.22E-01	SLIT1	-	2.96E-07	3.22E-01	7.92E-04	3609	
6	rs9348868	11476104	7.56E-01	NEDD9	1	's12407360	30137216	8.43E-01	N/A	-	2.96E-07	3.50E-01	3.10E-06	3610	
1	rs1501509	1.89E+08	3.10E-01	FAM5C	15	rs3743204	53577602	8.77E-02	DYX1C1	-	2.96E-07	4.58E-01	4.26E-05	3611	
5	rs1422092	1.03E+08	5.12E-01	N/A	17	rs748694	4496938	8.01E-01	ALOX15	-	2.96E-07	4.78E-01	2.89E-05	3612	
1	rs10900541	2.02E+08	3.46E-01	C1orf157	3	rs1838143	1.59E+08	1.97E-01	VEPH1	-	2.96E-07	4.78E-01	2.74E-04	3613	
4	rs2256587	29793136	8.67E-01	N/A	8	rs882229	62040177	7.82E-01	N/A	-	2.96E-07	6.72E-01	1.13E-04	3614	
3	rs6665	1.56E+08	3.92E-02	MME	11	rs615405	59761890	7.57E-03	N/A	-	2.96E-07	6.25E-01	5.94E-05	3615	
5	rs10055705	65978304	3.62E-01	MAST4	10	's11197065	84803122	9.37E-01	N/A	-	2.96E-07	7.69E-01	1.50E-04	3616	
8	rs2453666	1.01E+08	1.05E-01	SPAG1	11	's12419678	1.07E+08	7.73E-01	N/A	-	2.96E-07	2.74E-01	4.23E-06	3617	
6	rs1015811	28556065	8.46E-01	N/A	5	rs7443875	1.45E+08	6.07E-01	N/A	MHC	2.97E-07	7.07E-01	5.94E-05	3618	
2	rs10519330	76539476	1.08E-02	N/A	3	rs1482038	14296181	5.73E-01	N/A	-	2.97E-07	2.22E-01	1.52E-03	3619	
3	rs2633788	9589001	6.16E-01	LHFPL4	18	rs8091944	5548558	4.63E-01	EPB41L3	-	2.97E-07	6.94E-01	5.18E-05	3620	
2	rs12694884	2.32E+08	6.93E-01	SPATA3	3	rs1485727	59869101	1.19E-04	FHIT	-	2.97E-07	8.58E-01	5.84E-05	3621	
10	rs1679425	4482584	3.11E-01	N/A	16	's12598271	81163012	3.75E-01	N/A	-	2.97E-07	2.42E-01	6.55E-07	3622	
6	rs11760199	9068727	3.40E-01	N/A	20	rs719563	7090997	2.75E-01	N/A	-	2.97E-07	4.48E-01	1.89E-05	3623	
1	rs2168924	2.15E+08	8.89E-01	USH2A	2	's17032053	1.06E+08	5.49E-01	N/A	-	2.97E-07	9.88E-02	2.99E-07	3624	
5	rs12054892	57981007	6.54E-01	RAB3C	14	rs7150776	92183574	8.61E-01	RIN3	-	2.97E-07	4.07E-01	1.50E-05	3625	
2	rs2042542	2.18E+08	1.32E-01	TNS1	12	rs1245819	78166488	8.71E-01	SYT1	-	2.97E-07	8.15E-01	1.50E-05	3626	
2	rs2042542	2.18E+08	1.32E-01	TNS1	12	rs1268463	78167625	8.71E-01	SYT1	-	2.97E-07	8.15E-01	1.50E-05	3627	
2	rs6714466	2.25E+08	5.02E-01	DOCK10	10	rs7893153	43712417	1.24E-02	N/A	-	2.97E-07	6.56E-01	9.42E-06	3628	
2	rs7558808	2.26E+08	5.02E-01	DOCK10	10	rs7893153	43712417	1.24E-02	N/A	-	2.97E-07	6.56E-01	9.42E-06	3629	
2	rs546613	1.74E+08	3.86E-01	N/A	22	rs2154592	22277352	2.33E-01	C22orf43	-	2.97E-07	7.76E-01	1.65E-04	3630	
7	rs6946884	15846661	9.92E-01	N/A	7	rs9886124	52353870	1.30E-01	N/A	-	2.97E-07	8.44E-01	1.47E-04	3631	
8	rs7825918	49733525	2.28E-01	N/A	10	's11002569	79893734	3.93E-01	N/A	-	2.97E-07	2.70E-01	2.48E-05	3632	
4	rs4859332	34705058	6.50E-01	N/A	11	rs3740958	36429376	2.52E-02	PRR5L	-	2.97E-07	5.06E-01	1.20E-05	3633	
6	rs2883136	75534691	6.95E-01	N/A	11	rs2919040	96085117	2.21E-01	N/A	-	2.97E-07	7.22E-01	2.93E-05	3634	
10	rs12263663	9589327	8.11E-01	N/A	14	's12434022	82815943	6.02E-01	N/A	-	2.97E-07	4.37E-01	4.80E-06	3635	
6	rs11757522	1.58E+08	6.68E-02	N/A	17	's10515044	49047083	5.76E-01	N/A	-	2.97E-07	6.84E-01	2.75E-04	3636	
1	rs643841	1.11E+08	4.70E-01	N/A	7	rs1859545	82007492	5.51E-01	N/A	-	2.97E-07	1.95E-01	3.65E-03	3637	
3	rs2712353	1.15E+08	5.03E-01	ATP6V1A	12	's10841537	20496840	7.70E-02	PDE3A	-	2.98E-07	5.18E-01	6.51E-04	3638	
4	rs1394272	1.48E+08	5.59E-01	TTC29	15	rs3825846	76287162	3.75E-01	ACSBG1	-	2.98E-07	3.01E-01	1.11E-05	3639	
11	rs2341431	5550669	6.63E-01	OR52B6	12	rs7960093	25876110	1.12E-01	N/A	-	2.98E-07	3.06E-01	2.13E-06	3640	
3	rs745675	70788200	4.55E-01	N/A	21	rs1501813	17554542	4.84E-01	N/A	-	2.98E-07	9.72E-01	1.29E-04	3641	
3	rs4128782	89320422	6.97E-02	EPHA3	7	rs4731731	1.3E+08	6.07E-01	FLJ43663	-	2.98E-07	7.60E-01	4.98E-05	3642	
2	rs2121661	61415162	2.63E-01	USP34	8	rs2726555	59947966	8.90E-01	TOX	-	2.98E-07	3.57E-01	4.25E-04	3643	
7	rs816411	56138983	5.13E-01	CHCHD2	12	rs4758963	51950415	5.01E-01	ESPL1	-	2.98E-07	1.83E-01	8.53E-04	3644	
4	rs2218313	1.43E+08	2.64E-02	N/A	5	's12717973	1.48E+08	7.31E-01	FBXO38	-	2.98E-07	5.38E-01	2.93E-04	3645	
15	rs12913722	35080579	7.02E-01	MEIS2	15	rs4258562	93910483	5.83E-01	N/A	-	2.98E-07	9.50E-01	1.02E-04	3646	
4	rs6824816	1.4E+08	2.98E-01	N/A	10	rs4146592	98711592	5.01E-02	LCOR	-	2.98E-07	9.30E-01	7.53E-05	3647	
9	rs1322275	1.12E+08	9.48E-01	PALM2	12	rs3912631	51249120	7.51E-01	KRT71	-	2.99E-07	1.59E-01	2.30E-04	3648	
4	rs10003340	38385826	9.28E-01	KLF3	12	rs9971766	1.3E+08	7.24E-01	GPR133	-	2.99E-07	6.42E-01	1.92E-05	3649	
11	rs1118137	1.3E+08	1.37E-01	N/A	12	rs7962280	3189646	8.77E-01	TSPAN9	-	2.99E-07	9.52E-01	8.35E-05	3650	
1	rs2946534	18647370	6.65E-01	N/A	7	rs1005516	1.4E+08	7.49E-01	N/A	-	2.99E-07	7.04E-01	6.75E-04	3651	
8	rs972738	72651328	8.18E-01	N/A	18	rs9954856	28009142	5.44E-01	MEP1B	-	2.99E-07	6.54E-01	1.07E-05	3652	
8	rs12549058	72654792	8.18E-01	N/A	18	rs9954856	28009142	5.44E-01	MEP1B	-	2.99E-07	6.88E-01	1.22E-05	3653	
13	rs4942644	46970415	2.76E-01	N/A	19	rs7252293	11225495	1.50E-01	DOCK6	-	2.99E-07	4.16E-01	1.36E-05	3654	
13	rs4300533	46958034	2.76E-01	N/A	19	rs7252293	11225495	1.50E-01	DOCK6	-	2.99E-07	4.43E-01	1.63E-05	3655	
7	rs4727171	87953473	4.67E-01	N/A	18	rs242685	56914766	3.20E-01	N/A	-	2.99E-07	7.94E-01	4.50E-04	3656	
3	rs6763225	20390516	9.08E-02	N/A	14	rs1152490	55865987	1.95E-01	N/A	-	2.99E-07	5.97E-01	3.87E-06	3657	
6	rs6455738	1.62E+08	6.81E-01	PARK2	7	rs2058163	34301616	2.61E-01	N/A	-	3.00E-07	7.59E-01	3.19E-05	3658	
4	rs12499198	61771192	8.23E-01	N/A	5	rs7701571	1.25E+08	6.64E-01	N/A	-	3.00E-07	6.80E-01	8.92E-06	3659	
4	rs12499198	61771192	8.23E-01	N/A	5	rs2678180	1.25E+08	6.63E-01	N/A	-	3.00E-07	8.30E-01	1.50E-05	3660	
4	rs12499198	61771192	8.23E-01	N/A	5	rs2678181	1.25E+08	6.63E-01	N/A	-	3.00E-07	8.35E-01	1.54E-05	3661	
2	rs10178695	1.33E+08	1.61E-01	GPR39	11	's11215686	1.15E+08	4.70E-01	N/A	-	3.00E-07	8.49E-01	2.92E-05	3662	
16	rs1072123	50596711	4.09E-01	N/A	22	's12483892	33689572	5.41E-01	N/A	-	3.00E-07	8.82E-01	1.07E-04	3663	
6	rs9321203	1.3E+08	5.47E-02	N/A	14	rs1397163	91173949	1.20E-01	CATSPERB	-	3.00E-07	7.58E-02	1.37E-06	3664	
6	rs1011088	4426420	1.19E-01	N/A	16	's11648589	23203475	4.34E-01	SCNN1B	-	3.00E-07	4.43E-01	2.17E-04	3665	
6	rs12216269	1.38E+08	9.09E-01	N/A	20	rs6078603	12388470	4.96E-01	N/A	-	3.00E-07	N/A	N/A	3666	
12	rs2286646	5931752	5.05E-01	VWF	12	rs974728	11826119	5.72E-01	ETV6	-	3.00E-07	7.17E-01	7.85E-06	3667	
17	rs9907408	69582367	5.16E-01	N/A	19	rs4805084	39674399	1.27E-01	WTIP	-	3.00E-07	5.87E-01	7.82E-06	3668	
5	rs1864213	36663526	2.60E-01	SLC1A3	10	's10762266	70657325	7.20E-01	SUPV3L1	-	3.00E-07	3.55E-01	4.34E-04	3669	
7	rs2106292	87959974	4.65E-01	N/A	18	rs242685	56914766	3.20E-01	N/A	-	3.00E-07	8.41E-01	3.91E-04	3670	
6	rs7769239	1.24E+08	4.56E-01	TRDN	11	rs7940779	1.2E+08	5.47E-01	N/A	-	3.00E-07	7.29E-01	1.52E-04	3671	
5	rs244603	50958653	1.09E-01	N/A	18	rs1857705	24385115	1.45E-01	N/A	-	3.00E-07	7.53E-01	2.79E-05	3672	
5	rs244552	50978106	1.09E-01	N/A	18	rs1857705	24385115	1.45E-01	N/A	-	3.00E-07	8.33E-01	3.78E-05	3673	
5	rs36271	50983810	1.09E-01	N/A	18	rs1857705	24385115	1.45E-01	N/A	-	3.00E-07	8.65E-01	4.09E-05	3674	
3	rs9853873	58459561	2.24E-01	KCTD6	13	rs9540880	66204784	2.64E-01	PCDH9	-	3.00E-07	1.80E-01	6.83E-04	3675	
5	rs6860945	16600195	7.78E-01	FAM134B	9	rs7034380	14898056	5.82E-01	FREM1	-	3.00E-07	6.71E-01	2.87E-04	3676	
6	rs3868542	31253818	6.85E-02	TCF19	6	rs7750269	31379136	8.58E-01	N/A	MHC	3.01E-07	1.14E-01	2.14E-06	3677	
2	rs12478812	2.3E+08	4.41E-01	PID1	3	rs769641	3800923	8.96E-02	LRRN1	-	3.01E-07	9.32E-01	1.19E-04	3678	
10	rs11257069	11452059	7.55E-01	N/A	11	rs2186598	1.05E+08	9.64E-01	GRIA4	-	3.01E-07	9.86E-01	1.06E-04	3679	
13	rs7992439	70673459	1.70E-01	N/A	14	rs1243685	20168506	9.27E-02	OR6S1	-	3.01E-07	2.71E-01	5.36E-06	3680	
11	rs1790474	1.19E+08	4.13E-01	N/A	15	rs7175373	69990571	6.95E-01	MYO9A	-	3.01E-07	3.84E-01	8.37E-04	3681	
3	rs164207	32520695	5.95E-01	CMTM6	11	rs540723	1.25E+08	9.43E-01	CHEK1	-	3.01E-07	2.99E-01	5.96E-06	3682	
7	rs13225805	87968107	4.64E-01	N/A	18	rs242685	56914766	3.20E-01	N/A	-	3.01E-07	8.37E-01	4.21E-04	3683	
2	rs12992690	2.25E+08	3.23E-01	FAM124B	10	rs1417949	1.08E+08	2.35E-02	N/A	-	3.01E-07	5.69E-01	2.54E-05	3684	
1	rs7517475	66070749	3.91E-01	PDE4B	15	rs2304939	89290806	3.80E-01	UNC45A	-	3.01E-07	3.09E-01	3.99E-04	3685	
6	rs1001808	1.61E+08	5.33E-01	MAP3K4	8	rs7822258	65059969	9.08E-01	N/A	-	3.01E-07	4.36E-01	2.16E-05	3686	
5	rs3797462	76657430	2.87E-01	PDE8B	13	rs9550471	28962014	8.92E-01	MTUS2	-	3.02E-07	1.82E-01	2.59E-06	3687	
5	rs716866	1.69E+08	1.09E-01	SLIT3	9	rs1796993	74505258	4.66E-02	TMC1	-	3.02E-07	5.03E-01	1.41E-04	3688	
5	rs716866	1.69E+08	1.09E-01	SLIT3	9	rs2501917	74505943	4.66E-02	TMC1	-	3.02E-07	5.03E-01	1.41E-04	3689	
5	rs716866	1.69E+08	1.09E-01	SLIT3	9	rs2793150	74509273	4.66E-02	TMC1	-	3.02E-07	5.03E-01	1.41E-04	3690	
8	rs2542419	1.29E+08	4.30E-01	MIR1208	17	rs7211638	69465455	6.86E-01	N/A	-	3.02E-07	5.05E-01	3.53E-04	3691	
1	rs2164685	1.18E+08	6.60E-01	N/A	3	rs4681495	1.51E+08	1.80E-01	N/A	-	3.02E-07	6.00E-01	1.42E-04	3692	
8	rs10103181	65006365	3.05E-01	N/A	10	's11202059	81944823	3.91E-01	ANXA11	-	3.02E-07	8.56E-01	1.59E-04	3693	
5	rs46261	40258940	6.77E-01	N/A	15	rs2354040	21838205	5.31E-01	N/A	-	3.02E-07	N/A	N/A	3694	

SNP A					SNP B						Interaction P			Ranking	Cluster in top 100 interactions
CHR	SNP	Location	gle locus P va	Gene	CHR	SNP	Location	Single locus P valu	Gene	MHC region	Stage 1	Stage 2	Combined		
1	rs10920336	2E+08	5.13E-01	ARL8A	5	rs3103600	52928377	4.78E-01	NDUFS4	-	3.02E-07	8.28E-01	4.32E-05	3695	
1	rs2474373	61653302	4.74E-01	NFIA	4	rs2531198	10229443	9.18E-01	CLNK	-	3.02E-07	8.45E-01	6.80E-05	3696	
1	rs7518553	32087734	4.95E-01	N/A	3	rs1551213	85386752	9.66E-01	CADM2	-	3.02E-07	5.24E-01	1.66E-05	3697	
2	rs12992690	2.25E+08	3.23E-01	FAM124B	10	rs2027474	1.08E+08	3.71E-02	N/A	-	3.02E-07	6.92E-01	4.31E-05	3698	
3	rs4680974	78251414	8.35E-01	N/A	10	's10788198	1.23E+08	8.55E-01	N/A	-	3.02E-07	4.30E-01	4.63E-06	3699	
3	rs10511131	78275617	8.35E-01	N/A	10	's10788198	1.23E+08	8.55E-01	N/A	-	3.02E-07	5.92E-01	9.79E-06	3700	
2	rs831017	1.7E+08	3.65E-01	LRP2	4	's13110509	32457740	6.02E-01	N/A	-	3.02E-07	9.56E-04	9.26E-03	3701	
12	rs772570	61594801	4.98E-01	PPM1H	14	rs847297	71742850	9.21E-01	RGS6	-	3.02E-07	3.01E-01	3.85E-04	3702	
16	rs8191288	11673115	2.97E-01	TXNDC11	17	rs9889324	57481404	4.96E-01	MED13	-	3.03E-07	2.75E-01	2.76E-04	3703	
11	rs4570579	22282645	7.87E-01	N/A	12	rs1398541	37954128	3.93E-01	N/A	-	3.03E-07	3.77E-01	5.14E-06	3704	
5	rs2731714	1.72E+08	5.77E-01	SH3PXD2B	22	rs9609689	31763761	3.04E-01	SYN3	-	3.03E-07	4.71E-01	4.34E-04	3705	
7	rs2058152	13100464	3.88E-02	N/A	19	rs4801457	62485375	9.79E-01	ZNF460	-	3.03E-07	6.27E-01	6.95E-06	3706	
7	rs7784901	13097171	3.88E-02	N/A	19	rs4801457	62485375	9.79E-01	ZNF460	-	3.03E-07	6.27E-01	6.95E-06	3707	
8	rs4739241	21350702	2.58E-01	N/A	13	rs8000868	1.14E+08	8.47E-01	GAS6	-	3.03E-07	5.27E-01	5.98E-05	3708	
3	rs12487334	1.21E+08	5.21E-01	CD80	14	rs1885754	92238463	4.61E-01	RIN3	-	3.03E-07	5.48E-01	5.46E-06	3709	
3	rs12496520	1.21E+08	5.21E-01	CD80	14	rs1885754	92238463	4.61E-01	RIN3	-	3.03E-07	5.48E-01	5.46E-06	3710	
3	rs6779517	48359392	8.93E-01	SPINK8	5	rs1366397	23476753	2.74E-01	N/A	-	3.03E-07	1.75E-01	3.75E-06	3711	
14	rs4905315	94986984	1.99E-01	C14orf49	17	's12943620	76059253	2.95E-02	NPTX1	-	3.03E-07	4.47E-01	5.15E-06	3712	
1	rs697147	32924929	6.86E-01	SYNC	2	's16859517	2.2E+08	5.05E-01	NHEJ1	-	3.03E-07	8.20E-01	1.86E-05	3713	
12	rs7132629	73561463	5.79E-01	N/A	14	rs2747099	57532115	6.09E-01	C14orf37	-	3.03E-07	8.40E-01	7.47E-05	3714	
9	rs16919255	33431826	9.05E-02	NOL6	12	rs7310294	1.06E+08	9.82E-01	N/A	-	3.04E-07	1.54E-01	8.75E-06	3715	
2	rs6756027	64573758	5.05E-01	N/A	3	rs9852113	27577036	9.90E-01	N/A	-	3.04E-07	6.25E-01	3.06E-04	3716	
18	rs12458020	65332189	8.28E-01	DOK6	20	rs6123631	54828815	2.98E-01	N/A	-	3.04E-07	6.96E-01	3.89E-05	3717	
2	rs2652450	1.55E+08	7.37E-01	KCNJ3	9	rs1873738	80967681	1.18E-01	N/A	-	3.04E-07	5.53E-01	8.70E-06	3718	
10	rs10748526	82263059	9.67E-01	TSPAN14	17	's12603115	43603993	3.27E-03	SKAP1	-	3.04E-07	3.60E-01	6.46E-06	3719	
11	rs3016382	1.32E+08	1.99E-01	OPCML	15	rs2892364	90771909	6.42E-01	ST8SIA2	-	3.04E-07	3.03E-02	4.24E-03	3720	
4	rs10030530	16755617	9.45E-01	N/A	19	rs643786	55547761	5.81E-03	N/APSB	-	3.04E-07	6.40E-01	4.06E-05	3721	
5	rs3798004	9423945	5.10E-02	SEMA5A	11	rs1354269	19330820	9.39E-01	N/AV2	-	3.04E-07	6.77E-02	2.19E-03	3722	
8	rs4520196	1.21E+08	7.83E-01	ENPP2	9	's12346753	1.12E+08	3.59E-01	SVEP1	-	3.04E-07	6.60E-01	1.52E-05	3723	
7	rs4730257	77861452	2.67E-02	MAGI2	12	rs1049380	26380811	5.44E-01	ITPR2	-	3.04E-07	9.72E-01	2.16E-04	3724	
10	rs1228187	90428187	9.65E-01	LIPF	21	rs2837686	40828369	2.51E-01	DSCAM	-	3.04E-07	2.17E-01	8.28E-07	3725	
5	rs2379266	17791935	7.57E-01	N/A	15	rs1392857	51101682	1.87E-02	N/A	-	3.04E-07	N/A	N/A	3726	
4	rs1364886	1.42E+08	4.87E-01	RNF150	5	's10066478	1.64E+08	3.63E-01	N/A	-	3.05E-07	2.41E-01	4.74E-06	3727	
18	rs613582	68401652	4.66E-02	N/A	20	rs6035877	21460532	7.08E-01	NKX2-2	-	3.05E-07	8.53E-01	3.43E-05	3728	
2	rs2578007	1.83E+08	4.69E-01	N/A	12	rs1603572	82627746	9.51E-01	N/A	-	3.05E-07	1.83E-01	4.44E-06	3729	
9	rs1107108	70508637	4.38E-01	PIP5K1B	17	rs4789966	74747156	2.61E-01	HRNBP3	-	3.05E-07	2.72E-01	2.40E-06	3730	
5	rs4538612	33628213	7.02E-01	ADAMTS12	13	rs4942023	40573611	2.01E-01	WBP4	-	3.05E-07	3.11E-01	1.03E-03	3731	
18	rs1546564	32290294	4.79E-01	FHOD3	19	rs2013899	57360509	2.10E-01	ZNF836	-	3.05E-07	8.63E-01	8.87E-05	3732	
20	rs2904359	51410644	8.79E-01	N/A	21	's11701664	43380309	6.81E-01	CBS	-	3.05E-07	2.24E-01	3.33E-06	3733	
14	rs10130307	99325044	4.04E-02	EML1	16	rs1992113	57532372	9.96E-01	N/A	-	3.05E-07	2.58E-01	6.31E-06	3734	
6	rs4947296	31166157	4.31E-02	N/A	6	rs1265156	31250276	6.76E-01	TCF19	MHC	3.05E-07	1.05E-01	6.54E-07	3735	
6	rs7750269	31379136	8.58E-01	N/A	6	rs2394990	31448538	6.80E-02	HLA-B	MHC	3.05E-07	5.96E-01	1.15E-05	3736	
2	rs4849975	3679211	4.75E-01	COLEC11	13	rs2316640	54542925	2.70E-01	N/A	-	3.06E-07	2.37E-01	6.23E-04	3737	
2	rs716335	1.69E+08	1.84E-01	LASS6	3	rs826378	24144730	6.64E-02	THRB	-	3.06E-07	1.03E-01	9.51E-04	3738	
13	rs1022876	1.07E+08	3.28E-01	FAM155A	16	rs322598	62443472	6.86E-01	N/A	-	3.06E-07	3.81E-01	4.14E-04	3739	
11	rs10501540	82866094	3.34E-01	DLG2	13	rs9532449	39369698	1.50E-01	N/A	-	3.06E-07	6.79E-01	8.58E-06	3740	
9	rs10970902	32275225	1.95E-01	N/A	13	rs9565053	73330323	7.69E-01	KLF12	-	3.06E-07	7.16E-01	1.79E-05	3741	
2	rs17016629	35506420	1.82E-01	N/A	7	rs6467778	1.38E+08	5.20E-01	TRIM24	-	3.06E-07	7.26E-01	2.42E-04	3742	
12	rs9783446	86330283	2.25E-01	N/A	22	rs7287569	41483090	8.60E-01	N/A	-	3.06E-07	6.20E-01	1.82E-04	3743	
11	rs10892301	1.18E+08	2.96E-01	CXCR5	13	rs9521623	1.1E+08	4.91E-01	COL4A1	-	3.06E-07	7.34E-01	2.54E-05	3744	
10	rs703979	80614101	6.91E-01	ZMIZ1	11	rs3133875	1.32E+08	4.72E-01	NTM	-	3.07E-07	8.80E-02	8.51E-07	3745	
12	rs10492225	94985437	4.09E-01	N/A	21	rs2837914	41348235	7.00E-02	N/A	-	3.07E-07	8.08E-01	7.35E-05	3746	
1	rs4920295	18310826	3.33E-01	IGSF21	11	rs1003072	92721701	8.33E-01	CCDC67	-	3.07E-07	5.15E-01	2.77E-04	3747	
4	rs6852233	32440679	5.49E-01	N/A	5	rs2591699	5586625	7.41E-01	N/A	-	3.07E-07	7.44E-01	1.95E-05	3748	
2	rs6543264	1.05E+08	5.58E-01	N/A	17	rs9891860	46238130	7.68E-01	N/A	-	3.07E-07	4.13E-01	7.90E-06	3749	
3	rs7431223	1.35E+08	8.27E-01	SLCO2A1	5	rs6886072	1.37E+08	7.80E-01	SPOCK1	-	3.07E-07	9.11E-01	2.26E-04	3750	
13	rs1924078	94107745	9.78E-01	N/A	15	rs9806636	36503842	1.34E-01	N/A	-	3.07E-07	4.80E-01	4.29E-04	3751	
14	rs1544488	71635235	9.17E-01	RGS6	19	rs2195310	58337103	7.73E-01	ZNF415	-	3.07E-07	5.40E-01	2.20E-05	3752	
7	rs6462318	31976439	5.44E-01	N/A	19	rs892114	11127584	9.90E-01	SPC24	-	3.07E-07	6.78E-01	3.91E-04	3753	
3	rs6789596	66651431	4.29E-01	LRIG1	16	rs1865819	5796463	8.70E-01	N/A	-	3.07E-07	7.88E-01	1.16E-04	3754	
7	rs17166689	13068758	6.44E-02	N/A	19	rs3746228	62496174	5.82E-01	ZNF460	-	3.07E-07	N/A	N/A	3755	
13	rs2481873	61805091	7.22E-01	N/A	14	rs2332432	70151986	3.69E-01	MED6	-	3.08E-07	4.79E-01	1.82E-06	3756	
4	rs6449236	15963509	2.93E-01	N/A	12	rs7968452	23653255	2.09E-01	SOX5	-	3.08E-07	7.80E-01	2.21E-05	3757	
14	rs17109820	24939651	7.49E-02	N/A	21	rs2277820	46399800	1.58E-01	FTCD	-	3.08E-07	1.52E-02	5.21E-08	3758	
2	rs2705593	2.18E+08	4.63E-03	N/A	21	rs2832826	30652228	1.49E-01	KRTAP23-1	-	3.08E-07	1.75E-01	7.19E-04	3759	
7	rs1052981	37913026	3.09E-01	TXNDC3	13	rs9533423	42665963	5.22E-01	ENOX1	-	3.08E-07	6.94E-02	1.97E-03	3760	
3	rs7615943	62015047	6.45E-01	PTPRG	13	's12872160	1.06E+08	1.08E-01	N/A	-	3.08E-07	7.15E-01	6.50E-06	3761	
6	rs11155364	1.45E+08	7.90E-02	UTRN	13	rs1200530	65765795	9.60E-01	PCDH9	-	3.08E-07	7.02E-01	3.43E-05	3762	
10	rs12570061	31720584	9.58E-01	ZEB1	13	rs9567077	42442964	1.36E-01	EPSTI1	-	3.08E-07	1.66E-01	7.45E-04	3763	
4	rs1858925	1.5E+08	7.12E-01	N/A	18	rs650329	42533890	5.68E-02	ST8SIA5	-	3.08E-07	6.91E-01	8.29E-06	3764	
2	rs4666383	20435575	5.33E-02	N/A	2	rs889838	2.23E+08	2.72E-01	N/A	-	3.08E-07	7.08E-01	4.87E-05	3765	
8	rs1030080	5181456	1.42E-01	N/A	12	rs3825181	1.28E+08	7.31E-01	SLC15A4	-	3.08E-07	5.36E-01	4.58E-04	3766	
3	rs4449296	77477230	9.21E-01	ROBO2	11	rs2239695	1.18E+08	6.63E-01	CD3D	-	3.08E-07	5.93E-01	3.17E-04	3767	
2	rs7581224	84562726	8.96E-02	N/A	2	's10804253	2.18E+08	2.64E-01	N/A	-	3.08E-07	9.84E-01	4.84E-05	3768	
8	rs1707420	5595071	3.79E-01	N/A	9	's10739601	1.24E+08	5.89E-01	TTLL11	-	3.09E-07	3.23E-01	6.75E-06	3769	
1	rs1106151	1.11E+08	2.83E-01	KCN/A10	8	's11991905	79323262	1.17E-01	N/A	-	3.09E-07	7.73E-01	1.26E-04	3770	
9	rs1998247	17621552	3.02E-01	SH3GL2	9	rs7032919	27690275	5.78E-01	N/A	-	3.09E-07	2.35E-01	2.87E-06	3771	
4	rs1530611	5206255	4.95E-01	STK32B	17	rs647397	37932924	1.94E-01	ATP6V0A1	-	3.09E-07	7.34E-01	1.39E-04	3772	
4	rs1542986	1.34E+08	4.93E-01	PCDH10	12	's10845390	11660236	5.81E-01	N/A	-	3.09E-07	9.34E-01	4.17E-04	3773	
6	rs9356531	1.67E+08	4.17E-01	RPS6KA2	4	's13109642	1.16E+08	1.66E-01	N/A	-	3.09E-07	7.73E-02	6.18E-07	3774	
1	rs4926440	2.45E+08	1.43E-02	SCCPDH	20	rs2424239	19560584	2.37E-01	SLC24A3	-	3.09E-07	8.21E-01	7.53E-05	3775	
12	rs3847810	93103156	7.10E-01	PLXNC1	19	rs2396105	8477530	2.59E-01	HNRNPM	-	3.09E-07	1.10E-01	7.09E-07	3776	
11	rs2298725	1.2E+08	2.28E-01	GRIK4	19	rs7250065	34057029	2.65E-01	N/A	-	3.09E-07	6.14E-01	2.02E-05	3777	
2	rs10202039	50824106	6.35E-01	NRXN1	12	's10880480	42068852	8.50E-01	ADAMTS20	-	3.09E-07	6.58E-01	2.52E-05	3778	
1	rs12730329	85254288	5.38E-01	MCOLN2	8	's10102622	1.25E+08	7.00E-01	FER1L6	-	3.09E-07	8.54E-01	6.60E-05	3779	
3	rs12490870	1.28E+08	3.77E-01	N/A	5	's13188321	1.12E+08	7.84E-01	N/A	-	3.09E-07	8.91E-02	8.14E-07	3780	
1	rs7518553	32087734	4.95E-01	N/A	3	's12638015	85399338	8.66E-01	CADM2	-	3.09E-07	5.24E-01	1.66E-05	3781	
6	rs13204280	1.46E+08	4.27E-01	GRM1	10	rs1318662	26860966	6.27E-01	APBB1IP	-	3.09E-07	9.75E-01	1.24E-04	3782	
7	rs2700991	37347745	9.74E-01	ELMO1	13	rs7334971	40581627	6.96E-02	KBTBD6	-	3.10E-07	2.55E-01	1.23E-03	3783	
12	rs2245918	543776	5.66E-02	B4GALNT3	19	rs7259974	38472101	5.42E-01	CEBPA	-	3.10E-07	7.83E-02	5.05E-04	3784	
6	rs2748293	1.07E+08	3.93E-01	N/A	3	rs2364931	1.6E+08	2.45E-01	N/A	-	3.10E-07	1.89E-01	3.60E-03	3785	
6	rs2748293	1.07E+08	3.93E-01	N/A	3	rs4679851	1.6E+08	2.45E-01	N/A	-	3.10E-07	1.89E-01	3.60E-03	3786	
6	rs2748293	1.07E+08	3.93E-01	N/A	3	rs6778590	1.6E+08	2.45E-01	N/A	-	3.10E-07	2.17E-01	3.14E-03	3787	
13	rs2481873	61805091	7.22E-01	N/A	14	's10483832	70130590	3.54E-01	MED6	-	3.10E-07	5.17E-01	3.39E-06	3788	
13	rs2481873	61805091	7.22E-01	N/A	14	rs7156661	70131302	3.54E-01	MED6	-	3.10E-07	5.17E-01	3.39E-06	3789	
3	rs4522753	2041897	9.14E-01	N/A	15	rs2715803	95085514	4.90E-01	N/A	-	3.10E-07	N/A	N/A	3790	
4	rs2218313	1.43E+08	2.64E-02	N/A	5	rs7727161	1.48E+08	9.43E-01	HTR4	-	3.10E-07	5.82E-01	2.51E-04	3791	
7	rs2270080	7284115	5.35E-01	N/A	14	rs1959335	61549704	8.67E-01	SYT16	-	3.10E-07	2.37E-01	3.76E-04	3792	
1	rs6429216	2.39E+08	8.73E-01	N/A	3	rs925440	1.91E+08	4.09E-01	LEPREL1	-	3.10E-07	2.09E-01	8.70E-06	3793	
18	rs6566862	52950493	7.29E-01	N/A	20	rs2426210	48970895	7.60E-01	ADNP	-	3.10E-07	3.90E-01	2.71E-04	3794	
6	rs423137	42768633	7.53E-01	UBR2	13	rs9512551	26603697	5.38E-01	USP12	-	3.10E-07	5.36E-01	8.93E-05	3795	
6	rs423137	42768633	7.53E-01	UBR2	13	rs2484111	26630970	5.38E-01	USP12	-	3.10E-07	6.93E-01	1.57E-04	3796	
5	rs6883143	11225736	6.16E-03	CTNND2	9	rs4977246	20134936	3.07E-01	N/A	-	3.10E-07	9.98E-01	7.16E-05	3797	
7	rs10951658	41755259	4.59E-01	LOC285954	17	rs745318	70275393	7.75E-01	SLC9A3R1	-	3.11E-07	3.33E-01	1.73E-03	3798	
4	rs12505556	25286080	4.58E-02	N/A	10	's10826248	60338567	9.22E-01	N/A	-	3.11E-07	9.41E-01	9.59E-05	3799	
2	rs4439973	1.66E+08	9.80E-01	N/A	10	rs7895521	55526027	3.65E-01	PCDH15	-	3.11E-07	4.35E-02	N/A	3800	
5	rs725127	64135525	5.35E-01	SDCCAG10	15	rs2271902	76273244	8.65E-01	ACSBG1	-	3.11E-07	8.31E-01	6.02E-06	3801	
8	rs754539	18631372	5.96E-01	PSD3	18	rs1602895	26838554	8.16E-01	DSC3	-	3.11E-07	N/A	N/A	3802	
6	rs396243	31383153	2.17E-01	N/A	6	rs2523608	31430538	3.16E-01	HLA-B	MHC	3.11E-07	9.41E-01	4.58E-05	3803	
3	rs2715267	1.23E+08	3.50E-01	CD86	17	's16960503	19709516	1.85E-01	ULK2	-	3.12E-07	7.05E-01	2.89E-04	3804	
1	rs6660924	16602287	2.60E-01	C1orf144	2	's10929808	12486447	8.26E-01	N/A	-	3.12E-07	3.25E-01	8.41E-04	3805	
5	rs30983	1.47E+08	8.75E-01	N/A	14	rs2383414	32511542	9.35E-01	NPAS3	-	3.12E-07	7.64E-01	4.67E-05	3806	

SNP A					SNP B						Interaction P			Ranking	Cluster in top 100 interactions
CHR	SNP	Location	gle locus P va	Gene	CHR	SNP	Location	Single locus P valu	Gene	HC region	Stage 1	Stage 2	Combined		
4	rs6849151	1.54E+08	6.43E-01	N/A	14	rs4983413	1.05E+08	3.35E-01	MTA1	-	3.12E-07	5.80E-01	1.10E-03	3807	
8	rs3812429	71264273	4.68E-01	NCOA2	16	rs1013239	8170014	1.93E-01	N/A	-	3.12E-07	8.06E-01	1.44E-04	3808	
12	rs1420779	1.26E+08	1.84E-03	N/A	16	rs12931881	83436787	4.28E-03	CRISPLD2	-	3.12E-07	8.09E-01	3.72E-05	3809	
2	rs1024776	49023539	7.64E-01	FSHR	5	rs7708428	62129790	4.81E-01	N/A	-	3.12E-07	8.19E-01	1.09E-05	3810	
4	rs6828256	40113567	3.09E-01	RBM47	8	rs13271803	9264401	7.51E-01	N/A	-	3.12E-07	1.13E-01	5.04E-07	3811	
1	rs6699355	35157192	3.16E-01	DLGAP3	2	rs1030907	23468532	7.03E-01	KLHL29	-	3.12E-07	7.26E-01	3.09E-05	3812	
8	rs7829982	23262643	9.83E-01	LOXL2	12	rs4764755	99753004	8.38E-01	ANO4	-	3.12E-07	2.05E-01	1.52E-06	3813	
2	rs896637	2.09E+08	5.59E-01	N/A	7	rs10273717	20519346	1.20E-01	N/A	-	3.12E-07	3.47E-01	9.02E-04	3814	
5	rs1015610	26771763	6.81E-01	N/A	14	rs230703	64337222	3.74E-01	SPTB	-	3.12E-07	6.04E-01	1.10E-05	3815	
1	rs12567677	2.39E+08	6.75E-01	RGS7	11	rs1564582	1.08E+08	7.98E-01	EXPH5	-	3.12E-07	3.39E-01	2.62E-06	3816	
8	rs511114	93583685	7.04E-01	N/A	17	rs12453363	45576919	9.54E-02	PPP1R9B	-	3.12E-07	7.90E-02	7.55E-03	3817	
10	rs1751295	4486292	2.55E-01	N/A	16	rs12598271	81163012	3.75E-01	N/A	-	3.12E-07	2.42E-01	7.10E-07	3818	
5	rs3104733	25012004	9.16E-01	N/A	9	rs4977871	23633682	9.19E-01	N/A	-	3.12E-07	N/A	N/A	3819	
3	rs12636246	1.4E+08	1.89E-01	FAIM	8	rs12680577	69541263	4.09E-01	N/A	-	3.13E-07	3.26E-01	5.62E-04	3820	
15	rs8034570	95188934	1.99E-01	N/A	19	rs3746305	58734264	6.74E-01	ZNF331	-	3.13E-07	7.49E-01	1.01E-04	3821	
2	rs8179692	49046208	5.33E-01	FSHR	5	rs10462946	1.64E+08	4.71E-01	N/A	-	3.13E-07	3.42E-01	1.77E-04	3822	
2	rs6433709	1.79E+08	8.57E-02	PDE11A	3	rs11710921	57167334	7.06E-01	IL17RD	-	3.13E-07	3.86E-01	1.50E-04	3823	
6	rs11969025	1.69E+08	5.32E-02	SMOC2	20	rs13433265	15528747	4.60E-01	MACROD2	-	3.13E-07	4.22E-02	1.56E-03	3824	
4	rs1491411	1.76E+08	7.69E-01	N/A	8	rs4300027	6867985	7.89E-01	DEFA3	-	3.13E-07	2.80E-01	1.32E-03	3825	
4	rs1491411	1.76E+08	7.69E-01	N/A	8	rs4512398	6868004	7.89E-01	DEFA3	-	3.13E-07	2.80E-01	1.32E-03	3826	
4	rs2130938	1.76E+08	7.69E-01	N/A	8	rs4300027	6867985	7.89E-01	DEFA3	-	3.13E-07	2.80E-01	1.32E-03	3827	
4	rs2130938	1.76E+08	7.69E-01	N/A	8	rs4512398	6868004	7.89E-01	DEFA3	-	3.13E-07	2.80E-01	1.32E-03	3828	
4	rs923677	1.76E+08	7.69E-01	N/A	8	rs4300027	6867985	7.89E-01	DEFA3	-	3.13E-07	2.91E-01	1.26E-03	3829	
4	rs923677	1.76E+08	7.69E-01	N/A	8	rs4512398	6868004	7.89E-01	DEFA3	-	3.13E-07	2.91E-01	1.26E-03	3830	
13	rs7998868	94099764	9.29E-01	GPR180	15	rs9806636	36503842	1.34E-01	N/A	-	3.13E-07	3.71E-01	5.82E-04	3831	
6	rs2880404	1.69E+08	5.32E-02	SMOC2	20	rs13433265	15528747	4.60E-01	MACROD2	-	3.13E-07	N/A	N/A	3832	
9	rs3812537	70510552	2.37E-01	PIP5K1B	17	rs1869934	74746266	4.52E-01	HRNBP3	-	3.13E-07	3.72E-01	3.68E-06	3833	
1	rs12044451	75464916	4.91E-01	SLC44A5	5	rs10079545	1.62E+08	6.50E-01	N/A	-	3.13E-07	3.72E-01	3.68E-06	3834	
1	rs12046389	1.03E+08	9.05E-01	N/A	8	rs2445353	1.08E+08	7.80E-01	ANGPT1	-	3.13E-07	7.25E-01	2.88E-05	3835	
2	rs1015823	50541382	1.58E-01	NRXN1	18	rs1789476	55297903	4.65E-01	CCBE1	-	3.13E-07	6.45E-01	3.37E-05	3836	
6	rs9348862	31360726	7.72E-01	HLA-C	6	rs9266722	31457901	1.13E-01	N/A	MHC	3.13E-07	1.38E-01	2.31E-03	3837	
4	rs4693676	85301318	9.16E-01	N/A	20	rs6131256	11678686	7.81E-01	N/A	-	3.13E-07	3.49E-01	7.76E-04	3838	
6	rs9498131	1.49E+08	4.50E-01	N/A	9	rs2798062	92072427	9.72E-01	N/A	-	3.13E-07	3.49E-01	7.76E-04	3839	
2	rs1015823	50541382	1.58E-01	NRXN1	18	rs2013210	55297475	4.65E-01	CCBE1	-	3.13E-07	3.54E-01	1.20E-05	3840	
6	rs9357122	31368324	7.72E-01	N/A	6	rs9266722	31457901	1.13E-01	N/A	MHC	3.13E-07	3.98E-01	5.19E-05	3841	
6	rs3094205	31199841	5.91E-01	C6orf15	6	rs9368675	31380136	7.91E-01	N/A	MHC	3.14E-07	4.30E-01	8.38E-06	3842	
18	rs1546564	32290294	4.79E-01	FHOD3	19	rs7246914	57362717	1.36E-01	ZNF836	-	3.14E-07	5.04E-01	1.15E-05	3843	
5	rs11746792	1.72E+08	5.19E-01	DUSP1	20	rs1926065	54264744	8.87E-01	MC3R	-	3.14E-07	9.89E-01	1.41E-04	3844	
2	rs6709502	30864428	9.17E-01	CAPN13	11	rs1621764	1.16E+08	8.09E-01	N/A	-	3.14E-07	8.66E-02	1.12E-06	3845	
2	rs2306797	1.73E+08	2.91E-01	N/A	10	rs9418709	1.29E+08	5.24E-01	DOCK1	-	3.14E-07	2.12E-01	1.35E-06	3846	
9	rs1546049	1.16E+08	9.42E-01	COL27A1	11	rs10766597	19791607	4.41E-01	N/AV2	-	3.14E-07	4.27E-01	1.77E-05	3847	
9	rs2383022	17475598	2.76E-01	CNTLN	12	rs4760993	1.27E+08	5.27E-01	N/A	-	3.14E-07	3.64E-01	1.20E-03	3848	
5	rs1607561	23024828	8.54E-01	N/A	8	rs1915416	19200380	1.07E-01	N/A	-	3.14E-07	7.47E-02	2.31E-07	3849	
1	rs4596920	1.59E+08	1.89E-01	ITLN2	13	rs9538443	33189462	2.90E-01	N/A	-	3.14E-07	4.24E-01	8.88E-06	3850	
1	rs9783019	19722243	4.66E-01	N/A	5	rs325836	41073722	8.97E-01	HEATR7B2	-	3.14E-07	9.30E-01	2.09E-04	3851	
6	rs9393374	23294416	8.71E-01	N/A	19	rs36643	59332292	4.54E-01	CNOT3	-	3.14E-07	3.05E-01	1.14E-03	3852	
11	rs7936604	82514027	8.27E-01	N/A	15	rs2288258	70423855	6.81E-01	HEXA	-	3.14E-07	4.87E-01	6.13E-06	3853	
4	rs8192049	1.41E+08	5.80E-02	MGST2	5	rs4632783	92554065	3.86E-01	N/A	-	3.14E-07	5.52E-01	1.13E-05	3854	
6	rs6934929	65510506	6.41E-01	EYS	21	rs7276462	30351507	1.67E-01	N/A	-	3.15E-07	7.15E-03	2.49E-08	3855	
6	rs7771674	71510285	7.97E-02	SMAP1	4	rs10010241	1.66E+08	3.65E-01	KLHL2	-	3.15E-07	2.93E-01	1.47E-03	3856	
18	rs9652993	32292147	6.10E-01	FHOD3	19	rs2013899	57360509	2.10E-01	ZNF836	-	3.15E-07	8.26E-01	6.63E-05	3857	
4	rs4862611	1.87E+08	1.86E-03	N/A	22	rs882432	24677844	3.56E-02	MYO18B	-	3.15E-07	2.92E-01	6.50E-04	3858	
14	rs1205091	71349353	6.83E-01	N/A	20	rs226834	46292121	6.68E-01	N/A	-	3.15E-07	7.10E-01	2.92E-04	3859	
3	rs10865655	70940116	2.58E-01	N/A	20	rs2151507	56283834	2.15E-01	PPP4R1L	-	3.15E-07	8.98E-01	5.04E-05	3860	
1	rs1340592	67793691	4.96E-01	N/A	12	rs10875778	46910922	5.55E-01	N/A	-	3.15E-07	9.42E-01	4.78E-05	3861	
1	rs6662607	67794090	4.96E-01	N/A	12	rs10875778	46910922	5.55E-01	N/A	-	3.15E-07	9.42E-01	4.78E-05	3862	
1	rs6693292	67795756	4.96E-01	N/A	12	rs10875778	46910922	5.55E-01	N/A	-	3.15E-07	9.42E-01	4.78E-05	3863	
1	rs4655733	67798226	4.96E-01	N/A	12	rs10875778	46910922	5.55E-01	N/A	-	3.15E-07	#######	3.95E-05	3864	
3	rs10865655	70940116	2.58E-01	N/A	20	rs1045387	56374681	1.20E-02	RAB22A	-	3.15E-07	N/A	N/A	3865	
3	rs4330287	1.21E+08	4.44E-01	CD80	14	rs1885754	92238463	4.61E-01	RIN3	-	3.15E-07	5.83E-01	6.32E-06	3866	
10	rs1327014	1.3E+08	7.85E-01	N/A	20	rs389018	185497	9.45E-01	C20orf96	-	3.15E-07	6.19E-01	1.02E-05	3867	
1	rs1391819	63343139	8.94E-01	N/A	11	rs11041390	7432565	6.89E-01	SYT9	-	3.15E-07	1.09E-02	6.04E-08	3868	
3	rs7653155	1.05E+08	4.98E-01	N/A	9	rs7869607	90490207	2.68E-01	N/A	-	3.15E-07	4.47E-02	6.53E-07	3869	
15	rs4244559	35159903	6.45E-01	MEIS2	17	rs1975469	52797183	6.32E-01	MSI2	-	3.15E-07	6.96E-01	6.07E-04	3870	
6	rs2508015	31118179	8.45E-01	HCG22	6	rs9380215	31157634	4.29E-02	N/A	MHC	3.15E-07	1.98E-02	5.63E-08	3871	
5	rs712572	1.16E+08	5.19E-01	COMMD10	10	rs2475793	84629629	5.30E-01	NRG3	-	3.15E-07	6.81E-01	2.94E-06	3872	
8	rs10283038	14914993	5.14E-01	SGCZ	11	rs10831841	12502868	9.60E-03	PARVA	-	3.16E-07	4.85E-01	3.65E-04	3873	
6	rs7750881	1.65E+08	8.79E-01	N/A	2	rs12618269	34017690	2.17E-01	N/A	-	3.16E-07	4.03E-01	2.50E-06	3874	
3	rs336545	1.63E+08	5.72E-01	C3orf57	8	rs4977114	1.44E+08	6.95E-01	N/A	-	3.16E-07	1.32E-01	2.95E-06	3875	
3	rs336546	1.63E+08	5.72E-01	C3orf57	8	rs4977114	1.44E+08	6.95E-01	N/A	-	3.16E-07	1.32E-01	2.95E-06	3876	
7	rs6946058	1.16E+08	1.44E-02	TES	22	rs4822129	41153860	3.57E-01	NFAM1	-	3.16E-07	7.33E-01	6.63E-05	3877	
13	rs1323154	42872326	8.27E-01	ENOX1	14	rs4900550	1.02E+08	2.80E-01	RCOR1	-	3.16E-07	2.96E-01	6.69E-07	3878	
6	rs2763243	1.69E+08	6.71E-01	SMOC2	1	rs618451	2.36E+08	7.50E-02	N/A	-	3.16E-07	1.58E-01	4.69E-07	3879	
3	rs9874287	3506472	2.58E-02	N/A	11	rs4910330	11366514	9.37E-01	GALNTL4	-	3.16E-07	2.39E-01	2.08E-03	3880	
5	rs716866	1.69E+08	1.09E-01	SLIT3	9	rs1444835	74442041	1.62E-01	TMC1	-	3.16E-07	6.04E-01	7.40E-05	3881	
6	rs4713646	33714501	7.65E-01	ITPR3	3	rs6805120	1.67E+08	1.63E-01	N/A	MHC	3.17E-07	2.28E-02	1.48E-07	3882	
10	rs1679425	4482584	3.11E-01	N/A	16	rs4782717	81167218	2.78E-01	N/A	-	3.17E-07	2.49E-01	2.76E-03	3883	
4	rs1513697	1.23E+08	6.86E-01	N/A	5	rs26559	1.11E+08	3.67E-01	C5orf13	-	3.17E-07	2.54E-01	6.38E-07	3884	
2	rs6734108	1.05E+08	1.25E-01	N/A	9	rs10760528	1.3E+08	1.16E-01	N/AIF1	-	3.17E-07	4.60E-01	2.64E-04	3885	
3	rs6806077	16662811	5.54E-01	N/A	8	rs2514730	1.19E+08	2.12E-01	N/A	-	3.17E-07	N/A	N/A	3886	
5	rs293031	57999932	6.05E-01	RAB3C	14	rs3829948	92186579	5.37E-01	RIN3	-	3.17E-07	8.92E-02	3.96E-06	3887	
6	rs13216134	1.52E+08	3.72E-01	ESR1	3	rs2568911	1.96E+08	3.66E-01	N/A	-	3.17E-07	9.39E-01	4.72E-05	3888	
5	rs159009	58002899	6.05E-01	RAB3C	14	rs3829948	92186579	5.37E-01	RIN3	-	3.17E-07	9.39E-01	4.72E-05	3889	
7	rs2396623	1.13E+08	5.52E-01	N/A	15	rs12595242	85026338	2.72E-01	AGBL1	-	3.17E-07	7.77E-01	5.76E-05	3890	
1	rs4658609	2.42E+08	2.39E-01	N/A	18	rs1942336	52929100	5.55E-01	N/A	-	3.17E-07	6.71E-01	3.07E-05	3891	
2	rs4368280	1.93E+08	2.54E-01	TMEFF2	13	rs9317883	69622422	5.88E-01	ATXN8OS	-	3.17E-07	9.57E-01	1.34E-05	3892	
10	rs7906363	14302444	8.72E-01	FRMD4A	13	rs750989	1.12E+08	6.09E-01	C13orf28	-	3.17E-07	2.78E-01	6.32E-07	3893	
8	rs4840991	8467097	4.62E-01	N/A	14	rs10130641	74857206	8.76E-01	N/A	-	3.17E-07	N/A	N/A	3894	
6	rs9374070	1.1E+08	6.74E-01	N/A	12	rs1805474	13633417	8.43E-01	GRIN2B	-	3.17E-07	1.37E-01	1.34E-06	3895	
6	rs9374070	1.1E+08	6.74E-01	N/A	12	rs888150	13636311	8.43E-01	GRIN2B	-	3.17E-07	1.37E-01	1.34E-06	3896	
5	rs10939931	61512658	1.43E-01	N/A	7	rs4263662	7193952	8.74E-01	C1GALT1	-	3.17E-07	1.92E-01	1.45E-03	3897	
4	rs4407515	37361291	5.94E-01	RELL1	15	rs4965263	98105885	4.65E-01	LYSMD4	-	3.17E-07	6.46E-01	7.69E-04	3898	
2	rs6547818	21743084	2.76E-01	N/A	10	rs6585662	1.23E+08	2.36E-01	N/A	-	3.17E-07	4.79E-01	1.07E-05	3899	
13	rs17299557	92131341	7.25E-01	GPC5	18	rs10871618	63228905	1.89E-01	N/A	-	3.17E-07	6.42E-01	1.88E-04	3900	
13	rs4773539	89356301	5.20E-01	N/A	14	rs12431784	84014544	2.97E-01	N/A	-	3.18E-07	2.44E-02	9.07E-08	3901	
13	rs4773539	89356301	5.20E-01	N/A	14	rs1449108	84016968	2.97E-01	N/A	-	3.18E-07	2.44E-02	9.07E-08	3902	
5	rs1833813	32027313	8.77E-02	PDZD2	9	rs6559697	84905763	1.29E-01	N/A	-	3.18E-07	3.17E-02	2.46E-08	3903	
7	rs17159512	77394950	2.57E-01	PHTF2	20	rs6028080	59237873	1.55E-01	N/A	-	3.18E-07	7.09E-01	1.14E-05	3904	
6	rs6903174	1.65E+08	5.60E-01	N/A	21	rs2142077	40738065	6.47E-01	DSCAM	-	3.18E-07	4.76E-01	2.90E-04	3905	
10	rs497099	89980562	5.50E-02	N/A	19	rs7250425	18062757	8.45E-01	MAST3	-	3.18E-07	3.12E-01	2.55E-04	3906	
2	rs232571	38245817	4.34E-01	C2orf58	8	rs2634526	59847995	7.38E-01	N/A	-	3.18E-07	9.22E-01	2.40E-05	3907	
2	rs13407109	2.18E+08	9.06E-02	TNS1	12	rs1245840	78178449	9.17E-01	SYT1	-	3.18E-07	5.04E-01	1.02E-05	3908	
1	rs10493782	86223969	1.31E-01	COL24A1	5	rs1820167	52155113	6.97E-02	ITGA1	-	3.18E-07	2.26E-01	1.12E-03	3909	
3	rs9811451	32441111	4.38E-01	CMTM7	10	rs10999147	71550864	7.10E-01	H2AFY2	-	3.18E-07	4.71E-01	5.72E-06	3910	
4	rs6835433	10094085	2.79E-01	CLNK	9	rs1410846	23778680	4.60E-01	N/A	-	3.18E-07	5.56E-01	8.32E-04	3911	
3	rs6439099	1.29E+08	1.68E-01	KBTBD12	5	rs7705905	1.15E+08	2.56E-01	LVRN	-	3.18E-07	6.73E-01	1.27E-04	3912	
6	rs820089	35132673	2.90E-01	ANKS1A	9	rs372957	31962985	1.19E-01	N/A	MHC	3.18E-07	2.47E-01	1.12E-03	3913	
6	rs820089	35132673	2.90E-01	ANKS1A	9	rs453019	31975376	1.19E-01	N/A	MHC	3.18E-07	2.56E-01	1.07E-03	3914	
6	rs1028318	25698203	7.81E-01	N/A	2	rs3771846	75243000	7.15E-01	TACR1	MHC	3.18E-07	9.10E-01	3.60E-04	3915	
1	rs10489639	1.59E+08	7.88E-01	CD48	16	rs2199430	81394207	3.00E-01	CDH13	-	3.19E-07	6.61E-01	1.11E-04	3916	
3	rs2542360	9590418	6.09E-01	LHFPL4	18	rs8091944	5548558	4.63E-01	EPB41L3	-	3.19E-07	7.07E-01	1.16E-04	3917	
6	rs12525909	1.65E+08	7.54E-01	N/A	2	rs12618269	34017690	2.17E-01	N/A	-	3.19E-07	7.49E-01	1.15E-05	3918	

SNP A					SNP B						Interaction P			Ranking	Cluster in top 100 interactions
CHR	SNP	Location	gle locus P va	Gene	CHR	SNP	Location	Single locus P valu	Gene	MHC region	Stage 1	Stage 2	Combined		
4	rs12510598	1.36E+08	9.94E-01	N/A	7	rs3801317	36403431	5.53E-01	ANLN	-	3.19E-07	8.50E-01	4.55E-05	3919	
8	rs12056435	87218924	6.53E-02	ATP6V0D2	20	rs1005351	7020555	8.94E-01	N/A	-	3.19E-07	4.80E-01	8.00E-04	3920	
4	rs8192049	1.41E+08	5.80E-02	MGST2	5	s11738974	92563092	3.87E-01	N/A	-	3.19E-07	6.13E-01	1.45E-05	3921	
2	rs9711993	2.39E+08	7.23E-01	N/A	3	rs2220521	1.61E+08	8.82E-01	SCHIP1	-	3.19E-07	8.15E-01	1.16E-04	3922	
11	rs1943345	82553918	3.92E-01	PCF11	22	rs743185	27433217	6.71E-01	CHEK2	-	3.19E-07	2.81E-01	2.22E-06	3923	
1	rs1578624	1.72E+08	5.68E-01	N/A	10	s11016071	1.3E+08	5.72E-02	PTPRE	-	3.19E-07	2.85E-01	1.80E-06	3924	
1	rs1340592	67793691	4.96E-01	N/A	12	rs1471997	47009862	8.07E-01	N/A	-	3.19E-07	5.67E-01	2.59E-04	3925	
1	rs6662607	67794090	4.96E-01	N/A	12	rs1471997	47009862	8.07E-01	N/A	-	3.19E-07	5.67E-01	2.59E-04	3926	
1	rs6693292	67795756	4.96E-01	N/A	12	rs1471997	47009862	8.07E-01	N/A	-	3.19E-07	5.67E-01	2.59E-04	3927	
1	rs4655733	67798226	4.96E-01	N/A	12	rs1471997	47009862	8.07E-01	N/A	-	3.19E-07	6.22E-01	2.15E-04	3928	
3	rs4857176	97356654	2.54E-01	N/A	7	rs4380850	1.54E+08	1.06E-01	DPP6	-	3.19E-07	8.51E-01	6.51E-05	3929	
9	rs10816169	9719134	5.50E-01	PTPRD	15	rs2344853	84184767	1.10E-01	N/A	-	3.19E-07	6.51E-01	1.32E-05	3930	
3	rs2130369	1.01E+08	7.50E-01	C3orf26	19	s11880374	4587946	4.78E-01	TNFAIP8L1	-	3.19E-07	7.66E-01	2.93E-04	3931	
2	rs10185087	99790742	4.87E-01	AFF3	8	rs3885690	10864279	5.90E-01	XKR6	-	3.19E-07	8.56E-01	1.02E-04	3932	
5	rs712572	1.16E+08	5.19E-01	COMMD10	10	rs512064	84613706	3.18E-01	NRG3	-	3.19E-07	4.84E-03	9.78E-09	3933	
11	rs4755782	44124572	7.90E-01	EXT2	22	rs743803	47990194	1.82E-01	N/A	-	3.19E-07	1.77E-02	1.04E-07	3934	
15	rs2603229	98081022	5.54E-03	MEF2A	18	rs9965330	6754633	7.99E-01	N/A	-	3.19E-07	5.98E-01	4.26E-04	3935	
17	rs7209801	39678902	5.38E-01	SLC4A1	19	rs483808	7863481	2.21E-01	N/A	-	3.19E-07	1.95E-01	6.12E-07	3936	
6	rs2248386	31119226	7.23E-01	HCG22	6	rs9368675	31380136	7.91E-01	N/A	MHC	3.19E-07	5.54E-01	3.28E-06	3937	
6	rs4566922	8816712	6.24E-01	N/A	8	s17353731	35599010	7.29E-01	UNC5D	-	3.19E-07	7.90E-01	5.16E-05	3938	
2	rs1921266	67298196	2.15E-01	N/A	9	rs7038501	1.16E+08	1.57E-01	ZNF618	-	3.20E-07	4.21E-02	2.81E-07	3939	
7	rs2062403	68146581	5.93E-01	N/A	7	s10256186	69279720	1.55E-01	AUTS2	-	3.20E-07	3.23E-01	1.74E-04	3940	
7	rs2062403	68146581	5.93E-01	N/A	7	s10499812	69291926	1.55E-01	AUTS2	-	3.20E-07	3.23E-01	1.74E-04	3941	
15	rs488756	41402016	3.99E-02	ADAL	19	rs7256487	19716654	6.42E-02	ZNF14	-	3.20E-07	8.97E-01	5.96E-05	3942	
11	rs484092	1.07E+08	3.24E-01	ALKBH8	17	rs2074222	7070698	6.89E-02	DVL2	-	3.20E-07	N/A	N/A	3943	
3	rs713177	1.81E+08	9.34E-02	N/A	18	rs6506525	7678826	5.90E-01	PTPRM	-	3.20E-07	5.59E-02	1.94E-02	3944	
11	rs6590660	1.32E+08	9.90E-01	OPCML	14	rs2787448	83604750	5.80E-01	N/A	-	3.20E-07	8.52E-01	5.21E-05	3945	
6	rs9389952	1.42E+08	4.72E-01	N/A	6	rs9295361	1.67E+08	4.03E-01	RPS6KA2	-	3.20E-07	6.52E-01	1.20E-04	3946	
11	rs3751084	67540854	4.51E-01	UNC93B1	15	rs9806169	23415155	9.18E-01	N/A	-	3.20E-07	8.63E-01	2.17E-05	3947	
14	rs1868449	84090306	1.58E-01	N/A	19	rs778972	5818913	9.28E-01	FUT3	-	3.20E-07	N/A	N/A	3948	
7	rs2160193	13070161	6.14E-02	N/A	19	rs3746228	62496174	5.82E-01	ZNF460	-	3.20E-07	5.16E-01	6.19E-06	3949	
7	rs7793289	13073946	6.14E-02	N/A	19	rs3746228	62496174	5.82E-01	ZNF460	-	3.20E-07	6.16E-01	8.55E-06	3950	
2	rs2861680	79006430	1.64E-01	N/A	11	s11228236	55920138	3.50E-01	OR8U8	-	3.20E-07	6.56E-01	1.36E-04	3951	
1	rs476567	1.14E+08	8.28E-01	SYT6	22	rs4820028	29575745	4.21E-01	OSBP2	-	3.20E-07	8.42E-01	2.06E-05	3952	
1	rs12046158	2881573	1.29E-01	N/A	20	rs2876049	6602029	9.54E-01	N/A	-	3.20E-07	1.19E-01	5.71E-07	3953	
4	rs13152512	1.66E+08	9.00E-01	N/A	5	rs6894893	1.58E+08	9.24E-01	EBF1	-	3.20E-07	1.74E-01	1.06E-06	3954	
4	rs1512129	46597190	2.44E-01	COX7B2	17	s12601473	4272789	1.83E-01	SPNS3	-	3.20E-07	5.59E-01	1.27E-04	3955	
10	rs10711	62224480	6.47E-01	CDK1	17	rs1353618	28653255	3.18E-01	ACCN1	-	3.21E-07	3.48E-01	2.08E-04	3956	
8	rs1480391	50270973	3.23E-01	N/A	10	rs7077266	1E+08	5.43E-01	LOXL4	-	3.21E-07	5.14E-01	3.14E-04	3957	
17	rs2240226	39621744	3.99E-01	C17orf65	19	rs483808	7863481	2.21E-01	N/A	-	3.21E-07	7.49E-01	1.64E-05	3958	
6	rs2503322	1.27E+08	1.21E-01	RSPO3	21	rs2832373	29798982	2.68E-01	N/A	-	3.21E-07	3.79E-01	1.86E-05	3959	
1	rs11577259	2.42E+08	7.06E-01	N/A	11	rs475056	1.33E+08	7.01E-01	OPCML	-	3.21E-07	3.85E-01	2.09E-03	3960	
5	rs252452	1.42E+08	2.84E-01	ARHGAP26	12	s16910493	15052845	8.69E-01	N/A	-	3.21E-07	9.56E-01	5.34E-05	3961	
1	rs11802620	1.61E+08	6.02E-01	N/A	4	rs4401481	60397850	3.91E-01	N/A	-	3.21E-07	N/A	N/A	3962	
2	rs985891	2.3E+08	7.36E-01	PID1	3	rs769641	3800923	8.96E-02	LRRN1	-	3.21E-07	1.02E-01	1.22E-06	3963	
2	rs4971336	1017830	6.05E-01	SNTG2	5	rs1692356	71383640	8.06E-01	N/A	-	3.21E-07	7.31E-01	3.91E-04	3964	
3	rs13085838	64625627	3.84E-02	ADAMTS9	16	s12923918	24751904	6.69E-01	TNRC6A	-	3.21E-07	2.39E-02	1.32E-07	3965	
3	rs6783300	1.77E+08	1.38E-01	N/AALADL2	14	rs8005509	61616695	4.53E-01	SYT16	-	3.22E-07	N/A	N/A	3966	
6	rs6454267	83097859	3.68E-01	N/A	4	s11947222	12632405	1.04E-01	N/A	-	3.22E-07	9.02E-02	1.27E-03	3967	
5	rs6452143	23756112	3.87E-01	N/A	10	s11253779	16178495	5.46E-01	N/A	-	3.22E-07	6.41E-01	9.59E-05	3968	
9	rs1220793	1.31E+08	3.39E-01	N/A	11	s11603383	61794159	6.49E-01	SCGB2A2	-	3.22E-07	N/A	N/A	3969	
4	rs4689340	4415967	8.13E-01	N/A	5	rs2400176	1.45E+08	8.36E-01	N/A	-	3.22E-07	N/A	N/A	3970	
4	rs4689340	4415967	8.13E-01	N/A	5	rs7719533	1.45E+08	8.36E-01	N/A	-	3.22E-07	N/A	N/A	3971	
1	rs10493966	1.02E+08	9.02E-01	OLFM3	5	rs2560828	16619212	7.74E-01	FAM134B	-	3.22E-07	3.29E-01	3.21E-06	3972	
5	rs4360024	77719962	5.37E-01	SCAMP1	11	s11236029	73555872	3.42E-01	C2CD3	-	3.22E-07	6.50E-01	2.12E-05	3973	
1	rs1251575	76330372	5.00E-01	ST6GALN/AC3	2	s16843564	1.6E+08	9.48E-01	TANC1	-	3.22E-07	9.15E-01	1.04E-04	3974	
4	rs4698457	15826043	8.44E-01	FLJ39653	20	rs6010789	61031598	5.08E-01	N/A	-	3.22E-07	3.73E-01	1.05E-03	3975	
3	rs442800	1.39E+08	3.26E-01	N/A	8	rs904924	96599824	2.18E-01	N/A	-	3.22E-07	3.56E-01	1.54E-03	3976	
4	rs2866217	1.02E+08	1.38E-01	N/A	10	rs913295	14746460	4.56E-01	FAM107B	-	3.22E-07	4.17E-01	1.66E-04	3977	
3	rs13081659	68588088	1.80E-01	FAM19A1	4	s10018782	1.87E+08	7.83E-01	SORBS2	-	3.22E-07	2.27E-01	5.89E-07	3978	
4	rs17012036	1.28E+08	4.52E-01	N/A	8	rs1440332	73897644	2.54E-01	KCNB2	-	3.22E-07	4.28E-01	5.47E-06	3979	
9	rs12339359	1.24E+08	4.95E-01	TTLL11	21	rs2824961	18917889	7.40E-01	N/A	-	3.23E-07	4.81E-01	1.58E-04	3980	
7	rs6973935	13927105	6.35E-01	ETV1	7	rs6974985	1.49E+08	6.20E-01	KRBA1	-	3.23E-07	7.28E-01	4.97E-05	3981	
13	rs2282135	96287906	1.93E-01	HS6ST3	22	rs2027855	46216177	7.20E-01	N/A	-	3.23E-07	9.68E-01	3.20E-05	3982	
7	rs6969848	13926846	6.35E-01	ETV1	7	rs6974985	1.49E+08	6.20E-01	KRBA1	-	3.23E-07	N/A	N/A	3983	
11	rs1872902	1.03E+08	4.71E-01	PDGFD	12	s10770687	20701778	6.32E-01	PDE3A	-	3.23E-07	9.89E-01	6.85E-05	3984	
2	rs13410950	57506559	3.52E-01	N/A	2	rs730402	59948210	2.73E-02	N/A	-	3.23E-07	3.53E-03	4.04E-09	3985	
6	rs3020375	1.52E+08	6.30E-01	ESR1	3	rs861092	1.96E+08	7.35E-01	N/A	-	3.23E-07	4.31E-01	4.51E-04	3986	
1	rs10437024	82673852	2.74E-01	N/A	10	rs2495725	1.02E+08	8.19E-01	N/A	-	3.23E-07	5.06E-01	5.53E-04	3987	
8	rs6473031	78426035	1.45E-01	N/A	16	rs758456	5628146	9.77E-03	N/A	-	3.23E-07	7.41E-01	9.55E-05	3988	
1	rs12089641	65316390	9.34E-01	MIR101-1	16	s12928871	47640431	5.75E-02	N/A	-	3.23E-07	5.48E-01	3.24E-04	3989	
5	rs1181983	1.28E+08	6.83E-01	SLC27A6	9	rs3847227	1788170	7.23E-01	N/A	-	3.23E-07	7.58E-01	4.18E-05	3990	
13	rs12854616	80660902	6.63E-01	N/A	21	rs1892727	25154439	8.57E-01	N/A	-	3.23E-07	7.58E-01	2.72E-05	3991	
5	rs1551050	16586551	9.95E-01	FAM134B	9	rs7034380	14898056	5.82E-01	FREM1	-	3.23E-07	8.19E-01	1.71E-04	3992	
14	rs7144022	84831173	2.06E-01	N/A	21	rs2834371	34348725	7.30E-01	MRPS6	-	3.23E-07	2.83E-02	1.63E-07	3993	
10	rs12359500	1.1E+08	8.86E-01	N/A	15	rs1898885	38424914	6.19E-01	C15orf52	-	3.23E-07	6.67E-01	2.88E-04	3994	
5	rs6555834	1.68E+08	1.49E-01	SLIT3	10	s10764706	27874675	3.89E-01	RAB18	-	3.24E-07	6.13E-01	1.24E-05	3995	
8	rs2732972	5500796	7.16E-01	N/A	20	rs2267869	43403791	8.67E-03	SDC4	-	3.24E-07	5.27E-02	9.11E-06	3996	
3	rs4502548	24990545	3.81E-02	N/A	22	rs5770913	49360638	6.64E-01	CPT1B	-	3.24E-07	8.62E-01	1.41E-05	3997	
4	rs4407515	37361291	5.94E-01	RELL1	15	rs8027443	98103228	4.02E-01	LYSMD4	-	3.24E-07	5.52E-01	1.12E-03	3998	
18	rs9652993	32292147	6.10E-01	FHOD3	19	rs7246914	57362717	1.36E-01	ZNF836	-	3.24E-07	9.51E-01	1.05E-04	3999	
3	rs6773781	1.74E+08	4.07E-01	ECT2	18	rs1557320	22365762	4.83E-01	KCTD1	-	3.24E-07	8.49E-02	1.11E-06	4000	
17	rs4792606	15265393	4.16E-01	CDRT4	20	rs6125623	35679827	1.53E-01	N/A	-	3.24E-07	5.34E-01	3.13E-06	4001	
11	rs10831769	12201044	6.12E-01	MICAL2	18	rs2852346	35448497	5.22E-03	LOC647946	-	3.24E-07	9.34E-01	9.93E-06	4002	
5	rs6897932	35910332	6.50E-01	IL7R	21	rs2091895	40993998	1.65E-01	DSCAM	-	3.24E-07	3.92E-01	1.54E-03	4003	
5	rs962186	1.65E+08	1.92E-01	N/A	21	rs2236431	35335609	4.65E-01	RUNX1	-	3.24E-07	6.77E-01	8.56E-05	4004	
6	rs372578	7832222	6.84E-01	BMP6	1	s12146056	1.21E+08	5.66E-02	LOC647121	-	3.24E-07	2.07E-01	2.37E-06	4005	
2	rs4234085	2.09E+08	2.12E-01	N/A	7	rs6461503	20527521	1.19E-01	N/A	-	3.24E-07	6.07E-01	2.11E-04	4006	
6	rs9343977	81023720	2.70E-01	BCKDHB	4	s10020692	1.68E+08	8.89E-01	SPOCK3	-	3.24E-07	8.05E-01	6.10E-05	4007	
2	rs985891	2.3E+08	7.36E-01	PID1	3	rs6442831	3795517	9.99E-02	N/A	-	3.24E-07	8.90E-02	9.94E-07	4008	
11	rs983689	87861605	6.26E-01	GRM5	22	rs2413187	32194817	7.79E-01	LARGE	-	3.24E-07	1.39E-01	1.66E-07	4009	
1	rs12120863	89274110	9.04E-02	GBP1	13	rs2321995	58577784	3.86E-01	N/A	-	3.24E-07	1.55E-01	6.96E-07	4010	
11	rs10831769	12201044	6.12E-01	MICAL2	18	rs2726244	35471396	5.36E-03	LOC647946	-	3.24E-07	#######	1.08E-05	4011	
6	rs1362070	29850278	4.50E-02	HCG4	10	rs7083541	30997484	6.83E-01	N/A	MHC	3.25E-07	2.24E-01	2.08E-06	4012	
1	rs1188360	30984963	2.65E-01	N/A	16	s11648116	85609265	1.75E-01	N/A	-	3.25E-07	6.58E-01	5.99E-05	4013	
1	rs12035946	39177455	8.05E-01	RHBDL2	3	rs4550782	1.4E+08	2.27E-01	MRAS	-	3.25E-07	4.26E-02	5.32E-07	4014	
10	rs1925542	20986659	6.24E-01	N/A	14	s10483696	57622398	9.30E-01	C14orf37	-	3.25E-07	6.67E-01	1.38E-04	4015	
3	rs1826351	99269950	3.42E-02	OR5AC2	11	rs2897122	97129381	8.75E-01	N/A	-	3.25E-07	1.24E-01	1.34E-06	4016	
2	rs10211370	31134421	2.58E-01	GALNT14	13	rs4771203	27477477	2.12E-02	PRHOXNB	-	3.25E-07	2.78E-01	1.16E-03	4017	
9	rs1928423	73440207	7.97E-02	N/A	9	rs6477930	1.14E+08	2.40E-01	N/A	-	3.25E-07	4.80E-01	7.59E-04	4018	
2	rs4853333	77772523	7.72E-01	N/A	7	s12707217	1.34E+08	4.26E-01	AGBL3	-	3.25E-07	6.52E-01	2.93E-05	4019	
5	rs2594840	1.73E+08	4.46E-02	N/A	7	rs1229492	81402058	1.21E-01	CACN/A2D1	-	3.25E-07	3.36E-01	3.69E-06	4020	
11	rs2499958	4969470	5.23E-01	MMP26	15	rs3825915	99736820	6.82E-01	PCSK6	-	3.25E-07	4.53E-01	9.64E-05	4021	
19	rs1004046	35684479	3.06E-01	ZNF536	22	rs1024338	47919286	7.26E-02	N/A	-	3.25E-07	5.54E-01	1.60E-05	4022	
7	rs1581570	63862709	8.02E-01	N/A	8	s13278957	1.29E+08	6.15E-02	PVT1	-	3.25E-07	2.61E-01	7.05E-06	4023	
8	rs1912470	14474524	8.69E-01	SGCZ	16	rs6564661	77925428	6.01E-01	N/A	-	3.25E-07	1.12E-01	6.42E-03	4024	
2	rs10496401	1.06E+08	4.06E-01	N/A	19	rs3810342	43599425	1.44E-01	RYR1	-	3.25E-07	1.70E-01	2.62E-06	4025	
5	rs1477284	1.67E+08	1.94E-01	ODZ2	8	rs4549775	1.24E+08	4.80E-01	N/A	-	3.25E-07	2.45E-02	2.18E-08	4026	
6	rs9296264	38908879	4.36E-01	DN/AH8	8	rs2385107	1.26E+08	1.87E-01	N/A	-	3.25E-07	8.63E-02	1.58E-06	4027	
9	rs11141741	89101212	1.52E-01	N/A	10	s10998957	71198936	5.17E-01	N/A	-	3.25E-07	7.03E-01	3.40E-06	4028	
3	rs12497255	1.9E+08	4.41E-01	LPP	10	rs7097894	52387891	5.40E-02	N/A	-	3.25E-07	5.10E-02	7.60E-03	4029	
10	rs1880051	79665598	2.41E-01	N/A	19	rs1990932	51132140	9.77E-01	NOVA2	-	3.26E-07	5.90E-01	3.41E-04	4030	

SNP A					SNP B						Interaction P			Ranking	Cluster in top 100 interactions
CHR	SNP	Location	gle locus P va	Gene	CHR	SNP	Location	Single locus P valu	Gene	HC region	Stage 1	Stage 2	Combined		
5	rs4895293	1.2E+08	7.54E-01	N/A	20	rs580544	47638833	9.25E-01	N/A	-	3.26E-07	N/A	N/A	4031	
6	rs764650	14820472	5.25E-01	N/A	10	s12355470	92140942	7.41E-01	N/A	-	3.26E-07	8.29E-01	5.34E-04	4032	
1	rs2105422	1.89E+08	8.54E-01	N/A	17	rs11657083	6797819	8.22E-01	N/A	-	3.26E-07	8.93E-01	2.56E-05	4033	
19	rs7253483	13313550	5.47E-02	CACN/A1A	19	s10153462	51474446	6.13E-01	HIF3A	-	3.26E-07	1.90E-01	9.57E-04	4034	
1	rs697147	32924929	6.86E-01	SYNC	2	rs7588654	2.2E+08	5.46E-01	NHEJ1	-	3.26E-07	8.20E-01	1.93E-05	4035	
7	rs6467136	1.27E+08	5.90E-01	N/A	11	rs1478750	1.34E+08	4.25E-01	N/A	-	3.26E-07	N/A	N/A	4036	
2	rs7561066	2.15E+08	2.97E-01	SPAG16	13	rs4771997	96880094	7.13E-01	RAP2A	-	3.26E-07	3.25E-01	8.68E-04	4037	
12	rs7971473	69568041	6.83E-01	PTPRR	22	rs2275979	22534158	8.03E-01	SLC2A11	-	3.26E-07	2.86E-01	2.97E-04	4038	
11	rs2707098	19868309	3.39E-01	N/AV2	13	rs9520569	1.07E+08	3.76E-01	FAM155A	-	3.26E-07	2.40E-02	2.04E-02	4039	
8	rs2076958	1.13E+08	1.13E-01	N/A	20	rs16995121	8632506	5.45E-01	PLCB1	-	3.26E-07	8.47E-01	8.84E-05	4040	
4	rs12642398	89542237	4.46E-01	HERC6	10	rs1419099	1.15E+08	1.36E-01	NRAP	-	3.26E-07	9.27E-01	5.72E-05	4041	
5	rs7446783	38042733	1.54E-01	N/A	7	rs1181908	1.56E+08	8.15E-01	N/A	-	3.26E-07	1.46E-01	2.54E-06	4042	
16	rs2650494	28225941	6.64E-01	SBK1	22	rs7290806	46186516	3.83E-01	N/A	-	3.26E-07	2.26E-01	1.26E-06	4043	
3	rs7630566	1.68E+08	2.22E-01	N/A	8	rs7819156	84934035	4.28E-01	N/A	-	3.26E-07	2.77E-01	3.40E-04	4044	
2	rs13424790	1.88E+08	7.75E-01	TFPI	21	rs958924	26498774	5.76E-02	N/A	-	3.26E-07	4.46E-01	5.72E-04	4045	
2	rs7586970	1.88E+08	7.75E-01	TFPI	21	rs958924	26498774	5.76E-02	N/A	-	3.26E-07	4.46E-01	5.72E-04	4046	
10	rs2456761	62200548	7.14E-01	CDK1	17	rs1353618	28653255	3.18E-01	ACCN1	-	3.27E-07	5.10E-01	1.09E-04	4047	
7	rs736198	13069584	5.91E-02	N/A	19	rs3746228	62496174	5.82E-01	ZNF460	-	3.27E-07	5.19E-01	6.30E-06	4048	
2	rs2241752	50016262	7.60E-01	NRXN1	21	rs2822733	14819910	9.37E-01	SAMSN1	-	3.27E-07	6.94E-01	1.17E-04	4049	
1	rs10493208	56593239	6.62E-01	N/A	5	s12659150	25967191	4.97E-02	N/A	-	3.27E-07	1.96E-01	3.72E-04	4050	
2	rs1861764	2.05E+08	5.92E-01	PARD3B	19	rs4807863	6257971	8.37E-02	ACER1	-	3.27E-07	2.07E-01	4.81E-06	4051	
5	rs26505	96105143	6.10E-01	CAST	8	rs924864	4115253	1.59E-01	CSMD1	-	3.27E-07	6.30E-01	2.20E-05	4052	
8	rs17740228	1.19E+08	2.93E-01	N/A	19	rs1107920	38382536	5.86E-01	SLC7A10	-	3.27E-07	3.74E-01	2.07E-04	4053	
1	rs12040932	28183811	8.69E-02	EYA3	19	rs1870071	16366106	3.56E-01	EPS15L1	-	3.27E-07	7.74E-01	1.62E-05	4054	
2	rs3911199	1.88E+08	1.99E-01	N/A	21	rs958924	26498774	5.76E-02	N/A	-	3.27E-07	8.33E-02	5.02E-07	4055	
4	rs9993773	80653628	5.97E-02	N/A	7	rs2390961	24266172	2.98E-01	N/A	-	3.27E-07	1.48E-01	2.29E-06	4056	
1	rs1202812	58154568	7.98E-01	DAB1	20	rs6026369	56581846	2.97E-01	N/A	-	3.27E-07	7.53E-01	1.56E-04	4057	
4	rs6532503	95827046	9.78E-01	PDLIM5	16	s11644659	7691594	7.84E-01	A2BP1	-	3.27E-07	4.63E-01	6.52E-05	4058	
2	rs2528626	1.59E+08	1.37E-01	N/A	20	rs400420	54903747	1.89E-01	N/A	-	3.27E-07	9.93E-01	3.86E-05	4059	
4	rs1473094	30085252	9.67E-01	N/A	9	rs7854358	2749058	1.06E-02	N/A	-	3.27E-07	1.59E-01	5.94E-07	4060	
6	rs9380215	31157634	4.29E-02	N/A	6	rs1265156	31250276	6.76E-01	TCF19	MHC	3.27E-07	6.82E-01	8.42E-05	4061	
8	rs9773092	3203432	2.05E-01	CSMD1	12	rs6582285	73977054	5.01E-01	CAPS2	-	3.28E-07	8.91E-01	1.89E-05	4062	
4	rs6850999	80720962	6.97E-02	N/A	7	s10272997	70402452	5.44E-01	WBSCR17	-	3.28E-07	1.67E-01	2.49E-06	4063	
1	rs987293	18324487	3.40E-01	IGSF21	13	s10507989	90366295	4.82E-01	LOC144776	-	3.28E-07	1.80E-01	2.03E-06	4064	
8	rs6472126	64948465	2.45E-01	N/A	10	s11202059	81944823	3.91E-01	ANXA11	-	3.28E-07	8.56E-01	1.75E-04	4065	
3	rs475050	22998578	6.54E-01	N/A	13	rs7338544	46952662	7.38E-01	N/A	-	3.28E-07	6.23E-01	4.51E-06	4066	
4	rs11734262	61206591	1.38E-01	N/A	4	rs1000222	1.41E+08	2.25E-01	MGST2	-	3.28E-07	N/A	N/A	4067	
1	rs7520936	75317557	2.63E-01	N/A	16	rs9939422	62265086	1.45E-02	N/A	-	3.28E-07	4.43E-01	1.24E-04	4068	
9	rs4130590	1.29E+08	9.17E-01	GARNL3	10	s11255250	7706915	3.87E-01	ITIH5	-	3.28E-07	7.62E-01	8.54E-05	4069	
10	rs7918519	20218037	5.41E-01	PLXDC2	15	rs1401893	35314111	9.35E-01	N/A	-	3.28E-07	8.02E-01	2.08E-04	4070	
6	rs4629702	1.39E+08	5.89E-01	NHSL1	10	rs1153784	50559273	9.40E-01	CHAT	-	3.29E-07	1.08E-01	5.87E-07	4071	
2	rs4954804	1.39E+08	3.81E-01	N/A	16	rs1544357	19117111	1.90E-01	SYT17	-	3.29E-07	8.76E-01	3.55E-05	4072	
6	rs4240586	1.01E+08	6.76E-01	N/A	11	rs7928739	67590119	9.00E-02	CHKA	-	3.29E-07	7.01E-01	4.86E-05	4073	
1	rs11208380	64429559	8.74E-02	ROR1	15	rs2311843	25856949	5.73E-02	OCA2	-	3.29E-07	3.44E-01	3.84E-06	4074	
2	rs6436656	2.28E+08	4.34E-01	COL4A4	12	rs2097726	46105143	7.54E-01	N/A	-	3.29E-07	7.57E-01	6.79E-04	4075	
6	rs4709819	1.64E+08	4.73E-01	N/A	9	s10966450	24760659	3.09E-02	N/A	-	3.29E-07	8.53E-01	8.19E-05	4076	
3	rs2574720	11635412	1.97E-01	VGLL4	8	rs7838049	3077902	3.00E-01	CSMD1	-	3.29E-07	N/A	N/A	4077	
1	rs3820119	2.31E+08	8.54E-01	PCNXL2	3	rs4613466	83953554	9.75E-01	N/A	-	3.29E-07	N/A	N/A	4078	
1	rs4258226	1.89E+08	5.74E-01	N/A	9	rs4532674	1.13E+08	9.42E-02	MUSK	-	3.29E-07	2.58E-01	6.39E-06	4079	
4	rs7688965	1.82E+08	5.43E-01	N/A	21	rs2827845	23386781	1.78E-01	N/A	-	3.29E-07	8.93E-02	8.97E-04	4080	
6	rs3798221	1.61E+08	6.44E-01	LPA	7	rs7798471	6711482	4.88E-01	ZNF12	-	3.29E-07	6.32E-02	4.23E-07	4081	
10	rs1219719	1.26E+08	6.91E-01	CPXM2	14	rs736087	92102618	9.69E-01	RIN3	-	3.29E-07	2.00E-01	1.46E-06	4082	
2	rs1442763	1.17E+08	6.32E-01	N/A	15	rs9920389	93832564	9.64E-01	LOC145820	-	3.29E-07	9.29E-01	6.13E-05	4083	
6	rs130067	31226490	8.62E-01	TCF19	6	rs7761965	31381474	9.15E-01	N/A	MHC	3.29E-07	1.51E-01	9.56E-07	4084	
4	rs4834272	1.14E+08	4.80E-01	ALPK1	7	rs7778134	7891322	3.73E-02	N/A	-	3.30E-07	9.95E-01	7.86E-05	4085	
7	rs4719152	70669405	8.16E-02	WBSCR17	9	rs2300932	1.23E+08	6.11E-01	C5	-	3.30E-07	9.46E-02	2.76E-07	4086	
6	rs9360979	76900873	7.02E-01	N/A	2	rs6751246	2.09E+08	2.67E-01	CRYGC	-	3.30E-07	2.00E-01	2.29E-04	4087	
6	rs4240586	1.01E+08	6.76E-01	N/A	11	s10791957	67624124	6.97E-02	CHKA	-	3.30E-07	7.01E-01	5.17E-05	4088	
7	rs13227701	1.15E+08	6.99E-01	N/A	12	rs7295078	81066712	5.80E-01	N/A	-	3.30E-07	1.03E-02	1.74E-02	4089	
7	rs10266901	22235570	7.72E-01	RAPGEF5	19	rs2278004	54346233	1.61E-01	N/A	-	3.30E-07	2.66E-02	8.76E-07	4090	
4	rs1963656	24933840	2.08E-02	ZCCHC4	10	s10752113	7688196	6.87E-01	ITIH5	-	3.30E-07	5.07E-01	2.74E-04	4091	
5	rs1592890	1.27E+08	4.38E-01	MEGF10	16	rs9302846	7431239	3.90E-01	A2BP1	-	3.30E-07	2.67E-01	3.38E-06	4092	
1	rs2046616	92604157	3.47E-01	RPAP2	3	s10510803	59329572	6.53E-02	N/A	-	3.30E-07	3.69E-01	6.51E-04	4093	
10	rs1890628	1.3E+08	7.51E-01	N/A	11	s10831759	12177958	1.84E-01	MICAL2	-	3.30E-07	8.30E-01	9.73E-06	4094	
1	rs2297779	97772032	8.71E-02	DPYD	4	rs6811225	1.74E+08	2.91E-01	GALNTL6	-	3.30E-07	6.18E-01	3.69E-04	4095	
2	rs10496107	64222840	4.88E-01	PELI1	14	rs8008274	47746133	4.68E-01	N/A	-	3.30E-07	6.19E-02	3.80E-03	4096	
1	rs10797493	2.32E+08	1.14E-01	N/A	14	rs1349250	42409927	6.58E-01	N/A	-	3.30E-07	3.78E-01	9.65E-04	4097	
1	rs1603141	2.32E+08	1.14E-01	N/A	14	rs1349250	42409927	6.58E-01	N/A	-	3.30E-07	4.75E-01	6.88E-04	4098	
4	rs17023944	96598204	3.69E-01	UNC5C	7	s12667080	1.39E+08	8.74E-01	TBXAS1	-	3.31E-07	7.36E-01	8.75E-06	4099	
4	rs10012537	1.84E+08	9.76E-01	ODZ3	8	s13253691	18542967	7.46E-01	PSD3	-	3.31E-07	3.14E-01	1.36E-03	4100	
14	rs2352900	48441520	1.86E-01	N/A	15	rs2623255	50035348	1.42E-02	LEO1	-	3.31E-07	6.94E-01	2.47E-04	4101	
6	rs1883443	23569151	7.39E-01	N/A	3	rs282861	1.8E+08	7.29E-01	N/A	-	3.31E-07	7.75E-01	4.89E-05	4102	
5	rs13180336	1.49E+08	7.17E-01	CSNK1A1	18	rs7238178	37886955	8.10E-01	PIK3C3	-	3.31E-07	4.98E-02	5.87E-03	4103	
9	rs11103233	1.38E+08	9.44E-01	UBAC1	12	rs2407782	42564756	7.06E-01	TMEM117	-	3.31E-07	4.31E-01	2.35E-04	4104	
2	rs1918876	1.45E+08	8.78E-01	N/A	5	rs1371370	1.25E+08	2.66E-01	N/A	-	3.31E-07	7.63E-01	1.00E-04	4105	
6	rs764650	14820472	5.25E-01	N/A	10	s12762901	92157717	5.91E-01	N/A	-	3.31E-07	6.17E-01	7.99E-04	4106	
1	rs7526034	63359561	8.81E-01	N/A	11	rs11041391	7432637	5.65E-01	SYT9	-	3.31E-07	7.82E-01	2.22E-05	4107	
10	rs1572760	1.3E+08	9.01E-01	N/A	11	rs4370932	9559812	3.25E-01	WEE1	-	3.31E-07	5.08E-01	5.10E-06	4108	
5	rs4702718	10724040	8.93E-01	ANKRD33B	12	rs658719	3944655	7.20E-01	N/A	-	3.31E-07	4.10E-01	5.70E-06	4109	
3	rs3872610	1.14E+08	4.28E-01	C3orf17	11	s10902218	733813	9.12E-02	EPS8L2	-	3.32E-07	2.20E-01	2.25E-06	4110	
8	rs1912470	14474524	8.69E-01	SGCZ	16	s11642715	77904541	8.14E-01	N/A	-	3.32E-07	2.22E-01	3.66E-03	4111	
1	rs3006971	1.14E+08	1.19E-02	HIPK1	9	rs3780193	80122335	7.54E-01	PSAT1	-	3.32E-07	4.36E-01	4.46E-06	4112	
1	rs11122490	2.29E+08	4.44E-01	PGBD5	7	rs7800421	18029280	2.60E-01	PRPS1L1	-	3.32E-07	7.80E-02	2.13E-07	4113	
6	rs9392523	3750472	7.90E-01	N/A	5	rs1422780	1.39E+08	2.98E-02	PSD2	-	3.32E-07	8.58E-02	4.53E-07	4114	
4	rs12233660	1.5E+08	6.03E-01	N/A	18	rs650329	42533890	5.68E-02	ST8SIA5	-	3.32E-07	5.79E-01	7.87E-05	4115	
13	rs953937	89320971	6.27E-01	N/A	14	rs1449115	84073885	1.97E-01	N/A	-	3.32E-07	3.77E-02	2.24E-07	4116	
13	rs953937	89320971	6.27E-01	N/A	14	rs895889	84082471	1.97E-01	N/A	-	3.32E-07	4.07E-02	2.48E-07	4117	
1	rs12119911	5616797	8.09E-01	N/A	4	rs3919788	1.79E+08	5.02E-01	LOC285501	-	3.32E-07	5.51E-01	2.40E-03	4118	
1	rs3128678	4925039	7.99E-01	N/A	13	rs4941866	36880118	6.94E-01	N/A	-	3.32E-07	1.04E-02	2.05E-02	4119	
11	rs356247	1.27E+08	7.93E-01	N/A	16	rs4888091	79056411	2.75E-01	N/A	-	3.32E-07	5.26E-01	1.99E-05	4120	
9	rs1930021	27885371	9.56E-01	N/A	10	rs1572799	72181064	8.91E-02	ADAMTS14	-	3.32E-07	5.66E-01	7.28E-05	4121	
2	rs2916490	80045863	6.83E-01	CTNN/A2	7	s10247519	1.37E+08	7.87E-01	PTN	-	3.32E-07	7.20E-02	7.24E-03	4122	
8	rs2514809	95250422	7.09E-01	CDH17	17	rs2948549	22937916	2.93E-01	KSR1	-	3.32E-07	5.48E-01	1.58E-05	4123	
1	rs2479415	55271333	7.21E-01	PCSK9	7	rs541768	1.03E+08	3.45E-01	RELN	-	3.32E-07	8.43E-01	1.61E-05	4124	
1	rs6429195	2.38E+08	6.67E-01	FMN2	13	rs912975	45757278	1.56E-01	N/A	-	3.32E-07	3.71E-01	8.13E-06	4125	
1	rs2498962	62196074	7.63E-01	IN/ADL	3	rs7628487	1.3E+08	9.73E-01	N/A	-	3.33E-07	5.76E-01	3.77E-05	4126	
1	rs2482881	62196595	7.63E-01	IN/ADL	3	rs7628487	1.3E+08	9.73E-01	N/A	-	3.33E-07	5.81E-01	3.69E-05	4127	
1	rs12404105	15468767	1.24E-01	FHAD1	4	rs1880864	1.23E+08	8.43E-02	N/A	-	3.33E-07	9.42E-02	5.31E-07	4128	
10	rs7915236	60924819	9.66E-01	N/A	14	s11159868	88296625	9.31E-01	EML5	-	3.33E-07	2.98E-01	1.13E-03	4129	
2	rs1346787	55946116	8.78E-01	EFEMP1	18	rs715900	57397233	7.19E-01	N/A	-	3.33E-07	6.78E-01	3.69E-05	4130	
19	rs8112317	19998956	7.52E-01	ZNF682	21	rs2824797	18684231	5.80E-01	PRSS7	-	3.33E-07	5.32E-01	3.58E-04	4131	
1	rs766499	2.13E+08	4.42E-01	SMYD2	13	s11069721	1.08E+08	5.06E-02	N/A	-	3.33E-07	9.50E-01	2.41E-05	4132	
14	rs3759702	58631330	2.15E-03	N/A	15	s11637837	60835493	3.88E-01	TLN2	-	3.33E-07	8.84E-01	2.00E-05	4133	
8	rs7829925	83586789	7.73E-01	N/A	9	s10987314	1.28E+08	5.49E-01	FAM125B	-	3.33E-07	5.14E-01	6.74E-04	4134	
3	rs987944	21417257	7.70E-01	VENTXP7	5	rs6892080	84023778	6.73E-01	N/A	-	3.33E-07	8.47E-01	2.93E-05	4135	
7	rs6974363	47599712	6.43E-01	N/A	19	rs8102890	17236213	4.20E-01	ANKLE1	-	3.33E-07	6.21E-01	5.00E-04	4136	
5	rs1588265	59405551	5.93E-01	PDE4D	17	rs3785902	65619713	5.26E-02	KCNJ16	-	3.33E-07	7.20E-01	6.42E-05	4137	
2	rs11891588	2.16E+08	5.19E-01	N/A	16	s13336428	1472464	1.58E-01	CLCN7	-	3.33E-07	1.42E-01	1.97E-03	4138	
2	rs1363062	54135266	9.73E-01	N/A	5	rs995360	1.13E+08	4.00E-01	N/A	-	3.33E-07	5.53E-01	2.52E-04	4139	
2	rs1352043	1.41E+08	9.71E-01	LRP1B	14	s10483869	75098433	6.99E-01	BATF	-	3.33E-07	8.73E-01	2.02E-04	4140	
1	rs16839239	1.56E+08	8.84E-01	CD5L	8	rs7843846	1.41E+08	2.43E-01	TRAPPC9	-	3.33E-07	8.98E-01	6.50E-06	4141	
2	rs3769765	85126660	5.71E-01	KCMF1	21	rs914112	14437714	4.97E-01	LIPI	-	3.34E-07	7.68E-01	1.67E-04	4142	

SNP A					SNP B						Interaction P			Ranking	Cluster in top 100 interactions
CHR	SNP	Location	gle locus P va	Gene	CHR	SNP	Location	Single locus P valu	Gene	HC region	Stage 1	Stage 2	Combined		
2	rs3883936	85077757	5.71E-01	KCMF1	21	rs914112	14437714	4.97E-01	LIPI	-	3.34E-07	7.68E-01	1.67E-04	4143	
3	rs9853873	58459561	2.24E-01	KCTD6	13	rs7990984	66217726	2.89E-01	PCDH9	-	3.34E-07	2.21E-01	5.43E-04	4144	
10	rs1228187	90428187	9.65E-01	LIPF	21	rs2837682	40826362	6.19E-01	DSCAM	-	3.34E-07	3.34E-01	1.05E-04	4145	
6	rs412303	2799366	4.61E-01	SERPINB1	3	rs6794917	72632205	9.57E-01	N/A	-	3.34E-07	1.07E-01	1.57E-06	4146	
2	rs12053116	11462212	4.56E-01	N/A	7	rs10281617	1.53E+08	5.47E-01	N/A	-	3.34E-07	3.35E-01	1.83E-05	4147	
3	rs4685194	14553166	5.14E-01	GRIP2	9	rs1002420	78059344	5.32E-01	N/A	-	3.34E-07	7.79E-01	1.21E-04	4148	
4	rs1159315	46697833	4.76E-01	GABRA4	7	rs41815	1.06E+08	2.77E-01	N/A	-	3.34E-07	8.69E-01	4.86E-05	4149	
15	rs2412780	41443086	4.65E-02	ADAL	19	rs7256487	19716654	6.42E-02	ZNF14	-	3.34E-07	8.97E-01	6.10E-05	4150	
13	rs4773539	89356301	5.20E-01	N/A	14	rs1551013	84077293	1.21E-01	N/A	-	3.34E-07	4.64E-02	2.31E-07	4151	
10	rs12266064	90161951	2.47E-01	RNLS	13	rs1998835	25308742	2.34E-01	ATP8A2	-	3.34E-07	7.90E-01	2.28E-04	4152	
1	rs2821333	1.98E+08	1.14E-02	NR5A2	2	rs7587759	1.99E+08	6.59E-01	N/A	-	3.34E-07	4.65E-01	7.70E-04	4153	
8	rs1827135	1.29E+08	5.43E-01	MIR1208	17	rs7211638	69465455	6.86E-01	N/A	-	3.34E-07	4.97E-01	3.54E-04	4154	
6	rs9360979	76900873	7.02E-01	N/A	2	rs4375849	2.09E+08	2.62E-01	CRYGC	-	3.34E-07	2.00E-01	2.34E-04	4155	
6	rs9347476	1.61E+08	8.27E-03	MAP3K4	17	rs224547	3421768	3.75E-01	TRPV3	-	3.34E-07	1.92E-01	1.46E-03	4156	
5	rs7442753	1.72E+08	6.68E-02	N/A	10	rs1924715	91607363	4.15E-01	N/A	-	3.34E-07	8.98E-01	4.03E-05	4157	
1	rs10800013	1.63E+08	8.33E-01	N/A	8	rs4876153	2291741	2.11E-01	N/A	-	3.34E-07	N/A	N/A	4158	
3	rs4855477	68567763	2.53E-01	FAM19A1	4	rs10018782	1.87E+08	7.83E-01	SORBS2	-	3.34E-07	1.83E-01	3.42E-07	4159	
10	rs12266732	23636426	8.72E-01	C10orf67	20	rs1884748	5246668	4.34E-01	PROKR2	-	3.35E-07	7.71E-02	1.22E-06	4160	
10	rs1751295	4486292	2.55E-01	N/A	16	rs4782717	81167218	2.78E-01	N/A	-	3.35E-07	2.53E-01	6.94E-07	4161	
1	rs952477	1.46E+08	3.30E-02	GJA5	10	rs10824710	80482755	5.23E-01	LOC283050	-	3.35E-07	1.87E-01	7.55E-06	4162	
5	rs6452143	23756112	3.87E-01	N/A	10	rs1417096	16187485	5.17E-01	N/A	-	3.35E-07	6.64E-01	5.45E-05	4163	
10	rs3923324	1.25E+08	6.39E-01	N/A	12	rs1731760	76930722	8.22E-01	N/AV3	-	3.35E-07	7.21E-01	4.41E-04	4164	
6	rs9392247	8932862	6.08E-01	N/A	16	rs1017239	73915708	6.09E-01	CFDP1	-	3.35E-07	2.35E-02	5.03E-08	4165	
6	rs6568622	1.1E+08	8.38E-01	GPR6	5	rs1875478	1.69E+08	9.36E-01	DOCK2	-	3.35E-07	6.41E-01	4.69E-04	4166	
8	rs7814264	38959748	5.22E-01	PLEKHA2	15	rs401990	56450814	4.67E-01	N/A	-	3.35E-07	2.98E-01	1.96E-03	4167	
3	rs7611893	32295296	6.50E-02	CMTM8	10	rs7899885	69813780	1.16E-01	RUFY2	-	3.35E-07	3.90E-01	6.34E-04	4168	
2	rs11127258	30210165	8.47E-01	YPEL5	9	rs1555422	17611965	4.27E-01	SH3GL2	-	3.35E-07	4.42E-01	4.64E-06	4169	
2	rs6436559	2.26E+08	3.47E-01	KIAA1486	12	rs1012980	25075275	7.35E-01	N/A	-	3.35E-07	N/A	N/A	4170	
10	rs625039	1.03E+08	5.60E-02	FLJ41350	12	rs3858666	22795317	8.86E-01	N/A	-	3.35E-07	6.56E-01	4.27E-05	4171	
3	rs2629396	1.21E+08	9.07E-01	CD80	18	rs339866	20462599	6.44E-03	N/A	-	3.35E-07	8.89E-01	2.36E-05	4172	
5	rs7703740	1.65E+08	7.04E-01	N/A	21	rs2236479	45743560	2.20E-01	COL18A1	-	3.35E-07	N/A	N/A	4173	
6	rs4305722	49146347	2.05E-01	N/A	7	rs6968694	34183926	7.84E-01	N/A	-	3.36E-07	1.38E-01	7.14E-07	4174	
8	rs7018112	4030154	5.47E-01	CSMD1	13	rs206146	31854757	2.93E-01	BRCA2	-	3.36E-07	6.48E-01	N/A	4175	
2	rs6743301	85823031	8.01E-01	ATOH8	3	rs921160	63063832	9.79E-01	LOC285401	-	3.36E-07	6.27E-01	4.78E-05	4176	
6	rs9386578	1.07E+08	5.28E-01	QRSL1	3	rs16844883	1.1E+08	3.40E-01	N/A	-	3.36E-07	8.16E-01	4.59E-05	4177	
6	rs9400046	1.07E+08	5.28E-01	QRSL1	3	rs16844883	1.1E+08	3.40E-01	N/A	-	3.36E-07	8.16E-01	4.59E-05	4178	
1	rs1245489	94847043	3.09E-01	N/A	2	rs12466939	45340923	1.30E-01	N/A	-	3.36E-07	3.81E-01	1.35E-03	4179	
3	rs155524	37537145	7.07E-01	ITGA9	20	rs2425650	42936452	6.37E-01	YWHAB	-	3.36E-07	4.67E-01	2.26E-05	4180	
4	rs1382176	1.32E+08	8.37E-01	N/A	8	rs372976	82634163	4.55E-01	N/A	-	3.36E-07	7.97E-01	2.72E-05	4181	
4	rs1382176	1.32E+08	8.37E-01	N/A	8	rs396738	82634100	4.55E-01	N/A	-	3.36E-07	7.97E-01	2.72E-05	4182	
6	rs7756097	55142383	1.84E-01	HCRTR2	7	rs10267710	95578271	9.10E-01	DYNC1I1	-	3.36E-07	8.25E-01	2.07E-04	4183	
3	rs497316	1.74E+08	4.99E-01	NCEH1	11	rs4936118	1.3E+08	8.36E-01	N/A	-	3.36E-07	9.77E-01	4.67E-05	4184	
7	rs10255262	1.23E+08	4.02E-01	SLC13A1	17	rs17821469	1637177	4.76E-01	SMYD4	-	3.36E-07	2.45E-02	8.10E-03	4185	
11	rs689327	95888376	3.75E-01	N/A	12	rs12298037	51217814	3.41E-01	KRT71	-	3.36E-07	8.98E-01	5.86E-05	4186	
5	rs868548	1.07E+08	5.29E-01	N/A	16	rs1728394	84968026	1.23E-01	N/A	-	3.36E-07	9.72E-01	3.65E-04	4187	
11	rs11232180	79914436	3.63E-01	N/A	14	rs1434903	65773322	6.06E-02	N/A	-	3.36E-07	2.13E-01	7.88E-03	4188	
6	rs1059630	2730635	8.24E-01	WRNIP1	15	rs789884	34138757	4.05E-01	N/A	-	3.37E-07	9.38E-02	7.09E-03	4189	
6	rs3734557	40468443	9.70E-01	LRFN2	8	rs11779434	1.44E+08	6.20E-01	N/A	-	3.37E-07	9.70E-01	6.76E-05	4190	
6	rs2355199	1.57E+08	7.19E-01	N/A	1	rs596561	1.45E+08	8.84E-01	N/A	-	3.37E-07	1.75E-01	4.67E-07	4191	
6	rs10457834	1.49E+08	2.16E-01	N/A	9	rs2798062	92072427	9.72E-01	N/A	-	3.37E-07	3.85E-01	6.61E-05	4192	
6	rs4897043	1.49E+08	2.16E-01	N/A	9	rs2798062	92072427	9.72E-01	N/A	-	3.37E-07	3.85E-01	6.61E-05	4193	
5	rs1109105	1.68E+08	6.41E-01	SLIT3	18	rs556624	6873629	7.18E-01	ARHGAP28	-	3.37E-07	1.27E-01	2.94E-06	4194	
6	rs9395787	13552717	7.23E-01	GFOD1	6	rs11754023	1.19E+08	4.14E-01	N/A	-	3.37E-07	2.80E-01	4.91E-06	4195	
4	rs4834136	1.28E+08	3.28E-01	N/A	18	rs273700	21434903	6.88E-01	N/A	-	3.37E-07	6.84E-01	3.30E-05	4196	
3	rs7636734	37721855	4.56E-02	ITGA9	3	rs16846607	1.05E+08	1.09E-01	N/A	-	3.37E-07	7.48E-01	6.79E-05	4197	
3	rs6787571	1.8E+08	4.09E-02	N/A	9	rs2361093	596676	8.01E-01	KANK1	-	3.37E-07	2.77E-01	6.53E-04	4198	
4	rs4692317	25646700	5.87E-01	N/A	17	rs1896880	49924305	5.32E-01	N/A	-	3.37E-07	5.51E-01	5.02E-06	4199	
4	rs4693503	83869301	9.12E-01	SCD5	12	rs3819526	2306783	4.68E-01	CACN/A1C	-	3.37E-07	8.83E-01	6.81E-05	4200	
8	rs10808696	59867127	3.77E-01	TOX	9	rs7855088	99782074	6.54E-01	ANP32B	-	3.37E-07	5.60E-01	4.86E-04	4201	
6	rs11751484	1.28E+08	4.60E-01	N/A	14	rs2331481	21587111	1.27E-01	N/A	-	3.37E-07	4.92E-01	1.04E-05	4202	
2	rs3849377	1.01E+08	1.51E-01	NPAS2	7	rs7796391	1.26E+08	5.21E-01	GRM8	-	3.37E-07	6.15E-01	1.51E-04	4203	
8	rs7828550	82813796	9.93E-01	N/A	17	rs9894401	76357121	4.47E-01	RPTOR	-	3.37E-07	9.65E-01	1.76E-04	4204	
1	rs2236601	1.55E+08	2.44E-01	BCAN	7	rs700273	1.46E+08	7.36E-02	CNTN/AP2	-	3.37E-07	7.01E-01	1.05E-04	4205	
6	rs1407045	26584134	9.72E-01	BTN2A1	15	rs813299	29225595	8.06E-01	N/A	MHC	3.38E-07	7.44E-02	8.65E-07	4206	
4	rs6850637	28441643	9.68E-01	N/A	18	rs645951	8848624	5.45E-01	N/A	-	3.38E-07	8.93E-02	4.46E-03	4207	
8	rs351554	16051766	6.08E-01	MSR1	11	rs7950814	71905497	2.40E-01	N/A	-	3.38E-07	4.73E-01	6.33E-04	4208	
18	rs8090931	948056	8.57E-01	N/A	21	rs887772	14607323	6.65E-01	ABCC13	-	3.38E-07	5.19E-01	1.79E-04	4209	
5	rs7701794	1.7E+08	3.25E-01	KCNIP1	12	rs1466771	1.29E+08	2.60E-01	N/A	-	3.38E-07	5.59E-01	6.41E-04	4210	
6	rs2051115	1.4E+08	4.80E-01	N/A	13	rs2765153	23461279	7.99E-01	N/A	-	3.38E-07	7.62E-01	7.80E-05	4211	
14	rs10141952	66669922	1.78E-02	GPHN	16	rs12449282	10265020	5.28E-01	N/A	-	3.38E-07	9.60E-01	1.10E-04	4212	
2	rs1067359	44515501	1.95E-01	C2orf34	5	rs1445795	1.73E+08	2.27E-01	N/A	-	3.38E-07	8.36E-01	8.37E-06	4213	
3	rs393076	1.63E+08	7.34E-01	C3orf57	8	rs4977114	1.44E+08	6.95E-01	N/A	-	3.38E-07	9.32E-01	1.18E-04	4214	
14	rs10152034	25469206	8.47E-02	N/A	19	rs10418950	22402580	3.00E-01	ZNF98	-	3.38E-07	3.79E-01	6.34E-06	4215	
3	rs10937432	1.92E+08	5.92E-01	TMEM207	11	rs7950594	20243150	4.74E-01	N/A	-	3.38E-07	4.55E-01	1.20E-05	4216	
4	rs1355578	46594378	2.65E-01	COX7B2	17	rs12601473	4272789	1.83E-01	SPNS3	-	3.38E-07	5.36E-01	1.44E-04	4217	
1	rs4652781	1.81E+08	5.30E-01	LAMC2	11	rs10835057	26653876	2.24E-01	SLC5A12	-	3.38E-07	5.41E-01	7.11E-06	4218	
6	rs4461777	1633390	4.87E-01	GMDS	5	rs1238016	3181940	1.71E-01	N/A	-	3.38E-07	7.86E-01	9.09E-06	4219	
8	rs983977	24161212	7.35E-01	N/A	18	rs690336	8844219	8.40E-01	N/A	-	3.38E-07	1.68E-02	2.39E-02	4220	
14	rs176319	28095107	9.25E-02	N/A	14	rs11627968	36114849	9.79E-01	NKX2-8	-	3.38E-07	4.22E-01	2.74E-06	4221	
2	rs12711923	1.2E+08	1.54E-01	C1QL2	16	rs7188098	20233908	2.97E-01	GP2	-	3.38E-07	5.75E-01	7.08E-05	4222	
12	rs1558976	95606570	2.66E-01	N/A	15	rs12594730	37442648	6.90E-01	N/A	-	3.38E-07	6.07E-01	1.26E-05	4223	
3	rs6797725	77627512	6.51E-01	ROBO2	12	rs1666910	61563176	4.69E-01	PPM1H	-	3.38E-07	7.76E-01	5.40E-05	4224	
1	rs634294	85175585	2.17E-01	MCOLN2	3	rs6777810	1.02E+08	9.77E-02	TMEM45A	-	3.38E-07	8.61E-01	2.32E-05	4225	
2	rs10176482	29620651	8.22E-01	ALK	2	rs10514625	71292673	2.47E-02	PAIP2B	-	3.38E-07	N/A	N/A	4226	
4	rs10004154	1.12E+08	9.73E-02	ENPEP	15	rs8025706	91052293	9.84E-02	N/A	-	3.38E-07	2.84E-01	1.10E-03	4227	
4	rs11133136	1.77E+08	1.72E-01	SPATA4	13	rs7333999	46669259	3.68E-01	N/A	-	3.38E-07	5.02E-01	3.94E-04	4228	
4	rs12508055	1.83E+08	3.47E-01	N/A	18	rs1981370	72199803	1.86E-01	ZNF516	-	3.38E-07	6.80E-01	1.00E-04	4229	
3	rs1284745	1.04E+08	9.89E-01	N/A	13	rs7998964	47686991	6.92E-01	ITM2B	-	3.39E-07	1.10E-01	1.02E-06	4230	
3	rs1284745	1.04E+08	9.89E-01	N/A	13	rs9534995	47684077	6.92E-01	N/A	-	3.39E-07	1.10E-01	1.02E-06	4231	
3	rs1284745	1.04E+08	9.89E-01	N/A	13	rs9534981	47653682	6.92E-01	N/A	-	3.39E-07	1.26E-01	1.22E-06	4232	
5	rs10059139	64258718	1.65E-01	SDCCAG10	15	rs2271902	76273244	8.65E-01	ACSBG1	-	3.39E-07	8.63E-01	5.99E-06	4233	
1	rs2504053	1.78E+08	8.61E-01	FAM163A	11	rs10502173	1.13E+08	8.36E-01	TTC12	-	3.39E-07	6.29E-01	9.11E-05	4234	
12	rs17106030	67661043	5.90E-01	CPM	19	rs4802033	44671806	1.47E-01	TIMM50	-	3.39E-07	1.10E-01	1.25E-03	4235	
5	rs12522949	51014850	1.08E-01	N/A	18	rs1566819	24374735	1.49E-01	N/A	-	3.39E-07	6.41E-01	2.31E-05	4236	
5	rs12522949	51014850	1.08E-01	N/A	18	rs879500	24378686	1.49E-01	N/A	-	3.39E-07	6.41E-01	2.31E-05	4237	
7	rs7802654	93731669	6.96E-01	N/A	18	rs11083143	21424684	5.86E-01	N/A	-	3.39E-07	4.27E-01	1.53E-05	4238	
13	rs1171080	35385286	5.95E-01	DCLK1	13	rs9557470	1E+08	9.45E-01	TMTC4	-	3.39E-07	2.50E-01	8.33E-06	4239	
5	rs13180336	1.49E+08	7.17E-01	CSNK1A1	18	rs9956832	37818874	7.60E-01	PIK3C3	-	3.39E-07	5.02E-02	5.96E-03	4240	
9	rs7029669	1.09E+08	4.90E-01	N/A	10	rs12220941	96265029	4.42E-01	TBC1D12	-	3.39E-07	9.30E-01	7.01E-04	4241	
4	rs13137221	63216279	1.76E-01	N/A	20	rs1452320	12598325	1.09E-02	N/A	-	3.39E-07	8.05E-01	2.10E-06	4242	
6	rs6910759	7678861	9.35E-01	BMP6	2	rs2003298	11003601	5.14E-01	N/A	-	3.39E-07	5.23E-01	3.58E-04	4243	
3	rs7637878	1.35E+08	5.18E-02	BFSP2	4	rs2321859	1.42E+08	1.99E-01	RNF150	-	3.39E-07	7.75E-01	3.57E-05	4244	
4	rs4693676	85301318	9.16E-01	N/A	20	rs6078340	11716987	5.97E-01	N/A	-	3.39E-07	1.79E-01	1.02E-03	4245	
3	rs10212614	1.05E+08	8.96E-01	N/A	15	rs7169735	77090508	7.37E-02	RASGRF1	-	3.39E-07	3.79E-01	1.74E-04	4246	
3	rs7641572	1.05E+08	8.96E-01	N/A	15	rs7169735	77090508	7.37E-02	RASGRF1	-	3.39E-07	3.79E-01	1.74E-04	4247	
1	rs4658609	2.42E+08	2.39E-01	N/A	18	rs9956663	52932253	5.71E-01	N/A	-	3.39E-07	6.71E-01	3.23E-05	4248	
4	rs12504598	54284822	1.49E-01	N/A	9	rs4501686	11420659	8.61E-01	N/A	-	3.39E-07	9.74E-01	3.38E-04	4249	
1	rs4233351	1.58E+08	9.47E-01	N/A	3	rs12494196	85796570	2.19E-01	CADM2	-	3.40E-07	2.23E-01	8.93E-06	4250	
2	rs785259	1.91E+08	9.00E-01	C2orf88	14	rs4899357	70047887	3.99E-01	ADAM20	-	3.40E-07	6.92E-01	1.46E-04	4251	
2	rs2160328	2.06E+08	9.36E-01	NRP2	9	rs13295839	1.14E+08	4.66E-01	UGCG	-	3.40E-07	4.24E-02	8.44E-03	4252	
2	rs7605725	43495131	5.05E-01	THADA	13	rs4773843	94637496	6.38E-01	ABCC4	-	3.40E-07	5.44E-02	4.29E-03	4253	
1	rs11203423	17780092	4.50E-01	ARHGEF10L	7	rs7795893	1.04E+08	8.37E-01	LHFPL3	-	3.40E-07	2.70E-01	3.59E-04	4254	

SNP A					SNP B						Interaction P			Ranking	Cluster in top 100 interactions
CHR	SNP	Location	gle locus P va	Gene	CHR	SNP	Location	Single locus P valu	Gene	MHC region	Stage 1	Stage 2	Combined		
10	rs2254079	1.23E+08	5.45E-01	N/A	16	rs9933953	76467193	6.69E-01	VAT1L	-	3.40E-07	5.29E-01	1.34E-05	4255	
10	rs2918605	1.23E+08	5.45E-01	N/A	16	rs9933953	76467193	6.69E-01	VAT1L	-	3.40E-07	5.29E-01	1.34E-05	4256	
10	rs1871445	62218680	6.59E-01	CDK1	17	rs1353618	28653255	3.18E-01	ACCN1	-	3.40E-07	4.01E-01	1.96E-04	4257	
9	rs1934659	4123945	4.27E-01	GLIS3	21	rs7278004	44461309	9.54E-01	ICOSLG	-	3.40E-07	2.21E-01	1.54E-03	4258	
5	rs1427899	1.26E+08	3.90E-01	N/A	22	rs5760855	23830496	2.15E-01	KIAA1671	-	3.40E-07	2.75E-01	1.66E-03	4259	
6	rs6939257	1.52E+08	5.15E-01	ESR1	16	rs4595825	74948744	9.00E-01	CNTN/AP4	-	3.40E-07	6.88E-01	6.42E-04	4260	
2	rs12052617	1.03E+08	3.74E-01	N/A	10	s11258894	14223377	6.07E-01	FRMD4A	-	3.40E-07	3.58E-01	1.27E-03	4261	
2	rs7603249	1.43E+08	5.90E-01	N/A	17	s12941887	10847741	5.60E-01	N/A	-	3.40E-07	5.18E-01	1.68E-05	4262	
1	rs6699355	35157192	3.16E-01	DLGAP3	2	rs2577703	23445724	6.86E-01	KLHL29	-	3.41E-07	9.34E-01	4.55E-05	4263	
6	rs1359067	16804703	8.99E-01	ATXN1	14	s10083460	33502972	8.02E-01	EGLN3	-	3.41E-07	2.64E-01	7.39E-06	4264	
9	rs10813281	30514250	8.75E-01	N/A	21	rs9984627	33393473	3.80E-01	N/A	-	3.41E-07	8.08E-01	5.21E-05	4265	
11	rs1790474	1.19E+08	4.13E-01	N/A	15	rs2415128	69978127	6.51E-01	MYO9A	-	3.41E-07	9.86E-01	1.16E-04	4266	
17	rs6501526	67761913	5.02E-02	N/A	20	rs4911134	31915876	6.63E-01	CHMP4B	-	3.41E-07	3.37E-01	3.99E-06	4267	
17	rs8068238	67760317	5.02E-02	N/A	20	rs4911134	31915876	6.63E-01	CHMP4B	-	3.41E-07	3.37E-01	3.99E-06	4268	
3	rs1501892	1.23E+08	6.14E-01	CASR	10	rs2589446	56634203	2.40E-01	N/A	-	3.41E-07	2.92E-01	8.85E-04	4269	
2	rs2164861	1.83E+08	6.71E-01	PDE1A	4	s10020119	1.84E+08	9.64E-01	ODZ3	-	3.41E-07	4.87E-01	9.22E-06	4270	
6	rs6927442	1.45E+08	4.00E-01	UTRN	2	s12614817	49124921	5.01E-01	FSHR	-	3.41E-07	5.33E-01	4.44E-05	4271	
1	rs7544975	1.06E+08	1.91E-01	N/A	2	rs1374274	46908673	9.45E-01	N/A	-	3.41E-07	6.18E-01	5.50E-04	4272	
2	rs2042542	2.18E+08	1.32E-01	TNS1	12	rs1245840	78178449	9.17E-01	SYT1	-	3.41E-07	7.48E-01	1.34E-05	4273	
1	rs10495181	2.2E+08	2.04E-01	N/A	2	rs6705285	66562972	1.71E-01	MEIS1	-	3.41E-07	8.77E-01	8.69E-05	4274	
5	rs3797937	9244362	9.13E-01	SEMA5A	9	s10810975	18509313	9.75E-01	ADAMTSL1	-	3.41E-07	2.93E-02	4.78E-08	4275	
12	rs17027488	96741146	2.20E-01	N/A	16	rs7200819	4885986	2.92E-01	PPL	-	3.41E-07	1.15E-01	7.34E-06	4276	
5	rs6893965	1.74E+08	8.40E-01	N/A	13	rs7982606	91342134	2.22E-01	GPC5	-	3.41E-07	N/A	N/A	4277	
10	rs814620	90412116	8.85E-01	LIPF	21	rs2837688	40829567	3.03E-01	DSCAM	-	3.41E-07	4.07E-01	2.63E-06	4278	
5	rs1345592	1.69E+08	1.10E-01	SLIT3	9	rs1663762	74466718	1.36E-01	TMC1	-	3.42E-07	6.63E-01	7.33E-05	4279	
5	rs323700	2617243	6.01E-01	N/A	8	rs7824696	53389772	4.34E-01	ST18	-	3.42E-07	2.84E-01	4.73E-04	4280	
8	rs1063582	23223298	9.95E-01	LOXL2	19	s12052065	17102649	9.51E-01	MYO9B	-	3.42E-07	1.51E-01	8.39E-07	4281	
8	rs4273857	23228998	9.95E-01	LOXL2	19	s12052065	17102649	9.51E-01	MYO9B	-	3.42E-07	1.89E-01	1.16E-06	4282	
4	rs568752	1.46E+08	8.25E-01	N/A	7	rs3857649	1.55E+08	1.43E-02	N/A	-	3.42E-07	4.67E-01	3.01E-05	4283	
20	rs6132511	22327060	5.83E-01	LOC284788	20	rs2244440	36823420	2.11E-01	ACTR5	-	3.42E-07	4.33E-01	6.69E-06	4284	
9	rs12685329	715147	1.35E-01	KANK1	22	rs9612003	41821213	3.03E-01	BIK	-	3.42E-07	7.88E-01	1.34E-05	4285	
8	rs17343241	37384133	8.16E-01	N/A	13	rs4445762	1E+08	8.77E-02	N/A	-	3.42E-07	1.22E-01	6.14E-07	4286	
17	rs8081973	74152185	5.28E-02	N/A	20	rs162079	46218152	5.59E-01	N/A	-	3.42E-07	3.07E-01	1.05E-03	4287	
4	rs1357133	54478962	7.64E-01	N/A	17	rs9894442	67556774	6.47E-01	N/A	-	3.42E-07	6.91E-01	1.91E-04	4288	
13	rs879681	1.06E+08	2.38E-01	N/A	19	s12974420	46490500	9.05E-01	HNRNPUL1	-	3.43E-07	4.71E-02	9.20E-03	4289	
7	rs7800424	25102342	4.92E-01	N/A	10	rs927915	7744923	5.25E-01	ITIH5	-	3.43E-07	7.83E-01	2.11E-04	4290	
7	rs7800424	25102342	4.92E-01	N/A	10	s12763796	7735774	5.25E-01	ITIH5	-	3.43E-07	8.68E-01	1.65E-04	4291	
12	rs7307618	86287617	2.51E-01	N/A	22	rs5758913	41478203	8.66E-01	N/A	-	3.43E-07	5.24E-01	2.55E-04	4292	
12	rs7968713	86286718	2.51E-01	N/A	22	rs5758913	41478203	8.66E-01	N/A	-	3.43E-07	5.24E-01	2.55E-04	4293	
11	rs2015016	94126105	3.78E-01	AMOTL1	16	rs6500840	6795938	6.00E-01	A2BP1	-	3.43E-07	5.67E-01	8.48E-05	4294	
11	rs2851573	94127142	3.78E-01	AMOTL1	16	rs6500840	6795938	6.00E-01	A2BP1	-	3.43E-07	5.67E-01	8.48E-05	4295	
7	rs6966644	19360045	3.98E-01	N/A	13	rs7328412	71569296	5.20E-02	N/A	-	3.43E-07	6.31E-01	2.57E-05	4296	
8	rs1421247	15691228	9.32E-01	N/A	22	rs735455	20743793	9.48E-01	N/A	-	3.43E-07	6.90E-01	1.93E-05	4297	
1	rs769891	55704888	3.77E-01	N/A	8	rs1474239	20904371	1.42E-01	N/A	-	3.43E-07	8.51E-01	2.42E-05	4298	
7	rs6960241	1.11E+08	6.68E-02	DOCK4	15	rs752476	52032440	9.77E-01	N/A	-	3.43E-07	1.56E-01	3.76E-06	4299	
1	rs6680807	1.99E+08	7.42E-01	N/A	16	rs6499904	56223857	9.91E-01	GPR56	-	3.43E-07	3.39E-01	4.62E-04	4300	
9	rs12553421	1.13E+08	8.62E-01	MUSK	10	rs1409390	14667852	2.78E-01	FAM107B	-	3.43E-07	4.81E-01	2.06E-04	4301	
2	rs13404712	52797294	9.74E-01	N/A	4	s13138440	1.87E+08	3.94E-01	SORBS2	-	3.43E-07	3.75E-01	3.91E-04	4302	
6	rs2858331	32789255	1.42E-01	N/A	10	rs7895606	52228498	7.03E-01	A1CF	MHC	3.43E-07	4.16E-01	1.78E-06	4303	
9	rs944591	83372048	1.28E-01	TLE1	11	rs2845835	1.25E+08	8.13E-01	FEZ1	-	3.44E-07	7.18E-01	1.15E-04	4304	
8	rs10102198	28738513	3.13E-01	INTS9	12	rs7294478	7158081	7.76E-01	RBP5	-	3.44E-07	8.44E-02	2.75E-03	4305	
4	rs6835711	1.66E+08	3.23E-01	1-Mar	10	rs7094555	1.29E+08	7.56E-01	DOCK1	-	3.44E-07	2.96E-01	3.50E-06	4306	
2	rs4849084	1.13E+08	5.35E-01	N/A	12	rs2029939	61132374	8.84E-01	MON2	-	3.44E-07	4.63E-01	5.64E-04	4307	
15	rs7179965	22676011	6.11E-01	SNRPN	19	rs3752174	2465565	4.35E-01	GNG7	-	3.44E-07	2.52E-01	1.83E-06	4308	
17	rs2620018	69251595	1.62E-01	C17orf54	22	rs1640299	18478359	8.57E-01	TRMT2A	-	3.44E-07	4.13E-01	4.71E-06	4309	
6	rs2503661	92817083	2.15E-01	N/A	19	rs2682597	48635339	2.85E-01	N/A	-	3.44E-07	5.94E-01	1.74E-04	4310	
16	rs9930914	5965640	8.30E-01	N/A	21	rs2254505	17006156	1.80E-01	N/A	-	3.44E-07	9.95E-01	1.67E-04	4311	
6	rs4140396	22797053	1.67E-02	N/A	6	rs4712708	22817582	6.75E-01	N/A	-	3.44E-07	1.68E-01	2.07E-06	4312	
2	rs2528626	1.59E+08	1.37E-01	N/A	20	rs373217	54905130	2.73E-01	N/A	-	3.44E-07	6.17E-01	5.92E-06	4313	
6	rs6921516	1.61E+08	4.89E-01	LPA	7	rs7798471	6711482	4.88E-01	ZNF12	-	3.44E-07	N/A	N/A	4314	
5	rs11241882	1.26E+08	7.99E-01	N/A	15	rs2725580	52050006	7.12E-01	N/A	-	3.44E-07	6.80E-01	3.42E-04	4315	
5	rs1169654	24842350	3.66E-01	N/A	9	rs4740766	4294915	6.89E-01	GLIS3	-	3.44E-07	4.46E-01	2.18E-05	4316	
1	rs6672120	42350031	8.62E-01	N/A	2	rs227782	71720350	1.32E-01	DYSF	-	3.44E-07	5.06E-01	1.71E-03	4317	
2	rs2861680	79006430	1.64E-01	N/A	11	s11227133	55593196	2.25E-01	N/A	-	3.44E-07	5.08E-01	2.61E-04	4318	
10	rs814620	90412116	8.85E-01	LIPF	21	rs2837682	40826362	6.19E-01	DSCAM	-	3.44E-07	1.67E-01	3.04E-04	4319	
3	rs7613069	10600996	5.88E-01	N/A	14	s12433895	21692350	8.97E-02	N/A	-	3.44E-07	6.49E-01	8.14E-04	4320	
5	rs12523278	60599664	1.69E-01	N/A	7	rs6945560	19231029	1.98E-01	N/A	-	3.44E-07	8.30E-01	8.52E-05	4321	
1	rs4129186	2.13E+08	6.85E-01	PTPN14	13	s11069721	1.08E+08	5.06E-02	N/A	-	3.45E-07	4.71E-01	1.35E-05	4322	
4	rs4368681	1.1E+08	5.11E-01	COL25A1	18	rs2551402	59141002	8.89E-01	BCL2	-	3.45E-07	6.59E-01	1.04E-05	4323	
4	rs4368681	1.1E+08	5.11E-01	COL25A1	18	rs1462129	59131851	8.89E-01	BCL2	-	3.45E-07	7.32E-01	1.33E-05	4324	
3	rs2033857	1.66E+08	5.05E-01	N/A	8	rs6993893	1.15E+08	8.61E-01	N/A	-	3.45E-07	9.71E-01	2.52E-05	4325	
13	rs7335635	35814985	6.44E-01	SPG20	19	rs8111509	57003307	1.74E-01	FPR3	-	3.45E-07	7.13E-02	1.09E-02	4326	
11	rs1549489	4393046	8.44E-01	N/A	15	rs4906809	23737373	1.01E-01	N/A	-	3.45E-07	4.25E-01	1.08E-03	4327	
18	rs7238355	32291755	3.85E-01	FHOD3	19	rs919271	57360226	2.20E-01	ZNF836	-	3.45E-07	8.64E-01	8.16E-05	4328	
2	rs1542179	1.01E+08	1.26E-01	NPAS2	12	rs1015390	46630305	7.87E-01	TMEM106C	-	3.45E-07	4.31E-03	2.49E-02	4329	
11	rs4909926	10373830	2.52E-01	N/A	20	rs2903015	56598801	2.78E-01	N/A	-	3.45E-07	4.52E-02	1.67E-02	4330	
12	rs640014	51249181	3.51E-01	KRT71	19	rs175530	61377275	3.88E-01	ZNF444	-	3.45E-07	7.37E-01	2.47E-04	4331	
2	rs4675812	2.42E+08	7.38E-01	FARP2	15	rs2047667	51126053	4.76E-02	N/A	-	3.45E-07	1.98E-03	2.08E-02	4332	
13	rs9511484	24339945	5.54E-01	N/A	14	rs2070337	30289934	8.04E-01	SCFD1	-	3.45E-07	1.87E-01	4.22E-04	4333	
14	rs1299512	32653876	2.73E-01	NPAS3	15	rs1346037	59925203	7.83E-01	VPS13C	-	3.45E-07	2.38E-01	7.83E-04	4334	
1	rs729071	1.63E+08	3.62E-01	PBX1	8	rs7386394	1.43E+08	4.92E-01	N/A	-	3.45E-07	3.33E-01	4.20E-04	4335	
9	rs2129626	17061162	6.52E-04	N/A	19	rs887270	2501852	4.15E-01	GNG7	-	3.45E-07	3.93E-01	1.02E-05	4336	
9	rs2774633	17060633	6.52E-04	N/A	19	rs887270	2501852	4.15E-01	GNG7	-	3.45E-07	3.93E-01	1.02E-05	4337	
4	rs3804162	1.22E+08	8.94E-02	PRDM5	12	rs4463925	14153103	3.61E-01	N/A	-	3.45E-07	7.00E-01	6.23E-05	4338	
6	rs2504892	12667225	5.86E-01	N/A	7	rs1159329	67261520	9.33E-01	N/A	-	3.45E-07	8.99E-01	2.18E-05	4339	
6	rs9374070	1.1E+08	6.74E-01	N/A	12	rs1805510	13642519	9.10E-01	GRIN2B	-	3.45E-07	1.43E-01	1.08E-06	4340	
5	rs2909920	1.2E+08	8.44E-01	N/A	20	rs580544	47638833	9.25E-01	N/A	-	3.45E-07	3.47E-01	6.39E-06	4341	
1	rs4650224	73939547	8.27E-02	N/A	10	rs1227079	73122633	3.67E-01	CDH23	-	3.46E-07	2.01E-01	4.45E-03	4342	
6	rs6939257	1.52E+08	5.15E-01	ESR1	16	rs1428753	74961558	9.45E-01	CNTN/AP4	-	3.46E-07	4.01E-01	1.80E-03	4343	
3	rs1317443	1685731	6.52E-01	N/A	8	rs6994715	1.43E+08	8.41E-03	FLJ43860	-	3.46E-07	5.06E-01	5.37E-04	4344	
3	rs2715609	1.35E+08	4.13E-02	RAB6B	9	rs542808	1.1E+08	5.97E-02	N/A	-	3.46E-07	5.38E-01	1.14E-04	4345	
3	rs1606851	21812431	1.06E-01	N/A	3	rs1497762	1.61E+08	9.59E-01	SCHIP1	-	3.46E-07	2.29E-01	1.16E-06	4346	
9	rs10757736	28368229	4.29E-02	LINGO2	13	rs7330519	94673419	5.16E-01	ABCC4	-	3.46E-07	4.99E-01	2.21E-05	4347	
12	rs7300814	11998148	8.86E-03	N/A	20	rs2206900	7888429	1.27E-01	HAO1	-	3.46E-07	7.39E-01	2.13E-04	4348	
1	rs6701300	8123277	1.37E-01	N/A	7	rs1229492	81402058	1.21E-01	CACN/A2D1	-	3.46E-07	7.53E-01	7.56E-05	4349	
3	rs11706042	1.21E+08	6.10E-01	CDGAP	18	rs339866	20462599	6.44E-03	N/A	-	3.46E-07	8.86E-01	9.58E-05	4350	
3	rs11129702	36273457	6.02E-01	N/A	16	s12149452	6977388	9.69E-01	A2BP1	-	3.46E-07	6.68E-01	1.24E-05	4351	
4	rs7665758	46652454	1.94E-01	GABRA4	17	s12601473	4272789	1.83E-01	SPNS3	-	3.46E-07	3.81E-01	2.25E-04	4352	
1	rs4652997	34451653	4.14E-01	C1orf94	8	rs2056666	5251734	3.47E-01	N/A	-	3.46E-07	7.68E-01	3.10E-05	4353	
7	rs10255418	6037382	2.49E-03	EIF2AK1	14	rs1958643	53390192	7.78E-02	N/A	-	3.46E-07	2.92E-01	1.09E-05	4354	
6	rs1858038	87209749	5.60E-01	N/A	1	rs748142	48281640	6.07E-01	N/A	-	3.46E-07	5.80E-01	1.31E-03	4355	
6	rs9375683	1.3E+08	9.08E-01	N/A	21	rs2825529	19634654	1.41E-01	N/A	-	3.46E-07	6.91E-01	2.54E-05	4356	
4	rs1493708	76501826	7.84E-01	N/A	16	rs4787646	24549872	7.29E-01	N/A	-	3.46E-07	2.61E-01	1.19E-03	4357	
9	rs4878057	89126324	7.54E-01	N/A	12	rs7974686	995736	7.08E-01	ERC1	-	3.46E-07	3.65E-01	6.14E-04	4358	
5	rs10057782	1.6E+08	4.96E-01	N/A	12	rs3741781	1.08E+08	5.09E-01	SSH1	-	3.47E-07	2.56E-01	1.26E-03	4359	
11	rs4757259	12172122	1.36E-01	MICAL2	18	rs1573499	35409444	6.09E-04	LOC647946	-	3.47E-07	5.00E-01	8.72E-05	4360	
6	rs2504853	12643097	3.49E-01	N/A	7	s12698670	67256918	8.26E-01	N/A	-	3.47E-07	8.43E-01	5.38E-05	4361	
1	rs274587	74716053	5.38E-01	TNNI3K	1	rs863035	1.57E+08	8.74E-01	N/A	-	3.47E-07	7.88E-01	3.95E-05	4362	
1	rs274604	74729913	5.38E-01	TNNI3K	1	rs863035	1.57E+08	8.74E-01	N/A	-	3.47E-07	7.88E-01	3.95E-05	4363	
2	rs842064	1.6E+08	2.17E-01	TANC1	17	rs2165846	42296365	3.50E-01	WNT9B	-	3.47E-07	2.70E-01	2.36E-07	4364	
5	rs4416586	72530549	9.87E-01	N/A	8	s13269478	1.39E+08	8.93E-01	FAM135B	-	3.47E-07	9.34E-01	7.74E-05	4365	
7	rs1404634	1.43E+08	6.39E-01	N/A	18	rs4371250	72578561	8.81E-01	N/A	-	3.47E-07	9.79E-02	5.93E-07	4366	

SNP A					SNP B						Interaction P			Ranking	Cluster in top 100 interactions
CHR	SNP	Location	gle locus P va	Gene	CHR	SNP	Location	Single locus P valu	Gene	MHC region	Stage 1	Stage 2	Combined		
6	rs9502003	3384636	1.17E-01	SLC22A23	9	rs959066	1.04E+08	3.29E-03	N/A	-	3.47E-07	1.91E-01	3.89E-03	4367	
6	rs6940601	1.66E+08	2.20E-01	N/A	2	rs7590342	59113908	3.13E-01	N/A	-	3.47E-07	5.92E-01	3.81E-04	4368	
3	rs622205	1.84E+08	8.78E-01	ATP11B	5	rs1592937	1.81E+08	5.09E-01	TRIM7	-	3.47E-07	2.80E-01	1.84E-06	4369	
1	rs6702842	1.5E+08	1.51E-01	SNX27	4	rs6817264	39194505	8.61E-01	N/A	-	3.47E-07	3.81E-01	2.64E-04	4370	
5	rs13436137	3818085	6.43E-01	N/A	7	rs917117	28142830	7.24E-02	JAZF1	-	3.47E-07	5.20E-01	1.20E-05	4371	
16	rs1072123	50596711	4.09E-01	N/A	22	rs1013513	33683740	5.86E-01	N/A	-	3.47E-07	8.82E-01	1.22E-04	4372	
5	rs256219	1.33E+08	3.57E-01	N/A	16	rs17775878	26460280	1.73E-01	N/A	-	3.48E-07	4.80E-01	6.65E-06	4373	
6	rs2038475	1.55E+08	7.36E-01	N/A	9	rs10739098	6407677	2.18E-01	UHRF2	-	3.48E-07	7.17E-01	6.04E-05	4374	
9	rs10746796	89100633	1.87E-01	N/A	10	rs10998957	71198936	5.17E-01	N/A	-	3.48E-07	8.55E-01	6.64E-06	4375	
1	rs631272	12129342	4.26E-01	TNFRSF8	14	rs2000612	87685145	5.29E-01	N/A	-	3.48E-07	9.28E-01	1.13E-04	4376	
2	rs1615929	1.61E+08	6.08E-01	N/A	11	rs6592878	79405363	6.40E-01	N/A	-	3.48E-07	2.00E-01	1.44E-03	4377	
2	rs11126537	76598448	1.06E-01	N/A	3	rs1482038	14296181	5.73E-01	N/A	-	3.48E-07	6.68E-01	4.37E-05	4378	
2	rs11679162	76598844	1.06E-01	N/A	3	rs1482038	14296181	5.73E-01	N/A	-	3.48E-07	6.68E-01	4.37E-05	4379	
8	rs10156288	1.36E+08	5.46E-01	N/A	13	rs1340741	68418063	2.94E-01	N/A	-	3.48E-07	5.47E-02	2.72E-07	4380	
8	rs7013295	1.36E+08	5.46E-01	N/A	13	rs1340741	68418063	2.94E-01	N/A	-	3.48E-07	5.47E-02	2.72E-07	4381	
7	rs10486500	32117895	9.71E-01	N/A	14	rs12587447	93836479	8.24E-02	SERPIN/A10	-	3.48E-07	7.63E-01	5.48E-05	4382	
2	rs16824376	1.98E+08	7.80E-01	PLCL1	7	rs12699758	15930763	7.54E-01	N/A	-	3.48E-07	4.94E-01	6.16E-05	4383	
2	rs996427	1.98E+08	7.80E-01	PLCL1	7	rs12699758	15930763	7.54E-01	N/A	-	3.48E-07	4.94E-01	6.16E-05	4384	
2	rs1085448	44517309	6.63E-01	C2orf34	3	rs10933677	1.96E+08	7.25E-01	GP5	-	3.48E-07	9.21E-01	1.24E-04	4385	
8	rs983674	1.04E+08	3.95E-01	N/A	11	rs353626	35145821	4.67E-01	CD44	-	3.48E-07	1.45E-01	5.00E-07	4386	
13	rs4942324	43829134	8.92E-01	SERP2	19	rs7252746	53476029	4.99E-01	CCDC114	-	3.48E-07	2.47E-01	5.47E-04	4387	
2	rs872162	1.8E+08	9.32E-01	ZNF385B	3	rs358741	1.57E+08	8.76E-01	C3orf33	-	3.48E-07	4.32E-01	2.11E-04	4388	
3	rs13094598	35709235	3.90E-01	ARPP-21	9	rs7040543	1.33E+08	6.13E-01	ABL1	-	3.48E-07	5.78E-01	4.05E-05	4389	
5	rs12654281	1.07E+08	8.25E-01	N/A	10	rs538037	29481749	5.75E-01	N/A	-	3.48E-07	4.01E-01	5.93E-04	4390	
7	rs10255671	1.51E+08	4.48E-01	CRYGN	20	rs2148218	54324150	8.95E-01	N/A	-	3.48E-07	9.80E-02	4.57E-06	4391	
7	rs10255671	1.51E+08	4.48E-01	CRYGN	20	rs6024799	54338231	8.95E-01	N/A	-	3.48E-07	1.73E-01	9.61E-06	4392	
10	rs9663977	4507381	3.48E-01	N/A	16	rs4782717	81167218	2.78E-01	N/A	-	3.48E-07	2.31E-01	6.23E-07	4393	
12	rs12812221	55605603	5.43E-01	SDR9C7	15	rs10852212	25475248	1.27E-01	N/A	-	3.48E-07	4.03E-01	5.56E-06	4394	
1	rs12126518	94017704	5.48E-01	N/A	5	rs794731	1.74E+08	1.19E-01	N/A	-	3.48E-07	2.29E-01	3.05E-06	4395	
6	rs1265074	31221193	9.61E-01	TCF19	6	rs7750269	31379136	8.58E-01	N/A	MHC	3.48E-07	2.29E-01	3.05E-06	4396	
4	rs11732672	16133477	2.14E-01	LDB2	11	rs1514621	81759552	9.57E-01	N/A	-	3.48E-07	2.29E-01	3.05E-06	4397	
3	rs562516	59754716	8.44E-01	FHIT	16	rs3011249	61206793	2.25E-02	N/A	-	3.48E-07	4.36E-01	4.20E-04	4398	
6	rs1265093	31215166	9.61E-01	TCF19	6	rs7750269	31379136	8.58E-01	N/A	MHC	3.48E-07	1.45E-01	3.52E-03	4399	
6	rs1966	31215712	9.61E-01	TCF19	6	rs7750269	31379136	8.58E-01	N/A	MHC	3.48E-07	6.51E-01	1.36E-05	4400	
5	rs6862119	27393113	6.08E-02	N/A	11	rs217092	87610021	3.20E-02	N/A	-	3.49E-07	3.37E-01	1.30E-03	4401	
5	rs325339	27388996	6.08E-02	N/A	11	rs217092	87610021	3.20E-02	N/A	-	3.49E-07	N/A	N/A	4402	
1	rs4233351	1.58E+08	9.47E-01	N/A	3	rs11127905	85793026	2.44E-01	CADM2	-	3.49E-07	2.23E-01	8.99E-06	4403	
1	rs4233351	1.58E+08	9.47E-01	N/A	3	rs9860249	85770242	2.44E-01	CADM2	-	3.49E-07	2.23E-01	8.99E-06	4404	
7	rs1476882	19654304	3.29E-01	N/A	12	rs1353933	33035604	3.74E-01	N/A	-	3.49E-07	1.96E-01	6.48E-04	4405	
7	rs1421349	33866990	6.03E-01	N/A	9	rs7020876	84904859	6.69E-02	N/A	-	3.49E-07	2.15E-01	2.70E-03	4406	
6	rs7771674	71510285	7.97E-02	SMAP1	17	rs766304	64102446	9.81E-01	FAM20A	-	3.49E-07	7.69E-01	3.47E-05	4407	
1	rs631272	12129342	4.26E-01	TNFRSF8	14	rs1940550	87673873	4.41E-01	N/A	-	3.49E-07	9.28E-01	1.11E-04	4408	
1	rs1538370	1.63E+08	4.23E-01	N/A	13	rs970028	87375435	5.74E-01	N/A	-	3.49E-07	9.69E-01	1.80E-05	4409	
18	rs4491635	65337037	9.24E-01	DOK6	20	rs6123631	54828815	2.98E-01	N/A	-	3.49E-07	6.96E-01	4.06E-05	4410	
18	rs4545912	65331047	9.24E-01	DOK6	20	rs6123631	54828815	2.98E-01	N/A	-	3.49E-07	6.96E-01	4.06E-05	4411	
7	rs4717554	69908273	3.15E-02	AUTS2	17	rs12943089	73084524	3.59E-01	N/A	-	3.49E-07	9.47E-01	4.49E-05	4412	
6	rs7745179	92910090	9.16E-01	N/A	2	rs12617448	1.22E+08	9.88E-01	N/A	-	3.49E-07	3.25E-01	2.02E-04	4413	
8	rs7828550	82813796	9.93E-01	N/A	17	rs9674559	76330203	3.60E-01	RPTOR	-	3.49E-07	7.50E-01	5.31E-04	4414	
6	rs365515	1.46E+08	5.08E-01	N/A	8	rs4518624	1.31E+08	9.16E-01	N/A	-	3.49E-07	7.45E-02	7.91E-07	4415	
12	rs4882801	1.28E+08	3.44E-01	TMEM132C	13	rs9563011	50371905	8.96E-01	RN/ASEH2B	-	3.50E-07	9.57E-01	1.63E-04	4416	
3	rs4550855	99419735	6.43E-01	N/A	16	rs6500343	49448844	5.45E-01	N/A	-	3.50E-07	2.17E-01	9.86E-04	4417	
1	rs11586497	33823829	3.60E-01	CSMD2	10	rs12569498	1.26E+08	5.95E-01	LHPP	-	3.50E-07	3.02E-01	1.76E-06	4418	
3	rs4856387	82524502	9.74E-01	N/A	9	rs1808789	1.07E+08	9.30E-01	N/A	-	3.50E-07	5.55E-02	1.59E-07	4419	
6	rs9383633	1.5E+08	4.17E-01	RAET1K	9	rs4838278	1.27E+08	4.28E-01	MAPKAP1	-	3.50E-07	1.90E-01	5.15E-04	4420	
2	rs13382215	2.15E+08	7.25E-01	VWC2L	14	rs10137605	59379238	2.07E-01	RTN1	-	3.50E-07	4.45E-01	2.39E-05	4421	
6	rs6455688	1.61E+08	6.19E-01	LPA	7	rs7798471	6711482	4.88E-01	ZNF12	-	3.50E-07	1.51E-01	1.01E-06	4422	
4	rs7679087	1.35E+08	4.46E-01	N/A	10	rs2243812	1.35E+08	4.28E-01	LOC619207	-	3.50E-07	7.60E-01	4.48E-04	4423	
4	rs6447849	8135460	1.41E-01	ABLIM2	5	rs905350	3813907	4.91E-01	N/A	-	3.50E-07	8.51E-01	1.73E-04	4424	
6	rs2233984	31187243	4.20E-02	C6orf15	6	rs2394990	31448538	6.80E-02	HLA-B	MHC	3.50E-07	5.14E-02	4.20E-07	4425	
2	rs341605	44917691	3.10E-02	N/A	15	rs690316	41525172	1.24E-01	TP53BP1	-	3.50E-07	7.08E-01	3.23E-04	4426	
14	rs749395	69414527	2.94E-01	SMOC1	16	rs591940	55925679	4.39E-01	N/A	-	3.50E-07	8.61E-01	4.19E-05	4427	
6	rs3868542	31253818	6.85E-02	TCF19	6	rs9368675	31380136	7.91E-01	N/A	MHC	3.51E-07	9.63E-01	6.30E-05	4428	
5	rs1423236	67916441	9.31E-01	N/A	20	rs6123674	55196354	2.99E-02	BMP7	-	3.51E-07	2.99E-01	6.63E-06	4429	
8	rs924184	28352090	8.82E-01	FBXO16	8	rs7008604	1.25E+08	6.37E-01	FER1L6	-	3.51E-07	9.89E-01	3.04E-05	4430	
6	rs674026	1.51E+08	7.55E-02	IYD	10	rs11193430	1.09E+08	6.89E-01	N/A	-	3.51E-07	1.17E-01	3.88E-07	4431	
9	rs7021631	7493994	1.66E-02	N/A	15	rs11857297	69643146	6.16E-01	THSD4	-	3.51E-07	N/A	N/A	4432	
6	rs1536242	6876009	6.02E-01	N/A	5	rs217259	1.37E+08	4.99E-01	WNT8A	-	3.51E-07	6.27E-01	4.82E-05	4433	
2	rs6721784	2.38E+08	2.41E-02	LRRFIP1	14	rs4254262	1.04E+08	8.90E-01	N/A	-	3.51E-07	N/A	N/A	4434	
12	rs2448064	12118362	4.13E-01	BCL2L14	14	rs882270	30315684	9.65E-01	N/A	-	3.51E-07	5.34E-01	2.25E-04	4435	
5	rs2188464	1.36E+08	4.70E-02	N/A	19	rs4802605	54622533	5.19E-01	SLC17A7	-	3.51E-07	6.37E-01	8.59E-05	4436	
4	rs1963656	24933840	2.08E-02	ZCCHC4	5	rs6881214	24873656	6.85E-01	N/A	-	3.51E-07	1.16E-01	4.23E-07	4437	
8	rs16916039	52422619	9.65E-01	PXDNL	14	rs1476609	70291818	2.00E-01	MAP3K9	-	3.51E-07	1.34E-01	1.62E-06	4438	
8	rs7006959	28517242	3.41E-01	N/A	14	rs8004087	33560651	8.84E-01	N/A	-	3.51E-07	4.56E-01	2.23E-04	4439	
2	rs6746998	2.09E+08	1.89E-01	N/A	7	rs10273717	20519346	1.20E-01	N/A	-	3.51E-07	3.38E-01	6.68E-04	4440	
12	rs6488713	14834183	9.01E-01	WBP11	13	rs12875137	34199013	9.76E-01	N/A	-	3.51E-07	1.94E-01	4.82E-06	4441	
6	rs4714010	36855384	1.98E-01	CPNE5	15	rs720736	84937208	1.73E-01	AGBL1	MHC	3.52E-07	9.02E-01	1.99E-05	4442	
17	rs7219984	45051819	2.21E-01	SPOP	18	rs1539871	43721804	2.34E-01	SMAD2	-	3.52E-07	1.80E-01	6.97E-04	4443	
2	rs10171249	54346912	9.82E-01	ACYP2	2	rs4550612	1.43E+08	3.13E-01	N/A	-	3.52E-07	6.22E-01	1.03E-05	4444	
4	rs992363	1.76E+08	8.98E-01	N/A	8	rs4288398	6869048	3.91E-01	DEFA3	-	3.52E-07	7.30E-01	1.63E-04	4445	
8	rs7829925	83586789	7.73E-01	N/A	9	rs1548788	1.28E+08	4.99E-01	FAM125B	-	3.52E-07	5.13E-01	7.09E-04	4446	
4	rs9992520	1.2E+08	2.16E-02	N/A	18	rs12958117	18238667	6.26E-01	CTAGE1	-	3.52E-07	7.07E-01	2.84E-04	4447	
6	rs1220445	91712833	3.24E-01	N/A	6	rs2473517	1.39E+08	4.48E-01	N/A	-	3.52E-07	8.62E-01	6.12E-05	4448	
2	rs1515269	1.8E+08	9.87E-01	ZNF385B	3	rs358741	1.57E+08	8.76E-01	C3orf33	-	3.52E-07	2.45E-01	4.85E-04	4449	
2	rs3791289	1.35E+08	5.41E-01	MGAT5	17	rs8074370	74468926	5.41E-01	LGALS3BP	-	3.52E-07	3.23E-01	3.59E-06	4450	
3	rs6794945	1.35E+08	2.05E-01	SRPRB	9	rs542808	1.1E+08	5.97E-02	N/A	-	3.52E-07	5.27E-01	4.43E-05	4451	
8	rs9792175	11344528	6.56E-01	C8orf12	15	rs11247349	96851676	8.76E-01	FAM169B	-	3.52E-07	5.68E-01	4.57E-06	4452	
6	rs6927442	1.45E+08	4.00E-01	UTRN	2	rs11125198	49128954	4.37E-01	FSHR	-	3.52E-07	7.79E-01	1.27E-04	4453	
3	rs13081722	1.33E+08	7.94E-01	CPNE4	8	rs9644299	6032068	6.53E-02	N/A	-	3.52E-07	1.98E-01	8.65E-06	4454	
1	rs2504053	1.78E+08	8.61E-01	FAM163A	11	rs2303380	1.13E+08	5.91E-01	TTC12	-	3.52E-07	9.21E-01	3.35E-05	4455	
9	rs7865126	19092828	2.08E-01	PLIN2	14	rs1110386	94588593	8.34E-01	N/A	-	3.52E-07	1.71E-02	8.80E-07	4456	
1	rs7517475	66070749	3.91E-01	PDE4B	15	rs11073961	89284396	4.20E-01	UNC45A	-	3.52E-07	4.54E-01	2.27E-04	4457	
10	rs4993019	1.33E+08	6.54E-01	N/A	15	rs7497692	31850420	9.08E-01	RYR3	-	3.52E-07	8.98E-01	9.05E-05	4458	
4	rs869240	1.84E+08	3.74E-01	ODZ3	10	rs2279339	59815004	7.10E-01	UBE2D1	-	3.53E-07	4.69E-02	2.92E-07	4459	
6	rs4495304	31188697	4.80E-02	C6orf15	6	rs3130453	31232828	5.90E-01	TCF19	MHC	3.53E-07	9.22E-01	1.36E-04	4460	
2	rs1834619	17764966	5.69E-01	SMC6	8	rs10283194	34785928	7.98E-01	N/A	-	3.53E-07	3.41E-02	1.44E-02	4461	
2	rs10188985	85794585	8.93E-01	GNLY	18	rs11874608	18954411	1.92E-01	CABLES1	-	3.53E-07	5.83E-01	2.58E-05	4462	
12	rs7296020	61595594	4.84E-01	PPM1H	13	rs9523455	91398074	7.43E-01	GPC5	-	3.53E-07	7.49E-01	3.17E-04	4463	
9	rs12238357	12038206	4.82E-01	N/A	9	rs7049218	31015113	6.86E-02	N/A	-	3.53E-07	9.90E-01	2.18E-05	4464	
3	rs9846698	1.86E+08	7.18E-03	N/A	10	rs6593445	44679489	1.11E-01	N/A	-	3.53E-07	7.44E-01	9.47E-04	4465	
6	rs6928665	52548929	5.31E-01	TRAM2	18	rs4325639	9614796	9.98E-01	PPP4R1	-	3.53E-07	8.25E-01	2.10E-05	4466	
1	rs1409143	1.74E+08	3.05E-01	N/A	15	rs491274	44656818	2.01E-01	N/A	-	3.53E-07	1.07E-01	3.93E-03	4467	
1	rs6425361	1.74E+08	3.05E-01	N/A	15	rs491274	44656818	2.01E-01	N/A	-	3.53E-07	1.07E-01	3.93E-03	4468	
9	rs11103233	1.38E+08	9.44E-01	UBAC1	12	rs10785474	42604695	3.03E-01	TMEM117	-	3.53E-07	3.14E-01	4.03E-04	4469	
9	rs11103233	1.38E+08	9.44E-01	UBAC1	12	rs10785481	42611328	3.03E-01	TMEM117	-	3.53E-07	3.14E-01	4.03E-04	4470	
5	rs331390	91045508	8.16E-03	N/A	12	rs933420	2996301	8.04E-01	TEAD4	-	3.53E-07	6.12E-01	1.17E-04	4471	
5	rs2561063	91053281	8.16E-03	N/A	12	rs933420	2996301	8.04E-01	TEAD4	-	3.53E-07	6.87E-01	8.49E-05	4472	
12	rs11109097	96452568	5.25E-01	RMST	21	rs9983892	44898088	1.43E-01	C21orf29	-	3.53E-07	7.13E-01	4.72E-05	4473	
3	rs7626487	1.63E+08	6.10E-01	N/A	7	rs12674179	43179131	4.12E-01	HECW1	-	3.53E-07	N/A	N/A	4474	
6	rs7772351	88417736	3.23E-01	ORC3L	1	rs6687674	27409688	9.67E-01	N/A	-	3.54E-07	6.04E-01	2.92E-04	4475	
5	rs12656216	36196425	3.71E-01	SKP2	19	rs3746028	38009752	5.44E-01	SLC7A9	-	3.54E-07	5.21E-01	3.87E-04	4476	
5	rs10037027	85654459	2.73E-01	N/A	19	rs16979344	13751205	6.89E-01	CCDC130	-	3.54E-07	3.23E-02	5.84E-03	4477	
6	rs9383831	1.51E+08	3.69E-01	N/A	1	rs12030585	2E+08	2.92E-01	N/A	-	3.54E-07	7.90E-01	1.01E-05	4478	

SNP A					SNP B						Interaction P			Ranking	Cluster in top 100 interactions
CHR	SNP	Location	gle locus P va	Gene	CHR	SNP	Location	Single locus P valu	Gene	MHC region	Stage 1	Stage 2	Combined		
8	rs1405246	5345291	2.28E-01	N/A	14	rs8018930	94309119	5.71E-01	GSC	-	3.54E-07	2.67E-01	1.42E-03	4479	
15	rs17116519	23823618	9.64E-01	N/A	17	rs9899950	44183411	6.90E-01	TTLL6	-	3.54E-07	6.06E-01	2.20E-04	4480	
10	rs12766032	1.17E+08	5.38E-01	ATRNL1	13	rs1354033	84486546	1.17E-02	N/A	-	3.54E-07	7.23E-01	1.26E-03	4481	
6	rs4997129	856832	2.83E-01	N/A	5	's10072353	1.72E+08	1.55E-01	UBTD2	-	3.54E-07	9.77E-01	3.39E-05	4482	
8	rs4373508	59251479	4.35E-01	N/A	20	's11700048	6033006	4.64E-01	FERMT1	-	3.54E-07	2.91E-01	3.67E-05	4483	
8	rs4373508	59251479	4.35E-01	N/A	20	's11700084	6033216	4.64E-01	FERMT1	-	3.54E-07	3.40E-01	4.88E-05	4484	
5	rs6862959	38905836	6.57E-01	OSMR	17	rs4793859	52945965	2.68E-01	MSI2	-	3.54E-07	3.57E-01	1.08E-05	4485	
2	rs1220525	1.55E+08	7.30E-01	N/A	17	's12952904	13427823	4.34E-01	HS3ST3A1	-	3.54E-07	7.10E-01	4.72E-06	4486	
6	rs6912145	1.13E+08	4.48E-01	LAMA4	2	's12995942	16226902	7.82E-01	N/A	-	3.54E-07	1.24E-01	3.71E-04	4487	
6	rs7738951	1.13E+08	4.48E-01	LAMA4	2	's12995942	16226902	7.82E-01	N/A	-	3.54E-07	1.24E-01	3.71E-04	4488	
9	rs10810827	1767030	2.30E-01	N/A	14	rs1669794	45975802	8.14E-01	N/A	-	3.54E-07	1.72E-01	4.83E-06	4489	
4	rs12642503	1.81E+08	7.69E-01	N/A	7	's12698138	1.58E+08	8.50E-01	PTPRN2	-	3.54E-07	5.65E-01	3.95E-04	4490	
2	rs2216009	40444462	2.20E-02	SLC8A1	20	rs944268	59648074	9.38E-01	CDH4	-	3.54E-07	4.17E-01	1.69E-04	4491	
9	rs16922319	99393885	1.84E-01	TMOD1	10	rs2495718	1.02E+08	8.58E-01	N/A	-	3.55E-07	4.25E-01	4.69E-04	4492	
3	rs2712353	1.15E+08	5.03E-01	ATP6V1A	12	's10770664	20505291	2.75E-01	PDE3A	-	3.55E-07	5.94E-01	1.89E-05	4493	
6	rs9295957	31265572	2.18E-01	POU5F1	6	rs9357122	31368324	7.72E-01	N/A	MHC	3.55E-07	5.94E-01	1.89E-05	4494	
6	rs9295957	31265572	2.18E-01	POU5F1	6	rs9348862	31360726	7.72E-01	HLA-C	MHC	3.55E-07	N/A	N/A	4495	
13	rs9318228	73399211	4.98E-01	KLF12	16	rs734826	13397881	2.90E-01	N/A	-	3.55E-07	5.78E-01	1.78E-05	4496	
2	rs1385363	1.46E+08	3.18E-02	N/A	12	's17783679	4755323	2.81E-01	GALNT8	-	3.55E-07	5.27E-01	1.33E-03	4497	
2	rs4662248	1.46E+08	3.18E-02	N/A	12	's17783679	4755323	2.81E-01	GALNT8	-	3.55E-07	5.27E-01	1.33E-03	4498	
6	rs4235996	19576099	4.19E-01	N/A	1	rs6677214	1.98E+08	7.30E-01	N/A	-	3.55E-07	9.87E-01	7.75E-05	4499	
5	rs12516181	91074796	2.05E-01	N/A	16	rs1075072	53863076	6.97E-01	N/A	-	3.55E-07	2.29E-01	6.67E-06	4500	
8	rs10089434	3210534	1.83E-01	CSMD1	12	rs6582285	73977054	5.01E-01	CAPS2	-	3.55E-07	9.94E-01	2.75E-05	4501	
1	rs6600435	38907229	7.75E-01	N/A	3	rs2596617	24198314	1.15E-02	THRB	-	3.55E-07	1.35E-01	1.55E-06	4502	
2	rs6547148	77601021	6.85E-01	LRRTM4	13	rs884124	1.06E+08	7.21E-01	N/A	-	3.55E-07	2.45E-01	2.82E-06	4503	
3	rs9814549	1.01E+08	6.90E-01	C3orf26	8	's12682381	16946172	6.38E-01	EFHA2	-	3.55E-07	3.52E-01	6.42E-06	4504	
3	rs3821767	10898941	4.55E-01	SLC6A11	8	rs4736682	1.35E+08	9.37E-01	ST3GAL1	-	3.55E-07	9.07E-01	1.44E-05	4505	
6	rs1737031	29845460	4.76E-02	IFITM4P	10	rs7083541	30997484	6.83E-01	N/A	MHC	3.55E-07	7.25E-01	3.16E-05	4506	
5	rs12522949	51014850	1.08E-01	N/A	18	rs1857705	24385115	1.45E-01	N/A	-	3.55E-07	8.78E-01	1.16E-04	4507	
1	rs10800799	2E+08	7.20E-01	LMOD1	4	's13144541	79465233	5.01E-01	FRAS1	-	3.56E-07	5.01E-01	1.39E-03	4508	
6	rs197988	23701354	1.44E-01	N/A	22	rs5769389	47820135	9.90E-01	N/A	-	3.56E-07	5.40E-01	5.67E-05	4509	
8	rs1390357	18327160	6.45E-01	N/A	18	rs4479346	67700989	3.96E-01	N/A	-	3.56E-07	6.93E-01	9.90E-05	4510	
5	rs4615276	1.06E+08	1.15E-01	N/A	12	's12824017	61935103	2.85E-01	N/A	-	3.56E-07	8.11E-01	1.13E-04	4511	
2	rs6721784	2.38E+08	2.41E-02	LRRFIP1	14	's11622864	1.04E+08	4.14E-01	N/A	-	3.56E-07	N/A	N/A	4512	
1	rs1320594	53382960	9.77E-01	SLC1A7	1	rs1995873	2.16E+08	8.51E-01	N/A	-	3.56E-07	4.63E-01	2.49E-05	4513	
4	rs6819114	16156093	2.61E-01	LDB2	7	's10236729	64040200	5.24E-02	ZNF273	-	3.56E-07	5.05E-01	2.46E-05	4514	
1	rs10493673	80887077	7.44E-01	N/A	7	rs7458326	53285965	1.53E-02	N/A	-	3.56E-07	1.50E-01	1.52E-03	4515	
1	rs10493673	80887077	7.44E-01	N/A	7	rs6945796	53274224	1.53E-02	N/A	-	3.56E-07	1.81E-01	1.12E-03	4516	
1	rs12144807	79945399	6.54E-01	N/A	3	rs9879958	23163047	8.52E-03	N/A	-	3.56E-07	7.51E-01	3.85E-04	4517	
1	rs12144677	79944988	6.54E-01	N/A	3	rs9879958	23163047	8.52E-03	N/A	-	3.56E-07	9.36E-01	1.81E-04	4518	
10	rs12766032	1.17E+08	5.38E-01	ATRNL1	13	rs2496764	84477159	1.28E-02	N/A	-	3.56E-07	9.16E-01	6.81E-04	4519	
1	rs4130547	2.07E+08	8.78E-01	N/A	13	rs9522350	1.11E+08	2.41E-01	N/A	-	3.57E-07	6.45E-01	6.88E-05	4520	
1	rs4147822	94334077	5.28E-01	ABCA4	14	rs2430368	91457294	7.01E-02	FBLN5	-	3.57E-07	5.94E-02	6.36E-03	4521	
6	rs10948132	44418832	3.40E-01	SPATS1	1	rs8179271	1.54E+08	2.21E-01	ASH1L	-	3.57E-07	5.95E-01	8.16E-06	4522	
6	rs9367511	53118141	1.47E-01	GCM1	3	's10936353	1.65E+08	7.88E-01	N/A	-	3.57E-07	9.88E-01	2.79E-04	4523	
10	rs10763941	33802908	9.74E-01	N/A	11	rs563343	95882686	9.81E-01	N/A	-	3.57E-07	7.18E-01	1.58E-05	4524	
12	rs4766567	1.1E+08	1.30E-01	CUX2	20	rs6063947	51501548	2.15E-02	N/A	-	3.57E-07	2.29E-01	3.08E-06	4525	
16	rs7500355	84634601	7.85E-01	N/A	17	rs1960379	22098343	8.24E-01	N/A	-	3.57E-07	2.29E-01	3.08E-06	4526	
12	rs11179347	39672060	4.62E-02	CNTN1	18	rs168206	3683772	8.93E-01	DLGAP1	-	3.57E-07	2.29E-01	3.08E-06	4527	
6	rs1265074	31221193	9.61E-01	TCF19	6	rs9368675	31380136	7.91E-01	N/A	MHC	3.57E-07	4.52E-01	2.56E-05	4528	
6	rs1265093	31215166	9.61E-01	TCF19	6	rs9368675	31380136	7.91E-01	N/A	MHC	3.57E-07	2.99E-01	8.09E-03	4529	
6	rs1966	31215712	9.61E-01	TCF19	6	rs9368675	31380136	7.91E-01	N/A	MHC	3.57E-07	3.50E-01	6.42E-04	4530	
3	rs6787571	1.8E+08	4.09E-02	N/A	9	's10815463	668951	6.68E-01	KANK1	-	3.58E-07	9.09E-01	2.77E-05	4531	
10	rs2254535	72207879	8.64E-01	ADAMTS14	20	rs6113720	2257625	2.85E-01	TGM3	-	3.58E-07	6.39E-01	1.06E-03	4532	
2	rs2042542	2.18E+08	1.32E-01	TNS1	7	rs539840	54685819	1.58E-01	N/A	-	3.58E-07	4.10E-01	2.50E-04	4533	
6	rs1359067	16804703	8.99E-01	ATXN1	14	rs1809461	33501028	7.02E-01	EGLN3	-	3.58E-07	3.37E-01	1.14E-05	4534	
2	rs797703	47801295	8.31E-01	N/A	3	's12487334	1.21E+08	5.21E-01	CD80	-	3.58E-07	7.80E-01	2.98E-05	4535	
2	rs797703	47801295	8.31E-01	N/A	3	's12496520	1.21E+08	5.21E-01	CD80	-	3.58E-07	7.80E-01	2.98E-05	4536	
1	rs10873855	87770885	2.21E-02	N/A	22	rs926331	35840018	4.51E-01	TMPRSS6	-	3.58E-07	9.62E-01	6.55E-05	4537	
9	rs7874951	9968178	7.47E-02	PTPRD	21	rs2300395	35167068	6.29E-01	RUNX1	-	3.58E-07	1.48E-01	1.25E-06	4538	
2	rs10172608	57695579	6.23E-01	N/A	7	rs6969684	30204134	7.76E-01	N/A	-	3.58E-07	1.54E-01	7.40E-07	4539	
2	rs6705138	25441355	5.87E-01	DTNB	11	's10765675	93731257	6.51E-01	GPR83	-	3.58E-07	5.39E-01	4.37E-05	4540	
1	rs950302	1.65E+08	5.39E-01	DUSP27	12	rs2669010	75533191	8.86E-02	N/A	-	3.58E-07	N/A	N/A	4541	
5	rs4702704	10644672	3.92E-01	ANKRD33B	11	's10767804	29362561	7.92E-01	N/A	-	3.58E-07	9.59E-01	1.17E-04	4542	
6	rs969931	29602876	1.59E-01	N/A	7	's17204590	9233106	3.25E-01	N/A	MHC	3.58E-07	1.30E-01	1.58E-03	4543	
2	rs1595752	4863708	9.21E-02	N/A	8	rs7000712	1.33E+08	3.34E-01	EFR3A	-	3.59E-07	6.55E-01	9.28E-05	4544	
3	rs2131495	1.43E+08	5.28E-01	RASA2	20	rs6131220	11421176	6.47E-01	N/A	-	3.59E-07	3.41E-02	1.85E-07	4545	
1	rs10801687	89079849	5.85E-01	PKN2	16	's12443910	72711976	1.06E-01	N/A	-	3.59E-07	3.29E-01	8.19E-06	4546	
1	rs786911	89037836	5.85E-01	PKN2	16	's12443910	72711976	1.06E-01	N/A	-	3.59E-07	3.29E-01	8.19E-06	4547	
6	rs4895646	1.45E+08	6.08E-02	UTRN	13	rs9540711	65788311	9.70E-01	PCDH9	-	3.59E-07	7.86E-01	4.95E-05	4548	
5	rs10462906	1.74E+08	1.27E-01	N/A	9	rs504822	15232896	4.76E-01	TTC39B	-	3.59E-07	N/A	N/A	4549	
6	rs2233984	31187243	4.20E-02	C6orf15	6	rs130067	31226490	8.62E-01	TCF19	MHC	3.60E-07	2.73E-01	1.16E-04	4550	
10	rs431673	90484145	5.54E-01	LIPK	21	rs2837693	40832232	1.95E-01	DSCAM	-	3.60E-07	5.13E-01	2.02E-04	4551	
2	rs3112960	1.8E+08	6.24E-01	ZNF385B	3	rs358741	1.57E+08	8.76E-01	C3orf33	-	3.60E-07	9.97E-01	1.83E-04	4552	
5	rs607077	29531321	6.29E-01	N/A	7	rs8188345	61948628	2.18E-01	N/A	-	3.60E-07	N/A	N/A	4553	
8	rs923433	1.12E+08	3.90E-01	N/A	12	's11112403	1.04E+08	4.10E-01	APPL2	-	3.60E-07	5.96E-02	3.79E-07	4554	
9	rs11145038	78550841	2.72E-01	PRUNE2	11	's11026477	22234428	1.81E-01	ANO5	-	3.60E-07	6.19E-01	1.51E-05	4555	
8	rs1602091	1.28E+08	2.61E-01	N/A	16	rs2968161	83529138	3.80E-04	N/A	-	3.60E-07	3.03E-01	4.94E-06	4556	
8	rs6472100	64453126	6.41E-01	N/A	18	's12965296	8234875	2.97E-01	PTPRM	-	3.60E-07	3.62E-01	3.29E-04	4557	
2	rs1531968	2.4E+08	4.33E-01	N/A	2	rs2028446	35025447	4.74E-01	N/A	-	3.60E-07	1.69E-02	2.27E-08	4558	
7	rs7789215	77933529	9.47E-01	MAGI2	19	rs7254299	63576777	4.40E-01	ZNF497	-	3.60E-07	6.73E-01	2.44E-04	4559	
9	rs1537504	1.01E+08	8.11E-01	COL15A1	15	rs869884	75078653	4.88E-01	PSTPIP1	-	3.60E-07	7.34E-02	1.04E-07	4560	
9	rs1591029	25488831	8.02E-01	N/A	16	rs2127065	7141423	3.49E-01	RBFOX1	-	3.60E-07	6.46E-04	2.69E-09	4561	
2	rs13385191	20751746	7.46E-01	C2orf43	17	rs9914151	58959018	3.03E-01	KCNH6	-	3.60E-07	4.43E-02	5.64E-08	4562	
3	rs17803320	5466322	4.46E-01	N/A	3	's10511022	72626886	7.15E-01	N/A	-	3.60E-07	8.89E-01	8.79E-05	4563	
1	rs4147836	94289062	5.38E-01	ABCA4	15	rs189155	64115535	7.18E-01	MEGF11	-	3.60E-07	1.99E-01	1.90E-03	4564	
3	rs1526582	54871122	2.55E-01	CACN/A2D3	16	rs982205	27306957	2.20E-01	IL21R	-	3.60E-07	1.70E-01	2.74E-03	4565	
6	rs7745656	32788948	1.70E-01	N/A	10	rs7895606	52228498	7.03E-01	A1CF	MHC	3.60E-07	1.71E-01	1.08E-03	4566	
3	rs1463526	1.76E+08	4.94E-01	N/AALDL2	9	rs1332879	82881448	9.56E-01	N/A	-	3.60E-07	5.32E-01	1.19E-05	4567	
9	rs968357	76454609	4.53E-01	RORB	9	's10819538	1.31E+08	2.00E-01	N/A	-	3.61E-07	1.94E-01	1.93E-03	4568	
20	rs6132511	22327060	5.83E-01	LOC284788	20	rs2245231	36829534	2.32E-01	ACTR5	-	3.61E-07	5.10E-01	1.06E-05	4569	
9	rs1326809	1.19E+08	6.83E-01	ASTN2	17	rs801259	34580691	9.50E-04	PLXDC1	-	3.61E-07	5.61E-01	1.56E-04	4570	
7	rs289988	25480258	3.86E-02	N/A	15	's12903340	96760605	8.55E-01	N/A	-	3.61E-07	8.39E-01	1.53E-04	4571	
3	rs2350491	1.41E+08	4.26E-01	CLSTN2	3	's13326945	1.6E+08	4.06E-01	N/A	-	3.61E-07	5.67E-01	4.82E-04	4572	
5	rs4700436	56023055	7.81E-01	N/A	5	rs931452	1.19E+08	7.30E-01	N/A	-	3.61E-07	4.67E-01	1.08E-04	4573	
13	rs7999417	22451518	9.11E-01	N/A	22	rs6519519	23321863	8.84E-01	GGT1	-	3.61E-07	2.04E-01	2.87E-06	4574	
4	rs2314104	1.63E+08	9.04E-02	FSTL5	8	rs2035919	18151237	4.61E-01	N/A	-	3.61E-07	3.26E-02	3.43E-07	4575	
9	rs4130590	1.29E+08	9.17E-01	GARNL3	10	rs718299	7703675	2.88E-01	ITIH5	-	3.61E-07	6.52E-02	4.76E-07	4576	
1	rs1005716	1.81E+08	7.11E-01	LAMC2	11	rs4244525	26652919	2.96E-01	SLC5A12	-	3.61E-07	6.52E-02	4.76E-07	4577	
7	rs2188324	88162442	3.41E-01	N/A	13	rs9508666	29645725	6.46E-01	N/A	-	3.61E-07	6.52E-02	4.76E-07	4578	
11	rs12281121	80681472	5.23E-01	N/A	16	rs9938188	10389673	6.56E-02	N/A	-	3.61E-07	8.85E-01	1.34E-05	4579	
3	rs9851637	1.86E+08	7.18E-03	N/A	10	rs6593445	44679489	1.11E-01	N/A	-	3.61E-07	2.06E-01	9.77E-07	4580	
1	rs2494067	1.08E+08	8.74E-01	VAV3	9	rs3118536	1.36E+08	1.42E-01	RXRA	-	3.61E-07	2.47E-01	1.11E-06	4581	
6	rs998645	9399550	9.32E-01	N/A	4	's11937231	92898379	7.65E-02	N/A	-	3.61E-07	8.34E-01	8.68E-05	4582	
6	rs9375927	1.33E+08	9.95E-01	N/A	16	rs7192417	83591618	7.39E-01	ZDHHC7	-	3.61E-07	1.49E-01	2.05E-06	4583	
2	rs581720	74893122	2.30E-01	N/A	15	rs8039242	61107561	8.15E-01	TPM1	-	3.61E-07	5.39E-01	3.45E-05	4584	
3	rs485497	1.61E+08	1.03E-01	IL12A	11	rs7125644	1.22E+08	6.92E-01	CRTAM	-	3.61E-07	7.12E-01	1.08E-03	4585	
6	rs1966	31215712	9.61E-01	TCF19	6	rs7761965	31381474	9.15E-01	N/A	MHC	3.61E-07	2.03E-01	2.68E-03	4586	
6	rs1265093	31215166	9.61E-01	TCF19	6	rs7761965	31381474	9.15E-01	N/A	MHC	3.61E-07	2.60E-01	1.94E-03	4587	
6	rs9389037	1.33E+08	9.95E-01	N/A	16	rs7192417	83591618	7.39E-01	ZDHHC7	-	3.61E-07	4.71E-01	1.19E-04	4588	
6	rs1265074	31221193	9.61E-01	TCF19	6	rs7761965	31381474	9.15E-01	N/A	MHC	3.61E-07	7.07E-01	9.98E-05	4589	
5	rs1501791	81206460	6.50E-01	N/A	17	's12450654	40110303	8.29E-01	CCDC43	-	3.62E-07	3.47E-01	5.10E-04	4590	

SNP A					SNP B						Interaction P			Ranking	Cluster in top 100 interactions
CHR	SNP	Location	gle locus P va	Gene	CHR	SNP	Location	Single locus P valu	Gene	MHC region	Stage 1	Stage 2	Combined		
5	rs2116823	1.07E+08	7.00E-01	FBXL17	16	rs3760055	73048030	1.36E-02	GLG1	-	3.62E-07	8.09E-01	2.24E-05	4591	
5	rs10059539	7881404	6.42E-01	ADCY2	7	rs1023011	1.5E+08	6.10E-02	GBX1	-	3.62E-07	2.75E-01	2.47E-06	4592	
1	rs6424949	1.83E+08	9.93E-01	C1orf21	5	rs230068	13573973	4.11E-01	N/A	-	3.62E-07	2.81E-01	2.55E-06	4593	
1	rs6424949	1.83E+08	9.93E-01	C1orf21	5	rs230071	13565539	4.11E-01	N/A	-	3.62E-07	2.81E-01	2.55E-06	4594	
13	rs11620499	92173267	7.88E-01	GPC5	18	rs1087161	63228905	1.89E-01	N/A	-	3.62E-07	5.88E-01	2.56E-04	4595	
1	rs1125953	1.96E+08	9.32E-01	N/A	7	rs194825	1.04E+08	9.16E-01	ORC5L	-	3.62E-07	5.43E-01	1.28E-05	4596	
2	rs10187555	2.19E+08	7.58E-01	IL8RBP	7	rs1687252	1.08E+08	4.60E-01	N/A	-	3.62E-07	5.55E-01	1.13E-04	4597	
15	rs12442595	55831228	7.23E-01	N/A	20	rs1048575	11927195	8.28E-01	N/A	-	3.62E-07	9.13E-01	6.65E-05	4598	
8	rs11783232	13848841	8.21E-01	N/A	18	rs919093	6463820	5.11E-01	N/A	-	3.62E-07	9.73E-01	3.00E-04	4599	
6	rs3890820	1.01E+08	9.04E-01	N/A	11	rs7928739	67590119	9.00E-02	CHKA	-	3.62E-07	4.89E-01	2.20E-05	4600	
7	rs4074751	7006701	2.45E-02	N/A	13	rs9540823	33895343	4.65E-02	N/A	-	3.62E-07	7.85E-01	1.46E-05	4601	
1	rs7522419	17906086	5.76E-01	ARHGEF10L	4	rs6827740	1.2E+08	9.89E-01	SEC24D	-	3.62E-07	9.77E-01	8.34E-05	4602	
11	rs10833325	20415014	2.74E-01	PRMT3	14	rs1048330	25626745	7.37E-01	N/A	-	3.62E-07	6.90E-02	5.39E-03	4603	
2	rs7592671	1.68E+08	9.06E-01	XIRP2	19	rs889363	18622276	1.44E-01	KLHL26	-	3.62E-07	3.14E-01	3.74E-04	4604	
6	rs394754	42767957	8.05E-01	UBR2	13	rs9512551	26603697	5.38E-01	USP12	-	3.62E-07	5.14E-01	9.10E-05	4605	
6	rs394754	42767957	8.05E-01	UBR2	13	rs2484111	26630970	5.38E-01	USP12	-	3.62E-07	6.68E-01	1.60E-04	4606	
14	rs10130202	35235169	6.01E-01	RALGAPA1	19	rs36259	8232904	7.47E-01	LASS4	-	3.62E-07	9.29E-02	5.26E-07	4607	
4	rs7700133	1.86E+08	1.53E-02	N/A	14	rs2179932	30503224	9.59E-01	STRN3	-	3.62E-07	8.28E-01	2.86E-04	4608	
2	rs2300439	49048206	8.49E-02	FSHR	5	rs4145649	1.03E+08	3.12E-01	N/A	-	3.63E-07	1.34E-02	1.48E-02	4609	
5	rs37181	1.16E+08	5.66E-01	COMMD10	10	rs560083	84604824	4.67E-01	NRG3	-	3.63E-07	7.13E-03	1.51E-08	4610	
16	rs4782793	82031100	9.85E-03	CDH13	16	rs731258	82271433	4.87E-01	CDH13	-	3.63E-07	5.36E-01	1.99E-04	4611	
8	rs16939382	77948002	9.53E-01	ZFHX4	8	rs1443930	1.09E+08	8.89E-01	RSPO2	-	3.63E-07	4.76E-01	1.10E-05	4612	
2	rs6431850	7165870	3.51E-01	N/A	5	rs1007563	28422872	7.48E-01	N/A	-	3.63E-07	7.08E-02	2.60E-03	4613	
12	rs1870214	50884762	1.09E-01	LOC283404	22	rs738416	48482509	5.65E-01	N/A	-	3.63E-07	2.01E-02	1.76E-07	4614	
12	rs10743315	19360345	6.05E-01	PLEKHA5	21	rs7279115	41205602	7.41E-01	N/A	-	3.63E-07	3.05E-01	4.66E-03	4615	
6	rs9285499	1.44E+08	5.74E-02	AIG1	20	rs911168	58430478	6.24E-01	N/A	-	3.63E-07	1.44E-01	4.53E-03	4616	
1	rs4926749	48975936	8.86E-01	AGBL4	7	rs1176158	1.51E+08	7.66E-01	WDR86	-	3.63E-07	7.78E-01	8.53E-05	4617	
6	rs4320355	25570803	1.26E-01	N/A	11	rs1122551	1.02E+08	6.94E-01	N/A	MHC	3.63E-07	3.68E-01	1.95E-04	4618	
7	rs12113404	26189819	2.30E-01	NFE2L3	11	rs7950929	97023895	8.03E-01	N/A	-	3.64E-07	8.48E-01	7.81E-05	4619	
1	rs1340592	67793691	4.96E-01	N/A	12	rs716553	46927670	5.80E-01	N/A	-	3.64E-07	N/A	N/A	4620	
1	rs4655733	67798226	4.96E-01	N/A	12	rs716553	46927670	5.80E-01	N/A	-	3.64E-07	N/A	N/A	4621	
1	rs6662607	67794090	4.96E-01	N/A	12	rs716553	46927670	5.80E-01	N/A	-	3.64E-07	N/A	N/A	4622	
1	rs6693292	67795756	4.96E-01	N/A	12	rs716553	46927670	5.80E-01	N/A	-	3.64E-07	N/A	N/A	4623	
10	rs10741093	25854593	3.28E-01	GPR158	14	rs1289718	37572030	5.97E-01	N/A	-	3.64E-07	N/A	N/A	4624	
4	rs10517218	48150691	8.49E-01	N/A	16	rs7192553	81674884	9.75E-02	CDH13	-	3.64E-07	1.86E-01	1.72E-06	4625	
9	rs1332173	25490238	8.01E-01	N/A	16	rs2127065	7141423	3.49E-01	RBFOX1	-	3.64E-07	4.24E-04	1.76E-09	4626	
8	rs1467300	61163130	8.96E-01	N/A	20	rs2426472	51290219	3.51E-01	N/A	-	3.64E-07	2.40E-02	2.94E-07	4627	
3	rs6785998	61593709	7.64E-01	PTPRG	20	rs1530089	58423289	7.30E-01	N/A	-	3.64E-07	6.36E-02	1.81E-07	4628	
4	rs9993173	93564163	7.92E-01	GRID2	20	rs6044641	17058620	4.41E-01	N/A	-	3.64E-07	3.30E-01	4.47E-06	4629	
4	rs9993173	93564163	7.92E-01	GRID2	20	rs6135981	17059709	4.41E-01	N/A	-	3.64E-07	3.30E-01	4.47E-06	4630	
3	rs7626487	1.63E+08	6.10E-01	N/A	7	rs1022938	43210547	3.33E-01	HECW1	-	3.64E-07	9.46E-02	4.26E-06	4631	
13	rs982790	90029774	3.82E-01	N/A	13	rs816960	1.07E+08	2.38E-01	FAM155A	-	3.64E-07	4.05E-03	2.78E-08	4632	
7	rs10280848	67742124	8.09E-01	N/A	10	rs7923691	26281462	2.98E-01	MYO3A	-	3.64E-07	2.54E-01	7.17E-04	4633	
3	rs1284726	1.04E+08	2.34E-01	N/A	13	rs9534981	47653682	6.92E-01	N/A	-	3.65E-07	2.05E-01	1.38E-06	4634	
3	rs1284726	1.04E+08	2.34E-01	N/A	13	rs7998964	47686991	6.92E-01	ITM2B	-	3.65E-07	2.27E-01	1.59E-06	4635	
3	rs1284726	1.04E+08	2.34E-01	N/A	13	rs9534995	47684077	6.92E-01	N/A	-	3.65E-07	2.27E-01	1.59E-06	4636	
13	rs1323949	34329868	4.15E-01	N/A	14	rs1048352	41375682	9.86E-01	LRFN5	-	3.65E-07	4.39E-01	1.37E-04	4637	
15	rs11071185	53482122	5.92E-01	CCPG1	20	rs720489	11897114	4.17E-01	N/A	-	3.65E-07	5.70E-01	5.29E-04	4638	
15	rs8037097	46891401	1.26E-01	SHC4	18	rs2947325	693442	7.20E-01	YES1	-	3.65E-07	5.71E-01	6.32E-04	4639	
2	rs2861680	79006430	1.64E-01	N/A	11	rs1122871	56187792	3.86E-01	OR8U8	-	3.65E-07	6.54E-01	1.46E-04	4640	
5	rs17577590	1.78E+08	8.68E-01	AGXT2L2	10	rs1119765	1.18E+08	9.76E-01	C10orf96	-	3.65E-07	8.72E-01	8.74E-05	4641	
2	rs883423	1.61E+08	5.32E-01	N/A	3	rs1112957	34040753	7.33E-01	N/A	-	3.65E-07	9.80E-01	3.10E-04	4642	
7	rs2188324	88162442	3.41E-01	N/A	13	rs7997640	29642080	5.24E-01	N/A	-	3.65E-07	1.45E-01	5.25E-07	4643	
6	rs6923877	1.61E+08	5.49E-01	LPA	7	rs7798471	6711482	4.88E-01	ZNF12	-	3.65E-07	1.51E-01	1.05E-06	4644	
1	rs2026598	82722231	1.00E-01	N/A	3	rs1685420	1.71E+08	7.05E-01	MECOM	-	3.65E-07	2.89E-01	1.35E-03	4645	
6	rs2328537	1.44E+08	7.35E-01	PLAGL1	8	rs1095492	33649641	2.34E-02	N/A	-	3.65E-07	4.19E-02	1.37E-07	4646	
4	rs2165387	80710844	6.07E-02	N/A	4	rs1705642	1.72E+08	7.86E-01	N/A	-	3.65E-07	6.76E-01	2.13E-04	4647	
2	rs1108890	2.37E+08	2.96E-01	IQCA1	9	rs1081870	1.24E+08	2.70E-01	OR1J1	-	3.65E-07	9.20E-01	8.16E-05	4648	
2	rs7586860	1.85E+08	2.23E-01	ZNF804A	8	rs722311	18541896	2.56E-01	PSD3	-	3.66E-07	5.59E-01	6.32E-05	4649	
1	rs16837337	35655246	8.61E-01	ZMYM4	17	rs2091763	34483768	5.34E-01	PLXDC1	-	3.66E-07	6.91E-01	1.15E-05	4650	
1	rs16837337	35655246	8.61E-01	ZMYM4	17	rs3744077	34488719	5.34E-01	PLXDC1	-	3.66E-07	6.91E-01	1.15E-05	4651	
2	rs1477032	2.03E+08	4.85E-02	N/A	17	rs3744763	33164998	3.48E-01	HNF1B	-	3.66E-07	8.53E-01	5.32E-05	4652	
10	rs10821624	61407293	3.85E-02	C10orf40	10	rs2817694	98856512	3.60E-01	SLIT1	-	3.66E-07	4.56E-02	8.19E-03	4653	
10	rs3862863	61411255	3.85E-02	N/A	10	rs2817694	98856512	3.60E-01	SLIT1	-	3.66E-07	4.56E-02	8.19E-03	4654	
3	rs10513754	1.79E+08	1.99E-01	N/A	21	rs1108824	33506414	9.85E-01	IFN/AR2	-	3.66E-07	6.93E-01	1.55E-05	4655	
11	rs4937554	1.3E+08	6.14E-01	N/A	17	rs1695602	682800	6.15E-01	NXN	-	3.66E-07	3.11E-01	1.50E-05	4656	
2	rs3087386	99421938	9.51E-01	REV1	5	rs3776081	1.5E+08	6.89E-01	CDX1	-	3.66E-07	N/A	N/A	4657	
1	rs10494417	1.63E+08	2.35E-01	N/A	12	rs1078449	38983701	7.50E-01	LRRK2	-	3.66E-07	6.89E-01	1.86E-05	4658	
5	rs4921132	1.6E+08	8.00E-02	C1QTNF2	7	rs5012030	1.42E+08	5.59E-01	MOXD2	-	3.66E-07	8.82E-01	9.69E-04	4659	
7	rs12672847	9346003	4.11E-01	N/A	9	rs4878845	38673660	9.08E-01	N/A	-	3.66E-07	N/A	N/A	4660	
6	rs2764075	7300517	1.24E-01	N/A	21	rs641698	23324326	1.25E-01	N/A	-	3.66E-07	1.80E-01	4.04E-06	4661	
3	rs4128691	72432553	5.85E-01	N/A	11	rs523157	95574019	4.22E-01	MAML2	-	3.66E-07	5.95E-01	1.58E-05	4662	
6	rs10484284	1.3E+08	6.91E-01	N/A	9	rs7873365	6889149	7.98E-01	KDM4C	-	3.66E-07	6.87E-01	8.01E-06	4663	
4	rs869240	1.84E+08	3.74E-01	ODZ3	10	rs1100613	59826172	9.08E-01	TFAM	-	3.66E-07	9.80E-01	1.14E-04	4664	
10	rs1149952	9256962	4.81E-01	N/A	12	rs2695148	1.2E+08	3.23E-01	AN/APC5	-	3.67E-07	1.03E-01	5.39E-07	4665	
11	rs3934007	1.13E+08	5.65E-02	TMPRSS5	11	rs1182747	1.2E+08	3.90E-01	POU2F3	-	3.67E-07	7.03E-01	2.40E-04	4666	
2	rs2254958	37229795	7.81E-01	EIF2AK2	13	rs4907727	1.12E+08	1.48E-03	C13orf35	-	3.67E-07	N/A	N/A	4667	
2	rs2916490	80045863	6.83E-01	CTNN/A2	7	rs1267128	1.37E+08	8.17E-01	PTN	-	3.67E-07	7.82E-02	5.15E-03	4668	
2	rs13407916	1.09E+08	2.11E-01	SH3RF3	22	rs84458	35855154	2.56E-02	IL2RB	-	3.67E-07	7.95E-01	1.53E-04	4669	
9	rs3812537	70510552	2.37E-01	PIP5K1B	17	rs4789966	74747156	2.61E-01	HRNBP3	-	3.67E-07	6.63E-01	9.32E-06	4670	
9	rs1155556	1.19E+08	8.36E-01	ASTN2	17	rs657672	34572591	2.17E-03	PLXDC1	-	3.67E-07	2.32E-01	5.18E-04	4671	
9	rs1155556	1.19E+08	8.36E-01	ASTN2	17	rs657723	34572629	2.17E-03	PLXDC1	-	3.67E-07	2.32E-01	5.18E-04	4672	
7	rs40848	1.08E+08	7.89E-01	PNPLA8	8	rs1822958	1.16E+08	4.80E-01	N/A	-	3.67E-07	4.73E-01	1.98E-04	4673	
2	rs10865313	59970801	6.92E-01	N/A	20	rs1884885	31341559	7.95E-01	C20orf114	-	3.67E-07	8.12E-02	2.20E-07	4674	
2	rs7575977	1.06E+08	2.59E-01	N/A	8	rs2458285	1.04E+08	1.60E-01	N/A	-	3.67E-07	8.54E-01	3.01E-05	4675	
2	rs1580834	2.34E+08	9.57E-01	N/A	7	rs1717179	40924194	9.86E-01	N/A	-	3.67E-07	2.77E-01	6.63E-04	4676	
6	rs4129277	1.68E+08	3.07E-01	N/A	9	rs7846927	6831480	4.72E-01	KDM4C	-	3.67E-07	9.12E-01	3.79E-05	4677	
6	rs2523864	31126525	5.29E-01	HCG22	6	rs9368675	31380136	7.91E-01	N/A	MHC	3.68E-07	4.31E-01	6.41E-05	4678	
6	rs3890820	1.01E+08	9.04E-01	N/A	11	rs1079195	67624124	6.97E-02	CHKA	-	3.68E-07	2.02E-01	1.72E-03	4679	
6	rs6905786	99662477	7.59E-01	N/A	17	rs4362439	5945363	1.34E-01	WSCD1	-	3.68E-07	2.02E-01	1.72E-03	4680	
6	rs6902772	99657185	7.59E-01	N/A	17	rs4362439	5945363	1.34E-01	WSCD1	-	3.68E-07	2.76E-01	1.26E-03	4681	
4	rs2313565	1.62E+08	9.47E-01	N/A	9	rs7048074	13970085	5.75E-01	N/A	-	3.68E-07	4.89E-01	2.34E-05	4682	
2	rs4410257	1.76E+08	2.19E-01	N/A	22	rs1894707	25189086	5.21E-01	HPS4	-	3.68E-07	5.10E-01	4.50E-04	4683	
11	rs12795300	87118134	1.74E-01	N/A	14	rs1288256	40546007	7.05E-01	N/A	-	3.68E-07	5.12E-01	1.64E-05	4684	
3	rs12488085	55809911	3.00E-01	ERC2	19	rs1860565	62026834	7.53E-01	PEG3	-	3.68E-07	6.82E-01	8.25E-06	4685	
3	rs11709966	84602817	2.33E-01	N/A	12	rs1281027	63788725	4.93E-01	WIF1	-	3.68E-07	1.08E-01	1.23E-06	4686	
2	rs6729152	2.28E+08	3.45E-01	COL4A3	3	rs1546007	1.12E+08	2.86E-03	N/A	-	3.68E-07	2.81E-01	1.73E-03	4687	
12	rs2238079	2343619	3.69E-01	CACN/A1C	20	rs4811033	48392648	4.65E-01	N/A	-	3.68E-07	4.13E-01	5.91E-06	4688	
1	rs2117905	76825184	3.98E-01	ST6GALN/AC3	1	rs2815211	2.16E+08	4.92E-01	N/A	-	3.68E-07	5.41E-01	3.29E-04	4689	
6	rs6929928	99658969	7.59E-01	N/A	17	rs4362439	5945363	1.34E-01	WSCD1	-	3.68E-07	8.47E-01	2.50E-04	4690	
8	rs1351424	1.36E+08	8.70E-01	N/A	13	rs1256068	68455672	4.35E-01	N/A	-	3.68E-07	2.45E-02	1.57E-07	4691	
5	rs6595497	1.23E+08	2.73E-01	N/A	17	rs4789890	74855677	6.31E-01	HRNBP3	-	3.68E-07	5.10E-01	4.36E-06	4692	
3	rs1079472	45115270	5.92E-01	CDCP1	11	rs4354680	43345907	6.42E-01	TTC17	-	3.68E-07	5.59E-01	4.57E-05	4693	
3	rs1079472	45115270	5.92E-01	CDCP1	11	rs2004933	43432394	6.42E-01	TTC17	-	3.68E-07	6.45E-01	3.72E-05	4694	
1	rs4654545	4425464	2.77E-01	N/A	4	rs1194070	1.3E+08	6.92E-01	N/A	-	3.68E-07	5.74E-02	6.25E-03	4695	
11	rs1378357	11758937	8.32E-02	N/A	12	rs1281040	68731249	7.40E-01	N/A	-	3.68E-07	7.84E-01	9.31E-05	4696	
6	rs4895646	1.45E+08	6.08E-02	UTRN	13	rs2324908	65741222	9.23E-01	N/A	-	3.68E-07	8.83E-01	6.99E-05	4697	
3	rs1533939	1.44E+08	4.89E-01	PLS1	15	rs8026665	85201876	9.40E-01	AGBL1	-	3.68E-07	5.84E-01	1.75E-04	4698	
4	rs10001689	1.65E+08	4.99E-01	1-Mar	18	rs535452	58840553	6.46E-02	N/A	-	3.68E-07	7.63E-01	6.23E-05	4699	
2	rs7565158	2.13E+08	9.45E-01	N/A	7	rs6463994	10360633	3.57E-02	N/A	-	3.68E-07	8.54E-01	4.62E-05	4700	
6	rs1342740	16132379	4.44E-01	N/A	2	rs605980	67537207	3.77E-01	N/A	-	3.68E-07	N/A	N/A	4701	
8	rs6987909	26036647	9.71E-01	N/A	15	rs1124730	99842741	8.96E-01	PCSK6	-	3.68E-07	N/A	N/A	4702	

SNP A					SNP B						Interaction P			Ranking	Cluster in top 100 interactions
CHR	SNP	Location	gle locus P va	Gene	CHR	SNP	Location	ingle locus P valu	Gene	HC region	Stage 1	Stage 2	Combined		
1	rs10924366	2.44E+08	2.27E-01	SMYD3	5	s12188529	16582754	6.47E-01	FAM134B	-	3.69E-07	3.74E-01	3.39E-05	4703	
10	rs2163673	90477499	7.09E-01	LIPK	21	rs2837686	40828369	2.51E-01	DSCAM	-	3.69E-07	6.90E-02	1.89E-07	4704	
1	rs970741	1.68E+08	6.98E-01	F5	1	rs284113	1.87E+08	1.46E-01	N/A	-	3.69E-07	8.22E-01	2.35E-05	4705	
7	rs6978212	8499369	6.00E-01	NXPH1	18	s11564338	23972174	2.55E-01	CDH2	-	3.69E-07	2.46E-01	1.52E-03	4706	
7	rs6978212	8499369	6.00E-01	NXPH1	18	rs8094439	23966605	2.55E-01	CDH2	-	3.69E-07	2.46E-01	1.52E-03	4707	
6	rs7769927	44409085	7.15E-01	SPATS1	8	rs510395	20854474	5.80E-01	N/A	-	3.69E-07	4.67E-01	3.75E-04	4708	
4	rs6845251	1.84E+08	1.20E-01	WWC2	15	rs6495163	73252104	3.89E-01	N/A	-	3.69E-07	7.77E-01	1.80E-04	4709	
2	rs2600660	61403104	3.92E-01	USP34	8	rs2726555	59947966	8.90E-01	TOX	-	3.69E-07	3.62E-01	4.64E-04	4710	
3	rs2033857	1.66E+08	5.05E-01	N/A	8	s10107021	1.15E+08	7.82E-01	N/A	-	3.69E-07	9.71E-01	2.84E-05	4711	
5	rs1158196	58017591	5.70E-01	RAB3C	14	s11621843	92185877	4.54E-01	RIN3	-	3.69E-07	4.54E-01	1.36E-05	4712	
5	rs889268	37917929	2.47E-01	N/A	12	rs4763602	11020700	5.42E-01	PRH1	-	3.69E-07	6.77E-01	1.99E-04	4713	
11	rs870066	78487703	3.88E-01	ODZ4	11	rs3758770	1.28E+08	2.98E-01	FLI1	-	3.69E-07	3.10E-01	3.09E-04	4714	
11	rs870066	78487703	3.88E-01	ODZ4	11	rs7951996	1.28E+08	2.98E-01	FLI1	-	3.69E-07	3.10E-01	3.09E-04	4715	
14	rs2152402	32634017	1.73E-01	NPAS3	15	rs1346037	59925203	7.83E-01	VPS13C	-	3.69E-07	3.36E-01	4.77E-04	4716	
4	rs2044844	42551396	5.93E-01	N/A	10	s12411348	1.25E+08	2.74E-01	HMX3	-	3.69E-07	3.59E-01	3.83E-06	4717	
4	rs9985652	42553210	5.93E-01	N/A	10	s12411348	1.25E+08	2.74E-01	HMX3	-	3.69E-07	3.59E-01	3.83E-06	4718	
5	rs4921132	1.6E+08	8.00E-02	C1QTNF2	7	rs4329193	1.42E+08	5.55E-01	MOXD2	-	3.69E-07	9.01E-01	9.63E-04	4719	
1	rs3128678	4925039	7.99E-01	N/A	13	rs1322812	36877365	7.17E-01	N/A	-	3.69E-07	1.04E-02	2.16E-02	4720	
3	rs13088837	63434165	2.58E-01	SYNPR	10	rs1983127	70659907	6.72E-01	HKDC1	-	3.69E-07	1.77E-01	1.28E-06	4721	
1	rs1451915	1.94E+08	7.67E-01	N/A	8	rs7838977	1.4E+08	4.40E-01	N/A	-	3.69E-07	5.46E-01	3.52E-04	4722	
3	rs4857176	97356654	2.54E-01	N/A	7	s12703329	1.54E+08	2.15E-01	DPP6	-	3.69E-07	7.88E-01	2.23E-04	4723	
1	rs17019602	1.08E+08	5.64E-01	VAV3	9	s11243900	1.35E+08	5.82E-02	C9orf98	-	3.70E-07	3.10E-01	1.61E-05	4724	
8	rs7833351	95251354	6.61E-01	CDH17	17	rs2948549	22937916	2.93E-01	KSR1	-	3.70E-07	5.22E-01	1.57E-05	4725	
15	rs892775	31611822	1.40E-01	RYR3	17	rs9908987	60878953	1.38E-01	N/A	-	3.70E-07	1.71E-01	2.81E-06	4726	
6	rs1013147	57232840	9.75E-01	N/A	8	rs6991834	3115506	1.57E-01	CSMD1	-	3.70E-07	6.83E-01	2.83E-05	4727	
6	rs1013147	57232840	9.75E-01	N/A	8	rs7010127	3115425	1.57E-01	CSMD1	-	3.70E-07	6.83E-01	2.83E-05	4728	
1	rs2035015	1.08E+08	4.76E-01	VAV3	4	rs6850861	1.78E+08	8.83E-01	NEIL3	-	3.70E-07	3.34E-02	4.80E-03	4729	
4	rs6814124	1.51E+08	4.99E-01	N/A	9	rs1445206	9154473	9.39E-01	PTPRD	-	3.70E-07	9.63E-01	1.26E-04	4730	
1	rs7543486	14170601	6.45E-01	N/A	10	rs1923268	33414814	9.72E-01	N/A	-	3.70E-07	2.65E-01	2.67E-03	4731	
5	rs7708669	12454130	7.55E-01	N/A	7	rs217581	14893196	8.73E-01	N/A	-	3.70E-07	3.05E-01	7.20E-04	4732	
5	rs12654191	67529717	5.99E-01	N/A	10	rs2842149	1.28E+08	9.48E-01	N/A	-	3.70E-07	9.38E-02	1.19E-06	4733	
4	rs2583194	1.78E+08	2.23E-02	N/A	20	rs932905	48229538	2.71E-01	CEBPB	-	3.70E-07	3.25E-01	2.59E-05	4734	
2	rs4477859	1.6E+08	9.69E-01	7-Mar	18	rs7228959	7066464	6.14E-01	LAMA1	-	3.70E-07	8.67E-01	1.05E-04	4735	
5	rs1600178	51894999	9.08E-01	N/A	11	s10792712	83440566	3.08E-02	DLG2	-	3.71E-07	1.41E-01	5.79E-06	4736	
2	rs11691626	2.36E+08	2.24E-01	N/A	14	rs4399485	1.05E+08	8.53E-01	JAG2	-	3.71E-07	7.04E-01	2.42E-04	4737	
6	rs3130534	31317024	3.50E-01	N/A	6	rs1131896	31487094	3.40E-01	MICA	MHC	3.71E-07	9.18E-01	1.28E-04	4738	
7	rs4721998	21228763	7.69E-01	N/A	12	rs1035065	5666310	1.70E-01	ANO2	-	3.71E-07	8.87E-02	3.75E-03	4739	
3	rs7613069	10600996	5.88E-01	N/A	14	s10872983	21691822	1.07E-01	N/A	-	3.71E-07	6.43E-01	8.62E-04	4740	
1	rs10797006	1.57E+08	6.62E-01	CD1C	18	rs1940435	407559	6.83E-01	COLEC12	-	3.71E-07	9.03E-01	6.78E-05	4741	
1	rs6588480	53750707	4.14E-01	GLIS1	5	rs4958217	1.34E+08	7.30E-01	TCF7	-	3.71E-07	9.44E-01	8.82E-05	4742	
1	rs6588480	53750707	4.14E-01	GLIS1	5	rs30489	1.34E+08	7.30E-01	TCF7	-	3.71E-07	9.72E-01	1.19E-04	4743	
12	rs11612851	1.29E+08	3.15E-01	TMEM132D	16	rs4782474	87685048	1.76E-01	ACSF3	-	3.71E-07	2.53E-01	1.16E-06	4744	
12	rs7967165	950268	1.40E-01	N/A	17	rs9892829	11661322	5.30E-01	DN/AH9	-	3.71E-07	7.09E-02	2.25E-03	4745	
9	rs7034150	73262661	7.36E-01	N/A	16	rs231921	20569262	1.79E-02	ACSM1	-	3.71E-07	8.57E-01	1.71E-04	4746	
4	rs1458038	81383747	6.26E-01	N/A	5	s10462950	1.65E+08	2.37E-01	N/A	-	3.71E-07	4.43E-01	9.33E-04	4747	
5	rs2560412	5202621	4.31E-01	ADAMTS16	12	rs825959	89248606	9.78E-02	N/A	-	3.72E-07	1.87E-01	1.69E-03	4748	
1	rs1702002	54833136	7.12E-01	ACOT11	2	rs1521880	2.03E+08	4.46E-01	N/A	-	3.72E-07	2.64E-01	3.14E-05	4749	
6	rs6919094	10298044	4.47E-01	N/A	19	rs321907	57480101	2.91E-02	ZNF480	-	3.72E-07	9.59E-01	5.15E-05	4750	
2	rs2029771	2.38E+08	2.22E-02	LRRFIP1	14	rs4254262	1.04E+08	8.90E-01	N/A	-	3.72E-07	2.71E-01	6.69E-04	4751	
2	rs896637	2.09E+08	5.59E-01	N/A	7	rs2214244	20527828	1.05E-01	N/A	-	3.72E-07	4.05E-01	7.83E-04	4752	
14	rs894039	1.05E+08	1.30E-01	CDCA4	22	rs5749430	31136331	1.29E-01	BPIL2	-	3.72E-07	4.10E-01	2.42E-05	4753	
11	rs2298725	1.2E+08	2.28E-01	GRIK4	19	s10426038	34046581	2.40E-01	N/A	-	3.72E-07	6.50E-01	2.66E-05	4754	
1	rs169453	75337635	3.44E-01	N/A	5	rs828718	1.62E+08	5.73E-01	N/A	-	3.72E-07	9.08E-01	3.92E-04	4755	
3	rs1220472	1.52E+08	4.00E-01	N/A	8	s11994520	1.26E+08	5.93E-01	MTSS1	-	3.72E-07	2.10E-01	1.21E-06	4756	
3	rs1220472	1.52E+08	4.00E-01	N/A	8	s11995300	1.26E+08	5.93E-01	MTSS1	-	3.72E-07	2.10E-01	1.21E-06	4757	
3	rs1220472	1.52E+08	4.00E-01	N/A	8	rs4870911	1.26E+08	5.93E-01	MTSS1	-	3.72E-07	2.10E-01	1.21E-06	4758	
3	rs1387089	1915922	6.17E-01	N/A	10	rs242965	1.19E+08	2.93E-02	EMX2OS	-	3.72E-07	9.49E-01	1.53E-04	4759	
3	rs4244713	1.01E+08	5.69E-01	FILIP1L	9	rs4877434	90080934	9.39E-01	N/A	-	3.72E-07	2.51E-01	4.52E-03	4760	
6	rs10498874	71513774	6.64E-02	SMAP1	17	rs766304	64102446	9.81E-01	FAM20A	-	3.72E-07	6.19E-01	2.20E-05	4761	
11	rs1790158	74858897	1.10E-01	GDPD5	16	rs7191962	79763243	7.80E-01	PKD1L2	-	3.72E-07	7.98E-01	1.54E-04	4762	
2	rs7579812	1.17E+08	7.65E-01	N/A	15	rs9920389	93832564	9.64E-01	LOC145820	-	3.72E-07	7.99E-01	3.35E-04	4763	
1	rs1416531	2.15E+08	4.59E-02	ESRRG	2	rs6737361	1.41E+08	6.40E-01	LRP1B	-	3.72E-07	8.10E-01	2.86E-04	4764	
1	rs1781030	2.06E+08	4.10E-02	PLXN/A2	5	rs4631227	1.33E+08	5.10E-02	N/A	-	3.72E-07	9.64E-01	1.36E-04	4765	
6	rs6935416	5879999	9.34E-01	N/A	2	rs4668879	15221205	8.99E-01	NBAS	-	3.72E-07	3.76E-01	1.53E-03	4766	
2	rs13407916	1.09E+08	2.11E-01	SH3RF3	22	rs228942	35854565	1.59E-02	IL2RB	-	3.72E-07	8.69E-01	1.44E-04	4767	
2	rs1595066	2.12E+08	3.76E-01	ERBB4	3	rs4677287	73598809	1.07E-01	PDZRN3	-	3.72E-07	1.82E-01	9.48E-07	4768	
2	rs10210863	76802609	1.17E-01	N/A	12	rs2468343	1.04E+08	3.60E-01	SLC41A2	-	3.72E-07	1.89E-01	2.17E-06	4769	
2	rs2592813	1.7E+08	6.04E-01	PPIG	7	rs2158546	49596217	9.09E-01	N/A	-	3.72E-07	4.45E-01	3.34E-06	4770	
1	rs600734	75457991	2.70E-01	SLC44A5	5	s10079545	1.62E+08	6.50E-01	N/A	-	3.72E-07	7.64E-01	3.69E-05	4771	
12	rs12812221	55605603	5.43E-01	SDR9C7	15	s10852213	25475258	1.32E-01	N/A	-	3.73E-07	4.03E-01	5.82E-06	4772	
3	rs1464623	1.67E+08	5.46E-01	N/A	20	s11696207	54565194	1.95E-01	N/A	-	3.73E-07	2.66E-01	1.96E-06	4773	
5	rs9885155	1.72E+08	2.52E-01	ERGIC1	19	rs3852916	16253163	4.41E-01	N/A	-	3.73E-07	8.35E-01	3.23E-05	4774	
16	rs4783883	53750132	2.02E-01	N/A	22	rs2282667	21554058	9.35E-01	N/A	-	3.73E-07	9.32E-01	2.77E-05	4775	
5	rs1600178	51894999	9.08E-01	N/A	11	rs1945805	83438516	3.17E-02	DLG2	-	3.73E-07	6.18E-01	3.22E-04	4776	
8	rs12155668	17969431	3.06E-01	ASAH1	8	rs1473482	18641042	6.14E-02	PSD3	-	3.73E-07	1.00E-01	6.30E-07	4777	
6	rs480557	1.62E+08	3.94E-01	PARK2	8	rs4740033	85925158	5.24E-01	RALYL	-	3.73E-07	2.73E-01	1.64E-06	4778	
2	rs12621974	4871441	8.40E-02	N/A	8	s10956624	1.33E+08	3.62E-01	EFR3A	-	3.73E-07	5.73E-01	6.56E-05	4779	
1	rs12079158	29739034	2.13E-01	N/A	9	s11787664	1.17E+08	1.29E-01	TNC	-	3.73E-07	1.85E-01	2.21E-06	4780	
2	rs11890872	52111013	2.63E-02	N/A	3	rs939334	1.85E+08	4.28E-01	HTR3D	-	3.73E-07	1.92E-01	1.46E-03	4781	
12	rs6539598	80814114	3.92E-01	N/A	17	rs7208838	27492679	3.23E-01	RHOT1	-	3.74E-07	4.27E-03	5.28E-09	4782	
8	rs7828331	1.31E+08	3.79E-01	N/A	12	rs7968494	64417330	8.18E-01	N/A	-	3.74E-07	9.50E-01	8.48E-05	4783	
3	rs760316	60074008	8.69E-01	FHIT	14	rs3783845	88937234	2.59E-01	FOXN3	-	3.74E-07	4.79E-01	2.99E-04	4784	
8	rs7000801	1.22E+08	5.92E-01	MRPL13	22	rs5768016	46595486	9.66E-01	N/A	-	3.74E-07	7.44E-01	2.62E-05	4785	
10	rs3127086	1.15E+08	6.41E-01	NRAP	22	rs2092351	42905737	3.98E-01	PARVB	-	3.74E-07	8.53E-01	2.58E-04	4786	
3	rs9814549	1.01E+08	6.90E-01	C3orf26	19	s11880374	4587946	4.78E-01	TNFAIP8L1	-	3.74E-07	8.03E-01	2.95E-04	4787	
3	rs7610881	77619036	9.77E-01	ROBO2	12	rs1666910	61563176	4.69E-01	PPM1H	-	3.75E-07	9.23E-01	3.49E-05	4788	
11	rs7106847	19943938	7.51E-01	N/AV2	16	rs7202731	17219600	1.34E-02	XYLT1	-	3.75E-07	7.16E-01	2.88E-04	4789	
1	rs822555	98440134	6.46E-01	N/A	20	rs6064311	53981863	1.99E-01	N/A	-	3.75E-07	2.50E-01	2.17E-03	4790	
4	rs2194941	76196847	6.40E-01	PARM1	7	rs940728	70025889	1.33E-01	N/A	-	3.75E-07	5.80E-01	9.98E-05	4791	
4	rs17082557	53835173	3.32E-02	SCFD2	10	rs7099858	1.28E+08	1.11E-01	C10orf90	-	3.75E-07	2.14E-01	1.62E-03	4792	
13	rs2481873	61805091	7.22E-01	N/A	14	s17108246	70124745	3.09E-01	MED6	-	3.75E-07	5.60E-01	3.12E-06	4793	
1	rs2504053	1.78E+08	8.61E-01	FAM163A	11	rs2288159	1.13E+08	8.82E-01	TTC12	-	3.75E-07	6.70E-01	8.46E-05	4794	
11	rs4265593	11237381	9.47E-01	GALNTL4	14	rs6573766	23921886	4.11E-01	NFATC4	-	3.75E-07	8.45E-01	3.49E-04	4795	
2	rs7582124	1.6E+08	9.56E-01	7-Mar	18	rs7228959	7066464	6.14E-01	LAMA1	-	3.75E-07	8.67E-01	9.33E-05	4796	
12	rs491987	1.28E+08	5.36E-01	TMEM132D	22	rs5754727	20387273	3.09E-01	PPIL2	-	3.75E-07	6.10E-02	6.77E-03	4797	
10	rs563654	98468439	5.67E-01	PIK3AP1	16	rs364931	61206720	2.72E-02	N/A	-	3.75E-07	2.31E-01	1.41E-06	4798	
10	rs563654	98468439	5.67E-01	PIK3AP1	16	rs642081	61205572	2.72E-02	N/A	-	3.75E-07	2.31E-01	1.41E-06	4799	
5	rs6596287	1.36E+08	9.90E-01	SMAD5OS	10	rs2251068	28801907	4.20E-01	N/A	-	3.75E-07	3.90E-01	1.20E-03	4800	
6	rs9470187	36058188	5.66E-02	SLC26A8	1	s12029576	2.17E+08	1.13E-01	TGFB2	MHC	3.75E-07	7.80E-01	7.57E-05	4801	
6	rs807894	1.65E+08	1.25E-01	N/A	12	rs2730747	1.18E+08	5.47E-03	N/A	-	3.76E-07	N/A	N/A	4802	
8	rs9298280	77492308	3.47E-01	N/A	17	s11871749	14593162	8.66E-01	N/A	-	3.76E-07	1.55E-01	3.14E-06	4803	
12	rs1551845	77838929	6.92E-01	SYT1	17	rs7219984	45051819	2.21E-01	SPOP	-	3.76E-07	5.59E-01	1.14E-06	4804	
4	rs2570096	58820719	5.52E-01	N/A	10	s12774333	77838858	3.76E-01	C10orf11	-	3.76E-07	6.65E-01	1.16E-05	4805	
4	rs12507136	1.18E+08	2.41E-01	N/A	11	rs2433434	86131063	1.20E-01	N/A	-	3.76E-07	7.54E-01	5.62E-05	4806	
12	rs12820022	95680511	5.97E-01	N/A	14	rs3126174	49412701	2.58E-01	ARF6	-	3.76E-07	5.93E-01	7.58E-04	4807	
2	rs6727349	62530410	2.60E-02	N/A	15	rs1017520	96719022	4.14E-01	N/A	-	3.76E-07	6.34E-01	4.73E-04	4808	
6	rs6913797	1.46E+08	3.89E-01	GRM1	10	rs1318662	26860966	6.27E-01	APBB1IP	-	3.76E-07	8.76E-01	2.08E-04	4809	
3	rs13065354	1.64E+08	7.43E-01	N/A	8	s10111517	70063994	9.86E-01	N/A	-	3.76E-07	7.46E-01	8.00E-05	4810	
7	rs1916986	1.14E+08	4.42E-01	N/A	19	rs299175	61005340	7.65E-01	NLRP11	-	3.76E-07	N/A	N/A	4811	
8	rs13273876	4798506	9.88E-01	CSMD1	16	s12446517	6876909	8.91E-01	A2BP1	-	3.77E-07	3.07E-01	8.09E-04	4812	
2	rs10191556	2.42E+08	2.87E-01	THAP4	4	rs3796479	76914163	5.12E-01	USO1	-	3.77E-07	2.65E-02	3.10E-07	4813	
1	rs358693	91180775	5.40E-01	ZNF644	11	s10837424	40472369	6.70E-01	N/A	-	3.77E-07	4.77E-02	2.45E-07	4814	

SNP A					SNP B						Interaction P			Ranking	Cluster in top 100 interactions
CHR	SNP	Location	gle locus P va	Gene	CHR	SNP	Location	Single locus P valu	Gene	MHC region	Stage 1	Stage 2	Combined		
4	rs4692317	25646700	5.87E-01	N/A	17	s12944694	49926502	5.69E-01	N/A	-	3.77E-07	4.49E-01	3.35E-06	4815	
5	rs6452143	33756112	3.87E-01	N/A	10	rs7098374	16185793	5.48E-01	N/A	-	3.77E-07	6.41E-01	1.03E-04	4816	
11	rs2434484	43905584	8.47E-01	ALKBH3	14	rs1187629	94732144	2.73E-01	CLMN	-	3.77E-07	7.28E-01	2.48E-05	4817	
2	rs7569378	1.3E+08	2.16E-01	N/A	13	rs4405446	58102205	3.36E-01	N/A	-	3.77E-07	N/A	N/A	4818	
6	rs1331484	990503	3.23E-01	LOC285768	21	rs2826319	20778532	2.99E-01	N/A	-	3.77E-07	6.93E-02	1.45E-06	4819	
4	rs1512129	46597190	2.44E-01	COX7B2	17	s17223566	4274255	2.14E-01	SPNS3	-	3.77E-07	5.59E-01	1.45E-04	4820	
12	rs11067735	1.15E+08	5.90E-01	N/A	20	rs6064607	56001130	6.02E-01	N/A	-	3.77E-07	9.15E-01	2.73E-05	4821	
9	rs1543687	1.31E+08	2.60E-01	N/A	18	rs4798681	8727474	2.97E-01	KIAA0802	-	3.77E-07	1.11E-01	2.31E-03	4822	
2	rs3771719	1.6E+08	9.21E-01	CD302	18	rs9948462	7066836	6.08E-01	LAMA1	-	3.77E-07	8.72E-01	1.37E-04	4823	
4	rs12641060	14868093	4.27E-02	N/A	16	s11645643	10431666	1.08E-01	ATF7IP2	-	3.77E-07	8.60E-01	5.65E-05	4824	
6	rs912879	1.58E+08	5.23E-01	SNX9	20	rs6121828	59890894	4.69E-01	CDH4	-	3.77E-07	2.58E-02	1.63E-07	4825	
6	rs7747363	1.19E+08	6.33E-01	SLC35F1	22	rs2190742	15634399	9.23E-01	XKR3	-	3.77E-07	1.94E-01	8.93E-04	4826	
6	rs9344747	88714274	5.64E-01	N/A	10	rs7101032	59341804	3.82E-01	N/A	-	3.77E-07	7.40E-01	1.72E-04	4827	
6	rs4122189	31275906	7.70E-02	HCG27	6	rs9348862	31360726	7.72E-01	HLA-C	MHC	3.77E-07	4.79E-01	1.60E-05	4828	
6	rs4122189	31275906	7.70E-02	HCG27	6	rs9357122	31368324	7.72E-01	N/A	MHC	3.77E-07	4.79E-01	1.60E-05	4829	
6	rs996247	27301074	6.38E-01	N/A	15	rs813299	29225595	8.06E-01	N/A	MHC	3.78E-07	2.83E-01	8.37E-06	4830	
11	rs952131	1.29E+08	6.41E-01	N/A	19	rs8104657	24008904	4.16E-01	N/A	-	3.78E-07	3.72E-01	1.36E-05	4831	
2	rs932559	31406406	5.28E-01	XDH	3	rs9846710	1.47E+08	6.81E-02	N/A	-	3.78E-07	7.01E-01	4.69E-05	4832	
3	rs7628262	1.35E+08	1.64E-01	BFSP2	4	rs4627935	1.42E+08	1.59E-01	RNF150	-	3.78E-07	6.68E-01	1.10E-05	4833	
10	rs1326983	19976417	3.86E-01	N/A	14	rs238375	57129139	8.18E-01	SLC35F4	-	3.78E-07	6.85E-01	1.44E-04	4834	
1	rs2257749	61676044	2.41E-01	NFIA	4	rs2531198	10229443	9.18E-01	CLNK	-	3.78E-07	9.13E-01	3.49E-05	4835	
10	rs1326983	19976417	3.86E-01	N/A	14	rs1745690	57143154	8.18E-01	SLC35F4	-	3.78E-07	1.50E-02	3.12E-03	4836	
12	rs755660	60638722	5.92E-01	FAM19A2	16	rs1015990	8282369	9.27E-01	N/A	-	3.78E-07	6.42E-01	1.35E-05	4837	
2	rs1317981	10936131	5.02E-01	N/A	21	rs81481	41693232	9.74E-01	MX2	-	3.78E-07	8.39E-01	2.59E-05	4838	
3	rs4680758	28576547	9.13E-02	N/A	13	rs9550983	23021397	5.38E-01	N/A	-	3.78E-07	6.52E-01	8.13E-05	4839	
2	rs2577999	1.83E+08	4.99E-01	N/A	12	rs2645979	82541174	9.79E-01	N/A	-	3.78E-07	4.24E-01	1.48E-05	4840	
2	rs2971890	1.83E+08	4.99E-01	N/A	12	rs2645979	82541174	9.79E-01	N/A	-	3.78E-07	4.24E-01	1.48E-05	4841	
8	rs6470858	87301627	2.75E-01	SLC7A13	15	rs4319733	86020665	3.71E-01	N/A	-	3.78E-07	6.65E-01	3.84E-05	4842	
17	rs2072081	39683019	4.20E-01	SLC4A1	19	rs483808	7863481	2.21E-01	N/A	-	3.78E-07	7.61E-01	6.52E-05	4843	
2	rs7601184	1.06E+08	9.62E-01	N/A	2	rs831864	1.82E+08	8.34E-01	N/A	-	3.78E-07	2.13E-01	1.60E-06	4844	
8	rs4872441	26388640	2.98E-02	N/A	8	rs6471155	1.35E+08	1.98E-01	N/A	-	3.78E-07	5.64E-01	2.61E-05	4845	
8	rs2585470	26392094	2.98E-02	N/A	8	rs6471155	1.35E+08	1.98E-01	N/A	-	3.78E-07	N/A	N/A	4846	
4	rs7693914	1.75E+08	2.99E-01	N/A	5	rs2925071	1.09E+08	6.14E-01	N/A	-	3.78E-07	2.71E-01	4.14E-04	4847	
16	rs8059644	50288902	1.61E-01	N/A	21	rs2832409	29903116	9.83E-01	GRIK1	-	3.78E-07	3.38E-01	6.14E-04	4848	
11	rs2793476	29327278	7.22E-01	N/A	16	rs9934713	57758869	7.52E-01	N/A	-	3.79E-07	3.61E-01	2.77E-05	4849	
1	rs1268910	2.13E+08	3.18E-01	N/A	5	rs7715303	31080077	8.96E-05	N/A	-	3.79E-07	3.91E-01	1.92E-05	4850	
4	rs11929741	1.77E+08	9.08E-01	GPM6A	7	rs6954511	89933006	2.24E-01	N/A	-	3.79E-07	5.79E-01	2.13E-04	4851	
5	rs7716659	3363539	8.79E-01	N/A	20	rs2842	52700989	9.19E-01	DOK5	-	3.79E-07	7.12E-01	3.81E-05	4852	
7	rs2189903	8512593	7.70E-01	NXPH1	12	s11055306	13288136	6.45E-01	N/A	-	3.79E-07	8.83E-01	5.82E-05	4853	
1	rs4653112	35113235	4.91E-01	C1orf212	18	rs1917927	5752705	4.35E-01	N/A	-	3.79E-07	6.08E-01	7.79E-05	4854	
16	rs7500355	84634601	7.85E-01	N/A	17	rs2013347	22095317	9.42E-01	N/A	-	3.79E-07	2.40E-01	1.36E-03	4855	
3	rs7648743	66997379	1.13E-01	N/A	10	rs1113313	76683707	5.97E-01	COMTD1	-	3.79E-07	9.39E-01	3.69E-05	4856	
9	rs4324976	76158411	6.55E-01	N/A	10	s10509498	86451065	9.57E-01	N/A	-	3.79E-07	5.50E-01	3.65E-06	4857	
1	rs960472	2.36E+08	2.97E-01	RYR2	7	s11768588	1.55E+08	1.13E-01	N/A	-	3.79E-07	1.89E-02	1.16E-07	4858	
11	rs11028190	24795634	7.93E-01	LUZP2	18	s11663038	1163428	6.82E-01	N/A	-	3.79E-07	2.56E-01	1.37E-03	4859	
1	rs233104	85547774	7.93E-01	DDAH1	2	rs930655	70537959	9.35E-01	TGFA	-	3.79E-07	1.76E-01	1.81E-06	4860	
1	rs1325224	1.89E+08	7.58E-01	N/A	17	s11657083	6797819	8.22E-01	N/A	-	3.79E-07	2.91E-01	1.41E-03	4861	
6	rs969931	29602876	1.59E-01	N/A	10	rs7070343	33438257	5.29E-01	N/A	MHC	3.79E-07	1.40E-02	1.93E-02	4862	
6	rs3130453	31232828	5.90E-01	TCF19	6	rs7761965	31381474	9.15E-01	N/A	MHC	3.79E-07	1.33E-01	2.74E-05	4863	
1	rs6670655	1.63E+08	3.45E-01	PBX1	16	rs2247465	77632812	2.88E-01	WWOX	-	3.79E-07	5.62E-01	2.37E-04	4864	
8	rs10504093	51028383	6.53E-01	SNTG1	20	rs6014949	55186217	1.65E-01	BMP7	-	3.80E-07	1.98E-01	7.39E-04	4865	
14	rs1157565	26692755	5.10E-01	N/A	18	rs8093432	622651	7.38E-01	C18orf56	-	3.80E-07	6.93E-01	2.51E-05	4866	
6	rs472072	12745272	5.47E-01	N/A	12	rs7967362	1.29E+08	8.73E-01	N/A	-	3.80E-07	3.09E-01	8.62E-06	4867	
3	rs17034817	11632278	3.49E-01	VGLL4	4	s10025139	35989633	1.24E-01	DTHD1	-	3.80E-07	4.50E-01	8.17E-06	4868	
8	rs2584561	12971665	1.24E-01	DLC1	20	s10485804	54239111	7.83E-01	MC3R	-	3.80E-07	N/A	N/A	4869	
4	rs12502363	90932087	1.75E-01	SNCA	10	rs1932148	25857672	8.35E-01	GPR158	-	3.80E-07	1.54E-01	3.41E-06	4870	
4	rs3822095	90955540	1.75E-01	SNCA	10	rs1932148	25857672	8.35E-01	GPR158	-	3.80E-07	1.54E-01	3.41E-06	4871	
4	rs11100107	1.59E+08	2.74E-01	N/A	9	s12686399	29383359	4.88E-02	N/A	-	3.80E-07	5.31E-01	3.98E-04	4872	
2	rs1542179	1.01E+08	1.26E-01	NPAS2	12	s12721420	46645061	7.59E-01	TMEM106C	-	3.80E-07	2.30E-03	2.67E-02	4873	
14	rs7160583	76402161	1.39E-01	C14orf166B	17	s12601802	63750164	6.15E-01	ARSG	-	3.80E-07	1.01E-01	3.72E-03	4874	
4	rs12645956	83746281	3.50E-01	C4orf11	9	rs1358907	3158478	2.43E-01	N/A	-	3.80E-07	3.94E-01	6.01E-04	4875	
12	rs17022166	93111536	8.07E-01	PLXNC1	19	rs2396105	8477530	2.59E-01	HNRNPM	-	3.80E-07	1.25E-01	1.00E-06	4876	
2	rs2215669	2.11E+08	7.14E-01	UNC80	15	s10519198	76529809	1.35E-02	IREB2	-	3.80E-07	7.57E-01	2.31E-05	4877	
7	rs6973256	1.33E+08	5.41E-01	EXOC4	11	rs2467592	29154125	8.46E-02	N/A	-	3.80E-07	9.58E-01	1.13E-04	4878	
9	rs329376	36490619	2.82E-01	N/A	13	rs4567577	1.04E+08	8.70E-02	N/A	-	3.80E-07	N/A	N/A	4879	
6	rs736348	36859223	7.24E-01	CPNE5	5	s17651538	75588257	7.84E-01	SV2C	MHC	3.81E-07	7.74E-01	1.46E-04	4880	
1	rs11263839	36167312	1.78E-01	EIF2C3	7	s10499587	29601630	6.17E-01	N/A	-	3.81E-07	2.93E-01	7.14E-06	4881	
4	rs2165387	80710844	6.07E-02	N/A	7	rs9886302	70389420	4.37E-01	WBSCR17	-	3.81E-07	2.67E-01	3.88E-06	4882	
10	rs1570940	18725771	2.46E-01	CACNB2	13	s11069247	96313752	1.29E-01	N/A	-	3.81E-07	7.85E-01	4.40E-04	4883	
13	rs17089968	72697612	5.28E-02	N/A	20	rs4814019	11456980	9.36E-01	N/A	-	3.81E-07	9.73E-01	1.48E-05	4884	
2	rs4954353	1.33E+08	1.73E-01	NCKAP5	11	rs2512568	1.15E+08	5.17E-01	N/A	-	3.81E-07	6.06E-01	4.86E-05	4885	
1	rs1166906	2.07E+08	6.03E-01	N/A	2	rs1027928	2.39E+08	2.68E-01	N/A	-	3.81E-07	7.56E-01	6.86E-05	4886	
8	rs13261391	1144229	2.61E-01	N/A	22	rs9609560	31186999	4.06E-01	BPIL2	-	3.81E-07	2.41E-02	4.91E-07	4887	
4	rs1993656	1.51E+08	8.88E-01	DCLK2	22	rs5750423	36064398	3.60E-01	N/A	-	3.81E-07	2.26E-02	2.12E-07	4888	
15	rs2017626	22983935	1.51E-01	SNORD115-2	17	rs1828096	49668269	3.48E-01	N/A	-	3.81E-07	6.87E-01	2.96E-05	4889	
2	rs11901302	2376572	8.05E-01	N/A	5	rs2055873	1.26E+08	4.08E-01	3-Mar	-	3.81E-07	8.47E-01	1.50E-05	4890	
3	rs13085838	64625627	3.84E-02	ADAMTS9	16	rs2547033	24651158	4.73E-01	TNRC6A	-	3.81E-07	7.89E-01	1.33E-04	4891	
6	rs2508015	31118179	8.45E-01	HCG22	6	rs2233965	31188878	4.20E-03	C6orf15	MHC	3.81E-07	2.37E-02	1.65E-07	4892	
3	rs2056123	1.59E+08	9.69E-01	N/A	5	rs6450490	50334219	4.55E-01	N/A	-	3.82E-07	1.26E-01	3.60E-07	4893	
3	rs9883600	1.46E+08	9.15E-01	N/A	9	rs1332408	1.24E+08	4.21E-01	N/A	-	3.82E-07	4.81E-01	4.84E-06	4894	
10	rs3814170	15252220	3.47E-01	NMT2	21	rs2164171	26710929	6.66E-01	N/A	-	3.82E-07	1.09E-01	3.67E-03	4895	
1	rs12134393	1.59E+08	7.19E-01	N/A	3	s13067831	1.45E+08	2.76E-01	N/A	-	3.82E-07	9.29E-01	7.58E-05	4896	
1	rs3737792	1.59E+08	7.19E-01	N/A	3	s13067831	1.45E+08	2.76E-01	N/A	-	3.82E-07	9.29E-01	7.58E-05	4897	
9	rs7866059	72498917	7.83E-01	TRPM3	18	rs1625796	28369416	9.26E-01	N/A	-	3.82E-07	3.46E-01	6.49E-06	4898	
6	rs1842129	1.25E+08	1.93E-01	NKAIN2	5	rs7724739	33828213	6.65E-01	ADAMTS12	-	3.82E-07	8.08E-01	1.24E-04	4899	
4	rs2570096	58820719	5.52E-01	N/A	9	s10125617	72107987	3.56E-01	SMC5	-	3.82E-07	8.00E-01	1.71E-05	4900	
2	rs7426089	29556186	6.56E-02	ALK	8	rs7006961	1.06E+08	9.14E-01	N/A	-	3.82E-07	N/A	N/A	4901	
11	rs1522084	33762562	7.45E-01	FBXO3	14	rs2286425	73884992	3.34E-01	C14orf115	-	3.82E-07	3.97E-01	1.02E-05	4902	
5	rs7730204	90058340	7.16E-01	GPR98	17	s10491216	3267156	7.29E-01	OR1E1	-	3.83E-07	4.31E-01	9.08E-07	4903	
6	rs332496	93512596	3.60E-01	N/A	8	rs2014286	16951496	5.93E-01	EFHA2	-	3.83E-07	4.95E-01	9.65E-05	4904	
4	rs966380	98654412	2.56E-01	N/A	7	s10280700	52549994	6.13E-01	N/A	-	3.83E-07	3.69E-01	1.20E-03	4905	
5	rs2731714	1.72E+08	5.77E-01	SH3PXD2B	22	rs9607007	31769006	7.86E-01	SYN3	-	3.83E-07	6.55E-01	2.31E-04	4906	
6	rs12204812	1.25E+08	7.18E-01	NKAIN2	2	s16858290	12168764	5.66E-01	N/A	-	3.83E-07	3.22E-01	3.33E-06	4907	
1	rs2276401	1.57E+08	7.22E-01	SPTA1	21	rs2070423	31985225	5.14E-01	SFRS15	-	3.83E-07	3.36E-01	4.73E-06	4908	
1	rs2276401	1.57E+08	7.22E-01	SPTA1	21	s12626622	31981460	5.14E-01	SFRS15	-	3.83E-07	3.39E-01	4.84E-06	4909	
8	rs2341177	30124035	5.30E-01	DCTN6	9	s10760611	1.31E+08	8.02E-01	N/A	-	3.83E-07	4.08E-01	1.98E-03	4910	
5	rs10719	31437204	3.58E-01	RN/ASEN	20	rs1012891	7928390	3.29E-01	TMX4	-	3.83E-07	1.39E-01	1.19E-03	4911	
12	rs3782362	71171821	3.87E-01	TRHDE	16	rs4784779	55741226	9.21E-01	CPNE2	-	3.83E-07	7.47E-01	1.01E-04	4912	
1	rs12758773	1.89E+08	2.64E-01	N/A	3	rs2197126	19398565	5.56E-01	KCNH8	-	3.83E-07	9.68E-01	3.18E-05	4913	
6	rs887464	31253899	8.59E-02	TCF19	6	rs9357122	31368324	7.72E-01	N/A	MHC	3.84E-07	9.42E-02	6.70E-04	4914	
6	rs887464	31253899	8.59E-02	TCF19	6	rs9348862	31360726	7.72E-01	HLA-C	MHC	3.84E-07	4.78E-01	5.91E-06	4915	
1	rs7537880	2E+08	2.49E-01	N/A	13	rs1412817	65090650	9.21E-01	N/A	-	3.84E-07	7.88E-01	6.39E-05	4916	
10	rs2138690	1514715	1.64E-01	ADARB2	12	s10772982	17000624	2.83E-01	N/A	-	3.84E-07	6.74E-01	1.63E-04	4917	
11	rs1790474	1.19E+08	4.13E-01	N/A	15	rs2929525	70074910	6.08E-01	MYO9A	-	3.84E-07	9.30E-01	3.81E-05	4918	
6	rs2141150	1.53E+08	7.98E-01	SYNE1	5	rs1563551	83057582	8.70E-01	HAPLN1	-	3.84E-07	9.48E-01	1.38E-04	4919	
3	rs6419844	22391322	7.79E-01	N/A	10	rs1323072	77370241	9.75E-01	C10orf11	-	3.84E-07	3.48E-01	1.04E-05	4920	
7	rs17282271	23891070	9.24E-01	N/A	16	s12598492	58087166	3.34E-01	N/A	-	3.84E-07	5.48E-01	2.97E-05	4921	
3	rs10935384	1.42E+08	7.96E-01	CLSTN2	16	s10521115	19998146	1.63E-01	GPR139	-	3.84E-07	9.42E-01	9.26E-05	4922	
1	rs1387389	1.63E+08	3.06E-01	PBX1	8	s10888157	18449117	1.85E-01	PSD3	-	3.84E-07	4.67E-01	1.68E-05	4923	
1	rs7512480	63131404	5.99E-01	N/A	4	s13134705	31417795	6.99E-01	N/A	-	3.84E-07	4.67E-01	1.68E-05	4924	
3	rs9862823	1.81E+08	5.87E-01	USP13	12	rs7295506	71201098	6.40E-01	TRHDE	-	3.84E-07	5.72E-01	1.57E-04	4925	
5	rs10041997	1.2E+08	5.18E-01	N/A	20	rs557446	47632326	8.78E-01	PTGIS	-	3.84E-07	9.22E-01	1.53E-04	4926	

SNP A					SNP B						Interaction P			Ranking	Cluster in top 100 interactions
CHR	SNP	Location	gle locus P va	Gene	CHR	SNP	Location	ingle locus P valu	Gene	MHC region	Stage 1	Stage 2	Combined		
4	rs7673610	76679560	3.80E-02	THAP6	16	rs9652638	6438715	8.36E-01	A2BP1	-	3.84E-07	9.91E-02	1.59E-03	4927	
7	rs334523	47532029	7.13E-01	TNS3	15	rs17637182	23568636	3.70E-01	ATP10A	-	3.84E-07	1.99E-01	2.12E-03	4928	
2	rs908571	45171969	8.44E-01	N/A	21	rs2830017	26324026	4.03E-01	APP	-	3.84E-07	8.80E-01	3.61E-05	4929	
2	rs908571	45171969	8.44E-01	N/A	21	rs2830018	26324064	4.03E-01	APP	-	3.84E-07	8.80E-01	3.61E-05	4930	
1	rs4658931	2.29E+08	4.05E-01	FAM89A	9	rs7021746	93216082	5.23E-01	NFIL3	-	3.84E-07	2.25E-01	4.34E-06	4931	
6	rs13203475	1.5E+08	8.54E-01	N/A	9	rs1668978	25342327	1.10E-01	N/A	-	3.84E-07	6.77E-01	4.72E-05	4932	
12	rs9971641	86289970	2.82E-01	N/A	22	rs7287569	41483090	8.60E-01	N/A	-	3.84E-07	6.20E-01	2.06E-04	4933	
6	rs2145753	24834387	6.53E-01	C6orf62	6	rs2245704	66576477	1.36E-01	N/A	-	3.84E-07	N/A	N/A	4934	
1	rs1340985	74933312	4.80E-01	CRYZ	8	rs10103576	22620944	4.12E-01	PEBP4	-	3.84E-07	1.43E-01	1.64E-06	4935	
2	rs16986865	19752687	7.87E-01	N/A	13	rs10507508	41867782	6.05E-01	N/A	-	3.84E-07	2.30E-01	2.93E-06	4936	
3	rs3850186	1.73E+08	2.18E-01	PLD1	8	rs11787427	61391405	3.70E-01	N/A	-	3.84E-07	8.05E-01	6.22E-05	4937	
5	rs4645374	1.09E+08	1.52E-01	N/A	10	rs17454012	11377324	1.32E-01	CUGBP2	-	3.84E-07	9.61E-01	1.98E-04	4938	
6	rs226468	1760690	6.18E-01	GMDS	18	rs7243091	59031542	2.18E-01	BCL2	-	3.85E-07	9.68E-02	8.32E-07	4939	
6	rs7768952	1.23E+08	5.42E-01	CLVS2	1	rs17596572	1.5E+08	1.20E-01	HRNR	-	3.85E-07	1.09E-01	2.47E-03	4940	
5	rs4700799	1.79E+08	4.34E-01	ADAMTS2	9	rs2739257	1.4E+08	2.66E-01	CACN/A1B	-	3.85E-07	1.32E-01	5.09E-03	4941	
13	rs1446770	84006207	1.39E-01	N/A	18	rs1039610	74049795	9.22E-01	N/A	-	3.85E-07	5.98E-01	1.40E-05	4942	
13	rs7337326	84007088	1.39E-01	N/A	18	rs1039610	74049795	9.22E-01	N/A	-	3.85E-07	5.98E-01	1.40E-05	4943	
6	rs13216342	1.68E+08	3.38E-01	N/A	9	rs7846927	6831480	4.72E-01	KDM4C	-	3.85E-07	9.83E-01	2.32E-05	4944	
2	rs11893319	1.97E+08	1.77E-01	HECW2	3	rs16828799	1.75E+08	8.75E-01	NLGN1	-	3.85E-07	4.53E-01	5.62E-04	4945	
10	rs4838594	49339148	4.46E-01	ARHGAP22	19	rs4806147	40563043	6.89E-01	FFAR3	-	3.85E-07	4.62E-01	1.12E-05	4946	
2	rs1477032	2.03E+08	4.85E-02	N/A	4	rs1160185	1.21E+08	1.33E-01	N/A	-	3.85E-07	5.25E-01	1.53E-05	4947	
11	rs1060435	68612171	5.78E-02	TPCN2	17	rs1285294	75540276	1.71E-01	TBC1D16	-	3.85E-07	7.63E-01	8.73E-04	4948	
3	rs7373528	37725628	8.99E-02	ITGA9	3	rs4856085	1.05E+08	9.58E-02	N/A	-	3.85E-07	7.94E-01	7.36E-05	4949	
12	rs11059460	1.27E+08	2.00E-01	N/A	18	rs8098175	41773439	3.29E-01	KIAA1632	-	3.85E-07	5.83E-01	2.93E-04	4950	
13	rs1446770	84006207	1.39E-01	N/A	18	rs9951013	74048791	9.24E-01	N/A	-	3.85E-07	5.93E-01	1.38E-05	4951	
13	rs7337326	84007088	1.39E-01	N/A	18	rs9951013	74048791	9.24E-01	N/A	-	3.85E-07	5.93E-01	1.38E-05	4952	
11	rs10741762	19179719	7.95E-01	CSRP3	16	rs4780486	9532033	1.05E-03	N/A	-	3.85E-07	7.80E-01	8.52E-05	4953	
2	rs2540289	1.03E+08	6.72E-01	TMEM182	8	rs10100405	88960987	6.43E-01	DCAF4L2	-	3.85E-07	9.36E-01	7.83E-05	4954	
1	rs4339862	1.58E+08	6.96E-01	N/A	3	rs12494196	85796570	2.19E-01	CADM2	-	3.85E-07	2.04E-01	1.28E-05	4955	
6	rs2764075	7300517	1.24E-01	N/A	21	rs198070	23341733	3.67E-01	N/A	-	3.85E-07	4.69E-01	1.35E-05	4956	
6	rs4896802	1.46E+08	5.40E-02	N/A	7	rs6967748	45105688	1.27E-01	N/ACAD	-	3.85E-07	8.58E-01	6.39E-05	4957	
5	rs2526162	1.35E+08	4.86E-01	LECT2	15	rs12148733	70622069	9.56E-01	ARIH1	-	3.85E-07	8.86E-02	3.64E-07	4958	
2	rs17538082	2.09E+08	1.91E-01	N/A	7	rs916790	20539225	9.56E-02	N/A	-	3.85E-07	3.44E-01	5.16E-04	4959	
14	rs1885147	28063661	9.81E-01	N/A	15	rs8043356	58803461	7.65E-01	RORA	-	3.85E-07	8.51E-01	4.45E-04	4960	
8	rs7839217	59433595	4.65E-01	N/A	19	rs2116941	10195443	4.56E-01	S1PR2	-	3.85E-07	9.71E-01	2.86E-05	4961	
13	rs17233815	92156875	7.20E-01	GPC5	18	rs17077389	63326692	2.58E-02	N/A	-	3.85E-07	9.44E-01	1.69E-04	4962	
13	rs927694	94312964	4.27E-01	N/A	15	rs4620904	57817305	6.68E-01	N/A	-	3.86E-07	2.30E-01	3.05E-06	4963	
9	rs10969037	29248978	2.53E-01	N/A	10	rs7901487	53459394	4.79E-01	PRKG1	-	3.86E-07	6.54E-01	2.82E-05	4964	
5	rs950392	1.23E+08	3.41E-01	N/A	17	rs4789890	74855677	6.31E-01	HRNBP3	-	3.86E-07	6.62E-01	1.22E-05	4965	
1	rs1512579	1.18E+08	9.71E-01	N/A	20	rs6097587	51855423	3.44E-01	N/A	-	3.86E-07	8.55E-01	7.89E-05	4966	
2	rs3087386	99421938	9.51E-01	REV1	5	rs2304060	1.49E+08	9.70E-01	CSF1R	-	3.86E-07	N/A	N/A	4967	
13	rs4773200	1.1E+08	5.87E-01	COL4A2	14	rs12878670	41021898	9.18E-01	N/A	-	3.86E-07	5.24E-01	2.21E-04	4968	
12	rs1008012	33135446	3.63E-01	N/A	21	rs2823739	16583289	8.44E-01	C21orf34	-	3.86E-07	7.57E-01	3.45E-05	4969	
2	rs7564632	1.17E+08	6.76E-01	N/A	15	rs9920389	93832564	9.64E-01	LOC145820	-	3.86E-07	9.79E-01	4.78E-05	4970	
1	rs471202	30832985	2.44E-01	N/A	15	rs2304546	46890536	1.83E-01	SHC4	-	3.86E-07	1.22E-01	5.66E-07	4971	
5	rs2569208	1.72E+08	5.28E-01	SH3PXD2B	22	rs9609684	31759708	2.52E-01	SYN3	-	3.86E-07	2.73E-01	1.46E-03	4972	
10	rs17157052	1853551	8.36E-01	N/A	17	rs11650437	6313993	1.94E-01	PITPNM3	-	3.86E-07	5.58E-01	3.54E-05	4973	
7	rs6952869	1.33E+08	5.14E-01	EXOC4	18	rs341237	6391283	7.19E-01	L3MBTL4	-	3.86E-07	7.62E-02	2.99E-07	4974	
1	rs10926614	2.4E+08	5.44E-01	N/A	5	rs985890	1.72E+08	9.19E-01	NEURL1B	-	3.86E-07	6.33E-01	2.58E-05	4975	
4	rs4975057	80707369	7.87E-02	N/A	4	rs17056428	1.72E+08	7.86E-01	N/A	-	3.86E-07	6.76E-01	2.17E-04	4976	
1	rs7517484	42023043	9.35E-01	HIVEP3	15	rs8025614	39930204	3.55E-01	PLA2G4B	-	3.86E-07	6.94E-01	2.03E-04	4977	
4	rs4916612	3719953	2.64E-01	ADRA2C	16	rs2526063	72599222	3.84E-01	N/A	-	3.86E-07	4.66E-01	4.05E-04	4978	
6	rs3094205	31199841	5.91E-01	C6orf15	14	rs8014790	97513859	2.36E-01	C14orf64	MHC	3.87E-07	6.06E-01	1.92E-05	4979	
3	rs7613069	10600996	5.88E-01	N/A	14	rs12434307	21771960	9.12E-02	N/A	-	3.87E-07	6.13E-01	9.97E-05	4980	
3	rs2378570	1.92E+08	4.64E-01	TMEM207	11	rs7950594	20243150	4.54E-01	N/A	-	3.87E-07	7.49E-01	7.62E-04	4981	
3	rs301564	11778927	9.11E-01	N/A	4	rs2634073	1.12E+08	3.49E-01	N/A	-	3.87E-07	8.15E-01	6.13E-05	4982	
6	rs2328779	23568885	6.45E-01	N/A	3	rs282861	1.8E+08	7.29E-01	N/A	-	3.87E-07	8.46E-01	1.44E-04	4983	
5	rs2406422	1.21E+08	5.37E-01	N/A	18	rs10775437	6833259	9.39E-01	ARHGAP28	-	3.87E-07	1.40E-01	8.12E-07	4984	
6	rs4895646	1.45E+08	6.08E-02	UTRN	13	rs1200530	65765795	9.60E-01	PCDH9	-	3.87E-07	8.73E-01	1.38E-05	4985	
5	rs2652217	77016000	2.28E-01	TBCA	5	rs791645	1.73E+08	2.26E-01	N/A	-	3.87E-07	3.56E-02	1.78E-07	4986	
1	rs6701300	8123277	1.37E-01	N/A	10	rs11248405	1.25E+08	2.12E-01	HMX3	-	3.87E-07	6.32E-02	7.46E-03	4987	
8	rs1030080	5181456	1.42E-01	N/A	12	rs10773579	1.28E+08	5.78E-01	SLC15A4	-	3.87E-07	5.22E-01	6.53E-04	4988	
8	rs3864665	62093567	7.26E-01	N/A	22	rs2284063	36874244	2.55E-01	PLA2G6	-	3.87E-07	6.09E-01	1.67E-05	4989	
9	rs10960384	11924943	4.20E-01	N/A	22	rs5992838	16644831	1.46E-01	BID	-	3.87E-07	4.47E-02	3.41E-07	4990	
3	rs17032876	10445911	8.68E-01	ATP2B2	9	rs2274821	4712462	1.56E-01	AK3	-	3.87E-07	4.21E-01	4.48E-04	4991	
3	rs4522753	2041897	9.14E-01	N/A	15	rs2715792	95089815	5.24E-01	N/A	-	3.87E-07	N/A	N/A	4992	
8	rs298191	65434415	4.58E-01	N/A	16	rs16944688	84712349	8.18E-02	N/A	-	3.87E-07	7.13E-01	3.84E-04	4993	
3	rs3111310	23484601	5.09E-01	UBE2E2	19	rs12610520	10210118	9.39E-02	S1PR2	-	3.87E-07	2.23E-01	1.94E-06	4994	
3	rs9877410	32406070	9.42E-01	CMTM8	12	rs2250441	1.05E+08	7.29E-01	TCP11L2	-	3.87E-07	3.82E-01	3.85E-04	4995	
4	rs366831	1.67E+08	5.15E-01	N/A	16	rs11117407	84411812	7.47E-01	COX4I1	-	3.87E-07	N/A	N/A	4996	
4	rs892838	1.85E+08	7.41E-01	N/A	13	rs979244	25988036	5.10E-01	N/A	-	3.88E-07	9.78E-01	6.60E-05	4997	
8	rs10503296	4803438	6.89E-01	CSMD1	11	rs12797802	1.32E+08	3.01E-01	NTM	-	3.88E-07	6.75E-01	8.91E-05	4998	
1	rs4339862	1.58E+08	6.96E-01	N/A	3	rs11127905	85793026	2.44E-01	CADM2	-	3.88E-07	2.04E-01	1.48E-05	4999	
1	rs4339862	1.58E+08	6.96E-01	N/A	3	rs9860249	85770242	2.44E-01	CADM2	-	3.88E-07	2.04E-01	1.48E-05	5000	
3	rs7630566	1.68E+08	2.22E-01	N/A	8	rs6473497	84947068	4.09E-01	N/A	-	3.88E-07	2.90E-01	3.22E-04	5001	
1	rs3026913	21417188	9.30E-01	ECE1	1	rs2359934	2.12E+08	7.34E-01	N/A	-	3.88E-07	2.90E-01	2.17E-06	5002	
1	rs17115070	96628806	8.62E-01	N/A	5	rs10471333	67291220	9.55E-01	N/A	-	3.88E-07	3.20E-01	9.96E-04	5003	
1	rs7549180	2.43E+08	5.68E-01	KIF26B	18	rs1615035	19432843	1.66E-01	ANKRD29	-	3.88E-07	6.59E-01	1.22E-04	5004	
1	rs10900497	2.04E+08	8.79E-01	CDK18	12	rs2254827	1.03E+08	8.61E-01	N/A	-	3.88E-07	8.10E-01	1.55E-04	5005	
9	rs10491725	1.13E+08	8.57E-01	GNG10	16	rs2601828	4043872	9.45E-02	ADCY9	-	3.88E-07	4.95E-01	6.44E-04	5006	
7	rs7786289	1.12E+08	6.93E-01	DOCK4	11	rs7127610	84306633	5.16E-01	DLG2	-	3.88E-07	1.10E-01	2.71E-03	5007	
5	rs6887279	1.04E+08	5.70E-02	N/A	18	rs4797325	879905	2.66E-01	ADCYAP1	-	3.88E-07	7.89E-01	8.42E-06	5008	
5	rs2463506	9410942	4.19E-01	SEMA5A	16	rs4843975	85109283	8.14E-01	N/A	-	3.89E-07	2.88E-01	7.11E-06	5009	
6	rs9343974	80981364	1.68E-01	BCKDHB	4	rs10020692	1.68E+08	8.89E-01	SPOCK3	-	3.89E-07	6.60E-01	4.72E-05	5010	
1	rs16866144	30632446	9.45E-01	N/A	18	rs13381162	55096022	9.11E-01	RAX	-	3.89E-07	1.88E-01	2.54E-03	5011	
6	rs1452531	79148679	6.80E-02	N/A	1	rs232812	58851836	9.12E-01	N/A	-	3.89E-07	3.48E-01	2.33E-04	5012	
13	rs9594979	43069495	4.20E-01	ENOX1	14	rs4900550	1.02E+08	2.80E-01	RCOR1	-	3.89E-07	5.71E-01	6.89E-06	5013	
13	rs1329285	43068982	4.20E-01	ENOX1	14	rs4900550	1.02E+08	2.80E-01	RCOR1	-	3.89E-07	6.28E-01	8.66E-06	5014	
4	rs4695945	1.76E+08	8.61E-01	GLRA3	7	rs6466333	78127906	5.24E-01	MAGI2	-	3.89E-07	9.91E-01	8.44E-05	5015	
5	rs3733989	1.68E+08	2.01E-01	ODZ2	18	rs2612377	71771223	4.47E-01	N/A	-	3.89E-07	9.62E-02	7.26E-07	5016	
5	rs3733989	1.68E+08	2.01E-01	ODZ2	18	rs2732239	71762651	4.47E-01	N/A	-	3.89E-07	9.62E-02	7.26E-07	5017	
5	rs3733989	1.68E+08	2.01E-01	ODZ2	18	rs2926622	71764657	4.47E-01	N/A	-	3.89E-07	9.62E-02	7.26E-07	5018	
2	rs10168340	5641015	7.86E-01	N/A	10	rs12261821	79847770	1.17E-01	N/A	-	3.89E-07	6.94E-01	8.03E-05	5019	
1	rs10797006	1.57E+08	6.62E-01	CD1C	18	rs3932728	388147	5.84E-01	COLEC12	-	3.89E-07	8.61E-01	8.61E-05	5020	
2	rs17031608	66216670	6.44E-01	N/A	15	rs4128671	95771967	8.82E-01	N/A	-	3.89E-07	4.18E-01	5.81E-04	5021	
19	rs1005204	50342528	2.90E-01	NKPD1	20	rs1361051	53671644	6.08E-01	N/A	-	3.89E-07	5.10E-01	2.59E-04	5022	
6	rs9378518	806084	4.97E-01	N/A	1	rs11207426	59458507	2.71E-01	N/A	-	3.89E-07	9.71E-01	5.08E-05	5023	
4	rs10022014	1.19E+08	1.76E-01	N/A	20	rs6123551	54195825	1.06E-01	N/A	-	3.89E-07	1.06E-01	8.29E-04	5024	
10	rs7914952	14209168	8.19E-01	FRMD4A	14	rs1126133	61045031	1.28E-01	PRKCH	-	3.89E-07	8.43E-01	1.61E-04	5025	
1	rs10918897	1.67E+08	9.85E-01	N/A	17	rs7220422	5579942	9.21E-01	N/A	-	3.89E-07	N/A	N/A	5026	
3	rs2251423	62514776	5.94E-01	CADPS	9	rs12553755	1.16E+08	3.64E-01	COL27A1	-	3.90E-07	9.01E-01	7.11E-05	5027	
3	rs6792978	97025511	2.64E-01	N/A	12	rs1881959	60854638	2.72E-01	FAM19A2	-	3.90E-07	9.99E-02	2.71E-03	5028	
3	rs934438	97032728	2.64E-01	N/A	12	rs1881959	60854638	2.72E-01	FAM19A2	-	3.90E-07	9.99E-02	2.71E-03	5029	
2	rs7582990	10450320	8.69E-01	HPCAL1	14	rs883219	53362407	9.36E-01	N/A	-	3.90E-07	5.38E-01	1.91E-04	5030	
2	rs2305516	39052470	9.59E-01	LOC100271715	19	rs9636161	61592298	4.39E-01	ZNF542	-	3.90E-07	9.52E-01	1.32E-04	5031	
13	rs4773539	89356301	5.20E-01	N/A	14	rs2998303	84071041	1.50E-01	N/A	-	3.90E-07	5.52E-02	3.31E-07	5032	
6	rs4959998	4436492	5.90E-02	N/A	16	rs756813	23198310	4.85E-01	N/A	-	3.90E-07	7.15E-02	5.28E-07	5033	
11	rs930722	87740697	6.73E-01	N/A	17	rs2541244	50455167	7.92E-01	STXBP4	-	3.90E-07	3.13E-01	2.24E-04	5034	
11	rs930722	87740697	6.73E-01	N/A	17	rs1026961	50510665	7.92E-01	STXBP4	-	3.90E-07	3.24E-01	2.11E-04	5035	
6	rs11751484	1.28E+08	4.60E-01	N/A	14	rs10143413	21596721	1.12E-01	N/A	-	3.90E-07	5.16E-01	3.28E-04	5036	
6	rs11751484	1.28E+08	4.60E-01	N/A	14	rs2072614	21590891	1.12E-01	N/A	-	3.90E-07	4.14E-01	8.33E-06	5037	
6	rs1766208	1.07E+08	2.34E-01	ATG5	1	rs6687497	2.31E+08	6.40E-01	PCNXL2	-	3.90E-07	4.74E-01	1.09E-05	5038	

SNP A					SNP B						Interaction P			Ranking	Cluster in top 100 interactions
CHR	SNP	Location	gle locus P va	Gene	CHR	SNP	Location	Single locus P valu	Gene	MHC region	Stage 1	Stage 2	Combined		
6	rs9378688	2180632	4.95E-01	GMDS	10	rs315647	36493915	1.63E-01	N/A	-	3.90E-07	8.69E-02	9.93E-07	5039	
6	rs4947296	31166157	4.31E-02	N/A	6	rs3130453	31232828	5.90E-01	TCF19	MHC	3.90E-07	8.28E-01	2.10E-03	5040	
6	rs9392374	2168001	4.95E-01	GMDS	10	rs315647	36493915	1.63E-01	N/A	-	3.90E-07	9.28E-01	1.03E-03	5041	
6	rs9405550	2175063	4.95E-01	GMDS	10	rs315647	36493915	1.63E-01	N/A	-	3.90E-07	9.98E-01	1.30E-03	5042	
4	rs2231142	89271347	7.83E-01	ABCG2	9	rs10739694	1.3E+08	7.73E-01	SH2D3C	-	3.91E-07	5.11E-01	5.76E-05	5043	
5	rs10035580	16617901	7.32E-01	FAM134B	9	rs940120	14896868	8.68E-01	FREM1	-	3.91E-07	7.21E-01	2.12E-04	5044	
13	rs7984030	43528420	9.16E-01	N/A	18	rs16973990	36862780	3.43E-01	N/A	-	3.91E-07	N/A	N/A	5045	
2	rs17033166	67262298	7.40E-02	N/A	9	rs4979295	1.16E+08	1.77E-01	ZNF618	-	3.91E-07	5.50E-02	4.96E-07	5046	
4	rs1006467	7738552	3.15E-02	SORCS2	17	rs2663319	737553	9.44E-01	NXN	-	3.91E-07	7.15E-01	7.80E-04	5047	
2	rs874060	2.41E+08	3.07E-01	NDUFA10	16	rs4782717	81167218	2.78E-01	N/A	-	3.91E-07	9.87E-01	1.81E-04	5048	
12	rs10505911	23913427	1.61E-01	SOX5	20	rs805764	5624783	6.50E-01	N/A	-	3.91E-07	1.71E-03	3.62E-02	5049	
16	rs7500355	84634601	7.85E-01	N/A	17	rs1973661	22125162	8.70E-01	N/A	-	3.91E-07	2.14E-01	1.67E-03	5050	
6	rs2769004	6868655	6.36E-01	N/A	3	rs1985121	1.21E+08	5.30E-01	PLA1A	-	3.91E-07	6.61E-01	1.86E-05	5051	
6	rs2769004	6868655	6.36E-01	N/A	3	rs2670292	1.21E+08	5.30E-01	COX17	-	3.91E-07	6.61E-01	1.86E-05	5052	
6	rs2769004	6868655	6.36E-01	N/A	3	rs4688021	1.21E+08	5.30E-01	PLA1A	-	3.91E-07	6.61E-01	1.86E-05	5053	
6	rs2769006	6868881	6.36E-01	N/A	3	rs1985121	1.21E+08	5.30E-01	PLA1A	-	3.91E-07	6.61E-01	1.86E-05	5054	
6	rs2769006	6868881	6.36E-01	N/A	3	rs2670292	1.21E+08	5.30E-01	COX17	-	3.91E-07	6.61E-01	1.86E-05	5055	
6	rs2769006	6868881	6.36E-01	N/A	3	rs4688021	1.21E+08	5.30E-01	PLA1A	-	3.91E-07	6.61E-01	1.86E-05	5056	
1	rs1340985	74933312	4.80E-01	CRYZ	8	rs7823327	22618297	3.67E-01	PEBP4	-	3.91E-07	3.57E-01	1.40E-05	5057	
2	rs7597923	77088361	7.21E-01	LRRTM4	5	rs468828	3205858	8.16E-01	N/A	-	3.91E-07	4.52E-01	6.58E-04	5058	
2	rs1595752	4863708	9.21E-02	N/A	8	rs10956624	1.33E+08	3.62E-01	EFR3A	-	3.91E-07	6.55E-01	9.76E-05	5059	
6	rs911193	1.55E+08	9.23E-01	N/A	13	rs3916966	1.05E+08	2.03E-01	DAOA	-	3.91E-07	8.58E-01	1.59E-05	5060	
1	rs10782578	86848885	5.63E-01	N/A	5	rs12519981	21610367	8.39E-01	LOC728411	-	3.91E-07	3.05E-01	3.43E-06	5061	
2	rs11695426	1.88E+08	1.78E-01	N/A	21	rs958924	26498774	5.76E-02	N/A	-	3.91E-07	7.53E-01	1.77E-04	5062	
20	rs760998	36279152	4.78E-01	KIAA1755	20	rs6017202	41901496	1.28E-01	N/A	-	3.91E-07	9.22E-01	2.07E-04	5063	
10	rs11595366	11454821	7.08E-01	N/A	11	rs11226848	1.05E+08	8.16E-01	GRIA4	-	3.92E-07	8.59E-01	1.04E-04	5064	
6	rs4947296	31166157	4.31E-02	N/A	6	rs2394990	31448538	6.80E-02	HLA-B	MHC	3.92E-07	8.85E-01	1.54E-04	5065	
10	rs11595366	11454821	7.08E-01	N/A	11	rs10791774	1.05E+08	8.16E-01	GRIA4	-	3.92E-07	8.85E-01	1.54E-04	5066	
3	rs875581	1.58E+08	4.49E-01	KCN/AB1	12	rs11061542	1.31E+08	2.20E-01	N/A	-	3.92E-07	4.63E-01	5.03E-05	5067	
6	rs1065657	90103732	7.71E-02	UBE2J1	12	rs6490003	1.14E+08	9.76E-01	N/A	-	3.92E-07	5.41E-02	5.07E-07	5068	
7	rs10953556	77883184	8.70E-02	MAGI2	19	rs257675	63176021	6.07E-01	ZNF606	-	3.92E-07	8.13E-01	1.72E-05	5069	
2	rs13002663	1.45E+08	2.06E-01	ZEB2	17	rs8067882	18747856	2.48E-01	PRPSAP2	-	3.92E-07	9.97E-01	3.54E-04	5070	
1	rs12131363	2.37E+08	5.71E-01	N/A	11	rs12293251	12156242	7.25E-01	MICAL2	-	3.92E-07	7.52E-02	1.15E-06	5071	
3	rs12491180	1.89E+08	3.07E-01	N/A	4	rs4475174	65038105	2.01E-02	N/A	-	3.92E-07	8.45E-01	3.69E-05	5072	
1	rs12139561	94076827	9.62E-01	MIR760	5	rs794731	1.74E+08	1.19E-01	N/A	-	3.92E-07	1.82E-01	3.78E-03	5073	
7	rs2190529	1.1E+08	9.37E-01	IMMP2L	8	rs12676463	32459740	6.39E-01	NRG1	-	3.92E-07	8.64E-01	1.88E-05	5074	
9	rs1041681	29726564	9.95E-01	N/A	12	rs894010	60670094	4.92E-01	FAM19A2	-	3.92E-07	8.97E-01	4.04E-05	5075	
1	rs4925659	2.46E+08	4.01E-02	NLRP3	7	rs1016019	9945064	8.61E-01	N/A	-	3.92E-07	8.54E-01	7.86E-06	5076	
13	rs3956272	28447099	2.96E-01	N/A	19	rs4926176	12970908	8.59E-02	NFIX	-	3.92E-07	9.32E-01	1.42E-04	5077	
6	rs4709845	1.65E+08	1.34E-01	N/A	10	rs1896248	59890187	8.58E-01	N/A	-	3.93E-07	6.00E-01	1.34E-05	5078	
5	rs6864519	1751123	6.91E-01	N/A	15	rs2719889	23078573	1.06E-01	SNORD115-48	-	3.93E-07	2.53E-01	2.04E-06	5079	
2	rs1366765	99807781	1.97E-01	AFF3	8	rs3885690	10864279	5.90E-01	XKR6	-	3.93E-07	8.70E-01	1.27E-04	5080	
4	rs12502363	90932087	1.75E-01	SNCA	5	rs1500238	98647612	3.63E-01	N/A	-	3.93E-07	9.98E-01	1.55E-04	5081	
4	rs3822095	90955540	1.75E-01	SNCA	5	rs1500238	98647612	3.63E-01	N/A	-	3.93E-07	9.98E-01	1.55E-04	5082	
2	rs7571787	1.22E+08	1.28E-01	N/A	4	rs1036767	1.8E+08	5.82E-01	N/A	-	3.93E-07	8.38E-01	7.78E-05	5083	
13	rs2121288	68747353	4.74E-01	N/A	20	rs6017335	42444239	6.63E-01	HNF4A	-	3.93E-07	6.32E-01	3.18E-04	5084	
13	rs2481873	61805091	7.22E-01	N/A	14	rs2332431	70145604	3.86E-01	MED6	-	3.93E-07	5.17E-01	4.12E-06	5085	
1	rs1324377	81823630	2.52E-02	N/A	4	rs10519415	1.38E+08	9.47E-01	N/A	-	3.93E-07	N/A	N/A	5086	
14	rs2352900	48441520	1.86E-01	N/A	15	rs2570241	49921747	2.29E-02	TMOD3	-	3.93E-07	6.18E-01	3.42E-04	5087	
6	rs9470794	38214822	9.72E-01	ZFAND3	18	rs11877899	73733998	4.97E-01	N/A	-	3.93E-07	5.14E-02	4.63E-07	5088	
5	rs4425512	18990653	9.41E-01	N/A	5	rs34236	1.03E+08	7.49E-01	N/A	-	3.93E-07	8.90E-01	3.86E-04	5089	
6	rs9380215	31157634	4.29E-02	N/A	6	rs2394990	31448538	6.80E-02	HLA-B	MHC	3.93E-07	9.87E-01	1.20E-05	5090	
5	rs4309976	18990473	9.41E-01	N/A	5	rs34236	1.03E+08	7.49E-01	N/A	-	3.93E-07	9.93E-01	2.75E-04	5091	
6	rs887464	31253899	8.59E-02	TCF19	6	rs2894181	31282506	2.32E-02	HCG27	MHC	3.94E-07	N/A	N/A	5092	
1	rs2922240	11416419	2.36E-01	N/A	7	rs2893542	37520172	6.87E-01	N/A	-	3.94E-07	2.32E-01	4.37E-04	5093	
1	rs12239243	68645691	1.51E-01	N/A	21	rs16987903	31477873	2.80E-01	TIAM1	-	3.94E-07	7.43E-01	1.04E-04	5094	
4	rs4862611	1.87E+08	1.86E-03	N/A	11	rs11233078	81706676	6.44E-02	N/A	-	3.94E-07	3.48E-01	6.41E-06	5095	
3	rs9813521	97363067	3.05E-01	N/A	7	rs4380850	1.54E+08	1.06E-01	DPP6	-	3.94E-07	5.02E-01	2.07E-05	5096	
3	rs7617433	78966169	8.31E-02	ROBO1	21	rs12483420	41428996	6.97E-02	C21orf130	-	3.94E-07	6.01E-01	2.70E-05	5097	
5	rs1181983	1.28E+08	6.83E-01	SLC27A6	9	rs10117983	1805727	7.12E-01	N/A	-	3.94E-07	6.99E-01	1.19E-04	5098	
11	rs1025438	8862037	1.09E-01	ST5	13	rs625331	1.01E+08	5.54E-03	N/ALCN	-	3.94E-07	1.51E-01	1.13E-06	5099	
1	rs629079	72118919	8.42E-01	NEGR1	12	rs10774689	1.12E+08	1.85E-02	SLC24A6	-	3.94E-07	5.02E-01	1.18E-05	5100	
5	rs367615	1.09E+08	8.98E-02	N/A	20	rs1751846	57809323	3.88E-01	PHACTR3	-	3.94E-07	6.18E-01	6.02E-06	5101	
2	rs4675041	2.27E+08	2.28E-01	N/A	9	rs10120210	94324803	9.58E-01	ECM2	-	3.94E-07	7.48E-01	3.86E-04	5102	
4	rs3109836	26017925	9.71E-01	RBPJ	5	rs3105235	1.02E+08	2.11E-01	N/A	-	3.94E-07	1.65E-01	2.85E-03	5103	
6	rs1015157	1.16E+08	3.62E-01	N/A	8	rs2043967	33516574	9.43E-01	RNF122	-	3.94E-07	3.98E-01	1.12E-03	5104	
1	rs6540817	2.13E+08	6.69E-01	SMYD2	13	rs11069721	1.08E+08	5.06E-02	N/A	-	3.94E-07	4.58E-01	3.98E-04	5105	
1	rs6540819	2.13E+08	6.69E-01	SMYD2	13	rs11069721	1.08E+08	5.06E-02	N/A	-	3.94E-07	4.58E-01	3.98E-04	5106	
9	rs13287975	1.04E+08	2.90E-01	N/A	22	rs3091364	41744274	9.36E-01	N/A	-	3.94E-07	5.74E-01	2.75E-05	5107	
2	rs4332939	1.66E+08	8.53E-01	N/A	10	rs7895521	55526027	3.65E-01	PCDH15	-	3.94E-07	9.90E-01	6.14E-05	5108	
15	rs12900448	56586105	4.13E-01	LIPC	22	rs2399168	15884945	3.06E-03	CECR7	-	3.94E-07	N/A	N/A	5109	
1	rs6697016	2E+08	4.08E-01	TNNI1	15	rs10152611	68238590	5.90E-01	N/A	-	3.95E-07	6.32E-01	7.81E-05	5110	
6	rs4259245	33732199	1.45E-01	ITPR3	5	rs32745	35392931	9.60E-01	N/A	MHC	3.95E-07	9.42E-01	3.72E-05	5111	
13	rs456683	21862207	1.40E-01	N/A	15	rs8030397	31731603	1.79E-01	RYR3	-	3.95E-07	1.04E-01	1.24E-03	5112	
5	rs4298263	1.68E+08	8.90E-01	SLIT3	14	rs1057605	19825127	3.97E-01	TTC5	-	3.95E-07	8.47E-02	2.53E-03	5113	
4	rs7682791	1.26E+08	6.43E-01	N/A	11	rs7103380	46166835	1.31E-01	N/A	-	3.95E-07	4.84E-01	1.85E-04	5114	
17	rs6501526	67761913	5.02E-02	N/A	20	rs6087534	31907635	7.50E-01	CHMP4B	-	3.95E-07	5.99E-01	1.36E-05	5115	
17	rs8068238	67760317	5.02E-02	N/A	20	rs6087534	31907635	7.50E-01	CHMP4B	-	3.95E-07	5.99E-01	1.36E-05	5116	
4	rs4148155	89273691	8.33E-01	ABCG2	9	rs10819305	1.3E+08	6.61E-01	SH2D3C	-	3.95E-07	6.05E-01	8.66E-05	5117	
11	rs366257	5447119	2.05E-01	OR51I2	13	rs17058611	60173532	5.84E-01	N/A	-	3.95E-07	9.91E-01	7.80E-05	5118	
11	rs449937	5446921	2.05E-01	OR51I2	13	rs17058611	60173532	5.84E-01	N/A	-	3.95E-07	9.91E-01	7.80E-05	5119	
1	rs1319898	1.64E+08	2.64E-01	N/A	13	rs1444231	92525871	6.06E-01	N/A	-	3.95E-07	3.57E-01	9.47E-04	5120	
4	rs7682286	7471476	2.08E-01	SORCS2	14	rs4905360	95165775	9.98E-02	N/A	-	3.95E-07	4.41E-01	1.41E-06	5121	
6	rs2811231	55263663	7.58E-01	HCRTR2	18	rs1943796	69177259	6.85E-02	N/A	-	3.95E-07	5.44E-01	1.87E-05	5122	
6	rs2811232	55264771	7.58E-01	HCRTR2	18	rs1943796	69177259	6.85E-02	N/A	-	3.95E-07	5.44E-01	1.87E-05	5123	
11	rs875106	69683289	1.25E-01	ANO1	13	rs9285248	22477883	4.20E-02	N/A	-	3.95E-07	5.57E-01	2.90E-04	5124	
14	rs1566128	51584731	9.42E-01	NID2	20	rs6136179	17675223	8.24E-01	BANF2	-	3.96E-07	2.39E-01	1.17E-05	5125	
1	rs2282431	36557857	1.32E-01	THRAP3	7	rs7781554	98500304	1.19E-01	SMURF1	-	3.96E-07	3.85E-01	1.02E-03	5126	
7	rs6460666	70644605	1.31E-01	WBSCR17	9	rs2300932	1.23E+08	6.11E-01	C5	-	3.96E-07	1.20E-01	2.06E-06	5127	
6	rs10948479	49125374	1.87E-01	N/A	7	rs6968694	34183926	7.84E-01	N/A	-	3.96E-07	1.26E-01	7.14E-07	5128	
6	rs4382258	49133206	1.87E-01	N/A	7	rs6968694	34183926	7.84E-01	N/A	-	3.96E-07	1.38E-01	8.16E-07	5129	
6	rs4601116	49149287	1.87E-01	N/A	7	rs6968694	34183926	7.84E-01	N/A	-	3.96E-07	1.38E-01	8.16E-07	5130	
6	rs6458651	49124735	1.87E-01	N/A	7	rs6968694	34183926	7.84E-01	N/A	-	3.96E-07	1.38E-01	8.16E-07	5131	
6	rs10498874	71513774	6.64E-02	SMAP1	4	rs10010241	1.66E+08	3.65E-01	KLHL2	-	3.96E-07	3.59E-01	1.26E-03	5132	
2	rs12052592	1.61E+08	7.02E-01	N/A	11	rs6592878	79405363	6.40E-01	N/A	-	3.96E-07	3.87E-01	9.14E-04	5133	
6	rs2021678	1.02E+08	2.43E-01	GRIK2	20	rs6078117	1128222	8.67E-01	C20orf46	-	3.96E-07	6.31E-01	6.89E-06	5134	
2	rs4341884	2.36E+08	3.28E-01	SH3BP4	3	rs6550169	32888097	5.46E-01	TRIM71	-	3.96E-07	5.14E-01	5.45E-04	5135	
18	rs1284056	13819080	6.21E-01	MC5R	20	rs11700219	24788164	8.28E-01	N/A	-	3.96E-07	1.56E-01	3.54E-03	5136	
13	rs2174834	21866628	1.63E-01	N/A	15	rs8030397	31731603	1.79E-01	RYR3	-	3.96E-07	2.17E-01	5.95E-04	5137	
5	rs6892217	1.71E+08	4.33E-01	STK10	11	rs2298813	1.21E+08	5.91E-01	SORL1	-	3.96E-07	7.88E-01	5.32E-05	5138	
2	rs2339564	22837681	2.48E-01	N/A	2	rs7595229	2.03E+08	5.11E-01	N/A	-	3.96E-07	5.52E-01	3.22E-05	5139	
6	rs753849	48268824	9.36E-01	N/A	9	rs12685240	98118935	3.88E-01	SLC35D2	-	3.96E-07	5.56E-01	1.07E-05	5140	
3	rs12638746	89413745	1.10E-01	EPHA3	7	rs10259462	1.3E+08	5.50E-01	FLJ43663	-	3.96E-07	8.31E-01	2.04E-04	5141	
11	rs10830545	89950000	5.49E-01	N/A	13	rs1407578	60093488	4.39E-01	N/A	-	3.96E-07	6.54E-02	5.51E-07	5142	
7	rs2634080	56647326	1.18E-01	N/A	10	rs9423335	1.25E+08	9.95E-01	N/A	-	3.96E-07	8.56E-01	5.43E-05	5143	
2	rs11894060	1.41E+08	4.14E-01	LRP1B	2	rs1561609	1.64E+08	4.35E-01	FIGN	-	3.96E-07	1.42E-01	1.66E-06	5144	
12	rs1520780	1.18E+08	2.71E-01	N/A	15	rs12102256	35077585	1.40E-01	MEIS2	-	3.97E-07	1.49E-01	9.92E-07	5145	
5	rs16867696	88830576	2.27E-01	N/A	8	rs4875820	2354297	1.95E-01	N/A	-	3.97E-07	5.52E-01	1.74E-04	5146	
5	rs160909	3172423	2.69E-01	N/A	14	rs12892370	77945360	7.72E-01	NRXN3	-	3.97E-07	8.56E-01	3.60E-04	5147	
3	rs1512019	62537831	5.42E-01	CADPS	20	rs365516	14785965	7.92E-01	MACROD2	-	3.97E-07	9.88E-01	9.96E-05	5148	
4	rs12510912	1.85E+08	1.68E-01	N/A	20	rs3813914	24610065	1.34E-01	TMEM90B	-	3.97E-07	9.78E-01	1.01E-04	5149	
5	rs1422882	1.59E+08	3.47E-01	LOC285627	7	rs1005387	1.46E+08	5.78E-01	CNTN/AP2	-	3.97E-07	N/A	N/A	5150	

SNP A					SNP B						Interaction P			Ranking	Cluster in top 100 interactions
CHR	SNP	Location	gle locus P va	Gene	CHR	SNP	Location	ingle locus P valu	Gene	HC region	Stage 1	Stage 2	Combined		
3	rs1464623	1.67E+08	5.46E-01	N/A	20	rs4811713	54565885	1.74E-01	N/A	-	3.97E-07	3.35E-01	3.33E-06	5151	
13	rs4145275	1.03E+08	2.65E-02	N/A	16	rs11076522	48800875	7.48E-02	PAPD5	-	3.97E-07	2.05E-01	1.65E-03	5152	
7	rs6974901	47512004	5.04E-01	TNS3	10	rs2061049	56296804	2.25E-01	N/A	-	3.97E-07	4.36E-01	4.79E-04	5153	
12	rs10843050	9221872	5.65E-01	PZP	20	rs2223246	41378557	4.01E-01	N/A	-	3.97E-07	5.95E-01	4.88E-05	5154	
12	rs7958878	99646096	2.32E-01	N/A	17	rs12940357	9061595	3.98E-01	NTN1	-	3.97E-07	7.41E-01	2.22E-04	5155	
2	rs777589	61279125	3.60E-01	USP34	8	rs2726555	59947966	8.90E-01	TOX	-	3.97E-07	3.10E-01	6.47E-04	5156	
2	rs10192375	28745620	4.66E-02	N/A	5	rs17137209	1.01E+08	7.00E-01	N/A	-	3.97E-07	7.30E-01	5.10E-05	5157	
1	rs4658609	2.42E+08	2.39E-01	N/A	18	rs7230256	52950186	5.52E-01	N/A	-	3.97E-07	7.28E-01	3.64E-05	5158	
10	rs12766032	1.17E+08	5.38E-01	ATRNL1	13	rs2485241	84503947	1.35E-02	N/A	-	3.97E-07	7.77E-01	1.19E-03	5159	
9	rs10817344	1.14E+08	9.74E-02	N/A	12	rs7311671	60565928	3.93E-01	FAM19A2	-	3.97E-07	9.56E-01	5.30E-05	5160	
3	rs1501892	1.23E+08	6.14E-01	CASR	10	rs2680327	56663822	3.94E-01	N/A	-	3.98E-07	8.28E-02	3.73E-07	5161	
2	rs7565212	59591375	6.34E-01	N/A	11	rs385987	1.31E+08	3.63E-01	NTM	-	3.98E-07	3.57E-01	8.04E-04	5162	
3	rs1501892	1.23E+08	6.14E-01	CASR	10	rs2120763	56645255	3.94E-01	N/A	-	3.98E-07	3.69E-01	7.72E-04	5163	
6	rs11760047	1.54E+08	9.48E-01	N/A	9	rs2106373	1.36E+08	6.29E-01	VAV2	-	3.98E-07	6.36E-01	8.50E-05	5164	
6	rs4495304	31188697	4.80E-02	C6orf15	6	rs2394990	31448538	6.80E-02	HLA-B	MHC	3.98E-07	9.78E-01	2.72E-04	5165	
6	rs9397717	1.55E+08	6.24E-01	CNKSR3	12	rs10492029	1.12E+08	1.95E-01	OAS2	-	3.98E-07	4.94E-01	6.77E-05	5166	
2	rs6707521	1.98E+08	8.29E-01	PLCL1	7	rs12699758	15930763	7.54E-01	N/A	-	3.98E-07	9.65E-01	2.96E-05	5167	
19	rs4802856	56917579	4.09E-01	NCRN/A00085	22	rs132985	36893417	9.15E-01	PLA2G6	-	3.98E-07	2.67E-02	2.80E-02	5168	
8	rs6997474	59434386	4.69E-01	N/A	19	rs2116941	10195443	4.56E-01	S1PR2	-	3.98E-07	9.22E-01	4.68E-05	5169	
3	rs997559	10397920	7.82E-01	ATP2B2	5	rs3892476	1.33E+08	7.52E-01	N/A	-	3.98E-07	5.14E-02	4.69E-07	5170	
6	rs7759825	11064635	6.26E-01	SYCP2L	1	rs892975	2.23E+08	2.97E-01	CNIH3	-	3.98E-07	N/A	N/A	5171	
12	rs1805545	13679765	6.19E-01	GRIN2B	18	rs11083322	24938463	6.92E-01	N/A	-	3.98E-07	1.05E-01	5.42E-07	5172	
10	rs1338591	19916335	2.59E-01	N/A	10	rs758577	1.18E+08	3.98E-01	GFRA1	-	3.98E-07	N/A	N/A	5173	
11	rs1547893	1.31E+08	2.67E-01	NTM	12	rs11177428	67651838	9.77E-01	CPM	-	3.98E-07	9.45E-02	2.76E-03	5174	
2	rs701492	1.71E+08	4.00E-01	GAD1	3	rs7622240	85444335	7.52E-01	CADM2	-	3.98E-07	5.13E-01	4.81E-04	5175	
4	rs6837240	1.42E+08	2.60E-01	TBC1D9	16	rs11640300	3932784	8.21E-01	ADCY9	-	3.98E-07	5.64E-01	3.91E-06	5176	
6	rs9378518	806084	4.97E-01	N/A	1	rs12044604	59427090	5.87E-01	N/A	-	3.98E-07	4.10E-01	4.06E-06	5177	
10	rs11254314	17058324	1.98E-01	CUBN	11	rs10831311	94521927	7.04E-01	ENDOD1	-	3.98E-07	4.39E-01	3.45E-06	5178	
2	rs908571	45171969	8.44E-01	N/A	21	rs2226330	26321396	4.22E-01	APP	-	3.98E-07	8.80E-01	3.70E-05	5179	
1	rs599730	78366874	4.00E-01	GIPC2	7	rs177580	28465195	6.12E-01	CREB5	-	3.99E-07	4.42E-01	2.15E-04	5180	
4	rs1517945	1.38E+08	4.72E-01	N/A	5	rs2578483	1.09E+08	7.54E-01	N/A	-	3.99E-07	7.89E-02	1.08E-03	5181	
5	rs7713036	1.5E+08	3.41E-01	RBM22	11	rs4758270	7999192	2.37E-01	TUB	-	3.99E-07	6.31E-01	1.35E-03	5182	
1	rs513086	72082573	4.73E-01	NEGR1	1	rs11806852	2.09E+08	4.85E-01	KCNH1	-	3.99E-07	8.40E-01	1.13E-06	5183	
6	rs11751484	1.28E+08	4.60E-01	N/A	14	rs7157434	21598185	1.11E-01	N/A	-	3.99E-07	4.74E-01	1.10E-05	5184	
4	rs1355578	46594378	2.65E-01	COX7B2	17	rs17223566	4274255	2.14E-01	SPNS3	-	3.99E-07	5.36E-01	1.63E-04	5185	
6	rs2811231	55263663	7.58E-01	HCRTR2	18	rs11660744	69178521	7.73E-02	N/A	-	3.99E-07	6.00E-01	2.24E-05	5186	
6	rs2811232	55264771	7.58E-01	HCRTR2	18	rs11660744	69178521	7.73E-02	N/A	-	3.99E-07	6.00E-01	2.24E-05	5187	
10	rs7088704	82231910	8.87E-01	TSPAN14	13	rs518569	79663798	3.59E-01	N/A	-	3.99E-07	6.55E-01	2.99E-03	5188	
1	rs6697899	1.99E+08	2.83E-02	CAMSAP1L1	5	rs6868351	77998195	2.86E-01	LHFPL2	-	3.99E-07	9.59E-01	2.06E-04	5189	
11	rs492257	1.16E+08	1.55E-01	N/A	19	rs4926155	13321244	5.03E-01	CACN/A1A	-	3.99E-07	3.85E-01	7.47E-04	5190	
2	rs2693818	6121959	3.61E-02	N/A	5	rs4404633	63885913	1.57E-01	RGS7BP	-	3.99E-07	2.87E-01	1.61E-03	5191	
4	rs9307532	80650245	5.15E-02	N/A	7	rs16170	24263983	2.04E-01	N/A	-	3.99E-07	1.74E-01	2.96E-06	5192	
7	rs10266901	22235570	7.72E-01	RAPGEF5	19	rs2303053	54331814	1.72E-01	LIN7B	-	4.00E-07	3.05E-02	1.35E-06	5193	
8	rs6468204	33894290	5.28E-04	N/A	22	rs5746775	17930343	7.86E-02	LOC150185	-	4.00E-07	2.40E-01	1.94E-03	5194	
7	rs9641392	77861948	2.83E-02	MAGI2	12	rs1049380	26380811	5.44E-01	ITPR2	-	4.00E-07	9.72E-01	2.80E-04	5195	
3	rs631991	54613147	4.07E-01	CACN/A2D3	17	rs2521348	65011312	2.13E-02	MAP2K6	-	4.00E-07	7.02E-02	8.55E-07	5196	
3	rs9881461	1.16E+08	3.07E-01	ZBTB20	17	rs9894327	10222910	8.55E-01	MYH13	-	4.00E-07	7.77E-01	4.81E-05	5197	
5	rs282466	1.56E+08	7.48E-01	SGCD	9	rs4743285	1.01E+08	7.71E-01	GALNT12	-	4.00E-07	2.11E-02	2.84E-02	5198	
5	rs9293807	79600414	2.80E-01	SERINC5	20	rs208250	40849159	4.23E-01	PTPRT	-	4.00E-07	5.83E-01	1.52E-03	5199	
7	rs2373347	1.48E+08	2.70E-01	CNTN/AP2	15	rs7164503	66868444	7.60E-01	ANP32A	-	4.00E-07	1.92E-01	7.30E-04	5200	
6	rs657057	56402241	4.41E-02	N/A	5	rs1349589	1.02E+08	2.11E-01	SLCO4C1	-	4.00E-07	8.65E-01	7.05E-05	5201	
6	rs2248386	31119226	7.23E-01	HCG22	6	rs7761965	31381474	9.15E-01	N/A	MHC	4.01E-07	6.48E-01	2.66E-04	5202	
5	rs2560828	16619212	7.74E-01	FAM134B	9	rs940120	14896868	8.68E-01	FREM1	-	4.01E-07	1.02E-02	4.98E-08	5203	
5	rs297832	1.69E+08	7.83E-01	SLIT3	19	rs4807078	6255967	8.30E-02	ACER1	-	4.01E-07	8.58E-01	2.17E-04	5204	
1	rs9436816	68619836	3.94E-01	N/A	20	rs1886956	54313803	5.96E-01	N/A	-	4.01E-07	1.13E-01	3.19E-03	5205	
7	rs2062403	68146581	5.93E-01	N/A	7	rs6975814	69370528	5.09E-01	AUTS2	-	4.01E-07	5.70E-02	3.87E-03	5206	
8	rs7018112	4030154	5.47E-01	CSMD1	13	rs15869	31871012	3.02E-01	BRCA2	-	4.01E-07	4.83E-01	1.30E-05	5207	
2	rs11893319	1.97E+08	1.77E-01	HECW2	3	rs9840905	1.75E+08	9.57E-01	NLGN1	-	4.01E-07	7.69E-01	3.06E-04	5208	
8	rs3940483	62165144	6.29E-01	N/A	12	rs7980084	21114147	1.33E-01	LST-3TM12	-	4.01E-07	5.50E-01	1.19E-04	5209	
11	rs1996352	1.02E+08	4.37E-01	MMP7	20	rs6079468	14446396	6.38E-01	MACROD2	-	4.01E-07	3.19E-01	1.86E-04	5210	
10	rs1014246	1.18E+08	2.36E-01	HSPA12A	12	rs1860394	3309312	3.54E-01	N/A	-	4.01E-07	3.33E-01	1.73E-04	5211	
5	rs2259749	1.81E+08	3.61E-01	GNB2L1	7	rs9647713	72864743	7.71E-01	CLDN4	-	4.01E-07	3.43E-01	1.97E-06	5212	
10	rs1014246	1.18E+08	2.36E-01	HSPA12A	12	rs1860393	3309218	3.54E-01	N/A	MHC	4.01E-07	9.12E-01	8.26E-05	5213	
7	rs10246727	1.53E+08	4.18E-01	N/A	9	rs888229	1.28E+08	7.76E-01	N/A	-	4.01E-07	8.99E-02	2.52E-07	5214	
10	rs10749125	1.14E+08	3.58E-01	VTI1A	17	rs11079255	52147344	4.52E-01	N/A	-	4.01E-07	1.75E-01	4.27E-07	5215	
1	rs10802929	2.39E+08	4.97E-01	RGS7	5	rs4309976	18990473	9.41E-01	N/A	-	4.02E-07	4.98E-02	3.15E-03	5216	
1	rs10802929	2.39E+08	4.97E-01	RGS7	5	rs4425512	18990653	9.41E-01	N/A	-	4.02E-07	6.76E-02	2.54E-03	5217	
2	rs4671787	67261861	9.61E-01	N/A	12	rs10862641	75765471	1.35E-02	ZDHHC17	-	4.02E-07	4.29E-01	1.56E-03	5218	
7	rs874607	1.51E+08	9.05E-01	WDR86	9	rs2183864	12887342	3.18E-01	N/A	-	4.02E-07	N/A	N/A	5219	
5	rs17635646	1.68E+08	2.54E-01	SLIT3	10	rs10764706	27874675	3.89E-01	RAB18	-	4.02E-07	3.56E-01	2.80E-06	5220	
1	rs1335532	1.17E+08	1.78E-01	IGSF3	4	rs13116208	15039724	6.35E-01	C1QTNF7	-	4.02E-07	4.06E-01	1.71E-03	5221	
3	rs7615549	1.6E+08	6.22E-01	N/A	7	rs702479	12770960	2.89E-01	N/A	-	4.02E-07	4.93E-01	7.91E-04	5222	
2	rs4952561	42063034	7.95E-01	N/A	18	rs561802	605394	6.40E-01	CLUL1	-	4.02E-07	N/A	N/A	5223	
5	rs4866463	3742516	5.78E-01	N/A	22	rs2331112	24272670	8.95E-01	ADRBK2	-	4.02E-07	4.95E-01	5.15E-06	5224	
3	rs10513754	1.79E+08	1.99E-01	N/A	21	rs928738	33498255	5.26E-01	N/A	-	4.02E-07	1.95E-01	7.00E-04	5225	
3	rs10935384	1.42E+08	7.96E-01	CLSTN2	16	rs2355651	20024866	1.10E-01	N/A	-	4.02E-07	3.01E-01	8.09E-06	5226	
5	rs2042449	1469646	4.10E-01	SLC6A3	20	rs2425861	44362136	8.58E-01	N/A	-	4.02E-07	7.06E-01	N/A	5227	
3	rs4858553	23896118	2.37E-01	UBE2E1	10	rs2804069	10145593	2.47E-01	N/A	-	4.02E-07	9.78E-01	1.80E-05	5228	
5	rs1477625	1.01E+08	9.04E-01	N/A	12	rs529446	5091580	5.54E-01	N/A	-	4.02E-07	2.82E-01	2.28E-06	5229	
17	rs2196791	14594522	7.87E-01	N/A	17	rs7220683	73784880	2.07E-01	N/A	-	4.02E-07	5.98E-01	1.93E-05	5230	
4	rs9762579	1.66E+08	3.08E-01	TRIM61	13	rs4771012	26188493	2.32E-01	N/A	-	4.03E-07	4.06E-01	1.33E-03	5231	
3	rs4143282	5694146	9.09E-01	N/A	9	rs1416583	8200741	6.14E-01	N/A	-	4.03E-07	6.86E-01	7.97E-05	5232	
9	rs10961450	14237488	7.40E-01	N/A	13	rs2772179	40560028	9.05E-01	WBP4	-	4.03E-07	5.93E-01	2.32E-04	5233	
15	rs16968711	37380451	3.78E-01	N/A	22	rs713747	42759348	3.45E-01	PARVB	-	4.03E-07	8.07E-01	6.48E-05	5234	
11	rs11820229	59959174	8.62E-01	MS4A14	18	rs9964221	42151105	6.02E-01	RNF165	-	4.03E-07	3.11E-01	3.73E-06	5235	
13	rs3812874	60887176	3.87E-01	PCDH20	18	rs2848786	37281262	1.30E-02	N/A	-	4.03E-07	5.47E-01	6.46E-06	5236	
6	rs6907188	1.5E+08	6.86E-01	RAET1K	3	rs9866473	1.73E+08	6.06E-01	PLD1	-	4.04E-07	7.31E-01	4.92E-05	5237	
7	rs803166	90583560	5.06E-01	CDK14	10	rs2271271	75228873	4.09E-02	CAMK2G	-	4.04E-07	5.02E-01	2.07E-05	5238	
9	rs4879901	35508997	2.90E-01	RUSC2	9	rs1469598	1.16E+08	2.79E-01	COL27A1	-	4.04E-07	9.85E-01	3.05E-04	5239	
9	rs10120512	12447384	7.20E-01	N/A	10	rs1471384	67634379	4.80E-01	CTNN/A3	-	4.04E-07	2.28E-01	1.47E-03	5240	
7	rs40848	1.08E+08	7.89E-01	PNPLA8	8	rs6991398	1.16E+08	4.07E-01	N/A	-	4.04E-07	4.73E-01	2.18E-04	5241	
2	rs6738688	1.91E+08	6.57E-01	C2orf88	14	rs4899357	70047887	3.99E-01	ADAM20	-	4.04E-07	6.24E-01	2.53E-04	5242	
10	rs9663977	4507381	3.48E-01	N/A	16	rs8047711	81225172	3.35E-01	CDH13	-	4.04E-07	7.16E-01	1.40E-04	5243	
8	rs1481597	13458525	6.78E-01	C8orf48	11	rs10833283	3101248	7.58E-01	OSBPL5	-	4.04E-07	8.79E-01	3.79E-05	5244	
16	rs8050910	83696674	1.64E-02	KIAA0513	21	rs2843964	37356495	7.86E-02	TTC3	-	4.04E-07	1.40E-01	6.06E-04	5245	
10	rs1538098	3444147	6.98E-02	N/A	16	rs731710	86442048	3.16E-01	SLC7A5	-	4.04E-07	6.97E-01	2.01E-05	5246	
3	rs6792978	97025511	2.64E-01	N/A	12	rs10784285	60844581	2.64E-01	FAM19A2	-	4.04E-07	1.12E-01	2.19E-03	5247	
3	rs934438	97032728	2.64E-01	N/A	12	rs10784285	60844581	2.64E-01	FAM19A2	-	4.04E-07	1.12E-01	2.19E-03	5248	
8	rs950549	16885663	2.28E-01	FGF20	20	rs362569	10194733	6.56E-01	SN/AP25	-	4.04E-07	3.16E-02	2.21E-07	5249	
3	rs7611011	1.65E+08	4.51E-01	N/A	5	rs13162151	1.24E+08	9.33E-01	ZNF608	-	4.04E-07	5.01E-01	2.62E-04	5250	
2	rs722963	75369694	3.68E-01	N/A	10	rs953920	1.18E+08	1.93E-01	GFRA1	-	4.04E-07	5.32E-01	3.90E-05	5251	
1	rs543640	47705137	2.78E-02	N/A	12	rs1446527	63733304	7.29E-01	WIF1	-	4.04E-07	5.76E-02	4.87E-03	5252	
16	rs2312948	8600664	9.53E-01	N/A	17	rs9897184	69926356	4.67E-02	GPRC5C	-	4.05E-07	2.66E-01	2.05E-04	5253	
4	rs2218313	1.43E+08	2.64E-02	N/A	5	rs10043775	1.48E+08	7.84E-01	FBXO38	-	4.05E-07	6.58E-01	2.05E-04	5254	
4	rs2218313	1.43E+08	2.64E-02	N/A	5	rs9325095	1.48E+08	7.84E-01	FBXO38	-	4.05E-07	6.58E-01	2.05E-04	5255	
4	rs2218313	1.43E+08	2.64E-02	N/A	5	rs9325098	1.48E+08	7.84E-01	HTR4	-	4.05E-07	6.58E-01	2.05E-04	5256	
3	rs2037184	1.89E+08	2.28E-01	N/A	8	rs3110774	1.08E+08	3.04E-01	OXR1	-	4.05E-07	9.86E-01	2.53E-05	5257	
8	rs1707420	5595071	3.79E-01	N/A	9	rs9409243	1.24E+08	4.04E-01	TTLL11	-	4.05E-07	3.03E-01	7.21E-06	5258	
9	rs944591	83372048	1.28E-01	TLE1	11	rs4627097	1.25E+08	7.92E-01	STT3A	-	4.05E-07	N/A	N/A	5259	
3	rs1795306	1.06E+08	1.31E-01	N/A	8	rs7005602	58763548	1.78E-01	N/A	-	4.05E-07	2.05E-01	5.69E-06	5260	
4	rs1382176	1.32E+08	8.37E-01	N/A	8	rs2114695	82661686	2.74E-01	N/A	-	4.05E-07	7.15E-01	2.21E-05	5261	
3	rs11128068	89289912	8.95E-02	EPHA3	7	rs4731731	1.3E+08	6.07E-01	FLJ43663	-	4.05E-07	7.27E-01	5.45E-05	5262	

SNP A					SNP B						Interaction P			Ranking	Cluster in top 100 interactions
CHR	SNP	Location	gle locus P va	Gene	CHR	SNP	Location	ingle locus P valu	Gene	HC region	Stage 1	Stage 2	Combined		
11	rs1873098	1.04E+08	5.79E-01	N/A	16	rs7188834	10628697	5.52E-01	TEKT5	-	4.05E-07	9.88E-01	8.83E-05	5263	
6	rs3793048	70841467	9.61E-01	COL19A1	1	s10800925	2.02E+08	9.27E-01	N/A	-	4.05E-07	1.10E-01	2.80E-03	5264	
6	rs3806014	70852176	9.61E-01	COL19A1	1	s10800925	2.02E+08	9.27E-01	N/A	-	4.05E-07	1.34E-01	2.39E-03	5265	
2	rs1510112	39231824	9.08E-02	N/A	18	rs554440	36814255	9.50E-01	N/A	-	4.05E-07	7.31E-01	2.65E-04	5266	
2	rs1510112	39231824	9.08E-02	N/A	18	rs6507386	36791626	9.50E-01	N/A	-	4.05E-07	8.73E-01	1.66E-04	5267	
1	rs650298	16538195	2.34E-01	FBXO42	2	s10929808	12486447	8.26E-01	N/A	-	4.05E-07	4.46E-01	4.89E-04	5268	
12	rs559392	71168283	4.58E-01	TRHDE	16	rs2046532	55840586	1.11E-01	RSPRY1	-	4.05E-07	5.27E-01	2.07E-05	5269	
2	rs7604147	2.31E+08	8.53E-01	CAB39	12	rs7139067	95571241	4.31E-01	N/A	-	4.05E-07	4.03E-01	1.47E-04	5270	
10	rs2420724	1.22E+08	1.21E-02	PPAPDC1A	11	s12365860	56138083	2.99E-01	OR8U8	-	4.05E-07	8.09E-01	7.18E-05	5271	
4	rs10461165	89989606	4.18E-02	FAM13A	5	rs6863774	55730873	1.10E-01	N/A	-	4.05E-07	2.06E-01	1.96E-06	5272	
1	rs12132898	57494319	7.08E-01	DAB1	7	s10247649	95054183	5.98E-01	PDK4	-	4.05E-07	8.89E-01	3.63E-04	5273	
1	rs12132898	57494319	7.08E-01	DAB1	7	rs2073978	95053208	5.98E-01	PDK4	-	4.05E-07	8.89E-01	3.63E-04	5274	
3	rs809421	1.09E+08	4.77E-01	LOC344595	5	rs1650647	80006179	6.89E-01	MSH3	-	4.05E-07	9.52E-01	9.50E-05	5275	
7	rs4730257	77861452	2.67E-02	MAGI2	12	rs1049376	26382742	5.85E-01	ITPR2	-	4.05E-07	9.72E-01	2.56E-04	5276	
2	rs4538253	48887519	5.79E-01	N/A	12	rs1603810	62202949	6.29E-01	N/A	-	4.05E-07	N/A	N/A	5277	
9	rs1929409	11942643	3.07E-01	N/A	22	rs5992838	16644831	1.46E-01	BID	-	4.05E-07	2.01E-02	1.16E-07	5278	
4	rs7674113	83854700	2.10E-01	SCD5	7	rs38094	78071441	7.21E-01	MAGI2	-	4.05E-07	6.01E-01	1.48E-05	5279	
2	rs3816375	1.87E+08	8.71E-02	ITGAV	10	rs1926050	25458413	6.46E-01	N/A	-	4.06E-07	5.64E-01	6.49E-04	5280	
7	rs4727443	99431282	1.00E-01	ZKSCAN1	16	rs2068142	85189810	9.39E-01	FOXL1	-	4.06E-07	5.14E-01	2.38E-05	5281	
6	rs4624889	1.44E+08	4.45E-01	PHACTR2	11	s11021732	2197848	2.05E-01	N/A	-	4.06E-07	6.38E-01	4.99E-04	5282	
2	rs7585252	1.34E+08	1.52E-01	N/A	10	rs7086224	30857283	4.70E-01	N/A	-	4.06E-07	2.99E-01	3.41E-04	5283	
2	rs13407109	2.18E+08	9.06E-02	TNS1	12	s10861755	78185404	8.71E-01	SYT1	-	4.06E-07	5.26E-01	8.52E-06	5284	
6	rs2767576	1.57E+08	6.69E-02	N/A	7	s13221445	3560091	2.75E-02	SDK1	-	4.06E-07	5.37E-01	5.66E-03	5285	
5	rs4975526	1094105	9.76E-01	NKD2	11	rs7479626	7532894	9.57E-01	PPFIBP2	-	4.06E-07	7.66E-01	3.76E-05	5286	
5	rs4975526	1094105	9.76E-01	NKD2	11	rs7481625	7532926	9.57E-01	PPFIBP2	-	4.06E-07	7.66E-01	3.76E-05	5287	
11	rs10458992	1.25E+08	2.95E-01	CDON	12	rs2041385	6432554	3.97E-01	LOC678655	-	4.06E-07	9.84E-01	7.14E-04	5288	
11	rs10458992	1.25E+08	2.95E-01	CDON	12	rs2532497	6434471	3.97E-01	LOC678655	-	4.06E-07	9.84E-01	7.14E-04	5289	
4	rs7668220	1.57E+08	4.01E-01	GUCY1A3	5	rs2045990	1.01E+08	9.39E-01	N/A	-	4.06E-07	3.50E-01	1.14E-03	5290	
2	rs2896773	1.27E+08	5.54E-01	N/A	21	rs9978525	33869726	1.65E-01	CRYZL1	-	4.06E-07	5.35E-01	5.96E-04	5291	
2	rs754737	1.27E+08	5.54E-01	N/A	21	rs9978525	33869726	1.65E-01	CRYZL1	-	4.06E-07	5.35E-01	5.96E-04	5292	
7	rs4029796	71587226	6.99E-02	N/A	15	s12905875	96175633	3.43E-01	LOC91948	-	4.07E-07	7.89E-01	2.71E-04	5293	
2	rs7585163	2.06E+08	4.11E-01	PARD3B	9	s10759341	1.11E+08	2.15E-01	PTPN3	-	4.07E-07	9.39E-01	8.98E-05	5294	
13	rs2761367	31877647	1.37E-02	BRCA2	17	rs8066210	9963168	7.55E-01	GAS7	-	4.07E-07	2.95E-03	2.03E-08	5295	
9	rs4745511	77952115	7.17E-01	N/A	11	rs984371	55334274	3.18E-01	OR5D14	-	4.07E-07	4.72E-01	5.00E-04	5296	
7	rs17164162	11266179	6.32E-01	N/A	17	rs1534308	52740495	3.62E-02	MSI2	-	4.07E-07	7.29E-01	2.42E-05	5297	
6	rs636393	1.38E+08	6.03E-01	N/A	3	rs1579905	74711280	1.34E-01	N/A	-	4.07E-07	9.55E-01	7.39E-05	5298	
11	rs4648319	1.13E+08	7.38E-01	DRD2	16	rs1548808	9873708	6.50E-01	GRIN2A	-	4.07E-07	9.64E-01	1.86E-04	5299	
2	rs13013813	2.12E+08	6.44E-01	N/A	11	rs764848	36925579	3.79E-01	N/A	-	4.07E-07	2.68E-01	6.73E-04	5300	
2	rs13013813	2.12E+08	6.44E-01	N/A	11	s10836645	36941613	3.79E-01	N/A	-	4.07E-07	3.42E-01	4.70E-04	5301	
3	rs2573174	70965867	1.25E-01	N/A	20	rs1537479	56260074	5.33E-01	PPP4R1L	-	4.07E-07	N/A	N/A	5302	
3	rs10440084	84955494	1.83E-01	N/A	20	rs1015362	32202273	6.09E-01	N/A	-	4.07E-07	6.30E-01	2.41E-04	5303	
12	rs7296020	61595594	4.84E-01	PPM1H	13	rs1571596	91386827	7.95E-01	GPC5	-	4.07E-07	7.14E-01	3.89E-04	5304	
6	rs9405911	6227777	3.24E-01	F13A1	21	rs768039	26329891	6.37E-02	APP	-	4.07E-07	4.91E-02	5.08E-07	5305	
1	rs12022475	80075562	4.18E-02	N/A	10	rs615019	3553318	6.85E-01	N/A	-	4.07E-07	3.20E-01	6.47E-06	5306	
17	rs17564605	46215431	2.27E-01	C17orf73	18	rs4800633	21293512	7.53E-02	N/A	-	4.07E-07	4.76E-01	5.16E-05	5307	
6	rs7774407	33883369	7.12E-01	MLN	11	rs7112954	1127086	4.52E-01	N/A	MHC	4.07E-07	7.06E-01	1.47E-04	5308	
1	rs326910	96624140	9.19E-01	N/A	5	s10471333	67291220	9.55E-01	N/A	-	4.07E-07	3.40E-01	1.08E-03	5309	
6	rs2064212	1.39E+08	9.42E-01	KIAA1244	5	s17540829	16581227	6.92E-01	FAM134B	-	4.08E-07	2.56E-01	7.69E-06	5310	
6	rs515077	8125285	5.80E-01	N/A	7	rs2299219	86255781	3.01E-01	GRM3	-	4.08E-07	4.73E-01	2.53E-04	5311	
5	rs2591454	82753420	9.76E-01	N/A	11	rs7126761	13079129	7.61E-01	N/A	-	4.08E-07	8.49E-01	1.11E-05	5312	
4	rs2196978	80754993	5.99E-02	N/A	7	rs9886302	70389420	4.37E-01	WBSCR17	-	4.08E-07	3.35E-01	7.88E-06	5313	
9	rs4322101	1.28E+08	1.17E-01	N/A	20	rs1411206	50960763	8.42E-01	N/A	-	4.08E-07	6.10E-01	1.41E-05	5314	
4	rs7672573	16090777	5.24E-01	N/A	5	rs4246043	1.53E+08	4.78E-01	N/A	-	4.08E-07	7.53E-01	4.95E-04	5315	
4	rs7672573	16090777	5.24E-01	N/A	5	rs4958655	1.53E+08	4.78E-01	N/A	-	4.08E-07	7.53E-01	4.95E-04	5316	
11	rs876378	1.13E+08	4.80E-01	N/A	16	rs8047350	73236745	7.81E-01	RFWD3	-	4.08E-07	9.13E-01	1.82E-04	5317	
17	rs6501526	67761913	5.02E-02	N/A	20	rs2064505	31859046	7.12E-01	ZNF341	-	4.08E-07	5.65E-01	1.25E-05	5318	
17	rs8068238	67760317	5.02E-02	N/A	20	rs2064505	31859046	7.12E-01	ZNF341	-	4.08E-07	5.65E-01	1.25E-05	5319	
2	rs11300	2.02E+08	5.77E-01	ALS2	9	s10869600	77415635	9.47E-01	MIR548H3	-	4.08E-07	7.52E-01	4.13E-05	5320	
2	rs11680658	2.4E+08	8.77E-01	HDAC4	4	s11941589	1.4E+08	3.96E-01	N/A	-	4.08E-07	4.11E-01	1.55E-03	5321	
2	rs3769570	32867326	3.53E-01	N/A	4	rs4552542	75818014	9.42E-01	N/A	-	4.08E-07	9.00E-01	7.30E-05	5322	
2	rs6545648	57511448	3.54E-01	N/A	2	rs4672323	59936804	2.75E-02	N/A	-	4.08E-07	1.53E-02	2.39E-08	5323	
2	rs6545648	57511448	3.54E-01	N/A	2	rs2419402	59932754	2.75E-02	N/A	-	4.08E-07	1.56E-02	2.45E-08	5324	
12	rs2193140	16490730	8.21E-01	N/A	14	rs7155553	73171266	2.19E-01	ACOT6	-	4.08E-07	6.59E-01	1.85E-04	5325	
6	rs2691187	1.11E+08	9.24E-02	CDK19	1	s10798126	1.86E+08	8.20E-01	N/A	-	4.08E-07	6.70E-01	1.99E-04	5326	
12	rs10772938	16481578	8.21E-01	N/A	14	rs7155553	73171266	2.19E-01	ACOT6	-	4.08E-07	7.02E-01	1.62E-04	5327	
4	rs7665758	46652454	1.94E-01	GABRA4	17	s17223566	4274255	2.14E-01	SPNS3	-	4.08E-07	3.81E-01	2.55E-04	5328	
6	rs4947296	31166157	4.31E-02	N/A	6	rs3131003	31201461	6.51E-01	C6orf15	MHC	4.08E-07	5.50E-01	3.04E-05	5329	
10	rs1751274	4472350	3.58E-01	N/A	16	rs8047711	81225172	3.35E-01	CDH13	-	4.08E-07	7.18E-01	2.64E-04	5330	
1	rs6428727	1.18E+08	4.83E-01	SPAG17	2	rs7421653	2.34E+08	8.14E-01	INPP5D	-	4.09E-07	5.98E-01	1.26E-04	5331	
15	rs2462847	50499221	6.86E-01	MYO5A	19	rs255265	35046780	9.53E-01	N/A	-	4.09E-07	N/A	N/A	5332	
6	rs9388724	1.3E+08	7.93E-01	ARHGAP18	15	rs7175648	95247346	2.52E-01	N/A	-	4.09E-07	2.39E-01	8.86E-04	5333	
6	rs6932730	31462161	9.29E-02	MICA	5	rs1457115	1.11E+08	4.77E-01	CAMK4	MHC	4.09E-07	3.58E-01	1.01E-03	5334	
6	rs6932730	31462161	9.29E-02	MICA	5	rs6875225	1.11E+08	4.77E-01	CAMK4	MHC	4.09E-07	3.58E-01	1.01E-03	5335	
11	rs11222391	1.3E+08	8.45E-01	SNX19	16	rs4784182	60912752	1.71E-01	N/A	-	4.09E-07	5.47E-01	1.61E-05	5336	
1	rs2076620	29488905	4.38E-01	PTPRU	7	rs2657389	1.58E+08	5.37E-01	N/A	-	4.09E-07	4.19E-01	3.29E-04	5337	
8	rs10503617	18474753	6.82E-01	PSD3	10	rs871747	5741767	2.31E-01	ASB13	-	4.09E-07	4.49E-01	3.58E-04	5338	
1	rs868162	5856823	3.31E-01	NPHP4	12	s10848943	3814283	7.50E-01	PARP11	-	4.09E-07	7.95E-01	8.14E-05	5339	
1	rs1319776	2.16E+08	3.05E-01	N/A	9	s10123353	4175934	5.46E-01	GLIS3	-	4.09E-07	2.52E-01	2.44E-06	5340	
13	rs7328408	43532989	9.62E-01	N/A	18	s16973990	36862780	3.43E-01	N/A	-	4.09E-07	2.82E-01	4.65E-04	5341	
4	rs4340831	32442419	5.95E-01	N/A	5	rs2921571	5592894	6.48E-01	N/A	-	4.09E-07	8.01E-01	4.60E-05	5342	
7	rs13225455	1.03E+08	1.26E-01	RELN	20	rs2313152	60782438	4.05E-01	LOC100127888	-	4.09E-07	9.08E-02	1.38E-06	5343	
6	rs2508015	31118179	8.45E-01	HCG22	6	rs396243	31383153	2.17E-01	N/A	MHC	4.09E-07	2.29E-01	2.49E-03	5344	
4	rs1967559	57000456	5.50E-01	PPAT	16	s13332555	54442500	6.18E-01	N/A	-	4.09E-07	2.30E-01	4.08E-06	5345	
7	rs7790976	1.41E+08	5.67E-01	N/A	15	rs2242062	56621683	2.99E-01	LIPC	-	4.09E-07	5.92E-01	2.06E-04	5346	
2	rs1531968	2.4E+08	4.33E-01	N/A	4	rs1441209	35015347	4.44E-01	N/A	-	4.09E-07	1.69E-02	2.55E-08	5347	
6	rs7761965	31381474	9.15E-01	N/A	6	rs9266722	31457901	1.13E-01	N/A	MHC	4.09E-07	5.79E-01	1.60E-05	5348	
5	rs6555834	1.68E+08	1.49E-01	SLIT3	10	rs2505331	27816417	5.15E-01	RAB18	-	4.09E-07	9.64E-01	4.41E-05	5349	
1	rs7537318	2.45E+08	7.00E-02	SMYD3	8	s16886947	1.16E+08	9.74E-01	N/A	-	4.10E-07	5.58E-01	2.63E-05	5350	
1	rs10733099	1.18E+08	4.83E-01	SPAG17	2	rs7421653	2.34E+08	8.14E-01	INPP5D	-	4.10E-07	5.98E-01	1.27E-04	5351	
6	rs12528430	1.69E+08	3.07E-01	N/A	3	rs2399248	1.1E+08	9.05E-01	GUCA1C	-	4.10E-07	6.03E-01	2.65E-04	5352	
6	rs17182135	1.69E+08	3.07E-01	N/A	3	rs2399248	1.1E+08	9.05E-01	GUCA1C	-	4.10E-07	6.03E-01	2.65E-04	5353	
1	rs10800775	2E+08	6.35E-01	TNNT2	1	s12562849	2.31E+08	7.66E-01	SIPA1L2	-	4.10E-07	6.64E-01	3.74E-05	5354	
6	rs7769903	1.38E+08	3.50E-01	N/A	3	rs6786301	1.57E+08	5.11E-01	N/A	-	4.10E-07	5.50E-01	4.18E-04	5355	
4	rs3101281	29750478	2.88E-01	N/A	18	rs3902020	48065150	7.07E-01	N/A	-	4.10E-07	5.81E-01	1.69E-04	5356	
6	rs2073021	38239420	9.46E-01	ZFAND3	18	s11877899	73733998	4.97E-01	N/A	-	4.10E-07	9.41E-01	1.40E-05	5357	
6	rs11759483	38195657	9.46E-01	ZFAND3	18	s11877899	73733998	4.97E-01	N/A	-	4.10E-07	9.87E-01	1.20E-05	5358	
2	rs12987632	42986241	4.26E-01	N/A	4	rs6552407	1.81E+08	5.03E-03	N/A	-	4.10E-07	5.10E-01	1.27E-03	5359	
5	rs6453473	79030235	2.00E-01	CMYA5	5	rs4526101	1.26E+08	6.08E-01	GRAMD3	-	4.10E-07	4.76E-01	5.89E-04	5360	
8	rs10107668	33643721	2.78E-02	N/A	16	rs130021	3772472	7.22E-01	CREBBP	-	4.10E-07	8.48E-01	7.92E-05	5361	
1	rs6678713	1.89E+08	5.35E-01	FAM5C	15	rs600753	53546485	3.82E-01	DYX1C1	-	4.10E-07	9.31E-01	7.15E-05	5362	
13	rs828187	90129301	8.25E-01	N/A	15	rs7178006	24619527	8.21E-01	N/A	-	4.10E-07	N/A	N/A	5363	
6	rs1737727	35012466	6.45E-01	ANKS1A	9	rs372957	31962985	1.19E-01	N/A	MHC	4.10E-07	1.63E-01	1.03E-03	5364	
6	rs1737727	35012466	6.45E-01	ANKS1A	9	rs453019	31975376	1.19E-01	N/A	MHC	4.10E-07	1.71E-01	9.81E-04	5365	
2	rs4671787	67261861	9.61E-01	N/A	12	s10862509	75708572	1.89E-02	ZDHHC17	-	4.10E-07	4.28E-01	1.55E-03	5366	
3	rs13068206	1.94E+08	6.74E-01	FGF12	14	s11627673	96845032	9.62E-01	N/A	-	4.10E-07	9.41E-01	8.42E-05	5367	
4	rs4522850	8773890	8.78E-01	N/A	18	rs3786309	28226772	2.56E-02	FAM59A	-	4.10E-07	1.87E-01	6.96E-07	5368	
3	rs10513754	1.79E+08	1.99E-01	N/A	21	rs2834144	33501939	4.81E-01	N/A	-	4.10E-07	2.80E-01	5.43E-04	5369	
1	rs2343479	1.61E+08	4.66E-01	C1orf110	4	rs4401481	60397850	3.91E-01	N/A	-	4.10E-07	N/A	N/A	5370	
6	rs2327351	1.34E+08	3.81E-01	EYA4	19	rs8102503	51727276	7.25E-01	N/A	-	4.11E-07	2.90E-01	9.54E-04	5371	
3	rs9811329	1.57E+08	6.44E-01	PLCH1	14	rs2239220	71922329	3.85E-01	RGS6	-	4.11E-07	3.02E-01	1.18E-03	5372	
5	rs13190535	38686394	5.91E-01	N/A	16	rs1496955	75547875	5.60E-01	N/A	-	4.11E-07	8.27E-01	5.31E-05	5373	
4	rs9307532	80650245	5.15E-02	N/A	7	rs16480	24277594	2.38E-01	NPY	-	4.11E-07	1.95E-01	4.61E-06	5374	

SNP A					SNP B						Interaction P			Ranking	Cluster in top 100 interactions
CHR	SNP	Location	gle locus P va	Gene	CHR	SNP	Location	Single locus P valu	Gene	IHC region	Stage 1	Stage 2	Combined		
1	rs3738605	16595671	1.88E-01	C1orf144	2	rs10929808	12486447	8.26E-01	N/A	-	4.11E-07	4.75E-01	3.74E-04	5375	
18	rs748717	19083582	5.45E-01	CABLES1	18	rs8086877	49928133	6.74E-01	MBD2	-	4.11E-07	7.77E-01	2.48E-05	5376	
4	rs4833025	37933638	7.62E-01	N/A	8	rs2163372	1.26E+08	6.16E-01	MTSS1	-	4.11E-07	9.53E-01	6.22E-05	5377	
1	rs4653178	36568017	7.52E-01	STK40	13	rs1230472	45675502	6.29E-01	N/A	-	4.11E-07	9.76E-01	2.08E-05	5378	
17	rs7222435	14270245	1.45E-01	N/A	20	rs2208185	20961756	1.58E-01	N/A	-	4.11E-07	N/A	N/A	5379	
3	rs4678222	1.38E+08	8.99E-01	N/A	7	rs11514853	1.48E+08	7.55E-02	CNTN/AP2	-	4.11E-07	7.94E-01	6.13E-06	5380	
2	rs4610017	1.88E+08	1.53E-01	N/A	12	rs7968902	64649337	5.94E-01	HMGA2	-	4.11E-07	6.94E-02	2.69E-03	5381	
16	rs7500465	8598095	9.65E-01	N/A	17	rs9897184	69926356	4.67E-02	GPRC5C	-	4.11E-07	3.97E-01	3.60E-04	5382	
1	rs807247	25993456	5.33E-01	MAN1C1	11	rs1661409	1.01E+08	7.87E-01	KIAA1377	-	4.11E-07	3.08E-01	1.71E-05	5383	
1	rs4130463	2.38E+08	3.44E-01	N/A	2	rs10197959	2.3E+08	4.27E-01	DNER	-	4.12E-07	3.37E-01	3.89E-05	5384	
3	rs2365929	61608890	7.52E-01	PTPRG	4	rs4303965	8151172	4.06E-01	ABLIM2	-	4.12E-07	4.51E-01	7.88E-06	5385	
10	rs7919956	2330038	8.38E-01	N/A	11	rs835784	44820394	1.36E-01	TSPAN18	-	4.12E-07	8.94E-01	1.22E-04	5386	
19	rs929502	46822616	5.91E-01	CEACAM4	20	rs6063278	46634198	1.79E-01	N/A	-	4.12E-07	6.73E-01	2.94E-05	5387	
11	rs11042524	9858687	1.44E-01	SBF2	11	rs10766003	12872450	7.06E-01	TEAD1	-	4.12E-07	7.28E-01	6.67E-04	5388	
2	rs4627617	54513960	2.43E-01	N/A	5	rs10454913	73720859	6.88E-01	N/A	-	4.12E-07	2.82E-01	5.81E-03	5389	
3	rs6788471	1.77E+08	5.83E-01	N/A	12	rs3825080	56157822	7.63E-01	INHBE	-	4.12E-07	3.18E-01	1.13E-05	5390	
10	rs7906057	8566119	7.31E-01	N/A	11	rs956349	22374956	7.04E-01	SLC17A6	-	4.12E-07	5.92E-01	2.95E-04	5391	
10	rs7906057	8566119	7.31E-01	N/A	11	rs2665716	22377858	7.50E-01	N/A	-	4.12E-07	6.76E-01	3.10E-04	5392	
6	rs4713646	33714501	7.65E-01	ITPR3	3	rs637999	1.67E+08	8.73E-02	N/A	MHC	4.12E-07	8.86E-01	2.20E-04	5393	
5	rs277318	1.54E+08	6.51E-01	N/A	15	rs12440646	98665445	2.63E-01	ADAMTS17	-	4.12E-07	N/A	N/A	5394	
6	rs831481	37110160	1.82E-01	FGD2	1	rs6681392	1.53E+08	7.78E-02	KCNN3	-	4.12E-07	7.03E-01	8.78E-06	5395	
10	rs4256883	63577608	9.27E-02	N/A	11	rs1020734	41535671	3.94E-01	N/A	-	4.12E-07	8.43E-01	4.76E-05	5396	
3	rs7617131	39883849	6.57E-01	MYRIP	12	rs220583	13852010	1.88E-01	GRIN2B	-	4.12E-07	6.87E-01	8.37E-05	5397	
4	rs7664678	1.14E+08	5.16E-01	ALPK1	7	rs7778134	7891322	3.73E-02	N/A	-	4.12E-07	9.81E-01	8.70E-05	5398	
2	rs6545648	57511448	3.54E-01	N/A	2	rs6545760	59954716	2.82E-02	N/A	-	4.13E-07	1.06E-02	1.43E-08	5399	
3	rs6792702	1.51E+08	6.36E-01	RNF13	9	rs10514834	88605481	8.82E-01	N/A	-	4.13E-07	6.94E-01	4.06E-05	5400	
2	rs831017	1.7E+08	3.65E-01	LRP2	4	rs9992839	32447798	6.01E-01	N/A	-	4.13E-07	1.04E-03	9.62E-03	5401	
3	rs10510933	65123661	9.37E-02	N/A	12	rs609704	51221514	5.17E-01	KRT71	-	4.13E-07	3.37E-01	1.23E-03	5402	
3	rs7635505	64624234	1.80E-01	ADAMTS9	16	rs12923918	24751904	6.69E-01	TNRC6A	-	4.13E-07	2.26E-01	2.47E-06	5403	
6	rs3130685	31314185	9.60E-02	N/A	6	rs3130534	31317024	3.50E-01	N/A	MHC	4.13E-07	6.46E-01	8.46E-05	5404	
12	rs11059460	1.27E+08	2.00E-01	N/A	18	rs11082498	41756726	2.99E-01	KIAA1632	-	4.13E-07	5.73E-01	3.59E-04	5405	
13	rs16971228	1.07E+08	5.06E-01	FAM155A	18	rs2036660	70967007	2.32E-01	N/A	-	4.13E-07	N/A	N/A	5406	
1	rs12724129	11880226	9.76E-01	N/A	11	rs6589900	1.21E+08	7.03E-01	N/A	-	4.13E-07	1.64E-01	9.02E-07	5407	
12	rs12297113	7494612	2.61E-01	CD163L1	12	rs10505774	13327672	3.47E-01	N/A	-	4.13E-07	2.90E-01	4.95E-04	5408	
14	rs7148194	1.04E+08	9.05E-01	KIF26A	17	rs2880540	39043136	3.51E-01	N/A	-	4.14E-07	5.68E-01	1.50E-05	5409	
1	rs2046616	92604157	3.47E-01	RPAP2	3	rs12497701	59329494	5.42E-02	N/A	-	4.14E-07	3.29E-01	8.98E-04	5410	
2	rs4832401	17968469	3.73E-01	KCNS3	5	rs1374017	33871110	4.57E-01	ADAMTS12	-	4.14E-07	4.86E-01	5.17E-04	5411	
4	rs1452402	1.1E+08	1.85E-01	N/A	8	rs2934950	87945230	4.04E-01	CNBD1	-	4.14E-07	7.42E-01	4.82E-05	5412	
8	rs6991967	1.03E+08	2.39E-02	GRHL2	20	rs2425540	40859592	4.66E-01	PTPRT	-	4.14E-07	N/A	N/A	5413	
3	rs992089	1.6E+08	7.49E-01	N/A	10	rs11190298	1.02E+08	8.59E-01	DNMBP	-	4.14E-07	5.55E-01	3.70E-04	5414	
13	rs2174834	21866628	1.63E-01	N/A	15	rs1979739	31737586	3.55E-02	RYR3	-	4.14E-07	3.33E-01	4.11E-04	5415	
5	rs877886	7712279	7.77E-01	ADCY2	13	rs1409445	88127685	3.68E-01	N/A	-	4.14E-07	5.68E-01	2.39E-05	5416	
5	rs7700862	7699404	7.77E-01	ADCY2	13	rs1409445	88127685	3.68E-01	N/A	-	4.14E-07	5.90E-01	2.50E-05	5417	
7	rs6979644	1.44E+08	4.14E-02	TPK1	17	rs12949534	33222700	2.59E-01	N/A	-	4.14E-07	6.35E-01	3.24E-04	5418	
3	rs1185188	1.05E+08	4.54E-01	N/A	9	rs7869607	90490207	2.69E-01	N/A	-	4.15E-07	3.10E-02	5.24E-07	5419	
2	rs1466180	1.69E+08	7.53E-01	N/A	5	rs2548373	42963857	9.66E-02	N/A	-	4.15E-07	1.30E-01	2.89E-03	5420	
4	rs17005836	83746757	2.38E-01	C4orf11	9	rs1358907	3158478	2.43E-01	N/A	-	4.15E-07	2.15E-01	1.78E-03	5421	
15	rs11073466	94529525	6.77E-01	N/A	16	rs3785220	7644364	7.76E-01	A2BP1	-	4.15E-07	3.30E-01	3.49E-06	5422	
4	rs2310322	1.87E+08	8.02E-01	PDLIM3	14	rs9323293	56024440	2.29E-01	N/A	-	4.15E-07	9.23E-01	2.69E-04	5423	
6	rs1486039	76965256	6.49E-01	N/A	3	rs1995453	72031489	3.15E-01	N/A	-	4.15E-07	1.51E-01	1.20E-06	5424	
6	rs1976109	76981958	6.49E-01	N/A	3	rs1995453	72031489	3.15E-01	N/A	-	4.15E-07	1.51E-01	1.20E-06	5425	
6	rs2314204	76979633	6.49E-01	N/A	3	rs1995453	72031489	3.15E-01	N/A	-	4.15E-07	1.51E-01	1.20E-06	5426	
3	rs900327	45628440	8.56E-01	LIMD1	5	rs7448746	92602250	2.15E-01	N/A	-	4.15E-07	8.65E-01	1.60E-05	5427	
9	rs507586	1.35E+08	8.86E-01	GFI1B	11	rs7125406	1.33E+08	6.28E-01	N/A	-	4.15E-07	8.92E-02	3.47E-06	5428	
3	rs270150	1.88E+08	6.46E-01	N/A	10	rs1245907	1.12E+08	8.57E-02	N/A	-	4.15E-07	8.14E-01	1.21E-05	5429	
3	rs17216900	57158917	4.42E-01	IL17RD	4	rs13118455	1.81E+08	6.41E-01	N/A	-	4.15E-07	8.23E-01	5.11E-05	5430	
3	rs9422	1.25E+08	8.04E-01	MYLK	12	rs1293739	1.12E+08	1.91E-01	OAS2	-	4.15E-07	9.35E-01	2.96E-05	5431	
7	rs1859526	70527975	9.33E-01	WBSCR17	9	rs2199552	22886283	2.85E-01	N/A	-	4.15E-07	1.15E-01	2.97E-07	5432	
2	rs12469968	1.63E+08	6.12E-01	DPP4	7	rs705308	97533299	9.17E-01	N/A	-	4.15E-07	6.07E-01	5.06E-05	5433	
1	rs789360	2.31E+08	2.09E-01	N/A	14	rs8022093	76146916	7.72E-02	N/A	-	4.15E-07	9.98E-01	1.17E-04	5434	
1	rs2765501	1.56E+08	4.10E-01	CD5L	5	rs10038486	1.77E+08	9.07E-01	FAM153C	-	4.15E-07	2.75E-02	5.28E-03	5435	
3	rs9985428	1.04E+08	1.85E-01	N/A	10	rs7901814	24886122	6.72E-01	KIAA1217	-	4.15E-07	5.91E-01	7.72E-04	5436	
2	rs2447741	38250946	3.87E-01	C2orf58	20	rs6139808	5641718	5.97E-01	N/A	-	4.15E-07	3.68E-01	6.52E-06	5437	
13	rs4055938	33191277	7.40E-01	N/A	13	rs3007264	1.05E+08	7.02E-01	N/A	-	4.16E-07	4.06E-01	2.69E-04	5438	
6	rs9367231	46121345	8.34E-01	CLIC5	4	rs4691073	1.57E+08	1.35E-01	GUCY1A3	-	4.16E-07	4.78E-01	7.92E-04	5439	
8	rs6996294	33557468	3.22E-01	RNF122	11	rs1073985	84316412	5.03E-01	DLG2	-	4.16E-07	N/A	N/A	5440	
2	rs509063	2.39E+08	4.33E-01	TRAF3IP1	10	rs17097516	1.2E+08	8.73E-01	N/A	-	4.16E-07	8.81E-01	3.78E-05	5441	
11	rs1390073	44167215	8.39E-01	EXT2	22	rs743803	47990194	1.82E-01	N/A	-	4.16E-07	1.56E-01	5.15E-07	5442	
3	rs741495	10392929	9.47E-01	ATP2B2	13	rs9549123	39702332	8.79E-01	N/A	-	4.16E-07	3.29E-01	4.12E-06	5443	
5	rs1035856	77952104	1.47E-01	LHFPL2	15	rs3743501	57378371	4.15E-01	MYO1E	-	4.16E-07	5.97E-01	2.31E-05	5444	
11	rs11234568	85553870	3.17E-01	N/A	15	rs4965499	97809029	6.31E-01	N/A	-	4.16E-07	8.16E-01	8.39E-05	5445	
10	rs4553272	21878386	2.93E-01	N/A	18	rs17440876	10284089	9.95E-01	N/A	-	4.16E-07	1.02E-01	7.47E-07	5446	
1	rs10465918	18574486	1.15E-01	IGSF21	2	rs6727258	67093532	1.27E-01	N/A	-	4.16E-07	5.08E-01	1.07E-05	5447	
1	rs10874870	94874053	8.20E-02	N/A	12	rs3850970	31674144	7.08E-01	C12orf72	-	4.16E-07	8.01E-01	3.14E-05	5448	
5	rs10054105	1.11E+08	6.53E-01	N/A	17	rs4968624	59783283	5.19E-01	N/A	-	4.16E-07	8.31E-01	1.50E-05	5449	
1	rs4660538	41664286	4.56E-02	N/A	4	rs7670222	1.83E+08	2.88E-02	ODZ3	-	4.17E-07	6.96E-03	1.29E-02	5450	
5	rs2407065	81511479	7.26E-01	ATG10	5	rs11749320	86161696	5.93E-01	N/A	-	4.17E-07	8.05E-01	1.65E-05	5451	
2	rs6746998	2.09E+08	1.89E-01	N/A	7	rs2214244	20527828	1.05E-01	N/A	-	4.17E-07	4.48E-01	4.18E-04	5452	
3	rs164207	32520695	5.95E-01	CMTM6	11	rs12575533	1.25E+08	7.62E-01	N/A	-	4.17E-07	3.65E-01	1.85E-05	5453	
1	rs10914802	34117836	7.49E-01	CSMD2	7	rs2299460	1.26E+08	1.23E-01	GRM8	-	4.17E-07	7.01E-01	1.15E-04	5454	
4	rs2687466	32940629	6.06E-01	N/A	11	rs2041466	22769111	4.37E-02	GAS2	-	4.17E-07	3.56E-01	6.16E-05	5455	
6	rs2503829	5317881	9.89E-01	FARS2	4	rs12647248	1.23E+08	8.83E-01	N/A	-	4.17E-07	3.82E-01	7.41E-06	5456	
3	rs3863998	1.5E+08	4.24E-01	N/A	11	rs720749	1.09E+08	5.35E-01	N/A	-	4.17E-07	5.43E-01	4.49E-06	5457	
12	rs7314097	95398409	3.97E-01	N/A	15	rs8036295	51651969	2.81E-01	WDR72	-	4.17E-07	5.93E-01	1.00E-05	5458	
3	rs9814549	1.01E+08	6.90E-01	C3orf26	8	rs11203826	16943551	5.66E-01	EFHA2	-	4.17E-07	8.79E-01	1.44E-05	5459	
10	rs10823136	69525369	7.41E-01	MYPN	10	rs1036268	1.33E+08	6.84E-01	N/A	-	4.17E-07	5.97E-01	7.15E-05	5460	
3	rs4688634	65983350	4.46E-01	MAGI1	10	rs920259	60066071	1.45E-01	BICC1	-	4.17E-07	7.27E-01	1.82E-04	5461	
11	rs10830242	87500294	2.65E-01	RAB38	16	rs12597569	47250843	3.40E-01	N/A	-	4.17E-07	7.92E-01	1.28E-05	5462	
11	rs10830242	87500294	2.65E-01	RAB38	16	rs1477135	47284324	3.40E-01	N/A	-	4.17E-07	7.92E-01	1.28E-05	5463	
1	rs12760232	1.85E+08	8.07E-01	PLA2G4A	15	rs2291627	38047140	1.15E-01	EIF2AK4	-	4.17E-07	8.88E-01	6.15E-05	5464	
5	rs13172078	1.22E+08	8.46E-01	N/A	14	rs3783862	88851462	1.07E-01	FOXN3	-	4.17E-07	2.97E-01	3.34E-06	5465	
8	rs9297384	1.08E+08	1.17E-01	OXR1	10	rs3850698	1.03E+08	5.56E-01	TLX1	-	4.17E-07	4.42E-01	3.03E-04	5466	
1	rs11581576	48742578	7.66E-01	N/A	2	rs4464317	69290795	6.47E-02	ANTXR1	-	4.18E-07	4.41E-01	1.40E-04	5467	
1	rs1343124	48745397	7.66E-01	N/A	2	rs4464317	69290795	6.47E-02	ANTXR1	-	4.18E-07	4.41E-01	1.40E-04	5468	
6	rs879882	31247431	7.61E-01	TCF19	6	rs7770216	31448590	1.71E-01	HLA-B	MHC	4.18E-07	9.59E-03	6.91E-08	5469	
10	rs12779841	87744801	1.49E-01	GRID1	14	rs12894609	94837133	9.14E-01	CLMN	-	4.18E-07	6.54E-01	7.29E-04	5470	
6	rs6911049	1.04E+08	1.87E-02	N/A	13	rs11618082	1.03E+08	8.06E-01	SLC10A2	-	4.18E-07	N/A	N/A	5471	
13	rs456683	21862207	1.40E-01	N/A	15	rs1979739	31737586	3.55E-02	RYR3	-	4.18E-07	1.68E-01	8.68E-04	5472	
14	rs1279323	32654910	1.91E-01	NPAS3	15	rs1346037	59925203	7.83E-01	VPS13C	-	4.18E-07	2.16E-01	8.68E-04	5473	
1	rs946131	2.31E+08	6.58E-01	N/A	7	rs157911	1.3E+08	5.87E-01	MIR29B1	-	4.18E-07	3.43E-01	1.60E-05	5474	
4	rs2322560	20235195	6.00E-02	SLIT2	17	rs11656549	69040691	4.86E-01	SDK2	-	4.18E-07	1.89E-02	1.32E-07	5475	
6	rs9378518	806084	4.97E-01	N/A	1	rs3850543	59426218	5.46E-01	N/A	-	4.18E-07	4.10E-01	4.20E-06	5476	
4	rs162182	1.88E+08	9.49E-01	FAT1	9	rs10820089	1.04E+08	9.60E-01	N/A	-	4.18E-07	5.51E-01	3.86E-04	5477	
3	rs4502548	24990545	3.81E-02	N/A	22	rs1557502	49360864	6.19E-01	CPT1B	-	4.18E-07	8.62E-01	1.71E-05	5478	
3	rs9855392	1.31E+08	5.23E-01	COL6A4P2	16	rs933724	73464892	2.14E-01	WDR59	-	4.18E-07	4.43E-01	4.93E-04	5479	
8	rs3102542	96919800	3.31E-01	N/A	10	rs10218882	1.26E+08	5.84E-02	N/A	-	4.18E-07	N/A	N/A	5480	
8	rs10441573	77488759	5.40E-01	N/A	17	rs11871749	14593162	8.66E-01	N/A	-	4.19E-07	7.88E-01	4.92E-05	5481	
1	rs2185811	2.04E+08	5.58E-01	ELK4	3	rs2874805	72721090	2.24E-01	N/A	-	4.19E-07	1.63E-01	8.76E-04	5482	
6	rs1102563	27268074	8.40E-01	N/A	14	rs2147105	53475815	5.59E-01	BMP4	MHC	4.19E-07	4.13E-01	1.34E-03	5483	
8	rs2317630	57748410	4.30E-01	N/A	15	rs2243932	59770219	5.70E-01	N/A	-	4.19E-07	4.50E-01	4.96E-04	5484	
2	rs4954799	1.39E+08	3.47E-01	N/A	16	rs1544357	19117111	1.90E-01	SYT17	-	4.19E-07	9.11E-01	4.42E-05	5485	
2	rs7575596	2.35E+08	2.26E-01	N/A	11	rs12293101	5292175	2.75E-01	OR51B2	-	4.19E-07	1.39E-01	2.90E-03	5486	

SNP A					SNP B						Interaction P			Ranking	Cluster in top 100 interactions
CHR	SNP	Location	gle locus P va	Gene	CHR	SNP	Location	Single locus P valu	Gene	IHC region	Stage 1	Stage 2	Combined		
1	rs1749778	58236351	6.32E-02	DAB1	8	rs4734606	1.03E+08	3.01E-01	NCALD	-	4.19E-07	1.26E-01	9.52E-04	5487	
14	rs989909	74157997	7.17E-01	LTBP2	22	rs3747152	29850338	4.54E-01	INPP5J	-	4.19E-07	2.08E-01	3.09E-06	5488	
5	rs1158196	58017591	5.70E-01	RAB3C	14	rs3829948	92186579	5.37E-01	RIN3	-	4.19E-07	3.40E-01	8.45E-06	5489	
2	rs17442753	19509571	6.90E-01	N/A	12	rs1683163	25124885	2.18E-01	LRMP	-	4.19E-07	6.54E-03	1.79E-08	5490	
6	rs2273006	34959112	4.80E-01	ANKS1A	9	rs287572	31994713	1.35E-01	N/A	MHC	4.19E-07	1.49E-01	1.14E-03	5491	
6	rs2273006	34959112	4.80E-01	ANKS1A	9	rs287573	31994571	1.35E-01	N/A	MHC	4.19E-07	1.49E-01	1.14E-03	5492	
6	rs2273006	34959112	4.80E-01	ANKS1A	9	rs407791	32012207	1.35E-01	N/A	MHC	4.19E-07	1.49E-01	1.14E-03	5493	
6	rs2273006	34959112	4.80E-01	ANKS1A	9	rs453809	32006697	1.35E-01	N/A	MHC	4.19E-07	N/A	N/A	5494	
7	rs6945759	1.58E+08	2.35E-01	PTPRN2	11	rs10769176	45939961	7.97E-01	PHF21A	-	4.20E-07	N/A	N/A	5495	
1	rs1886394	1.55E+08	4.87E-01	N/A	20	rs6087095	9923888	5.53E-01	N/A	-	4.20E-07	4.71E-01	2.23E-03	5496	
5	rs11748629	88832536	2.26E-01	N/A	8	rs4875820	2354297	1.95E-01	N/A	-	4.20E-07	5.07E-01	2.11E-04	5497	
2	rs10165036	5634870	7.59E-01	N/A	10	rs12261821	79847770	1.17E-01	N/A	-	4.20E-07	7.14E-01	8.95E-05	5498	
2	rs6433791	1.8E+08	8.19E-01	N/A	18	rs2617939	23552981	7.21E-01	N/A	-	4.20E-07	4.86E-01	6.48E-04	5499	
8	rs4237068	21266056	4.28E-01	N/A	11	rs922774	1.09E+08	2.56E-01	N/A	-	4.20E-07	9.42E-01	1.25E-04	5500	
5	rs4916818	90074285	8.68E-01	GPR98	17	rs10491216	3267156	7.29E-01	OR1E1	-	4.20E-07	6.56E-01	1.27E-06	5502	
7	rs6979066	82260873	1.50E-01	PCLO	16	rs1861315	50859323	5.32E-01	N/A	-	4.20E-07	7.10E-03	1.08E-08	5503	
4	rs12648158	1.89E+08	6.61E-01	N/A	8	rs13250449	10475278	3.30E-01	N/A	-	4.20E-07	7.70E-01	7.69E-05	5501	
9	rs1360523	25489528	7.55E-01	N/A	16	rs2127065	7141423	3.49E-01	RBFOX1	-	4.20E-07	5.57E-04	2.69E-09	5505	
7	rs17167656	13927831	5.60E-01	ETV1	7	rs6974985	1.49E+08	6.20E-01	KRBA1	-	4.20E-07	7.28E-01	5.91E-05	5504	
1	rs11265455	1.59E+08	8.55E-01	SLAMF1	15	rs2959930	70385668	6.97E-01	BRUNOL6	-	4.21E-07	5.09E-02	1.86E-07	5506	
3	rs4858553	23896118	2.37E-01	UBE2E1	10	rs2762637	10134843	2.37E-01	N/A	-	4.21E-07	9.92E-01	2.09E-05	5507	
1	rs10915971	2.25E+08	2.28E-01	LIN9	16	rs1420591	47607079	3.28E-02	N/A	-	4.21E-07	5.67E-01	8.83E-04	5508	
3	rs2135511	43343543	3.87E-01	SNRK	17	rs4078429	75949967	1.87E-01	RNF213	-	4.21E-07	9.46E-01	8.38E-05	5509	
4	rs17351704	83864655	7.03E-01	SCD5	12	rs3819526	2306783	4.68E-01	CACN/A1C	-	4.21E-07	8.17E-01	4.71E-05	5510	
6	rs3020375	1.52E+08	6.30E-01	ESR1	3	rs2568911	1.96E+08	3.66E-01	N/A	-	4.21E-07	N/A	N/A	5511	
6	rs6900214	67144853	4.90E-01	N/A	16	rs17697705	64460451	4.63E-01	N/A	-	4.21E-07	8.13E-01	4.67E-05	5512	
2	rs6437294	2.41E+08	1.05E-02	OTOS	5	rs9293322	82220254	3.84E-01	N/A	-	4.22E-07	4.13E-01	4.74E-04	5513	
3	rs6767029	68559900	5.42E-01	FAM19A1	4	rs10018782	1.87E+08	7.83E-01	SORBS2	-	4.22E-07	5.33E-01	7.11E-06	5514	
4	rs11936270	1.89E+08	1.69E-01	N/A	11	rs10769958	8771691	8.33E-01	ST5	-	4.22E-07	5.86E-01	4.33E-05	5515	
5	rs3822476	77749577	4.95E-01	SCAMP1	11	rs1800849	73397813	6.34E-01	UCP3	-	4.22E-07	6.97E-01	2.79E-05	5516	
3	rs2228291	1.86E+08	7.82E-01	THPO	15	rs4778036	90537185	6.23E-01	N/A	-	4.22E-07	7.74E-01	8.67E-05	5517	
1	rs9730586	74734405	7.35E-01	TNNI3K	1	rs959163	1.02E+08	2.53E-01	N/A	-	4.22E-07	8.51E-01	4.38E-04	5518	
1	rs1963273	1.7E+08	3.68E-01	FMO4	16	rs208636	22822746	9.23E-01	HS3ST2	-	4.22E-07	6.49E-01	1.20E-04	5519	
3	rs168150	1.13E+08	1.06E-01	TMPRSS7	15	rs7183545	36762771	6.64E-01	C15orf53	-	4.22E-07	1.33E-01	1.77E-03	5520	
6	rs9380215	31157634	4.29E-02	N/A	6	rs3131003	31201461	6.51E-01	C6orf15	MHC	4.22E-07	5.32E-01	2.84E-05	5521	
11	rs1441505	25816359	2.82E-01	N/A	22	rs9616022	45267548	9.31E-01	CELSR1	-	4.22E-07	1.99E-01	6.84E-03	5522	
8	rs6578039	1.41E+08	8.38E-03	KCNK9	16	rs7192675	83101985	1.60E-01	KIAA1609	-	4.22E-07	3.47E-01	4.92E-06	5523	
6	rs9397717	1.55E+08	6.24E-01	CNKSR3	12	rs1635135	1.12E+08	2.15E-01	OAS2	-	4.22E-07	6.36E-01	8.84E-05	5524	
10	rs11257069	11452059	7.55E-01	N/A	11	rs10791774	1.05E+08	8.16E-01	GRIA4	-	4.22E-07	9.00E-01	1.55E-04	5525	
10	rs11257069	11452059	7.55E-01	N/A	11	rs11226848	1.05E+08	8.16E-01	GRIA4	-	4.22E-07	9.00E-01	1.55E-04	5526	
6	rs10946578	22854096	8.77E-01	N/A	4	rs6815581	1.39E+08	1.52E-01	N/A	-	4.22E-07	4.09E-01	5.81E-04	5527	
1	rs804136	15006255	8.36E-01	KIAA1026	3	rs4305421	1.34E+08	3.51E-02	N/A	-	4.22E-07	5.11E-01	2.30E-05	5528	
4	rs4699831	96185917	7.16E-01	BMPR1B	15	rs1145170	81022977	5.17E-01	CPEB1	-	4.23E-07	3.11E-01	3.22E-06	5529	
9	rs1491124	1.08E+08	9.07E-01	N/A	10	rs12573087	1.01E+08	1.21E-01	N/A	-	4.23E-07	4.69E-02	3.43E-07	5530	
6	rs9380215	31157634	4.29E-02	N/A	6	rs3130453	31232828	5.90E-01	TCF19	MHC	4.23E-07	8.70E-01	1.19E-04	5531	
1	rs1105489	2.46E+08	4.50E-01	C1orf150	2	rs1405343	76318008	3.10E-01	N/A	-	4.23E-07	4.48E-02	9.68E-03	5532	
12	rs7309071	81138807	1.07E-01	N/A	20	rs2904362	51580068	1.25E-01	N/A	-	4.23E-07	8.07E-01	2.74E-05	5533	
6	rs9362180	86067798	7.62E-01	N/A	9	rs11265909	91267966	3.52E-01	SEMA4D	-	4.23E-07	6.41E-01	2.03E-04	5534	
17	rs4792542	14529207	2.86E-01	N/A	22	rs2845393	15905970	3.40E-01	CECR7	-	4.23E-07	3.90E-01	1.87E-03	5535	
8	rs16912432	85103642	8.81E-01	N/A	18	rs4890506	40873376	8.96E-01	SETBP1	-	4.23E-07	6.63E-01	4.59E-05	5536	
8	rs6991736	85116394	8.81E-01	N/A	18	rs4890506	40873376	8.96E-01	SETBP1	-	4.23E-07	6.63E-01	4.59E-05	5537	
11	rs7937641	1.13E+08	9.99E-01	N/A	18	rs2027742	73210278	5.31E-01	N/A	-	4.23E-07	7.26E-01	7.71E-06	5538	
6	rs4594925	1.69E+08	8.29E-01	N/A	5	rs4235460	65724797	4.53E-01	N/A	-	4.23E-07	5.45E-01	3.93E-05	5539	
11	rs1790474	1.19E+08	4.13E-01	N/A	15	rs11634608	70042772	6.68E-01	MYO9A	-	4.23E-07	9.86E-01	1.35E-04	5540	
7	rs2108554	12549605	4.82E-02	N/A	7	rs7794764	85088091	5.62E-01	N/A	-	4.23E-07	3.10E-01	5.81E-06	5541	
7	rs6460282	65328681	1.27E-01	TPST1	7	rs1018995	1.47E+08	1.22E-01	CNTN/AP2	-	4.23E-07	8.98E-01	3.88E-05	5542	
14	rs387419	55603832	3.08E-01	N/A	20	rs6024282	53674348	3.44E-01	N/A	-	4.23E-07	1.71E-01	8.04E-06	5543	
6	rs1123969	1.55E+08	1.18E-01	N/A	1	rs1999506	2.35E+08	4.26E-01	EDARADD	-	4.23E-07	2.28E-01	6.37E-04	5544	
5	rs359457	1.73E+08	6.98E-01	N/A	8	rs4077788	65933222	6.61E-01	N/A	-	4.23E-07	4.22E-01	2.92E-05	5545	
7	rs7800092	1.31E+08	9.76E-02	N/A	16	rs6500343	49448844	5.45E-01	N/A	-	4.23E-07	6.94E-01	1.21E-04	5546	
15	rs8041357	72656491	1.21E-01	ARID3B	18	rs2187116	68697667	9.09E-01	NETO1	-	4.23E-07	8.49E-01	9.45E-05	5547	
10	rs4980259	1.25E+08	5.34E-01	N/A	17	rs2191176	14745432	2.59E-01	N/A	-	4.23E-07	6.34E-01	6.49E-05	5548	
7	rs7803705	77932576	8.74E-01	MAGI2	20	rs12625612	42279088	8.94E-01	C20orf111	-	4.23E-07	7.81E-01	1.68E-04	5549	
7	rs7803705	77932576	8.74E-01	MAGI2	20	rs4812799	42280108	8.94E-01	C20orf111	-	4.23E-07	7.81E-01	1.68E-04	5550	
4	rs4240307	1.19E+08	7.96E-01	N/A	5	rs2114961	1.35E+08	3.60E-01	N/A	-	4.24E-07	3.09E-01	2.92E-04	5551	
1	rs12027754	63362033	5.97E-01	N/A	11	rs11041390	7432565	6.89E-01	SYT9	-	4.24E-07	9.85E-01	1.29E-05	5552	
6	rs9376770	1.44E+08	7.64E-01	PHACTR2	13	rs12430792	45363200	7.64E-01	N/A	-	4.24E-07	5.03E-01	8.69E-06	5553	
10	rs7903114	1.25E+08	8.27E-01	N/A	21	rs2826601	21193520	8.89E-01	N/A	-	4.24E-07	4.76E-01	1.02E-05	5554	
15	rs1896799	82038855	3.04E-01	SH3GL3	20	rs6081456	18907491	2.48E-02	N/A	-	4.24E-07	8.11E-01	2.41E-04	5555	
6	rs2745626	1411547	5.37E-01	N/A	8	rs2014238	76461908	3.07E-01	N/A	-	4.24E-07	5.04E-01	8.45E-06	5556	
2	rs904162	47363828	4.44E-01	N/A	7	rs1319470	1.17E+08	3.49E-01	N/A	-	4.24E-07	8.94E-02	6.07E-07	5557	
4	rs7673610	76679560	3.80E-02	THAP6	16	rs2795571	6435492	9.03E-01	A2BP1	-	4.24E-07	9.11E-02	1.54E-03	5558	
5	rs10515015	65974608	2.26E-01	MAST4	10	rs11197219	84837248	9.50E-01	N/A	-	4.24E-07	6.37E-01	1.46E-04	5559	
6	rs906658	54307077	9.23E-01	TIN/AG	11	rs651861	78359137	4.97E-01	ODZ4	-	4.24E-07	1.97E-01	8.01E-04	5560	
6	rs9364407	1.68E+08	1.38E-01	N/A	4	rs6552787	1.86E+08	3.52E-01	N/A	-	4.24E-07	6.27E-01	1.88E-04	5561	
2	rs2577999	1.83E+08	4.99E-01	N/A	12	rs1846035	82545653	9.72E-01	N/A	-	4.25E-07	5.01E-01	2.30E-05	5562	
2	rs2971890	1.83E+08	4.99E-01	N/A	12	rs1846035	82545653	9.72E-01	N/A	-	4.25E-07	5.01E-01	2.30E-05	5563	
2	rs7603696	35427381	6.79E-01	N/A	11	rs835764	44868055	4.14E-01	TSPAN18	-	4.25E-07	2.56E-01	1.15E-03	5564	
8	rs10086120	96860951	6.63E-01	N/A	15	rs4774982	55853182	9.82E-01	N/A	-	4.25E-07	1.99E-03	8.56E-09	5565	
2	rs308003	6753810	2.84E-01	N/A	5	rs2304066	1.46E+08	2.71E-01	N/A	-	4.25E-07	5.37E-01	3.58E-04	5566	
5	rs976207	1.59E+08	7.34E-02	N/A	9	rs1953003	28295521	3.86E-01	LINGO2	-	4.25E-07	5.61E-01	2.05E-04	5567	
5	rs156319	33434957	4.24E-01	N/A	7	rs13237471	1.31E+08	1.84E-01	MKLN1	-	4.25E-07	7.13E-01	1.96E-04	5568	
6	rs2523849	31133030	6.18E-01	HCG22	6	rs9380215	31157634	4.29E-02	N/A	MHC	4.25E-07	8.51E-01	5.61E-05	5569	
7	rs2365695	79158672	2.79E-01	N/A	10	rs12221133	12293603	9.93E-01	NUDT5	-	4.25E-07	7.11E-02	2.03E-03	5570	
2	rs1454545	2.21E+08	8.85E-01	N/A	12	rs1909327	61960235	6.38E-02	N/A	-	4.25E-07	3.49E-01	1.99E-03	5571	
1	rs3890764	61734541	7.49E-01	N/A	8	rs7842199	77794324	2.30E-01	ZFHX4	-	4.25E-07	9.08E-01	1.58E-05	5572	
2	rs1109426	1.21E+08	4.16E-01	N/A	3	rs882788	1.89E+08	8.95E-01	N/A	-	4.25E-07	1.11E-01	3.48E-03	5573	
3	rs12152230	35728583	4.12E-01	ARPP-21	9	rs3802347	1.33E+08	8.87E-01	ABL1	-	4.25E-07	3.11E-01	7.30E-04	5574	
6	rs1570258	93125026	3.84E-01	N/A	15	rs6493549	50315485	3.84E-01	MYO5C	-	4.25E-07	4.66E-01	9.26E-04	5575	
4	rs7661550	46581758	2.94E-01	COX7B2	17	rs12601473	4272789	1.83E-01	SPNS3	-	4.25E-07	5.36E-01	1.62E-04	5576	
6	rs2235384	11884617	1.72E-01	C6orf105	11	rs12419062	35206246	3.94E-01	CD44	-	4.26E-07	3.71E-02	9.96E-03	5577	
1	rs1333065	1.59E+08	5.97E-01	LY9	13	rs261475	81237360	3.99E-01	N/A	-	4.26E-07	8.56E-01	6.08E-05	5578	
5	rs4585491	91537859	2.39E-02	N/A	9	rs10819317	1.3E+08	9.82E-02	ST6GALN/AC4	-	4.26E-07	5.89E-01	7.88E-05	5579	
5	rs6579767	1.49E+08	3.01E-01	HMGXB3	10	rs2505366	32699393	6.73E-01	N/A	-	4.26E-07	7.85E-03	3.06E-08	5580	
9	rs1155556	1.19E+08	8.36E-01	ASTN2	17	rs544198	34572555	1.93E-03	PLXDC1	-	4.26E-07	2.32E-01	5.72E-04	5581	
2	rs6735868	1.03E+08	7.37E-01	N/A	4	rs10470823	26549785	9.62E-01	STIM2	-	4.26E-07	5.28E-01	5.84E-05	5582	
6	rs9450765	88401767	3.03E-01	ORC3L	1	rs6687674	27409688	9.67E-01	N/A	-	4.26E-07	6.12E-01	3.18E-04	5583	
4	rs17050402	1.4E+08	7.16E-01	N/A	10	rs10794204	1.27E+08	2.72E-01	N/A	-	4.26E-07	8.69E-01	1.03E-04	5584	
4	rs17050402	1.4E+08	7.16E-01	N/A	10	rs10901876	1.27E+08	2.72E-01	N/A	-	4.26E-07	8.69E-01	1.03E-04	5585	
3	rs6550901	24788964	7.02E-01	N/A	11	rs2166706	92331180	4.72E-01	MTNR1B	-	4.26E-07	8.81E-01	2.00E-05	5586	
3	rs9828692	1.35E+08	5.16E-02	BFSP2	4	rs2321859	1.42E+08	1.99E-01	RNF150	-	4.26E-07	8.76E-01	5.71E-05	5587	
6	rs9322188	1.5E+08	4.39E-02	KATN/A1	4	rs1842534	34244104	3.80E-01	N/A	-	4.26E-07	8.85E-02	1.03E-03	5588	
1	rs2236591	2.27E+08	1.27E-01	N/A	8	rs4737264	56273876	5.95E-01	XKR4	-	4.26E-07	5.89E-01	4.35E-05	5589	
2	rs12477036	2.3E+08	4.94E-01	PID1	3	rs6442831	3795517	9.99E-02	N/A	-	4.26E-07	5.41E-02	8.24E-07	5590	
6	rs2130357	27994809	6.14E-01	OR2B2	1	rs11184182	1.05E+08	4.73E-01	N/A	MHC	4.26E-07	1.53E-01	7.50E-06	5591	
2	rs7581202	1.99E+08	8.77E-01	PLCL1	7	rs12699758	15930763	7.54E-01	N/A	-	4.26E-07	4.27E-01	9.65E-05	5592	
2	rs1378729	1.75E+08	9.00E-01	CHN1	5	rs6898404	83237939	4.08E-01	N/A	-	4.26E-07	5.19E-01	3.38E-06	5593	
2	rs4438515	3082104	8.90E-01	N/A	10	rs4980031	80561637	5.52E-02	ZMIZ1	-	4.26E-07	8.26E-01	2.79E-05	5594	
2	rs10171249	54346912	9.82E-01	ACYP2	2	rs4330055	1.43E+08	2.91E-01	N/A	-	4.27E-07	1.65E-01	8.63E-04	5595	
1	rs4847269	94189807	4.16E-01	N/A	4	rs7668386	1.67E+08	2.22E-01	N/A	-	4.27E-07	9.06E-01	1.69E-04	5596	
2	rs3792074	2.31E+08	8.70E-01	CAB39	12	rs7139067	95571241	4.31E-01	N/A	-	4.27E-07	4.03E-01	1.52E-04	5597	
2	rs1598024	1.23E+08	5.11E-01	N/A	14	rs10144310	39591908	9.54E-01	N/A	-	4.27E-07	4.31E-01	2.56E-04	5598	

SNP A					SNP B						Interaction P			Ranking	Cluster in top 100 interactions
CHR	SNP	Location	gle locus P va	Gene	CHR	SNP	Location	Single locus P valu	Gene	MHC region	Stage 1	Stage 2	Combined		
3	rs1460118	53684891	4.45E-01	CACN/A1D	4	rs966299	1.82E+08	4.39E-01	N/A	-	4.27E-07	8.77E-01	1.09E-04	5599	
3	rs7618109	13684182	4.15E-01	LOC285375	5	rs3776319	1.42E+08	4.71E-01	ARHGAP26	-	4.27E-07	5.75E-01	4.66E-05	5600	
12	rs10772693	13622511	3.81E-01	GRIN2B	18	rs11083322	24938463	6.92E-01	N/A	-	4.27E-07	8.10E-01	5.41E-05	5601	
2	rs2276607	19932861	5.84E-01	N/A	3	rs4684917	13219384	1.75E-01	N/A	-	4.27E-07	8.37E-01	7.48E-05	5602	
12	rs10845886	14161348	4.46E-01	N/A	17	rs17810148	9658805	1.38E-01	GLP2R	-	4.27E-07	1.88E-01	2.39E-05	5603	
11	rs7106847	19943938	7.51E-01	N/AV2	16	rs4782022	17211055	1.10E-02	XYLT1	-	4.27E-07	9.04E-01	1.71E-04	5604	
7	rs4721181	13066067	1.03E-01	N/A	19	rs1859955	62514181	6.47E-01	ZNF460	-	4.27E-07	5.96E-01	1.24E-05	5605	
14	rs10220359	35196872	6.90E-01	RALGAPA1	19	rs36259	8232904	7.47E-01	LASS4	-	4.27E-07	9.29E-02	5.98E-07	5606	
9	rs2806096	1.2E+08	2.76E-01	N/A	20	rs680379	12917400	2.86E-01	N/A	-	4.27E-07	1.57E-01	1.43E-06	5607	
4	rs4558821	1.57E+08	4.26E-01	N/A	9	rs10967899	27325339	9.80E-01	MOBKL2B	-	4.27E-07	3.00E-01	1.35E-03	5608	
5	rs6555834	1.68E+08	1.49E-01	SLIT3	10	rs592818	27808006	5.02E-01	N/A	-	4.27E-07	8.78E-01	3.38E-05	5609	
5	rs171667	1.79E+08	4.22E-01	ADAMTS2	9	rs1545909	7645364	9.51E-01	N/A	-	4.27E-07	7.77E-01	1.56E-05	5610	
2	rs3100232	29210173	4.05E-01	CLIP4	17	rs9901291	17482475	4.48E-03	N/A	-	4.28E-07	3.84E-01	3.10E-06	5611	
2	rs1829731	19525417	6.93E-01	N/A	12	rs10842458	25118569	1.92E-01	LRMP	-	4.28E-07	2.15E-02	8.08E-08	5612	
1	rs1736557	1.69E+08	9.58E-01	FMO3	11	rs4598653	28505842	1.13E-01	N/A	-	4.28E-07	6.02E-01	2.21E-05	5613	
12	rs11107828	93889967	1.48E-01	NDUFA12	20	rs6017995	44770445	6.23E-01	SLC2A10	-	4.28E-07	N/A	N/A	5614	
4	rs1449622	61688491	2.49E-01	N/A	18	rs12458846	69206580	6.29E-01	N/A	-	4.28E-07	3.60E-01	4.77E-06	5615	
12	rs6539598	80814114	3.92E-01	N/A	17	rs7210733	27591452	4.04E-01	RHOT1	-	4.28E-07	8.12E-03	1.22E-08	5616	
15	rs7170993	53475261	5.43E-01	CCPG1	20	rs4401268	11893421	4.17E-01	N/A	-	4.28E-07	4.28E-01	9.41E-04	5617	
11	rs2729385	57019569	3.46E-01	SLC43A1	21	rs1475584	27142601	2.58E-01	ADAMTS1	-	4.28E-07	8.22E-01	4.36E-05	5618	
9	rs10813616	31540664	4.48E-01	N/A	15	rs2279168	83878193	1.90E-01	AKAP13	-	4.28E-07	1.97E-03	8.42E-09	5619	
3	rs9849797	1.45E+08	4.69E-02	SLC9A9	17	rs9889335	66626741	5.16E-01	N/A	-	4.28E-07	1.74E-01	4.54E-06	5620	
6	rs2523849	31133030	6.18E-01	HCG22	6	rs4947296	31166157	4.31E-02	N/A	MHC	4.28E-07	4.97E-01	1.17E-05	5621	
3	rs7429119	1.86E+08	2.03E-01	N/A	13	rs9539014	60502737	5.08E-01	N/A	-	4.28E-07	1.44E-01	2.14E-03	5622	
3	rs9818538	1.86E+08	2.03E-01	N/A	13	rs9539014	60502737	5.08E-01	N/A	-	4.28E-07	1.44E-01	2.14E-03	5623	
3	rs4678717	34890112	2.60E-01	N/A	8	rs4925813	1.46E+08	3.21E-01	KIAA1688	-	4.28E-07	6.23E-01	5.28E-05	5624	
3	rs4831129	1.17E+08	2.46E-01	LSAMP	8	rs6984840	10169975	9.13E-01	MSRA	-	4.28E-07	6.99E-01	2.68E-04	5625	
12	rs10784919	70156530	7.16E-01	LGR5	18	rs16975208	37538035	7.09E-02	N/A	-	4.28E-07	N/A	N/A	5626	
10	rs1992293	80261371	7.35E-01	N/A	18	rs721526	24049212	4.61E-01	N/A	-	4.29E-07	5.02E-02	1.11E-02	5627	
4	rs2165387	80710844	6.07E-02	N/A	7	rs10272997	70402452	5.44E-01	WBSCR17	-	4.29E-07	1.67E-01	3.07E-06	5628	
8	rs7011466	25875922	7.61E-01	EBF2	19	rs4932844	23214035	7.49E-01	N/A	-	4.29E-07	8.04E-01	2.10E-04	5629	
8	rs7011466	25875922	7.61E-01	EBF2	19	rs6511422	23184318	7.49E-01	N/A	-	4.29E-07	8.04E-01	2.10E-04	5630	
8	rs7011466	25875922	7.61E-01	EBF2	19	rs7254300	23222276	7.49E-01	N/A	-	4.29E-07	8.04E-01	2.10E-04	5631	
6	rs6458486	46115804	9.72E-01	CLIC5	7	rs11772608	1.58E+08	8.38E-01	PTPRN2	-	4.29E-07	7.84E-01	2.35E-05	5632	
6	rs716434	1.56E+08	9.74E-01	N/A	14	rs181484	68427927	4.63E-01	ACTN1	-	4.29E-07	4.92E-01	2.17E-04	5633	
5	rs1432925	1.66E+08	4.81E-01	N/A	8	rs10504930	94813989	5.77E-01	FAM92A1	-	4.29E-07	5.02E-01	2.79E-05	5634	
5	rs1432925	1.66E+08	4.81E-01	N/A	8	rs2914944	94793219	5.77E-01	N/A	-	4.29E-07	5.02E-01	2.79E-05	5635	
5	rs1432925	1.66E+08	4.81E-01	N/A	8	rs6471393	94784467	5.77E-01	N/A	-	4.29E-07	5.02E-01	2.79E-05	5636	
6	rs9371389	1.56E+08	9.74E-01	N/A	14	rs181484	68427927	4.63E-01	ACTN1	-	4.29E-07	5.35E-01	1.85E-04	5637	
8	rs2697675	91002125	8.04E-01	NBN	8	rs3134245	96665925	6.55E-01	N/A	-	4.29E-07	5.41E-01	3.49E-05	5638	
6	rs9383633	1.5E+08	4.17E-01	RAET1K	9	rs2900229	1.28E+08	5.04E-01	MAPKAP1	-	4.29E-07	1.66E-01	6.92E-04	5639	
4	rs6850000	1.67E+08	1.89E-02	CPE	5	rs12517451	79955829	3.08E-01	DHFR	-	4.29E-07	4.13E-01	3.51E-05	5640	
8	rs2616496	89130414	4.68E-01	MMP16	8	rs6577679	1.37E+08	7.60E-01	N/A	-	4.29E-07	3.64E-01	1.57E-03	5641	
4	rs6827817	88550011	8.95E-01	HSD17B11	12	rs11063880	5806526	7.05E-02	ANO2	-	4.29E-07	6.23E-01	6.15E-04	5642	
6	rs2274090	37928946	7.70E-01	ZFAND3	18	rs11877899	73733998	4.97E-01	N/A	-	4.29E-07	9.85E-01	1.29E-05	5643	
9	rs10974873	4933454	4.63E-01	N/A	16	rs12932445	71627389	2.79E-01	ZFHX3	-	4.30E-07	9.20E-03	1.19E-08	5644	
8	rs12543466	1.09E+08	1.08E-01	N/A	8	rs10105826	1.32E+08	8.22E-01	N/A	-	4.30E-07	9.05E-02	7.37E-07	5645	
5	rs7707754	79474682	6.36E-01	SERINC5	12	rs11182800	43712783	9.74E-01	DBX2	-	4.30E-07	9.09E-01	1.28E-05	5646	
5	rs6896456	1.35E+08	3.00E-01	N/A	8	rs10102337	22704701	5.17E-01	PEBP4	-	4.30E-07	3.21E-01	5.20E-06	5647	
9	rs12236072	1.21E+08	4.01E-01	N/A	16	rs2075738	76619660	4.31E-01	CLEC3A	-	4.30E-07	6.60E-01	1.16E-05	5648	
9	rs17195438	79320366	2.00E-01	GN/A14	14	rs10142258	72879970	5.50E-01	NUMB	-	4.30E-07	8.40E-02	3.82E-03	5649	
9	rs17195438	79320366	2.00E-01	GN/A14	14	rs10483853	72826052	5.50E-01	NUMB	-	4.30E-07	9.88E-02	3.27E-03	5650	
9	rs17195438	79320366	2.00E-01	GN/A14	14	rs8013402	72818251	5.50E-01	NUMB	-	4.30E-07	9.88E-02	3.27E-03	5651	
2	rs1545224	88204931	6.60E-02	FABP1	4	rs10517394	57966443	2.47E-01	N/A	-	4.30E-07	3.27E-01	1.21E-03	5652	
5	rs619146	1.13E+08	6.85E-01	MCC	19	rs4805602	35897140	2.21E-01	N/A	-	4.30E-07	6.38E-01	1.99E-04	5653	
1	rs17115070	96628806	8.62E-01	N/A	5	rs2372183	67233711	7.49E-01	N/A	-	4.30E-07	2.29E-01	1.54E-03	5654	
6	rs2745443	1.33E+08	6.67E-01	VNN1	11	rs7940244	70899222	9.24E-01	N/ADSYN1	-	4.30E-07	4.07E-01	1.22E-03	5655	
4	rs1909805	1.32E+08	7.22E-01	N/A	8	rs372976	82634163	4.55E-01	N/A	-	4.30E-07	5.86E-01	9.78E-06	5656	
4	rs1909805	1.32E+08	7.22E-01	N/A	8	rs396738	82634100	4.55E-01	N/A	-	4.30E-07	5.86E-01	9.78E-06	5657	
2	rs4556998	1.06E+08	4.51E-01	NCK2	19	rs3810342	43599425	1.44E-01	RYR1	-	4.30E-07	1.46E-01	2.63E-06	5658	
9	rs4978465	1.14E+08	1.98E-01	N/A	12	rs4764104	9981700	2.62E-01	CLEC2A	-	4.31E-07	1.71E-01	2.75E-03	5659	
1	rs766910	22745274	7.03E-01	ZBTB40	10	rs11004362	55972380	8.45E-01	PCDH15	-	4.31E-07	1.97E-01	1.05E-03	5660	
6	rs9347476	1.61E+08	8.27E-03	MAP3K4	17	rs224546	3419621	6.36E-01	TRPV3	-	4.31E-07	2.26E-01	1.16E-03	5661	
8	rs1805860	91092043	5.65E-01	DECR1	12	rs7300317	50918840	7.41E-01	LOC283404	-	4.31E-07	6.29E-01	1.62E-05	5662	
1	rs6424262	2.31E+08	4.25E-01	PCNXL2	4	rs13119882	1.34E+08	9.80E-01	N/A	-	4.31E-07	9.29E-01	8.44E-05	5663	
5	rs11134509	1.68E+08	4.65E-01	WWC1	9	rs945464	9176346	4.40E-01	PTPRD	-	4.31E-07	5.41E-02	2.78E-07	5664	
8	rs4384011	1.2E+08	2.67E-01	N/A	13	rs3014939	45068610	1.62E-02	FAM194B	-	4.31E-07	6.72E-02	4.92E-03	5665	
8	rs4384011	1.2E+08	2.67E-01	N/A	13	rs3014940	45070829	1.62E-02	FAM194B	-	4.31E-07	6.72E-02	4.92E-03	5666	
7	rs7806789	1.21E+08	3.76E-01	N/A	13	rs16970118	1.07E+08	2.42E-01	FAM155A	-	4.31E-07	2.52E-01	1.64E-05	5667	
1	rs9435130	9190514	6.08E-01	N/A	11	rs518427	59240269	8.26E-01	OR10V1	-	4.31E-07	6.76E-01	2.01E-05	5668	
2	rs2889323	1.04E+08	3.76E-01	LOC150568	7	rs82	11571857	3.54E-01	THSD7A	-	4.31E-07	8.75E-01	2.86E-05	5669	
7	rs2062403	68146581	5.93E-01	N/A	7	rs6958981	69063930	1.96E-01	AUTS2	-	4.31E-07	5.84E-01	6.88E-05	5670	
2	rs4622658	1.3E+08	2.51E-01	N/A	5	rs10067065	1.24E+08	5.91E-01	N/A	-	4.31E-07	3.19E-02	4.42E-07	5671	
2	rs4355062	1.3E+08	2.17E-01	N/A	8	rs2167316	58669656	5.81E-01	N/A	-	4.31E-07	2.85E-01	1.51E-06	5672	
15	rs2353499	21757903	3.97E-01	N/A	17	rs4794737	52855906	3.47E-01	MSI2	-	4.31E-07	9.44E-01	1.64E-04	5673	
2	rs2298948	75780073	9.45E-01	C2orf3	3	rs9870165	1.28E+08	5.57E-01	PLXN/A1	-	4.32E-07	8.57E-02	1.57E-07	5674	
8	rs4354357	1.39E+08	1.25E-01	FAM135B	19	rs1793978	50529109	2.65E-01	KLC3	-	4.32E-07	2.94E-01	6.29E-06	5675	
9	rs7849611	1.21E+08	1.09E-02	DBC1	17	rs9905064	51522603	4.66E-01	N/A	-	4.32E-07	4.09E-01	6.59E-06	5676	
19	rs8110509	35673479	8.80E-01	ZNF536	22	rs6009503	47918047	1.89E-01	N/A	-	4.32E-07	7.39E-01	1.23E-04	5677	
8	rs6599528	1.46E+08	8.55E-01	CPSF1	10	rs1471824	72405746	7.51E-01	N/A	-	4.32E-07	8.02E-01	4.37E-04	5678	
6	rs1564601	1.49E+08	8.21E-01	UST	10	rs6482268	23685520	6.10E-01	C10orf67	-	4.32E-07	5.95E-01	4.66E-06	5679	
2	rs7570764	1.34E+08	8.98E-01	N/A	12	rs1451904	1.29E+08	8.19E-01	TMEM132D	-	4.32E-07	3.42E-01	1.07E-05	5680	
6	rs1125513	5722130	2.93E-01	FARS2	2	rs6715170	42941808	4.89E-01	N/A	-	4.32E-07	5.92E-01	3.28E-05	5681	
6	rs11756073	1.7E+08	1.82E-01	N/A	4	rs1391980	1.29E+08	2.52E-01	PGRMC2	-	4.32E-07	7.57E-01	3.12E-05	5682	
14	rs11627558	25161910	9.62E-01	N/A	20	rs6086493	8475885	6.74E-01	PLCB1	-	4.32E-07	3.02E-01	1.50E-05	5683	
7	rs1476452	87920309	1.77E-02	N/A	9	rs2281785	2171854	4.47E-01	SMARCA2	-	4.32E-07	3.07E-01	1.97E-06	5684	
5	rs2731818	94821797	5.99E-01	FAM81B	9	rs6476554	3637685	6.61E-01	N/A	-	4.32E-07	8.78E-01	6.31E-05	5685	
1	rs9970083	2.13E+08	5.77E-01	SMYD2	13	rs10492664	1.08E+08	6.04E-02	N/A	-	4.32E-07	6.41E-01	2.17E-04	5686	
1	rs6678619	54534938	7.12E-02	SSBP3	8	rs10113207	78251495	7.86E-01	N/A	-	4.33E-07	4.24E-01	1.57E-05	5687	
2	rs10210465	2.31E+08	2.50E-01	SP110	11	rs1276252	1.02E+08	4.49E-01	N/A	-	4.33E-07	9.28E-01	2.89E-04	5688	
3	rs12485456	1.59E+08	9.34E-01	VEPH1	18	rs8088164	53979610	6.35E-02	NEDD4L	-	4.33E-07	6.54E-01	3.84E-05	5689	
3	rs7630566	1.68E+08	2.22E-01	N/A	8	rs7821219	84959724	3.97E-01	N/A	-	4.33E-07	2.90E-01	3.15E-04	5690	
5	rs2648731	52107951	2.20E-01	ITGA1	14	rs970662	32243135	2.78E-01	AKAP6	-	4.33E-07	4.21E-01	3.81E-06	5691	
9	rs563006	18740076	4.24E-01	ADAMTSL1	14	rs1958987	29409562	7.60E-01	PRKD1	-	4.33E-07	5.42E-01	3.78E-06	5692	
1	rs2590691	2.07E+08	6.33E-02	N/A	5	rs4631227	1.33E+08	5.10E-02	N/A	-	4.33E-07	8.11E-01	2.49E-04	5693	
2	rs12476979	1.02E+08	2.90E-02	MAP4K4	12	rs7300686	1.27E+08	2.36E-01	N/A	-	4.33E-07	1.54E-01	4.32E-03	5694	
2	rs6732726	1.02E+08	2.90E-02	MAP4K4	12	rs7300686	1.27E+08	2.36E-01	N/A	-	4.33E-07	1.66E-01	3.86E-03	5695	
9	rs7029536	1.29E+08	8.13E-02	GARNL3	10	rs1334722	7715013	5.58E-01	ITIH5	-	4.33E-07	2.88E-01	1.24E-05	5696	
2	rs2015245	14662597	2.63E-01	N/A	8	rs10097475	1.14E+08	1.98E-01	CSMD3	-	4.33E-07	7.70E-01	4.27E-05	5697	
7	rs255112	30703658	8.13E-01	CRHR2	17	rs9303381	51480301	6.68E-01	N/A	-	4.33E-07	1.90E-01	5.27E-04	5698	
5	rs11960862	78132976	4.27E-01	ARSB	10	rs10903372	1161734	9.26E-01	WDR37	-	4.33E-07	5.79E-01	8.02E-04	5699	
1	rs684231	1.14E+08	8.48E-01	SYT6	11	rs1873873	1.29E+08	5.77E-01	N/A	-	4.33E-07	8.16E-01	5.93E-05	5700	
10	rs12219393	18795615	8.52E-01	CACNB2	13	rs1331278	96310944	4.55E-02	N/A	-	4.33E-07	4.76E-01	2.44E-06	5701	
5	rs10514108	78141103	4.51E-01	ARSB	10	rs11250250	1107514	2.58E-01	WDR37	-	4.33E-07	5.58E-01	1.30E-03	5702	
6	rs6924759	82529275	8.54E-01	FAM46A	14	rs9806105	79676933	3.82E-01	N/A	-	4.33E-07	5.95E-01	4.03E-04	5703	
4	rs7441352	55608691	8.59E-01	N/A	7	rs320088	29049787	7.65E-01	CPVL	-	4.33E-07	8.54E-01	1.27E-04	5704	
14	rs1205083	71359386	5.25E-01	N/A	20	rs226834	46292121	6.68E-01	N/A	-	4.33E-07	3.03E-01	1.09E-03	5705	
8	rs351562	16043410	9.56E-01	MSR1	11	rs7950814	71905497	2.40E-01	N/A	-	4.33E-07	3.89E-01	9.95E-04	5706	
1	rs12132898	57494319	7.08E-01	DAB1	1	rs2789726	2.15E+08	2.98E-01	ESRRG	-	4.33E-07	6.23E-01	1.43E-05	5707	
10	rs1866159	80553089	1.09E-01	ZMIZ1	16	rs9927150	764099	1.45E-01	RPUSD1	-	4.33E-07	7.60E-01	6.11E-05	5708	
2	rs207431	31406898	6.50E-01	XDH	3	rs9814423	1.48E+08	6.44E-01	N/A	-	4.34E-07	5.37E-01	4.34E-05	5709	
9	rs4741165	11519226	4.87E-01	N/A	12	rs1463752	1.27E+08	5.41E-01	N/A	-	4.34E-07	2.26E-01	8.46E-06	5710	

SNP A					SNP B						Interaction P			Ranking	Cluster in top 100 interactions
CHR	SNP	Location	gle locus P va	Gene	CHR	SNP	Location	Single locus P valu	Gene	HC region	Stage 1	Stage 2	Combined		
9	rs4741165	11519226	4.87E-01	N/A	12	rs2131705	1.27E+08	5.41E-01	N/A	-	4.34E-07	N/A	N/A	5711	
6	rs2248386	31119226	7.23E-01	HCG22	6	rs7750269	31379136	8.58E-01	N/A	MHC	4.34E-07	1.77E-02	1.36E-07	5712	
5	rs13161496	1.6E+08	9.75E-02	ATP10B	12	rs2653447	99968774	5.35E-02	ANO4	-	4.34E-07	1.45E-01	2.25E-07	5713	
2	rs2118836	96526699	8.13E-01	NEURL3	8	rs1012640	17356776	1.98E-01	N/A	-	4.34E-07	6.98E-01	1.17E-04	5714	
6	rs157695	91275194	1.31E-01	MAP3K7	7	rs11979455	1.33E+08	6.11E-01	N/A	-	4.34E-07	4.47E-01	2.18E-04	5715	
1	rs4920502	18687921	3.22E-01	KLHDC7A	12	rs1641730	12084602	9.87E-01	N/A	-	4.34E-07	5.85E-01	2.07E-05	5716	
4	rs17024592	96949609	8.17E-01	N/A	13	rs7999200	23538701	5.14E-01	N/A	-	4.34E-07	9.38E-01	2.07E-05	5717	
5	rs6453292	76516517	2.53E-01	N/A	19	rs888663	18345922	2.19E-01	PGPEP1	-	4.35E-07	3.94E-01	7.64E-04	5718	
1	rs4654897	20064023	3.71E-01	OTUD3	4	rs4689786	7635460	5.98E-01	SORCS2	-	4.35E-07	5.80E-01	1.92E-05	5719	
6	rs4715607	56264084	2.92E-01	N/A	16	rs4483832	12734286	1.81E-01	CPPED1	-	4.35E-07	9.54E-01	2.51E-05	5720	
1	rs2281960	1.65E+08	6.11E-01	GPA33	12	rs2669013	75547079	6.30E-01	N/A	-	4.35E-07	N/A	N/A	5721	
1	rs2281962	1.65E+08	6.11E-01	GPA33	12	rs2669013	75547079	6.30E-01	N/A	-	4.35E-07	N/A	N/A	5722	
4	rs13150890	1.9E+08	8.91E-01	N/A	5	rs13170849	72917884	3.03E-01	UTP15	-	4.35E-07	3.57E-01	6.94E-06	5723	
10	rs10906736	14747699	5.93E-01	FAM107B	20	rs218460	56645554	4.68E-01	N/A	-	4.35E-07	4.79E-01	4.97E-04	5724	
2	rs11899692	47321835	6.25E-01	N/A	3	rs11710036	1.46E+08	3.50E-01	N/A	-	4.35E-07	5.67E-01	7.08E-04	5725	
2	rs11899692	47321835	6.25E-01	N/A	3	rs12107704	1.46E+08	3.50E-01	N/A	-	4.35E-07	5.67E-01	7.08E-04	5726	
3	rs1284736	1.04E+08	1.83E-01	N/A	10	rs7901814	24886122	6.72E-01	KIAA1217	-	4.35E-07	5.91E-01	7.83E-04	5727	
3	rs1494837	27695237	1.75E-01	N/A	8	rs1471294	1.01E+08	1.03E-01	RGS22	-	4.35E-07	8.74E-02	4.63E-03	5728	
2	rs7577925	1.34E+08	1.97E-03	NCKAP5	12	rs1401982	88513730	1.71E-01	ATP2B1	-	4.35E-07	7.63E-01	2.82E-05	5729	
2	rs843713	54359574	7.46E-01	ACYP2	3	rs1355467	73796830	1.82E-01	N/A	-	4.35E-07	1.23E-01	1.31E-06	5730	
3	rs2365794	20131306	5.03E-01	KAT2B	12	rs6538508	93474460	2.40E-01	TMCC3	-	4.35E-07	6.41E-01	3.18E-04	5731	
14	rs7140236	70181627	9.81E-01	TTC9	15	rs936227	72919012	9.02E-01	LMAN1L	-	4.35E-07	6.10E-02	2.15E-06	5732	
1	rs12089641	65316390	9.34E-01	MIR101-1	16	rs8050477	47628695	3.25E-02	N/A	-	4.35E-07	4.63E-01	5.13E-04	5733	
6	rs4296898	1.54E+08	8.39E-01	N/A	17	rs2288278	44016291	7.84E-01	MIR10A	-	4.35E-07	9.75E-01	1.34E-04	5734	
6	rs3778089	1.52E+08	1.47E-01	ESR1	3	rs2568911	1.96E+08	3.66E-01	N/A	-	4.35E-07	N/A	N/A	5735	
12	rs2245906	544049	6.02E-02	B4GALNT3	19	rs7259974	38472101	5.42E-01	CEBPA	-	4.36E-07	1.08E-01	4.79E-04	5736	
2	rs1067350	44523450	9.60E-01	C2orf34	3	rs13082706	1.96E+08	7.66E-01	ATP13A3	-	4.36E-07	5.17E-01	3.37E-05	5737	
2	rs2042542	2.18E+08	1.32E-01	TNS1	12	rs10861755	78185404	8.71E-01	SYT1	-	4.36E-07	8.15E-01	1.97E-05	5738	
5	rs13163323	27881924	8.32E-01	N/A	21	rs2833640	32371952	9.46E-01	NCRN/A00159	-	4.36E-07	9.20E-01	7.92E-05	5739	
7	rs10266620	31924075	5.17E-02	N/A	10	rs3781191	89452970	5.28E-01	PAPSS2	-	4.36E-07	3.60E-01	6.32E-06	5740	
14	rs2922641	84046658	2.64E-01	N/A	19	rs778972	5818913	9.28E-01	FUT3	-	4.36E-07	3.90E-01	1.09E-03	5741	
6	rs13437134	1.16E+08	3.72E-01	N/A	4	rs6826343	1.67E+08	1.70E-01	N/A	-	4.36E-07	9.56E-01	1.62E-04	5742	
1	rs1251575	76330372	5.00E-01	ST6GALN/AC3	2	rs264658	1.6E+08	9.27E-01	TANC1	-	4.36E-07	9.18E-01	1.36E-04	5743	
2	rs1921266	67298196	2.15E-01	N/A	9	rs4979295	1.16E+08	1.77E-01	ZNF618	-	4.36E-07	4.21E-02	3.57E-07	5744	
6	rs2294757	1.33E+08	5.47E-01	VNN1	1	rs4450009	1.56E+08	4.00E-01	N/A	-	4.36E-07	2.46E-01	2.30E-06	5745	
10	rs1272383	1.17E+08	4.51E-01	ATRNL1	19	rs2304226	34153511	9.43E-01	LOC148145	-	4.36E-07	3.73E-01	1.16E-05	5746	
5	rs17099403	1.42E+08	9.18E-01	N/A	11	rs579711	77533825	7.98E-01	ALG8	-	4.36E-07	5.69E-01	3.51E-05	5747	
1	rs3524	2.3E+08	9.49E-01	TSN/AX-DISC1	10	rs16931023	75353799	1.16E-01	C10orf55	-	4.36E-07	6.42E-01	3.70E-05	5748	
13	rs4773539	89356301	5.20E-01	N/A	14	rs1961196	84055613	3.25E-01	N/A	-	4.36E-07	4.15E-02	2.02E-07	5749	
3	rs6770065	21367311	4.02E-01	N/A	8	rs6530778	14357664	3.34E-01	SGCZ	-	4.36E-07	2.17E-01	1.39E-03	5750	
1	rs12040932	28183811	8.69E-02	EYA3	19	rs11878602	16416153	3.89E-01	EPS15L1	-	4.36E-07	6.29E-01	1.03E-05	5751	
2	rs7577243	2.41E+08	3.92E-01	PP14571	20	rs2050083	40000537	2.33E-02	N/A	-	4.36E-07	7.59E-01	1.17E-04	5752	
1	rs1410244	1.65E+08	4.30E-01	DUSP27	12	rs2669010	75533191	8.86E-02	N/A	-	4.36E-07	N/A	N/A	5753	
4	rs6835823	1.55E+08	5.78E-01	N/A	15	rs12911590	95127752	4.94E-01	SPATA8	-	4.36E-07	6.15E-01	1.49E-05	5754	
6	rs234427	4574653	8.11E-01	N/A	6	rs1765028	1.48E+08	3.38E-01	N/A	-	4.36E-07	4.42E-02	5.56E-08	5755	
5	rs716866	1.69E+08	1.09E-01	SLIT3	9	rs1444826	74536439	2.27E-02	TMC1	-	4.36E-07	4.62E-01	2.11E-04	5756	
9	rs10115619	72496357	8.34E-01	TRPM3	18	rs1625796	28369416	9.26E-01	N/A	-	4.36E-07	3.46E-01	7.00E-06	5757	
9	rs4617221	72495543	8.34E-01	TRPM3	18	rs1625796	28369416	9.26E-01	N/A	-	4.36E-07	3.46E-01	7.00E-06	5758	
6	rs3818408	10496639	7.25E-01	TFAP2A	2	rs4973451	2.32E+08	1.81E-02	C2orf57	-	4.36E-07	7.01E-01	4.53E-05	5759	
2	rs7586283	59357259	7.08E-01	N/A	14	rs7147299	38223515	4.78E-01	N/A	-	4.36E-07	1.50E-01	6.59E-04	5760	
3	rs7628692	1.45E+08	1.39E-01	SLC9A9	10	rs2297325	24863082	1.88E-01	KIAA1217	-	4.37E-07	7.29E-01	2.65E-04	5761	
11	rs7105932	94781090	3.27E-01	N/A	18	rs2657769	67690248	2.12E-01	N/A	-	4.37E-07	8.82E-01	2.70E-05	5762	
11	rs7108171	94790890	3.27E-01	N/A	18	rs2657769	67690248	2.12E-01	N/A	-	4.37E-07	8.82E-01	2.70E-05	5763	
1	rs669444	57091690	3.37E-01	C8A	4	rs12498616	1.32E+08	6.41E-01	N/A	-	4.37E-07	5.36E-01	1.02E-05	5764	
18	rs1484738	66091557	2.84E-01	SOCS6	22	rs743260	26329140	1.28E-01	N/A	-	4.37E-07	3.80E-01	5.11E-05	5765	
6	rs12527293	98306852	2.59E-02	N/A	14	rs1544488	71635235	9.17E-01	RGS6	-	4.37E-07	7.95E-01	1.77E-04	5766	
10	rs10795169	4505698	4.03E-01	N/A	22	rs735119	48970873	7.18E-01	SELO	-	4.37E-07	4.89E-01	1.12E-05	5767	
2	rs168293	8641753	2.14E-01	N/A	17	rs890431	44041839	4.49E-01	HOXB6	-	4.37E-07	2.85E-01	1.02E-03	5768	
6	rs1935949	1.09E+08	3.03E-01	FOXO3	13	rs1105576	99178430	6.33E-01	CLYBL	-	4.37E-07	6.29E-01	2.41E-03	5769	
6	rs2636601	1.34E+08	3.18E-01	N/A	13	rs7322222	96933162	7.88E-01	RAP2A	-	4.37E-07	9.63E-01	2.64E-04	5770	
6	rs2677818	1.34E+08	3.18E-01	N/A	13	rs7322222	96933162	7.88E-01	RAP2A	-	4.37E-07	9.71E-01	2.55E-04	5771	
6	rs2677820	1.34E+08	3.18E-01	N/A	13	rs7322222	96933162	7.88E-01	RAP2A	-	4.37E-07	9.71E-01	2.55E-04	5772	
6	rs10900941	3348874	6.72E-01	SLC22A23	6	rs7742857	96685540	1.04E-01	FUT9	-	4.37E-07	3.65E-01	8.07E-04	5773	
1	rs640554	1.08E+08	3.03E-01	NTNG1	3	rs6778609	17163396	5.38E-01	TBC1D5	-	4.37E-07	9.24E-01	6.61E-05	5774	
10	rs7477069	54212144	4.45E-01	MBL2	18	rs1787824	50137100	1.72E-01	C18orf54	-	4.37E-07	8.15E-02	4.71E-07	5775	
5	rs92631	1.19E+08	1.57E-01	N/A	12	rs10770476	19384370	2.69E-01	PLEKHA5	-	4.37E-07	7.32E-01	2.88E-04	5776	
5	rs2548659	59419643	7.22E-01	PDE4D	17	rs3785902	65619713	5.26E-02	KCNJ16	-	4.37E-07	9.07E-01	5.57E-05	5777	
12	rs2270151	5931221	3.63E-01	VWF	12	rs974728	11826119	5.72E-01	ETV6	-	4.38E-07	5.94E-01	7.37E-06	5778	
6	rs4997129	856832	2.83E-01	N/A	5	rs6876698	1.72E+08	1.35E-01	UBTD2	-	4.38E-07	8.93E-01	3.02E-05	5779	
12	rs1450994	96555782	6.23E-01	N/A	13	rs9554169	27313850	9.24E-01	N/A	-	4.38E-07	1.32E-01	7.26E-07	5780	
1	rs6704127	55842970	8.76E-01	N/A	16	rs4578668	63193638	4.55E-01	N/A	-	4.38E-07	6.31E-01	3.02E-04	5781	
1	rs1335051	1.07E+08	5.64E-01	N/A	5	rs75325	18117992	4.87E-01	N/A	-	4.38E-07	7.50E-01	6.98E-05	5782	
6	rs9344790	89116996	3.91E-01	N/A	10	rs1470996	1.26E+08	7.68E-01	FAM53B	-	4.38E-07	2.31E-01	4.00E-06	5783	
13	rs9511255	18516304	9.62E-01	DKFZp686A162	21	rs2252292	36991312	7.40E-01	SIM2	-	4.38E-07	3.25E-01	5.01E-04	5784	
1	rs6681821	2.07E+08	6.16E-02	N/A	5	rs4631227	1.33E+08	5.10E-02	N/A	-	4.38E-07	6.12E-01	4.06E-04	5785	
8	rs4735795	424668	4.58E-01	FBXO25	16	rs1018159	77512959	5.13E-01	WWOX	-	4.38E-07	8.93E-01	3.27E-05	5786	
2	rs3900546	1.02E+08	3.62E-01	N/A	12	rs4131522	1.02E+08	8.96E-01	N/A	-	4.38E-07	2.98E-01	5.02E-04	5787	
1	rs972099	1.71E+08	9.83E-01	N/A	10	rs7073239	85043865	7.54E-01	N/A	-	4.38E-07	4.57E-01	2.79E-05	5788	
6	rs6570451	1.42E+08	7.78E-01	N/A	10	rs1904382	65220692	1.59E-01	N/A	-	4.38E-07	5.44E-02	1.10E-06	5789	
5	rs7735817	24588073	7.68E-01	CDH10	16	rs8047519	49026539	2.62E-01	N/A	-	4.38E-07	7.52E-02	1.05E-07	5790	
2	rs551660	44730514	3.43E-01	C2orf34	2	rs2380885	1.41E+08	7.66E-01	LRP1B	-	4.38E-07	2.94E-01	1.79E-05	5791	
3	rs7646289	24776800	7.23E-01	N/A	11	rs2166706	92331180	4.72E-01	MTNR1B	-	4.38E-07	6.79E-01	8.53E-06	5792	
9	rs7038242	28795269	8.33E-02	N/A	15	rs4513061	84772636	8.10E-01	AGBL1	-	4.38E-07	#######	2.21E-05	5793	
2	rs10202499	1.61E+08	6.51E-01	N/A	11	rs6592878	79405363	6.40E-01	N/A	-	4.39E-07	2.00E-01	1.64E-03	5794	
2	rs10930002	1.61E+08	6.51E-01	N/A	11	rs6592878	79405363	6.40E-01	N/A	-	4.39E-07	2.00E-01	1.64E-03	5795	
2	rs1595066	2.12E+08	3.76E-01	ERBB4	13	rs1415707	97294596	1.04E-01	N/A	-	4.39E-07	8.82E-01	9.86E-05	5796	
12	rs10862392	80842509	3.70E-01	N/A	17	rs7208838	27492679	3.23E-01	RHOT1	-	4.39E-07	4.27E-03	6.12E-09	5797	
5	rs4379221	1.64E+08	9.09E-01	N/A	7	rs12669418	1.46E+08	1.95E-02	CNTN/AP2	-	4.39E-07	8.47E-01	7.71E-05	5798	
5	rs1501727	33599737	3.76E-01	ADAMTS12	8	rs4740010	85433884	7.11E-01	RALYL	-	4.39E-07	6.85E-01	1.18E-04	5799	
8	rs1393979	1.07E+08	8.37E-01	N/A	11	rs2370643	1.21E+08	2.16E-01	N/A	-	4.39E-07	1.32E-02	4.92E-08	5800	
2	rs264604	1.6E+08	1.71E-01	TANC1	17	rs2165846	42296365	3.50E-01	WNT9B	-	4.39E-07	1.78E-01	1.40E-07	5801	
4	rs4450911	24327595	2.69E-01	N/A	13	rs2313000	30494445	7.22E-01	N/A	-	4.39E-07	N/A	N/A	5802	
1	rs12035823	1.03E+08	5.47E-01	N/A	4	rs11100890	1.39E+08	8.35E-01	N/A	-	4.40E-07	6.31E-01	1.93E-04	5803	
12	rs4913287	66944537	9.23E-01	IL22	15	rs6492997	39328463	4.90E-01	CHP	-	4.40E-07	7.89E-01	2.66E-05	5804	
12	rs4913287	66944537	9.23E-01	IL22	15	rs7175912	39342541	4.90E-01	CHP	-	4.40E-07	8.35E-01	3.10E-05	5805	
6	rs214445	9507024	4.84E-01	N/A	9	rs10981012	1.13E+08	1.65E-01	C9orf84	-	4.40E-07	5.24E-01	1.97E-05	5806	
3	rs9872768	1.48E+08	1.86E-01	N/A	8	rs12114032	20891612	8.29E-01	N/A	-	4.40E-07	6.40E-01	2.56E-04	5807	
6	rs1419638	29463498	1.74E-01	OR12D3	2	rs4663902	2.39E+08	5.94E-01	N/A	MHC	4.40E-07	7.77E-01	3.89E-05	5808	
6	rs3130573	31214247	2.01E-01	PSORS1C1	12	rs902062	1.28E+08	1.35E-01	TMEM132C	MHC	4.40E-07	7.98E-01	4.70E-05	5809	
1	rs7541544	4191702	8.36E-01	N/A	4	rs10049681	95903403	9.11E-01	BMPR1B	-	4.40E-07	9.15E-01	1.54E-04	5810	
5	rs4145160	1.53E+08	6.37E-01	GRIA1	8	rs1901090	1.34E+08	9.03E-01	KCNQ3	-	4.40E-07	9.81E-01	1.67E-05	5811	
2	rs728672	2.35E+08	5.74E-01	TRPM8	5	rs12152801	1.48E+08	4.37E-01	HTR4	-	4.40E-07	7.65E-01	3.93E-05	5812	
1	rs1832787	18499504	2.02E-01	IGSF21	3	rs6783423	29737953	7.89E-01	N/A	-	4.40E-07	9.93E-01	1.18E-04	5813	
1	rs4908382	28156065	2.56E-01	EYA3	3	rs721572	1.45E+08	7.91E-01	C3orf58	-	4.40E-07	8.13E-02	3.03E-03	5814	
4	rs12509042	1.55E+08	8.84E-01	N/A	11	rs648067	1.19E+08	4.25E-01	N/A	-	4.40E-07	7.97E-01	5.44E-06	5815	
4	rs7663313	1.55E+08	9.53E-01	N/A	11	rs648067	1.19E+08	4.25E-01	N/A	-	4.40E-07	9.83E-01	1.32E-05	5816	
2	rs7586087	15222505	7.98E-01	NBAS	7	rs6977642	18966254	5.48E-03	HDAC9	-	4.40E-07	5.17E-01	1.93E-05	5817	
12	rs17313674	85963295	8.78E-01	N/A	16	rs4997772	80462796	1.94E-01	PLCG2	-	4.40E-07	8.61E-01	2.39E-04	5818	
10	rs4752275	1.21E+08	5.29E-01	GRK5	16	rs4888108	72620781	6.18E-03	N/A	-	4.40E-07	5.43E-02	8.16E-03	5819	
8	rs10086002	10429327	8.75E-01	T-SP1	9	rs872039	2409538	5.30E-01	N/A	-	4.40E-07	7.51E-01	9.15E-05	5820	
10	rs1751291	4482612	2.98E-01	N/A	16	rs3852716	81170828	4.46E-01	N/A	-	4.41E-07	3.97E-01	1.91E-06	5821	
18	rs7238168	32291551	4.02E-01	FHOD3	19	rs919271	57360226	2.20E-01	ZNF836	-	4.41E-07	8.64E-01	9.42E-05	5822	

SNP A					SNP B						Interaction P			Ranking	Cluster in top 100 interactions
CHR	SNP	Location	gle locus P va	Gene	CHR	SNP	Location	Single locus P valu	Gene	MHC region	Stage 1	Stage 2	Combined		
4	rs17583808	1.66E+08	5.57E-01	N/A	20	rs6130274	41047898	7.73E-02	PTPRT	-	4.41E-07	9.97E-02	1.12E-02	5823	
6	rs454165	1.66E+08	5.89E-01	PDE10A	7	rs1799384	47452490	2.50E-01	TNS3	-	4.41E-07	3.09E-01	6.19E-04	5824	
2	rs4669725	11448167	6.07E-01	N/A	3	rs9283633	1.66E+08	4.36E-01	SI	-	4.41E-07	4.88E-01	1.97E-03	5825	
2	rs4669725	11448167	6.07E-01	N/A	3	rs9825346	1.66E+08	4.36E-01	SI	-	4.41E-07	5.08E-01	1.85E-03	5826	
7	rs4607527	1.41E+08	7.87E-02	MGAM	8	rs7386095	1.39E+08	6.69E-01	N/A	-	4.41E-07	5.56E-01	5.62E-06	5827	
2	rs1487395	1.88E+08	1.73E-01	N/A	21	rs958924	26498774	5.76E-02	N/A	-	4.41E-07	7.53E-01	1.83E-04	5828	
1	rs1373291	1.12E+08	9.94E-01	KCND3	10	rs2388260	2580305	1.52E-01	N/A	-	4.41E-07	8.07E-01	3.96E-05	5829	
1	rs1373291	1.12E+08	9.94E-01	KCND3	10	rs2388268	2585331	1.52E-01	N/A	-	4.41E-07	8.17E-01	4.11E-05	5830	
3	rs10511051	76747249	9.79E-01	N/A	4	rs4388065	1.76E+08	8.29E-01	ADAM29	-	4.41E-07	2.92E-01	5.08E-04	5831	
6	rs911566	4421201	3.95E-02	N/A	16	rs756813	23198310	4.85E-01	N/A	-	4.41E-07	4.49E-01	3.85E-04	5832	
1	rs2013526	1.65E+08	9.88E-01	ILDR2	22	rs4819558	15996217	3.00E-01	CECR5	-	4.41E-07	N/A	N/A	5833	
6	rs9362770	91463530	6.77E-01	N/A	8	rs10504661	79009024	8.90E-01	N/A	-	4.41E-07	8.39E-02	6.48E-07	5834	
6	rs4928434	58432168	7.99E-01	N/A	4	rs4364309	1.59E+08	3.57E-01	N/A	-	4.41E-07	2.08E-01	6.06E-04	5835	
2	rs10490511	80802274	8.48E-02	N/A	4	rs1969399	1.82E+08	9.51E-01	N/A	-	4.41E-07	4.77E-01	9.96E-04	5836	
7	rs1285933	1.41E+08	9.22E-01	CLEC5A	15	rs4775214	57924997	1.59E-01	N/A	-	4.41E-07	8.77E-01	1.87E-05	5837	
11	rs10908210	69283997	7.39E-01	FGF4	13	rs603894	1.02E+08	1.89E-01	C13orf39	-	4.42E-07	2.39E-01	1.09E-06	5838	
2	rs12692087	1.3E+08	2.36E-01	N/A	7	rs515906	1.54E+08	1.75E-01	DPP6	-	4.42E-07	5.47E-01	1.74E-05	5839	
6	rs1360738	1.43E+08	1.34E-01	N/A	4	rs1391010	76300577	9.83E-01	N/A	-	4.42E-07	6.31E-01	1.39E-05	5840	
6	rs9321880	1.43E+08	1.34E-01	N/A	4	rs1391010	76300577	9.83E-01	N/A	-	4.42E-07	N/A	N/A	5841	
7	rs2058152	13100464	3.88E-02	N/A	19	rs3746228	62496174	5.82E-01	ZNF460	-	4.42E-07	8.66E-02	1.49E-06	5842	
4	rs10029516	1.14E+08	6.49E-01	ANK2	16	rs12918743	11531746	1.20E-01	LITAF	-	4.42E-07	3.61E-01	4.72E-06	5843	
11	rs586421	94504982	1.62E-01	ENDOD1	20	rs928064	54837966	1.08E-01	N/A	-	4.42E-07	4.21E-01	2.52E-03	5844	
7	rs7784901	13097171	3.88E-02	N/A	19	rs3746228	62496174	5.82E-01	ZNF460	-	4.42E-07	6.91E-01	1.27E-05	5845	
6	rs3094205	31199841	5.91E-01	C6orf15	6	rs7750269	31379136	8.58E-01	N/A	MHC	4.42E-07	6.91E-01	1.27E-05	5846	
7	rs6973203	1.47E+08	1.84E-01	CNTN/AP2	19	rs422318	6902434	3.99E-01	EMR1	-	4.42E-07	1.63E-02	1.68E-07	5847	
8	rs4871564	1.26E+08	9.34E-01	N/A	17	rs12602762	23219007	8.77E-01	C17orf108	-	4.42E-07	8.32E-01	8.96E-05	5848	
8	rs4871562	1.26E+08	9.34E-01	N/A	17	rs12602762	23219007	8.77E-01	C17orf108	-	4.42E-07	8.93E-01	7.52E-05	5849	
4	rs2614563	1.8E+08	1.82E-01	N/A	8	rs1380634	80400448	1.07E-01	N/A	-	4.42E-07	5.60E-01	1.41E-04	5850	
4	rs2702386	1.8E+08	1.82E-01	N/A	8	rs1380634	80400448	1.07E-01	N/A	-	4.42E-07	N/A	N/A	5851	
6	rs2295199	1.35E+08	1.94E-01	N/A	19	rs17254521	7189695	9.97E-01	INSR	-	4.42E-07	7.06E-02	9.63E-03	5852	
6	rs9341388	73562825	7.18E-01	KCNQ5	12	rs10880377	41552055	6.29E-01	N/A	-	4.42E-07	8.24E-01	7.99E-04	5853	
5	rs2567816	1.21E+08	6.31E-01	N/A	7	rs10464419	1.54E+08	1.52E-01	DPP6	-	4.42E-07	1.32E-01	6.59E-06	5854	
2	rs1921627	77026201	5.37E-01	LRRTM4	5	rs1038046	3198652	5.89E-01	N/A	-	4.42E-07	7.17E-01	1.91E-05	5855	
2	rs7572482	1.92E+08	5.02E-01	STAT4	15	rs7167270	94576562	9.93E-01	N/A	-	4.42E-07	5.10E-01	7.24E-06	5856	
6	rs3823301	1.7E+08	1.11E-01	FAM120B	12	rs1351266	28168605	2.89E-01	N/A	-	4.42E-07	2.44E-01	3.41E-06	5857	
8	rs13258079	1.22E+08	6.33E-01	MTBP	22	rs5768016	46595486	9.66E-01	N/A	-	4.42E-07	7.44E-01	2.96E-05	5858	
4	rs1367528	1.17E+08	8.74E-01	N/A	4	rs9999397	1.34E+08	5.88E-01	N/A	-	4.42E-07	7.46E-01	5.11E-04	5859	
4	rs6449012	13926214	8.95E-01	N/A	4	rs11937354	96756747	1.91E-01	N/A	-	4.43E-07	5.03E-01	4.98E-04	5860	
4	rs6843137	1.3E+08	6.63E-01	N/A	19	rs4801731	52958023	1.21E-01	EHD2	-	4.43E-07	5.43E-01	2.28E-05	5861	
18	rs1517033	55088717	7.53E-01	RAX	20	rs168525	58370741	3.12E-01	N/A	-	4.43E-07	9.08E-01	6.32E-05	5862	
2	rs12612419	1.6E+08	9.90E-01	WDSUB1	10	rs4439449	1.14E+08	4.11E-01	VTI1A	-	4.43E-07	5.87E-01	3.77E-04	5863	
5	rs10035580	16617901	7.32E-01	FAM134B	9	rs7034380	14898056	5.82E-01	FREM1	-	4.43E-07	4.81E-01	6.11E-04	5864	
10	rs1925542	20986659	6.24E-01	N/A	14	rs12433260	57622646	9.30E-01	C14orf37	-	4.43E-07	4.26E-02	6.99E-07	5865	
6	rs164548	94128329	5.15E-01	EPHA7	3	rs12629430	1.09E+08	1.21E-01	LOC344595	-	4.43E-07	3.77E-01	7.70E-04	5866	
5	rs17708295	17022440	5.82E-01	N/A	22	rs4481093	44462694	9.37E-01	ATXN10	-	4.43E-07	3.91E-01	1.37E-05	5867	
15	rs294547	92647763	3.11E-01	MCTP2	20	rs6085256	5655548	3.06E-01	N/A	-	4.43E-07	6.12E-01	1.68E-05	5868	
11	rs1382775	79673362	9.86E-02	N/A	11	rs543876	1.19E+08	6.46E-01	N/A	-	4.43E-07	6.29E-01	1.42E-04	5869	
5	rs1423611	50806871	7.37E-01	N/A	12	rs11045812	21212749	1.32E-01	SLCO1B1	-	4.43E-07	9.87E-01	3.17E-04	5870	
6	rs4707657	91922402	7.63E-01	N/A	12	rs10860226	96537446	6.03E-01	N/A	-	4.43E-07	3.55E-01	3.30E-04	5871	
3	rs1378661	1.62E+08	8.01E-01	N/A	20	rs761865	24021113	2.28E-01	N/A	-	4.43E-07	8.64E-01	3.00E-05	5872	
5	rs6881283	91091413	2.28E-01	N/A	16	rs1075072	53863076	6.97E-01	N/A	-	4.43E-07	2.82E-01	1.12E-05	5873	
7	rs6954727	40974397	7.75E-01	N/A	8	rs10108175	88832035	6.38E-01	N/A	-	4.43E-07	5.17E-01	1.43E-05	5874	
6	rs483366	1.62E+08	3.18E-01	PARK2	8	rs7004118	85928307	7.04E-01	RALYL	-	4.43E-07	9.54E-01	4.14E-05	5875	
12	rs10845539	12471314	1.13E-01	LOH12CR1	12	rs1402318	76753767	8.81E-01	N/AV3	-	4.43E-07	8.24E-01	8.42E-05	5876	
8	rs6992419	20609295	9.39E-01	N/A	13	rs4884202	82305944	2.09E-01	N/A	-	4.43E-07	9.88E-01	7.12E-05	5877	
2	rs6431957	5457870	5.83E-02	N/A	16	rs2795571	6435492	9.03E-01	A2BP1	-	4.44E-07	5.31E-01	1.60E-05	5878	
6	rs2844665	31114834	8.49E-01	HCG22	22	rs2071761	43636606	5.15E-01	PHF21B	MHC	4.44E-07	8.50E-01	8.34E-05	5879	
1	rs219002	36899099	7.55E-01	N/A	4	rs1967096	1.76E+08	8.60E-02	N/A	-	4.44E-07	7.37E-01	1.28E-05	5880	
2	rs788018	1.98E+08	8.83E-01	SF3B1	10	rs10736312	1.24E+08	3.97E-01	BTBD16	-	4.44E-07	2.16E-01	3.77E-06	5881	
2	rs788018	1.98E+08	8.83E-01	SF3B1	10	rs2421013	1.24E+08	3.97E-01	BTBD16	-	4.44E-07	2.16E-01	3.77E-06	5882	
9	rs7029669	1.09E+08	4.90E-01	N/A	10	rs11187968	96217420	4.05E-01	TBC1D12	-	4.44E-07	8.89E-01	9.04E-04	5883	
3	rs4686910	1.89E+08	9.80E-01	N/A	20	rs6081622	19441514	2.89E-01	SLC24A3	-	4.44E-07	1.44E-01	1.09E-03	5884	
6	rs674327	1.53E+08	5.82E-01	N/A	20	rs6077218	7443442	8.49E-01	N/A	-	4.44E-07	1.21E-01	6.73E-07	5885	
9	rs10960390	11938271	9.23E-02	N/A	10	rs17578297	71079206	8.60E-01	C10orf35	-	4.44E-07	7.68E-01	2.31E-05	5886	
2	rs962052	1.51E+08	9.58E-01	N/A	7	rs4724965	7273557	9.03E-01	C1GALT1	-	4.44E-07	1.53E-01	1.15E-02	5887	
3	rs12489904	20359631	4.21E-01	N/A	14	rs1188692	55859765	1.29E-01	N/A	-	4.44E-07	6.21E-01	4.75E-05	5888	
3	rs1300120	1.04E+08	2.85E-01	N/A	13	rs9534981	47653682	6.92E-01	N/A	-	4.44E-07	2.83E-01	2.57E-06	5889	
1	rs487344	88490122	5.06E-01	N/A	7	rs4725035	7821066	3.94E-01	N/A	-	4.44E-07	3.06E-01	4.00E-06	5890	
3	rs1300120	1.04E+08	2.85E-01	N/A	13	rs7998964	47686991	6.92E-01	ITM2B	-	4.44E-07	3.15E-01	2.99E-06	5891	
3	rs1300120	1.04E+08	2.85E-01	N/A	13	rs9534995	47684077	6.92E-01	N/A	-	4.44E-07	3.15E-01	2.99E-06	5892	
8	rs4469412	33684920	9.17E-01	N/A	18	rs6505558	10445680	3.89E-01	APCDD1	-	4.44E-07	N/A	N/A	5893	
2	rs2595203	46045695	2.94E-01	PRKCE	3	rs9654034	29318469	8.54E-01	N/A	-	4.44E-07	1.32E-01	3.82E-06	5894	
11	rs10790202	1.17E+08	5.47E-01	DSCAML1	12	rs1396208	24449587	3.61E-01	SOX5	-	4.44E-07	3.36E-01	3.82E-06	5895	
6	rs7770797	6566961	8.95E-01	LOC285780	10	rs11188985	98757832	7.12E-01	SLIT1	-	4.44E-07	6.17E-01	3.66E-04	5896	
9	rs10964716	20801311	6.67E-01	KIAA1797	12	rs4765110	1.26E+08	2.97E-01	N/A	-	4.44E-07	8.13E-01	5.25E-05	5897	
11	rs11222391	1.3E+08	8.45E-01	SNX19	18	rs10454095	18031438	5.72E-02	GATA6	-	4.45E-07	1.78E-01	9.11E-07	5898	
6	rs2764208	34822300	4.22E-01	SNRPC	11	rs12420859	44681103	6.76E-01	N/A	MHC	4.45E-07	2.86E-01	1.67E-06	5899	
7	rs2058163	34301616	2.61E-01	N/A	13	rs7997335	53936920	8.84E-01	N/A	-	4.45E-07	2.86E-01	1.67E-06	5900	
6	rs9462015	34847704	4.22E-01	SNRPC	11	rs12420859	44681103	6.76E-01	N/A	MHC	4.45E-07	9.15E-01	7.50E-05	5901	
4	rs4640717	66029475	5.28E-01	EPHA5	12	rs11176761	66189993	8.19E-01	N/A	-	4.45E-07	9.28E-01	2.60E-04	5902	
15	rs5026360	98566891	7.71E-01	ADAMTS17	17	rs9897263	74002853	3.65E-01	DN/AH17	-	4.45E-07	3.94E-01	6.91E-04	5903	
9	rs11103233	1.38E+08	9.44E-01	UBAC1	12	rs7965873	42641064	4.06E-01	TMEM117	-	4.45E-07	3.14E-01	4.30E-04	5904	
5	rs2695990	1.66E+08	7.16E-01	N/A	15	rs1961839	87268458	4.60E-01	MFGE8	-	4.45E-07	7.60E-01	1.64E-04	5905	
6	rs259723	37896198	9.34E-01	ZFAND3	16	rs231921	20569262	1.79E-02	ACSM1	-	4.45E-07	8.94E-01	8.62E-05	5906	
5	rs12697762	85155175	7.21E-01	N/A	11	rs11219100	1.23E+08	7.18E-01	N/A	-	4.45E-07	1.96E-01	1.21E-06	5907	
1	rs1452607	2.13E+08	5.83E-01	KCNK2	5	rs10070522	57821867	3.20E-01	GAPT	-	4.45E-07	1.69E-01	6.34E-03	5908	
1	rs1452612	2.13E+08	5.83E-01	KCNK2	5	rs10070522	57821867	3.20E-01	GAPT	-	4.45E-07	2.00E-01	4.94E-03	5909	
1	rs1333065	1.59E+08	5.97E-01	LY9	13	rs261474	81237286	3.55E-01	N/A	-	4.45E-07	8.44E-01	6.63E-05	5910	
6	rs9328172	3385313	5.00E-02	SLC22A23	2	rs1921651	2.24E+08	1.34E-01	N/A	-	4.45E-07	1.20E-02	4.24E-08	5911	
7	rs1345934	1.36E+08	1.76E-01	N/A	11	rs12281086	1.1E+08	2.52E-01	ARHGAP20	-	4.45E-07	3.03E-02	2.18E-08	5912	
7	rs1345934	1.36E+08	1.76E-01	N/A	11	rs7127275	1.1E+08	2.52E-01	ARHGAP20	-	4.45E-07	3.57E-01	3.76E-04	5913	
7	rs4460277	43234995	6.55E-01	HECW1	15	rs17680945	27027109	3.43E-01	APBA2	-	4.45E-07	3.57E-01	3.76E-04	5914	
6	rs9262155	30808476	3.99E-01	MDC1	6	rs2524123	31373293	7.02E-01	N/A	MHC	4.45E-07	3.69E-01	9.33E-06	5915	
1	rs1001373	1.7E+08	2.20E-02	N/A	9	rs12376350	89192731	3.19E-01	N/A	-	4.45E-07	1.42E-01	3.05E-03	5916	
2	rs2709549	1.33E+08	9.50E-01	NCKAP5	11	rs2512568	1.15E+08	5.17E-01	N/A	-	4.45E-07	2.72E-01	1.89E-06	5917	
3	rs2600226	12903762	4.43E-01	IQSEC1	11	rs2467059	85076646	2.91E-01	SYTL2	-	4.45E-07	8.70E-01	3.59E-05	5918	
6	rs9405622	3152257	5.14E-02	TUBB2B	8	rs10504826	87759135	1.46E-01	CNGB3	-	4.45E-07	3.14E-01	7.89E-06	5919	
5	rs756699	1.33E+08	1.33E-01	TCF7	7	rs10263303	1.57E+08	1.02E-01	PTPRN2	-	4.46E-07	3.40E-01	1.39E-04	5920	
11	rs2285030	63351793	5.63E-01	MARK2	20	rs2297056	44429857	7.67E-01	SLC35C2	-	4.46E-07	4.29E-01	1.85E-05	5921	
11	rs2285030	63351793	5.63E-01	MARK2	20	rs10211808	44419171	7.67E-01	SLC35C2	-	4.46E-07	4.62E-01	2.14E-05	5922	
7	rs4265124	1.04E+08	7.94E-01	N/A	13	rs4885537	77875455	1.46E-01	N/A	-	4.46E-07	4.66E-01	1.80E-04	5923	
3	rs4858021	21827143	8.93E-03	N/A	3	rs4679863	1.61E+08	8.68E-01	SCHIP1	-	4.46E-07	2.07E-01	2.24E-06	5924	
6	rs6458566	47469747	9.31E-01	N/A	14	rs7156053	39998166	5.56E-01	N/A	-	4.46E-07	6.43E-01	5.88E-06	5925	
5	rs6887279	1.04E+08	5.70E-02	N/A	18	rs2040177	882310	1.75E-01	ADCYAP1	-	4.46E-07	7.89E-01	9.24E-06	5926	
8	rs7386222	36908749	3.48E-01	KCNU1	12	rs345659	1.27E+08	6.04E-01	N/A	-	4.46E-07	8.85E-01	4.10E-05	5927	
17	rs11650399	19456287	5.90E-01	N/A	21	rs2826056	20459129	9.51E-01	N/A	-	4.46E-07	8.90E-01	5.04E-05	5928	
3	rs1599796	1.21E+08	5.30E-01	CD80	18	rs339866	20462599	6.44E-03	N/A	-	4.46E-07	9.53E-01	9.69E-05	5929	
3	rs3755579	1.21E+08	5.30E-01	KTELC1	18	rs339866	20462599	6.44E-03	N/A	-	4.46E-07	9.53E-01	9.69E-05	5930	
2	rs6430516	1.35E+08	7.45E-01	TMEM163	4	rs10520357	1.78E+08	4.46E-01	N/A	-	4.46E-07	2.39E-01	1.65E-06	5931	
5	rs12516921	1.65E+08	2.00E-01	N/A	19	rs10412551	51605589	1.00E+00	CCDC8	-	4.46E-07	2.81E-01	9.15E-06	5932	
1	rs1414529	2.15E+08	6.14E-01	ESRRG	4	rs6822279	99964728	1.96E-01	N/A	-	4.46E-07	N/A	N/A	5933	
6	rs2273238	90383081	1.31E-01	ANKRD6	8	rs2045029	27978493	2.74E-01	C8orf80	-	4.46E-07	2.55E-01	8.58E-06	5934	

SNP A					SNP B						Interaction P			Ranking	Cluster in top 100 interactions
CHR	SNP	Location	gle locus P va	Gene	CHR	SNP	Location	Single locus P valu	Gene	MHC region	Stage 1	Stage 2	Combined		
4	rs2871153	1.82E+08	8.72E-01	N/A	19	rs7259371	38226481	8.16E-01	RHPN2	-	4.46E-07	8.65E-01	1.43E-05	5935	
1	rs2776747	24748970	4.42E-01	C1orf130	2	rs17006285	70834394	7.99E-01	N/A	-	4.46E-07	6.83E-03	3.32E-08	5936	
4	rs7666914	1.73E+08	4.68E-01	GALNTL6	9	rs11998869	1.35E+08	8.36E-01	DBH	-	4.46E-07	2.03E-01	2.79E-06	5937	
6	rs2817200	24692345	1.05E-01	KIAA0319	4	rs2340252	1.57E+08	2.93E-01	N/A	-	4.46E-07	4.71E-01	3.42E-04	5938	
4	rs16890501	14062550	4.50E-01	N/A	8	rs2978010	1.35E+08	8.56E-01	ST3GAL1	-	4.47E-07	1.21E-01	9.74E-07	5939	
4	rs2622629	89313088	6.90E-01	ABCG2	21	rs2837964	41442516	7.16E-02	BACE2	-	4.47E-07	3.44E-01	3.91E-04	5940	
7	rs157920	1.3E+08	7.66E-02	MIR29B1	22	rs2283670	44626834	9.17E-01	ATXN10	-	4.47E-07	4.09E-01	6.83E-04	5941	
12	rs10843492	29742986	4.65E-01	N/A	18	rs2159637	9828349	9.67E-01	RAB31	-	4.47E-07	4.29E-01	2.87E-04	5942	
4	rs1512129	46597190	2.44E-01	COX7B2	17	rs8068010	4278869	2.49E-01	SPNS3	-	4.47E-07	5.08E-01	1.89E-04	5943	
9	rs1412059	1.21E+08	9.57E-01	N/A	18	rs633163	2287887	9.30E-01	N/A	-	4.47E-07	3.68E-01	1.95E-03	5944	
8	rs7814222	1.21E+08	5.34E-01	COL14A1	22	rs16985531	26491703	8.13E-01	MN1	-	4.47E-07	5.32E-01	3.05E-04	5945	
7	rs12666922	19176462	8.62E-01	N/A	14	rs2295388	1E+08	2.66E-01	MEG3	-	4.47E-07	7.99E-01	5.19E-05	5946	
4	rs4340831	32442419	5.95E-01	N/A	5	rs2591699	5586625	7.41E-01	N/A	-	4.47E-07	8.89E-01	3.99E-05	5947	
6	rs9458499	1.63E+08	9.55E-01	PARK2	3	rs6774264	62468862	4.79E-02	CADPS	-	4.47E-07	N/A	N/A	5948	
6	rs296424	1.34E+08	5.44E-01	EYA4	1	rs11584093	54769601	7.91E-01	ACOT11	-	4.47E-07	5.65E-01	7.27E-06	5949	
6	rs296425	1.34E+08	5.44E-01	EYA4	1	rs11584093	54769601	7.91E-01	ACOT11	-	4.47E-07	5.65E-01	7.27E-06	5950	
6	rs6931504	1.49E+08	5.91E-01	SASH1	2	rs11682769	7278731	5.36E-01	N/A	-	4.47E-07	1.59E-02	2.69E-02	5951	
6	rs6931504	1.49E+08	5.91E-01	SASH1	2	rs13396157	7277175	5.36E-01	N/A	-	4.47E-07	1.59E-02	2.69E-02	5952	
9	rs1887787	1.33E+08	9.39E-01	RAPGEF1	22	rs5770651	48463535	8.37E-02	N/A	-	4.47E-07	2.48E-01	6.06E-06	5953	
1	rs10915971	2.25E+08	2.28E-01	LIN9	16	rs8050477	47628695	3.25E-02	N/A	-	4.47E-07	3.04E-01	1.74E-03	5954	
8	rs1538728	1.31E+08	7.47E-01	ASAP1	13	rs9512928	27411301	6.17E-01	PDX1	-	4.47E-07	8.53E-01	5.06E-05	5955	
2	rs17013592	77088275	7.17E-01	LRRTM4	5	rs2165515	3227032	8.89E-01	N/A	-	4.47E-07	9.98E-01	1.97E-04	5956	
6	rs9342394	65485313	1.00E+00	EYS	3	rs662959	1.61E+08	7.18E-01	IL12A	-	4.47E-07	7.40E-01	5.37E-05	5957	
8	rs1866897	70688850	8.46E-01	SULF1	12	rs11172162	38129079	1.23E-01	N/A	-	4.48E-07	7.64E-01	1.17E-04	5958	
11	rs11232180	79914436	3.63E-01	N/A	14	rs8022194	65776943	7.91E-02	N/A	-	4.48E-07	2.60E-01	8.05E-03	5959	
3	rs12107377	1.94E+08	3.20E-01	FGF12	4	rs2047246	1.77E+08	8.59E-02	GPM6A	-	4.48E-07	5.40E-01	3.38E-04	5960	
2	rs4852246	71165151	2.41E-01	N/AGK	7	rs757196	1.12E+08	1.08E-01	DOCK4	-	4.48E-07	3.22E-01	3.38E-04	5961	
6	rs9382120	52535698	4.82E-02	TRAM2	9	rs1328403	1.34E+08	6.43E-01	DDX31	-	4.48E-07	6.24E-01	9.12E-06	5962	
1	rs795576	75246566	6.56E-01	N/A	5	rs828718	1.62E+08	5.73E-01	N/A	-	4.48E-07	8.15E-01	2.32E-04	5963	
4	rs5024785	1.82E+08	9.19E-01	N/A	19	rs7259371	38226481	8.16E-01	RHPN2	-	4.48E-07	8.71E-01	1.43E-05	5964	
17	rs322173	22687706	1.42E-02	N/A	21	rs928873	42166440	9.14E-02	PRDM15	-	4.48E-07	N/A	N/A	5965	
11	rs11232180	79914436	3.63E-01	N/A	14	rs7145240	65778052	7.77E-02	N/A	-	4.48E-07	2.60E-01	7.97E-03	5966	
17	rs9907360	66913916	3.31E-01	N/A	21	rs2834732	35308597	7.01E-01	RUNX1	-	4.48E-07	3.32E-01	1.27E-05	5967	
2	rs12993541	41092961	5.50E-01	N/A	2	rs10200201	1.13E+08	7.60E-01	FBLN7	-	4.48E-07	2.08E-01	9.03E-06	5968	
3	rs4839632	1.45E+08	4.77E-02	SLC9A9	17	rs9889335	66626741	5.16E-01	N/A	-	4.48E-07	2.22E-01	7.14E-06	5969	
8	rs1619829	1.41E+08	4.12E-02	KCNK9	17	rs612720	44273203	4.77E-01	CALCOCO2	-	4.48E-07	6.35E-01	2.63E-05	5970	
3	rs9813521	97363067	3.05E-01	N/A	7	rs12703329	1.54E+08	2.15E-01	DPP6	-	4.48E-07	9.48E-01	1.02E-04	5971	
2	rs4233870	9728099	9.47E-01	N/A	9	rs10815019	4537288	9.23E-01	SLC1A1	-	4.48E-07	3.13E-01	3.98E-06	5972	
7	rs10246184	50177586	5.43E-01	C7orf72	12	rs492806	91379989	5.65E-01	N/A	-	4.48E-07	2.28E-01	3.00E-06	5973	
3	rs16943	51849719	7.98E-01	IQCF3	9	rs12552164	30007230	7.98E-01	N/A	-	4.48E-07	3.81E-01	5.12E-06	5974	
5	rs252152	1.41E+08	3.95E-01	N/A	8	rs1510465	90645751	4.23E-01	N/A	-	4.48E-07	4.03E-01	3.33E-06	5975	
6	rs7769547	39156735	5.43E-01	GLP1R	7	rs846936	1.05E+08	8.30E-02	ATXN7L1	-	4.49E-07	6.80E-01	5.14E-04	5976	
3	rs6775796	74786379	7.90E-02	N/A	17	rs2279730	68865580	3.35E-01	SDK2	-	4.49E-07	2.72E-01	3.55E-04	5977	
2	rs17031608	66216670	6.44E-01	N/A	4	rs4695935	1.76E+08	3.68E-01	N/A	-	4.49E-07	7.01E-01	1.26E-05	5978	
7	rs3778810	1.37E+08	7.63E-01	DGKI	9	rs10814284	3583961	6.04E-01	N/A	-	4.49E-07	7.23E-01	1.39E-04	5979	
5	rs17635646	1.68E+08	2.54E-01	SLIT3	10	rs2505331	27816417	5.15E-01	RAB18	-	4.49E-07	7.59E-01	1.25E-05	5980	
8	rs2218279	1.07E+08	6.00E-01	ZFPM2	19	rs4801516	62915910	2.21E-01	ZNF671	-	4.49E-07	1.29E-01	2.59E-03	5981	
1	rs10800778	2E+08	2.07E-01	TNNI1	7	rs2074866	12646979	2.98E-01	SCIN	-	4.49E-07	5.52E-01	2.13E-05	5982	
4	rs6845545	61762810	9.92E-01	N/A	5	rs2678181	1.25E+08	6.63E-01	N/A	-	4.49E-07	7.74E-01	8.21E-05	5983	
5	rs12654056	1.01E+08	8.58E-01	N/A	11	rs10792641	82167402	1.25E-01	N/A	-	4.49E-07	7.78E-01	2.51E-04	5984	
4	rs6845545	61762810	9.92E-01	N/A	5	rs2678180	1.25E+08	6.63E-01	N/A	-	4.49E-07	7.81E-01	7.99E-05	5985	
6	rs672706	1.64E+08	2.65E-01	N/A	9	rs10966450	24760659	3.09E-02	N/A	-	4.50E-07	7.99E-01	9.89E-05	5986	
6	rs2517552	31115569	8.54E-01	HCG22	22	rs2071761	43636606	5.15E-01	PHF21B	MHC	4.50E-07	8.21E-01	2.43E-04	5987	
4	rs6845545	61762810	9.92E-01	N/A	5	rs7701571	1.25E+08	6.64E-01	N/A	-	4.50E-07	9.26E-01	4.97E-05	5988	
12	rs10492095	5431461	8.87E-03	NTF3	13	rs2153454	65203559	5.56E-01	N/A	-	4.50E-07	2.82E-01	4.65E-03	5989	
1	rs12037569	1.1E+08	4.37E-01	SORT1	5	rs7710000	1.55E+08	1.55E-02	N/A	-	4.50E-07	4.48E-01	1.24E-05	5990	
6	rs9355443	1.64E+08	5.34E-01	N/A	17	rs3179840	44722535	3.71E-01	ZNF652	-	4.50E-07	1.73E-01	9.27E-07	5991	
3	rs16825206	1.57E+08	6.78E-01	PLCH1	14	rs2239220	71922329	3.85E-01	RGS6	-	4.50E-07	3.63E-01	1.45E-03	5992	
1	rs274587	74716053	5.38E-01	TNNI3K	1	rs959163	1.02E+08	2.53E-01	N/A	-	4.50E-07	8.67E-01	1.73E-04	5993	
1	rs274604	74729913	5.38E-01	TNNI3K	1	rs959163	1.02E+08	2.53E-01	N/A	-	4.50E-07	8.67E-01	1.73E-04	5994	
5	rs7716821	25659711	7.27E-01	N/A	11	rs12363688	26001568	7.50E-01	N/A	-	4.50E-07	7.77E-01	5.66E-05	5995	
3	rs2130369	1.01E+08	7.50E-01	C3orf26	8	rs12682381	16946172	6.38E-01	EFHA2	-	4.50E-07	8.60E-01	9.97E-06	5996	
1	rs4970609	38865036	2.56E-01	N/A	9	rs7858089	28399300	3.65E-01	LINGO2	-	4.50E-07	4.22E-01	1.88E-04	5997	
2	rs7587228	2.28E+08	2.98E-01	MFF	3	rs1546007	1.12E+08	2.86E-03	N/A	-	4.50E-07	4.84E-01	8.04E-06	5998	
18	rs9956612	8989233	8.07E-01	N/A	20	rs232286	43771100	9.09E-01	WFDC10B	-	4.50E-07	8.60E-01	4.94E-04	5999	
8	rs731685	59741470	8.55E-01	NSMAF	20	rs6121337	30145660	4.41E-01	N/A	-	4.50E-07	7.36E-01	4.27E-05	6000	
3	rs1461820	28766590	7.62E-01	N/A	5	rs32125	11397903	3.09E-01	CTNND2	-	4.50E-07	3.03E-01	1.38E-06	6001	
14	rs8009573	50622379	6.04E-01	TRIM9	18	rs16948569	5633771	6.99E-01	N/A	-	4.50E-07	7.11E-01	1.70E-04	6002	
2	rs4672495	62374748	2.76E-01	N/A	9	rs1381919	22927343	7.70E-01	N/A	-	4.50E-07	7.46E-01	3.07E-04	6003	
1	rs987293	18324487	3.40E-01	IGSF21	13	rs9523229	90454387	5.58E-01	N/A	-	4.50E-07	2.36E-01	4.24E-06	6004	
4	rs4689741	7472399	2.79E-01	SORCS2	14	rs6575528	95133483	1.34E-02	N/A	-	4.51E-07	6.33E-01	6.90E-06	6005	
3	rs6769422	11698224	7.77E-01	VGLL4	10	rs780825	16952140	2.29E-01	CUBN	-	4.51E-07	4.03E-01	1.28E-05	6006	
4	rs11942220	93558414	7.39E-01	GRID2	20	rs6044641	17058620	4.41E-01	N/A	-	4.51E-07	3.51E-01	6.09E-06	6007	
4	rs11942220	93558414	7.39E-01	GRID2	20	rs6135981	17059709	4.41E-01	N/A	-	4.51E-07	3.51E-01	6.09E-06	6008	
1	rs12760395	89812949	1.42E-01	LRRC8B	7	rs7793355	1.24E+08	2.39E-01	N/A	-	4.51E-07	3.78E-01	1.49E-03	6009	
1	rs922106	89798107	1.42E-01	LRRC8B	7	rs7793355	1.24E+08	2.39E-01	N/A	-	4.51E-07	3.78E-01	1.49E-03	6010	
1	rs4503376	1.08E+08	3.64E-01	VAV3	4	rs1860621	57781793	9.31E-01	N/A	-	4.51E-07	5.18E-01	3.49E-05	6011	
3	rs7619413	24799810	8.05E-01	N/A	11	rs1387153	92313476	4.04E-01	N/A	-	4.51E-07	8.24E-01	1.69E-05	6012	
9	rs4237258	75018527	9.63E-01	N/A	9	rs10819243	1.29E+08	7.51E-01	ZBTB34	-	4.51E-07	3.03E-01	8.36E-06	6013	
3	rs168150	1.13E+08	1.06E-01	TMPRSS7	15	rs7177672	36762585	6.21E-01	C15orf53	-	4.51E-07	1.68E-01	1.17E-03	6014	
2	rs17038242	49191301	4.24E-01	FSHR	14	rs1468503	74031105	2.41E-01	LTBP2	-	4.51E-07	7.47E-02	5.84E-07	6015	
1	rs7532282	1.58E+08	5.02E-01	N/A	3	rs12494196	85796570	2.19E-01	CADM2	-	4.51E-07	1.59E-01	1.49E-06	6016	
11	rs1790158	74858897	1.10E-01	GDPD5	16	rs1901819	79761689	9.85E-01	PKD1L2	-	4.51E-07	9.50E-01	4.70E-04	6017	
8	rs2427715	17510484	1.96E-01	PDGFRL	11	rs585768	93192503	4.36E-01	C11orf90	-	4.51E-07	2.95E-01	4.06E-06	6018	
10	rs982764	90759978	3.64E-01	ACTA2	11	rs7358476	86870272	1.10E-01	N/A	-	4.51E-07	4.14E-01	1.23E-03	6019	
16	rs11149582	82199007	9.04E-01	CDH13	22	rs16990207	31052207	4.59E-01	N/A	-	4.51E-07	5.32E-01	6.17E-05	6020	
3	rs355090	8621781	4.54E-02	C3orf32	7	rs17617404	12767576	7.11E-01	N/A	-	4.52E-07	N/A	N/A	6021	
13	rs9517971	99526202	9.41E-01	PCCA	18	rs3863524	18715280	7.36E-01	N/A	-	4.52E-07	N/A	N/A	6022	
9	rs690364	92396585	6.74E-01	N/A	11	rs4936808	1.23E+08	2.52E-01	N/A	-	4.52E-07	3.93E-01	2.44E-06	6023	
6	rs3117016	33203494	5.42E-01	HLA-DPB2	1	rs2144438	1.85E+08	4.91E-01	N/A	MHC	4.52E-07	6.87E-01	2.81E-04	6024	
1	rs2225875	1.8E+08	8.66E-01	CACN/A1E	7	rs1404807	1.45E+08	3.66E-01	N/A	-	4.52E-07	7.38E-01	5.93E-06	6025	
6	rs6919571	36494442	5.48E-01	PXT1	7	rs2694158	1.02E+08	8.06E-01	CUX1	MHC	4.52E-07	4.59E-01	1.95E-03	6026	
1	rs513086	72082573	4.73E-01	NEGR1	1	rs6540636	2.09E+08	5.24E-01	KCNH1	-	4.52E-07	8.74E-01	1.30E-06	6027	
8	rs1457185	4628942	8.07E-01	CSMD1	13	rs9530873	78681045	8.73E-02	N/A	-	4.52E-07	1.34E-01	1.70E-06	6028	
8	rs1457185	4628942	8.07E-01	CSMD1	13	rs9545015	78680916	8.73E-02	N/A	-	4.52E-07	1.34E-01	1.70E-06	6029	
7	rs10487725	31506482	5.17E-01	CCDC129	7	rs675242	47556332	3.71E-01	TNS3	-	4.52E-07	1.40E-01	4.08E-03	6030	
3	rs9851577	1.27E+08	1.08E-01	ALDH1L1	16	rs1991867	81076729	2.92E-02	N/A	-	4.52E-07	5.65E-01	1.04E-03	6031	
17	rs12453818	8775320	1.76E-01	PIK3R5	22	rs136958	26145491	2.90E-01	N/A	-	4.52E-07	9.24E-01	5.45E-05	6032	
6	rs2269706	30760851	1.54E-01	MDC1	6	rs6937313	97319863	2.80E-01	N/A	MHC	4.52E-07	2.45E-02	1.58E-07	6033	
17	rs11656925	66450507	5.02E-01	N/A	19	rs408687	22022963	6.99E-01	ZNF257	-	4.52E-07	2.48E-01	2.05E-03	6034	
17	rs11656925	66450507	5.02E-01	N/A	19	rs409835	22022511	6.99E-01	ZNF257	-	4.52E-07	2.48E-01	2.05E-03	6035	
17	rs11656925	66450507	5.02E-01	N/A	19	rs411020	22022942	6.99E-01	ZNF257	-	4.52E-07	2.48E-01	2.05E-03	6036	
11	rs549289	1.17E+08	8.02E-01	DSCAML1	15	rs1078001	56955157	2.85E-01	FAM63B	-	4.52E-07	1.67E-01	1.98E-03	6037	
1	rs12732780	60255648	3.34E-01	C1orf87	9	rs669441	27127242	4.87E-02	TEK	-	4.52E-07	N/A	N/A	6038	
13	rs1927544	26331248	7.04E-01	N/A	13	rs13378888	1.12E+08	9.06E-01	N/A	-	4.52E-07	4.92E-02	6.74E-07	6039	
3	rs9825812	1.28E+08	8.33E-01	N/A	10	rs7098301	70925096	2.13E-01	TSPAN15	-	4.52E-07	7.07E-01	4.26E-05	6040	
2	rs4671787	67261861	9.61E-01	N/A	12	rs1979743	75761099	1.41E-02	ZDHHC17	-	4.53E-07	5.27E-01	9.76E-04	6041	
6	rs851998	1.52E+08	3.84E-01	N/A	10	rs1659840	13142854	7.05E-01	N/A	-	4.53E-07	8.98E-01	2.17E-05	6042	
10	rs7069105	14884581	1.26E-01	CDNF	18	rs477347	3511704	1.90E-01	DLGAP1	-	4.53E-07	2.04E-02	1.89E-08	6043	
1	rs6699355	35157192	3.16E-01	DLGAP3	2	rs2577756	23455061	6.22E-01	KLHL29	-	4.53E-07	9.12E-01	5.08E-05	6044	
11	rs1790474	1.19E+08	4.13E-01	N/A	15	rs2957734	70048439	6.94E-01	MYO9A	-	4.53E-07	9.47E-01	1.53E-04	6045	
11	rs1790474	1.19E+08	4.13E-01	N/A	15	rs1481862	70032702	6.94E-01	MYO9A	-	4.53E-07	9.86E-01	1.38E-04	6046	

SNP A					SNP B						Interaction P			Ranking	Cluster in top 100 interactions
CHR	SNP	Location	gle locus P va	Gene	CHR	SNP	Location	ingle locus P valu	Gene	HC region	Stage 1	Stage 2	Combined		
11	rs1790474	1.19E+08	4.13E-01	N/A	15	rs2306489	70014525	6.94E-01	MYO9A	-	4.53E-07	9.86E-01	1.38E-04	6047	
1	rs951405	80293967	2.54E-01	N/A	3	rs7626500	1.55E+08	2.13E-01	N/A	-	4.53E-07	7.68E-02	4.36E-06	6048	
4	rs6822279	99964728	1.96E-01	N/A	11	rs10790395	1.2E+08	4.48E-01	GRIK4	-	4.53E-07	4.15E-01	1.31E-05	6049	
6	rs11969025	1.69E+08	5.32E-02	SMOC2	9	rs1332440	25291050	1.62E-01	N/A	-	4.53E-07	2.97E-01	2.88E-04	6050	
2	rs938095	1.12E+08	9.07E-01	N/A	7	rs7806966	1.04E+08	2.49E-02	LHFPL3	-	4.53E-07	7.69E-01	3.74E-04	6051	
6	rs2880404	1.69E+08	5.32E-02	SMOC2	9	rs1332440	25291050	1.62E-01	N/A	-	4.53E-07	N/A	N/A	6052	
9	rs1412059	1.21E+08	9.57E-01	N/A	18	rs627202	2282491	9.49E-01	N/A	-	4.53E-07	3.59E-01	2.03E-03	6053	
2	rs2861680	79006430	1.64E-01	N/A	11	rs4939020	55606111	2.52E-01	OR8I2	-	4.53E-07	5.08E-01	3.07E-04	6054	
2	rs17538082	2.09E+08	1.91E-01	N/A	7	rs1989886	20549647	1.18E-01	N/A	-	4.53E-07	6.38E-01	2.73E-04	6055	
6	rs11758699	1.44E+08	3.75E-01	AIG1	6	rs10782333	1.55E+08	9.71E-01	CNKSR3	-	4.53E-07	1.26E-01	8.24E-07	6056	
4	rs992363	1.76E+08	8.98E-01	N/A	8	rs4300027	6867985	7.89E-01	DEFA3	-	4.53E-07	3.95E-01	7.05E-04	6057	
4	rs992363	1.76E+08	8.98E-01	N/A	8	rs4512398	6868004	7.89E-01	DEFA3	-	4.53E-07	3.95E-01	7.05E-04	6058	
2	rs4477859	1.6E+08	9.69E-01	7-Mar	18	rs9948462	7066836	6.08E-01	LAMA1	-	4.53E-07	8.72E-01	1.54E-04	6059	
1	rs12746343	1.89E+08	7.62E-01	N/A	3	rs2197126	19398565	5.56E-01	KCNH8	-	4.53E-07	9.64E-01	2.46E-05	6060	
6	rs318457	2883121	8.67E-01	N/A	11	rs1491850	27706301	3.53E-01	BDNF	-	4.53E-07	1.83E-01	2.61E-06	6061	
3	rs10865655	70940116	2.58E-01	N/A	20	rs1537479	56260074	5.33E-01	PPP4R1L	-	4.53E-07	7.45E-01	6.43E-05	6062	
5	rs12653782	1.56E+08	9.52E-01	SGCD	17	rs7405830	73984363	8.13E-01	DN/AH17	-	4.53E-07	5.88E-01	1.02E-04	6063	
6	rs820089	35132673	2.90E-01	ANKS1A	9	rs287572	31994713	1.35E-01	N/A	MHC	4.53E-07	2.56E-01	1.29E-03	6064	
6	rs820089	35132673	2.90E-01	ANKS1A	9	rs287573	31994571	1.35E-01	N/A	MHC	4.53E-07	2.56E-01	1.29E-03	6065	
8	rs10110766	36928456	3.45E-01	KCNU1	12	rs345659	1.27E+08	6.04E-01	N/A	-	4.53E-07	2.56E-01	1.29E-03	6066	
6	rs820089	35132673	2.90E-01	ANKS1A	9	rs453809	32006697	1.35E-01	N/A	MHC	4.53E-07	8.85E-01	4.13E-05	6067	
6	rs820089	35132673	2.90E-01	ANKS1A	9	rs407791	32012207	1.35E-01	N/A	MHC	4.53E-07	N/A	N/A	6068	
6	rs2328537	1.44E+08	7.35E-01	PLAGL1	8	rs11782575	33614727	1.69E-02	N/A	-	4.54E-07	5.71E-02	2.68E-07	6069	
5	rs1485458	96856499	6.31E-01	N/A	13	rs723603	1.03E+08	7.61E-01	N/A	-	4.54E-07	1.90E-01	2.31E-03	6070	
9	rs1331346	12394591	8.13E-01	N/A	10	rs7916078	67519845	3.31E-02	CTNN/A3	-	4.54E-07	4.43E-01	5.34E-06	6071	
8	rs10216980	3032368	3.12E-01	CSMD1	8	rs1126681	97674976	8.90E-01	SDC2	-	4.54E-07	4.43E-01	3.16E-06	6072	
7	rs6949736	48330883	8.45E-02	ABCA13	10	rs11015882	27901946	4.28E-01	N/A	-	4.54E-07	5.98E-01	1.46E-05	6073	
1	rs12745240	1.6E+08	1.03E-02	ATF6	5	rs7701785	1.19E+08	3.79E-01	N/A	-	4.54E-07	2.53E-01	N/A	6074	
5	rs6555834	1.68E+08	1.49E-01	SLIT3	10	rs2505300	27830113	3.98E-01	RAB18	-	4.54E-07	9.76E-01	5.68E-05	6075	
6	rs2186061	1.26E+08	7.31E-01	N/A	5	rs2132537	78103740	5.06E-02	ARSB	-	4.54E-07	6.11E-01	1.95E-05	6076	
6	rs17053778	1.26E+08	7.31E-01	N/A	5	rs2132537	78103740	5.06E-02	ARSB	-	4.54E-07	6.12E-01	2.03E-05	6077	
1	rs2224153	95668595	1.47E-01	N/A	9	rs10757699	27940863	6.33E-01	LINGO2	-	4.54E-07	6.99E-01	2.86E-05	6078	
3	rs13093115	63312961	6.35E-01	SYNPR	12	rs11168337	46662797	8.45E-01	TMEM106C	-	4.54E-07	6.57E-01	1.10E-04	6079	
6	rs2496379	48354822	9.46E-01	N/A	9	rs12685240	98118935	3.88E-01	SLC35D2	-	4.54E-07	5.53E-01	1.36E-05	6080	
5	rs6890478	1.72E+08	2.33E-01	UBTD2	13	rs4771482	1.04E+08	3.54E-01	N/A	-	4.54E-07	6.66E-01	9.71E-06	6081	
7	rs3111297	1.53E+08	6.87E-01	N/A	16	rs11076898	5215560	5.53E-01	N/A	-	4.54E-07	7.88E-02	5.75E-07	6082	
6	rs2842363	7212581	1.11E-02	RREB1	9	rs2769195	76560591	6.02E-01	TRPM6	-	4.54E-07	3.52E-01	1.77E-03	6083	
1	rs2240335	17547124	1.17E-02	PADI4	13	rs568474	71822612	7.95E-01	N/A	-	4.54E-07	6.61E-01	2.37E-04	6084	
6	rs3131003	31201461	6.51E-01	C6orf15	6	rs396243	31383153	2.17E-01	N/A	MHC	4.54E-07	N/A	N/A	6085	
6	rs957387	15429838	7.84E-01	JARID2	21	rs2837808	41045971	4.06E-01	DSCAM	-	4.55E-07	1.89E-01	2.33E-03	6086	
6	rs957387	15429838	7.84E-01	JARID2	21	rs9976793	41042747	4.06E-01	DSCAM	-	4.55E-07	1.89E-01	2.33E-03	6087	
3	rs9876304	1.33E+08	7.53E-01	N/A	9	rs1334069	3592018	4.85E-01	N/A	-	4.55E-07	6.11E-01	5.31E-06	6088	
7	rs4720301	4480332	3.82E-01	N/A	10	rs1762004	29055329	3.43E-01	N/A	-	4.55E-07	8.29E-01	4.80E-05	6089	
2	rs1871780	1.58E+08	2.96E-01	N/A	9	rs540412	96936436	1.65E-01	FANCC	-	4.55E-07	7.17E-01	1.42E-04	6090	
2	rs3892295	20492363	8.69E-01	RHOB	9	rs1930384	80756494	1.09E-01	N/A	-	4.55E-07	4.16E-01	2.08E-05	6091	
6	rs675497	75114270	6.59E-01	N/A	8	rs7010570	1.22E+08	9.84E-01	N/A	-	4.55E-07	N/A	N/A	6092	
2	rs6707186	1.28E+08	9.86E-01	MYO7B	21	rs2831689	28569675	2.54E-01	N/A	-	4.55E-07	6.94E-01	4.50E-05	6093	
17	rs2107565	20029084	5.41E-01	CYTSB	18	rs1493917	29646831	1.67E-01	N/A	-	4.55E-07	7.10E-01	7.49E-05	6094	
17	rs2703807	20049781	5.41E-01	CYTSB	18	rs1493917	29646831	1.67E-01	N/A	-	4.55E-07	7.10E-01	7.49E-05	6095	
1	rs1074541	89282087	8.51E-02	GBP1	13	rs2321997	58561359	4.61E-01	N/A	-	4.55E-07	5.09E-01	1.30E-05	6096	
9	rs7045254	27946578	7.60E-01	LINGO2	12	rs4590981	1.05E+08	5.73E-01	N/A	-	4.55E-07	7.11E-01	4.16E-04	6097	
1	rs535857	65264782	9.79E-01	N/A	10	rs7075837	21066895	6.94E-01	N/A	-	4.55E-07	9.22E-01	3.46E-04	6098	
1	rs1134647	2.13E+08	8.01E-01	SMYD2	13	rs10492664	1.08E+08	6.04E-02	N/A	-	4.55E-07	5.18E-01	3.31E-04	6099	
1	rs12124666	2.13E+08	8.01E-01	SMYD2	13	rs10492664	1.08E+08	6.04E-02	N/A	-	4.55E-07	5.18E-01	3.31E-04	6100	
2	rs1914507	76863507	7.98E-01	LRRTM4	10	rs4751955	1.18E+08	4.04E-01	GFRA1	-	4.55E-07	6.99E-01	3.32E-04	6101	
7	rs11763033	26611167	4.89E-01	N/A	12	rs2270484	61400423	7.55E-02	PPM1H	-	4.55E-07	9.94E-01	3.62E-05	6102	
3	rs4511884	14616816	3.22E-01	N/A	19	rs2270048	2277215	4.97E-01	SPPL2B	-	4.55E-07	3.58E-01	2.16E-06	6103	
1	rs4915746	61725162	2.89E-01	N/A	3	rs1017788	1.66E+08	2.58E-01	N/A	-	4.55E-07	3.93E-01	1.34E-03	6104	
5	rs339424	14034968	8.30E-01	N/A	9	rs7042473	98386391	4.94E-01	CDC14B	-	4.55E-07	9.60E-01	1.10E-04	6105	
1	rs12024734	48737857	8.69E-01	N/A	2	rs4464317	69290795	6.47E-02	ANTXR1	-	4.55E-07	3.14E-01	2.48E-04	6106	
2	rs4618005	1.82E+08	1.34E-01	N/A	3	rs12496951	1.02E+08	5.55E-01	ABI3BP	-	4.55E-07	9.16E-01	1.49E-04	6107	
5	rs17218390	66316182	6.50E-01	MAST4	20	rs8121494	45927980	5.59E-01	N/A	-	4.56E-07	3.90E-02	6.94E-03	6108	
9	rs4877367	89388071	1.01E-01	DAPK1	12	rs1317758	1.15E+08	7.72E-01	N/A	-	4.56E-07	5.38E-01	6.06E-06	6109	
1	rs2296799	2.29E+08	1.96E-01	COG2	5	rs4895350	1.22E+08	7.13E-01	SNCAIP	-	4.56E-07	7.37E-01	3.50E-05	6110	
2	rs1440063	2.24E+08	9.63E-01	N/A	20	rs1204765	49153010	7.86E-01	N/A	-	4.56E-07	8.51E-01	5.00E-05	6111	
10	rs2744069	42694232	1.62E-01	N/A	21	rs724128	18617132	3.79E-01	PRSS7	-	4.56E-07	9.63E-01	1.85E-04	6112	
14	rs7154215	48879212	1.35E-02	N/A	16	rs7205534	80759119	5.53E-01	MPHOSPH6	-	4.56E-07	4.51E-01	1.18E-05	6113	
1	rs6664865	1.61E+08	7.45E-01	N/A	12	rs1450072	96748321	9.73E-01	N/A	-	4.56E-07	6.75E-01	1.34E-04	6114	
10	rs881631	77636503	8.28E-01	C10orf11	11	rs7944652	32818969	5.82E-01	PRRG4	-	4.56E-07	6.03E-01	9.15E-06	6115	
6	rs2130357	27994809	6.14E-01	OR2B2	1	rs7539245	1.05E+08	4.73E-01	N/A	MHC	4.56E-07	1.52E-01	7.47E-06	6116	
2	rs4673807	2.15E+08	5.15E-01	SPAG16	11	rs1528758	90482436	2.78E-01	N/A	-	4.56E-07	7.02E-01	1.56E-04	6117	
4	rs6535244	82509042	1.95E-01	N/A	13	rs4942311	43765641	1.16E-01	N/A	-	4.56E-07	2.27E-01	1.56E-03	6118	
5	rs930413	1.25E+08	5.72E-01	N/A	8	rs1461223	28195157	3.61E-01	N/A	-	4.56E-07	7.22E-01	5.02E-05	6119	
4	rs4692317	25646700	5.87E-01	N/A	17	rs1991548	49927900	6.13E-01	N/A	-	4.56E-07	4.71E-01	4.22E-06	6120	
12	rs7307618	86287617	2.51E-01	N/A	22	rs7287569	41483090	8.60E-01	N/A	-	4.56E-07	6.52E-01	2.08E-04	6121	
12	rs7968713	86286718	2.51E-01	N/A	22	rs7287569	41483090	8.60E-01	N/A	-	4.56E-07	6.52E-01	2.08E-04	6122	
5	rs4866542	3335671	8.29E-01	N/A	16	rs4887899	75830083	2.84E-01	N/A	-	4.56E-07	N/A	N/A	6123	
1	rs12040542	9514086	4.02E-01	SLC25A33	7	rs1203570	40799122	1.63E-01	N/A	-	4.56E-07	4.76E-01	5.56E-04	6124	
8	rs923429	1.12E+08	3.60E-01	N/A	12	rs11112403	1.04E+08	4.10E-01	APPL2	-	4.56E-07	7.56E-01	1.39E-04	6125	
6	rs9473021	47235282	7.88E-01	N/A	16	rs1872678	49551265	8.88E-01	N/A	-	4.56E-07	9.26E-01	8.30E-05	6126	
1	rs1062827	1.59E+08	1.23E-01	F11R	1	rs1253618	2.35E+08	5.72E-01	EDARADD	-	4.57E-07	8.77E-02	1.70E-06	6127	
1	rs11807930	1.59E+08	1.23E-01	F11R	1	rs1253618	2.35E+08	5.72E-01	EDARADD	-	4.57E-07	8.77E-02	1.70E-06	6128	
1	rs2297221	1.59E+08	1.23E-01	F11R	1	rs1253618	2.35E+08	5.72E-01	EDARADD	-	4.57E-07	8.77E-02	1.70E-06	6129	
6	rs9348862	31360726	7.72E-01	HLA-C	6	rs2394990	31448538	6.80E-02	HLA-B	MHC	4.57E-07	3.05E-01	1.43E-05	6130	
9	rs2076904	1.29E+08	2.31E-02	FAM129B	14	rs1955634	57555964	4.62E-01	C14orf37	-	4.57E-07	3.05E-01	1.43E-05	6131	
6	rs9357122	31368324	7.72E-01	N/A	6	rs2394990	31448538	6.80E-02	HLA-B	MHC	4.57E-07	4.07E-01	5.29E-06	6132	
6	rs1209198	46125585	8.77E-01	CLIC5	10	rs11197936	1.19E+08	1.27E-01	SLC18A2	-	4.57E-07	3.05E-01	9.82E-06	6133	
2	rs10169420	1.83E+08	4.31E-01	N/A	12	rs2645979	82541174	9.79E-01	N/A	-	4.57E-07	4.05E-01	1.75E-05	6134	
8	rs16886892	87202047	2.31E-01	ATP6V0D2	20	rs1005351	7020555	8.94E-01	N/A	-	4.57E-07	6.68E-01	5.86E-04	6135	
12	rs12312186	66857437	5.09E-02	IFNG	17	rs16974282	65203957	5.52E-01	N/A	-	4.57E-07	1.06E-01	1.69E-03	6136	
5	rs251235	1.72E+08	1.80E-01	C5orf41	12	rs1881227	87562931	9.76E-01	N/A	-	4.57E-07	2.88E-01	7.72E-06	6137	
6	rs1419638	29463498	1.74E-01	OR12D3	2	rs2166855	2.39E+08	4.94E-01	N/A	MHC	4.57E-07	6.66E-01	2.92E-05	6138	
7	rs2062403	68146581	5.93E-01	N/A	7	rs917717	69161370	3.10E-01	AUTS2	-	4.57E-07	1.94E-01	1.01E-03	6139	
5	rs2560828	16619212	7.74E-01	FAM134B	9	rs7034380	14898056	5.82E-01	FREM1	-	4.57E-07	4.22E-01	7.57E-04	6140	
7	rs1551634	1.56E+08	9.72E-01	N/A	9	rs1806706	97389742	6.23E-01	N/A	-	4.57E-07	9.44E-01	4.69E-05	6141	
7	rs3808035	1.03E+08	8.82E-03	RELN	22	rs4345013	25711837	4.45E-02	N/A	-	4.57E-07	3.72E-01	3.26E-03	6142	
3	rs9872768	1.48E+08	1.86E-01	N/A	4	rs16990391	34524071	3.13E-01	N/A	-	4.57E-07	6.76E-01	2.99E-04	6143	
6	rs4495304	31188697	4.80E-02	C6orf15	6	rs130067	31226490	8.62E-01	TCF19	MHC	4.57E-07	5.96E-02	4.62E-07	6144	
1	rs10863652	2.2E+08	7.39E-01	DUSP10	2	rs13405506	2.38E+08	6.55E-01	RAMP1	-	4.57E-07	2.34E-01	2.15E-06	6145	
2	rs17036168	47133109	1.47E-01	TTC7A	12	rs7302422	48931738	6.44E-02	LIMA1	-	4.57E-07	7.24E-01	5.73E-04	6146	
7	rs6961353	55501341	9.62E-02	VOPP1	18	rs9945709	68529825	8.59E-01	N/A	-	4.57E-07	7.54E-01	1.88E-04	6147	
3	rs1541614	1.87E+08	1.72E-01	DGKG	20	rs465757	15528283	9.21E-01	MACROD2	-	4.57E-07	6.91E-01	3.57E-05	6148	
2	rs2380706	15897443	7.57E-01	N/A	10	rs3858284	88520461	1.99E-01	BMPR1A	-	4.57E-07	9.78E-01	1.46E-04	6149	
5	rs16902128	85527586	8.83E-01	N/A	12	rs11829762	1.06E+08	5.39E-01	CRY1	-	4.57E-07	2.48E-02	3.93E-07	6150	
1	rs11117745	2.15E+08	2.20E-01	ESRRG	8	rs11987973	1.16E+08	1.92E-01	N/A	-	4.57E-07	6.86E-01	1.36E-05	6151	
1	rs12074413	51388967	8.27E-02	C1orf185	3	rs775229	1.14E+08	4.63E-01	WDR52	-	4.58E-07	7.05E-02	3.57E-06	6152	
7	rs194849	1.04E+08	8.54E-01	ORC5L	13	rs4885537	77875455	1.46E-01	N/A	-	4.58E-07	5.44E-01	1.30E-04	6153	
6	rs2444830	81397894	2.20E-01	N/A	2	rs10930467	1.72E+08	2.77E-01	N/A	-	4.58E-07	9.38E-01	4.17E-05	6154	
4	rs2635205	1.19E+08	6.83E-01	N/A	7	rs741304	36769525	9.92E-01	N/A	-	4.58E-07	8.25E-01	1.35E-04	6155	
3	rs9876553	1.62E+08	4.85E-01	NMD3	8	rs4977114	1.44E+08	6.95E-01	N/A	-	4.58E-07	8.74E-02	2.36E-06	6156	
3	rs9290075	1.63E+08	4.85E-01	N/A	8	rs4977114	1.44E+08	6.95E-01	N/A	-	4.58E-07	1.01E-01	2.99E-06	6157	
6	rs231948	1.56E+08	5.37E-01	NOX3	12	rs2900367	16028049	6.09E-01	DERA	-	4.58E-07	5.34E-02	7.33E-03	6158	

SNP A					SNP B						Interaction P			Ranking	Cluster in top 100 interactions
CHR	SNP	Location	gle locus P va	Gene	CHR	SNP	Location	ingle locus P valu	Gene	HC region	Stage 1	Stage 2	Combined		
1	rs326910	96624140	9.19E-01	N/A	5	rs2372183	67233711	7.49E-01	N/A	-	4.58E-07	1.75E-01	2.59E-03	6159	
6	rs4715902	14573278	7.81E-01	N/A	10	rs7090670	1.03E+08	4.46E-01	BTRC	-	4.58E-07	4.92E-01	4.29E-04	6160	
2	rs7589636	57543537	5.47E-01	N/A	2	rs730402	59948210	2.73E-02	N/A	-	4.59E-07	1.47E-03	2.11E-09	6161	
1	rs163401	1.93E+08	3.90E-02	N/A	13	rs2439959	77555600	3.52E-02	N/A	-	4.59E-07	5.14E-02	3.77E-07	6162	
1	rs2039759	1.92E+08	4.44E-01	N/A	22	rs131853	46141822	2.23E-01	N/A	-	4.59E-07	1.09E-01	4.16E-07	6163	
5	rs3762991	52121160	2.81E-01	ITGA1	14	rs970662	32243135	2.78E-01	AKAP6	-	4.59E-07	5.31E-01	3.98E-06	6164	
4	rs4241918	1.4E+08	4.18E-01	N/A	10	rs4146592	98711592	5.01E-02	LCOR	-	4.59E-07	8.69E-01	1.15E-04	6165	
3	rs6414591	59087928	4.45E-01	N/A	4	rs2940779	1.26E+08	1.11E-01	FAT4	-	4.59E-07	2.46E-01	2.17E-06	6166	
5	rs17745927	1.7E+08	9.72E-01	KCNIP1	7	rs6976331	1.28E+08	4.24E-01	N/A	-	4.59E-07	3.40E-01	9.20E-06	6167	
8	rs1985958	1.25E+08	6.80E-01	KLHL38	11	rs7951026	1.3E+08	4.99E-01	N/A	-	4.59E-07	1.85E-01	6.44E-04	6168	
1	rs7542125	2.01E+08	4.99E-01	KLHL12	1	s10926744	2.41E+08	4.05E-01	PLD5	-	4.59E-07	1.93E-01	4.79E-06	6169	
9	rs10819907	97938109	8.46E-01	N/A	12	s11112414	1.04E+08	4.48E-01	APPL2	-	4.59E-07	5.83E-01	4.44E-04	6170	
5	rs1037112	13427095	1.63E-01	N/A	13	rs14067	1.13E+08	4.50E-01	ADPRHL1	-	4.59E-07	5.88E-01	6.49E-04	6171	
1	rs241301	2.27E+08	8.36E-01	N/A	11	rs1160627	37068443	3.76E-01	N/A	-	4.59E-07	6.58E-01	5.81E-05	6172	
1	rs2639760	2.27E+08	8.36E-01	N/A	11	rs1160627	37068443	3.76E-01	N/A	-	4.59E-07	6.58E-01	5.81E-05	6173	
9	rs10819490	1.31E+08	6.98E-01	N/A	14	s10140947	99110135	3.75E-01	CCDC85C	-	4.59E-07	5.41E-01	4.62E-04	6174	
5	rs10069382	31957022	1.47E-01	PDZD2	17	rs7220419	43263404	6.44E-01	OSBPL7	-	4.59E-07	N/A	N/A	6175	
3	rs9881202	15256030	4.74E-01	SH3BP5	22	rs7364173	49023156	5.00E-01	HDAC10	-	4.59E-07	4.31E-01	1.06E-05	6176	
5	rs7724310	1.6E+08	8.22E-01	N/A	9	rs3789281	1.18E+08	5.32E-01	PAPPA	-	4.59E-07	6.27E-01	8.52E-06	6177	
16	rs12447690	86855625	1.91E-01	N/A	21	rs2834484	34738811	1.90E-01	KCNE1	-	4.59E-07	1.27E-01	2.52E-07	6178	
1	rs290826	97246295	2.48E-01	N/A	8	rs2978257	30620257	6.01E-01	GTF2E2	-	4.59E-07	3.01E-01	2.90E-05	6179	
4	rs6834348	1.19E+08	7.30E-01	N/A	5	rs2114961	1.35E+08	3.60E-01	N/A	-	4.59E-07	6.60E-01	7.52E-05	6180	
9	rs1375209	1.05E+08	7.36E-01	N/A	21	rs9983113	39237186	5.50E-01	N/A	-	4.59E-07	7.42E-03	2.05E-02	6181	
7	rs343719	35593876	3.67E-01	N/A	19	rs4805602	35897140	2.21E-01	N/A	-	4.59E-07	3.38E-01	1.06E-03	6182	
14	rs2239281	72090506	7.35E-01	RGS6	20	rs6066320	45487220	5.11E-01	N/A	-	4.59E-07	5.19E-01	1.70E-03	6183	
10	rs16932566	77131018	4.12E-01	N/A	14	rs6571501	31797984	8.20E-02	N/A	-	4.60E-07	2.42E-01	3.38E-06	6184	
10	rs3858154	71268065	7.98E-02	COL13A1	14	rs1950663	30024969	2.00E-01	N/A	-	4.60E-07	9.89E-01	8.80E-05	6185	
5	rs1348473	1.2E+08	8.23E-01	N/A	14	rs1958059	33256781	3.87E-01	NPAS3	-	4.60E-07	4.69E-02	8.69E-08	6186	
11	rs10830545	89950000	5.49E-01	N/A	13	rs7334528	60087939	4.60E-01	N/A	-	4.60E-07	5.72E-02	4.04E-07	6187	
2	rs1018124	2.34E+08	4.52E-01	UGT1A10	14	s10137862	50518114	5.13E-01	TRIM9	-	4.60E-07	4.22E-01	7.24E-04	6188	
3	rs6442772	3009425	7.40E-01	CNTN4	7	s10488426	1.34E+08	3.68E-01	LRGUK	-	4.60E-07	9.99E-01	4.17E-05	6189	
14	rs1952576	43992264	1.80E-01	N/A	14	rs7156399	95104682	7.38E-01	N/A	-	4.60E-07	3.56E-01	1.84E-04	6190	
6	rs3757169	1.37E+08	9.38E-01	PEX7	8	rs2979419	62167326	2.62E-01	N/A	-	4.60E-07	6.88E-01	6.27E-04	6191	
1	rs11264177	35819589	6.66E-01	TFAP2E	17	rs4795343	34472851	4.95E-01	PLXDC1	-	4.60E-07	3.81E-01	5.12E-06	6192	
6	rs1449679	1.56E+08	8.18E-01	N/A	2	rs6431983	9228055	5.24E-01	N/A	-	4.60E-07	5.26E-01	9.31E-05	6193	
9	rs1751449	22186863	3.75E-02	N/A	20	rs3212191	42472196	7.21E-01	HNF4A	-	4.60E-07	1.87E-01	7.68E-04	6194	
12	rs1812730	1.24E+08	2.04E-01	NCOR2	15	rs563940	41986601	4.96E-01	FRMD5	-	4.60E-07	3.33E-01	2.65E-05	6195	
8	rs10092844	72375844	9.01E-01	EYA1	15	rs7183955	58836861	5.11E-01	RORA	-	4.60E-07	4.62E-01	1.72E-03	6196	
6	rs4710090	1.67E+08	8.67E-02	RPS6KA2	17	s11651877	1799581	2.22E-01	RTN4RL1	-	4.60E-07	6.58E-01	1.15E-04	6197	
9	rs10810402	15576534	1.54E-01	C9orf93	13	rs7328894	60686950	6.37E-01	N/A	-	4.60E-07	9.21E-01	3.01E-05	6198	
9	rs10810402	15576534	1.54E-01	C9orf93	13	rs7329289	60687123	6.37E-01	N/A	-	4.60E-07	9.21E-01	3.01E-05	6199	
4	rs4862611	1.87E+08	1.86E-03	N/A	11	rs7928596	81675813	7.26E-02	N/A	-	4.61E-07	1.82E-01	6.75E-04	6200	
6	rs12661581	1.48E+08	8.47E-01	N/A	12	s12425791	653745	3.72E-01	NINJ2	-	4.61E-07	5.70E-01	3.96E-05	6201	
6	rs9384817	1.12E+08	4.04E-01	WISP3	22	rs5751997	23517544	1.79E-01	PIWIL3	-	4.61E-07	7.68E-01	2.68E-04	6202	
16	rs7500939	62349448	1.60E-01	N/A	17	rs4789374	72423955	4.59E-01	MGAT5B	-	4.61E-07	6.61E-01	5.69E-04	6203	
14	rs4272953	48540230	9.05E-01	N/A	16	s10514506	79278578	9.46E-01	CDYL2	-	4.61E-07	3.12E-01	2.98E-06	6204	
6	rs3117004	33204744	4.44E-01	HLA-DPB2	1	rs2144438	1.85E+08	4.91E-01	N/A	MHC	4.61E-07	6.43E-01	3.16E-04	6205	
4	rs1872293	1.43E+08	3.52E-01	INPP4B	8	rs4733166	33691038	2.68E-02	N/A	-	4.61E-07	9.64E-01	1.33E-04	6206	
2	rs6545648	57511448	3.54E-01	N/A	2	rs7349361	59930506	1.93E-02	N/A	-	4.61E-07	1.09E-02	1.87E-08	6207	
11	rs952131	1.29E+08	6.41E-01	N/A	19	rs6511680	24017416	3.77E-01	N/A	-	4.61E-07	2.58E-01	8.37E-06	6208	
6	rs1062470	31192414	5.89E-01	C6orf15	14	rs8014790	97513859	2.36E-01	C14orf64	MHC	4.61E-07	1.39E-01	9.62E-07	6209	
2	rs2580344	1.22E+08	8.76E-01	TFCP2L1	10	rs6597745	1.28E+08	6.87E-01	ADAM12	-	4.61E-07	2.05E-01	1.02E-06	6210	
12	rs10860416	98216138	1.43E-01	ANKS1B	14	rs1959482	70197453	9.06E-02	TTC9	-	4.61E-07	4.83E-01	3.57E-06	6211	
10	rs1888606	90447613	9.07E-01	LIPF	21	rs2837682	40826362	6.19E-01	DSCAM	-	4.61E-07	4.91E-01	1.10E-04	6212	
4	rs705117	72826979	5.15E-01	GC	13	s11618409	23658270	3.19E-01	SPATA13	-	4.61E-07	3.98E-01	9.88E-04	6213	
10	rs1679414	4495344	2.71E-01	N/A	16	rs8047711	81225172	3.35E-01	CDH13	-	4.61E-07	8.51E-01	1.02E-04	6214	
3	rs2046227	1.02E+08	6.40E-01	GPR128	10	rs4880745	1008585	6.63E-01	GTPBP4	-	4.62E-07	1.71E-02	3.32E-08	6215	
3	rs2046227	1.02E+08	6.40E-01	GPR128	10	rs7069123	1014214	6.63E-01	GTPBP4	-	4.62E-07	2.08E-02	4.18E-08	6216	
5	rs375183	31772339	5.44E-01	N/A	14	rs7149097	1.03E+08	5.74E-01	CDC42BPB	-	4.62E-07	2.89E-01	1.07E-03	6217	
5	rs25767	1.23E+08	5.69E-01	N/A	10	s10997485	68458948	1.45E-01	CTNN/A3	-	4.62E-07	7.72E-01	5.79E-05	6218	
7	rs9791389	1224774	5.76E-01	UNCX	16	rs2283540	24038271	6.59E-01	PRKCB	-	4.62E-07	7.95E-01	1.66E-04	6219	
5	rs4425512	18990653	9.41E-01	N/A	5	rs1363258	1.03E+08	6.55E-01	N/A	-	4.62E-07	8.83E-01	2.81E-04	6220	
5	rs4309976	18990473	9.41E-01	N/A	5	rs1363258	1.03E+08	6.55E-01	N/A	-	4.62E-07	9.95E-01	1.95E-04	6221	
3	rs2062834	1.15E+08	5.25E-01	ZNF80	19	rs4807249	2331982	7.92E-01	TMPRSS9	-	4.62E-07	9.69E-01	1.59E-04	6222	
7	rs3735613	1.07E+08	8.56E-01	PRKAR2B	22	rs7289126	36958252	3.27E-01	N/A	-	4.62E-07	5.01E-01	8.88E-06	6223	
1	rs6672985	2.05E+08	9.49E-01	N/A	4	rs9996449	2371145	8.31E-01	ZFYVE28	-	4.62E-07	1.53E-01	1.37E-03	6224	
7	rs4947404	52263829	1.33E-01	N/A	10	rs1144518	28664142	6.10E-01	N/A	-	4.62E-07	8.77E-01	3.16E-04	6225	
1	rs6671446	63681568	4.37E-01	ITGB3BP	16	rs2641674	83510605	7.87E-01	CRISPLD2	-	4.62E-07	4.05E-01	3.88E-04	6226	
1	rs10779849	2.29E+08	3.61E-01	COG2	10	rs2487707	70435098	6.93E-01	KIAA1279	-	4.62E-07	3.95E-01	4.50E-04	6227	
10	rs2163673	90477499	7.09E-01	LIPK	21	rs2834278	34141871	2.22E-01	ITSN1	-	4.62E-07	9.59E-02	1.17E-02	6228	
16	rs7500355	84634601	7.85E-01	N/A	17	rs8080725	22116859	8.98E-01	N/A	-	4.62E-07	3.08E-01	9.40E-04	6229	
4	rs9307912	1.39E+08	6.67E-01	PCDH18	9	rs7035310	98308511	1.78E-01	CDC14B	-	4.62E-07	3.37E-01	2.94E-06	6230	
1	rs17102086	46495526	7.73E-01	RAD54L	13	rs9572575	70408794	5.98E-02	N/A	-	4.62E-07	8.94E-01	7.26E-05	6231	
7	rs7786719	43043641	2.38E-01	N/A	11	rs609036	58210105	8.98E-03	N/A	-	4.63E-07	3.60E-01	1.04E-03	6232	
1	rs2273367	63793684	4.38E-01	EFCAB7	16	rs2641674	83510605	7.87E-01	CRISPLD2	-	4.63E-07	4.05E-01	3.88E-04	6233	
1	rs6693255	63772414	4.38E-01	ITGB3BP	16	rs2641674	83510605	7.87E-01	CRISPLD2	-	4.63E-07	4.05E-01	3.88E-04	6234	
2	rs1362466	2.07E+08	7.06E-01	GPR1	9	s10992192	91926950	7.12E-01	N/A	-	4.63E-07	4.58E-01	3.81E-04	6235	
11	rs3107606	1.22E+08	9.25E-01	C11orf63	18	rs2364890	65595729	6.97E-01	DOK6	-	4.63E-07	7.29E-01	1.28E-04	6236	
5	rs7379517	1.8E+08	3.10E-01	N/A	14	s12878113	80102281	5.96E-01	C14orf145	-	4.63E-07	7.96E-01	1.50E-04	6237	
5	rs7380299	1.8E+08	3.10E-01	N/A	14	s12878113	80102281	5.96E-01	C14orf145	-	4.63E-07	7.96E-01	1.50E-04	6238	
6	rs9455852	1.69E+08	6.79E-01	N/A	11	rs1540082	85979365	8.72E-01	ME3	-	4.63E-07	3.03E-01	7.55E-04	6239	
4	rs218268	55105844	4.94E-01	N/A	10	rs1801234	17019667	4.15E-04	CUBN	-	4.63E-07	9.86E-01	5.44E-05	6240	
4	rs218269	55105896	4.94E-01	N/A	10	rs1801234	17019667	4.15E-04	CUBN	-	4.63E-07	9.88E-01	5.50E-05	6241	
3	rs6796803	1.88E+08	2.59E-02	KNG1	10	rs7905723	1.08E+08	2.35E-01	SORCS1	-	4.63E-07	6.10E-01	3.36E-04	6242	
2	rs7590923	1.46E+08	1.47E-01	N/A	9	rs582337	92893278	1.05E-02	N/A	-	4.63E-07	3.08E-01	6.72E-04	6243	
10	rs11254314	17058324	1.98E-01	CUBN	11	rs521191	94524021	6.86E-01	ENDOD1	-	4.63E-07	4.30E-01	3.55E-06	6244	
15	rs4966036	97247272	8.94E-01	IGF1R	20	rs6093838	41510850	7.02E-01	SFRS6	-	4.63E-07	7.62E-01	5.40E-05	6245	
2	rs318369	80342285	6.96E-01	CTNN/A2	3	rs973538	73989561	8.85E-01	N/A	-	4.63E-07	8.98E-01	3.57E-04	6246	
7	rs10280848	67742124	8.09E-01	N/A	10	rs7069921	26258799	4.96E-01	MYO3A	-	4.63E-07	2.39E-01	9.48E-04	6247	
2	rs701492	1.71E+08	4.00E-01	GAD1	3	s12630512	85183388	9.01E-01	CADM2	-	4.63E-07	4.06E-01	8.30E-04	6248	
2	rs2577999	1.83E+08	4.99E-01	N/A	12	rs1603572	82627746	9.51E-01	N/A	-	4.63E-07	1.49E-01	3.01E-06	6249	
2	rs2971890	1.83E+08	4.99E-01	N/A	12	rs1603572	82627746	9.51E-01	N/A	-	4.63E-07	1.49E-01	3.01E-06	6250	
12	rs4768560	43256238	4.05E-01	NELL2	18	rs535796	22574324	9.92E-01	N/A	-	4.63E-07	3.28E-01	3.39E-06	6251	
5	rs159009	58002899	6.05E-01	RAB3C	14	s11621843	92185877	4.54E-01	RIN3	-	4.64E-07	9.47E-01	9.76E-05	6252	
5	rs293031	57999932	6.05E-01	RAB3C	14	s11621843	92185877	4.54E-01	RIN3	-	4.64E-07	9.47E-01	9.76E-05	6253	
11	rs4545549	11237494	8.89E-01	GALNTL4	14	rs6573766	23921886	4.11E-01	NFATC4	-	4.64E-07	8.45E-01	3.76E-04	6254	
15	rs8025338	35681159	4.29E-01	N/A	22	rs4824083	48485654	2.32E-01	N/A	-	4.64E-07	9.15E-01	5.68E-05	6255	
11	rs7939909	42612422	9.53E-01	N/A	20	rs7264443	46087381	9.11E-01	N/A	-	4.64E-07	3.82E-02	3.77E-07	6256	
6	rs9376375	1.39E+08	5.65E-01	ECT2L	7	rs1670340	1.58E+08	1.04E-02	PTPRN2	-	4.64E-07	5.15E-01	2.75E-05	6257	
8	rs11986414	1734357	3.57E-01	CLN8	19	rs7257025	55848043	6.90E-02	SYT3	-	4.64E-07	1.53E-01	3.66E-03	6258	
12	rs12425217	1.28E+08	3.66E-01	TMEM132D	14	s17116495	45389174	9.01E-01	N/A	-	4.64E-07	9.35E-01	6.99E-05	6259	
3	rs10804661	1.4E+08	4.94E-01	FOXL2	3	rs7641416	1.92E+08	5.75E-01	N/A	-	4.64E-07	7.75E-01	7.20E-05	6260	
3	rs10804661	1.4E+08	4.94E-01	FOXL2	3	rs2363961	1.92E+08	5.75E-01	N/A	-	4.64E-07	7.90E-01	7.62E-05	6261	
6	rs1538185	1.3E+08	5.30E-01	N/A	21	rs2825529	19634654	1.41E-01	N/A	-	4.64E-07	7.83E-01	1.64E-04	6262	
16	rs12445596	12718185	2.27E-01	CPPED1	20	rs6073055	41406604	1.64E-01	N/A	-	4.64E-07	9.25E-01	1.67E-04	6263	
1	rs2336938	2.05E+08	7.38E-01	N/A	7	s12539814	1.44E+08	6.60E-01	TPK1	-	4.64E-07	3.24E-01	8.86E-06	6264	
4	rs4693676	85301318	9.16E-01	N/A	20	rs2423587	11654310	8.27E-01	N/A	-	4.65E-07	3.00E-01	1.17E-03	6265	
6	rs959619	1.14E+08	6.24E-01	MARCKS	1	rs2131887	2.19E+08	9.42E-01	N/A	-	4.65E-07	9.65E-01	1.10E-04	6266	
3	rs9290392	1.72E+08	7.90E-01	SLC7A14	11	rs2282661	20648784	5.32E-01	NELL1	-	4.65E-07	7.52E-01	9.53E-05	6267	
6	rs1062470	31192414	5.89E-01	C6orf15	6	rs9368675	31380136	7.91E-01	N/A	MHC	4.65E-07	1.10E-01	2.21E-06	6268	
2	rs4536605	2.36E+08	7.12E-01	N/A	3	s17709863	4671646	7.33E-01	ITPR1	-	4.65E-07	6.94E-01	2.25E-04	6269	
14	rs6573467	62471338	4.83E-02	KCNH5	18	rs1367865	48215053	8.55E-01	DCC	-	4.65E-07	9.04E-01	6.43E-05	6270	

SNP A					SNP B						Interaction P			Ranking	Cluster in top 100 interactions
CHR	SNP	Location	gle locus P va	Gene	CHR	SNP	Location	Single locus P valu	Gene	MHC region	Stage 1	Stage 2	Combined		
3	rs843350	1.85E+08	2.95E-01	VWA5B2	13	rs2985923	66653746	1.50E-01	PCDH9	-	4.65E-07	5.29E-02	2.45E-03	6271	
6	rs159415	1.34E+08	5.46E-01	EYA4	1	rs11584093	54769601	7.91E-01	ACOT11	-	4.65E-07	4.60E-01	4.94E-06	6272	
4	rs12509909	81294224	7.17E-02	N/A	20	rs6090443	61624230	7.46E-01	PPDPF	-	4.65E-07	5.10E-01	3.38E-04	6273	
2	rs10932374	2.12E+08	3.38E-01	ERBB4	13	rs1415707	97294596	1.04E-01	N/A	-	4.65E-07	9.75E-01	8.38E-05	6274	
11	rs475688	64120867	8.12E-01	NRXN2	12	rs7304431	43286606	4.07E-01	NELL2	-	4.65E-07	2.03E-01	1.16E-03	6275	
3	rs9849797	1.45E+08	4.69E-02	SLC9A9	17	rs1859962	66620348	5.57E-01	N/A	-	4.65E-07	1.72E-01	5.66E-06	6276	
10	rs7477069	54212144	4.45E-01	MBL2	18	rs3730783	50069281	1.43E-01	POLI	-	4.65E-07	2.25E-01	2.89E-06	6277	
1	rs2948045	53824486	7.93E-01	GLIS1	8	rs2593095	38587762	1.79E-01	RNF5P1	-	4.65E-07	2.44E-01	3.30E-06	6278	
7	rs4730287	1.08E+08	7.75E-01	N/A	7	rs6464342	1.53E+08	7.66E-01	N/A	-	4.65E-07	5.59E-01	1.67E-05	6279	
6	rs1028318	25698203	7.81E-01	N/A	2	rs3821318	75240819	6.73E-01	TACR1	MHC	4.65E-07	9.82E-01	8.93E-05	6280	
10	rs10788283	1.24E+08	2.17E-01	BTBD16	17	rs8074645	5683518	1.44E-01	N/A	-	4.66E-07	5.38E-01	6.86E-05	6281	
15	rs867043	36297219	5.79E-01	N/A	16	rs11150564	29574237	6.15E-01	SPN	-	4.66E-07	8.49E-01	1.08E-04	6282	
7	rs10155893	1.31E+08	1.51E-01	N/A	21	rs416707	21048681	4.96E-01	C21orf131	-	4.66E-07	3.01E-01	7.37E-06	6283	
5	rs17692371	1.29E+08	9.90E-01	CHSY3	19	rs12463337	20935770	3.58E-01	ZNF85	-	4.66E-07	N/A	N/A	6284	
1	rs7543486	14170601	6.45E-01	N/A	10	rs2755997	33415197	9.30E-01	N/A	-	4.66E-07	3.03E-01	2.49E-03	6285	
12	rs964997	1.25E+08	4.28E-01	TMEM132B	15	rs4267279	31977983	8.52E-01	AVEN	-	4.66E-07	8.47E-01	6.84E-05	6286	
11	rs12795300	87118134	1.74E-01	N/A	14	rs11626128	40565293	6.42E-01	N/A	-	4.66E-07	8.56E-01	1.70E-05	6287	
6	rs9379638	10744123	6.89E-01	GCNT2	14	rs9285592	24468411	9.67E-01	STXBP6	-	4.66E-07	4.80E-01	3.06E-06	6288	
1	rs12058205	1.89E+08	6.51E-01	FAM5C	15	rs8040756	53585891	1.08E-01	DYX1C1	-	4.66E-07	5.69E-01	5.72E-05	6289	
7	rs2040933	1.24E+08	6.57E-01	N/A	10	rs805722	1.06E+08	9.63E-01	COL17A1	-	4.66E-07	8.28E-01	9.86E-05	6290	
5	rs10046065	1.24E+08	6.30E-01	N/A	11	rs1940640	94000460	4.42E-01	PIWIL4	-	4.66E-07	4.63E-01	6.98E-04	6291	
3	rs12631386	1.35E+08	4.49E-02	N/A	5	rs2060428	1.37E+08	6.22E-01	SPOCK1	-	4.66E-07	6.49E-01	1.36E-03	6292	
6	rs11759320	1.48E+08	2.98E-01	N/A	5	rs6870285	1.25E+08	5.79E-01	N/A	-	4.66E-07	1.76E-01	5.26E-06	6293	
2	rs4851794	1.06E+08	6.23E-01	N/A	8	rs2458285	1.04E+08	1.60E-01	N/A	-	4.66E-07	4.03E-01	2.06E-04	6294	
12	rs1683709	1.27E+08	5.57E-01	TMEM132C	14	rs227019	22064646	3.29E-01	N/A	-	4.66E-07	7.72E-01	1.18E-04	6295	
7	rs17171480	35552194	6.06E-01	N/A	8	rs1463259	82900300	4.00E-01	SNX16	-	4.66E-07	5.20E-01	1.82E-04	6296	
6	rs4594993	29378477	2.80E-01	OR14J1	4	rs4833753	1.23E+08	5.94E-02	N/A	MHC	4.66E-07	6.78E-01	5.61E-05	6297	
5	rs323700	2617243	6.01E-01	N/A	8	rs2885068	53405328	3.95E-01	ST18	-	4.67E-07	1.84E-01	9.06E-04	6298	
6	rs847848	35014146	5.60E-01	ANKS1A	9	rs372957	31962985	1.19E-01	N/A	MHC	4.67E-07	1.13E-01	1.56E-03	6299	
6	rs847848	35014146	5.60E-01	ANKS1A	9	rs453019	31975376	1.19E-01	N/A	MHC	4.67E-07	1.18E-01	1.48E-03	6300	
2	rs7425117	99811462	2.20E-01	AFF3	8	rs3885690	10864279	5.90E-01	XKR6	-	4.67E-07	8.38E-01	1.58E-04	6301	
1	rs4649419	2.31E+08	5.59E-01	C1orf57	13	rs2765584	1.03E+08	5.14E-01	N/A	-	4.67E-07	9.46E-01	1.12E-04	6302	
1	rs12140479	70595826	2.66E-01	ANKRD13C	8	rs961465	69732037	6.09E-02	C8orf34	-	4.67E-07	1.14E-01	2.88E-03	6303	
4	rs2196978	80754993	5.99E-02	N/A	7	rs10272997	70402452	5.44E-01	WBSCR17	-	4.67E-07	1.91E-01	3.22E-06	6304	
6	rs7741397	1.66E+08	8.81E-01	N/A	3	rs6764089	77104959	5.26E-01	N/A	-	4.67E-07	7.23E-01	2.00E-05	6305	
3	rs10510920	64892123	3.04E-01	MIR548A2	3	rs12636725	1.51E+08	2.92E-01	WWTR1	-	4.67E-07	9.12E-01	3.68E-05	6306	
3	rs4312641	15975891	3.46E-01	N/A	4	rs1395652	60231878	6.00E-01	N/A	-	4.67E-07	N/A	N/A	6307	
9	rs10511574	11941204	3.79E-01	N/A	22	rs5992838	16644831	1.46E-01	BID	-	4.67E-07	2.73E-02	1.90E-07	6308	
16	rs2386570	50690049	6.58E-01	N/A	16	rs11642248	81374764	5.23E-01	CDH13	-	4.67E-07	4.84E-01	3.07E-04	6309	
8	rs2511692	1.04E+08	2.82E-01	N/A	13	rs4885319	75057105	7.13E-01	UCHL3	-	4.67E-07	5.65E-01	1.75E-05	6310	
5	rs11745467	85695581	2.77E-01	N/A	10	rs1856591	44918624	3.29E-01	LOC100133308	-	4.67E-07	7.52E-01	1.18E-04	6311	
6	rs474519	10902522	4.30E-01	MAK	18	rs10454095	18031438	5.72E-02	GATA6	-	4.68E-07	5.28E-01	9.05E-04	6312	
9	rs4978813	1.11E+08	4.45E-01	PTPN3	17	rs8068489	69363038	8.35E-01	N/A	-	4.68E-07	6.16E-01	1.59E-04	6313	
12	rs11109097	96452568	5.25E-01	RMST	21	rs12482870	44905807	2.59E-01	C21orf29	-	4.68E-07	6.66E-01	4.44E-05	6314	
4	rs4862611	1.87E+08	1.86E-03	N/A	11	rs17143395	81687956	6.24E-02	N/A	-	4.68E-07	2.31E-01	5.14E-04	6315	
12	rs1656535	41734204	5.59E-01	N/A	12	rs1527790	76647901	1.07E-01	N/A	-	4.68E-07	7.91E-01	1.25E-05	6316	
8	rs4831871	12995282	4.86E-02	DLC1	20	rs10485804	54239111	7.83E-01	MC3R	-	4.68E-07	N/A	N/A	6317	
2	rs1476698	2.42E+08	7.09E-01	FARP2	15	rs2047667	51126053	4.76E-02	N/A	-	4.68E-07	3.52E-03	1.91E-02	6318	
12	rs987839	21266105	6.20E-01	SLCO1B1	21	rs6586245	41927730	2.66E-01	N/A	-	4.68E-07	3.92E-01	1.15E-05	6319	
2	rs888011	2.02E+08	9.75E-01	ALS2CR4	11	rs2277279	1.05E+08	4.76E-01	GRIA4	-	4.68E-07	3.06E-02	2.87E-07	6320	
8	rs10108007	61310616	4.24E-01	CA8	16	rs1423904	59023860	6.69E-01	N/A	-	4.68E-07	8.23E-01	6.59E-05	6321	
4	rs10027979	88675465	8.89E-01	SPARCL1	9	rs4618799	18109661	7.02E-01	N/A	-	4.68E-07	5.65E-01	7.55E-06	6322	
2	rs259833	1.8E+08	8.75E-01	ZNF385B	4	rs10015715	58946804	7.11E-01	N/A	-	4.69E-07	9.36E-01	7.14E-05	6323	
1	rs6660548	22552666	3.08E-01	N/A	7	rs10277168	42271762	2.73E-01	N/A	-	4.69E-07	6.65E-02	2.08E-02	6324	
3	rs442800	1.39E+08	3.26E-01	N/A	8	rs1500906	96601801	1.79E-01	N/A	-	4.69E-07	1.88E-01	4.15E-03	6325	
3	rs442800	1.39E+08	3.26E-01	N/A	8	rs1500909	96602267	1.79E-01	N/A	-	4.69E-07	1.88E-01	4.15E-03	6326	
13	rs17294949	44524937	5.94E-01	N/A	16	rs7404068	71847434	2.01E-01	N/A	-	4.69E-07	8.67E-01	8.14E-05	6327	
2	rs7558782	45389180	7.44E-01	N/A	15	rs11070857	49644512	4.49E-01	DMXL2	-	4.69E-07	5.36E-01	1.76E-05	6328	
1	rs2065140	1.76E+08	1.21E-01	N/A	3	rs1456669	1.49E+08	3.45E-01	N/A	-	4.69E-07	5.24E-02	4.12E-07	6329	
6	rs879882	31247431	7.61E-01	TCF19	6	rs2596560	31463297	2.87E-01	MICA	MHC	4.69E-07	5.38E-01	5.40E-04	6330	
6	rs3818408	10496639	7.25E-01	TFAP2A	2	rs1075708	2.32E+08	2.20E-02	C2orf57	-	4.69E-07	6.93E-01	4.61E-05	6331	
7	rs2471189	1.22E+08	7.66E-01	CADPS2	7	rs10241449	1.38E+08	7.83E-01	ATP6V0A4	-	4.69E-07	N/A	N/A	6332	
13	rs6492238	1.1E+08	9.41E-02	N/A	16	rs8045995	81255449	3.88E-01	CDH13	-	4.69E-07	1.68E-01	4.27E-03	6333	
7	rs2726005	85398458	8.77E-01	N/A	13	rs17086849	28064399	5.75E-01	N/A	-	4.69E-07	5.60E-01	2.99E-04	6334	
6	rs2690107	25438517	2.75E-01	N/A	8	rs6982250	70575004	8.99E-01	SULF1	MHC	4.69E-07	1.15E-01	4.19E-03	6335	
19	rs10425594	44581531	5.59E-01	SAMD4B	20	rs3828016	1865290	9.92E-01	SIRPA	-	4.69E-07	4.66E-01	9.38E-04	6336	
1	rs17102086	46495526	7.73E-01	RAD54L	13	rs9572574	70408059	6.02E-02	N/A	-	4.69E-07	9.43E-01	6.23E-05	6337	
7	rs7806522	34056348	8.00E-01	BMPER	7	rs13228720	37555747	4.44E-01	N/A	-	4.69E-07	5.71E-01	2.17E-04	6338	
13	rs11617984	18520143	5.78E-01	DKFZp686A162	13	rs9546698	83931934	2.41E-01	N/A	-	4.69E-07	8.74E-01	7.06E-05	6339	
4	rs2131466	28426304	8.00E-01	N/A	9	rs7846724	12808692	6.34E-01	C9orf150	-	4.69E-07	7.52E-01	7.02E-05	6340	
3	rs4395346	38826088	9.80E-01	SCN10A	14	rs10140652	19761728	6.10E-01	OR11H6	-	4.70E-07	2.65E-02	3.12E-03	6341	
3	rs7627881	38822156	9.80E-01	SCN10A	14	rs10140652	19761728	6.10E-01	OR11H6	-	4.70E-07	2.65E-02	3.12E-03	6342	
18	rs10513886	52034647	7.39E-01	N/A	20	rs6030208	40459279	6.73E-01	PTPRT	-	4.70E-07	7.66E-02	5.60E-03	6343	
2	rs11893319	1.97E+08	1.77E-01	HECW2	3	rs1393450	1.75E+08	6.49E-01	NLGN1	-	4.70E-07	7.35E-01	2.71E-04	6344	
6	rs851971	1.52E+08	4.53E-01	N/A	10	rs1659840	13142854	7.05E-01	N/A	-	4.70E-07	9.51E-01	2.51E-05	6345	
14	rs12432098	30160309	7.28E-01	G2E3	14	rs1952773	89398746	1.91E-01	C14orf143	-	4.70E-07	5.60E-01	3.54E-05	6346	
2	rs13020025	2.3E+08	7.12E-01	PID1	19	rs2255598	24248960	9.93E-01	N/A	-	4.70E-07	8.01E-01	1.08E-04	6347	
6	rs11153450	1.14E+08	5.83E-01	N/A	1	rs2131887	2.19E+08	9.42E-01	N/A	-	4.70E-07	9.65E-01	1.12E-04	6348	
1	rs1855886	82408707	9.33E-01	N/A	3	rs304077	4509629	3.69E-01	ITPR1	-	4.70E-07	3.35E-02	1.03E-07	6349	
7	rs10954732	75449085	9.92E-03	STYXL1	18	rs616329	61182880	9.06E-01	N/A	-	4.70E-07	6.52E-01	1.46E-04	6350	
3	rs6550306	34873129	3.77E-01	N/A	8	rs4925813	1.46E+08	3.21E-01	KIAA1688	-	4.70E-07	7.29E-01	5.02E-05	6351	
6	rs4122189	31275906	7.70E-02	HCG27	18	rs2732229	71742290	5.44E-01	N/A	MHC	4.70E-07	5.59E-01	4.03E-04	6352	
2	rs10929012	2.35E+08	9.14E-02	N/A	8	rs6421410	18002533	1.18E-01	ASAH1	-	4.70E-07	N/A	N/A	6353	
2	rs10929014	2.35E+08	9.14E-02	N/A	8	rs6421410	18002533	1.18E-01	ASAH1	-	4.70E-07	N/A	N/A	6354	
1	rs6701300	8123277	1.37E-01	N/A	10	rs4980264	1.25E+08	1.92E-01	HMX3	-	4.71E-07	6.15E-02	9.04E-03	6355	
13	rs726697	1.06E+08	4.64E-01	N/A	17	rs7216333	48262329	6.61E-01	N/A	-	4.71E-07	1.17E-01	4.38E-06	6356	
7	rs17158641	77306373	2.24E-01	PHTF2	20	rs6028080	59237873	1.55E-01	N/A	-	4.71E-07	9.19E-01	3.04E-05	6357	
14	rs7150049	21271728	1.77E-01	N/A	16	rs4780986	10722731	6.88E-01	N/A	-	4.71E-07	7.75E-01	3.66E-04	6358	
5	rs17708295	17022440	5.82E-01	N/A	22	rs138150	44484794	9.42E-01	ATXN10	-	4.71E-07	8.59E-01	3.62E-05	6359	
8	rs4876860	1.2E+08	2.21E-01	N/A	13	rs3014937	45066410	1.59E-02	FAM194B	-	4.71E-07	8.64E-02	3.77E-03	6360	
6	rs1331484	990503	3.23E-01	LOC285768	21	rs2826320	20779301	2.45E-01	N/A	-	4.71E-07	1.38E-01	5.48E-06	6361	
11	rs12273302	19208280	8.61E-01	E2F8	16	rs12930687	9528370	8.01E-04	N/A	-	4.71E-07	8.50E-01	1.70E-04	6362	
6	rs675497	75114270	6.59E-01	N/A	8	rs4871160	1.22E+08	9.92E-01	N/A	-	4.71E-07	7.91E-02	1.63E-03	6363	
11	rs4396252	94014342	9.83E-01	N/A	14	rs3759704	58631036	9.00E-01	N/A	-	4.71E-07	N/A	N/A	6364	
8	rs4237068	21266056	4.28E-01	N/A	11	rs11213013	1.09E+08	2.26E-01	N/A	-	4.71E-07	9.27E-01	1.41E-04	6365	
2	rs714546	1.33E+08	4.56E-01	GPR39	7	rs227951	23577154	9.20E-01	CLK2P	-	4.71E-07	2.48E-01	1.96E-03	6366	
1	rs12133873	2.44E+08	9.87E-01	SMYD3	16	rs748815	52965305	5.39E-01	N/A	-	4.71E-07	4.82E-01	2.45E-05	6367	
1	rs12137320	2.44E+08	9.87E-01	SMYD3	16	rs748815	52965305	5.39E-01	N/A	-	4.71E-07	6.45E-01	4.24E-05	6368	
14	rs3829948	92186579	5.37E-01	RIN3	16	rs16959429	56467155	4.05E-01	CNGB1	-	4.71E-07	3.58E-01	5.29E-04	6369	
9	rs869995	28014654	1.20E-01	LINGO2	16	rs8047611	86374093	7.30E-01	N/A	-	4.71E-07	6.72E-01	6.29E-04	6370	
14	rs3829948	92186579	5.37E-01	RIN3	16	rs247032	56470180	4.05E-01	CNGB1	-	4.71E-07	7.76E-01	1.48E-04	6371	
5	rs9784716	82965743	7.54E-01	HAPLN1	16	rs322613	62427536	9.55E-01	N/A	-	4.71E-07	1.88E-01	3.37E-03	6372	
4	rs7681966	1.84E+08	4.36E-01	ODZ3	10	rs2279339	59815004	7.10E-01	UBE2D1	-	4.71E-07	8.50E-01	2.00E-04	6373	
8	rs11135723	23217149	1.75E-01	LOXL2	19	rs12052065	17102649	9.51E-01	MYO9B	-	4.72E-07	4.93E-01	5.14E-06	6374	
7	rs6466401	1.12E+08	5.50E-01	DOCK4	11	rs7127610	84306633	5.16E-01	DLG2	-	4.72E-07	1.38E-01	2.32E-03	6375	
2	rs1470504	77076318	7.97E-01	LRRTM4	5	rs160891	3187466	5.99E-01	N/A	-	4.72E-07	9.67E-01	4.56E-05	6376	
7	rs3807655	77656483	1.16E-01	MAGI2	13	rs2298230	52506480	8.49E-01	OLFM4	-	4.72E-07	5.52E-02	3.56E-07	6377	
6	rs3127337	1.66E+08	1.80E-01	T	3	rs9848736	1.92E+08	8.00E-01	N/A	-	4.72E-07	5.88E-01	7.96E-06	6378	
3	rs11715745	63466181	5.22E-02	SYNPR	3	rs1844396	1.79E+08	1.88E-01	N/A	-	4.72E-07	9.27E-01	1.86E-05	6379	
2	rs1834619	17764966	5.69E-01	SMC6	8	rs11994327	34696584	7.25E-01	N/A	-	4.72E-07	5.96E-02	1.09E-02	6380	
4	rs963065	1.02E+08	5.27E-01	PPP3CA	14	rs2148692	84870539	7.10E-01	N/A	-	4.72E-07	7.97E-01	3.04E-05	6381	
8	rs12549171	13816259	3.01E-01	N/A	12	rs11114975	80588517	4.17E-01	PPFIA2	-	4.72E-07	2.63E-01	5.30E-04	6382	

SNP A					SNP B						Interaction P			Ranking	Cluster in top 100 interactions
CHR	SNP	Location	gle locus P va	Gene	CHR	SNP	Location	Single locus P valu	Gene	MHC region	Stage 1	Stage 2	Combined		
3	rs1667746	24274509	6.55E-01	THRB	7	rs2161	97930442	8.96E-01	N/A	-	4.72E-07	3.33E-01	1.42E-05	6383	
2	rs4233870	9728099	9.47E-01	N/A	9	s16921457	4535973	9.31E-01	SLC1A1	-	4.72E-07	3.44E-01	2.77E-06	6384	
5	rs34697	66334393	5.25E-02	MAST4	9	rs1538844	6953799	8.24E-02	KDM4C	-	4.72E-07	7.64E-01	1.49E-04	6385	
2	rs7582990	10450320	8.69E-01	HPCAL1	14	rs1958636	53365399	7.95E-01	N/A	-	4.72E-07	4.97E-01	2.78E-04	6386	
3	rs7636734	37721855	4.56E-02	ITGA9	3	rs4856085	1.05E+08	9.58E-02	N/A	-	4.72E-07	8.38E-01	7.59E-05	6387	
14	rs12101174	60868578	6.05E-01	PRKCH	20	rs4809440	61076763	5.34E-02	SLC17A9	-	4.73E-07	4.10E-01	1.67E-05	6388	
6	rs6936833	1.33E+08	6.73E-01	VNN1	11	rs7940244	70899222	9.24E-01	N/ADSYN1	-	4.73E-07	4.45E-01	1.17E-03	6389	
11	rs1790474	1.19E+08	4.13E-01	N/A	15	rs2957740	70094745	5.68E-01	MYO9A	-	4.73E-07	9.41E-01	1.55E-04	6390	
1	rs602117	2.38E+08	8.64E-01	CHRM3	4	rs1425377	65119238	4.89E-01	N/A	-	4.73E-07	4.08E-01	1.40E-05	6391	
1	rs1408859	29638736	6.06E-01	N/A	3	rs925820	2843988	4.43E-01	CNTN4	-	4.73E-07	5.18E-01	1.88E-05	6392	
17	rs9909526	14596417	6.56E-01	N/A	17	rs7220683	73784880	2.07E-01	N/A	-	4.73E-07	5.37E-01	1.13E-05	6393	
1	rs1509019	1.61E+08	6.64E-01	RGS5	8	s10101849	5238068	4.26E-01	N/A	-	4.73E-07	9.31E-01	4.09E-05	6394	
1	rs1509019	1.61E+08	6.64E-01	RGS5	8	rs6559010	5237624	4.26E-01	N/A	-	4.73E-07	9.31E-01	4.09E-05	6395	
10	rs7095662	29419122	4.39E-01	N/A	15	s17491052	93800733	7.17E-01	LOC145820	-	4.73E-07	2.30E-01	1.50E-03	6396	
2	rs1031293	2.07E+08	6.55E-01	N/A	14	rs2269967	72807795	6.44E-01	NUMB	-	4.73E-07	9.02E-02	1.68E-06	6397	
10	rs602618	1.13E+08	9.60E-01	ADRA2A	11	rs524288	56662487	6.35E-01	N/A	-	4.73E-07	2.21E-01	6.66E-07	6398	
4	rs1355578	46594378	2.65E-01	COX7B2	17	rs8068010	4278869	2.49E-01	SPNS3	-	4.73E-07	4.86E-01	2.13E-04	6399	
2	rs4674952	2.26E+08	5.40E-01	DOCK10	10	rs7895918	43712074	1.50E-02	N/A	-	4.73E-07	7.42E-01	1.67E-05	6400	
20	rs6140981	9533873	1.62E-01	PAK7	20	rs1015581	14467512	4.24E-01	MACROD2	-	4.73E-07	1.04E-01	2.89E-03	6401	
14	rs10162396	78751203	8.87E-01	NRXN3	17	rs1010044	67804921	2.92E-01	N/A	-	4.73E-07	5.96E-01	4.93E-06	6402	
2	rs7562668	71051131	7.59E-01	ATP6V1B1	13	rs2146443	1.12E+08	2.86E-01	N/A	-	4.73E-07	6.72E-01	2.11E-04	6403	
12	rs4762367	96075155	1.93E-01	N/A	19	rs2334253	50808317	2.61E-01	N/A	-	4.73E-07	8.54E-01	2.57E-05	6404	
12	rs4913277	66868439	5.36E-02	IL26	17	rs2285572	65226809	3.31E-01	N/A	-	4.74E-07	7.62E-01	2.98E-05	6405	
1	rs4269797	63401236	1.52E-01	N/A	13	rs7318772	29578623	1.96E-01	N/A	-	4.74E-07	4.55E-01	1.15E-05	6406	
6	rs4577811	1.51E+08	5.19E-02	PLEKHG1	3	rs4684747	10947003	1.59E-01	SLC6A11	-	4.74E-07	6.01E-01	5.30E-05	6407	
14	rs17124954	88440873	6.70E-01	N/A	16	rs7199026	70098727	8.99E-02	CHST4	-	4.74E-07	5.64E-02	3.77E-03	6408	
2	rs4665135	1.61E+08	9.50E-01	N/A	2	rs6712733	1.89E+08	7.76E-01	N/A	-	4.74E-07	1.63E-01	1.09E-03	6409	
18	rs4399595	8217344	9.76E-01	PTPRM	22	rs135044	42175652	8.62E-01	MPPED1	-	4.74E-07	4.65E-01	3.65E-04	6410	
2	rs2422245	64595770	5.59E-01	AFTPH	3	s13089360	81005517	2.78E-01	N/A	-	4.74E-07	9.12E-01	8.30E-05	6411	
10	rs10823571	71986873	2.28E-01	KIAA1274	10	rs7094645	72466324	2.68E-01	N/A	-	4.74E-07	8.47E-01	5.10E-05	6412	
8	rs7823353	1.43E+08	6.14E-01	N/A	12	rs2696004	1.04E+08	7.91E-01	N/A	-	4.74E-07	4.47E-01	4.83E-04	6413	
14	rs11627134	74168449	6.49E-01	LTBP2	22	rs3747152	29850338	4.54E-01	INPP5J	-	4.74E-07	2.08E-01	3.38E-06	6414	
5	rs152787	14631546	8.72E-01	FAM105A	9	s11790480	38374578	1.93E-01	ALDH1B1	-	4.74E-07	5.99E-03	2.88E-02	6415	
17	rs17247848	28008297	5.07E-01	MYO1D	18	rs3813086	46442790	4.62E-01	MAPK4	-	4.74E-07	5.95E-01	1.00E-03	6416	
6	rs9374668	1.18E+08	4.91E-01	DCBLD1	7	s10234234	1.34E+08	8.83E-01	N/A	-	4.75E-07	2.32E-02	6.15E-08	6417	
6	rs9688888	1.69E+08	9.98E-03	N/A	8	rs6468274	34658236	8.79E-01	N/A	-	4.75E-07	9.83E-01	1.14E-04	6418	
5	rs4513681	1.48E+08	4.93E-01	N/A	17	rs9303521	41160977	3.77E-01	N/A	-	4.75E-07	3.88E-01	1.98E-06	6419	
6	rs2038774	1.51E+08	4.70E-01	PPP1R14C	18	rs2042717	72323185	2.83E-01	ZNF516	-	4.75E-07	8.17E-01	9.39E-05	6420	
7	rs217510	28484580	9.20E-01	CREB5	14	s10140845	80087990	9.57E-01	C14orf145	-	4.75E-07	2.74E-01	2.20E-06	6421	
7	rs217517	28488170	9.20E-01	CREB5	14	s10140845	80087990	9.57E-01	C14orf145	-	4.75E-07	3.28E-01	3.39E-06	6422	
2	rs4395295	1.33E+08	7.88E-01	GPR39	4	s13113943	1.11E+08	9.66E-01	N/A	-	4.75E-07	3.80E-01	2.26E-06	6423	
5	rs463691	4322779	3.89E-01	N/A	12	rs4764755	99753004	8.38E-01	ANO4	-	4.75E-07	1.24E-01	7.07E-07	6424	
6	rs13216260	1.51E+08	1.16E-01	MTHFD1L	13	rs9561545	93815594	7.90E-01	GPC6	-	4.75E-07	8.95E-01	2.11E-05	6425	
5	rs13158939	86981886	1.72E-01	N/A	19	s12460438	22867619	1.63E-01	N/A	-	4.75E-07	6.28E-01	7.97E-06	6426	
5	rs13158939	86981886	1.72E-01	N/A	19	rs7246529	22856963	1.63E-01	N/A	-	4.75E-07	6.43E-01	8.35E-06	6427	
2	rs6430452	1.34E+08	6.73E-01	N/A	12	rs1451904	1.29E+08	8.19E-01	TMEM132D	-	4.76E-07	2.66E-01	7.31E-06	6428	
6	rs1335024	1.03E+08	2.31E-01	GRIK2	15	s11636976	31296239	2.84E-01	N/A	-	4.76E-07	7.68E-01	1.52E-04	6429	
2	rs427375	8299357	7.08E-01	N/A	8	s11787080	1.43E+08	7.59E-01	N/A	-	4.76E-07	2.43E-01	1.18E-05	6430	
9	rs944591	83372048	1.28E-01	TLE1	11	rs6590146	1.25E+08	8.72E-01	FEZ1	-	4.76E-07	9.26E-01	1.16E-04	6431	
3	rs17033281	10664671	2.92E-01	N/A	17	rs4968857	64932028	5.49E-02	MAP2K6	-	4.76E-07	1.41E-01	9.75E-04	6432	
5	rs1600178	51894999	9.08E-01	N/A	11	rs2625518	83425743	3.63E-02	DLG2	-	4.76E-07	4.98E-01	5.58E-04	6433	
4	rs7655423	1.12E+08	4.16E-01	N/A	11	rs2291842	5676243	8.66E-02	TRIM5	-	4.76E-07	8.65E-01	1.76E-04	6434	
5	rs866821	87036066	1.39E-01	N/A	19	s12460438	22867619	1.63E-01	N/A	-	4.76E-07	9.14E-01	2.01E-05	6435	
5	rs866821	87036066	1.39E-01	N/A	19	rs7246529	22856963	1.63E-01	N/A	-	4.76E-07	9.33E-01	2.10E-05	6436	
2	rs735887	47053450	6.26E-01	TTC7A	2	rs236833	2.06E+08	2.29E-01	PARD3B	-	4.77E-07	7.38E-01	1.43E-04	6437	
5	rs173878	1.73E+08	1.84E-01	BNIP1	12	rs1881227	87562931	9.76E-01	N/A	-	4.77E-07	8.02E-01	6.69E-05	6438	
3	rs11709290	1.4E+08	3.45E-01	ESYT3	8	s12680577	69541263	4.09E-01	N/A	-	4.77E-07	1.56E-01	1.07E-03	6439	
3	rs4543005	1825547	5.30E-01	N/A	14	rs7148018	74026906	7.46E-01	LTBP2	-	4.77E-07	6.24E-01	1.20E-04	6440	
10	rs313483	4567247	5.12E-01	N/A	12	s11829149	3947434	1.88E-01	N/A	-	4.77E-07	4.90E-01	7.65E-04	6441	
1	rs17047703	2.17E+08	1.84E-01	RRP15	12	s10784294	61108334	9.66E-01	N/A	-	4.77E-07	9.26E-01	8.73E-05	6442	
7	rs3807669	77648804	1.30E-01	MAGI2	13	rs2298230	52506480	8.49E-01	OLFM4	-	4.77E-07	5.69E-02	3.72E-07	6443	
7	rs6961353	55501341	9.62E-02	VOPP1	18	rs4321280	68532326	7.24E-01	N/A	-	4.77E-07	N/A	N/A	6444	
11	rs2846418	1.08E+08	4.85E-01	EXPH5	20	rs1201981	58729618	8.82E-01	N/A	-	4.77E-07	6.19E-01	3.72E-04	6445	
4	rs6838346	1.23E+08	2.28E-01	N/A	11	s12792538	98748297	7.68E-01	CNTN5	-	4.77E-07	6.31E-01	1.48E-05	6446	
3	rs9824811	1.57E+08	4.93E-01	N/A	4	rs203443	15857774	6.21E-01	FLJ39653	-	4.77E-07	9.76E-01	1.39E-04	6447	
5	rs17635646	1.68E+08	2.54E-01	SLIT3	10	rs592818	27808006	5.02E-01	N/A	-	4.78E-07	6.60E-01	9.33E-06	6448	
4	rs1506801	1.02E+08	5.55E-01	PPP3CA	4	s12509055	1.69E+08	5.12E-01	N/A	-	4.78E-07	3.49E-01	5.74E-07	6449	
3	rs10865559	77489198	7.66E-01	ROBO2	11	rs2239695	1.18E+08	6.63E-01	CD3D	-	4.78E-07	5.32E-01	3.71E-04	6450	
4	rs17060468	1.75E+08	7.26E-01	KIAA1712	15	rs4243086	72484137	3.39E-01	SEMA7A	-	4.78E-07	6.54E-01	2.33E-05	6451	
8	rs6469053	1.07E+08	9.47E-01	N/A	11	rs2370643	1.21E+08	2.16E-01	N/A	-	4.78E-07	1.79E-02	5.04E-08	6452	
5	rs16893170	64155371	5.65E-01	SDCCAG10	15	rs2271902	76273244	8.65E-01	ACSBG1	-	4.78E-07	9.93E-01	3.93E-06	6453	
5	rs2367114	64159843	5.65E-01	SDCCAG10	15	rs2271902	76273244	8.65E-01	ACSBG1	-	4.78E-07	9.93E-01	3.93E-06	6454	
6	rs6932930	34785081	2.64E-01	C6orf106	5	rs3010277	72050156	1.27E-01	N/A	MHC	4.78E-07	1.39E-02	1.57E-06	6455	
6	rs6932930	34785081	2.64E-01	C6orf106	5	rs3010278	72049329	1.27E-01	N/A	MHC	4.78E-07	1.39E-02	1.57E-06	6456	
3	rs3749213	1.24E+08	6.77E-01	KPN/A1	10	rs7086109	1.15E+08	9.73E-01	N/A	-	4.78E-07	4.57E-01	1.20E-05	6457	
3	rs7632823	54290709	1.09E-01	CACN/A2D3	20	rs6082420	2145870	1.69E-01	N/A	-	4.78E-07	N/A	N/A	6458	
14	rs178490	57596355	5.66E-01	C14orf37	15	rs6496239	84149393	1.48E-01	KLHL25	-	4.78E-07	2.14E-01	2.16E-03	6459	
13	rs1757387	1.03E+08	3.23E-01	N/A	20	rs6135395	15284682	7.79E-01	MACROD2	-	4.78E-07	4.34E-01	1.22E-05	6460	
1	rs7532282	1.58E+08	5.02E-01	N/A	3	s11127905	85793026	2.44E-01	CADM2	-	4.78E-07	1.59E-01	1.55E-06	6461	
1	rs7532282	1.58E+08	5.02E-01	N/A	3	rs9860249	85770242	2.44E-01	CADM2	-	4.78E-07	1.59E-01	1.55E-06	6462	
3	rs909017	22779841	9.68E-01	N/A	17	rs4609888	1808354	3.07E-01	RTN4RL1	-	4.78E-07	8.75E-01	1.43E-05	6463	
1	rs11582551	20345766	9.81E-02	PLA2G2F	8	rs7833497	1.28E+08	2.51E-01	N/A	-	4.78E-07	2.87E-01	2.10E-03	6464	
3	rs11709882	1.61E+08	2.24E-01	N/A	20	rs1469781	118642	3.26E-01	DEFB128	-	4.78E-07	5.95E-01	3.01E-05	6465	
15	rs867043	36297219	5.79E-01	N/A	16	rs3764276	29580704	3.61E-01	QPRT	-	4.78E-07	4.13E-01	1.08E-03	6466	
6	rs6570854	1.49E+08	7.06E-01	SASH1	18	rs2847297	12787694	4.25E-01	PTPN2	-	4.78E-07	8.84E-01	8.21E-05	6467	
2	rs1067350	44523450	9.60E-01	C2orf34	3	rs9874152	1.96E+08	8.70E-01	ATP13A3	-	4.79E-07	5.27E-01	3.69E-05	6468	
2	rs1072581	2.26E+08	5.38E-01	DOCK10	10	rs7895918	43712074	1.50E-02	N/A	-	4.79E-07	7.42E-01	1.67E-05	6469	
9	rs4745511	77952115	7.17E-01	N/A	11	s11231529	55437912	2.40E-01	OR5W2	-	4.79E-07	3.38E-01	9.79E-04	6470	
7	rs6976604	29580803	4.48E-01	PRR15	19	rs7247411	21367210	8.92E-02	ZNF493	-	4.79E-07	N/A	N/A	6471	
17	rs1982815	34313660	1.30E-01	LASP1	21	rs2839493	42668766	1.80E-01	TMPRSS3	-	4.79E-07	1.28E-02	4.97E-03	6472	
1	rs1033190	18401435	6.46E-01	IGSF21	3	rs1347449	1.2E+08	7.97E-01	IGSF11	-	4.79E-07	1.44E-01	4.48E-03	6473	
7	rs12530984	12799614	3.49E-01	N/A	10	rs7898559	66695250	2.39E-01	N/A	-	4.79E-07	1.95E-01	1.38E-05	6474	
6	rs3868542	31253818	6.85E-02	TCF19	6	rs3130685	31314185	9.60E-02	N/A	MHC	4.79E-07	3.76E-01	7.75E-04	6475	
11	rs1628349	61651058	1.21E-01	INCENP	12	s11178238	69115060	4.54E-01	KCNMB4	-	4.79E-07	7.74E-01	1.84E-05	6476	
3	rs9844576	39183388	4.18E-01	CSRNP1	7	s11761526	4934651	2.83E-01	MMD2	-	4.79E-07	3.41E-01	2.17E-06	6477	
2	rs16847823	2.13E+08	7.67E-01	ERBB4	8	rs2979715	80724814	6.41E-01	STMN2	-	4.79E-07	1.25E-01	1.03E-02	6478	
7	rs2365281	1.56E+08	2.10E-01	N/A	16	rs2925990	80115507	2.21E-01	CMIP	-	4.79E-07	2.32E-01	4.19E-06	6479	
5	rs2648727	52110158	3.09E-01	ITGA1	14	rs970662	32243135	2.78E-01	AKAP6	-	4.79E-07	5.35E-01	4.11E-06	6480	
18	rs10775450	27939881	1.81E-01	N/A	18	rs7232315	53717230	6.60E-01	N/A	-	4.79E-07	3.65E-01	3.82E-06	6481	
7	rs11763946	46184672	2.61E-02	N/A	8	rs4831320	14895787	9.72E-01	SGCZ	-	4.79E-07	3.98E-02	1.63E-06	6482	
3	rs10510919	64892679	3.37E-01	MIR548A2	3	s12636725	1.51E+08	2.92E-01	WWTR1	-	4.79E-07	6.71E-01	9.59E-05	6483	
3	rs3892905	76125986	4.32E-01	N/A	11	rs658624	1.18E+08	7.38E-01	SCN2B	-	4.79E-07	3.09E-01	9.03E-06	6484	
15	rs2034247	78399486	8.59E-01	N/A	19	rs8102072	57180523	5.80E-01	ZNF350	-	4.79E-07	4.66E-01	1.43E-05	6485	
4	rs12507167	52613460	7.74E-01	SPATA18	5	rs216148	1.49E+08	2.51E-01	HMGXB3	-	4.79E-07	7.25E-01	2.74E-04	6486	
4	rs16875599	24001535	2.97E-01	N/A	19	rs2435068	20528316	4.58E-02	ZNF737	-	4.80E-07	1.41E-01	9.28E-03	6487	
5	rs16899073	29554957	5.51E-01	N/A	12	rs1879560	77393236	6.79E-01	N/A	-	4.80E-07	3.43E-01	2.83E-05	6488	
6	rs9405882	5864285	7.96E-01	N/A	2	rs4668879	15221205	8.99E-01	NBAS	-	4.80E-07	6.70E-01	5.15E-04	6489	
2	rs1595825	1.99E+08	9.23E-01	PLCL1	7	s12699758	15930763	7.54E-01	N/A	-	4.80E-07	7.00E-01	3.06E-05	6490	
5	rs1600178	51894999	9.08E-01	N/A	11	rs963146	83423444	3.73E-02	DLG2	-	4.80E-07	4.98E-01	5.54E-04	6491	
4	rs6825574	1.25E+08	6.10E-01	N/A	7	rs1242775	1.57E+08	8.48E-02	PTPRN2	-	4.80E-07	8.46E-01	1.01E-04	6492	
14	rs7154449	65371529	6.27E-02	N/A	16	rs4984633	1040307	2.16E-01	LOC146336	-	4.80E-07	4.47E-01	2.59E-04	6493	
9	rs1888157	1.28E+08	1.48E-01	N/A	18	rs7236619	19898877	6.56E-01	TTC39C	-	4.80E-07	8.65E-01	4.94E-04	6494	

SNP A					SNP B						Interaction P			Ranking	Cluster in top 100 interactions
CHR	SNP	Location	gle locus P va	Gene	CHR	SNP	Location	Single locus P valu	Gene	HC region	Stage 1	Stage 2	Combined		
1	rs7519595	1.75E+08	5.81E-02	ASTN1	4	rs3815464	25280141	9.09E-01	N/A	-	4.80E-07	1.23E-01	3.15E-06	6495	
4	rs4314329	31960561	3.19E-01	N/A	8	rs1016646	20307688	5.76E-01	N/A	-	4.80E-07	9.40E-01	3.02E-05	6496	
3	rs2574720	11635412	1.97E-01	VGLL4	8	rs4546683	3077005	2.46E-01	CSMD1	-	4.80E-07	1.11E-01	1.96E-03	6497	
3	rs10514734	77687380	9.95E-01	ROBO2	5	rs9327428	1.26E+08	5.43E-01	3-Mar	-	4.80E-07	7.86E-01	1.78E-04	6498	
1	rs987293	18324487	3.40E-01	IGSF21	13	rs4372573	90417134	5.26E-01	N/A	-	4.80E-07	2.24E-01	4.15E-06	6499	
3	rs1917072	78289877	9.02E-01	N/A	10	rs10788198	1.23E+08	8.55E-01	N/A	-	4.80E-07	6.91E-01	2.58E-05	6500	
12	rs4767944	1.11E+08	8.85E-01	ACAD10	14	rs10141858	97886923	1.51E-01	N/A	-	4.81E-07	2.88E-01	8.91E-06	6501	
6	rs9394169	33886942	7.87E-01	MLN	11	rs7112954	1127086	4.52E-01	N/A	MHC	4.81E-07	3.50E-01	8.92E-06	6502	
8	rs2514710	1.01E+08	1.50E-01	SPAG1	11	rs12419678	1.07E+08	7.73E-01	N/A	-	4.81E-07	6.47E-01	1.78E-04	6503	
2	rs10187622	1.88E+08	8.82E-01	TFPI	18	rs4940002	46449193	4.85E-01	MAPK4	-	4.81E-07	7.88E-01	5.40E-04	6504	
2	rs10179730	1.88E+08	9.34E-01	TFPI	18	rs4940002	46449193	4.85E-01	MAPK4	-	4.81E-07	7.88E-01	4.91E-04	6505	
1	rs868584	2.31E+08	7.01E-01	N/A	7	rs157911	1.3E+08	5.87E-01	MIR29B1	-	4.81E-07	3.43E-01	1.75E-05	6506	
3	rs993558	1.59E+08	5.44E-01	N/A	11	rs1783217	58225294	3.15E-01	GLYAT	-	4.81E-07	8.49E-01	2.55E-04	6507	
4	rs1948250	1.19E+08	5.57E-01	N/A	7	rs741304	36769525	9.92E-01	N/A	-	4.81E-07	8.85E-01	1.75E-04	6508	
8	rs411279	90877299	1.63E-01	RIPK2	17	rs10451228	73195845	8.35E-02	N/A	-	4.81E-07	9.34E-02	2.02E-06	6509	
2	rs2274412	2.16E+08	2.71E-01	ABCA12	17	rs11078308	15411904	9.73E-01	FAM18B2	-	4.81E-07	1.44E-01	5.54E-04	6510	
5	rs1600178	51894999	9.08E-01	N/A	11	rs1384751	83432571	3.75E-02	DLG2	-	4.81E-07	6.18E-01	3.72E-04	6511	
7	rs2023910	21524379	8.73E-01	SP4	8	rs17347502	4618657	5.03E-01	CSMD1	-	4.81E-07	6.45E-01	2.35E-05	6512	
8	rs2697675	91002125	8.04E-01	NBN	8	rs3104899	96689580	6.18E-01	N/A	-	4.81E-07	7.91E-01	6.38E-05	6513	
1	rs845451	2.08E+08	9.45E-01	SERTAD4	12	rs7963902	4988938	9.11E-01	N/A	-	4.81E-07	7.98E-01	1.60E-04	6514	
15	rs4778368	21764708	4.65E-01	N/A	17	rs4794737	52855906	3.47E-01	MSI2	-	4.81E-07	9.97E-01	1.45E-04	6515	
11	rs286911	34613797	6.17E-02	EHF	17	rs11658990	29790363	3.01E-01	N/A	-	4.81E-07	1.40E-01	2.71E-03	6516	
1	rs313710	86024424	8.56E-01	COL24A1	4	rs7659060	1.4E+08	7.60E-01	N/A	-	4.81E-07	1.43E-01	1.44E-03	6517	
17	rs10512556	66449189	5.04E-01	N/A	19	rs408687	22022963	6.99E-01	ZNF257	-	4.81E-07	3.20E-01	1.34E-03	6518	
17	rs10512556	66449189	5.04E-01	N/A	19	rs409835	22022511	6.99E-01	ZNF257	-	4.81E-07	3.20E-01	1.34E-03	6519	
17	rs10512556	66449189	5.04E-01	N/A	19	rs411020	22022942	6.99E-01	ZNF257	-	4.81E-07	3.20E-01	1.34E-03	6520	
17	rs11653075	50228743	6.77E-01	N/A	20	rs2206426	40719457	9.61E-01	PTPRT	-	4.81E-07	5.28E-01	4.33E-04	6521	
2	rs10185841	1.46E+08	9.99E-02	N/A	3	rs9845872	62816561	8.94E-01	CADPS	-	4.81E-07	7.64E-02	5.83E-07	6522	
2	rs4854547	69196037	8.62E-01	ANTXR1	5	rs4975684	1131642	9.89E-01	SLC12A7	-	4.81E-07	1.02E-01	1.12E-06	6523	
5	rs710998	3644916	6.03E-02	IRX1	22	rs11704609	44630591	3.50E-01	ATXN10	-	4.81E-07	4.25E-01	1.99E-05	6524	
13	rs2148443	22885650	3.12E-01	SACS	16	rs244810	82827257	8.79E-01	KCNG4	-	4.81E-07	7.39E-01	4.61E-05	6525	
5	rs6870688	93391891	8.26E-02	FAM172A	8	rs3134521	96868093	2.74E-01	N/A	-	4.81E-07	2.96E-01	3.12E-06	6526	
5	rs1026985	1.79E+08	9.77E-02	N/A	12	rs10848548	1653512	6.14E-01	ADIPOR2	-	4.81E-07	4.86E-01	3.35E-04	6527	
1	rs758518	1.17E+08	9.71E-02	CD58	4	rs2192356	15033689	5.94E-01	C1QTNF7	-	4.81E-07	8.05E-01	6.11E-04	6528	
7	rs1880957	80192165	6.79E-01	SEMA3C	7	rs10464592	96068622	9.27E-01	N/A	-	4.82E-07	6.59E-01	3.76E-04	6529	
6	rs1199389	8336115	7.69E-01	N/A	17	rs638538	65727723	5.11E-01	N/A	-	4.82E-07	6.63E-02	5.41E-03	6530	
6	rs500110	1.48E+08	2.41E-01	N/A	1	rs10864334	8142693	6.24E-01	N/A	-	4.82E-07	2.55E-01	2.33E-03	6531	
6	rs500110	1.48E+08	2.41E-01	N/A	1	rs7524007	8140793	6.24E-01	N/A	-	4.82E-07	2.55E-01	2.33E-03	6532	
6	rs500110	1.48E+08	2.41E-01	N/A	1	rs11121117	8139890	6.24E-01	N/A	-	4.82E-07	2.98E-01	1.96E-03	6533	
5	rs877886	7712279	7.77E-01	ADCY2	13	rs1326426	88073196	2.43E-01	N/A	-	4.82E-07	4.62E-01	2.13E-05	6534	
5	rs7700862	7699404	7.77E-01	ADCY2	13	rs1326426	88073196	2.43E-01	N/A	-	4.82E-07	4.82E-01	2.24E-05	6535	
2	rs1177234	61113567	5.69E-01	PUS10	8	rs2726555	59947966	8.90E-01	TOX	-	4.82E-07	1.58E-01	7.37E-04	6536	
6	rs1514348	1.52E+08	6.65E-01	ESR1	3	rs7632980	64602857	1.06E-01	ADAMTS9	-	4.82E-07	4.80E-01	7.48E-05	6537	
16	rs7188406	70179708	1.42E-01	TAT	20	rs6123045	49552249	4.33E-01	NFATC2	-	4.82E-07	6.89E-01	9.12E-06	6538	
5	rs10056967	33089900	7.00E-01	N/A	10	rs1925819	15492765	9.69E-01	N/A	-	4.82E-07	4.35E-01	1.15E-06	6539	
6	rs7761614	97494900	7.25E-01	KLHL32	19	rs740867	61886961	1.13E-02	ZNF835	-	4.82E-07	3.50E-02	4.51E-06	6540	
6	rs2252551	34730654	3.57E-01	C6orf106	5	rs2338637	72037229	8.11E-02	N/A	MHC	4.82E-07	3.50E-02	4.51E-06	6541	
5	rs10942656	91323193	9.67E-01	N/A	8	rs10098729	1.29E+08	6.01E-02	N/A	-	4.82E-07	6.29E-02	5.38E-03	6542	
6	rs2815003	34743787	3.57E-01	C6orf106	5	rs2338637	72037229	8.11E-02	N/A	MHC	4.82E-07	5.58E-01	2.01E-04	6543	
5	rs2964145	5401640	4.16E-01	N/A	8	rs10488368	180568	1.15E-01	ZNF596	-	4.82E-07	7.96E-01	6.55E-05	6544	
1	rs10889187	60783346	3.61E-01	N/A	5	rs10056028	1.69E+08	9.38E-02	SLIT3	-	4.82E-07	5.88E-01	1.11E-05	6545	
1	rs11207633	60779770	3.61E-01	N/A	5	rs10056028	1.69E+08	9.38E-02	SLIT3	-	4.82E-07	6.62E-01	1.54E-05	6546	
8	rs10216986	3032515	3.00E-01	CSMD1	8	rs1126681	97674976	8.90E-01	SDC2	-	4.83E-07	4.43E-01	3.30E-06	6547	
8	rs17319192	3032637	3.00E-01	CSMD1	8	rs1126681	97674976	8.90E-01	SDC2	-	4.83E-07	5.10E-01	4.34E-06	6548	
2	rs10803494	1.44E+08	8.53E-01	ARHGAP15	20	rs4811626	53812550	1.41E-01	N/A	-	4.83E-07	7.95E-01	2.30E-05	6549	
1	rs2886208	2.43E+08	9.22E-01	EFCAB2	10	rs6480209	68177427	3.49E-03	CTNN/A3	-	4.83E-07	6.27E-01	3.94E-05	6550	
1	rs203777	1.66E+08	5.46E-01	BRP44	13	rs9574952	81200154	3.28E-02	N/A	-	4.83E-07	6.49E-01	4.17E-05	6551	
1	rs1470438	1.02E+08	6.01E-01	N/A	5	rs6860945	16600195	7.78E-01	FAM134B	-	4.83E-07	7.34E-01	1.71E-04	6552	
11	rs3902836	1.13E+08	3.61E-01	TMPRSS5	16	rs8047350	73236745	7.81E-01	RFWD3	-	4.83E-07	7.53E-01	8.52E-05	6553	
3	rs4855919	1.18E+08	8.75E-01	N/A	10	rs10822321	66041735	2.75E-01	N/A	-	4.83E-07	8.01E-01	3.14E-05	6554	
6	rs16882886	52673487	2.45E-01	TMEM14A	7	rs12670938	10734918	5.00E-01	N/A	-	4.83E-07	2.17E-01	4.44E-06	6555	
1	rs4465172	2.13E+08	6.30E-02	PTPN14	18	rs9960247	10269250	3.17E-01	N/A	-	4.83E-07	9.75E-01	4.83E-05	6556	
1	rs12037907	67745023	9.25E-01	N/A	3	rs11927581	29043754	5.63E-02	N/A	-	4.83E-07	2.35E-01	2.18E-05	6557	
6	rs2021678	1.02E+08	2.43E-01	GRIK2	20	rs6074272	1130173	9.26E-01	C20orf46	-	4.83E-07	7.08E-01	1.04E-05	6558	
3	rs11715745	63466181	5.22E-02	SYNPR	3	rs12490085	1.79E+08	1.74E-01	N/A	-	4.83E-07	8.53E-01	1.80E-05	6559	
1	rs16860761	1.67E+08	2.55E-01	XCL2	22	rs9626344	44236582	4.46E-01	N/A	-	4.83E-07	N/A	N/A	6560	
2	rs1027901	2.11E+08	6.72E-02	LANCL1	16	rs8060485	57460142	3.78E-01	N/A	-	4.83E-07	7.97E-01	2.06E-04	6561	
11	rs984333	25822965	3.11E-01	N/A	22	rs9616022	45267548	9.31E-01	CELSR1	-	4.83E-07	2.04E-01	7.59E-03	6562	
4	rs4862612	1.87E+08	6.27E-04	N/A	22	rs1012480	24661264	1.12E-01	MYO18B	-	4.83E-07	2.58E-01	7.88E-04	6563	
4	rs7665758	46652454	1.94E-01	GABRA4	17	rs8068010	4278869	2.49E-01	SPNS3	-	4.83E-07	3.40E-01	3.30E-04	6564	
11	rs486621	78603685	2.89E-02	ODZ4	17	rs1515022	74017503	3.27E-01	DN/AH17	-	4.83E-07	6.26E-01	4.73E-04	6565	
4	rs10003340	38385826	9.28E-01	KLF3	12	rs11061312	1.3E+08	8.31E-01	GPR133	-	4.83E-07	4.79E-01	1.63E-05	6566	
1	rs7550796	2.02E+08	8.28E-01	SOX13	10	rs10822248	65346801	1.22E-02	N/A	-	4.83E-07	2.51E-01	5.09E-06	6567	
6	rs1322197	1.65E+08	1.36E-02	N/A	17	rs7220603	13232871	1.56E-01	N/A	-	4.83E-07	6.38E-01	4.85E-05	6568	
7	rs4947404	52263829	1.33E-01	N/A	10	rs1249328	28674932	3.44E-01	N/A	-	4.84E-07	5.56E-01	1.57E-03	6569	
3	rs6799396	1.51E+08	4.55E-01	N/A	18	rs4890245	38853168	5.24E-01	RIT2	-	4.84E-07	1.00E-01	1.80E-06	6570	
3	rs6799396	1.51E+08	4.55E-01	N/A	18	rs9962882	38855296	5.24E-01	RIT2	-	4.84E-07	1.27E-01	2.65E-06	6571	
5	rs1993657	1.14E+08	8.75E-01	KCNN2	21	rs2826757	21550171	5.55E-01	NCAM2	-	4.84E-07	5.02E-01	2.75E-04	6572	
4	rs13144189	1.79E+08	4.04E-01	LOC285501	10	rs10736128	1E+08	7.48E-01	N/A	-	4.84E-07	8.56E-01	2.24E-05	6573	
3	rs2133952	1.58E+08	1.90E-01	N/A	8	rs7835921	96345468	3.63E-02	C8orf37	-	4.84E-07	2.68E-01	2.01E-03	6574	
12	rs10859814	93902069	5.59E-01	NDUFA12	15	rs1693534	57069254	3.42E-01	RNF111	-	4.84E-07	N/A	N/A	6575	
12	rs4923674	93910102	5.59E-01	NDUFA12	15	rs1693534	57069254	3.42E-01	RNF111	-	4.84E-07	N/A	N/A	6576	
7	rs6964347	3673289	2.83E-02	SDK1	20	rs11086468	36229826	8.53E-01	TGM2	-	4.84E-07	N/A	N/A	6577	
7	rs6964347	3673289	2.83E-02	SDK1	20	rs761002	36230414	8.53E-01	TGM2	-	4.84E-07	N/A	N/A	6578	
13	rs12877189	36340165	2.54E-02	SMAD9	15	rs12912995	88809540	2.59E-01	IQGAP1	-	4.84E-07	5.94E-01	2.68E-04	6579	
1	rs10489163	30665013	6.12E-01	N/A	11	rs2510957	94010968	1.52E-01	PIWIL4	-	4.84E-07	4.96E-01	1.34E-04	6580	
18	rs1421113	26355659	8.56E-01	N/A	21	rs2826880	21826525	2.06E-01	NCAM2	-	4.84E-07	6.39E-01	2.94E-04	6581	
4	rs9312270	1.82E+08	3.77E-01	N/A	17	rs11650777	65856078	3.76E-01	N/A	-	4.84E-07	6.76E-01	3.62E-04	6582	
4	rs1872293	1.43E+08	3.52E-01	INPP4B	8	rs10454341	33707440	2.59E-02	N/A	-	4.84E-07	9.64E-01	1.38E-04	6583	
4	rs4566706	1.89E+08	7.37E-01	N/A	5	rs38065	10037418	3.07E-01	N/A	-	4.84E-07	1.61E-01	3.87E-06	6584	
6	rs4712047	13698164	7.40E-01	N/A	4	rs9307395	1.15E+08	3.28E-01	CAMK2D	-	4.84E-07	4.85E-01	1.08E-05	6585	
1	rs9970083	2.13E+08	5.77E-01	SMYD2	13	rs12584299	1.08E+08	7.00E-02	N/A	-	4.84E-07	6.06E-01	2.67E-04	6586	
2	rs6704827	41869279	6.25E-01	N/A	12	rs4931000	32032762	8.59E-01	C12orf35	-	4.84E-07	9.69E-01	5.09E-04	6587	
10	rs17127304	1.12E+08	2.93E-01	N/A	14	rs17257668	24505046	4.07E-01	STXBP6	-	4.85E-07	3.23E-01	3.14E-05	6588	
1	rs10492947	5622062	9.03E-01	N/A	4	rs3919788	1.79E+08	5.02E-01	LOC285501	-	4.85E-07	5.51E-01	2.80E-03	6589	
7	rs10499441	14472219	4.24E-01	DGKB	16	rs7196165	65036953	9.74E-02	BEAN	-	4.85E-07	1.36E-01	2.16E-06	6590	
2	rs12470592	79662790	4.09E-02	CTNN/A2	13	rs7337804	35000523	9.71E-01	NBEA	-	4.85E-07	2.15E-01	8.39E-06	6591	
3	rs6799396	1.51E+08	4.55E-01	N/A	18	rs9945309	38883414	5.22E-01	RIT2	-	4.85E-07	2.64E-01	1.04E-05	6592	
3	rs7609823	24354748	5.47E-01	THRB	14	rs1241771	91760538	9.11E-01	N/A	-	4.85E-07	4.78E-01	1.79E-05	6593	
3	rs7609823	24354748	5.47E-01	THRB	14	rs1241768	91757980	9.11E-01	N/A	-	4.85E-07	6.07E-01	3.00E-05	6594	
3	rs2629396	1.21E+08	9.07E-01	CD80	18	rs7240925	20484577	1.06E-01	N/A	-	4.85E-07	1.49E-01	7.37E-07	6595	
2	rs1011397	1.44E+08	4.25E-01	GTDC1	18	rs11080562	12260142	9.88E-01	CIDEA	-	4.85E-07	7.14E-01	1.51E-05	6596	
7	rs2108554	12549605	4.82E-02	N/A	7	rs4377888	85091282	6.48E-01	N/A	-	4.85E-07	3.10E-01	6.53E-06	6597	
7	rs10251901	1.21E+08	2.05E-01	C7orf58	16	rs9302822	6482926	8.98E-01	A2BP1	-	4.85E-07	6.97E-01	3.00E-05	6598	
11	rs441703	29779181	9.14E-01	N/A	13	rs586205	21263974	6.44E-01	N/A	-	4.85E-07	7.44E-01	1.72E-05	6599	
4	rs12507136	1.18E+08	2.41E-01	N/A	11	rs3096716	86134830	1.22E-01	N/A	-	4.85E-07	7.18E-01	9.06E-05	6600	
13	rs7987351	1.12E+08	7.57E-01	C13orf28	18	rs4600536	1767013	3.47E-02	N/A	-	4.85E-07	7.70E-01	1.79E-04	6601	
5	rs160874	3114140	3.77E-01	N/A	10	rs1710313	1.28E+08	7.92E-01	ADAM12	-	4.85E-07	1.24E-01	3.10E-03	6602	
1	rs1074541	89282087	8.51E-02	GBP1	13	rs2321995	58577784	3.86E-01	N/A	-	4.85E-07	5.09E-01	1.38E-05	6603	
8	rs1508569	1.19E+08	4.47E-01	N/A	14	rs8014143	33197925	4.91E-01	NPAS3	-	4.85E-07	9.15E-01	1.26E-04	6604	
13	rs7335635	35814985	6.44E-01	SPG20	19	rs17761865	57016046	2.07E-01	FPR3	-	4.85E-07	6.29E-02	1.39E-02	6605	
4	rs1558707	12459848	3.38E-01	N/A	10	rs2503872	43113631	8.14E-01	N/A	-	4.85E-07	2.65E-01	6.04E-04	6606	

SNP A					SNP B						Interaction P			Ranking	Cluster in top 100 interactions
CHR	SNP	Location	gle locus P va	Gene	CHR	SNP	Location	ingle locus P valu	Gene	MHC region	Stage 1	Stage 2	Combined		
14	rs1241517	24694856	8.12E-01	N/A	19	rs1012531	57810090	8.10E-02	ZNF83	-	4.85E-07	9.06E-01	1.24E-04	6607	
7	rs6460519	68551289	6.83E-01	N/A	13	rs7321128	59211318	8.85E-01	DIAPH3	-	4.85E-07	4.65E-01	2.24E-05	6608	
7	rs12718664	52495255	1.01E-01	N/A	18	rs7233920	35670316	3.79E-02	N/A	-	4.85E-07	8.74E-01	1.63E-05	6609	
4	rs6822800	78325465	7.96E-02	CCNG2	17	rs10432046	28661025	4.68E-01	ACCN1	-	4.85E-07	3.02E-01	3.19E-06	6610	
2	rs3771150	1.02E+08	9.55E-01	IL18RAP	11	rs7119726	70136496	4.88E-02	SHANK2	-	4.86E-07	4.73E-01	9.12E-06	6611	
1	rs2795425	2.31E+08	5.94E-01	N/A	8	rs428838	88550018	1.17E-01	N/A	-	4.86E-07	9.27E-02	5.14E-07	6612	
5	rs1527820	1.66E+08	7.85E-01	N/A	9	rs1001389	3574543	8.34E-01	N/A	-	4.86E-07	2.06E-01	1.20E-03	6613	
5	rs2650353	1.66E+08	7.85E-01	N/A	9	rs1001389	3574543	8.34E-01	N/A	-	4.86E-07	2.06E-01	1.20E-03	6614	
2	rs4673340	2.07E+08	3.56E-01	N/A	7	rs18	11564000	3.26E-01	THSD7A	-	4.86E-07	4.07E-01	1.29E-05	6615	
8	rs4909895	1.36E+08	7.36E-01	N/A	13	rs1340741	68418063	2.94E-01	N/A	-	4.86E-07	9.83E-02	2.06E-06	6616	
12	rs9435	22734474	9.98E-01	ETNK1	13	rs9537164	55013404	7.89E-01	N/A	-	4.86E-07	8.62E-01	2.76E-05	6617	
4	rs6535244	82509042	1.95E-01	N/A	13	rs1998659	43778783	1.18E-01	N/A	-	4.86E-07	1.75E-01	2.23E-03	6618	
9	rs1375211	1.05E+08	7.30E-01	N/A	21	rs9983113	39237186	5.50E-01	N/A	-	4.86E-07	4.10E-03	3.39E-02	6619	
6	rs1399221	1.63E+08	6.79E-01	PACRG	4	rs7683874	7526324	5.54E-01	SORCS2	-	4.86E-07	5.72E-01	3.34E-05	6620	
4	rs6826949	1.15E+08	9.93E-01	ANK2	11	rs1482795	7806921	9.81E-01	OR5P3	-	4.86E-07	6.61E-01	3.43E-04	6621	
18	rs9956612	8989233	8.07E-01	N/A	20	rs232291	43767808	8.10E-01	WFDC10B	-	4.86E-07	6.70E-01	6.39E-04	6622	
5	rs7724626	9477801	1.32E-01	SEMA5A	7	rs974937	34012527	9.62E-01	BMPER	-	4.86E-07	7.95E-01	6.43E-05	6623	
1	rs1006147	15918696	9.12E-01	PLEKHM2	18	rs7237483	42571079	7.40E-01	ST8SIA5	-	4.86E-07	7.17E-01	1.74E-04	6624	
18	rs505140	614631	1.55E-01	CLUL1	21	rs220179	42433102	2.06E-01	UMODL1	-	4.86E-07	8.56E-01	2.47E-05	6625	
2	rs6753156	2.03E+08	9.79E-01	NOP58	4	rs1377916	91916000	9.71E-01	FAM190A	-	4.86E-07	3.83E-01	4.86E-04	6626	
1	rs559798	2.43E+08	9.42E-01	C1orf100	11	rs6485413	43250079	7.17E-01	N/A	-	4.86E-07	9.18E-01	7.09E-05	6627	
5	rs11744711	9752040	3.51E-01	LOC285692	7	rs7797887	47652893	3.67E-01	C7orf65	-	4.87E-07	3.43E-01	6.54E-05	6628	
7	rs17170878	1.48E+08	2.12E-01	CNTN/AP2	17	rs10871516	245185	3.19E-01	C17orf97	-	4.87E-07	3.47E-01	4.63E-04	6629	
5	rs3849045	1.37E+08	5.74E-01	BRD8	18	rs1893152	2831862	7.81E-02	EMILIN2	-	4.87E-07	7.84E-01	1.40E-04	6630	
3	rs4839632	1.45E+08	4.77E-02	SLC9A9	17	rs1859962	66620348	5.57E-01	N/A	-	4.87E-07	2.18E-01	8.82E-06	6631	
5	rs32018	66736793	7.99E-01	N/A	10	rs359288	17370186	5.24E-02	N/A	-	4.87E-07	9.60E-01	2.49E-05	6632	
1	rs1562866	2.04E+08	4.19E-02	TMCC2	10	rs10887514	87421528	6.48E-01	GRID1	-	4.87E-07	7.43E-01	4.57E-05	6633	
1	rs10797493	2.32E+08	1.14E-01	N/A	14	rs2060609	42408480	6.01E-01	N/A	-	4.87E-07	4.24E-01	9.65E-04	6634	
8	rs7814198	87310963	1.46E-01	SLC7A13	15	rs4319733	86020665	3.71E-01	N/A	-	4.87E-07	4.93E-01	2.38E-05	6635	
1	rs1603141	2.32E+08	1.14E-01	N/A	14	rs2060609	42408480	6.01E-01	N/A	-	4.87E-07	5.28E-01	6.88E-04	6636	
1	rs554455	65272403	4.25E-01	N/A	10	rs4748702	21053851	8.02E-01	N/A	-	4.87E-07	9.95E-01	8.29E-05	6637	
5	rs819569	72867949	9.30E-01	ANKRA2	18	rs304398	1083206	5.47E-01	N/A	-	4.87E-07	2.90E-01	1.24E-03	6638	
9	rs10117522	26709332	7.51E-01	N/A	12	rs416594	48612443	8.43E-01	AQP2	-	4.87E-07	1.87E-01	1.35E-03	6639	
4	rs4698832	1.11E+08	3.84E-01	N/A	13	rs903777	28365098	2.46E-01	N/A	-	4.87E-07	4.93E-01	1.07E-05	6640	
3	rs2037184	1.89E+08	2.28E-01	N/A	8	rs3110776	1.08E+08	9.57E-02	OXR1	-	4.87E-07	6.35E-01	2.03E-04	6641	
2	rs3771719	1.6E+08	9.21E-01	CD302	18	rs7228959	7066464	6.14E-01	LAMA1	-	4.87E-07	8.67E-01	1.24E-04	6642	
7	rs7788797	18166730	6.32E-01	N/A	11	rs17155314	60320042	3.03E-01	MS4A10	-	4.88E-07	5.05E-01	3.92E-04	6643	
8	rs16939382	77948002	9.53E-01	ZFHX4	8	rs2443779	1.09E+08	9.31E-01	RSPO2	-	4.88E-07	8.42E-01	9.94E-05	6644	
1	rs1339965	2.45E+08	3.41E-02	SMYD3	9	rs557495	1.34E+08	9.85E-01	C9orf171	-	4.88E-07	8.11E-01	1.79E-04	6645	
7	rs1371607	12429581	1.12E-01	VWDE	7	rs2110871	77918484	9.79E-01	MAGI2	-	4.88E-07	9.23E-01	1.18E-04	6646	
10	rs1907350	77864818	9.71E-01	C10orf11	11	rs10790683	1.24E+08	3.46E-01	N/A	-	4.88E-07	N/A	N/A	6647	
10	rs16936162	35928643	4.99E-01	GJD4	13	rs4772827	85761708	1.65E-01	N/A	-	4.88E-07	3.22E-01	1.46E-06	6648	
5	rs10516081	1.71E+08	2.68E-01	RANBP17	7	rs11763596	67061656	1.04E-01	N/A	-	4.88E-07	7.43E-01	3.42E-04	6649	
1	rs7532151	89161532	6.84E-01	CCBL2	20	rs386365	48470468	5.13E-01	N/A	-	4.88E-07	2.16E-01	7.12E-04	6650	
3	rs10513826	1.18E+08	9.08E-01	LSAMP	17	rs1849733	30774032	3.42E-01	SLFN13	-	4.88E-07	8.32E-01	6.45E-05	6651	
5	rs16876434	5907993	9.25E-01	N/A	9	rs544253	6132157	1.14E-01	N/A	-	4.88E-07	3.23E-01	2.23E-04	6652	
1	rs1287949	2.25E+08	1.60E-01	N/A	14	rs8016079	91828198	8.08E-01	N/A	-	4.88E-07	3.75E-01	4.51E-05	6653	
1	rs10910288	2.32E+08	1.00E-01	N/A	14	rs1349250	42409927	6.58E-01	N/A	-	4.88E-07	3.78E-01	1.24E-03	6654	
1	rs11265289	1.58E+08	3.74E-01	C1orf204	3	rs2681092	1.5E+08	1.80E-02	CP	-	4.88E-07	8.87E-01	2.00E-05	6655	
3	rs7618500	1.54E+08	3.66E-01	N/A	16	rs12917932	85008323	5.57E-02	N/A	-	4.88E-07	4.10E-01	9.69E-06	6656	
10	rs1853204	1.15E+08	1.28E-01	N/A	11	rs11213916	1.11E+08	7.82E-01	BTG4	-	4.89E-07	2.47E-01	3.94E-06	6657	
9	rs1505641	80384461	5.83E-01	N/A	19	rs7253114	42680565	8.14E-01	ZNF793	-	4.89E-07	5.53E-01	1.93E-04	6658	
7	rs10233278	1.17E+08	2.30E-01	CTTNBP2	21	rs1783364	24425455	5.03E-01	N/A	-	4.89E-07	2.54E-01	6.06E-06	6659	
7	rs17166711	13075823	7.09E-02	N/A	19	rs4801457	62485375	9.79E-01	ZNF460	-	4.89E-07	6.42E-01	9.87E-06	6660	
7	rs12533128	13071663	7.09E-02	N/A	19	rs4801457	62485375	9.79E-01	ZNF460	-	4.89E-07	7.18E-01	1.43E-05	6661	
7	rs7778887	13071534	7.09E-02	N/A	19	rs4801457	62485375	9.79E-01	ZNF460	-	4.89E-07	7.18E-01	1.43E-05	6662	
13	rs17072214	21281905	5.14E-01	N/A	19	rs12461274	38599376	6.36E-01	PEPD	-	4.89E-07	7.27E-02	8.17E-07	6663	
9	rs7031834	6776734	4.42E-01	KDM4C	13	rs2313626	19586150	8.45E-01	N/A	-	4.89E-07	7.09E-01	3.18E-05	6664	
9	rs11794756	6775243	4.42E-01	KDM4C	13	rs2313626	19586150	8.45E-01	N/A	-	4.89E-07	7.50E-01	3.72E-05	6665	
3	rs458458	8160570	9.96E-01	N/A	9	rs6415812	98927782	6.88E-01	N/A	-	4.89E-07	N/A	N/A	6666	
6	rs9402524	1.34E+08	6.11E-01	N/A	13	rs7322222	96933162	7.88E-01	RAP2A	-	4.89E-07	8.70E-01	4.52E-04	6667	
7	rs1035169	1.04E+08	7.40E-01	ORC5L	13	rs4885537	77875455	1.46E-01	N/A	-	4.89E-07	5.44E-01	1.38E-04	6668	
3	rs7373878	1.26E+08	6.01E-01	ITGB5	9	rs10969854	30743998	5.56E-02	N/A	-	4.89E-07	6.52E-01	2.42E-04	6669	
5	rs7720838	40522653	1.17E-01	N/A	15	rs4238558	29933027	4.94E-01	N/A	-	4.90E-07	6.17E-02	3.38E-03	6670	
4	rs6850999	80720962	6.97E-02	N/A	4	rs17056428	1.72E+08	7.86E-01	N/A	-	4.90E-07	6.76E-01	2.51E-04	6671	
8	rs2442517	6278932	4.91E-01	MCPH1	12	rs7313455	55139498	2.48E-01	TIMELESS	-	4.90E-07	3.25E-02	5.49E-03	6672	
7	rs2001618	1.33E+08	5.22E-01	EXOC4	20	rs6041636	12744624	8.63E-01	N/A	-	4.90E-07	2.50E-01	8.39E-04	6673	
3	rs17070650	64347702	5.24E-01	N/A	3	rs6549181	69197060	3.66E-01	TMF1	-	4.90E-07	2.39E-01	2.20E-06	6674	
8	rs4961205	87723331	7.35E-02	CNGB3	18	rs12232728	40613836	5.35E-02	SETBP1	-	4.90E-07	5.44E-01	2.16E-04	6675	
2	rs6708879	2.27E+08	2.55E-01	N/A	9	rs10120210	94324803	9.58E-01	ECM2	-	4.90E-07	1.87E-01	1.89E-06	6676	
1	rs10926974	2.41E+08	7.49E-01	CEP170	8	rs10112382	1.29E+08	6.80E-01	N/A	-	4.90E-07	8.43E-01	7.38E-05	6677	
6	rs9387896	1.21E+08	2.65E-01	N/A	1	rs972099	1.71E+08	9.83E-01	N/A	-	4.90E-07	8.86E-01	3.08E-04	6678	
8	rs2172350	19573855	2.20E-02	CSGALN/ACT1	13	rs1077756	74338831	4.69E-02	N/A	-	4.90E-07	8.95E-01	4.94E-05	6679	
6	rs12209486	98123109	8.68E-02	N/A	19	rs1270284	39976681	5.42E-01	N/A	-	4.90E-07	8.98E-01	6.52E-05	6680	
2	rs4343438	1.86E+08	1.16E-01	N/A	3	rs7433389	18249117	7.25E-01	N/A	-	4.90E-07	3.47E-01	1.15E-05	6681	
1	rs419145	32951743	3.53E-01	SYNC	2	rs1220224	34660284	6.29E-01	N/A	-	4.90E-07	7.39E-01	5.80E-05	6682	
6	rs2523864	31126525	5.29E-01	HCG22	6	rs7750269	31379136	8.58E-01	N/A	MHC	4.91E-07	1.08E-01	1.55E-06	6683	
4	rs9993773	80653628	5.97E-02	N/A	7	rs16170	24263983	2.04E-01	N/A	-	4.91E-07	1.74E-01	3.83E-06	6684	
1	rs7554674	68501106	5.41E-01	N/A	5	rs6892958	1.47E+08	1.16E-01	JAKMIP2	-	4.91E-07	9.12E-01	2.75E-04	6685	
6	rs1555772	1.22E+08	4.39E-01	N/A	3	rs656571	74400453	1.50E-01	CNTN3	-	4.91E-07	2.43E-01	5.33E-06	6686	
5	rs34022	1.42E+08	1.65E-01	FGF1	18	rs16973056	12389481	5.41E-01	SLMO1	-	4.91E-07	6.18E-01	2.32E-04	6687	
6	rs9505257	7626569	6.10E-01	N/A	2	rs2003298	11003601	5.14E-01	N/A	-	4.91E-07	1.51E-01	4.70E-03	6688	
3	rs3111310	23484601	5.09E-01	UBE2E2	10	rs12220213	1.32E+08	4.15E-01	N/A	-	4.91E-07	9.07E-02	2.60E-06	6689	
4	rs7668697	42598764	9.93E-01	GRXCR1	4	rs1946687	1.34E+08	8.61E-01	N/A	-	4.91E-07	9.35E-02	2.16E-06	6690	
4	rs1562842	42572559	9.93E-01	GRXCR1	4	rs1946687	1.34E+08	8.61E-01	N/A	-	4.91E-07	9.64E-02	2.07E-06	6691	
13	rs1924578	96267186	2.15E-01	HS6ST3	22	rs713999	46210776	3.83E-01	N/A	-	4.91E-07	6.49E-01	2.71E-04	6692	
2	rs6712681	80377689	6.22E-02	CTNN/A2	17	rs1861954	8139229	4.26E-01	N/A	-	4.91E-07	8.81E-01	2.29E-04	6693	
7	rs11768025	1.43E+08	6.20E-01	FAM115A	14	rs935340	75660959	1.19E-01	N/A	-	4.91E-07	9.15E-02	2.80E-03	6694	
6	rs551962	1.62E+08	3.33E-01	PARK2	8	rs4740033	85925158	5.24E-01	RALYL	-	4.91E-07	3.76E-01	1.68E-06	6695	
1	rs12120788	1.56E+08	4.86E-01	N/A	18	rs546000	20257114	7.45E-01	IMPACT	-	4.91E-07	1.71E-02	3.81E-08	6696	
1	rs12120788	1.56E+08	4.86E-01	N/A	18	rs604345	20257300	7.45E-01	IMPACT	-	4.91E-07	1.71E-02	3.81E-08	6697	
9	rs4977334	19839403	5.36E-01	N/A	10	rs2670223	79797061	9.54E-02	N/A	-	4.91E-07	2.04E-01	5.98E-06	6698	
14	rs2150167	47185921	4.05E-01	MDGA2	16	rs734710	82831935	7.58E-01	KCNG4	-	4.91E-07	4.48E-01	5.52E-04	6699	
2	rs547773	30767885	2.33E-01	N/A	10	rs4752697	1.16E+08	8.31E-01	ABLIM1	-	4.91E-07	2.23E-01	3.22E-06	6700	
2	rs12618646	50025162	8.47E-01	NRXN1	21	rs2822733	14819910	9.37E-01	SAMSN1	-	4.91E-07	6.46E-01	2.46E-04	6701	
4	rs13110363	1.63E+08	2.81E-01	FSTL5	13	rs671810	1.01E+08	7.54E-02	N/ALCN	-	4.91E-07	6.74E-01	9.35E-06	6702	
6	rs879882	31247431	7.61E-01	TCF19	6	rs7761965	31381474	9.15E-01	N/A	MHC	4.92E-07	1.25E-02	7.95E-08	6703	
1	rs6701300	8123277	1.37E-01	N/A	10	rs10794596	1.25E+08	2.89E-01	HMX3	-	4.92E-07	4.88E-02	9.45E-03	6704	
1	rs10493248	58503366	7.66E-01	DAB1	10	rs10509957	1.14E+08	5.37E-01	TECTB	-	4.92E-07	6.74E-01	3.94E-04	6705	
1	rs2148210	5278276	2.74E-01	N/A	4	rs1497400	1.44E+08	6.31E-01	INPP4B	-	4.92E-07	8.21E-01	7.40E-05	6706	
6	rs2883118	75699704	3.70E-01	N/A	14	rs712285	33696953	8.59E-01	N/A	-	4.92E-07	2.56E-01	1.20E-03	6707	
13	rs7335534	33608780	6.41E-01	N/A	18	rs949292	56319787	7.64E-01	N/A	-	4.92E-07	4.38E-01	1.97E-05	6708	
10	rs10905918	11288555	6.56E-01	CUGBP2	16	rs7500313	86968379	4.47E-01	N/A	-	4.92E-07	N/A	N/A	6709	
11	rs11600456	1.28E+08	4.18E-01	ETS1	17	rs739559	65001825	1.86E-02	MAP2K6	-	4.92E-07	1.62E-01	5.96E-03	6710	
9	rs10739815	1.04E+08	1.71E-02	N/A	14	rs1998355	29113020	8.24E-01	PRKD1	-	4.92E-07	7.06E-01	2.28E-04	6711	
3	rs12490596	24979491	7.14E-01	N/A	17	rs9908987	60878953	1.38E-01	N/A	-	4.92E-07	1.61E-01	4.15E-03	6712	
9	rs1998247	17621552	3.02E-01	SH3GL2	9	rs7033375	27690428	7.41E-01	N/A	-	4.92E-07	2.35E-01	4.64E-06	6713	
8	rs3864665	62093567	7.26E-01	N/A	22	rs3761445	36925357	8.56E-01	PLA2G6	-	4.92E-07	9.11E-01	5.44E-05	6714	
5	rs10942722	74219900	4.09E-01	N/A	18	rs497254	54029897	3.02E-01	NEDD4L	-	4.92E-07	2.51E-01	6.40E-04	6715	
3	rs6549009	85169093	6.19E-01	CADM2	17	rs2261081	899093	4.41E-01	ABR	-	4.92E-07	N/A	N/A	6716	
3	rs808887	1.2E+08	4.38E-01	N/A	15	rs8041396	78443883	2.09E-02	N/A	-	4.92E-07	1.35E-01	4.60E-03	6717	
9	rs7849191	4978761	2.15E-01	JAK2	10	rs10762785	79894711	5.77E-01	N/A	-	4.92E-07	2.58E-01	2.47E-06	6718	

SNP A					SNP B						Interaction P			Ranking	Cluster in top 100 interactions
CHR	SNP	Location	gle locus P va	Gene	CHR	SNP	Location	Single locus P valu	Gene	MHC region	Stage 1	Stage 2	Combined		
14	rs11621843	92185877	4.54E-01	RIN3	16	rs16959429	56467155	4.05E-01	CNGB1	-	4.92E-07	3.27E-01	6.32E-04	6719	
1	rs3736118	53456912	3.93E-01	CPT2	17	rs4794122	45683412	2.10E-01	N/A	-	4.92E-07	7.16E-01	5.03E-05	6720	
14	rs11621843	92185877	4.54E-01	RIN3	16	rs247032	56470180	4.05E-01	CNGB1	-	4.92E-07	7.30E-01	1.78E-04	6721	
1	rs16860526	1.67E+08	3.67E-01	N/A	14	rs4981439	22317163	4.94E-01	SLC7A7	-	4.92E-07	3.81E-01	2.33E-04	6722	
1	rs10925374	2.36E+08	2.21E-01	RYR2	10	rs7086823	56607632	3.27E-01	N/A	-	4.92E-07	N/A	N/A	6723	
6	rs9374553	1.16E+08	2.47E-01	N/A	14	rs1890982	1.04E+08	9.79E-01	KIF26A	-	4.93E-07	1.19E-02	7.57E-03	6724	
6	rs2812489	1.3E+08	6.60E-01	N/A	5	rs7710314	85811870	4.27E-01	N/A	-	4.93E-07	N/A	N/A	6725	
6	rs807898	1.65E+08	1.00E+00	N/A	12	rs10851011	1.18E+08	5.40E-02	N/A	-	4.93E-07	6.10E-01	4.08E-06	6726	
3	rs587612	1.95E+08	5.96E-01	N/A	17	rs7216812	11882208	3.12E-01	MAP2K4	-	4.93E-07	7.51E-01	5.47E-05	6727	
20	rs2423808	14469106	4.81E-01	MACROD2	21	rs4920107	42755622	7.96E-01	RSPH1	-	4.93E-07	6.74E-01	1.52E-04	6728	
3	rs1561030	1.79E+08	7.50E-01	N/A	10	rs1152699	1.27E+08	3.71E-01	CTBP2	-	4.93E-07	6.90E-01	5.79E-05	6729	
2	rs3112970	1.8E+08	7.31E-01	ZNF385B	3	rs358741	1.57E+08	8.76E-01	C3orf33	-	4.93E-07	9.89E-01	6.71E-04	6730	
2	rs10196671	1.87E+08	6.51E-01	N/A	10	rs10823403	70967730	3.46E-01	N/A	-	4.93E-07	N/A	N/A	6731	
3	rs1039878	68557818	6.98E-01	FAM19A1	4	rs10018782	1.87E+08	7.83E-01	SORBS2	-	4.93E-07	2.86E-01	3.17E-06	6732	
2	rs6758220	74191092	7.27E-01	TET3	3	rs4450813	1.35E+08	8.87E-02	RYK	-	4.93E-07	5.07E-01	6.13E-06	6733	
9	rs7853037	7007391	6.43E-01	KDM4C	13	rs7985904	97330758	3.60E-01	N/A	-	4.93E-07	7.68E-01	4.48E-05	6734	
15	rs17561907	36288221	7.51E-01	N/A	16	rs3764276	29580704	3.61E-01	QPRT	-	4.93E-07	9.96E-01	1.54E-04	6735	
12	rs7298815	5189961	9.89E-01	N/A	17	rs7210806	59843219	8.77E-01	N/A	-	4.94E-07	8.05E-01	8.73E-05	6736	
5	rs2913774	1.78E+08	8.47E-01	COL23A1	8	rs7007275	54269163	8.49E-01	N/A	-	4.94E-07	1.27E-01	3.53E-03	6737	
5	rs2913774	1.78E+08	8.47E-01	COL23A1	8	rs7844660	54268837	8.49E-01	N/A	-	4.94E-07	1.99E-01	2.31E-03	6738	
16	rs1395577	7596973	8.24E-01	A2BP1	21	rs220153	42417692	2.48E-01	UMODL1	-	4.94E-07	4.92E-01	3.30E-04	6739	
2	rs10932374	2.12E+08	3.38E-01	ERBB4	3	rs4677287	73598809	1.07E-01	PDZRN3	-	4.94E-07	2.09E-01	1.68E-06	6740	
7	rs389153	1.53E+08	3.08E-02	N/A	8	rs7016292	5789482	9.96E-01	N/A	-	4.94E-07	N/A	N/A	6741	
6	rs332496	93512596	3.60E-01	N/A	8	rs11203826	16943551	5.66E-01	EFHA2	-	4.94E-07	4.68E-01	1.35E-04	6742	
6	rs2353783	1.56E+08	9.80E-01	N/A	14	rs181484	68427927	4.63E-01	ACTN1	-	4.94E-07	4.52E-01	2.71E-04	6743	
17	rs1971773	8574035	7.61E-01	CCDC42	19	rs3843043	46125771	3.99E-01	CYP2B7P1	-	4.95E-07	2.67E-01	5.23E-04	6744	
17	rs1971773	8574035	7.61E-01	CCDC42	19	rs3844443	46123775	3.99E-01	CYP2B7P1	-	4.95E-07	2.67E-01	5.23E-04	6745	
1	rs1335647	89605052	9.73E-01	GBP6	15	rs16974359	92723701	5.27E-01	MCTP2	-	4.95E-07	4.03E-01	2.22E-05	6746	
8	rs12541274	5485819	3.04E-01	N/A	15	rs1516855	44629062	7.44E-01	N/A	-	4.95E-07	2.42E-02	4.49E-03	6747	
15	rs2017626	22983935	1.51E-01	SNORD115-2	17	rs1396087	49667564	3.20E-01	N/A	-	4.95E-07	6.31E-01	2.98E-05	6748	
2	rs1347930	42903972	2.65E-01	N/A	18	rs12458297	5441029	5.85E-01	EPB41L3	-	4.95E-07	8.22E-01	1.07E-04	6749	
1	rs1562866	2.04E+08	4.19E-02	TMCC2	10	rs7097285	87420101	6.66E-01	GRID1	-	4.95E-07	8.82E-01	3.80E-05	6750	
2	rs16987836	20627392	4.24E-01	N/A	2	rs11902056	1.43E+08	5.87E-01	KYNU	-	4.95E-07	5.77E-02	4.34E-03	6751	
3	rs11717277	54195911	9.71E-01	CACN/A2D3	20	rs6039487	9453078	9.95E-01	PAK7	-	4.95E-07	3.20E-01	1.23E-03	6752	
1	rs4384209	66051713	8.66E-01	PDE4B	12	rs7976243	7977350	2.24E-01	SLC2A3	-	4.95E-07	2.52E-01	9.43E-04	6753	
6	rs6930429	1.44E+08	6.49E-01	PLAGL1	3	rs6772225	55251558	1.06E-01	N/A	-	4.95E-07	7.74E-02	4.54E-03	6754	
11	rs930722	87740697	6.73E-01	N/A	17	rs2628299	50515945	7.26E-01	STXBP4	-	4.95E-07	3.13E-01	2.68E-04	6755	
2	rs2195610	2.18E+08	2.05E-01	N/A	8	rs13273672	11649790	4.06E-01	NEIL2	-	4.95E-07	4.58E-01	1.36E-03	6756	
13	rs4284530	84153070	5.92E-01	N/A	16	rs223881	55944067	4.22E-01	CX3CL1	-	4.96E-07	7.61E-01	1.16E-04	6757	
1	rs1572750	2.23E+08	1.66E-01	DN/AH14	15	rs8035632	83499613	4.67E-01	PDE8A	-	4.96E-07	4.76E-01	2.06E-05	6758	
1	rs4570460	2.01E+08	8.03E-02	KDM5B	8	rs2447236	1.08E+08	8.29E-02	N/A	-	4.96E-07	5.38E-01	3.89E-05	6759	
5	rs10078721	92446445	8.04E-01	N/A	15	rs12148268	56546751	6.34E-01	LIPC	-	4.96E-07	7.55E-01	8.46E-05	6760	
7	rs10280848	67742124	8.09E-01	N/A	10	rs2153345	26258401	4.98E-01	MYO3A	-	4.96E-07	2.55E-01	8.84E-04	6761	
6	rs236258	79159396	7.91E-02	N/A	1	rs232812	58851836	9.12E-01	N/A	-	4.96E-07	2.84E-01	3.67E-04	6762	
4	rs1996182	25066599	9.95E-01	N/A	5	rs354670	1.01E+08	3.17E-01	N/A	-	4.96E-07	5.87E-01	3.58E-05	6763	
9	rs10818322	1.21E+08	7.03E-01	N/A	18	rs633163	2287887	9.30E-01	N/A	-	4.96E-07	4.50E-01	1.21E-03	6764	
1	rs164983	91339045	5.42E-01	N/A	1	rs17360740	1.76E+08	3.77E-01	N/A	-	4.96E-07	5.46E-01	7.64E-05	6765	
6	rs4145199	40459819	6.11E-01	LRFN2	8	rs11779434	1.44E+08	6.20E-01	N/A	-	4.96E-07	6.92E-01	2.11E-05	6766	
6	rs9493096	1.32E+08	3.50E-01	N/A	12	rs3858688	1.02E+08	6.56E-01	N/A	-	4.96E-07	N/A	N/A	6767	
6	rs10872455	1.36E+08	8.68E-01	PDE7B	2	rs10804036	1.54E+08	1.46E-02	N/A	-	4.96E-07	9.47E-02	3.79E-06	6768	
14	rs12586452	53275921	9.08E-01	N/A	15	rs6416582	91537949	3.44E-01	N/A	-	4.96E-07	6.70E-01	2.79E-05	6769	
1	rs1898240	1.98E+08	5.80E-01	N/A	5	rs2010560	21118841	2.86E-01	N/A	-	4.96E-07	2.22E-01	7.40E-07	6770	
5	rs2968019	58380905	8.06E-01	PDE4D	18	rs1899894	55183633	1.38E-01	LMAN1	-	4.97E-07	4.74E-01	7.55E-06	6771	
3	rs7622138	34629706	1.94E-01	N/A	4	rs969340	55485150	4.31E-01	N/A	-	4.97E-07	9.06E-01	1.40E-04	6772	
1	rs11203366	17530121	9.93E-03	PADI4	13	rs568474	71822612	7.95E-01	N/A	-	4.97E-07	6.83E-01	2.31E-04	6773	
3	rs9832999	1.23E+08	3.65E-01	CASR	11	rs11026661	3328263	3.84E-02	ZNF195	-	4.97E-07	2.43E-01	8.75E-04	6774	
10	rs1679414	4495344	2.71E-01	N/A	16	rs3852716	81170828	4.46E-01	N/A	-	4.97E-07	3.22E-01	1.39E-06	6775	
10	rs7100993	47148027	3.97E-02	ANTXRL	11	rs1459014	13513831	9.75E-01	N/A	-	4.97E-07	2.16E-02	2.87E-07	6776	
3	rs12630584	1.91E+08	1.56E-01	TP63	11	rs11034871	38567581	9.60E-02	N/A	-	4.97E-07	3.04E-01	4.30E-06	6777	
3	rs7650285	1.91E+08	1.56E-01	TP63	11	rs11034871	38567581	9.60E-02	N/A	-	4.97E-07	3.04E-01	4.30E-06	6778	
16	rs2312948	8600664	9.53E-01	N/A	17	rs4789659	69909931	4.36E-02	N/A	-	4.97E-07	3.53E-01	1.60E-04	6779	
2	rs7563917	77066611	7.93E-01	LRRTM4	5	rs160890	3187879	9.49E-01	N/A	-	4.97E-07	5.74E-01	3.15E-04	6780	
2	rs1861612	2.3E+08	6.42E-01	DNER	8	rs13279626	70933356	6.02E-01	N/A	-	4.97E-07	6.74E-02	1.23E-03	6781	
6	rs1190050	1.06E+08	8.50E-02	PREP	3	rs3872720	1.88E+08	8.81E-01	N/A	-	4.97E-07	4.74E-01	3.31E-05	6782	
13	rs9508819	30173913	6.61E-01	N/A	16	rs12933233	85669131	9.81E-01	N/A	-	4.97E-07	3.10E-02	1.25E-02	6783	
5	rs186740	54585874	2.12E-01	DHX29	7	rs10281702	1.46E+08	5.08E-02	CNTN/AP2	-	4.97E-07	3.62E-01	4.52E-04	6784	
3	rs4128782	89320422	6.97E-02	EPHA3	7	rs765966	1.3E+08	4.31E-01	FLJ43663	-	4.97E-07	7.50E-01	6.97E-05	6785	
4	rs7690692	98417796	4.25E-01	N/A	10	rs11593108	1.01E+08	6.50E-02	N/A	-	4.98E-07	2.79E-01	3.43E-06	6786	
3	rs10935384	1.42E+08	7.96E-01	CLSTN2	16	rs11642377	20017154	1.27E-01	N/A	-	4.98E-07	3.01E-01	9.50E-06	6787	
5	rs2194026	87837029	9.49E-01	N/A	8	rs4737930	70046059	6.04E-01	N/A	-	4.98E-07	3.37E-01	4.87E-06	6788	
2	rs6437294	2.41E+08	1.05E-02	OTOS	5	rs4285197	82218715	3.53E-01	N/A	-	4.98E-07	4.13E-01	5.30E-04	6789	
6	rs7759825	11064635	6.26E-01	SYCP2L	8	rs2455052	97754387	4.87E-01	PGCP	-	4.98E-07	N/A	N/A	6790	
4	rs4580651	7892387	6.94E-01	AFAP1	18	rs8086733	27901867	7.72E-01	RNF125	-	4.98E-07	1.56E-01	2.13E-03	6791	
6	rs7761965	31381474	9.15E-01	N/A	6	rs2844575	31442924	5.47E-01	HLA-B	MHC	4.98E-07	2.88E-01	9.53E-06	6792	
12	rs10848896	3622861	8.80E-01	EFCAB4B	14	rs1958037	33354237	1.12E-01	NPAS3	-	4.98E-07	6.07E-02	9.93E-08	6793	
4	rs7657164	1.69E+08	4.42E-01	DDX60	15	rs1898110	45677642	7.99E-01	N/A	-	4.98E-07	1.96E-01	1.32E-03	6794	
1	rs290826	97246295	2.48E-01	N/A	8	rs9649911	30629011	5.56E-01	GTF2E2	-	4.98E-07	3.01E-01	3.05E-05	6795	
12	rs2641558	61465046	3.84E-01	PPM1H	17	rs679210	72557578	6.10E-01	N/A	-	4.98E-07	3.47E-01	4.59E-06	6796	
13	rs9556980	98047555	7.77E-01	N/A	22	rs138606	43358052	3.31E-01	NCRN/A00207	-	4.98E-07	5.02E-01	2.41E-05	6797	
6	rs17141268	5720674	2.41E-01	FARS2	2	rs6715170	42941808	4.89E-01	N/A	-	4.98E-07	5.07E-01	2.38E-05	6798	
8	rs11782575	33614727	1.69E-02	N/A	16	rs130021	3772472	7.22E-01	CREBBP	-	4.98E-07	9.48E-01	6.54E-05	6799	
3	rs2063483	1.7E+08	2.37E-01	N/A	12	rs394119	48591274	8.09E-01	LOC283332	-	4.99E-07	2.31E-02	3.59E-03	6800	
3	rs3851374	1.7E+08	2.37E-01	N/A	12	rs394119	48591274	8.09E-01	LOC283332	-	4.99E-07	2.31E-02	3.59E-03	6801	
8	rs1480391	50270973	3.23E-01	N/A	10	rs11189510	99952263	4.94E-01	C10orf28	-	4.99E-07	4.84E-01	4.82E-04	6802	
8	rs1480391	50270973	3.23E-01	N/A	10	rs4281427	99907747	4.94E-01	C10orf28	-	4.99E-07	4.84E-01	4.82E-04	6803	
2	rs4258776	33626029	7.37E-01	RASGRP3	9	rs10821164	95387050	7.43E-01	FAM120A	-	4.99E-07	3.74E-01	2.82E-04	6804	
2	rs2909448	1.63E+08	9.70E-01	DPP4	11	rs16937773	43949775	8.47E-01	N/A	-	4.99E-07	6.63E-01	1.34E-04	6805	
7	rs10260011	84547292	6.49E-01	SEMA3D	18	rs1551233	42349363	1.86E-01	LOXHD1	-	4.99E-07	5.55E-01	5.02E-04	6806	
6	rs483366	1.62E+08	3.18E-01	PARK2	8	rs1946401	85941322	5.68E-01	RALYL	-	4.99E-07	8.82E-01	3.58E-05	6807	
3	rs9867793	67243323	2.83E-01	N/A	9	rs390253	1.07E+08	3.78E-01	N/A	-	4.99E-07	3.04E-01	5.84E-07	6808	
1	rs3827730	50710436	1.77E-01	FAF1	13	rs9315776	39901022	5.07E-01	LOC646982	-	4.99E-07	4.93E-01	1.32E-05	6809	
3	rs1920230	1.25E+08	1.63E-01	CCDC14	9	rs13291990	1.15E+08	3.84E-01	N/A	-	4.99E-07	6.26E-01	4.75E-05	6810	
1	rs845451	2.08E+08	9.45E-01	SERTAD4	12	rs11612475	4968898	9.94E-01	N/A	-	4.99E-07	6.85E-01	3.15E-04	6811	
17	rs7224942	46821956	2.78E-01	N/A	20	rs6072793	40618938	6.45E-01	PTPRT	-	4.99E-07	4.16E-02	3.89E-06	6812	
17	rs17247848	28008297	5.07E-01	MYO1D	18	rs2051308	46448639	6.07E-01	MAPK4	-	4.99E-07	5.66E-01	1.26E-03	6813	
11	rs4938222	1.15E+08	2.15E-01	N/A	14	rs8012632	32312100	2.02E-02	AKAP6	-	4.99E-07	5.71E-01	1.94E-04	6814	
7	rs17165486	12009756	8.46E-01	N/A	14	rs768750	32017697	2.25E-01	AKAP6	-	4.99E-07	7.75E-01	4.41E-04	6815	
11	rs748807	1.31E+08	6.42E-01	NTM	20	rs6114758	2453124	2.23E-01	TMC2	-	4.99E-07	8.79E-01	3.53E-04	6816	
11	rs1144745	59555589	3.95E-01	PLAC1L	16	rs7206380	78029215	5.90E-01	N/A	-	4.99E-07	9.80E-01	1.09E-04	6817	
1	rs9436717	65607890	9.33E-01	DN/AJC6	4	rs13125519	1.81E+08	2.92E-01	N/A	-	4.99E-07	1.88E-01	2.11E-04	6818	
19	rs1054685	19595539	5.06E-01	PBX4	21	rs2410415	41563272	1.63E-01	BACE2	-	4.99E-07	1.42E-01	6.52E-06	6819	
6	rs942465	17696137	4.97E-01	FAM8A1	7	rs11772815	28357572	3.06E-01	CREB5	-	4.99E-07	5.56E-01	4.99E-04	6820	
3	rs936154	36462496	5.36E-01	STAC	14	rs1408965	36340385	8.09E-01	SLC25A21	-	4.99E-07	6.72E-01	6.20E-05	6821	
5	rs408005	98050220	1.20E-01	N/A	22	rs134848	44422472	9.41E-01	N/A	-	4.99E-07	7.44E-01	5.50E-04	6822	
2	rs4670719	37522991	3.92E-01	N/A	19	rs1293703	58834663	6.87E-01	DPRX	-	4.99E-07	7.64E-01	4.62E-05	6823	
3	rs9882278	7542652	4.90E-01	GRM7	7	rs6945244	32225798	6.04E-01	N/A	-	4.99E-07	8.83E-01	2.14E-05	6824	
9	rs7869846	1767728	3.44E-01	N/A	14	rs11157503	45963326	6.99E-01	N/A	-	5.00E-07	5.37E-02	4.71E-07	6825	
3	rs1284745	1.04E+08	9.89E-01	N/A	13	rs9534986	47665333	6.35E-01	N/A	-	5.00E-07	1.10E-01	1.38E-06	6826	
1	rs2506058	1.92E+08	2.48E-01	N/A	9	rs690232	92422258	7.74E-01	DIRAS2	-	5.00E-07	5.05E-01	6.93E-05	6827	
5	rs17155896	1.03E+08	2.26E-01	N/A	12	rs2047482	1.14E+08	8.50E-01	N/A	-	5.00E-07	N/A	N/A	6828	
7	rs10486920	79532689	9.86E-01	N/A	11	rs1018206	92435804	4.20E-01	N/A	-	5.00E-07	4.94E-02	8.52E-03	6829	
2	rs11899692	47321835	6.25E-01	N/A	3	rs953859	1.46E+08	3.15E-01	N/A	-	5.00E-07	5.67E-01	7.58E-04	6830	

SNP A					SNP B						Interaction P			Ranking	Cluster in top 100 interactions
CHR	SNP	Location	gle locus P va	Gene	CHR	SNP	Location	Single locus P valu	Gene	MHC region	Stage 1	Stage 2	Combined		
7	rs160342	28630355	1.95E-01	CREB5	9	rs4645624	24024180	5.59E-01	N/A	-	5.00E-07	2.82E-02	9.00E-08	6831	
1	rs2126643	1.03E+08	9.08E-01	N/A	8	rs2445353	1.08E+08	7.80E-01	ANGPT1	-	5.00E-07	3.10E-01	1.33E-05	6832	
4	rs6850999	80720962	6.97E-02	N/A	7	rs2390961	24266172	2.98E-01	N/A	-	5.00E-07	2.96E-01	9.37E-06	6833	
9	rs10114562	1.16E+08	3.37E-01	N/A	18	rs1546010	62496449	3.87E-01	N/A	-	5.00E-07	5.76E-01	1.88E-05	6834	
13	rs9541712	68481740	1.99E-01	N/A	20	rs2427204	59640796	8.70E-01	CDH4	-	5.00E-07	7.79E-01	1.06E-04	6835	
5	rs9292435	18993604	9.27E-01	N/A	5	rs34236	1.03E+08	7.49E-01	N/A	-	5.00E-07	9.21E-01	3.54E-04	6836	
4	rs17008912	1.26E+08	4.39E-01	N/A	17	rs2376586	74018260	1.21E-01	DN/AH17	-	5.00E-07	1.29E-01	5.16E-04	6837	
5	rs7711972	40026136	6.97E-01	N/A	21	rs2210268	40685128	9.43E-01	DSCAM	-	5.00E-07	2.24E-01	6.61E-06	6838	
1	rs527762	85701802	6.43E-01	DDAH1	3	rs1389903	30880388	6.08E-01	GADL1	-	5.00E-07	6.03E-01	1.19E-05	6839	
6	rs2858331	32789255	1.42E-01	N/A	10	rs10994392	52200825	6.17E-01	ASAH2B	MHC	5.00E-07	1.48E-01	8.02E-04	6840	
8	rs10504458	70769716	4.74E-01	SLCO5A1	20	rs297740	4364301	7.87E-01	N/A	-	5.00E-07	3.38E-01	2.65E-06	6841	
8	rs1037550	83314011	2.12E-01	N/A	16	rs1990684	48985055	4.69E-01	N/A	-	5.00E-07	4.82E-01	5.26E-04	6842	
5	rs330356	1.25E+08	8.73E-01	N/A	16	rs2526269	2996585	4.66E-01	CLDN9	-	5.00E-07	9.65E-01	1.36E-04	6843	
19	rs2240669	4158940	9.86E-01	ANKRD24	21	rs2834463	34639337	7.52E-01	KCNE2	-	5.00E-07	N/A	N/A	6844	
6	rs7739011	3057529	1.89E-01	BPHL	5	rs11745574	79223631	1.48E-01	N/A	-	5.00E-07	2.93E-01	2.54E-04	6845	
11	rs713217	69288261	4.04E-01	FGF4	13	rs603894	1.02E+08	1.89E-01	C13orf39	-	5.00E-07	1.54E-01	7.11E-07	6846	
2	rs10520221	79044007	5.14E-01	N/A	18	rs2319555	64326814	3.00E-01	N/A	-	5.01E-07	2.45E-02	5.24E-02	6847	
8	rs993883	5179603	1.13E-01	N/A	12	rs3825181	1.28E+08	7.31E-01	SLC15A4	-	5.01E-07	5.36E-01	5.88E-04	6848	
12	rs10862392	80842509	3.70E-01	N/A	17	rs7210733	27591452	4.04E-01	RHOT1	-	5.01E-07	8.12E-03	1.41E-08	6849	
4	rs7661550	46581758	2.94E-01	COX7B2	17	rs17223566	4274255	2.14E-01	SPNS3	-	5.01E-07	5.36E-01	1.84E-04	6850	
8	rs11203975	18564773	3.50E-02	PSD3	11	rs11220164	1.25E+08	5.25E-01	CHEK1	-	5.01E-07	7.76E-01	5.58E-05	6851	
1	rs7418365	9457890	1.94E-01	N/A	1	rs11162519	78852154	7.88E-01	IFI44L	-	5.01E-07	8.90E-01	1.90E-04	6852	
3	rs35837	63988289	2.92E-01	PSMD6	19	rs10410581	58989046	7.77E-01	NLRP12	-	5.01E-07	N/A	N/A	6853	
1	rs11163437	82421530	7.61E-01	N/A	3	rs304077	4509629	3.69E-01	ITPR1	-	5.01E-07	9.61E-02	5.38E-07	6854	
3	rs12490397	21451300	5.76E-01	ZNF385D	5	rs347736	19583914	2.36E-01	CDH18	-	5.01E-07	1.80E-01	1.08E-05	6855	
9	rs10819125	1.28E+08	2.01E-01	N/A	10	rs2132377	84628885	6.41E-01	NRG3	-	5.01E-07	6.23E-01	1.25E-04	6856	
5	rs10746553	9977172	7.24E-01	N/A	19	rs12611086	39318772	1.81E-01	N/A	-	5.01E-07	5.40E-01	1.53E-04	6857	
6	rs3800229	1.09E+08	3.04E-01	FOXO3	13	rs1105576	99178430	6.33E-01	CLYBL	-	5.01E-07	6.29E-01	2.49E-03	6858	
6	rs9377138	1.49E+08	8.74E-01	SASH1	13	rs12430285	30981270	9.66E-01	N/A	-	5.01E-07	7.03E-01	1.04E-04	6859	
4	rs7669469	1.82E+08	9.27E-01	N/A	15	rs1280396	55519020	4.41E-01	CGNL1	-	5.01E-07	7.21E-01	6.96E-05	6860	
1	rs7540516	2.05E+08	1.70E-01	IL19	10	rs1904836	44437128	4.05E-01	N/A	-	5.01E-07	6.43E-01	1.23E-05	6861	
10	rs1413615	99793187	9.45E-01	CRTAC1	13	rs9536324	52443516	1.01E-01	N/A	-	5.01E-07	2.25E-01	4.22E-06	6862	
11	rs1505193	4666863	8.15E-01	OR51E2	17	rs6505400	29580727	9.86E-01	N/A	-	5.01E-07	2.87E-01	1.66E-03	6863	
7	rs4728435	1.38E+08	7.90E-02	N/A	11	rs754410	1.15E+08	3.67E-01	N/A	-	5.01E-07	5.28E-01	8.92E-06	6864	
10	rs10736121	99796738	9.45E-01	CRTAC1	13	rs9536324	52443516	1.01E-01	N/A	-	5.01E-07	N/A	N/A	6865	
6	rs2233984	31187243	4.20E-02	C6orf15	6	rs1265074	31221193	9.61E-01	TCF19	MHC	5.01E-07	5.32E-02	4.58E-07	6866	
6	rs2233984	31187243	4.20E-02	C6orf15	6	rs1265093	31215166	9.61E-01	TCF19	MHC	5.01E-07	5.32E-02	4.58E-07	6867	
6	rs2233984	31187243	4.20E-02	C6orf15	6	rs1966	31215712	9.61E-01	TCF19	MHC	5.01E-07	5.32E-02	4.58E-07	6868	
3	rs1824212	21625652	9.20E-01	ZNF385D	7	rs4304246	43248856	6.72E-01	HECW1	-	5.01E-07	9.86E-02	3.71E-03	6869	
9	rs11103306	1.38E+08	5.50E-01	N/ACC2	11	rs10894417	1.31E+08	8.89E-01	NTM	-	5.01E-07	7.29E-01	1.13E-05	6870	
10	rs12570061	31720584	9.58E-01	ZEB1	13	rs10492659	42440382	9.49E-01	EPSTI1	-	5.02E-07	1.53E-01	1.30E-03	6871	
1	rs9803825	1.08E+08	4.67E-01	VAV3	9	rs11243900	1.35E+08	5.82E-02	C9orf98	-	5.02E-07	3.68E-01	2.89E-05	6872	
1	rs804136	15006255	8.36E-01	KIAA1026	3	rs10804615	1.34E+08	2.80E-02	N/A	-	5.02E-07	8.47E-01	1.11E-04	6873	
3	rs7637803	1.53E+08	3.99E-02	MED12L	8	rs7820557	1.1E+08	7.99E-01	N/A	-	5.02E-07	4.04E-01	9.85E-05	6874	
11	rs4755622	42607106	9.86E-01	N/A	20	rs7264443	46087381	9.11E-01	N/A	-	5.02E-07	3.82E-02	3.99E-07	6875	
6	rs7768952	1.23E+08	5.42E-01	CLVS2	1	rs1552991	1.5E+08	1.63E-01	HRNR	-	5.02E-07	1.10E-01	3.27E-03	6876	
5	rs13190397	21851481	9.72E-01	CDH12	9	rs12375503	30028860	6.52E-01	N/A	-	5.02E-07	5.22E-01	1.47E-05	6877	
5	rs6452000	21840233	9.72E-01	CDH12	9	rs12375503	30028860	6.52E-01	N/A	-	5.02E-07	5.22E-01	1.47E-05	6878	
6	rs9385976	1.01E+08	7.22E-01	MCHR2	11	rs7928739	67590119	9.00E-02	CHKA	-	5.02E-07	5.91E-01	4.41E-05	6879	
6	rs2051630	1.3E+08	3.65E-01	ARHGAP18	20	rs6040993	11880255	9.18E-01	N/A	-	5.02E-07	8.72E-01	2.14E-04	6880	
4	rs9999931	31286083	8.12E-01	N/A	12	rs7974366	12829195	8.78E-01	APOLD1	-	5.02E-07	8.85E-01	5.56E-05	6881	
4	rs7695237	31285919	8.12E-01	N/A	12	rs7974366	12829195	8.78E-01	APOLD1	-	5.02E-07	9.05E-01	5.89E-05	6882	
6	rs2051630	1.3E+08	3.65E-01	ARHGAP18	20	rs6078404	11877122	9.18E-01	N/A	-	5.02E-07	9.95E-01	1.48E-04	6883	
4	rs371796	1.32E+08	1.85E-01	N/A	12	rs4760933	69430025	4.52E-01	PTPRR	-	5.02E-07	8.35E-01	6.23E-05	6884	
10	rs3128720	42693853	1.62E-01	N/A	21	rs724128	18617132	3.79E-01	PRSS7	-	5.02E-07	9.63E-01	1.95E-04	6885	
8	rs6983144	29659120	4.96E-01	C8orf75	20	rs127929	22270223	6.18E-01	N/A	-	5.02E-07	5.66E-01	6.51E-05	6886	
5	rs245820	1.42E+08	4.55E-02	ARHGAP26	18	rs4131293	21026067	4.08E-01	ZNF521	-	5.02E-07	1.52E-01	9.33E-07	6887	
1	rs807247	25993456	5.33E-01	MAN1C1	11	rs1299588	1.01E+08	6.68E-01	KIAA1377	-	5.02E-07	4.19E-01	2.87E-05	6888	
11	rs12418045	94152718	3.13E-01	AMOTL1	16	rs6500840	6795938	6.00E-01	A2BP1	-	5.02E-07	5.18E-01	8.40E-05	6889	
6	rs4947296	31166157	4.31E-02	N/A	6	rs130067	31226490	8.62E-01	TCF19	MHC	5.02E-07	8.53E-02	7.86E-07	6890	
6	rs2072633	32027557	5.29E-01	SKIV2L	18	rs1489169	44129658	2.17E-02	N/A	MHC	5.02E-07	1.23E-01	3.83E-06	6891	
12	rs4763825	12470053	1.21E-01	LOH12CR1	12	rs1402318	76753767	8.81E-01	N/AV3	-	5.02E-07	8.24E-01	8.98E-05	6892	
9	rs10980452	1.12E+08	2.69E-01	SVEP1	11	rs7932321	10480052	8.52E-01	N/A	-	5.02E-07	3.29E-02	5.35E-08	6893	
1	rs10923615	1.19E+08	3.15E-01	N/A	3	rs1383407	78990861	1.92E-01	ROBO1	-	5.02E-07	8.88E-02	9.49E-07	6894	
13	rs1886033	99356474	5.92E-01	CLYBL	20	rs6039885	10299696	3.29E-01	N/A	-	5.03E-07	8.00E-02	1.84E-03	6895	
1	rs3863641	46615803	5.52E-01	FAAH	22	rs2092136	31168255	2.38E-01	BPIL2	-	5.03E-07	2.37E-01	7.93E-06	6896	
10	rs1228187	90428187	9.65E-01	LIPF	21	rs2837688	40829567	3.03E-01	DSCAM	-	5.03E-07	2.58E-01	1.63E-06	6897	
4	rs7656930	1.47E+08	5.35E-01	N/A	7	rs10277450	70001719	2.35E-01	N/A	-	5.03E-07	5.25E-01	1.14E-05	6898	
6	rs6922865	36202166	9.23E-01	MAPK14	9	rs997758	91854240	1.29E-01	N/A	MHC	5.03E-07	8.85E-01	3.95E-04	6899	
1	rs10889978	41634712	1.59E-01	N/A	12	rs7133845	93507755	6.13E-01	TMCC3	-	5.03E-07	9.40E-01	1.52E-04	6900	
3	rs301564	11778927	9.11E-01	N/A	4	rs10032150	1.12E+08	4.59E-01	N/A	-	5.03E-07	4.07E-01	5.91E-04	6901	
8	rs10108007	61310616	4.24E-01	CA8	16	rs4784108	59011776	6.74E-01	N/A	-	5.03E-07	8.03E-01	8.32E-05	6902	
9	rs7860062	84736837	8.07E-01	N/A	12	rs7960369	28605769	9.14E-01	CCDC91	-	5.03E-07	1.61E-01	3.09E-03	6903	
1	rs1339356	2.15E+08	2.22E-01	ESRRG	10	rs12262126	5608776	8.34E-01	N/A	-	5.03E-07	8.69E-01	7.37E-05	6904	
1	rs6697899	1.99E+08	2.83E-02	CAMSAP1L1	5	rs341908	78000473	2.58E-01	N/A	-	5.03E-07	N/A	N/A	6905	
6	rs9374070	1.1E+08	6.74E-01	N/A	12	rs7970177	13630255	9.77E-01	GRIN2B	-	5.03E-07	2.31E-01	4.20E-06	6906	
13	rs1171080	35385286	5.95E-01	DCLK1	13	rs1051518	1E+08	4.04E-01	TMTC4	-	5.03E-07	7.78E-01	4.21E-05	6907	
4	rs2124236	61388951	7.72E-01	N/A	9	rs2482705	93222323	3.01E-01	NFIL3	-	5.03E-07	8.72E-01	2.55E-05	6908	
2	rs1378729	1.75E+08	9.00E-01	CHN1	5	rs4077503	83249374	5.14E-01	N/A	-	5.03E-07	4.80E-01	3.54E-06	6909	
2	rs1378729	1.75E+08	9.00E-01	CHN1	5	rs10805814	83238685	5.14E-01	N/A	-	5.03E-07	5.29E-01	4.18E-06	6910	
2	rs1378729	1.75E+08	9.00E-01	CHN1	5	rs13163997	83236774	5.14E-01	N/A	-	5.03E-07	6.16E-01	5.87E-06	6911	
10	rs1679425	4482584	3.11E-01	N/A	16	rs3852716	81170828	4.46E-01	N/A	-	5.03E-07	3.97E-01	2.11E-06	6912	
1	rs695117	1.86E+08	6.04E-01	N/A	11	rs2574824	1.13E+08	5.06E-01	NCAM1	-	5.03E-07	6.59E-01	1.77E-04	6913	
1	rs695117	1.86E+08	6.04E-01	N/A	11	rs965560	1.13E+08	5.06E-01	NCAM1	-	5.03E-07	6.59E-01	1.77E-04	6914	
3	rs9871884	32104083	6.05E-01	GPD1L	8	rs6981972	2760863	7.24E-01	CSMD1	-	5.03E-07	7.78E-01	1.45E-04	6915	
3	rs7645818	36505422	4.88E-01	STAC	10	rs1118359	14324700	4.75E-01	FRMD4A	-	5.03E-07	7.61E-01	4.17E-04	6916	
4	rs549815	63958061	9.12E-01	N/A	13	rs4772447	1.02E+08	8.51E-01	FGF14	-	5.04E-07	2.87E-01	8.82E-04	6917	
9	rs10818322	1.21E+08	7.03E-01	N/A	18	rs627202	2282491	9.49E-01	N/A	-	5.04E-07	4.42E-01	1.25E-03	6918	
1	rs11203368	17539095	1.03E-02	PADI4	13	rs568474	71822612	7.95E-01	N/A	-	5.04E-07	6.43E-01	2.62E-04	6919	
9	rs669223	83609578	3.50E-01	N/A	14	rs10467770	20969471	8.71E-01	N/A	-	5.04E-07	7.21E-01	3.79E-04	6920	
16	rs7500465	8598095	9.65E-01	N/A	17	rs4789659	69909931	4.36E-02	N/A	-	5.04E-07	4.91E-01	3.23E-04	6921	
3	rs2062059	1E+08	9.02E-01	N/A	11	rs7935882	11384153	5.43E-01	GALNTL4	-	5.04E-07	6.14E-01	1.08E-05	6922	
6	rs966984	67509371	8.98E-01	N/A	10	rs11204199	47959596	5.01E-01	ZNF488	-	5.04E-07	1.11E-01	3.16E-03	6923	
4	rs2540841	12456173	1.83E-01	N/A	10	rs2503872	43113631	8.14E-01	N/A	-	5.04E-07	1.69E-01	1.01E-03	6924	
6	rs575649	1.53E+08	9.42E-01	N/A	15	rs4965242	97818746	8.85E-01	N/A	-	5.04E-07	1.94E-01	1.97E-06	6925	
5	rs984288	1.03E+08	3.19E-02	N/A	17	rs16966855	27088333	7.47E-01	N/A	-	5.04E-07	2.21E-01	3.93E-03	6926	
6	rs539107	1.53E+08	9.42E-01	N/A	15	rs4965242	97818746	8.85E-01	N/A	-	5.04E-07	2.38E-01	2.63E-06	6927	
4	rs448469	1.39E+08	6.97E-01	N/A	7	rs7789801	52063148	9.49E-01	N/A	-	5.04E-07	4.97E-02	2.87E-07	6928	
2	rs2357887	11082742	6.91E-01	N/A	15	rs2030619	59078972	7.69E-01	RORA	-	5.04E-07	5.96E-02	1.37E-03	6929	
6	rs7356965	1.04E+08	3.46E-01	N/A	13	rs4573787	28091434	1.39E-01	N/A	-	5.04E-07	1.32E-01	4.03E-03	6930	
5	rs7710223	1.09E+08	7.08E-01	N/A	12	rs7315359	57234394	1.90E-01	N/A	-	5.04E-07	5.22E-01	4.54E-04	6931	
11	rs2403441	19479964	3.13E-01	N/AV2	18	rs1031287	18600005	1.85E-01	N/A	-	5.04E-07	7.00E-01	1.78E-04	6932	
11	rs2403441	19479964	3.13E-01	N/AV2	18	rs1072782	18611634	1.85E-01	N/A	-	5.04E-07	7.00E-01	1.78E-04	6933	
9	rs1842636	32100777	3.20E-01	N/A	21	rs4817449	32359302	6.17E-01	NCRN/A00159	-	5.04E-07	9.65E-02	2.07E-03	6934	
9	rs7854371	32101669	3.20E-01	N/A	21	rs4817449	32359302	6.17E-01	NCRN/A00159	-	5.04E-07	9.65E-02	2.07E-03	6935	
1	rs623400	1.12E+08	7.42E-01	DENND2D	3	rs2961186	1.06E+08	9.24E-01	N/A	-	5.04E-07	9.21E-01	7.89E-05	6936	
1	rs623400	1.12E+08	7.42E-01	DENND2D	3	rs6437526	1.06E+08	9.24E-01	N/A	-	5.04E-07	9.32E-01	7.63E-05	6937	
1	rs623400	1.12E+08	7.42E-01	DENND2D	3	rs7648727	1.06E+08	9.24E-01	N/A	-	5.04E-07	9.32E-01	7.63E-05	6938	
6	rs9385976	1.01E+08	7.22E-01	MCHR2	11	rs10791957	67624124	6.97E-02	CHKA	-	5.04E-07	5.91E-01	4.70E-05	6939	
12	rs10876227	50689377	8.08E-02	ACVR1B	17	rs10491120	32062557	3.94E-01	N/A	-	5.04E-07	3.11E-01	4.42E-06	6940	
5	rs961606	18720287	5.03E-01	N/A	12	rs955801	71678350	8.64E-01	N/A	-	5.04E-07	5.15E-01	4.14E-04	6941	
6	rs1884043	47239133	2.12E-01	N/A	11	rs7113061	40483594	5.08E-01	N/A	-	5.05E-07	1.90E-01	2.89E-03	6942	

SNP A					SNP B						Interaction P				
CHR	SNP	Location	gle locus P va	Gene	CHR	SNP	Location	Single locus P valu	Gene	MHC region	Stage 1	Stage 2	Combined	Ranking	Cluster in top 100 interactions
12	rs11535963	18245787	9.69E-01	N/A	18	rs2717128	73116620	4.92E-01	GALR1	-	5.05E-07	4.52E-01	6.72E-04	6943	
4	rs2298999	1.11E+08	9.63E-01	N/A	7	rs3778878	38499182	6.37E-01	AMPH	-	5.05E-07	8.03E-01	2.68E-04	6944	
6	rs9474397	53107883	8.77E-02	GCM1	3	rs9841414	1.65E+08	7.88E-01	N/A	-	5.05E-07	6.68E-01	1.42E-04	6945	
1	rs617322	1.8E+08	2.29E-02	N/A	5	rs17105785	1.46E+08	4.03E-01	PPP2R2B	-	5.05E-07	7.44E-01	3.14E-04	6946	
2	rs1400847	76925651	8.79E-01	LRRTM4	10	rs2209688	36176611	7.60E-02	N/A	-	5.05E-07	2.97E-01	1.98E-05	6947	
10	rs1250590	80653792	4.11E-01	ZMIZ1	11	rs2514897	1.27E+08	3.08E-01	N/A	-	5.05E-07	5.91E-01	5.48E-04	6948	
6	rs911193	1.55E+08	9.23E-01	N/A	13	rs2391191	1.05E+08	2.25E-01	DAOA	-	5.05E-07	8.06E-01	1.53E-05	6949	
11	rs1676887	74696944	6.51E-01	ARRB1	15	rs549811	32793614	3.37E-01	N/A	-	5.05E-07	2.58E-01	4.94E-06	6950	
3	rs6443578	1.8E+08	1.00E-01	KCNMB2	5	rs13362504	56014886	9.88E-01	N/A	-	5.05E-07	4.48E-01	3.29E-05	6951	
1	rs12726043	1.98E+08	5.83E-01	N/A	8	rs2010560	21118841	2.86E-01	N/A	-	5.05E-07	2.22E-01	7.49E-07	6952	
7	rs2188324	88162442	3.41E-01	N/A	13	rs609045	29644051	5.64E-01	N/A	-	5.06E-07	1.68E-01	8.76E-07	6953	
3	rs4413325	83960374	7.39E-01	N/A	8	rs12682123	2777203	1.69E-01	CSMD1	-	5.06E-07	8.01E-01	8.86E-05	6954	
2	rs6736495	1.05E+08	3.94E-01	N/A	3	rs6806453	1.33E+08	6.74E-01	CPNE4	-	5.06E-07	6.28E-01	6.66E-06	6955	
6	rs602414	1.38E+08	4.34E-01	N/A	3	rs1579905	74711280	1.34E-01	N/A	-	5.06E-07	9.08E-01	7.62E-05	6956	
9	rs1415221	72670538	1.92E-01	TRPM3	16	rs8047876	80178923	5.88E-01	CMIP	-	5.06E-07	8.55E-01	1.05E-04	6957	
1	rs4144543	1.18E+08	4.46E-01	SPAG17	2	rs7421653	2.34E+08	8.14E-01	INPP5D	-	5.06E-07	6.40E-01	1.28E-04	6958	
8	rs1533308	22725338	5.39E-01	PEBP4	8	rs16909722	1.4E+08	2.89E-01	COL22A1	-	5.06E-07	5.22E-01	6.12E-04	6959	
5	rs6885306	3790730	9.94E-01	N/A	13	rs12427758	33949169	2.14E-01	N/A	-	5.06E-07	7.37E-01	8.74E-05	6960	
9	rs10961183	13625017	2.87E-01	N/A	9	rs4877361	89330745	5.32E-01	DAPK1	-	5.06E-07	6.49E-01	6.55E-05	6961	
7	rs11761833	1.52E+08	6.31E-01	N/A	19	rs17570	38570677	8.07E-02	PEPD	-	5.06E-07	6.25E-01	6.74E-05	6962	
2	rs13417603	16679487	3.64E-01	FAM49A	11	rs891437	1.21E+08	2.96E-01	N/A	-	5.06E-07	7.08E-01	2.53E-05	6963	
1	rs778411	56478862	5.35E-01	N/A	5	rs2052527	1.66E+08	7.26E-01	N/A	-	5.06E-07	9.55E-01	2.91E-04	6964	
6	rs9372252	1.11E+08	2.32E-01	DDO	13	rs4771309	98073315	8.62E-01	N/A	-	5.06E-07	1.85E-01	5.65E-06	6965	
6	rs332496	93512596	3.60E-01	N/A	8	rs17488918	16957026	4.51E-01	EFHA2	-	5.06E-07	4.68E-01	4.72E-04	6966	
2	rs934748	1.13E+08	2.21E-01	CHCHD5	8	rs3758063	87754664	2.21E-01	CNGB3	-	5.06E-07	1.34E-01	1.15E-06	6967	
10	rs11200896	85910065	5.67E-01	GHITM	16	rs731710	86442048	3.16E-01	SLC7A5	-	5.06E-07	2.81E-01	5.01E-04	6968	
2	rs13409846	2.19E+08	2.63E-01	PNKD	4	rs3762864	1.4E+08	4.37E-01	ELF2	-	5.06E-07	3.16E-01	1.66E-03	6969	
8	rs899920	1.36E+08	8.74E-01	N/A	13	rs1340745	68414215	1.43E-01	N/A	-	5.07E-07	6.21E-02	1.26E-06	6970	
3	rs4388016	99441797	6.29E-01	N/A	16	rs6500343	49448844	5.45E-01	N/A	-	5.07E-07	1.17E-01	3.42E-03	6971	
2	rs6748488	30805423	5.14E-01	CAPN13	16	rs1038106	7105351	2.71E-01	A2BP1	-	5.07E-07	1.56E-01	1.36E-06	6972	
5	rs17707884	1.48E+08	6.59E-02	N/A	16	rs8062393	76975312	7.54E-01	WWOX	-	5.07E-07	2.47E-01	5.56E-06	6973	
7	rs864249	28235781	1.94E-01	N/A	16	rs4368165	20098136	4.35E-01	N/A	-	5.07E-07	6.46E-02	1.29E-07	6974	
6	rs12208688	3728762	7.93E-01	N/A	10	rs10905843	10926420	9.03E-01	N/A	-	5.07E-07	3.54E-01	8.66E-04	6975	
7	rs2190930	1.24E+08	6.60E-01	N/A	10	rs805722	1.06E+08	9.63E-01	COL17A1	-	5.07E-07	6.86E-01	1.71E-04	6976	
6	rs12205120	1.55E+08	4.76E-01	IPCEF1	10	rs10904177	4102318	9.30E-01	N/A	-	5.07E-07	8.45E-01	1.90E-04	6977	
2	rs698809	44556482	9.07E-01	C2orf34	3	rs9874152	1.96E+08	8.70E-01	ATP13A3	-	5.07E-07	6.31E-01	6.97E-05	6978	
2	rs10171249	54346912	9.82E-01	ACYP2	2	rs13416800	1.43E+08	2.64E-01	N/A	-	5.07E-07	1.49E-01	1.07E-03	6979	
2	rs10171249	54346912	9.82E-01	ACYP2	2	rs4429409	1.43E+08	2.64E-01	N/A	-	5.07E-07	1.65E-01	9.62E-04	6980	
2	rs341605	44917691	3.10E-02	N/A	15	rs3809482	41449094	5.41E-02	ADAL	-	5.07E-07	5.55E-01	7.62E-05	6981	
2	rs341605	44917691	3.10E-02	N/A	15	rs6493084	41434919	5.41E-02	ADAL	-	5.07E-07	5.55E-01	7.62E-05	6982	
2	rs341605	44917691	3.10E-02	N/A	15	rs9920879	41427062	5.41E-02	ADAL	-	5.07E-07	5.55E-01	7.62E-05	6983	
2	rs12104503	1.88E+08	6.47E-01	CALCRL	21	rs958924	26498774	5.76E-02	N/A	-	5.07E-07	6.28E-01	5.39E-04	6984	
4	rs10005964	77689324	7.97E-01	SHROOM3	17	rs930525	10635980	2.67E-01	N/A	-	5.07E-07	9.85E-01	3.41E-05	6985	
5	rs12697130	53943184	6.98E-01	N/A	10	rs7101190	71258355	2.31E-01	COL13A1	-	5.07E-07	2.30E-01	2.04E-06	6986	
2	rs1851779	1.98E+08	9.34E-01	BOLL	7	rs12699758	15930763	7.54E-01	N/A	-	5.07E-07	4.94E-01	8.19E-05	6987	
2	rs2293255	1.98E+08	9.34E-01	BOLL	7	rs12699758	15930763	7.54E-01	N/A	-	5.07E-07	4.94E-01	8.19E-05	6988	
5	rs990859	39993218	6.20E-01	N/A	21	rs2210268	40685128	9.43E-01	DSCAM	-	5.07E-07	2.24E-01	6.85E-06	6989	
5	rs1465687	1.55E+08	3.87E-01	N/A	11	rs504847	94043963	1.01E-01	N/A	-	5.07E-07	7.63E-01	4.75E-05	6990	
1	rs17261915	26629443	3.49E-01	LIN28	2	rs12612120	1.74E+08	5.89E-01	N/A	-	5.07E-07	9.31E-01	2.00E-04	6991	
6	rs6909426	1.57E+08	6.39E-01	ARID1B	2	rs17784678	52223385	2.20E-01	N/A	-	5.07E-07	3.48E-02	1.21E-02	6992	
6	rs6917637	1.57E+08	6.39E-01	ARID1B	2	rs17784678	52223385	2.20E-01	N/A	-	5.07E-07	4.10E-02	1.09E-02	6993	
4	rs9993773	80653628	5.97E-02	N/A	7	rs16480	24277594	2.38E-01	NPY	-	5.07E-07	1.95E-01	6.03E-06	6994	
7	rs17172499	45217818	8.10E-01	N/A	15	rs920686	63298482	2.89E-02	CILP	-	5.07E-07	2.41E-01	1.31E-03	6995	
5	rs1506174	40169821	1.82E-01	N/A	17	rs2289587	7948375	3.44E-01	N/A	-	5.07E-07	3.97E-01	5.35E-04	6996	
9	rs12380341	1.07E+08	8.87E-01	N/A	14	rs2250789	1.01E+08	9.50E-01	DYNC1H1	-	5.07E-07	6.58E-01	3.23E-05	6997	
3	rs2875057	5689429	1.03E-01	N/A	14	rs698329	47602215	4.37E-01	N/A	-	5.08E-07	2.93E-01	6.58E-06	6998	
7	rs4728435	1.38E+08	7.90E-02	N/A	11	rs11215584	1.15E+08	3.31E-01	N/A	-	5.08E-07	4.66E-01	7.14E-06	6999	
12	rs3748283	99491044	3.41E-01	NR1H4	18	rs1612474	26923837	2.86E-01	DSC2	-	5.08E-07	3.37E-01	3.06E-04	7000	
1	rs10753195	1.7E+08	4.64E-02	DNM3	8	rs2945745	1.35E+08	2.74E-01	N/A	-	5.08E-07	6.33E-01	1.04E-03	7001	
1	rs10753196	1.7E+08	4.64E-02	DNM3	8	rs2945745	1.35E+08	2.74E-01	N/A	-	5.08E-07	6.33E-01	1.04E-03	7002	
6	rs2432808	5316348	8.97E-01	FARS2	4	rs12647248	1.23E+08	8.83E-01	N/A	-	5.08E-07	6.83E-01	2.13E-05	7003	
11	rs16910400	12020875	3.50E-01	N/A	16	rs12447346	74532394	5.14E-01	N/A	-	5.08E-07	1.59E-01	1.80E-06	7004	
4	rs7656270	82782793	5.13E-02	N/A	11	rs4586138	11955605	4.04E-02	USP47	-	5.08E-07	N/A	N/A	7005	
2	rs6716984	47716579	4.31E-02	N/A	12	rs16919514	32277681	6.16E-01	BICD1	-	5.08E-07	1.19E-01	1.41E-06	7006	
8	rs7011466	25875922	7.61E-01	EBF2	19	rs295367	23233776	7.97E-01	N/A	-	5.08E-07	8.40E-01	2.69E-04	7007	
3	rs4389439	29404741	8.89E-01	N/A	5	rs3896348	18204313	2.02E-01	N/A	-	5.08E-07	N/A	N/A	7008	
4	rs7688965	1.82E+08	5.43E-01	N/A	21	rs4816892	23373171	1.93E-01	N/A	-	5.08E-07	6.23E-02	1.69E-03	7009	
6	rs2243384	1.18E+08	3.68E-02	ROS1	9	rs10514815	94296516	9.41E-01	ECM2	-	5.08E-07	9.15E-01	3.10E-04	7010	
6	rs2243384	1.18E+08	3.68E-02	ROS1	9	rs7869742	94295995	9.41E-01	ECM2	-	5.08E-07	9.55E-01	2.09E-04	7011	
1	rs12035088	53797428	2.21E-01	GLIS1	4	rs11722340	22889073	3.68E-02	N/A	-	5.08E-07	7.97E-02	3.92E-07	7012	
7	rs6973848	1.27E+08	9.55E-02	N/A	8	rs2575735	97603827	1.89E-01	SDC2	-	5.08E-07	5.24E-01	2.86E-05	7013	
5	rs4866470	3795035	6.89E-01	N/A	13	rs12427758	33949169	2.14E-01	N/A	-	5.08E-07	7.21E-01	8.21E-05	7014	
1	rs4603156	1.08E+08	3.33E-02	NTNG1	3	rs6548570	78599853	5.78E-01	N/A	-	5.09E-07	6.16E-01	4.10E-05	7015	
12	rs10778881	81174214	1.47E-01	N/A	20	rs2904362	51580068	1.25E-01	N/A	-	5.09E-07	6.88E-01	1.73E-05	7016	
12	rs7132518	81168474	1.47E-01	N/A	20	rs2904362	51580068	1.25E-01	N/A	-	5.09E-07	6.88E-01	1.73E-05	7017	
12	rs7135409	81168293	1.47E-01	N/A	20	rs2904362	51580068	1.25E-01	N/A	-	5.09E-07	6.88E-01	1.73E-05	7018	
6	rs204247	13830502	5.75E-01	RANBP9	7	rs12536153	1.09E+08	1.13E-01	N/A	-	5.09E-07	8.29E-01	5.95E-04	7019	
1	rs12116812	84513524	7.83E-01	N/A	11	rs669244	1.22E+08	4.14E-01	N/A	-	5.09E-07	9.35E-01	5.62E-05	7020	
4	rs3114381	40228054	3.52E-01	RBM47	14	rs6572313	21751816	7.24E-02	N/A	-	5.09E-07	8.72E-01	2.43E-04	7021	
10	rs2394840	73243371	6.85E-01	CDH23	15	rs524908	41975283	4.02E-01	FRMD5	-	5.09E-07	1.86E-01	1.35E-06	7022	
3	rs1385331	1.98E+08	5.07E-01	LRRC33	15	rs8025419	56376746	2.92E-01	N/A	-	5.09E-07	6.73E-01	2.64E-05	7023	
1	rs12035356	1.53E+08	8.11E-01	KCNN3	13	rs11839072	1.08E+08	5.31E-01	MYO16	-	5.09E-07	8.85E-01	8.23E-05	7024	
6	rs9346979	1.64E+08	5.80E-01	N/A	17	rs3179840	44722535	3.71E-01	ZNF652	-	5.09E-07	1.64E-01	8.83E-07	7025	
6	rs6908296	1.5E+08	3.97E-01	N/A	9	rs1668978	25342327	1.10E-01	N/A	-	5.09E-07	5.24E-01	5.85E-05	7026	
5	rs17391168	77962789	1.45E-01	LHFPL2	10	rs7094555	1.29E+08	7.56E-01	DOCK1	-	5.09E-07	8.71E-01	1.05E-04	7027	
1	rs623400	1.12E+08	7.42E-01	DENND2D	3	rs3996212	1.06E+08	9.42E-01	N/A	-	5.09E-07	9.21E-01	7.95E-05	7028	
1	rs704874	32889210	9.30E-01	ZBTB8OS	7	rs4285401	1.53E+08	1.97E-02	N/A	-	5.10E-07	9.94E-01	7.26E-05	7029	
4	rs2540844	12461217	2.05E-01	N/A	10	rs2503872	43113631	8.14E-01	N/A	-	5.10E-07	2.18E-01	7.70E-04	7030	
11	rs12290503	98866701	4.24E-01	CNTN5	13	rs7319745	19623636	2.88E-01	GJA3	-	5.10E-07	3.01E-01	3.15E-04	7031	
1	rs10799300	2.24E+08	3.75E-01	DN/AH14	22	rs5994572	31188428	8.06E-02	BPIL2	-	5.10E-07	9.79E-01	4.81E-05	7032	
4	rs4862612	1.87E+08	6.27E-04	N/A	11	rs11233078	81706676	6.44E-02	N/A	-	5.10E-07	8.68E-02	1.79E-03	7033	
6	rs674026	1.51E+08	7.55E-02	IYD	10	rs4636589	1.09E+08	8.36E-01	N/A	-	5.10E-07	1.21E-01	5.62E-07	7034	
6	rs831496	37100215	4.36E-01	FGD2	17	rs1465983	72454404	6.73E-01	MGAT5B	-	5.10E-07	3.44E-01	6.34E-06	7035	
8	rs1563389	1.21E+08	8.44E-01	COL14A1	22	rs16985531	26491703	8.13E-01	MN1	-	5.10E-07	2.14E-01	7.97E-04	7036	
8	rs7004099	1.21E+08	8.44E-01	COL14A1	22	rs16985531	26491703	8.13E-01	MN1	-	5.10E-07	2.14E-01	7.97E-04	7037	
9	rs10967704	27078014	3.02E-01	N/A	17	rs3760275	50855658	2.36E-01	MMD	-	5.11E-07	6.33E-01	2.39E-04	7038	
2	rs13017014	1.68E+08	5.49E-01	XIRP2	19	rs889362	18619255	5.85E-02	KLHL26	-	5.11E-07	6.16E-02	2.17E-03	7039	
2	rs6710739	73132498	8.40E-01	SFXN5	16	rs4781693	15793744	9.69E-01	MYH11	-	5.11E-07	4.61E-01	5.20E-06	7040	
13	rs9316498	49958956	4.96E-01	N/A	13	rs1278114	1.12E+08	9.60E-01	TUBGCP3	-	5.11E-07	6.59E-01	4.81E-05	7041	
3	rs6763266	1.21E+08	3.41E-01	C3orf15	19	rs370229	11145483	6.57E-01	SPC24	-	5.11E-07	9.00E-01	6.56E-05	7042	
6	rs1006012	53966408	8.93E-01	N/A	1	rs3795811	2.27E+08	5.59E-01	OBSCN	-	5.11E-07	9.55E-01	1.64E-04	7043	
4	rs17290361	59064272	5.01E-02	N/A	17	rs11077813	71848140	3.71E-01	PRPSAP1	-	5.11E-07	1.55E-01	1.65E-03	7044	
8	rs1635668	5591685	3.93E-01	N/A	13	rs2226042	59188076	8.65E-01	DIAPH3	-	5.11E-07	9.08E-01	9.70E-05	7045	
5	rs10041997	1.2E+08	5.18E-01	N/A	20	rs580544	47638833	9.25E-01	N/A	-	5.11E-07	9.94E-01	8.62E-05	7046	
2	rs6431850	7165870	3.51E-01	N/A	5	rs1582924	28365424	6.36E-01	N/A	-	5.11E-07	6.44E-02	2.01E-03	7047	
6	rs2758937	1.65E+08	1.16E-01	N/A	4	rs7694043	96157791	5.95E-01	BMPR1B	-	5.11E-07	2.08E-01	9.23E-04	7048	
2	rs10496132	66251051	2.35E-02	N/A	12	rs1503583	59289463	6.62E-01	N/A	-	5.11E-07	5.21E-01	3.29E-04	7049	
2	rs13383903	66254338	2.35E-02	N/A	12	rs1503583	59289463	6.62E-01	N/A	-	5.11E-07	5.21E-01	3.29E-04	7050	
9	rs7859384	81312105	2.09E-01	N/A	19	rs2285515	40352290	6.76E-01	FXYD5	-	5.11E-07	6.28E-01	1.25E-04	7051	
1	rs1772849	2E+08	1.30E-01	PKP1	11	rs907203	7925138	2.42E-01	NLRP10	-	5.12E-07	2.71E-02	1.42E-02	7052	
1	rs2595961	2.03E+08	2.10E-01	NFASC	15	rs11630255	21800234	2.97E-01	N/A	-	5.12E-07	8.95E-01	1.64E-04	7053	
7	rs11763980	4134517	5.56E-01	SDK1	13	rs9545555	80274474	1.04E-01	N/A	-	5.12E-07	2.72E-01	1.22E-05	7054	

SNP A					SNP B						Interaction P			Ranking	Cluster in top 100 interactions
CHR	SNP	Location	gle locus P va	Gene	CHR	SNP	Location	Single locus P valu	Gene	MHC region	Stage 1	Stage 2	Combined		
15	rs11071185	53482122	5.92E-01	CCPG1	20	rs6078410	11894861	2.75E-01	N/A	-	5.12E-07	3.68E-01	3.50E-03	7055	
2	rs17862647	51518397	5.33E-01	N/A	11	rs7122101	41185350	4.39E-01	N/A	-	5.12E-07	6.97E-01	3.51E-05	7056	
1	rs17102086	46495526	7.73E-01	RAD54L	13	rs7984814	70400632	6.37E-02	N/A	-	5.12E-07	9.82E-01	4.61E-05	7057	
6	rs2076270	1.39E+08	8.90E-01	KIAA1244	13	rs2481952	27434901	2.34E-01	CDX2	-	5.12E-07	7.20E-02	4.94E-03	7058	
2	rs4408769	42949290	2.28E-01	N/A	7	rs4236317	3101119	8.02E-01	N/A	-	5.12E-07	N/A	N/A	7059	
1	rs12725738	2.41E+08	5.35E-01	N/A	8	s10098621	21322806	6.93E-01	N/A	-	5.12E-07	7.02E-01	1.73E-05	7060	
1	rs12725738	2.41E+08	5.35E-01	N/A	8	rs9325883	21322213	6.93E-01	N/A	-	5.12E-07	7.02E-01	1.73E-05	7061	
8	rs12547275	1.08E+08	9.41E-01	OXR1	10	rs730080	1.19E+08	6.50E-01	N/A	-	5.12E-07	7.22E-01	2.31E-05	7062	
2	rs736085	53688587	7.79E-01	N/A	16	rs9928718	73634239	5.52E-01	ZNRF1	-	5.12E-07	9.00E-01	1.68E-04	7063	
9	rs3935562	1.2E+08	4.07E-01	N/A	11	rs481597	93709664	9.58E-02	N/A	-	5.12E-07	2.46E-01	4.28E-06	7064	
14	rs2021986	47220619	3.71E-01	MDGA2	16	rs734710	82831935	7.58E-01	KCNG4	-	5.12E-07	6.02E-01	3.69E-04	7065	
1	rs11203423	17780092	4.50E-01	ARHGEF10L	7	rs2428161	1.04E+08	9.74E-01	N/A	-	5.12E-07	4.11E-02	3.27E-03	7066	
6	rs13206042	5667397	3.40E-01	FARS2	12	s10842067	22843998	1.80E-01	N/A	-	5.12E-07	4.84E-01	9.15E-05	7067	
10	rs10466303	10810026	2.28E-01	N/A	21	rs2837756	40917744	2.49E-01	DSCAM	-	5.12E-07	9.83E-01	3.20E-04	7068	
9	rs10124903	25649613	6.42E-01	TUSC1	14	rs741275	88934721	8.84E-01	FOXN3	-	5.12E-07	N/A	N/A	7069	
5	rs4916688	90058536	9.62E-01	GPR98	17	s10491216	3267156	7.29E-01	OR1E1	-	5.12E-07	7.63E-01	2.22E-06	7070	
3	rs7372263	1.3E+08	1.59E-01	RAB7A	14	rs3817625	21275287	7.83E-01	N/A	-	5.12E-07	8.27E-01	3.18E-04	7071	
1	rs12727575	33824892	3.93E-01	CSMD2	10	s12569498	1.26E+08	5.95E-01	LHPP	-	5.13E-07	1.93E-01	1.16E-06	7072	
2	rs12995304	1.39E+08	9.98E-02	N/A	8	rs7816586	27425251	8.02E-01	EPHX2	-	5.13E-07	4.16E-01	9.45E-06	7073	
20	rs1475348	14469824	5.12E-01	MACROD2	21	rs4920107	42755622	7.96E-01	RSPH1	-	5.13E-07	7.04E-01	1.49E-04	7074	
7	rs17152356	25718996	1.49E-01	N/A	21	rs9978523	33511140	1.84E-01	IFN/AR2	-	5.13E-07	1.12E-01	5.65E-07	7075	
1	rs6429280	2.4E+08	5.83E-01	KMO	15	rs3784651	92726277	7.71E-01	MCTP2	-	5.13E-07	3.56E-01	1.59E-05	7076	
9	rs1931093	87022289	6.79E-01	N/A	13	rs7984163	79056401	5.41E-01	N/A	-	5.13E-07	8.30E-01	7.58E-05	7077	
6	rs2858331	32789255	1.42E-01	N/A	10	rs4245009	52247821	7.47E-01	A1CF	MHC	5.13E-07	2.37E-01	8.93E-04	7078	
2	rs6729271	2.38E+08	3.09E-01	RAMP1	11	rs4258388	1.23E+08	1.39E-01	GRAMD1B	-	5.13E-07	4.18E-01	5.58E-04	7079	
2	rs1906901	66250961	2.76E-02	N/A	12	rs1503583	59289463	6.62E-01	N/A	-	5.13E-07	5.25E-01	3.20E-04	7080	
3	rs2246861	1E+08	4.21E-01	N/A	11	rs7935882	11384153	5.43E-01	GALNTL4	-	5.13E-07	7.17E-01	9.73E-06	7081	
2	rs10210465	2.31E+08	2.50E-01	SP110	11	s11225370	1.02E+08	2.54E-01	N/A	-	5.13E-07	9.80E-01	2.68E-04	7082	
2	rs6434930	1.98E+08	5.02E-01	HSPD1	10	s10736312	1.24E+08	3.97E-01	BTBD16	-	5.13E-07	2.30E-01	5.79E-06	7083	
2	rs6434930	1.98E+08	5.02E-01	HSPD1	10	rs2421013	1.24E+08	3.97E-01	BTBD16	-	5.13E-07	2.30E-01	5.79E-06	7084	
2	rs3792159	1.98E+08	5.40E-01	MOBKL3	10	s10736312	1.24E+08	3.97E-01	BTBD16	-	5.13E-07	2.30E-01	4.61E-06	7085	
2	rs3792159	1.98E+08	5.40E-01	MOBKL3	10	rs2421013	1.24E+08	3.97E-01	BTBD16	-	5.13E-07	2.30E-01	4.61E-06	7086	
10	rs10509579	91485752	7.64E-01	KIF20B	12	rs7958724	1.25E+08	1.05E-01	N/A	-	5.13E-07	2.88E-01	1.72E-04	7087	
10	rs1886997	91488234	7.64E-01	KIF20B	12	rs7958724	1.25E+08	1.05E-01	N/A	-	5.13E-07	2.88E-01	1.72E-04	7088	
4	rs7656347	1.85E+08	5.30E-01	N/A	12	rs7300564	78119084	7.81E-01	SYT1	-	5.13E-07	4.23E-01	2.70E-06	7089	
7	rs2040933	1.24E+08	6.57E-01	N/A	10	rs1747677	1.06E+08	9.81E-01	COL17A1	-	5.13E-07	5.79E-01	2.46E-04	7090	
6	rs2277090	84994249	3.66E-01	KIAA1009	13	rs9300855	1.03E+08	1.36E-01	N/A	-	5.13E-07	2.80E-02	1.03E-02	7091	
1	rs7540190	2.37E+08	1.04E-01	N/A	4	rs6842810	1.43E+08	9.88E-01	N/A	-	5.13E-07	3.34E-01	2.39E-04	7092	
1	rs2075630	86725189	6.58E-01	CLCA1	4	rs1516691	1.54E+08	8.51E-01	N/A	-	5.13E-07	4.73E-01	8.23E-06	7093	
1	rs4540634	1.61E+08	8.60E-01	N/A	20	rs4814626	17509041	3.03E-01	DSTN	-	5.14E-07	1.65E-01	1.58E-06	7094	
5	rs13436137	3818085	6.43E-01	N/A	7	rs2008296	28112089	5.00E-02	JAZF1	-	5.14E-07	4.67E-01	1.62E-05	7095	
5	rs10039219	25919165	1.95E-01	N/A	12	rs4620789	81632526	3.93E-01	TMTC2	-	5.14E-07	8.93E-01	6.46E-05	7096	
2	rs1510548	2.15E+08	2.34E-01	SPAG16	13	rs9516948	96928731	8.96E-01	RAP2A	-	5.14E-07	7.90E-01	1.63E-04	7097	
2	rs2732827	1.03E+08	6.78E-01	MFSD9	8	s10100405	88960987	6.43E-01	DCAF4L2	-	5.14E-07	9.39E-01	1.42E-04	7098	
9	rs1332179	21410677	2.83E-01	IFN/A8	19	rs7254781	11680193	2.06E-02	ZNF823	-	5.14E-07	9.47E-01	7.32E-05	7099	
6	rs2469	43407301	4.71E-01	ZNF318	13	rs1981060	38650044	5.89E-01	N/A	-	5.14E-07	1.96E-01	1.62E-03	7100	
3	rs11706042	1.21E+08	6.10E-01	CDGAP	18	rs7240925	20484577	1.06E-01	N/A	-	5.14E-07	3.10E-01	9.35E-06	7101	
7	rs10226243	18934528	4.40E-02	HDAC9	10	s12769200	70887790	8.42E-01	TSPAN15	-	5.14E-07	1.09E-02	9.63E-08	7102	
3	rs875581	1.58E+08	4.49E-01	KCN/AB1	12	rs2141099	1.3E+08	1.92E-01	N/A	-	5.14E-07	7.73E-01	1.60E-04	7103	
6	rs2508015	31118179	8.45E-01	HCG22	6	rs2233984	31187243	4.20E-02	C6orf15	MHC	5.14E-07	7.91E-01	6.67E-05	7104	
11	rs3758832	87712046	9.34E-01	CTSC	14	s12434120	56493360	7.66E-01	N/A	-	5.14E-07	6.15E-02	7.65E-03	7105	
1	rs1572416	1.55E+08	7.60E-01	ARHGEF11	4	rs734526	7648352	7.83E-01	SORCS2	-	5.14E-07	6.36E-01	1.90E-05	7106	
13	rs2057609	78038844	1.93E-01	N/A	19	rs9636161	61592298	4.39E-01	ZNF542	-	5.14E-07	1.10E-01	4.38E-03	7107	
6	rs7745656	32788948	1.70E-01	N/A	10	s10994392	52200825	6.17E-01	ASAH2B	MHC	5.14E-07	2.40E-01	1.41E-03	7108	
5	rs2707772	66749767	9.98E-01	N/A	10	rs359288	17370186	5.24E-02	N/A	-	5.14E-07	7.94E-01	6.84E-05	7109	
5	rs712572	1.16E+08	5.19E-01	COMMD10	10	rs560083	84604824	4.67E-01	NRG3	-	5.14E-07	7.13E-03	2.00E-08	7110	
2	rs2593713	1.44E+08	7.82E-01	N/A	3	s10933952	1.1E+08	5.35E-02	DZIP3	-	5.14E-07	1.16E-01	2.00E-06	7111	
9	rs10814410	36587	2.05E-01	FAM138C	9	rs7863447	14145418	5.81E-01	N/A	-	5.14E-07	5.57E-01	2.25E-05	7112	
4	rs2320566	17841558	5.53E-01	N/A	10	s11018214	1.29E+08	4.79E-01	N/A	-	5.15E-07	1.49E-01	1.53E-03	7113	
4	rs10007789	1.66E+08	4.50E-01	TRIM61	13	rs1374010	26193317	2.59E-01	N/A	-	5.15E-07	3.77E-01	2.40E-03	7114	
9	rs2998711	80313512	6.58E-01	N/A	15	rs999787	53079743	8.58E-01	N/A	-	5.15E-07	7.81E-01	5.86E-06	7115	
9	rs3010713	80310810	6.58E-01	N/A	15	rs999787	53079743	8.58E-01	N/A	-	5.15E-07	8.47E-01	7.45E-06	7116	
11	rs10742412	4882769	2.18E-01	OR51A7	22	rs763217	43496258	6.23E-01	PRR5	-	5.15E-07	N/A	N/A	7117	
14	rs1169467	36416374	4.09E-01	SLC25A21	17	rs2924250	43698118	7.13E-03	SKAP1	-	5.15E-07	3.22E-01	1.70E-05	7118	
17	rs12602618	74866743	6.73E-01	HRNBP3	22	rs6519064	36177411	6.64E-02	MFNG	-	5.15E-07	3.02E-01	7.10E-04	7119	
5	rs336073	54476342	4.53E-01	CDC20B	19	rs415647	3074113	9.77E-01	GN/A15	-	5.15E-07	3.11E-01	7.26E-04	7120	
4	rs17040984	1.63E+08	9.50E-02	FSTL5	8	rs6586730	18165587	8.14E-01	N/A	-	5.15E-07	4.25E-01	7.36E-06	7121	
2	rs1378729	1.75E+08	9.00E-01	CHN1	5	rs4274957	83243219	4.41E-01	N/A	-	5.15E-07	4.80E-01	3.43E-06	7122	
2	rs2373106	37524430	2.39E-01	N/A	19	rs1293703	58834663	6.87E-01	DPRX	-	5.15E-07	7.00E-01	3.85E-05	7123	
7	rs3113288	68170082	6.14E-01	N/A	11	rs7946653	7394175	9.33E-01	SYT9	-	5.15E-07	N/A	N/A	7124	
7	rs17169351	16218692	2.46E-01	ISPD	9	rs781098	31619602	7.24E-01	N/A	-	5.15E-07	1.67E-01	4.21E-06	7125	
4	rs13148340	1.39E+08	6.30E-01	N/A	16	rs1463462	17161560	9.44E-01	XYLT1	-	5.15E-07	2.28E-01	1.26E-06	7126	
4	rs6537055	1.39E+08	6.30E-01	N/A	16	rs1463462	17161560	9.44E-01	XYLT1	-	5.15E-07	3.06E-01	2.18E-06	7127	
2	rs10932327	1.56E+08	5.59E-01	N/A	10	rs192710	70983142	4.51E-01	NEUROG3	-	5.15E-07	5.49E-01	4.03E-04	7128	
2	rs10203676	1.56E+08	5.59E-01	N/A	10	rs192710	70983142	4.51E-01	NEUROG3	-	5.15E-07	5.79E-01	3.61E-04	7129	
2	rs1482308	1.56E+08	5.59E-01	N/A	10	rs192710	70983142	4.51E-01	NEUROG3	-	5.15E-07	5.79E-01	3.61E-04	7130	
6	rs4839977	99336295	7.10E-01	N/A	2	s12476858	20117235	1.44E-01	LAPTM4A	-	5.15E-07	6.09E-02	5.96E-07	7131	
2	rs3116507	2.04E+08	8.99E-01	N/A	12	rs537938	3264359	8.36E-01	TSPAN9	-	5.15E-07	1.66E-01	1.23E-06	7132	
2	rs1177269	61140949	1.72E-01	KIAA1841	8	rs2726555	59947966	8.90E-01	TOX	-	5.15E-07	1.74E-01	8.28E-04	7133	
9	rs1004800	14552698	2.78E-02	N/A	17	rs7211216	9987832	1.30E-01	GAS7	-	5.15E-07	5.13E-01	5.26E-05	7134	
3	rs2895285	1.06E+08	7.62E-01	N/A	11	rs7932792	9660453	4.63E-01	SWAP70	-	5.16E-07	5.94E-02	1.57E-02	7135	
2	rs4672665	2.13E+08	9.70E-01	N/A	12	rs4764852	1.01E+08	5.46E-01	DRAM1	-	5.16E-07	1.74E-01	3.23E-06	7136	
2	rs6704827	41869279	6.25E-01	N/A	12	rs2388982	32025507	8.15E-01	C12orf35	-	5.16E-07	5.13E-01	2.98E-03	7137	
10	rs1925542	20986659	6.24E-01	N/A	14	s11626913	57634347	8.73E-01	C14orf37	-	5.16E-07	2.80E-02	4.34E-07	7138	
6	rs1199389	8336115	7.69E-01	N/A	17	rs236567	65754141	2.05E-01	N/A	-	5.16E-07	1.71E-01	1.33E-03	7139	
2	rs4536647	2.28E+08	5.32E-01	COL4A4	8	rs4243888	1.39E+08	7.31E-01	FAM135B	-	5.16E-07	1.65E-01	1.24E-06	7140	
3	rs9818348	54346185	1.46E-01	CACN/A2D3	20	rs6137352	2146288	5.96E-02	N/A	-	5.16E-07	9.08E-01	6.61E-05	7141	
1	rs11163047	80780548	6.98E-01	N/A	4	s16880691	28082146	6.88E-01	N/A	-	5.16E-07	5.50E-01	2.81E-04	7142	
2	rs7603916	1.46E+08	7.70E-01	N/A	14	rs6107	94127023	1.17E-01	SERPIN/A5	-	5.16E-07	2.39E-01	7.92E-06	7143	
3	rs4679232	1.26E+08	8.10E-01	HEG1	11	s10831444	88168143	6.59E-02	GRM5	-	5.16E-07	6.22E-02	1.76E-03	7144	
7	rs3779456	27181082	2.46E-02	HOXA7	14	rs4982736	22698306	1.93E-01	SLC7A8	-	5.16E-07	2.30E-01	1.09E-05	7145	
7	rs3779456	27181082	2.46E-02	HOXA7	14	rs7150157	22697830	1.93E-01	SLC7A8	-	5.16E-07	2.30E-01	1.09E-05	7146	
8	rs2511735	1.04E+08	9.11E-01	ODF1	18	rs584970	72176763	8.92E-01	N/A	-	5.16E-07	3.35E-02	1.22E-02	7147	
5	rs12351	95155007	2.90E-02	RHOBTB3	14	rs7148107	42437346	7.21E-01	N/A	-	5.16E-07	6.66E-01	2.00E-04	7148	
8	rs7014409	5344747	2.26E-01	N/A	14	rs8018930	94309119	5.71E-01	GSC	-	5.16E-07	N/A	N/A	7149	
9	rs10990533	1.05E+08	7.78E-01	N/A	21	rs9983113	39237186	5.50E-01	N/A	-	5.16E-07	5.70E-03	2.54E-02	7150	
18	rs9807656	40600954	7.79E-01	SETBP1	22	rs5762049	25992213	1.49E-02	N/A	-	5.16E-07	1.69E-01	7.45E-07	7151	
2	rs2931465	1.41E+08	7.27E-01	LRP1B	5	rs1585224	32697244	4.34E-01	N/A	-	5.16E-07	2.00E-01	2.01E-04	7152	
5	rs16899073	29554957	5.51E-01	N/A	12	s11109922	77386192	5.76E-01	N/A	-	5.16E-07	3.45E-01	3.12E-05	7153	
8	rs12543507	1.09E+08	8.32E-02	N/A	8	s10105826	1.32E+08	8.22E-01	N/A	-	5.16E-07	N/A	N/A	7154	
8	rs7837572	18709420	7.72E-01	PSD3	11	rs3133226	1.2E+08	3.77E-02	GRIK4	-	5.17E-07	3.21E-01	9.54E-04	7155	
10	rs10823136	69525369	7.41E-01	MYPN	10	rs1036270	1.33E+08	7.29E-01	N/A	-	5.17E-07	5.73E-01	9.84E-05	7156	
1	rs4233307	2.13E+08	5.65E-02	PTPN14	18	rs9960247	10269250	3.17E-01	N/A	-	5.17E-07	8.91E-01	4.27E-05	7157	
1	rs6540839	2.13E+08	5.65E-02	PTPN14	18	rs9960247	10269250	3.17E-01	N/A	-	5.17E-07	8.91E-01	4.27E-05	7158	
3	rs13077612	1.35E+08	5.91E-01	RAB6B	3	rs493186	1.91E+08	6.97E-01	N/A	-	5.17E-07	3.02E-01	1.18E-05	7159	
5	rs1158196	58017591	5.70E-01	RAB3C	14	rs7150776	92183574	8.61E-01	RIN3	-	5.17E-07	3.62E-01	1.69E-05	7160	
10	rs1907721	78420926	4.72E-01	KCNMA1	20	rs6047565	21605602	5.78E-01	N/A	-	5.17E-07	4.13E-01	1.85E-03	7161	
3	rs2629396	1.21E+08	9.07E-01	CD80	18	s11873104	20566685	1.56E-01	N/A	-	5.17E-07	4.90E-01	4.87E-06	7162	
3	rs9815397	72400160	4.54E-02	N/A	10	s11002552	79870936	7.15E-02	N/A	-	5.17E-07	6.72E-01	9.13E-05	7163	
6	rs6932496	11497723	6.95E-01	NEDD9	1	s12407360	30137216	8.43E-01	N/A	-	5.17E-07	3.85E-01	9.72E-06	7164	
13	rs1931950	73881784	6.29E-01	N/A	14	s11626456	81580316	3.32E-01	N/A	-	5.17E-07	8.25E-01	4.92E-05	7165	
10	rs1751295	4486292	2.55E-01	N/A	16	rs3852716	81170828	4.46E-01	N/A	-	5.17E-07	3.96E-01	2.24E-06	7166	

SNP A					SNP B						Interaction P				
CHR	SNP	Location	gle locus P va	Gene	CHR	SNP	Location	ingle locus P valu	Gene	IHC region	Stage 1	Stage 2	Combined	Ranking	Cluster in top 100 interactions
5	rs381034	60927962	7.83E-01	N/A	18	rs903733	32438195	7.11E-01	FHOD3	-	5.17E-07	5.17E-01	2.67E-03	7167	
4	rs11724795	1.49E+08	6.95E-01	ARHGAP10	8	rs2272641	23350706	3.13E-01	ENTPD4	-	5.17E-07	3.66E-01	8.73E-06	7168	
6	rs510849	1.62E+08	4.00E-01	PARK2	8	rs4740033	85925158	5.24E-01	RALYL	-	5.17E-07	3.76E-01	1.18E-06	7169	
1	rs606149	1.92E+08	7.23E-01	N/A	14	rs6573604	64857694	1.15E-01	N/A	-	5.17E-07	9.88E-02	5.13E-03	7170	
1	rs10903128	25235925	7.89E-01	N/A	17	rs4985650	5761818	8.96E-01	N/A	-	5.17E-07	7.75E-01	2.00E-04	7171	
4	rs11096754	34674459	8.16E-01	N/A	11	rs3740958	36429376	2.52E-02	PRR5L	-	5.17E-07	N/A	N/A	7172	
14	rs11158751	68101178	8.01E-01	RAD51L1	20	rs1080026	42846612	5.00E-01	RIMS4	-	5.18E-07	5.11E-01	2.09E-05	7173	
2	rs16825115	2.29E+08	5.37E-01	N/A	3	rs6804761	1.71E+08	9.05E-01	SEC62	-	5.18E-07	2.35E-01	1.03E-03	7174	
8	rs13260745	20608472	9.76E-01	N/A	13	rs4884202	82305944	2.09E-01	N/A	-	5.18E-07	9.11E-01	3.30E-05	7175	
4	rs9993793	16749737	9.46E-01	N/A	19	rs643786	55547761	5.81E-03	N/APSB	-	5.18E-07	6.35E-01	4.57E-05	7176	
3	rs9917841	1.64E+08	7.92E-01	N/A	8	s10111517	70063994	9.86E-01	N/A	-	5.18E-07	6.83E-01	1.08E-04	7177	
13	rs953937	89320971	6.27E-01	N/A	14	s12431784	84014544	2.97E-01	N/A	-	5.18E-07	1.34E-02	7.02E-08	7178	
13	rs953937	89320971	6.27E-01	N/A	14	rs1449108	84016968	2.97E-01	N/A	-	5.18E-07	1.34E-02	7.02E-08	7179	
3	rs11920780	1.36E+08	5.41E-01	EPHB1	4	rs2165289	1.18E+08	8.08E-01	N/A	-	5.18E-07	3.50E-02	5.50E-07	7180	
17	rs917343	66910901	9.59E-01	N/A	20	rs6119407	31867302	6.93E-01	CHMP4B	-	5.18E-07	3.30E-01	8.55E-04	7181	
7	rs3807827	8197116	8.52E-01	ICA1	9	rs7871964	23654696	5.09E-01	N/A	-	5.18E-07	4.88E-01	1.92E-04	7182	
13	rs9316871	21759921	6.03E-01	N/A	13	rs7329652	46394115	2.14E-01	N/A	-	5.18E-07	5.90E-01	2.56E-04	7183	
3	rs2133884	56821109	8.39E-01	ARHGEF3	8	rs6557594	22711133	2.99E-01	PEBP4	-	5.18E-07	1.21E-01	1.17E-06	7184	
3	rs2657606	16151302	2.50E-01	N/A	8	rs7464161	1.4E+08	6.55E-01	N/A	-	5.18E-07	8.07E-01	1.50E-04	7185	
10	rs2394616	71442025	7.95E-01	N/A	13	rs9576397	37381521	8.20E-01	N/A	-	5.19E-07	3.17E-01	5.39E-04	7186	
5	rs4975727	2111143	5.84E-01	N/A	20	rs4814877	19564837	6.23E-01	SLC24A3	-	5.19E-07	8.55E-01	4.34E-05	7187	
9	rs10815480	6902767	7.21E-01	KDM4C	14	s17098608	31763855	6.29E-01	N/A	-	5.19E-07	4.47E-03	1.92E-02	7188	
2	rs7604050	1.17E+08	6.14E-01	N/A	2	s12619432	2.14E+08	6.33E-01	SPAG16	-	5.19E-07	4.17E-02	3.87E-07	7189	
10	rs670482	84253897	3.21E-01	NRG3	13	rs9573305	73293493	3.29E-01	KLF12	-	5.19E-07	4.85E-01	3.90E-05	7190	
10	rs7079799	14146320	2.45E-01	FRMD4A	12	rs2263487	45537094	2.68E-02	N/A	-	5.19E-07	9.17E-01	1.58E-04	7191	
12	rs9435	22734474	9.98E-01	ETNK1	13	rs658343	55025869	8.40E-01	N/A	-	5.19E-07	8.62E-01	2.85E-05	7192	
2	rs4852124	2.4E+08	4.24E-01	N/A	5	rs32598	83478888	4.50E-01	EDIL3	-	5.19E-07	7.08E-01	7.22E-06	7193	
5	rs1505074	82293747	8.76E-01	N/A	5	rs1833710	1.48E+08	9.49E-01	HTR4	-	5.19E-07	9.91E-01	4.49E-05	7194	
4	rs13138512	1.2E+08	7.79E-01	SYNPO2	13	rs7321643	55956435	9.73E-01	N/A	-	5.19E-07	4.47E-01	1.38E-05	7195	
4	rs13138512	1.2E+08	7.79E-01	SYNPO2	13	rs7994712	55952332	9.73E-01	N/A	-	5.19E-07	4.47E-01	1.38E-05	7196	
6	rs13192959	53608748	2.32E-01	KLHL31	2	rs2674075	59649524	6.05E-01	N/A	-	5.19E-07	N/A	N/A	7197	
6	rs1326543	53605181	2.32E-01	KLHL31	2	rs2674075	59649524	6.05E-01	N/A	-	5.19E-07	N/A	N/A	7198	
17	rs105025	39342967	3.05E-01	MPP2	18	rs4891904	66962989	9.00E-01	N/A	-	5.19E-07	5.25E-02	2.56E-07	7199	
7	rs8180796	73472036	4.84E-01	CLIP2	16	rs4787384	26633023	9.94E-01	N/A	-	5.19E-07	6.86E-01	2.79E-04	7200	
5	rs2279980	57949136	5.54E-01	RAB3C	14	rs4904964	92169620	6.24E-01	RIN3	-	5.19E-07	7.98E-01	2.65E-04	7201	
6	rs9370225	53654545	9.52E-02	KLHL31	5	rs32652	1.19E+08	1.50E-01	TNFAIP8	-	5.19E-07	2.28E-02	4.82E-07	7202	
3	rs3749404	31720390	1.77E-01	N/A	18	rs8082866	3415211	3.88E-01	TGIF1	-	5.19E-07	2.44E-02	1.08E-02	7203	
1	rs851121	18960352	5.40E-01	PAX7	14	rs1168987	35467431	3.15E-01	N/A	-	5.19E-07	1.23E-01	4.55E-03	7204	
3	rs1826351	99269950	3.42E-02	OR5AC2	11	rs7949197	97143884	9.52E-01	N/A	-	5.19E-07	3.66E-01	2.61E-05	7205	
5	rs10491237	10965382	4.28E-01	N/A	8	rs1994370	62303895	6.96E-01	N/A	-	5.19E-07	5.01E-01	4.50E-05	7206	
9	rs10760932	1.05E+08	5.61E-01	CYLC2	19	rs400658	6573352	9.69E-01	N/A	-	5.19E-07	6.11E-01	1.97E-04	7207	
7	rs2192017	1.22E+08	8.28E-01	CADPS2	7	s10241449	1.38E+08	7.83E-01	ATP6V0A4	-	5.19E-07	8.85E-01	5.07E-05	7208	
8	rs2737375	31179757	6.46E-02	N/A	20	rs1345368	15458343	6.95E-01	MACROD2	-	5.20E-07	2.73E-02	1.36E-02	7209	
9	rs10780298	81084044	4.79E-01	N/A	17	rs2319453	47208020	8.73E-01	CA10	-	5.20E-07	2.24E-01	4.91E-06	7210	
6	rs9398455	98122073	7.52E-01	N/A	21	s10222063	41216672	7.06E-01	N/A	-	5.20E-07	5.56E-01	4.99E-04	7211	
7	rs3808035	1.03E+08	8.82E-03	RELN	22	rs5761894	25718110	7.83E-03	N/A	-	5.20E-07	3.83E-01	3.33E-03	7212	
3	rs999764	23486342	5.20E-01	UBE2E2	19	s12610520	10210118	9.39E-02	S1PR2	-	5.20E-07	2.45E-01	2.36E-06	7213	
3	rs342913	1.48E+08	6.17E-02	PLSCR5	4	rs1947202	1.8E+08	5.93E-01	N/A	-	5.20E-07	7.70E-01	5.04E-05	7214	
4	rs13111136	30345822	1.25E-01	PCDH7	5	s17651643	16945438	6.10E-02	MYO10	-	5.20E-07	N/A	N/A	7215	
5	rs890779	1.17E+08	9.40E-01	N/A	7	s10807744	71279200	1.46E-01	CALN1	-	5.20E-07	3.73E-01	3.51E-05	7216	
4	rs2290847	1.51E+08	6.60E-01	LRBA	5	rs4260724	1.52E+08	2.46E-01	N/A	-	5.20E-07	4.91E-01	1.17E-05	7217	
8	rs2927345	6718913	7.45E-01	DEFB1	11	rs6483763	21302450	2.80E-02	NELL1	-	5.20E-07	6.04E-01	4.42E-04	7218	
8	rs5743463	6719584	7.45E-01	DEFB1	11	rs6483763	21302450	2.80E-02	NELL1	-	5.20E-07	6.04E-01	4.42E-04	7219	
2	rs6545648	57511448	3.54E-01	N/A	2	rs7566185	59945105	3.77E-02	N/A	-	5.20E-07	5.76E-03	1.07E-08	7220	
2	rs1177265	61192336	9.23E-02	KIAA1841	8	rs2726555	59947966	8.90E-01	TOX	-	5.20E-07	9.72E-02	2.54E-03	7221	
2	rs896637	2.09E+08	5.59E-01	N/A	7	rs6461503	20527521	1.19E-01	N/A	-	5.20E-07	4.05E-01	9.36E-04	7222	
1	rs1134647	2.13E+08	8.01E-01	SMYD2	13	s12584299	1.08E+08	7.00E-02	N/A	-	5.20E-07	4.84E-01	4.13E-04	7223	
1	rs12124666	2.13E+08	8.01E-01	SMYD2	13	s12584299	1.08E+08	7.00E-02	N/A	-	5.20E-07	4.84E-01	4.13E-04	7224	
8	rs11203975	18564773	3.50E-02	PSD3	11	s11220155	1.25E+08	5.18E-01	CHEK1	-	5.21E-07	7.99E-01	5.38E-05	7225	
9	rs10983400	1.19E+08	4.62E-01	ASTN2	12	rs7978850	10613852	4.94E-01	KLRA1	-	5.21E-07	8.35E-01	1.25E-04	7226	
9	rs10868248	71703997	9.40E-01	C9orf135	12	rs1054637	21576375	8.40E-01	GOLT1B	-	5.21E-07	4.28E-01	8.30E-06	7227	
2	rs12614028	10341404	2.68E-01	HPCAL1	16	rs30935	53878002	7.36E-01	N/A	-	5.21E-07	9.02E-01	3.68E-05	7228	
8	rs551496	93555883	9.54E-01	N/A	13	rs1359390	77682214	5.97E-01	N/A	-	5.21E-07	4.69E-01	5.24E-05	7229	
1	rs2873096	53802444	4.99E-01	GLIS1	4	s11725723	1.11E+08	5.94E-01	N/A	-	5.21E-07	7.06E-01	2.32E-04	7230	
4	rs1978916	80666284	5.89E-02	N/A	7	rs2390961	24266172	2.98E-01	N/A	-	5.21E-07	1.32E-01	2.67E-06	7231	
2	rs1430642	79721176	7.19E-01	CTNNA2	7	rs4376436	94244361	7.29E-01	N/A	-	5.21E-07	5.36E-01	4.57E-05	7232	
1	rs2492604	42026375	9.89E-01	HIVEP3	15	rs8025614	39930204	3.55E-01	PLA2G4B	-	5.21E-07	5.87E-01	3.48E-04	7233	
1	rs978528	17222385	2.63E-01	ATP13A2	11	rs1574128	1.28E+08	9.77E-01	KCNJ1	-	5.21E-07	6.16E-01	1.59E-05	7234	
7	rs2066984	70518586	4.56E-01	WBSCR17	7	s12707374	1.37E+08	6.77E-01	CREB3L2	-	5.21E-07	6.88E-01	1.62E-04	7235	
1	rs12022475	80075562	4.18E-02	N/A	10	rs7086377	3586526	5.88E-01	N/A	-	5.21E-07	3.11E-01	9.82E-06	7236	
2	rs4669725	11448167	6.07E-01	N/A	3	rs9883965	1.66E+08	4.54E-01	SI	-	5.21E-07	4.57E-01	2.55E-03	7237	
2	rs7598757	77781822	6.62E-01	N/A	18	s12964454	70389710	2.81E-01	CNDP1	-	5.21E-07	2.40E-01	8.95E-04	7238	
6	rs2071286	32287874	3.66E-01	GPSM3	4	rs4692694	1.7E+08	5.90E-02	N/A	MHC	5.21E-07	4.21E-01	3.75E-04	7239	
3	rs1381798	38412475	6.28E-01	XYLB	12	rs961810	91154907	6.30E-01	N/A	-	5.21E-07	4.32E-01	2.70E-03	7240	
3	rs6779681	1.39E+08	8.78E-02	N/A	15	s11070359	40145872	4.50E-01	PLA2G4D	-	5.22E-07	8.97E-01	1.07E-04	7241	
1	rs819666	2.35E+08	3.68E-01	ACTN2	4	rs7669416	1.4E+08	1.68E-01	N/A	-	5.22E-07	5.32E-01	1.69E-05	7242	
1	rs10754115	1.93E+08	1.38E-01	N/A	5	rs27114	14498585	4.08E-01	TRIO	-	5.22E-07	6.41E-01	4.65E-05	7243	
6	rs1226493	47096607	4.02E-01	GPR110	22	rs4821797	37346128	4.82E-01	LOC646851	-	5.22E-07	6.63E-01	1.03E-04	7244	
4	rs2061231	45333604	5.46E-01	N/A	8	rs4960992	87985797	2.78E-01	CNBD1	-	5.22E-07	1.33E-01	1.53E-02	7245	
4	rs7687343	1.52E+08	2.07E-01	LRBA	10	rs9423479	3156345	6.44E-01	PFKP	-	5.22E-07	8.05E-01	9.19E-05	7246	
1	rs489933	5848388	1.86E-01	NPHP4	12	rs7300043	3828974	1.06E-01	PARP11	-	5.22E-07	5.97E-01	3.42E-05	7247	
2	rs1857706	49131455	7.68E-01	FSHR	7	rs6963278	21046932	4.18E-01	N/A	-	5.22E-07	4.93E-01	6.34E-04	7248	
3	rs1284726	1.04E+08	2.34E-01	N/A	13	rs9534986	47665333	6.35E-01	N/A	-	5.22E-07	2.27E-01	2.14E-06	7249	
12	rs12228293	65408694	7.51E-01	N/A	15	rs2083013	60188774	8.94E-01	N/A	-	5.22E-07	4.93E-01	3.12E-05	7250	
6	rs12190699	91173809	9.04E-01	N/A	1	rs7547610	89469191	4.87E-01	N/A	-	5.23E-07	2.09E-01	2.31E-03	7251	
1	rs10800813	2E+08	2.97E-01	N/A	14	rs4981422	21867816	9.51E-01	N/A	-	5.23E-07	3.84E-01	7.36E-06	7252	
2	rs1861612	2.3E+08	6.42E-01	DNER	8	rs3829049	70925162	6.73E-01	SLCO5A1	-	5.23E-07	7.70E-02	7.96E-04	7253	
1	rs7518553	32087734	4.95E-01	N/A	3	rs7622240	85444335	7.52E-01	CADM2	-	5.23E-07	5.29E-01	2.07E-05	7254	
3	rs9311474	52313892	1.13E-01	DN/AH1	7	rs7809589	1.51E+08	4.91E-01	PRKAG2	-	5.23E-07	7.68E-01	2.41E-05	7255	
8	rs11203975	18564773	3.50E-02	PSD3	11	s11220153	1.25E+08	5.26E-01	CHEK1	-	5.23E-07	8.21E-01	5.21E-05	7256	
2	rs1470414	2.42E+08	7.32E-01	PPP1R7	10	rs7077238	1.3E+08	7.67E-01	CLRN3	-	5.23E-07	8.98E-01	4.35E-04	7257	
5	rs293649	1.06E+08	7.59E-01	N/A	14	s10148134	78252844	5.63E-01	NRXN3	-	5.23E-07	4.90E-01	1.45E-05	7258	
5	rs1288205	1.62E+08	1.66E-01	N/A	5	s10055695	1.78E+08	6.03E-01	N/A	-	5.23E-07	2.17E-01	1.98E-03	7259	
7	rs1178118	18718523	9.40E-03	HDAC9	13	rs4769088	19620066	6.47E-01	GJA3	-	5.23E-07	5.38E-01	6.73E-06	7260	
8	rs1351424	1.36E+08	8.70E-01	N/A	13	rs9541675	68457371	3.97E-01	N/A	-	5.23E-07	2.69E-02	2.27E-07	7261	
8	rs2439520	97588101	5.97E-01	SDC2	11	rs1398259	1.15E+08	7.48E-01	CADM1	-	5.23E-07	2.82E-02	3.34E-07	7262	
11	rs4611172	94473918	3.46E-01	ENDOD1	18	rs9941423	479385	4.78E-02	COLEC12	-	5.23E-07	8.20E-01	3.51E-05	7263	
1	rs1980684	96663224	7.61E-01	N/A	8	s10504553	75038957	6.47E-01	TMEM70	-	5.23E-07	3.05E-03	2.82E-02	7264	
7	rs1015931	1.48E+08	7.03E-01	CNTN/AP2	14	rs1017141	80639431	7.34E-01	TSHR	-	5.23E-07	9.20E-02	4.89E-03	7265	
7	rs2972102	1.48E+08	7.03E-01	CNTN/AP2	14	rs1017141	80639431	7.34E-01	TSHR	-	5.23E-07	1.17E-01	3.52E-03	7266	
2	rs6732000	64868700	7.19E-01	N/A	21	rs995819	38366840	8.01E-01	DSCR4	-	5.23E-07	4.26E-01	8.38E-04	7267	
1	rs3119186	1.01E+08	1.12E-01	N/A	2	rs2288644	9996621	4.20E-01	GRHL1	-	5.23E-07	6.93E-01	1.20E-05	7268	
9	rs10811278	19888792	5.22E-01	N/A	10	rs7915543	94971879	1.97E-01	N/A	-	5.23E-07	9.45E-01	1.28E-04	7269	
6	rs6937213	20481752	5.25E-02	N/A	18	rs1587989	60554118	8.65E-01	N/A	-	5.23E-07	1.06E-01	1.23E-06	7270	
1	rs623400	1.12E+08	7.42E-01	DENND2D	3	rs7428418	1.06E+08	9.96E-01	N/A	-	5.23E-07	7.73E-01	2.83E-05	7271	
2	rs6735981	2.05E+08	9.72E-01	PARD3B	19	rs4807863	6257971	8.37E-02	ACER1	-	5.23E-07	2.74E-01	5.39E-06	7272	
11	rs10839632	6848280	7.62E-01	OR10A4	18	rs4940460	55196114	2.85E-02	LMAN1	-	5.24E-07	5.93E-01	3.50E-05	7273	
7	rs4607527	1.41E+08	7.87E-02	MGAM	8	rs907461	1.39E+08	6.69E-01	N/A	-	5.24E-07	6.55E-01	1.22E-05	7274	
5	rs7706759	1.78E+08	4.31E-01	COL23A1	18	rs9950852	69317869	9.09E-01	N/A	-	5.24E-07	N/A	N/A	7275	
12	rs3850005	1.28E+08	3.85E-01	TMEM132D	14	rs3783941	80644758	8.52E-01	TSHR	-	5.24E-07	1.22E-01	6.99E-03	7276	
6	rs9295957	31265572	2.18E-01	POU5F1	18	rs2732229	71742290	5.44E-01	N/A	MHC	5.24E-07	5.99E-01	3.46E-04	7277	
1	rs11260657	5404973	6.43E-01	N/A	11	rs486052	83813054	8.55E-01	DLG2	-	5.24E-07	9.48E-01	6.46E-05	7278	

SNP A					SNP B						Interaction P			Ranking	Cluster in top 100 interactions
CHR	SNP	Location	gle locus P va	Gene	CHR	SNP	Location	Single locus P valu	Gene	MHC region	Stage 1	Stage 2	Combined		
12	rs2111343	95979264	9.44E-01	N/A	15	rs1480086	96446213	5.97E-01	N/A	-	5.24E-07	5.36E-01	3.06E-05	7279	
10	rs9663977	4507381	3.48E-01	N/A	16	s12598271	81163012	3.75E-01	N/A	-	5.24E-07	2.22E-01	9.60E-07	7280	
12	rs53025	29565117	7.82E-01	N/A	13	rs7981459	1.05E+08	4.55E-01	N/A	-	5.24E-07	4.21E-01	1.28E-05	7281	
7	rs11983119	9047577	9.64E-01	N/A	7	rs2214740	46906589	9.74E-01	N/A	-	5.24E-07	8.91E-02	2.96E-03	7282	
7	rs10279330	35613505	3.30E-01	N/A	19	rs4805602	35897140	2.21E-01	N/A	-	5.24E-07	2.99E-01	1.32E-03	7283	
6	rs6941942	1.11E+08	4.58E-01	N/A	1	s10798126	1.86E+08	8.20E-01	N/A	-	5.24E-07	3.83E-01	7.56E-04	7284	
2	rs174253	1.6E+08	9.19E-01	BAZ2B	2	rs514898	2.2E+08	2.70E-01	N/A	-	5.24E-07	5.31E-01	3.88E-05	7285	
4	rs557785	1.12E+08	1.57E-02	ENPEP	8	rs1994792	96378313	6.70E-01	N/A	-	5.24E-07	N/A	N/A	7286	
9	rs7042130	77281975	4.69E-01	N/A	13	rs7999126	86769337	9.01E-01	N/A	-	5.24E-07	N/A	N/A	7287	
9	rs7853037	7007391	6.43E-01	KDM4C	13	rs7982669	97339220	4.65E-01	N/A	-	5.25E-07	9.51E-01	1.33E-04	7288	
6	rs6918423	27302051	6.03E-01	N/A	15	rs813299	29225595	8.06E-01	N/A	MHC	5.25E-07	9.62E-01	7.22E-05	7289	
6	rs3800312	27318593	6.03E-01	PRSS16	15	rs813299	29225595	8.06E-01	N/A	MHC	5.25E-07	9.95E-01	1.11E-04	7290	
10	rs10785907	91563972	3.28E-01	N/A	16	s17192054	6530267	3.59E-01	A2BP1	-	5.25E-07	6.01E-01	2.60E-04	7291	
1	rs1004766	37597095	9.31E-01	N/A	2	s11687313	2.01E+08	8.42E-01	N/A	-	5.25E-07	9.98E-01	4.72E-05	7292	
12	rs1354156	1.14E+08	1.51E-02	TBX3	17	s11655550	34863649	5.95E-02	MED1	-	5.25E-07	8.48E-03	8.85E-08	7293	
18	rs1546564	32290294	4.79E-01	FHOD3	19	rs919271	57360226	2.20E-01	ZNF836	-	5.25E-07	8.64E-01	1.11E-04	7294	
6	rs2295261	47344839	6.30E-01	TNFRSF21	10	rs7074064	88673102	9.77E-01	BMPR1A	-	5.25E-07	9.72E-01	3.59E-04	7295	
6	rs4342445	1.6E+08	6.60E-01	SOD2	12	rs7133563	5308881	6.30E-01	N/A	-	5.25E-07	5.36E-01	2.66E-05	7296	
6	rs9365085	1.6E+08	6.60E-01	SOD2	12	rs7133563	5308881	6.30E-01	N/A	-	5.25E-07	5.36E-01	2.66E-05	7297	
2	rs10169420	1.83E+08	4.31E-01	N/A	12	rs1846035	82545653	9.72E-01	N/A	-	5.25E-07	4.83E-01	2.86E-05	7298	
17	rs8077733	28043066	3.83E-01	MYO1D	18	rs3911593	46436326	7.35E-01	MAPK4	-	5.25E-07	4.79E-01	1.16E-03	7299	
7	rs4724070	41749884	3.09E-01	LOC285954	17	rs895690	70296244	2.37E-01	SLC9A3R1	-	5.25E-07	5.95E-01	4.78E-04	7300	
3	rs4683070	45174624	4.12E-01	CDCP1	20	s12329405	14281754	5.20E-02	MACROD2	-	5.25E-07	6.33E-01	3.60E-05	7301	
7	rs6960014	14307870	4.40E-01	DGKB	20	rs2697515	26033338	6.54E-01	FAM182A	-	5.25E-07	8.07E-01	6.76E-05	7302	
2	rs2031028	2991297	6.14E-01	N/A	4	rs1992981	27328888	6.94E-01	N/A	-	5.25E-07	9.29E-01	8.99E-05	7303	
9	rs2148600	26562236	4.99E-01	N/A	11	s11215437	1.15E+08	6.38E-01	CADM1	-	5.25E-07	4.80E-01	5.07E-04	7304	
11	rs1442717	20122076	8.67E-01	DBX1	13	s10492482	1.09E+08	2.55E-01	N/A	-	5.25E-07	5.81E-01	1.77E-05	7305	
1	rs7532302	1.55E+08	1.39E-01	NES	7	rs6466069	1.05E+08	9.94E-01	PUS7	-	5.25E-07	8.44E-01	2.91E-04	7306	
1	rs7516314	89823419	1.10E-01	LRRC8B	7	rs7793355	1.24E+08	2.39E-01	N/A	-	5.25E-07	4.03E-01	1.45E-03	7307	
5	rs10477862	1.06E+08	8.56E-02	N/A	12	s10506439	61930601	4.64E-01	N/A	-	5.25E-07	7.78E-01	4.17E-05	7308	
1	rs3949904	62620678	4.41E-01	N/A	4	rs9992060	1.89E+08	5.12E-01	N/A	-	5.26E-07	1.30E-01	9.01E-03	7309	
9	rs7855908	70706902	5.79E-01	PIP5K1B	9	rs4515651	90095083	2.25E-01	N/A	-	5.26E-07	2.79E-02	1.81E-07	7310	
3	rs958144	89077362	4.21E-01	N/A	17	rs4796460	6131231	5.37E-01	N/A	-	5.26E-07	9.31E-01	4.32E-05	7311	
2	rs6704827	41869279	6.25E-01	N/A	12	rs4931005	32036267	9.10E-01	C12orf35	-	5.26E-07	8.13E-01	2.37E-04	7312	
7	rs13244987	1.57E+08	6.87E-01	N/A	12	rs7133582	1.16E+08	7.16E-01	KSR2	-	5.26E-07	6.64E-01	6.03E-05	7313	
12	rs11052333	32994537	4.58E-01	N/A	12	s11615997	1.28E+08	3.34E-01	TMEM132D	-	5.26E-07	4.34E-01	6.20E-04	7314	
4	rs633398	1.19E+08	2.43E-01	NDST3	10	rs4028374	16511634	8.46E-01	PTER	-	5.26E-07	8.12E-01	3.64E-04	7315	
6	rs3752534	1.64E+08	2.08E-01	N/A	17	rs4794053	43124550	4.39E-01	KPNB1	-	5.26E-07	9.60E-02	1.51E-03	7316	
7	rs798328	77733361	8.28E-01	MAGI2	13	rs9576336	37152339	7.39E-02	TRPC4	-	5.26E-07	2.12E-01	1.32E-06	7317	
14	rs955119	85994405	1.04E-01	N/A	19	rs251896	44510231	2.01E-01	SAMD4B	-	5.26E-07	9.40E-01	3.56E-04	7318	
4	rs1478095	1.32E+08	6.79E-01	N/A	8	rs372976	82634163	4.55E-01	N/A	-	5.26E-07	N/A	N/A	7319	
4	rs1478095	1.32E+08	6.79E-01	N/A	8	rs396738	82634100	4.55E-01	N/A	-	5.26E-07	N/A	N/A	7320	
1	rs4652753	1.81E+08	2.95E-01	N/A	3	rs184452	64125246	5.83E-01	PRICKLE2	-	5.26E-07	7.43E-01	1.57E-04	7321	
2	rs2540100	59604268	7.95E-01	N/A	3	rs6439301	1.33E+08	8.79E-01	MRPL3	-	5.27E-07	1.94E-01	1.47E-06	7322	
9	rs10124022	72472635	7.39E-01	TRPM3	18	rs1625796	28369416	9.26E-01	N/A	-	5.27E-07	3.23E-01	6.88E-06	7323	
19	rs919803	35678944	1.57E-01	ZNF536	22	rs5769941	47928225	6.71E-02	N/A	-	5.27E-07	3.53E-01	1.23E-05	7324	
6	rs4618489	85230221	9.25E-01	N/A	4	s12506399	5975695	8.44E-01	N/A	-	5.27E-07	9.58E-01	7.16E-05	7325	
6	rs9362053	85234736	9.25E-01	N/A	4	s12506399	5975695	8.44E-01	N/A	-	5.27E-07	9.58E-01	7.16E-05	7326	
2	rs6709502	30864428	9.17E-01	CAPN13	11	rs483058	1.16E+08	9.05E-01	N/A	-	5.27E-07	2.66E-01	1.59E-06	7327	
4	rs7657164	1.69E+08	4.42E-01	DDX60	15	s11633288	45664002	9.88E-01	N/A	-	5.27E-07	2.83E-01	7.27E-04	7328	
14	rs11844935	30734018	5.71E-01	HECTD1	18	s16943693	23314984	5.93E-03	N/A	-	5.27E-07	7.52E-01	1.24E-04	7329	
8	rs4291239	1.03E+08	3.97E-01	N/A	13	rs7326356	71581505	1.62E-01	N/A	-	5.27E-07	5.05E-01	2.00E-05	7330	
5	rs9327438	1.27E+08	5.38E-01	MEGF10	7	s10225002	1.58E+08	2.26E-02	PTPRN2	-	5.27E-07	7.17E-01	2.41E-05	7331	
5	rs9327438	1.27E+08	5.38E-01	MEGF10	7	s10265417	1.58E+08	2.26E-02	PTPRN2	-	5.27E-07	7.17E-01	2.41E-05	7332	
6	rs942723	1.65E+08	8.26E-01	N/A	2	s13387662	34016918	2.26E-01	N/A	-	5.27E-07	9.73E-01	5.39E-05	7333	
6	rs6932930	34785081	2.64E-01	C6orf106	5	rs3010240	72045895	1.43E-01	N/A	MHC	5.27E-07	1.39E-02	1.65E-06	7334	
7	rs7807486	8775211	7.61E-01	NXPH1	15	rs2007355	49934917	6.32E-02	TMOD3	-	5.28E-07	3.30E-01	7.80E-06	7335	
5	rs2526150	1.35E+08	5.12E-01	FBXL21	15	s12148733	70622069	9.56E-01	ARIH1	-	5.28E-07	6.41E-02	3.27E-07	7336	
3	rs259489	21916263	1.63E-01	N/A	12	rs1080902	41884180	7.83E-01	N/A	-	5.28E-07	8.53E-01	4.36E-05	7337	
1	rs10863725	2.07E+08	8.94E-01	N/A	2	s10191044	18579682	1.89E-01	RDH14	-	5.28E-07	8.79E-01	6.12E-05	7338	
1	rs10863725	2.07E+08	8.94E-01	N/A	2	s10495687	18580060	1.89E-01	RDH14	-	5.28E-07	8.79E-01	6.12E-05	7339	
1	rs7552260	30624265	7.04E-01	N/A	18	rs657773	55097571	7.94E-01	RAX	-	5.28E-07	2.44E-01	2.35E-03	7340	
2	rs7586962	2.15E+08	8.48E-02	BARD1	7	s12698902	69475255	2.51E-01	AUTS2	-	5.28E-07	8.06E-01	1.65E-04	7341	
2	rs2304674	2.39E+08	1.83E-01	PER2	20	s17744800	21422355	4.36E-01	NKX2-2	-	5.28E-07	7.09E-01	4.61E-05	7342	
3	rs6443863	1.85E+08	6.71E-01	MCF2L2	20	rs2024594	46951201	7.98E-01	N/A	-	5.28E-07	8.43E-01	1.23E-04	7343	
1	rs1466613	79409914	4.91E-01	N/A	4	rs2626608	1.73E+08	5.00E-01	GALNTL6	-	5.28E-07	3.57E-01	2.44E-06	7344	
2	rs341605	44917691	3.10E-02	N/A	15	rs2602141	41511938	1.58E-01	TP53BP1	-	5.28E-07	5.03E-01	7.74E-04	7345	
4	rs2310129	29711586	3.44E-01	N/A	18	rs3902020	48065150	7.07E-01	N/A	-	5.28E-07	6.07E-01	2.23E-04	7346	
12	rs7976486	74660715	3.38E-01	N/A	13	rs1119911	30979769	7.70E-01	N/A	-	5.28E-07	9.19E-02	2.40E-03	7347	
8	rs477516	16758362	9.63E-01	N/A	9	rs1413258	20166246	5.03E-01	N/A	-	5.28E-07	4.05E-01	1.06E-03	7348	
3	rs6786950	903456	9.05E-01	N/A	7	rs2402852	1.26E+08	3.71E-01	GRM8	-	5.28E-07	8.15E-01	1.60E-04	7349	
4	rs13149605	1.88E+08	4.46E-01	N/A	9	rs1571231	37401834	1.64E-01	GRHPR	-	5.29E-07	6.72E-01	9.61E-05	7350	
12	rs10506937	85825624	7.99E-01	N/A	16	rs4243218	80458129	5.86E-01	PLCG2	-	5.29E-07	7.03E-01	3.43E-04	7351	
10	rs2387667	1420761	1.95E-01	ADARB2	17	rs8078179	14856327	9.16E-01	N/A	-	5.29E-07	7.98E-01	2.52E-04	7352	
14	rs11625068	61803905	8.47E-01	N/A	18	rs4890490	40691266	4.60E-01	SETBP1	-	5.29E-07	1.76E-01	8.59E-06	7353	
5	rs877886	7712279	7.77E-01	ADCY2	13	rs1326419	88080653	3.54E-01	N/A	-	5.29E-07	5.30E-01	3.03E-05	7354	
5	rs7700862	7699404	7.77E-01	ADCY2	13	rs1326419	88080653	3.54E-01	N/A	-	5.29E-07	5.51E-01	3.15E-05	7355	
6	rs9384296	1.56E+08	7.99E-01	TIAM2	1	s11206140	53538022	3.82E-02	LRP8	-	5.29E-07	1.54E-01	4.14E-06	7356	
3	rs7635505	64624234	1.80E-01	ADAMTS9	16	rs2547033	24651158	4.73E-01	TNRC6A	-	5.29E-07	1.64E-01	2.17E-06	7357	
5	rs249663	1.42E+08	8.54E-01	N/A	8	rs6470155	1.25E+08	4.92E-01	N/A	-	5.29E-07	3.16E-01	1.89E-06	7358	
5	rs3822476	77749577	4.95E-01	SCAMP1	11	rs1944971	73601066	3.55E-01	PPME1	-	5.29E-07	7.02E-01	3.44E-05	7359	
1	rs1340592	67793691	4.96E-01	N/A	12	rs2732439	47030466	7.19E-01	N/A	-	5.29E-07	9.11E-01	9.57E-05	7360	
1	rs6662607	67794090	4.96E-01	N/A	12	rs2732439	47030466	7.19E-01	N/A	-	5.29E-07	9.11E-01	9.57E-05	7361	
1	rs6693292	67795756	4.96E-01	N/A	12	rs2732439	47030466	7.19E-01	N/A	-	5.29E-07	9.11E-01	9.57E-05	7362	
1	rs4655733	67798226	4.96E-01	N/A	12	rs2732439	47030466	7.19E-01	N/A	-	5.29E-07	9.75E-01	7.83E-05	7363	
3	rs2653321	1.89E+08	6.66E-01	N/A	22	s13055229	26149250	1.13E-01	N/A	-	5.29E-07	2.51E-01	8.48E-04	7364	
3	rs2130369	1.01E+08	7.50E-01	C3orf26	8	s11203826	16943551	5.66E-01	EFHA2	-	5.29E-07	8.30E-01	9.98E-06	7365	
8	rs4439163	72496335	8.31E-01	N/A	21	rs2838808	45445534	8.25E-01	ADARB1	-	5.29E-07	5.29E-01	1.71E-05	7366	
12	rs10774178	3853496	8.18E-01	PARP11	13	rs7993566	97349660	7.09E-01	N/A	-	5.29E-07	4.56E-01	1.58E-05	7367	
2	rs6705773	2.21E+08	2.92E-01	N/A	5	s17702421	1.36E+08	6.28E-01	N/A	-	5.29E-07	9.79E-01	2.05E-04	7368	
1	rs1321646	1.57E+08	3.95E-01	CD1B	2	rs2114631	1.6E+08	5.19E-01	LY75	-	5.29E-07	3.46E-01	1.15E-03	7369	
3	rs4686997	1.9E+08	8.61E-01	LPP	14	rs2332909	72233067	4.87E-01	DPF3	-	5.30E-07	4.56E-01	1.60E-03	7370	
7	rs7789227	12089416	2.68E-01	N/A	7	s10252758	71052221	4.14E-01	CALN1	-	5.30E-07	5.56E-01	1.10E-03	7371	
3	rs697357	1.67E+08	1.61E-02	N/A	11	rs7925271	4621233	5.42E-01	OR51E1	-	5.30E-07	5.65E-01	4.08E-05	7372	
1	rs10494739	1.94E+08	7.09E-01	N/A	1	s10900497	2.04E+08	8.79E-01	CDK18	-	5.30E-07	6.31E-01	5.13E-05	7373	
11	rs11037877	5483740	7.33E-01	UBQLN3	18	s11564338	23972174	2.55E-01	CDH2	-	5.30E-07	9.69E-01	4.48E-04	7374	
11	rs11037877	5483740	7.33E-01	UBQLN3	18	rs8094439	23966605	2.55E-01	CDH2	-	5.30E-07	9.69E-01	4.48E-04	7375	
10	rs4748341	17087787	1.73E-01	CUBN	11	rs1145403	99199515	2.75E-01	CNTN5	-	5.30E-07	3.08E-01	9.49E-07	7376	
7	rs736198	13069584	5.91E-02	N/A	19	rs4801457	62485375	9.79E-01	ZNF460	-	5.30E-07	4.60E-01	5.64E-06	7377	
1	rs2024788	61659856	4.03E-01	NFIA	4	rs2531208	10241502	8.98E-01	CLNK	-	5.30E-07	5.66E-01	1.78E-04	7378	
4	rs4862432	1.86E+08	2.49E-02	N/A	14	rs2179932	30503224	9.59E-01	STRN3	-	5.30E-07	8.66E-01	2.77E-04	7379	
4	rs298988	1.2E+08	1.77E-01	SEC24D	4	s10006257	1.46E+08	9.20E-01	N/A	-	5.30E-07	5.50E-01	2.69E-05	7380	
6	rs4715646	57008299	4.50E-01	BEND6	2	rs775806	1.22E+08	8.02E-01	N/A	-	5.31E-07	4.83E-01	8.40E-06	7381	
6	rs6455534	1.69E+08	1.53E-01	SMOC2	21	rs8128228	40395436	9.46E-01	DSCAM	-	5.31E-07	2.83E-01	1.64E-06	7382	
15	rs6495119	72721493	7.68E-01	EDC3	21	rs1012959	36983492	9.81E-01	SIM2	-	5.31E-07	3.90E-01	7.17E-06	7383	
7	rs6979644	1.44E+08	4.14E-02	TPK1	17	rs7406059	33216095	2.94E-01	N/A	-	5.31E-07	6.16E-01	3.48E-04	7384	
5	rs2562262	1.25E+08	6.94E-01	N/A	14	rs4465523	19983363	4.03E-01	TMEM55B	-	5.31E-07	9.42E-01	9.40E-05	7385	
17	rs8073602	74849485	9.24E-01	HRNBP3	18	rs8091916	3182682	6.18E-02	MYOM1	-	5.31E-07	6.11E-01	6.31E-04	7386	
5	rs2731714	1.72E+08	5.77E-01	SH3PXD2B	22	rs137537	31633500	7.10E-02	SYN3	-	5.31E-07	2.13E-01	1.32E-03	7387	
7	rs6460980	12838836	4.21E-01	N/A	13	rs4886227	59888507	7.91E-01	TDRD3	-	5.31E-07	2.67E-01	4.63E-06	7388	
6	rs1265110	31227401	8.90E-01	TCF19	6	rs3130473	31307187	2.29E-01	N/A	MHC	5.31E-07	2.54E-01	6.49E-06	7389	
2	rs12612419	1.6E+08	9.90E-01	WDSUB1	10	s12255141	1.14E+08	4.35E-01	VTI1A	-	5.31E-07	6.57E-01	3.28E-04	7390	

SNP A					SNP B						Interaction P			Ranking	Cluster in top 100 interactions
CHR	SNP	Location	gle locus P va	Gene	CHR	SNP	Location	Single locus P valu	Gene	HC region	Stage 1	Stage 2	Combined		
3	rs3905902	12000779	6.46E-01	N/A	10	rs11254053	16607306	8.69E-01	PTER	-	5.31E-07	3.89E-01	4.55E-04	7391	
3	rs9857596	6571774	6.65E-01	N/A	4	rs7691785	1.66E+08	2.37E-01	N/A	-	5.31E-07	1.09E-02	1.87E-02	7392	
8	rs7007064	63261986	5.07E-01	N/A	9	rs3118853	89483169	8.61E-02	DAPK1	-	5.31E-07	2.62E-01	1.47E-03	7393	
10	rs2025468	10656491	3.55E-01	N/A	19	rs425655	52063456	2.78E-01	AP2S1	-	5.31E-07	5.72E-01	3.29E-04	7394	
10	rs2025468	10656491	3.55E-01	N/A	19	rs7259209	52095694	2.78E-01	GRLF1	-	5.31E-07	5.72E-01	3.29E-04	7395	
8	rs11203713	15486984	4.01E-01	TUSC3	12	rs353897	1.17E+08	6.39E-01	TAOK3	-	5.32E-07	1.07E-01	5.84E-03	7396	
4	rs13150890	1.9E+08	8.91E-01	N/A	5	rs980631	72927514	3.41E-01	UTP15	-	5.32E-07	3.81E-01	8.80E-06	7397	
5	rs10941232	35090396	7.53E-02	PRLR	8	rs1487233	1.28E+08	1.03E-01	N/A	-	5.32E-07	1.21E-01	1.97E-06	7398	
13	rs9543825	74465245	1.35E-03	N/A	18	rs17621618	22748311	4.63E-01	CHST9	-	5.32E-07	5.77E-01	4.39E-05	7399	
4	rs1966546	31036506	8.11E-01	N/A	11	rs2640093	90984244	7.29E-01	N/A	-	5.32E-07	N/A	N/A	7400	
2	rs1533971	35990142	1.23E-01	N/A	11	rs11042978	2154994	3.22E-01	N/A	-	5.32E-07	2.71E-01	9.13E-04	7401	
3	rs1997061	1.72E+08	2.64E-01	PRKCI	4	rs2029886	1.22E+08	1.34E-01	N/A	-	5.32E-07	5.58E-01	2.58E-05	7402	
13	rs17233815	92156875	7.20E-01	GPC5	18	rs2291172	63333372	5.45E-02	N/A	-	5.32E-07	8.81E-01	9.34E-05	7403	
9	rs1854442	23689886	9.25E-01	N/A	14	rs4899635	76602425	2.51E-01	N/A	-	5.32E-07	5.51E-02	7.43E-07	7404	
4	rs874950	32976358	9.07E-01	N/A	11	rs2109681	22770039	8.58E-02	GAS2	-	5.32E-07	7.15E-02	4.63E-06	7405	
2	rs16986865	19752687	7.87E-01	N/A	13	rs17638544	41854621	7.32E-01	N/A	-	5.32E-07	2.04E-01	3.10E-06	7406	
6	rs6910409	1.23E+08	3.45E-01	CLVS2	1	rs17596572	1.5E+08	1.20E-01	HRNR	-	5.32E-07	2.35E-01	1.01E-03	7407	
5	rs1345592	1.69E+08	1.10E-01	SLIT3	9	rs1444832	74497539	1.90E-01	TMC1	-	5.32E-07	2.21E-01	6.11E-04	7408	
3	rs6798021	5444862	1.90E-01	N/A	8	rs7834601	1.43E+08	6.16E-01	N/A	-	5.32E-07	2.86E-01	4.52E-04	7409	
4	rs7681982	59940049	7.61E-01	N/A	7	rs17165845	93360865	5.99E-01	GNGT1	-	5.32E-07	7.27E-01	1.64E-04	7410	
14	rs1890723	48512327	8.65E-01	N/A	16	rs10514506	79278578	9.46E-01	CDYL2	-	5.33E-07	7.88E-01	1.40E-05	7411	
3	rs1461820	28766590	7.62E-01	N/A	5	rs32128	11396384	2.81E-01	CTNND2	-	5.33E-07	3.03E-01	1.58E-06	7412	
3	rs2630243	1.95E+08	7.53E-01	N/A	20	rs12479923	38039524	2.06E-01	N/A	-	5.33E-07	1.64E-02	3.99E-03	7413	
7	rs7807056	8774929	7.61E-01	NXPH1	15	rs2007355	49934917	6.32E-02	TMOD3	-	5.33E-07	3.72E-01	9.51E-06	7414	
16	rs7190485	14048591	7.16E-01	N/A	19	rs7250902	768708	8.58E-01	N/A	-	5.33E-07	7.46E-01	1.31E-04	7415	
6	rs9277027	33106216	3.69E-01	N/A	14	rs4540991	26620498	2.14E-02	N/A	MHC	5.33E-07	2.82E-01	2.35E-03	7416	
14	rs7148194	1.04E+08	9.05E-01	KIF26A	17	rs4793006	39045489	3.83E-01	N/A	-	5.33E-07	5.68E-01	1.78E-05	7417	
6	rs3130267	33414772	3.22E-01	DAXX	2	rs1344936	12167200	8.58E-01	N/A	MHC	5.33E-07	8.30E-01	1.23E-04	7418	
6	rs567513	86060923	8.10E-01	N/A	9	rs11265909	91267966	3.52E-01	SEMA4D	-	5.33E-07	8.61E-01	1.07E-04	7419	
19	rs17833971	38620583	9.68E-01	PEPD	19	rs16975865	47702683	3.29E-01	CEACAM1	-	5.33E-07	5.43E-01	3.24E-05	7420	
8	rs7002130	1.39E+08	3.23E-01	N/A	10	rs7072336	1.04E+08	7.69E-01	NOLC1	-	5.33E-07	7.26E-01	2.23E-04	7421	
5	rs294039	1.03E+08	9.72E-01	N/A	10	rs4451642	1.13E+08	5.16E-01	N/A	-	5.33E-07	N/A	N/A	7422	
16	rs17287570	16062604	1.95E-01	ABCC1	19	rs1056143	13926488	8.03E-01	PODNL1	-	5.33E-07	5.26E-01	8.16E-04	7423	
1	rs2576241	2.15E+08	7.10E-01	ESRRG	9	rs7020062	1.14E+08	8.71E-01	N/A	-	5.33E-07	7.77E-01	6.57E-05	7424	
15	rs10152229	99780180	3.42E-01	PCSK6	21	rs8128163	39211000	4.81E-01	N/A	-	5.34E-07	7.10E-01	2.47E-04	7425	
16	rs7500355	84634601	7.85E-01	N/A	17	rs4795494	22501836	9.47E-01	N/A	-	5.34E-07	2.06E-01	1.77E-03	7426	
3	rs6549383	71349575	2.78E-01	FOXP1	14	rs2895811	99203695	1.13E-01	HHIPL1	-	5.34E-07	3.23E-01	7.23E-06	7427	
5	rs149352	94224367	7.77E-01	MCTP1	8	rs997258	1.46E+08	2.94E-01	N/A	-	5.34E-07	2.07E-01	7.41E-04	7428	
11	rs7936604	82514027	8.27E-01	N/A	15	rs12915032	70431715	6.37E-01	HEXA	-	5.34E-07	5.65E-01	1.10E-05	7429	
7	rs9641392	77861948	2.83E-02	MAGI2	12	rs1049376	26382742	5.85E-01	ITPR2	-	5.34E-07	9.72E-01	3.31E-04	7430	
2	rs7592322	35930844	1.76E-01	N/A	19	rs12610468	9850470	9.36E-01	OLFM2	-	5.34E-07	5.50E-01	6.36E-06	7431	
7	rs7789227	12089416	2.68E-01	N/A	7	rs6460701	71062107	5.36E-01	CALN1	-	5.34E-07	5.00E-01	9.62E-04	7432	
2	rs12469242	15892005	8.01E-01	N/A	10	rs3858284	88520461	1.99E-01	BMPR1A	-	5.34E-07	9.14E-01	2.24E-04	7433	
15	rs12324434	53501727	5.02E-01	CCPG1	20	rs4401268	11893421	4.17E-01	N/A	-	5.34E-07	9.51E-01	1.65E-04	7434	
6	rs9374809	1.2E+08	7.48E-02	N/A	1	rs749390	1.89E+08	4.35E-02	N/A	-	5.34E-07	5.13E-01	1.13E-05	7435	
1	rs6677529	1.61E+08	7.01E-01	NOS1AP	22	rs713821	25059005	1.71E-01	N/A	-	5.35E-07	1.55E-01	5.71E-07	7436	
4	rs7670291	39366980	4.57E-01	UBE2K	4	rs1498020	1.26E+08	9.18E-01	N/A	-	5.35E-07	3.75E-01	1.56E-05	7437	
9	rs7470806	1.2E+08	7.09E-01	N/A	15	rs7179994	25997365	7.80E-01	OCA2	-	5.35E-07	2.87E-01	5.65E-06	7438	
6	rs2169100	93477143	5.01E-01	N/A	10	rs4256883	63577608	9.79E-02	N/A	-	5.35E-07	4.18E-01	2.89E-05	7439	
6	rs9380215	31157634	4.29E-02	N/A	6	rs130067	31226490	8.62E-01	TCF19	MHC	5.35E-07	5.96E-02	5.33E-07	7440	
1	rs9730586	74734405	7.35E-01	TNNI3K	1	rs11164301	1.02E+08	2.21E-01	N/A	-	5.35E-07	8.10E-01	5.93E-04	7441	
6	rs12196881	40936923	1.22E-01	N/A	8	rs1662248	26499553	1.69E-02	DPYSL2	-	5.35E-07	2.84E-02	3.15E-07	7442	
4	rs1320763	3383007	1.24E-02	RGS12	5	rs13173741	87233291	5.32E-01	N/A	-	5.35E-07	4.90E-01	3.57E-04	7443	
2	rs1430642	79721176	7.19E-01	CTNN/A2	7	rs6965194	94246494	7.30E-01	N/A	-	5.35E-07	6.03E-01	5.15E-05	7444	
1	rs1493081	2.27E+08	9.40E-01	N/A	12	rs4760933	69430025	4.48E-01	PTPRR	-	5.35E-07	6.90E-01	4.84E-05	7445	
6	rs9378310	1671161	4.51E-02	GMDS	2	rs2890439	13375247	5.43E-01	N/A	-	5.35E-07	7.89E-02	8.98E-03	7446	
9	rs871495	89327436	1.97E-01	DAPK1	12	rs10082832	1.24E+08	4.76E-01	DHX37	-	5.35E-07	2.06E-01	1.75E-05	7447	
3	rs9311726	59761072	9.41E-01	FHIT	16	rs3011249	61206793	2.25E-02	N/A	-	5.35E-07	3.09E-01	7.71E-04	7448	
14	rs6572524	48440753	2.04E-01	N/A	15	rs2623255	50035348	1.42E-02	LEO1	-	5.35E-07	6.94E-01	3.31E-04	7449	
6	rs6597004	3773229	7.05E-02	N/A	3	rs11716029	1.72E+08	5.87E-01	TNIK	-	5.36E-07	3.17E-01	5.18E-06	7450	
6	rs7745656	32788948	1.70E-01	N/A	10	rs4245009	52247821	7.47E-01	A1CF	MHC	5.36E-07	9.79E-02	2.34E-03	7451	
2	rs12619674	81300586	6.24E-01	N/A	4	rs11132160	1.84E+08	9.03E-01	DCTD	-	5.36E-07	1.90E-01	2.01E-06	7452	
17	rs2604953	36869956	5.87E-01	KRT38	21	rs9984330	24880617	4.51E-01	N/A	-	5.36E-07	4.44E-01	1.45E-05	7453	
1	rs12058205	1.89E+08	6.51E-01	FAM5C	15	rs3743204	53577602	8.77E-02	DYX1C1	-	5.36E-07	8.24E-01	1.29E-04	7454	
9	rs1147377	1.31E+08	3.41E-01	N/A	18	rs4798681	8727474	2.97E-01	KIAA0802	-	5.36E-07	1.11E-01	2.91E-03	7455	
6	rs9376770	1.44E+08	7.64E-01	PHACTR2	13	rs7996510	45368378	7.15E-01	N/A	-	5.36E-07	5.86E-01	1.77E-05	7456	
6	rs7453084	8297003	5.04E-01	N/A	2	rs1861074	71083241	6.71E-01	ANKRD53	-	5.36E-07	2.62E-01	2.10E-03	7457	
8	rs7007530	38932203	3.32E-01	PLEKHA2	14	rs1176968	40503056	8.90E-01	N/A	-	5.36E-07	4.19E-02	7.07E-07	7458	
1	rs235922	1.7E+08	1.40E-01	MYOC	2	rs6759703	1.54E+08	7.80E-01	N/A	-	5.36E-07	8.35E-02	6.29E-03	7459	
6	rs442745	33490219	8.11E-01	CUTA	6	rs717120	1.36E+08	4.48E-01	AHI1	MHC	5.36E-07	3.30E-01	9.89E-04	7460	
6	rs559087	1.46E+08	7.16E-02	N/A	1	rs1288479	53551492	3.28E-01	LRP8	-	5.36E-07	7.46E-01	3.30E-05	7461	
3	rs4676593	38817916	9.69E-01	SCN10A	14	rs10140652	19761728	6.10E-01	OR11H6	-	5.37E-07	2.65E-02	3.99E-03	7462	
7	rs6462540	34320990	9.11E-01	N/A	13	rs693039	53944792	9.51E-01	N/A	-	5.37E-07	2.47E-01	1.55E-03	7463	
2	rs2396327	2.27E+08	9.04E-01	N/A	10	rs17100087	84203231	2.62E-01	NRG3	-	5.37E-07	2.23E-01	1.01E-05	7464	
3	rs9874792	55835566	2.37E-01	ERC2	11	rs2345345	6534153	1.45E-02	DNHD1	-	5.37E-07	3.73E-01	2.48E-03	7465	
6	rs2524123	31373293	7.02E-01	N/A	6	rs9266825	31490861	3.44E-01	MICA	MHC	5.37E-07	5.26E-01	2.11E-05	7466	
1	rs9428015	89041928	5.42E-01	PKN2	16	rs7187327	72734213	8.14E-02	N/A	-	5.37E-07	3.83E-01	1.56E-05	7467	
6	rs4896611	1.43E+08	1.20E-01	N/A	4	rs1391010	76300577	9.83E-01	N/A	-	5.37E-07	6.31E-01	1.61E-05	7468	
3	rs13061150	1.15E+08	7.50E-01	ATP6V1A	12	rs4762758	20505926	3.12E-01	PDE3A	-	5.37E-07	4.95E-01	9.54E-04	7469	
5	rs2973737	1.78E+08	2.89E-01	COL23A1	21	rs2850080	36510920	7.17E-01	DOPEY2	-	5.37E-07	4.39E-01	4.72E-04	7470	
2	rs4832131	85137224	7.91E-01	KCMF1	4	rs4235294	21830023	2.24E-02	N/A	-	5.37E-07	6.25E-01	5.70E-05	7471	
2	rs13033411	1.5E+08	3.27E-01	LYPD6B	13	rs4911004	84802577	2.78E-01	N/A	-	5.37E-07	1.62E-01	1.41E-06	7472	
5	rs1508869	1.15E+08	2.81E-02	LVRN	8	rs7834992	1.2E+08	8.57E-01	SAMD12	-	5.37E-07	2.57E-01	2.10E-06	7473	
15	rs8041357	72656491	1.21E-01	ARID3B	18	rs9963622	9718453	5.26E-01	RAB31	-	5.38E-07	5.76E-01	4.86E-04	7474	
11	rs790343	83282554	3.77E-02	DLG2	18	rs633734	63690918	6.74E-01	N/A	-	5.38E-07	6.40E-01	6.19E-06	7475	
1	rs2996016	41633970	2.03E-01	N/A	12	rs7133845	93507755	6.13E-01	TMCC3	-	5.38E-07	7.73E-01	9.50E-05	7476	
6	rs10945513	1.69E+08	7.23E-01	SMOC2	16	rs818380	63985635	8.93E-01	LOC283867	-	5.38E-07	5.86E-02	4.54E-07	7477	
5	rs4246818	1.78E+08	7.55E-01	COL23A1	21	rs2156411	36502677	5.73E-01	DOPEY2	-	5.38E-07	1.58E-01	1.96E-06	7478	
6	rs942723	1.65E+08	8.26E-01	N/A	2	rs12618269	34017690	2.17E-01	N/A	-	5.38E-07	8.40E-01	3.37E-05	7479	
14	rs12587506	57638555	4.02E-01	C14orf37	18	rs9951013	74048791	9.24E-01	N/A	-	5.38E-07	9.36E-01	5.44E-05	7480	
10	rs11193960	84114305	2.14E-01	NRG3	13	rs7981707	90765165	4.04E-01	N/A	-	5.38E-07	6.27E-01	2.82E-04	7481	
2	rs6568	1.27E+08	8.12E-01	GYPC	8	rs11136028	27959504	8.51E-02	C8orf80	-	5.38E-07	7.36E-01	1.75E-04	7482	
10	rs12573002	71212324	5.93E-01	COL13A1	22	rs5770953	49409804	9.14E-01	ARSA	-	5.38E-07	N/A	N/A	7483	
1	rs2133123	2.15E+08	4.16E-01	ESRRG	16	rs274542	75980097	4.51E-01	ADAMTS18	-	5.38E-07	3.39E-01	1.54E-03	7484	
14	rs10047954	36097797	5.90E-01	N/A	15	rs7359253	54901363	4.30E-01	N/A	-	5.38E-07	3.66E-01	9.68E-06	7485	
2	rs2118844	1.35E+08	5.89E-01	MGAT5	2	rs7570933	1.48E+08	6.16E-01	N/A	-	5.38E-07	1.19E-01	4.17E-03	7486	
8	rs1376842	83055073	6.14E-01	N/A	12	rs12227156	45295695	6.25E-02	N/A	-	5.39E-07	3.24E-02	4.07E-07	7487	
6	rs4870302	1.5E+08	8.38E-01	PPP1R14C	9	rs10780770	88373314	7.97E-01	N/A	-	5.39E-07	2.15E-01	1.60E-03	7488	
15	rs12909171	97796941	3.15E-01	N/A	17	rs4795800	28871806	2.64E-01	ACCN1	-	5.39E-07	7.63E-02	2.08E-07	7489	
7	rs6467148	1.27E+08	2.07E-01	SND1	17	rs2453589	19429443	1.12E-01	SLC47A1	-	5.39E-07	4.01E-01	9.50E-04	7490	
5	rs3822476	77749577	4.95E-01	SCAMP1	11	rs7126049	73061030	5.43E-01	RAB6A	-	5.39E-07	7.33E-01	2.64E-04	7491	
3	rs6441419	1.63E+08	6.08E-01	N/A	7	rs12674179	43179131	4.12E-01	HECW1	-	5.39E-07	N/A	N/A	7492	
4	rs767513	1.56E+08	2.40E-01	N/A	12	rs2141859	1.03E+08	2.12E-01	N/A	-	5.39E-07	3.93E-02	3.57E-07	7493	
6	rs9483751	1.35E+08	5.43E-01	N/A	2	rs722087	3883857	9.67E-01	N/A	-	5.39E-07	3.94E-01	5.68E-06	7494	
4	rs4699434	96725634	8.68E-01	N/A	5	rs10942498	86576064	1.58E-01	N/A	-	5.39E-07	4.03E-01	3.11E-05	7495	
6	rs4713646	33714501	7.65E-01	ITPR3	3	rs1371901	1.67E+08	1.42E-01	N/A	MHC	5.39E-07	6.43E-01	6.55E-04	7496	
1	rs2673962	2.33E+08	9.95E-01	N/A	3	rs9857101	88573932	9.77E-01	N/A	-	5.39E-07	6.89E-01	2.96E-04	7497	
12	rs11608740	2483590	4.39E-01	CACN/A1C	16	rs10852694	8004159	5.38E-02	N/A	-	5.39E-07	3.36E-01	3.12E-03	7498	
12	rs11608740	2483590	4.39E-01	CACN/A1C	16	rs9934058	8004042	5.38E-02	N/A	-	5.39E-07	3.36E-01	3.12E-03	7499	
6	rs9342268	91991774	1.86E-01	N/A	2	rs10166760	1.74E+08	7.22E-02	N/A	-	5.39E-07	5.75E-02	1.34E-07	7500	
5	rs2973737	1.78E+08	2.89E-01	COL23A1	21	rs2247051	36499456	7.18E-01	DOPEY2	-	5.39E-07	3.40E-01	7.20E-04	7501	
2	rs6731334	2.37E+08	5.08E-01	IQCA1	9	rs10818702	1.24E+08	2.70E-01	OR1J1	-	5.39E-07	7.54E-01	1.38E-04	7502	

SNP A					SNP B						Interaction P			Ranking	Cluster in top 100 interactions
CHR	SNP	Location	gle locus P va	Gene	CHR	SNP	Location	Single locus P valu	Gene	HC region	Stage 1	Stage 2	Combined		
2	rs1531968	2.4E+08	4.33E-01	N/A	4	rs6531358	35032724	4.82E-01	N/A	-	5.39E-07	1.27E-02	2.09E-08	7503	
2	rs1015230	6269313	1.46E-01	N/A	19	rs722571	12672379	7.95E-01	DHPS	-	5.40E-07	1.54E-02	3.28E-08	7504	
1	rs7528002	2.45E+08	2.33E-02	SCCPDH	20	rs2424239	19560584	2.37E-01	SLC24A3	-	5.40E-07	7.38E-01	1.24E-04	7505	
7	rs2969071	2479968	1.70E-01	N/A	9	rs1634352	80503640	1.86E-01	N/A	-	5.40E-07	8.17E-01	1.25E-04	7506	
1	rs4844686	2.07E+08	1.37E-02	N/A	12	rs2304818	39602648	8.58E-02	CNTN1	-	5.40E-07	8.79E-01	1.68E-04	7507	
1	rs10157528	2.32E+08	3.43E-01	SLC35F3	5	s16872154	73991036	8.31E-01	ENC1	-	5.40E-07	1.69E-01	1.51E-03	7508	
4	rs1404372	57898362	5.00E-01	N/A	5	rs2973522	11128691	7.21E-01	CTNND2	-	5.40E-07	6.41E-01	1.23E-04	7509	
6	rs4437459	24964302	7.51E-01	FAM65B	11	rs4540821	35914185	1.00E-01	LDLRAD3	-	5.40E-07	4.51E-01	9.71E-04	7510	
1	rs12036568	2.12E+08	2.79E-01	N/A	4	rs7669821	39198618	5.99E-01	N/A	-	5.40E-07	4.68E-01	3.75E-06	7511	
2	rs11679592	2385495	7.48E-01	N/A	8	s17149871	9242040	7.31E-01	N/A	-	5.40E-07	5.35E-01	4.06E-04	7512	
2	rs4971336	1017830	6.05E-01	SNTG2	5	rs1692395	71389241	8.04E-01	N/A	-	5.40E-07	7.29E-01	5.38E-04	7513	
4	rs1394272	1.48E+08	5.59E-01	TTC29	15	rs8033802	76286842	2.87E-01	ACSBG1	-	5.40E-07	3.01E-01	1.76E-05	7514	
6	rs9398296	1.12E+08	6.12E-01	WISP3	22	rs5751997	23517544	1.79E-01	PIWIL3	-	5.40E-07	5.48E-01	5.31E-04	7515	
2	rs11893319	1.97E+08	1.77E-01	HECW2	3	s16828950	1.75E+08	6.72E-01	NLGN1	-	5.40E-07	7.35E-01	2.97E-04	7516	
2	rs16834182	1.93E+08	8.92E-01	TMEFF2	4	rs997572	35725168	4.99E-01	ARAP2	-	5.40E-07	7.55E-01	2.23E-04	7517	
9	rs1874105	14842141	6.85E-01	FREM1	14	s10136168	61720094	1.24E-01	N/A	-	5.40E-07	7.79E-01	1.48E-04	7518	
7	rs6978845	1.54E+08	9.80E-01	DPP6	22	rs132405	43509092	1.47E-01	PRR5	-	5.40E-07	N/A	N/A	7519	
3	rs16854433	1.1E+08	8.92E-01	HHLA2	10	rs869963	87285190	3.38E-01	N/A	-	5.41E-07	1.48E-01	3.03E-03	7520	
5	rs3112410	2568875	9.66E-01	N/A	8	rs7824696	53389772	4.34E-01	ST18	-	5.41E-07	2.31E-01	1.66E-03	7521	
1	rs1109223	2.22E+08	3.26E-01	CAPN8	4	rs1980360	83913307	9.20E-01	SCD5	-	5.41E-07	4.55E-01	1.47E-05	7522	
2	rs7556765	41672351	5.27E-01	N/A	17	s11078877	10860250	8.61E-01	N/A	-	5.41E-07	8.67E-01	8.44E-05	7523	
10	rs1881735	49246884	3.65E-01	N/A	20	rs6063141	45779866	4.18E-01	SULF2	-	5.41E-07	2.86E-02	2.89E-07	7524	
4	rs6850999	80720962	6.97E-02	N/A	7	rs16480	24277594	2.38E-01	NPY	-	5.41E-07	3.08E-01	1.46E-05	7525	
1	rs10916042	2.25E+08	1.61E-01	N/A	12	rs1488643	94140216	7.65E-01	FGD6	-	5.41E-07	3.64E-01	2.43E-05	7526	
3	rs9869100	77494923	4.96E-01	ROBO2	11	rs2239695	1.18E+08	6.63E-01	CD3D	-	5.41E-07	5.92E-01	3.58E-04	7527	
4	rs2063822	70988012	8.06E-01	CSN1S2A	10	rs2619111	1.19E+08	6.03E-01	KCNK18	-	5.41E-07	7.51E-01	9.41E-06	7528	
9	rs17771589	24248863	8.18E-01	N/A	12	s11051659	31945102	6.68E-01	N/A	-	5.41E-07	N/A	N/A	7529	
1	rs4147592	1.64E+08	8.02E-01	MGST3	2	s10197841	1.46E+08	4.89E-01	N/A	-	5.41E-07	2.47E-01	9.98E-04	7530	
1	rs4147592	1.64E+08	8.02E-01	MGST3	2	s13426482	1.46E+08	4.89E-01	N/A	-	5.41E-07	2.47E-01	9.98E-04	7531	
5	rs12697730	82944230	4.26E-01	N/A	19	rs916694	40384243	9.02E-01	N/A	-	5.41E-07	3.73E-01	1.05E-05	7532	
7	rs4723037	30966625	1.87E-01	GHRHR	15	rs4601989	65239008	4.20E-01	SMAD3	-	5.41E-07	6.69E-01	1.97E-04	7533	
5	rs2607148	82947341	4.26E-01	N/A	19	rs916694	40384243	9.02E-01	N/A	-	5.41E-07	N/A	N/A	7534	
3	rs962268	28182767	8.92E-01	N/A	3	rs6809777	30647366	6.94E-01	TGFBR2	-	5.41E-07	9.49E-01	2.06E-05	7535	
2	rs10191556	2.42E+08	2.87E-01	THAP4	4	rs6847598	76969380	6.04E-01	USO1	-	5.41E-07	1.53E-02	1.92E-07	7536	
6	rs4715646	57008299	4.50E-01	BEND6	2	rs280210	1.22E+08	8.01E-01	N/A	-	5.41E-07	4.83E-01	8.52E-06	7537	
11	rs728341	1.33E+08	7.86E-01	OPCML	16	rs4786964	7085568	4.11E-03	A2BP1	-	5.42E-07	3.66E-01	8.77E-06	7538	
15	rs11858872	55831992	7.86E-01	N/A	20	s10485751	11927195	8.28E-01	N/A	-	5.42E-07	8.81E-01	8.26E-05	7539	
6	rs13197916	5872558	8.81E-01	N/A	2	rs4668879	15221205	8.99E-01	NBAS	-	5.42E-07	2.36E-01	3.29E-03	7540	
11	rs11026661	3328263	3.84E-02	ZNF195	13	rs1571069	1.02E+08	6.24E-01	BIVM	-	5.42E-07	2.28E-01	1.07E-03	7541	
13	rs4584703	84163820	5.63E-01	N/A	16	rs223881	55944067	4.22E-01	CX3CL1	-	5.42E-07	7.61E-01	1.25E-04	7542	
5	rs3892476	1.33E+08	7.52E-01	N/A	7	rs856589	46700832	1.76E-01	N/A	-	5.42E-07	8.58E-01	1.32E-04	7543	
1	rs857123	56978254	9.57E-01	C1orf168	17	rs2214448	29418554	1.58E-01	ACCN1	-	5.42E-07	9.49E-01	7.09E-05	7544	
6	rs6929192	75691542	3.17E-01	N/A	14	rs712285	33696953	8.59E-01	N/A	-	5.42E-07	N/A	N/A	7545	
3	rs7431530	29357545	3.66E-01	N/A	22	rs9622429	35331441	5.96E-01	CACNG2	-	5.42E-07	8.59E-01	3.14E-04	7546	
8	rs16915724	94263226	8.31E-01	N/A	22	rs2190742	15634399	9.23E-01	XKR3	-	5.42E-07	N/A	N/A	7547	
1	rs2992736	18670290	9.82E-01	KLHDC7A	2	rs2887087	33548577	8.64E-01	RASGRP3	-	5.42E-07	3.08E-01	2.82E-06	7548	
3	rs812124	30750967	9.07E-01	GADL1	3	rs6769495	1.75E+08	2.10E-03	NLGN1	-	5.42E-07	3.24E-01	8.95E-04	7549	
3	rs9866032	8526112	4.30E-01	N/A	14	s11158959	24439995	9.41E-01	STXBP6	-	5.42E-07	4.96E-01	2.74E-04	7550	
7	rs4947618	51599655	4.53E-01	N/A	10	s12414926	15714011	3.40E-01	ITGA8	-	5.42E-07	6.16E-01	2.10E-05	7551	
7	rs4947617	51596856	4.53E-01	N/A	10	s12414926	15714011	3.40E-01	ITGA8	-	5.42E-07	6.70E-01	2.55E-05	7552	
6	rs9490225	1.22E+08	2.46E-01	N/A	3	rs524431	74383584	3.74E-01	CNTN3	-	5.43E-07	4.98E-02	6.03E-07	7553	
2	rs13407493	1.02E+08	5.04E-01	N/A	2	s16850533	2.35E+08	5.53E-01	N/A	-	5.43E-07	6.77E-01	5.01E-04	7554	
9	rs10982461	1.17E+08	3.08E-01	TNFSF8	13	rs1334825	68827182	6.82E-01	N/A	-	5.43E-07	8.65E-01	4.45E-05	7555	
1	rs7544003	31461675	6.64E-02	NKAIN1	14	s10130620	28940257	7.75E-02	N/A	-	5.43E-07	N/A	N/A	7556	
2	rs12612899	52509436	7.11E-03	N/A	14	s11159103	74341107	9.84E-01	YLPM1	-	5.43E-07	3.50E-01	1.76E-05	7557	
6	rs3823055	1.61E+08	9.03E-01	PLG	1	rs2453200	2.42E+08	5.37E-01	N/A	-	5.43E-07	3.54E-01	3.37E-03	7558	
3	rs12491696	21889150	6.98E-01	N/A	19	s12977050	35520535	8.54E-01	N/A	-	5.43E-07	1.81E-02	4.49E-02	7559	
6	rs4947296	31166157	4.31E-02	N/A	6	rs3130534	31317024	3.50E-01	N/A	MHC	5.43E-07	1.71E-01	2.85E-06	7560	
2	rs4662670	1.3E+08	8.36E-01	LOC389033	13	rs3742207	1.1E+08	3.69E-01	COL4A1	-	5.43E-07	3.54E-01	5.40E-06	7561	
2	rs10192881	79415518	6.27E-01	N/A	11	rs6590919	1.01E+08	4.91E-01	N/A	-	5.43E-07	6.60E-01	7.06E-05	7562	
5	rs11745906	83610946	6.03E-01	EDIL3	20	rs6110407	14657451	5.12E-01	MACROD2	-	5.43E-07	1.33E-01	4.06E-03	7563	
6	rs9363764	68288763	7.99E-01	N/A	4	rs6843121	1.13E+08	7.67E-01	N/A	-	5.43E-07	1.35E-01	1.30E-06	7564	
6	rs4713420	31101546	1.78E-02	N/A	6	rs3095250	31316319	6.90E-02	N/A	MHC	5.43E-07	9.18E-01	4.91E-04	7565	
4	rs10520473	1.82E+08	5.10E-01	N/A	13	rs9594808	42242401	8.39E-01	C13orf30	-	5.43E-07	1.25E-01	5.73E-03	7566	
12	rs11107847	93910922	2.10E-01	NDUFA12	15	rs10518996	57164738	4.83E-01	RNF111	-	5.43E-07	5.62E-01	2.26E-04	7567	
4	rs6835572	1.82E+08	5.19E-01	N/A	17	s11650777	65856078	3.76E-01	N/A	-	5.43E-07	6.89E-01	4.62E-05	7568	
1	rs2184345	2.31E+08	3.18E-01	N/A	7	rs7797216	53125393	2.18E-01	N/A	-	5.44E-07	3.88E-01	8.75E-06	7569	
12	rs7959314	23962261	3.69E-01	SOX5	22	rs112544	19676485	1.08E-01	MGC16703	-	5.44E-07	4.11E-01	8.23E-05	7570	
7	rs4719162	70764626	3.97E-01	WBSCR17	12	rs952367	77167621	8.74E-01	N/A	-	5.44E-07	4.19E-01	7.98E-06	7571	
6	rs10499303	1.56E+08	5.17E-01	TFB1M	3	rs2195914	1.29E+08	6.64E-01	KBTBD12	-	5.44E-07	8.56E-01	8.78E-05	7572	
6	rs6917887	1.3E+08	3.98E-01	ARHGAP18	20	rs6040993	11880255	9.18E-01	N/A	-	5.44E-07	9.15E-01	2.05E-04	7573	
6	rs6917887	1.3E+08	3.98E-01	ARHGAP18	20	rs6078404	11877122	9.18E-01	N/A	-	5.44E-07	9.61E-01	1.42E-04	7574	
2	rs1430170	1.34E+08	6.74E-03	NCKAP5	12	rs1401982	88513730	1.71E-01	ATP2B1	-	5.44E-07	3.77E-01	3.46E-06	7575	
8	rs7003953	21660734	6.22E-02	GFRA2	14	rs2371462	80490527	5.39E-01	TSHR	-	5.44E-07	5.11E-01	3.37E-04	7576	
8	rs3015779	36967630	7.76E-01	N/A	11	rs481168	1.23E+08	9.92E-01	ZNF202	-	5.44E-07	8.14E-01	2.39E-04	7577	
1	rs12049279	87826079	8.23E-01	N/A	5	rs2368554	97822610	1.72E-01	N/A	-	5.44E-07	1.13E-01	8.65E-07	7578	
13	rs327130	28419831	9.93E-01	N/A	16	s11862527	24679648	6.29E-01	TNRC6A	-	5.44E-07	1.50E-01	2.75E-03	7579	
13	rs327130	28419831	9.93E-01	N/A	16	s12598296	24684011	6.29E-01	TNRC6A	-	5.44E-07	1.50E-01	2.75E-03	7580	
11	rs790343	83282554	3.77E-02	DLG2	18	rs8084950	63688332	6.73E-01	N/A	-	5.44E-07	7.33E-01	8.43E-06	7581	
3	rs2200392	1.57E+08	1.72E-01	N/A	3	rs1002993	1.91E+08	1.38E-01	LEPREL1	-	5.44E-07	7.72E-01	8.67E-05	7582	
1	rs4846689	2.19E+08	3.35E-01	N/A	2	rs2600726	2.37E+08	1.46E-01	N/A	-	5.44E-07	8.83E-01	3.01E-05	7583	
1	rs1123250	1.1E+08	6.30E-01	SLC6A17	2	rs243021	60438323	8.73E-01	N/A	-	5.44E-07	4.50E-01	1.40E-05	7584	
4	rs17023944	96598204	3.69E-01	UNC5C	16	rs3751832	77771773	4.68E-01	WWOX	-	5.44E-07	5.82E-01	3.66E-04	7585	
3	rs11918985	1.95E+08	4.33E-01	N/A	5	rs6449720	63685866	2.77E-01	RNF180	-	5.44E-07	8.50E-01	1.54E-04	7586	
8	rs3098233	1.04E+08	3.71E-01	CTHRC1	20	rs6063397	47449616	1.99E-01	KCNB1	-	5.45E-07	7.51E-02	8.68E-04	7587	
12	rs10902507	1.31E+08	5.86E-01	GALNT9	17	rs8069412	33104144	5.22E-01	HNF1B	-	5.45E-07	2.67E-01	6.16E-06	7588	
6	rs926774	47229234	8.43E-01	N/A	5	rs9327220	1.21E+08	7.94E-01	N/A	-	5.45E-07	4.77E-01	4.48E-04	7589	
18	rs9652993	32292147	6.10E-01	FHOD3	19	rs919271	57360226	2.20E-01	ZNF836	-	5.45E-07	8.26E-01	8.43E-05	7590	
3	rs875581	1.58E+08	4.49E-01	KCN/AB1	12	rs7485772	1.3E+08	7.50E-01	N/A	-	5.45E-07	1.49E-01	2.27E-03	7591	
3	rs9830035	1.73E+08	6.63E-01	PLD1	13	rs1230455	45702138	5.90E-01	N/A	-	5.45E-07	3.65E-01	1.99E-06	7592	
15	rs1665050	57080897	6.37E-01	RNF111	15	rs2060060	84701996	9.27E-01	AGBL1	-	5.45E-07	9.01E-01	3.63E-05	7593	
3	rs2344816	88772146	4.35E-02	N/A	11	rs9971402	92396199	6.89E-01	N/A	-	5.45E-07	1.22E-01	3.04E-06	7594	
3	rs1495838	88758266	4.35E-02	N/A	11	rs9971402	92396199	6.89E-01	N/A	-	5.45E-07	1.48E-01	4.04E-06	7595	
4	rs13130336	8443721	3.36E-01	ACOX3	17	s17836676	68630211	8.76E-01	N/A	-	5.45E-07	3.65E-01	1.16E-03	7596	
5	rs7720237	1.09E+08	1.14E-01	N/A	10	s17454012	11377324	1.32E-01	CUGBP2	-	5.45E-07	9.61E-01	2.65E-04	7597	
2	rs1368233	1894304	8.39E-01	MYT1L	13	rs1992744	68657677	3.99E-01	N/A	-	5.45E-07	9.87E-01	6.27E-05	7598	
14	rs12587506	57638555	4.02E-01	C14orf37	18	rs1039610	74049795	9.22E-01	N/A	-	5.45E-07	9.33E-01	5.44E-05	7599	
5	rs830230	1.65E+08	6.59E-01	N/A	13	s10507466	37361657	7.50E-01	TRPC4	-	5.45E-07	1.31E-01	2.07E-06	7600	
1	rs226081	44619079	7.76E-01	N/A	3	rs9882054	1.69E+08	3.19E-01	N/A	-	5.45E-07	3.94E-01	1.52E-05	7601	
1	rs2154229	2.07E+08	3.21E-02	N/A	5	rs4631227	1.33E+08	5.10E-02	N/A	-	5.45E-07	8.53E-01	2.55E-04	7602	
2	rs1513887	1.77E+08	9.61E-01	N/A	4	s17050362	1.4E+08	7.81E-01	N/A	-	5.46E-07	3.43E-01	4.21E-06	7603	
2	rs1513887	1.77E+08	9.61E-01	N/A	4	rs3919615	1.4E+08	7.81E-01	N/A	-	5.46E-07	3.62E-01	4.79E-06	7604	
6	rs1342740	16132379	4.44E-01	N/A	2	rs685211	67532362	3.77E-01	N/A	-	5.46E-07	N/A	N/A	7605	
4	rs17040984	1.63E+08	9.50E-02	FSTL5	8	rs7357578	18186633	5.75E-01	N/A	-	5.46E-07	6.30E-01	9.16E-06	7606	
19	rs278184	36108379	1.34E-01	N/A	20	rs6134656	1274001	2.07E-01	SDCBP2	-	5.46E-07	9.53E-01	2.49E-04	7607	
9	rs2185414	1.07E+08	8.76E-01	N/A	10	s11819138	10219789	1.34E-01	N/A	-	5.46E-07	2.67E-01	4.28E-06	7608	
2	rs17042099	602629	3.63E-02	N/A	19	rs7188	11136139	8.51E-01	SPC24	-	5.46E-07	2.88E-01	2.44E-04	7609	
6	rs4716289	18690865	6.33E-01	MIR548A1	11	s10839822	7598983	1.17E-01	PPFIBP2	-	5.46E-07	2.60E-01	4.26E-06	7610	
12	rs10846824	1.24E+08	6.74E-01	AACS	14	rs883519	53322290	7.94E-01	N/A	-	5.46E-07	8.60E-01	4.66E-05	7611	
14	rs1885189	88457081	5.47E-01	N/A	16	rs8044561	70108642	8.90E-02	CHST4	-	5.46E-07	4.91E-03	1.79E-02	7612	
1	rs1287634	5847958	3.75E-01	NPHP4	12	rs7300043	3828974	1.06E-01	PARP11	-	5.46E-07	6.00E-01	2.22E-05	7613	
10	rs7087652	24760104	1.40E-01	KIAA1217	16	rs998109	68673020	8.44E-01	PDXDC2	-	5.46E-07	9.24E-01	5.67E-05	7614	

SNP A					SNP B						Interaction P			Ranking	Cluster in top 100 interactions
CHR	SNP	Location	gle locus P va	Gene	CHR	SNP	Location	Single locus P valu	Gene	HC region	Stage 1	Stage 2	Combined		
1	rs1157688	4536627	6.86E-01	N/A	1	rs12093992	2.38E+08	2.66E-01	N/A	-	5.46E-07	9.54E-01	1.06E-04	7615	
16	rs7500355	84634601	7.85E-01	N/A	17	rs7213544	22034311	9.87E-01	N/A	-	5.46E-07	2.61E-01	1.12E-03	7616	
5	rs750063	1.54E+08	3.82E-01	GALNT10	17	rs8081781	14385239	5.30E-01	N/A	-	5.46E-07	5.74E-01	6.26E-05	7617	
1	rs270765	44796002	7.32E-01	RNF220	8	rs1615810	5555216	4.96E-01	N/A	-	5.46E-07	2.75E-01	3.49E-06	7618	
2	rs1843758	1.73E+08	8.06E-01	N/A	4	rs10805125	44005235	8.84E-01	KCTD8	-	5.46E-07	4.26E-01	1.13E-03	7619	
3	rs3851997	1.33E+08	2.94E-01	N/A	18	rs4796915	11218713	7.61E-01	N/A	-	5.46E-07	7.09E-01	1.38E-04	7620	
6	rs1265156	31250276	6.76E-01	TCF19	6	rs9394047	31344229	1.23E-01	HLA-C	MHC	5.47E-07	3.00E-01	4.09E-06	7621	
5	rs4703537	81516127	7.28E-01	ATG10	5	rs11749320	86161696	5.93E-01	N/A	-	5.47E-07	8.05E-01	1.67E-05	7622	
1	rs7514610	1.56E+08	9.24E-01	N/A	10	rs10752261	12370719	4.42E-01	N/A	-	5.47E-07	3.93E-01	1.13E-03	7623	
2	rs12477036	2.3E+08	4.94E-01	PID1	3	rs1352409	3785723	1.45E-01	N/A	-	5.47E-07	8.99E-03	5.04E-08	7624	
6	rs602414	1.38E+08	4.34E-01	N/A	12	rs10772757	14295548	4.15E-02	N/A	-	5.47E-07	4.99E-01	9.61E-06	7625	
4	rs4697055	23876434	1.38E-01	N/A	5	rs10474733	2057160	7.20E-02	N/A	-	5.47E-07	5.13E-01	1.14E-05	7626	
10	rs6585278	1.16E+08	8.57E-01	ABLIM1	19	rs1035937	38830785	7.88E-01	CHST8	-	5.47E-07	9.16E-01	5.01E-05	7627	
14	rs989909	74157997	7.17E-01	LTBP2	22	rs2240432	29851404	5.25E-01	INPP5J	-	5.47E-07	2.05E-01	3.65E-06	7628	
9	rs4979054	1.14E+08	3.99E-01	N/A	11	rs531767	78337603	1.73E-01	ODZ4	-	5.47E-07	4.72E-01	5.44E-05	7629	
6	rs3778084	1.52E+08	1.73E-01	ESR1	3	rs2568911	1.96E+08	3.66E-01	N/A	-	5.47E-07	N/A	N/A	7630	
4	rs2231142	89271347	7.83E-01	ABCG2	9	rs10819305	1.3E+08	6.61E-01	SH2D3C	-	5.47E-07	5.68E-01	1.22E-04	7631	
5	rs306107	1.11E+08	5.85E-01	CAMK4	9	rs7035592	27224029	1.27E-01	NCRN/A00032	-	5.47E-07	6.00E-01	1.43E-04	7632	
4	rs2131466	28426304	8.00E-01	N/A	9	rs6474732	12805091	2.53E-01	C9orf150	-	5.47E-07	7.52E-01	4.56E-05	7633	
2	rs6725806	1.03E+08	4.53E-01	SLC9A2	3	rs1813256	1.47E+08	5.56E-01	N/A	-	5.47E-07	4.20E-02	3.33E-07	7634	
11	rs17214070	4347807	2.77E-01	OR52B4	12	rs7955684	1.28E+08	8.86E-02	TMEM132D	-	5.47E-07	3.30E-01	2.10E-03	7635	
5	rs564195	1.78E+08	3.20E-01	COL23A1	10	rs7914626	72151924	7.56E-01	ADAMTS14	-	5.47E-07	3.60E-01	2.38E-03	7636	
11	rs11224280	96358989	4.47E-01	N/A	13	rs9542922	71826381	6.18E-01	N/A	-	5.47E-07	9.69E-01	3.98E-05	7637	
7	rs17166689	13068758	6.44E-02	N/A	19	rs4801457	62485375	9.79E-01	ZNF460	-	5.47E-07	N/A	N/A	7638	
6	rs1419638	29463498	1.74E-01	OR12D3	2	rs9287631	2.39E+08	6.36E-01	N/A	MHC	5.47E-07	5.24E-01	2.32E-05	7639	
1	rs585075	18893960	6.59E-01	PAX7	13	rs4885913	55035424	5.13E-01	N/A	-	5.48E-07	9.19E-02	4.28E-07	7640	
15	rs4886456	73802922	9.11E-01	ODF3L1	18	rs1942405	67482471	1.27E-01	N/A	-	5.48E-07	1.27E-01	1.31E-06	7641	
4	rs2313565	1.62E+08	9.47E-01	N/A	9	rs870409	13977094	9.46E-01	N/A	-	5.48E-07	9.14E-01	1.72E-04	7642	
10	rs7918519	20218037	5.41E-01	PLXDC2	15	rs16964798	35283503	3.05E-01	N/A	-	5.48E-07	5.60E-01	9.13E-04	7643	
12	rs10771301	26819693	2.20E-01	ITPR2	18	rs1991621	69620005	8.81E-01	N/A	-	5.48E-07	N/A	N/A	7644	
16	rs8056267	63850483	5.36E-01	N/A	20	rs761262	15726775	7.52E-02	MACROD2	-	5.48E-07	1.33E-01	2.84E-06	7645	
12	rs11107828	93889967	1.48E-01	NDUFA12	20	rs4810539	44757415	6.23E-01	SLC2A10	-	5.48E-07	6.17E-01	1.61E-05	7646	
2	rs1378428	78535975	9.45E-01	N/A	9	rs871807	1.16E+08	9.50E-01	ZNF618	-	5.48E-07	4.19E-01	5.03E-04	7647	
7	rs4283980	1.04E+08	1.62E-01	LHFPL3	16	rs8055043	80537939	8.22E-02	PLCG2	-	5.48E-07	7.63E-01	4.25E-05	7648	
8	rs7461956	6760323	1.09E-01	DEFA6	10	rs3843023	92781365	3.20E-01	N/A	-	5.48E-07	5.56E-02	2.29E-02	7649	
6	rs174402	1.12E+08	7.12E-01	N/A	6	rs197681	1.23E+08	8.34E-01	PKIB	-	5.48E-07	8.66E-01	1.53E-04	7650	
4	rs4312793	44119244	1.92E-01	KCTD8	13	rs9520146	1.06E+08	7.81E-01	N/A	-	5.48E-07	6.73E-01	2.27E-05	7651	
3	rs1914812	1.18E+08	4.42E-01	N/A	9	rs2282683	78307571	6.05E-01	GCNT1	-	5.48E-07	3.18E-01	7.25E-06	7652	
3	rs1914812	1.18E+08	4.42E-01	N/A	9	rs707739	78308723	6.05E-01	GCNT1	-	5.48E-07	3.18E-01	7.25E-06	7653	
13	rs2164172	1.09E+08	4.20E-01	N/A	18	rs7244352	32192058	6.74E-01	FHOD3	-	5.49E-07	2.46E-01	8.71E-07	7654	
6	rs1790022	1.62E+08	2.17E-01	PARK2	9	rs927319	4146304	6.61E-01	GLIS3	-	5.49E-07	3.85E-01	1.14E-05	7655	
6	rs9398296	1.12E+08	6.12E-01	WISP3	3	rs9820339	1.51E+08	7.77E-01	WWTR1	-	5.49E-07	4.86E-01	2.33E-03	7656	
11	rs10833513	21323102	5.78E-01	NELL1	18	rs7243707	41486272	4.79E-01	SLC14A2	-	5.49E-07	6.76E-01	1.04E-03	7657	
3	rs17220622	46554129	2.14E-01	LRRC2	12	rs3817012	1.21E+08	1.89E-01	CLIP1	-	5.49E-07	8.05E-01	6.00E-04	7658	
1	rs11164665	1.03E+08	7.21E-01	N/A	12	rs697635	50569015	9.28E-01	ANKRD33	-	5.49E-07	6.72E-01	3.62E-04	7659	
2	rs12467286	1.96E+08	9.95E-01	N/A	11	rs542284	95696359	9.26E-01	MAML2	-	5.49E-07	9.01E-01	2.12E-04	7660	
3	rs9874470	1.17E+08	1.48E-01	LSAMP	8	rs6984840	10169975	9.13E-01	MSRA	-	5.49E-07	5.60E-01	3.73E-04	7661	
1	rs7418397	1.05E+08	2.50E-02	N/A	12	rs2671436	1E+08	4.99E-01	SLC5A8	-	5.49E-07	3.22E-01	1.25E-05	7662	
10	rs878259	1E+08	1.00E-01	LOXL4	14	rs10138087	65602555	1.31E-01	N/A	-	5.49E-07	3.27E-01	2.35E-05	7663	
1	rs6702660	4923290	9.64E-01	N/A	13	rs9576264	36886791	9.77E-01	N/A	-	5.49E-07	7.36E-03	3.33E-02	7664	
8	rs10504458	70769716	4.74E-01	SLCO5A1	20	rs402899	4368450	7.38E-01	N/A	-	5.49E-07	1.42E-01	8.96E-04	7665	
8	rs1543157	1.29E+08	5.43E-01	N/A	19	rs8112582	8148511	4.19E-01	N/A	-	5.50E-07	6.73E-01	9.67E-05	7666	
4	rs12641060	14868093	4.27E-02	N/A	16	rs1970817	10483325	8.64E-02	ATF7IP2	-	5.50E-07	8.21E-01	2.65E-05	7667	
1	rs1737617	10773385	8.47E-01	CASZ1	12	rs2072511	94905363	5.05E-01	AMDHD1	-	5.50E-07	3.41E-01	1.37E-05	7668	
10	rs6560807	2334268	1.96E-01	N/A	14	rs4906459	1.04E+08	8.88E-01	N/A	-	5.50E-07	3.62E-01	1.24E-05	7669	
2	rs1568766	1.3E+08	1.61E-01	N/A	7	rs515906	1.54E+08	1.75E-01	DPP6	-	5.50E-07	4.17E-01	1.91E-05	7670	
5	rs691360	18110132	6.01E-01	N/A	13	rs2067649	57660246	9.30E-01	N/A	-	5.50E-07	4.48E-02	1.00E-07	7671	
5	rs4868438	1.74E+08	1.72E-01	N/A	18	rs1436907	6374431	7.68E-01	L3MBTL4	-	5.50E-07	1.60E-01	2.04E-06	7672	
3	rs9835427	1.17E+08	4.15E-01	LSAMP	8	rs6984840	10169975	9.13E-01	MSRA	-	5.50E-07	6.78E-01	2.46E-04	7673	
5	rs751768	66809711	7.20E-02	N/A	9	rs7036602	1.04E+08	4.93E-01	N/A	-	5.50E-07	4.50E-01	1.85E-04	7674	
2	rs4953288	45979475	3.29E-01	PRKCE	8	rs11203945	18137238	7.24E-01	N/AT1	-	5.50E-07	7.06E-01	3.29E-05	7675	
1	rs1470438	1.02E+08	6.01E-01	N/A	5	rs1551050	16586551	9.95E-01	FAM134B	-	5.50E-07	7.40E-01	1.43E-04	7676	
16	rs11149981	75927639	7.93E-01	ADAMTS18	16	rs1035572	79924772	1.65E-01	GAN	-	5.50E-07	1.08E-01	1.27E-06	7677	
8	rs241182	28703345	3.09E-01	INTS9	12	rs7294478	7158081	7.76E-01	RBP5	-	5.50E-07	6.16E-02	5.06E-03	7678	
6	rs7741153	15064906	9.28E-01	N/A	2	rs7574768	1.41E+08	3.18E-01	N/A	-	5.50E-07	2.78E-01	7.36E-06	7679	
3	rs9815177	1.48E+08	7.47E-01	N/A	11	rs11826249	81030361	8.15E-01	N/A	-	5.50E-07	6.37E-01	2.28E-04	7680	
1	rs11119972	2.11E+08	7.34E-01	ATF3	1	rs1619856	2.33E+08	3.89E-01	TARBP1	-	5.50E-07	8.69E-01	2.54E-05	7681	
3	rs11719844	1.76E+08	8.09E-01	N/A	13	rs9506776	21518850	6.63E-01	N/A	-	5.51E-07	6.58E-01	1.96E-05	7682	
3	rs231998	1.74E+08	1.56E-01	N/A	18	rs4797283	7239740	2.25E-01	LRRC30	-	5.51E-07	9.51E-01	2.87E-05	7683	
3	rs9828937	1.78E+08	2.21E-01	N/A	13	rs1323682	1.07E+08	2.81E-01	FAM155A	-	5.51E-07	5.25E-01	4.03E-04	7684	
6	rs9480921	1.1E+08	6.83E-01	CCDC162	7	rs10245351	78062680	7.43E-01	MAGI2	-	5.51E-07	N/A	N/A	7685	
6	rs9350866	81477869	8.88E-01	N/A	8	rs2205263	1.17E+08	1.04E-01	N/A	-	5.51E-07	1.77E-01	1.31E-03	7686	
6	rs9350866	81477869	8.88E-01	N/A	8	rs2205262	1.17E+08	1.04E-01	N/A	-	5.51E-07	1.79E-01	1.30E-03	7687	
6	rs9350866	81477869	8.88E-01	N/A	8	rs2205264	1.17E+08	1.04E-01	N/A	-	5.51E-07	1.79E-01	1.30E-03	7688	
6	rs9350866	81477869	8.88E-01	N/A	8	rs2223057	1.17E+08	1.04E-01	N/A	-	5.51E-07	N/A	N/A	7689	
9	rs320266	1.04E+08	8.47E-01	N/A	20	rs6112923	20433588	1.45E-01	RALGAPA2	-	5.51E-07	9.24E-01	1.85E-04	7690	
5	rs12523655	1.7E+08	5.99E-01	KCNIP1	7	rs6965333	1.28E+08	5.74E-01	N/A	-	5.51E-07	N/A	N/A	7691	
1	rs1125952	1.96E+08	6.85E-01	N/A	7	rs194834	1.04E+08	9.34E-01	ORC5L	-	5.51E-07	6.55E-01	3.38E-04	7692	
6	rs6568622	1.1E+08	8.38E-01	GPR6	5	rs4385221	1.69E+08	8.39E-01	DOCK2	-	5.51E-07	2.22E-01	2.37E-03	7693	
10	rs215493	91154150	9.20E-01	IFIT1L	14	rs4899457	39410936	9.92E-01	N/A	-	5.51E-07	2.40E-01	2.30E-04	7694	
7	rs1012951	80774676	2.03E-01	N/A	10	rs10881895	92927999	4.38E-01	N/A	-	5.51E-07	7.11E-01	1.01E-05	7695	
16	rs9936415	77386046	9.30E-01	WWOX	17	rs1899650	32129054	1.65E-01	N/A	-	5.51E-07	8.35E-01	5.64E-05	7696	
5	rs2120349	1.13E+08	1.97E-01	MCC	12	rs11180712	74526998	1.45E-01	N/A	-	5.51E-07	9.88E-01	7.45E-04	7697	
14	rs10137893	68142631	8.12E-01	RAD51L1	15	rs10519772	31094034	6.74E-01	FMN1	-	5.51E-07	4.50E-02	1.45E-07	7698	
5	rs160766	3163148	7.66E-01	N/A	17	rs12600765	74837318	4.25E-01	HRNBP3	-	5.51E-07	1.56E-01	2.44E-06	7699	
1	rs1062827	1.59E+08	1.23E-01	F11R	18	rs8092575	68807530	4.64E-01	N/A	-	5.51E-07	5.86E-01	2.06E-04	7700	
1	rs11807930	1.59E+08	1.23E-01	F11R	18	rs8092575	68807530	4.64E-01	N/A	-	5.51E-07	5.86E-01	2.06E-04	7701	
1	rs2297221	1.59E+08	1.23E-01	F11R	18	rs8092575	68807530	4.64E-01	N/A	-	5.51E-07	5.86E-01	2.06E-04	7702	
12	rs1920947	1.14E+08	9.43E-01	N/A	22	rs451840	16696526	2.10E-01	MICAL3	-	5.51E-07	9.25E-01	1.09E-04	7703	
1	rs12046158	2881573	1.29E-01	N/A	20	rs11087739	6600824	8.99E-01	N/A	-	5.52E-07	8.42E-02	5.49E-07	7704	
7	rs6966644	19360045	3.98E-01	N/A	13	rs9564874	71564341	7.04E-02	N/A	-	5.52E-07	6.31E-01	3.44E-05	7705	
9	rs10810402	15576534	1.54E-01	C9orf93	13	rs7328574	60686962	6.80E-01	N/A	-	5.52E-07	9.21E-01	3.48E-05	7706	
2	rs2013613	19674758	3.25E-01	N/A	12	rs6486795	1.21E+08	7.72E-01	ORAI1	-	5.52E-07	9.87E-01	5.79E-05	7707	
4	rs2597786	17132046	8.10E-01	QDPR	11	rs12278912	1.24E+08	7.20E-01	NRGN	-	5.52E-07	8.64E-01	2.65E-04	7708	
1	rs1776148	2.4E+08	6.52E-01	EXO1	7	rs2189448	15614109	9.93E-01	MEOX2	-	5.52E-07	9.10E-01	2.55E-06	7709	
10	rs1006286	1.2E+08	5.74E-01	N/A	10	rs1999414	1.22E+08	3.28E-01	N/A	-	5.52E-07	7.57E-01	5.47E-04	7710	
5	rs860439	3637368	1.81E-01	IRX1	9	rs10992420	94490629	2.81E-01	IPPK	-	5.52E-07	4.93E-01	3.58E-05	7711	
11	rs599190	69138317	5.90E-01	N/A	22	rs2413485	36523866	3.78E-02	H1F0	-	5.52E-07	5.06E-01	2.94E-04	7712	
6	rs1923747	17334064	4.80E-01	N/A	14	rs229586	64333100	3.76E-01	SPTB	-	5.52E-07	9.18E-01	3.36E-04	7713	
6	rs2281476	1.44E+08	9.91E-01	PLAGL1	3	rs6772225	55251558	1.06E-01	N/A	-	5.52E-07	1.48E-01	1.68E-03	7714	
5	rs17155896	1.03E+08	2.26E-01	N/A	12	rs1292011	1.14E+08	6.79E-01	N/A	-	5.52E-07	9.24E-01	2.24E-04	7715	
6	rs9503155	2314491	4.99E-01	N/A	5	rs153252	83501745	2.27E-01	EDIL3	-	5.52E-07	4.27E-01	6.01E-06	7716	
5	rs10471421	55696266	8.82E-02	N/A	11	rs2251835	1.16E+08	2.34E-01	N/A	-	5.52E-07	5.65E-01	2.44E-04	7717	
1	rs10920333	2E+08	9.55E-01	GPR37L1	20	rs1885284	21418454	9.46E-01	N/A	-	5.52E-07	8.85E-01	1.83E-04	7718	
5	rs17745927	1.7E+08	9.72E-01	KCNIP1	7	rs6467202	1.28E+08	4.41E-01	FAM71F2	-	5.52E-07	3.56E-01	1.18E-05	7719	
4	rs7663480	84987302	5.25E-01	N/A	9	rs6474967	15750988	5.75E-01	C9orf93	-	5.52E-07	2.54E-01	1.67E-05	7720	
9	rs7863832	1.13E+08	3.33E-01	GNG10	19	rs17599667	35042506	1.64E-02	N/A	-	5.52E-07	8.58E-01	4.92E-05	7721	
4	rs1547909	1.87E+08	4.44E-01	SORBS2	11	rs618929	95868655	7.44E-01	N/A	-	5.52E-07	6.46E-02	8.68E-07	7722	
10	rs731016	71206453	8.01E-01	N/A	22	rs6010044	49448804	4.82E-01	SHANK3	-	5.52E-07	5.95E-01	9.85E-04	7723	
4	rs17258848	1.28E+08	9.43E-01	N/A	15	rs11637659	65229440	4.59E-01	SMAD3	-	5.53E-07	1.98E-03	2.24E-08	7724	
11	rs669834	79043373	8.37E-01	N/A	18	rs7232740	5246587	4.97E-01	LOC339290	-	5.53E-07	1.24E-01	5.22E-03	7725	
17	rs9906543	32593335	3.97E-01	ACACA	17	rs4789106	69034532	1.94E-01	SDK2	-	5.53E-07	2.60E-01	1.90E-05	7726	

SNP A					SNP B						Interaction P			Ranking	Cluster in top 100 interactions
CHR	SNP	Location	gle locus P va	Gene	CHR	SNP	Location	Single locus P valu	Gene	MHC region	Stage 1	Stage 2	Combined		
6	rs4959998	4436492	5.90E-02	N/A	16	rs11648589	23203475	4.34E-01	SCNN1B	-	5.53E-07	6.00E-01	1.92E-04	7727	
2	rs12151639	71565457	9.31E-02	DYSF	7	rs12667810	9032697	2.73E-02	N/A	-	5.53E-07	2.00E-01	4.67E-03	7728	
6	rs851967	1.52E+08	3.49E-01	N/A	10	rs1659840	13142854	7.05E-01	N/A	-	5.53E-07	9.08E-01	3.19E-05	7729	
13	rs947076	97235209	7.22E-01	N/A	16	rs2270461	88499846	7.04E-01	MC1R	-	5.53E-07	4.53E-01	2.90E-05	7730	
4	rs13126626	1.84E+08	1.09E-01	N/A	5	rs10447222	1.5E+08	7.87E-01	RPS14	-	5.53E-07	7.14E-01	3.05E-04	7731	
3	rs9872768	1.48E+08	1.86E-01	N/A	4	rs2055931	34528519	3.95E-01	N/A	-	5.53E-07	7.87E-01	2.18E-04	7732	
12	rs1709696	1.27E+08	5.19E-01	TMEM132C	14	rs227019	22064646	3.29E-01	N/A	-	5.53E-07	8.72E-01	9.22E-05	7733	
6	rs4709756	1.64E+08	3.66E-01	N/A	1	rs2186104	2.32E+08	2.53E-01	SLC35F3	-	5.53E-07	3.84E-01	2.10E-05	7734	
1	rs2605082	2.18E+08	8.95E-01	N/A	1	rs2490371	2.35E+08	9.42E-02	RYR2	-	5.53E-07	5.84E-01	1.52E-05	7735	
7	rs10265774	1.56E+08	1.75E-01	LMBR1	17	rs9895907	5521311	2.74E-01	N/A	-	5.53E-07	9.57E-01	6.52E-05	7736	
7	rs10265774	1.56E+08	1.75E-01	LMBR1	17	rs9915238	5520461	3.13E-01	N/A	-	5.53E-07	9.57E-01	6.52E-05	7737	
7	rs10263303	1.57E+08	1.02E-01	PTPRN2	9	rs7863180	1.04E+08	5.20E-01	GRIN3A	-	5.53E-07	3.63E-02	7.61E-03	7738	
2	rs1424916	2.18E+08	5.75E-01	TNS1	3	rs740836	43769564	3.71E-01	N/A	-	5.53E-07	2.33E-01	2.60E-06	7739	
3	rs7612632	1.52E+08	5.85E-01	CLRN1OS	11	rs4755854	44605807	1.09E-01	CD82	-	5.53E-07	7.89E-01	8.41E-05	7740	
7	rs1534001	1.56E+08	4.98E-02	N/A	9	rs2015843	1.28E+08	4.25E-01	N/A	-	5.53E-07	2.62E-05	2.62E-05	7741	
1	rs1469267	1.64E+08	1.60E-01	RXRG	21	rs222954	26839258	8.30E-01	CYYR1	-	5.53E-07	3.08E-01	4.55E-04	7742	
1	rs12141538	43104003	3.77E-01	ZNF691	15	rs4984407	92422405	6.93E-01	N/A	-	5.54E-07	2.46E-01	1.34E-06	7743	
15	rs4702	89227564	2.93E-02	FES	21	rs373521	26179531	7.64E-01	APP	-	5.54E-07	2.89E-01	3.35E-06	7744	
6	rs6910563	19718814	2.28E-01	N/A	8	rs2028229	4643403	7.62E-01	CSMD1	-	5.54E-07	6.47E-01	2.27E-05	7745	
6	rs6934188	19719138	2.28E-01	N/A	8	rs2028229	4643403	7.62E-01	CSMD1	-	5.54E-07	6.47E-01	2.27E-05	7746	
6	rs4711987	51897548	3.42E-02	PKHD1	7	rs6973946	52171488	1.61E-01	N/A	-	5.54E-07	9.75E-01	6.48E-05	7747	
1	rs2206047	25067287	2.47E-02	N/A	4	rs17292184	62677428	4.92E-01	N/A	-	5.54E-07	5.84E-01	2.15E-04	7748	
15	rs12148851	93469960	5.52E-01	N/A	16	rs8047963	54347071	7.26E-01	N/A	-	5.54E-07	9.09E-01	1.77E-04	7749	
9	rs1337706	1.04E+08	8.55E-01	N/A	16	rs12600108	54865932	3.76E-01	GN/AO1	-	5.54E-07	8.47E-02	3.32E-06	7750	
1	rs920307	1.89E+08	2.98E-01	N/A	3	rs2197126	19398565	5.56E-01	KCNH8	-	5.54E-07	6.03E-01	1.34E-04	7751	
6	rs647108	1.38E+08	4.75E-01	N/A	3	rs1579905	74711280	1.34E-01	N/A	-	5.54E-07	6.76E-01	3.73E-05	7752	
3	rs7624435	97082944	2.66E-01	N/A	12	rs1881959	60854638	2.72E-01	FAM19A2	-	5.54E-07	9.81E-02	3.49E-03	7753	
13	rs726697	1.06E+08	4.64E-01	N/A	17	rs10515025	48161244	7.47E-01	N/A	-	5.54E-07	1.81E-01	9.49E-06	7754	
13	rs726697	1.06E+08	4.64E-01	N/A	17	rs4794378	48161698	7.47E-01	N/A	-	5.54E-07	1.81E-01	9.49E-06	7755	
13	rs726697	1.06E+08	4.64E-01	N/A	17	rs7216414	48155324	7.47E-01	N/A	-	5.54E-07	1.81E-01	9.49E-06	7756	
13	rs726697	1.06E+08	4.64E-01	N/A	17	rs866976	48156155	7.47E-01	N/A	-	5.54E-07	1.81E-01	9.49E-06	7757	
15	rs8025338	35681159	4.29E-01	N/A	22	rs738414	48481780	2.57E-01	N/A	-	5.54E-07	9.22E-01	6.53E-05	7758	
3	rs7432792	70564770	1.05E-01	N/A	22	rs5757290	37529882	6.56E-01	DN/AL4	-	5.54E-07	N/A	N/A	7759	
10	rs6479812	52394805	1.88E-01	N/A	20	rs6132222	19539835	2.14E-01	SLC24A3	-	5.55E-07	2.59E-01	8.17E-04	7760	
4	rs9996986	1.87E+08	8.69E-01	SORBS2	22	rs2187793	46066667	5.79E-01	N/A	-	5.55E-07	8.70E-01	3.28E-04	7761	
3	rs234062	1.74E+08	6.37E-01	N/A	10	rs6585679	85667401	7.20E-02	N/A	-	5.55E-07	7.99E-01	1.66E-04	7762	
10	rs4457653	4933518	3.48E-01	tAKR	11	rs656759	1.02E+08	3.72E-01	N/A	-	5.55E-07	4.19E-01	3.80E-05	7763	
8	rs10106907	96855324	7.33E-01	N/A	15	rs4774982	55853182	9.82E-01	N/A	-	5.55E-07	7.99E-01	8.11E-05	7764	
14	rs1885189	88457081	5.47E-01	N/A	16	rs7199026	70098727	8.99E-02	CHST4	-	5.55E-07	4.10E-03	2.06E-02	7765	
8	rs992617	18040775	8.84E-01	N/A	11	rs2403547	19944988	7.83E-01	N/AV2	-	5.55E-07	5.79E-01	3.00E-05	7766	
1	rs4950760	2E+08	9.08E-01	ELF3	9	rs10964427	2008825	3.19E-01	SMARCA2	-	5.55E-07	8.30E-01	3.32E-04	7767	
12	rs758637	3564728	2.42E-02	PRMT8	16	rs4073828	80527996	7.74E-01	PLCG2	-	5.55E-07	8.95E-01	5.03E-05	7768	
12	rs7958980	41545011	1.55E-01	N/A	13	rs724852	75369530	7.59E-01	N/A	-	5.55E-07	5.21E-02	2.48E-03	7769	
3	rs9815385	64899900	1.23E-01	MIR548A2	3	rs12636725	1.51E+08	2.92E-01	WWTR1	-	5.55E-07	7.41E-01	1.38E-05	7770	
2	rs1370600	1.34E+08	8.59E-01	NCKAP5	17	rs8069937	40564152	6.46E-01	ACBD4	-	5.55E-07	9.64E-01	1.20E-04	7771	
7	rs10255671	1.51E+08	4.48E-01	CRYGN	20	rs988166	54354265	9.39E-01	C20orf108	-	5.55E-07	1.44E-01	1.15E-05	7772	
2	rs10200446	1.3E+08	9.47E-01	N/A	13	rs1543001	1.07E+08	1.33E-01	N/A	-	5.55E-07	N/A	N/A	7773	
6	rs1520	39634845	1.02E-01	KIF6	12	rs1356954	76074319	2.85E-01	N/A	-	5.55E-07	6.25E-02	5.26E-03	7774	
6	rs1520	39634845	1.02E-01	KIF6	12	rs771655	76069560	2.85E-01	N/A	-	5.55E-07	6.25E-02	5.26E-03	7775	
10	rs7916448	24129585	6.01E-01	KIAA1217	10	rs10509103	60917716	5.79E-01	N/A	-	5.55E-07	N/A	N/A	7776	
6	rs9402970	1.39E+08	5.89E-01	KIAA1244	5	rs17540829	16581227	6.92E-01	FAM134B	-	5.56E-07	2.64E-01	1.29E-05	7777	
10	rs3817218	17020931	5.76E-04	CUBN	14	rs2355659	49553790	7.51E-01	C14orf182	-	5.56E-07	7.68E-01	3.78E-04	7778	
5	rs10054105	1.11E+08	6.53E-01	N/A	17	rs2070782	59785865	4.81E-01	N/A	-	5.56E-07	8.31E-01	1.88E-05	7779	
2	rs4668976	15818201	1.72E-01	N/A	10	rs4880384	1.34E+08	9.99E-01	N/A	-	5.56E-07	9.12E-01	5.09E-05	7780	
5	rs655599	1.18E+08	3.85E-01	N/A	12	rs6582630	37029775	2.82E-02	ALG10B	-	5.56E-07	N/A	N/A	7781	
3	rs9211	12914546	7.97E-01	IQSEC1	9	rs7037974	1.17E+08	9.47E-01	1-Dec	-	5.56E-07	3.00E-01	1.25E-05	7782	
2	rs9283527	2.15E+08	4.89E-01	SPAG16	14	rs243296	32796044	2.33E-01	NPAS3	-	5.56E-07	8.31E-01	2.44E-04	7783	
5	rs11742240	35917133	2.68E-01	IL7R	21	rs2091895	40993998	1.65E-01	DSCAM	-	5.56E-07	8.60E-01	4.84E-04	7784	
11	rs10458992	1.25E+08	2.95E-01	CDON	12	rs1001127	6472004	6.42E-01	MRPL51	-	5.56E-07	N/A	N/A	7785	
1	rs1360564	1.08E+08	3.42E-01	VAV3	4	rs6850861	1.78E+08	8.83E-01	NEIL3	-	5.56E-07	N/A	N/A	7786	
9	rs2295932	1.01E+08	6.78E-01	N/A	16	rs2967875	83193101	2.17E-01	COTL1	-	5.56E-07	1.14E-01	7.85E-07	7787	
6	rs9389037	1.33E+08	9.95E-01	N/A	16	rs1566458	83576432	5.21E-01	ZDHHC7	-	5.56E-07	1.84E-01	3.72E-03	7788	
6	rs9375927	1.33E+08	9.95E-01	N/A	16	rs1566458	83576432	5.21E-01	ZDHHC7	-	5.56E-07	2.37E-01	2.72E-03	7789	
18	rs1540028	69386571	9.71E-01	N/A	19	rs4453628	2416737	1.74E-01	LMNB2	-	5.56E-07	3.60E-01	2.04E-05	7790	
6	rs9344674	87859142	3.70E-01	CGA	11	rs6589485	1.15E+08	2.27E-01	CADM1	-	5.56E-07	6.06E-01	2.51E-05	7791	
10	rs6599700	1.25E+08	9.00E-01	N/A	21	rs2826601	21193520	8.89E-01	N/A	-	5.56E-07	6.22E-01	3.02E-05	7792	
1	rs7513079	12708081	3.33E-01	AADACL3	8	rs2741098	6677686	2.86E-01	XKR5	-	5.56E-07	9.55E-01	6.15E-05	7793	
7	rs10281326	1.47E+08	3.71E-01	CNTN/AP2	11	rs16831	1.27E+08	6.34E-01	N/A	-	5.56E-07	9.71E-01	1.74E-04	7794	
3	rs7639399	14358820	6.42E-01	N/A	5	rs257244	97378881	7.60E-01	N/A	-	5.56E-07	N/A	N/A	7795	
8	rs2977391	59257784	4.73E-01	N/A	20	rs11700048	6033006	4.64E-01	FERMT1	-	5.56E-07	1.60E-01	1.98E-05	7796	
8	rs2977391	59257784	4.73E-01	N/A	20	rs11700084	6033216	4.64E-01	FERMT1	-	5.56E-07	1.91E-01	2.64E-05	7797	
3	rs10934586	1.24E+08	8.37E-01	CCDC58	10	rs7086109	1.15E+08	9.73E-01	N/A	-	5.56E-07	2.71E-01	6.02E-06	7798	
5	rs1482483	1.13E+08	9.00E-01	N/A	10	rs4881075	3119472	9.14E-01	PFKP	-	5.56E-07	5.11E-01	6.70E-04	7799	
6	rs2015519	85510501	2.72E-01	TBX18	1	rs6664249	2.38E+08	9.24E-01	N/A	-	5.56E-07	6.83E-01	4.69E-04	7800	
9	rs2291681	14780841	7.32E-01	FREM1	19	rs4803449	46426506	9.22E-01	AXL	-	5.56E-07	9.19E-01	5.63E-05	7801	
4	rs869240	1.84E+08	3.74E-01	ODZ3	10	rs1049432	59825126	7.48E-01	TFAM	-	5.56E-07	9.80E-01	1.50E-04	7802	
6	rs1010549	1.64E+08	5.13E-01	N/A	9	rs10966450	24760659	3.09E-02	N/A	-	5.56E-07	N/A	N/A	7803	
6	rs4959847	3461503	2.56E-02	N/A	4	rs11723268	54480980	6.98E-01	N/A	-	5.56E-07	7.12E-01	6.02E-05	7804	
12	rs7296020	61595594	4.84E-01	PPM1H	13	rs7319605	91405375	7.92E-01	GPC5	-	5.57E-07	8.10E-01	3.47E-04	7805	
1	rs12046389	1.03E+08	9.05E-01	N/A	17	rs9899594	29363775	2.26E-01	ACCN1	-	5.57E-07	9.17E-01	3.85E-04	7806	
4	rs6848033	1.55E+08	8.56E-01	KIAA0922	20	rs2064505	31859046	7.12E-01	ZNF341	-	5.57E-07	2.21E-01	5.90E-04	7807	
20	rs2206801	11959405	4.23E-01	N/A	22	rs1006253	33903871	8.62E-01	N/A	-	5.57E-07	6.15E-01	4.91E-04	7808	
20	rs2206801	11959405	4.23E-01	N/A	22	rs2213877	33904374	8.62E-01	N/A	-	5.57E-07	6.15E-01	4.91E-04	7809	
7	rs10245681	1.35E+08	9.51E-01	N/A	9	rs484397	1.37E+08	9.45E-01	N/A	-	5.57E-07	7.70E-01	3.61E-04	7810	
9	rs10810827	1767030	2.30E-01	N/A	14	rs1376889	45987357	6.90E-01	N/A	-	5.57E-07	1.60E-01	3.41E-06	7811	
5	rs10805919	77628680	8.71E-01	AP3B1	13	rs7317926	53073513	7.78E-02	N/A	-	5.57E-07	3.23E-01	6.16E-04	7812	
6	rs10458124	1388098	7.98E-01	N/A	8	rs12679850	1009757	4.98E-01	N/A	-	5.57E-07	6.42E-01	8.38E-05	7813	
6	rs9393291	22542472	2.21E-01	N/A	14	rs8005371	33087261	6.25E-01	NPAS3	-	5.57E-07	7.11E-01	2.78E-05	7814	
2	rs12992898	68887661	6.39E-01	ARHGAP25	17	rs4293443	68382106	8.81E-01	SLC39A11	-	5.57E-07	7.68E-01	1.34E-05	7815	
5	rs31299	16766465	8.85E-01	MYO10	9	rs17587560	8994122	1.97E-01	PTPRD	-	5.57E-07	3.73E-02	2.18E-03	7816	
5	rs10036373	23945676	3.75E-01	N/A	10	rs11253773	16171295	5.97E-01	N/A	-	5.57E-07	7.13E-01	2.45E-05	7817	
5	rs10473666	23919986	3.75E-01	N/A	10	rs11253773	16171295	5.97E-01	N/A	-	5.57E-07	N/A	N/A	7818	
3	rs7651713	1.13E+08	9.45E-01	N/A	7	rs2107552	30827230	1.54E-01	FAM188B	-	5.57E-07	6.98E-01	3.01E-04	7819	
1	rs10922563	89319391	2.84E-01	GBP1	13	rs9538282	58519787	2.47E-01	N/A	-	5.57E-07	3.16E-01	8.46E-06	7820	
11	rs11604602	1.13E+08	3.99E-01	TMPRSS5	16	rs8047350	73236745	7.81E-01	RFWD3	-	5.57E-07	7.22E-01	8.47E-05	7821	
3	rs902417	1.85E+08	2.31E-01	VWA5B2	13	rs2985923	66653746	1.50E-01	PCDH9	-	5.58E-07	4.90E-02	2.99E-03	7822	
2	rs3769581	32822620	7.28E-01	N/A	14	rs3861621	77478622	7.86E-02	ADCK1	-	5.58E-07	1.44E-01	5.67E-03	7823	
6	rs2268650	39138379	4.51E-01	GLP1R	8	rs2970694	37049213	9.58E-01	N/A	-	5.58E-07	2.83E-01	1.46E-03	7824	
6	rs6937258	93450044	5.04E-01	N/A	10	rs4256883	63577608	9.27E-02	N/A	-	5.58E-07	3.59E-01	2.19E-05	7825	
6	rs1265110	31227401	8.90E-01	TCF19	6	rs9348862	31360726	7.72E-01	HLA-C	MHC	5.58E-07	2.95E-01	4.30E-06	7826	
6	rs1265110	31227401	8.90E-01	TCF19	6	rs9357122	31368324	7.72E-01	N/A	MHC	5.58E-07	2.95E-01	4.30E-06	7827	
10	rs878321	15597121	4.70E-02	ITGA8	22	rs4282002	26350555	9.72E-01	N/A	-	5.58E-07	6.00E-01	3.21E-06	7828	
4	rs2321684	1.42E+08	9.54E-01	RNF150	14	rs1188551	19724187	9.78E-02	OR11G2	-	5.58E-07	9.79E-01	8.60E-05	7829	
4	rs2321684	1.42E+08	9.54E-01	RNF150	14	rs1188553	19724802	9.78E-02	OR11G2	-	5.58E-07	9.79E-01	8.60E-05	7830	
2	rs7587880	2.15E+08	4.53E-01	SPAG16	14	rs243296	32796044	2.33E-01	NPAS3	-	5.58E-07	8.31E-01	2.45E-04	7831	
5	rs716866	1.69E+08	1.09E-01	SLIT3	9	rs1444832	74497539	1.90E-01	TMC1	-	5.58E-07	1.63E-01	9.15E-04	7832	
4	rs4328901	25338157	9.22E-01	SEL1L3	4	rs6843675	1.06E+08	9.70E-01	N/A	-	5.58E-07	7.98E-01	2.87E-05	7833	
7	rs12540373	7796065	8.75E-01	N/A	9	rs783772	1.38E+08	4.07E-01	PAEP	-	5.58E-07	4.54E-01	4.67E-05	7834	
13	rs9521509	1.09E+08	4.27E-01	IRS2	14	rs12589158	81468914	2.87E-01	N/A	-	5.58E-07	6.88E-01	8.02E-05	7835	
1	rs2224648	1.96E+08	3.54E-01	N/A	10	rs10795505	17780153	4.98E-01	STAM	-	5.58E-07	7.61E-01	2.50E-04	7836	
3	rs333507	78923643	6.68E-02	ROBO1	21	rs12483420	41428996	6.97E-02	C21orf130	-	5.58E-07	3.04E-01	2.54E-06	7837	
3	rs7650002	78926964	6.68E-02	ROBO1	21	rs12483420	41428996	6.97E-02	C21orf130	-	5.58E-07	3.04E-01	2.54E-06	7838	

SNP A					SNP B						Interaction P			Ranking	Cluster in top 100 interactions
CHR	SNP	Location	gle locus P va	Gene	CHR	SNP	Location	ingle locus P valu	Gene	HC region	Stage 1	Stage 2	Combined		
3	rs717478	21240113	4.43E-01	N/A	17	rs4790519	3403485	2.02E-01	TRPV3	-	5.58E-07	5.66E-01	3.99E-04	7839	
6	rs6900214	67144853	4.90E-01	N/A	13	rs562992	1.1E+08	7.11E-02	COL4A1	-	5.58E-07	6.11E-01	1.45E-04	7840	
6	rs2146342	11310343	3.55E-01	NEDD9	7	's12539524	22081979	3.34E-01	N/A	-	5.58E-07	1.53E-01	1.95E-03	7841	
5	rs2132502	18712631	7.05E-01	N/A	8	rs1531590	76198999	9.00E-01	N/A	-	5.58E-07	6.41E-01	1.45E-03	7842	
2	rs1859712	1.02E+08	7.93E-01	MAP4K4	16	's11076066	53632581	8.63E-02	N/A	-	5.58E-07	7.23E-01	1.88E-04	7843	
11	rs7129746	38870235	2.67E-01	N/A	13	rs4772971	1.08E+08	5.04E-01	N/A	-	5.58E-07	7.40E-01	2.95E-05	7844	
8	rs6981109	82102994	7.81E-01	PAG1	20	rs6088301	31833015	7.29E-01	ZNF341	-	5.59E-07	8.66E-02	4.44E-07	7845	
11	rs2566231	4491658	5.06E-01	N/A	18	rs2322094	5427967	1.18E-01	EPB41L3	-	5.59E-07	6.14E-01	5.80E-05	7846	
2	rs954845	6804068	2.36E-01	N/A	15	rs6598321	98646388	5.98E-01	ADAMTS17	-	5.59E-07	N/A	N/A	7847	
7	rs2190930	1.24E+08	6.60E-01	N/A	10	rs1747677	1.06E+08	9.81E-01	COL17A1	-	5.59E-07	1.08E-01	1.36E-06	7848	
2	rs3769185	1.74E+08	7.80E-01	ZAK	18	rs4539677	43190135	4.57E-01	N/A	-	5.59E-07	1.13E-01	5.23E-03	7849	
6	rs3130453	31232828	5.90E-01	TCF19	6	rs1131896	31487094	3.40E-01	MICA	MHC	5.59E-07	4.22E-01	4.67E-04	7850	
2	rs744556	1.9E+08	7.77E-01	WDR75	8	rs2200330	20299494	2.05E-01	N/A	-	5.59E-07	7.13E-01	5.93E-05	7851	
1	rs3790843	1.98E+08	4.74E-01	NR5A2	2	rs1517499	2.26E+08	8.86E-01	KIAA1486	-	5.59E-07	2.20E-01	2.14E-06	7852	
2	rs295789	1.57E+08	2.70E-01	N/A	9	rs6479604	97150069	8.31E-01	N/A	-	5.59E-07	9.52E-01	4.06E-05	7853	
2	rs295793	1.57E+08	2.70E-01	N/A	9	rs6479604	97150069	8.31E-01	N/A	-	5.59E-07	9.74E-01	4.37E-05	7854	
2	rs295800	1.57E+08	2.70E-01	N/A	9	rs6479604	97150069	8.31E-01	N/A	-	5.59E-07	9.74E-01	4.37E-05	7855	
4	rs13110363	1.63E+08	2.81E-01	FSTL5	13	rs1151375	1.01E+08	8.61E-02	N/ALCN	-	5.59E-07	6.84E-01	1.73E-05	7856	
13	rs9510171	21858130	4.89E-01	N/A	15	rs8030397	31731603	1.79E-01	RYR3	-	5.59E-07	1.88E-01	8.53E-04	7857	
7	rs6460664	70638550	2.18E-01	WBSCR17	16	rs750950	86430748	8.59E-01	SLC7A5	-	5.59E-07	6.53E-01	7.88E-04	7858	
11	rs4755969	45306615	3.46E-01	N/A	22	rs926331	35840018	4.51E-01	TMPRSS6	-	5.59E-07	3.92E-01	1.25E-03	7859	
12	rs1395342	51085568	7.06E-01	KRT75	14	rs8010709	50365250	3.15E-02	NIN	-	5.59E-07	6.85E-01	7.56E-05	7860	
8	rs11136659	3585782	7.74E-03	CSMD1	8	rs4875302	4028885	2.97E-01	CSMD1	-	5.59E-07	8.18E-01	1.12E-04	7861	
6	rs7742508	70834071	2.99E-01	COL19A1	8	rs2294119	1.45E+08	1.95E-02	ZC3H3	-	5.59E-07	5.28E-01	1.04E-04	7862	
5	rs1348473	1.2E+08	8.23E-01	N/A	14	rs1958055	33254537	4.21E-01	NPAS3	-	5.59E-07	5.18E-02	1.20E-07	7863	
14	rs11157994	53521052	6.80E-01	N/A	18	rs7506045	11977272	3.81E-01	IMPA2	-	5.59E-07	5.39E-01	2.53E-05	7864	
1	rs4240918	2.15E+08	6.52E-01	N/A	10	rs1002936	5725215	1.03E-01	ASB13	-	5.59E-07	2.96E-01	4.06E-04	7865	
10	rs7090538	1.27E+08	7.36E-01	CTBP2	18	rs1380711	71644131	6.79E-02	N/A	-	5.60E-07	5.72E-01	1.84E-04	7866	
10	rs7090538	1.27E+08	7.36E-01	CTBP2	18	's11150915	71645804	6.79E-02	N/A	-	5.60E-07	6.04E-01	1.67E-04	7867	
2	rs12618856	2515922	7.68E-01	N/A	11	's10833362	20565955	7.18E-01	SLC6A5	-	5.60E-07	6.06E-01	5.97E-05	7868	
10	rs666101	14468193	1.20E-01	N/A	13	rs7336806	26487593	9.23E-01	N/A	-	5.60E-07	N/A	N/A	7869	
4	rs4495109	55611307	9.13E-01	N/A	16	rs3855650	65016360	9.39E-01	BEAN	-	5.60E-07	N/A	N/A	7870	
5	rs4868506	1.75E+08	8.40E-01	N/A	7	rs4449730	1.32E+08	5.26E-04	N/A	-	5.60E-07	1.01E-02	4.07E-02	7871	
2	rs10179483	2.04E+08	8.49E-01	N/A	12	rs537938	3264359	8.36E-01	TSPAN9	-	5.60E-07	1.86E-01	1.87E-06	7872	
3	rs4575830	34938207	2.88E-01	N/A	8	rs4925813	1.46E+08	3.21E-01	KIAA1688	-	5.60E-07	6.75E-01	5.50E-05	7873	
6	rs3018	5883367	9.10E-01	N/A	2	rs4668879	15221205	8.99E-01	NBAS	-	5.60E-07	9.32E-01	4.88E-04	7874	
2	rs7425902	2.39E+08	2.54E-01	N/A	14	's10149590	23263944	6.60E-01	N/A	-	5.60E-07	3.05E-01	8.06E-04	7875	
9	rs7035562	1.25E+08	2.52E-01	CRB2	14	's17113911	42671419	5.97E-01	N/A	-	5.60E-07	2.44E-01	1.78E-03	7876	
1	rs2224648	1.96E+08	3.54E-01	N/A	10	rs3780960	17727559	3.96E-01	STAM	-	5.60E-07	8.23E-01	1.90E-04	7877	
8	rs7465378	1.43E+08	4.91E-01	N/A	15	rs2542597	84117079	9.10E-01	MIR1276	-	5.60E-07	1.62E-01	9.63E-04	7878	
3	rs2071387	1.41E+08	6.07E-01	RBP1	4	's12647535	39433024	6.34E-01	UBE2K	-	5.60E-07	9.10E-01	6.01E-05	7879	
3	rs2071387	1.41E+08	6.07E-01	RBP1	4	rs724256	39424302	6.34E-01	UBE2K	-	5.60E-07	9.10E-01	6.01E-05	7880	
2	rs2001427	22951846	4.97E-01	N/A	10	rs2092833	83239996	4.15E-01	N/A	-	5.60E-07	N/A	N/A	7881	
7	rs4728178	1.29E+08	3.28E-01	N/A	14	's17126895	53317833	6.46E-01	N/A	-	5.60E-07	7.89E-01	2.39E-05	7882	
1	rs10917811	1.62E+08	8.49E-02	N/A	4	's17590593	1.14E+08	7.24E-01	ANK2	-	5.60E-07	5.67E-02	3.94E-03	7883	
6	rs332496	93512596	3.60E-01	N/A	8	rs3862107	16960135	5.76E-01	EFHA2	-	5.60E-07	3.31E-01	8.93E-04	7884	
2	rs1843758	1.73E+08	8.06E-01	N/A	4	rs1490452	44003304	8.80E-01	KCTD8	-	5.60E-07	5.13E-01	9.01E-04	7885	
5	rs4700752	1.78E+08	5.38E-01	N/A	7	's12537447	10854274	3.29E-01	N/A	-	5.60E-07	5.76E-01	1.18E-05	7886	
5	rs4700971	1.78E+08	5.38E-01	N/A	7	's12537447	10854274	3.29E-01	N/A	-	5.60E-07	5.76E-01	1.18E-05	7887	
15	rs4984516	94529167	6.68E-01	N/A	16	rs3785220	7644364	7.76E-01	A2BP1	-	5.61E-07	5.62E-01	1.36E-05	7888	
3	rs860309	1.96E+08	1.06E-01	N/A	9	rs7038375	80865574	3.30E-01	N/A	-	5.61E-07	7.31E-01	1.28E-03	7889	
9	rs10760165	1.23E+08	9.87E-01	GSN	13	rs928021	69604009	4.49E-01	ATXN8OS	-	5.61E-07	7.24E-01	2.67E-04	7890	
3	rs1370743	1.22E+08	5.55E-01	N/A	5	rs6595591	1.25E+08	2.60E-01	N/A	-	5.61E-07	7.65E-02	1.89E-06	7891	
5	rs10064418	1.25E+08	1.27E-01	N/A	10	's10510050	1.21E+08	8.33E-01	N/A	-	5.61E-07	3.07E-01	1.27E-03	7892	
4	rs1491380	23364356	9.87E-01	N/A	16	rs3912990	53192399	3.88E-01	N/A	-	5.61E-07	4.80E-01	1.70E-05	7893	
4	rs11132603	1.9E+08	5.75E-01	N/A	11	rs1943761	1.01E+08	4.04E-01	N/A	-	5.61E-07	8.68E-01	2.14E-04	7894	
4	rs2889670	1.9E+08	5.75E-01	N/A	11	rs1943761	1.01E+08	4.04E-01	N/A	-	5.61E-07	8.68E-01	2.14E-04	7895	
7	rs10950803	2639592	4.18E-01	IQCE	11	rs2220086	83285502	2.06E-01	DLG2	-	5.61E-07	6.04E-01	2.39E-05	7896	
5	rs3733989	1.68E+08	2.01E-01	ODZ2	18	rs4519422	71790009	5.10E-02	N/A	-	5.61E-07	7.32E-02	6.71E-07	7897	
6	rs9352581	79003608	4.41E-01	N/A	7	rs765951	1.49E+08	7.40E-01	N/A	-	5.61E-07	1.90E-01	3.01E-06	7898	
6	rs2817064	35849941	7.74E-01	C6orf126	22	rs565979	19358946	3.62E-01	POM121L4P	MHC	5.61E-07	9.75E-01	6.29E-05	7899	
3	rs6441419	1.63E+08	6.08E-01	N/A	7	's10229386	43210547	3.33E-01	HECW1	-	5.61E-07	9.46E-02	5.77E-06	7900	
5	rs6452162	23853128	4.72E-02	N/A	10	rs4748247	16167564	5.20E-01	N/A	-	5.61E-07	5.80E-01	5.40E-04	7901	
5	rs12653782	1.56E+08	9.52E-01	SGCD	17	rs618743	73981463	7.14E-01	DN/AH17	-	5.61E-07	6.35E-01	1.36E-04	7902	
1	rs4655724	67771577	4.44E-01	N/A	12	rs1471997	47009862	8.07E-01	N/A	-	5.61E-07	1.38E-01	4.30E-03	7903	
1	rs7539855	67770721	4.44E-01	N/A	12	rs1471997	47009862	8.07E-01	N/A	-	5.61E-07	1.38E-01	4.30E-03	7904	
3	rs6793237	2844449	7.92E-01	CNTN4	22	rs9614732	44408187	7.21E-02	N/A	-	5.61E-07	5.50E-01	5.80E-04	7905	
2	rs2116435	54925978	2.30E-01	EML6	16	rs7186985	27156744	4.64E-01	NSMCE1	-	5.61E-07	N/A	N/A	7906	
9	rs1591029	25488831	8.02E-01	N/A	16	rs899305	7140564	2.91E-01	RBFOX1	-	5.61E-07	2.42E-03	1.52E-08	7907	
3	rs2365794	20131306	5.03E-01	KAT2B	12	rs6538509	93474528	2.24E-01	TMCC3	-	5.61E-07	6.41E-01	3.71E-04	7908	
2	rs4527188	2.22E+08	9.99E-02	N/A	3	's12633980	126789	4.20E-01	N/A	-	5.62E-07	2.23E-01	1.36E-03	7909	
1	rs10918897	1.67E+08	9.85E-01	N/A	17	's11658364	5578000	7.47E-01	N/A	-	5.62E-07	3.04E-01	6.96E-04	7910	
6	rs1325833	54004114	4.11E-01	C6orf142	15	rs1500652	95894238	3.26E-01	N/A	-	5.62E-07	5.13E-01	2.11E-05	7911	
9	rs10867274	80592007	1.23E-01	N/A	12	rs1506384	30491520	8.29E-01	N/A	-	5.62E-07	9.90E-01	1.76E-05	7912	
1	rs630538	54370863	3.98E-02	CDCP2	21	rs2825928	20274865	8.44E-01	N/A	-	5.62E-07	3.76E-01	8.28E-06	7913	
4	rs4864014	1.32E+08	1.40E-01	N/A	11	rs2060942	99302537	2.24E-01	CNTN5	-	5.62E-07	5.70E-01	1.30E-05	7914	
6	rs9447374	75706288	2.98E-01	N/A	14	rs712285	33696953	8.59E-01	N/A	-	5.62E-07	2.56E-01	1.27E-03	7915	
2	rs13410903	1.56E+08	7.52E-01	N/A	8	's17668341	1026696	9.50E-01	N/A	-	5.62E-07	3.90E-01	2.30E-05	7916	
13	rs9541712	68481740	1.99E-01	N/A	20	's11204437	59650650	9.75E-01	CDH4	-	5.62E-07	7.01E-01	1.54E-04	7917	
5	rs2115251	1.03E+08	1.07E-02	N/A	21	rs2836285	38578375	4.86E-01	KCNJ15	-	5.62E-07	8.84E-01	1.92E-05	7918	
8	rs13255574	6386085	3.62E-01	MCPH1	16	rs2253910	10663548	3.83E-01	TEKT5	-	5.62E-07	9.10E-01	3.65E-05	7919	
1	rs1360903	86345051	2.62E-01	COL24A1	2	rs2168395	33592801	9.34E-01	RASGRP3	-	5.62E-07	9.56E-01	1.44E-04	7920	
3	rs10510998	70599111	2.53E-01	N/A	22	rs5757290	37529882	6.56E-01	DN/AL4	-	5.62E-07	N/A	N/A	7921	
10	rs11201843	87698090	6.50E-01	GRID1	11	rs4930431	67050884	4.54E-01	CDK2AP2	-	5.62E-07	6.15E-01	4.75E-05	7922	
1	rs10910522	2.33E+08	5.43E-01	N/A	5	's10940145	67326728	9.96E-01	N/A	-	5.62E-07	6.60E-01	2.54E-05	7923	
3	rs17236986	1.83E+08	1.00E-01	N/A	10	's10508240	2719953	4.62E-01	N/A	-	5.62E-07	4.44E-01	1.13E-05	7924	
2	rs10496557	1.2E+08	3.47E-01	N/A	4	rs2903269	1.03E+08	3.42E-01	N/A	-	5.62E-07	7.76E-01	6.85E-05	7925	
5	rs2963894	9930169	4.71E-01	LOC285692	13	's17088289	70938213	4.58E-02	DACH1	-	5.62E-07	1.76E-01	4.96E-06	7926	
16	rs12443623	56662165	6.63E-01	N/A	21	rs4818955	43653515	2.50E-01	SIK1	-	5.63E-07	1.93E-01	1.17E-05	7927	
5	rs4912652	1.43E+08	9.63E-01	N/A	10	rs2854992	73256591	3.39E-01	CDH23	-	5.63E-07	9.20E-01	2.56E-04	7928	
1	rs6429216	2.39E+08	8.73E-01	N/A	3	rs7612998	1.91E+08	5.88E-01	LEPREL1	-	5.63E-07	3.50E-01	3.42E-05	7929	
6	rs1572801	1.65E+08	7.52E-01	N/A	10	rs7072606	49603980	1.18E-01	WDFY4	-	5.63E-07	3.15E-01	2.02E-04	7930	
7	rs389153	1.53E+08	3.08E-02	N/A	8	's11136948	5786984	9.36E-01	N/A	-	5.63E-07	5.26E-01	5.71E-04	7931	
5	rs1541913	1.65E+08	6.06E-01	N/A	15	rs8035124	89906712	3.42E-01	N/A	-	5.63E-07	6.57E-02	2.07E-03	7932	
5	rs33294	68106034	3.68E-01	N/A	5	rs4147470	1.49E+08	4.88E-01	ABLIM3	-	5.63E-07	9.59E-02	2.99E-03	7933	
3	rs6789670	1.72E+08	2.03E-02	SLC7A14	15	rs7162561	61452818	7.60E-01	CA12	-	5.63E-07	N/A	N/A	7934	
7	rs1551098	33564322	9.00E-01	BBS9	21	rs2822892	15044008	3.39E-01	N/A	-	5.63E-07	7.09E-01	8.09E-06	7935	
1	rs6588502	54335744	5.60E-01	C1orf83	17	rs7211140	69685505	6.98E-01	N/A	-	5.63E-07	6.23E-01	3.62E-05	7936	
1	rs2376280	1.03E+08	7.35E-01	N/A	12	rs697635	50569015	9.28E-01	ANKRD33	-	5.63E-07	6.72E-01	3.70E-04	7937	
15	rs2278309	31830119	8.73E-02	RYR3	15	rs4779113	77649940	6.88E-01	N/A	-	5.63E-07	7.56E-01	1.08E-04	7938	
15	rs2278309	31830119	8.73E-02	RYR3	15	rs4779114	77650100	6.88E-01	N/A	-	5.63E-07	7.56E-01	1.08E-04	7939	
8	rs1635668	5591685	3.93E-01	N/A	13	rs1353523	59215544	8.67E-01	DIAPH3	-	5.63E-07	9.23E-01	1.03E-04	7940	
11	rs4492782	97370202	2.69E-01	N/A	19	rs4807471	3399694	1.10E-01	NFIC	-	5.63E-07	1.21E-01	3.24E-03	7941	
4	rs13434618	72685352	5.91E-01	N/A	4	rs7665426	1.76E+08	3.40E-01	N/A	-	5.63E-07	3.52E-01	1.88E-06	7942	
8	rs7829925	83586789	7.73E-01	N/A	9	rs7039136	1.28E+08	9.18E-01	FAM125B	-	5.63E-07	5.54E-01	6.65E-04	7943	
4	rs13434618	72685352	5.91E-01	N/A	4	rs7655606	1.76E+08	3.40E-01	N/A	-	5.63E-07	N/A	N/A	7944	
1	rs2817178	3037112	8.46E-01	PRDM16	16	's11647913	6612412	2.68E-01	A2BP1	-	5.64E-07	3.10E-01	1.12E-03	7945	
6	rs7769547	39156735	5.43E-01	GLP1R	7	rs846931	1.05E+08	1.08E-01	ATXN7L1	-	5.64E-07	7.30E-01	3.44E-04	7946	
6	rs9398763	1.25E+08	6.09E-01	NKAIN2	6	rs7768039	1.63E+08	8.16E-01	PARK2	-	5.64E-07	9.96E-01	7.47E-05	7947	
7	rs6976604	29580803	4.48E-01	PRR15	19	rs8103325	21340051	1.00E-01	ZNF738	-	5.64E-07	N/A	N/A	7948	
1	rs2491864	2.41E+08	5.14E-01	N/A	11	rs1529530	44472551	1.84E-01	N/A	-	5.64E-07	2.64E-01	4.30E-06	7949	
3	rs12152230	35728583	4.12E-01	ARPP-21	9	rs7040543	1.33E+08	6.13E-01	ABL1	-	5.64E-07	5.24E-01	6.28E-05	7950	

SNP A					SNP B						Interaction P			Ranking	Cluster in top 100 interactions
CHR	SNP	Location	gle locus P va	Gene	CHR	SNP	Location	Single locus P valu	Gene	MHC region	Stage 1	Stage 2	Combined		
7	rs12532271	8132548	4.44E-02	ICA1	7	s10255671	1.51E+08	4.48E-01	CRYGN	-	5.64E-07	2.06E-01	1.13E-05	7951	
4	rs1909805	1.32E+08	7.22E-01	N/A	8	rs2114695	82661686	2.74E-01	N/A	-	5.64E-07	5.14E-01	8.65E-06	7952	
1	rs12096993	2.18E+08	5.84E-01	N/A	7	rs1076283	1.49E+08	2.59E-01	N/A	-	5.64E-07	5.83E-01	9.14E-05	7953	
3	rs557077	55506307	2.34E-01	ERC2	3	s12496746	57167967	9.29E-01	IL17RD	-	5.64E-07	3.43E-01	5.99E-06	7954	
1	rs4951090	2.03E+08	6.58E-01	LRRN2	7	rs2299395	1.03E+08	8.00E-01	RELN	-	5.64E-07	4.06E-01	9.45E-06	7955	
4	rs17019139	93283210	8.24E-01	N/A	15	s16967121	36710299	6.81E-01	N/A	-	5.64E-07	9.91E-01	1.07E-04	7956	
6	rs624243	70977333	5.86E-01	COL19A1	18	rs2217127	33475628	3.01E-02	N/A	-	5.64E-07	8.52E-02	1.82E-03	7957	
6	rs807515	24627865	6.59E-03	ALDH5A1	7	rs987252	51660260	8.05E-01	N/A	-	5.64E-07	7.14E-01	4.94E-05	7958	
2	rs11685620	16039315	1.84E-01	N/A	4	s11098092	1.12E+08	2.97E-01	N/A	-	5.64E-07	8.56E-01	2.77E-04	7959	
8	rs7001469	15841353	3.68E-01	N/A	16	rs8043555	27685332	2.55E-01	KIAA0556	-	5.64E-07	9.67E-01	7.54E-05	7960	
10	rs2710771	92431406	1.13E-01	N/A	10	rs181499	1.19E+08	4.00E-01	N/A	-	5.64E-07	2.27E-01	1.52E-06	7961	
3	rs6766004	28988908	2.46E-01	N/A	15	rs1568657	81517183	7.10E-01	BTBD1	-	5.64E-07	2.67E-01	7.53E-04	7962	
7	rs7809837	48692714	6.64E-01	N/A	9	s12683817	79240630	8.39E-01	GN/A14	-	5.64E-07	3.65E-01	3.59E-03	7963	
6	rs7769239	1.24E+08	4.56E-01	TRDN	6	rs1777220	1.26E+08	5.13E-01	N/A	-	5.64E-07	4.44E-01	4.49E-05	7964	
7	rs741299	36319013	4.51E-01	EEPD1	18	rs1849885	25192611	3.25E-01	N/A	-	5.64E-07	N/A	N/A	7965	
7	rs537276	17491609	8.35E-01	N/A	14	rs4624074	33814177	7.11E-01	N/A	-	5.64E-07	7.04E-01	5.15E-05	7966	
5	rs401681	1375087	9.51E-01	CLPTM1L	17	rs4251739	31172196	3.31E-01	TAF15	-	5.64E-07	9.18E-01	4.63E-04	7967	
9	rs1486806	11737626	1.52E-01	N/A	11	rs3930459	7910534	7.59E-01	OR10A6	-	5.65E-07	4.03E-01	8.04E-06	7968	
9	rs1486806	11737626	1.52E-01	N/A	11	rs7933807	7906367	7.59E-01	OR10A6	-	5.65E-07	4.03E-01	8.04E-06	7969	
2	rs13024316	1.42E+08	9.16E-01	LRP1B	7	rs7789801	52063148	9.49E-01	N/A	-	5.65E-07	2.89E-01	2.56E-03	7970	
9	rs2570961	8835429	1.69E-01	PTPRD	22	rs6008906	45330103	9.35E-01	CELSR1	-	5.65E-07	5.14E-01	9.38E-06	7971	
7	rs923336	1.35E+08	2.18E-01	LUZP6	13	rs9318901	82198270	2.33E-01	N/A	-	5.65E-07	8.14E-01	2.69E-04	7972	
11	rs10500628	4883523	1.97E-01	OR51A7	22	rs763217	43496258	6.23E-01	PRR5	-	5.65E-07	N/A	N/A	7973	
11	rs10500629	4883261	1.97E-01	OR51A7	22	rs763217	43496258	6.23E-01	PRR5	-	5.65E-07	N/A	N/A	7974	
1	rs10493817	88899475	6.20E-01	N/A	16	rs7187327	72734213	8.14E-02	N/A	-	5.65E-07	9.42E-01	1.19E-04	7975	
9	rs4604527	84714389	9.85E-01	N/A	9	s10991799	91843986	5.47E-01	N/A	-	5.65E-07	2.00E-02	2.24E-07	7976	
2	rs1193629	1.76E+08	8.87E-01	CHN1	5	rs4455541	83232076	4.71E-01	N/A	-	5.65E-07	4.53E-01	2.94E-06	7977	
1	rs1175111	1.9E+08	6.00E-01	N/A	12	s17091714	41380669	4.95E-01	N/A	-	5.65E-07	5.88E-01	3.56E-06	7978	
2	rs6755271	10427491	5.52E-01	HPCAL1	14	rs4901454	53372596	6.69E-01	N/A	-	5.65E-07	9.23E-01	1.17E-04	7979	
4	rs13112125	1.25E+08	1.58E-01	N/A	8	rs4531042	60251242	1.76E-01	N/A	-	5.65E-07	2.84E-01	1.70E-03	7980	
13	rs2164172	1.09E+08	4.20E-01	N/A	18	rs488173	32156598	7.31E-01	FHOD3	-	5.65E-07	6.20E-01	1.30E-05	7981	
2	rs4123497	46876947	1.86E-01	N/A	10	s16934609	54301082	4.91E-01	N/A	-	5.65E-07	6.92E-01	1.29E-04	7982	
14	rs198230	56311203	5.16E-01	N/A	17	rs882847	4329478	9.30E-02	SPNS2	-	5.65E-07	8.31E-01	6.64E-05	7983	
9	rs1332173	25490238	8.01E-01	N/A	16	rs899305	7140564	2.91E-01	RBFOX1	-	5.66E-07	2.42E-03	1.54E-08	7984	
6	rs989467	1.02E+08	8.65E-01	GRIK2	6	s13201016	1.57E+08	1.55E-01	N/A	-	5.66E-07	7.30E-02	1.22E-06	7985	
2	rs4853388	78502930	9.45E-01	N/A	9	rs871807	1.16E+08	9.50E-01	ZNF618	-	5.66E-07	4.19E-01	5.05E-04	7986	
3	rs3845917	1.23E+08	6.66E-01	CASR	10	rs2589446	56634203	2.40E-01	N/A	-	5.66E-07	6.12E-01	3.11E-04	7987	
1	rs10749738	63565743	7.57E-01	FOXD3	22	rs4822836	25768108	5.39E-01	N/A	-	5.66E-07	9.95E-01	1.25E-04	7988	
2	rs12621974	4871441	8.40E-02	N/A	8	rs7000712	1.33E+08	3.34E-01	EFR3A	-	5.66E-07	5.73E-01	8.38E-05	7989	
2	rs4146018	1.24E+08	6.30E-01	N/A	16	rs7199004	87195552	6.57E-01	ZC3H18	-	5.66E-07	7.85E-01	9.92E-05	7990	
1	rs922104	89814487	1.62E-01	LRRC8B	7	rs7793355	1.24E+08	2.39E-01	N/A	-	5.66E-07	3.37E-01	1.95E-03	7991	
7	rs1994464	1.35E+08	1.78E-01	N/A	13	rs9318901	82198270	2.33E-01	N/A	-	5.66E-07	8.16E-01	2.61E-04	7992	
17	rs12937692	39611956	4.58E-01	C17orf65	19	rs483808	7863481	2.21E-01	N/A	-	5.66E-07	8.74E-01	1.30E-04	7993	
2	rs13014896	1.72E+08	7.20E-01	GORASP2	8	s10956564	1.32E+08	5.01E-01	ADCY8	-	5.66E-07	1.27E-02	1.69E-07	7994	
10	rs11013317	18611417	5.40E-01	CACNB2	20	rs2180377	24072082	9.55E-01	N/A	-	5.66E-07	3.89E-02	2.08E-07	7995	
6	rs11754948	85759109	6.99E-01	N/A	20	rs1571167	53212274	2.49E-01	N/A	-	5.66E-07	2.07E-01	1.05E-03	7996	
11	rs4936353	1.16E+08	5.80E-01	N/A	14	rs2877672	70106012	3.91E-01	MED6	-	5.66E-07	1.04E-01	5.75E-07	7997	
6	rs4140634	1.2E+08	3.71E-01	MAN1A1	4	s13125519	1.81E+08	2.92E-01	N/A	-	5.66E-07	4.39E-01	1.48E-05	7998	
13	rs9543280	72700672	2.27E-02	N/A	20	rs4814019	11456980	9.36E-01	N/A	-	5.66E-07	9.13E-01	5.08E-04	7999	
2	rs7561984	2.3E+08	4.76E-01	PID1	3	rs6442831	3795517	9.99E-02	N/A	-	5.67E-07	7.98E-02	3.87E-06	8000	
2	rs7588090	2.3E+08	4.76E-01	PID1	3	rs6442831	3795517	9.99E-02	N/A	-	5.67E-07	7.98E-02	3.87E-06	8001	
10	rs2170643	1.27E+08	6.44E-01	MMP21	13	rs449520	76001336	1.46E-02	N/A	-	5.67E-07	9.84E-01	5.82E-05	8002	
7	rs17132513	2630740	8.24E-01	IQCE	10	s11254336	17115579	4.70E-01	CUBN	-	5.67E-07	1.84E-01	1.33E-06	8003	
8	rs10503236	3990061	8.99E-01	CSMD1	16	rs1599549	8085493	9.35E-01	N/A	-	5.67E-07	6.67E-01	2.79E-05	8004	
6	rs3757340	31029861	5.23E-03	DPCR1	6	rs2223971	78703467	2.39E-01	N/A	MHC	5.67E-07	6.73E-01	2.11E-04	8005	
6	rs7738087	939790	2.78E-01	LOC285768	3	rs6770575	68679728	3.20E-01	FAM19A1	-	5.67E-07	5.80E-02	4.46E-07	8006	
16	rs7196682	25415341	9.46E-01	N/A	17	s16951016	47559290	1.21E-01	CA10	-	5.67E-07	1.59E-01	7.98E-07	8007	
3	rs9866656	20701727	9.76E-01	N/A	12	s10160900	82187560	1.48E-01	N/A	-	5.67E-07	1.87E-01	3.36E-06	8008	
1	rs2816062	18776489	1.09E-01	N/A	13	s12869335	54242849	4.86E-01	N/A	-	5.67E-07	3.28E-01	1.09E-05	8009	
1	rs931590	1.86E+08	1.70E-01	N/A	17	rs2109174	14454035	2.07E-01	N/A	-	5.67E-07	4.16E-01	9.37E-06	8010	
3	rs12630901	84342357	9.03E-02	N/A	13	rs3015350	1.05E+08	3.67E-01	N/A	-	5.67E-07	5.71E-01	8.20E-05	8011	
12	rs4767535	1.16E+08	8.74E-01	NOS1	18	rs4566256	31651305	9.69E-01	N/A	-	5.67E-07	7.42E-01	7.95E-05	8012	
2	rs11901161	2.4E+08	6.64E-01	N/A	13	rs9559087	1.07E+08	9.50E-01	FAM155A	-	5.68E-07	4.07E-01	1.11E-03	8013	
1	rs640692	1.74E+08	2.04E-01	N/A	15	rs491274	44656818	2.01E-01	N/A	-	5.68E-07	4.53E-01	4.29E-04	8014	
8	rs2511692	1.04E+08	2.82E-01	N/A	13	rs7982613	75068514	7.59E-01	UCHL3	-	5.68E-07	5.94E-01	2.26E-05	8015	
11	rs11608125	85598629	8.35E-01	N/A	13	rs7330967	52407458	1.83E-01	N/A	-	5.68E-07	9.80E-01	1.91E-04	8016	
6	rs11154263	1.25E+08	2.17E-01	NKAIN2	20	rs1329820	16108141	9.79E-01	N/A	-	5.68E-07	4.23E-01	4.54E-05	8017	
3	rs10222671	1.07E+08	3.47E-01	N/A	16	rs8062451	81558637	8.96E-01	CDH13	-	5.68E-07	1.64E-01	1.82E-06	8018	
4	rs1486786	30261294	4.46E-01	N/A	10	rs2059996	77071681	6.20E-01	N/A	-	5.68E-07	3.65E-01	3.93E-03	8019	
14	rs17115288	44098618	7.06E-01	N/A	14	rs7156399	95104682	7.38E-01	N/A	-	5.68E-07	4.12E-01	1.28E-04	8020	
6	rs9397717	1.55E+08	6.24E-01	CNKSR3	12	rs1293739	1.12E+08	1.91E-01	OAS2	-	5.68E-07	5.58E-01	1.50E-04	8021	
7	rs11975489	77814840	4.58E-02	MAGI2	16	rs3924409	84585738	4.64E-01	N/A	-	5.68E-07	7.39E-01	3.66E-05	8022	
11	rs4910896	4118132	3.58E-01	RRM1	17	rs7209298	28877062	6.60E-01	ACCN1	-	5.68E-07	1.71E-01	1.26E-03	8023	
11	rs4910907	4125427	3.58E-01	RRM1	17	rs7209298	28877062	6.60E-01	ACCN1	-	5.68E-07	1.76E-01	1.21E-03	8024	
1	rs767654	18394276	4.61E-01	IGSF21	17	rs4789963	74741972	3.26E-01	HRNBP3	-	5.68E-07	5.05E-01	1.10E-04	8025	
6	rs1535616	1.34E+08	3.92E-01	MGC34034	5	rs6889043	1.47E+08	7.55E-02	N/A	-	5.69E-07	6.89E-01	1.58E-05	8026	
15	rs7171933	21765294	4.98E-01	N/A	17	rs4794737	52855906	3.47E-01	MSI2	-	5.69E-07	9.25E-01	1.30E-04	8027	
5	rs434192	5803925	6.11E-01	N/A	18	rs1941193	24840424	3.55E-01	N/A	-	5.69E-07	1.18E-01	6.72E-07	8028	
5	rs434192	5803925	6.11E-01	N/A	18	s10502527	24834125	3.55E-01	N/A	-	5.69E-07	1.19E-01	6.80E-07	8029	
6	rs807515	24627865	6.59E-03	ALDH5A1	7	s10233000	51661855	7.95E-01	N/A	-	5.69E-07	7.14E-01	4.97E-05	8030	
19	rs12459777	36105034	1.35E-01	N/A	20	rs6134656	1274001	2.07E-01	SDCBP2	-	5.69E-07	9.53E-01	2.53E-04	8031	
8	rs187089	15690991	9.82E-01	N/A	22	rs735455	20743793	9.48E-01	N/A	-	5.69E-07	7.69E-01	3.62E-05	8032	
10	rs2839658	31696055	5.20E-02	ZEB1	19	rs2864419	2134175	2.28E-01	DOT1L	-	5.69E-07	7.76E-01	2.95E-04	8033	
2	rs6714182	30853337	9.98E-01	CAPN13	5	rs6889916	22394616	6.58E-01	CDH12	-	5.69E-07	2.69E-01	1.26E-03	8034	
2	rs2578007	1.83E+08	4.69E-01	N/A	12	rs2645979	82541174	9.79E-01	N/A	-	5.69E-07	4.49E-01	2.93E-05	8035	
1	rs2997338	41644665	3.21E-02	N/A	5	s11960332	1.8E+08	6.87E-01	FLT4	-	5.69E-07	5.34E-03	1.97E-08	8036	
2	rs17016629	35506420	1.82E-01	N/A	7	rs1874326	1.38E+08	5.59E-01	TRIM24	-	5.69E-07	7.96E-01	2.71E-04	8037	
9	rs7864368	1.2E+08	3.17E-01	N/A	19	rs1235382	49711347	8.98E-01	CEACAM20	-	5.69E-07	8.84E-01	2.68E-04	8038	
1	rs7554629	79367862	4.81E-03	N/A	22	rs1573764	25415223	6.59E-01	MIAT	-	5.69E-07	N/A	N/A	8039	
1	rs7554629	79367862	4.81E-03	N/A	22	rs1573768	25415554	6.59E-01	MIAT	-	5.69E-07	N/A	N/A	8040	
1	rs7554629	79367862	4.81E-03	N/A	22	rs4820699	25418298	6.59E-01	MIAT	-	5.69E-07	N/A	N/A	8041	
1	rs7554629	79367862	4.81E-03	N/A	22	rs766071	25418786	6.59E-01	MIAT	-	5.69E-07	N/A	N/A	8042	
9	rs895229	3873042	1.04E-01	GLIS3	15	rs7172407	71637454	6.54E-01	C15orf60	-	5.69E-07	2.51E-01	1.81E-05	8043	
2	rs1346620	99819360	2.43E-01	AFF3	8	rs3885690	10864279	5.90E-01	XKR6	-	5.69E-07	7.69E-01	2.06E-04	8044	
4	rs7681428	1.72E+08	6.90E-01	N/A	7	rs2215040	1.02E+08	7.37E-03	FBXL13	-	5.69E-07	9.21E-01	1.69E-04	8045	
3	rs6442159	10218304	5.86E-01	IRAK2	13	rs7999797	1.09E+08	6.89E-01	IRS2	-	5.69E-07	9.26E-01	2.73E-05	8046	
1	rs12142922	89262503	9.20E-02	GBP3	13	rs2321997	58561359	4.61E-01	N/A	-	5.69E-07	1.80E-01	1.35E-06	8047	
7	rs10480737	79005941	2.99E-01	N/A	18	rs4346287	60381151	5.74E-01	N/A	-	5.69E-07	8.11E-01	1.89E-05	8048	
5	rs1501841	60983240	7.44E-01	FLJ37543	9	rs4978738	1.11E+08	3.81E-01	N/A	-	5.69E-07	8.88E-01	2.54E-04	8049	
5	rs13179657	86982256	9.66E-01	N/A	10	rs1931901	7695358	1.69E-01	ITIH5	-	5.69E-07	9.44E-01	3.28E-05	8050	
5	rs16902739	87040272	9.66E-01	N/A	10	rs1931901	7695358	1.69E-01	ITIH5	-	5.69E-07	9.44E-01	3.28E-05	8051	
1	rs12239182	2.02E+08	9.37E-01	SOX13	10	s10822248	65346801	1.22E-02	N/A	-	5.70E-07	1.59E-01	2.39E-06	8052	
2	rs895514	43064710	7.97E-01	N/A	14	s11157503	45963326	6.99E-01	N/A	-	5.70E-07	8.40E-01	7.02E-05	8053	
2	rs465119	80329653	5.48E-01	CTNN/A2	9	rs4836656	1.31E+08	7.18E-01	N/A	-	5.70E-07	3.93E-01	5.29E-06	8054	
1	rs6695161	41178251	2.96E-01	N/A	4	s13128971	44275968	7.37E-01	N/A	-	5.70E-07	6.11E-01	2.61E-04	8055	
6	rs9495051	1.39E+08	8.49E-01	N/A	2	rs7569111	2.36E+08	1.62E-01	AGAP1	-	5.70E-07	7.78E-01	1.11E-04	8056	
5	rs11949795	18999415	8.81E-01	N/A	5	rs34236	1.03E+08	7.49E-01	N/A	-	5.70E-07	9.21E-01	3.89E-04	8057	
5	rs7720020	19001099	8.81E-01	N/A	5	rs34236	1.03E+08	7.49E-01	N/A	-	5.70E-07	9.21E-01	3.89E-04	8058	
8	rs2410319	15883488	5.34E-01	N/A	10	s11018401	1.3E+08	4.63E-01	PTPRE	-	5.70E-07	7.15E-01	6.13E-05	8059	
2	rs10167695	50912249	6.42E-01	NRXN1	3	rs2639210	81196639	3.61E-01	N/A	-	5.70E-07	9.26E-01	1.12E-04	8060	
2	rs842064	1.6E+08	2.17E-01	TANC1	17	rs2083797	42276928	3.93E-01	WNT9B	-	5.70E-07	1.19E-01	1.04E-07	8061	
10	rs874758	88090069	1.75E-01	GRID1	10	s10509835	1.09E+08	9.75E-01	N/A	-	5.70E-07	1.90E-01	7.14E-06	8062	

SNP A					SNP B						Interaction P			Ranking	Cluster in top 100 interactions
CHR	SNP	Location	gle locus P va	Gene	CHR	SNP	Location	ingle locus P valu	Gene	HC region	Stage 1	Stage 2	Combined		
11	rs11232180	79914436	3.63E-01	N/A	14	rs6573656	65780836	7.09E-02	N/A	-	5.70E-07	2.31E-01	8.65E-03	8063	
3	rs6789465	43907049	2.27E-01	N/A	8	rs3862082	17514969	2.66E-01	PDGFRL	-	5.70E-07	9.42E-02	1.12E-02	8064	
8	rs3864665	62093567	7.26E-01	N/A	22	rs6001027	36875565	2.10E-01	PLA2G6	-	5.70E-07	5.28E-01	1.57E-05	8065	
6	rs1343244	82133707	5.14E-01	N/A	4	rs2305949	55675213	6.54E-01	KDR	-	5.70E-07	4.04E-02	5.54E-03	8066	
6	rs9352999	82125494	5.14E-01	N/A	4	rs2305949	55675213	6.54E-01	KDR	-	5.70E-07	4.04E-02	5.54E-03	8067	
4	rs2868382	83756330	2.88E-01	SCD5	9	rs1358907	3158478	2.43E-01	N/A	-	5.70E-07	2.01E-01	2.37E-03	8068	
16	rs12929721	82030355	1.07E-02	CDH13	16	rs731258	82271433	4.87E-01	CDH13	-	5.70E-07	4.98E-01	2.98E-04	8069	
6	rs2504892	12667225	5.86E-01	N/A	7	rs12698670	67256918	8.26E-01	N/A	-	5.70E-07	8.99E-01	3.22E-05	8070	
4	rs1563844	60541497	6.14E-01	N/A	7	rs2192017	1.22E+08	8.28E-01	CADPS2	-	5.70E-07	9.84E-01	1.80E-04	8071	
13	rs953937	89320971	6.27E-01	N/A	14	rs1551013	84077293	1.21E-01	N/A	-	5.71E-07	2.57E-02	1.81E-07	8072	
1	rs10889294	62402950	9.72E-01	IN/ADL	18	rs7237761	958021	5.30E-01	N/A	-	5.71E-07	3.37E-01	4.05E-06	8073	
4	rs10938692	8169461	3.72E-01	ABLIM2	20	rs962814	55822433	3.92E-01	N/A	-	5.71E-07	2.82E-01	7.99E-04	8074	
4	rs7670919	1.75E+08	6.73E-01	N/A	8	rs12677963	1.31E+08	7.69E-01	N/A	-	5.71E-07	6.32E-01	1.95E-05	8075	
4	rs7670919	1.75E+08	6.73E-01	N/A	8	rs6470742	1.31E+08	7.69E-01	N/A	-	5.71E-07	6.51E-01	2.14E-05	8076	
8	rs1825000	61372500	4.19E-01	CA8	14	rs2184282	19950000	9.09E-01	TEP1	-	5.71E-07	2.28E-01	1.15E-05	8077	
8	rs7842871	61372013	4.19E-01	CA8	14	rs2184282	19950000	9.09E-01	TEP1	-	5.71E-07	2.28E-01	1.15E-05	8078	
5	rs961536	75948599	9.95E-01	IQGAP2	8	rs2223053	1.17E+08	2.97E-01	N/A	-	5.71E-07	7.81E-01	1.40E-04	8079	
12	rs4763947	13261503	3.31E-02	EMP1	20	rs2423107	5433138	2.18E-01	LOC149837	-	5.71E-07	3.98E-01	1.48E-05	8080	
1	rs4926345	68733064	5.87E-01	DEPDC1	14	rs17123886	87431359	4.36E-01	N/A	-	5.71E-07	5.96E-01	1.88E-04	8081	
1	rs7527841	68708712	5.87E-01	DEPDC1	14	rs17123886	87431359	4.36E-01	N/A	-	5.71E-07	5.96E-01	1.88E-04	8082	
5	rs4360024	77719962	5.37E-01	SCAMP1	11	rs1800849	73397813	6.34E-01	UCP3	-	5.71E-07	6.44E-01	2.79E-05	8083	
2	rs10932374	2.12E+08	3.38E-01	ERBB4	13	rs9517097	97307303	1.40E-01	N/A	-	5.71E-07	6.83E-01	4.16E-05	8084	
4	rs1435313	1.34E+08	6.31E-01	N/A	14	rs17124069	51054331	1.13E-01	FRMD6	-	5.71E-07	1.69E-01	4.81E-06	8085	
11	rs10891201	1.1E+08	4.36E-01	N/A	19	rs7507911	7249095	2.70E-01	INSR	-	5.71E-07	9.16E-01	4.64E-04	8086	
1	rs12082808	2.37E+08	1.05E-01	N/A	4	rs6842810	1.43E+08	9.88E-01	N/A	-	5.71E-07	3.34E-01	2.40E-04	8087	
5	rs216535	1.11E+08	6.28E-01	CAMK4	9	rs7035592	27224029	1.27E-01	NCRN/A00032	-	5.71E-07	5.57E-01	1.85E-04	8088	
1	rs10874095	80101868	1.24E-01	N/A	10	rs10795059	3582082	5.85E-01	N/A	-	5.71E-07	7.22E-02	1.76E-06	8089	
4	rs4385097	63426495	8.33E-01	N/A	16	rs9928573	10728243	9.73E-01	NUBP1	-	5.71E-07	3.08E-01	1.05E-03	8090	
12	rs7296020	61595594	4.84E-01	PPM1H	13	rs1411517	91448474	7.42E-01	GPC5	-	5.71E-07	9.13E-01	2.47E-04	8091	
3	rs7648830	66997448	8.94E-02	N/A	10	rs1113313	76683707	5.97E-01	COMTD1	-	5.71E-07	9.39E-01	4.65E-05	8092	
3	rs1154631	16667935	3.44E-01	N/A	8	rs10505328	1.19E+08	8.38E-01	N/A	-	5.72E-07	2.45E-01	7.16E-04	8093	
12	rs7959574	96615419	5.75E-01	N/A	15	rs12915737	30871133	4.53E-02	FMN1	-	5.72E-07	3.99E-01	6.22E-04	8094	
8	rs7003858	20046275	5.18E-01	SLC18A1	15	rs12595143	51572775	5.95E-01	N/A	-	5.72E-07	4.97E-01	1.17E-05	8095	
11	rs1528658	13946856	7.85E-02	SPON1	14	rs3829948	92186579	5.37E-01	RIN3	-	5.72E-07	2.63E-01	4.25E-05	8096	
6	rs1888274	53040467	5.50E-01	FBXO9	3	rs10936353	1.65E+08	7.88E-01	N/A	-	5.72E-07	6.38E-01	3.74E-05	8097	
5	rs10515804	1.59E+08	2.53E-03	TTC1	10	rs7895170	1.16E+08	3.54E-01	DCLRE1A	-	5.72E-07	1.70E-01	2.59E-06	8098	
10	rs7917581	73851763	6.43E-01	MIR1256	15	rs4522364	48129516	5.11E-02	ATP8B4	-	5.72E-07	2.97E-01	5.18E-03	8099	
2	rs2311552	1.12E+08	3.74E-01	N/A	8	rs7002414	62820244	8.72E-01	N/A	-	5.72E-07	3.53E-01	5.79E-06	8100	
7	rs2464614	3590577	2.15E-01	SDK1	19	rs741448	34429788	7.00E-01	N/A	-	5.72E-07	5.27E-01	5.59E-04	8101	
9	rs1933679	95748843	8.32E-01	BARX1	11	rs4939456	60319217	3.57E-02	MS4A10	-	5.72E-07	1.06E-01	2.44E-06	8102	
1	rs12407446	1.57E+08	6.61E-01	CD1A	18	rs1940435	407559	6.83E-01	COLEC12	-	5.72E-07	7.48E-01	1.10E-04	8103	
6	rs1876155	1.01E+08	7.67E-01	N/A	1	rs4233164	1.74E+08	5.50E-01	TNR	-	5.72E-07	5.93E-01	1.21E-04	8104	
5	rs4502857	1.2E+08	2.78E-01	N/A	11	rs1982496	1.06E+08	2.75E-01	GUCY1A2	-	5.73E-07	9.60E-01	1.15E-04	8105	
2	rs2033137	1.85E+08	5.32E-01	N/A	3	rs9789945	31452286	4.10E-01	N/A	-	5.73E-07	2.33E-01	5.18E-06	8106	
7	rs4732134	1.35E+08	6.11E-01	NUP205	13	rs2419887	25549661	7.29E-01	N/A	-	5.73E-07	7.96E-01	5.65E-05	8107	
1	rs10803421	13829071	8.81E-01	PDPN	11	rs12364154	70029656	5.09E-01	SHANK2	-	5.73E-07	4.83E-01	2.21E-06	8108	
6	rs9385975	1.01E+08	6.33E-01	MCHR2	11	rs10791957	67624124	6.97E-02	CHKA	-	5.73E-07	7.14E-01	6.75E-05	8109	
2	rs4954112	1.35E+08	8.12E-01	N/A	21	rs2832392	29840088	2.51E-01	GRIK1	-	5.73E-07	6.92E-01	1.27E-05	8110	
2	rs10184017	2658488	4.51E-01	N/A	14	rs10142843	28784615	8.66E-01	N/A	-	5.73E-07	9.51E-01	2.83E-05	8111	
8	rs10441655	13794892	3.21E-01	N/A	12	rs11114975	80588517	4.17E-01	PPFIA2	-	5.73E-07	1.38E-01	1.71E-03	8112	
1	rs2821330	1.98E+08	1.67E-02	NR5A2	2	rs7587759	1.99E+08	6.59E-01	N/A	-	5.73E-07	4.70E-01	1.09E-03	8113	
8	rs1033345	1248521	4.69E-01	N/A	13	rs9554684	99838057	6.66E-01	PCCA	-	5.73E-07	8.89E-01	3.14E-05	8114	
8	rs754539	18631372	5.96E-01	PSD3	18	rs12607553	26841557	7.22E-01	DSC3	-	5.73E-07	9.78E-01	6.22E-05	8115	
1	rs202248	1.66E+08	7.63E-01	DCAF6	18	rs732034	13793344	6.02E-01	N/A	-	5.74E-07	4.96E-01	1.15E-05	8116	
16	rs4787394	26874852	3.03E-01	N/A	20	rs17770331	1563308	9.57E-01	SIRPB1	-	5.74E-07	6.40E-01	5.42E-04	8117	
6	rs9385975	1.01E+08	6.33E-01	MCHR2	11	rs7928739	67590119	9.00E-02	CHKA	-	5.74E-07	7.14E-01	6.39E-05	8118	
1	rs4844678	2.07E+08	7.95E-02	N/A	10	rs715687	84561933	8.55E-01	NRG3	-	5.74E-07	8.84E-01	1.14E-04	8119	
10	rs3858304	1.32E+08	5.58E-01	N/A	20	rs8114499	8069097	9.79E-01	PLCB1	-	5.74E-07	5.60E-01	3.92E-05	8120	
7	rs7793289	13073946	6.14E-02	N/A	19	rs4801457	62485375	9.79E-01	ZNF460	-	5.74E-07	5.52E-01	8.38E-06	8121	
7	rs2160193	13070161	6.14E-02	N/A	19	rs4801457	62485375	9.79E-01	ZNF460	-	5.74E-07	5.71E-01	9.30E-06	8122	
9	rs2417226	1.03E+08	2.10E-01	N/A	14	rs1956218	32144994	4.70E-02	AKAP6	-	5.74E-07	8.81E-01	6.57E-05	8123	
2	rs6746998	2.09E+08	1.89E-01	N/A	7	rs6461503	20527521	1.19E-01	N/A	-	5.74E-07	4.48E-01	5.00E-04	8124	
14	rs10147954	66486836	1.29E-02	GPHN	16	rs12449282	10265020	5.28E-01	N/A	-	5.74E-07	9.62E-01	1.90E-04	8125	
1	rs4652795	1.82E+08	7.52E-01	NMN/AT2	8	rs6994396	1.15E+08	8.90E-01	N/A	-	5.74E-07	2.58E-01	9.09E-06	8126	
6	rs2508015	31118179	8.45E-01	HCG22	6	rs4495304	31188697	4.80E-02	C6orf15	MHC	5.74E-07	1.09E-02	1.06E-07	8127	
2	rs6726480	2.05E+08	8.63E-01	PARD3B	19	rs4807863	6257971	8.37E-02	ACER1	-	5.74E-07	2.28E-01	4.33E-06	8128	
6	rs1570155	20490579	3.98E-02	E2F3	18	rs1587989	60554118	8.65E-01	N/A	-	5.74E-07	1.01E-01	1.25E-06	8129	
3	rs7624435	97082944	2.66E-01	N/A	12	rs10784285	60844581	2.64E-01	FAM19A2	-	5.74E-07	1.10E-01	2.84E-03	8130	
2	rs2528626	1.59E+08	1.37E-01	N/A	20	rs2249634	54904032	1.71E-01	N/A	-	5.74E-07	2.98E-01	2.75E-06	8131	
7	rs2714862	17914665	3.91E-01	SNX13	14	rs734606	94272240	2.92E-01	N/A	-	5.74E-07	3.55E-01	1.95E-03	8132	
7	rs2714862	17914665	3.91E-01	SNX13	14	rs8006054	94273061	2.92E-01	N/A	-	5.74E-07	3.55E-01	1.95E-03	8133	
8	rs10108007	61310616	4.24E-01	CA8	16	rs1862746	59019859	7.18E-01	N/A	-	5.75E-07	8.23E-01	7.64E-05	8134	
2	rs1400795	2.24E+08	1.76E-02	N/A	7	rs1477250	1.31E+08	4.99E-02	PODXL	-	5.75E-07	9.97E-01	9.49E-05	8135	
5	rs4998063	79636449	8.89E-01	LOC644936	17	rs11077598	67786460	2.04E-01	N/A	-	5.75E-07	6.20E-02	3.48E-07	8136	
5	rs4702704	10644672	3.92E-01	ANKRD33B	11	rs11030669	29330054	9.44E-01	N/A	-	5.75E-07	1.08E-01	2.78E-03	8137	
5	rs1502638	3802205	7.33E-01	N/A	13	rs12427758	33949169	2.14E-01	N/A	-	5.75E-07	7.63E-01	8.23E-05	8138	
6	rs313210	86065675	5.72E-01	N/A	10	rs7921890	5355630	1.82E-01	N/A	-	5.75E-07	8.67E-01	8.41E-05	8139	
6	rs17143510	8239017	9.52E-01	N/A	9	rs11243810	1.34E+08	4.63E-01	C9orf171	-	5.75E-07	9.65E-01	6.00E-05	8140	
1	rs11211212	46114226	2.24E-01	MAST2	16	rs1046623	83157772	1.15E-01	COTL1	-	5.75E-07	3.04E-01	5.88E-04	8141	
2	rs12614105	28528629	8.58E-01	N/A	12	rs1669925	41172484	8.84E-01	PRICKLE1	-	5.75E-07	5.18E-01	3.19E-05	8142	
4	rs1268789	79499717	8.75E-01	FRAS1	11	rs6578870	7519655	5.27E-01	PPFIBP2	-	5.75E-07	9.35E-01	2.14E-04	8143	
4	rs375726	79500774	8.75E-01	FRAS1	11	rs6578870	7519655	5.27E-01	PPFIBP2	-	5.75E-07	9.35E-01	2.14E-04	8144	
7	rs181047	87962850	6.03E-01	N/A	18	rs154899	56949141	5.31E-01	N/A	-	5.75E-07	7.97E-01	6.59E-04	8145	
7	rs181049	87960886	6.03E-01	N/A	18	rs154899	56949141	5.31E-01	N/A	-	5.75E-07	8.80E-01	5.27E-04	8146	
1	rs703797	14538729	7.23E-01	N/A	14	rs2812086	37728519	8.37E-01	SSTR1	-	5.75E-07	1.63E-01	4.05E-04	8147	
11	rs1528658	13946856	7.85E-02	SPON1	14	rs11629324	92190472	7.82E-01	RIN3	-	5.75E-07	4.07E-01	1.41E-04	8148	
3	rs2367773	63222968	8.24E-01	SYNPR	4	rs11100291	1.62E+08	8.92E-01	N/A	-	5.75E-07	2.11E-01	1.82E-03	8149	
2	rs17538082	2.09E+08	1.91E-01	N/A	7	rs10273717	20519346	1.20E-01	N/A	-	5.75E-07	3.08E-01	9.31E-04	8150	
4	rs354849	1.42E+08	8.33E-01	N/A	14	rs2356914	50986010	5.29E-01	N/A	-	5.75E-07	4.49E-01	1.17E-03	8151	
4	rs4692446	29853056	4.28E-01	N/A	5	rs2910472	61466188	7.44E-01	N/A	-	5.75E-07	7.76E-01	3.22E-05	8152	
4	rs4975057	80707369	7.87E-02	N/A	7	rs2390961	24266172	2.98E-01	N/A	-	5.76E-07	2.96E-01	1.05E-05	8153	
5	rs415024	9445358	5.95E-02	SEMA5A	18	rs999146	65702326	4.85E-01	CD226	-	5.76E-07	4.14E-01	2.42E-04	8154	
1	rs11185168	1.08E+08	1.12E-01	VAV3	14	rs4906007	1E+08	1.09E-02	N/A	-	5.76E-07	6.85E-01	4.71E-05	8155	
13	rs4512966	1.1E+08	7.15E-01	COL4A2	14	rs438931	76816063	1.14E-01	MIR1260	-	5.76E-07	1.67E-01	1.82E-06	8156	
9	rs11144766	77954821	6.42E-01	N/A	11	rs984371	55334274	3.18E-01	OR5D14	-	5.76E-07	5.07E-01	5.30E-04	8157	
4	rs4585357	1.9E+08	2.38E-01	N/A	12	rs4387423	81941727	7.51E-01	TMTC2	-	5.76E-07	5.38E-01	6.76E-04	8158	
4	rs10032655	1.9E+08	2.38E-01	N/A	12	rs4387423	81941727	7.51E-01	TMTC2	-	5.76E-07	5.38E-01	6.66E-04	8159	
5	rs6871540	23896821	3.05E-02	N/A	10	rs1417096	16187485	5.17E-01	N/A	-	5.76E-07	4.33E-01	5.03E-04	8160	
10	rs12766032	1.17E+08	5.38E-01	ATRNL1	13	rs2485247	84507278	3.47E-02	N/A	-	5.76E-07	7.87E-01	1.31E-03	8161	
6	rs9401123	98289833	1.34E-01	N/A	8	rs1544844	72681728	4.86E-01	N/A	-	5.76E-07	8.47E-01	6.38E-05	8162	
2	rs1879948	1.76E+08	1.05E-01	N/A	22	rs1894707	25189086	5.21E-01	HPS4	-	5.76E-07	9.46E-01	1.89E-04	8163	
6	rs590641	81023202	1.86E-01	BCKDHB	4	rs10020692	1.68E+08	8.89E-01	SPOCK3	-	5.76E-07	9.70E-01	1.17E-04	8164	
6	rs1575536	93966647	2.34E-01	N/A	10	rs6585251	1.16E+08	8.22E-01	NHLRC2	-	5.76E-07	9.58E-01	4.55E-05	8165	
10	rs3125309	69152443	3.20E-01	N/A	12	rs886540	1461019	3.82E-01	ERC1	-	5.76E-07	6.16E-01	3.50E-04	8166	
13	rs3956272	28447099	2.96E-01	N/A	19	rs8103241	12983612	6.34E-02	NFIX	-	5.76E-07	7.93E-01	1.01E-04	8167	
10	rs874758	88090069	1.75E-01	GRID1	10	rs1416410	1.09E+08	4.16E-01	N/A	-	5.76E-07	9.76E-01	2.59E-04	8168	
4	rs1689004	1.81E+08	3.97E-01	N/A	5	rs2963265	1423832	9.68E-01	N/A	-	5.76E-07	9.82E-01	5.27E-05	8169	
5	rs350089	40210203	2.93E-01	N/A	16	rs4780954	10589498	1.60E-01	EMP2	-	5.76E-07	8.95E-01	1.14E-04	8170	
1	rs11804163	36757321	2.49E-01	N/A	3	rs9864105	54393641	8.05E-01	CACN/A2D3	-	5.76E-07	4.94E-01	9.31E-05	8171	
2	rs797700	47801605	8.82E-01	N/A	3	rs6438537	1.21E+08	4.07E-01	CD80	-	5.76E-07	8.06E-01	5.02E-05	8172	
6	rs16868805	62782774	1.55E-01	KHDRBS2	13	rs1618139	1.03E+08	9.11E-02	N/A	-	5.77E-07	3.60E-02	2.86E-03	8173	
8	rs890014	5835084	7.22E-01	N/A	12	rs2108639	1843645	3.17E-01	CACN/A2D4	-	5.77E-07	2.97E-01	5.16E-06	8174	

SNP A					SNP B						Interaction P			Ranking	Cluster in top 100 interactions
CHR	SNP	Location	gle locus P va	Gene	CHR	SNP	Location	ingle locus P valu	Gene	HC region	Stage 1	Stage 2	Combined		
4	rs2202507	1.45E+08	5.74E-01	N/A	12	rs7966207	1.01E+08	9.55E-01	GNPTAB	-	5.77E-07	5.17E-01	9.29E-06	8175	
6	rs1737727	35012466	6.45E-01	ANKS1A	9	rs407791	32012207	1.35E-01	N/A	MHC	5.77E-07	3.35E-02	1.29E-02	8176	
3	rs1494837	27695237	1.75E-01	N/A	8	rs1866788	1.01E+08	1.07E-01	SPAG1	-	5.77E-07	1.66E-01	2.71E-03	8177	
6	rs1737727	35012466	6.45E-01	ANKS1A	9	rs287572	31994713	1.35E-01	N/A	MHC	5.77E-07	1.71E-01	1.19E-03	8178	
8	rs6984093	1.25E+08	4.00E-01	FER1L6	17	rs7218965	57622877	7.10E-01	N/A	-	5.77E-07	1.71E-01	1.19E-03	8179	
8	rs2511733	1.04E+08	9.45E-01	ODF1	18	rs584970	72176763	8.92E-01	N/A	-	5.77E-07	1.71E-01	1.19E-03	8180	
10	rs7100844	1.33E+08	9.56E-01	N/A	11	rs800140	2290251	6.46E-02	C11orf21	-	5.77E-07	8.88E-01	5.56E-05	8181	
4	rs1364886	1.42E+08	4.87E-01	RNF150	20	rs2207542	59609117	8.19E-01	CDH4	-	5.77E-07	9.60E-01	3.71E-05	8182	
6	rs1737727	35012466	6.45E-01	ANKS1A	9	rs287573	31994571	1.35E-01	N/A	MHC	5.77E-07	N/A	N/A	8183	
6	rs1737727	35012466	6.45E-01	ANKS1A	9	rs453809	32006697	1.35E-01	N/A	MHC	5.77E-07	N/A	N/A	8184	
1	rs6656611	16388392	4.78E-01	ARHGEF19	2	rs17447598	2.01E+08	6.92E-01	SPATS2L	-	5.77E-07	1.30E-01	2.21E-03	8185	
7	rs6969674	18929863	5.77E-02	HDAC9	10	rs7070665	70919814	8.87E-01	TSPAN15	-	5.77E-07	2.54E-01	1.48E-03	8186	
3	rs6441301	1.61E+08	1.14E-01	N/A	8	rs1425902	54281767	6.56E-02	OPRK1	-	5.77E-07	3.74E-01	2.82E-06	8187	
12	rs1396226	73586112	5.53E-01	N/A	15	rs716980	37161386	6.34E-02	N/A	-	5.77E-07	8.17E-01	2.02E-04	8188	
1	rs2276401	1.57E+08	7.22E-01	SPTA1	21	rs2833476	31969189	6.31E-01	SFRS15	-	5.77E-07	4.35E-01	1.42E-05	8189	
1	rs6702660	4923290	9.64E-01	N/A	13	rs4941866	36880118	6.94E-01	N/A	-	5.77E-07	5.53E-03	3.42E-02	8190	
4	rs10022598	1.3E+08	5.74E-01	N/A	9	rs884540	8250902	2.32E-01	N/A	-	5.77E-07	1.44E-01	1.80E-03	8191	
9	rs10819641	1.01E+08	5.52E-01	TGFBR1	10	rs2366878	1.28E+08	7.87E-02	N/A	-	5.77E-07	1.53E-01	5.06E-03	8192	
1	rs17371526	85299361	8.46E-01	WDR63	8	rs17270769	1.25E+08	6.56E-01	FER1L6	-	5.77E-07	9.21E-01	7.85E-05	8193	
7	rs4719152	70669405	8.16E-02	WBSCR17	9	rs3789311	1.23E+08	7.66E-01	CEP110	-	5.77E-07	9.28E-02	3.92E-07	8194	
16	rs8060937	81277337	3.78E-02	CDH13	17	rs4796274	32183977	5.59E-01	N/A	-	5.78E-07	2.90E-01	6.02E-04	8195	
5	rs889268	37917929	2.47E-01	N/A	12	rs2900554	11025968	4.75E-01	PRH1	-	5.78E-07	6.77E-01	2.59E-04	8196	
11	rs2403441	19479964	3.13E-01	N/AV2	18	rs12954266	18612312	2.06E-01	N/A	-	5.78E-07	7.00E-01	1.92E-04	8197	
1	rs1031675	2.2E+08	1.27E-01	N/A	11	rs7103207	3353807	5.87E-02	LOC650368	-	5.78E-07	7.31E-01	2.42E-04	8198	
2	rs4675141	2.28E+08	6.97E-01	N/A	8	rs4243888	1.39E+08	7.31E-01	FAM135B	-	5.78E-07	1.14E-01	5.92E-07	8199	
7	rs3110788	1.35E+08	9.18E-01	SLC13A4	16	rs1478705	7086270	7.88E-01	A2BP1	-	5.78E-07	2.11E-01	9.64E-04	8200	
2	rs10174591	2.28E+08	6.98E-01	COL4A4	8	rs4243888	1.39E+08	7.31E-01	FAM135B	-	5.78E-07	1.14E-01	5.92E-07	8201	
1	rs4078315	2.17E+08	1.56E-01	N/A	20	rs12624817	7870861	9.87E-01	HAO1	-	5.78E-07	5.47E-01	6.08E-04	8202	
1	rs7517054	1.96E+08	4.15E-01	NEK7	12	rs7960795	1.01E+08	1.61E-01	GNPTAB	-	5.78E-07	6.93E-01	8.14E-06	8203	
9	rs10511900	32285707	2.21E-01	N/A	13	rs9565053	73330323	7.69E-01	KLF12	-	5.78E-07	7.16E-01	2.71E-05	8204	
2	rs1834619	17764966	5.69E-01	SMC6	8	rs13254921	34760826	6.57E-01	N/A	-	5.78E-07	5.09E-02	1.31E-02	8205	
2	rs1834619	17764966	5.69E-01	SMC6	8	rs2685607	34755686	6.57E-01	N/A	-	5.78E-07	5.09E-02	1.31E-02	8206	
14	rs176338	28081367	8.18E-01	N/A	15	rs8043356	58803461	7.65E-01	RORA	-	5.78E-07	6.69E-01	1.16E-03	8207	
10	rs431673	90484145	5.54E-01	LIPK	21	rs2834278	34141871	2.22E-01	ITSN1	-	5.78E-07	2.12E-01	7.25E-03	8208	
4	rs2158199	1.14E+08	4.02E-02	ANK2	13	rs9318552	77923788	2.86E-01	N/A	-	5.78E-07	2.96E-01	8.86E-04	8209	
1	rs3806365	1.61E+08	1.45E-01	RGS5	5	rs7448547	1.08E+08	7.00E-01	N/A	-	5.78E-07	8.05E-01	2.52E-04	8210	
1	rs3806368	1.61E+08	1.45E-01	RGS5	5	rs7448547	1.08E+08	7.00E-01	N/A	-	5.78E-07	8.42E-01	2.86E-04	8211	
11	rs11037175	42927616	1.95E-01	N/A	13	rs4943461	36730514	8.97E-01	N/A	-	5.78E-07	4.84E-01	2.25E-05	8212	
2	rs2861680	79006430	1.64E-01	N/A	11	rs11228719	56196812	5.52E-01	OR8U8	-	5.78E-07	5.58E-01	2.74E-04	8213	
10	rs2457857	14100685	3.57E-01	FRMD4A	12	rs2263487	45537094	2.68E-02	N/A	-	5.78E-07	7.35E-01	1.84E-05	8214	
8	rs17755518	2583513	6.43E-01	N/A	12	rs2277383	50600655	3.61E-01	ACVRL1	-	5.79E-07	2.86E-01	1.52E-03	8215	
1	rs2282431	36557857	1.32E-01	THRAP3	7	rs7799478	98519158	2.19E-01	SMURF1	-	5.79E-07	5.09E-01	4.46E-04	8216	
1	rs7418327	86428028	6.59E-01	N/A	12	rs11107877	93970892	1.50E-01	NR2C1	-	5.79E-07	2.63E-01	7.78E-06	8217	
10	rs10884387	1.09E+08	4.56E-01	SORCS1	17	rs998180	47751533	2.92E-01	N/A	-	5.79E-07	8.29E-01	7.56E-05	8218	
5	rs2783487	54453150	4.94E-01	CDC20B	19	rs415647	3074113	9.77E-01	GN/A15	-	5.79E-07	2.21E-01	1.27E-03	8219	
6	rs6925151	1.5E+08	4.69E-01	RAET1E	10	rs7067847	1.23E+08	8.36E-01	N/A	-	5.79E-07	2.64E-01	1.01E-05	8220	
1	rs1879744	2.39E+08	6.03E-01	RGS7	8	rs1022430	75373718	8.22E-01	JPH1	-	5.79E-07	4.38E-01	4.82E-06	8221	
6	rs472072	12745272	5.47E-01	N/A	12	rs11060728	1.29E+08	9.40E-01	N/A	-	5.79E-07	3.35E-01	1.31E-05	8222	
3	rs13072528	29271196	3.61E-01	N/A	4	rs4689050	6814016	9.17E-01	N/A	-	5.79E-07	5.05E-01	6.47E-06	8223	
1	rs274587	74716053	5.38E-01	TNNI3K	1	rs11164301	1.02E+08	2.21E-01	N/A	-	5.79E-07	8.34E-01	2.34E-04	8224	
1	rs274604	74729913	5.38E-01	TNNI3K	1	rs11164301	1.02E+08	2.21E-01	N/A	-	5.79E-07	8.34E-01	2.34E-04	8225	
6	rs6926382	1.62E+08	9.98E-01	AGPAT4	2	rs2861754	67931211	5.93E-02	N/A	-	5.79E-07	7.73E-01	3.00E-04	8226	
4	rs704352	2456284	8.39E-01	N/A	16	rs2920706	55015988	9.20E-01	AMFR	-	5.79E-07	1.41E-01	7.05E-04	8227	
3	rs12493232	77495032	5.37E-01	ROBO2	11	rs2239695	1.18E+08	6.63E-01	CD3D	-	5.79E-07	5.92E-01	3.58E-04	8228	
1	rs12036568	2.12E+08	2.79E-01	N/A	19	rs10426302	59903359	1.92E-01	LILRP2	-	5.79E-07	1.56E-01	9.93E-04	8229	
15	rs10152229	99780180	3.42E-01	PCSK6	21	rs8128155	39210983	8.62E-01	N/A	-	5.79E-07	8.81E-01	2.50E-04	8230	
6	rs7739011	3057529	1.89E-01	BPHL	5	rs10514161	79222927	1.66E-01	N/A	-	5.80E-07	2.93E-01	2.75E-04	8231	
1	rs7535209	12572663	3.54E-01	DHRS3	16	rs12597345	61798718	6.13E-01	N/A	-	5.80E-07	6.00E-01	6.10E-04	8232	
1	rs7535209	12572663	3.54E-01	DHRS3	16	rs12930214	61798839	6.13E-01	N/A	-	5.80E-07	6.00E-01	6.10E-04	8233	
4	rs6554340	56822605	3.67E-01	KIAA1211	10	rs11004720	56494729	2.12E-01	N/A	-	5.80E-07	3.64E-01	1.53E-03	8234	
10	rs7074840	1.28E+08	1.63E-01	N/A	12	rs12578390	42010813	5.20E-01	N/A	-	5.80E-07	3.53E-01	1.15E-03	8235	
3	rs10933931	1.09E+08	8.65E-01	CD47	19	rs501168	3697087	6.17E-01	APBA3	-	5.80E-07	8.49E-01	9.60E-05	8236	
1	rs12037907	67745023	9.25E-01	N/A	3	rs6796246	29064003	4.69E-02	N/A	-	5.80E-07	3.98E-01	6.34E-05	8237	
5	rs13178161	86987264	1.24E-01	N/A	19	rs12460438	22867619	1.63E-01	N/A	-	5.80E-07	9.14E-01	2.31E-05	8238	
5	rs13178161	86987264	1.24E-01	N/A	19	rs7246529	22856963	1.63E-01	N/A	-	5.80E-07	9.33E-01	2.41E-05	8239	
5	rs318108	92959503	6.47E-01	FAM172A	10	rs12412647	49654308	9.16E-01	WDFY4	-	5.80E-07	8.08E-01	7.67E-05	8240	
1	rs1736559	1.69E+08	8.99E-01	FMO3	11	rs4598653	28505842	1.13E-01	N/A	-	5.80E-07	5.81E-01	2.42E-05	8241	
7	rs2534575	38337999	4.87E-01	N/A	10	rs7904918	1.14E+08	4.04E-02	ACSL5	-	5.80E-07	8.32E-01	9.36E-05	8242	
13	rs879681	1.06E+08	2.38E-01	N/A	19	rs2304232	46454365	8.53E-01	HNRNPUL1	-	5.80E-07	3.65E-02	1.43E-02	8243	
6	rs12211975	37884200	9.82E-01	ZFAND3	16	rs231921	20569262	1.79E-02	ACSM1	-	5.80E-07	6.96E-01	6.49E-05	8244	
14	rs1769621	33493429	7.51E-02	EGLN3	19	rs367825	3014409	2.38E-01	TLE2	-	5.80E-07	7.54E-01	7.45E-05	8245	
2	rs1863676	2.31E+08	9.31E-01	N/A	18	rs4800466	19145025	6.12E-01	C18orf45	-	5.80E-07	7.92E-01	4.65E-04	8246	
4	rs12643033	1.7E+08	5.92E-01	PALLD	12	rs7134738	26249437	5.13E-01	SSPN	-	5.80E-07	9.85E-01	4.88E-05	8247	
1	rs10493695	81825975	1.38E-02	N/A	4	rs10519415	1.38E+08	9.47E-01	N/A	-	5.80E-07	N/A	N/A	8248	
1	rs11118618	2.19E+08	6.08E-01	N/A	3	rs3755625	1.58E+08	6.49E-01	KCN/AB1	-	5.81E-07	1.41E-01	1.46E-03	8249	
15	rs16963122	34539761	7.14E-02	N/A	17	rs159279	29716300	3.29E-01	CCL13	-	5.81E-07	2.70E-01	4.69E-06	8250	
7	rs10951847	46193477	2.32E-02	N/A	8	rs4831320	14895787	9.72E-01	SGCZ	-	5.81E-07	3.42E-02	1.60E-06	8251	
6	rs6923034	74493546	8.15E-01	CD109	2	rs1169255	35116338	1.39E-01	N/A	-	5.81E-07	4.99E-01	1.91E-05	8252	
2	rs205621	74821748	4.19E-01	N/A	5	rs752283	1.78E+08	8.21E-01	COL23A1	-	5.81E-07	8.13E-01	2.30E-04	8253	
6	rs9267947	32319196	8.78E-01	NOTCH4	15	rs12912995	88809540	2.59E-01	IQGAP1	MHC	5.81E-07	3.89E-02	1.34E-07	8254	
6	rs9392523	3750472	7.90E-01	N/A	5	rs11167794	1.39E+08	2.96E-02	PSD2	-	5.81E-07	1.11E-01	1.01E-06	8255	
7	rs270904	1.37E+08	7.97E-01	DGKI	7	rs12703402	1.41E+08	5.51E-01	AGK	-	5.81E-07	5.79E-01	3.52E-05	8256	
6	rs12000	28335415	4.34E-01	NKAPL	13	rs7317836	1.01E+08	2.93E-02	N/ALCN	MHC	5.81E-07	6.99E-01	1.47E-04	8257	
5	rs350089	40210203	2.93E-01	N/A	16	rs10852323	10589107	1.60E-01	EMP2	-	5.81E-07	7.56E-01	1.92E-04	8258	
6	rs2508015	31118179	8.45E-01	HCG22	6	rs4947296	31166157	4.31E-02	N/A	MHC	5.81E-07	1.46E-02	1.46E-07	8259	
6	rs2237236	26559532	9.88E-01	BTN3A3	15	rs1378847	29234640	5.84E-01	N/A	MHC	5.81E-07	2.04E-01	1.08E-03	8260	
14	rs8016794	52070396	9.02E-01	TXNDC16	15	rs2293028	31802205	7.95E-01	RYR3	-	5.81E-07	8.49E-01	8.54E-05	8261	
4	rs17867524	1.19E+08	3.61E-01	N/A	20	rs6123551	54195825	1.06E-01	N/A	-	5.82E-07	1.07E-01	1.33E-03	8262	
4	rs17867526	1.19E+08	3.61E-01	N/A	20	rs6123551	54195825	1.06E-01	N/A	-	5.82E-07	1.07E-01	1.33E-03	8263	
13	rs9598051	33216946	8.31E-01	N/A	18	rs1788103	65689804	4.58E-01	CD226	-	5.82E-07	2.69E-01	1.07E-06	8264	
13	rs9598051	33216946	8.31E-01	N/A	18	rs1790588	65686164	4.58E-01	DOK6	-	5.82E-07	2.69E-01	1.07E-06	8265	
2	rs16865276	6568763	2.73E-01	N/A	4	rs6448587	28171088	8.03E-01	N/A	-	5.82E-07	3.58E-01	1.34E-03	8266	
2	rs10193873	33895727	7.83E-01	N/A	5	rs10155508	1.36E+08	2.98E-01	N/A	-	5.82E-07	5.59E-01	3.71E-04	8267	
8	rs633979	12977952	8.41E-02	DLC1	20	rs10485804	54239111	7.83E-01	MC3R	-	5.82E-07	6.04E-01	3.72E-05	8268	
6	rs4706341	90259096	4.66E-01	ANKRD6	17	rs7207955	72434856	4.20E-01	MGAT5B	-	5.82E-07	7.68E-01	1.03E-04	8269	
3	rs1355538	1.67E+08	2.10E-02	BCHE	11	rs7925271	4621233	5.42E-01	OR51E1	-	5.82E-07	7.84E-01	1.18E-04	8270	
3	rs9867490	1.2E+08	3.21E-01	N/A	7	rs2894771	57421511	8.87E-01	N/A	-	5.82E-07	6.72E-03	4.09E-08	8271	
14	rs7145382	38998918	1.18E-01	N/A	17	rs538399	35864191	9.81E-01	IGFBP4	-	5.82E-07	2.02E-01	1.24E-03	8272	
8	rs12548349	72692958	9.29E-01	N/A	18	rs9954856	28009142	5.44E-01	MEP1B	-	5.82E-07	6.18E-01	1.46E-05	8273	
7	rs6462540	34320990	9.11E-01	N/A	13	rs9316728	53978870	8.29E-01	N/A	-	5.82E-07	3.37E-01	1.03E-03	8274	
6	rs9452048	93352725	2.41E-01	N/A	4	rs2305958	1.29E+08	9.36E-01	HSPA4L	-	5.82E-07	3.91E-01	1.77E-04	8275	
3	rs7610202	1.6E+08	9.84E-01	N/A	9	rs11143705	75637499	3.31E-01	N/A	-	5.82E-07	7.87E-01	4.42E-05	8276	
5	rs1826675	62102994	2.07E-02	N/A	9	rs6476824	4196769	9.65E-01	GLIS3	-	5.82E-07	8.25E-01	4.92E-05	8277	
3	rs9870894	1.61E+08	5.95E-01	SCHIP1	3	rs10936353	1.65E+08	7.88E-01	N/A	-	5.82E-07	8.26E-02	6.62E-03	8278	
3	rs10470336	1.61E+08	5.95E-01	SCHIP1	3	rs10936353	1.65E+08	7.88E-01	N/A	-	5.82E-07	9.78E-02	5.68E-03	8279	
3	rs1565352	1.61E+08	5.95E-01	SCHIP1	3	rs10936353	1.65E+08	7.88E-01	N/A	-	5.82E-07	9.78E-02	5.68E-03	8280	
3	rs3773271	13621819	6.59E-01	FBLN2	4	rs637495	37806344	3.78E-02	TBC1D1	-	5.82E-07	4.79E-01	3.08E-03	8281	
1	rs754224	2.26E+08	4.80E-01	SN/AP47	17	rs1405187	47260598	1.82E-02	CA10	-	5.82E-07	8.15E-01	3.27E-05	8282	
11	rs7114583	1.22E+08	5.33E-01	C11orf63	16	rs9928833	25879924	7.83E-01	HS3ST4	-	5.82E-07	1.18E-01	6.08E-03	8283	
18	rs1473633	4224452	6.66E-03	N/A	18	rs11663391	12506768	4.05E-01	SPIRE1	-	5.82E-07	6.37E-01	7.65E-04	8284	
4	rs2062763	1.61E+08	6.90E-01	N/A	14	rs11157364	43306266	1.31E-02	N/A	-	5.82E-07	8.49E-01	1.78E-04	8285	
4	rs1224511	1.61E+08	6.90E-01	N/A	14	rs11157364	43306266	1.31E-02	N/A	-	5.82E-07	9.76E-01	1.14E-04	8286	

SNP A					SNP B						Interaction P			Ranking	Cluster in top 100 interactions
CHR	SNP	Location	gle locus P va	Gene	CHR	SNP	Location	Single locus P valu	Gene	MHC region	Stage 1	Stage 2	Combined		
3	rs4683070	45174624	4.12E-01	CDCP1	20	rs6110252	14272140	7.09E-02	MACROD2	-	5.82E-07	6.95E-01	4.83E-05	8287	
1	rs12031183	2.44E+08	6.51E-01	SMYD3	9	s10733728	1.05E+08	2.95E-01	N/A	-	5.83E-07	1.54E-01	1.28E-06	8288	
1	rs11578784	2.22E+08	5.54E-02	N/A	10	rs1409391	14667831	1.29E-01	FAM107B	-	5.83E-07	2.02E-01	1.56E-06	8289	
15	rs753857	47645190	1.09E-01	C15orf33	16	rs8057936	64472852	6.82E-01	N/A	-	5.83E-07	1.19E-01	1.73E-06	8290	
7	rs12705744	1.1E+08	3.82E-01	IMMP2L	8	s16879442	32444084	9.18E-01	NRG1	-	5.83E-07	7.45E-01	8.90E-06	8291	
4	rs1379707	81399403	2.13E-01	FGF5	21	rs2839378	46909417	1.50E-01	PRMT2	-	5.83E-07	N/A	N/A	8292	
2	rs10167828	1.17E+08	5.91E-01	N/A	15	rs9920389	93832564	9.64E-01	LOC145820	-	5.83E-07	9.71E-01	7.45E-05	8293	
7	rs533887	83136139	9.16E-02	SEMA3E	15	rs2073592	99689829	5.44E-01	PCSK6	-	5.83E-07	N/A	N/A	8294	
3	rs1675521	99728322	1.05E-01	CLDND1	8	rs2252460	14679062	9.27E-01	SGCZ	-	5.83E-07	6.36E-01	2.63E-05	8295	
5	rs12518125	1.69E+08	4.38E-02	DOCK2	11	rs924110	1.33E+08	7.03E-01	N/A	-	5.83E-07	7.88E-01	1.45E-05	8296	
8	rs12676876	69767325	1.32E-01	C8orf34	10	rs1331155	1.08E+08	7.37E-01	N/A	-	5.83E-07	2.77E-01	8.04E-04	8297	
1	rs4540634	1.61E+08	8.60E-01	N/A	20	rs6044895	17515578	1.66E-01	DSTN	-	5.83E-07	2.92E-01	4.17E-06	8298	
5	rs10513024	53254741	1.12E-01	ARL15	20	rs6139726	5351246	5.49E-01	N/A	-	5.83E-07	N/A	N/A	8299	
2	rs16855155	1.69E+08	1.88E-01	STK39	8	rs4324958	1.22E+08	7.37E-01	SNTB1	-	5.84E-07	5.38E-01	1.26E-04	8300	
2	rs12618497	12068272	2.85E-01	N/A	7	rs2657237	9599702	5.15E-01	N/A	-	5.84E-07	8.20E-01	4.01E-05	8301	
6	rs270378	7707714	2.39E-01	BMP6	17	rs4536508	61813793	7.85E-02	PRKCA	-	5.84E-07	1.98E-01	5.67E-06	8302	
6	rs270378	7707714	2.39E-01	BMP6	17	rs7211558	61812941	7.85E-02	PRKCA	-	5.84E-07	1.98E-01	5.67E-06	8303	
6	rs2747690	1.64E+08	7.58E-01	PACRG	12	s10848548	1653512	6.14E-01	ADIPOR2	-	5.84E-07	5.39E-01	1.02E-05	8304	
1	rs1077884	1.14E+08	1.64E-01	SYT6	10	rs7923262	71895542	4.77E-01	KIAA1274	-	5.84E-07	9.51E-01	3.05E-05	8305	
6	rs9381237	43428388	4.32E-01	ZNF318	13	rs1981060	38650044	5.89E-01	N/A	-	5.84E-07	1.52E-01	2.47E-03	8306	
14	rs1743506	95213333	4.49E-01	TCL1B	20	rs292887	37761688	9.18E-01	N/A	-	5.84E-07	2.72E-01	6.08E-04	8307	
1	rs2148210	5278276	2.74E-01	N/A	4	rs1353624	1.44E+08	7.07E-01	INPP4B	-	5.84E-07	7.95E-01	9.45E-05	8308	
12	rs12812221	55605603	5.43E-01	SDR9C7	15	rs4778154	25468103	4.83E-02	GABRG3	-	5.84E-07	2.59E-02	1.32E-07	8309	
7	rs7806221	1.35E+08	4.50E-01	NUP205	9	s10760025	1.21E+08	5.52E-02	DBC1	-	5.84E-07	6.01E-01	2.09E-05	8310	
1	rs12407446	1.57E+08	6.61E-01	CD1A	18	rs3932728	388147	5.84E-01	COLEC12	-	5.84E-07	7.15E-01	1.36E-04	8311	
1	rs3892225	2.17E+08	2.20E-01	TGFB2	10	rs7087965	16729689	1.26E-01	RSU1	-	5.84E-07	7.76E-01	8.13E-05	8312	
2	rs10931507	1.92E+08	7.13E-01	N/A	12	rs2168029	68020908	6.01E-01	LYZ	-	5.84E-07	9.73E-02	3.21E-03	8313	
1	rs927019	63854867	2.99E-01	PGM1	4	rs936551	811490	8.43E-01	CPLX1	-	5.84E-07	5.61E-01	1.62E-04	8314	
2	rs6730381	2.22E+08	8.96E-02	N/A	3	s12633980	126789	4.20E-01	N/A	-	5.84E-07	2.58E-01	1.07E-03	8315	
11	rs1886657	34313612	9.78E-01	ABTB2	17	s11649949	3619690	2.27E-01	ITGAE	-	5.84E-07	4.08E-01	1.66E-03	8316	
4	rs10000121	1.58E+08	1.69E-01	N/A	5	rs1370182	30748837	1.13E-01	N/A	-	5.84E-07	4.44E-01	2.98E-04	8317	
13	rs10851266	1.11E+08	8.98E-01	N/A	21	rs2835555	37273701	3.77E-01	HLCS	-	5.84E-07	1.39E-01	5.03E-07	8318	
13	rs10851266	1.11E+08	8.98E-01	N/A	21	rs2835557	37273783	3.77E-01	HLCS	-	5.84E-07	1.39E-01	5.03E-07	8319	
6	rs12214182	23974032	9.37E-01	N/A	14	s17117189	82249803	1.90E-01	N/A	-	5.84E-07	4.78E-01	2.47E-05	8320	
6	rs12214182	23974032	9.37E-01	N/A	14	rs8011971	82246445	1.90E-01	N/A	-	5.84E-07	4.78E-01	2.47E-05	8321	
7	rs11768025	1.43E+08	6.20E-01	FAM115A	14	rs2543352	75656117	1.77E-01	N/A	-	5.85E-07	9.87E-02	2.35E-03	8322	
3	rs1877267	1.7E+08	4.57E-01	N/A	5	rs759135	1.48E+08	8.02E-01	N/A	-	5.85E-07	6.69E-01	3.50E-05	8323	
8	rs6471711	59444379	4.28E-01	N/A	19	rs2116941	10195443	4.56E-01	S1PR2	-	5.85E-07	9.71E-01	3.90E-05	8324	
3	rs2675181	9172231	4.32E-01	SRGAP3	3	rs4677766	1.96E+08	3.65E-01	N/A	-	5.85E-07	2.96E-01	2.80E-03	8325	
2	rs666126	21135940	6.80E-01	APOB	12	rs1564647	1.32E+08	9.71E-01	CHFR	-	5.85E-07	8.36E-01	1.81E-04	8326	
9	rs10993045	95883066	1.10E-01	PTPDC1	15	rs304129	99085506	5.39E-01	N/A	-	5.85E-07	5.90E-02	3.61E-07	8327	
2	rs749460	1.35E+08	3.65E-01	TMEM163	8	s10503296	4803438	6.89E-01	CSMD1	-	5.85E-07	1.33E-01	5.49E-06	8328	
14	rs941924	99962502	3.27E-01	WDR25	17	rs2362396	75469049	5.17E-01	N/A	-	5.85E-07	5.18E-01	8.96E-04	8329	
12	rs12372711	25418920	8.63E-01	N/A	13	rs2002363	1E+08	8.78E-01	N/A	-	5.85E-07	2.12E-01	4.80E-07	8330	
1	rs10489745	1.64E+08	6.44E-01	RXRG	18	rs1443321	68035323	7.44E-01	N/A	-	5.85E-07	1.17E-01	2.52E-03	8331	
1	rs11208905	66785643	3.50E-01	SGIP1	13	s12860689	53213806	6.53E-01	N/A	-	5.85E-07	1.13E-01	2.21E-03	8332	
2	rs1001098	2.19E+08	7.69E-01	N/A	5	rs400691	1.25E+08	9.51E-01	N/A	-	5.85E-07	5.37E-01	1.53E-05	8333	
13	rs4770597	23605466	4.72E-02	N/A	22	s16988632	19315012	1.99E-01	N/A	-	5.85E-07	1.37E-01	2.03E-06	8334	
13	rs6561302	45664566	3.99E-01	LCP1	20	rs2423487	10161095	5.14E-01	SN/AP25	-	5.85E-07	2.66E-01	2.29E-06	8335	
2	rs10170020	80373325	2.46E-02	CTNN/A2	8	rs2170116	16157007	3.72E-01	N/A	-	5.85E-07	N/A	N/A	8336	
2	rs12996794	2.38E+08	2.62E-01	RAMP1	21	rs2835165	36232226	3.98E-01	N/A	-	5.86E-07	5.29E-01	1.21E-05	8337	
3	rs6783300	1.77E+08	1.38E-01	N/AALADL2	14	rs2000316	61614119	6.46E-01	SYT16	-	5.86E-07	N/A	N/A	8338	
8	rs12547343	26889115	6.19E-02	N/A	8	s16901359	1.27E+08	9.11E-02	N/A	-	5.86E-07	5.62E-02	7.58E-03	8339	
8	rs1481646	26887222	6.19E-02	N/A	8	s16901359	1.27E+08	9.11E-02	N/A	-	5.86E-07	5.62E-02	7.58E-03	8340	
2	rs2595202	46045521	5.22E-01	PRKCE	3	rs9654034	29318469	8.54E-01	N/A	-	5.86E-07	1.04E-01	2.99E-06	8341	
14	rs11625068	61803905	8.47E-01	N/A	18	s12454763	40688613	2.90E-01	SETBP1	-	5.86E-07	2.07E-01	1.06E-05	8342	
3	rs6444054	1.87E+08	5.38E-01	MAP3K13	16	s16956086	54660259	3.62E-01	N/A	-	5.86E-07	7.64E-01	4.89E-04	8343	
2	rs6738097	1.01E+08	9.79E-01	NPAS2	8	rs6995462	3304322	6.06E-01	CSMD1	-	5.86E-07	3.28E-01	1.35E-05	8344	
3	rs13089230	1.79E+08	7.71E-01	N/A	10	rs1152699	1.27E+08	3.71E-01	CTBP2	-	5.86E-07	6.90E-01	6.71E-05	8345	
3	rs4683474	1.41E+08	8.62E-01	CLSTN2	9	rs7040955	1.04E+08	8.57E-01	N/A	-	5.86E-07	4.85E-01	5.24E-06	8346	
6	rs6935051	1.5E+08	5.88E-01	RAET1L	3	rs2124147	1.73E+08	6.44E-01	PLD1	-	5.86E-07	7.34E-01	6.01E-05	8347	
2	rs1427550	19673258	3.29E-01	N/A	12	rs6486795	1.21E+08	7.72E-01	ORAI1	-	5.86E-07	9.49E-01	4.89E-05	8348	
13	rs9576292	36998697	4.66E-01	N/A	14	s11627948	29657861	9.60E-01	N/A	-	5.86E-07	N/A	N/A	8349	
8	rs1489365	1.12E+08	3.30E-01	N/A	12	s11112403	1.04E+08	4.10E-01	APPL2	-	5.86E-07	4.56E-01	3.85E-04	8350	
2	rs831017	1.7E+08	3.65E-01	LRP2	4	rs7663866	32445371	5.98E-01	N/A	-	5.86E-07	8.67E-04	1.32E-02	8351	
6	rs1265203	4323996	4.59E-01	N/A	5	rs6596651	1.05E+08	6.06E-02	N/A	-	5.86E-07	8.33E-01	8.21E-05	8352	
6	rs7755190	44559792	9.73E-01	N/A	9	s10491586	907348	6.30E-01	DMRT1	-	5.86E-07	8.96E-01	7.06E-05	8353	
11	rs10832519	15768506	2.92E-01	N/A	15	rs1000914	99768456	7.16E-01	PCSK6	-	5.87E-07	2.05E-01	1.67E-06	8354	
11	rs10832519	15768506	2.92E-01	N/A	15	rs1108993	99768718	7.16E-01	PCSK6	-	5.87E-07	2.05E-01	1.67E-06	8355	
11	rs10832519	15768506	2.92E-01	N/A	15	s11633098	99769346	7.16E-01	PCSK6	-	5.87E-07	2.05E-01	1.67E-06	8356	
11	rs10832519	15768506	2.92E-01	N/A	15	s12916267	99770553	7.16E-01	PCSK6	-	5.87E-07	2.29E-01	2.02E-06	8357	
1	rs766499	2.13E+08	4.42E-01	SMYD2	13	s10492664	1.08E+08	6.04E-02	N/A	-	5.87E-07	7.98E-01	1.49E-05	8358	
1	rs6661776	11734714	3.42E-01	AGTRAP	9	s12338330	30743635	8.78E-01	N/A	-	5.87E-07	8.99E-01	1.97E-04	8359	
9	rs12350232	73221968	2.09E-01	N/A	10	rs2384167	54625302	9.79E-01	N/A	-	5.87E-07	9.15E-01	2.70E-05	8360	
1	rs12120788	1.56E+08	4.86E-01	N/A	18	rs582110	20249434	7.02E-01	IMPACT	-	5.87E-07	1.71E-02	4.42E-08	8361	
8	rs10954965	34450799	8.24E-01	N/A	18	rs7240570	46059417	9.92E-01	MBD1	-	5.87E-07	4.85E-01	6.14E-04	8362	
3	rs17035210	36528606	3.87E-01	STAC	21	rs2834940	35758791	2.08E-02	N/A	-	5.87E-07	5.98E-02	1.36E-02	8363	
10	rs582449	78864354	3.77E-01	KCNMA1	13	s12430668	1.1E+08	7.83E-01	N/A	-	5.87E-07	6.17E-01	1.44E-04	8364	
1	rs1538961	2.44E+08	9.74E-01	KIF26B	4	rs2168267	63090174	4.31E-01	N/A	-	5.87E-07	8.01E-01	3.24E-05	8365	
4	rs11732071	1.9E+08	2.38E-01	N/A	12	rs4387423	81941727	7.51E-01	TMTC2	-	5.87E-07	5.38E-01	6.80E-04	8366	
6	rs2764075	7300517	1.24E-01	N/A	21	rs198078	23347197	3.75E-01	N/A	-	5.87E-07	4.69E-01	1.81E-05	8367	
2	rs2123774	33494820	5.95E-01	LTBP1	3	rs1124184	1.72E+08	1.84E-01	TNIK	-	5.87E-07	5.52E-01	5.66E-04	8368	
4	rs1348060	67875452	8.39E-01	N/A	9	rs4339720	25109058	7.78E-01	N/A	-	5.87E-07	5.15E-02	2.25E-02	8369	
8	rs950549	16885663	2.28E-01	FGF20	20	rs362584	10202475	7.13E-01	SN/AP25	-	5.87E-07	5.61E-02	3.18E-07	8370	
18	rs11660621	73145738	2.09E-01	N/A	22	rs2205815	33298614	1.22E-02	N/A	-	5.87E-07	5.54E-01	1.87E-04	8371	
3	rs6439416	1.35E+08	1.01E-01	N/A	4	rs4627935	1.42E+08	1.59E-01	RNF150	-	5.87E-07	9.11E-01	1.33E-04	8372	
8	rs11783979	1.06E+08	8.72E-01	DPYS	22	rs2896022	42766283	3.60E-01	PARVB	-	5.88E-07	2.36E-01	2.18E-03	8373	
1	rs7418397	1.05E+08	2.50E-02	N/A	12	rs7962305	1E+08	3.30E-01	SLC5A8	-	5.88E-07	4.92E-01	3.17E-05	8374	
2	rs10167695	50912249	6.42E-01	NRXN1	3	rs6548746	81203808	5.05E-01	N/A	-	5.88E-07	9.13E-01	1.47E-04	8375	
2	rs4446102	45851371	9.48E-01	PRKCE	13	rs348008	79702503	5.16E-01	N/A	-	5.88E-07	9.62E-01	8.50E-05	8376	
3	rs4685929	5424157	8.32E-01	N/A	8	s12678139	71556304	1.04E-01	N/A	-	5.88E-07	4.42E-01	2.60E-06	8377	
14	rs4272953	48540230	9.05E-01	N/A	16	rs930744	79273958	7.60E-01	CDYL2	-	5.88E-07	2.33E-01	2.45E-06	8378	
2	rs6752720	54815856	6.59E-01	EML6	18	rs7242862	51656014	9.71E-01	N/A	-	5.88E-07	4.84E-01	4.12E-06	8379	
3	rs9878957	1.33E+08	1.82E-01	N/A	4	rs1598859	1.04E+08	8.57E-01	NFKB1	-	5.88E-07	9.01E-01	5.40E-05	8380	
7	rs6462370	32482895	2.43E-02	AVL9	14	rs1476610	70267508	1.24E-01	MAP3K9	-	5.88E-07	8.20E-02	3.88E-07	8381	
6	rs1624440	26541308	9.34E-01	BTN3A3	15	rs1378847	29234640	5.84E-01	N/A	MHC	5.88E-07	3.33E-01	5.87E-04	8382	
1	rs12039012	35710472	6.63E-01	KIAA0319L	17	rs4795343	34472851	4.95E-01	PLXDC1	-	5.88E-07	5.74E-01	1.18E-05	8383	
1	rs12562622	35677767	6.63E-01	ZMYM4	17	rs4795343	34472851	4.95E-01	PLXDC1	-	5.88E-07	5.79E-01	1.22E-05	8384	
1	rs11485264	74970121	7.05E-01	CRYZ	7	rs7779412	1.49E+08	7.42E-01	N/A	-	5.88E-07	1.67E-01	2.17E-03	8385	
10	rs1314013	31605361	9.02E-01	N/A	13	rs9567077	42442964	1.36E-01	EPSTI1	-	5.88E-07	1.77E-01	9.64E-04	8386	
1	rs17120530	99724648	8.60E-01	N/A	20	s12151880	8847426	4.62E-01	N/A	-	5.88E-07	7.46E-01	1.59E-05	8387	
5	rs2526162	1.35E+08	4.86E-01	LECT2	7	rs4357206	5369132	4.48E-01	TNRC18	-	5.88E-07	6.93E-01	2.29E-04	8388	
1	rs6540451	2.06E+08	4.90E-01	PLXN/A2	21	rs858008	37987500	4.45E-01	KCNJ6	-	5.88E-07	3.85E-02	3.25E-07	8389	
13	rs12866357	87376672	8.09E-01	N/A	15	rs2048305	97665539	7.65E-01	LRRC28	-	5.88E-07	5.96E-01	5.16E-05	8390	
14	rs10148795	32915073	4.18E-01	NPAS3	15	rs4244899	90073528	9.74E-01	N/A	-	5.89E-07	3.15E-01	1.12E-05	8391	
6	rs721101	1.56E+08	4.00E-01	TFB1M	3	rs2195914	1.29E+08	6.64E-01	KBTBD12	-	5.89E-07	9.05E-01	6.28E-05	8392	
5	rs11952632	21849060	9.32E-01	CDH12	9	s12375503	30028860	6.52E-01	N/A	-	5.89E-07	5.22E-01	1.65E-05	8393	
4	rs4069861	1.84E+08	1.59E-01	N/A	18	rs1521428	4228612	2.14E-01	N/A	-	5.89E-07	5.94E-01	6.88E-05	8394	
10	rs4933471	89875567	9.51E-01	N/A	12	rs6488388	7554728	4.56E-01	CD163	-	5.89E-07	8.83E-01	1.57E-04	8395	
10	rs7069053	89876184	9.51E-01	N/A	12	rs6488388	7554728	4.56E-01	CD163	-	5.89E-07	8.83E-01	1.57E-04	8396	
5	rs10069382	31957022	1.47E-01	PDZD2	17	rs2074191	43271724	6.94E-01	OSBPL7	-	5.89E-07	N/A	N/A	8397	
3	rs10935419	1.42E+08	1.55E-01	N/A	4	rs3797025	1.65E+08	9.89E-01	1-Mar	-	5.89E-07	3.48E-01	2.67E-03	8398	

SNP A					SNP B						Interaction P				Cluster in top 100 interactions
CHR	SNP	Location	gle locus P va	Gene	CHR	SNP	Location	Single locus P valu	Gene	MHC region	Stage 1	Stage 2	Combined	Ranking	
7	rs10245821	89212857	1.85E-01	N/A	12	rs2900367	16028049	6.09E-01	DERA	-	5.89E-07	7.10E-01	1.51E-05	8399	
2	rs11893319	1.97E+08	1.77E-01	HECW2	3	rs9290477	1.75E+08	6.93E-01	NLGN1	-	5.89E-07	8.10E-01	3.49E-04	8400	
2	rs13413808	2.26E+08	4.20E-01	N/A	4	rs2159972	18666528	1.66E-01	N/A	-	5.89E-07	9.99E-01	4.86E-05	8401	
5	rs17509298	1.16E+08	6.63E-01	N/A	22	rs4824056	48314077	9.46E-01	N/A	-	5.89E-07	N/A	N/A	8402	
10	rs4917420	1.06E+08	2.66E-01	N/A	12	rs4761936	50063395	2.29E-01	GALNT6	-	5.89E-07	3.23E-02	4.77E-07	8403	
16	rs2023929	70794457	3.53E-01	N/A	18	rs1843184	61704351	6.23E-01	CDH7	-	5.89E-07	4.72E-02	7.28E-03	8404	
4	rs17828324	61908200	1.90E-01	N/A	3	rs3923441	88408675	2.10E-01	N/A	-	5.90E-07	2.93E-01	9.14E-06	8405	
3	rs9288967	1.14E+08	5.45E-01	C3orf17	11	rs10902218	733813	9.12E-02	EPS8L2	-	5.90E-07	3.92E-01	9.59E-06	8406	
6	rs1135056	71018554	2.68E-01	COL9A1	13	rs9586002	1.02E+08	7.04E-01	LOC121952	-	5.90E-07	4.37E-01	3.18E-04	8407	
2	rs1430104	1.15E+08	2.68E-01	DPP10	10	rs10444205	6776567	2.08E-01	N/A	-	5.90E-07	5.94E-01	6.43E-05	8408	
6	rs4712728	23050192	1.83E-02	N/A	12	rs10848287	1.3E+08	9.21E-02	GPR133	-	5.90E-07	5.07E-01	5.86E-03	8409	
4	rs2135354	1.14E+08	4.30E-01	ANK2	19	rs2965155	49880311	8.88E-01	CEACAM16	-	5.90E-07	7.99E-01	4.94E-05	8410	
6	rs7760349	57220087	9.93E-01	N/A	8	rs6991834	3115506	1.57E-01	CSMD1	-	5.90E-07	5.86E-01	2.85E-05	8411	
6	rs7760349	57220087	9.93E-01	N/A	8	rs7010127	3115425	1.57E-01	CSMD1	-	5.90E-07	5.86E-01	2.85E-05	8412	
12	rs1033232	60033642	5.14E-01	N/A	13	rs1924206	21457190	4.58E-01	N/A	-	5.90E-07	3.87E-02	1.57E-06	8413	
7	rs763397	10686855	8.84E-02	N/A	18	rs1376119	20536712	1.13E-02	N/A	-	5.90E-07	9.08E-01	9.69E-05	8414	
15	rs999047	41476208	7.78E-02	TP53BP1	19	rs7256487	19716654	6.42E-02	ZNF14	-	5.90E-07	9.85E-01	1.08E-04	8415	
10	rs12766316	1405366	1.71E-01	ADARB2	17	rs8078179	14856327	9.16E-01	N/A	-	5.90E-07	6.65E-01	3.80E-04	8416	
6	rs2131283	50427324	9.51E-01	N/A	12	rs7304109	60484742	1.12E-01	FAM19A2	-	5.90E-07	8.36E-01	1.03E-03	8417	
12	rs7313825	12499123	1.17E-01	LOH12CR1	12	rs1402318	76753767	8.81E-01	N/AV3	-	5.90E-07	8.64E-01	1.10E-04	8418	
4	rs7657174	82775969	1.07E-01	N/A	11	rs4586138	11955605	4.04E-02	USP47	-	5.90E-07	N/A	N/A	8419	
6	rs6596805	1240151	2.83E-01	FOXQ1	6	rs1047953	22294843	4.49E-01	FLJ22536	-	5.90E-07	N/A	N/A	8420	
10	rs2163673	90477499	7.09E-01	LIPK	21	rs2837688	40829567	3.03E-01	DSCAM	-	5.90E-07	8.65E-02	3.83E-07	8421	
2	rs12052709	1.22E+08	6.14E-01	CLASP1	14	rs2254320	91440894	1.57E-02	FBLN5	-	5.90E-07	4.63E-01	1.67E-03	8422	
4	rs12649725	63401029	5.50E-01	N/A	12	rs1436124	94807563	6.05E-01	CCDC38	-	5.90E-07	3.97E-02	6.95E-07	8423	
6	rs3130559	31205280	7.50E-01	C6orf15	6	rs2227956	31886251	5.64E-03	VARS	MHC	5.91E-07	4.60E-01	7.19E-06	8424	
15	rs723434	72130383	7.63E-01	PML	18	rs7505745	4089242	8.65E-01	N/A	-	5.91E-07	1.54E-01	5.51E-04	8425	
8	rs957608	14611359	8.59E-01	SGCZ	11	rs10833450	21033719	4.49E-01	NELL1	-	5.91E-07	1.56E-01	1.09E-03	8426	
5	rs12523326	1.2E+08	2.25E-01	N/A	11	rs1982496	1.06E+08	2.75E-01	GUCY1A2	-	5.91E-07	9.07E-01	7.26E-05	8427	
4	rs1497125	1.47E+08	2.07E-01	MMAA	17	rs216219	2094041	6.27E-01	SMG6	-	5.91E-07	3.16E-01	1.95E-03	8428	
1	rs10915668	4873161	6.30E-01	N/A	11	rs10835810	31679060	7.96E-01	ELP4	-	5.91E-07	5.29E-01	2.60E-05	8429	
8	rs7829063	1.07E+08	3.28E-01	N/A	9	rs10967306	2630805	6.31E-01	FLJ35024	-	5.91E-07	6.13E-01	2.16E-05	8430	
1	rs12120788	1.56E+08	4.86E-01	N/A	18	rs489631	20243022	7.04E-01	IMPACT	-	5.91E-07	5.14E-03	1.43E-08	8431	
12	rs12318001	1.03E+08	6.33E-01	N/A	18	rs786034	14930293	5.61E-01	N/A	-	5.91E-07	3.26E-02	9.33E-03	8432	
2	rs1357204	53462867	1.47E-03	N/A	8	rs6991885	1.43E+08	2.65E-01	TSN/ARE1	-	5.91E-07	5.22E-01	1.02E-05	8433	
5	rs10515241	95988347	3.03E-01	N/A	14	rs11851301	94244019	8.05E-01	N/A	-	5.91E-07	8.19E-01	2.90E-04	8434	
1	rs4847269	94189807	4.16E-01	N/A	4	rs10517867	1.67E+08	1.90E-01	N/A	-	5.91E-07	8.49E-01	2.49E-04	8435	
5	rs11953210	1.24E+08	2.51E-01	ZNF608	19	rs7256033	54209025	1.28E-02	SN/AR-G2	-	5.91E-07	5.14E-01	2.36E-04	8436	
8	rs895270	82067849	9.09E-01	PAG1	10	rs4747900	11374684	6.49E-02	CUGBP2	-	5.91E-07	4.34E-02	7.16E-03	8437	
2	rs20349	1.34E+08	7.04E-01	N/A	2	rs1477500	1.81E+08	8.98E-01	N/A	-	5.91E-07	4.18E-01	7.79E-06	8438	
6	rs9464303	55886911	8.13E-01	N/A	3	rs4858348	21730685	3.92E-01	ZNF385D	-	5.92E-07	1.51E-01	5.80E-06	8439	
6	rs4574618	55924686	8.13E-01	N/A	3	rs4858348	21730685	3.92E-01	ZNF385D	-	5.92E-07	1.69E-01	7.06E-06	8440	
6	rs895641	55893820	8.13E-01	N/A	3	rs4858348	21730685	3.92E-01	ZNF385D	-	5.92E-07	1.69E-01	7.06E-06	8441	
1	rs1418784	90769062	7.66E-01	N/A	7	rs1345525	1.31E+08	4.06E-01	N/A	-	5.92E-07	2.64E-01	8.91E-04	8442	
1	rs2789405	1.86E+08	3.34E-01	N/A	20	rs8183283	50670380	3.11E-01	N/A	-	5.92E-07	2.95E-01	3.74E-06	8443	
6	rs10943465	78293015	1.26E-01	N/A	14	rs1254910	61572745	1.95E-01	SYT16	-	5.92E-07	7.31E-01	4.33E-04	8444	
6	rs12204397	16601341	7.29E-01	ATXN1	10	rs10904833	16979982	6.06E-01	CUBN	-	5.92E-07	6.04E-01	2.95E-05	8445	
6	rs1408263	18515722	1.65E-02	RNF144B	11	rs616354	77265621	5.31E-01	INTS4	-	5.92E-07	3.20E-01	3.90E-06	8446	
5	rs7723716	67246186	4.70E-01	N/A	5	rs34992	80302822	3.48E-01	RASGRF2	-	5.92E-07	5.27E-01	3.65E-04	8447	
8	rs1866788	1.01E+08	1.07E-01	SPAG1	12	rs7305093	1.3E+08	3.09E-01	GPR133	-	5.92E-07	3.33E-01	1.02E-03	8448	
1	rs4233060	2.32E+08	5.71E-02	N/A	4	rs1656235	36970225	7.27E-01	KIAA1239	-	5.92E-07	4.09E-01	7.08E-06	8449	
5	rs10059312	66714796	1.06E-01	N/A	18	rs2847297	12787694	4.25E-01	PTPN2	-	5.92E-07	5.58E-01	4.67E-04	8450	
2	rs3769583	32821625	5.93E-01	N/A	14	rs3861621	77478622	7.86E-02	ADCK1	-	5.92E-07	1.35E-01	6.18E-03	8451	
7	rs227944	23569843	9.65E-01	N/A	20	rs6070505	56560874	3.55E-01	N/A	-	5.92E-07	3.85E-01	2.94E-05	8452	
4	rs6826277	78639678	6.15E-01	CXCL13	12	rs4996094	50069928	4.23E-01	GALNT6	-	5.93E-07	8.28E-02	8.61E-04	8453	
3	rs331158	78975042	5.95E-02	ROBO1	10	rs1797	1.2E+08	8.98E-01	N/A	-	5.93E-07	4.58E-01	2.27E-03	8454	
2	rs1419950	2.3E+08	3.09E-01	PID1	18	rs2541872	61783932	3.81E-01	N/A	-	5.93E-07	8.29E-01	1.27E-04	8455	
2	rs1914791	49787517	8.33E-01	N/A	5	rs338271	34839003	1.37E-01	RAI14	-	5.93E-07	7.05E-01	6.37E-05	8456	
10	rs723483	1.11E+08	6.16E-01	N/A	20	rs1883522	1074724	3.51E-02	PSMF1	-	5.93E-07	3.29E-01	2.13E-05	8457	
11	rs1793000	20663510	7.50E-01	NELL1	12	rs10879741	72903036	3.75E-01	N/A	-	5.93E-07	3.90E-01	2.35E-03	8458	
6	rs1472364	38098736	6.38E-01	ZFAND3	18	rs4075498	47826748	5.02E-02	N/A	-	5.93E-07	5.36E-01	6.38E-06	8459	
8	rs2960109	1.39E+08	5.40E-01	N/A	14	rs10873327	25202900	8.83E-03	N/A	-	5.93E-07	6.44E-01	2.83E-04	8460	
1	rs6673771	1.01E+08	4.49E-01	S1PR1	19	rs1047333	42334005	4.22E-02	ZNF585A	-	5.93E-07	7.46E-01	8.47E-06	8461	
3	rs7611893	32295296	6.50E-02	CMTM8	10	rs10509301	69814683	1.06E-01	RUFY2	-	5.93E-07	2.33E-01	2.10E-03	8462	
4	rs1563844	60541497	6.14E-01	N/A	5	rs1019810	15651629	8.04E-01	FBXL7	-	5.93E-07	7.43E-01	8.65E-06	8463	
8	rs7007801	3303507	8.29E-01	CSMD1	14	rs4362328	68141480	3.44E-01	RAD51L1	-	5.93E-07	8.93E-03	1.89E-02	8464	
6	rs9397523	1.53E+08	8.99E-02	SYNE1	10	rs3922590	1.33E+08	4.08E-01	N/A	-	5.94E-07	3.32E-02	1.46E-06	8465	
6	rs742224	11878800	9.06E-01	C6orf105	20	rs6056750	9539204	5.92E-01	PAK7	-	5.94E-07	1.55E-01	4.53E-03	8466	
10	rs6482067	20175733	9.69E-01	PLXDC2	10	rs10763792	30875579	8.56E-01	N/A	-	5.94E-07	9.24E-02	1.14E-06	8467	
1	rs275137	1.66E+08	6.89E-01	DCAF6	9	rs306538	1.35E+08	8.88E-01	DDX31	-	5.94E-07	6.81E-01	6.84E-04	8468	
8	rs6981770	17623070	7.80E-03	MTUS1	13	rs9315678	38841803	4.74E-01	LHFP	-	5.94E-07	7.02E-01	7.42E-04	8469	
2	rs7570609	1.28E+08	8.76E-01	MYO7B	21	rs2831689	28569675	2.54E-01	N/A	-	5.94E-07	7.53E-01	5.09E-05	8470	
4	rs7661550	46581758	2.94E-01	COX7B2	17	rs8068010	4278869	2.49E-01	SPNS3	-	5.94E-07	4.86E-01	2.40E-04	8471	
12	rs7965347	31672121	4.53E-01	C12orf72	12	rs4759987	1.28E+08	5.12E-01	TMEM132D	-	5.94E-07	9.97E-01	1.24E-04	8472	
6	rs3096700	32329760	9.20E-01	N/A	8	rs2279460	59670929	2.71E-02	NSMAF	MHC	5.94E-07	3.29E-01	1.26E-03	8473	
6	rs3804288	1.47E+08	8.40E-01	RAB32	5	rs2085448	1.68E+08	2.55E-01	WWC1	-	5.94E-07	4.01E-01	2.63E-03	8474	
1	rs2810587	41745682	2.48E-01	HIVEP3	13	rs4886104	58250558	4.20E-01	N/A	-	5.94E-07	N/A	N/A	8475	
2	rs3769643	2.28E+08	6.95E-01	COL4A4	8	rs4243888	1.39E+08	7.31E-01	FAM135B	-	5.94E-07	1.14E-01	6.12E-07	8476	
9	rs10739815	1.04E+08	1.71E-02	N/A	14	rs2273814	29132953	5.89E-01	PRKD1	-	5.94E-07	4.09E-01	5.59E-04	8477	
6	rs442124	95061419	2.68E-01	N/A	6	rs9375582	1.29E+08	3.80E-01	N/A	-	5.94E-07	7.42E-01	5.21E-04	8478	
6	rs417343	95064745	2.68E-01	N/A	6	rs9375582	1.29E+08	3.80E-01	N/A	-	5.94E-07	8.04E-01	4.31E-04	8479	
2	rs2975784	2.41E+08	6.13E-01	N/A	3	rs7619096	84281765	7.67E-02	N/A	-	5.94E-07	8.65E-01	2.86E-04	8480	
1	rs10746500	12738903	4.06E-01	C1orf158	8	rs2977798	6684860	1.55E-01	XKR5	-	5.94E-07	2.56E-01	4.04E-06	8481	
5	rs594140	1.72E+08	2.02E-01	C5orf41	12	rs1881227	87562931	9.76E-01	N/A	-	5.94E-07	2.88E-01	9.44E-06	8482	
10	rs11595694	1.24E+08	2.56E-01	BTBD16	17	rs8074645	5683518	1.44E-01	N/A	-	5.94E-07	3.22E-02	3.42E-03	8483	
4	rs10003328	30296150	4.01E-01	N/A	10	rs2059996	77071681	6.20E-01	N/A	-	5.94E-07	3.26E-01	5.06E-03	8484	
4	rs4862611	1.87E+08	1.86E-03	N/A	11	rs11233070	81677116	6.36E-02	N/A	-	5.95E-07	1.82E-01	8.30E-04	8485	
4	rs3796592	1.57E+08	5.57E-01	GUCY1A3	16	rs2301763	2885091	2.14E-01	FLYWCH2	-	5.95E-07	2.00E-01	6.72E-04	8486	
3	rs1878173	13617947	7.10E-01	FBLN2	4	rs637495	37806344	3.78E-02	TBC1D1	-	5.95E-07	3.25E-01	4.19E-03	8487	
2	rs10165206	1.86E+08	1.03E-01	N/A	3	rs7433389	18249117	7.25E-01	N/A	-	5.95E-07	3.47E-01	1.33E-05	8488	
8	rs2245198	98472949	2.31E-01	N/A	12	rs7138803	48533735	8.62E-01	FAIM2	-	5.95E-07	3.93E-01	5.14E-04	8489	
6	rs9382658	56579361	9.61E-01	DST	11	rs11228925	56679937	7.32E-01	N/A	-	5.95E-07	2.11E-01	6.02E-07	8490	
4	rs549815	63958061	9.12E-01	N/A	13	rs2607642	1.02E+08	7.96E-01	FGF14	-	5.95E-07	3.92E-01	6.19E-04	8491	
2	rs11677276	2.18E+08	8.94E-01	TNS1	12	rs7958606	1.27E+08	5.18E-01	TMEM132C	-	5.95E-07	8.07E-01	6.47E-04	8492	
3	rs2276768	30705243	1.10E-01	TGFBR2	8	rs2960106	1.39E+08	3.74E-01	N/A	-	5.95E-07	3.46E-01	2.01E-04	8493	
5	rs17457012	38792000	9.44E-01	N/A	16	rs1947771	76445767	7.38E-01	VAT1L	-	5.95E-07	4.41E-01	4.59E-06	8494	
4	rs4862612	1.87E+08	6.27E-04	N/A	11	rs17143395	81687956	6.24E-02	N/A	-	5.95E-07	8.65E-02	2.07E-03	8495	
4	rs7665839	11275490	9.56E-01	N/A	21	rs1014602	17263238	6.54E-01	N/A	-	5.95E-07	4.35E-01	1.03E-03	8496	
3	rs1378800	1.59E+08	1.51E-01	VEPH1	13	rs7992004	1.09E+08	9.19E-01	MYO16	-	5.95E-07	8.61E-01	1.94E-05	8497	
5	rs4008125	1.74E+08	8.11E-01	N/A	8	rs7836624	1838029	9.71E-01	ARHGEF10	-	5.95E-07	9.22E-02	1.15E-06	8498	
6	rs807898	1.65E+08	1.00E+00	N/A	12	rs4126012	1.18E+08	4.63E-02	N/A	-	5.95E-07	5.20E-01	3.64E-06	8499	
5	rs389333	1.25E+08	6.22E-01	N/A	14	rs17277375	19979312	4.37E-01	TMEM55B	-	5.95E-07	9.99E-01	1.23E-04	8500	
5	rs420280	1.25E+08	6.22E-01	N/A	14	rs17277375	19979312	4.37E-01	TMEM55B	-	5.95E-07	9.99E-01	1.23E-04	8501	
4	rs533595	1.06E+08	2.64E-01	N/A	9	rs932705	1.18E+08	9.04E-01	PAPPA	-	5.95E-07	1.18E-01	4.49E-07	8502	
2	rs17296210	2.33E+08	6.58E-02	EIF4E2	15	rs765763	20428330	7.43E-01	CYFIP1	-	5.95E-07	1.31E-01	2.01E-06	8503	
1	rs270764	44796929	8.32E-01	RNF220	8	rs1615810	5555216	4.96E-01	N/A	-	5.95E-07	2.75E-01	3.68E-06	8504	
5	rs1114534	1.16E+08	6.14E-01	N/A	20	rs6069674	54264681	7.71E-01	MC3R	-	5.95E-07	5.54E-01	1.18E-03	8505	
3	rs6786950	903456	9.05E-01	N/A	7	rs2237782	1.26E+08	2.66E-01	GRM8	-	5.95E-07	8.68E-01	2.19E-04	8506	
4	rs4862612	1.87E+08	6.27E-04	N/A	11	rs7928596	81675813	7.26E-02	N/A	-	5.95E-07	6.40E-02	2.66E-03	8507	
1	rs155646	1.12E+08	1.75E-01	N/A	8	rs6992951	18074257	7.61E-01	N/AT1	-	5.95E-07	2.79E-01	9.08E-06	8508	
8	rs966703	49805796	2.29E-01	EFCAB1	11	rs10840300	9753303	5.43E-01	SBF2	-	5.95E-07	9.72E-01	2.48E-04	8509	
2	rs10183172	2.42E+08	5.08E-01	SNED1	11	rs835764	44868055	4.14E-01	TSPAN18	-	5.95E-07	9.74E-01	4.00E-05	8510	

SNP A					SNP B						Interaction P			Ranking	Cluster in top 100 interactions
CHR	SNP	Location	gle locus P va	Gene	CHR	SNP	Location	Single locus P valu	Gene	MHC region	Stage 1	Stage 2	Combined		
7	rs17132513	2630740	8.24E-01	IQCE	10	rs1907360	17145674	4.78E-01	CUBN	-	5.96E-07	2.02E-01	1.54E-06	8511	
3	rs7623065	22360379	1.74E-01	N/A	14	rs10150688	1.06E+08	2.72E-01	N/A	-	5.96E-07	N/A	N/A	8512	
4	rs4698742	14085628	5.31E-01	N/A	8	rs2978043	1.35E+08	7.11E-01	ST3GAL1	-	5.96E-07	7.47E-02	5.40E-07	8513	
6	rs12660608	1.23E+08	8.18E-02	CLVS2	1	rs12030667	1.5E+08	1.59E-01	N/A	-	5.96E-07	1.24E-01	1.89E-03	8514	
6	rs4618489	85230221	9.25E-01	N/A	4	rs10016978	5979088	7.92E-01	N/A	-	5.96E-07	9.56E-01	8.30E-05	8515	
6	rs9362053	85234736	9.25E-01	N/A	4	rs10016978	5979088	7.92E-01	N/A	-	5.96E-07	9.56E-01	8.30E-05	8516	
3	rs2239624	1.88E+08	2.65E-01	DGKG	7	rs2299418	93870261	9.95E-02	COL1A2	-	5.96E-07	4.47E-01	6.88E-05	8517	
8	rs11776172	5192944	2.49E-01	N/A	12	rs3825181	1.28E+08	7.31E-01	SLC15A4	-	5.96E-07	5.90E-01	4.77E-04	8518	
2	rs13025722	53525558	8.86E-01	N/A	14	rs2069600	95782782	1.58E-02	BDKRB2	-	5.96E-07	6.22E-01	1.19E-05	8519	
6	rs2764075	7300517	1.24E-01	N/A	21	rs244268	23314957	1.13E-01	N/A	-	5.96E-07	1.81E-01	5.11E-06	8520	
7	rs4128374	70648071	1.51E-01	WBSCR17	9	rs2300932	1.23E+08	6.11E-01	C5	-	5.96E-07	1.20E-01	2.89E-06	8521	
7	rs1551098	33564322	9.00E-01	BBS9	21	rs9636594	15050542	3.07E-01	N/A	-	5.96E-07	7.26E-01	8.79E-06	8522	
10	rs7894083	5933274	5.85E-02	ANKRD16	16	rs4888867	77449191	1.21E-01	WWOX	-	5.96E-07	3.86E-02	8.84E-07	8523	
5	rs37545	82270553	8.00E-01	N/A	14	rs3751289	61053696	2.25E-01	PRKCH	-	5.96E-07	8.05E-01	1.76E-04	8524	
5	rs37545	82270553	8.00E-01	N/A	14	rs10483739	61053005	2.25E-01	PRKCH	-	5.96E-07	8.13E-01	1.65E-04	8525	
7	rs10488617	37903558	1.53E-01	TXNDC3	15	rs10519835	31684497	8.50E-01	RYR3	-	5.96E-07	9.29E-01	3.76E-04	8526	
1	rs986034	46999414	1.14E-01	N/A	8	rs4620270	1.26E+08	8.89E-01	N/A	-	5.96E-07	8.39E-01	1.63E-03	8527	
1	rs12568675	85697052	6.54E-01	DDAH1	12	rs10507259	1.14E+08	7.42E-01	N/A	-	5.97E-07	1.64E-01	1.84E-05	8528	
4	rs7687468	58807916	4.05E-01	N/A	10	rs12774333	77838858	3.76E-01	C10orf11	-	5.97E-07	5.95E-01	1.74E-05	8529	
4	rs1480967	34845120	7.85E-01	N/A	4	rs1346715	1.27E+08	8.39E-02	N/A	-	5.97E-07	6.04E-01	3.33E-05	8530	
5	rs7736632	1.59E+08	4.34E-01	LOC285627	7	rs1005387	1.46E+08	5.78E-01	CNTN/AP2	-	5.97E-07	N/A	N/A	8531	
7	rs17621204	11322531	3.16E-01	N/A	17	rs17819558	51001239	1.50E-01	N/A	-	5.97E-07	3.18E-01	4.30E-03	8532	
8	rs2514809	95250422	7.09E-01	CDH17	17	rs940009	22924910	3.78E-01	KSR1	-	5.97E-07	5.45E-01	2.66E-05	8533	
10	rs4750244	12713166	7.68E-01	CAMK1D	16	rs1364301	81941269	1.09E-01	CDH13	-	5.97E-07	5.84E-01	6.40E-04	8534	
4	rs6848941	1.02E+08	1.83E-01	N/A	17	rs639679	44254253	9.84E-01	CALCOCO2	-	5.97E-07	9.19E-01	2.01E-04	8535	
3	rs10935636	1.48E+08	8.02E-01	N/A	13	rs1974254	1.03E+08	1.82E-01	N/A	-	5.97E-07	6.00E-01	3.77E-05	8536	
6	rs9342394	65485313	1.00E+00	EYS	3	rs2243131	1.61E+08	3.19E-01	IL12A	-	5.97E-07	6.18E-01	4.76E-05	8537	
10	rs814628	90419632	7.73E-01	LIPF	21	rs2837693	40832232	1.95E-01	DSCAM	-	5.97E-07	1.88E-01	7.76E-04	8538	
12	rs17097506	45709198	9.67E-01	N/A	22	rs2018340	41720263	8.72E-01	N/A	-	5.97E-07	2.73E-01	2.30E-03	8539	
6	rs7763695	34180539	6.05E-01	GRM4	2	rs12616994	15291126	6.39E-01	NBAS	MHC	5.97E-07	3.83E-01	1.50E-03	8540	
1	rs1506950	1.64E+08	1.92E-01	RXRG	18	rs17072163	59740628	5.87E-01	SERPINB2	-	5.97E-07	8.26E-01	1.15E-04	8541	
12	rs1553380	56897140	2.79E-01	N/A	21	rs2257906	18085674	7.74E-01	C21orf91	-	5.97E-07	1.56E-01	4.65E-03	8542	
9	rs1332349	77015553	6.51E-02	N/A	21	rs648648	33019856	4.18E-01	SYNJ1	-	5.97E-07	1.61E-01	2.02E-03	8543	
1	rs7517475	66070749	3.91E-01	PDE4B	15	rs749938	89293881	4.12E-01	UNC45A	-	5.97E-07	3.29E-01	6.69E-04	8544	
7	rs17735647	53031873	2.98E-01	N/A	18	rs2169014	8354598	2.87E-01	PTPRM	-	5.97E-07	6.43E-01	7.23E-04	8545	
4	rs1353583	1.9E+08	6.65E-01	N/A	11	rs1943761	1.01E+08	4.04E-01	N/A	-	5.97E-07	8.22E-01	2.59E-04	8546	
3	rs1018175	43337601	2.22E-01	SNRK	17	rs4078429	75949967	1.87E-01	RNF213	-	5.97E-07	9.99E-01	7.74E-05	8547	
1	rs761092	11600782	7.60E-01	N/A	7	rs10257188	92430599	9.82E-01	N/A	-	5.97E-07	1.31E-01	8.58E-06	8548	
4	rs767002	1.81E+08	3.53E-01	N/A	12	rs3825251	1.08E+08	6.46E-01	SVOP	-	5.97E-07	6.68E-02	5.06E-03	8549	
6	rs192021	3676722	3.56E-01	C6orf145	18	rs1354274	73544313	6.29E-01	N/A	-	5.97E-07	3.87E-01	7.02E-04	8550	
6	rs192021	3676722	3.56E-01	C6orf145	18	rs1995448	73552199	6.29E-01	N/A	-	5.97E-07	3.87E-01	7.02E-04	8551	
5	rs4700646	63990500	7.85E-01	N/A	9	rs2820957	7215030	9.25E-01	N/A	-	5.97E-07	4.43E-01	4.15E-06	8552	
2	rs10490204	1.02E+08	9.98E-01	IL18RAP	11	rs7119726	70136496	4.88E-02	SHANK2	-	5.97E-07	4.73E-01	1.06E-05	8553	
1	rs2090281	1.64E+08	3.48E-02	ALDH9A1	8	rs7820543	1.21E+08	8.51E-01	DEPDC6	-	5.97E-07	8.74E-01	6.86E-05	8554	
15	rs9920450	53480496	5.42E-01	CCPG1	20	rs4401268	11893421	4.17E-01	N/A	-	5.97E-07	5.31E-01	8.17E-04	8555	
8	rs1480691	14808735	7.02E-01	SGCZ	22	rs5762879	27616180	3.77E-01	ZNRF3	-	5.97E-07	5.34E-01	5.16E-04	8556	
6	rs3798979	69738381	5.05E-01	BAI3	14	rs175646	74993419	5.10E-01	JDP2	-	5.97E-07	6.28E-01	5.00E-05	8557	
15	rs6576311	22284089	7.23E-01	N/A	21	rs7276462	30351507	1.67E-01	N/A	-	5.98E-07	5.77E-01	2.19E-05	8558	
6	rs559102	53560019	6.43E-01	N/A	8	rs1494754	1.16E+08	1.09E-01	N/A	-	5.98E-07	9.76E-01	1.11E-04	8559	
1	rs4845391	1.53E+08	9.66E-01	KCNN3	4	rs17644018	70966894	5.35E-01	CSN1S2A	-	5.98E-07	2.24E-01	1.12E-05	8560	
2	rs7597348	2.32E+08	6.17E-02	NMUR1	15	rs11635018	51846856	3.02E-01	WDR72	-	5.98E-07	2.61E-01	6.63E-04	8561	
7	rs17164162	11266179	6.32E-01	N/A	17	rs941284	52757004	1.36E-01	MSI2	-	5.98E-07	4.87E-01	5.48E-04	8562	
2	rs4674952	2.26E+08	5.40E-01	DOCK10	10	rs7893153	43712417	1.24E-02	N/A	-	5.98E-07	6.64E-01	1.46E-05	8563	
6	rs12205120	1.55E+08	4.76E-01	IPCEF1	10	rs2069178	4118024	7.87E-01	N/A	-	5.98E-07	7.12E-01	2.81E-04	8564	
8	rs12544454	1.09E+08	1.25E-01	N/A	8	rs10105826	1.32E+08	8.22E-01	N/A	-	5.98E-07	9.05E-02	9.54E-07	8565	
5	rs10045058	1.24E+08	6.96E-01	N/A	11	rs1940640	94000460	4.42E-01	PIWIL4	-	5.98E-07	N/A	N/A	8566	
1	rs2772304	68409168	9.26E-02	GPR177	14	rs1953553	19730945	9.93E-01	OR11G2	-	5.98E-07	5.69E-01	9.54E-06	8567	
6	rs4513756	91944287	9.40E-01	N/A	1	rs1342780	90971264	5.87E-01	BARHL2	-	5.98E-07	6.17E-01	3.19E-05	8568	
6	rs3778082	1.52E+08	1.77E-01	ESR1	3	rs2568911	1.96E+08	3.66E-01	N/A	-	5.98E-07	N/A	N/A	8569	
3	rs9837076	1.14E+08	1.15E-01	N/A	19	rs12462307	63476544	3.48E-01	ZNF544	-	5.98E-07	3.92E-01	3.05E-04	8570	
3	rs4449296	77477230	9.21E-01	ROBO2	11	rs3782046	1.18E+08	4.53E-01	CD3D	-	5.98E-07	7.65E-01	2.82E-04	8571	
8	rs2407741	4922282	1.34E-01	N/A	15	rs11638799	48203888	2.11E-02	ATP8B4	-	5.98E-07	7.70E-01	3.85E-04	8572	
1	rs1217057	1.56E+08	4.85E-01	KIRREL	13	rs9518810	1.02E+08	5.48E-01	TPP2	-	5.98E-07	8.01E-01	2.08E-04	8573	
5	rs33376	1.71E+08	8.11E-01	N/A	8	rs2948587	8493973	3.40E-01	N/A	-	5.98E-07	5.92E-01	9.58E-06	8574	
9	rs17778819	27289620	6.83E-01	NCRN/A00032	11	rs7928347	1.2E+08	9.89E-01	GRIK4	-	5.98E-07	6.47E-01	3.16E-04	8575	
4	rs6845251	1.84E+08	1.20E-01	WWC2	15	rs2899793	73247816	3.52E-01	N/A	-	5.98E-07	7.81E-01	2.67E-04	8576	
2	rs4850931	1E+08	8.67E-01	CHST10	3	rs6777841	1.88E+08	1.38E-01	N/A	-	5.99E-07	8.51E-02	7.02E-07	8577	
3	rs2049233	1.98E+08	6.09E-01	PAK2	4	rs6419260	83823263	8.56E-01	SCD5	-	5.99E-07	7.96E-01	3.19E-04	8578	
6	rs1407180	1.18E+08	4.25E-02	ROS1	9	rs10820964	94081457	9.62E-01	IARS	-	5.99E-07	N/A	N/A	8579	
2	rs12694364	2.16E+08	5.20E-01	N/A	3	rs6797616	81313323	7.54E-02	N/A	-	5.99E-07	7.38E-02	6.31E-07	8580	
7	rs10266901	22235570	7.72E-01	RAPGEF5	19	rs3764625	54340863	3.30E-01	N/A	-	5.99E-07	8.64E-02	1.11E-05	8581	
2	rs984682	63199425	5.20E-01	C2orf86	20	rs966534	5735785	9.38E-02	C20orf196	-	5.99E-07	6.99E-01	1.11E-04	8582	
2	rs7582382	98753392	8.87E-01	N/A	5	rs4866662	2749580	8.51E-01	N/A	-	5.99E-07	8.39E-01	6.89E-05	8583	
7	rs10265394	26194302	2.30E-01	NFE2L3	11	rs7950929	97023895	8.03E-01	N/A	-	5.99E-07	9.01E-01	1.28E-04	8584	
13	rs1323946	19572044	7.20E-01	ZMYM2	20	rs2869313	46011510	3.26E-01	N/A	-	5.99E-07	5.64E-01	3.13E-04	8585	
8	rs1062998	23758017	4.33E-01	STC1	12	rs10505755	10203229	3.82E-01	OLR1	-	5.99E-07	9.71E-01	1.15E-04	8586	
8	rs1062998	23758017	4.33E-01	STC1	12	rs3736233	10204532	3.82E-01	OLR1	-	5.99E-07	9.71E-01	1.15E-04	8587	
8	rs1062998	23758017	4.33E-01	STC1	12	rs3736234	10204401	3.82E-01	OLR1	-	5.99E-07	9.71E-01	1.15E-04	8588	
8	rs1062998	23758017	4.33E-01	STC1	12	rs6488265	10207064	3.82E-01	OLR1	-	5.99E-07	9.71E-01	1.15E-04	8589	
3	rs4678999	33458748	6.60E-01	UBP1	8	rs4487751	40684150	6.22E-01	ZMAT4	-	5.99E-07	3.61E-02	1.78E-03	8590	
5	rs590575	55762933	6.54E-01	N/A	10	rs10900250	42390831	9.92E-01	ZNF33B	-	5.99E-07	1.74E-01	1.23E-03	8591	
2	rs4675374	2.05E+08	5.19E-01	ICOS	13	rs2388079	28898621	8.35E-01	MTUS2	-	5.99E-07	5.66E-01	4.40E-05	8592	
3	rs6803988	46696666	2.83E-01	ALS2CL	4	rs4597805	46759253	3.50E-02	GABRB1	-	5.99E-07	9.71E-01	6.13E-05	8593	
4	rs9762579	1.66E+08	3.08E-01	TRIM61	13	rs1374010	26193317	2.59E-01	N/A	-	5.99E-07	4.50E-01	1.84E-03	8594	
11	rs1462183	40033122	8.26E-01	N/A	19	rs266849	56040902	9.87E-01	KLK15	-	5.99E-07	2.10E-02	8.30E-08	8595	
1	rs1935571	96186923	9.54E-01	N/A	4	rs12513097	1.28E+08	3.70E-01	N/A	-	5.99E-07	8.44E-01	1.75E-04	8596	
2	rs16854563	2.16E+08	2.75E-01	N/A	5	rs1012176	11373538	8.64E-01	CTNND2	-	5.99E-07	4.17E-01	1.70E-04	8597	
17	rs4791759	7618652	5.09E-01	DN/AH2	20	rs2027237	4125104	9.56E-02	SMOX	-	5.99E-07	6.54E-01	1.50E-05	8598	
2	rs921876	1.09E+08	2.05E-01	N/A	3	rs2670104	1.33E+08	7.90E-01	N/A	-	5.99E-07	8.48E-01	1.01E-04	8599	
2	rs2678272	2.16E+08	2.75E-01	N/A	5	rs1012176	11373538	8.64E-01	CTNND2	-	5.99E-07	N/A	N/A	8600	
6	rs9358604	23033510	1.39E-02	N/A	12	rs10848287	1.3E+08	9.21E-02	GPR133	-	6.00E-07	5.50E-01	3.86E-03	8601	
2	rs166379	1.81E+08	1.97E-02	CWC22	12	rs10843938	31439162	2.86E-01	DENND5B	-	6.00E-07	6.55E-01	7.35E-05	8602	
2	rs166379	1.81E+08	1.97E-02	CWC22	12	rs7313889	31463494	2.86E-01	DENND5B	-	6.00E-07	6.55E-01	7.35E-05	8603	
2	rs3210400	2.39E+08	4.66E-01	SCLY	3	rs710451	1.88E+08	1.02E-01	MASP1	-	6.00E-07	4.80E-01	1.55E-04	8604	
2	rs3927971	8693553	8.53E-01	N/A	7	rs10250152	20478452	5.35E-02	N/A	-	6.00E-07	2.84E-02	3.59E-07	8605	
2	rs3927971	8693553	8.53E-01	N/A	7	rs11971231	20472872	5.35E-02	N/A	-	6.00E-07	2.84E-02	3.59E-07	8606	
1	rs4949631	76671543	6.92E-01	ST6GALN/AC3	3	rs6792584	67614078	1.34E-01	N/A	-	6.00E-07	6.70E-01	3.95E-05	8607	
5	rs9325009	1.46E+08	7.15E-01	N/A	12	rs2072137	1.12E+08	1.26E-01	OAS2	-	6.00E-07	1.88E-01	2.06E-03	8608	
8	rs4579575	1.39E+08	1.41E-01	FAM135B	19	rs1793978	50529109	2.65E-01	KLC3	-	6.00E-07	2.94E-01	7.89E-06	8609	
1	rs9699681	2.13E+08	8.99E-01	PTPN14	18	rs12604302	27573421	7.98E-01	N/A	-	6.00E-07	9.11E-01	1.20E-04	8610	
11	rs1039750	94449474	8.02E-01	ENDOD1	15	rs1124962	92601453	3.30E-02	N/A	-	6.00E-07	1.40E-02	2.69E-02	8611	
8	rs1390586	50809815	9.60E-01	N/A	20	rs6014949	55186217	1.65E-01	BMP7	-	6.00E-07	1.22E-01	1.92E-03	8612	
1	rs12239394	2.03E+08	4.27E-01	NFASC	15	rs4326996	61567335	8.75E-01	USP3	-	6.00E-07	4.89E-01	7.07E-04	8613	
10	rs7097370	19049704	2.15E-01	N/A	12	rs10771391	27966867	9.98E-01	N/A	-	6.00E-07	7.85E-01	1.73E-04	8614	
1	rs2810587	41745682	2.48E-01	HIVEP3	13	rs9538206	58259825	2.94E-01	N/A	-	6.00E-07	5.44E-01	3.38E-05	8615	
7	rs2043978	62099233	8.06E-01	N/A	10	rs2297922	80498251	6.49E-01	LOC283050	-	6.00E-07	5.77E-01	1.43E-05	8616	
1	rs12140479	70595826	2.66E-01	ANKRD13C	8	rs2196255	69738891	7.00E-02	C8orf34	-	6.01E-07	9.40E-02	4.07E-03	8617	
11	rs1505193	4666863	8.15E-01	OR51E2	22	rs5768016	46595486	9.66E-01	N/A	-	6.01E-07	1.06E-01	2.96E-07	8618	
5	rs9784716	82965743	7.54E-01	HAPLN1	16	rs322609	62432854	9.57E-01	N/A	-	6.01E-07	1.74E-01	4.29E-03	8619	
2	rs1470874	1.08E+08	5.98E-01	SULT1C3	5	rs173878	1.73E+08	1.84E-01	BNIP1	-	6.01E-07	4.72E-01	1.54E-03	8620	
1	rs12042089	1.98E+08	7.54E-01	N/A	2	rs6734151	5387284	4.49E-01	N/A	-	6.01E-07	7.28E-02	7.94E-07	8621	
2	rs6756848	66215108	5.77E-01	N/A	15	rs4128671	95771967	8.82E-01	N/A	-	6.01E-07	5.99E-01	2.33E-04	8622	

SNP A					SNP B						Interaction P				
CHR	SNP	Location	gle locus P va	Gene	CHR	SNP	Location	Single locus P valu	Gene	MHC region	Stage 1	Stage 2	Combined	Ranking	Cluster in top 100 interactions
1	rs12119911	5616797	8.09E-01	N/A	4	rs10866218	1.79E+08	5.02E-01	LOC285501	-	6.01E-07	6.79E-01	2.32E-03	8623	
6	rs742543	3667442	1.34E-01	C6orf145	12	rs7135583	1.29E+08	8.49E-01	TMEM132D	-	6.01E-07	2.62E-01	1.32E-05	8624	
5	rs889268	37917929	2.47E-01	N/A	12	rs10772420	11065543	5.65E-01	PRH1	-	6.01E-07	6.77E-01	2.61E-04	8625	
5	rs889268	37917929	2.47E-01	N/A	12	rs1551191	11220900	5.65E-01	PRH1	-	6.01E-07	6.77E-01	2.61E-04	8626	
5	rs889268	37917929	2.47E-01	N/A	12	rs2010481	11071607	5.65E-01	PRH1	-	6.01E-07	6.77E-01	2.61E-04	8627	
5	rs889268	37917929	2.47E-01	N/A	12	rs2600359	11175030	5.65E-01	PRH1	-	6.01E-07	6.77E-01	2.61E-04	8628	
5	rs889268	37917929	2.47E-01	N/A	12	rs2708389	11096007	5.65E-01	PRH1	-	6.01E-07	6.77E-01	2.61E-04	8629	
5	rs889268	37917929	2.47E-01	N/A	12	rs7486717	11204215	5.65E-01	PRH1	-	6.01E-07	6.77E-01	2.61E-04	8630	
1	rs11121648	5846375	3.33E-01	NPHP4	12	rs10848943	3814283	7.50E-01	PARP11	-	6.01E-07	8.07E-01	1.05E-04	8631	
5	rs4704108	73428422	5.84E-01	N/A	7	rs42322	28657021	9.27E-02	CREB5	-	6.01E-07	3.07E-02	6.41E-03	8632	
4	rs12509055	1.69E+08	5.12E-01	N/A	9	rs7866311	1.31E+08	3.28E-01	METTL11A	-	6.01E-07	2.42E-01	1.58E-03	8633	
3	rs6800560	24825476	1.33E-02	N/A	4	rs4269197	1.16E+08	9.95E-01	N/A	-	6.01E-07	7.34E-01	2.54E-05	8634	
1	rs1833036	2.15E+08	6.81E-01	ESRRG	20	rs8567	61992759	2.99E-01	TPD52L2	-	6.01E-07	2.89E-01	8.42E-04	8635	
1	rs1001304	2.11E+08	1.99E-01	ATF3	2	rs842663	74900714	2.82E-01	HK2	-	6.01E-07	6.92E-01	3.80E-05	8636	
3	rs9422	1.25E+08	8.04E-01	MYLK	12	rs10492029	1.12E+08	1.95E-01	OAS2	-	6.01E-07	8.61E-01	2.92E-05	8637	
10	rs1248423	29322308	7.62E-01	N/A	13	rs4771419	1.02E+08	9.85E-01	FGF14	-	6.01E-07	2.58E-01	1.21E-05	8638	
13	rs11616409	80469691	4.13E-01	N/A	14	rs1953417	64948662	6.54E-02	FUT8	-	6.01E-07	3.45E-01	5.14E-06	8639	
4	rs10035076	1.52E+08	6.37E-01	LRBA	13	rs9301361	1.09E+08	3.45E-01	MYO16	-	6.01E-07	4.30E-01	3.20E-05	8640	
9	rs4745511	77952115	7.17E-01	N/A	11	rs10897314	55378348	2.77E-01	OR5D16	-	6.01E-07	4.72E-01	6.40E-04	8641	
1	rs514163	1.64E+08	5.44E-01	UCK2	16	rs42270	12468247	6.78E-01	SNX29	-	6.01E-07	6.63E-01	1.58E-04	8642	
4	rs1878685	1.69E+08	6.05E-01	N/A	12	rs7970396	59254503	6.20E-01	N/A	-	6.01E-07	7.67E-01	5.17E-04	8643	
11	rs1787666	65005665	3.80E-01	MALAT1	12	rs7980937	77250807	9.01E-01	N/A	-	6.02E-07	7.98E-01	1.88E-05	8644	
3	rs7623065	22360379	1.74E-01	N/A	14	rs10151262	1.06E+08	3.78E-01	N/A	-	6.02E-07	N/A	N/A	8645	
2	rs10182124	1.87E+08	7.40E-01	N/A	10	rs10823403	70967730	3.46E-01	N/A	-	6.02E-07	2.67E-01	1.85E-06	8646	
8	rs10101117	29922269	8.58E-01	LOC286135	14	rs1554957	77077624	2.75E-01	SPTLC2	-	6.02E-07	2.74E-01	9.28E-06	8647	
2	rs1106518	69181722	2.66E-01	ANTXR1	18	rs2868929	10097749	2.97E-01	N/A	-	6.02E-07	8.42E-01	1.11E-04	8648	
2	rs7582076	1.17E+08	6.33E-01	N/A	15	rs9920389	93832564	9.64E-01	LOC145820	-	6.02E-07	9.04E-01	1.50E-04	8649	
1	rs6686875	68491604	5.52E-01	N/A	12	rs1986727	1.05E+08	6.06E-01	N/A	-	6.02E-07	3.92E-01	2.87E-06	8650	
7	rs1917361	1.24E+08	2.36E-01	N/A	11	rs495330	64478484	9.67E-01	C11orf85	-	6.02E-07	4.20E-01	2.25E-05	8651	
6	rs2764075	7300517	1.24E-01	N/A	21	rs198072	23344509	3.65E-01	N/A	-	6.02E-07	4.69E-01	1.85E-05	8652	
6	rs2764075	7300517	1.24E-01	N/A	21	rs398570	23338997	3.65E-01	N/A	-	6.02E-07	4.69E-01	1.85E-05	8653	
2	rs11688532	1.28E+08	1.78E-01	N/A	7	rs2043221	14432417	3.54E-01	DGKB	-	6.02E-07	5.71E-01	4.98E-04	8654	
2	rs12466450	36474457	3.37E-01	CRIM1	20	rs3933284	879743	7.84E-01	RSPO4	-	6.02E-07	9.55E-01	6.52E-04	8655	
1	rs12125484	92584784	3.43E-01	RPAP2	3	rs10510803	59329572	6.53E-02	N/A	-	6.02E-07	3.24E-01	1.12E-03	8656	
9	rs1475784	1.38E+08	8.78E-01	N/A	15	rs7166977	64720857	6.18E-01	N/A	-	6.02E-07	N/A	N/A	8657	
7	rs2346444	1.33E+08	5.91E-01	EXOC4	18	rs341237	6391283	7.19E-01	L3MBTL4	-	6.03E-07	7.63E-02	4.25E-07	8658	
2	rs12695010	2.41E+08	4.64E-01	N/A	7	rs2057761	26136637	4.47E-01	N/A	-	6.03E-07	5.27E-01	2.85E-05	8659	
6	rs2747690	1.64E+08	7.58E-01	PACRG	12	rs2286383	1761633	4.27E-01	ADIPOR2	-	6.03E-07	8.23E-01	3.43E-05	8660	
4	rs12505957	1.55E+08	6.88E-01	N/A	11	rs648067	1.19E+08	4.25E-01	N/A	-	6.03E-07	6.68E-01	7.26E-06	8661	
6	rs1407180	1.18E+08	4.25E-02	ROS1	9	rs10739927	94091902	9.03E-01	IARS	-	6.03E-07	9.71E-01	2.37E-04	8662	
6	rs1407180	1.18E+08	4.25E-02	ROS1	9	rs7023004	94138533	9.03E-01	CENPP	-	6.03E-07	9.71E-01	2.37E-04	8663	
11	rs623018	1.06E+08	8.27E-01	GUCY1A2	20	rs4811895	55639715	7.93E-02	PMEPA1	-	6.03E-07	8.20E-02	2.78E-03	8664	
1	rs11208758	66059788	5.82E-01	PDE4B	15	rs2304939	89290806	3.80E-01	UNC45A	-	6.03E-07	2.23E-01	9.67E-04	8665	
1	rs908319	2.33E+08	6.89E-01	N/A	12	rs1468795	66536637	4.80E-01	N/A	-	6.03E-07	7.75E-01	5.43E-04	8666	
1	rs938455	2.33E+08	6.89E-01	N/A	12	rs1468795	66536637	4.80E-01	N/A	-	6.03E-07	8.37E-01	4.44E-04	8667	
7	rs10250954	22441474	5.74E-01	MGC87042	16	rs4785763	88594437	5.14E-01	AFG3L1	-	6.03E-07	9.39E-01	3.79E-05	8668	
9	rs16915442	1E+08	9.32E-02	GABBR2	11	rs1007369	1.19E+08	9.34E-01	N/A	-	6.03E-07	2.85E-02	6.65E-03	8669	
2	rs11692361	66577203	4.35E-01	MEIS1	19	rs11882529	7561810	2.76E-01	KIAA1543	-	6.03E-07	8.19E-02	1.47E-02	8670	
7	rs799929	79935512	5.25E-01	GNAT3	7	rs10271646	1.24E+08	8.14E-01	POT1	-	6.03E-07	1.13E-01	6.86E-07	8671	
7	rs799929	79935512	5.25E-01	GNAT3	7	rs727505	1.24E+08	8.14E-01	POT1	-	6.03E-07	1.13E-01	6.86E-07	8672	
7	rs799929	79935512	5.25E-01	GNAT3	7	rs7801661	1.24E+08	8.14E-01	POT1	-	6.03E-07	1.13E-01	6.86E-07	8673	
7	rs799929	79935512	5.25E-01	GNAT3	7	rs1541416	1.24E+08	8.14E-01	POT1	-	6.03E-07	1.18E-01	7.37E-07	8674	
7	rs799929	79935512	5.25E-01	GNAT3	7	rs720614	1.24E+08	8.14E-01	POT1	-	6.03E-07	1.18E-01	7.37E-07	8675	
12	rs12578470	1.29E+08	4.01E-01	TMEM132D	14	rs11847318	30287393	7.26E-01	SCFD1	-	6.03E-07	6.52E-01	2.80E-04	8676	
7	rs4947995	55276124	6.95E-01	N/A	11	rs10891479	1.12E+08	1.52E-01	NCAM1	-	6.03E-07	8.79E-01	3.22E-04	8677	
7	rs10265924	1.56E+08	1.67E-01	LMBR1	17	rs9895907	5521311	2.74E-01	N/A	-	6.03E-07	9.57E-01	7.02E-05	8678	
7	rs10265924	1.56E+08	1.67E-01	LMBR1	17	rs9915238	5520461	3.13E-01	N/A	-	6.03E-07	9.57E-01	7.02E-05	8679	
15	rs2631700	37767618	3.35E-01	FSIP1	21	rs2298515	24980244	5.12E-01	N/A	-	6.03E-07	2.19E-01	4.37E-07	8680	
5	rs9327697	1.34E+08	1.15E-01	PCBD2	14	rs1777685	82201687	8.47E-01	N/A	-	6.03E-07	3.14E-01	1.16E-05	8681	
7	rs10486860	96436762	3.89E-01	DLX6AS	11	rs948028	1.2E+08	5.74E-01	GRIK4	-	6.03E-07	3.73E-01	2.15E-05	8682	
9	rs3010716	80313592	5.32E-01	N/A	15	rs999787	53079743	8.58E-01	N/A	-	6.03E-07	7.81E-01	7.04E-06	8683	
7	rs17171480	35552194	6.06E-01	N/A	8	rs1489587	82878407	5.97E-01	SNX16	-	6.03E-07	7.32E-01	8.24E-05	8684	
7	rs17171480	35552194	6.06E-01	N/A	8	rs1470787	82903057	5.97E-01	SNX16	-	6.03E-07	7.93E-01	1.00E-04	8685	
7	rs17171480	35552194	6.06E-01	N/A	8	rs774468	82885666	5.97E-01	SNX16	-	6.03E-07	7.93E-01	1.00E-04	8686	
11	rs1446160	25045160	6.12E-01	LUZP2	13	rs9599328	68151863	4.00E-01	N/A	-	6.04E-07	2.29E-02	1.03E-07	8687	
3	rs557077	55506307	2.34E-01	ERC2	3	rs3968279	57162118	9.26E-01	IL17RD	-	6.04E-07	4.33E-01	9.45E-06	8688	
8	rs7000324	91105089	4.90E-01	DECR1	12	rs7300317	50918840	7.41E-01	LOC283404	-	6.04E-07	5.57E-01	1.69E-05	8689	
8	rs11985492	5863519	1.12E-01	N/A	12	rs11068208	1.16E+08	3.33E-01	RNFT2	-	6.04E-07	5.63E-01	3.33E-05	8690	
7	rs4717047	1.56E+08	3.65E-02	N/A	9	rs2015843	1.28E+08	4.25E-01	N/A	-	6.04E-07	2.81E-05	2.81E-05	8691	
6	rs7776061	2432060	7.25E-01	N/A	7	rs2111815	1.32E+08	4.69E-01	CHCHD3	-	6.04E-07	6.18E-01	2.64E-04	8692	
6	rs2325963	2424401	7.25E-01	N/A	7	rs2111815	1.32E+08	4.69E-01	CHCHD3	-	6.04E-07	6.81E-01	2.19E-04	8693	
6	rs12665316	1.62E+08	7.18E-01	PARK2	3	rs9830791	57066615	5.67E-01	ARHGEF3	-	6.04E-07	8.90E-02	1.82E-03	8694	
13	rs9550270	1.14E+08	7.77E-01	GAS6	21	rs1999328	38852576	5.82E-01	ERG	-	6.04E-07	4.87E-01	2.53E-05	8695	
2	rs7425274	1.61E+08	2.11E-01	RBMS1	19	rs8100011	49128573	3.05E-01	ZNF45	-	6.04E-07	7.41E-01	1.72E-04	8696	
4	rs10007789	1.66E+08	4.50E-01	TRIM61	13	rs4771012	26188493	2.32E-01	N/A	-	6.04E-07	3.36E-01	2.23E-03	8697	
1	rs10874095	80101868	1.24E-01	N/A	10	rs1890583	3580269	9.20E-01	N/A	-	6.04E-07	8.66E-02	1.93E-06	8698	
3	rs9846698	1.86E+08	7.18E-03	N/A	10	rs7067780	44672425	2.24E-01	N/A	-	6.04E-07	6.50E-01	1.23E-03	8699	
9	rs2890733	6998732	7.97E-01	KDM4C	13	rs7985904	97330758	3.60E-01	N/A	-	6.04E-07	8.10E-01	3.45E-05	8700	
5	rs10053880	1.13E+08	4.61E-01	N/A	9	rs7849752	95431811	1.72E-01	PHF2	-	6.04E-07	8.35E-01	2.56E-04	8701	
5	rs211037	1.61E+08	5.60E-03	GABRG2	11	rs4758104	6390645	3.69E-01	APBB1	-	6.04E-07	4.37E-01	1.54E-05	8702	
2	rs1034380	8290469	5.90E-01	N/A	14	rs1950356	36278007	2.25E-01	SLC25A21	-	6.04E-07	6.86E-01	5.31E-04	8703	
8	rs3780087	6555609	1.24E-01	AGPAT5	10	rs11244948	1.28E+08	6.71E-01	ADAM12	-	6.04E-07	2.16E-01	7.34E-06	8704	
4	rs731284	25480199	2.66E-01	SEL1L3	10	rs754713	44117093	2.13E-01	N/A	-	6.04E-07	8.15E-01	1.55E-05	8705	
1	rs7536260	2.39E+08	1.34E-01	N/A	20	rs910382	51132596	1.74E-02	TSHZ2	-	6.04E-07	7.54E-01	4.22E-04	8706	
2	rs12478463	2.41E+08	3.88E-01	N/A	22	rs137766	42397074	5.12E-01	EFCAB6	-	6.04E-07	7.60E-01	1.68E-04	8707	
6	rs11153000	1.07E+08	8.04E-01	AIM1	2	rs1370512	1.19E+08	8.37E-01	N/A	-	6.05E-07	4.07E-02	1.03E-02	8708	
2	rs2359682	2.07E+08	8.98E-02	DYTN	14	rs2236185	68140517	5.40E-02	RAD51L1	-	6.05E-07	5.43E-01	5.88E-04	8709	
1	rs859448	1.74E+08	5.33E-01	TNR	2	rs10183647	16931006	6.11E-01	N/A	-	6.05E-07	7.58E-01	1.78E-04	8710	
2	rs6705911	1.83E+08	7.14E-01	PPP1R1C	10	rs1316459	1.29E+08	7.58E-01	N/A	-	6.05E-07	8.38E-01	4.97E-04	8711	
10	rs1273180	1.3E+08	2.48E-01	N/A	16	rs4608363	74563413	2.46E-01	N/A	-	6.05E-07	8.60E-01	4.50E-05	8712	
2	rs12478812	2.3E+08	4.41E-01	PID1	3	rs1352409	3785723	1.45E-01	N/A	-	6.05E-07	9.79E-03	4.33E-08	8713	
1	rs1924267	58020360	7.30E-01	DAB1	9	rs11138361	81622122	5.36E-01	N/A	-	6.05E-07	7.12E-01	8.67E-05	8714	
10	rs10828047	20716201	2.54E-01	N/A	21	rs2833294	31429521	7.20E-01	TIAM1	-	6.05E-07	1.11E-02	5.71E-07	8715	
1	rs6671680	1.91E+08	1.19E-01	N/A	9	rs4842022	1.37E+08	4.20E-01	N/A	-	6.05E-07	1.92E-01	5.77E-06	8716	
12	rs4765228	1.24E+08	4.46E-01	N/A	16	rs11076110	54227607	7.21E-01	N/A	-	6.05E-07	3.03E-01	1.26E-03	8717	
5	rs4242263	36451121	1.14E-02	N/A	13	rs9524937	94799350	8.77E-01	N/A	-	6.05E-07	9.85E-01	1.05E-04	8718	
9	rs3739440	37021074	7.19E-02	PAX5	13	rs4771640	1.09E+08	7.81E-01	IRS2	-	6.05E-07	6.27E-01	1.19E-04	8719	
6	rs915013	1.63E+08	6.13E-01	PACRG	20	rs211863	37887581	5.97E-01	N/A	-	6.05E-07	2.72E-02	1.00E-02	8720	
5	rs2344483	1.35E+08	7.99E-01	N/A	18	rs8086028	10727779	1.33E-01	FAM38B	-	6.05E-07	9.54E-02	2.77E-03	8721	
6	rs2439538	13416062	5.23E-01	TBC1D7	2	rs6710260	69183622	5.99E-01	ANTXR1	-	6.05E-07	7.60E-01	1.32E-04	8722	
8	rs1520933	64826826	1.54E-01	N/A	10	rs11202059	81944823	3.91E-01	ANXA11	-	6.05E-07	7.93E-01	3.35E-04	8723	
2	rs1072581	2.26E+08	5.38E-01	DOCK10	10	rs7893153	43712417	1.24E-02	N/A	-	6.06E-07	6.64E-01	1.47E-05	8724	
7	rs12540373	7796065	8.75E-01	N/A	8	rs729360	20174246	2.99E-01	LZTS1	-	6.06E-07	8.88E-01	8.13E-05	8725	
7	rs12540373	7796065	8.75E-01	N/A	8	rs729361	20174275	2.99E-01	LZTS1	-	6.06E-07	9.56E-01	9.76E-05	8726	
1	rs10864471	10772232	8.38E-01	CASZ1	12	rs2072511	94905363	5.05E-01	AMDHD1	-	6.06E-07	3.41E-01	1.47E-05	8727	
7	rs7784835	13157446	6.21E-02	N/A	19	rs10411733	62482800	4.72E-01	ZNF460	-	6.06E-07	2.64E-01	2.76E-06	8728	
6	rs1888274	53040467	5.50E-01	FBXO9	3	rs9841414	1.65E+08	7.88E-01	N/A	-	6.06E-07	5.94E-01	3.71E-05	8729	
9	rs960908	19448939	6.79E-01	ACER2	12	rs3741595	1.21E+08	7.91E-01	ORAI1	-	6.06E-07	4.79E-01	4.54E-04	8730	
3	rs1562464	1.62E+08	7.94E-01	PPM1L	16	rs4783276	81384445	2.94E-01	CDH13	-	6.06E-07	9.65E-01	9.80E-05	8731	
5	rs17113732	1.52E+08	3.34E-01	N/A	13	rs9517587	98526988	5.39E-01	DOCK9	-	6.06E-07	3.73E-01	4.51E-06	8732	
3	rs793494	1.01E+08	7.38E-01	MIR548G	9	rs4877434	90080934	9.39E-01	N/A	-	6.06E-07	2.91E-01	4.11E-03	8733	
2	rs1667059	2925338	8.31E-01	N/A	9	rs2987392	1.34E+08	8.00E-02	N/A	-	6.06E-07	8.17E-01	2.66E-05	8734	

SNP A					SNP B						Interaction P				
CHR	SNP	Location	gle locus P va	Gene	CHR	SNP	Location	ingle locus P valu	Gene	HC region	Stage 1	Stage 2	Combined	Ranking	Cluster in top 100 interactions
2	rs10495824	34604049	3.79E-01	N/A	9	rs10435864	1.15E+08	5.39E-01	FKBP15	-	6.07E-07	2.70E-02	1.08E-07	8735	
11	rs1474500	4080688	2.64E-01	STIM1	17	rs7209298	28877062	6.60E-01	ACCN1	-	6.07E-07	1.94E-01	1.02E-03	8736	
9	rs10120316	7419449	4.50E-01	N/A	19	rs515391	13603702	1.61E-01	N/A	-	6.07E-07	9.25E-01	4.85E-05	8737	
1	rs502036	1.78E+08	1.05E-01	TOR1AIP2	8	rs2269693	17237645	1.60E-01	MTMR7	-	6.07E-07	1.99E-01	1.21E-03	8738	
3	rs3911812	1.33E+08	6.39E-01	N/A	15	rs7183261	92129998	3.06E-01	N/A	-	6.07E-07	2.55E-01	3.20E-06	8739	
1	rs11240748	2.03E+08	7.99E-01	PIK3C2B	3	rs4973706	18896776	4.22E-01	N/A	-	6.07E-07	6.77E-01	1.91E-04	8740	
3	rs1163740	77795273	9.82E-01	ROBO2	7	rs2192073	17634341	3.98E-01	N/A	-	6.07E-07	8.80E-01	1.05E-04	8741	
4	rs4834136	1.28E+08	3.28E-01	N/A	18	rs273754	21445436	5.74E-01	N/A	-	6.07E-07	N/A	N/A	8742	
3	rs7612811	21355529	3.76E-01	N/A	8	rs6530778	14357664	3.34E-01	SGCZ	-	6.07E-07	2.39E-01	1.62E-03	8743	
6	rs4594925	1.69E+08	8.29E-01	N/A	5	rs1469419	65694877	3.67E-01	N/A	-	6.07E-07	4.05E-01	2.87E-05	8744	
1	rs2594289	11536855	1.91E-01	PTCHD2	21	rs11088668	18448920	2.54E-01	N/A	-	6.07E-07	2.04E-01	1.34E-06	8745	
3	rs13068206	1.94E+08	6.74E-01	FGF12	14	rs8008701	96843914	8.87E-01	N/A	-	6.07E-07	6.11E-01	5.05E-05	8746	
4	rs2869678	88652904	7.53E-01	SPARCL1	9	rs4618799	18109661	7.02E-01	N/A	-	6.07E-07	3.03E-01	2.39E-06	8747	
3	rs1513181	1.9E+08	7.51E-01	LPP	14	rs2332909	72233067	4.87E-01	DPF3	-	6.07E-07	3.65E-01	2.35E-03	8748	
3	rs7644727	1.45E+08	8.34E-01	SLC9A9	8	rs2816489	5849941	8.18E-01	N/A	-	6.07E-07	4.12E-01	3.33E-03	8749	
6	rs7762377	1.33E+08	9.42E-01	TAAR1	13	rs7991685	50890718	4.35E-01	INTS6	-	6.08E-07	4.93E-01	1.57E-03	8750	
1	rs10494690	1.92E+08	7.64E-01	N/A	22	rs131853	46141822	2.23E-01	N/A	-	6.08E-07	6.88E-02	3.38E-07	8751	
1	rs4657760	1.92E+08	7.64E-01	N/A	22	rs131853	46141822	2.23E-01	N/A	-	6.08E-07	8.39E-02	4.31E-07	8752	
12	rs1483757	1.16E+08	3.69E-01	NOS1	18	rs12457903	12537561	3.20E-01	SPIRE1	-	6.08E-07	9.96E-01	2.05E-05	8753	
3	rs4680602	1.68E+08	4.45E-01	N/A	10	rs6482574	27095902	7.09E-01	N/A	-	6.08E-07	N/A	N/A	8754	
6	rs9369677	47431213	7.48E-01	N/A	19	rs492734	53977807	8.11E-01	BCAT2	-	6.08E-07	4.91E-01	7.68E-04	8755	
4	rs2570096	58820719	5.52E-01	N/A	9	rs1927099	72025395	4.23E-01	MAMDC2	-	6.08E-07	7.83E-01	2.15E-05	8756	
3	rs1449996	36140983	1.58E-01	N/A	10	rs7897550	17104998	6.52E-01	CUBN	-	6.08E-07	2.51E-01	1.67E-05	8757	
3	rs1992991	36123334	1.58E-01	N/A	10	rs7897550	17104998	6.52E-01	CUBN	-	6.08E-07	2.51E-01	1.67E-05	8758	
2	rs6714466	2.25E+08	5.02E-01	DOCK10	10	rs7076199	43695611	3.38E-02	N/A	-	6.08E-07	5.69E-01	1.11E-05	8759	
2	rs7558808	2.26E+08	5.02E-01	DOCK10	10	rs7076199	43695611	3.38E-02	N/A	-	6.08E-07	5.69E-01	1.11E-05	8760	
2	rs4851111	1.06E+08	7.20E-01	C2orf40	2	rs17006117	1.22E+08	9.88E-01	N/A	-	6.08E-07	1.68E-05	1.68E-05	8761	
4	rs4689830	7759789	2.48E-01	SORCS2	12	rs10735933	64746061	7.54E-01	N/A	-	6.08E-07	1.51E-01	8.57E-03	8762	
10	rs7083204	4504452	2.55E-01	N/A	16	rs4782717	81167218	2.78E-01	N/A	-	6.08E-07	N/A	N/A	8763	
3	rs1021701	25033761	9.90E-01	N/A	8	rs272610	81644892	1.05E-01	N/A	-	6.08E-07	1.26E-01	4.22E-06	8764	
6	rs1331644	1.46E+08	3.38E-01	GRM1	12	rs214680	45965615	9.84E-02	N/A	-	6.08E-07	6.21E-02	5.50E-03	8765	
3	rs6788821	60099620	4.10E-01	FHIT	20	rs2424244	19595527	2.18E-01	SLC24A3	-	6.08E-07	9.09E-01	2.10E-04	8766	
2	rs7421557	1.8E+08	6.78E-01	ZNF385B	3	rs358741	1.57E+08	8.76E-01	C3orf33	-	6.08E-07	9.89E-01	7.47E-04	8767	
2	rs7593072	1.41E+08	4.00E-01	LRP1B	5	rs1585224	32697244	4.34E-01	N/A	-	6.09E-07	2.40E-01	3.21E-04	8768	
6	rs1012252	18733774	8.73E-01	N/A	10	rs10905067	7012015	6.11E-01	N/A	-	6.09E-07	8.35E-01	1.08E-05	8769	
6	rs7748349	18737049	8.73E-01	N/A	10	rs10905067	7012015	6.11E-01	N/A	-	6.09E-07	8.35E-01	1.08E-05	8770	
12	rs2075362	95131020	1.47E-01	ELK3	22	rs5997752	29468869	9.11E-01	OSBP2	-	6.09E-07	1.34E-01	6.91E-06	8771	
8	rs1445560	82047632	5.71E-01	PAG1	21	rs4816682	40723632	1.17E-01	DSCAM	-	6.09E-07	3.92E-01	4.47E-04	8772	
18	rs7236709	43780889	9.98E-01	N/A	21	rs686364	30509664	7.09E-01	CLDN8	-	6.09E-07	4.47E-01	1.09E-04	8773	
3	rs4142860	3371403	4.00E-01	N/A	11	rs7937803	1.22E+08	9.50E-01	N/A	-	6.09E-07	5.66E-01	3.41E-05	8774	
4	rs828152	16672662	9.12E-01	N/A	17	rs7211818	76303498	7.82E-01	RPTOR	-	6.09E-07	6.15E-01	2.99E-04	8775	
2	rs6714955	16564378	8.60E-01	N/A	9	rs970480	73460052	7.68E-01	N/A	-	6.09E-07	7.88E-01	1.45E-04	8776	
2	rs7604116	16562832	8.60E-01	N/A	9	rs970480	73460052	7.68E-01	N/A	-	6.09E-07	7.88E-01	1.45E-04	8777	
4	rs12500207	1.66E+08	6.26E-01	N/A	20	rs6130274	41047898	7.73E-02	PTPRT	-	6.09E-07	2.63E-01	3.24E-03	8778	
2	rs13410950	57506559	3.52E-01	N/A	2	rs4672323	59936804	2.75E-02	N/A	-	6.09E-07	1.48E-02	3.48E-08	8779	
2	rs13410950	57506559	3.52E-01	N/A	2	rs2419402	59932754	2.75E-02	N/A	-	6.09E-07	1.51E-02	3.58E-08	8780	
4	rs7691951	1.54E+08	1.67E-01	N/A	9	rs566618	1.37E+08	7.83E-02	N/A	-	6.09E-07	6.44E-01	7.24E-04	8781	
12	rs12318001	1.03E+08	6.33E-01	N/A	18	rs786038	14928839	5.50E-01	N/A	-	6.09E-07	3.15E-02	9.65E-03	8782	
10	rs10763625	19074957	8.33E-01	N/A	17	rs3967769	9921751	7.44E-01	GAS7	-	6.09E-07	2.70E-02	8.95E-08	8783	
10	rs1981251	61797933	3.88E-01	ANK3	16	rs7194009	19285314	7.18E-02	N/A	-	6.09E-07	3.43E-01	2.38E-03	8784	
6	rs781736	1.25E+08	4.59E-01	N/A	4	rs4235294	21830023	2.24E-02	N/A	-	6.09E-07	8.01E-01	5.54E-05	8785	
8	rs503179	96916516	9.97E-01	N/A	13	rs4500590	34979108	7.02E-01	NBEA	-	6.10E-07	6.51E-03	3.66E-08	8786	
6	rs2523864	31126525	5.29E-01	HCG22	6	rs9380215	31157634	4.29E-02	N/A	MHC	6.10E-07	4.21E-01	1.08E-04	8787	
11	rs1207273	70393683	3.47E-01	SHANK2	15	rs3110075	85573499	6.60E-01	N/A	-	6.10E-07	4.33E-01	3.13E-04	8788	
1	rs7546890	1.59E+08	8.08E-01	F11R	2	rs17586405	2.32E+08	6.61E-01	N/A	-	6.10E-07	6.80E-01	1.29E-04	8789	
12	rs9804747	57572877	2.21E-01	LRIG3	19	rs12980498	23488097	8.63E-01	N/A	-	6.10E-07	9.66E-01	2.64E-05	8790	
4	rs7682684	17849661	5.26E-01	N/A	10	rs11018214	1.29E+08	4.79E-01	N/A	-	6.10E-07	9.95E-02	1.98E-03	8791	
2	rs7569763	2778476	9.79E-01	N/A	7	rs42322	28657021	9.27E-02	CREB5	-	6.10E-07	1.40E-01	1.69E-06	8792	
6	rs9479734	1.5E+08	7.99E-01	PPP1R14C	9	rs10780770	88373314	7.97E-01	N/A	-	6.10E-07	1.94E-01	1.64E-03	8793	
5	rs1030179	1.23E+08	6.89E-01	N/A	10	rs4935677	58332093	2.60E-01	N/A	-	6.10E-07	4.44E-01	3.37E-04	8794	
7	rs2373885	1.5E+08	6.21E-02	KCNH2	8	rs6981476	1.4E+08	2.72E-01	COL22A1	-	6.10E-07	8.65E-01	8.86E-04	8795	
2	rs7573324	1.44E+08	5.75E-01	N/A	3	rs2603133	1.1E+08	8.72E-02	MYH15	-	6.10E-07	2.68E-01	8.57E-06	8796	
4	rs6822279	99964728	1.96E-01	N/A	11	rs10892609	1.2E+08	3.51E-01	GRIK4	-	6.10E-07	4.15E-01	1.56E-05	8797	
6	rs4896802	1.46E+08	5.40E-02	N/A	7	rs3801407	45110028	1.45E-01	N/ACAD	-	6.10E-07	7.43E-01	8.55E-05	8798	
6	rs11961178	87786587	8.78E-01	HTR1E	5	rs4298224	8947289	9.93E-01	N/A	-	6.10E-07	6.65E-02	5.12E-07	8799	
3	rs4325955	30518421	4.48E-01	N/A	5	rs7702304	96057851	3.78E-01	CAST	-	6.10E-07	5.43E-01	1.41E-05	8800	
3	rs12494312	1.76E+08	4.95E-01	N/A	20	rs4810496	44387690	8.39E-01	N/A	-	6.10E-07	6.07E-01	4.25E-04	8801	
2	rs12623155	1.05E+08	1.50E-01	N/A	7	rs7786445	1.55E+08	3.07E-01	N/A	-	6.10E-07	7.85E-01	3.18E-04	8802	
1	rs12089317	22625714	4.12E-01	N/A	11	rs609261	1.08E+08	7.75E-01	ATM	-	6.10E-07	8.36E-01	5.72E-05	8803	
16	rs1684578	55627151	7.80E-01	NLRC5	17	rs2042005	5674282	5.95E-01	N/A	-	6.11E-07	1.49E-01	3.26E-06	8804	
3	rs936154	36462496	5.36E-01	STAC	14	rs1325541	36348098	7.13E-01	SLC25A21	-	6.11E-07	7.13E-01	8.74E-05	8805	
3	rs936154	36462496	5.36E-01	STAC	14	rs1749935	36347898	7.13E-01	SLC25A21	-	6.11E-07	7.13E-01	8.74E-05	8806	
2	rs7609111	76828891	1.89E-01	LRRTM4	5	rs37775	52663553	5.59E-01	N/A	-	6.11E-07	N/A	N/A	8807	
2	rs10928940	1.1E+08	8.08E-01	LOC389033	13	rs3742207	1.1E+08	3.69E-01	COL4A1	-	6.11E-07	3.54E-01	5.91E-06	8808	
3	rs4465894	33191738	8.24E-01	SUSD5	3	rs1395006	1.33E+08	6.48E-01	N/A	-	6.11E-07	9.33E-01	2.04E-04	8809	
12	rs983369	1.27E+08	3.50E-01	N/A	14	rs7155581	57119444	9.88E-01	SLC35F4	-	6.11E-07	1.75E-01	2.18E-03	8810	
7	rs12673905	1.38E+08	4.85E-01	ZC3HAV1	17	rs9893164	9309408	9.72E-01	STX8	-	6.11E-07	2.90E-01	5.96E-05	8811	
6	rs9394952	43509083	3.01E-01	ABCC10	8	rs10112865	88509387	5.95E-01	N/A	-	6.11E-07	3.68E-01	1.51E-05	8812	
6	rs9394952	43509083	3.01E-01	ABCC10	8	rs4961036	88516372	5.95E-01	N/A	-	6.11E-07	4.65E-01	2.56E-05	8813	
1	rs4655724	67771577	4.44E-01	N/A	12	rs2732439	47030466	7.19E-01	N/A	-	6.11E-07	2.59E-01	1.67E-03	8814	
1	rs7539855	67770721	4.44E-01	N/A	12	rs2732439	47030466	7.19E-01	N/A	-	6.11E-07	2.59E-01	1.67E-03	8815	
7	rs4724070	41749884	3.09E-01	LOC285954	17	rs745318	70275393	7.75E-01	SLC9A3R1	-	6.11E-07	3.26E-01	4.15E-03	8816	
1	rs3205087	3790102	1.00E+00	DFFB	14	rs1059264	99838916	2.41E-01	SLC25A29	-	6.11E-07	5.57E-01	3.91E-04	8817	
14	rs3829948	92186579	5.37E-01	RIN3	16	rs247044	56473419	3.60E-01	CNGB1	-	6.11E-07	5.92E-01	1.89E-04	8818	
7	rs4730257	77861452	2.67E-02	MAGI2	12	rs9442	26379772	6.67E-01	ITPR2	-	6.11E-07	9.32E-01	3.74E-04	8819	
5	rs307177	6140348	3.52E-01	N/A	5	rs4958761	1.54E+08	8.67E-01	N/A	-	6.11E-07	N/A	N/A	8820	
11	rs286911	34613797	6.17E-02	EHF	17	rs969979	29795821	3.96E-01	N/A	-	6.11E-07	1.97E-01	2.18E-03	8821	
8	rs2623638	3616003	2.52E-01	CSMD1	9	rs690140	92372895	5.82E-01	N/A	-	6.11E-07	5.91E-01	1.05E-04	8822	
20	rs158534	52149424	3.38E-01	N/A	21	rs2830657	27363903	3.61E-01	N/A	-	6.11E-07	N/A	N/A	8823	
7	rs6944987	8802381	9.72E-01	N/A	17	rs9912563	72491280	4.19E-01	N/A	-	6.11E-07	1.82E-01	2.09E-06	8824	
4	rs12498640	1.26E+08	3.84E-01	N/A	2	rs2200257	1.74E+08	8.54E-01	GALNTL6	-	6.12E-07	1.86E-01	1.92E-03	8825	
3	rs1387015	1.14E+08	3.56E-01	CD200R1L	3	rs7616603	1.21E+08	2.34E-01	CDGAP	-	6.12E-07	9.81E-01	4.18E-05	8826	
2	rs13410950	57506559	3.52E-01	N/A	2	rs6545760	59954716	2.82E-02	N/A	-	6.12E-07	9.97E-03	2.08E-08	8827	
1	rs835004	99857976	7.14E-01	N/A	22	rs2041076	29765945	3.77E-01	N/A	-	6.12E-07	4.84E-02	1.32E-02	8828	
3	rs1482171	73445898	2.76E-01	N/A	5	rs2029401	92916785	7.58E-01	FLJ42709	-	6.12E-07	2.72E-01	1.03E-02	8829	
5	rs353465	1.76E+08	3.89E-03	UIMC1	10	rs2388273	2592415	2.62E-01	N/A	-	6.12E-07	9.43E-01	2.26E-05	8830	
2	rs13407109	2.18E+08	9.06E-02	TNS1	12	rs10861795	78210673	7.80E-01	SYT1	-	6.12E-07	5.41E-01	1.20E-05	8831	
2	rs13407109	2.18E+08	9.06E-02	TNS1	12	rs2272500	78210044	7.80E-01	SYT1	-	6.12E-07	5.41E-01	1.20E-05	8832	
2	rs13424874	2.05E+08	6.57E-01	N/A	3	rs784718	1.31E+08	5.61E-01	TRH	-	6.12E-07	3.08E-01	1.44E-03	8833	
13	rs724706	47020735	7.89E-01	N/A	20	rs552068	47521405	8.01E-01	KCNB1	-	6.12E-07	3.87E-01	4.00E-06	8834	
3	rs26796	10390314	4.54E-01	ATP2B2	12	rs10876844	54336973	7.02E-01	OR10P1	-	6.12E-07	3.92E-01	2.47E-03	8835	
2	rs6758953	45853912	7.25E-01	PRKCE	2	rs16848342	1.64E+08	8.34E-01	N/A	-	6.12E-07	8.41E-01	6.21E-05	8836	
1	rs3013086	13665979	5.25E-01	N/A	1	rs4659374	26651857	2.79E-01	HMGN2	-	6.12E-07	9.41E-01	3.10E-04	8837	
3	rs16864010	1.9E+08	3.13E-01	N/A	12	rs646323	17592322	3.51E-01	N/A	-	6.12E-07	2.61E-01	4.11E-06	8838	
5	rs6898119	1.5E+08	3.39E-01	RBM22	11	rs4758270	7999192	2.37E-01	TUB	-	6.12E-07	6.31E-01	1.68E-03	8839	
1	rs12046389	1.03E+08	9.05E-01	N/A	8	rs2514861	1.08E+08	6.44E-01	ANGPT1	-	6.12E-07	N/A	N/A	8840	
7	rs2158146	88574463	8.40E-01	ZNF804B	19	rs7256177	23101703	5.72E-01	N/A	-	6.12E-07	5.29E-02	1.51E-06	8841	
7	rs2158146	88574463	8.40E-01	ZNF804B	19	rs7257716	23119554	5.72E-01	N/A	-	6.12E-07	5.29E-02	1.51E-06	8842	
8	rs10505007	1.02E+08	4.05E-01	N/A	14	rs1777069	42275132	7.58E-01	N/A	-	6.12E-07	4.77E-01	1.06E-05	8843	
7	rs2214810	26606626	9.64E-01	N/A	12	rs10778270	1.03E+08	1.02E-01	STAB2	-	6.12E-07	7.32E-01	4.00E-04	8844	
4	rs3114381	40228054	3.52E-01	RBM47	14	rs2075493	21701518	7.45E-02	N/A	-	6.12E-07	6.76E-01	1.17E-04	8845	
2	rs2250565	1.5E+08	2.84E-02	LYPD6B	14	rs1275689	68882032	3.17E-01	GALNTL1	-	6.12E-07	9.52E-02	8.77E-07	8846	

SNP A					SNP B						Interaction P			Ranking	Cluster in top 100 interactions
CHR	SNP	Location	gle locus P va	Gene	CHR	SNP	Location	Single locus P valu	Gene	MHC region	Stage 1	Stage 2	Combined		
6	rs7761723	65387111	7.96E-01	EYS	6	rs9403008	1.39E+08	8.72E-02	ECT2L	-	6.12E-07	2.06E-01	2.30E-06	8847	
19	rs8092	3074635	4.94E-01	GN/A15	21	rs715157	34869845	2.08E-02	RCAN1	-	6.12E-07	5.51E-01	4.45E-05	8848	
5	rs2269954	1.38E+08	8.02E-01	BRD8	22	rs137916	48833840	2.20E-01	TTLL8	-	6.12E-07	8.78E-01	4.36E-05	8849	
2	rs2380674	15848838	9.58E-01	N/A	2	rs2195477	51189362	6.45E-01	N/A	-	6.13E-07	3.54E-01	1.51E-05	8850	
5	rs12519172	1.61E+08	6.94E-01	N/A	13	rs9565131	74540692	1.47E-01	N/A	-	6.13E-07	9.37E-01	2.57E-04	8851	
11	rs1790474	1.19E+08	4.13E-01	N/A	15	rs3784313	69974573	6.54E-01	MYO9A	-	6.13E-07	9.70E-01	1.78E-04	8852	
6	rs2395471	31348671	7.52E-01	HLA-C	6	rs6929796	31630648	6.69E-01	NFKBIL1	MHC	6.13E-07	1.95E-01	2.35E-06	8853	
3	rs10510938	65241112	4.14E-01	N/A	11	rs12184451	91406410	4.26E-01	N/A	-	6.13E-07	5.96E-01	1.33E-05	8854	
7	rs6467153	1.27E+08	1.79E-01	SND1	17	rs2453589	19429443	1.12E-01	SLC47A1	-	6.13E-07	4.44E-01	7.86E-04	8855	
10	rs7088704	82231910	8.87E-01	TSPAN14	13	rs9531028	79665833	4.85E-01	N/A	-	6.13E-07	5.40E-01	5.08E-03	8856	
14	rs7149088	26690636	5.71E-01	N/A	18	rs8093432	622651	7.38E-01	C18orf56	-	6.13E-07	6.74E-01	3.01E-05	8857	
14	rs7154679	26691701	5.71E-01	N/A	18	rs8093432	622651	7.38E-01	C18orf56	-	6.13E-07	6.74E-01	3.01E-05	8858	
14	rs7493689	26691037	5.71E-01	N/A	18	rs8093432	622651	7.38E-01	C18orf56	-	6.13E-07	6.74E-01	3.01E-05	8859	
2	rs1370352	2.12E+08	7.68E-01	N/A	22	rs139383	37857855	3.80E-01	CBX7	-	6.14E-07	3.15E-01	1.11E-03	8860	
2	rs4663126	2.35E+08	4.68E-02	N/A	13	rs4517635	23933155	8.30E-01	PARP4	-	6.14E-07	4.91E-01	6.66E-05	8861	
3	rs869557	65708881	3.29E-01	MAGI1	8	rs305306	1.38E+08	4.63E-01	N/A	-	6.14E-07	7.42E-01	6.94E-05	8862	
3	rs2372079	30278778	1.81E-01	N/A	18	rs1791303	55606206	5.86E-01	N/A	-	6.14E-07	N/A	N/A	8863	
1	rs3806340	88921381	6.67E-01	PKN2	16	rs2716563	72727157	3.95E-02	N/A	-	6.14E-07	2.05E-01	4.46E-06	8864	
9	rs1411936	12395940	6.76E-01	N/A	10	rs7916078	67519845	3.31E-02	CTNN/A3	-	6.14E-07	4.66E-01	5.47E-06	8865	
2	rs979976	1.37E+08	6.60E-01	THSD7B	4	rs2583636	83372203	6.35E-01	N/A	-	6.14E-07	5.18E-01	1.92E-04	8866	
9	rs498924	1.1E+08	1.55E-01	N/A	13	rs2755223	39942303	5.21E-01	LOC646982	-	6.14E-07	9.51E-01	1.34E-04	8867	
9	rs10511682	20801421	7.24E-01	KIAA1797	12	rs4765110	1.26E+08	2.97E-01	N/A	-	6.14E-07	8.13E-01	6.35E-05	8868	
2	rs10207099	75374192	7.98E-01	N/A	10	rs953920	1.18E+08	1.93E-01	GFRA1	-	6.14E-07	9.16E-01	2.51E-04	8869	
2	rs2593713	1.44E+08	7.82E-01	N/A	3	's1306978(	1.1E+08	6.12E-02	KIAA1524	-	6.14E-07	1.61E-01	5.98E-06	8870	
1	rs1546204	73981721	9.62E-01	N/A	10	rs7906694	1.2E+08	5.23E-01	CASC2	-	6.14E-07	9.39E-01	6.52E-04	8871	
1	rs2634999	1.63E+08	5.26E-01	N/A	3	rs7618962	1.83E+08	5.30E-01	N/A	-	6.14E-07	8.51E-02	5.87E-07	8872	
4	rs13102102	81292696	8.28E-02	N/A	20	rs6090443	61624230	7.46E-01	PPDPF	-	6.14E-07	5.04E-01	4.02E-04	8873	
9	rs1505641	80384461	5.83E-01	N/A	19	rs1382357	42669449	6.60E-01	ZNF569	-	6.14E-07	6.33E-01	1.76E-04	8874	
13	rs1329292	43164920	6.44E-01	ENOX1	13	rs9595049	43491911	2.85E-01	LOC121838	-	6.14E-07	3.16E-02	2.52E-07	8875	
8	rs3940483	62165144	6.29E-01	N/A	12	's1104577(	21169459	1.32E-01	SLCO1B1	-	6.15E-07	6.44E-01	9.11E-05	8876	
3	rs7615155	1.21E+08	8.53E-01	CD80	18	rs339866	20462599	6.44E-03	N/A	-	6.15E-07	8.09E-01	2.58E-05	8877	
15	rs4485306	91483233	6.19E-01	N/A	18	's1187533	14255834	5.16E-01	N/A	-	6.15E-07	2.55E-01	9.38E-04	8878	
3	rs9848505	1.73E+08	6.80E-01	PLD1	13	rs1230455	45702138	5.90E-01	N/A	-	6.15E-07	4.09E-01	2.95E-06	8879	
3	rs11718502	42504086	3.62E-01	VIPR1	20	's1169791	843325	6.83E-01	ANGPT4	-	6.15E-07	4.25E-01	1.04E-05	8880	
1	rs6583069	1.09E+08	4.81E-01	SLC25A24	2	rs6719434	12780356	4.32E-02	TRIB2	-	6.15E-07	4.54E-01	9.00E-04	8881	
14	rs7154905	66709646	1.54E-02	GPHN	16	's1244928	10265020	5.28E-01	N/A	-	6.15E-07	8.61E-01	1.11E-04	8882	
7	rs4717112	72903353	4.94E-01	WBSCR27	16	rs1094928	64511973	2.29E-01	N/A	-	6.15E-07	1.19E-02	6.04E-08	8883	
3	rs2133600	1.79E+08	9.80E-01	N/A	5	rs7724797	31131278	5.83E-01	N/A	-	6.15E-07	2.13E-01	1.50E-06	8884	
5	rs716866	1.69E+08	1.09E-01	SLIT3	9	rs1663762	74466718	1.36E-01	TMC1	-	6.15E-07	5.48E-01	1.57E-04	8885	
16	rs7192957	59563069	5.24E-01	N/A	17	rs886078	47090559	7.36E-01	CA10	-	6.15E-07	2.55E-01	1.69E-03	8886	
1	rs3000802	2.26E+08	2.15E-01	N/A	2	rs7575156	1.81E+08	3.32E-01	N/A	-	6.15E-07	5.03E-01	1.95E-04	8887	
12	rs11108568	95448949	5.87E-01	N/A	15	rs8036295	51651969	2.81E-01	WDR72	-	6.15E-07	5.60E-01	1.09E-04	8888	
9	rs10858377	1.37E+08	5.00E-01	N/A	18	rs8088785	43806288	1.38E-01	ZBTB7C	-	6.15E-07	N/A	N/A	8889	
2	rs1023632	1.5E+08	2.92E-02	LYPD6B	14	rs1275689	68882032	3.17E-01	GALNTL1	-	6.16E-07	9.48E-02	8.78E-07	8890	
6	rs13201016	1.57E+08	1.55E-01	N/A	19	rs3786657	14367632	5.50E-01	DDX39	-	6.16E-07	5.27E-01	5.97E-06	8891	
4	rs7679904	98410731	4.75E-01	N/A	10	's1159310	1.01E+08	6.50E-02	N/A	-	6.16E-07	3.14E-01	4.98E-06	8892	
1	rs12030578	21921251	5.63E-01	USP48	4	rs4435811	1.18E+08	2.88E-01	N/A	-	6.16E-07	5.92E-01	3.49E-05	8893	
3	rs7651713	1.13E+08	9.45E-01	N/A	7	rs6960287	30823866	1.55E-01	FAM188B	-	6.16E-07	8.72E-01	1.92E-04	8894	
2	rs2347857	19781723	8.76E-01	N/A	4	rs4690032	2693329	2.15E-01	TNIP2	-	6.16E-07	3.45E-01	1.76E-05	8895	
1	rs274587	74716053	5.38E-01	TNNI3K	1	's1049397(	1.02E+08	5.05E-01	OLFM3	-	6.16E-07	8.33E-01	9.25E-05	8896	
1	rs274604	74729913	5.38E-01	TNNI3K	1	's1049397(	1.02E+08	5.05E-01	OLFM3	-	6.16E-07	8.33E-01	9.25E-05	8897	
5	rs108609	56937664	7.86E-01	N/A	14	rs7148001	48189937	8.57E-01	N/A	-	6.16E-07	2.17E-01	7.86E-04	8898	
5	rs108609	56937664	7.86E-01	N/A	14	rs5004293	48191689	8.57E-01	N/A	-	6.16E-07	2.60E-01	6.27E-04	8899	
4	rs4546314	1.19E+08	7.45E-01	N/A	5	rs2114961	1.35E+08	3.60E-01	N/A	-	6.16E-07	N/A	N/A	8900	
3	rs16943	51849719	7.98E-01	IQCF3	9	's1223682	29968410	6.11E-01	N/A	-	6.16E-07	6.07E-01	1.65E-05	8901	
1	rs631272	12129342	4.26E-01	TNFRSF8	14	rs8007372	87698870	7.32E-01	KCNK10	-	6.16E-07	8.74E-01	1.45E-04	8902	
4	rs921853	34661236	9.09E-01	N/A	11	rs3740958	36429376	2.52E-02	PRR5L	-	6.16E-07	8.55E-01	7.65E-05	8903	
16	rs7500355	84634601	7.85E-01	N/A	17	rs4889744	22048100	9.66E-01	N/A	-	6.16E-07	2.76E-01	1.13E-03	8904	
4	rs12642398	89542237	4.46E-01	HERC6	10	rs3121480	1.15E+08	5.66E-02	NRAP	-	6.16E-07	9.58E-01	6.04E-05	8905	
4	rs6837103	52410697	9.66E-01	DCUN1D4	5	rs216148	1.49E+08	2.51E-01	HMGXB3	-	6.17E-07	5.75E-01	5.95E-04	8906	
3	rs3009500	1.33E+08	2.42E-01	N/A	10	rs1953734	1.13E+08	4.20E-02	N/A	-	6.17E-07	8.45E-01	1.30E-04	8907	
5	rs6555665	1.65E+08	8.43E-01	N/A	22	rs2587113	16708503	1.18E-01	MICAL3	-	6.17E-07	N/A	N/A	8908	
3	rs9839376	1.8E+08	1.76E-01	KCNMB2	5	's1336250	56014886	9.88E-01	N/A	-	6.17E-07	4.48E-01	3.26E-05	8909	
5	rs4835860	1.22E+08	2.56E-01	N/A	12	rs1158818	66463164	5.69E-01	N/A	-	6.17E-07	5.05E-01	2.56E-04	8910	
1	rs1146394	85719788	4.53E-01	DDAH1	18	rs555107	7455949	2.61E-01	N/A	-	6.17E-07	8.21E-01	3.15E-04	8911	
11	rs1894213	74837056	6.08E-02	GDPD5	16	rs7191962	79763243	7.80E-01	PKD1L2	-	6.17E-07	8.71E-01	5.01E-04	8912	
11	rs482458	74836097	6.08E-02	GDPD5	16	rs7191962	79763243	7.80E-01	PKD1L2	-	6.17E-07	8.71E-01	5.01E-04	8913	
11	rs482503	74836081	6.08E-02	GDPD5	16	rs7191962	79763243	7.80E-01	PKD1L2	-	6.17E-07	8.71E-01	5.01E-04	8914	
14	rs7160556	27866669	3.39E-01	N/A	14	's1712873	92541968	6.03E-02	ITPK1	-	6.17E-07	2.44E-01	2.19E-03	8915	
6	rs488367	1.25E+08	8.36E-01	NKAIN2	16	's1292729	5186015	1.66E-01	N/A	-	6.17E-07	5.29E-01	1.62E-05	8916	
6	rs6935051	1.5E+08	5.88E-01	RAET1L	3	rs9290426	1.73E+08	7.40E-01	PLD1	-	6.17E-07	7.14E-01	5.76E-05	8917	
6	rs942492	7335966	1.16E-01	N/A	2	's1269162	1.42E+08	3.61E-01	LRP1B	-	6.17E-07	8.59E-01	8.02E-05	8918	
1	rs4845394	1.53E+08	9.78E-01	KCNN3	3	's1332442(	1.92E+08	1.82E-01	N/A	-	6.17E-07	8.59E-01	1.92E-04	8919	
5	rs2010010	1.54E+08	4.14E-01	GALNT10	17	rs8081781	14385239	5.30E-01	N/A	-	6.17E-07	5.22E-01	5.54E-05	8920	
4	rs1519097	9411951	2.28E-01	DRD5	5	rs4140810	1.61E+08	6.34E-01	GABRG2	-	6.17E-07	8.42E-01	7.38E-05	8921	
3	rs11918522	1.14E+08	5.37E-01	CD200R1L	7	rs9639018	48084209	9.73E-02	C7orf57	-	6.17E-07	4.86E-01	1.14E-05	8922	
1	rs4660652	39230446	3.01E-02	AKIRIN1	13	rs9543238	72618119	7.27E-01	N/A	-	6.17E-07	9.53E-01	9.39E-05	8923	
5	rs4868133	1.71E+08	2.36E-01	FBXW11	18	's1108051	11375531	6.02E-01	N/A	-	6.17E-07	5.00E-01	2.68E-05	8924	
1	rs708753	2.25E+08	3.28E-01	C1orf95	14	rs1077989	67045575	9.86E-01	TMEM229B	-	6.17E-07	5.69E-01	3.36E-04	8925	
10	rs1415880	8417258	6.83E-01	N/A	16	rs1713468	12680426	3.05E-01	CPPED1	-	6.18E-07	2.09E-01	5.46E-06	8926	
4	rs4975057	80707369	7.87E-02	N/A	7	rs16480	24277594	2.38E-01	NPY	-	6.18E-07	3.08E-01	1.62E-05	8927	
8	rs10503236	3990061	8.99E-01	CSMD1	16	rs896398	8053854	5.70E-01	N/A	-	6.18E-07	8.16E-01	5.24E-05	8928	
6	rs2179367	1.5E+08	6.33E-01	ZC3H12D	8	rs1380994	1.01E+08	3.92E-01	RGS22	-	6.18E-07	3.34E-01	4.85E-04	8929	
4	rs11100846	1.45E+08	3.92E-01	N/A	5	rs9292871	43340159	9.92E-01	HMGCS1	-	6.18E-07	3.99E-01	2.14E-06	8930	
1	rs6426156	2.23E+08	7.64E-01	CNIH3	19	rs4807928	1399180	9.68E-01	DAZAP1	-	6.18E-07	3.19E-01	4.29E-06	8931	
6	rs4709755	1.64E+08	4.16E-01	N/A	1	rs2186104	2.32E+08	2.53E-01	SLC35F3	-	6.18E-07	3.97E-01	2.41E-05	8932	
6	rs2756318	836210	2.56E-02	N/A	14	rs4902118	61775417	4.45E-01	N/A	-	6.18E-07	N/A	N/A	8933	
4	rs12507136	1.18E+08	2.41E-01	N/A	11	rs2433435	86131348	1.10E-01	N/A	-	6.18E-07	8.01E-01	7.81E-05	8934	
14	rs11627134	74168449	6.49E-01	LTBP2	22	rs2240432	29851404	5.25E-01	INPP5J	-	6.18E-07	2.05E-01	3.99E-06	8935	
10	rs2505971	27165547	6.04E-01	N/A	16	rs7197983	52461190	9.04E-02	FTO	-	6.18E-07	3.37E-01	4.22E-04	8936	
3	rs17714443	55836171	2.13E-01	ERC2	19	rs1860565	62026834	7.53E-01	PEG3	-	6.18E-07	4.30E-01	8.37E-05	8937	
5	rs1205720	9428383	4.08E-01	SEMA5A	18	's1051398	65703318	9.47E-02	CD226	-	6.18E-07	8.66E-01	1.68E-05	8938	
7	rs6976604	29580803	4.48E-01	PRR15	19	rs1781873	21269271	1.11E-01	ZNF708	-	6.18E-07	N/A	N/A	8939	
3	rs16854451	1.1E+08	8.89E-01	HHLA2	10	rs869963	87285190	3.38E-01	N/A	-	6.19E-07	1.35E-01	3.96E-03	8940	
3	rs12634962	1.1E+08	8.89E-01	HHLA2	10	rs869963	87285190	3.38E-01	N/A	-	6.19E-07	1.57E-01	3.27E-03	8941	
3	rs1489919	1.61E+08	6.50E-01	SCHIP1	20	rs6134049	10771506	7.23E-01	N/A	-	6.19E-07	4.86E-01	1.07E-05	8942	
8	rs1443930	1.09E+08	8.89E-01	RSPO2	9	's1179324	81968977	4.73E-01	N/A	-	6.19E-07	7.17E-01	1.48E-04	8943	
7	rs1476106	78482013	9.21E-01	MAGI2	8	rs6985478	25836069	6.09E-01	EBF2	-	6.19E-07	8.41E-01	1.34E-04	8944	
1	rs1176529	1.55E+08	2.15E-01	CYCSP52	18	rs9966360	3988885	7.68E-01	N/A	-	6.19E-07	N/A	N/A	8945	
6	rs4896469	1.4E+08	4.77E-01	N/A	3	rs7649938	6190542	7.52E-01	N/A	-	6.19E-07	8.29E-01	4.57E-05	8946	
10	rs7097370	19049704	2.15E-01	N/A	12	rs39635	28017512	9.44E-02	PTHLH	-	6.19E-07	2.74E-01	2.80E-05	8947	
3	rs9851637	1.86E+08	7.18E-03	N/A	10	rs7067780	44672425	2.24E-01	N/A	-	6.19E-07	6.20E-01	1.39E-03	8948	
4	rs1567474	1.79E+08	7.79E-01	N/A	20	's1190822	823368	8.53E-01	ANGPT4	-	6.19E-07	7.17E-01	4.70E-04	8949	
4	rs1567475	1.79E+08	7.79E-01	N/A	20	's1190822	823368	8.53E-01	ANGPT4	-	6.19E-07	7.17E-01	4.70E-04	8950	
6	rs2327478	1.35E+08	2.47E-01	N/A	1	rs6672870	85664045	3.09E-01	DDAH1	-	6.19E-07	9.42E-01	3.55E-05	8951	
2	rs11903252	37146394	5.36E-01	HEATR5B	5	rs4045344	648870	8.28E-01	CEP72	-	6.19E-07	7.68E-02	2.15E-03	8952	
3	rs12637098	1.96E+08	6.26E-01	N/A	7	rs1568824	55448883	9.41E-01	LANCL2	-	6.19E-07	5.08E-03	1.82E-02	8953	
2	rs10179068	2.16E+08	4.66E-01	N/A	3	rs6797616	81313323	7.54E-02	N/A	-	6.19E-07	3.46E-02	2.65E-07	8954	
9	rs2150199	1.19E+08	5.00E-01	ASTN2	13	's1161977	44123531	9.28E-01	N/A	-	6.19E-07	9.66E-01	8.63E-05	8955	
5	rs1288205	1.62E+08	1.66E-01	N/A	5	rs7713731	1.78E+08	2.06E-01	N/A	-	6.19E-07	2.70E-01	8.87E-04	8956	
4	rs1545763	1.17E+08	1.99E-01	N/A	18	rs1033060	46680315	2.76E-01	ME2	-	6.20E-07	6.75E-01	5.58E-05	8957	
5	rs1152168	1.03E+08	7.35E-02	N/A	12	rs2046383	29995409	2.63E-01	N/A	-	6.20E-07	3.02E-01	6.07E-06	8958	

SNP A					SNP B						Interaction P			Ranking	Cluster in top 100 interactions
CHR	SNP	Location	gle locus P va	Gene	CHR	SNP	Location	Single locus P valu	Gene	HC region	Stage 1	Stage 2	Combined		
10	rs10903731	2318519	2.42E-01	N/A	14	rs4906459	1.04E+08	8.88E-01	N/A	-	6.20E-07	3.58E-01	1.47E-05	8959	
2	rs17734815	30175483	8.49E-01	N/A	9	rs1555422	17611965	4.27E-01	SH3GL2	-	6.20E-07	4.42E-01	7.34E-06	8960	
1	rs7545391	61850935	6.14E-01	N/A	10	rs3740415	1.04E+08	7.13E-01	TMEM180	-	6.20E-07	6.91E-01	4.94E-04	8961	
1	rs7545391	61850935	6.14E-01	N/A	10	rs7342070	1.04E+08	7.13E-01	C10orf95	-	6.20E-07	8.56E-01	3.30E-04	8962	
4	rs2611021	1.81E+08	5.29E-02	N/A	16	rs1566045	49579304	4.21E-01	N/A	-	6.20E-07	1.07E-01	9.26E-03	8963	
6	rs2328779	23568885	6.45E-01	N/A	6	rs12202603	36351472	7.94E-01	PNPLA1	MHC	6.20E-07	8.33E-01	3.90E-05	8964	
10	rs17110864	89364066	8.95E-01	N/A	16	rs11648776	82122361	5.90E-02	CDH13	-	6.20E-07	7.28E-01	4.81E-05	8965	
3	rs7610039	24332086	4.49E-01	THRB	11	rs10488764	1.1E+08	3.21E-01	FDX1	-	6.20E-07	1.40E-01	5.90E-03	8966	
2	rs601400	1.66E+08	5.54E-01	N/A	10	rs2490846	37140774	7.05E-02	N/A	-	6.20E-07	3.87E-01	6.76E-06	8967	
6	rs3818408	10496639	7.25E-01	TFAP2A	2	rs1865373	2.32E+08	4.54E-02	C2orf57	-	6.20E-07	4.34E-01	1.75E-05	8968	
8	rs10088527	1.13E+08	1.46E-01	N/A	20	rs16995121	8632506	5.45E-01	PLCB1	-	6.20E-07	5.64E-01	1.63E-05	8969	
11	rs10750352	1.26E+08	6.61E-01	KIRREL3	12	rs2284422	13855111	3.90E-02	GRIN2B	-	6.20E-07	6.06E-01	9.35E-04	8970	
6	rs9461680	31351326	4.16E-01	HLA-C	13	rs4479095	98070051	6.42E-01	N/A	MHC	6.20E-07	6.41E-01	1.15E-04	8971	
4	rs4864014	1.32E+08	1.40E-01	N/A	11	rs1349784	99311421	2.15E-01	CNTN5	-	6.21E-07	8.06E-01	3.38E-05	8972	
4	rs1048447	68166067	3.20E-01	UBA6	12	rs4768582	43675084	4.51E-01	DBX2	-	6.21E-07	9.51E-01	8.13E-05	8973	
4	rs1843593	68281593	3.20E-01	LOC550112	12	rs4768582	43675084	4.51E-01	DBX2	-	6.21E-07	9.51E-01	8.13E-05	8974	
1	rs4927071	54452500	3.24E-01	SSBP3	18	rs12607576	13236168	9.33E-01	C18orf1	-	6.21E-07	6.14E-01	3.34E-05	8975	
6	rs7752992	20533437	9.32E-01	E2F3	2	rs1446738	1.34E+08	4.91E-01	N/A	-	6.21E-07	9.74E-01	6.30E-05	8976	
13	rs9603180	36895401	8.29E-01	N/A	17	rs1388164	29068522	6.13E-01	ACCN1	-	6.21E-07	8.84E-01	5.67E-04	8977	
1	rs11118465	2.18E+08	1.42E-01	SLC30A10	10	rs2893959	66053372	8.59E-01	N/A	-	6.21E-07	7.77E-01	1.22E-04	8978	
4	rs10001661	89468396	5.50E-01	N/A	20	rs2424253	19921873	4.76E-02	RIN2	-	6.21E-07	3.61E-01	8.60E-04	8979	
13	rs9742122	84196912	2.70E-01	N/A	16	rs16942541	85617984	2.75E-01	N/A	-	6.21E-07	1.11E-01	3.02E-03	8980	
4	rs2197670	5270240	7.69E-01	STK32B	4	rs4695338	47843579	9.82E-01	TEC	-	6.21E-07	3.64E-01	7.40E-04	8981	
6	rs4280956	23064231	5.80E-02	N/A	12	rs10848287	1.3E+08	9.21E-02	GPR133	-	6.21E-07	5.02E-01	5.00E-03	8982	
3	rs13067383	61332457	7.04E-01	N/A	17	rs11656549	69040691	4.86E-01	SDK2	-	6.21E-07	6.89E-01	2.39E-04	8983	
1	rs2693958	7463878	5.27E-01	CAMTA1	4	rs1518000	18398373	6.78E-01	N/A	-	6.21E-07	8.07E-01	5.94E-05	8984	
1	rs1193188	7462117	5.27E-01	CAMTA1	4	rs1518000	18398373	6.78E-01	N/A	-	6.21E-07	8.41E-01	6.63E-05	8985	
6	rs6921041	1.36E+08	9.29E-01	PDE7B	14	rs1028535	58569128	1.01E-01	N/A	-	6.22E-07	2.03E-01	3.68E-03	8986	
6	rs6921041	1.36E+08	9.29E-01	PDE7B	14	rs2064770	58570725	1.01E-01	N/A	-	6.22E-07	2.03E-01	3.68E-03	8987	
19	rs1005204	50342528	2.90E-01	NKPD1	20	rs572715	53672362	8.41E-01	N/A	-	6.22E-07	5.25E-01	3.26E-04	8988	
3	rs12495053	24375132	9.29E-01	THRB	14	rs1241771	91760538	9.11E-01	N/A	-	6.22E-07	6.43E-01	4.72E-05	8989	
8	rs12679346	34188001	4.96E-01	N/A	16	rs3094773	2755238	4.17E-01	SRRM2	-	6.22E-07	6.58E-01	1.56E-04	8990	
3	rs12495053	24375132	9.29E-01	THRB	14	rs1241768	91757980	9.11E-01	N/A	-	6.22E-07	7.77E-01	7.44E-05	8991	
2	rs7594227	96969475	4.59E-01	FAM178B	4	rs4693763	87450873	3.44E-01	MAPK10	-	6.22E-07	3.62E-01	3.55E-05	8992	
2	rs7594227	96969475	4.59E-01	FAM178B	4	rs4299551	87454804	3.44E-01	MAPK10	-	6.22E-07	3.76E-01	3.79E-05	8993	
8	rs7824600	1.22E+08	7.20E-01	MTBP	22	rs5768016	46595486	9.66E-01	N/A	-	6.22E-07	6.41E-01	2.67E-05	8994	
6	rs12660854	95455862	4.94E-01	N/A	1	rs16848614	2.27E+08	6.52E-01	HIST3H2A	-	6.22E-07	3.77E-01	4.27E-06	8995	
11	rs421694	26567094	5.60E-01	ANO3	22	rs1372070	20724749	8.98E-01	N/A	-	6.22E-07	8.84E-01	1.27E-04	8996	
14	rs1998192	28514419	4.38E-02	N/A	18	rs1947494	63190117	7.67E-01	N/A	-	6.22E-07	9.19E-01	5.88E-05	8997	
1	rs2151124	62192891	8.25E-01	IN/ADL	3	rs7628487	1.3E+08	9.73E-01	N/A	-	6.22E-07	5.81E-01	5.64E-05	8998	
9	rs10739386	1.15E+08	5.40E-01	ZFP37	13	rs1323553	48160257	4.86E-01	CYSLTR2	-	6.22E-07	8.61E-01	9.82E-05	8999	
2	rs12466600	52528250	3.80E-03	N/A	10	rs997225	67952976	8.78E-01	CTNN/A3	-	6.22E-07	6.42E-02	1.15E-06	9000	
10	rs10508570	19228576	7.14E-01	N/A	12	rs762718	11835595	1.75E-01	ETV6	-	6.22E-07	3.38E-01	1.34E-03	9001	
4	rs2085600	89998932	5.46E-02	FAM13A	5	rs6863774	55730873	1.10E-01	N/A	-	6.22E-07	N/A	N/A	9002	
6	rs13217925	70642371	7.42E-01	COL19A1	15	rs1317722	88382020	2.18E-01	ZNF710	-	6.22E-07	2.70E-01	9.03E-04	9003	
6	rs10498870	70643053	7.42E-01	COL19A1	15	rs1317722	88382020	2.18E-01	ZNF710	-	6.22E-07	6.00E-01	4.93E-04	9004	
6	rs647108	1.38E+08	4.75E-01	N/A	12	rs10772757	14295548	4.15E-02	N/A	-	6.22E-07	4.96E-01	1.04E-05	9005	
6	rs911566	4421201	3.95E-02	N/A	16	rs11648589	23203475	4.34E-01	SCNN1B	-	6.22E-07	5.12E-01	2.45E-04	9006	
5	rs6876694	4442590	4.59E-01	N/A	12	rs10850931	1.17E+08	7.84E-01	KSR2	-	6.22E-07	4.66E-01	3.11E-05	9007	
6	rs2503661	92817083	2.15E-01	N/A	19	rs4803630	48642923	3.09E-01	LYPD3	-	6.22E-07	5.94E-01	2.61E-04	9008	
5	rs17154889	1.02E+08	9.95E-01	PAM	10	rs2419320	1.12E+08	2.95E-01	N/A	-	6.23E-07	2.99E-01	4.53E-06	9009	
2	rs6733165	1.7E+08	5.66E-01	C2orf77	7	rs2158546	49596217	9.09E-01	N/A	-	6.23E-07	4.46E-01	5.43E-06	9010	
4	rs1918179	1.23E+08	3.16E-01	N/A	15	rs12910396	83229107	7.07E-01	SLC28A1	-	6.23E-07	5.74E-01	1.25E-04	9011	
10	rs10904442	5252361	3.43E-01	AKR1C4	16	rs11648508	56621014	5.32E-01	MMP15	-	6.23E-07	7.81E-01	9.32E-05	9012	
2	rs11688109	29987967	8.36E-01	ALK	11	rs4300383	42610668	8.20E-01	N/A	-	6.23E-07	5.02E-01	1.57E-05	9013	
6	rs1057985	44293879	6.85E-01	SLC29A1	8	rs4841386	10481589	6.08E-01	RP1L1	-	6.23E-07	9.58E-02	3.94E-06	9014	
2	rs6433123	1.52E+08	9.93E-01	N/A	10	rs11595073	1.24E+08	4.90E-01	TACC2	-	6.23E-07	8.84E-02	5.14E-03	9015	
7	rs17171480	35552194	6.06E-01	N/A	8	rs10958045	82885549	3.66E-01	SNX16	-	6.23E-07	6.78E-01	6.86E-05	9016	
3	rs7653315	1.33E+08	4.04E-01	N/A	15	rs7183261	92129998	3.06E-01	N/A	-	6.23E-07	2.67E-01	4.93E-06	9017	
3	rs9857771	1.03E+08	1.69E-01	LOC152225	15	rs2116207	84729595	4.73E-01	AGBL1	-	6.23E-07	2.90E-01	7.41E-04	9018	
4	rs7659432	1.82E+08	7.97E-01	N/A	19	rs7259371	38226481	8.16E-01	RHPN2	-	6.23E-07	8.03E-01	1.03E-04	9019	
1	rs10911412	1.82E+08	9.70E-01	RGL1	16	rs12933084	82918034	1.52E-01	WFDC1	-	6.23E-07	5.79E-01	7.04E-05	9020	
6	rs6929774	33670698	3.67E-01	C6orf227	9	rs296639	92880480	2.69E-01	N/A	MHC	6.23E-07	7.39E-01	6.83E-04	9021	
2	rs6719991	2.41E+08	8.27E-01	AGXT	8	rs7835759	19226201	3.02E-01	N/A	-	6.23E-07	5.68E-01	2.91E-05	9022	
14	rs1279329	32645873	3.69E-01	NPAS3	15	rs1346037	59925203	7.83E-01	VPS13C	-	6.24E-07	2.29E-01	1.18E-03	9023	
1	rs12089317	22625714	4.12E-01	N/A	11	rs12787445	1.08E+08	3.47E-01	ACAT1	-	6.24E-07	7.90E-01	1.07E-04	9024	
6	rs2064501	1.38E+08	5.73E-02	IL22RA2	7	rs4404863	1.36E+08	1.50E-01	N/A	-	6.24E-07	1.90E-01	8.53E-06	9025	
1	rs766499	2.13E+08	4.42E-01	SMYD2	13	rs12584299	1.08E+08	7.00E-02	N/A	-	6.24E-07	8.02E-01	1.66E-05	9026	
2	rs780049	1.25E+08	1.26E-01	CNTN/AP5	10	rs10903382	1189313	6.90E-01	NCRN/A00200	-	6.24E-07	9.37E-01	2.38E-04	9027	
8	rs12155569	1.22E+08	7.17E-01	MTBP	22	rs5768016	46595486	9.66E-01	N/A	-	6.24E-07	6.08E-01	2.40E-05	9028	
6	rs2297846	1.45E+08	8.19E-01	UTRN	10	rs12415753	31456921	7.29E-01	N/A	-	6.24E-07	7.14E-01	7.03E-05	9029	
8	rs2882460	62688450	3.00E-01	ASPH	18	rs10853232	13373207	2.24E-01	C18orf1	-	6.24E-07	8.18E-01	4.38E-05	9030	
2	rs12714386	3116122	9.11E-01	N/A	3	rs9850126	7962521	1.42E-01	N/A	-	6.24E-07	N/A	N/A	9031	
7	rs7799285	1.01E+08	8.62E-01	RABL5	18	rs328196	42469804	8.73E-01	LOXHD1	-	6.24E-07	7.03E-01	3.70E-04	9032	
9	rs619589	29533979	1.46E-01	N/A	9	rs1858555	1.07E+08	1.99E-01	NIPSN/AP3B	-	6.24E-07	9.52E-01	9.09E-05	9033	
13	rs9549507	1.12E+08	1.13E-01	N/A	14	rs2332635	25603564	2.97E-01	N/A	-	6.24E-07	9.29E-01	5.14E-05	9034	
10	rs1992293	80261371	7.35E-01	N/A	18	rs4800293	24067275	5.19E-01	N/A	-	6.25E-07	4.46E-02	1.43E-02	9035	
14	rs17098186	98546126	7.09E-02	N/A	17	rs8064352	72407940	7.21E-01	MGAT5B	-	6.25E-07	9.92E-01	7.96E-05	9036	
14	rs17098186	98546126	7.09E-02	N/A	17	rs8065442	72407954	7.21E-01	MGAT5B	-	6.25E-07	9.92E-01	7.96E-05	9037	
3	rs6793774	1.55E+08	7.89E-01	N/A	17	rs7502835	71010218	2.23E-01	CASKIN2	-	6.25E-07	5.34E-01	9.68E-06	9038	
2	rs13417226	8249928	5.08E-01	N/A	7	rs4729228	95419185	1.02E-01	DYNC1I1	-	6.25E-07	3.97E-01	1.46E-05	9039	
4	rs907362	1.85E+08	7.44E-01	N/A	15	rs4243082	76469131	7.52E-01	N/A	-	6.25E-07	9.01E-01	7.62E-05	9040	
2	rs874060	2.41E+08	3.07E-01	NDUFA10	16	rs12598271	81163012	3.75E-01	N/A	-	6.25E-07	9.60E-01	2.81E-04	9041	
12	rs12812221	55605603	5.43E-01	SDR9C7	15	rs7161838	25472401	3.84E-02	N/A	-	6.25E-07	N/A	N/A	9042	
6	rs9368675	31380136	7.91E-01	N/A	6	rs2523608	31430538	3.16E-01	HLA-B	MHC	6.25E-07	1.38E-01	N/A	9043	
2	rs4669614	10792122	5.50E-02	ATP6V1C2	3	rs1077524	13905134	3.84E-01	WNT7A	-	6.25E-07	3.27E-01	4.18E-05	9044	
2	rs4669614	10792122	5.50E-02	ATP6V1C2	3	rs6768050	13905491	3.84E-01	WNT7A	-	6.25E-07	3.49E-01	3.78E-05	9045	
4	rs4689408	6393114	9.67E-01	PPP2R2C	10	rs364974	17386272	9.25E-01	ST8SIA6	-	6.25E-07	6.93E-01	3.05E-05	9046	
2	rs10933620	2.41E+08	4.61E-01	N/A	8	rs7845852	4144545	2.92E-01	CSMD1	-	6.25E-07	1.54E-01	9.64E-04	9047	
1	rs767707	1.66E+08	9.63E-01	N/A	18	rs12964156	24831978	4.76E-01	N/A	-	6.25E-07	1.72E-01	1.69E-06	9048	
3	rs4234265	33822939	5.20E-01	PDCD6IP	13	rs2038712	21757616	3.79E-02	N/A	-	6.25E-07	2.60E-01	3.30E-04	9049	
1	rs2673962	2.33E+08	9.95E-01	N/A	3	rs9829982	88568692	8.64E-01	N/A	-	6.25E-07	5.04E-01	6.26E-04	9050	
4	rs445275	3712796	5.56E-01	N/A	5	rs6881045	1.24E+08	9.79E-01	N/A	-	6.25E-07	2.28E-01	1.67E-06	9051	
1	rs1325432	92713438	2.83E-01	GFI1	2	rs4850410	1.98E+08	4.05E-01	N/A	-	6.25E-07	5.14E-01	1.08E-05	9052	
6	rs1569579	24621615	3.83E-03	ALDH5A1	7	rs987252	51660260	8.05E-01	N/A	-	6.26E-07	5.35E-01	2.30E-05	9053	
7	rs4721181	13066067	1.03E-01	N/A	19	rs3746228	62496174	5.82E-01	ZNF460	-	6.26E-07	6.36E-01	1.88E-05	9054	
1	rs2272784	1.73E+08	4.95E-01	KIAA0040	8	rs7008943	28395310	8.96E-01	FBXO16	-	6.26E-07	4.10E-01	4.05E-04	9055	
1	rs3753555	1.73E+08	4.95E-01	KIAA0040	8	rs7008943	28395310	8.96E-01	FBXO16	-	6.26E-07	4.15E-01	4.00E-04	9056	
17	rs2259992	895040	8.79E-01	ABR	17	rs12942260	9708106	6.96E-01	GLP2R	-	6.26E-07	4.51E-01	1.60E-05	9057	
8	rs2407747	4931242	4.21E-01	N/A	15	rs11638799	48203888	2.11E-02	ATP8B4	-	6.26E-07	7.73E-01	4.85E-04	9058	
14	rs11159347	25017276	8.68E-01	N/A	15	rs3931347	68490126	5.60E-01	N/A	-	6.26E-07	7.28E-01	6.71E-04	9059	
2	rs6757623	16424588	1.04E-01	N/A	7	rs2920486	1.35E+08	9.37E-01	N/A	-	6.26E-07	3.48E-01	3.31E-03	9060	
2	rs7578883	78928227	9.72E-02	N/A	9	rs1156155	1.19E+08	8.96E-01	ASTN2	-	6.26E-07	4.37E-01	6.31E-04	9061	
2	rs16827195	1.48E+08	4.23E-01	N/A	11	rs10892384	1.19E+08	1.43E-01	N/A	-	6.26E-07	7.74E-01	8.31E-05	9062	
1	rs2076571	54590687	8.72E-01	SSBP3	12	rs1527047	76297680	9.83E-01	N/A	-	6.26E-07	3.28E-01	1.07E-03	9063	
8	rs4279641	1.21E+08	6.59E-01	COL14A1	22	rs16985531	26491703	8.13E-01	MN1	-	6.26E-07	5.62E-01	2.89E-04	9064	
1	rs819639	2.35E+08	1.08E-01	ACTN2	10	rs1892545	61982455	6.47E-01	N/A	-	6.26E-07	8.91E-01	7.18E-05	9065	
9	rs7032844	1E+08	5.23E-02	GABBR2	11	rs1007369	1.19E+08	9.34E-01	N/A	-	6.26E-07	9.66E-02	4.96E-03	9066	
8	rs12549316	33384253	2.83E-01	FUT10	14	rs11847817	38089551	5.85E-01	N/A	-	6.26E-07	3.84E-01	1.52E-05	9067	
16	rs10852363	13048123	2.67E-02	SHISA9	17	rs12452913	66615939	1.45E-01	N/A	-	6.27E-07	5.68E-01	9.71E-05	9068	
5	rs2930059	10806399	6.79E-01	DAP	12	rs658719	3944655	7.20E-01	N/A	-	6.27E-07	1.59E-01	1.65E-06	9069	
7	rs13225365	1.47E+08	8.39E-01	CNTN/AP2	8	rs2702971	1.34E+08	8.61E-01	SLA	-	6.27E-07	2.27E-02	1.38E-02	9070	

SNP A					SNP B						Interaction P			Ranking	Cluster in top 100 interactions
CHR	SNP	Location	gle locus P va	Gene	CHR	SNP	Location	Single locus P valu	Gene	MHC region	Stage 1	Stage 2	Combined		
1	rs803675	44125824	1.95E-01	ST3GAL3	18	rs1720464	1003959	9.85E-01	N/A	-	6.27E-07	8.43E-01	1.89E-05	9071	
1	rs803675	44125824	1.95E-01	ST3GAL3	18	rs789040	1007587	9.85E-01	N/A	-	6.27E-07	8.43E-01	1.89E-05	9072	
1	rs803675	44125824	1.95E-01	ST3GAL3	18	rs789071	998339	9.85E-01	N/A	-	6.27E-07	9.61E-01	2.42E-05	9073	
1	rs803675	44125824	1.95E-01	ST3GAL3	18	rs789072	998417	9.85E-01	N/A	-	6.27E-07	9.61E-01	2.42E-05	9074	
12	rs12230272	1.24E+08	8.24E-01	N/A	13	rs9542786	71412634	3.39E-02	N/A	-	6.27E-07	2.78E-01	3.30E-06	9075	
2	rs968763	7946176	3.61E-01	N/A	14	rs4142176	76109537	4.29E-01	N/A	-	6.27E-07	7.08E-01	1.44E-04	9076	
1	rs1122396	1.99E+08	2.50E-01	TMEM9	5	rs10065187	1.74E+08	2.82E-01	N/A	-	6.27E-07	8.17E-01	2.46E-05	9077	
6	rs9358619	9390197	3.16E-01	N/A	9	rs2297879	35652251	3.82E-01	SIT1	-	6.27E-07	9.06E-01	1.67E-04	9078	
1	rs1538686	1.95E+08	9.57E-01	KCNT2	10	rs4750552	14771455	7.55E-01	FAM107B	-	6.27E-07	9.39E-01	1.07E-04	9079	
6	rs12212773	1.55E+08	1.06E-01	IPCEF1	10	rs7341996	4111705	7.54E-01	N/A	-	6.27E-07	5.11E-01	3.88E-04	9080	
8	rs17418933	4598479	5.45E-01	CSMD1	8	rs10504149	53972009	9.58E-01	N/A	-	6.27E-07	8.40E-01	6.40E-05	9081	
1	rs10864504	11398790	1.07E-01	N/A	15	rs4965726	99202209	4.48E-01	N/A	-	6.27E-07	3.70E-01	8.91E-06	9082	
1	rs12049593	2.32E+08	3.22E-01	SLC35F3	4	rs1397453	67184018	5.92E-01	N/A	-	6.27E-07	6.53E-01	3.95E-04	9083	
3	rs7633179	56907748	7.99E-01	ARHGEF3	3	rs11914998	1.49E+08	8.61E-01	N/A	-	6.27E-07	N/A	N/A	9084	
7	rs2392362	35367972	5.04E-02	N/A	8	rs11779508	1.43E+08	7.59E-01	FLJ43860	-	6.28E-07	4.83E-01	8.16E-04	9085	
6	rs9487771	1.12E+08	8.81E-01	N/A	18	rs636173	11986337	2.75E-01	IMPA2	-	6.28E-07	6.71E-01	1.59E-04	9086	
9	rs10812053	24472743	7.55E-01	N/A	15	rs1077015	99718796	5.21E-01	PCSK6	-	6.28E-07	9.60E-01	4.88E-05	9087	
11	rs10840332	9937077	9.64E-01	SBF2	21	rs2824920	18881236	9.16E-01	N/A	-	6.28E-07	6.17E-01	N/A	9088	
10	rs6480474	72441686	4.22E-01	N/A	20	rs1201889	58690346	9.74E-01	N/A	-	6.28E-07	6.53E-01	4.01E-05	9089	
11	rs516450	1.02E+08	9.54E-02	MMP10	20	rs6056801	968318	1.37E-01	N/A	-	6.28E-07	N/A	N/A	9090	
14	rs4901494	53738263	9.60E-01	N/A	16	rs11150556	81828042	1.99E-01	CDH13	-	6.28E-07	N/A	N/A	9091	
2	rs6729109	1.05E+08	5.15E-02	N/A	11	rs5028798	34562011	1.26E-01	N/A	-	6.28E-07	N/A	N/A	9092	
2	rs919868	1.74E+08	5.53E-01	N/A	2	rs10209084	2.4E+08	4.29E-01	HDAC4	-	6.28E-07	2.74E-01	9.42E-06	9093	
4	rs6448494	10708616	7.44E-01	N/A	13	rs1359066	48550422	5.19E-01	FNDC3A	-	6.28E-07	4.79E-01	6.20E-04	9094	
2	rs6709502	30864428	9.17E-01	CAPN13	11	rs563272	1.16E+08	7.58E-01	N/A	-	6.28E-07	2.38E-01	1.69E-06	9095	
2	rs4669167	5310781	6.00E-01	N/A	9	rs3739795	1.02E+08	3.34E-01	ERP44	-	6.28E-07	4.03E-01	6.35E-04	9096	
3	rs9422	1.25E+08	8.04E-01	MYLK	12	rs1635135	1.12E+08	2.15E-01	OAS2	-	6.28E-07	8.60E-01	3.05E-05	9097	
10	rs7909341	1.34E+08	9.28E-01	N/A	22	rs16992538	43363969	4.92E-01	NCRN/A00207	-	6.28E-07	6.06E-01	2.12E-05	9098	
11	rs10750352	1.26E+08	6.61E-01	KIRREL3	12	rs2160732	13872593	3.79E-02	GRIN2B	-	6.28E-07	6.75E-01	7.54E-04	9099	
1	rs7418397	1.05E+08	2.50E-02	N/A	12	rs164364	1E+08	7.79E-01	SLC5A8	-	6.28E-07	8.05E-01	7.82E-05	9100	
1	rs648011	2.24E+08	5.54E-01	DN/AH14	4	rs11726933	1.05E+08	8.59E-01	N/A	-	6.28E-07	5.28E-01	5.35E-04	9101	
7	rs10250954	22441474	5.74E-01	MGC87042	16	rs4238833	88578190	7.85E-01	AFG3L1	-	6.28E-07	9.81E-01	5.92E-05	9102	
8	rs12681931	23305045	2.01E-01	LOXL2	18	rs1560434	69656491	9.19E-01	N/A	-	6.28E-07	2.90E-01	1.48E-05	9103	
5	rs4571472	15774360	3.10E-01	FBXL7	10	rs7075349	64097655	2.74E-01	ZNF365	-	6.28E-07	1.93E-03	3.38E-09	9104	
9	rs7873308	23415710	6.87E-01	N/A	11	rs2000560	87328786	6.43E-02	N/A	-	6.28E-07	1.31E-01	1.18E-06	9105	
2	rs897877	2.19E+08	6.02E-01	PNKD	4	rs3762864	1.4E+08	4.37E-01	ELF2	-	6.28E-07	2.04E-01	3.16E-03	9106	
6	rs6931002	1.58E+08	4.57E-01	SNX9	11	rs2850303	1.13E+08	6.79E-01	NCAM1	-	6.29E-07	1.50E-01	3.31E-03	9107	
9	rs7870760	1.34E+08	4.42E-02	NTNG2	15	rs7167668	99720676	6.25E-01	PCSK6	-	6.29E-07	7.27E-01	4.11E-05	9108	
1	rs1324727	1.19E+08	3.52E-01	N/A	7	rs39446	25095313	3.67E-01	N/A	-	6.29E-07	9.69E-01	1.09E-04	9109	
14	rs4901977	59858929	6.13E-01	N/A	16	rs4275868	7289778	9.72E-01	A2BP1	-	6.29E-07	2.90E-02	1.97E-07	9110	
6	rs4534020	1.21E+08	1.83E-01	N/A	6	rs11756851	1.55E+08	7.33E-01	N/A	-	6.29E-07	2.83E-01	2.44E-04	9111	
8	rs11203713	15486984	4.01E-01	TUSC3	12	rs795484	1.17E+08	6.00E-01	TAOK3	-	6.29E-07	6.92E-02	9.75E-03	9112	
12	rs7976486	74660715	3.38E-01	N/A	13	rs11147467	30974678	8.93E-01	N/A	-	6.29E-07	7.75E-02	3.74E-03	9113	
1	rs10494336	1.58E+08	7.15E-01	ATP1A2	8	rs7005426	1.19E+08	1.00E-01	N/A	-	6.29E-07	2.30E-01	6.36E-06	9114	
14	rs6572524	48440753	2.04E-01	N/A	15	rs2570241	49921747	2.29E-02	TMOD3	-	6.29E-07	6.18E-01	4.55E-04	9115	
4	rs2165387	80710844	6.07E-02	N/A	7	rs2390961	24266172	2.98E-01	N/A	-	6.29E-07	2.96E-01	1.11E-05	9116	
1	rs12086212	24581327	6.75E-01	GRHL3	5	rs13180182	1.51E+08	7.83E-02	SLC36A1	-	6.29E-07	7.03E-01	4.33E-04	9117	
1	rs3765431	24578997	6.75E-01	GRHL3	5	rs13180182	1.51E+08	7.83E-02	SLC36A1	-	6.29E-07	7.03E-01	4.33E-04	9118	
3	rs9815931	1.87E+08	9.56E-02	DGKG	16	rs12925884	74997300	7.42E-01	CNTN/AP4	-	6.29E-07	4.96E-01	1.91E-03	9119	
12	rs4764273	16507430	5.26E-01	N/A	14	rs7155553	73171266	2.19E-01	ACOT6	-	6.29E-07	7.40E-01	1.71E-04	9120	
6	rs9404224	1.03E+08	7.25E-01	N/A	3	rs7431637	1.45E+08	2.55E-01	SLC9A9	-	6.29E-07	8.55E-01	1.05E-04	9121	
2	rs1454410	1.66E+08	8.30E-01	SLC38A11	22	rs738089	19102598	4.09E-01	ZNF74	-	6.29E-07	2.88E-02	3.46E-07	9122	
6	rs7453429	15379675	8.41E-02	JARID2	2	rs1730586	36234999	9.69E-01	N/A	-	6.29E-07	1.06E-01	7.34E-03	9123	
6	rs7752992	20533437	9.32E-01	E2F3	2	rs931313	1.34E+08	2.74E-01	N/A	-	6.29E-07	5.56E-01	1.09E-05	9124	
3	rs1847830	1.33E+08	2.10E-01	N/A	4	rs1598859	1.04E+08	8.57E-01	NFKB1	-	6.29E-07	8.95E-01	5.41E-05	9125	
7	rs6946884	15846661	9.92E-01	N/A	7	rs1035041	52419727	5.49E-02	N/A	-	6.29E-07	7.33E-01	2.43E-04	9126	
9	rs1889006	18607804	4.71E-01	ADAMTSL1	13	rs7987126	52829614	1.35E-01	N/A	-	6.29E-07	8.47E-01	7.19E-06	9127	
8	rs12544945	63611710	4.60E-01	NKAIN3	12	rs749137	1.06E+08	6.14E-01	BTBD11	-	6.29E-07	7.70E-01	1.47E-04	9128	
11	rs621922	93994962	2.89E-01	PIWIL4	21	rs12626360	16938048	6.20E-01	N/A	-	6.30E-07	6.55E-02	8.47E-07	9129	
6	rs2170614	2589629	2.83E-01	MYLK4	15	rs2030592	35102817	4.89E-01	MEIS2	-	6.30E-07	1.03E-01	1.89E-03	9130	
4	rs7670864	1.58E+08	5.45E-01	N/A	19	rs259270	37848843	5.40E-01	ANKRD27	-	6.30E-07	2.39E-01	9.80E-06	9131	
3	rs2239624	1.88E+08	2.65E-01	DGKG	7	rs42530	93890431	1.99E-01	COL1A2	-	6.30E-07	5.24E-01	7.62E-05	9132	
2	rs13404712	52797294	9.74E-01	N/A	4	rs13144135	1.87E+08	3.94E-01	SORBS2	-	6.30E-07	5.27E-01	4.42E-06	9133	
2	rs10930874	1.8E+08	7.84E-01	ZNF385B	7	rs6463815	8506230	2.97E-01	NXPH1	-	6.30E-07	5.92E-01	4.71E-05	9134	
3	rs6796724	1.74E+08	5.99E-01	NCEH1	7	rs13240443	57212608	5.19E-01	ZNF479	-	6.30E-07	5.57E-01	1.18E-05	9135	
1	rs10789446	38813821	9.21E-01	N/A	12	rs17353923	16219962	5.92E-01	N/A	-	6.30E-07	4.57E-01	2.29E-03	9136	
4	rs2715388	1.8E+08	3.74E-01	N/A	7	rs10261275	11905958	6.94E-01	N/A	-	6.30E-07	8.38E-01	2.45E-05	9137	
2	rs1974689	64999950	7.68E-01	N/A	15	rs9672398	51666533	7.53E-01	WDR72	-	6.30E-07	2.97E-01	1.14E-03	9138	
6	rs2063474	24053250	2.20E-01	N/A	7	rs2385143	1.04E+08	5.45E-01	N/A	-	6.30E-07	5.06E-01	5.46E-05	9139	
1	rs3121168	56570711	3.74E-01	N/A	5	rs2052527	1.66E+08	7.26E-01	N/A	-	6.30E-07	1.88E-01	3.77E-06	9140	
13	rs9598051	33216946	8.31E-01	N/A	18	rs763361	65682622	4.97E-01	DOK6	-	6.30E-07	2.17E-01	7.85E-07	9141	
6	rs1569579	24621615	3.83E-03	ALDH5A1	7	rs10233000	51661855	7.95E-01	N/A	-	6.30E-07	5.35E-01	2.31E-05	9142	
2	rs4308140	1.02E+08	4.77E-01	N/A	5	rs9687411	35030357	9.17E-01	AGXT2	-	6.30E-07	9.25E-01	2.95E-04	9143	
12	rs2239104	2493804	4.29E-01	CACN/A1C	21	rs457920	41716628	4.76E-01	MX1	-	6.30E-07	2.47E-01	3.61E-03	9144	
6	rs1564601	1.49E+08	8.21E-01	UST	10	rs4748864	23670201	6.74E-01	C10orf67	-	6.30E-07	5.95E-01	5.80E-06	9145	
7	rs17142116	69978722	3.38E-01	N/A	22	rs136140	45767259	4.44E-02	TBC1D22A	-	6.30E-07	8.67E-01	1.07E-04	9146	
9	rs3128519	89376025	2.59E-01	DAPK1	17	rs8064660	50330822	9.53E-01	TOM1L1	-	6.30E-07	9.53E-01	6.84E-05	9147	
5	rs1465687	1.55E+08	3.87E-01	N/A	11	rs11020878	94032763	9.67E-02	N/A	-	6.30E-07	8.18E-01	9.05E-05	9148	
17	rs9907360	66913916	3.31E-01	N/A	21	rs9976900	35306233	6.97E-01	RUNX1	-	6.30E-07	2.14E-01	8.16E-06	9149	
4	rs874950	32976358	9.07E-01	N/A	11	rs2041466	22769111	4.37E-02	GAS2	-	6.30E-07	2.90E-01	8.79E-05	9150	
6	rs4618489	85230221	9.25E-01	N/A	4	rs13146468	5961370	4.15E-01	CRMP1	-	6.30E-07	2.93E-01	1.01E-05	9151	
6	rs9362053	85234736	9.25E-01	N/A	4	rs13146468	5961370	4.15E-01	CRMP1	-	6.30E-07	2.93E-01	1.01E-05	9152	
6	rs1001965	1.5E+08	3.44E-01	N/A	9	rs980462	25343887	1.40E-01	N/A	-	6.30E-07	9.11E-01	2.83E-04	9153	
5	rs11959895	10109951	6.70E-01	N/A	15	rs8038972	67178532	6.33E-01	MIR548H4	-	6.31E-07	2.13E-01	1.65E-06	9154	
4	rs2614563	1.8E+08	1.82E-01	N/A	8	rs4074500	80419832	1.77E-01	N/A	-	6.31E-07	5.18E-01	2.26E-04	9155	
4	rs2702386	1.8E+08	1.82E-01	N/A	8	rs4074500	80419832	1.77E-01	N/A	-	6.31E-07	N/A	N/A	9156	
16	rs8049015	19300744	4.96E-01	N/A	18	rs1465943	48315280	7.67E-01	DCC	-	6.31E-07	6.18E-02	1.07E-02	9157	
6	rs948521	1.09E+08	2.20E-01	ARMC2	2	rs2032953	2.32E+08	3.56E-01	N/A	-	6.31E-07	1.24E-01	4.94E-06	9158	
13	rs4142313	42995658	4.52E-01	ENOX1	14	rs12590361	1.02E+08	5.10E-01	RCOR1	-	6.31E-07	7.19E-01	1.04E-05	9159	
9	rs12685329	715147	1.35E-01	KANK1	22	rs8142159	41820578	3.33E-01	BIK	-	6.31E-07	8.24E-01	2.55E-05	9160	
2	rs6724530	1.2E+08	9.04E-02	N/A	11	rs532751	74806655	9.59E-01	GDPD5	-	6.31E-07	7.78E-01	1.02E-04	9161	
13	rs9534651	46919997	2.74E-01	N/A	19	rs4804579	11219700	7.85E-02	DOCK6	-	6.31E-07	6.59E-02	5.37E-07	9162	
10	rs10508362	8714527	7.25E-01	N/A	12	rs974728	11826119	5.72E-01	ETV6	-	6.31E-07	7.92E-01	1.21E-04	9163	
12	rs10506125	37406554	6.58E-01	CPNE8	15	rs8038332	91111579	3.77E-01	N/A	-	6.31E-07	N/A	N/A	9164	
5	rs6892878	1.25E+08	8.05E-01	N/A	16	rs4889066	78607771	5.13E-01	N/A	-	6.31E-07	7.21E-01	1.01E-04	9165	
8	rs4738873	62248730	8.62E-01	N/A	22	rs134260	33875092	4.63E-01	N/A	-	6.31E-07	9.16E-01	2.96E-04	9166	
5	rs11743924	1.25E+08	8.05E-01	N/A	16	rs4889066	78607771	5.13E-01	N/A	-	6.31E-07	9.34E-01	2.81E-04	9167	
1	rs2890085	11399128	1.05E-01	N/A	15	rs4965726	99202209	4.48E-01	N/A	-	6.31E-07	3.77E-01	9.10E-06	9168	
10	rs2862833	90765609	7.44E-01	FAS	20	rs6063248	46444226	3.46E-01	LOC284749	-	6.31E-07	7.80E-01	1.82E-04	9169	
3	rs658724	61790490	4.27E-01	PTPRG	14	rs438402	44018211	9.20E-01	N/A	-	6.32E-07	6.38E-01	3.90E-05	9170	
2	rs12994560	75658556	9.34E-01	FAM176A	8	rs6473088	79172145	3.72E-01	N/A	-	6.32E-07	7.18E-01	1.25E-04	9171	
18	rs16946301	24998498	1.12E-01	N/A	20	rs6078121	11225755	1.99E-01	N/A	-	6.32E-07	7.59E-01	1.58E-04	9172	
2	rs7575223	1.42E+08	7.86E-01	LRP1B	12	rs11609325	1.27E+08	5.22E-01	TMEM132C	-	6.32E-07	9.68E-01	4.92E-05	9173	
2	rs4672884	2.19E+08	5.09E-01	PNKD	4	rs2292837	1.4E+08	2.54E-01	ELF2	-	6.32E-07	3.85E-01	1.50E-03	9174	
6	rs2063474	24053250	2.20E-01	N/A	7	rs10259086	1.04E+08	6.23E-01	N/A	-	6.32E-07	4.75E-01	4.37E-05	9175	
3	rs2366672	1.94E+08	6.44E-02	FGF12	10	rs6560722	1322488	5.23E-01	ADARB2	-	6.32E-07	9.95E-01	1.15E-04	9176	
1	rs1774825	57022146	9.57E-01	C1orf168	10	rs2814930	3279771	9.21E-01	N/A	-	6.32E-07	5.98E-02	1.33E-03	9177	
5	rs2112197	1.43E+08	5.26E-01	N/A	9	rs2298181	1.29E+08	4.46E-01	FAM129B	-	6.32E-07	2.72E-01	1.15E-03	9178	
5	rs17179868	11081344	5.44E-01	CTNND2	9	rs10814848	4100947	9.30E-01	GLIS3	-	6.32E-07	3.42E-01	6.57E-04	9179	
14	rs11621843	92185877	4.54E-01	RIN3	16	rs247044	56473419	3.60E-01	CNGB1	-	6.32E-07	5.49E-01	2.31E-04	9180	
1	rs11587479	7745672	2.87E-01	CAMTA1	8	rs1133076	1.34E+08	8.05E-01	SLA	-	6.32E-07	4.66E-02	2.73E-03	9181	
7	rs12670282	1.38E+08	4.88E-01	ZC3HAV1	17	rs9893164	9309408	9.72E-01	STX8	-	6.32E-07	2.90E-01	6.08E-05	9182	

SNP A					SNP B						Interaction P			Ranking	Cluster in top 100 interactions
CHR	SNP	Location	gle locus P va	Gene	CHR	SNP	Location	Single locus P valu	Gene	MHC region	Stage 1	Stage 2	Combined		
2	rs238625	8305547	6.67E-01	N/A	8	rs11787080	1.43E+08	7.59E-01	N/A	-	6.32E-07	3.02E-01	2.06E-05	9183	
12	rs7296020	61595594	4.84E-01	PPM1H	13	rs2352192	91373182	8.48E-01	GPC5	-	6.32E-07	8.35E-01	1.89E-04	9184	
2	rs9750891	2.34E+08	8.23E-01	INPP5D	4	rs4698457	15826043	8.44E-01	FLJ39653	-	6.32E-07	9.70E-01	1.43E-04	9185	
4	rs918402	1.82E+08	2.52E-01	N/A	13	rs9534199	45189527	6.31E-01	SPERT	-	6.32E-07	1.66E-03	1.50E-08	9186	
14	rs8010088	36259816	1.26E-01	SLC25A21	18	rs2365332	42295148	7.84E-01	RNF165	-	6.32E-07	6.68E-01	9.52E-04	9187	
10	rs11596728	12685893	8.39E-01	CAMK1D	16	rs151696	17976386	8.06E-01	N/A	-	6.32E-07	4.62E-01	4.21E-04	9188	
5	rs7705243	35155303	8.67E-01	PRLR	16	rs11632150	43839682	5.18E-01	N/A	-	6.32E-07	6.13E-01	1.57E-04	9189	
12	rs721793	66720281	1.22E-01	N/A	15	rs12439503	98679389	7.51E-01	ADAMTS17	-	6.32E-07	N/A	N/A	9190	
9	rs2841490	87117835	4.48E-01	N/A	15	rs17648050	91063933	4.00E-01	N/A	-	6.32E-07	2.45E-02	1.34E-07	9191	
2	rs2289200	2.16E+08	8.14E-01	FN1	7	rs6952109	88033266	5.62E-01	N/A	-	6.32E-07	6.77E-01	2.41E-04	9192	
5	rs492842	78445743	7.97E-01	BHMT	16	rs1423797	63846744	1.23E-01	N/A	-	6.32E-07	8.62E-01	9.43E-05	9193	
11	rs4930351	65823240	1.20E-01	TMEM151A	18	rs2849210	63661045	1.99E-01	N/A	-	6.33E-07	2.63E-01	3.90E-06	9194	
10	rs4749844	9326574	8.09E-01	N/A	16	rs1383361	72231717	8.81E-01	N/A	-	6.33E-07	6.35E-01	3.46E-05	9195	
1	rs17625634	1.76E+08	7.60E-01	N/A	16	rs11150110	77489180	4.14E-02	WWOX	-	6.33E-07	6.65E-01	1.95E-05	9196	
3	rs16854239	1.71E+08	3.84E-01	MECOM	8	rs4872278	24911501	3.94E-01	N/A	-	6.33E-07	6.81E-01	5.10E-05	9197	
12	rs2283306	2359651	3.69E-01	CACN/A1C	20	rs4811033	48392648	4.65E-01	N/A	-	6.33E-07	5.04E-01	5.16E-04	9198	
2	rs883844	1.92E+08	8.59E-01	GLS	2	rs6436670	2.28E+08	3.77E-01	COL4A3	-	6.33E-07	5.30E-01	1.71E-05	9199	
12	rs1108385	2352591	3.69E-01	CACN/A1C	20	rs4811033	48392648	4.65E-01	N/A	-	6.33E-07	5.51E-01	4.44E-04	9200	
6	rs3943166	76986284	4.64E-01	N/A	11	rs4550210	40729088	1.06E-01	N/A	-	6.33E-07	7.52E-01	1.56E-04	9201	
6	rs2844665	31114834	8.49E-01	HCG22	22	rs2071762	43636988	5.14E-01	PHF21B	MHC	6.33E-07	8.38E-01	2.64E-04	9202	
7	rs306721	79740223	3.85E-01	N/A	8	rs1455583	63975240	1.06E-01	NKAIN3	-	6.33E-07	5.16E-02	5.87E-07	9203	
11	rs16907631	21164119	1.45E-01	NELL1	12	rs10841780	21309410	9.69E-01	SLCO1A2	-	6.33E-07	3.52E-01	7.20E-04	9204	
11	rs260858	1.03E+08	1.08E-01	PDGFD	19	rs11668141	3708409	1.06E-01	APBA3	-	6.33E-07	8.37E-02	4.08E-06	9205	
10	rs2814317	88047586	7.83E-01	GRID1	10	rs4917503	1.09E+08	8.54E-01	N/A	-	6.33E-07	1.04E-01	1.36E-06	9206	
1	rs1410397	1.92E+08	4.39E-01	N/A	22	rs131853	46141822	2.23E-01	N/A	-	6.34E-07	1.09E-01	5.89E-07	9207	
1	rs1113239	1.92E+08	4.39E-01	N/A	22	rs131853	46141822	2.23E-01	N/A	-	6.34E-07	1.30E-01	7.43E-07	9208	
8	rs209973	17595134	4.36E-01	MTUS1	12	rs17129591	40286489	5.32E-01	N/A	-	6.34E-07	3.25E-01	1.41E-05	9209	
4	rs4241809	1.87E+08	5.84E-01	SORBS2	5	rs2962365	17308515	9.31E-01	BASP1	-	6.34E-07	1.75E-01	2.10E-06	9210	
12	rs3847896	23051233	1.41E-02	N/A	16	rs1273422	8756514	9.67E-01	ABAT	-	6.34E-07	9.25E-01	9.97E-05	9211	
1	rs17100624	97793543	1.62E-01	DPYD	10	rs2420255	1.18E+08	2.57E-01	GFRA1	-	6.34E-07	8.38E-01	2.16E-04	9212	
5	rs10040610	6406815	2.75E-01	MED10	9	rs3789892	92611380	8.51E-01	N/A	-	6.34E-07	3.01E-01	2.38E-06	9213	
9	rs1410964	24262883	6.51E-01	N/A	12	rs11051659	31945102	6.68E-01	N/A	-	6.34E-07	2.08E-01	1.28E-03	9214	
8	rs1565741	16799856	1.33E-01	N/A	10	rs10509252	67491726	6.44E-01	CTNN/A3	-	6.34E-07	3.30E-01	1.00E-03	9215	
6	rs10447418	1.31E+08	2.49E-01	SAMD3	9	rs2418076	1.12E+08	3.34E-01	TXN	-	6.34E-07	6.89E-01	5.74E-05	9216	
8	rs9644041	25377198	8.17E-01	KCTD9	12	rs10492261	1.14E+08	8.29E-01	N/A	-	6.34E-07	1.68E-01	1.75E-06	9217	
8	rs9644041	25377198	8.17E-01	KCTD9	12	rs11067378	1.14E+08	8.29E-01	N/A	-	6.34E-07	1.78E-01	1.92E-06	9218	
3	rs779165	22077711	2.06E-01	N/A	9	rs13288677	1.37E+08	6.20E-01	COL5A1	-	6.34E-07	3.34E-01	1.44E-03	9219	
20	rs4811050	48414077	8.82E-01	N/A	22	rs12159191	43022888	1.97E-01	KIAA1644	-	6.34E-07	1.34E-01	2.03E-06	9220	
15	rs12592193	80303094	6.37E-01	EFTUD1	22	rs5751902	23326630	2.18E-01	GGT1	-	6.34E-07	3.93E-01	9.53E-06	9221	
5	rs1152168	1.03E+08	7.35E-02	N/A	12	rs1463604	30005035	3.95E-01	N/A	-	6.34E-07	7.03E-01	2.05E-05	9222	
5	rs889268	37917929	2.47E-01	N/A	12	rs10845252	11010287	3.83E-01	PRH1	-	6.34E-07	7.18E-01	2.69E-04	9223	
13	rs10492646	1.06E+08	7.68E-01	N/A	17	rs1492219	48083872	8.58E-01	N/A	-	6.35E-07	3.27E-01	1.98E-05	9224	
2	rs12712127	1.02E+08	3.78E-01	N/A	3	rs7622275	1.49E+08	7.49E-01	N/A	-	6.35E-07	9.91E-01	1.37E-04	9225	
5	rs1445978	60890827	3.01E-01	ZSWIM6	13	rs16968984	1.06E+08	4.53E-01	N/A	-	6.35E-07	4.39E-01	5.11E-06	9226	
2	rs952822	52704577	2.36E-01	N/A	5	rs13161840	1.2E+08	4.89E-01	PRR16	-	6.35E-07	4.55E-01	3.52E-04	9227	
1	rs10873827	87527272	2.21E-01	N/A	1	rs631230	1.15E+08	9.64E-01	N/A	-	6.35E-07	7.61E-01	2.83E-05	9228	
10	rs4750665	15603456	4.86E-02	ITGA8	22	rs4282002	26350555	9.72E-01	N/A	-	6.35E-07	5.27E-01	4.07E-06	9229	
8	rs3750306	1.39E+08	7.03E-01	FAM135B	12	rs11059243	1.27E+08	1.69E-01	N/A	-	6.35E-07	N/A	N/A	9230	
14	rs2352900	48441520	1.86E-01	N/A	15	rs8031045	49931596	2.60E-02	TMOD3	-	6.35E-07	5.46E-01	4.56E-04	9231	
5	rs334470	16565364	7.81E-01	FAM134B	19	rs4804000	51968294	4.75E-01	SLC1A5	-	6.35E-07	5.35E-01	1.03E-04	9232	
1	rs1132185	12743457	4.07E-01	C1orf158	8	rs2741098	6677686	2.86E-01	XKR5	-	6.35E-07	7.74E-01	4.16E-05	9233	
12	rs10770444	8341081	6.06E-01	N/A	18	rs2847286	12807815	5.51E-01	PTPN2	-	6.35E-07	9.86E-01	2.23E-04	9234	
9	rs2251530	86002760	4.40E-01	N/A	9	rs581558	1.27E+08	8.25E-01	GOLGA1	-	6.35E-07	7.84E-01	9.98E-05	9235	
1	rs3010888	12710219	4.08E-01	AADACL3	8	rs2741098	6677686	2.86E-01	XKR5	-	6.35E-07	9.39E-01	6.56E-05	9236	
4	rs6552935	1.87E+08	1.33E-02	SORBS2	20	rs2426843	57814240	7.05E-01	PHACTR3	-	6.35E-07	9.93E-02	5.46E-03	9237	
5	rs10447192	8949731	9.66E-01	N/A	20	rs6074558	13045311	9.77E-01	SPTLC3	-	6.35E-07	1.93E-01	1.12E-03	9238	
8	rs4602870	68905107	7.27E-02	N/A	17	rs11868948	10154767	5.03E-01	MYH13	-	6.35E-07	6.83E-01	1.63E-04	9239	
8	rs4602870	68905107	7.27E-02	N/A	17	rs4451985	10157958	5.03E-01	MYH13	-	6.35E-07	8.01E-01	1.22E-04	9240	
6	rs9689723	1.5E+08	6.32E-02	KATN/A1	4	rs1842534	34244104	3.80E-01	N/A	-	6.35E-07	9.01E-02	1.43E-03	9241	
6	rs732498	1.6E+08	5.30E-01	SOD2	12	rs7133563	5308881	6.30E-01	N/A	-	6.35E-07	5.80E-01	3.05E-05	9242	
2	rs2341991	16854392	8.05E-01	N/A	21	rs6518237	45627380	4.76E-01	N/A	-	6.35E-07	6.56E-01	4.07E-04	9243	
8	rs10102945	61469669	7.77E-01	N/A	20	rs2425464	39997455	4.56E-01	N/A	-	6.35E-07	4.25E-02	1.83E-07	9244	
1	rs4908382	28156065	2.56E-01	EYA3	10	rs478839	89721850	9.83E-01	PTEN	-	6.36E-07	1.71E-01	3.20E-06	9245	
8	rs12544945	63611710	4.60E-01	NKAIN3	12	rs7973935	1.06E+08	6.57E-01	BTBD11	-	6.36E-07	7.70E-01	1.47E-04	9246	
1	rs735937	95108357	2.74E-01	SLC44A3	7	rs567184	1.05E+08	2.78E-01	ATXN7L1	-	6.36E-07	5.87E-01	3.86E-04	9247	
2	rs4675140	2.28E+08	5.70E-01	COL4A4	8	rs4243888	1.39E+08	7.31E-01	FAM135B	-	6.36E-07	9.04E-02	4.92E-07	9248	
5	rs7725801	1.22E+08	6.92E-01	SNCAIP	11	rs11037217	43006402	8.33E-01	N/A	-	6.36E-07	7.17E-01	7.07E-05	9249	
1	rs7514281	1.88E+08	4.90E-01	N/A	4	rs10028606	1.89E+08	4.98E-01	N/A	-	6.36E-07	2.62E-01	9.62E-04	9250	
6	rs9356355	1.66E+08	1.05E-01	C6orf118	5	rs10079374	17863895	8.53E-01	N/A	-	6.36E-07	7.14E-01	4.03E-05	9251	
4	rs1108256	77987610	4.33E-01	N/A	8	rs4909749	1.39E+08	1.25E-01	FAM135B	-	6.36E-07	7.21E-01	3.59E-06	9252	
3	rs4560291	62187202	4.94E-01	PTPRG	9	rs1442528	17319964	7.90E-01	CNTLN	-	6.36E-07	9.12E-01	1.51E-04	9253	
2	rs4616447	1.7E+08	2.67E-01	N/A	17	rs244292	50585733	4.53E-01	STXBP4	-	6.36E-07	7.53E-02	5.83E-07	9254	
6	rs2714342	7199640	1.56E-01	RREB1	14	rs5028394	68141443	1.18E-01	RAD51L1	-	6.36E-07	5.80E-01	1.45E-05	9255	
1	rs12033458	18330155	2.84E-01	IGSF21	13	rs10507989	90366295	4.82E-01	LOC144776	-	6.36E-07	1.17E-01	1.98E-06	9256	
3	rs12488746	76751352	8.44E-01	N/A	4	rs4388065	1.76E+08	8.29E-01	ADAM29	-	6.36E-07	3.50E-01	6.67E-04	9257	
6	rs650849	93158360	2.11E-01	N/A	10	rs4935336	54117352	2.63E-01	N/A	-	6.36E-07	7.98E-01	2.28E-04	9258	
10	rs1149952	9256962	4.81E-01	N/A	12	rs1647253	1.2E+08	3.23E-01	AN/APC5	-	6.36E-07	1.03E-01	8.43E-07	9259	
10	rs11201128	86370207	7.10E-01	N/A	20	rs6114101	23394322	3.37E-01	CST11	-	6.36E-07	9.18E-03	2.08E-07	9260	
5	rs6888963	1.15E+08	3.50E-01	N/A	11	rs3781710	34472826	7.96E-01	ELF5	-	6.36E-07	5.34E-01	6.08E-06	9261	
5	rs1824788	58476015	3.45E-01	PDE4D	10	rs7922657	25381004	9.13E-01	N/A	-	6.36E-07	5.92E-01	5.80E-05	9262	
3	rs9830614	916639	6.88E-01	N/A	7	rs2402852	1.26E+08	3.71E-01	GRM8	-	6.36E-07	6.58E-01	7.06E-04	9263	
2	rs4853948	2925053	9.25E-01	N/A	3	rs1396414	3471478	7.61E-01	N/A	-	6.36E-07	N/A	N/A	9264	
8	rs993883	5179603	1.13E-01	N/A	12	rs10773579	1.28E+08	5.78E-01	SLC15A4	-	6.37E-07	5.22E-01	8.38E-04	9265	
4	rs4362823	1.03E+08	3.70E-01	N/A	14	rs4144016	29079928	2.42E-01	N/A	-	6.37E-07	9.02E-01	7.03E-05	9266	
7	rs10499928	96222714	1.07E-01	N/A	14	rs10149476	1.06E+08	1.84E-01	N/A	-	6.37E-07	2.93E-01	6.50E-06	9267	
7	rs990902	96246861	1.07E-01	N/A	14	rs10149476	1.06E+08	1.84E-01	N/A	-	6.37E-07	2.93E-01	6.50E-06	9268	
3	rs1300120	1.04E+08	2.85E-01	N/A	13	rs9534986	47665333	6.35E-01	N/A	-	6.37E-07	3.15E-01	3.99E-06	9269	
6	rs1265156	31250276	6.76E-01	TCF19	6	rs9348862	31360726	7.72E-01	HLA-C	MHC	6.37E-07	8.49E-01	5.13E-05	9270	
6	rs1265156	31250276	6.76E-01	TCF19	6	rs9357122	31368324	7.72E-01	N/A	MHC	6.37E-07	8.49E-01	5.13E-05	9271	
14	rs932543	47239589	3.98E-01	N/A	16	rs734710	82831935	7.58E-01	KCNG4	-	6.37E-07	8.61E-01	2.06E-04	9272	
1	rs1321776	1.76E+08	2.94E-01	N/A	7	rs10269431	11937854	6.48E-01	N/A	-	6.37E-07	7.48E-01	3.73E-04	9273	
3	rs12487078	1.28E+08	4.87E-01	N/A	5	rs13188923	1.12E+08	5.60E-01	N/A	-	6.37E-07	1.96E-01	3.73E-06	9274	
2	rs6434981	1.99E+08	8.18E-01	N/A	19	rs11670254	10504352	9.69E-01	MIR1238	-	6.37E-07	3.68E-01	4.88E-04	9275	
1	rs1617731	2.07E+08	5.48E-02	N/A	5	rs4631227	1.33E+08	5.10E-02	N/A	-	6.37E-07	8.11E-01	3.21E-04	9276	
1	rs1934206	1.93E+08	2.42E-01	N/A	20	rs366496	53690857	1.71E-01	N/A	-	6.37E-07	6.73E-01	5.28E-05	9277	
2	rs3769184	1.74E+08	9.80E-01	ZAK	18	rs4539677	43190135	4.57E-01	N/A	-	6.37E-07	6.47E-02	1.03E-02	9278	
5	rs7717186	1.46E+08	4.91E-01	N/A	19	rs6508962	33798612	5.53E-01	N/A	-	6.37E-07	9.90E-02	5.42E-07	9279	
1	rs12086324	69453778	2.65E-01	N/A	4	rs6833157	23603253	1.57E-01	N/A	-	6.37E-07	1.09E-01	1.99E-06	9280	
5	rs2569208	1.72E+08	5.28E-01	SH3PXD2B	22	rs9609689	31763761	3.04E-01	SYN3	-	6.37E-07	2.46E-01	2.18E-03	9281	
4	rs4580639	25254263	7.14E-01	N/A	11	rs12789646	1.31E+08	2.47E-01	NTM	-	6.37E-07	6.26E-01	1.69E-04	9282	
6	rs9378518	806084	4.97E-01	N/A	1	rs331635	59438130	5.19E-01	N/A	-	6.37E-07	9.37E-01	7.42E-05	9283	
8	rs6991624	1.15E+08	3.32E-01	N/A	19	rs8103853	63269574	6.55E-01	ZNF135	-	6.38E-07	3.89E-01	3.73E-06	9284	
13	rs7327643	65593386	5.40E-01	N/A	16	rs729633	52909791	5.10E-01	N/A	-	6.38E-07	9.42E-01	2.53E-04	9285	
3	rs17498471	6332198	6.63E-01	N/A	14	rs1887064	20695102	9.70E-01	OR5AU1	-	6.38E-07	9.60E-01	1.18E-04	9286	
12	rs964997	1.25E+08	4.28E-01	TMEM132B	15	rs2077422	31978578	8.89E-01	AVEN	-	6.38E-07	8.85E-01	9.47E-05	9287	
7	rs17168135	1.34E+08	3.50E-01	CALD1	8	rs4733828	1.29E+08	1.24E-01	PVT1	-	6.38E-07	1.24E-01	4.59E-06	9288	
4	rs13106255	1.85E+08	4.83E-01	N/A	14	rs4899251	68219567	3.01E-01	N/A	-	6.38E-07	4.95E-01	1.00E-03	9289	
1	rs609516	2.35E+08	8.71E-01	N/A	8	rs281712	1.09E+08	9.95E-01	N/A	-	6.38E-07	1.03E-01	1.24E-03	9290	
19	rs7256487	19716654	6.42E-02	ZNF14	22	rs2092117	33251246	4.00E-01	N/A	-	6.38E-07	2.40E-01	1.31E-03	9291	
6	rs3095250	31316319	6.90E-02	N/A	6	rs9266409	31444547	1.40E-01	HLA-B	MHC	6.38E-07	1.78E-01	1.12E-05	9292	
10	rs11254294	17033583	3.68E-04	CUBN	14	rs2355659	49553790	7.51E-01	C14orf182	-	6.38E-07	8.26E-01	3.31E-04	9293	
5	rs1423611	50806871	7.37E-01	N/A	12	rs2306283	21221005	1.30E-01	SLCO1B1	-	6.38E-07	9.60E-01	3.57E-04	9294	

SNP A					SNP B						Interaction P			Ranking	Cluster in top 100 interactions
CHR	SNP	Location	gle locus P va	Gene	CHR	SNP	Location	Single locus P valu	Gene	MHC region	Stage 1	Stage 2	Combined		
4	rs4558821	1.57E+08	4.26E-01	N/A	12	rs2701623	1.12E+08	8.21E-01	DTX1	-	6.38E-07	2.58E-01	2.56E-06	9295	
3	rs6782826	1.14E+08	3.68E-01	N/A	16	rs1244838	84705528	5.48E-01	N/A	-	6.38E-07	8.08E-01	5.17E-05	9296	
10	rs1186361	1.29E+08	3.75E-01	DOCK1	17	rs9891348	50929373	1.18E-01	N/A	-	6.38E-07	7.10E-01	8.59E-05	9297	
6	rs1893556	1.63E+08	5.41E-01	PARK2	8	rs218019	1.19E+08	1.93E-01	N/A	-	6.38E-07	1.61E-01	3.12E-03	9298	
10	rs7098200	75769563	9.12E-01	ADK	15	rs1996142	95507602	8.77E-02	N/A	-	6.38E-07	3.03E-01	9.29E-06	9299	
8	rs7001469	15841353	3.68E-01	N/A	16	rs7184872	27374838	4.93E-01	GTF3C1	-	6.38E-07	8.16E-01	5.50E-05	9300	
4	rs4546199	1.64E+08	9.51E-01	N/A	9	rs3118561	1.37E+08	1.70E-01	N/A	-	6.38E-07	N/A	N/A	9301	
1	rs7418187	56706132	9.31E-01	N/A	11	rs1078985	1.11E+08	8.70E-01	PIH1D2	-	6.39E-07	7.86E-01	6.36E-04	9302	
4	rs10002827	1.45E+08	3.88E-01	N/A	5	rs9292871	43340159	9.92E-01	HMGCS1	-	6.39E-07	3.84E-01	2.18E-06	9303	
7	rs7801539	1.17E+08	9.79E-01	CTTNBP2	16	rs1862453	27857577	4.07E-01	GSG1L	-	6.39E-07	1.19E-02	8.86E-08	9304	
7	rs12701102	31522149	5.92E-01	CCDC129	7	rs675242	47556332	3.71E-01	TNS3	-	6.39E-07	2.46E-01	2.84E-03	9305	
4	rs9992791	45217342	8.87E-01	N/A	18	rs558673	5898869	7.01E-01	TMEM200C	-	6.39E-07	3.27E-01	5.13E-04	9306	
6	rs4495304	31188697	4.80E-02	C6orf15	6	rs1265074	31221193	9.61E-01	TCF19	MHC	6.39E-07	5.32E-02	5.59E-07	9307	
6	rs4495304	31188697	4.80E-02	C6orf15	6	rs1265093	31215166	9.61E-01	TCF19	MHC	6.39E-07	5.32E-02	5.59E-07	9308	
6	rs4495304	31188697	4.80E-02	C6orf15	6	rs1966	31215712	9.61E-01	TCF19	MHC	6.39E-07	5.32E-02	5.59E-07	9309	
6	rs3130453	31232828	5.90E-01	TCF19	6	rs3130685	31314185	9.60E-02	N/A	MHC	6.39E-07	3.05E-01	1.11E-05	9310	
5	rs3828699	1.26E+08	5.49E-01	LMNB1	19	rs3786669	55178751	3.89E-02	SIGLEC16	-	6.39E-07	6.34E-01	2.35E-05	9311	
1	rs2024788	61659856	4.03E-01	NFIA	4	rs2720372	10226723	8.99E-01	CLNK	-	6.39E-07	5.66E-01	1.94E-04	9312	
12	rs2673730	1.07E+08	5.03E-01	N/A	17	rs7223756	74794846	1.98E-01	HRNBP3	-	6.39E-07	8.92E-01	1.87E-04	9313	
12	rs11045834	21232363	4.07E-02	SLCO1B1	14	rs181484	68427927	4.63E-01	ACTN1	-	6.39E-07	3.56E-02	1.53E-02	9314	
1	rs2024788	61659856	4.03E-01	NFIA	4	rs978591	10227767	8.47E-01	CLNK	-	6.39E-07	5.66E-01	1.94E-04	9315	
4	rs6814124	1.51E+08	4.99E-01	N/A	9	rs7861959	9141280	9.21E-01	PTPRD	-	6.39E-07	7.57E-01	2.62E-04	9316	
15	rs12441340	22875057	2.86E-01	SNORD116-3	18	rs1550716	35493076	1.30E-03	LOC647946	-	6.39E-07	9.22E-02	1.68E-03	9317	
4	rs11943009	1.34E+08	7.64E-01	N/A	5	rs1818350	90269689	2.09E-01	GPR98	-	6.39E-07	3.98E-01	1.25E-05	9318	
4	rs11100040	1.57E+08	7.77E-01	N/A	19	rs4808526	16737099	6.66E-01	NWD1	-	6.39E-07	9.32E-01	1.30E-04	9319	
6	rs2677818	1.34E+08	3.18E-01	N/A	13	rs9513249	96957705	6.69E-01	N/A	-	6.39E-07	9.57E-01	3.52E-04	9320	
6	rs2677820	1.34E+08	3.18E-01	N/A	13	rs9513249	96957705	6.69E-01	N/A	-	6.39E-07	9.57E-01	3.52E-04	9321	
6	rs2636601	1.34E+08	3.18E-01	N/A	13	rs9513249	96957705	6.69E-01	N/A	-	6.39E-07	9.68E-01	3.65E-04	9322	
16	rs8061907	81574017	1.49E-01	CDH13	22	rs5763284	28229724	2.21E-01	NEFH	-	6.40E-07	1.83E-01	1.50E-03	9323	
4	rs2614563	1.8E+08	1.82E-01	N/A	8	rs1095793	80439275	7.60E-01	N/A	-	6.40E-07	6.29E-01	2.36E-04	9324	
8	rs17283426	1.06E+08	9.86E-01	ZFPM2	11	rs1249582	68522471	7.82E-01	MRGPRD	-	6.40E-07	7.29E-01	3.43E-05	9325	
10	rs8578	98343411	7.82E-01	TM9SF3	11	rs1228306	98665712	8.47E-01	CNTN5	-	6.40E-07	9.62E-01	2.91E-04	9326	
4	rs7661705	60539135	5.95E-01	N/A	7	rs2192017	1.22E+08	8.28E-01	CADPS2	-	6.40E-07	9.95E-01	2.00E-04	9327	
4	rs2702386	1.8E+08	1.82E-01	N/A	8	rs1095793	80439275	7.60E-01	N/A	-	6.40E-07	N/A	N/A	9328	
15	rs7180351	68239801	7.04E-01	N/A	19	rs2965261	58467498	9.88E-01	ZNF677	-	6.40E-07	3.65E-01	3.29E-04	9329	
4	rs1478095	1.32E+08	6.79E-01	N/A	8	rs2114695	82661686	2.74E-01	N/A	-	6.40E-07	N/A	N/A	9330	
5	rs10515518	1.42E+08	2.25E-01	ARHGAP26	11	rs1123489	86348490	5.82E-01	FZD4	-	6.40E-07	9.51E-01	5.57E-05	9331	
4	rs13119453	1.86E+08	6.91E-01	IRF2	11	rs1073643	96986617	9.20E-01	N/A	-	6.40E-07	7.90E-02	5.66E-06	9332	
3	rs333507	78923643	6.68E-02	ROBO1	21	rs2007397	41438062	1.27E-02	C21orf130	-	6.40E-07	7.97E-02	3.81E-07	9333	
3	rs7650002	78926964	6.68E-02	ROBO1	21	rs2007397	41438062	1.27E-02	C21orf130	-	6.40E-07	7.97E-02	3.81E-07	9334	
3	rs7623391	1.66E+08	3.36E-01	N/A	4	rs7697897	1.79E+08	1.72E-01	LOC285501	-	6.40E-07	5.32E-01	8.34E-06	9335	
1	rs12120414	56615717	7.99E-01	N/A	5	rs7732252	26006423	3.76E-01	N/A	-	6.40E-07	1.29E-01	8.39E-04	9336	
1	rs2317955	1.57E+08	5.40E-01	N/A	2	rs2114631	1.6E+08	5.19E-01	LY75	-	6.40E-07	3.67E-01	9.91E-04	9337	
6	rs9380215	31157634	4.29E-02	N/A	6	rs3130534	31317024	3.50E-01	N/A	MHC	6.40E-07	1.21E-01	2.09E-06	9338	
6	rs1076673	6572183	9.01E-01	LOC285780	2	rs6542834	98588371	2.67E-01	INPP4A	-	6.40E-07	4.85E-01	2.46E-04	9339	
5	rs216535	1.11E+08	6.28E-01	CAMK4	9	rs4878368	27223367	1.26E-01	NCRNA00032	-	6.40E-07	6.12E-01	1.21E-05	9340	
3	rs7645792	1.42E+08	3.90E-01	N/A	12	rs10450	55270873	6.76E-01	BAZ2A	-	6.40E-07	2.15E-01	2.39E-06	9341	
1	rs6702660	4923290	9.64E-01	N/A	13	rs1322812	36877365	7.17E-01	N/A	-	6.41E-07	5.53E-03	3.58E-02	9342	
4	rs1500453	21902341	9.12E-01	N/A	17	rs1051251	62642338	1.53E-01	HELZ	-	6.41E-07	9.24E-03	2.02E-07	9343	
2	rs833764	36047684	6.67E-01	N/A	8	rs3943615	54109850	2.27E-02	N/A	-	6.41E-07	1.88E-01	2.63E-06	9344	
2	rs10931519	1.93E+08	5.31E-01	TMEFF2	4	rs2611074	66677216	7.19E-01	N/A	-	6.41E-07	3.98E-01	1.44E-05	9345	
1	rs16845221	2.24E+08	5.89E-01	N/A	3	rs4857355	99444827	7.71E-01	N/A	-	6.41E-07	1.91E-01	6.94E-04	9346	
11	rs2555141	6615883	6.85E-01	DCHS1	16	rs205355	28012030	9.04E-01	XPO6	-	6.41E-07	1.06E-01	2.41E-03	9347	
6	rs9295638	25074668	3.49E-01	N/A	1	rs1091462	33071409	6.91E-01	S100PBP	MHC	6.41E-07	8.95E-01	4.61E-04	9348	
8	rs1443930	1.09E+08	8.89E-01	RSPO2	9	rs4877161	81984071	5.30E-01	N/A	-	6.41E-07	6.20E-01	2.08E-04	9349	
5	rs6450270	54287290	6.72E-01	N/A	7	rs1008549	9629332	2.00E-01	PER4	-	6.41E-07	9.57E-01	6.36E-05	9350	
2	rs2033172	1.37E+08	3.96E-01	N/A	8	rs2447504	99195082	5.73E-01	HRSP12	-	6.41E-07	4.63E-01	1.79E-03	9351	
1	rs2808019	2.19E+08	2.72E-02	RAB3GAP2	8	rs1050470	80373804	3.99E-02	N/A	-	6.41E-07	5.23E-01	1.46E-03	9352	
1	rs2808019	2.19E+08	2.72E-02	RAB3GAP2	8	rs1460162	80389955	3.99E-02	N/A	-	6.41E-07	5.23E-01	1.46E-03	9353	
9	rs10114562	1.16E+08	3.37E-01	N/A	18	rs9941427	62514281	3.52E-01	N/A	-	6.41E-07	5.76E-01	2.19E-05	9354	
8	rs11261474	86481902	3.43E-01	CA1	10	rs2394375	68825642	3.00E-01	CTNN/A3	-	6.42E-07	6.38E-01	1.76E-05	9355	
11	rs601715	87461466	9.12E-02	N/A	12	rs1353360	69793828	6.32E-01	TSPAN8	-	6.42E-07	2.30E-01	4.13E-06	9356	
2	rs10804016	1.88E+08	1.12E-01	N/A	12	rs7968902	64649337	5.94E-01	HMGA2	-	6.42E-07	3.29E-01	4.91E-04	9357	
3	rs9790156	1.41E+08	4.24E-01	CLSTN2	3	rs1332694	1.6E+08	4.06E-01	N/A	-	6.42E-07	6.34E-01	4.35E-04	9358	
4	rs10005964	77689324	7.97E-01	SHROOM3	15	rs1373470	84429110	5.00E-01	N/A	-	6.42E-07	6.63E-01	1.48E-05	9359	
12	rs10859807	93886698	1.39E-01	NDUFA12	20	rs6017995	44770445	6.23E-01	SLC2A10	-	6.42E-07	N/A	N/A	9360	
5	rs691360	18110132	6.01E-01	N/A	13	rs9316975	57661603	8.78E-01	N/A	-	6.42E-07	4.48E-02	1.14E-07	9361	
6	rs10484679	1.48E+08	4.97E-01	N/A	14	rs1243600	32600921	8.34E-01	NPAS3	-	6.42E-07	9.66E-01	8.37E-05	9362	
4	rs4690795	1.66E+08	1.12E-01	N/A	13	rs4771012	26188493	2.32E-01	N/A	-	6.42E-07	5.22E-01	6.85E-04	9363	
1	rs1925032	1.56E+08	5.23E-01	KIRREL	7	rs453	1.12E+08	6.05E-01	DOCK4	-	6.42E-07	8.54E-01	1.10E-03	9364	
2	rs4671178	54136997	9.21E-01	N/A	5	rs995360	1.13E+08	4.00E-01	N/A	-	6.42E-07	6.08E-01	3.04E-04	9365	
3	rs1550158	14381627	5.19E-02	N/A	5	rs257258	97396129	8.06E-01	N/A	-	6.42E-07	9.97E-01	9.73E-05	9366	
1	rs12036282	2.2E+08	1.25E-01	N/A	9	rs1096254	16709445	6.36E-01	BNC2	-	6.42E-07	4.20E-02	2.69E-02	9367	
2	rs4664403	1.62E+08	9.28E-01	N/A	11	rs7483643	91326575	2.78E-01	N/A	-	6.43E-07	2.82E-01	4.94E-06	9368	
3	rs2613946	1.14E+08	6.20E-02	N/A	5	rs7710003	1.15E+08	6.21E-01	LVRN	-	6.43E-07	4.73E-01	3.72E-05	9369	
1	rs867921	1.55E+08	4.75E-01	RHBG	2	rs1703921	49810189	9.92E-01	N/A	-	6.43E-07	9.70E-01	3.95E-04	9370	
1	rs2684866	1.61E+08	7.02E-01	DDR2	11	rs7127924	44042263	4.97E-01	ACCS	-	6.43E-07	2.36E-01	7.62E-06	9371	
11	rs2632051	19485215	4.09E-01	N/AV2	18	rs1031287	18600005	1.85E-01	N/A	-	6.43E-07	7.00E-01	2.50E-04	9372	
11	rs2632051	19485215	4.09E-01	N/AV2	18	rs1072782	18611634	1.85E-01	N/A	-	6.43E-07	7.00E-01	2.50E-04	9373	
6	rs2517552	31115569	8.54E-01	HCG22	22	rs2071762	43636988	5.14E-01	PHF21B	MHC	6.43E-07	7.03E-01	1.84E-04	9374	
12	rs11063723	5489075	8.88E-02	NTF3	16	rs8045798	12601561	5.26E-01	N/A	-	6.43E-07	9.02E-01	2.66E-04	9375	
3	rs11712058	31921677	4.43E-01	N/A	5	rs4869637	36311907	5.44E-02	RANBP3L	-	6.43E-07	3.24E-02	2.13E-07	9376	
4	rs7690570	37329901	7.09E-01	RELL1	4	rs6532966	1.03E+08	8.73E-01	N/A	-	6.43E-07	9.99E-01	2.11E-04	9377	
6	rs569919	1.61E+08	9.54E-02	SLC22A3	22	rs196067	37183623	7.63E-01	KDELR3	-	6.43E-07	2.74E-01	1.29E-03	9378	
5	rs32209	1.28E+08	4.32E-01	FBN2	10	rs1413582	1.21E+08	5.08E-01	GRK5	-	6.43E-07	6.05E-01	7.53E-04	9379	
5	rs39937	1.28E+08	4.32E-01	FBN2	10	rs1413582	1.21E+08	5.08E-01	GRK5	-	6.43E-07	6.05E-01	7.53E-04	9380	
2	rs4669725	11448167	6.07E-01	N/A	10	rs1090410	3862876	9.86E-01	N/A	-	6.43E-07	8.45E-01	1.90E-05	9381	
2	rs6711866	2.02E+08	8.77E-01	MPP4	15	rs3794586	31662556	5.27E-01	RYR3	-	6.43E-07	8.67E-01	1.33E-04	9382	
6	rs11754106	1.58E+08	6.75E-02	N/A	17	rs1051504	49047083	5.76E-01	N/A	-	6.43E-07	5.86E-01	6.03E-04	9383	
18	rs8088944	32512127	2.57E-01	FHOD3	20	rs1015401	35723550	2.43E-01	N/A	-	6.43E-07	1.99E-01	2.67E-03	9384	
1	rs405731	54806170	1.66E-01	ACOT11	12	rs4762096	64114263	1.79E-01	N/A	-	6.43E-07	2.06E-01	5.64E-06	9385	
1	rs6665760	1.55E+08	4.55E-01	RHBG	10	rs1360246	1.1E+08	2.70E-01	N/A	-	6.43E-07	2.26E-01	5.39E-06	9386	
1	rs559798	2.43E+08	9.42E-01	C1orf100	11	rs1103742	43336082	5.52E-01	TTC17	-	6.43E-07	9.35E-01	7.75E-05	9387	
6	rs12530461	559353	9.75E-01	EXOC2	14	rs3742345	94945095	6.36E-01	C14orf139	-	6.43E-07	3.97E-02	1.29E-02	9388	
6	rs17755831	548377	9.75E-01	EXOC2	14	rs3742345	94945095	6.36E-01	C14orf139	-	6.43E-07	3.97E-02	1.29E-02	9389	
6	rs2753212	83052893	2.32E-01	N/A	3	rs1153606	25549190	3.57E-01	RARB	-	6.43E-07	1.95E-01	4.02E-06	9390	
3	rs4130493	87896144	8.19E-02	N/A	3	rs4912486	1.86E+08	1.13E-01	CHRD	-	6.43E-07	7.99E-01	8.78E-05	9391	
5	rs10057387	1.72E+08	3.26E-01	N/A	19	rs2111505	37471459	5.00E-02	N/A	-	6.44E-07	6.75E-03	3.92E-08	9392	
15	rs813299	29225595	8.06E-01	N/A	17	rs2671822	30621795	1.79E-01	SLFN5	-	6.44E-07	1.99E-01	1.21E-02	9393	
3	rs776105	77825099	8.41E-01	N/A	12	rs2335817	3164497	6.41E-01	TSPAN9	-	6.44E-07	7.32E-01	1.62E-04	9394	
3	rs1997421	77823644	8.41E-01	N/A	12	rs2335817	3164497	6.41E-01	TSPAN9	-	6.44E-07	N/A	N/A	9395	
4	rs6848033	1.55E+08	8.56E-01	KIAA0922	20	rs2626552	31848505	9.49E-01	ZNF341	-	6.44E-07	1.13E-01	1.39E-03	9396	
5	rs12659680	1.52E+08	4.37E-02	N/A	20	rs1151924	15505228	6.57E-01	MACROD2	-	6.44E-07	9.56E-01	1.42E-04	9397	
14	rs1299512	32653876	2.73E-01	NPAS3	17	rs350615	66067303	9.25E-01	N/A	-	6.44E-07	1.34E-01	3.21E-03	9398	
1	rs11161943	87427194	7.56E-01	LOC339524	16	rs7359414	302639	5.67E-01	AXIN1	-	6.44E-07	1.51E-01	6.01E-04	9399	
8	rs6998301	1.3E+08	5.69E-01	N/A	19	rs8112582	8148511	4.19E-01	N/A	-	6.44E-07	6.46E-01	1.17E-04	9400	
8	rs7841408	1.29E+08	5.69E-01	N/A	19	rs8112582	8148511	4.19E-01	N/A	-	6.44E-07	7.03E-01	9.67E-05	9401	
8	rs11776972	1.29E+08	5.69E-01	N/A	19	rs8112582	8148511	4.19E-01	N/A	-	6.44E-07	N/A	N/A	9402	
15	rs7176207	52416467	8.77E-01	UNC13C	21	rs9975938	41011248	5.49E-01	DSCAM	-	6.44E-07	2.72E-02	2.33E-07	9403	
3	rs3813264	1.4E+08	3.16E-01	ESYT3	8	rs1268057	69541263	4.09E-01	N/A	-	6.44E-07	2.03E-01	8.91E-04	9404	
5	rs1989916	1.37E+08	3.49E-01	N/A	18	rs1296023	59404470	6.14E-01	SERPINB12	-	6.44E-07	3.22E-01	1.58E-05	9405	
1	rs11205801	51257214	3.00E-01	N/A	2	rs1153738	1.83E+08	6.59E-01	PPP1R1C	-	6.44E-07	7.06E-01	8.35E-05	9406	

SNP A					SNP B						Interaction P			Ranking	Cluster in top 100 interactions
CHR	SNP	Location	gle locus P va	Gene	CHR	SNP	Location	ingle locus P valu	Gene	HC region	Stage 1	Stage 2	Combined		
6	rs9498131	1.49E+08	4.50E-01	N/A	9	rs2798060	92068763	9.20E-01	N/A	-	6.44E-07	6.56E-01	1.49E-04	9407	
11	rs790343	83282554	3.77E-02	DLG2	18	rs587742	63687584	7.16E-01	N/A	-	6.44E-07	6.88E-01	8.34E-06	9408	
12	rs2731032	43763341	5.52E-01	RACGAP1P	17	rs11652075	75793488	7.82E-01	CARD14	-	6.44E-07	9.03E-01	5.22E-05	9409	
6	rs208436	66954191	9.87E-01	N/A	1	rs12408956	41424539	6.56E-01	SCMH1	-	6.44E-07	N/A	N/A	9410	
3	rs10865655	70940116	2.58E-01	N/A	20	rs6070406	56292157	3.07E-01	PPP4R1L	-	6.45E-07	9.95E-01	5.47E-05	9411	
8	rs2720770	3617832	2.50E-01	CSMD1	21	rs2254505	17006156	1.80E-01	N/A	-	6.45E-07	6.58E-01	1.14E-03	9412	
1	rs10493113	42931097	6.74E-01	YBX1	10	rs2420801	92904402	7.74E-01	NUDT9P1	-	6.45E-07	7.15E-01	1.89E-05	9413	
3	rs11918416	63480231	6.21E-01	SYNPR	7	rs6948429	89247424	9.77E-01	N/A	-	6.45E-07	3.30E-02	1.57E-07	9414	
6	rs7762850	1.65E+08	6.55E-03	N/A	15	rs3848117	55923241	5.53E-01	N/A	-	6.45E-07	5.57E-01	8.13E-04	9415	
5	rs465560	4321276	4.71E-01	N/A	12	rs4764755	99753004	8.38E-01	ANO4	-	6.45E-07	1.24E-01	8.85E-07	9416	
5	rs32209	1.28E+08	4.32E-01	FBN2	10	rs4752305	1.21E+08	4.17E-01	GRK5	-	6.45E-07	8.17E-01	3.64E-04	9417	
5	rs39937	1.28E+08	4.32E-01	FBN2	10	rs4752305	1.21E+08	4.17E-01	GRK5	-	6.45E-07	8.17E-01	3.64E-04	9418	
5	rs7707487	1.29E+08	9.04E-01	ADAMTS19	15	rs2307449	87664932	4.79E-01	FANCI	-	6.45E-07	N/A	N/A	9419	
3	rs13085838	64625627	3.84E-02	ADAMTS9	16	rs6497755	24694696	4.03E-01	TNRC6A	-	6.45E-07	3.26E-02	3.43E-07	9420	
12	rs550434	71157865	4.27E-01	TRHDE	16	rs7197204	55779366	2.15E-01	FAM192A	-	6.45E-07	8.76E-01	9.45E-05	9421	
2	rs4952404	40414134	1.51E-01	SLC8A1	15	rs2118784	49238477	9.91E-01	N/A	-	6.45E-07	7.28E-02	1.50E-06	9422	
3	rs4855413	69683544	8.73E-01	N/A	16	rs8059582	12687941	9.97E-01	CPPED1	-	6.45E-07	3.90E-01	1.22E-05	9423	
13	rs953937	89320971	6.27E-01	N/A	14	rs2998303	84071041	1.50E-01	N/A	-	6.45E-07	3.14E-02	2.57E-07	9424	
2	rs7349387	1.41E+08	7.60E-01	LRP1B	21	rs1009123	39129666	4.85E-01	ETS2	-	6.45E-07	2.24E-01	3.05E-03	9425	
11	rs905770	65913341	3.92E-01	SLC29A2	11	rs664499	75565334	3.38E-01	WNT11	-	6.45E-07	5.54E-01	5.89E-05	9426	
10	rs2607840	87960202	3.48E-01	GRID1	13	rs9583760	89442220	8.24E-01	N/A	-	6.45E-07	7.28E-01	1.12E-04	9427	
6	rs161070	1.44E+08	2.14E-01	PEX3	2	rs13408281	1.71E+08	6.09E-01	UBR3	-	6.45E-07	6.60E-01	9.13E-05	9428	
2	rs6716242	94823140	2.70E-01	ANKRD20B	14	rs1950967	93333889	4.16E-01	PRIMA1	-	6.45E-07	7.40E-01	3.35E-04	9429	
6	rs12524492	1.23E+08	9.39E-01	PKIB	4	rs3899492	1.81E+08	9.31E-01	N/A	-	6.45E-07	9.83E-01	6.84E-05	9430	
6	rs7743671	1.23E+08	9.39E-01	PKIB	4	rs3899492	1.81E+08	9.31E-01	N/A	-	6.45E-07	9.83E-01	6.84E-05	9431	
9	rs7860062	84736837	8.07E-01	N/A	9	rs12685781	91801450	5.89E-01	N/A	-	6.45E-07	1.06E-01	2.81E-06	9432	
9	rs1327999	14542236	3.07E-01	N/A	19	rs2109069	4670443	4.31E-01	DPP9	-	6.45E-07	5.08E-01	2.85E-04	9433	
10	rs1831474	20076086	7.38E-01	N/A	17	rs442548	71884974	9.68E-01	UBE2O	-	6.45E-07	6.53E-01	4.78E-04	9434	
3	rs3856913	1.89E+08	7.12E-01	N/A	22	rs13055229	26149250	1.13E-01	N/A	-	6.46E-07	2.86E-01	8.37E-04	9435	
3	rs7614825	1.89E+08	7.12E-01	N/A	22	rs13055229	26149250	1.13E-01	N/A	-	6.46E-07	2.86E-01	8.37E-04	9436	
18	rs9964724	33413122	1.07E-01	BRUNOL4	19	rs12232877	24095263	4.11E-01	ZNF254	-	6.46E-07	6.55E-01	2.16E-03	9437	
7	rs12706912	80090231	2.59E-01	CD36	13	rs9316153	45185022	8.65E-01	SPERT	-	6.46E-07	8.83E-01	2.14E-05	9438	
7	rs17519442	11638659	1.37E-01	THSD7A	8	rs4738265	73606130	6.59E-01	KCNB2	-	6.46E-07	8.98E-01	5.03E-05	9439	
14	rs4272953	48540230	9.05E-01	N/A	16	rs930743	79273633	5.97E-01	CDYL2	-	6.46E-07	2.84E-01	3.69E-06	9440	
6	rs1873249	34161402	4.37E-01	GRM4	8	rs7463093	94001877	8.97E-01	N/A	MHC	6.46E-07	3.40E-01	5.68E-06	9441	
11	rs1058678	62117613	6.87E-01	EML3	15	rs11633650	31251260	2.07E-01	N/A	-	6.46E-07	N/A	N/A	9442	
2	rs2001427	22951846	4.97E-01	N/A	10	rs1997974	83243624	3.19E-01	N/A	-	6.46E-07	N/A	N/A	9443	
2	rs2311552	1.12E+08	3.74E-01	N/A	11	rs11823466	23578823	5.32E-01	N/A	-	6.46E-07	3.91E-01	1.11E-03	9444	
11	rs3751084	67540854	4.51E-01	UNC93B1	15	rs11631211	23412204	8.68E-01	N/A	-	6.46E-07	8.86E-01	1.07E-04	9445	
4	rs2323262	21753534	1.58E-01	N/A	9	rs7034424	88764722	6.56E-01	GAS1	-	6.46E-07	4.87E-01	7.83E-04	9446	
5	rs1600178	51894999	9.08E-01	N/A	11	rs2625510	83418247	6.12E-01	DLG2	-	6.46E-07	5.36E-01	5.49E-04	9447	
9	rs7045243	86024539	1.01E-01	N/A	13	rs6490697	21577011	1.52E-02	N/A	-	6.46E-07	9.10E-01	1.45E-04	9448	
18	rs3745013	2905219	4.57E-01	EMILIN2	20	rs6015003	55465368	6.77E-01	N/A	-	6.46E-07	N/A	N/A	9449	
15	rs507736	27504337	3.34E-02	FAM189A1	20	rs4814966	2022922	4.53E-01	STK35	-	6.46E-07	7.03E-01	1.29E-04	9450	
7	rs6463699	7516181	2.02E-01	COL28A1	10	rs2184352	1.22E+08	6.00E-01	N/A	-	6.46E-07	2.33E-01	8.95E-06	9451	
3	rs6845	1.86E+08	8.15E-01	PSMD2	14	rs2295136	88004455	1.52E-01	PTPN21	-	6.46E-07	9.26E-01	2.99E-05	9452	
2	rs3770725	64668269	2.88E-01	N/A	3	rs826385	24162088	3.25E-01	THRB	-	6.46E-07	4.53E-01	9.12E-06	9453	
4	rs2702383	1.8E+08	3.58E-01	N/A	9	rs10984491	1.21E+08	8.71E-02	DBC1	-	6.46E-07	5.89E-01	1.39E-05	9454	
4	rs6820131	23835016	1.96E-01	N/A	15	rs4337270	84343916	2.04E-01	N/A	-	6.46E-07	5.16E-01	4.44E-04	9455	
3	rs1161149	8462491	3.62E-01	N/A	13	rs3015345	1.05E+08	7.02E-01	N/A	-	6.47E-07	9.68E-01	1.17E-04	9456	
9	rs1360523	25489528	7.55E-01	N/A	16	rs899305	7140564	2.91E-01	A2BP1	-	6.47E-07	2.17E-03	1.52E-08	9457	
1	rs4651010	1.77E+08	9.70E-01	N/A	9	rs2245644	76514071	4.30E-01	TRPM6	-	6.47E-07	N/A	N/A	9458	
6	rs1856269	1.46E+08	9.10E-02	N/A	7	rs6967748	45105688	1.27E-01	N/ACAD	-	6.47E-07	8.58E-01	7.31E-05	9459	
8	rs270065	3273722	3.89E-01	CSMD1	22	rs761419	46324898	1.70E-01	N/A	-	6.47E-07	8.01E-01	1.83E-04	9460	
1	rs7522521	81651526	3.22E-01	N/A	2	rs7575607	31493959	3.12E-01	XDH	-	6.47E-07	3.46E-01	1.61E-05	9461	
1	rs16825336	1735586	6.09E-01	GNB1	5	rs2278662	1.79E+08	2.77E-01	RASGEF1C	-	6.47E-07	5.75E-01	2.47E-04	9462	
3	rs10865655	70940116	2.58E-01	N/A	20	rs6128300	56294445	5.34E-01	PPP4R1L	-	6.47E-07	7.37E-01	8.51E-05	9463	
3	rs10865655	70940116	2.58E-01	N/A	20	rs6128302	56299786	5.34E-01	RAB22A	-	6.47E-07	7.37E-01	8.51E-05	9464	
2	rs10191556	2.42E+08	2.87E-01	THAP4	4	rs7665811	76852349	6.59E-01	USO1	-	6.47E-07	2.72E-01	1.09E-05	9465	
3	rs12638861	56818068	8.98E-01	ARHGEF3	9	rs1535753	85094880	1.39E-01	FRMD3	-	6.47E-07	9.76E-01	3.36E-04	9466	
6	rs879882	31247431	7.61E-01	TCF19	6	rs9368675	31380136	7.91E-01	N/A	MHC	6.47E-07	4.97E-02	6.47E-07	9467	
19	rs1824794	22881436	2.70E-01	N/A	21	rs2836749	39209108	5.72E-01	N/A	-	6.47E-07	3.18E-01	7.05E-06	9468	
11	rs4556569	87116245	2.68E-01	N/A	14	rs12882560	40546007	7.05E-01	N/A	-	6.47E-07	8.34E-01	3.54E-05	9469	
9	rs10810285	14881670	3.19E-01	FREM1	17	rs317345	29208355	2.10E-01	ACCN1	-	6.48E-07	4.94E-01	1.05E-03	9470	
13	rs6490720	21780871	5.41E-01	N/A	13	rs17069892	77919317	2.91E-01	N/A	-	6.48E-07	6.16E-01	3.11E-05	9471	
5	rs2112564	1.29E+08	2.09E-01	N/A	9	rs3920797	1206635	2.77E-01	N/A	-	6.48E-07	N/A	N/A	9472	
5	rs10719	31437204	3.58E-01	RN/ASEN	20	rs6038995	7907777	3.98E-01	TMX4	-	6.48E-07	1.67E-01	1.64E-03	9473	
2	rs11123997	1.05E+08	7.12E-01	N/A	7	rs7803999	48427272	8.62E-04	ABCA13	-	6.48E-07	8.50E-01	2.51E-04	9474	
1	rs12126587	18350856	1.88E-01	IGSF21	16	rs12051478	20871819	1.53E-01	DN/AH3	-	6.48E-07	7.44E-01	9.61E-05	9475	
1	rs12126587	18350856	1.88E-01	IGSF21	16	rs3743696	20882570	1.53E-01	DN/AH3	-	6.48E-07	7.44E-01	9.61E-05	9476	
1	rs9727014	18348774	1.88E-01	IGSF21	16	rs12051478	20871819	1.53E-01	DN/AH3	-	6.48E-07	7.44E-01	9.61E-05	9477	
1	rs9727014	18348774	1.88E-01	IGSF21	16	rs3743696	20882570	1.53E-01	DN/AH3	-	6.48E-07	7.44E-01	9.61E-05	9478	
6	rs2752989	63468600	7.85E-02	N/A	8	rs12548301	62130234	7.87E-01	N/A	-	6.48E-07	7.64E-01	1.50E-04	9479	
4	rs1036016	1.47E+08	9.90E-01	SLC10A7	7	rs1362178	1.26E+08	8.96E-01	GRM8	-	6.48E-07	1.42E-01	1.98E-03	9480	
21	rs2236483	45750482	7.95E-01	COL18A1	22	rs2001121	25448181	5.25E-01	N/A	-	6.48E-07	4.48E-01	3.50E-04	9481	
12	rs10847417	1.27E+08	9.96E-01	N/A	17	rs8078613	210420	7.59E-02	N/A	-	6.48E-07	1.84E-01	4.51E-03	9482	
16	rs251796	67952935	5.20E-01	NIP7	16	rs1518604	78876091	4.19E-01	N/A	-	6.48E-07	3.70E-01	7.17E-04	9483	
2	rs12471723	2.19E+08	7.72E-02	IL8RBP	13	rs17065993	75757088	6.11E-01	N/A	-	6.48E-07	8.39E-01	4.90E-05	9484	
1	rs11119208	2.07E+08	3.46E-01	N/A	11	rs10750473	1.31E+08	6.72E-01	NTM	-	6.48E-07	4.05E-02	5.18E-03	9485	
1	rs3789375	94237720	1.08E-01	ABCA4	20	rs6057638	30802993	1.90E-02	COMMD7	-	6.48E-07	1.72E-01	1.88E-03	9486	
6	rs7453029	92424364	9.93E-01	N/A	2	rs16856834	10947834	4.30E-01	N/A	-	6.48E-07	2.31E-01	1.23E-05	9487	
6	rs7453029	92424364	9.93E-01	N/A	2	rs6725242	10946166	4.30E-01	N/A	-	6.48E-07	2.91E-01	1.86E-05	9488	
18	rs9956612	8989233	8.07E-01	N/A	20	rs980984	43766466	9.79E-01	WFDC10B	-	6.48E-07	8.60E-01	6.03E-04	9489	
6	rs9294333	86086103	7.16E-01	N/A	9	rs11265909	91267966	3.52E-01	SEMA4D	-	6.49E-07	6.41E-01	2.53E-04	9490	
3	rs3849514	1.04E+08	7.31E-01	N/A	4	rs11942384	21124180	3.94E-02	KCNIP4	-	6.49E-07	4.50E-02	4.07E-03	9491	
6	rs1580731	19481304	1.24E-01	N/A	13	rs12429969	41017842	5.95E-02	N/A	-	6.49E-07	4.29E-01	1.58E-05	9492	
6	rs499915	86061346	8.57E-01	N/A	9	rs11265909	91267966	3.52E-01	SEMA4D	-	6.49E-07	7.53E-01	1.83E-04	9493	
1	rs12411052	1.11E+08	6.46E-02	KCN/A2	9	rs10982124	1.16E+08	7.46E-01	COL27A1	-	6.49E-07	9.45E-01	2.00E-04	9494	
17	rs218693	6532476	1.92E-01	SLC13A5	18	rs8095324	22385657	5.44E-01	KCTD1	-	6.49E-07	5.71E-01	2.93E-05	9495	
4	rs11249478	70969062	8.34E-01	CSN1S2A	10	rs2619111	1.19E+08	6.03E-01	KCNK18	-	6.49E-07	7.51E-01	1.06E-05	9496	
4	rs4694267	70986395	8.34E-01	CSN1S2A	10	rs2619111	1.19E+08	6.03E-01	KCNK18	-	6.49E-07	7.51E-01	1.06E-05	9497	
1	rs1113239	1.92E+08	4.39E-01	N/A	22	rs11090762	46133989	5.61E-01	N/A	-	6.49E-07	8.56E-02	8.82E-07	9498	
1	rs1410397	1.92E+08	4.39E-01	N/A	22	rs11090762	46133989	5.61E-01	N/A	-	6.49E-07	9.65E-02	1.09E-06	9499	
13	rs1446385	68692163	5.55E-01	N/A	20	rs6017335	42444239	6.63E-01	HNF4A	-	6.49E-07	8.36E-01	2.18E-04	9500	
3	rs4686399	1.87E+08	4.19E-02	TRA2B	7	rs302127	83169251	2.49E-01	N/A	-	6.49E-07	8.89E-01	5.67E-05	9501	
4	rs1585428	93307687	8.02E-01	N/A	15	rs16967121	36710299	6.81E-01	N/A	-	6.49E-07	9.91E-01	1.17E-04	9502	
8	rs10504093	51028383	6.53E-01	SNTG1	20	rs6025422	55183188	3.01E-01	BMP7	-	6.49E-07	7.63E-02	5.99E-03	9503	
3	rs2060022	65974795	3.63E-01	MAGI1	3	rs11721056	1.48E+08	6.49E-01	N/A	-	6.49E-07	1.57E-01	1.59E-03	9504	
3	rs6767345	65975145	3.63E-01	MAGI1	3	rs11721056	1.48E+08	6.49E-01	N/A	-	6.49E-07	1.86E-01	1.29E-03	9505	
1	rs1175128	1.9E+08	4.58E-01	N/A	12	rs17091714	41380669	4.95E-01	N/A	-	6.49E-07	7.27E-01	8.83E-06	9506	
7	rs7808376	1.35E+08	6.57E-01	N/A	13	rs706598	50067538	6.78E-01	N/A	-	6.49E-07	9.51E-01	9.11E-05	9507	
8	rs12541274	5485819	3.04E-01	N/A	15	rs10519060	44632813	7.50E-01	N/A	-	6.49E-07	4.24E-02	2.72E-03	9508	
4	rs6448254	23864940	8.48E-02	N/A	18	rs7237881	2624943	7.01E-01	NDC80	-	6.49E-07	1.68E-01	8.76E-06	9509	
3	rs6550923	24899499	4.53E-01	N/A	7	rs1534573	84393275	1.76E-01	N/A	-	6.49E-07	5.11E-01	8.51E-05	9510	
1	rs614486	46911406	6.00E-01	ATPAF1	3	rs2291862	4817231	4.64E-01	ITPR1	-	6.49E-07	7.17E-01	3.18E-04	9511	
1	rs647999	46889144	6.00E-01	ATPAF1	3	rs2291862	4817231	4.64E-01	ITPR1	-	6.49E-07	7.34E-01	2.97E-04	9512	
7	rs17159305	1.12E+08	4.85E-01	DOCK4	11	rs11828539	84304563	5.25E-01	DLG2	-	6.49E-07	1.52E-01	3.13E-03	9513	
4	rs7677282	53276822	6.33E-01	SNORA26	17	rs11871090	72573388	2.93E-01	N/A	-	6.50E-07	1.23E-02	7.80E-08	9514	
7	rs7803276	77933808	6.10E-02	MAGI2	16	rs8057212	79437862	2.41E-01	N/A	-	6.50E-07	2.15E-01	1.14E-03	9515	
8	rs11787055	41537292	8.18E-01	GINS4	11	rs1793594	1.31E+08	7.70E-01	N/A	-	6.50E-07	2.72E-01	6.21E-04	9516	
8	rs7002916	41536109	8.18E-01	GINS4	11	rs1793594	1.31E+08	7.70E-01	N/A	-	6.50E-07	2.72E-01	6.21E-04	9517	
13	rs12428170	28069890	8.76E-02	N/A	19	rs7246479	60516144	9.84E-01	TMEM150B	-	6.50E-07	8.29E-01	2.72E-05	9518	

SNP A					SNP B						Interaction P			Ranking	Cluster in top 100 interactions
CHR	SNP	Location	gle locus P va	Gene	CHR	SNP	Location	Single locus P valu	Gene	HC region	Stage 1	Stage 2	Combined		
10	rs11259537	15275960	5.69E-01	FAM171A1	11	rs1939469	75913868	3.64E-01	C11orf30	-	6.50E-07	1.72E-01	1.07E-03	9519	
2	rs1250226	2.16E+08	5.73E-01	N/A	3	rs6797616	81313323	7.54E-02	N/A	-	6.50E-07	7.59E-02	6.48E-07	9520	
7	rs3808035	1.03E+08	8.82E-03	RELN	22	rs4822832	25717402	1.00E-02	N/A	-	6.50E-07	3.30E-01	4.41E-03	9521	
4	rs1352573	1.19E+08	1.53E-01	N/A	17	rs180750	12303225	3.31E-01	N/A	-	6.50E-07	7.92E-01	3.01E-05	9522	
11	rs12273302	19208280	8.61E-01	E2F8	16	rs4780486	9532033	1.05E-03	N/A	-	6.50E-07	7.98E-01	2.18E-04	9523	
2	rs13395807	2.29E+08	4.86E-01	N/A	11	rs836132	34511767	6.81E-01	ELF5	-	6.50E-07	6.66E-02	2.90E-07	9524	
1	rs2296452	78199071	8.61E-01	N/A	5	rs6860842	77775106	6.76E-01	SCAMP1	-	6.50E-07	5.48E-01	1.91E-04	9525	
6	rs6906699	19952334	1.52E-01	ID4	13	rs9524516	93940835	9.12E-01	DCT	-	6.50E-07	9.30E-01	5.05E-05	9526	
9	rs11142066	89849415	6.41E-01	N/A	12	rs7975710	26425333	8.50E-01	ITPR2	-	6.50E-07	5.11E-02	8.74E-07	9527	
1	rs1338078	1.12E+08	7.84E-01	N/A	3	rs1865160	510673	5.17E-01	N/A	-	6.50E-07	9.78E-01	8.66E-05	9528	
17	rs10459889	66453810	1.59E-01	N/A	19	rs408687	22022963	6.99E-01	ZNF257	-	6.50E-07	3.67E-01	6.75E-04	9529	
17	rs10459889	66453810	1.59E-01	N/A	19	rs409835	22022511	6.99E-01	ZNF257	-	6.50E-07	3.67E-01	6.75E-04	9530	
17	rs10459889	66453810	1.59E-01	N/A	19	rs411020	22022942	6.99E-01	ZNF257	-	6.50E-07	3.67E-01	6.75E-04	9531	
2	rs12614708	22421743	8.59E-01	N/A	3	rs13080704	1.1E+08	2.95E-01	TRAT1	-	6.50E-07	5.57E-01	2.54E-05	9532	
4	rs17021755	1.48E+08	9.94E-02	POU4F2	20	rs6125931	48228820	2.44E-01	CEBPB	-	6.51E-07	4.44E-02	3.35E-03	9533	
14	rs17124954	88440873	6.70E-01	N/A	16	rs10500558	70109022	7.80E-02	CHST4	-	6.51E-07	6.55E-02	3.83E-03	9534	
14	rs17124954	88440873	6.70E-01	N/A	16	rs7202662	70102989	7.80E-02	CHST4	-	6.51E-07	6.55E-02	3.83E-03	9535	
1	rs10493425	67782715	4.50E-01	N/A	12	rs1471997	47009862	8.07E-01	N/A	-	6.51E-07	7.78E-02	4.05E-03	9536	
2	rs7584904	1.05E+08	8.67E-01	N/A	20	rs2423967	15529484	1.83E-01	MACROD2	-	6.51E-07	3.25E-01	3.58E-05	9537	
6	rs2246016	93321027	4.12E-01	N/A	2	rs10173035	1.68E+08	3.08E-02	N/A	-	6.51E-07	5.20E-01	1.02E-03	9538	
7	rs1637837	1.48E+08	9.21E-01	CNTN/AP2	10	rs7901838	66928346	6.98E-01	N/A	-	6.51E-07	6.16E-01	1.66E-04	9539	
7	rs1637837	1.48E+08	9.21E-01	CNTN/AP2	10	rs10822576	66950838	6.98E-01	N/A	-	6.51E-07	6.62E-01	1.41E-04	9540	
7	rs1637837	1.48E+08	9.21E-01	CNTN/AP2	10	rs7099767	66952127	6.98E-01	N/A	-	6.51E-07	6.62E-01	1.41E-04	9541	
10	rs9423582	5291811	5.86E-01	N/A	20	rs6068993	52874904	1.22E-01	N/A	-	6.51E-07	9.17E-01	8.04E-05	9542	
1	rs807247	25993456	5.33E-01	MAN1C1	11	rs10791545	1.01E+08	4.47E-01	KIAA1377	-	6.51E-07	3.47E-01	2.87E-05	9543	
1	rs807247	25993456	5.33E-01	MAN1C1	11	rs2119734	1.01E+08	4.47E-01	KIAA1377	-	6.51E-07	3.47E-01	2.87E-05	9544	
14	rs7160845	76107877	1.14E-02	N/A	16	rs415595	11271193	5.49E-01	PRM3	-	6.51E-07	2.86E-01	9.54E-05	9545	
2	rs2578007	1.83E+08	4.69E-01	N/A	12	rs1846035	82545653	9.72E-01	N/A	-	6.51E-07	5.33E-01	4.68E-05	9546	
5	rs983280	59480894	4.55E-01	PDE4D	17	rs3785902	65619713	5.26E-02	KCNJ16	-	6.51E-07	5.69E-01	1.49E-04	9547	
3	rs769798	3750107	3.64E-01	N/A	16	rs280858	76155263	4.92E-01	N/A	-	6.51E-07	7.70E-01	5.23E-05	9548	
3	rs12629639	1.55E+08	2.97E-01	N/A	13	rs1431270	1.09E+08	9.33E-01	MYO16	-	6.51E-07	9.29E-01	1.71E-04	9549	
5	rs10045947	1.79E+08	1.93E-01	RNF130	9	rs1888171	27677159	6.23E-01	N/A	-	6.52E-07	6.54E-01	9.46E-05	9550	
11	rs1790158	74858897	1.10E-01	GDPD5	16	rs7198127	79758130	9.90E-01	PKD1L2	-	6.52E-07	9.67E-01	3.29E-04	9551	
16	rs1465350	54235278	7.60E-01	N/A	17	rs8069706	23346964	2.84E-01	N/A	-	6.52E-07	7.03E-01	4.35E-05	9552	
11	rs475688	64120867	8.12E-01	NRXN2	12	rs1470803	43281923	3.70E-01	NELL2	-	6.52E-07	2.03E-01	1.42E-03	9553	
4	rs11131397	63282780	8.65E-01	N/A	12	rs11063667	5403395	8.03E-03	NTF3	-	6.52E-07	2.22E-01	6.94E-06	9554	
10	rs1143870	9333647	6.47E-01	N/A	20	rs6021231	49525694	8.13E-01	NFATC2	-	6.52E-07	1.68E-02	9.24E-03	9555	
7	rs150607	28534544	2.11E-01	CREB5	17	rs4790738	4915716	3.88E-01	ZFP3	-	6.52E-07	5.65E-01	3.92E-05	9556	
3	rs9857030	4611821	8.94E-01	ITPR1	4	rs2911890	37472706	6.97E-01	N/A	-	6.52E-07	6.12E-01	8.61E-05	9557	
2	rs908733	1.82E+08	1.44E-01	N/A	14	rs12883541	96638001	7.85E-01	N/A	-	6.52E-07	9.89E-01	1.38E-04	9558	
1	rs7514281	1.88E+08	4.90E-01	N/A	4	rs10017989	1.89E+08	4.97E-01	N/A	-	6.52E-07	2.64E-01	9.57E-04	9559	
2	rs7575596	2.35E+08	2.26E-01	N/A	11	rs6578604	5299185	2.00E-01	OR51B2	-	6.52E-07	2.72E-01	1.72E-03	9560	
2	rs10189097	1.76E+08	1.01E-01	N/A	8	rs916551	17998578	1.48E-02	ASAH1	-	6.52E-07	8.61E-01	4.56E-04	9561	
2	rs13397558	1.76E+08	1.01E-01	N/A	8	rs916551	17998578	1.48E-02	ASAH1	-	6.52E-07	8.61E-01	4.56E-04	9562	
2	rs2551214	2335000	2.21E-01	N/A	3	rs12497339	45198645	1.89E-01	N/A	-	6.52E-07	9.63E-01	1.94E-04	9563	
12	rs11047574	24736444	7.80E-01	N/A	13	rs7999638	89274415	8.79E-01	N/A	-	6.52E-07	4.30E-01	5.47E-04	9564	
3	rs2918214	1.17E+08	7.48E-01	LSAMP	17	rs1380796	74696514	5.44E-01	HRNBP3	-	6.52E-07	9.09E-01	5.14E-04	9565	
2	rs7559746	2.27E+08	8.46E-01	N/A	13	rs2036717	75645031	4.28E-01	N/A	-	6.52E-07	2.23E-01	2.19E-03	9566	
8	rs12679072	57728418	8.23E-01	N/A	15	rs2243932	59770219	5.70E-01	N/A	-	6.53E-07	9.89E-01	6.59E-05	9567	
3	rs7625682	29466227	4.31E-01	N/A	11	rs4468331	3121988	3.10E-01	OSBPL5	-	6.53E-07	2.18E-02	2.01E-07	9568	
6	rs1951358	1.09E+08	2.23E-01	ARMC2	2	rs2032953	2.32E+08	3.56E-01	N/A	-	6.53E-07	1.58E-01	6.53E-06	9569	
1	rs12089317	22625714	4.12E-01	N/A	11	rs6589006	1.08E+08	4.79E-01	ACAT1	-	6.53E-07	7.63E-01	1.63E-04	9570	
4	rs7698564	48157924	7.79E-01	N/A	16	rs7192553	81674884	9.75E-02	CDH13	-	6.53E-07	1.86E-01	2.65E-06	9571	
8	rs10504444	70527562	4.00E-01	SULF1	9	rs10125663	1.17E+08	2.63E-01	1-Dec	-	6.53E-07	2.78E-01	5.12E-04	9572	
11	rs7109004	20849339	6.14E-02	NELL1	12	rs1512735	84240895	9.74E-01	N/A	-	6.53E-07	3.99E-01	1.03E-03	9573	
1	rs11208380	64429559	8.74E-02	ROR1	15	rs2594935	25858633	1.48E-01	OCA2	-	6.53E-07	4.20E-01	1.41E-05	9574	
5	rs4869417	92570216	6.44E-01	N/A	17	rs12450242	74960720	6.25E-01	HRNBP3	-	6.53E-07	7.27E-01	1.80E-05	9575	
2	rs6724135	1.15E+08	3.24E-01	N/A	12	rs7134402	12988594	3.76E-01	GPRC5D	-	6.53E-07	7.59E-01	5.19E-04	9576	
4	rs4235040	82762778	1.30E-01	N/A	11	rs4586138	11955605	4.04E-02	USP47	-	6.53E-07	N/A	N/A	9577	
6	rs3213541	1.01E+08	3.35E-01	SIM1	7	rs886505	8752582	9.14E-01	NXPH1	-	6.53E-07	1.10E-01	2.91E-06	9578	
14	rs12147701	77599568	5.40E-01	N/A	18	rs3888654	3587864	4.11E-01	DLGAP1	-	6.53E-07	2.87E-01	9.48E-04	9579	
6	rs1062470	31192414	5.89E-01	C6orf15	6	rs7750269	31379136	8.58E-01	N/A	MHC	6.53E-07	6.20E-01	8.30E-04	9580	
2	rs4671787	67261861	9.61E-01	N/A	12	rs762785	75783898	1.00E-02	ZDHHC17	-	6.53E-07	6.06E-01	9.84E-04	9581	
2	rs4671787	67261861	9.61E-01	N/A	12	rs762786	75783639	1.00E-02	ZDHHC17	-	6.53E-07	6.06E-01	9.84E-04	9582	
7	rs12669548	14492752	9.82E-01	DGKB	11	rs1482219	97912888	6.17E-01	N/A	-	6.53E-07	2.11E-01	7.22E-04	9583	
3	rs2110084	45358858	4.25E-01	N/A	12	rs7304462	1.14E+08	8.14E-01	N/A	-	6.53E-07	7.26E-01	2.10E-04	9584	
11	rs7930843	1.09E+08	4.26E-01	ZC3H12C	20	rs6093346	38500329	7.60E-01	N/A	-	6.54E-07	8.47E-01	3.89E-05	9585	
2	rs1064767	2.42E+08	4.36E-01	HDLBP	11	rs1899506	27098252	5.50E-02	BBOX1	-	6.54E-07	2.74E-01	2.94E-03	9586	
6	rs4324759	1.66E+08	1.04E-01	C6orf118	5	rs10079374	17863895	8.53E-01	N/A	-	6.54E-07	7.52E-01	4.55E-05	9587	
2	rs10170117	1.62E+08	6.43E-01	N/A	20	rs2104351	14869539	1.03E-01	MACROD2	-	6.54E-07	2.12E-01	3.99E-03	9588	
3	rs2218746	28991443	3.16E-01	N/A	15	rs1568657	81517183	7.10E-01	BTBD1	-	6.54E-07	2.38E-01	9.70E-04	9589	
4	rs2980089	4318199	8.86E-01	LYAR	5	rs7734808	1.21E+08	7.93E-01	N/A	-	6.54E-07	9.58E-01	1.38E-04	9590	
2	rs7597923	77088361	7.21E-01	LRRTM4	5	rs597099	3192158	7.14E-01	N/A	-	6.54E-07	1.53E-01	2.71E-03	9591	
3	rs6804523	1.78E+08	5.85E-01	N/A	7	rs2108225	1.07E+08	5.36E-01	SLC26A3	-	6.54E-07	2.37E-01	5.37E-04	9592	
12	rs12579771	44019689	6.72E-01	ANO6	13	rs2119541	78514623	2.23E-01	N/A	-	6.54E-07	1.23E-01	8.45E-07	9593	
3	rs1978683	1.48E+08	7.98E-01	N/A	4	rs2055931	34528519	3.95E-01	N/A	-	6.54E-07	7.43E-01	3.00E-05	9594	
1	rs1337575	90711835	9.45E-01	N/A	10	rs965265	55757429	5.20E-01	PCDH15	-	6.54E-07	4.15E-01	5.33E-06	9595	
1	rs1337575	90711835	9.45E-01	N/A	10	rs1935468	55753731	5.39E-01	PCDH15	-	6.54E-07	4.15E-01	5.04E-06	9596	
12	rs1492332	39144721	1.67E-01	N/A	17	rs9908150	17054786	1.62E-01	PLD6	-	6.54E-07	8.20E-01	9.44E-05	9597	
6	rs2753162	1.25E+08	4.58E-01	NKAIN2	2	rs11691779	97533360	6.37E-01	ANKRD36B	-	6.54E-07	9.73E-01	1.03E-04	9598	
8	rs352775	15680133	6.86E-01	TUSC3	18	rs8093852	68855454	1.39E-01	N/A	-	6.54E-07	5.04E-01	9.47E-06	9599	
5	rs12654341	1.52E+08	3.75E-02	N/A	20	rs1151924	15505228	6.57E-01	MACROD2	-	6.54E-07	9.56E-01	1.44E-04	9600	
4	rs2168489	94066927	6.46E-01	GRID2	9	rs3931884	13792750	1.24E-01	N/A	-	6.55E-07	6.81E-02	2.65E-03	9601	
1	rs505877	1.64E+08	6.53E-01	UCK2	16	rs42270	12468247	6.78E-01	SNX29	-	6.55E-07	9.48E-01	3.38E-04	9602	
5	rs6871540	23896821	3.05E-02	N/A	10	rs4748247	16167564	5.20E-01	N/A	-	6.55E-07	6.59E-01	3.59E-04	9603	
6	rs1188852	1.39E+08	1.33E-01	ECT2L	7	rs13227951	65850543	3.33E-01	RABGEF1	-	6.55E-07	1.62E-01	4.64E-06	9604	
10	rs7076988	34433738	3.25E-01	N/A	18	rs8087457	23871623	3.56E-01	CDH2	-	6.55E-07	3.24E-01	1.88E-03	9605	
2	rs6543316	1.06E+08	3.04E-01	N/A	8	rs2458285	1.04E+08	1.60E-01	N/A	-	6.55E-07	8.86E-01	2.73E-05	9606	
9	rs3739618	3816081	8.83E-01	GLIS3	19	rs10404382	15162389	7.16E-01	NOTCH3	-	6.55E-07	1.68E-02	1.62E-02	9607	
8	rs6983129	11628545	7.12E-01	GATA4	8	rs17620722	16029553	9.43E-01	MSR1	-	6.55E-07	9.28E-01	7.13E-05	9608	
7	rs12703402	1.41E+08	5.51E-01	AGK	11	rs968845	39986201	6.50E-01	N/A	-	6.55E-07	9.49E-01	7.80E-05	9609	
10	rs11254765	6899850	9.50E-01	N/A	11	rs10790338	1.19E+08	4.12E-01	PVRL1	-	6.55E-07	6.39E-02	2.00E-02	9610	
12	rs2010932	1956243	6.35E-01	DCP1B	17	rs178890	26820930	5.32E-01	RAB11FIP4	-	6.55E-07	3.56E-01	1.14E-05	9611	
14	rs1959375	44021538	2.55E-01	N/A	14	rs1033769	95104103	3.18E-01	N/A	-	6.55E-07	5.19E-01	1.13E-04	9612	
8	rs2173117	11642539	6.92E-01	GATA4	14	rs10145908	62823082	4.33E-01	RHOJ	-	6.55E-07	8.10E-01	6.44E-04	9613	
6	rs2754822	1.09E+08	1.35E-02	ARMC2	4	rs11100443	1.64E+08	6.18E-01	N/A	-	6.56E-07	1.81E-01	2.70E-06	9614	
1	rs3738055	89199490	6.25E-01	CCBL2	20	rs386365	48470468	5.13E-01	N/A	-	6.56E-07	1.24E-01	8.64E-04	9615	
1	rs4655890	89217553	6.25E-01	CCBL2	20	rs386365	48470468	5.13E-01	N/A	-	6.56E-07	1.55E-01	8.64E-04	9616	
6	rs2296048	5815349	4.96E-01	N/A	1	rs2301877	1.82E+08	1.82E-01	NMN/AT2	-	6.56E-07	9.48E-01	8.27E-05	9617	
6	rs748903	5808342	4.96E-01	N/A	1	rs2301877	1.82E+08	1.82E-01	NMN/AT2	-	6.56E-07	9.48E-01	8.27E-05	9618	
14	rs4074077	1.04E+08	8.74E-01	N/A	21	rs2833181	31260401	5.33E-02	N/A	-	6.56E-07	2.22E-01	1.04E-05	9619	
2	rs341605	44917691	3.10E-02	N/A	15	rs506120	41589316	9.55E-02	TP53BP1	-	6.56E-07	5.04E-01	8.04E-04	9620	
5	rs4921367	1.6E+08	3.81E-01	N/A	9	rs3789281	1.18E+08	5.32E-01	PAPPA	-	6.56E-07	6.38E-01	1.73E-04	9621	
5	rs12187771	90030739	1.04E-01	GPR98	14	rs12437105	68231084	3.18E-01	N/A	-	6.56E-07	7.10E-01	1.26E-04	9622	
1	rs10753239	31134811	3.93E-01	SDC3	2	rs1861720	2.07E+08	8.09E-01	N/A	-	6.56E-07	7.77E-01	5.45E-05	9623	
1	rs6683918	1.04E+08	6.40E-01	N/A	12	rs4763135	62733222	6.93E-01	SRGAP1	-	6.56E-07	8.96E-01	8.11E-05	9624	
16	rs13336699	82426672	3.37E-01	N/A	20	rs6048372	22752426	8.43E-01	N/A	-	6.56E-07	9.91E-01	7.49E-05	9625	
1	rs7523434	1.65E+08	1.28E-01	GPA33	12	rs2669017	75564882	3.00E-01	N/A	-	6.56E-07	7.18E-01	1.24E-04	9626	
4	rs1441722	1.65E+08	9.06E-01	1-Mar	21	rs2014791	22067821	9.65E-01	N/A	-	6.56E-07	9.02E-01	2.66E-05	9627	
6	rs3130559	31205280	7.50E-01	C6orf15	21	rs9976900	35306233	6.97E-01	RUNX1	MHC	6.56E-07	1.73E-02	2.00E-07	9628	
2	rs11676822	16857559	9.69E-01	N/A	21	rs6518237	45627380	4.76E-01	N/A	-	6.56E-07	8.83E-01	1.67E-04	9629	
14	rs2596114	80198982	5.74E-01	C14orf145	15	rs1623020	45612582	6.09E-02	N/A	-	6.56E-07	1.38E-01	6.85E-06	9630	

SNP A					SNP B						Interaction P			Ranking	Cluster in top 100 interactions
CHR	SNP	Location	gle locus P va	Gene	CHR	SNP	Location	ingle locus P valu	Gene	HC region	Stage 1	Stage 2	Combined		
2	rs1125383	1.05E+08	1.42E-01	N/A	9	rs2181029	27316904	1.05E-01	MOBKL2B	-	6.56E-07	5.40E-01	5.06E-05	9631	
6	rs969931	29602876	1.59E-01	N/A	10	rs2180563	33430188	4.72E-01	N/A	MHC	6.56E-07	9.99E-01	5.65E-05	9632	
8	rs579096	20846271	9.14E-01	N/A	9	rs10815111	4901930	7.16E-01	N/A	-	6.56E-07	N/A	N/A	9633	
11	rs669834	79043373	8.37E-01	N/A	18	rs4798335	5270622	5.42E-01	ZFP161	-	6.56E-07	7.50E-02	8.85E-03	9634	
6	rs9359457	81904850	7.73E-01	N/A	4	rs1473221	60466092	1.78E-01	N/A	-	6.56E-07	2.68E-01	1.14E-03	9635	
6	rs7749819	55442364	1.00E+00	HMGCLL1	10	rs12414556	1.27E+08	5.51E-01	N/A	-	6.56E-07	6.15E-01	1.33E-04	9636	
6	rs9475303	55445021	1.00E+00	HMGCLL1	10	rs12414556	1.27E+08	5.51E-01	N/A	-	6.56E-07	6.15E-01	1.33E-04	9637	
6	rs4566922	8816712	6.24E-01	N/A	8	rs7826624	35586829	6.46E-01	UNC5D	-	6.56E-07	7.77E-01	7.48E-05	9638	
1	rs12711521	11013503	2.32E-01	MASP2	14	rs1467526	51172942	9.42E-01	FRMD6	-	6.57E-07	5.83E-01	1.76E-05	9639	
8	rs3735830	74020431	9.67E-01	KCNB2	10	rs11238609	43453997	8.09E-01	ZNF32	-	6.57E-07	7.67E-01	2.68E-05	9640	
10	rs9299535	77786105	7.01E-01	C10orf11	11	rs670342	78329325	9.25E-01	ODZ4	-	6.57E-07	N/A	N/A	9641	
3	rs11714758	21973157	3.62E-01	N/A	11	rs16930396	14598458	1.37E-01	PSMA1	-	6.57E-07	1.01E-01	2.65E-03	9642	
1	rs1556795	97751905	4.03E-01	DPYD	8	rs925030	25298518	7.28E-01	DOCK5	-	6.57E-07	1.98E-01	5.84E-06	9643	
2	rs6435267	2.06E+08	8.74E-01	PARD3B	8	rs7016966	14118719	4.01E-01	SGCZ	-	6.57E-07	1.55E-01	1.40E-06	9644	
1	rs1193233	7472713	8.95E-01	CAMTA1	14	rs4981673	27958722	6.99E-01	N/A	-	6.57E-07	1.53E-01	5.03E-06	9645	
4	rs236754	1.03E+08	8.32E-01	N/A	10	rs2399659	11278201	2.44E-01	CUGBP2	-	6.57E-07	8.53E-01	1.95E-04	9646	
6	rs1555064	1.64E+08	2.00E-01	PACRG	14	rs2242531	21067948	7.35E-01	METTL3	-	6.57E-07	3.09E-01	5.78E-06	9647	
8	rs10503617	18474753	6.82E-01	PSD3	10	rs871748	5741801	2.59E-01	ASB13	-	6.57E-07	4.98E-01	3.82E-04	9648	
6	rs270398	7710839	2.09E-01	BMP6	17	rs4536508	61813793	7.85E-02	PRKCA	-	6.57E-07	1.86E-01	5.41E-06	9649	
6	rs270398	7710839	2.09E-01	BMP6	17	rs7211558	61812941	7.85E-02	PRKCA	-	6.57E-07	1.86E-01	5.41E-06	9650	
3	rs263412	10217671	7.61E-01	IRAK2	10	rs2889780	50231196	6.87E-01	DRGX	-	6.57E-07	3.27E-01	2.56E-04	9651	
2	rs6730963	1.33E+08	9.52E-01	NCKAP5	17	rs8069937	40564152	6.46E-01	ACBD4	-	6.57E-07	8.66E-01	2.07E-04	9652	
2	rs3820926	2.28E+08	4.59E-01	COL4A4	17	rs4239112	10096227	3.32E-01	N/A	-	6.57E-07	N/A	N/A	9653	
6	rs847848	35014146	5.60E-01	ANKS1A	9	rs287572	31994713	1.35E-01	N/A	MHC	6.57E-07	1.18E-01	1.78E-03	9654	
6	rs847848	35014146	5.60E-01	ANKS1A	9	rs287573	31994571	1.35E-01	N/A	MHC	6.57E-07	1.18E-01	1.78E-03	9655	
6	rs847848	35014146	5.60E-01	ANKS1A	9	rs407791	32012207	1.35E-01	N/A	MHC	6.57E-07	1.18E-01	1.78E-03	9656	
2	rs13000806	1.93E+08	3.10E-01	TMEFF2	20	rs2210578	10084402	6.71E-01	N/A	-	6.57E-07	1.28E-01	3.07E-06	9657	
5	rs7716678	1.5E+08	3.02E-01	NDST1	13	rs9575933	35808743	9.12E-02	SPG20	-	6.57E-07	5.94E-01	1.56E-05	9658	
6	rs847848	35014146	5.60E-01	ANKS1A	9	rs453809	32006697	1.35E-01	N/A	MHC	6.57E-07	N/A	N/A	9659	
2	rs11688532	1.28E+08	1.78E-01	N/A	7	rs1431534	14430643	3.87E-01	DGKB	-	6.58E-07	5.71E-01	5.25E-04	9660	
3	rs7619876	1.14E+08	3.65E-01	N/A	19	rs12462307	63476544	3.48E-01	ZNF544	-	6.58E-07	5.86E-01	2.42E-04	9661	
2	rs13024316	1.42E+08	9.16E-01	LRP1B	7	rs10278980	52066867	8.99E-01	N/A	-	6.58E-07	2.89E-01	2.83E-03	9662	
4	rs13108458	32614391	4.50E-02	N/A	20	rs6037513	3171225	7.60E-01	C20orf194	-	6.58E-07	7.10E-01	3.44E-04	9663	
12	rs825090	1.01E+08	3.89E-01	MYBPC1	14	rs17114437	43352817	5.00E-02	N/A	-	6.58E-07	2.33E-01	8.24E-06	9664	
4	rs7440592	66137501	6.34E-02	EPHA5	9	rs16908681	1.39E+08	6.46E-02	FAM135B	-	6.58E-07	3.69E-01	2.70E-06	9665	
4	rs4624709	66152139	5.53E-02	EPHA5	8	rs16908681	1.39E+08	6.46E-02	FAM135B	-	6.58E-07	4.44E-01	4.15E-06	9666	
3	rs1828652	1.47E+08	9.13E-01	N/A	11	rs10837475	40656383	2.70E-01	N/A	-	6.58E-07	6.83E-01	3.37E-05	9667	
4	rs17033388	1.57E+08	7.07E-01	GUCY1A3	5	rs2045990	1.01E+08	9.39E-01	N/A	-	6.58E-07	7.44E-01	2.35E-04	9668	
5	rs4704506	77690583	5.04E-01	SCAMP1	11	rs7126049	73061030	5.43E-01	RAB6A	-	6.58E-07	9.01E-01	1.77E-04	9669	
11	rs12576767	1.17E+08	1.90E-01	N/A	22	rs5997110	25366505	3.47E-01	CRYBA4	-	6.58E-07	8.63E-01	1.93E-04	9670	
8	rs4368963	42463395	9.89E-01	SLC20A2	10	rs7922447	1.02E+08	5.04E-01	DNMBP	-	6.58E-07	6.43E-01	1.05E-04	9671	
7	rs10488536	92719598	1.26E-01	CCDC132	13	rs9541313	67737117	4.18E-01	N/A	-	6.58E-07	3.20E-02	2.87E-07	9672	
2	rs10170117	1.62E+08	6.43E-01	N/A	20	rs4299400	14869606	9.44E-02	MACROD2	-	6.58E-07	2.12E-01	4.26E-03	9673	
3	rs843790	1.54E+08	4.31E-02	N/A	8	rs328300	38463656	1.14E-01	N/A	-	6.58E-07	2.44E-01	1.22E-05	9674	
10	rs2395023	71126317	7.01E-01	N/A	18	rs3902020	48065150	7.07E-01	N/A	-	6.58E-07	1.99E-01	2.79E-03	9675	
6	rs4541741	41772010	1.22E-01	TFEB	11	rs9326286	1.26E+08	2.37E-01	KIRREL3	-	6.58E-07	4.64E-01	2.60E-04	9676	
2	rs10171698	79011181	2.64E-01	N/A	11	rs6591324	55941921	3.16E-01	OR8U8	-	6.58E-07	5.35E-01	3.24E-04	9677	
2	rs12618471	1.87E+08	6.59E-01	N/A	3	rs7433389	18249117	7.25E-01	N/A	-	6.59E-07	4.90E-01	4.48E-05	9678	
1	rs4970610	38865078	1.51E-01	N/A	9	rs7858089	28399300	3.65E-01	LINGO2	-	6.59E-07	4.64E-01	2.04E-04	9679	
9	rs2273766	76887451	2.03E-01	OSTF1	18	rs1523232	24230826	1.07E-01	N/A	-	6.59E-07	8.69E-01	1.85E-05	9680	
9	rs1392521	8369313	2.54E-01	PTPRD	18	rs2156025	66885986	3.67E-01	N/A	-	6.59E-07	1.32E-01	7.12E-03	9681	
3	rs16852880	1.7E+08	5.05E-01	N/A	12	rs394119	48591274	8.09E-01	LOC283332	-	6.59E-07	7.66E-03	7.88E-03	9682	
3	rs6801861	3674454	1.60E-01	N/A	21	rs2833047	31025370	6.76E-01	KRTAP21-3	-	6.59E-07	6.91E-02	3.02E-07	9683	
3	rs6801861	3674454	1.60E-01	N/A	21	rs2833073	31040071	6.76E-01	KRTAP21-2	-	6.59E-07	6.91E-02	3.02E-07	9684	
6	rs12206684	18761302	6.54E-01	N/A	2	rs1522664	1.48E+08	4.22E-01	N/A	-	6.59E-07	7.57E-02	3.10E-06	9685	
3	rs154961	6491544	4.25E-01	N/A	12	rs6488326	10819446	3.01E-01	N/A	-	6.59E-07	5.87E-01	9.49E-05	9686	
5	rs10041997	1.2E+08	5.18E-01	N/A	20	rs527643	47639993	9.65E-01	N/A	-	6.59E-07	9.28E-01	9.09E-05	9687	
2	rs4850931	1E+08	8.67E-01	CHST10	3	rs11916012	1.88E+08	1.30E-01	N/A	-	6.60E-07	8.51E-02	7.36E-07	9688	
2	rs1861168	49037518	6.54E-01	FSHR	5	rs10462946	1.64E+08	4.71E-01	N/A	-	6.60E-07	4.06E-01	1.63E-04	9689	
5	rs6594929	1.15E+08	9.45E-01	LVRN	13	rs12863583	26426322	4.69E-01	N/A	-	6.60E-07	4.11E-01	7.07E-06	9690	
9	rs649891	10420602	8.75E-01	PTPRD	12	rs1497176	40519549	9.86E-01	N/A	-	6.60E-07	5.91E-01	9.19E-04	9691	
4	rs7687468	58807916	4.05E-01	N/A	9	rs10868806	72143304	3.29E-01	SMC5	-	6.60E-07	5.76E-01	1.11E-05	9692	
11	rs10891914	1.15E+08	8.34E-01	N/A	13	rs7989892	45528642	1.85E-01	CPB2	-	6.60E-07	6.55E-01	5.22E-04	9693	
2	rs6740698	54577245	8.28E-01	SPTBN1	11	rs7110039	1.22E+08	2.27E-03	C11orf63	-	6.60E-07	9.00E-01	7.04E-05	9694	
6	rs17058986	99537921	6.53E-01	N/A	11	rs17432191	1.04E+08	6.53E-01	N/A	-	6.60E-07	2.81E-01	3.82E-04	9695	
1	rs2686228	2.39E+08	9.03E-01	RGS7	2	rs1574392	2.28E+08	4.68E-01	COL4A4	-	6.60E-07	3.64E-01	5.94E-04	9696	
1	rs10753374	4188905	3.76E-01	N/A	9	rs2781102	1.22E+08	7.99E-01	N/A	-	6.60E-07	7.61E-01	1.79E-05	9697	
13	rs10047744	29310337	4.56E-01	N/A	16	rs7190076	79065556	6.67E-01	N/A	-	6.60E-07	2.09E-01	3.62E-03	9698	
7	rs10486378	19952661	3.53E-02	N/A	20	rs1182484	57777748	4.12E-01	PHACTR3	-	6.60E-07	6.09E-01	1.10E-05	9699	
15	rs2034247	78399486	8.59E-01	N/A	19	rs8104890	57211420	3.57E-01	ZNF614	-	6.60E-07	6.95E-01	2.44E-05	9700	
9	rs10739593	1.23E+08	9.58E-01	GSN	13	rs928021	69604009	4.49E-01	ATXN8OS	-	6.60E-07	7.24E-01	2.87E-04	9701	
7	rs10256379	1.41E+08	4.49E-01	N/A	14	rs8015016	95192161	2.86E-02	TCL6	-	6.60E-07	1.30E-01	3.90E-07	9702	
9	rs17195438	79320366	2.00E-01	GN/A14	14	rs10135747	72959205	5.45E-01	NUMB	-	6.60E-07	1.33E-01	2.87E-03	9703	
2	rs6437268	2.41E+08	4.02E-01	OR6B3	9	rs1873738	80967681	1.18E-01	N/A	-	6.60E-07	3.86E-01	1.35E-03	9704	
3	rs13061155	60244988	4.99E-01	FHIT	8	rs1713736	1.24E+08	4.74E-01	WDR67	-	6.60E-07	4.29E-01	1.55E-03	9705	
3	rs4593008	2034893	8.35E-01	N/A	15	rs2715803	95085514	4.90E-01	N/A	-	6.60E-07	7.74E-01	1.98E-04	9706	
6	rs3822992	1.53E+08	1.95E-01	SYNE1	12	rs838916	1.24E+08	4.63E-01	SCARB1	-	6.60E-07	6.35E-01	2.25E-05	9707	
3	rs4685766	4582533	2.81E-01	ITPR1	3	rs2324938	86096826	5.01E-01	CADM2	-	6.60E-07	6.29E-01	3.52E-05	9708	
7	rs7789227	12089416	2.68E-01	N/A	7	rs974134	71049725	2.78E-01	CALN1	-	6.60E-07	7.30E-01	6.12E-04	9709	
9	rs4842090	1.38E+08	2.93E-01	CAPN8	20	rs1777356	5794123	2.43E-01	C20orf196	-	6.60E-07	3.93E-01	7.42E-04	9710	
7	rs11974150	30727735	7.75E-02	N/A	22	rs135896	43156806	4.96E-01	N/A	-	6.60E-07	4.82E-01	4.50E-04	9711	
5	rs677881	1.18E+08	5.65E-01	N/A	12	rs6582630	37029775	2.82E-02	ALG10B	-	6.61E-07	N/A	N/A	9712	
4	rs12643813	1.63E+08	8.11E-01	N/A	13	rs17650122	28482893	1.39E-01	MTUS2	-	6.61E-07	2.41E-01	6.15E-06	9713	
4	rs4619965	1.19E+08	7.28E-01	N/A	5	rs2114961	1.35E+08	3.60E-01	N/A	-	6.61E-07	3.62E-01	2.95E-04	9714	
3	rs2686107	1.99E+08	7.18E-01	BDH1	11	rs7938378	11566292	5.63E-01	GALNTL4	-	6.61E-07	5.12E-02	4.20E-07	9715	
6	rs6910409	1.23E+08	3.45E-01	CLVS2	1	rs1552991	1.5E+08	1.63E-01	HRNR	-	6.61E-07	2.41E-01	1.13E-03	9716	
10	rs3858282	90424391	8.17E-01	LIPF	21	rs2837693	40832232	1.95E-01	DSCAM	-	6.61E-07	2.83E-01	3.01E-04	9717	
18	rs6566862	52950493	7.29E-01	N/A	20	rs4811099	48936359	7.16E-01	BCAS4	-	6.61E-07	3.56E-01	4.92E-04	9718	
6	rs934149	53204945	8.79E-01	N/A	3	rs1799852	1.35E+08	5.73E-01	TF	-	6.61E-07	1.81E-01	9.73E-07	9719	
10	rs12777530	21184987	2.89E-01	N/A	12	rs1009016	1.13E+08	2.89E-01	N/A	-	6.61E-07	2.41E-01	3.16E-06	9720	
2	rs1594304	1.83E+08	8.07E-01	PDE1A	22	rs17001868	39108177	1.20E-02	ADSL	-	6.61E-07	5.11E-01	3.23E-05	9721	
2	rs1914507	76863507	7.98E-01	LRRTM4	10	rs7909875	1.18E+08	2.77E-01	GFRA1	-	6.61E-07	8.61E-01	1.20E-04	9722	
5	rs7718088	1.8E+08	3.52E-01	RASGEF1C	7	rs160357	28635027	3.56E-01	CREB5	-	6.61E-07	8.88E-01	9.08E-05	9723	
12	rs7314370	1.14E+08	2.38E-01	N/A	20	rs6136213	17798340	8.88E-01	N/A	-	6.61E-07	6.05E-01	1.15E-05	9724	
5	rs1816398	1.56E+08	4.39E-01	N/A	17	rs8078686	43090705	4.98E-01	KPNB1	-	6.61E-07	2.94E-01	1.75E-03	9725	
4	rs633398	1.19E+08	2.43E-01	NDST3	10	rs10904733	16508848	9.98E-01	PTER	-	6.61E-07	6.56E-01	4.63E-05	9726	
5	rs10040022	19000599	8.35E-01	N/A	5	rs34236	1.03E+08	7.49E-01	N/A	-	6.61E-07	N/A	N/A	9727	
3	rs7651261	1.83E+08	3.46E-01	N/A	11	rs5030207	32395053	7.11E-01	WT1	-	6.62E-07	9.13E-02	6.60E-07	9728	
6	rs539298	1.61E+08	8.47E-02	SLC22A3	11	rs12278958	1.28E+08	5.96E-01	RICS	-	6.62E-07	7.18E-01	2.42E-04	9729	
4	rs1948573	1.9E+08	6.64E-01	N/A	11	rs1943761	1.01E+08	4.04E-01	N/A	-	6.62E-07	8.28E-01	2.65E-04	9730	
9	rs11142845	73286348	2.55E-01	N/A	9	rs4743764	1.07E+08	9.91E-01	ABCA1	-	6.62E-07	3.44E-01	3.32E-03	9731	
3	rs6803623	1.23E+08	2.85E-01	CASR	11	rs11026661	3328263	3.84E-02	ZNF195	-	6.62E-07	2.43E-01	1.00E-03	9732	
13	rs9318075	71923967	2.90E-02	N/A	15	rs8037097	46891401	1.26E-01	SHC4	-	6.62E-07	7.29E-01	6.09E-05	9733	
12	rs1392313	57823606	4.36E-01	N/A	13	rs4362282	1.12E+08	5.74E-01	N/A	-	6.62E-07	8.27E-01	5.19E-05	9734	
1	rs1155002	60146363	2.62E-01	CYP2J2	18	rs347542	48234610	1.30E-01	DCC	-	6.62E-07	4.85E-01	1.17E-05	9735	
5	rs4701768	7204564	1.41E-01	N/A	5	rs4571496	36466512	1.64E-02	N/A	-	6.62E-07	7.34E-01	4.69E-05	9736	
4	rs2043644	1.49E+08	8.15E-01	ARHGAP10	11	rs560640	1.25E+08	6.07E-02	CDON	-	6.62E-07	3.65E-01	5.70E-06	9737	
6	rs688198	93737778	4.46E-01	N/A	14	rs1204982	71289922	4.06E-02	SIPA1L1	-	6.62E-07	9.10E-01	4.81E-04	9738	
9	rs3802404	7023514	4.26E-01	KDM4C	13	rs7982669	97339220	4.65E-01	N/A	-	6.62E-07	5.48E-01	2.69E-04	9739	
11	rs1940080	84048322	6.12E-01	DLG2	17	rs9911897	5555845	6.63E-01	N/A	-	6.63E-07	3.91E-01	1.40E-03	9740	
9	rs4978717	1.1E+08	1.03E-01	N/A	20	rs2207541	59613519	5.58E-01	CDH4	-	6.63E-07	6.75E-01	4.94E-04	9741	
4	rs6850538	1.79E+08	3.96E-01	LOC285501	10	rs10736128	1E+08	7.48E-01	N/A	-	6.63E-07	8.40E-01	7.71E-05	9742	

SNP A					SNP B						Interaction P			Ranking	Cluster in top 100 interactions
CHR	SNP	Location	gle locus P va	Gene	CHR	SNP	Location	Single locus P valu	Gene	HC region	Stage 1	Stage 2	Combined		
14	rs4899673	77236707	4.21E-01	C14orf156	18	s12326518	19139126	4.70E-01	C18orf45	-	6.63E-07	9.75E-01	4.44E-05	9743	
13	rs7991698	20012824	2.48E-01	CRYL1	15	rs1453854	46016892	9.06E-01	N/A	-	6.63E-07	3.88E-01	1.07E-05	9744	
1	rs10489729	1.83E+08	1.79E-01	C1orf21	16	rs8050676	22723494	2.20E-01	MIR548D2	-	6.63E-07	1.32E-02	1.54E-02	9745	
1	rs845451	2.08E+08	9.45E-01	SERTAD4	12	rs7967556	4974791	9.59E-01	N/A	-	6.63E-07	6.51E-01	3.77E-04	9746	
2	rs3754944	2.31E+08	9.01E-01	SP100	7	s12703951	1.47E+08	9.39E-01	CNTN/AP2	-	6.63E-07	N/A	N/A	9747	
2	rs3771790	74924075	5.95E-01	HK2	10	s12572137	50185252	3.48E-01	C10orf71	-	6.63E-07	3.02E-01	2.60E-04	9748	
1	rs1727098	39761914	6.66E-02	BMP8A	12	rs4767311	1.14E+08	2.88E-01	N/A	-	6.63E-07	8.27E-01	3.19E-04	9749	
6	rs7741438	5668117	4.89E-01	FARS2	4	rs7693492	1.4E+08	1.12E-01	N/A	-	6.63E-07	3.25E-01	1.96E-05	9750	
2	rs2194408	53397123	7.00E-01	N/A	12	s12579294	3160206	5.99E-01	TSPAN9	-	6.63E-07	7.50E-01	1.20E-04	9751	
12	rs7298815	5189961	9.89E-01	N/A	17	rs8080666	59840612	8.26E-01	N/A	-	6.63E-07	8.74E-01	1.28E-04	9752	
10	rs4933211	93358143	4.39E-01	LOC100188947	12	s10847727	1.28E+08	7.76E-01	GLT1D1	-	6.63E-07	7.33E-01	1.02E-04	9753	
18	rs2033104	34351851	2.33E-01	N/A	20	s16995991	9439492	6.09E-03	C20orf103	-	6.63E-07	7.35E-01	6.73E-05	9754	
9	rs7869846	1767728	3.44E-01	N/A	14	rs1376889	45987357	6.90E-01	N/A	-	6.63E-07	1.39E-01	3.06E-06	9755	
8	rs7833351	95251354	6.61E-01	CDH17	17	rs940009	22924910	3.78E-01	KSR1	-	6.63E-07	5.19E-01	2.63E-05	9756	
10	rs6560722	1322488	5.23E-01	ADARB2	11	rs683323	1.25E+08	8.67E-02	PUS3	-	6.63E-07	8.17E-01	1.21E-04	9757	
5	rs2057831	1.38E+08	8.16E-01	CDC23	22	rs137916	48833840	2.20E-01	TTLL8	-	6.63E-07	8.78E-01	4.56E-05	9758	
5	rs2864	1.38E+08	8.16E-01	BRD8	22	rs137916	48833840	2.20E-01	TTLL8	-	6.63E-07	8.78E-01	4.56E-05	9759	
3	rs9874803	1.14E+08	4.57E-01	C3orf17	11	s10902218	733813	9.12E-02	EPS8L2	-	6.64E-07	2.77E-01	5.41E-06	9760	
9	rs10817970	1.19E+08	3.37E-01	ASTN2	16	rs3751688	88161940	3.42E-02	CPNE7	-	6.64E-07	3.98E-01	5.12E-06	9761	
1	rs1252071	1.76E+08	6.75E-01	N/A	3	s17038909	1442145	4.92E-01	N/A	-	6.64E-07	5.02E-01	2.09E-05	9762	
3	rs1566548	1.6E+08	6.22E-01	N/A	7	rs702479	12770960	2.89E-01	N/A	-	6.64E-07	5.50E-01	7.16E-04	9763	
2	rs1052431	1.03E+08	8.08E-01	SLC9A2	8	s10100405	88960987	6.43E-01	DCAF4L2	-	6.64E-07	5.41E-01	3.09E-05	9764	
7	rs2287126	37223275	7.85E-01	ELMO1	13	rs6492677	93200111	6.38E-01	GPC6	-	6.64E-07	9.32E-02	1.18E-06	9765	
7	rs2287126	37223275	7.85E-01	ELMO1	13	rs7993501	93196981	6.38E-01	GPC6	-	6.64E-07	9.32E-02	1.18E-06	9766	
15	rs10518725	51603044	2.99E-01	WDR72	20	rs6022965	52154471	7.37E-01	N/A	-	6.64E-07	8.01E-02	1.66E-06	9767	
7	rs354052	1.49E+08	7.76E-01	ZNF767	10	rs7076844	19866244	5.90E-01	N/A	-	6.64E-07	3.72E-01	2.41E-03	9768	
2	rs4852033	2.4E+08	2.71E-01	N/A	17	rs9911645	10632225	2.46E-01	N/A	-	6.64E-07	8.14E-01	7.66E-05	9769	
4	rs6856910	1.86E+08	4.53E-01	IRF2	5	rs6871988	1.53E+08	2.98E-01	N/A	-	6.64E-07	9.52E-01	9.79E-05	9770	
18	rs7236709	43780889	9.98E-01	N/A	21	rs524662	30506260	5.33E-01	CLDN8	-	6.64E-07	3.40E-01	6.46E-05	9771	
3	rs698675	1.42E+08	2.52E-01	TRIM42	5	s11745195	26242976	4.92E-01	N/A	-	6.64E-07	8.05E-01	1.98E-05	9772	
11	rs16933	44921948	8.37E-02	TP53I11	12	s11063009	4109753	2.93E-01	N/A	-	6.64E-07	5.14E-01	1.56E-05	9773	
2	rs1807143	76040607	1.38E-01	N/A	10	rs1219732	1.26E+08	2.28E-01	CPXM2	-	6.64E-07	6.67E-01	4.60E-05	9774	
5	rs12656216	36196425	3.71E-01	SKP2	10	rs650058	95368537	8.84E-01	PDE6C	-	6.65E-07	2.02E-02	1.24E-07	9775	
5	rs298026	58976968	4.11E-01	PDE4D	8	s10956489	1.31E+08	5.98E-01	N/A	-	6.65E-07	3.29E-01	2.21E-05	9776	
6	rs9484143	1.39E+08	7.03E-01	KIAA1244	2	rs7569111	2.36E+08	1.62E-01	AGAP1	-	6.65E-07	5.62E-01	5.04E-05	9777	
1	rs1103577	1.57E+08	2.54E-01	N/A	13	rs1112938	84307597	6.89E-02	N/A	-	6.65E-07	N/A	N/A	9778	
1	rs1103577	1.57E+08	2.54E-01	N/A	13	rs1358044	84316787	6.89E-02	N/A	-	6.65E-07	N/A	N/A	9779	
16	rs9925768	83735148	7.15E-01	N/A	17	rs6501697	69677322	7.87E-01	N/A	-	6.65E-07	1.62E-01	5.83E-04	9780	
10	rs4751691	1.21E+08	6.55E-01	EIF3A	17	rs7219850	15324395	3.55E-01	CDRT4	-	6.65E-07	2.60E-01	3.60E-06	9781	
1	rs1620977	72501730	7.62E-01	NEGR1	11	rs2510877	1.2E+08	1.22E-01	N/A	-	6.65E-07	3.87E-01	2.81E-05	9782	
2	rs12619205	66579371	1.00E-01	MEIS1	15	s12593813	65823906	9.76E-01	MAP2K5	-	6.65E-07	9.45E-01	8.68E-05	9783	
6	rs1199389	8336115	7.69E-01	N/A	17	rs236597	65747520	2.25E-01	N/A	-	6.65E-07	1.15E-01	2.34E-03	9784	
6	rs9459093	1.58E+08	4.97E-01	SYNJ2	3	rs7429855	74609855	2.42E-01	CNTN3	-	6.65E-07	1.26E-01	2.72E-07	9785	
5	rs2286394	1.4E+08	9.27E-01	WDR55	7	rs4141213	19218681	3.08E-01	N/A	-	6.65E-07	6.74E-01	1.70E-05	9786	
1	rs2296796	2.29E+08	1.34E-01	COG2	11	rs652024	81461531	3.08E-02	N/A	-	6.65E-07	4.55E-01	5.24E-04	9787	
5	rs13185746	1.12E+08	5.16E-01	N/A	16	s11074516	9980913	3.73E-01	GRIN2A	-	6.65E-07	8.47E-01	1.07E-04	9788	
5	rs10067709	1.12E+08	5.16E-01	N/A	16	s11074516	9980913	3.73E-01	GRIN2A	-	6.65E-07	8.89E-01	1.21E-04	9789	
5	rs4866602	3742363	7.06E-01	N/A	22	rs2331112	24272670	8.95E-01	ADRBK2	-	6.65E-07	4.08E-01	5.13E-06	9790	
8	rs1805867	91100250	3.99E-01	DECR1	15	rs4775941	49447122	7.78E-01	GLDN	-	6.65E-07	4.46E-01	9.72E-05	9791	
1	rs7536307	77730636	2.26E-01	AK5	21	rs2839641	43472790	9.99E-01	CRYAA	-	6.65E-07	7.34E-01	2.81E-04	9792	
2	rs17016629	35506420	1.82E-01	N/A	7	rs2353354	1.38E+08	3.66E-01	SVOPL	-	6.65E-07	8.29E-01	2.46E-04	9793	
5	rs10939975	62875449	4.76E-01	N/A	21	rs2238724	44677377	9.84E-01	TRPM2	-	6.66E-07	2.92E-01	6.90E-06	9794	
6	rs1130656	1.61E+08	9.49E-01	PLG	1	rs2453200	2.42E+08	5.37E-01	N/A	-	6.66E-07	3.84E-01	3.27E-03	9795	
4	rs11466640	38455298	4.92E-01	TLR10	19	rs2617640	58591685	7.17E-01	ZNF525	-	6.66E-07	9.19E-01	1.12E-04	9796	
6	rs2235718	1552602	1.38E-01	GMDS	1	s11102213	1.09E+08	4.65E-01	PRPF38B	-	6.66E-07	7.77E-03	4.57E-08	9797	
4	rs1519238	1.25E+08	7.80E-01	N/A	9	rs4745520	70821553	7.12E-01	FXN	-	6.66E-07	5.03E-01	1.85E-05	9798	
4	rs1390264	20995345	6.57E-01	KCNIP4	20	rs6139011	3042175	2.03E-01	UBOX5	-	6.66E-07	2.02E-01	2.35E-03	9799	
1	rs4394682	19710124	5.72E-01	N/A	2	rs1454382	52772433	7.99E-01	N/A	-	6.66E-07	9.59E-01	1.88E-04	9800	
11	rs1944720	78988045	1.68E-01	N/A	18	rs3842993	64393252	2.79E-01	N/A	-	6.66E-07	2.91E-01	1.10E-03	9801	
17	rs4795715	27966241	7.52E-01	MYO1D	18	rs8097986	46445557	7.03E-01	MAPK4	-	6.66E-07	5.73E-01	6.88E-04	9802	
6	rs6454267	83097859	3.68E-01	N/A	4	rs2903472	12665619	2.99E-02	N/A	-	6.66E-07	1.39E-01	3.13E-03	9803	
12	rs7300210	20615619	2.90E-01	PDE3A	15	s12593630	44460804	6.21E-02	N/A	-	6.66E-07	1.46E-01	4.15E-06	9804	
8	rs6999653	434485	7.57E-01	C8orf42	21	rs6517254	35002160	2.86E-01	CLIC6	-	6.66E-07	2.59E-01	4.05E-06	9805	
9	rs7031694	71412996	8.16E-01	APBA1	10	rs7904413	17655653	2.38E-01	PTPLA	-	6.66E-07	4.17E-01	1.05E-04	9806	
10	rs2224865	85412834	9.12E-01	N/A	14	s12587615	20323006	7.23E-01	FAM12B	-	6.67E-07	2.10E-01	1.09E-05	9807	
4	rs1513765	1.39E+08	1.21E-01	N/A	15	rs1992215	65205991	6.25E-01	SMAD3	-	6.67E-07	2.09E-01	1.52E-05	9808	
5	rs2918285	1.49E+08	3.78E-01	ABLIM3	18	rs3809950	65449850	1.94E-01	DOK6	-	6.67E-07	1.60E-01	3.45E-03	9809	
5	rs4277904	1.49E+08	3.78E-01	ABLIM3	18	rs3809950	65449850	1.94E-01	DOK6	-	6.67E-07	1.60E-01	3.45E-03	9810	
6	rs110691	1.24E+08	8.60E-01	NKAIN2	15	rs2623468	37214957	1.63E-01	N/A	-	6.67E-07	1.10E-02	2.98E-08	9811	
4	rs9312076	61933672	9.26E-01	N/A	17	rs4791235	60539518	5.54E-01	N/A	-	6.67E-07	6.43E-01	1.11E-05	9812	
1	rs10489227	1.76E+08	2.67E-01	N/A	7	s10269431	11937854	6.48E-01	N/A	-	6.67E-07	7.48E-01	3.87E-04	9813	
1	rs12038173	1.76E+08	2.67E-01	N/A	7	s10269431	11937854	6.48E-01	N/A	-	6.67E-07	7.48E-01	3.87E-04	9814	
1	rs1885026	1.76E+08	2.67E-01	N/A	7	s10269431	11937854	6.48E-01	N/A	-	6.67E-07	7.48E-01	3.87E-04	9815	
4	rs11939830	1.57E+08	7.81E-01	N/A	15	rs1908194	55641802	1.28E-01	CGNL1	-	6.67E-07	5.26E-01	9.30E-05	9816	
8	rs17819759	58503057	7.55E-01	N/A	18	rs1421265	52617413	6.52E-01	WDR7	-	6.67E-07	6.92E-01	3.44E-04	9817	
8	rs11990437	58519126	7.55E-01	N/A	18	rs1421265	52617413	6.52E-01	WDR7	-	6.67E-07	7.47E-01	2.96E-04	9818	
1	rs12142922	89262503	9.20E-02	GBP3	13	rs2321995	58577784	3.86E-01	N/A	-	6.67E-07	1.80E-01	1.57E-06	9819	
3	rs9809977	52141187	5.49E-01	WDR51A	9	rs944812	1.34E+08	7.45E-01	C9orf171	-	6.67E-07	2.13E-01	1.49E-05	9820	
3	rs9817653	52123273	5.49E-01	WDR51A	9	rs944812	1.34E+08	7.45E-01	C9orf171	-	6.67E-07	2.13E-01	1.49E-05	9821	
2	rs1016883	1.99E+08	9.21E-01	PLCL1	7	s12699758	15930763	7.54E-01	N/A	-	6.67E-07	7.00E-01	4.01E-05	9822	
8	rs17432531	4647993	3.54E-01	CSMD1	18	rs568188	8109001	3.77E-01	PTPRM	-	6.67E-07	3.22E-01	1.89E-03	9823	
11	rs577948	1.22E+08	2.84E-01	LOC399959	12	s12814930	29325607	4.56E-01	FAR2	-	6.67E-07	7.29E-01	6.16E-05	9824	
3	rs1127732	33880570	4.74E-01	PDCD6IP	13	rs2038712	21757616	3.79E-02	N/A	-	6.67E-07	2.55E-01	2.79E-04	9825	
2	rs2564025	5923146	1.30E-01	N/A	9	rs4620360	29937473	6.60E-01	N/A	-	6.67E-07	N/A	N/A	9826	
1	rs1832787	18499504	2.02E-01	IGSF21	3	rs1866773	29753247	7.15E-01	N/A	-	6.68E-07	7.19E-01	6.00E-05	9827	
3	rs11712075	43338209	3.96E-01	SNRK	5	rs292960	58097645	9.44E-01	RAB3C	-	6.68E-07	5.22E-01	4.20E-06	9828	
2	rs2896773	1.27E+08	5.54E-01	N/A	21	rs9808753	33709182	4.86E-01	IFNGR2	-	6.68E-07	6.04E-01	3.36E-04	9829	
2	rs754737	1.27E+08	5.54E-01	N/A	21	rs9808753	33709182	4.86E-01	IFNGR2	-	6.68E-07	6.04E-01	3.36E-04	9830	
3	rs13059129	1.65E+08	4.40E-01	N/A	8	rs7823200	10585168	2.10E-01	C8orf74	-	6.68E-07	8.40E-01	3.74E-05	9831	
3	rs13096857	1.65E+08	4.40E-01	N/A	8	rs7823200	10585168	2.10E-01	C8orf74	-	6.68E-07	8.40E-01	3.74E-05	9832	
3	rs6770461	1.65E+08	4.40E-01	N/A	8	rs7823200	10585168	2.10E-01	C8orf74	-	6.68E-07	8.40E-01	3.74E-05	9833	
12	rs4762400	96602255	5.45E-01	N/A	13	rs9554169	27313850	9.24E-01	N/A	-	6.68E-07	1.21E-01	6.56E-07	9834	
1	rs99174	1.78E+08	6.35E-02	LHX4	10	rs4237340	6754985	9.15E-03	N/A	-	6.68E-07	4.85E-01	1.14E-05	9835	
1	rs12126518	94017704	5.48E-01	N/A	5	rs6556137	1.74E+08	6.43E-02	N/A	-	6.68E-07	1.44E-01	4.96E-03	9836	
2	rs10180930	1.27E+08	6.06E-01	N/A	21	rs9978525	33869726	1.65E-01	CRYZL1	-	6.68E-07	4.54E-01	1.04E-03	9837	
2	rs6715733	1.27E+08	6.06E-01	N/A	21	rs9978525	33869726	1.65E-01	CRYZL1	-	6.68E-07	4.54E-01	1.04E-03	9838	
2	rs10178199	1.27E+08	6.61E-01	N/A	21	rs9978525	33869726	1.65E-01	CRYZL1	-	6.68E-07	4.54E-01	1.04E-03	9839	
6	rs969931	29602876	1.59E-01	N/A	10	rs970654	33439740	6.52E-01	N/A	MHC	6.68E-07	2.31E-01	4.69E-06	9840	
2	rs6735994	2.03E+08	9.53E-01	N/A	4	rs1377916	91916000	9.71E-01	FAM190A	-	6.68E-07	3.46E-01	7.43E-04	9841	
2	rs2015245	14662597	2.63E-01	N/A	8	rs2355754	1.14E+08	2.27E-01	CSMD3	-	6.68E-07	9.52E-01	9.61E-05	9842	
7	rs10280848	67742124	8.09E-01	N/A	10	rs3824699	26395998	4.96E-01	MYO3A	-	6.69E-07	3.15E-01	7.56E-04	9843	
15	rs1403590	23848717	2.16E-01	N/A	17	s11657462	52818145	2.83E-01	MSI2	-	6.69E-07	6.85E-01	2.14E-05	9844	
7	rs270904	1.37E+08	7.97E-01	DGKI	7	s12538056	1.41E+08	7.46E-01	AGK	-	6.69E-07	5.09E-01	2.85E-05	9845	
2	rs10189976	99662070	8.28E-01	AFF3	20	rs4816145	9445388	4.61E-02	C20orf103	-	6.69E-07	8.07E-01	1.66E-05	9846	
6	rs1433739	40531300	9.40E-01	LRFN2	19	rs2604874	45949656	5.08E-01	ITPKC	-	6.69E-07	3.71E-01	7.62E-06	9847	
6	rs7770051	1.56E+08	2.42E-02	TIAM2	17	rs7213136	32184076	3.85E-01	N/A	-	6.69E-07	2.57E-01	4.93E-03	9848	
9	rs626533	3116263	7.02E-02	N/A	20	rs2423808	14469106	4.81E-01	MACROD2	-	6.69E-07	1.99E-03	1.32E-08	9849	
6	rs2523849	31133030	6.18E-01	HCG22	6	rs2233984	31187243	4.20E-02	C6orf15	MHC	6.69E-07	7.07E-01	2.03E-03	9850	
6	rs1857859	1.01E+08	3.28E-01	SIM1	7	rs886505	8752582	9.14E-01	NXPH1	-	6.69E-07	6.20E-01	8.45E-04	9851	
5	rs6859765	2705947	2.96E-02	N/A	7	rs679423	83176689	3.40E-01	N/A	-	6.69E-07	9.09E-01	1.53E-05	9852	
2	rs9679491	1.22E+08	9.41E-02	N/A	7	s12535032	79133193	3.27E-01	N/A	-	6.69E-07	3.30E-01	4.88E-06	9853	
1	rs12120336	84518161	5.84E-01	SAMD13	11	rs669244	1.22E+08	4.14E-01	N/A	-	6.69E-07	8.75E-01	9.62E-05	9854	

SNP A					SNP B						Interaction P			Ranking	Cluster in top 100 interactions
CHR	SNP	Location	gle locus P va	Gene	CHR	SNP	Location	ingle locus P valu	Gene	HC region	Stage 1	Stage 2	Combined		
2	rs4500939	2.29E+08	8.48E-01	SPHKAP	4	rs1401910	56867735	9.49E-02	KIAA1211	-	6.69E-07	2.74E-02	2.99E-02	9855	
3	rs1917524	65535207	9.66E-01	MAGI1	18	rs2850463	31389109	9.82E-01	N/A	-	6.69E-07	8.64E-01	6.69E-05	9856	
6	rs9465831	20719905	7.22E-01	CDKAL1	20	rs713406	56389876	4.24E-01	VAPB	-	6.70E-07	4.16E-01	1.15E-03	9857	
5	rs246660	1.42E+08	3.22E-02	ARHGAP26	18	rs4131293	21026067	4.08E-01	ZNF521	-	6.70E-07	1.71E-01	1.25E-06	9858	
6	rs484406	92497627	8.78E-01	N/A	11	rs216486	16777742	4.09E-01	PLEKHA7	-	6.70E-07	9.66E-01	4.21E-04	9859	
4	rs4699831	96185917	7.16E-01	BMPR1B	15	rs4779046	81139827	7.98E-01	AP3B2	-	6.70E-07	5.43E-01	2.03E-05	9860	
1	rs10493809	88353971	7.71E-01	N/A	11	rs4944992	74541928	5.21E-03	SLCO2B1	-	6.70E-07	3.26E-01	3.00E-04	9861	
6	rs794114	1.65E+08	5.41E-01	N/A	2	's13387662	34016918	2.26E-01	N/A	-	6.70E-07	6.92E-01	2.46E-05	9862	
10	rs12217563	31781279	1.56E-02	ZEB1	19	rs2074552	2165057	1.08E-01	DOT1L	-	6.70E-07	9.85E-01	2.25E-04	9863	
6	rs9383633	1.5E+08	4.17E-01	RAET1K	9	's13293034	1.27E+08	4.15E-01	MAPKAP1	-	6.70E-07	1.71E-01	8.04E-04	9864	
2	rs11680124	71656459	7.96E-01	DYSF	3	rs1530734	54171506	3.02E-01	CACN/A2D3	-	6.70E-07	5.14E-01	3.01E-05	9865	
1	rs4847386	93193495	6.66E-01	FAM69A	18	rs749309	64573576	6.57E-02	CCDC102B	-	6.70E-07	8.93E-01	1.56E-04	9866	
6	rs6940079	20345367	4.89E-01	N/A	22	rs397709	16869048	8.73E-01	MICAL3	-	6.70E-07	N/A	N/A	9867	
8	rs2409096	8729887	1.70E-01	MFHAS1	21	rs4143412	27317398	1.06E-01	N/A	-	6.70E-07	3.48E-01	2.02E-03	9868	
1	rs6701594	78749158	9.42E-01	PTGFR	10	's11257877	12622719	8.07E-01	CAMK1D	-	6.70E-07	1.31E-01	2.91E-06	9869	
12	rs12582525	95573516	6.78E-02	N/A	15	's12594730	37442648	6.90E-01	N/A	-	6.70E-07	2.64E-01	3.14E-06	9870	
9	rs1575506	84978642	6.88E-01	N/A	13	rs204566	31559712	9.31E-01	FRY	-	6.70E-07	6.20E-01	6.41E-04	9871	
1	rs1575983	75034784	6.69E-01	N/A	7	rs7779412	1.49E+08	7.42E-01	N/A	-	6.71E-07	9.04E-02	3.19E-03	9872	
10	rs927935	14862497	4.34E-01	FAM107B	16	rs7192390	58144475	1.38E-01	N/A	-	6.71E-07	1.01E-01	7.04E-03	9873	
5	rs2120349	1.13E+08	1.97E-01	MCC	12	's11180719	74531784	1.30E-01	N/A	-	6.71E-07	9.88E-01	8.36E-04	9874	
7	rs1839115	1.14E+08	6.07E-02	N/A	18	rs7229532	71822526	2.09E-02	N/A	-	6.71E-07	3.95E-01	1.36E-03	9875	
6	rs17058986	99537921	6.53E-01	N/A	11	's11226342	1.04E+08	7.81E-01	N/A	-	6.71E-07	8.68E-01	2.05E-05	9876	
1	rs6670965	5619264	7.11E-01	N/A	9	rs4742469	8211247	1.90E-01	N/A	-	6.71E-07	9.58E-01	1.14E-04	9877	
8	rs12676876	69767325	1.32E-01	C8orf34	10	rs7918637	1.08E+08	4.52E-01	N/A	-	6.71E-07	5.73E-01	3.00E-04	9878	
3	rs7653203	34963263	2.37E-01	N/A	8	rs4925813	1.46E+08	3.21E-01	KIAA1688	-	6.71E-07	8.62E-01	4.80E-05	9879	
9	rs7027958	89408167	9.00E-01	DAPK1	18	rs7236585	68490525	5.74E-01	N/A	-	6.71E-07	4.82E-01	1.87E-04	9880	
2	rs4851111	1.06E+08	7.20E-01	C2orf40	2	rs4315535	1.22E+08	9.89E-01	N/A	-	6.71E-07	N/A	N/A	9881	
10	rs10828431	23663529	6.97E-01	C10orf67	10	rs3127449	78939737	2.30E-01	KCNMA1	-	6.71E-07	4.68E-02	3.52E-03	9882	
12	rs2079548	1.25E+08	3.16E-01	N/A	13	rs648241	96126061	9.10E-01	HS6ST3	-	6.71E-07	7.24E-01	1.28E-04	9883	
1	rs1103577	1.57E+08	2.54E-01	N/A	13	rs1358045	84317028	7.01E-02	N/A	-	6.71E-07	N/A	N/A	9884	
1	rs1103577	1.57E+08	2.54E-01	N/A	13	rs1554724	84314824	7.01E-02	N/A	-	6.71E-07	N/A	N/A	9885	
4	rs6837396	7470118	4.87E-01	SORCS2	14	rs6575528	95133483	1.34E-02	N/A	-	6.71E-07	2.85E-01	1.74E-06	9886	
4	rs10517791	1.65E+08	9.65E-01	1-Mar	5	rs407360	2661294	4.02E-01	N/A	-	6.71E-07	8.66E-01	8.28E-05	9887	
6	rs9394952	43509083	3.01E-01	ABCC10	5	's10042881	1.64E+08	6.35E-01	N/A	-	6.71E-07	N/A	N/A	9888	
1	rs7536290	2.29E+08	6.46E-01	COG2	3	rs635255	1.75E+08	6.62E-01	NLGN1	-	6.71E-07	2.06E-01	1.78E-03	9889	
5	rs1363880	33867062	8.01E-01	ADAMTS12	16	's16955751	80288734	3.15E-01	CMIP	-	6.71E-07	2.54E-01	6.84E-06	9890	
11	rs4237707	15401081	3.42E-01	N/A	17	rs9893316	6226092	6.30E-01	N/A	-	6.72E-07	1.34E-01	1.51E-06	9891	
4	rs7699839	1.55E+08	2.45E-01	N/A	22	rs2017317	43577722	4.18E-01	ARHGAP8	-	6.72E-07	9.28E-01	5.67E-05	9892	
4	rs218269	55105896	4.94E-01	N/A	10	rs7913144	17025772	3.26E-04	CUBN	-	6.72E-07	9.73E-01	8.31E-05	9893	
4	rs218268	55105844	4.94E-01	N/A	10	rs7913144	17025772	3.26E-04	CUBN	-	6.72E-07	9.75E-01	8.22E-05	9894	
5	rs12186500	1.8E+08	6.78E-01	OR2Y1	11	rs2237886	2767307	6.31E-01	KCNQ1	-	6.72E-07	4.86E-01	2.47E-05	9895	
7	rs12668378	53976764	6.25E-01	N/A	11	rs4943997	72137803	1.49E-01	STARD10	-	6.72E-07	7.79E-01	6.65E-04	9896	
11	rs7127839	41969521	4.60E-01	N/A	17	rs4969113	68873429	5.85E-01	SDK2	-	6.72E-07	N/A	N/A	9897	
7	rs17668139	53036313	4.40E-01	N/A	18	rs2169014	8354598	2.87E-01	PTPRM	-	6.72E-07	7.08E-01	6.17E-04	9898	
7	rs17735671	53034984	4.40E-01	N/A	18	rs2169014	8354598	2.87E-01	PTPRM	-	6.72E-07	8.53E-01	4.54E-04	9899	
5	rs7710366	1.14E+08	1.78E-01	KCNN2	10	rs1540957	10228122	7.48E-01	N/A	-	6.72E-07	8.67E-01	2.54E-04	9900	
14	rs1570265	95105664	1.29E-01	N/A	22	rs2051582	35888302	3.16E-01	IL2RB	-	6.72E-07	9.35E-01	3.61E-05	9901	
6	rs9460417	19924836	6.98E-01	N/A	17	rs9898958	52597241	8.45E-01	N/A	-	6.72E-07	9.69E-01	3.88E-05	9902	
1	rs13375867	1E+08	4.39E-01	SASS6	20	rs2022259	5412732	8.55E-01	LOC149837	-	6.72E-07	3.30E-01	7.12E-04	9903	
11	rs883752	44593415	5.75E-01	CD82	14	rs744439	95277543	7.26E-01	N/A	-	6.72E-07	7.53E-01	1.09E-04	9904	
1	rs7418365	9457890	1.94E-01	N/A	1	rs987495	78868169	7.90E-01	IFI44L	-	6.72E-07	9.30E-01	1.44E-04	9905	
8	rs11136431	1778840	7.32E-01	ARHGEF10	19	rs4808883	18915720	8.32E-02	HOMER3	-	6.72E-07	9.84E-01	2.93E-04	9906	
1	rs1329961	85823288	3.32E-01	DDAH1	10	rs7069288	20992966	3.74E-01	N/A	-	6.72E-07	7.05E-01	8.83E-05	9907	
2	rs10490386	1.5E+08	2.60E-02	LYPD6B	14	rs1275689	68882032	3.17E-01	GALNTL1	-	6.72E-07	9.57E-02	9.33E-07	9908	
2	rs16846013	2.12E+08	2.86E-01	ERBB4	13	rs1415707	97294596	1.04E-01	N/A	-	6.72E-07	9.40E-01	8.14E-05	9909	
4	rs10518379	1.22E+08	1.36E-01	PRDM5	12	rs4463925	14153103	3.61E-01	N/A	-	6.73E-07	4.57E-01	4.38E-05	9910	
6	rs9366347	20474041	3.52E-01	N/A	8	rs7839852	28761984	4.66E-01	INTS9	-	6.73E-07	9.31E-01	7.12E-05	9911	
7	rs38536	81629463	5.58E-01	CACN/A2D1	17	's16957494	8490252	8.30E-02	MYH10	-	6.73E-07	1.38E-02	1.25E-07	9912	
7	rs1357887	54379297	8.25E-01	N/A	11	's11025966	21197248	8.19E-01	NELL1	-	6.73E-07	3.21E-01	2.46E-04	9913	
5	rs1549225	1.75E+08	7.03E-01	N/A	18	rs1370827	41812161	3.78E-01	KIAA1632	-	6.73E-07	6.68E-01	9.39E-06	9914	
2	rs17452616	1.16E+08	4.34E-01	DPP10	9	rs2006996	1.17E+08	2.03E-01	N/A	-	6.73E-07	6.78E-01	6.86E-05	9915	
3	rs9830035	1.73E+08	6.63E-01	PLD1	13	rs625052	45700669	6.41E-01	N/A	-	6.73E-07	3.65E-01	2.33E-06	9916	
1	rs333970	1.1E+08	9.57E-01	CSF1	14	's10134155	46895886	8.42E-01	MDGA2	-	6.73E-07	6.92E-01	4.25E-05	9917	
2	rs7597348	2.32E+08	6.17E-02	NMUR1	15	rs8023615	51862838	2.76E-01	N/A	-	6.73E-07	2.69E-01	6.94E-04	9918	
7	rs324383	34737020	8.38E-01	N/A	10	's11257470	6317562	8.80E-01	PFKFB3	-	6.73E-07	4.29E-01	1.92E-05	9919	
9	rs563006	18740076	4.24E-01	ADAMTSL1	14	's17096099	29402713	8.65E-01	PRKD1	-	6.73E-07	4.37E-01	3.05E-06	9920	
6	rs375435	42769382	4.69E-01	UBR2	9	's12552818	1.04E+08	3.09E-02	N/A	-	6.73E-07	6.38E-01	5.41E-04	9921	
14	rs11158751	68101178	8.01E-01	RAD51L1	20	rs1078342	42829628	3.94E-01	RIMS4	-	6.73E-07	5.38E-01	3.12E-05	9922	
2	rs12617902	2.13E+08	9.26E-01	N/A	12	rs4764852	1.01E+08	5.46E-01	DRAM1	-	6.73E-07	1.75E-01	3.65E-06	9923	
1	rs7526237	2.45E+08	1.90E-02	SCCPDH	20	rs2424239	19560584	2.37E-01	SLC24A3	-	6.73E-07	5.95E-01	1.86E-04	9924	
4	rs7681514	1.28E+08	3.01E-01	N/A	18	rs273700	21434903	6.88E-01	N/A	-	6.73E-07	6.84E-01	5.21E-05	9925	
6	rs3873380	31370417	5.25E-01	N/A	18	rs1443005	26237044	6.06E-01	N/A	MHC	6.73E-07	8.19E-01	1.59E-04	9926	
10	rs2394678	71728195	7.85E-01	LRRC20	16	's12918376	55115682	8.71E-01	BBS2	-	6.73E-07	8.65E-01	5.97E-05	9927	
6	rs990060	89131055	3.84E-01	N/A	10	rs1470996	1.26E+08	7.68E-01	FAM53B	-	6.74E-07	3.93E-01	1.46E-05	9928	
3	rs9828099	1.15E+08	5.63E-01	ATP6V1A	12	rs4762758	20505926	3.12E-01	PDE3A	-	6.74E-07	4.21E-01	1.79E-03	9929	
4	rs12509678	1.82E+08	8.36E-01	N/A	19	rs2072382	11013650	6.99E-01	SMARCA4	-	6.74E-07	8.09E-01	1.43E-04	9930	
1	rs1418784	90769062	7.66E-01	N/A	7	rs7795671	1.31E+08	5.20E-01	N/A	-	6.74E-07	3.84E-01	4.23E-04	9931	
3	rs9862823	1.81E+08	5.87E-01	USP13	12	rs954147	71189568	4.27E-01	TRHDE	-	6.74E-07	2.29E-01	8.13E-06	9932	
1	rs10493966	1.02E+08	9.02E-01	OLFM3	5	's10035580	16617901	7.32E-01	FAM134B	-	6.74E-07	3.66E-01	7.98E-06	9933	
3	rs3772796	1.26E+08	9.45E-01	KALRN	11	rs563748	59523075	1.02E-01	N/A	-	6.74E-07	9.37E-01	1.02E-04	9934	
3	rs3772796	1.26E+08	9.45E-01	KALRN	11	rs569225	59522484	1.02E-01	N/A	-	6.74E-07	9.37E-01	1.02E-04	9935	
6	rs9353446	87837443	7.02E-01	CGA	8	rs3829037	1.26E+08	1.57E-01	MTSS1	-	6.74E-07	N/A	N/A	9936	
6	rs9390184	1.45E+08	1.13E-01	UTRN	13	rs9540711	65788311	9.70E-01	PCDH9	-	6.74E-07	7.01E-01	6.32E-05	9937	
9	rs4837357	1.31E+08	6.43E-01	C9orf106	11	rs2202150	88782954	5.55E-01	NOX4	-	6.74E-07	1.09E-01	1.28E-06	9938	
5	rs10068079	66329400	2.19E-01	MAST4	20	rs8121494	45927980	5.59E-01	N/A	-	6.74E-07	1.83E-01	2.69E-03	9939	
2	rs10496205	77347215	7.08E-01	LRRTM4	3	rs7432284	1.88E+08	1.98E-01	DGKG	-	6.74E-07	3.87E-01	4.92E-04	9940	
1	rs3844253	2.4E+08	6.55E-01	N/A	13	rs4491389	68964317	4.87E-01	N/A	-	6.74E-07	4.61E-01	1.10E-05	9941	
6	rs1265110	31227401	8.90E-01	TCF19	6	rs9267649	31932807	1.17E-02	C6orf48	MHC	6.74E-07	5.40E-01	1.11E-05	9942	
3	rs11706042	1.21E+08	6.10E-01	CDGAP	18	's11873104	20566685	1.56E-01	N/A	-	6.74E-07	6.25E-01	2.20E-05	9943	
4	rs6845027	1.79E+08	5.03E-01	N/A	12	rs1427988	20204704	6.90E-01	N/A	-	6.74E-07	5.52E-01	8.55E-05	9944	
9	rs2025391	1.16E+08	5.19E-01	ZNF618	12	rs1063843	1.2E+08	8.62E-01	P2RX4	-	6.74E-07	5.54E-01	3.21E-06	9945	
4	rs550625	1.69E+08	5.16E-01	DDX60	5	rs6861486	1.51E+08	4.06E-01	SPARC	-	6.74E-07	8.19E-02	1.24E-03	9946	
4	rs550625	1.69E+08	5.16E-01	DDX60	5	rs7719521	1.51E+08	4.06E-01	SPARC	-	6.74E-07	8.19E-02	1.24E-03	9947	
4	rs2242233	745025	2.78E-01	PCGF3	12	rs1020461	77095317	4.18E-02	N/AV3	-	6.74E-07	1.57E-01	2.20E-03	9948	
6	rs2274154	33965503	6.13E-01	N/A	8	's11780161	59237988	2.28E-01	FAM110B	MHC	6.74E-07	4.94E-01	9.71E-06	9949	
6	rs2596464	31520940	9.43E-01	HCP5	5	's10043332	3181346	3.07E-01	N/A	MHC	6.74E-07	2.36E-01	6.29E-06	9950	
9	rs3935562	1.2E+08	4.07E-01	N/A	11	rs663703	93699170	1.69E-01	FOLR4	-	6.74E-07	N/A	N/A	9951	
6	rs13210693	1.1E+08	7.04E-01	CCDC162	12	rs7970177	13630255	9.77E-01	GRIN2B	-	6.75E-07	1.44E-01	2.12E-06	9952	
16	rs12445989	50689464	7.18E-01	N/A	16	's11642248	81374764	5.23E-01	CDH13	-	6.75E-07	5.24E-01	3.23E-04	9953	
2	rs10176482	29620651	8.22E-01	ALK	2	rs1870384	71324662	3.02E-02	PAIP2B	-	6.75E-07	1.73E-01	5.21E-03	9954	
5	rs2063184	25338740	7.77E-01	N/A	9	rs823907	1.04E+08	2.07E-01	N/A	-	6.75E-07	3.83E-01	6.22E-06	9955	
15	rs624191	37880127	3.09E-01	FSIP1	16	rs837346	9320854	7.48E-01	N/A	-	6.75E-07	8.60E-01	6.86E-05	9956	
8	rs1434757	69205139	6.73E-01	PREX2	20	rs6098874	53887694	8.96E-01	N/A	-	6.75E-07	8.70E-02	1.15E-06	9957	
8	rs1840795	59384230	3.73E-01	N/A	18	rs9965812	41956731	3.64E-01	HAUS1	-	6.75E-07	5.97E-01	2.27E-04	9958	
6	rs3130696	31351863	1.36E-01	HLA-C	6	rs2253907	31444849	7.98E-01	HLA-B	MHC	6.75E-07	8.49E-01	2.78E-04	9959	
3	rs1969481	54909443	8.95E-01	CACN/A2D3	11	's12577139	1.29E+08	2.98E-04	BARX2	-	6.75E-07	9.29E-01	2.94E-04	9960	
4	rs4834136	1.28E+08	3.28E-01	N/A	18	rs273743	21440273	6.02E-01	N/A	-	6.75E-07	N/A	N/A	9961	
5	rs10038754	67186521	6.42E-01	N/A	8	rs2740940	3858860	1.07E-01	CSMD1	-	6.75E-07	6.09E-01	5.06E-05	9962	
3	rs2643823	27621118	6.63E-01	N/A	4	's10222974	77767192	8.17E-01	SHROOM3	-	6.75E-07	N/A	N/A	9963	
1	rs1287295	2.36E+08	7.98E-01	N/A	3	rs2918226	1.17E+08	8.84E-01	LSAMP	-	6.75E-07	2.41E-01	3.91E-06	9964	
9	rs11144766	77954821	6.42E-01	N/A	11	's11231529	55437912	2.40E-01	OR5W2	-	6.75E-07	3.67E-01	1.03E-03	9965	
6	rs1408442	18448266	1.01E-01	N/A	18	rs4940094	47326799	4.73E-01	N/A	-	6.75E-07	2.36E-01	3.44E-03	9966	

SNP A					SNP B						Interaction P			Ranking	Cluster in top 100 interactions
CHR	SNP	Location	gle locus P va	Gene	CHR	SNP	Location	Single locus P valu	Gene	MHC region	Stage 1	Stage 2	Combined		
6	rs1570682	18447816	1.01E-01	N/A	18	rs4940094	47326799	4.73E-01	N/A	-	6.75E-07	2.72E-01	2.81E-03	9967	
2	rs4261706	2.29E+08	7.85E-01	N/A	4	rs7663484	1.83E+08	1.69E-01	N/A	-	6.75E-07	4.41E-01	2.52E-05	9968	
2	rs4261706	2.29E+08	7.85E-01	N/A	4	rs10520478	1.83E+08	1.69E-01	N/A	-	6.75E-07	4.90E-01	3.40E-05	9969	
4	rs995302	1.64E+08	6.15E-01	N/A	13	rs7331053	22946593	9.15E-01	N/A	-	6.75E-07	1.05E-01	2.69E-03	9970	
6	rs207269	90884890	4.28E-01	N/A	17	rs2412324	45887792	3.39E-01	ACSF2	-	6.75E-07	1.92E-01	3.77E-05	9971	
17	rs4247113	228978	3.41E-01	N/A	22	rs5762713	27331789	9.86E-01	TTC28	-	6.75E-07	6.32E-01	1.90E-04	9972	
17	rs917343	66910901	9.59E-01	N/A	20	rs11908310	31923904	6.53E-01	CHMP4B	-	6.75E-07	9.46E-02	4.15E-03	9973	
5	rs149352	94224367	7.77E-01	MCTP1	8	rs4925813	1.46E+08	3.21E-01	KIAA1688	-	6.75E-07	2.00E-01	9.11E-04	9974	
1	rs428913	1.51E+08	1.35E-01	SPRR2F	7	rs4718885	68527153	5.67E-01	N/A	-	6.75E-07	2.36E-01	8.15E-05	9975	
4	rs1400363	1.04E+08	5.58E-01	N/A	16	rs11075616	64631826	7.21E-01	N/A	-	6.75E-07	7.59E-01	2.24E-04	9976	
6	rs8180555	52323747	1.78E-01	PAQR8	5	rs6887463	10585639	1.73E-01	N/A	-	6.75E-07	9.99E-01	1.72E-04	9977	
1	rs4623759	1.06E+08	5.89E-01	N/A	4	rs4862943	32267666	9.85E-01	N/A	-	6.76E-07	8.24E-01	2.12E-04	9978	
2	rs12712127	1.02E+08	3.78E-01	N/A	3	rs6776532	1.49E+08	8.34E-01	N/A	-	6.76E-07	N/A	N/A	9979	
5	rs6451392	38625773	8.09E-01	LIFR	19	rs8112157	7762561	8.90E-01	CLEC4GP1	-	6.76E-07	7.58E-01	2.49E-04	9980	
6	rs9342488	66395599	5.83E-02	EYS	18	rs1944572	44120241	3.35E-01	N/A	-	6.76E-07	3.51E-01	4.57E-04	9981	
6	rs9354270	66397310	5.83E-02	EYS	18	rs1944572	44120241	3.35E-01	N/A	-	6.76E-07	3.51E-01	4.57E-04	9982	
3	rs4683070	45174624	4.12E-01	CDCP1	20	rs6135128	14290719	4.45E-02	MACROD2	-	6.76E-07	6.33E-01	4.28E-05	9983	
9	rs10780262	80652948	6.91E-01	N/A	9	rs4448343	97306191	1.13E-01	PTCH1	-	6.77E-07	2.77E-01	5.43E-06	9984	
8	rs2653416	17770143	5.19E-01	FGL1	16	rs208625	22836323	6.40E-02	HS3ST2	-	6.77E-07	5.79E-01	6.74E-05	9985	
3	rs10514728	71950859	1.52E-01	N/A	11	rs1303932	40333204	9.59E-01	N/A	-	6.77E-07	2.67E-01	3.51E-03	9986	
2	rs1905925	1.41E+08	9.36E-01	LRP1B	4	rs7672552	23620706	1.34E-01	N/A	-	6.77E-07	6.21E-01	3.03E-04	9987	
2	rs6430913	1.41E+08	9.36E-01	LRP1B	4	rs7672552	23620706	1.34E-01	N/A	-	6.77E-07	6.21E-01	3.03E-04	9988	
5	rs4835942	1.27E+08	9.25E-01	FLJ33630	7	rs2969078	2445555	6.51E-01	CHST12	-	6.77E-07	3.72E-01	1.85E-03	9989	
4	rs7680050	1.85E+08	9.17E-01	N/A	8	rs6983185	1.32E+08	5.53E-01	N/A	-	6.77E-07	6.24E-01	5.48E-05	9990	
6	rs6902731	75274664	2.02E-01	N/A	11	rs10750171	1.2E+08	7.13E-01	POU2F3	-	6.77E-07	8.63E-01	3.32E-05	9991	
3	rs7628262	1.35E+08	1.64E-01	BFSP2	4	rs2042630	1.42E+08	8.40E-02	RNF150	-	6.77E-07	4.74E-01	6.11E-04	9992	
2	rs4047536	44232458	9.70E-01	PPM1B	16	rs17266222	13457361	3.98E-02	N/A	-	6.77E-07	5.44E-01	1.74E-03	9993	
9	rs7048503	79659381	8.79E-01	GN/AQ	17	rs8073077	76809616	1.48E-01	AZI1	-	6.77E-07	8.55E-01	2.13E-04	9994	
5	rs3805525	31437710	4.89E-01	RN/ASEN	20	rs1012891	7928390	3.29E-01	TMX4	-	6.77E-07	1.19E-01	2.57E-03	9995	
2	rs1317824	540644	4.10E-01	N/A	18	rs812573	984149	5.88E-01	N/A	-	6.77E-07	6.78E-01	2.82E-05	9996	
10	rs11201128	86370207	7.10E-01	N/A	20	rs6048810	23394429	3.23E-01	CST11	-	6.77E-07	9.18E-03	2.21E-07	9997	
8	rs11989948	5159862	1.24E-01	N/A	12	rs3825181	1.28E+08	7.31E-01	SLC15A4	-	6.77E-07	6.72E-01	4.82E-04	9998	
14	rs4900876	48493149	9.10E-01	N/A	16	rs10514506	79278578	9.46E-01	CDYL2	-	6.77E-07	9.14E-01	2.69E-05	9999	
3	rs9854853	1.28E+08	9.36E-01	N/A	10	rs1660627	34510310	7.97E-01	N/A	-	6.77E-07	N/A	N/A	10000	
