# Supplementary material for: How Genome-Wide SNP-SNP Interactions Relate to Nasopharyngeal Carcinoma Susceptibility
Source: PLoS One. 2013 Dec 23;8(12):e83034. doi: 10.1371/journal.pone.0083034 (PMC3871583; doi:10.1371/journal.pone.0083034)
Supplement: Table S2 — Suggestive interactions associated with NPC susceptibility. (PDF) [file pone.0083034.s006.pdf]

Table S2. Suggestive interactions associated with NPC susceptibility

Ranking[a]	Region A									Region B									Interaction P value						Region	LD[d]	
	CHR	SNP	position	Minor Allele	MAF in Case	MAF in Control	Major Allele	Single Locus P[b]	Nearest Gene	CHR	SNP	Position	Minor Allele	MAF in Case	MAF in Control	Major Allele	Single Locus P[b]	Nearst Gene	Stage 1	Permutation[c]	Stage 2	Permutation[c]	Combined	Permutation[c]		D'	r2
984	7	rs2237353	28,798,214	C	0.26	0.27	A	5.03E-01	CREB5	17	rs1607979	51,333,509	G	0.25	0.25	A	3.78E-01	KIF2B	9.96E-08	1.00E-04	5.87E-04	9.00E-04	9.86E-11	1.00E-04	1		
2574	7	rs2237361	28,832,297	G	0.27	0.27	A	3.48E-01	CREB5	17	rs1607979	51,333,509	G	0.25	0.25	A	3.78E-01	KIF2B	2.25E-07	1.00E-04	1.60E-03	1.80E-03	7.44E-10	1.00E-04	1		
66	7	rs6460664	71,000,614	C	0.21	0.19	A	2.18E-01	WBSCR17	9	rs2300932	123,770,436	A	0.18	0.18	C	6.11E-01	C5	9.63E-09	1.00E-04	9.76E-03	8.60E-03	8.86E-10	1.00E-04	2		
109	7	rs6460671	71,020,307	G	0.22	0.20	A	2.65E-01	WBSCR17	9	rs2300932	123,770,436	A	0.18	0.18	C	6.11E-01	C5	1.47E-08	1.00E-04	8.46E-03	6.70E-03	1.49E-09	1.00E-04	2		
214	7	rs6460671	71,020,307	G	0.22	0.20	A	2.65E-01	WBSCR17	9	rs3789311	123,938,998	G	0.18	0.19	A	7.66E-01	CNTRL	2.67E-08	1.00E-04	8.49E-03	7.00E-03	2.68E-09	1.00E-04	2		
2376	4	rs6821696	117,226,309	A	0.30	0.31	G	2.49E-01	MIR1973	10	rs1380439	127,768,110	A	0.43	0.43	G	6.89E-01	ADAM12	2.12E-07	1.00E-04	8.37E-04	8.00E-04	8.90E-10	1.00E-04	3		
300	2	rs10933155	227,686,907	A	0.25	0.22	G	5.33E-01	RHBDD1	21	rs1888469	39,899,504	A	0.48	0.49	G	9.91E-01	ERG	3.67E-08	1.00E-04	1.45E-03	1.10E-03	1.04E-09	1.00E-04	4		
388	7	rs10487781	14,869,205	G	0.41	0.44	A	4.38E-01	DGKB	7	rs17154507	107,477,267	A	0.35	0.35	G	8.30E-01	DLD	4.58E-08	1.00E-04	2.35E-03	1.60E-03	1.43E-09	1.00E-04	5		
4627	9	rs1332173	25,499,778	G	0.38	0.39	A	8.01E-01	TUSC1	16	rs2127065	7,201,422	A	0.35	0.33	C	3.49E-01	RBFOX1	3.64E-07	1.00E-04	4.24E-04	4.00E-04	1.76E-09	1.00E-04	6		
5505	9	rs1360523	25,499,778	A	0.38	0.39	C	7.55E-01	TUSC1	16	rs2127065	7,201,422	A	0.35	0.33	C	3.49E-01	RBFOX1	4.20E-07	1.00E-04	5.57E-04	6.00E-04	2.69E-09	1.00E-04	6		
4561	9	rs1591029	25,500,488	G	0.38	0.39	A	8.02E-01	TUSC1	16	rs2127065	7,201,422	A	0.35	0.33	C	3.49E-01	RBFOX1	3.60E-07	1.00E-04	6.46E-04	7.00E-04	2.69E-09	1.00E-04	6		
7908	9	rs1591029	25,499,081	G	0.38	0.39	A	7.55E-01	TUSC1	16	rs899305	7,200,563	G	0.37	0.35	A	2.91E-01	RBFOX1	5.61E-07	1.00E-04	2.42E-03	2.60E-03	1.52E-08	1.00E-04	6		
9458	9	rs1360523	25,499,778	A	0.38	0.39	C	8.02E-01	TUSC1	16	rs899305	7,200,563	G	0.37	0.35	A	2.91E-01	RBFOX1	6.47E-07	1.00E-04	2.17E-03	2.30E-03	1.52E-08	1.00E-04	6		
7988	9	rs1332173	25,500,488	G	0.38	0.39	A	8.01E-01	TUSC1	16	rs899305	7,200,563	G	0.37	0.35	A	2.91E-01	RBFOX1	5.66E-07	1.00E-04	2.42E-03	2.60E-03	1.54E-08	1.00E-04	6		
2517	2	rs6726261	25,153,986	G	0.34	0.33	A	4.51E-01	ADCY3	9	rs11140659	87,162,611	A	0.42	0.43	G	4.23E-01	NTRK2	2.21E-07	1.00E-04	3.85E-03	3.30E-03	1.92E-09	1.00E-04	7		
6163	2	rs7589636	57,690,033	A	0.45	0.44	G	5.47E-01	VRK2	2	rs730402	60,094,705	A	0.38	0.45	G	2.73E-02	MIR4432	4.59E-07	1.00E-04	1.47E-03	1.20E-03	2.11E-09	1.00E-04	8		
2409	2	rs6545648	57,657,943	A	0.41	0.40	G	3.54E-01	VRK2	2	rs730402	60,094,705	A	0.38	0.45	G	2.73E-02	MIR4432	2.14E-07	1.00E-04	3.61E-03	2.60E-03	2.72E-09	1.00E-04	8		
3985	2	rs13410950	57,653,054	G	0.41	0.40	A	3.52E-01	VRK2	2	rs730402	60,094,705	A	0.38	0.45	G	2.73E-02	MIR4432	3.23E-07	1.00E-04	3.53E-03	2.60E-03	4.04E-09	1.00E-04	8		
7221	2	rs6545648	57,657,943	A	0.41	0.40	G	3.54E-01	VRK2	2	rs7566185	60,091,600	G	0.39	0.45	A	3.77E-02	MIR4432	5.20E-07	1.00E-04	5.76E-03	4.00E-03	1.07E-08	1.00E-04	8		
8827	2	rs13410950	57,653,054	G	0.41	0.40	A	3.52E-01	VRK2	2	rs6545760	60,101,211	G	0.37	0.45	A	2.82E-02	MIR4432	6.12E-07	1.00E-04	9.97E-03	7.30E-03	2.08E-08	1.00E-04	8		
581	1	rs4660176	41,293,649	A	0.26	0.27	G	3.08E-01	KCNQ4	7	rs740576	153,586,831	A	0.23	0.23	G	8.98E-01	DPP6	6.33E-08	1.00E-04	2.97E-03	3.70E-03	2.11E-09	1.00E-04	9		
9105	5	rs4571472	15,721,360	A	0.20	0.18	G	3.10E-01	FBXL7	10	rs7075349	64,427,649	G	0.31	0.32	A	2.74E-01	ZNF365	6.28E-07	1.00E-04	1.93E-03	1.60E-03	3.38E-09	1.00E-04	10		
274	6	rs9403288	141,672,900	G	0.30	0.28	A	3.94E-01	AK097143	10	rs1904382	65,550,685	G	0.23	0.20	A	1.59E-01	REEP3	3.32E-08	1.00E-04	8.32E-03	7.90E-03	4.94E-09	1.00E-04	11		
273	6	rs9399364	141,692,825	G	0.30	0.28	A	3.94E-01	AK097143	10	rs1904382	65,550,685	G	0.23	0.20	A	1.59E-01	REEP3	3.32E-08	1.00E-04	8.32E-03	7.90E-03	4.94E-09	1.00E-04	11		
4782	12	rs6539598	82,290,233	G	0.32	0.31	A	3.92E-01	PPFIA2	17	rs7208838	30,468,566	G	0.22	0.22	A	3.23E-01	RHOT1	3.74E-07	1.00E-04	4.27E-03	4.40E-03	5.28E-09	1.00E-04	12		
5797	12	rs10862392	82,318,628	A	0.32	0.31	G	3.70E-01	PPFIA2	17	rs7208838	30,468,566	G	0.22	0.22	A	3.23E-01	RHOT1	4.39E-07	1.00E-04	4.27E-03	4.40E-03	6.12E-09	1.00E-04	12		
5617	12	rs6539598	82,290,233	G	0.32	0.31	A	3.92E-01	PPFIA2	17	rs7210733	30,567,339	G	0.22	0.22	A	4.04E-01	RHOT1	4.28E-07	1.00E-04	8.12E-03	8.40E-03	1.22E-08	1.00E-04	12		
6849	12	rs10862392	82,318,628	A	0.32	0.31	G	3.70E-01	PPFIA2	17	rs7210733	30,567,339	G	0.22	0.22	A	4.04E-01	RHOT1	5.01E-07	1.00E-04	8.12E-03	8.40E-03	1.41E-08	1.00E-04	12		
2515	5	rs37181	115,630,004	C	0.41	0.41	A	7.61E-01	COMMD10	10	rs512064	84,623,726	A	0.36	0.37	C	1.69E-01	NRG3	2.20E-07	1.00E-04	4.84E-03	3.80E-03	7.30E-09	1.00E-04	13		
3934	5	rs712572	115,634,127	G	0.41	0.41	A	5.19E-01	COMMD10	10	rs512064	84,623,726	A	0.36	0.37	C	3.18E-01	NRG3	3.19E-07	1.00E-04	4.84E-03	3.80E-03	9.78E-09	1.00E-04	13		
4611	5	rs37181	115,630,004	C	0.41	0.41	A	5.66E-01	COMMD10	10	rs560083	84,614,844	A	0.34	0.36	G	4.67E-01	NRG3	3.63E-07	1.00E-04	7.13E-03	5.40E-03	1.51E-08	1.00E-04	13		
7110	5	rs712572	115,634,127	G	0.41	0.41	A	5.19E-01	COMMD10	10	rs560083	84,614,844	A	0.34	0.36	G	4.67E-01	NRG3	5.14E-07	1.00E-04	7.13E-03	5.40E-03	2.00E-08	1.00E-04	13		
5503	7	rs6979066	82,422,937	G	0.47	0.48	A	1.50E-01	PCLO	16	rs1861315	52,302,072	A	0.41	0.41	C	5.32E-01	Mir_548	4.20E-07	1.00E-04	7.10E-03	6.10E-03	1.08E-08	1.00E-04	14		
3591	10	rs1413615	99,803,197	G	0.19	0.20	A	9.45E-01	CRTAC1	13	rs2018259	53,523,952	A	0.40	0.42	G	7.74E-02	OLFM4	2.95E-07	1.00E-04	2.62E-03	1.80E-03	1.10E-08	1.00E-04	15		
4632	13	rs982790	91,232,023	G	0.43	0.44	A	3.82E-01	BC038529	13	rs816960	108,522,521	A	0.48	0.49	G	2.38E-01	FAM155A	3.64E-07	1.00E-04	4.05E-03	4.20E-03	2.78E-08	1.00E-04	16		
5645	9	rs10974873	4,943,704	G	0.35	0.32	A	4.63E-01	JAK2	16	rs12932445	73,069,888	G	0.36	0.36	A	2.79E-01	ZFHX3	4.30E-07	1.00E-04	9.20E-03	8.10E-03	1.19E-08	1.00E-04	17		
8431	1	rs12120788	157,237,624	C	0.13	0.11	A	4.86E-01	ETV3	18	rs489631	21,989,024	G	0.34	0.35	A	7.04E-01	IMPACT	5.91E-07	1.00E-04	5.14E-03	4.80E-03	1.43E-08	1.00E-04	18		
9186	4	rs918402	181,691,988	C	0.30	0.31	A	2.52E-01	LINC00290	13	rs9534199	46,291,526	A	0.51	0.49	G	6.31E-01	SPERT	6.32E-07	1.00E-04	1.66E-03	9.00E-04	1.50E-08	1.00E-04	19		
3089	7	rs4728351	75,364,117	A	0.27	0.28	G	2.44E-01	HIP1	8	rs10112728	68,574,359	G	0.12	0.17	A	7.10E-02	CPA6	2.65E-07	1.00E-04	5.82E-03	5.60E-03	1.51E-08	1.00E-04	20		
1729	7	rs757359	75,367,376	G	0.28	0.28	A	3.05E-01	HIP1	8	rs10112728	68,574,359	G	0.12	0.17	A	7.10E-02	CPA6	1.61E-07	1.00E-04	8.93E-03	8.30E-03	1.93E-08	1.00E-04	20		
5490	2	rs17442753	19,646,340	A	0.21	0.20	G	6.90E-01	OSR1	12	rs1683163	25,233,618	A	0.30	0.34	G	2.18E-01	LRMP	4.19E-07	1.00E-04	6.54E-03	7.10E-03	1.79E-08	1.00E-04	21		
8036	1	rs2997338	41,872,328	C	0.37	0.43	A	3.21E-02	EDN2	5	rs11960332	180,071,276	G	0.46	0.45	A	6.87E-01	FLT4	5.69E-07	1.00E-04	5.34E-03	4.80E-03	1.97E-08	1.00E-04	22		
5296	13	rs2761367	32,979,647	A	0.32	0.27	C	1.63E-02	N4BP2L1	17	rs8066210	10,022,443	G	0.36	0.38	A	3.42E-01	GAS7	4.07E-07	1.00E-04	2.95E-03	2.20E-03	2.03E-08	1.00E-04	23		
7726	4	rs17258848	127,536,771	G	0.23	0.26	A	9.43E-01	INTU	15	rs11637659	67,442,386	A	0.30	0.31	G	4.59E-01	SMAD3	5.53E-07	1.00E-04	1.98E-03	1.80E-03	2.24E-08	1.00E-04	24		
3856	6	rs6934929	65,453,785	G	0.25	0.28	A	6.41E-01	EYS	21	rs7276462	31,429,636	A	0.13	0.14	G	1.67E-01	GRIK1	3.15E-07	1.00E-04	7.15E-03	4.10E-03	2.49E-08	1.00E-04	25		
1849	6	rs6907357	96,111,622	C	0.13	0.11	A	7.07E-01	MANEA	22	rs9617490	49,059,556	A	0.52	0.43	G	4.81E-03	FAM19A5	1.72E-07	1.00E-04	6.38E-03	4.50E-03	1.17E-08	1.00E-04	26		
5580	5	rs6579767	149,406,271	G	0.25	0.26	A	3.01E-01	HMGXB3	10	rs2505366	32,659,387	A	0.29	0.31	G	6.73E-01	EPC1	4.26E-07	1.00E-04	7.85E-03	6.60E-03	3.06E-08	1.00E-04	27		
5936	1	rs2776747	24876383	G	0.24	0.24	A	9.07E-01	C1orf130	2	rs17006285	70980886	G	0.32	0.32	A	9.17E-01	ADD2	4.46E-07	1.00E-04	6.83E-03	6.30E-03	3.32E-08	1.00E-04	28		
2407	12	rs10128762	74,681,897	A	0.27	0.29	G	7.81E-01	LOC10050737	21	rs2824444	19,063,193	A	0.36	0.36	G	2.72E-01	BTG3	2.14E-07	1.00E-04	1.56E-03	1.80E-03	3.39E-08	1.00E-04	29		
3250	12	rs10128762	74,681,897	A	0.27	0.29	G	7.81E-01	LOC10050737	21	rs2824447	19,065,455	A	0.36	0.36	G	2.47E-01	BTG3	2.73E-07	1.00E-04	1.56E-03	1.80E-03	4.24E-08	1.00E-04	29		
454	12	rs10128762	74,681,897	A	0.27	0.29	G	7.81E-01	LOC10050737	21	rs928250	19,055,529	A	0.39	0.38	C	4.43E-01	BTG3	4.97E-08	1.00E-04	5.70E-03	5.70E-03	9.21E-08	1.00E-04	29		
9395	5	rs10057387	172,230,002	G	0.48	0.48	A	3.26E-01	DUSP1	19	rs2111505	32,779,618	A	0.34	0.32	C	5.00E-02	ZNF507	6.44E-07	1.00E-04	6.75E-03	5.80E-03	3.92E-08	1.00E-04	30		
8272	3	rs9867490	118,364,856	C	0.40	0.42	A	3.21E-01	EU250752	7	rs2894771	57,417,568	G	0.47	0.47	A	8.87E-01	MIR3147	5.82E-07	1.00E-04	6.72E-03	7.00E-03	4.09E-08	1.00E-04	31		
7624	2	rs12477036	230,034,082	G	0.15	0.17	A	4.94E-01	PID1	3	rs1352409	3,810,973	G	0.27	0.28	A	1.45E-01	SUMF1	5.47E-07	1.00E-04	8.99E-03	7.40E-03	5.04E-08	1.00E-04	32		
9798	6	rs2235718	1,607,603	A	0.26	0.23	G	1.38E-01	FOXC1	1	rs11102213	109,221,612	G	0.20	0.17	A	4.65E-01	PRPF38B	6.66E-07	1.00E-04	7.77E-03	8.90E-03	4.57E-08	1.00E-04	33		
3129	7	rs6973203	147,868,281	A	0.23	0.23	G	1.84E-01	CNTN/AP2	19	rs376620	6,945,861	G	0.28	0.29	A	5.37E-01	EMR1	2.67E-07	1.00E-04	9.69E-03	8.50E-03	7.76E-08	1.00E-04	34		
7293	12	rs1354156	115,135,430	G	0.14	0.16	A	1.51E-02	TBX3	17	rs11655550	37,610,123	G	0.23	0.26	A	5.95E-02	MED1	5.25E-07	1.00E-04	8.48E-03	8.30E-03	8.85E-08	1.00E-04	35		
9345	4	rs1500453	22,293,242	A	0.31	0.34	G	9.12E-01	N/A	17	rs10512514	65,211,876	A	0.30	0.31	C	1.53E-01	HELZ	6.41E-07	1.00E-04	9.24E-03	8.80E-03	2.02E-07	1.00E-04	36		
9260	10	rs11201128	86,380,227	G	0.37	0.38	A	7.10E-01	FAM190B	20	rs6114101	23,446,322	A	0.29	0.28	G	3.37E-01	CST11	6.36E-07	1.00E-04	9.18E-03	8.90E-03	2.08E-07	1.00E-04	37		
9997	10	rs11201128	86,380,227	G	0.37	0.38	A	7.10E-01	FAM190B	20	rs6048810	23,446,429	A	0.29	0.28	G	3.23E-01	CST11	6.77E-07	1.00E-04	9.18E-03	8.90E-03	2.21E-07	1.00E-04	37		
**MHC region**																											
5620	6	rs2523849	31,025,051	G	0.31	0.27	A	4.48E-01	HCG22	6	rs4947296	31,058,178	G	0.30	0.23	A	1.90E-01	C6orf15	4.28E-07	1.00E-04	1.97E-03	1.60E-03	8.42E-09	1.00E-04	38	0.141	0.016
5568	6	rs2523849	31,025,051	G	0.31	0.27	A	4.48E-01	HCG22	6	rs9380215	31,049,655	A	0.30	0.23	G	9.82E-01	C6orf15	4.25E-07	1.00E-04	1.99E-03	1.60E-03	8.56E-09	1.00E-04	38	0.139	0.016
9849	6	rs2523849	31,025,051	G	0.31	0.27	A	4.48E-01	HCG22	6	rs2233984	31,079,264	A	0.30	0.23	G	4.81E-01	C6orf15	6.69E-07	1.00E-04	1.99E-03	1.60E-03	1.32E-08	1.00E-04	38	0.136	0.015
8789	6	rs2523864	31,018,546	A	0.31	0.29	G	9.97E-01	HCG22	6	rs9380215	31,049,655	A	0.30	0.23	G	9.82E-01	C6orf15	6.10E-07	1.00E-04	6.51E-03	6.30E-03	3.66E-08	1.00E-04	38	0.137	0.014
5471	6	rs879882	31,139,452	G	0.43	0.44	A	7.61E-01	POU5F1	6	rs7770216	31,340,611	A	0.38	0.37	G	1.71E-01	HLA-B	4.18E-07	1.00E-04	9.59E-03	9.90E-03	6.91E-08	1.00E-04	39	0.090	0.006
3082	6	rs7761965	31,273,495	A	0.38	0.37	G	8.90E-01	HLA-B	6	rs2596501	31,321,211	A	0.41	0.43	G	4.68E-02	HLA-B	2.64E-07	1.00E-04	7.40E-03	7.20E-03	1.81E-08	1.00E-04	39	0.232	0.042

[a] *P*-value ranking in the Discovery analysis
[b] Single-locus logistic regression association *p*-value from GWAS analysis
[c] Permutation *p*-values calculated after 10,000 permutation test.
[d] LD value calculated by combined control samples
