# Supplementary material for: How Genome-Wide SNP-SNP Interactions Relate to Nasopharyngeal Carcinoma Susceptibility
Source: PLoS One. 2013 Dec 23;8(12):e83034. doi: 10.1371/journal.pone.0083034 (PMC3871583; doi:10.1371/journal.pone.0083034)
Supplement: Table S3 — Suggestive regions associated with NPC susceptibility. (PDF) [file pone.0083034.s007.pdf]

Wen-Hui Su, Chang Gung University, whsu@mail.cgu.edu.tw

Table S3. Suggestive regions associated with NPC susceptibility

Region A									Region B									Interaction P values						Region	Significant Interaction Number
Chr.	Start bp	End bp	SNP[a]	Location	Distance	Note[a]	Gene	Single Locus *P*[b]	Chr.	Start bp	End bp	SNP	Location	Distance	Note[a]	Gene	Single locus *P*[b]	Stage 1	Permutation[d]	Stage 2	Permputation[d]	Combined	Permutation[d]		
7	28,798,214	28,832,297	rs2237353	intron			*CREB5*	5.03E-01	17	51,333,509	51,333,509	rs1607979	upstream	566,730		*KIF2B*	3.78E-01	9.96E-08	1.00E-04	5.87E-04	9.00E-04	9.86E-11	1.00E-04	1	2
7	71,000,614	71,020,307	rs6460664	intron			*WBSCR17*	2.18E-01	9	123,770,436	123,938,998	rs2300932	intron			*C5*	6.11E-01	9.63E-09	1.00E-04	9.76E-03	8.60E-03	8.86E-10	1.00E-04	2	3
4	117,226,309	117,226,309	rs6821696	downstream	5,428		*MIR1973*	2.49E-01	10	127,768,110	127,768,110	rs1380439	intron			*ADAM12*	6.89E-01	2.12E-07	1.00E-04	8.37E-04	8.00E-04	8.90E-10	1.00E-04	3	1
2	227,686,907	227,686,907	rs10933155	upstream	13,764		*RHBDD1*	5.33E-01	21	39,899,504	39,899,504	rs1888469	intron			*ERG*	9.91E-01	3.67E-08	1.00E-04	1.45E-03	1.10E-03	1.04E-09	1.00E-04	4	1
7	14,869,205	14,869,205	rs10487781	intron			*DGKB*	4.38E-01	7	107,477,267	107,477,267	rs17154507	upstream	54,319	DNAse I hyper 38, TF Chip-Seq site	*DLD*	8.30E-01	4.58E-08	1.00E-04	2.35E-03	1.60E-03	1.43E-09	1.00E-04	5	1
9	25,499,081	25,500,488	rs1332173	upstream	175,899		*TUSC1*	8.01E-01	16	7,200,563	7,200,563	rs2127065	intron			*RBFOX1*	3.49E-01	3.64E-07	1.00E-04	4.24E-04	4.00E-04	1.76E-09	1.00E-04	6	6
2	25,153,986	25,153,986	rs6726261	upstream	11,931	DNAse I hyper 43, TF Chip-Seq,	*ADCY3*	4.51E-01	9	87,162,611	87,162,611	rs11140659	upstream	120,855		*NTRK2*	4.23E-01	2.21E-07	1.00E-04	3.85E-03	3.30E-03	1.92E-09	1.00E-04	7	1
2	57,653,054	57,690,033	rs7589636	upstream	444,753		*VRK2*	5.47E-01	2	60,077,001	60,101,211	rs730402	upstream	513,286		*MIR4432*	2.73E-02	4.59E-07	1.00E-04	1.47E-03	1.20E-03	2.11E-09	1.00E-04	8	5
1	41,293,649	41,293,649	rs4660176	intron			*KCNQ4*	3.08E-01	7	153,586,831	153,586,831	rs740576	intron			*DPP6*	8.98E-01	6.33E-08	1.00E-04	2.97E-03	3.70E-03	2.11E-09	1.00E-04	9	1
5	15,721,360	15,721,360	rs4571472	intron			*FBXL7*	3.10E-01	10	64,427,649	64,427,649	rs7075349	intron		DNaseI hyper 3	*ZNF365*	2.74E-01	6.28E-07	1.00E-04	1.93E-03	1.60E-03	3.38E-09	1.00E-04	10	1
6	141,692,825	141,672,900	rs9399364	downstream	209,954		*AK097143*	3.94E-01	10	65,550,685	65,550,685	rs1904382	downstream	165,802		*REEP3*	1.59E-01	3.32E-08	1.00E-04	8.32E-03	7.90E-03	4.94E-09	1.00E-04	11	2
12	82,290,233	82,318,628	rs6539598	upstream	137,947		*PPFIA2*	3.92E-01	17	30,468,566	30,567,339	rs7208838	upstream	907	DNaseI hyper 122, TF CHIP-seq	*RHOT1*	3.23E-01	3.74E-07	1.00E-04	4.27E-03	4.40E-03	5.28E-09	1.00E-04	12	4
5	115,630,004	115,634,127	rs37181	upstream	1,026		*COMMD10*	7.61E-01	10	84,614,844	84,623,726	rs512064	intron			*NRG3*	1.69E-01	2.20E-07	1.00E-04	4.84E-03	3.80E-03	7.30E-09	1.00E-04	13	4
7	82,422,937	82,422,937	rs6979066	intron			*PCLO*	1.50E-01	16	52,302,072	52,302,072	rs1861315	upstream	12,611		*Mir_548*	5.32E-01	4.20E-07	1.00E-04	7.10E-03	6.10E-03	1.08E-08	1.00E-04	14	1
10	99,803,197	99,803,197	rs1413615	upstream	12,612		*CRTAC1*	9.45E-01	13	53,523,952	53,523,952	rs2018259	upstream	78,924		*OLFM4*	7.74E-02	2.95E-07	1.00E-04	2.62E-03	1.80E-03	1.10E-08	1.00E-04	15	1
6	96,111,622	96,111,622	rs6907357	downstream	54,294		*MANEA*	3.82E-01	22	49,059,556	49,059,556	rs9617490	intron			*FAM19A5*	2.38E-01	1.72E-07	1.00E-04	6.38E-03	4.20E-03	1.17E-08	1.00E-04	16	1
9	4,943,704	4,943,704	rs10974873	upstream	41,382		*JAK2*	4.63E-01	16	73,069,888	73,069,888	rs12932445	intron			*ZFHX3*	2.79E-01	4.30E-07	1.00E-04	9.20E-03	8.10E-03	1.19E-08	1.00E-04	17	1
1	157,237,624	157,237,624	rs12120788	upstream	129,247		*ETV3*	4.86E-01	18	21,989,024	21,989,024	rs489631	upstream	17,585		*IMPACT*	7.04E-01	5.91E-07	1.00E-04	5.14E-03	4.80E-03	1.43E-08	1.00E-04	18	1
4	181,691,988	181,691,988	rs918402	downstream	293,255		*LINC00290*	2.52E-01	13	46,291,526	46,291,526	rs9534199	downstream	2,833		*SPERT*	6.31E-01	6.32E-07	1.00E-04	1.66E-03	9.00E-04	1.50E-08	1.00E-04	19	1
7	75,364,117	75,367,376	rs4728351	intron		DNaseIhyper 86, TF CHIP-seq	*HIP1*	2.44E-01	8	68,574,359	68,574,359	rs10112728	intron			*CPA6*	7.10E-02	2.65E-07	1.00E-04	5.82E-03	5.60E-03	1.51E-08	1.00E-04	20	2
2	19,646,340	19,646,340	rs17442753	upstream	87,968		*OSR1*	6.90E-01	12	25,233,618	25,233,618	rs1683163	intron			*LRMP*	2.18E-01	4.19E-07	1.00E-04	6.54E-03	7.10E-03	1.79E-08	1.00E-04	21	1
1	41,872,328	41,872,328	rs2997338	downstream	72,118		*EDN2*	3.21E-02	5	180,071,276	180,071,276	rs11960332	intron			*FLT4*	6.87E-01	5.69E-07	1.00E-04	5.34E-03	4.80E-03	1.97E-08	1.00E-04	22	1
13	32,979,647	32,979,647	rs2761367	intron			*N4BP2L1*	1.63E-02	17	10,022,443	10,022,443	rs8066210	intron			*GAS7*	3.42E-01	4.07E-07	1.00E-04	2.95E-03	2.20E-03	2.03E-08	1.00E-04	23	1
4	127,536,771	127,536,771	rs17258848	upstream	1,017,319		*INTU*	9.43E-01	15	67,442,386	67,442,386	rs11637659	intron		H3K27Ac, TF CHIP-seq	*SMAD3*	4.59E-01	5.53E-07	1.00E-04	1.98E-03	1.80E-03	2.24E-08	1.00E-04	24	1
6	65,453,785	65,453,785	rs6934929	intron			*EYS*	6.41E-01	21	31,429,636	31,429,636	rs7276462	upstream	117,354		*GRIK1*	1.67E-01	3.15E-07	1.00E-04	7.15E-03	4.10E-03	2.49E-08	1.00E-04	25	1
13	91,232,023	91,232,023	rs982790	downstream	44,428		*BC038529*	7.07E-01	13	108,522,521	108,522,521	rs816960	intron			*FAM155A*	4.81E-03	3.64E-07	1.00E-04	4.05E-03	4.50E-03	2.78E-08	1.00E-04	26	1
5	149,406,271	149,406,271	rs6579767	coding-synon,			*HMGXB3*	3.01E-01	10	32,659,387	32,659,387	rs2505366	intron			*EPC1*	6.73E-01	4.26E-07	1.00E-04	7.85E-03	6.60E-03	3.06E-08	1.00E-04	27	1
1	24,876,383	24,876,383	rs2776747	upstream	6,183		*C1orf130*	9.07E-01	2	70,980,886	70,980,886	rs17006285	intron			*ADD2*	9.17E-01	4.46E-07	1.00E-04	6.83E-03	6.30E-03	3.32E-08	1.00E-04	28	1
12	74,681,897	74,681,897	rs10128762	intron			*LOC100507377*	7.81E-01	21	19,055,529	19,065,455	rs2824444	upstream	77,929	H3K27Ac, DNaseI hyperseneitivity	*BTG3*	2.72E-01	2.14E-07	1.00E-04	1.56E-03	1.80E-03	3.39E-08	1.00E-04	29	3
5	172,230,002	172,230,002	rs10057387	upstream	31,799	DNAse I hyper 98, H3K27Ac, CHIP-seq	*DUSP1*	3.26E-01	19	32,779,618	32,779,618	rs2111505	upstream	56,895		*ZNF507*	5.00E-02	6.44E-07	1.00E-04	6.75E-03	5.80E-03	3.92E-08	1.00E-04	30	1
3	118,364,856	118,364,856	rs9867490	intron			*EU250752*	3.21E-01	7	57,417,568	57,417,568	rs2894771	upstream	55,463		*MIR3147*	8.87E-01	5.82E-07	1.00E-04	6.72E-03	7.00E-03	4.09E-08	1.00E-04	31	1
2	230,034,082	230,068,786	rs12478812	intron			*PID1*	4.41E-01	3	3,810,973	3,810,973	rs1352409	downstream	591,856		*SUMF1*	1.45E-01	6.05E-07	1.00E-04	9.79E-03	1.07E-02	4.33E-08	1.00E-04	32	1
6	1,607,603	1,607,603	rs2235718	upstream	3,078	Dnase I hyper 55	*FOXC1*	1.38E-01	1	109,221,612	109,221,612	rs11102213	upstream	13,320	DNAse I hyper 4, CHIP-seq	*PRPF38B*	4.65E-01	6.66E-07	1.00E-04	7.77E-03	8.90E-03	4.57E-08	1.00E-04	33	1
7	147,868,281	147,868,281	rs6973203	intron			*CNTNAP2*	1.84E-01	19	6,945,861	6,951,434	rs376620	downstream	5,397		*EMR1*	5.37E-01	2.67E-07	1.00E-04	9.69E-03	8.50E-03	7.76E-08	1.00E-04	34	1
12	115,135,430	115,135,430	rs1354156	upstream	13,461		*TBX3*	1.51E-02	17	37,610,123	37,610,123	rs11655550	upstream	2,596	CHIP-seq	*MED1*	5.95E-02	5.25E-07	1.00E-04	8.48E-03	8.30E-03	8.85E-08	1.00E-04	35	1
4	22,293,242	22,293,242	rs1500453	downstream	35,747		*LOC100505912*	9.12E-01	17	65,211,876	65,211,876	rs10512514	intron			*HELZ*	1.53E-01	6.41E-07	1.00E-04	9.24E-03	8.80E-03	2.02E-07	1.00E-04	36	1
10	86,380,227	86,380,227	rs11201128	upstream	246,466		*FAM190B*	7.10E-01	20	23,446,322	23,446,429	rs6114101	upstream	12,840		*CST11*	3.37E-01	6.36E-07	1.00E-04	9.18E-03	8.90E-03	2.08E-07	1.00E-04	37	2
**MHC region**																									
6	31,018,546	31,273,495	rs2523849	intron			*HCG22*	4.48E-01	6	31,049,655	31,340,611	rs4947296	downstream	20,822		*C6orf15*	1.90E-01	4.28E-07	1.00E-04	1.97E-03	1.60E-03	8.42E-09	1.00E-04	38	5
6	31,273,495	31,139,452	rs7761965	downstream	48,764		*HLA-B*	8.90E-01	6	31,321,211	31,340,611	rs2596501	downstream	1,298	CHIP-seq	*HLA-B*	4.68E-02	2.64E-07	1.00E-04	7.40E-03	7.20E-03	1.81E-08	1.00E-04	39	4

[a]The most significant interaction pairs in the Discovery analysis.

[b]Transcription related elements annotated by UCSC genome browser (http://genome.ucsc.edu/).

[c] Single-locus ogistic regression association *p* -value from GWAS analysis

[d] Permutation *p*- values calculated after 10,000 permutation test. Permutation *p* -value >0.05 was highlighted with red color.
