# Supplementary material for: How Genome-Wide SNP-SNP Interactions Relate to Nasopharyngeal Carcinoma Susceptibility
Source: PLoS One. 2013 Dec 23;8(12):e83034. doi: 10.1371/journal.pone.0083034 (PMC3871583; doi:10.1371/journal.pone.0083034)
Supplement: Table S4 — LD analysis of SNPs in interacting region. (PDF) [file pone.0083034.s008.pdf]

Table S4. LD analysis of SNPs in interacting region

Cluster*	Region*	SNP1	SNP2	D'	r^2
1	A	rs2237353	rs2237361	0.94	0.851
2	A	rs6460664	rs6460671	1	0.954
2	B	rs2300932	rs3789311	0.966	0.904
6	A	rs1591029	rs1360523	1	1
		rs1591029	rs1332173	1	0.993
		rs1360523	rs1332173	1	0.993
6	B	rs899305	rs2127065	1	0.946
8	A	rs1341095	rs6545648	1	0.993
		rs1341095	rs7589636	0.958	0.761
		rs6545648	rs7589636	0.966	0.768
8	B	rs7566185	rs730402	1	0.993
		rs7566185	rs6545760	0.948	0.862
		rs730402	rs6545760	0.956	0.869
11	A	rs9403288	rs9399364	1	1
12	A	rs6539598	rs1086239	1	0.992
12	B	rs7208838	rs7210733	0.989	0.948
13	A	rs37181	rs712572	1	0.993
13	B	rs560083	rs512064	1	0.909
20	A	rs4728351	rs757359	1	0.992
29	B	rs928250	rs2824444	0.992	0.907
		rs928250	rs2824447	0.992	0.907
		rs2824444	rs2824447	1	1
32	A	rs1247703	rs1247881	0.806	0.64
37	A	rs2523864	rs2523849	0.965	0.886
37	B	rs6114101	rs6048810	1	1
38	A	rs2523864	rs2523849	0.965	0.886
38	B	rs9380215	rs4947296	1	0.99
		rs4947296	rs2233984	1	0.99
		rs9380215	rs2233984	0.99	0.981
39	A	rs879882	rs7761965	0.373	0.108
39	B	rs2596501	rs7770216	0.218	0.02

*Cluster and Region annotated in Supplementary Table S2
