# Supplementary material for: How Genome-Wide SNP-SNP Interactions Relate to Nasopharyngeal Carcinoma Susceptibility
Source: PLoS One. 2013 Dec 23;8(12):e83034. doi: 10.1371/journal.pone.0083034 (PMC3871583; doi:10.1371/journal.pone.0083034)
Supplement: Table S5 — Haplotype analysis of the interaction regions located in the MHC region. (PDF) [file pone.0083034.s009.pdf]

Supplementary Table S5. Haplotype analysis of the interaction regions located in the MHC region

Haplotype					Haplotype frequency[a]				Global association *p*-value		
					Pooled	Control	Case	OR (95% c.i.) [a]	Stage 1	Stage 2	Combined
**Zone I**											
rs2523864	rs2523849	rs9380215	rs4947296	rs2233984							
A	A	G	A	G	0.02	0.02	0.01	0.69 (0.32,1.45)			
A	G	A	G	A	0.06	0.08	0.04	0.56 (0.34,0.93)			
A	G	G	A	G	0.22	0.18	0.26	1.95 (1.49,2.54)			
G	A	A	G	A	0.20	0.14	0.25	2.34 (2.34,3.09)			
G	A	G	A	G	0.49	0.56	0.43	1.00			
Rare					0.01	0.01	0.01	0.55 (0.12,2.48)			
Global association *p*-value									1.03E-06	5.81E-04	8.35E-10
**Zone 2**											
rs879882	rs7761965	rs2596501	rs7770216								
A	A	A	A		0.04	0.05	0.03	0.39 (0.21,0.73)			
A	A	A	C		0.04	0.05	0.03	0.45 (0.24,0.82)			
A	G	A	A		0.03	0.04	0.03	0.77 (0.39,1.53)			
A	G	G	A		0.07	0.08	0.06	0.53 (0.33,0.86)			
A	G	G	C		0.17	0.16	0.18	0.80 (0.56,1.14)			
G	A	A	A		0.02	0.02	0.02	0.70 (0.27,1.80)			
G	A	A	C		0.06	0.08	0.04	0.43 (0.25,0.73)			
G	A	G	A		0.17	0.13	0.21	1.18 (0.84,1.65)			
G	A	G	C		0.03	0.02	0.03	0.94 (0.43,2.07)			
G	G	A	C		0.02	0.02	0.01	0.38 (0.11,1.33)			
G	G	G	A		0.03	0.03	0.03	0.59 (0.30,1.16)			
G	G	G	C		0.10	0.14	0.07	0.39 (0.25,0.61)			
A	G	A	C		0.20	0.16	0.23	1.00			
Rare					0.01	0.01	0.01	0.70 (0.22,2.22)			
Global association *p*-value									1.40E-04	5.77E-02	2.00E-06

[a]Calculated using Combined dataset.
