# Supplementary material for: How Genome-Wide SNP-SNP Interactions Relate to Nasopharyngeal Carcinoma Susceptibility
Source: PLoS One. 2013 Dec 23;8(12):e83034. doi: 10.1371/journal.pone.0083034 (PMC3871583; doi:10.1371/journal.pone.0083034)
Supplement: Table S6 — Interaction P values for SNP pairs identified by other GWAS studies. (PDF) [file pone.0083034.s010.pdf]

Supplementary Table S6. Interaction P values of the SNPs identified in previous GWAS studies

SNP1						SNP2					Interaction p-value		
SNP	Chr.	Position	Nearest Gene	Taiwan GWAS P	China GWAS	SNP	Chr.	Position	Nearest Gene	GWAS P	Stage 1	Stage 2	Combined
rs29232	6	29719410	GABBR1	**7.53E-09**	**3.90E-18**	rs33790	3	45271744	N/A	7.59E-02	**8.92E-05**	6.39E-01	**6.38E-03**
rs29232	6	29719410	GABBR1	**7.53E-09**	**3.90E-18**	rs12706933	7	129895384	MESTIT1	9.95E-01	**2.73E-05**	3.70E-01	**2.11E-04**
rs29232	6	29719410	GABBR1	**7.53E-09**	**3.90E-18**	rs1512406	8	139368347	FAM135B	7.77E-01	**1.37E-06**	8.76E-01	**7.85E-05**
rs29232	6	29719410	GABBR1	**7.53E-09**	**3.90E-18**	rs4838313	9	127832779	N/A	5.71E-01	**4.31E-05**	2.22E-01	**1.89E-04**
rs29232	6	29719410	GABBR1	**7.53E-09**	**3.90E-18**	rs10217721	9	127850667	N/A	7.02E-02	**8.75E-05**	8.02E-01	**7.72E-03**
rs29232	6	29719410	GABBR1	**7.53E-09**	**3.90E-18**	rs7496968	15	25984569	OCA2	6.76E-01	**4.42E-05**	7.57E-01	**2.39E-03**
rs29232	6	29719410	GABBR1	**7.53E-09**	**3.90E-18**	rs7179994	15	25997365	OCA2	7.80E-01	**1.36E-05**	8.81E-01	**3.03E-04**
rs29232	6	29719410	GABBR1	**7.53E-09**	**3.90E-18**	rs12596786	16	83261468	KLHL36	7.29E-01	**9.08E-05**	4.47E-02	**5.80E-06**
rs29232	6	29719410	GABBR1	**7.53E-09**	**3.90E-18**	rs16944618	16	83281757	USP10	5.26E-01	**2.20E-05**	1.62E-01	**1.25E-05**
rs29232	6	29719410	GABBR1	**7.53E-09**	**3.90E-18**	rs7217478	17	9548288	USP43	8.35E-01	**1.10E-05**	5.06E-01	**1.46E-04**
rs29232	6	29719410	GABBR1	**7.53E-09**	**3.90E-18**	rs873870	19	19599554	PBX4	4.07E-01	**9.48E-05**	8.50E-01	**1.80E-03**
rs29232	6	29719410	GABBR1	**7.53E-09**	**3.90E-18**	rs3891632	20	12302280	N/A	2.65E-01	**4.02E-05**	8.54E-01	**3.35E-03**
rs29232	6	29719410	GABBR1	**7.53E-09**	**3.90E-18**	rs6082457	20	21504715	N/A	6.38E-01	**6.78E-05**	8.81E-01	**2.38E-03**
rs29232	6	29719410	GABBR1	**7.53E-09**	**3.90E-18**	rs2839557	21	42863867	SLC37A1	6.47E-01	**3.38E-06**	1.13E-01	1.63E-02
rs29232	6	29719410	GABBR1	**7.53E-09**	**3.90E-18**	rs451954	21	42869023	SLC37A1	5.92E-01	**7.76E-06**	1.55E-01	1.72E-02
rs29232	6	29719410	GABBR1	**7.53E-09**	**3.90E-18**	rs401074	21	42886560	SLC37A1	9.93E-01	**1.90E-05**	1.80E-01	1.51E-02
rs29232	6	29719410	GABBR1	**7.53E-09**	**3.90E-18**	rs401809	21	42891021	SLC37A1	8.20E-01	**3.01E-05**	1.31E-01	3.76E-02
rs29232	6	29719410	GABBR1	**7.53E-09**	**3.90E-18**	rs433632	21	42893492	SLC37A1	8.18E-01	**7.98E-05**	1.05E-01	6.66E-02
rs2517713	6	30026078	HLA-A	**9.02E-09**	N/A	rs3122126	1	100500726	DBT	6.57E-01	**8.77E-05**	N/A	N/A
rs2517713	6	30026078	HLA-A	**9.02E-09**	N/A	rs4579779	1	100508491	RTCD1	6.57E-01	**8.77E-05**	N/A	N/A
rs2517713	6	30026078	HLA-A	**9.02E-09**	N/A	rs4504908	1	100517386	RTCD1	6.57E-01	**8.77E-05**	N/A	N/A
rs2517713	6	30026078	HLA-A	**9.02E-09**	N/A	rs7549726	1	169676139	N/A	8.60E-01	**5.44E-05**	N/A	N/A
rs2517713	6	30026078	HLA-A	**9.02E-09**	N/A	rs2347284	2	19553715	N/A	7.90E-01	**8.13E-05**	N/A	N/A
rs2517713	6	30026078	HLA-A	**9.02E-09**	N/A	rs4675431	2	204814338	N/A	4.85E-01	**9.40E-05**	N/A	N/A
rs2517713	6	30026078	HLA-A	**9.02E-09**	N/A	rs12488144	3	45806446	SLC6A20	**1.35E-03**	**4.24E-05**	N/A	N/A
rs2517713	6	30026078	HLA-A	**9.02E-09**	N/A	rs12506479	4	74811025	IL8	4.08E-02	**9.51E-05**	N/A	N/A
rs2517713	6	30026078	HLA-A	**9.02E-09**	N/A	rs6837842	4	189873238	N/A	7.73E-01	**5.01E-05**	N/A	N/A
rs2517713	6	30026078	HLA-A	**9.02E-09**	N/A	rs2438632	5	79427949	SERINC5	3.23E-01	**6.30E-05**	N/A	N/A
rs2517713	6	30026078	HLA-A	**9.02E-09**	N/A	rs2882826	6	67136078	N/A	9.03E-01	**7.12E-05**	N/A	N/A
rs2517713	6	30026078	HLA-A	**9.02E-09**	N/A	rs6905051	6	141846121	N/A	2.07E-01	**4.71E-05**	N/A	N/A
rs2517713	6	30026078	HLA-A	**9.02E-09**	N/A	rs2982683	6	152340128	ESR1	7.58E-01	**9.53E-05**	N/A	N/A
rs2517713	6	30026078	HLA-A	**9.02E-09**	N/A	rs726281	6	152344271	ESR1	9.43E-01	**5.31E-05**	N/A	N/A
rs2517713	6	30026078	HLA-A	**9.02E-09**	N/A	rs17199454	8	122144107	N/A	2.98E-01	**3.86E-05**	N/A	N/A
rs2517713	6	30026078	HLA-A	**9.02E-09**	N/A	rs520887	10	78720167	KCNMA1	5.81E-01	**6.07E-06**	N/A	N/A
rs2517713	6	30026078	HLA-A	**9.02E-09**	N/A	rs583507	10	78735335	KCNMA1	4.79E-01	**5.67E-06**	N/A	N/A
rs2517713	6	30026078	HLA-A	**9.02E-09**	N/A	rs11023194	11	14326345	N/A	3.99E-01	**8.41E-05**	N/A	N/A
rs2517713	6	30026078	HLA-A	**9.02E-09**	N/A	rs12785600	11	20043788	N/AV2	4.60E-01	**6.24E-05**	N/A	N/A
rs2517713	6	30026078	HLA-A	**9.02E-09**	N/A	rs658976	11	73775114	PGM2L1	8.19E-01	**2.80E-05**	N/A	N/A
rs2517713	6	30026078	HLA-A	**9.02E-09**	N/A	rs639228	11	73775613	PGM2L1	7.46E-01	**8.35E-05**	N/A	N/A
rs2517713	6	30026078	HLA-A	**9.02E-09**	N/A	rs639525	11	73782753	PGM2L1	7.25E-01	**7.53E-05**	N/A	N/A
rs2517713	6	30026078	HLA-A	**9.02E-09**	N/A	rs1351229	12	13309829	N/A	5.48E-01	**1.41E-05**	N/A	N/A
rs2517713	6	30026078	HLA-A	**9.02E-09**	N/A	rs2061868	12	13313104	N/A	2.14E-01	**2.95E-05**	N/A	N/A
rs2517713	6	30026078	HLA-A	**9.02E-09**	N/A	rs8059056	16	76324079	NUDT7	9.61E-01	**3.73E-05**	N/A	N/A
rs2517713	6	30026078	HLA-A	**9.02E-09**	N/A	rs16946398	16	76324992	NUDT7	9.61E-01	**3.73E-05**	N/A	N/A
rs2517713	6	30026078	HLA-A	**9.02E-09**	N/A	rs4496199	17	13743140	N/A	5.59E-01	**8.77E-05**	N/A	N/A
rs2517713	6	30026078	HLA-A	**9.02E-09**	N/A	rs2364890	18	65595729	DOK6	6.97E-01	**2.84E-05**	N/A	N/A
rs2517713	6	30026078	HLA-A	**9.02E-09**	N/A	rs6137384	20	21473800	N/A	5.00E-01	**2.17E-05**	N/A	N/A
rs2517713	6	30026078	HLA-A	**9.02E-09**	N/A	rs6035881	20	21475128	N/A	5.02E-01	**1.19E-05**	N/A	N/A
rs2517713	6	30026078	HLA-A	**9.02E-09**	N/A	rs4813430	20	21484958	N/A	7.11E-01	**2.71E-05**	N/A	N/A
rs2975042	6	30028515	HLA-A	**9.15E-09**	N/A	rs3122126	1	100500726	DBT	6.57E-01	**7.62E-05**	N/A	N/A
rs2975042	6	30028515	HLA-A	**9.15E-09**	N/A	rs4579779	1	100508491	RTCD1	6.57E-01	**7.62E-05**	N/A	N/A
rs2975042	6	30028515	HLA-A	**9.15E-09**	N/A	rs4504908	1	100517386	RTCD1	6.57E-01	**7.62E-05**	N/A	N/A
rs2975042	6	30028515	HLA-A	**9.15E-09**	N/A	rs7549726	1	169676139	N/A	8.60E-01	**3.04E-05**	N/A	N/A
rs2975042	6	30028515	HLA-A	**9.15E-09**	N/A	rs12488144	3	45806446	SLC6A20	**1.35E-03**	**3.30E-05**	N/A	N/A
rs2975042	6	30028515	HLA-A	**9.15E-09**	N/A	rs2346649	4	63584319	N/A	2.61E-01	**7.96E-05**	N/A	N/A
rs2975042	6	30028515	HLA-A	**9.15E-09**	N/A	rs6837842	4	189873238	N/A	7.73E-01	**7.33E-05**	N/A	N/A
rs2975042	6	30028515	HLA-A	**9.15E-09**	N/A	rs2438632	5	79427949	SERINC5	3.23E-01	**5.54E-05**	N/A	N/A
rs2975042	6	30028515	HLA-A	**9.15E-09**	N/A	rs2882826	6	67136078	N/A	9.03E-01	**8.15E-05**	N/A	N/A
rs2975042	6	30028515	HLA-A	**9.15E-09**	N/A	rs6905051	6	141846121	N/A	2.07E-01	**3.77E-05**	N/A	N/A
rs2975042	6	30028515	HLA-A	**9.15E-09**	N/A	rs2982683	6	152340128	ESR1	7.58E-01	**9.19E-05**	N/A	N/A
rs2975042	6	30028515	HLA-A	**9.15E-09**	N/A	rs726281	6	152344271	ESR1	9.43E-01	**4.07E-05**	N/A	N/A
rs2975042	6	30028515	HLA-A	**9.15E-09**	N/A	rs17199454	8	122144107	N/A	2.98E-01	**3.29E-05**	N/A	N/A
rs2975042	6	30028515	HLA-A	**9.15E-09**	N/A	rs520887	10	78720167	KCNMA1	5.81E-01	**9.71E-06**	N/A	N/A
rs2975042	6	30028515	HLA-A	**9.15E-09**	N/A	rs583507	10	78735335	KCNMA1	4.79E-01	**9.10E-06**	N/A	N/A
rs2975042	6	30028515	HLA-A	**9.15E-09**	N/A	rs12785600	11	20043788	N/AV2	4.60E-01	**8.11E-05**	N/A	N/A
rs2975042	6	30028515	HLA-A	**9.15E-09**	N/A	rs658976	11	73775114	PGM2L1	8.19E-01	**1.98E-05**	N/A	N/A
rs2975042	6	30028515	HLA-A	**9.15E-09**	N/A	rs639228	11	73775613	PGM2L1	7.46E-01	**6.09E-05**	N/A	N/A
rs2975042	6	30028515	HLA-A	**9.15E-09**	N/A	rs639525	11	73782753	PGM2L1	7.25E-01	**5.49E-05**	N/A	N/A
rs2975042	6	30028515	HLA-A	**9.15E-09**	N/A	rs1351229	12	13309829	N/A	5.48E-01	**2.80E-05**	N/A	N/A
rs2975042	6	30028515	HLA-A	**9.15E-09**	N/A	rs2061868	12	13313104	N/A	2.14E-01	**5.85E-05**	N/A	N/A
rs2975042	6	30028515	HLA-A	**9.15E-09**	N/A	rs8059056	16	76324079	NUDT7	9.61E-01	**2.82E-05**	N/A	N/A
rs2975042	6	30028515	HLA-A	**9.15E-09**	N/A	rs16946398	16	76324992	NUDT7	9.61E-01	**2.82E-05**	N/A	N/A
rs2975042	6	30028515	HLA-A	**9.15E-09**	N/A	rs2364890	18	65595729	DOK6	6.97E-01	**4.18E-05**	N/A	N/A
rs2975042	6	30028515	HLA-A	**9.15E-09**	N/A	rs6137384	20	21473800	N/A	5.00E-01	**8.73E-06**	N/A	N/A
rs2975042	6	30028515	HLA-A	**9.15E-09**	N/A	rs6035881	20	21475128	N/A	5.02E-01	**4.71E-06**	N/A	N/A
rs2975042	6	30028515	HLA-A	**9.15E-09**	N/A	rs4813430	20	21484958	N/A	7.11E-01	**1.10E-05**	N/A	N/A
rs2860580	6	30014670	HLA-A	**1.20E-08**	**1.34E-28**	rs3122126	1	100500726	DBT	6.57E-01	**7.67E-05**	7.46E-01	**1.06E-03**
rs2860580	6	30014670	HLA-A	**1.20E-08**	**1.34E-28**	rs4579779	1	100508491	RTCD1	6.57E-01	**7.67E-05**	7.46E-01	**1.06E-03**
rs2860580	6	30014670	HLA-A	**1.20E-08**	**1.34E-28**	rs4504908	1	100517386	RTCD1	6.57E-01	**7.67E-05**	7.46E-01	**1.06E-03**
rs2860580	6	30014670	HLA-A	**1.20E-08**	**1.34E-28**	rs10801954	1	117611372	N/A	5.94E-01	**9.76E-05**	4.48E-02	2.06E-01
rs2860580	6	30014670	HLA-A	**1.20E-08**	**1.34E-28**	rs7549726	1	169676139	N/A	8.60E-01	**6.69E-05**	7.17E-01	**3.02E-03**
rs2860580	6	30014670	HLA-A	**1.20E-08**	**1.34E-28**	rs2347284	2	19553715	N/A	7.90E-01	**8.23E-05**	9.30E-01	**4.60E-03**
rs2860580	6	30014670	HLA-A	**1.20E-08**	**1.34E-28**	rs4675431	2	204814338	N/A	4.85E-01	**8.74E-05**	9.88E-01	**1.26E-03**

SNP1						SNP2					Interaction p-value		
SNP	Chr.	Position	Nearest Gene	Taiwan GWAS P	China GWAS	SNP	Chr.	Position	Nearest Gene	GWAS P	Stage 1	Stage 2	Combined
rs2860580	6	30014670	HLA-A	**1.20E-08**	**1.34E-28**	rs12488144	3	45806446	SLC6A20	**1.35E-03**	**4.96E-05**	1.88E-01	2.05E-02
rs2860580	6	30014670	HLA-A	**1.20E-08**	**1.34E-28**	rs6837842	4	189873238	N/A	7.73E-01	**5.38E-05**	8.06E-01	**8.79E-03**
rs2860580	6	30014670	HLA-A	**1.20E-08**	**1.34E-28**	rs2438632	5	79427949	SERINC5	3.23E-01	**6.67E-05**	7.69E-01	**6.15E-03**
rs2860580	6	30014670	HLA-A	**1.20E-08**	**1.34E-28**	rs4236134	6	55551311	HMGCLL1	9.32E-01	**9.39E-05**	7.56E-01	**5.24E-03**
rs2860580	6	30014670	HLA-A	**1.20E-08**	**1.34E-28**	rs9367646	6	55574331	N/A	9.31E-01	**8.80E-05**	7.17E-01	**5.53E-03**
rs2860580	6	30014670	HLA-A	**1.20E-08**	**1.34E-28**	rs2882826	6	67136078	N/A	9.03E-01	**9.92E-05**	5.54E-01	1.11E-02
rs2860580	6	30014670	HLA-A	**1.20E-08**	**1.34E-28**	rs6905051	6	141846121	N/A	2.07E-01	**4.33E-05**	3.26E-01	**3.85E-04**
rs2860580	6	30014670	HLA-A	**1.20E-08**	**1.34E-28**	rs2982683	6	152340128	ESR1	7.58E-01	**7.59E-05**	3.31E-01	1.19E-02
rs2860580	6	30014670	HLA-A	**1.20E-08**	**1.34E-28**	rs726281	6	152344271	ESR1	9.43E-01	**5.21E-05**	4.63E-01	**4.89E-03**
rs2860580	6	30014670	HLA-A	**1.20E-08**	**1.34E-28**	rs17199454	8	122144107	N/A	2.98E-01	**4.29E-05**	6.30E-01	**3.15E-04**
rs2860580	6	30014670	HLA-A	**1.20E-08**	**1.34E-28**	rs520887	10	78720167	KCNMA1	5.81E-01	**8.74E-06**	3.18E-01	**2.68E-03**
rs2860580	6	30014670	HLA-A	**1.20E-08**	**1.34E-28**	rs583507	10	78735335	KCNMA1	4.79E-01	**8.23E-06**	3.18E-01	**2.69E-03**
rs2860580	6	30014670	HLA-A	**1.20E-08**	**1.34E-28**	rs11023194	11	14326345	N/A	3.99E-01	**7.82E-05**	6.43E-01	1.87E-02
rs2860580	6	30014670	HLA-A	**1.20E-08**	**1.34E-28**	rs12785600	11	20043788	N/AV2	4.60E-01	**6.24E-05**	6.22E-01	**4.13E-04**
rs2860580	6	30014670	HLA-A	**1.20E-08**	**1.34E-28**	rs658976	11	73775114	PGM2L1	8.19E-01	**3.54E-05**	2.00E-01	5.99E-02
rs2860580	6	30014670	HLA-A	**1.20E-08**	**1.34E-28**	rs639525	11	73782753	PGM2L1	7.25E-01	**9.46E-05**	2.68E-01	6.70E-02
rs2860580	6	30014670	HLA-A	**1.20E-08**	**1.34E-28**	rs1351229	12	13309829	N/A	5.48E-01	**1.66E-05**	9.99E-01	**7.12E-04**
rs2860580	6	30014670	HLA-A	**1.20E-08**	**1.34E-28**	rs2061868	12	13313104	N/A	2.14E-01	**3.71E-05**	6.28E-01	**3.97E-03**
rs2860580	6	30014670	HLA-A	**1.20E-08**	**1.34E-28**	rs8059056	16	76324079	NUDT7	9.61E-01	**4.44E-05**	2.92E-01	**1.64E-04**
rs2860580	6	30014670	HLA-A	**1.20E-08**	**1.34E-28**	rs16946398	16	76324992	NUDT7	9.61E-01	**4.44E-05**	2.92E-01	**1.64E-04**
rs2860580	6	30014670	HLA-A	**1.20E-08**	**1.34E-28**	rs4496199	17	13743140	N/A	5.59E-01	**8.57E-05**	6.56E-01	**3.88E-03**
rs2860580	6	30014670	HLA-A	**1.20E-08**	**1.34E-28**	rs2364890	18	65595729	DOK6	6.97E-01	**2.46E-05**	4.93E-01	**2.53E-04**
rs2860580	6	30014670	HLA-A	**1.20E-08**	**1.34E-28**	rs6137384	20	21473800	N/A	5.00E-01	**2.09E-05**	4.81E-01	**7.36E-03**
rs2860580	6	30014670	HLA-A	**1.20E-08**	**1.34E-28**	rs6035881	20	21475128	N/A	5.02E-01	**1.14E-05**	5.41E-01	**4.27E-03**
rs2860580	6	30014670	HLA-A	**1.20E-08**	**1.34E-28**	rs4813430	20	21484958	N/A	7.11E-01	**2.60E-05**	N/A	N/A
rs9260734	6	30040645	HCG9	**7.70E-08**	**9.80E-22**	rs3122126	1	100500726	DBT	6.57E-01	**3.48E-05**	9.20E-01	**1.62E-03**
rs9260734	6	30040645	HCG9	**7.70E-08**	**9.80E-22**	rs4579779	1	100508491	RTCD1	6.57E-01	**3.48E-05**	9.20E-01	**1.62E-03**
rs9260734	6	30040645	HCG9	**7.70E-08**	**9.80E-22**	rs4504908	1	100517386	RTCD1	6.57E-01	**3.48E-05**	9.20E-01	**1.62E-03**
rs9260734	6	30040645	HCG9	**7.70E-08**	**9.80E-22**	rs17019005	2	4329744	N/A	5.43E-02	**6.56E-05**	1.20E-01	2.59E-02
rs9260734	6	30040645	HCG9	**7.70E-08**	**9.80E-22**	rs2278581	2	42874418	HAAO	9.88E-01	**9.15E-05**	3.24E-02	8.15E-02
rs9260734	6	30040645	HCG9	**7.70E-08**	**9.80E-22**	rs2937715	5	75603809	SV2C	9.78E-01	**6.40E-05**	4.24E-01	**1.03E-03**
rs9260734	6	30040645	HCG9	**7.70E-08**	**9.80E-22**	rs2438632	5	79427949	SERINC5	3.23E-01	**2.73E-05**	2.85E-01	1.54E-02
rs9260734	6	30040645	HCG9	**7.70E-08**	**9.80E-22**	rs6905051	6	141846121	N/A	2.07E-01	**2.55E-05**	6.45E-01	**1.08E-03**
rs9260734	6	30040645	HCG9	**7.70E-08**	**9.80E-22**	rs9376628	6	141902024	N/A	1.46E-01	**3.64E-05**	7.98E-01	**1.43E-03**
rs9260734	6	30040645	HCG9	**7.70E-08**	**9.80E-22**	rs11757988	6	141928389	N/A	3.00E-01	**2.29E-05**	5.53E-01	**8.00E-04**
rs9260734	6	30040645	HCG9	**7.70E-08**	**9.80E-22**	rs38819	7	117495983	N/A	5.28E-01	**9.97E-05**	N/A	N/A
rs9260734	6	30040645	HCG9	**7.70E-08**	**9.80E-22**	rs17199454	8	122144107	N/A	2.98E-01	**1.79E-05**	9.54E-01	**4.23E-04**
rs9260734	6	30040645	HCG9	**7.70E-08**	**9.80E-22**	rs7864480	9	4049101	GLIS3	4.15E-01	**6.60E-05**	2.04E-01	**2.75E-04**
rs9260734	6	30040645	HCG9	**7.70E-08**	**9.80E-22**	rs2601735	10	14867137	FAM107B	2.41E-01	**7.51E-05**	1.11E-01	**8.77E-05**
rs9260734	6	30040645	HCG9	**7.70E-08**	**9.80E-22**	rs520887	10	78720167	KCNMA1	5.81E-01	**8.83E-06**	9.90E-02	**9.11E-03**
rs9260734	6	30040645	HCG9	**7.70E-08**	**9.80E-22**	rs583507	10	78735335	KCNMA1	4.79E-01	**7.74E-06**	9.90E-02	**8.91E-03**
rs9260734	6	30040645	HCG9	**7.70E-08**	**9.80E-22**	rs10824534	10	78807844	KCNMA1	3.30E-01	**8.16E-05**	1.30E-01	3.26E-02
rs9260734	6	30040645	HCG9	**7.70E-08**	**9.80E-22**	rs1371457	10	78817839	KCNMA1	6.18E-01	**4.42E-05**	1.50E-01	1.99E-02
rs9260734	6	30040645	HCG9	**7.70E-08**	**9.80E-22**	rs12785600	11	20043788	N/AV2	4.60E-01	**5.72E-06**	8.37E-01	**1.39E-04**
rs9260734	6	30040645	HCG9	**7.70E-08**	**9.80E-22**	rs1351229	12	13309829	N/A	5.48E-01	**2.98E-05**	5.62E-01	**3.85E-04**
rs9260734	6	30040645	HCG9	**7.70E-08**	**9.80E-22**	rs2061868	12	13313104	N/A	2.14E-01	**3.65E-05**	8.90E-01	**2.62E-03**
rs9260734	6	30040645	HCG9	**7.70E-08**	**9.80E-22**	rs932539	14	58614370	N/A	3.62E-01	**5.16E-05**	3.87E-01	**4.50E-04**
rs9260734	6	30040645	HCG9	**7.70E-08**	**9.80E-22**	rs11630247	15	23649253	ATP10A	4.36E-01	**5.87E-05**	2.58E-01	2.00E-02
rs9260734	6	30040645	HCG9	**7.70E-08**	**9.80E-22**	rs8053463	16	27205537	NSMCE1	1.89E-01	**9.91E-05**	9.57E-01	**5.55E-03**
rs9260734	6	30040645	HCG9	**7.70E-08**	**9.80E-22**	rs11081236	18	6433601	N/A	5.84E-01	**5.46E-05**	7.70E-01	**9.39E-04**
rs9260734	6	30040645	HCG9	**7.70E-08**	**9.80E-22**	rs2364890	18	65595729	DOK6	6.97E-01	**6.98E-05**	3.49E-01	**2.46E-04**
rs9260734	6	30040645	HCG9	**7.70E-08**	**9.80E-22**	rs6137384	20	21473800	N/A	5.00E-01	**5.20E-05**	3.35E-01	2.07E-02
rs9260734	6	30040645	HCG9	**7.70E-08**	**9.80E-22**	rs6035881	20	21475128	N/A	5.02E-01	**2.88E-05**	3.99E-01	1.22E-02
rs9260734	6	30040645	HCG9	**7.70E-08**	**9.80E-22**	rs6082457	20	21504715	N/A	6.38E-01	**7.16E-05**	1.57E-01	**1.10E-04**
rs9260734	6	30040645	HCG9	**7.70E-08**	**9.80E-22**	rs2839397	21	42159411	PRDM15	9.83E-02	**8.63E-05**	3.90E-01	**8.38E-04**
rs3869062	6	30042870	HCG9	**3.06E-07**	**2.90E-20**	rs3122126	1	100500726	DBT	6.57E-01	**4.39E-05**	9.22E-01	**1.71E-03**
rs3869062	6	30042870	HCG9	**3.06E-07**	**2.90E-20**	rs4579779	1	100508491	RTCD1	6.57E-01	**4.39E-05**	9.22E-01	**1.71E-03**
rs3869062	6	30042870	HCG9	**3.06E-07**	**2.90E-20**	rs4504908	1	100517386	RTCD1	6.57E-01	**4.39E-05**	9.22E-01	**1.71E-03**
rs3869062	6	30042870	HCG9	**3.06E-07**	**2.90E-20**	rs7549726	1	169676139	N/A	8.60E-01	**7.87E-05**	4.10E-01	**8.10E-04**
rs3869062	6	30042870	HCG9	**3.06E-07**	**2.90E-20**	rs6733573	2	204840871	N/A	3.35E-01	**8.94E-05**	3.29E-01	**2.61E-04**
rs3869062	6	30042870	HCG9	**3.06E-07**	**2.90E-20**	rs7590649	2	204842857	N/A	3.35E-01	**8.94E-05**	2.60E-01	**1.84E-04**
rs3869062	6	30042870	HCG9	**3.06E-07**	**2.90E-20**	rs2861203	3	174433247	N/A	6.12E-01	**8.98E-05**	6.55E-01	**2.84E-03**
rs3869062	6	30042870	HCG9	**3.06E-07**	**2.90E-20**	rs1981693	4	16889095	N/A	1.39E-01	**8.10E-05**	5.75E-01	1.12E-02
rs3869062	6	30042870	HCG9	**3.06E-07**	**2.90E-20**	rs4543093	4	154323909	TRIM2	6.96E-01	**9.66E-05**	N/A	N/A
rs3869062	6	30042870	HCG9	**3.06E-07**	**2.90E-20**	rs2937715	5	75603809	SV2C	9.78E-01	**8.18E-05**	4.15E-01	**9.09E-04**
rs3869062	6	30042870	HCG9	**3.06E-07**	**2.90E-20**	rs2438632	5	79427949	SERINC5	3.23E-01	**1.26E-05**	4.00E-01	**4.71E-03**
rs3869062	6	30042870	HCG9	**3.06E-07**	**2.90E-20**	rs4236134	6	55551311	HMGCLL1	9.32E-01	**9.34E-05**	7.38E-01	**1.19E-03**
rs3869062	6	30042870	HCG9	**3.06E-07**	**2.90E-20**	rs9367646	6	55574331	N/A	9.31E-01	**8.71E-05**	7.71E-01	**1.23E-03**
rs3869062	6	30042870	HCG9	**3.06E-07**	**2.90E-20**	rs2882826	6	67136078	N/A	9.03E-01	**8.91E-05**	9.71E-01	**3.85E-03**
rs3869062	6	30042870	HCG9	**3.06E-07**	**2.90E-20**	rs6905051	6	141846121	N/A	2.07E-01	**1.87E-05**	7.57E-01	**1.03E-03**
rs3869062	6	30042870	HCG9	**3.06E-07**	**2.90E-20**	rs9376628	6	141902024	N/A	1.46E-01	**2.33E-05**	7.83E-01	**1.05E-03**
rs3869062	6	30042870	HCG9	**3.06E-07**	**2.90E-20**	rs11757988	6	141928389	N/A	3.00E-01	**2.89E-05**	5.37E-01	**7.59E-04**
rs3869062	6	30042870	HCG9	**3.06E-07**	**2.90E-20**	rs12536735	7	142875457	N/A	6.29E-01	**3.51E-05**	9.19E-01	**1.34E-03**
rs3869062	6	30042870	HCG9	**3.06E-07**	**2.90E-20**	rs1525108	7	142898876	N/A	4.25E-01	**9.93E-05**	6.84E-01	**1.39E-03**
rs3869062	6	30042870	HCG9	**3.06E-07**	**2.90E-20**	rs17199454	8	122144107	N/A	2.98E-01	**5.76E-05**	9.47E-01	**9.00E-04**
rs3869062	6	30042870	HCG9	**3.06E-07**	**2.90E-20**	rs7864480	9	4049101	GLIS3	4.15E-01	**2.00E-05**	1.93E-01	**8.35E-05**
rs3869062	6	30042870	HCG9	**3.06E-07**	**2.90E-20**	rs10817497	9	115453572	N/A	1.54E-01	**7.63E-05**	6.58E-01	**1.24E-03**
rs3869062	6	30042870	HCG9	**3.06E-07**	**2.90E-20**	rs520887	10	78720167	KCNMA1	5.81E-01	**1.77E-05**	5.96E-02	1.76E-02
rs3869062	6	30042870	HCG9	**3.06E-07**	**2.90E-20**	rs583507	10	78735335	KCNMA1	4.79E-01	**1.55E-05**	5.96E-02	1.71E-02
rs3869062	6	30042870	HCG9	**3.06E-07**	**2.90E-20**	rs12785600	11	20043788	N/AV2	4.60E-01	**1.37E-05**	8.08E-01	**2.55E-04**
rs3869062	6	30042870	HCG9	**3.06E-07**	**2.90E-20**	rs1351229	12	13309829	N/A	5.48E-01	**4.04E-05**	7.71E-01	**7.11E-04**
rs3869062	6	30042870	HCG9	**3.06E-07**	**2.90E-20**	rs11630247	15	23649253	ATP10A	4.36E-01	**3.31E-05**	2.98E-01	1.20E-02
rs3869062	6	30042870	HCG9	**3.06E-07**	**2.90E-20**	rs2364890	18	65595729	DOK6	6.97E-01	**9.02E-05**	2.58E-01	**1.96E-04**
rs3869062	6	30042870	HCG9	**3.06E-07**	**2.90E-20**	rs6035877	20	21460532	NKX2-2	7.08E-01	**9.30E-05**	2.29E-01	3.76E-02
rs3869062	6	30042870	HCG9	**3.06E-07**	**2.90E-20**	rs6137384	20	21473800	N/A	5.00E-01	**1.07E-05**	2.22E-01	1.59E-02

SNP1						SNP2					Interaction p-value		
SNP	Chr.	Position	Nearest Gene	Taiwan GWAS P	China GWAS	SNP	Chr.	Position	Nearest Gene	GWAS P	Stage 1	Stage 2	Combined
rs3869062	6	30042870	HCG9	3.06E-07	2.90E-20	rs6035881	20	21475128	N/A	5.02E-01	5.76E-06	2.74E-01	9.02E-03
rs3869062	6	30042870	HCG9	3.06E-07	2.90E-20	rs4813430	20	21484958	N/A	7.11E-01	2.26E-05	N/A	N/A
rs3869062	6	30042870	HCG9	3.06E-07	2.90E-20	rs6082457	20	21504715	N/A	6.38E-01	3.49E-05	1.52E-01	4.52E-05
rs16896923	6	30108666	NCRNA00171	3.11E-06	4.20E-11	rs4912018	1	19890308	TMCO4	2.19E-01	8.29E-05	N/A	N/A
rs16896923	6	30108666	NCRNA00171	3.11E-06	4.20E-11	rs10917518	1	19890325	TMCO4	4.95E-01	2.89E-05	N/A	N/A
rs16896923	6	30108666	NCRNA00171	3.11E-06	4.20E-11	rs1858409	2	2430444	N/A	2.89E-02	8.19E-05	N/A	N/A
rs16896923	6	30108666	NCRNA00171	3.11E-06	4.20E-11	rs889248	5	63940824	RGS7BP	4.84E-01	3.67E-05	N/A	N/A
rs16896923	6	30108666	NCRNA00171	3.11E-06	4.20E-11	rs918604	5	63949443	RGS7BP	4.76E-01	3.62E-05	N/A	N/A
rs16896923	6	30108666	NCRNA00171	3.11E-06	4.20E-11	rs1266831	6	52205995	IL17F	4.05E-01	5.94E-05	N/A	N/A
rs16896923	6	30108666	NCRNA00171	3.11E-06	4.20E-11	rs9655042	7	4836277	RADIL	4.48E-01	4.06E-05	N/A	N/A
rs16896923	6	30108666	NCRNA00171	3.11E-06	4.20E-11	rs1351229	12	13309829	N/A	5.48E-01	1.62E-05	N/A	N/A
rs16896923	6	30108666	NCRNA00171	3.11E-06	4.20E-11	rs2061868	12	13313104	N/A	2.14E-01	7.46E-05	N/A	N/A
rs16896923	6	30108666	NCRNA00171	3.11E-06	4.20E-11	rs1373422	12	42325914	N/A	8.42E-01	7.50E-05	N/A	N/A
rs16896923	6	30108666	NCRNA00171	3.11E-06	4.20E-11	rs11169821	12	50062992	GALNT6	6.53E-01	8.22E-05	N/A	N/A
rs16896923	6	30108666	NCRNA00171	3.11E-06	4.20E-11	rs4913307	12	65150558	N/A	3.43E-01	5.55E-05	N/A	N/A
rs16896923	6	30108666	NCRNA00171	3.11E-06	4.20E-11	rs576810	13	74822785	TBC1D4	1.41E-02	6.20E-05	N/A	N/A
rs16896923	6	30108666	NCRNA00171	3.11E-06	4.20E-11	rs11625845	14	28334303	C14orf23	9.28E-01	4.58E-05	N/A	N/A
rs16896923	6	30108666	NCRNA00171	3.11E-06	4.20E-11	rs8074124	17	33785091	SOCS7	6.54E-01	2.54E-05	N/A	N/A
rs16896923	6	30108666	NCRNA00171	3.11E-06	4.20E-11	rs6089236	20	59529751	CDH4	8.07E-01	9.43E-06	N/A	N/A
rs16896923	6	30108666	NCRNA00171	3.11E-06	4.20E-11	rs1041274	20	59532539	CDH4	7.30E-01	5.66E-05	N/A	N/A
rs16896923	6	30108666	NCRNA00171	3.11E-06	4.20E-11	rs6061612	20	59540967	CDH4	7.63E-01	8.62E-05	N/A	N/A
rs16896923	6	30108666	NCRNA00171	3.11E-06	4.20E-11	rs2834843	21	35502660	N/A	6.26E-01	1.09E-05	N/A	N/A
rs16896923	6	30108666	NCRNA00171	3.11E-06	4.20E-11	rs5752318	22	25122113	N/A	4.66E-01	1.98E-05	N/A	N/A
rs5009448	6	30048467	HCG9	3.96E-06	2.00E-18	rs10917518	1	19890325	TMCO4	4.95E-01	9.60E-05	N/A	N/A
rs5009448	6	30048467	HCG9	3.96E-06	2.00E-18	rs1342738	1	117590806	N/A	8.65E-01	2.30E-05	1.77E-01	3.04E-02
rs5009448	6	30048467	HCG9	3.96E-06	2.00E-18	rs11124523	2	36704768	N/A	6.44E-01	6.63E-05	5.41E-01	5.01E-03
rs5009448	6	30048467	HCG9	3.96E-06	2.00E-18	rs4569679	3	87773719	N/A	7.88E-01	5.74E-05	5.39E-01	6.23E-04
rs5009448	6	30048467	HCG9	3.96E-06	2.00E-18	rs2861203	3	174433247	N/A	6.12E-01	2.55E-05	5.60E-01	8.46E-04
rs5009448	6	30048467	HCG9	3.96E-06	2.00E-18	rs1497431	4	67897598	N/A	8.71E-01	4.11E-05	8.99E-01	6.63E-04
rs5009448	6	30048467	HCG9	3.96E-06	2.00E-18	rs4236134	6	55551311	HMGCLL1	9.32E-01	3.30E-05	6.17E-01	6.38E-04
rs5009448	6	30048467	HCG9	3.96E-06	2.00E-18	rs9367646	6	55574331	N/A	9.31E-01	3.06E-05	6.56E-01	6.85E-04
rs5009448	6	30048467	HCG9	3.96E-06	2.00E-18	rs4710341	6	68173593	N/A	3.07E-01	7.91E-05	2.80E-01	2.92E-02
rs5009448	6	30048467	HCG9	3.96E-06	2.00E-18	rs6905051	6	141846121	N/A	2.07E-01	6.31E-05	9.41E-01	2.96E-03
rs5009448	6	30048467	HCG9	3.96E-06	2.00E-18	rs9376628	6	141902024	N/A	1.46E-01	8.03E-05	8.20E-01	5.43E-03
rs5009448	6	30048467	HCG9	3.96E-06	2.00E-18	rs11757988	6	141928389	N/A	3.00E-01	3.58E-05	7.03E-01	5.41E-03
rs5009448	6	30048467	HCG9	3.96E-06	2.00E-18	rs1477578	7	49773641	VWC2	6.18E-01	1.17E-05	2.32E-01	2.53E-03
rs5009448	6	30048467	HCG9	3.96E-06	2.00E-18	rs11975846	7	81201270	HGF	5.68E-01	9.42E-05	6.67E-02	5.45E-02
rs5009448	6	30048467	HCG9	3.96E-06	2.00E-18	rs2217092	7	135635590	N/A	2.89E-01	7.43E-05	8.78E-01	1.79E-03
rs5009448	6	30048467	HCG9	3.96E-06	2.00E-18	rs10253012	7	135661486	N/A	2.40E-01	6.72E-05	9.91E-01	1.49E-03
rs5009448	6	30048467	HCG9	3.96E-06	2.00E-18	rs12155586	8	9011268	PPP1R3B	2.53E-01	3.28E-05	4.95E-01	4.43E-04
rs5009448	6	30048467	HCG9	3.96E-06	2.00E-18	rs4921809	8	17609341	MTUS1	3.81E-01	1.39E-05	5.65E-01	2.52E-03
rs5009448	6	30048467	HCG9	3.96E-06	2.00E-18	rs7830863	8	17611182	MTUS1	4.47E-01	2.65E-05	6.99E-01	2.58E-03
rs5009448	6	30048467	HCG9	3.96E-06	2.00E-18	rs7039700	9	9673443	PTPRD	9.78E-01	6.58E-05	3.64E-01	2.48E-04
rs5009448	6	30048467	HCG9	3.96E-06	2.00E-18	rs1327407	9	19742860	SLC24A2	9.83E-01	1.23E-05	2.76E-01	2.56E-05
rs5009448	6	30048467	HCG9	3.96E-06	2.00E-18	rs10514825	9	19744476	SLC24A2	8.96E-01	9.85E-05	4.31E-01	1.28E-04
rs5009448	6	30048467	HCG9	3.96E-06	2.00E-18	rs2383119	9	19746769	SLC24A2	3.64E-01	4.01E-05	2.74E-01	6.29E-05
rs5009448	6	30048467	HCG9	3.96E-06	2.00E-18	rs2808541	9	100397228	GABBR2	2.03E-01	1.42E-05	1.20E-01	3.65E-05
rs5009448	6	30048467	HCG9	3.96E-06	2.00E-18	rs2151216	9	100399213	GABBR2	2.39E-01	3.52E-05	1.20E-01	7.31E-05
rs5009448	6	30048467	HCG9	3.96E-06	2.00E-18	rs3739915	9	134266951	TTF1	5.24E-01	5.79E-05	5.60E-01	7.07E-04
rs5009448	6	30048467	HCG9	3.96E-06	2.00E-18	rs4481939	10	90824653	N/A	1.35E-01	4.98E-06	2.42E-01	4.02E-05
rs5009448	6	30048467	HCG9	3.96E-06	2.00E-18	rs10509564	10	90868929	N/A	2.55E-01	2.93E-05	1.91E-01	4.69E-05
rs5009448	6	30048467	HCG9	3.96E-06	2.00E-18	rs17465248	10	90875116	N/A	2.22E-01	2.28E-05	1.91E-01	3.87E-05
rs5009448	6	30048467	HCG9	3.96E-06	2.00E-18	rs1937346	10	90879367	N/A	7.96E-02	3.91E-06	1.97E-01	1.67E-05
rs5009448	6	30048467	HCG9	3.96E-06	2.00E-18	rs10887915	10	90883230	N/A	2.56E-01	1.58E-05	1.91E-01	2.90E-05
rs5009448	6	30048467	HCG9	3.96E-06	2.00E-18	rs10749129	10	115177211	N/A	9.77E-02	6.11E-05	4.72E-01	4.09E-03
rs5009448	6	30048467	HCG9	3.96E-06	2.00E-18	rs12785600	11	20043788	N/AV2	4.60E-01	2.68E-05	7.45E-01	4.81E-04
rs5009448	6	30048467	HCG9	3.96E-06	2.00E-18	rs10835128	11	27183167	N/A	6.81E-01	3.00E-05	8.31E-01	1.21E-03
rs5009448	6	30048467	HCG9	3.96E-06	2.00E-18	rs1442930	11	27189991	N/A	5.88E-01	3.45E-05	7.10E-01	1.00E-03
rs5009448	6	30048467	HCG9	3.96E-06	2.00E-18	rs10835138	11	27215475	N/A	6.61E-01	2.05E-05	7.17E-01	5.80E-04
rs5009448	6	30048467	HCG9	3.96E-06	2.00E-18	rs10835141	11	27217382	N/A	7.96E-01	3.52E-05	6.80E-01	7.82E-04
rs5009448	6	30048467	HCG9	3.96E-06	2.00E-18	rs11046677	12	22928034	N/A	5.04E-01	1.30E-05	4.61E-01	2.06E-03
rs5009448	6	30048467	HCG9	3.96E-06	2.00E-18	rs11176241	12	65194084	N/A	4.42E-01	5.90E-05	5.86E-01	9.75E-04
rs5009448	6	30048467	HCG9	3.96E-06	2.00E-18	rs824209	15	21598494	N/A	9.00E-01	3.14E-05	1.95E-01	8.10E-03
rs5009448	6	30048467	HCG9	3.96E-06	2.00E-18	rs1027010	15	51399446	N/A	9.59E-01	6.33E-05	8.72E-01	2.49E-03
rs5009448	6	30048467	HCG9	3.96E-06	2.00E-18	rs7497064	15	64777213	SMAD6	9.28E-01	6.58E-05	8.39E-02	1.18E-05
rs5009448	6	30048467	HCG9	3.96E-06	2.00E-18	rs1902815	16	19981996	GPR139	5.25E-01	9.72E-05	2.68E-03	3.40E-01
rs5009448	6	30048467	HCG9	3.96E-06	2.00E-18	rs9894473	17	68903231	SDK2	3.45E-01	5.42E-05	1.50E-01	2.38E-02
rs5009448	6	30048467	HCG9	3.96E-06	2.00E-18	rs549827	18	5976416	L3MBTL4	4.42E-01	5.76E-05	4.50E-01	6.99E-03
rs5009448	6	30048467	HCG9	3.96E-06	2.00E-18	rs4798424	18	5977788	L3MBTL4	4.77E-01	5.02E-05	4.81E-01	5.83E-03
rs5009448	6	30048467	HCG9	3.96E-06	2.00E-18	rs3848732	20	11706999	N/A	8.79E-01	4.59E-05	6.12E-01	4.58E-04
rs5009448	6	30048467	HCG9	3.96E-06	2.00E-18	rs761684	20	15806290	MACROD2	3.02E-01	4.52E-05	8.08E-01	2.92E-04
rs5009448	6	30048467	HCG9	3.96E-06	2.00E-18	rs6082457	20	21504715	N/A	6.38E-01	4.29E-05	3.19E-02	4.85E-06
rs5009448	6	30048467	HCG9	3.96E-06	2.00E-18	rs4809647	20	45795165	SULF2	8.50E-01	2.54E-05	9.36E-02	8.76E-06
rs2076483	6	29679524	GABBR1	1.23E-05	1.30E-12	rs771296	2	6988986	RNF144A	2.93E-01	8.68E-05	1.25E-01	2.84E-02
rs2076483	6	29679524	GABBR1	1.23E-05	1.30E-12	rs7560249	2	36851578	N/A	7.76E-01	7.00E-05	8.36E-01	2.03E-03
rs2076483	6	29679524	GABBR1	1.23E-05	1.30E-12	rs3856435	2	175601525	N/A	4.21E-01	7.49E-05	6.44E-01	7.33E-03
rs2076483	6	29679524	GABBR1	1.23E-05	1.30E-12	rs7587476	2	215362132	BARD1	4.53E-01	1.60E-05	5.89E-01	2.56E-04
rs2076483	6	29679524	GABBR1	1.23E-05	1.30E-12	rs33766	3	45255069	TMEM158	6.96E-01	4.46E-05	7.57E-02	2.07E-05
rs2076483	6	29679524	GABBR1	1.23E-05	1.30E-12	rs33785	3	45268267	N/A	1.01E-01	7.81E-05	5.53E-01	3.10E-04
rs2076483	6	29679524	GABBR1	1.23E-05	1.30E-12	rs12506479	4	74811025	IL8	4.08E-02	9.51E-05	3.29E-01	4.00E-02
rs2076483	6	29679524	GABBR1	1.23E-05	1.30E-12	rs4459954	4	166012451	N/A	4.17E-01	9.25E-05	7.62E-01	1.09E-03
rs2076483	6	29679524	GABBR1	1.23E-05	1.30E-12	rs13434896	4	189581756	N/A	3.87E-01	7.32E-05	7.35E-01	9.10E-04
rs2076483	6	29679524	GABBR1	1.23E-05	1.30E-12	rs7690014	4	189581857	N/A	3.87E-01	7.32E-05	7.35E-01	9.10E-04
rs2076483	6	29679524	GABBR1	1.23E-05	1.30E-12	rs10060705	5	63937060	RGS7BP	4.59E-01	8.16E-05	7.07E-01	1.91E-03
rs2076483	6	29679524	GABBR1	1.23E-05	1.30E-12	rs724254	7	7153079	N/A	3.83E-01	8.76E-05	5.18E-01	7.44E-03

SNP1						SNP2					Interaction p-value		
SNP	Chr.	Position	Nearest Gene	Taiwan GWAS P	China GWAS	SNP	Chr.	Position	Nearest Gene	GWAS P	Stage 1	Stage 2	Combined
rs2076483	6	29679524	GABBR1	**1.23E-05**	**1.30E-12**	rs2722311	7	37878719	TXNDC3	2.27E-01	**8.77E-05**	4.49E-01	**1.57E-03**
rs2076483	6	29679524	GABBR1	**1.23E-05**	**1.30E-12**	rs2722309	7	37879060	TXNDC3	2.25E-01	**7.72E-05**	4.49E-01	**1.44E-03**
rs2076483	6	29679524	GABBR1	**1.23E-05**	**1.30E-12**	rs2217092	7	135635590	N/A	2.89E-01	**8.17E-05**	6.16E-01	**5.03E-04**
rs2076483	6	29679524	GABBR1	**1.23E-05**	**1.30E-12**	rs10253012	7	135661486	N/A	2.40E-01	**1.65E-05**	3.81E-01	**9.73E-05**
rs2076483	6	29679524	GABBR1	**1.23E-05**	**1.30E-12**	rs1882092	7	135673778	N/A	3.73E-01	**2.19E-05**	4.54E-01	**1.34E-04**
rs2076483	6	29679524	GABBR1	**1.23E-05**	**1.30E-12**	rs12671681	7	135674364	N/A	6.57E-01	**1.36E-05**	2.92E-01	**2.90E-05**
rs2076483	6	29679524	GABBR1	**1.23E-05**	**1.30E-12**	rs2954610	8	3769301	CSMD1	8.81E-01	**4.17E-05**	7.41E-02	**4.96E-05**
rs2076483	6	29679524	GABBR1	**1.23E-05**	**1.30E-12**	rs7357519	8	4399763	CSMD1	3.82E-01	**1.69E-05**	3.36E-01	**5.79E-05**
rs2076483	6	29679524	GABBR1	**1.23E-05**	**1.30E-12**	rs4609193	8	12734835	N/A	2.16E-01	**9.25E-05**	8.55E-01	**1.69E-03**
rs2076483	6	29679524	GABBR1	**1.23E-05**	**1.30E-12**	rs9802064	8	17313332	MTMR7	4.60E-01	**3.36E-05**	4.38E-01	**6.98E-03**
rs2076483	6	29679524	GABBR1	**1.23E-05**	**1.30E-12**	rs335244	8	18689427	PSD3	4.67E-02	**5.80E-05**	5.42E-01	**4.05E-03**
rs2076483	6	29679524	GABBR1	**1.23E-05**	**1.30E-12**	rs3808900	9	124032585	RBM18	3.96E-01	**9.16E-05**	1.57E-01	5.23E-02
rs2076483	6	29679524	GABBR1	**1.23E-05**	**1.30E-12**	rs9602618	13	35736074	C13orf38	4.22E-01	**9.63E-05**	4.74E-01	**4.93E-04**
rs2076483	6	29679524	GABBR1	**1.23E-05**	**1.30E-12**	rs1359480	13	107413961	N/A	9.89E-02	**4.28E-05**	7.49E-01	**2.37E-03**
rs2076483	6	29679524	GABBR1	**1.23E-05**	**1.30E-12**	rs1952589	14	56175083	C14orf101	6.68E-01	**3.03E-05**	5.38E-01	**2.50E-04**
rs2076483	6	29679524	GABBR1	**1.23E-05**	**1.30E-12**	rs4429270	15	89828778	N/A	1.63E-01	**6.68E-05**	4.44E-01	**2.04E-04**
rs2076483	6	29679524	GABBR1	**1.23E-05**	**1.30E-12**	rs2014341	21	33267837	N/A	1.79E-01	**5.42E-05**	5.29E-01	**2.69E-04**
rs2076483	6	29679524	GABBR1	**1.23E-05**	**1.30E-12**	rs2839557	21	42863867	SLC37A1	6.47E-01	**9.47E-05**	3.82E-02	1.01E-01
rs2076483	6	29679524	GABBR1	**1.23E-05**	**1.30E-12**	rs1788414	21	42878403	SLC37A1	6.36E-01	**7.81E-05**	4.13E-02	7.94E-02
rs2076483	6	29679524	GABBR1	**1.23E-05**	**1.30E-12**	rs450508	21	42879076	SLC37A1	6.36E-01	**7.81E-05**	4.13E-02	7.94E-02
rs2076483	6	29679524	GABBR1	**1.23E-05**	**1.30E-12**	rs401074	21	42886560	SLC37A1	9.93E-01	**4.04E-05**	1.02E-01	3.50E-02
rs2076483	6	29679524	GABBR1	**1.23E-05**	**1.30E-12**	rs133504	22	47290141	FAM19A5	5.68E-01	**4.23E-05**	4.39E-01	**7.06E-03**
rs2267633	6	29678820	GABBR1	**1.23E-05**	**1.20E-12**	rs771296	2	6988986	RNF144A	2.93E-01	**8.68E-05**	1.25E-01	2.84E-02
rs2267633	6	29678820	GABBR1	**1.23E-05**	**1.20E-12**	rs7560249	2	36851578	N/A	7.76E-01	**7.00E-05**	8.36E-01	**2.03E-03**
rs2267633	6	29678820	GABBR1	**1.23E-05**	**1.20E-12**	rs3856435	2	175601525	N/A	4.21E-01	**7.49E-05**	6.44E-01	**7.33E-03**
rs2267633	6	29678820	GABBR1	**1.23E-05**	**1.20E-12**	rs7587476	2	215362132	BARD1	4.53E-01	**1.60E-05**	5.89E-01	**2.56E-04**
rs2267633	6	29678820	GABBR1	**1.23E-05**	**1.20E-12**	rs33766	3	45255069	TMEM158	6.96E-01	**4.46E-05**	7.57E-02	**2.07E-05**
rs2267633	6	29678820	GABBR1	**1.23E-05**	**1.20E-12**	rs33785	3	45268267	N/A	1.01E-01	**7.81E-05**	5.53E-01	**3.10E-04**
rs2267633	6	29678820	GABBR1	**1.23E-05**	**1.20E-12**	rs12506479	4	74811025	IL8	4.08E-02	**9.51E-05**	3.29E-01	4.00E-02
rs2267633	6	29678820	GABBR1	**1.23E-05**	**1.20E-12**	rs4459954	4	166012451	N/A	4.17E-01	**9.25E-05**	7.62E-01	**1.09E-03**
rs2267633	6	29678820	GABBR1	**1.23E-05**	**1.20E-12**	rs13434896	4	189581756	N/A	3.87E-01	**7.32E-05**	7.35E-01	**9.10E-04**
rs2267633	6	29678820	GABBR1	**1.23E-05**	**1.20E-12**	rs7690014	4	189581857	N/A	3.87E-01	**7.32E-05**	7.35E-01	**9.10E-04**
rs2267633	6	29678820	GABBR1	**1.23E-05**	**1.20E-12**	rs10060705	5	63937060	RGS7BP	4.59E-01	**8.16E-05**	7.07E-01	**1.91E-03**
rs2267633	6	29678820	GABBR1	**1.23E-05**	**1.20E-12**	rs724254	7	7153079	N/A	3.83E-01	**8.76E-05**	5.18E-01	**7.44E-03**
rs2267633	6	29678820	GABBR1	**1.23E-05**	**1.20E-12**	rs2722311	7	37878719	TXNDC3	2.27E-01	**8.77E-05**	4.49E-01	**1.57E-03**
rs2267633	6	29678820	GABBR1	**1.23E-05**	**1.20E-12**	rs2722309	7	37879060	TXNDC3	2.25E-01	**7.72E-05**	4.49E-01	**1.44E-03**
rs2267633	6	29678820	GABBR1	**1.23E-05**	**1.20E-12**	rs2217092	7	135635590	N/A	2.89E-01	**8.17E-05**	6.16E-01	**5.03E-04**
rs2267633	6	29678820	GABBR1	**1.23E-05**	**1.20E-12**	rs10253012	7	135661486	N/A	2.40E-01	**1.65E-05**	3.81E-01	**9.73E-05**
rs2267633	6	29678820	GABBR1	**1.23E-05**	**1.20E-12**	rs1882092	7	135673778	N/A	3.73E-01	**2.19E-05**	4.54E-01	**1.34E-04**
rs2267633	6	29678820	GABBR1	**1.23E-05**	**1.20E-12**	rs12671681	7	135674364	N/A	6.57E-01	**1.36E-05**	2.92E-01	**2.90E-05**
rs2267633	6	29678820	GABBR1	**1.23E-05**	**1.20E-12**	rs2954610	8	3769301	CSMD1	8.81E-01	**4.17E-05**	7.41E-02	**4.96E-05**
rs2267633	6	29678820	GABBR1	**1.23E-05**	**1.20E-12**	rs7357519	8	4399763	CSMD1	3.82E-01	**1.69E-05**	3.36E-01	**5.79E-05**
rs2267633	6	29678820	GABBR1	**1.23E-05**	**1.20E-12**	rs4609193	8	12734835	N/A	2.16E-01	**9.25E-05**	8.55E-01	**1.69E-03**
rs2267633	6	29678820	GABBR1	**1.23E-05**	**1.20E-12**	rs9802064	8	17313332	MTMR7	4.60E-01	**3.36E-05**	4.38E-01	**6.98E-03**
rs2267633	6	29678820	GABBR1	**1.23E-05**	**1.20E-12**	rs335244	8	18689427	PSD3	4.67E-02	**5.80E-05**	5.42E-01	**4.05E-03**
rs2267633	6	29678820	GABBR1	**1.23E-05**	**1.20E-12**	rs3808900	9	124032585	RBM18	3.96E-01	**9.16E-05**	1.57E-01	5.23E-02
rs2267633	6	29678820	GABBR1	**1.23E-05**	**1.20E-12**	rs9602618	13	35736074	C13orf38	4.22E-01	**9.63E-05**	4.74E-01	**4.93E-04**
rs2267633	6	29678820	GABBR1	**1.23E-05**	**1.20E-12**	rs1359480	13	107413961	N/A	9.89E-02	**4.28E-05**	7.49E-01	**2.37E-03**
rs2267633	6	29678820	GABBR1	**1.23E-05**	**1.20E-12**	rs1952589	14	56175083	C14orf101	6.68E-01	**3.03E-05**	5.38E-01	**2.50E-04**
rs2267633	6	29678820	GABBR1	**1.23E-05**	**1.20E-12**	rs4429270	15	89828778	N/A	1.63E-01	**6.68E-05**	4.44E-01	**2.04E-04**
rs2267633	6	29678820	GABBR1	**1.23E-05**	**1.20E-12**	rs2014341	21	33267837	N/A	1.79E-01	**5.42E-05**	5.29E-01	**2.69E-04**
rs2267633	6	29678820	GABBR1	**1.23E-05**	**1.20E-12**	rs2839557	21	42863867	SLC37A1	6.47E-01	**9.47E-05**	3.82E-02	1.01E-01
rs2267633	6	29678820	GABBR1	**1.23E-05**	**1.20E-12**	rs1788414	21	42878403	SLC37A1	6.36E-01	**7.81E-05**	4.13E-02	7.94E-02
rs2267633	6	29678820	GABBR1	**1.23E-05**	**1.20E-12**	rs450508	21	42879076	SLC37A1	6.36E-01	**7.81E-05**	4.13E-02	7.94E-02
rs2267633	6	29678820	GABBR1	**1.23E-05**	**1.20E-12**	rs401074	21	42886560	SLC37A1	9.93E-01	**4.04E-05**	1.02E-01	3.50E-02
rs2267633	6	29678820	GABBR1	**1.23E-05**	**1.20E-12**	rs133504	22	47290141	FAM19A5	5.68E-01	**4.23E-05**	4.39E-01	**7.06E-03**
rs29230	6	29684372	GABBR1	**1.71E-05**	**1.30E-12**	rs771296	2	6988986	RNF144A	2.93E-01	**6.21E-05**	2.00E-01	1.84E-02
rs29230	6	29684372	GABBR1	**1.71E-05**	**1.30E-12**	rs7560249	2	36851578	N/A	7.76E-01	**6.67E-05**	8.86E-01	**2.46E-03**
rs29230	6	29684372	GABBR1	**1.71E-05**	**1.30E-12**	rs7587476	2	215362132	BARD1	4.53E-01	**2.14E-05**	7.05E-01	**3.86E-04**
rs29230	6	29684372	GABBR1	**1.71E-05**	**1.30E-12**	rs33766	3	45255069	TMEM158	6.96E-01	**3.54E-05**	5.30E-02	**1.29E-05**
rs29230	6	29684372	GABBR1	**1.71E-05**	**1.30E-12**	rs33785	3	45268267	N/A	1.01E-01	**5.76E-05**	5.48E-01	**2.94E-04**
rs29230	6	29684372	GABBR1	**1.71E-05**	**1.30E-12**	rs33790	3	45271744	N/A	7.59E-02	**9.78E-05**	5.28E-01	**4.09E-04**
rs29230	6	29684372	GABBR1	**1.71E-05**	**1.30E-12**	rs13434896	4	189581756	N/A	3.87E-01	**8.88E-05**	8.36E-01	**1.21E-03**
rs29230	6	29684372	GABBR1	**1.71E-05**	**1.30E-12**	rs7690014	4	189581857	N/A	3.87E-01	**8.88E-05**	8.36E-01	**1.21E-03**
rs29230	6	29684372	GABBR1	**1.71E-05**	**1.30E-12**	rs2129470	5	1989443	N/A	7.90E-01	**8.98E-05**	7.20E-01	**7.92E-03**
rs29230	6	29684372	GABBR1	**1.71E-05**	**1.30E-12**	rs10060705	5	63937060	RGS7BP	4.59E-01	**4.87E-05**	5.44E-01	**1.25E-03**
rs29230	6	29684372	GABBR1	**1.71E-05**	**1.30E-12**	rs2217092	7	135635590	N/A	2.89E-01	**4.62E-05**	4.68E-01	**3.09E-04**
rs29230	6	29684372	GABBR1	**1.71E-05**	**1.30E-12**	rs9642048	7	135637615	N/A	9.17E-01	**6.97E-05**	3.09E-01	**3.23E-04**
rs29230	6	29684372	GABBR1	**1.71E-05**	**1.30E-12**	rs11976656	7	135660559	N/A	9.88E-01	**6.27E-05**	2.78E-01	**2.56E-04**
rs29230	6	29684372	GABBR1	**1.71E-05**	**1.30E-12**	rs10253012	7	135661486	N/A	2.40E-01	**8.94E-06**	2.80E-01	**5.83E-05**
rs29230	6	29684372	GABBR1	**1.71E-05**	**1.30E-12**	rs4579471	7	135670796	N/A	8.44E-01	**7.47E-05**	3.47E-01	**4.78E-04**
rs29230	6	29684372	GABBR1	**1.71E-05**	**1.30E-12**	rs1882092	7	135673778	N/A	3.73E-01	**1.25E-05**	3.53E-01	**8.43E-05**
rs29230	6	29684372	GABBR1	**1.71E-05**	**1.30E-12**	rs12671681	7	135674364	N/A	6.57E-01	**8.22E-06**	2.16E-01	**1.84E-05**
rs29230	6	29684372	GABBR1	**1.71E-05**	**1.30E-12**	rs12707297	7	135675753	N/A	6.01E-01	**8.43E-05**	1.98E-01	**1.12E-04**
rs29230	6	29684372	GABBR1	**1.71E-05**	**1.30E-12**	rs11778973	8	3676205	CSMD1	2.52E-01	**9.54E-05**	4.84E-01	**8.28E-04**
rs29230	6	29684372	GABBR1	**1.71E-05**	**1.30E-12**	rs2954610	8	3769301	CSMD1	8.81E-01	**5.53E-05**	9.25E-02	**6.90E-05**
rs29230	6	29684372	GABBR1	**1.71E-05**	**1.30E-12**	rs7357519	8	4399763	CSMD1	3.82E-01	**2.95E-05**	4.30E-01	**9.04E-05**
rs29230	6	29684372	GABBR1	**1.71E-05**	**1.30E-12**	rs9802064	8	17313332	MTMR7	4.60E-01	**2.83E-05**	5.28E-01	**5.35E-03**
rs29230	6	29684372	GABBR1	**1.71E-05**	**1.30E-12**	rs335244	8	18689427	PSD3	4.67E-02	**7.79E-05**	5.88E-01	**3.80E-03**
rs29230	6	29684372	GABBR1	**1.71E-05**	**1.30E-12**	rs3808900	9	124032585	RBM18	3.96E-01	**6.68E-05**	2.16E-01	3.96E-02
rs29230	6	29684372	GABBR1	**1.71E-05**	**1.30E-12**	rs1359480	13	107413961	N/A	9.89E-02	**3.82E-05**	7.89E-01	**2.08E-03**
rs29230	6	29684372	GABBR1	**1.71E-05**	**1.30E-12**	rs1952589	14	56175083	C14orf101	6.68E-01	**4.18E-05**	6.04E-01	**3.50E-04**
rs29230	6	29684372	GABBR1	**1.71E-05**	**1.30E-12**	rs734446	15	77115237	RASGRF1	4.18E-01	**7.54E-05**	4.85E-01	1.62E-02
rs29230	6	29684372	GABBR1	**1.71E-05**	**1.30E-12**	rs4429270	15	89828778	N/A	1.63E-01	**6.95E-05**	3.59E-01	**1.27E-04**
rs29230	6	29684372	GABBR1	**1.71E-05**	**1.30E-12**	rs2014341	21	33267837	N/A	1.79E-01	**5.10E-05**	4.20E-01	**1.84E-04**

SNP1						SNP2					Interaction p-value		
SNP	Chr.	Position	Nearest Gene	Taiwan GWAS P	China GWAS	SNP	Chr.	Position	Nearest Gene	GWAS P	Stage 1	Stage 2	Combined
rs29230	6	29684372	GABBR1	**1.71E-05**	**1.30E-12**	rs1788414	21	42878403	SLC37A1	6.36E-01	**9.86E-05**	3.13E-02	9.62E-02
rs29230	6	29684372	GABBR1	**1.71E-05**	**1.30E-12**	rs450508	21	42879076	SLC37A1	6.36E-01	**9.86E-05**	3.13E-02	9.62E-02
rs29230	6	29684372	GABBR1	**1.71E-05**	**1.30E-12**	rs401074	21	42886560	SLC37A1	9.93E-01	**5.27E-05**	1.10E-01	3.37E-02
rs29230	6	29684372	GABBR1	**1.71E-05**	**1.30E-12**	rs133504	22	47290141	FAM19A5	5.68E-01	**5.04E-05**	3.88E-01	**8.62E-03**
rs3129055	6	29778240	HLA-F	**2.27E-04**	**3.00E-07**	rs7529822	1	247055082	SH3BP5L	7.87E-01	**2.24E-05**	5.53E-01	**3.39E-04**
rs3129055	6	29778240	HLA-F	**2.27E-04**	**3.00E-07**	rs2390601	2	168223608	N/A	4.56E-01	**6.03E-05**	6.61E-01	**1.78E-03**
rs3129055	6	29778240	HLA-F	**2.27E-04**	**3.00E-07**	rs7673403	4	32550339	N/A	8.16E-02	**2.36E-05**	7.83E-01	**9.97E-04**
rs3129055	6	29778240	HLA-F	**2.27E-04**	**3.00E-07**	rs7436505	4	32583628	N/A	6.95E-02	**2.46E-05**	9.46E-01	**1.31E-03**
rs3129055	6	29778240	HLA-F	**2.27E-04**	**3.00E-07**	rs13108458	4	32614391	N/A	4.50E-02	**1.76E-05**	8.02E-01	**1.92E-03**
rs3129055	6	29778240	HLA-F	**2.27E-04**	**3.00E-07**	rs7697063	4	32623704	N/A	7.31E-02	**8.94E-06**	6.61E-01	**8.71E-04**
rs3129055	6	29778240	HLA-F	**2.27E-04**	**3.00E-07**	rs10034211	4	32627937	N/A	5.44E-02	**1.12E-05**	6.82E-01	**9.37E-04**
rs3129055	6	29778240	HLA-F	**2.27E-04**	**3.00E-07**	rs7699693	4	181532217	N/A	5.14E-01	**7.98E-05**	7.81E-01	**3.05E-03**
rs3129055	6	29778240	HLA-F	**2.27E-04**	**3.00E-07**	rs11737709	4	183920409	ODZ3	8.11E-01	**3.69E-05**	3.08E-01	1.06E-02
rs3129055	6	29778240	HLA-F	**2.27E-04**	**3.00E-07**	rs12152801	5	147888538	HTR4	4.37E-01	**6.23E-05**	8.23E-01	**4.23E-03**
rs3129055	6	29778240	HLA-F	**2.27E-04**	**3.00E-07**	rs2071596	6	31614670	NFKBIL1	4.97E-01	**1.69E-05**	4.54E-01	**2.88E-03**
rs3129055	6	29778240	HLA-F	**2.27E-04**	**3.00E-07**	rs6929796	6	31630648	NFKBIL1	6.69E-01	**9.79E-05**	5.31E-01	**6.73E-03**
rs3129055	6	29778240	HLA-F	**2.27E-04**	**3.00E-07**	rs4244456	8	19754748	INTS10	4.74E-01	**1.90E-05**	4.56E-01	**4.21E-03**
rs3129055	6	29778240	HLA-F	**2.27E-04**	**3.00E-07**	rs2551698	8	30700119	N/A	9.65E-01	**5.79E-05**	3.71E-01	**7.69E-04**
rs3129055	6	29778240	HLA-F	**2.27E-04**	**3.00E-07**	rs4762975	12	20642793	PDE3A	2.50E-01	**9.10E-05**	7.72E-01	**5.01E-03**
rs3129055	6	29778240	HLA-F	**2.27E-04**	**3.00E-07**	rs11046676	12	22928001	N/A	3.71E-02	**3.44E-05**	1.16E-01	2.30E-02
rs3129055	6	29778240	HLA-F	**2.27E-04**	**3.00E-07**	rs10466797	12	22937090	N/A	8.97E-02	**5.44E-05**	1.44E-01	2.26E-02
rs3129055	6	29778240	HLA-F	**2.27E-04**	**3.00E-07**	rs2712668	12	98110506	ANKS1B	9.35E-01	**7.29E-05**	9.65E-02	**2.73E-05**
rs3129055	6	29778240	HLA-F	**2.27E-04**	**3.00E-07**	rs1545040	19	50821839	N/A	3.77E-01	**8.94E-05**	5.11E-01	**1.38E-03**
rs3129055	6	29778240	HLA-F	**2.27E-04**	**3.00E-07**	rs2839557	21	42863867	SLC37A1	6.47E-01	**4.62E-05**	3.47E-01	1.63E-02
rs3129055	6	29778240	HLA-F	**2.27E-04**	**3.00E-07**	rs451954	21	42869023	SLC37A1	5.92E-01	**4.67E-05**	4.07E-01	1.32E-02
rs3129055	6	29778240	HLA-F	**2.27E-04**	**3.00E-07**	rs401074	21	42886560	SLC37A1	9.93E-01	**2.41E-05**	6.75E-01	**2.89E-03**
rs9258122	6	29779719	HLA-F	**2.27E-04**	**2.40E-07**	rs7529822	1	247055082	SH3BP5L	7.87E-01	**2.24E-05**	5.53E-01	**3.39E-04**
rs9258122	6	29779719	HLA-F	**2.27E-04**	**2.40E-07**	rs2390601	2	168223608	N/A	4.56E-01	**6.03E-05**	6.61E-01	**1.78E-03**
rs9258122	6	29779719	HLA-F	**2.27E-04**	**2.40E-07**	rs7673403	4	32550339	N/A	8.16E-02	**2.36E-05**	7.83E-01	**9.97E-04**
rs9258122	6	29779719	HLA-F	**2.27E-04**	**2.40E-07**	rs7436505	4	32583628	N/A	6.95E-02	**2.46E-05**	9.46E-01	**1.31E-03**
rs9258122	6	29779719	HLA-F	**2.27E-04**	**2.40E-07**	rs13108458	4	32614391	N/A	4.50E-02	**1.76E-05**	8.02E-01	**1.92E-03**
rs9258122	6	29779719	HLA-F	**2.27E-04**	**2.40E-07**	rs7697063	4	32623704	N/A	7.31E-02	**8.94E-06**	6.61E-01	**8.71E-04**
rs9258122	6	29779719	HLA-F	**2.27E-04**	**2.40E-07**	rs10034211	4	32627937	N/A	5.44E-02	**1.12E-05**	6.82E-01	**9.37E-04**
rs9258122	6	29779719	HLA-F	**2.27E-04**	**2.40E-07**	rs7699693	4	181532217	N/A	5.14E-01	**7.98E-05**	7.81E-01	**3.05E-03**
rs9258122	6	29779719	HLA-F	**2.27E-04**	**2.40E-07**	rs11737709	4	183920409	ODZ3	8.11E-01	**3.69E-05**	3.08E-01	1.06E-02
rs9258122	6	29779719	HLA-F	**2.27E-04**	**2.40E-07**	rs12152801	5	147888538	HTR4	4.37E-01	**6.23E-05**	8.23E-01	**4.23E-03**
rs9258122	6	29779719	HLA-F	**2.27E-04**	**2.40E-07**	rs2071596	6	31614670	NFKBIL1	4.97E-01	**1.69E-05**	4.54E-01	**2.88E-03**
rs9258122	6	29779719	HLA-F	**2.27E-04**	**2.40E-07**	rs6929796	6	31630648	NFKBIL1	6.69E-01	**9.79E-05**	5.31E-01	**6.73E-03**
rs9258122	6	29779719	HLA-F	**2.27E-04**	**2.40E-07**	rs4244456	8	19754748	INTS10	4.74E-01	**1.90E-05**	4.56E-01	**4.21E-03**
rs9258122	6	29779719	HLA-F	**2.27E-04**	**2.40E-07**	rs2551698	8	30700119	N/A	9.65E-01	**5.79E-05**	3.71E-01	**7.69E-04**
rs9258122	6	29779719	HLA-F	**2.27E-04**	**2.40E-07**	rs4762975	12	20642793	PDE3A	2.50E-01	**9.10E-05**	7.72E-01	**5.01E-03**
rs9258122	6	29779719	HLA-F	**2.27E-04**	**2.40E-07**	rs11046676	12	22928001	N/A	3.71E-02	**3.44E-05**	1.16E-01	2.30E-02
rs9258122	6	29779719	HLA-F	**2.27E-04**	**2.40E-07**	rs10466797	12	22937090	N/A	8.97E-02	**5.44E-05**	1.44E-01	2.26E-02
rs9258122	6	29779719	HLA-F	**2.27E-04**	**2.40E-07**	rs2712668	12	98110506	ANKS1B	9.35E-01	**7.29E-05**	9.65E-02	**2.73E-05**
rs9258122	6	29779719	HLA-F	**2.27E-04**	**2.40E-07**	rs1545040	19	50821839	N/A	3.77E-01	**8.94E-05**	5.11E-01	**1.38E-03**
rs9258122	6	29779719	HLA-F	**2.27E-04**	**2.40E-07**	rs2839557	21	42863867	SLC37A1	6.47E-01	**4.62E-05**	3.47E-01	1.63E-02
rs9258122	6	29779719	HLA-F	**2.27E-04**	**2.40E-07**	rs451954	21	42869023	SLC37A1	5.92E-01	**4.67E-05**	4.07E-01	1.32E-02
rs9258122	6	29779719	HLA-F	**2.27E-04**	**2.40E-07**	rs401074	21	42886560	SLC37A1	9.93E-01	**2.41E-05**	6.75E-01	**2.89E-03**
rs2894207	6	31371730	HLA-B/C	**5.01E-03**	**1.22E-16**	rs7649284	3	118364404	N/A	9.72E-02	**4.64E-05**	N/A	N/A
rs2894207	6	31371730	HLA-B/C	**5.01E-03**	**1.22E-16**	rs7429580	3	181267464	N/A	1.98E-01	**2.05E-05**	N/A	N/A
rs2894207	6	31371730	HLA-B/C	**5.01E-03**	**1.22E-16**	rs852604	5	10952234	N/A	7.51E-01	**3.16E-05**	N/A	N/A
rs2894207	6	31371730	HLA-B/C	**5.01E-03**	**1.22E-16**	rs9373821	6	106513692	N/A	8.06E-01	**5.79E-05**	N/A	N/A
rs2894207	6	31371730	HLA-B/C	**5.01E-03**	**1.22E-16**	rs1529021	6	147329532	N/A	4.13E-01	**6.88E-05**	N/A	N/A
rs2894207	6	31371730	HLA-B/C	**5.01E-03**	**1.22E-16**	rs9355003	6	168802701	SMOC2	2.52E-01	**5.62E-05**	N/A	N/A
rs2894207	6	31371730	HLA-B/C	**5.01E-03**	**1.22E-16**	rs9346563	6	168828486	SMOC2	1.43E-01	**5.75E-06**	N/A	N/A
rs2894207	6	31371730	HLA-B/C	**5.01E-03**	**1.22E-16**	rs4731630	7	129214437	MIR96	1.74E-01	**2.49E-05**	N/A	N/A
rs2894207	6	31371730	HLA-B/C	**5.01E-03**	**1.22E-16**	rs10900213	10	45224720	ALOX5	3.34E-01	**2.74E-05**	N/A	N/A
rs2894207	6	31371730	HLA-B/C	**5.01E-03**	**1.22E-16**	rs1864758	10	62473423	N/A	3.72E-01	**7.24E-05**	N/A	N/A
rs2894207	6	31371730	HLA-B/C	**5.01E-03**	**1.22E-16**	rs7974440	12	57699069	N/A	1.43E-01	**3.44E-05**	N/A	N/A
rs2894207	6	31371730	HLA-B/C	**5.01E-03**	**1.22E-16**	rs12591292	15	59880917	N/A	2.35E-01	**4.94E-05**	N/A	N/A
rs2894207	6	31371730	HLA-B/C	**5.01E-03**	**1.22E-16**	rs299175	19	61005340	NLRP11	7.65E-01	**4.96E-05**	N/A	N/A
rs9510787	13	23103195	TNFRSF19	**1.78E-02**	**6.32E-07**	rs2149028	1	188837471	N/A	6.36E-01	**9.30E-05**	2.78E-01	1.90E-02
rs9510787	13	23103195	TNFRSF19	**1.78E-02**	**6.32E-07**	rs937932	2	22565821	N/A	3.05E-01	**3.29E-05**	1.31E-01	4.25E-02
rs9510787	13	23103195	TNFRSF19	**1.78E-02**	**6.32E-07**	rs2303291	2	24284688	ITSN2	5.53E-01	**8.40E-05**	8.50E-01	**1.79E-03**
rs9510787	13	23103195	TNFRSF19	**1.78E-02**	**6.32E-07**	rs9814223	3	24337256	THRB	5.72E-01	**7.99E-05**	7.79E-01	**9.53E-04**
rs9510787	13	23103195	TNFRSF19	**1.78E-02**	**6.32E-07**	rs7649511	3	24340665	THRB	6.79E-01	**8.94E-05**	9.10E-01	**1.27E-03**
rs9510787	13	23103195	TNFRSF19	**1.78E-02**	**6.32E-07**	rs1560073	5	5766635	N/A	5.00E-01	**6.64E-05**	7.63E-01	**6.88E-03**
rs9510787	13	23103195	TNFRSF19	**1.78E-02**	**6.32E-07**	rs185123	5	139497381	C5orf53	4.37E-01	**8.38E-05**	8.31E-02	6.74E-02
rs9510787	13	23103195	TNFRSF19	**1.78E-02**	**6.32E-07**	rs4130208	5	158281490	EBF1	4.51E-01	**8.36E-05**	2.95E-02	**2.81E-05**
rs9510787	13	23103195	TNFRSF19	**1.78E-02**	**6.32E-07**	rs9485136	6	147343555	N/A	2.08E-01	**8.21E-05**	2.60E-01	**2.50E-04**
rs9510787	13	23103195	TNFRSF19	**1.78E-02**	**6.32E-07**	rs1990433	7	78621051	MAGI2	8.16E-01	**5.46E-05**	2.82E-01	1.24E-02
rs9510787	13	23103195	TNFRSF19	**1.78E-02**	**6.32E-07**	rs7789660	7	78646314	MAGI2	3.55E-01	**2.45E-05**	1.97E-01	**9.41E-03**
rs9510787	13	23103195	TNFRSF19	**1.78E-02**	**6.32E-07**	rs8192813	7	139225646	TBXAS1	5.51E-01	**5.38E-05**	6.90E-01	**6.40E-04**
rs9510787	13	23103195	TNFRSF19	**1.78E-02**	**6.32E-07**	rs2578240	9	90016585	N/A	1.18E-01	**1.13E-05**	N/A	N/A
rs9510787	13	23103195	TNFRSF19	**1.78E-02**	**6.32E-07**	rs3739602	9	94649385	ANKRD19	5.42E-01	**7.67E-05**	5.71E-01	**1.32E-03**
rs9510787	13	23103195	TNFRSF19	**1.78E-02**	**6.32E-07**	rs7350438	10	5683517	N/A	4.22E-01	**9.16E-05**	2.55E-01	1.82E-02
rs9510787	13	23103195	TNFRSF19	**1.78E-02**	**6.32E-07**	rs10508016	13	94798750	N/A	6.67E-01	**6.66E-05**	N/A	N/A
rs9510787	13	23103195	TNFRSF19	**1.78E-02**	**6.32E-07**	rs9590247	13	94811006	N/A	7.26E-01	**1.78E-05**	7.30E-01	**5.28E-03**
rs9510787	13	23103195	TNFRSF19	**1.78E-02**	**6.32E-07**	rs7992512	13	94811886	N/A	6.78E-01	**2.04E-05**	8.05E-01	**4.82E-03**
rs9510787	13	23103195	TNFRSF19	**1.78E-02**	**6.32E-07**	rs6030129	20	40380158	PTPRT	3.69E-01	**5.00E-05**	2.15E-01	**1.31E-04**
rs9510787	13	23103195	TNFRSF19	**1.78E-02**	**6.32E-07**	rs6072688	20	40389980	PTPRT	2.37E-01	**5.28E-05**	4.41E-01	**4.86E-04**
rs9510787	13	23103195	TNFRSF19	**1.78E-02**	**6.32E-07**	rs2227158	20	40393620	PTPRT	3.19E-01	**3.22E-05**	3.45E-01	**2.87E-04**
rs9510787	13	23103195	TNFRSF19	**1.78E-02**	**6.32E-07**	rs2823521	21	16197041	N/A	7.22E-01	**4.45E-05**	3.07E-01	**7.92E-03**
rs1412829	9	22033926	CDKN2A-CDKN2	**3.96E-02**	**2.78E-05**	rs10493275	1	60228385	C1orf87	5.94E-01	**3.31E-05**	N/A	N/A
rs1412829	9	22033926	CDKN2A-CDKN2	**3.96E-02**	**2.78E-05**	rs4129625	3	19212787	KCNH8	4.21E-01	**4.38E-05**	5.77E-02	**5.08E-03**

SNP1						SNP2					Interaction p-value		
SNP	Chr.	Position	Nearest Gene	Taiwan GWAS P	China GWAS	SNP	Chr.	Position	Nearest Gene	GWAS P	Stage 1	Stage 2	Combined
rs1412829	9	22033926	CDKN2A-CDKN2	**3.96E-02**	**2.78E-05**	rs11711956	3	54353640	CACN/A2D3	4.54E-01	**6.06E-05**	9.80E-02	9.51E-02
rs1412829	9	22033926	CDKN2A-CDKN2	**3.96E-02**	**2.78E-05**	rs2941011	4	37233755	C4orf19	2.44E-01	**5.62E-05**	1.07E-01	3.22E-02
rs1412829	9	22033926	CDKN2A-CDKN2	**3.96E-02**	**2.78E-05**	rs11942503	4	37237649	C4orf19	1.77E-01	**5.98E-05**	9.80E-02	3.42E-02
rs1412829	9	22033926	CDKN2A-CDKN2	**3.96E-02**	**2.78E-05**	rs2871247	4	182516400	N/A	5.94E-01	**9.98E-05**	3.77E-01	**9.38E-04**
rs1412829	9	22033926	CDKN2A-CDKN2	**3.96E-02**	**2.78E-05**	rs299596	5	34067771	C1QTNF3	4.77E-01	**1.07E-05**	2.69E-01	**1.53E-05**
rs1412829	9	22033926	CDKN2A-CDKN2	**3.96E-02**	**2.78E-05**	rs11760085	6	150633122	PPP1R14C	7.02E-01	**5.75E-05**	9.62E-01	**2.97E-03**
rs1412829	9	22033926	CDKN2A-CDKN2	**3.96E-02**	**2.78E-05**	rs3844600	10	49353903	ARHGAP22	5.94E-01	**1.20E-05**	8.29E-01	**2.12E-03**
rs1412829	9	22033926	CDKN2A-CDKN2	**3.96E-02**	**2.78E-05**	rs3916175	20	12928826	SPTLC3	8.89E-01	**4.35E-05**	7.16E-01	**4.99E-03**
rs6774494	3	170565327	MDS1-EVI1	2.93E-01	**6.53E-06**	rs1674877	1	4396519	LOC284661	3.50E-01	**5.82E-05**	5.98E-01	**6.78E-04**
rs6774494	3	170565327	MDS1-EVI1	2.93E-01	**6.53E-06**	rs780586	1	4404787	N/A	5.04E-01	**5.04E-05**	6.76E-01	**1.92E-03**
rs6774494	3	170565327	MDS1-EVI1	2.93E-01	**6.53E-06**	rs236291	1	93893230	BCAR3	6.52E-01	**3.48E-05**	1.32E-01	**7.43E-05**
rs6774494	3	170565327	MDS1-EVI1	2.93E-01	**6.53E-06**	rs236285	1	93905376	BCAR3	3.58E-01	**3.63E-05**	9.32E-02	**5.06E-05**
rs6774494	3	170565327	MDS1-EVI1	2.93E-01	**6.53E-06**	rs6702036	1	93924177	BCAR3	5.19E-01	**2.85E-05**	1.90E-01	**9.52E-05**
rs6774494	3	170565327	MDS1-EVI1	2.93E-01	**6.53E-06**	rs6660740	1	93930160	BCAR3	3.35E-01	**4.98E-05**	4.57E-02	**2.06E-05**
rs6774494	3	170565327	MDS1-EVI1	2.93E-01	**6.53E-06**	rs6744525	2	30439770	N/A	5.37E-01	**7.07E-05**	N/A	N/A
rs6774494	3	170565327	MDS1-EVI1	2.93E-01	**6.53E-06**	rs7641246	3	1766598	N/A	3.94E-01	**9.37E-05**	7.60E-01	**3.07E-03**
rs6774494	3	170565327	MDS1-EVI1	2.93E-01	**6.53E-06**	rs1881364	3	10946358	SLC6A11	1.30E-01	**7.37E-06**	9.05E-01	**4.07E-04**
rs6774494	3	170565327	MDS1-EVI1	2.93E-01	**6.53E-06**	rs12490768	3	39538369	MOBP	9.15E-01	**9.98E-05**	6.20E-01	**2.92E-04**
rs6774494	3	170565327	MDS1-EVI1	2.93E-01	**6.53E-06**	rs9686756	5	30406995	N/A	9.06E-01	**9.07E-05**	1.89E-01	**1.39E-04**
rs6774494	3	170565327	MDS1-EVI1	2.93E-01	**6.53E-06**	rs3799380	6	26575161	BTN3A3	9.76E-01	**3.28E-05**	1.70E-01	1.27E-02
rs6774494	3	170565327	MDS1-EVI1	2.93E-01	**6.53E-06**	rs9296015	6	32326967	N/A	5.38E-01	**7.48E-05**	4.61E-01	**3.83E-03**
rs6774494	3	170565327	MDS1-EVI1	2.93E-01	**6.53E-06**	rs4959089	6	32327703	N/A	5.38E-01	**7.48E-05**	4.61E-01	**3.83E-03**
rs6774494	3	170565327	MDS1-EVI1	2.93E-01	**6.53E-06**	rs6901782	6	43066878	PEX6	1.44E-01	**1.72E-05**	7.53E-01	**3.16E-03**
rs6774494	3	170565327	MDS1-EVI1	2.93E-01	**6.53E-06**	rs4714651	6	43078485	C6orf153	1.74E-01	**8.58E-05**	5.80E-01	1.07E-02
rs6774494	3	170565327	MDS1-EVI1	2.93E-01	**6.53E-06**	rs2026898	6	87554494	N/A	3.17E-02	**4.72E-05**	6.20E-01	**1.06E-03**
rs6774494	3	170565327	MDS1-EVI1	2.93E-01	**6.53E-06**	rs2419601	10	113796371	N/A	2.10E-01	**5.72E-05**	6.52E-01	**2.98E-03**
rs6774494	3	170565327	MDS1-EVI1	2.93E-01	**6.53E-06**	rs669244	11	121729762	N/A	4.14E-01	**7.72E-05**	8.37E-02	**2.43E-04**
rs6774494	3	170565327	MDS1-EVI1	2.93E-01	**6.53E-06**	rs2007044	12	2215221	CACN/A1C	8.04E-01	**6.35E-05**	5.47E-01	**1.46E-04**
rs6774494	3	170565327	MDS1-EVI1	2.93E-01	**6.53E-06**	rs2283292	12	2241913	CACN/A1C	8.42E-01	**1.88E-05**	6.99E-01	**1.80E-04**
rs6774494	3	170565327	MDS1-EVI1	2.93E-01	**6.53E-06**	rs2239038	12	2244391	CACN/A1C	8.95E-01	**3.16E-05**	8.02E-01	**2.11E-04**
rs6774494	3	170565327	MDS1-EVI1	2.93E-01	**6.53E-06**	rs9572286	13	69262275	KLHL1	1.76E-01	**9.13E-05**	8.25E-01	**1.36E-03**
rs6774494	3	170565327	MDS1-EVI1	2.93E-01	**6.53E-06**	rs11619836	13	69281877	KLHL1	1.98E-01	**8.27E-05**	8.63E-01	**1.13E-03**
rs6774494	3	170565327	MDS1-EVI1	2.93E-01	**6.53E-06**	rs1188187	14	55589993	N/A	1.53E-01	**6.93E-05**	**2.94E-03**	3.82E-01
rs6774494	3	170565327	MDS1-EVI1	2.93E-01	**6.53E-06**	rs7188834	16	10628697	TEKT5	5.52E-01	**3.71E-05**	6.22E-01	**4.82E-04**
rs6774494	3	170565327	MDS1-EVI1	2.93E-01	**6.53E-06**	rs4074471	16	25866851	HS3ST4	7.17E-01	**5.78E-05**	9.56E-01	**3.30E-03**
rs6774494	3	170565327	MDS1-EVI1	2.93E-01	**6.53E-06**	rs16978310	18	41077512	N/A	9.65E-01	**9.05E-05**	5.56E-01	**2.85E-04**
rs6774494	3	170565327	MDS1-EVI1	2.93E-01	**6.53E-06**	rs898438	18	48485785	DCC	4.23E-01	**9.82E-05**	3.23E-01	2.60E-02
rs6774494	3	170565327	MDS1-EVI1	2.93E-01	**6.53E-06**	rs4809015	19	19922125	ZNF93	6.53E-01	**4.42E-05**	8.81E-01	**1.17E-03**
rs1572072	13	23025210	TNFRSF19	3.15E-01	**3.52E-04**	rs13005761	2	40391203	SLC8A1	9.95E-02	**2.89E-05**	7.63E-01	**7.83E-04**
rs1572072	13	23025210	TNFRSF19	3.15E-01	**3.52E-04**	rs759387	2	40401900	SLC8A1	6.94E-02	**5.85E-05**	9.75E-01	**2.43E-03**
rs1572072	13	23025210	TNFRSF19	3.15E-01	**3.52E-04**	rs6745475	2	130175179	N/A	4.93E-02	**3.04E-05**	**8.10E-03**	**4.45E-07**
rs1572072	13	23025210	TNFRSF19	3.15E-01	**3.52E-04**	rs12206002	6	145581160	N/A	6.42E-01	**8.19E-06**	9.87E-01	**4.59E-04**
rs1572072	13	23025210	TNFRSF19	3.15E-01	**3.52E-04**	rs993701	6	145588357	N/A	8.82E-01	**9.27E-05**	9.41E-01	**1.76E-03**
rs1572072	13	23025210	TNFRSF19	3.15E-01	**3.52E-04**	rs2513755	8	95166845	N/A	5.43E-01	**9.78E-05**	4.86E-01	**4.63E-04**
rs1572072	13	23025210	TNFRSF19	3.15E-01	**3.52E-04**	rs10809863	9	12946312	N/A	1.14E-02	**2.00E-05**	9.29E-01	**1.39E-03**
rs1572072	13	23025210	TNFRSF19	3.15E-01	**3.52E-04**	rs10809868	9	12949428	N/A	1.32E-02	**3.49E-05**	9.89E-01	**2.26E-03**
rs1572072	13	23025210	TNFRSF19	3.15E-01	**3.52E-04**	rs11635176	15	58367625	N/A	2.94E-01	**6.67E-05**	3.59E-02	**2.31E-05**
rs1572072	13	23025210	TNFRSF19	3.15E-01	**3.52E-04**	rs16942401	15	58369749	N/A	2.94E-01	**6.13E-05**	3.70E-02	**2.06E-05**
rs1572072	13	23025210	TNFRSF19	3.15E-01	**3.52E-04**	rs12903708	15	58380597	N/A	2.10E-01	**2.67E-05**	8.69E-02	**2.87E-05**
rs1572072	13	23025210	TNFRSF19	3.15E-01	**3.52E-04**	rs11635321	15	58383100	N/A	2.10E-01	**2.67E-05**	1.02E-01	**3.34E-05**
rs1572072	13	23025210	TNFRSF19	3.15E-01	**3.52E-04**	rs1509808	15	58386569	N/A	2.09E-01	**1.84E-05**	7.22E-02	**1.63E-05**
rs1572072	13	23025210	TNFRSF19	3.15E-01	**3.52E-04**	rs4238349	15	58392217	N/A	2.35E-01	**3.45E-05**	7.22E-02	**2.67E-05**
rs1572072	13	23025210	TNFRSF19	3.15E-01	**3.52E-04**	rs218655	17	6514688	MED31	4.94E-01	**5.95E-05**	4.16E-01	**3.40E-04**
rs1572072	13	23025210	TNFRSF19	3.15E-01	**3.52E-04**	rs7214006	17	36600822	KRTAP4-2	2.88E-01	**4.07E-05**	9.87E-01	**1.53E-03**
rs1572072	13	23025210	TNFRSF19	3.15E-01	**3.52E-04**	rs17804219	18	70073499	N/A	7.18E-01	**6.91E-05**	9.58E-01	**8.71E-04**
rs1572072	13	23025210	TNFRSF19	3.15E-01	**3.52E-04**	rs6517568	21	40056753	IGSF5	3.67E-01	**7.30E-05**	3.01E-01	**9.20E-03**

Note: Significant SNP rs28421666 discovered by China's NPC GWAS study was not inclueded in Taiwan's GWAS study, therefore did not list in this table.
Note': P values lower than 0.05 was shown in bold face.
